# Organocatalytic enantioconvergent α-substitution of sulfonium salts by nitrogen nucleophiles

**DOI:** 10.1039/d6sc04100k

**Published:** 2026-07-30

**Authors:** Zhiyang Li, Yao Luo, Alex C. Bissember, Jianwei Sun

**Affiliations:** a Department of Chemistry and the Hong Kong Branch of Chinese National Engineering Research Centre for Tissue Restoration & Reconstruction, The Hong Kong University of Science and Technology Clear Water Bay Kowloon Hong Kong SAR 999077 China sunjw@ust.hk; b School of Natural Sciences – Chemistry, University of Tasmania Hobart Tasmania 7001 Australia

## Abstract

Chiral α-amino carbonyl compounds are essential building blocks in the synthesis of natural products and pharmaceuticals. Direct enantioconvergent nucleophilic substitution represents a powerful approach, but it has not been well established for the synthesis of α-amino ketones. Herein, we report a robust organocatalytic protocol with α-keto sulfonium salts. Utilizing a chiral phosphoric acid (CPA) in an aqueous/organic biphasic system, we achieved enantioconvergent substitution with free amines in high yields and excellent enantioselectivity. Mechanistic studies suggest that the CPA plays a dual role as both the source of chirality and a phase-transfer catalyst, facilitating the reaction *via* a dynamic kinetic resolution of a chiral sulfonium ion pair. This mild protocol was successfully extended to asymmetric azidation using a neutral hydrogen-bond-donor (HBD) catalyst.

## Abstract 2

An efficient and robust phase-transfer catalytic protocol was developed for the enantioconvergent synthesis of chiral α-amino and α-azido ketones. This protocol is complementary to transition-metal-catalyzed approaches.

## Graphical abstract

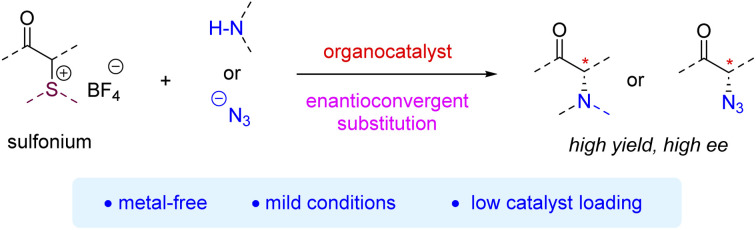

## Introduction

**Scheme 1:**
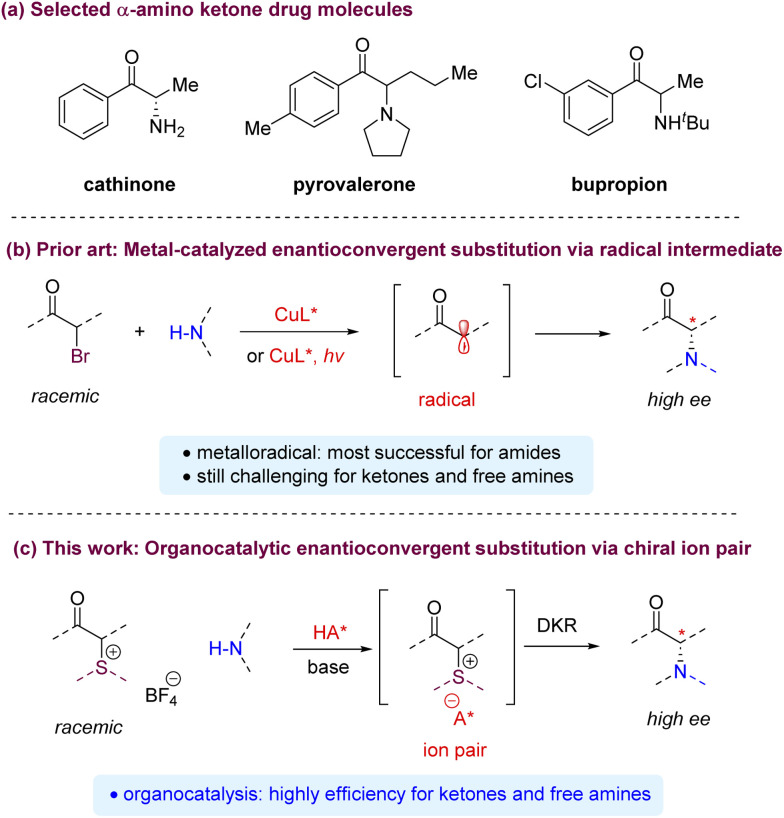
Introduction to chiral amino ketones and their enantioconvergent synthesis. (a) Selected α-amino ketone drug molecules. (b) Prior art using metal-catalyzed strategy. (c) The present reaction design.

Chiral α-amino carbonyl compounds, particularly α-amino ketones, are prevalent in natural products, pharmaceuticals, and functional materials (Scheme 1a).^1^ These compounds also serve as vital precursors to synthetically valuable chiral building blocks and ligands, such as amino alcohols and diamines.^2^ The versatility of these structures has inspired significant investigations into diverse synthetic methodologies.^3–5^ Among them, the enantioconvergent substitution of the readily available α-halo carbonyl compounds by amines represents arguably the most direct and versatile approach to enantioenriched α-amino carbonyl compounds. Indeed, as pioneered by the Fu and Liu laboratories, copper-based catalyst systems are particularly powerful in promoting this type of transformation *via* putative metalloradical intermediates (Scheme 1b).^5^ High efficiency and enantioselectivity could be achieved with different carbonyl compounds and nitrogen sources, but mostly for amide carbonyls and acidic nitrogen nucleophiles. In contrast, the use of free amines as nucleophiles for ketones remains less explored. More recently, our laboratory developed a complementary approach for the direct substitution of racemic α-bromo ketones by free amines. However, this strategy was limited to kinetic resolution and enantioconvergence could not be achieved.^6^

Our laboratory has been interested in deploying sulfonium and sulfoxonium ylides in enantioselective transformations.^7–12^ This includes employing these substrates for the synthesis of enantioenriched α-amino ketones derivatives *via* an asymmetric N–H insertion reaction with excellent yield and selectivity.^7^ Nevertheless, several inherent limitations remain to be addressed in formal insertion chemistry in these systems. For example, the relatively poor stability of sulfur ylides and the non-trivial additional step required for their synthesis restrict their scope, utility, and applications. Moreover, the previous protocol, which is performed under acidic conditions, features rather narrow amine nucleophile scope. Low efficiency was observed for both electron-deficient aryl amines and high basic aliphatic amines.^7^

In this context, we envisioned that stable α-keto sulfonium salts, which serve as synthetic precursors to sulfonium ylides, and possess readily racemizable α-stereogenic centers, may represent ideal substrates for enantioconvergent aminations under basic conditions. We envisaged this manifold would provide broader substrate scope. However, successful implementation requires identification of an effective chiral anion-exchange protocol that enables the use of a chiral anion in catalytic quantities, while simultaneously suppressing the competing racemic background reaction.^12^ We, therefore, hypothesized that a phase-transfer system could ensure that the asymmetric bond-forming step proceeds in the organic phase, while the sulfonium substrates remain sequestered in the aqueous or solid phase when not associated with the chiral catalyst (Scheme 1c). Herein, we report the development of this protocol and its extension to asymmetric azidation, providing an attractive complement to existing methods.

## Results and discussion

Our study commenced with racemic sulfonium salt 1a and *p*-anisidine 2a as model substrates, using Cs_2_CO_3_ as the base in toluene (Table 1). Chiral phosphoric acid (CPA) A,^13^ which was the optimal catalyst in our previous study on sulfonium ylides,^7^ promoted the enantioselective formation of the desired product, but with moderate efficiency and enantioselectivity (entry 1). Screening of other chiral CPAs revealed that while SPINOL-derived catalyst B1 did not improve the outcome (entry 2), B2 provided both high efficiency and excellent enantioselectivity (entry 3). Although these reactions were generally clean, reaction rates varied significantly; this variation may stem from differences in phase-transfer efficiency between the solid and organic phases.

**Table 1:** Evaluation of conditions for enantioselective amination of 1a^a^

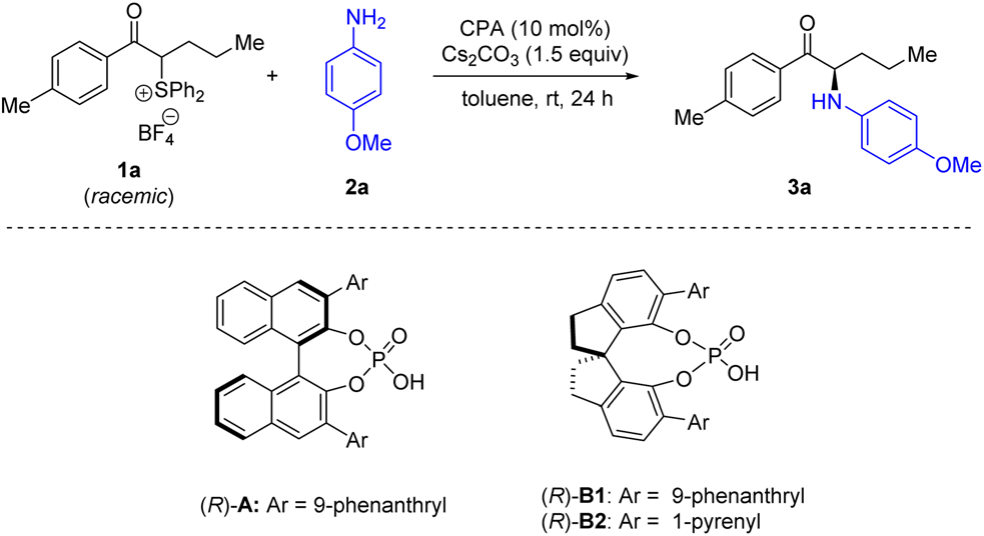					
Entry	Catalyst	Base (equiv.)	*t* (h)	Yield (%)	ee (%)
1	(*R*)-A	Cs_2_CO_3_ (1.5)	24	46	−87
2	(*R*)-B1	Cs_2_CO_3_ (1.5)	24	23	87
3	(*R*)-B2	Cs_2_CO_3_ (1.5)	24	>95	95
4^b^	(*R*)-B2	Cs_2_CO_3_ (1.5)	2	>95	91
5^b^	(*R*)-B2	K_2_CO_3_ (1.5)	2	>95	95
6^b^	(*R*)-B2	Na_2_CO_3_ (1.5)	2	>95	72
7^b^	(*R*)-B2	NaHCO_3_ (1.5)	2	>95	73
8^b^	(*R*)-B2	K_2_CO_3_ (1.0)	2	>95	95

**Change from entry 8**					
9^b^	CH_2_Cl_2_ in place of toluene			>95	93
10^b^	EtOAc in place of toluene			>95	87
11^b^	*n*-Hexane in place of toluene			90	57
12^b^	1.0 mol% (*R*)-B2			>95	95
13^c^	1a (0.4 mmol), 1.0 mol% (*R*)-B2			96	84
14^c^^,^^d^	1a (0.4 mmol), 1.0 mol% (*R*)-B2			93	95

a Reaction conditions: 1a (0.05 mmol), 2a (0.055 mmol), catalyst (10 mol%), toluene (0.5 mL). Yield is based on ^1^H NMR spectroscopic analysis of the crude reaction mixture using CH_2_Br_2_ as an internal standard. The ee value was determined by chiral HPLC analysis.

b H_2_O (0.5 mL) was added.

c Performed at 0.4 mmol scale, H_2_O (4 mL), Isolated yield is provided.

d Performed at 0 °C for 4 h.

We next investigated whether an aqueous/organic biphasic system could accelerate the reaction. Indeed, the simple addition of water allowed the reaction to reach completion within 2 h, albeit with a slight erosion in enantioselectivity, likely due to an accelerated background reaction (entry 4). However, switching the base to K_2_CO_3_ restored the excellent enantioselectivity (entry 5). In contrast, Na_2_CO_3_ and NaHCO_3_ led to dramatic decreases in enantiocontrol (entries 6–7). Reducing the K_2_CO_3_ loading to one equivalent did not compromise the results (entry 8). While replacing toluene with CH_2_Cl_2_ maintained the level of enantiocontrol; other solvents (*e.g.*, EtOAc and hexane) were significantly inferior (entries 9–11). This phase-transfer system proved robust enough to allow a reduction in catalyst loading to 1 mol% without a loss of efficiency (entry 12). Although scaling the reaction to 0.4 mmol resulted in a significant decrease in enantioselectivity in the first instance (84% ee, entry 13), lowering the temperature to 0 °C successfully restored the high enantioselectivity without negatively affecting chemical efficiency (entry 14).

**Scheme 2:**
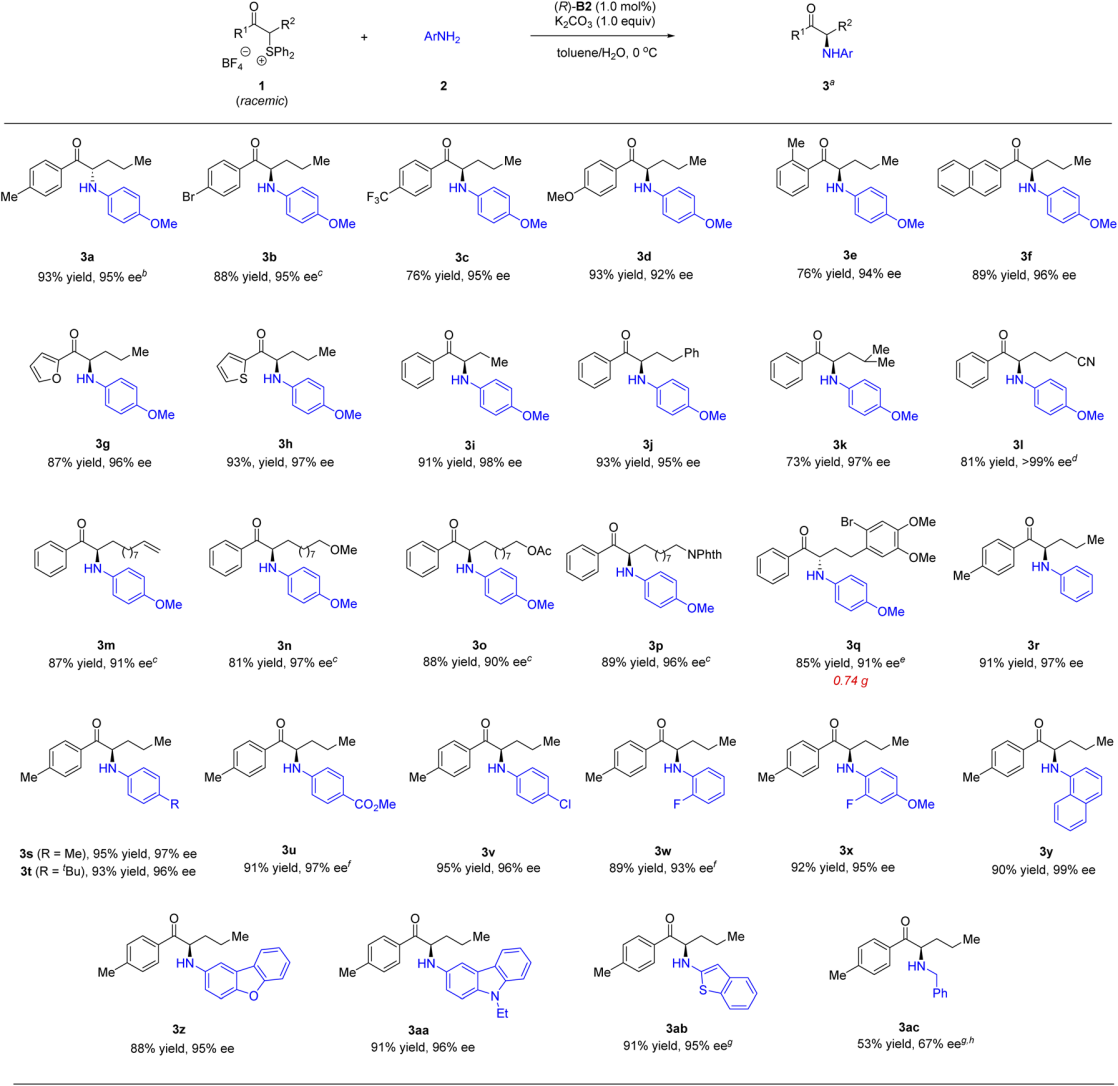
Reaction scope for the synthesis of chiral amino ketones. ^*a*^Reaction conditions: 1 (0.4 mmol), (*R*)-B2 (4.0 µmol), K_2_CO_3_ (0.4 mmol), amine (0.44 mmol), toluene (4 mL), H_2_O (4 mL), 0 °C, 5 h. Isolated yield is provided. The ee value was determined by chiral HPLC analysis. ^*b*^(*S*)-B2 (4.0 µmol) was used. ^*c*^Run at −10 °C with toluene (4 mL) and saturated brine (4 mL) as solvent. ^*d*^Run at −20 °C with toluene (4 mL) and saturated brine (4 mL) as solvent. ^*e*^Run in 1.8 mmol scale at −20 °C with toluene (18 mL) and saturated brine (18 mL) as solvent; (*S*)-B2 (18.0 µmol) was used. ^*f*^5 mol% (*R*)-B2 was used. ^*g*^Run with 10 mol% of (*R*)-B2. ^*h*^Run at room temperature.

**Scheme 3:**
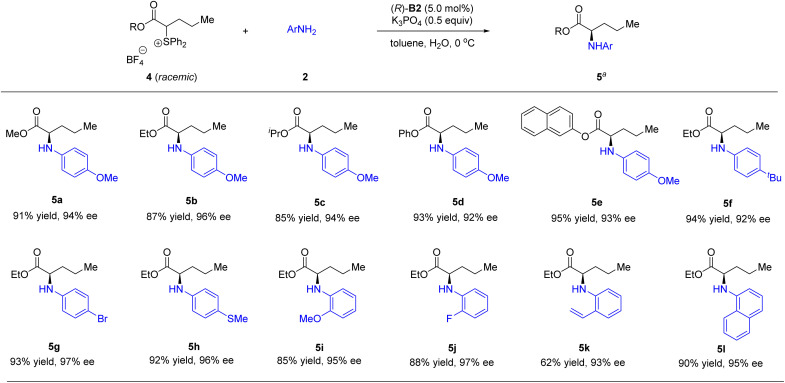
Reaction scope for the synthesis of chiral amino esters. ^*a*^Reaction conditions: 4 (0.4 mmol), (*R*)-B2 (20.0 µmol), K_3_PO_4_ (0.2 mmol), amine (0.44 mmol), toluene (4 mL), H_2_O (4 mL), 0 °C, 5 h. Isolated yield is provided. The ee value was determined by chiral HPLC analysis.

With the optimized conditions in hand, we examined the substrate scope with respect to both α-keto sulfonium salts and amines (Schemes 2 and 3). Aryl ketones bearing diverse electronic and steric properties (3a–3e) were well-suited for this protocol, delivering chiral α-amino ketones with consistently high enantioselectivity. Naphthyl (3f) and heteroaryl (3g and 3h) ketones performed equally well. Furthermore, various alkyl side chains (3i–3p) had little influence on the reaction performance. This mild protocol tolerated a wide range of functional groups, including nitriles, olefins, ethers, esters, and phthalimides. The robustness of the method was further demonstrated by a 0.7 gram synthesis of 3q, which proceeded without any loss in efficiency or enantioselectivity.

Additionally, various substituted aniline nucleophiles, bearing either electron-donating or electron-withdrawing groups, exhibited excellent reactivity, providing the corresponding α-amino ketones with enantioselectivities as high as 98% ee. Notably, while our previous CPA-catalyzed homogeneous protocol using α-keto sulfonium ylides was incompatible with electron-deficient anilines, this phase-transfer system proved highly efficient for such substrates, including those bearing strong electron-withdrawing ester substituents (3u), thereby offering a valuable complement to existing strategies. To our delight, benzylamine also participated successfully in this reaction, affording 3ac in moderate yield with good enantioselectivity. Previous protocols employ sulfur ylides under acidic conditions which are not compatible with basic aliphatic amines.^7*a*^ Importantly, this result provides important new substrate scope and highlights the capacity of this approach to efficiently engage challenging basic nucleophiles in these transformations. While moderate enantioselectivity was obtained directly from aliphatic amines, the *para*-methoxyphenyl group in 3a could be oxidatively cleaved (without erosion of ee) to generate the corresponding free primary amine (see the SI for details), thus readily permitting subsequent *N*-alkylation or other amine functionalization.

Beyond α-amino ketones, this protocol was successfully extended to the synthesis of α-amino esters (Scheme 3). By switching the base from K_2_CO_3_ to K_3_PO_4_, ester hydrolysis was effectively suppressed, allowing various chiral α-amino esters to be obtained with high efficiency and excellent enantioselectivity. Importantly, several sensitive functional groups that are typically incompatible with metal-carbene chemistry remained intact under these mild conditions, highlighting the superior functional group tolerance of this approach.

**Scheme 4:**
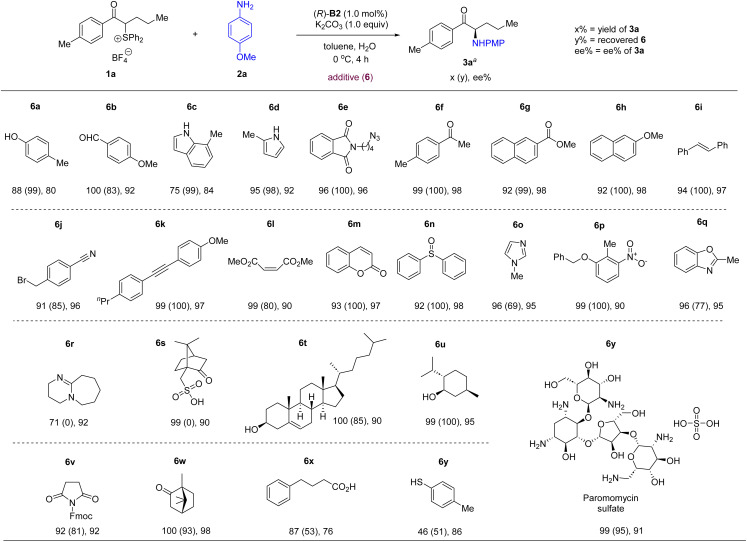
Functional group compatibility. *^a^*Reaction conditions: 1a (0.05 mmol), 2a (0.055 mmol), (*R*)-B2 (1 mol%), K_2_CO_3_ (0.05 mmol), toluene (0.5 mL), H_2_O (0.5 mL), 0 °C, 4 h. Yield is based on analysis of the ^1^H NMR spectroscopy of the crude reaction mixture using CH_2_Br_2_ as an internal standard. The ee value was determined by chiral HPLC analysis.

To further investigate the functional group tolerance of this asymmetric phase-transfer catalytic protocol, we examined the effects of various additives (6) on the standard reaction of 1a (Scheme 4). The reaction exhibited broad compatibility with a wide range of additives bearing common functionalities, including aldehydes (6b), amides, azides (6e), ketones (6f), esters (6g), ethers (6h), nitriles (6j), alkynes (6k), alkenes (6l), α,β-unsaturated esters (6m), sulfoxides (6n), nitro groups (6p), and oxazolines (6q). In the majority of these cases, both high efficiency and enantioselectivity were maintained. DBU (6r) and CSA (6s) were also evaluated, and they showed minimal influence on the reaction performance. The low recovery of certain additives may derive from their good water solubility. Limitations were observed for carboxylic acids or thiols (6x and 6y). These functional groups may interfere with ion-pairing interactions and/or serve as competing nucleophiles. Encouraged by these results, we evaluated the reaction in the presence of complex natural products and drug molecules, such as menthol, camphor, and the antibiotic paromomycin; none of these showed a detrimental effect on the reaction outcome. These results confirm the robustness of our protocol and its potential for the late-stage functionalization of complex molecules.

**Scheme 5:**
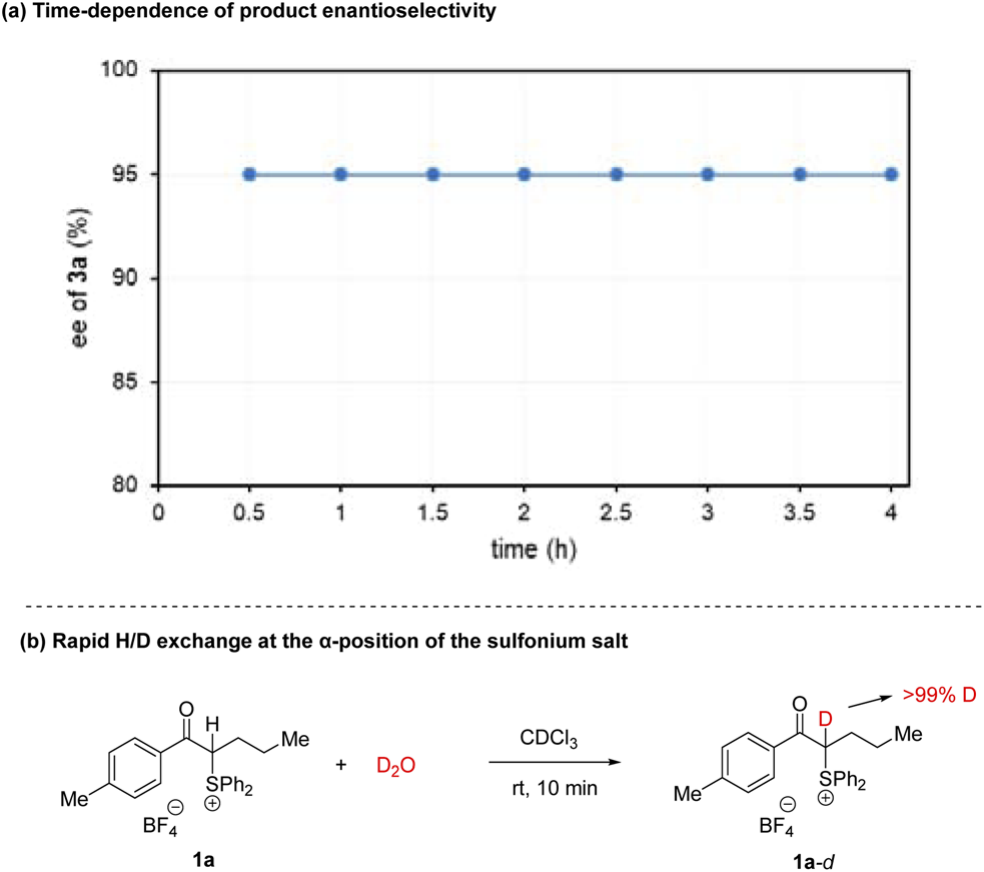
Mechanistic experiments. (a) Time-dependence of product enantioselectivity. (b) Rapid H/D exchange at the α-position of the sulfonium salt.

Next, we carried out some additional studies to gain insights into the mechanism, particularly on the proposed dynamic kinetic resolution (DKR) pathway. Firstly, the product enantiopurity was monitored during reaction progress, which indicated that the enantioselectivity remained constant (95% ee) over time (Scheme 5a). This result is consistent with the DKR mechanism. In addition, the α-keto sulfonium salt underwent rapid H/D exchange with D_2_O, suggesting facile deprotonation and protonation at the stereogenic α-carbon (Scheme 5b). This finding supports rapid interconversion between the two substrate enantiomers under the reaction conditions. Taken together, the foregoing experiments are consistent with the viability of a DKR process in this chemistry.

**Scheme 6:**
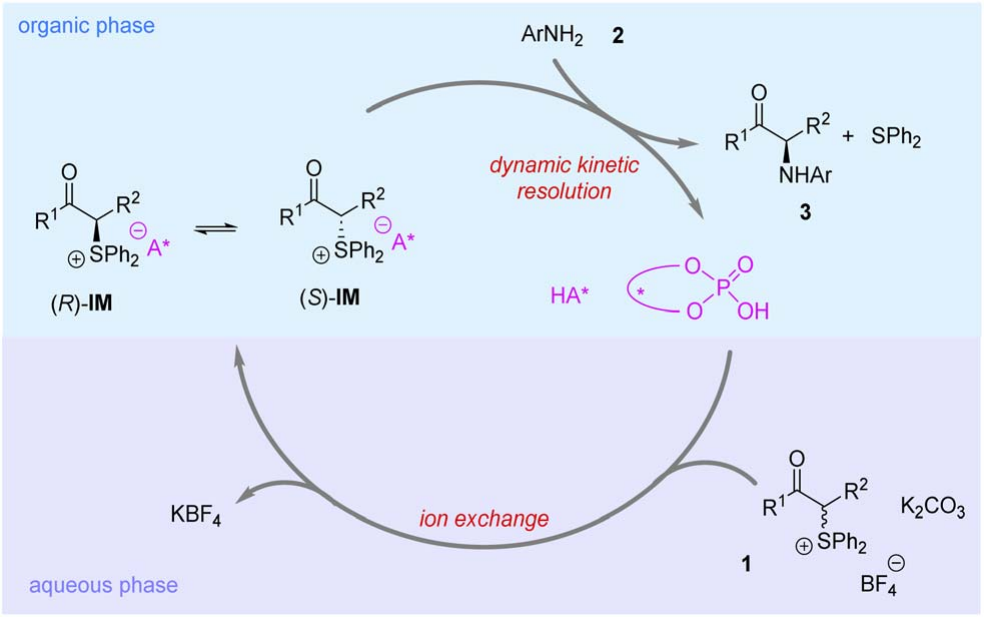
Proposed mechanism for the enantioconvergent amination of sulfonium salts.

Based on above results, a proposed mechanism is depicted in Scheme 6. The base and sulfonium salts are soluble in the aqueous phase but are insoluble in the organic phase in which the amine nucleophile is partitioned. Upon the addition of the base, an ion exchange between the chiral phosphate anion and BF_4_^−^ generates the chiral sulfonium ion pair IM. This species enters the organic phase due to its increased lipophilicity. Subsequently, the amine nucleophile reacts preferentially with the matched stereoisomer, (*S*)-IM, to form the enantioenriched product 3. The mismatched isomer, (*R*)-IM, undergoes racemization to (*S*)-IM at the labile α-stereocenter, allowing the reaction to proceed *via* DKR.^7,11^ In this catalytic cycle, the CPA serves a dual role, acting as both the source of chirality for enantiocontrol and as a phase-transfer catalyst to facilitate the reaction.

**Scheme 7:**
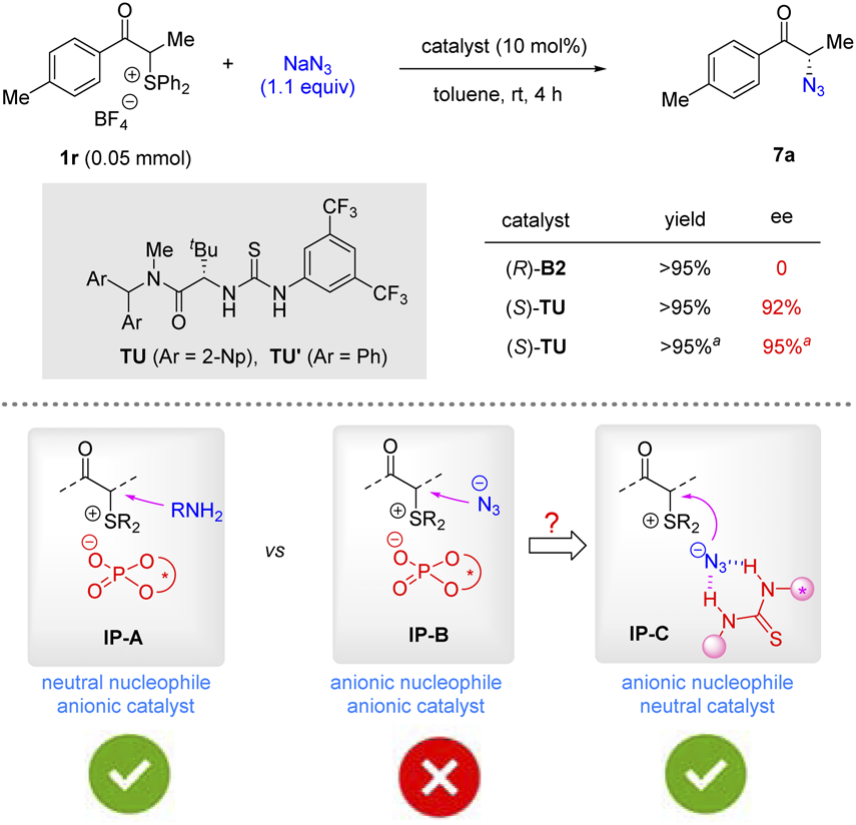
Extension of enantioconvergent α-substitution strategy to permit enantioselective azidation. ^*a*^Run with *o*-xylene as solvent at −15 °C for 24 h.

The exceptional functional group tolerance of this mild protocol prompted us to explore its potential for other C–N bond-forming reactions, particularly those typically incompatible with metal-carbene chemistry, such as α-azidation.^14,15^ We therefore investigated the reaction using NaN_3_ as the nucleophile (Scheme 7). Although catalyst (*R*)-B2 provided the α-azido ketone 7a in quantitative yield, it afforded no enantioselectivity. We reasoned that while the chiral ion pair IP-A is effective for reactions with neutral amine nucleophiles, it is superseded by the formation of an achiral ion pair between the sulfonium cation and the anionic azide (IP-B), leading to a racemic background reaction. To suppress this competing process, we hypothesized that a neutral hydrogen-bond-donor (HBD) catalyst capable of tightly binding the azide anion could be employed.^11,16^ Indeed, the chiral thiourea TU catalyzed the formation of 7a with excellent enantioselectivity (92% ee). Minor optimization using *o*-xylene as the solvent at −15 °C further improved the enantioselectivity to 95% ee.

**Scheme 8:**
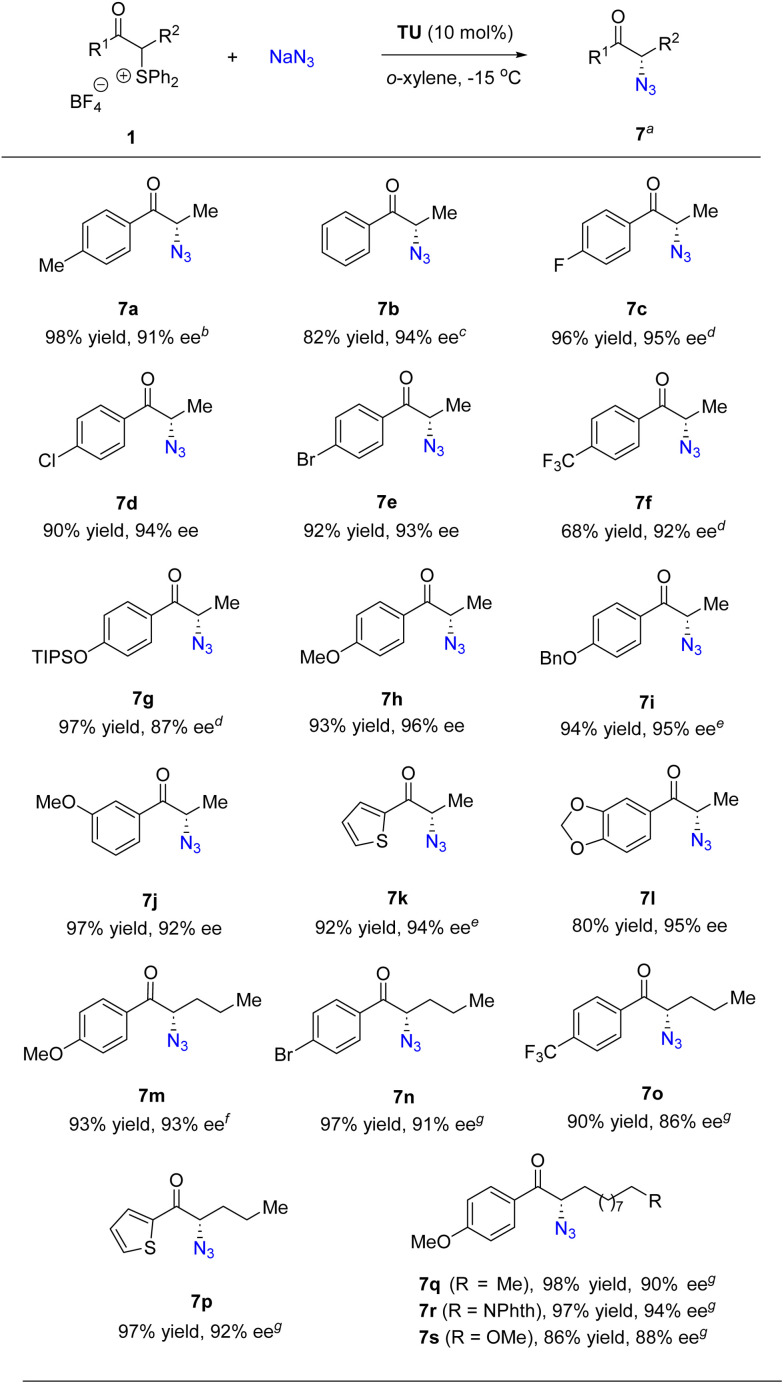
Scope for the synthesis of chiral α-azido ketones. ^*a*^Reaction conditions: 1 (0.5 mmol), TU (0.05 mmol), NaN_3_ (0.55 mmol), *o*-xylene (5 mL), −15 °C. Isolated yield. The ee value was determined by chiral HPLC analysis. ^*b*^Run with 1 mol% of TU. ^*c*^Run at −30 °C with *m*-xylene (5 mL). ^*d*^Run at −25 °C with 20 mol% of TU and *m*-xylene (5 mL). ^*e*^Run with 20 mol% of TU. ^*f*^Run with 10 mol% of TU′. ^*g*^Run at −30 °C with 20 mol% of TU′ and *m*-xylene (5 mL).

Next, we investigated the generality of this asymmetric azidation process (Scheme 8). A wide range of α-azido ketones bearing various substituents, including halides, CF_3_, silyl-protected alcohols, and heterocycles at different positions were smoothly obtained in high yields and with excellent enantioselectivity. This expanded scope not only underscores the superiority of this protocol over conventional diazo chemistry but also highlights its advantages over sulfur ylide-based formal N–H insertion strategies.

## Conclusions

In summary, we have developed a highly efficient and robust asymmetric phase-transfer catalytic protocol for the synthesis of chiral α-amino carbonyl compounds and α-azido ketones. By utilizing stable and easily accessible sulfonium salts as direct substrates, we successfully bypassed the safety hazards associated with α-diazo compounds and the stability issues inherent to α-keto sulfur ylides. The chiral organic catalysts serve a dual role, acting as both the source of enantiocontrol and the phase-transfer agent. This strategy provides high enantioselectivities (up to 98% ee) across a broad substrate scope and exhibits exceptional functional group compatibility, tolerating sensitive moieties incompatible with metal-carbene chemistry. Mechanistic insights further enabled the smooth extension of this protocol to asymmetric azidation mediated by a neutral hydrogen-bond-donor catalyst. The operational simplicity, scalability, and tolerance for sensitive functionalities make this protocol a powerful, safer alternative to traditional diazo-based transformations and open new avenues for developing asymmetric phase-transfer processes involving unstable intermediates.

## Author contributions

Z. L. and Y. L. conceived the project, performed the experiments, and wrote the paper. A. B. analyzed the results, discussed the mechanism and revised the paper. J. S. conceived and directed the project and wrote the paper. All the authors discussed the results and commented on the manuscript.

## Conflicts of interest

All the authors have no competing interests to declare.

## Supplementary Material

SC-OLF-D6SC04100K-s001

## Data Availability

Supplementary information: all data, including experimental details, characterization data, NMR spectra and HPLC traces are available in SI. See DOI: https://doi.org/10.1039/d6sc04100k.
