# Supplementary material for: Organocatalytic enantioconvergent α-substitution of sulfonium salts by nitrogen nucleophiles
Source: Chem Sci. 2026 Jul 30;17(38):18213–9. doi: 10.1039/d6sc04100k (PMC13458546; doi:10.1039/d6sc04100k)
Supplement: Supplementary file 1 [file SC-017-D6SC04100K-s001.pdf]

Supplementary Information (SI) for Chemical Science.
This journal is © The Royal Society of Chemistry 2026

Supporting Information

Organocatalytic Enantioconvergent α-Substitution of Sulfonium Salts by Nitrogen Nucleophiles

Zhiyang Li,[+a] Yao Luo,[+a] Alex C. Bissember[b] and Jianwei Sun[a*]

[a]*Department of Chemistry and the Hong Kong Branch of Chinese National Engineering Research Centre for Tissue Restoration & Reconstruction, The Hong Kong University of Science and Technology, Clear Water Bay, Kowloon, Hong Kong SAR 999077, China*

[b]*School of Natural Sciences – Chemistry, University of Tasmania, Hobart, Tasmania 7001, Australia*

[+]*These authors contributed equally to this work.*

Table of Contents

I. General Information S-2

II. Synthesis of the Sulfonium Substrates S-4

III. Catalytic Asymmetric N–H Insertion S-30

IV. Catalytic Asymmetric H–N_3 Insertion S-63

V. Mechanistic Studies S-78

VI. Deprotection of *p*-Methoxyphenyl Group S-80

VII. Determination of Absolute Stereochemistry S-81

VIII. References S-84

NMR Spectra and HPLC Traces

I. General Information

Flash column chromatography was performed over silica gel (200-300 or 300-400 mesh) purchased from Qindao Haiyang Co., China or SiliCycle® Inc., Canada. All air or moisture sensitive reactions were conducted in oven-dried glassware under nitrogen atmosphere using anhydrous solvents. Tetrahydrofuran was distilled from sodium/benzophenone. Anhydrous *N,N*-dimethyl formamide, dichloromethane, methanol, toluene, diethyl ether, acetonitrile, and *n*-hexane were purified by the Innovative® solvent purification system. Other anhydrous solvents were purchased from Sigma-Aldrich®, J&K® and Energy® and used as received. Chemicals were purchased from commercial suppliers, such as Sigma-Aldrich®, J&K®, Energy® and used without further purification unless otherwise stated. Chiral organocatalysts were purchased from Daicel® Chiral Technologies (China) Co., Ltd. and used as received. NMR spectra were recorded with a Bruker AVII, AVIII or NEO 400 spectrometer at 400 MHz (^{1}H NMR), 101 MHz (^{13}C NMR) and 376 MHz (^{19}F NMR). Chemical shifts (δ values) were reported in ppm down field from an internal standard (^{1}H NMR: Me_4Si at 0.00 ppm and ^{13}C NMR: $CDCl_3$ at 77.00 ppm). Data for ^{1}H NMR were recorded as follows: chemical shift (δ, ppm), multiplicity (s = singlet; d = doublet; t = triplet; q = quarter; p = pentet; sept = septet; m = multiplet; br = broad), coupling constant (Hz), integration. The mass spectra were collected from a Waters Xevo G2-XS Tof, with ESI source; or a Waters GCT premier with EI/CI source. Optical rotations were measured on a JASCO P-2000 polarimeter or an AUTOPOL I Automatic polarimeter with $[\alpha]_D$ values reported in degrees; concentration (c) is in 10 mg/mL. Enantioselectivities were recorded on an Agilent HPLC instrument, using a chiral stationary phase column (Daicel CHIRALPAK® AD-H, AD-3, AS-H, IC, CHIRALCEL® OD-H). The chiral HPLC methods were calibrated with the corresponding racemic mixtures. Unless otherwise noted, the racemic samples

in this study were prepared using the racemic phosphoric acid catalyst 1,1'-binaphthyl-2,2'-diyl hydrogen phosphate or a background reaction between **1** and **4** (or **2** and NaN_3) together with K_2CO_3 in DCM at room temperature.

II. Synthesis of the Sulfonium Substrates

General Procedure A

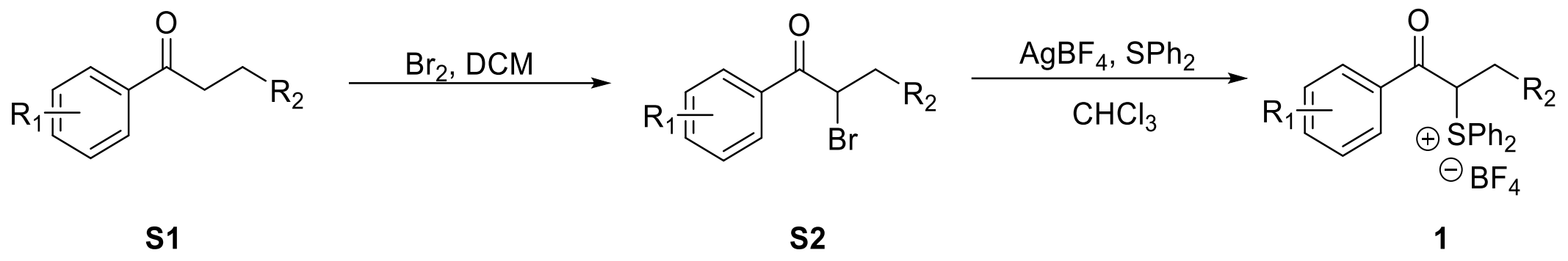

At 0 °C, to a solution of ketone **S1** (1.0 equiv) in DCM (0.1 M) was added dropwise bromine (1.0 equiv). The solution was kept stirring at room temperature for 12 h. Then, a saturated aqueous solution of $Na_2S_2O_3$ (15 mL) was slowly added. The mixture was stirred at room temperature for 15 min before the organic layer was separated. The aqueous layer was extracted with DCM (30 mL × 3). The combined organic layers were dried over anhydrous Na_2SO_4, filtered, and concentrated. The crude mixture was purified by flash silica gel column chromatography to give the *α*-bromo ketone **S2**.

To an oven-dried 100-mL sealed tube were added the *α*-bromo ketone **S2** (1.0 eqiuv), diphenyl sulfide (2.0 equiv), and dry $CHCl_3$ (0.5 M). Then, $AgBF_4$ (1.0 equiv) was added quickly before the tube was carefully sealed. The reaction mixture was kept stirring at 85 °C for 12 h. Next, the mixture was cooled to room temperature, filtered through a short pad of celite, and washed with DCM (100 mL). The filtrate was concentrated and purified by flash column chromatography on silica gel to afford the sulfonium salt **1**.

General Procedure B

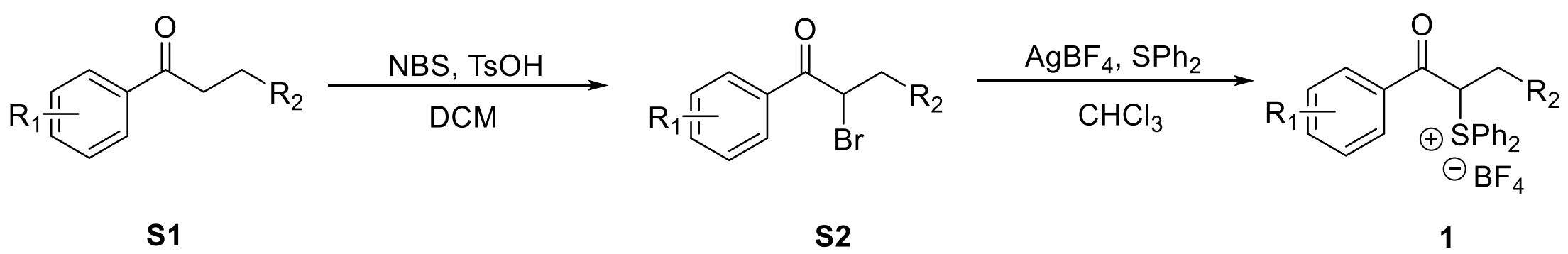

At 0 °C, to a solution of ketone **S1** (1.0 equiv) in DCM (0.2 M) was added *p*-

toluenesulfonic acid monohydrate (0.1 equiv). The mixture was stirred at room temperature for 5 min before *N*-bromosuccinimide (1.1 equiv) was added in small portions. Then, the mixture was heated with refluxing for 12 h before cooling to room temperature. Next, a saturated aqueous solution of $NaHCO_3$ (30 mL) was slowly added. The mixture was stirred at room temperature for 15 min before the organic layer was separated. The aqueous layerh was extracted with DCM (40 mL × 3). The combined organic layers were dried over anhydrous Na_2SO_4, filtered, and concentrated. The crude mixture was purified by flash silica gel column chromatography to give the α-bromo ketone **S2**.

To an oven-dried 100-mL sealed tube were added the α-bromo ketone **S2** (1.0 eqiuv), diphenyl sulfide (2.0 equiv), and dry $CHCl_3$ (0.5 M). Then, $AgBF_4$ (1.0 equiv) was added quickly before the tube was carefully sealed. The reaction mixture was kept stirring at 85 °C for 12 h. Next, the mixture was cooled to room temperature, filtered through a short pad of celite, and washed with DCM (100 mL). The filtrate was concentrated and purified by flash column chromatography on silica gel to afford the sulfonium salt **1**.

General Procedure C

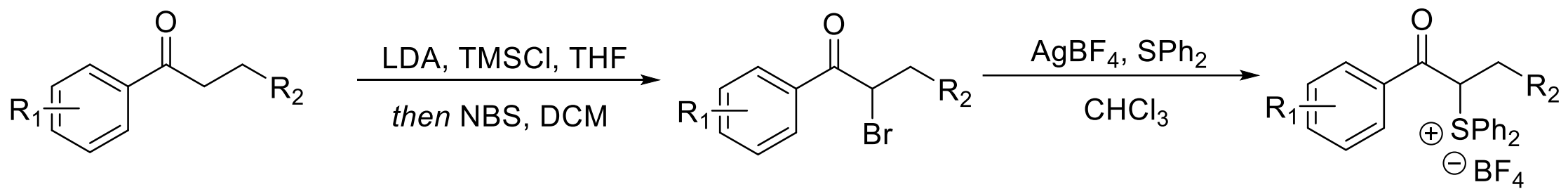

S1 **S2** **1**

At –78 °C, to a solution of LDA (1.3 equiv) in THF (1.5 mL) was slowly added a solution of **S1** (1.0 equiv) in THF (3.0 mL). The solution was stirred at the same temperature for 30 min before trimethylsilyl chloride (2.0 equiv) was added dropwise. After stirring at room temperature for 2 h, the solvent was removed under vacuum. Then, anhydrous DCM (9.0 mL) and *N*-bromosuccinimide (1.0 equiv, portionwise) were added. The mixture was

stirred at room temperature for another 16 h before quenching by water (10 mL). The organic layer was separated, and the aqueous layer was extracted with DCM (15 mL × 3). The combined organic layers were dried over anhydrous Na_2SO_4, filtered, and concentrated. The crude mixture was purified by flash silica gel column chromatography to give the α-bromo ketone **S2**.

To an oven-dried 100-mL sealed tube were added the α-bromo ketone **S2** (1.0 eqiuv), diphenyl sulfide (2.0 equiv), and dry $CHCl_3$ (0.5 M). Then, $AgBF_4$ (1.0 equiv) was added quickly before the tube was carefully sealed. The reaction mixture was kept stirring at 85 °C for 12 h. Next, the mixture was cooled to room temperature, filtered through a short pad of celite, and washed with DCM (100 mL). The filtrate was concentrated and purified by flash column chromatography on silica gel to afford the sulfonium salt **1**.

General Procedure D

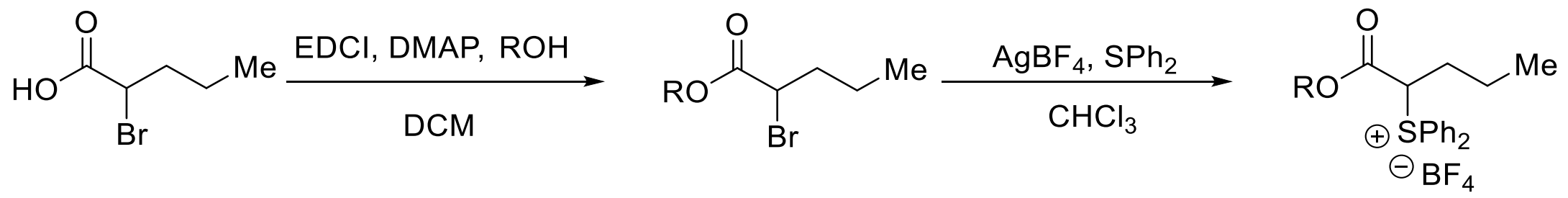

S3 **S4** **4**

At room temperature, to a solution of 2-bromopentanoic acid (1.0 equiv) in DCM (0.55 M) were added alcohol ROH (1.0 equiv), EDCI (1.16 equiv), and DMAP (0.26 equiv). The solution was stirred at the same temperature for 12 h before quenching by water (20 mL). The organic layer was separated, and the aqueous layer was extracted with DCM (20 mL × 3). The combined organic layers were dried over anhydrous Na_2SO_4, filtered, and concentrated. The crude mixture was purified by flash silica gel column chromatography to give the α-bromo ester **S4**.

To an oven-dried 100-mL sealed tube were added the α-bromo ketone **S4**

(1.0 eqiuv), diphenyl sulfide (2.0 equiv), and dry $CHCl_3$ (0.5 M). Then, $AgBF_4$ (1.0 equiv) was added quickly before the tube was carefully sealed. The reaction mixture was kept stirring at 85 °C for 24 h. Next, the mixture was cooled to room temperature, filtered through a short pad of celite, and washed with DCM (100 mL). The filtrate was concentrated and purified by flash column chromatography on silica gel to afford the sulfonium salt **4**.

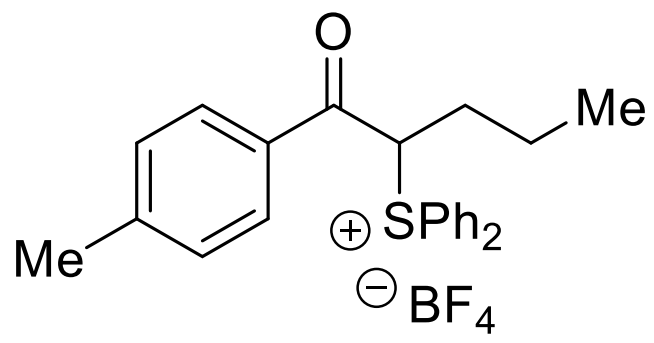

1a

(1-Oxo-1-(*p*-tolyl)pentan-2-yl)diphenylsulfonium tetrafluoroborate (1a) was prepared as a yellow solid from 2-bromo-1-(*p*-tolyl)pentan-1-one (4.2 g, 16.7 mmol), diphenyl sulfide (6.2 g, 33.4 mmol), and $AgBF_4$ (3.3 g, 16.7 mmol) according to the General Procedure A (eluent: hexanes/ethyl acetate = 10:1 → 0.5:1) in 82% yield.

^{1}H NMR (400 MHz, $CDCl_3$) δ 8.28 – 8.25 (m, 2H), 8.11 – 8.07 (m, 4H), 7.76 – 7.72 (m, 3H), 7.55 – 7.49 (m, 3H), 7.32 (d, J = 8.1 Hz, 2H), 7.04 – 7.02 (m, 1H), 2.35 (s, 3H), 2.30 – 2.21 (m, 1H), 1.97 – 1.87 (m, 1H), 1.44 – 1.28 (m, 2H), 0.72 (t, J = 7.3 Hz, 3H).

^{13}C NMR (100 MHz, $CDCl_3$) δ 192.8, 147.2, 135.2, 134.4, 132.1, 131.9, 131.4, 130.7, 130.4, 130.2, 129.6, 124.9, 122.0, 68.0, 31.3, 21.8, 18.3, 13.7.

HRMS (ES+) Calcd for $C_{24}H_{25}OS$ $[M-BF_4]^+$: 361.1621, found: 361.1632.

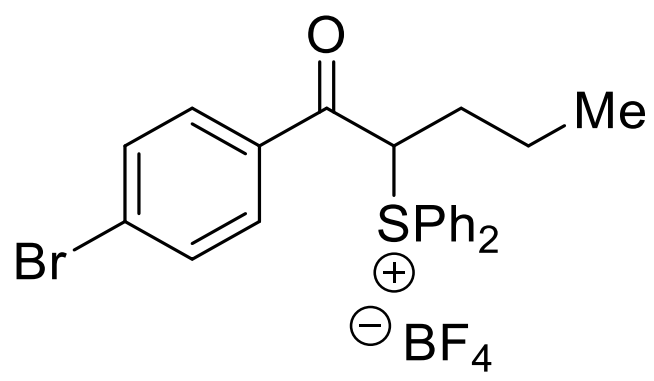

1b

(1-(4-Bromophenyl)-1-oxopentan-2-yl)diphenylsulfonium tetrafluoroborate

(1b) was prepared as a red solid from 2-bromo-1-(4-bromophenyl)pentan-1-one (5.3 g, 16.7 mmol), diphenyl sulfide (6.2 g, 33.4 mmol), and $AgBF_4$ (3.3 g, 16.7 mmol) according to the General Procedure B (eluent: hexanes/ethyl acetate = 10:1 → 0.5:1) in 49% yield.

^{1}H NMR (400 MHz, $CDCl_3$) δ 8.26 – 8.34 (m, 2H), 8.11 – 8.06 (m, 4H), 7.79 – 7.76 (m, 3H), 7.72 – 7.69 (m, 2H), 7.61 – 7.56 (m, 3H), 7.08 – 7.05 (m, 1H), 2.30 – 2.21 (m, 1H), 1.98 – 1.89 (m, 1H), 1.40 – 1.32 (m, 2H), 0.76 (t, J = 7.3 Hz, 3H).

^{13}C NMR (100 MHz, $CDCl_3$) δ 192.9, 135.4, 134.7, 133.0, 132.1, 132.0, 131.8, 131.7, 131.6, 131.0, 130.5, 124.7, 121.7, 68.0, 31.3, 18.4, 13.7.

HRMS (ES+) Calcd for $C_{23}H_{22}BrOS$ $[M-BF_4]^+$: 425.0569, found: 425.0581.

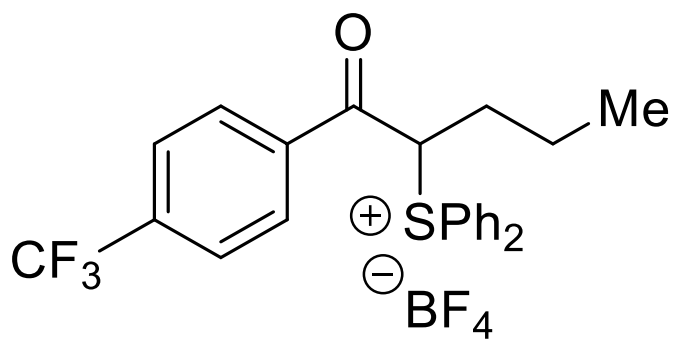

1c

(1-Oxo-1-(4-(trifluoromethyl)phenyl)pentan-2-yl)diphenylsulfonium tetrafluoroborate (1c) was prepared as a yellow solid from 2-bromo-1-(4-(trifluoromethyl)phenyl)pentan-1-one (0.9 g, 3.0 mmol), diphenyl sulfide (1.1 g, 6.0 mmol), and $AgBF_4$ (0.6 g, 3.0 mmol) according to the General Procedure B (eluent: hexanes/ethyl acetate = 10:1 → 0.5:1) in 45% yield.

^{1}H NMR (400 MHz, $CDCl_3$) δ 8.35 – 8.27 (m, 4H), 8.16 – 8.13 (m, 2H), 7.81 – 7.76 (m, 5H), 7.62 – 7.54 (m, 3H), 7.13 – 7.10 (m, 1H), 2.33 – 2.23 (m, 1H), 2.01 – 1.91 (m, 1H), 1.45 – 1.28 (m, 2H), 0.74 (t, J = 7.3 Hz, 3H).

^{13}C NMR (100 MHz, $CDCl_3$) δ 193.0, 136.3 (q, J = 32.9 Hz), 135.6, 135.4, 134.7, 132.1, 132.0, 131.6, 130.5, 129.9, 126.5 (q, J = 3.7 Hz), 124.6, 123.0 (q, J = 271.0 Hz), 121.6, 68.0, 31.1, 18.3, 13.7.

^{19}F NMR (376 MHz, $CDCl_3$) δ –63.5, –150.4, –150.5.

HRMS (ES+) Calcd for $C_{24}H_{22}F_3OS$ $[M-BF_4]^+$: 415.1338, found: 415.1349.

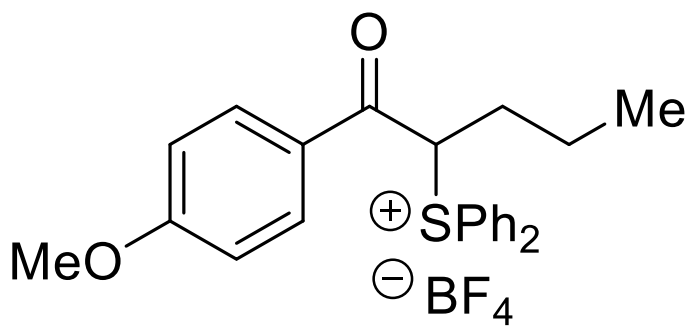

1d

(1-(4-Methoxyphenyl)-1-oxopentan-2-yl)diphenylsulfonium tetrafluoroborate (1d) was prepared as a yellow solid from 2-bromo-1-(4-methoxyphenyl)pentan-1-one (1.4 g, 5.0 mmol), diphenyl sulfide (1.9 g, 10.0 mmol), and $AgBF_4$ (1.0 g, 5.0 mmol) according to the General Procedure B (eluent: hexanes/ethyl acetate = 10:1 → 0.5:1) in 51% yield.

^{1}H NMR (400 MHz, $CDCl_3$) δ 8.25 – 8.18 (m, 4H), 8.09 – 8.07 (m, 2H), 7.78 – 7.76 (m, 3H), 7.58 – 7.53 (m, 3H), 7.05 – 7.00 (m, 3H), 3.87 (s, 3H), 2.29 – 2.20 (m, 1H), 1.98 – 1.88 (m, 1H), 1.41 – 1.35 (m, 2H), 0.76 (t, J = 7.3 Hz, 3H).

^{13}C NMR (100 MHz, $CDCl_3$) δ 191.4, 165.8, 132.3, 134.4, 132.4, 132.1, 131.9, 131.5, 130.5, 126.1, 124.9, 122.2, 114.9, 68.1, 55.8, 31.6, 18.5, 13.8.

HRMS (ES+) Calcd for $C_{24}H_{25}O_2S$ $[M-BF_4]^+$: 377.1570, found:377.1582.

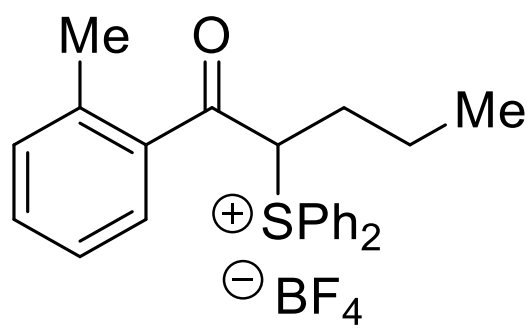

1e

(1-Oxo-1-(o-tolyl)pentan-2-yl)diphenylsulfonium tetrafluoroborate (1e) was prepared as a yellow solid from 2-bromo-1-(*o*-tolyl)pentan-1-one (1.3 g, 5.0 mmol), diphenyl sulfide (1.9 g, 10.0 mmol), and $AgBF_4$ (1.0 g, 5.0 mmol) according to the General Procedure A (eluent: hexanes/ethyl acetate = 10:1 → 0.5:1) in 43% yield.

^{1}H NMR (400 MHz, $CDCl_3$) δ 8.36 – 8.33 (m, 1H), 8.27 – 8.25 (m, 2H), 8.19 – 8.16 (m, 2H), 7.77 – 7.72 (m, 3H), 7.59 – 7.56 (m, 3H), 7.49 – 7.43 (m, 2H), 7.31 – 7.22 (m, 1H), 7.09 – 7.07 (m, 1H), 2.40 (s, 3H), 2.27 – 2.18 (m, 1H), 1.89 – 1.80 (m, 1H), 1.45 – 1.21 (m, 2H), 0.71 (t, J = 7.3 Hz, 3H).

^{13}C NMR (100 MHz, $CDCl_3$) δ 195.4, 140.3, 135.1, 134.3, 134.2, 132.4, 132.1, 131.9, 131.44, 131.38, 130.4, 129.1, 127.4, 125.3, 122.1, 70.2, 31.0, 21.5, 18.1, 13.7.

HRMS (ES+) Calcd for $C_{24}H_{25}OS$ $[M-BF_4]^+$: 361.1621, found: 361.1632.

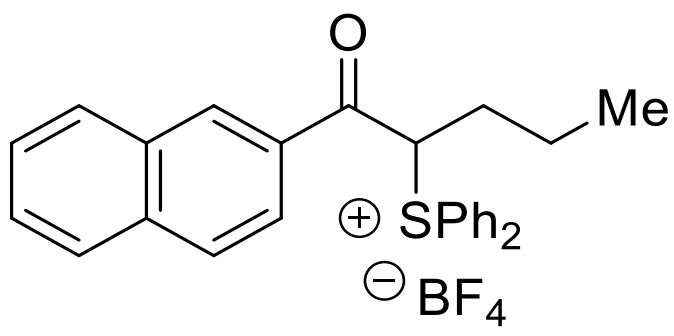

1f

(1-(Naphthalen-2-yl)-1-oxopentan-2-yl)diphenylsulfonium tetrafluoroborate (1f) was prepared as a red solid from 2-bromo-1-(naphthalen-2-yl)pentan-1-one (1.5 g, 5.0 mmol), diphenyl sulfide (1.9 g, 10.0 mmol, 2.0 equiv), and $AgBF_4$ (1.0 g, 5.0 mmol) according to the General Procedure B (eluent: hexanes/ethyl acetate = 10:1 → 0.5:1) in 66% yield.

^{1}H NMR (400 MHz, $CDCl_3$) δ 9.13 (s, 1H), 8.42 (d, J = 8.0 Hz, 1H), 8.32 – 8.30 (m, 2H), 8.16 – 8.14 (m, 2H), 7.94 – 7.78 (m, 6H), 7.66 – 7.53 (m, 5H), 7.34 – 7.31 (m, 1H), 2.42 – 2.33 (m, 1H), 2.05 – 1.95 (m, 1H), 1.46 – 1.36 (m, 2H), 0.74 (t, J = 7.3, 3H).

^{13}C NMR (100 MHz, $CDCl_3$) δ 193.4, 136.5, 135.4, 134.5, 133.8, 132.8, 132.2, 132.0, 131.5, 131.4, 130.5, 130.3, 130.2, 129.2, 127.5, 127.3, 124.9, 122.9, 122.0, 68.3, 31.7, 18.5, 13.7.

HRMS (ES+) Calcd for $C_{27}H_{25}OS$ $[M-BF_4]^+$: 397.1621, found: 397.1631.

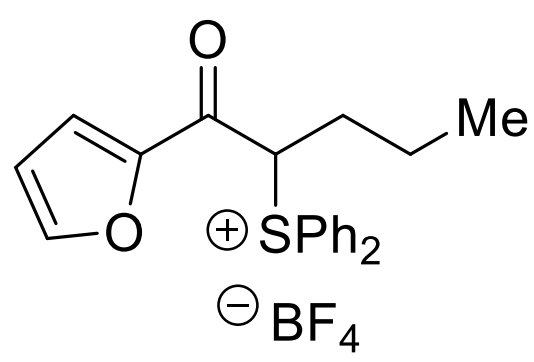

1g

(1-(Furan-2-yl)-1-oxopentan-2-yl)diphenylsulfonium tetrafluoroborate (1g) was prepared as a white solid from 2-bromo-1-(furan-2-yl)pentan-1-one (1.1 g, 5.0 mmol, 1.0 equiv), diphenyl sulfide (1.9 g, 10.0 mmol, 2.0 equiv), and $AgBF_4$

(1.0 g, 5.0 mmol) according to the General Procedure B (eluent: hexanes/ethyl acetate = 10:1 → 0.5:1) in 47% yield.

^{1}H NMR (400 MHz, $CDCl_3$) δ 8.26 – 8.23 (m, 2H), 8.07 – 8.02 (m, 3H), 7.78 – 7.71 (m, 4H), 7.60 – 7.53 (m, 3H), 6.64 – 6.61 (m, 2H), 2.16 – 1.94 (m, 2H), 1.54 – 1.33 (m, 2H), 0.79 (t, *J* = 7.3 Hz, 3H).

^{13}C NMR (100 MHz, $CDCl_3$) δ 179.5, 150.4, 149.5, 135.4, 134.8, 132.0, 131.5, 130.9, 130.8, 125.0, 124.0, 121.5, 113.9, 66.0, 31.9, 18.8, 13.7.

HRMS (ES+) Calcd for $C_{21}H_{21}O_2S$ $[M{-}BF_4]^+$: 337.1257, found: 337.1269.

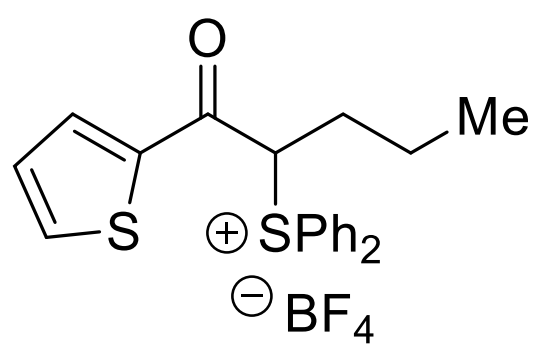

1h

(1-Oxo-1-(thiophen-2-yl)pentan-2-yl)diphenylsulfonium tetrafluoroborate (1h) was prepared as a white solid from 2-bromo-1-(thiophen-2-yl)pentan-1-one (2.5 g, 10.0 mmol, 1.0 equiv), diphenyl sulfide (3.7 g, 20.0 mmol, 2.0 equiv), and $AgBF_4$ (2.0 g, 10.0 mmol) according to the General Procedure B (eluent: hexanes/ethyl acetate = 10:1 → 0.5:1) in 41% yield.

^{1}H NMR (400 MHz, $CDCl_3$) δ 8.47 – 8.46 (m, 1H), 8.26 – 8.24 (m, 2H), 8.07 – 8.05 (m, 2H), 7.79 – 7.73 (m, 4H), 7.57 – 7.50 (m, 3H), 7.21 – 7.19 (m, 1H), 6.80 – 6.78 (m, 1H), 2.25 – 2.03 (m, 1H), 2.01 – 1.93 (m, 1H), 1.49 – 1.38 (m, 2H), 0.76 (t, *J* = 7.3, 3H).

^{13}C NMR (100 MHz, $CDCl_3$) δ 185.2, 140.1, 138.3, 137.6, 135.3, 134.6, 132.0, 131.9, 131.4, 130.6, 130.1, 124.2, 121.7, 67.0, 32.0, 18.7, 13.6.

HRMS (ES+) Calcd for $C_{21}H_{21}OS_2$ $[M{-}BF_4]^+$: 353.1028, found: 353.1039.

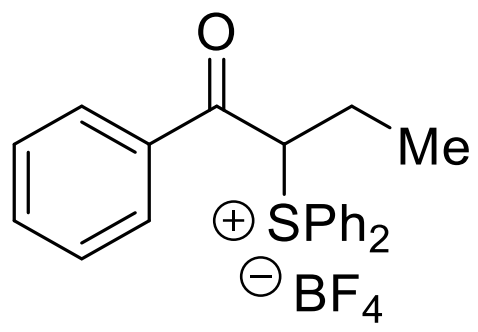

1i

(1-Oxo-1-phenylbutan-2-yl)diphenylsulfonium tetrafluoroborate (1i) was prepared as a yellow solid from 2-bromo-1-phenylbutan-1-one (3.8 g, 16.7 mmol, 1.0 equiv), diphenyl sulfide (6.2 g, 33.4 mmol), and $AgBF_4$ (3.3 g, 16.7 mmol) according to the General Procedure B (eluent: hexanes/ethyl acetate = 10:1 → 0.5:1) in 21% yield.

^{1}H NMR (400 MHz, $CDCl_3$) δ 8.29 – 8.26 (m, 2H), 8.19 – 8.17 (m, 2H), 8.14 – 8.11 (m, 2H), 7.76 – 7.74 (m, 3H), 7.65 – 7.51 (m, 6H), 7.11 – 7.09 (m, 1H), 2.47 – 2.37 (m, 1H), 2.09 – 1.98 (m, 1H), 0.94 (t, J = 7.5 Hz, 3H).

^{13}C NMR (100 MHz, $CDCl_3$) δ 193.4, 135.6, 135.3, 134.5, 133.0, 132.1, 131.9, 131.5, 130.5, 129.5 (2C), 124.8, 121.8, 69.1, 22.9, 8.9.

HRMS (ES+) Calcd for $C_{22}H_{21}OS$ $[M-BF_4]^+$: 333.1308, found: 333.1317.

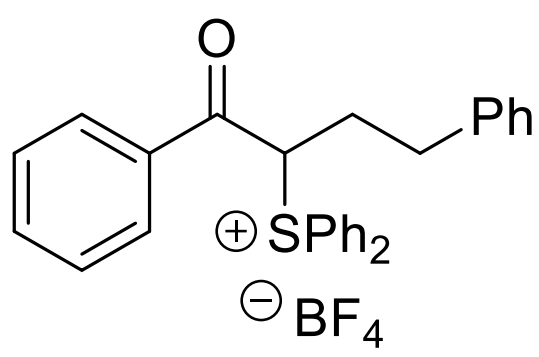

1j

(1-Oxo-1,4-diphenylbutan-2-yl)diphenylsulfonium tetrafluoroborate (1j) was prepared as a white solid from 2-bromo-1,4-diphenylbutan-1-one (5.0 g, 16.5 mmol), diphenyl sulfide (6.1 g, 33.0 mmol), and $AgBF_4$ (3.2 g, 16.5 mmol) according to the General Procedure B (eluent: DCM/MeOH = 50:1, 4.3 g, 52% yield).

^{1}H NMR (400 MHz, $CDCl_3$) δ 8.24 – 8.20 (m, 4H), 8.15 – 8.12 (m, 2H), 7.73 – 7.70 (m, 3H), 7.65 – 7.61 (m, 1H), 7.57 – 7.50 (m, 5H), 7.17 – 7.12 (m, 4H), 6.90 – 6.88 (m, 2H), 2.73 – 2.64 (m, 3H), 2.39 – 2.26 (m, 1H).

^{13}C NMR (100 MHz, $CDCl_3$) δ 193.4, 138.4, 135.7, 135.3, 134.6, 133.2, 132.2, 131.9,

131.5, 130.5, 129.62, 129.55, 128.7, 128.5, 126.9, 125.1, 121.9, 67.7, 31.0, 30.8.

HRMS (CI+) Calcd for $C_{28}H_{24}OS$ [M–HBF$_4$]$^+$: 408.1548, found: 408.1560.

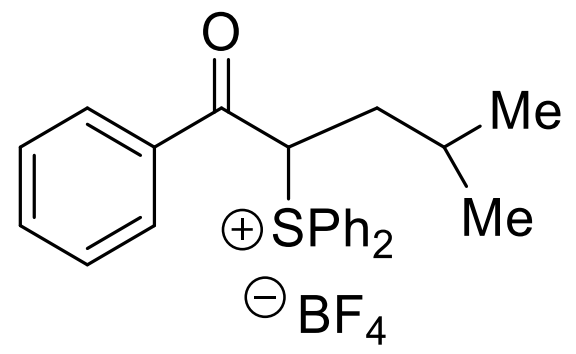

1k

(4-Methyl-1-oxo-1-phenylpentan-2-yl)diphenylsulfonium tetrafluoroborate (1k) was prepared as a yellow solid from 2-bromo-4-methyl-1-phenylpentan-1-one (1.2 g, 4.7 mmol), diphenyl sulfide (1.6 mL, 9.4 mmol), and $AgBF_4$ (0.9 g, 4.7 mmol) according to the General Procedure B (eluent: hexanes/ethyl acetate = 10:1 → 0.5:1) in 38% yield.

¹H NMR (400 MHz, $CDCl_3$) *δ* 8.32 – 8.29 (m, 2H), 8.17 – 8.12 (m, 4H), 7.77 – 7.75 (m, 3H), 7.63 – 7.59 (m, 1H), 7.54 – 7.50 (m, 5H), 6.97 – 6.94 (m, 1H), 2.15 – 2.09 (m, 1H), 2.03 – 1.96 (m, 1H), 1.66 – 1.56 (m, 1H), 0.80 – 0.76 (m, 6H).

¹³C NMR (100 MHz, $CDCl_3$) *δ* 194.1, 135.5, 135.3, 134.5, 133.7, 132.2, 131.9, 131.5, 130.5, 129.5, 129.4, 124.8, 122.1, 66.4, 38.4, 25.6, 23.3, 22.1.

HRMS (ES+) Calcd for $C_{24}H_{25}OS$ [M–BF$_4$]$^+$: 361.1621, found: 361.163.

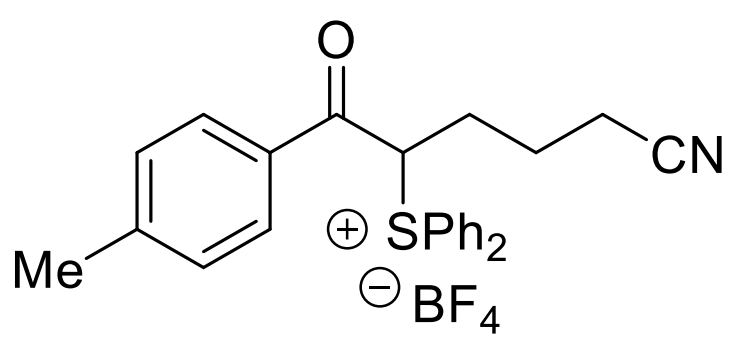

1l

(4-Cyano-1-oxo-1-(*p*-tolyl)butan-2-yl)diphenylsulfonium tetrafluoroborate (1l) was prepared as a light yellow viscous oil from 5-bromo-6-oxo-6-(*p*-tolyl)hexanenitrile (1.2 g, 4.3 mmol), diphenyl sulfide (1.6 g, 8.6 mmol), and $AgBF_4$ (0.8 g, 4.3 mmol) according to the General Procedure B (eluent: DCM/MeOH = 50:1, 1.1 g, 55% yield).

¹H NMR (400 MHz, $CDCl_3$) δ 8.32 – 8.29 (m, 2H), 8.11 – 8.09 (m, 4H), 8.81 – 7.79

(m, 3H), 7.65 – 7.56 (m, 3H), 7.37 (d, J = 8.2 Hz, 2H), 7.04 (dd, J = 5.7, 3.8 Hz, 1H), 2.45 – 2.13 (m, 7H), 1.78 – 1.71 (m, 2H).

^{13}C NMR (100 MHz, $CDCl_3$) δ 192.4, 147.9, 135.7, 134.8, 132.3, 132.2, 131.7, 130.8, 130.6, 130.5, 129.8, 124.4, 121.4, 118.2, 66.2, 28.7, 21.9, 21.4, 16.8.

HRMS (CI+) Calcd for $C_{25}H_{23}NOS$ $[M–HBF_4]^+$: 385.1500, found: 385.1514.

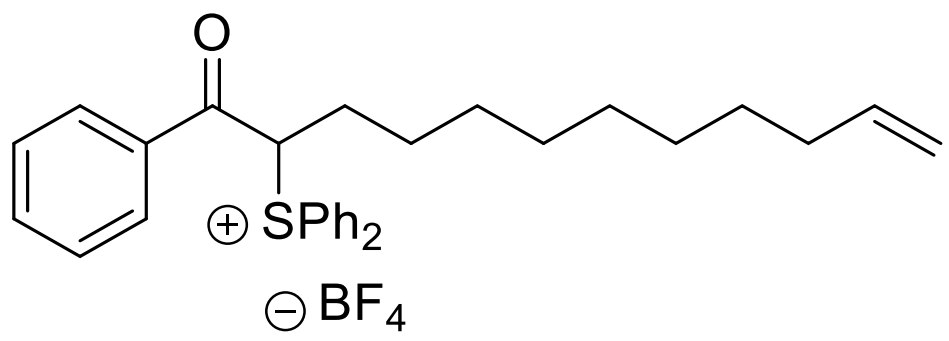

1m

(1-Oxo-1-phenyldodec-11-en-2-yl)diphenylsulfonium tetrafluoroborate (1m) was prepared as a light green oil from 2-bromo-1-phenyldodec-11-en-1-one (1.5 g, 4.5 mmol), diphenyl sulfide (1.6 g, 9.0 mmol), and $AgBF_4$ (0.88 g, 4.5 mmol) according to the General Procedure C (eluent: hexanes/ethyl acetate = 10:1 → 0.5:1) in 49% yield.

^{1}H NMR (400 MHz, $CDCl_3$) *δ* 8.29 – 8.26 (m, 2H), 8.18 – 8.11 (m, 4H), 7.77 – 7.72 (m, 3H), 7.63 – 7.59 (m, 1H), 7.56 – 7.49 (m, 5H), 7.09 – 7.06 (m, 1H), 5.77 – 5.67 (m, 1H), 4.94 – 4.85 (m, 2H), 2.36 – 2.27 (m, 1H), 1.99 – 1.90 (m, 3H), 1.58 – 1.02 (m, 12H).

^{13}C NMR (100 MHz, $CDCl_3$) *δ* 193.4, 138.9, 135.5, 135.2, 134.4, 133.0, 132.1, 131.8, 131.4, 130.4, 129.4 (2C), 124.8, 121.9, 114.1, 68.3, 33.5, 29.0, 28.8, 28.75, 28.65, 28.6, 28.5, 24.3.

HRMS (ES+) Calcd for $C_{30}H_{35}OS$ $[M–BF_4]^+$: 443.2403, found: 443.2406.

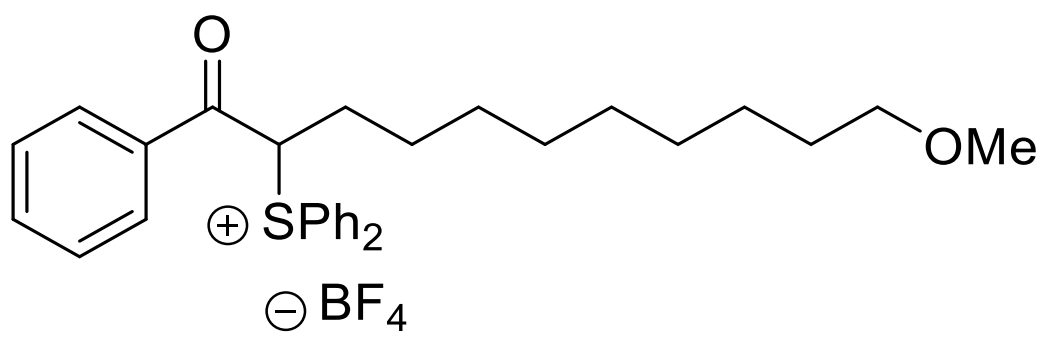

1n

(11-Methoxy-1-oxo-1-phenylundecan-2-yl)diphenylsulfonium

tetrafluoroborate (1n) was prepared as a brown oil from 2-bromo-11-methoxy-1-phenylundecan-1-one (0.5 g, 1.4 mmol), diphenyl sulfide (0.5 g, 2.8 mmol), and $AgBF_4$ (0.3 g, 1.4 mmol) according to the General Procedure C (eluent: hexanes/ethyl acetate = 10:1 → 0.5:1) in 65% yield.

^{1}H NMR (400 MHz, $CDCl_3$) δ 8.26 – 8.24 (m, 2H), 8.20 – 8.17 (m, 2H), 8.13 – 8.11 (m, 2H), 7.78 – 7.76 (m, 3H), 7.67 – 7.63 (m, 1H), 7.60 – 7.54 (m, 5H), 7.16 – 7.13 (m, 1H), 3.36 – 3.30 (m, 5H), 2.34 – 2.29 (m, 1H), 1.99 – 1.90 (m, 1H), 1.51 – 1.44 (m, 2H), 1.31 – 1.04 (m, 12H).

^{13}C NMR (100 MHz, $CDCl_3$) δ 193.7, 135.7, 135.3, 134.5, 132.9, 132.2, 132.0, 131.6, 130.5, 129.58, 129.56, 124.8, 121.8, 72.8, 68.8, 58.5, 29.5, 29.15, 29.06, 29.0, 28.8, 28.6, 25.9, 24.4.

HRMS (ES+) Calcd for $C_{30}H_{37}O_2S$ $[M-BF_4]^+$: 461.2509, found: 461.2510.

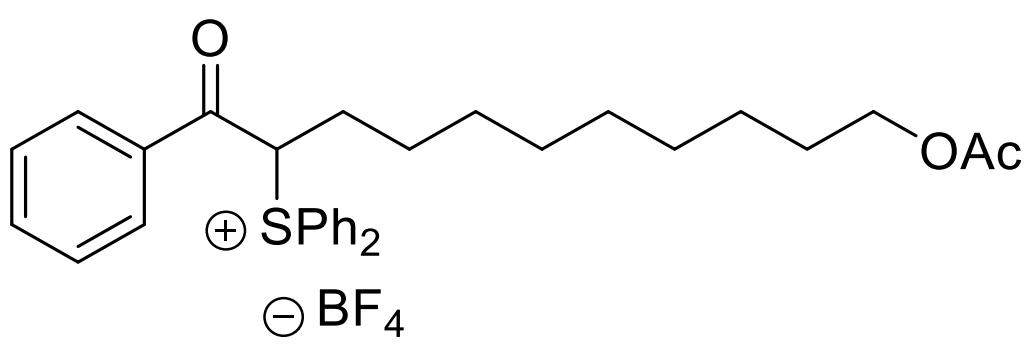

1o

(11-Acetoxy-1-oxo-1-phenylundecan-2-yl)diphenylsulfonium tetrafluoroborate (1o) was prepared as a brown oil from 10-bromo-11-oxo-11-phenylundecyl acetate (0.9 mg, 2.4 mmol) , diphenyl sulfide (0.9 g, 4.7 mmol), and $AgBF_4$ (0.4 g, 2.4 mmol) according to the General Procedure A (eluent: hexanes/ethyl acetate = 10:1 → 0.5:1) in 30% yield.

^{1}H NMR (400 MHz, $CDCl_3$) δ 8.27 – 8.25 (m, 2H), 8.19 – 8.10 (m, 4H), 7.77 – 7.76 (m, 3H), 7.66 – 7.63 (m, 1H), 7.57 – 7.53 (m, 5H), 7.14 – 7.12 (m, 1H), 3.99 (t, J = 6.8 Hz, 2H), 2.33 – 2.29 (m, 1H), 2.01 (s, 3H), 1.98 – 1.91 (m, 1H), 1.56 – 1.49 (m, 2H), 1.35 – 1.04 (m, 12H).

^{13}C NMR (100 MHz, $CDCl_3$) δ 193.6, 171.2, 135.7, 135.3, 134.5, 133.0, 132.1, 131.9, 131.5, 130.5, 129.5 (2C), 124.8, 121.9, 68.6, 64.4, 29.1, 28.92, 28.89, 28.8, 28.6, 28.4, 25.7, 24.4, 21.0.

HRMS (ES+) Calcd for $C_{31}H_{37}O_3S$ $[M-BF_4]^+$: 489.2458, found: 489.2458.

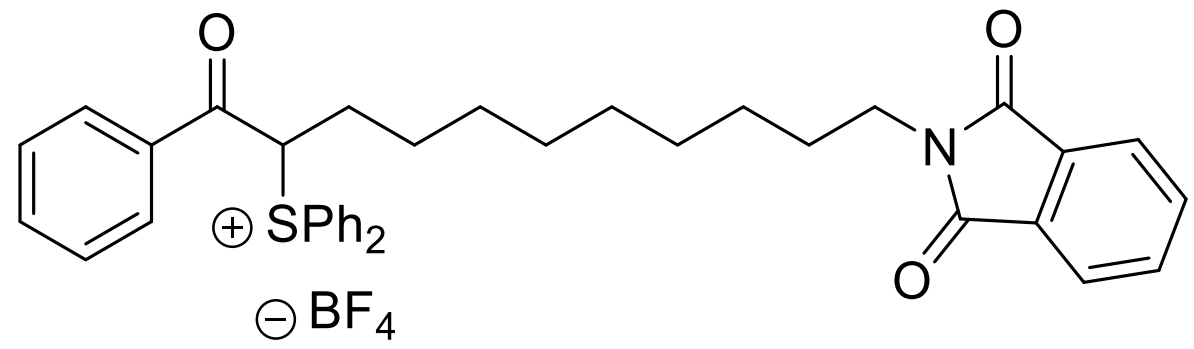

1p

(11-(1,3-Dioxoisoindolin-2-yl)-1-oxo-1-phenylundecan-2-yl)diphenylsulfonium tetrafluoroborate (1p) was prepared as a light yellow solid from 2-(10-bromo-11-oxo-11-phenylundecyl)isoindoline-1,3-dione (1.4 g, 3.0 mmol) , diphenyl sulfide (1.1 g, 6.0 mmol), and $AgBF_4$ (0.6 g, 3.0 mmol) according to the General Procedure A (eluent: hexanes/ethyl acetate = 10:1 → 0.5:1) in 55% yield.
^{1}H NMR (400 MHz, $CDCl_3$) δ 8.27 – 8.22 (m, 2H), 8.18 – 8.16 (m, 2H), 8.12 – 8.10 (m, 2H), 7.85 – 7.79 (m, 2H), 7.77 – 7.73 (m, 3H), 7.72 – 7.67 (m, 2H), 7.66 – 7.62 (m, 1H), 7.60 – 7.52 (m, 5H), 7.14 – 7.12 (m, 1H), 3.61 (t, J = 7.3 Hz, 2H), 2.37 – 2.28 (m, 1H), 1.97 – 1.88 (m, 1H), 1.61 – 1.54 (m, 2H), 1.33 – 1.02 (m, 12H).
^{13}C NMR (100 MHz, $CDCl_3$) δ 193.6, 168.4, 135.7, 135.3, 134.5, 133.9, 132.9, 132.2, 132.04, 131.96, 131.5, 130.5, 129.6, 129.5, 124.8, 123.1, 121.8, 68.8, 37.9, 29.0, 28.9, 28.80, 28.78, 28.6, 28.4, 26.6, 24.3.
HRMS (ES+) Calcd for $C_{37}H_{38}NO_3S$ $[M-BF_4]^+$: 576.2567, found: 576.2571.

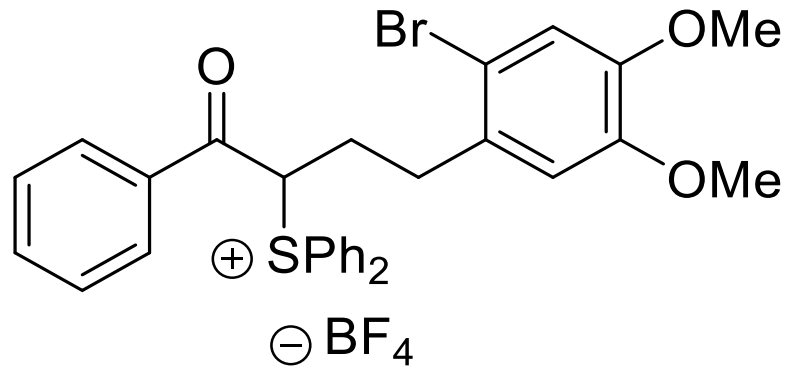

1q

(4-(2-Bromo-4,5-dimethoxyphenyl)-1-oxo-1-phenylbutan-2-yl)diphenylsulfonium tetrafluoroborate (1q) was prepared as a yellow solid from 2-bromo-4-(2-bromo-4,5-dimethoxyphenyl)-1-phenylbutan-1-one (1.3 g, 2.8 mmol) , diphenyl sulfide (1.0 g, 5.6 mmol), and $AgBF_4$ (0.6 g, 2.8 mmol) according to the General Procedure A (eluent: hexanes/ethyl acetate = 10:1 →

0.5:1) in 38% yield.

^{1}H NMR (400 MHz, $CDCl_3$) δ 8.27 – 8.27 (m, 2H), 8.18 (d, *J* = 7.5 Hz, 2H), 8.12 – 8.10 (m, 2H), 7.75 – 7.73 (m, 3H), 7.65 – 7.61 (m, 1H), 7.56 – 7.51 (m, 5H), 7.18 – 7.16 (m, 1H), 6.79 (s, 1H), 6.46 (s, 1H), 3.76 (s, 3H), 3.69 (s, 3H), 2.75 – 2.54 (m, 3H), 2.41 – 2.32 (m, 1H).

^{13}C NMR (100 MHz, $CDCl_3$) δ 193.3, 148.5, 148.4, 135.6, 135.3, 134.5, 133.3, 132.3, 131.9, 131.5, 130.4, 129.53, 129.47, 129.4, 125.0, 121.8, 115.3, 113.7, 113.2, 67.5, 56.1, 56.0, 31.3, 29.6.

HRMS (ES+) Calcd for $C_{30}H_{28}BrO_3S$ $[M–BF_4]^+$: 547.0937, found: 547.0947.

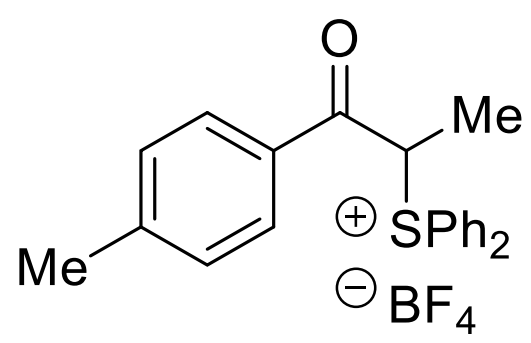

1r

(1-Oxo-1-(*p*-tolyl)propan-2-yl)diphenylsulfonium tetrafluoroborate (1r) was prepared as a colorless solid from 2-bromo-1-(*p*-tolyl)propan-1-one (2.0 g, 8.8 mmol), diphenyl sulfide (3.3 g, 17.6 mmol), and $AgBF_4$ (1.7 g, 8.8 mmol) in dry DCE (34 mL) according to the General Procedure A (24 h, eluent: DCM/MeOH = 50:1) in 57% yield.

^{1}H NMR (400 MHz, $CDCl_3$) δ 8.27 – 8.24 (m, 2H), 8.12 – 8.08 (m, 4H), 7.78 – 7.74 (m, 3H), 7.61 – 7.55 (m, 3H), 7.35 (d, *J* = 8.1 Hz, 2H), 7.02 (q, *J* = 7.2 Hz, 1H), 2.39 (s, 3H), 1.77 (d, *J* = 7.2 Hz, 3H).

^{13}C NMR (100 MHz, $CDCl_3$) δ 192.6, 146.7, 134.6, 133.9, 131.6, 131.3, 130.9, 129.8, 129.7, 129.3, 128.9, 124.5, 121.4, 63.1, 21.3, 15.7.

HRMS (CI+) Calcd for $C_{22}H_{20}OS$ $[M–HBF_4]^+$: 332.1235, found: 332.1231.

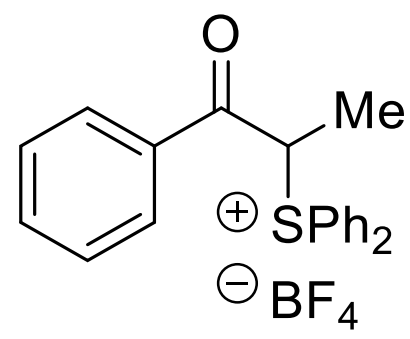

1s

(1-Oxo-1-phenylpropan-2-yl)diphenylsulfonium tetrafluoroborate (1s) was prepared as a colorless solid from 2-bromo-1-phenylpropan-1-one (2.8 g, 12.9 mmol) , diphenyl sulfide (4.8 g, 25.8 mmol), and $AgBF_4$ (2.5 g, 12.9 mmol) in dry DCE (50 mL) according to the General Procedure A (24 h, eluent: DCM/MeOH = 50:1) in 88% yield.

^{1}H NMR (400 MHz, $CDCl_3$) δ 8.31 – 8.23 (m, 2H), 8.23 – 8.17 (m, 2H), 8.15 – 8.09 (m, 2H), 7.81 – 7.73 (m, 3H), 7.70 – 7.52 (m, 6H), 7.08 (q, *J* = 7.2 Hz, 1H), 1.78 (d, *J* = 7.2 Hz, 3H).

^{13}C NMR (100 MHz, $CDCl_3$) δ 193.3, 135.2, 134.7, 134.0, 131.7, 131.39, 131.35, 131.0, 129.8, 129.2, 129.0, 124.4, 121.2, 63.3, 15.7.

HRMS (CI+) Calcd for $C_{21}H_{18}OS$ $[M–HBF_4]^+$: 318.1078, found: 318.1080.

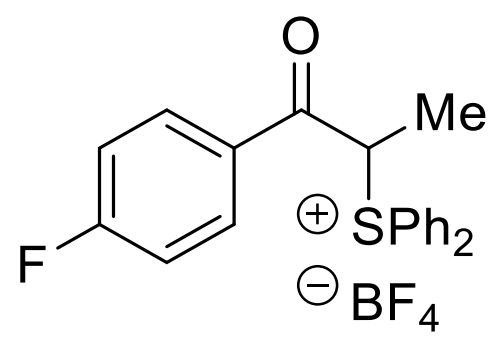

1t

(1-(4-Fluorophenyl)-1-oxopropan-2-yl)diphenylsulfonium tetrafluoroborate (1t) was prepared as a white solid from 2-bromo-1-(4-fluorophenyl)propan-1-one (2.4 g, 10.4 mmol), diphenyl sulfide (3.9 g, 20.8 mmol), and $AgBF_4$ (2.0 g, 10.4 mmol) in dry DCE (40 mL) according to the General Procedure A (12 h, eluent: DCM/MeOH = 50:1) in 55% yield.

^{1}H NMR (400 MHz, $CDCl_3$) δ 8.29 – 8.25 (m, 4H), 8.13 – 8.11 (m, 2H), 7.79 – 7.74 (m, 3H), 7.66 – 7.57 (m, 3H), 7.24 – 7.20 (m, 2H), 7.01 (q, *J* = 7.2 Hz, 1H), 1.78 (d, *J* = 7.2 Hz, 3H).

^{13}C NMR (100 MHz, $CDCl_3$) δ 191.6, 166.6 (d, *J* = 258.2 Hz), 134.7, 134.0, 132.3

(d, J = 10.0 Hz), 131.6, 131.3, 131.0, 129.8, 127.9 (d, J = 2.8 Hz), 124.4, 121.2, 116.3 (d, J = 22.0 Hz), 62.8, 15.6.

^{19}F NMR (376 MHz, $CDCl_3$) δ –99.5, –150.71, –150.77.

HRMS (CI+) Calcd for $C_{21}H_{17}FOS$ [M–HBF_4]$^+$: 336.0984, found: 336.0984.

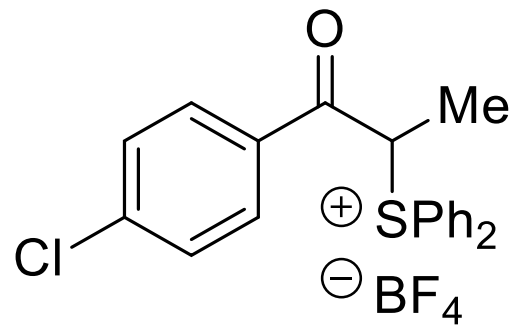

1u

(1-(4-Chlorophenyl)-1-oxopropan-2-yl)diphenylsulfonium tetrafluoroborate (1u) was prepared as a white solid from 2-bromo-1-(4-chlorophenyl)propan-1-one (2.5 g, 10.0 mmol), diphenyl sulfide (3.7 g, 20.0 mmol), and $AgBF_4$ (1.9 g, 10.0 mmol) in dry DCE (40 mL) according to the General Procedure A (12 h, eluent: DCM/MeOH = 50:1) in 64% yield.

^{1}H NMR (400 MHz, $CDCl_3$) δ 8.26 – 8.24 (m, 2H), 8.16 – 8.09 (m, 4H), 7.76 – 7.75 (m, 3H), 7.64 – 7.57 (m, 3H), 7.52 – 7.48 (m, 2H), 6.99 (q, J = 7.2 Hz, 1H), 1.76 (d, J = 7.0 Hz, 3H).

^{13}C NMR (100 MHz, $CDCl_3$) δ 192.7, 142.6, 135.3, 134.6, 132.2, 131.9, 131.6, 131.1, 130.4 (2C), 129.9, 124.9, 121.7, 63.4, 16.1.

HRMS (ES+) Calcd for $C_{21}H_{18}ClOS$ [M–BF_4]$^+$: 353.0761, found: 353.0766.

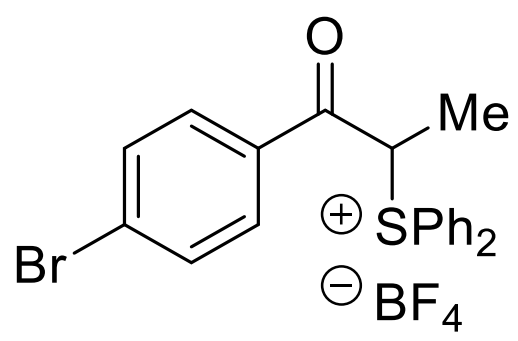

1v

(1-(4-Bromophenyl)-1-oxopropan-2-yl)diphenylsulfonium tetrafluoroborate (1v) was prepared as a colorless solid from 2-bromo-1-(4-bromophenyl)-propan-1-one (2.3 g, 8.0 mmol), diphenyl sulfide (3.0 g, 16.0 mmol), and $AgBF_4$ (1.6 g, 8.0 mmol) in dry DCE (31 mL) according to the General Procedure A (12h,

eluent: DCM/MeOH = 50:1) in 72% yield.

^{1}H NMR (400 MHz, $CDCl_3$) δ 8.26 – 8.23 (m, 2H), 8.12 – 8.06 (m, 4H), 7.80 – 7.76 (m, 3H), 7.75 – 7.70 (m, 2H), 7.67 – 7.59 (m, 3H), 7.04 (q, *J* = 7.2 Hz, 1H), 1.76 (d, *J* = 7.2 Hz, 3H).

^{13}C NMR (100 MHz, $CDCl_3$) δ 192.5, 134.8, 134.1, 132.4, 131.6, 131.4, 131.2, 131.1, 130.6, 130.1, 129.8, 124.2, 121.0, 62.9, 15.6.

HRMS (CI+) Calcd for $C_{21}H_{17}BrOS$ [M–HBF4]$^{+}$: 396.0183, found: 396.0193.

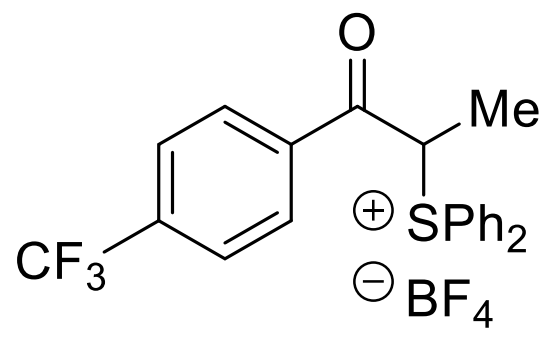

1w

(1-Oxo-1-(4-(trifluoromethyl)phenyl)propan-2-yl)diphenylsulfonium tetra-fluoroborate (1w) was prepared as a white solid from 2-bromo-1-(4-(trifluoromethyl)phenyl)propan-1-one (2.8 g, 10.0 mmol), diphenyl sulfide (3.7 g, 20.0 mmol), and $AgBF_4$ (1.9 g, 10.0 mmol) in dry DCE (38 mL) according to the General Procedure A (24 h, eluent: DCM/MeOH = 50:1) in 60% yield.

^{1}H NMR (400 MHz, $CDCl_3$) δ 8.33 (d, *J* = 8.2 Hz, 2H), 8.28 – 8.26 (m, 2H), 8.15 – 8.12 (m, 2H), 7.81 – 7.73 (m, 5H), 7.65 – 7.57 (m, 3H), 7.06 (q, *J* = 7.2 Hz, 1H), 1.77 (d, *J* = 7.2 Hz, 3H).

^{13}C NMR (100 MHz, $CDCl_3$) δ 192.6, 135.8 (q, *J* = 32.8 Hz), 134.9, 134.2, 134.1, 131.6, 131.4, 131.1, 129.8, 129.6, 126.0 (q, *J* = 3.6 Hz), 124.2, 122.5 (q, *J* = 271.5 Hz), 121.0, 62.8, 15.3.

^{19}F NMR (376 MHz, $CDCl_3$) δ –63.5, –150.45, –150.50.

HRMS (CI+) Calcd for $C_{22}H_{17}F_3OS$ [M–HBF_4]$^{+}$: 386.0952, found: 386.0952.

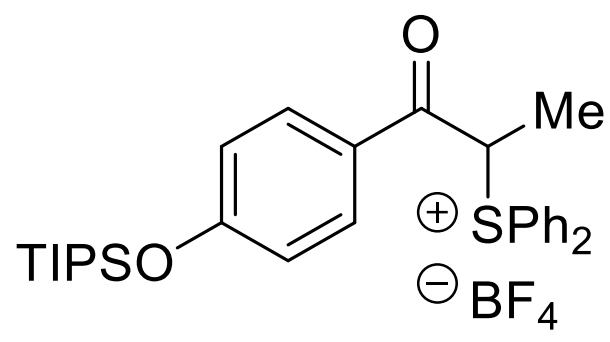

1x

(1-Oxo-1-(4-((Triisopropylsilyl)oxy)phenyl)propan-2-yl)diphenylsulfonium tetrafluoroborate (1x) was prepared as an orange foam from 2-bromo-1-(4-((triisopropylsilyl)oxy)phenyl)propan-1-one (2.5 g, 6.3 mmol), diphenyl sulfide (2.4 g, 12.6 mmol), and $AgBF_4$ (1.2 g, 6.3 mmol) according to the General Procedure A (24 h, eluent: DCM/MeOH = 50:1) in 73% yield.

^{1}H NMR (400 MHz, $CDCl_3$) δ 8.23 – 8.21 (m, 2H), 8.12 – 8.07 (m, 4H), 7.76 – 7.74 (m, 3H), 7.61 – 7.55 (m, 3H), 7.02 – 7.96 (m, 3H), 1.76 (d, *J* = 7.1 Hz, 3H), 1.33 – 1.22 (m, 3H), 1.08 – 1.06 (m, 18H).

^{13}C NMR (100 MHz, $CDCl_3$) δ 191.5, 162.9, 134.9, 134.2, 132.2, 132.1, 131.7, 131.3, 130.2, 125.2, 125.1, 122.0, 120.5, 63.4, 17.6, 16.2, 12.5.

HRMS (ES+) Calcd for $C_{30}H_{39}O_2SSi$ $[M–BF_4]^+$: 491.2435, found: 491.2441.

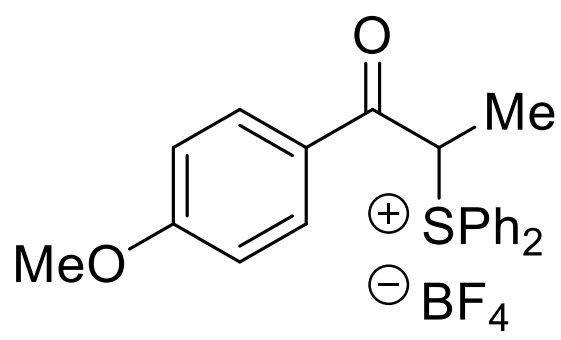

1y

(1-(4-Methoxyphenyl)-1-oxopropan-2-yl)diphenylsulfonium tetrafluoroborate (1y) was prepared as a colorless solid from 2-bromo-1-(4-methoxy-phenyl)propan-1-one (3.0 g, 12.3 mmol), diphenyl sulfide (4.6 g, 24.6 mmol), and $AgBF_4$ (2.4 g, 12.3 mmol) in dry DCE (48 mL) according to the General Procedure A (24 h, eluent: DCM/MeOH = 50:1) in 56% yield.

^{1}H NMR (400 MHz, $CDCl_3$) δ 8.25 – 8.23 (m, 2H), 8.21 – 8.17 (m, 2H), 8.10 – 8.08 (m, 2H), 7.78 – 7.75 (m, 3H), 7.62 – 7.56 (m, 3H), 7.03 – 6.97 (m, 3H), 3.86 (s, 3H), 1.77 (d, *J* = 7.2 Hz, 3H).

^{13}C NMR (100 MHz, $CDCl_3$) δ 191.2, 165.1, 134.6, 133.9, 131.9, 131.6, 131.3, 130.9,

129.7, 124.6, 124.3, 121.4, 114.3, 63.1, 55.2, 15.9.

HRMS (CI+) Calcd for $C_{22}H_{20}O_2S$ [M–HBF_4]$^{+}$: 348.1184, found: 348.1177.

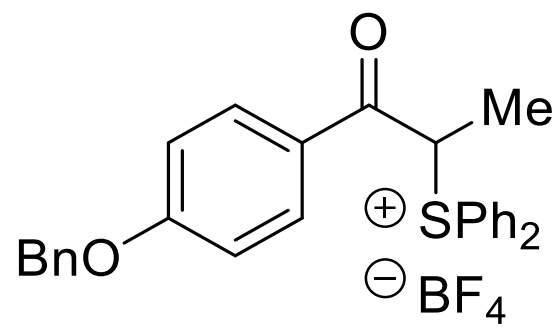

1z

(1-(4-(Benzyloxy)phenyl)-1-oxopropan-2-yl)diphenylsulfonium tetrafluoroborate (1z) was prepared as a white solid from 1-(4-(benzyloxy)phenyl)-2-iodopropan-1-one (1.7 g, 4.6 mmol), diphenyl sulfide (1.7 g, 9.2 mmol), and $AgBF_4$ (1.3 g, 6.9 mmol, 1.5 equiv) according to the General Procedure A at room temperature (24 h, eluent: DCM/MeOH = 50:1) in 81% yield.

^{1}H NMR (400 MHz, $CDCl_3$) δ 8.28 – 8.13 (m, 4H), 8.11 – 8.04 (m, 2H), 7.73 (dd, *J* = 4.9, 1.8 Hz, 3H), 7.62 – 7.49 (m, 3H), 7.42 – 7.23 (m, 5H), 7.11 – 7.03 (m, 2H), 6.96 (q, *J* = 7.1 Hz, 1H), 5.09 (d, *J* = 1.3 Hz, 2H), 1.75 (d, *J* = 7.1 Hz, 3H).

^{13}C NMR (100 MHz, $CDCl_3$) δ 191.6, 164.7, 135.6, 135.1, 134.4, 132.4, 132.2, 131.8, 131.4, 130.3, 128.6, 128.3, 127.5, 125.1, 125.1, 122.0, 115.5, 70.3, 63.5, 16.4.

HRMS (ES+) Calcd for $C_{28}H_{25}O_2S$ [M–BF_4]$^+$: 425.1570, found: 425.1574.

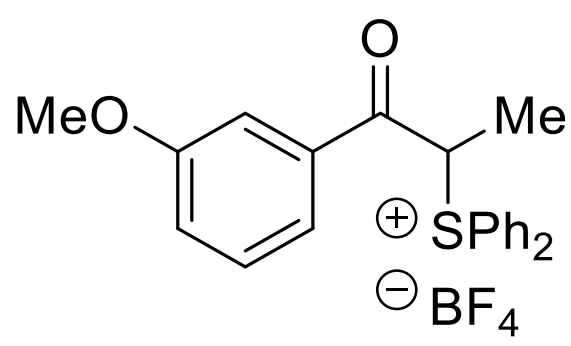

1aa

(1-(3-Methoxyphenyl)-1-oxopropan-2-yl)diphenylsulfonium tetrafluoroborate (1aa) was prepared as a white solid from 2-bromo-1-(3-methoxyphenyl)propan-1-one (4.8 g, 20.0 mmol), diphenyl sulfide (7.4 g, 40.0 mmol), and $AgBF_4$ (3.8 g, 12.3 mmol) in dry DCE (40 mL) according to the General Procedure A (24 h, eluent: DCM/MeOH = 50:1) in 80% yield.

^{1}H NMR (400 MHz, $CDCl_3$) δ 8.26 – 8.24 (m, 2H), 8.12 – 8.10 (m, 2H), 7.82 – 7.80 (m, 1H), 7.73 – 7.72 (m, 3H), 7.61 – 7.53 (m, 4H), 7.44 – 7.40 (m, 1H), 7.17 – 7.15 (m, 1H), 6.96 (q, *J* = 7.2 Hz, 1H), 3.85 (s, 3H), 1.75 (d, *J* = 7.2 Hz, 3H).

^{13}C NMR (100 MHz, $CDCl_3$) δ 193.5, 160.2, 135.2, 134.5, 133.3, 132.3, 131.8, 131.5, 130.6, 130.3, 125.0, 123.3, 122.5, 121.8, 112.1, 63.8, 55.7, 16.2.

HRMS (ES+) Calcd for $C_{22}H_{21}O_2S$ $[M–BF_4]^+$: 349.1257, found: 349.1262.

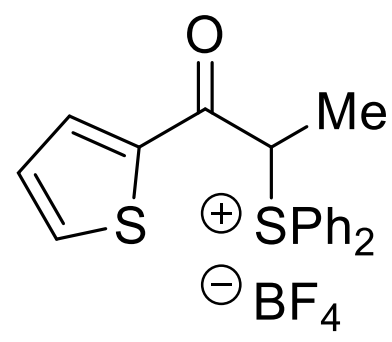

1ab

(1-Oxo-1-(thiophen-2-yl)propan-2-yl)diphenylsulfonium tetrafluoroborate (1ab) was prepared as a light orange solid from 2-bromo-1-(thiophen-2-yl)propan-1-one (2.2 g, 10.0 mmol), diphenyl sulfide (3.7 g, 20.0 mmol), and $AgBF_4$ (1.9 g, 10.0 mmol) in dry DCE (38 mL) according to the General Procedure A (12 h, eluent: DCM/MeOH = 50:1) in 59% yield.

^{1}H NMR (400 MHz, $CDCl_3$) δ 8.50 (d, J = 4.0 Hz, 1H), 8.26 – 8.22 (m, 2H), 8.11 – 8.08 (m, 2H), 7.83-7.76 (m, 4H), 7.66 – 7.58 (m, 3H), 7.28 – 7.26 (m, 1H), 6.89 (q, *J* = 7.2 Hz, 1H), 1.83 (d, *J* = 7.2 Hz, 3H).

^{13}C NMR (100 MHz, $CDCl_3$) δ 185.2, 138.1, 137.7, 137.3, 134.8, 134.1, 131.6, 131.4, 131.0, 129.9, 129.7, 124.0, 121.0, 62.6, 16.3.

HRMS (CI+) Calcd for $C_{19}H_{16}OS_2$ $[M–HBF_4]^+$: 324.0643, found: 324.0654.

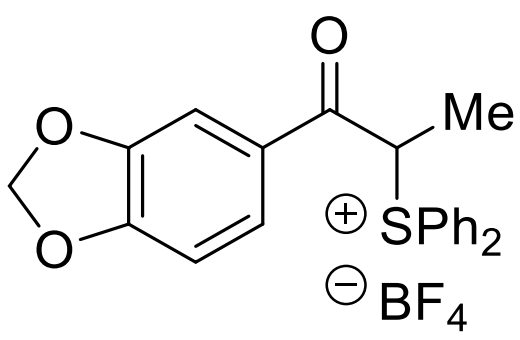

1ac

(1-(Benzo[d][1,3]dioxol-5-yl)-1-oxopropan-2-yl)diphenylsulfonium tetra-fluoroborate (1ac) was prepared as an white solid from 1-(benzo[d][1,3]-

dioxol-5-yl)-2-bromopropan-1-one (1.3 g, 5.0 mmol), diphenyl sulfide (1.9 g, 10.0 mmol), and $AgBF_4$ (1.0 g, 5.0 mmol) according to the General Procedure A (24 h, eluent: DCM/MeOH = 50:1) in 62% yield.

^{1}H NMR (400 MHz, $CDCl_3$) δ 8.26 – 8.18 (m, 2H), 8.12 – 7.97 (m, 3H), 7.79 – 7.70 (m, 3H), 7.65 – 7.52 (m, 3H), 7.41 (d, *J* = 1.8 Hz, 1H), 7.00 – 6.88 (m, 2H), 6.02 (dd, *J* = 5.8, 1.2 Hz, 2H), 1.75 (d, *J* = 7.1 Hz, 3H).

^{13}C NMR (100 MHz, $CDCl_3$) δ 191.4, 154.2, 148.8, 135.2, 134.5, 132.2, 131.9, 131.5, 130.3, 128.2, 126.6, 125.0, 121.9, 109.1, 108.1, 102.4, 63.6, 16.5.

HRMS (ES+) Calcd for $C_{22}H_{19}O_3S$ $[M–BF_4]^+$: 363.1049, found: 363.1056.

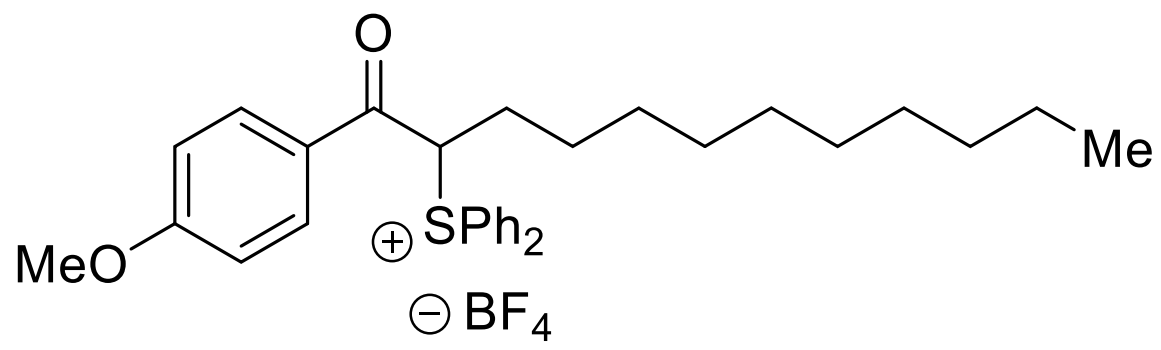

1ad

(1-(4-Methoxyphenyl)-1-oxododecan-2-yl)diphenylsulfonium tetrafluoroborate (1ad) was prepared as a red crude from 2-bromo-1-(4-methoxyphenyl)dodecan-1-one (1.6 g, 4.3 mmol), diphenyl sulfide (1.6 g, 8.7 mmol), and $AgBF_4$ (0.8 g, 4.3 mmol) according to the General Procedure A (24 h, eluent: DCM → ethyl acetate) in 29% yield.

^{1}H NMR (400 MHz, $CDCl_3$) δ 8.28 – 8.13 (m, 4H), 8.12 – 8.04 (m, 2H), 7.79 – 7.71 (m, 3H), 7.59 – 7.49 (m, 3H), 7.06 – 6.95 (m, 3H), 3.84 (s, 3H), 2.35 – 2.21 (m, 1H), 2.01 – 1.88 (m, 1H), 1.50 – 0.98 (m, 16H), 0.83 (t, *J* = 7.0 Hz, 3H).

^{13}C NMR (100 MHz, $CDCl_3$) δ 191.4, 165.7, 135.2, 134.4, 132.3, 132.1, 131.9, 131.5, 130.4 (2C), 126.0, 125.0, 122.1, 114.8, 68.4, 55.8, 31.8, 29.4, 29.3, 29.1, 29.0, 28.7, 24.5, 22.6, 14.0.

HRMS (ES+) Calcd for $C_{31}H_{39}O_2S$ $[M–BF_4]^+$: 475.2665, found: 475.2670.

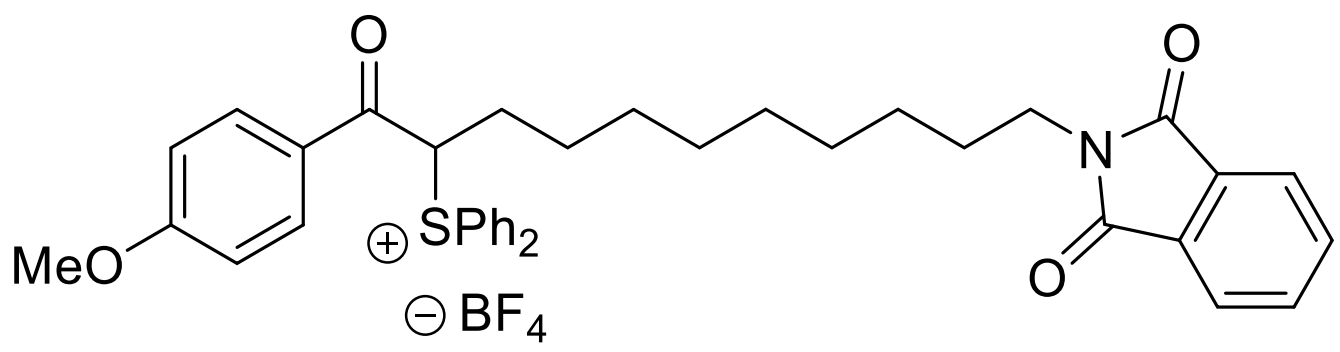

1ae

(11-(1,3-Dioxoisoindolin-2-yl)-1-(4-methoxyphenyl)-1-oxoundecan-2-yl)diphenylsulfonium tetrafluoroborate (1ae) was prepared as a red from 2-(10-bromo-11-(4-methoxyphenyl)-11-oxoundecyl)isoindoline-1,3-dione (2.2 g, 4.5 mmol), diphenyl sulfide (1.7 g, 8.9 mmol), and $AgBF_4$ (0.9 g, 4.4 mmol) according to the General Procedure A (24 h, eluent: DCM → ethyl acetate) in 24% yield.

^{1}H NMR (400 MHz, $CDCl_3$) δ 8.27 – 8.20 (m, 2H), 8.20 – 8.12 (m, 2H), 8.11 – 8.04 (m, 2H), 7.84 – 7.65 (m, 6H), 7.59 – 7.49 (m, 3H), 7.06 – 6.95 (m, 3H), 3.83 (s, 3H), 3.61 (t, *J* = 7.4 Hz, 2H), 2.33 – 2.20 (m, 1H), 1.98 – 1.85 (m, 1H), 1.62 – 1.53 (m, 2H), 1.41 – 0.93 (m, 13H).

^{13}C NMR (100 MHz, $CDCl_3$) δ 191.3, 168.4, 165.6, 135.2, 134.3, 133.8, 132.3, 132.1, 132.0, 131.9, 131.4, 130.4, 125.9, 124.9, 123.0, 122.1, 114.8, 68.3, 55.7, 37.8, 29.3, 28.9, 28.84, 28.79, 28.6, 28.4, 26.6, 24.4.

HRMS (ES+) Calcd for $C_{38}H_{40}NO_4S$ $[M–BF_4]^+$: 606.2673, found: 606.2684.

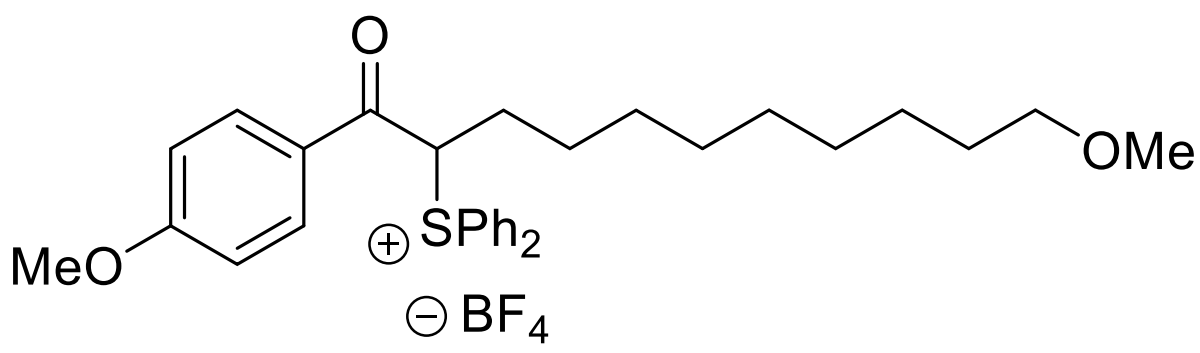

1af

(11-Methoxy-1-(4-methoxyphenyl)-1-oxoundecan-2-yl)diphenylsulfonium tetrafluoroborate (1af) was prepared as a red viscous oil from 2-bromo-11-methoxy-1-(4-methoxyphenyl)undecan-1-one (1.7 g, 4.4 mmol), diphenyl sulfide (1.7 g, 8.9 mmol), and $AgBF_4$ (0.9 g, 4.4 mmol) according to the General Procedure A (24 h, eluent: DCM → ethyl acetate) in 31% yield.

^{1}H NMR (400 MHz, $CDCl_3$) δ 8.27 – 8.19 (m, 2H), 8.18 – 8.11 (m, 2H), 8.10 – 8.03

(m, 2H), 7.76 – 7.69 (m, 3H), 7.56 – 7.45 (m, 3H), 7.01 – 6.92 (m, 3H), 3.80 (s, 3H), 3.32 – 3.24 (m, 5H), 2.29 – 2.20 (m, 1H), 1.98 – 1.86 (m, 1H), 1.51 – 1.39 (m, 2H), 1.23 – 1.15 (m, 2H), 1.15 – 0.98 (m, 10H).

^{13}C NMR (100 MHz, $CDCl_3$) δ 191.1, 165.5, 135.1, 134.3, 132.2, 132.0, 131.8, 131.3, 130.3, 125.9, 124.9, 122.1, 114.7, 72.7, 68.0, 58.4, 55.6, 29.4, 29.3, 29.0, 28.9, 28.8, 28.5, 25.8, 24.4.

HRMS (ES+) Calcd for $C_{31}H_{39}O_3S$ $[M–BF_4]^+$: 491.2614, found: 491.2623.

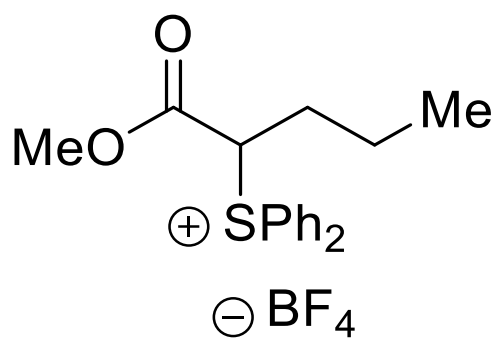

4a

(1-Methoxy-1-oxopentan-2-yl)diphenylsulfonium tetrafluoroborate (4a) was prepared as a light yellow solid from methyl 2-bromopentanoate (2.3 g, 11.6 mmol), diphenyl sulfide (4.3 g, 23.2 mmol), and $AgBF_4$ (2.3 g, 11.6 mmol) according to the General Procedure D (eluent: hexanes/ethyl acetate = 10:1 → ethyl acetate) in 18% yield.

^{1}H NMR (400 MHz, $CDCl_3$) *δ* 8.23 – 8.16 (m, 4H), 7.79 – 7.65 (m, 6H), 5.83 – 5.80 (m, 1H), 3.71 (s, 3H), 2.14 – 2.04 (m, 1H), 1.94 – 1.85 (m, 1H), 1.56 – 1.46 (m, 2H), 0.86 (t, *J* = 7.3 Hz, 3H).

^{13}C NMR (100 MHz, $CDCl_3$) *δ* 166.2, 135.2, 134.7, 131.8, 131.7, 131.4, 130.6, 124.1, 121.9, 61.4, 54.0, 30.3, 18.9, 13.3.

HRMS (ES+) Calcd for $C_{18}H_{21}O_2S$ $[M–BF_4]^+$: 301.1257, found: 301.1259.

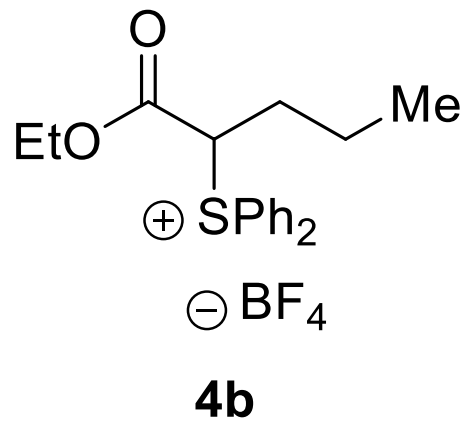

4b

(1-Ethoxy-1-oxopentan-2-yl)diphenylsulfonium tetrafluoroborate (4b) was

prepared as a light yellow solid from ethyl 2-bromopentanoate (2.4 g, 11.6 mmol), diphenyl sulfide (3.7 mL, 23.2 mmol), and $AgBF_4$ (2.3 g, 11.6 mmol) according to the General Procedure D (eluent: hexanes/ethyl acetate = 10:1 → ethyl acetate) in 9% yield.

^{1}H NMR (400 MHz, $CDCl_3$) δ 8.15 – 8.13 (m, 4H), 7.77 – 7.66 (m, 6H), 5.91 – 5.89 (m, 1H), 4.21 – 4.12 (m, 2H), 2.12 – 2.06 (m, 1H), 1.90 – 1.83 (m, 1H), 1.54 – 1.45 (m, 2H), 1.14 (t, *J* = 7.1 Hz, 3H), 0.88 (t, *J* = 7.3 Hz, 3H).

^{13}C NMR (100 MHz, $CDCl_3$) δ 166.3, 135.4, 134.9, 132.0, 131.8, 131.6, 130.9, 124.2, 122.0, 63.9, 62.2, 30.5, 19.0, 13.7, 13.6.

HRMS (ES+) Calcd for $C_{19}H_{23}O_2S$ $[M{-}BF_4^-]^+$: 315.1413, found: 315.1423.

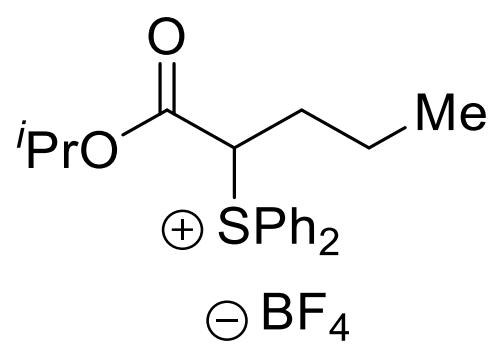

4c

(1-Isopropoxy-1-oxopentan-2-yl)diphenylsulfonium tetrafluoroborate (4c) was prepared as a brown oil from isopropyl 2-bromopentanoate (2.6 g, 11.6 mmol), diphenyl sulfide (3.7 mL, 23.2 mmol), and $AgBF_4$ (2.3 g, 11.6 mmol) according to the General Procedure D (eluent: hexanes/ethyl acetate = 10:1 → ethyl acetate) in 7% yield.

^{1}H NMR (400 MHz, $CDCl_3$) δ 8.17 – 8.14 (m, 4H), 7.73 – 7.64 (m, 6H), 5.73 – 5.70 (m, 1H), 4.97 – 4.88 (m, 1H), 2.07 – 1.98 (m, 1H), 1.91 – 1.82 (m, 1H), 1.55 – 1.46 (m, 2H), 1.12 (d, *J* = 6.2 Hz, 3H), 1.07 (d, *J* = 6.3 Hz, 3H), 0.86 (t, *J* = 7.3 Hz, 3H).

^{13}C NMR (100 MHz, $CDCl_3$) δ 165.2, 135.3, 134.8, 131.9, 131.7, 131.5, 130.8, 124.3, 122.1, 72.5, 61.7, 30.5, 21.3, 19.0, 13.5.

HRMS (ES+) Calcd for $C_{20}H_{25}NO_2S$ $[M{-}BF_4^-]^+$: 329.1570, found: 329.1569.

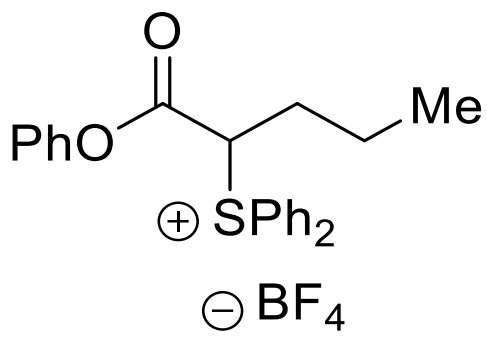

4d

(1-Oxo-1-phenoxypentan-2-yl)diphenylsulfonium tetrafluoroborate (4d) was prepared as a light yellow solid from phenyl 2-bromopentanoate (3.0 g, 11.6 mmol), diphenyl sulfide (4.3 g, 23.2 mmol), and $AgBF_4$ (2.3 g, 11.6 mmol) according to the General Procedure D (eluent: hexanes/Ethyl acetate = 10:1 → ethyl acetate) in 6% yield.

^{1}H NMR (400 MHz, $CDCl_3$) δ 8.24 – 8.22 (m, 4H), 7.74 – 7.63 (m, 6H), 7.30 – 7.26 (m, 2H), 7.22 – 7.13 (m, 1H), 6.89 – 6.83 (m, 2H), 6.13 – 6.10 (m, 1H), 2.26 – 2.18 (m, 1H), 2.05 – 1.96 (m, 1H), 1.71 – 1.61 (m, 2H), 0.92 (t, J = 7.2 Hz, 3H).

^{13}C NMR (100 MHz, $CDCl_3$) δ 164.9, 149.5, 135.4, 135.0, 132.0, 131.8, 131.6, 130.9, 129.6, 126.9, 123.9, 121.8, 120.6, 61.5, 30.5, 19.1, 13.5.

HRMS (ES+) Calcd for $C_{23}H_{23}O_2S$ $[M{-}BF_4]^+$: 363.1413, found: 363.1417.

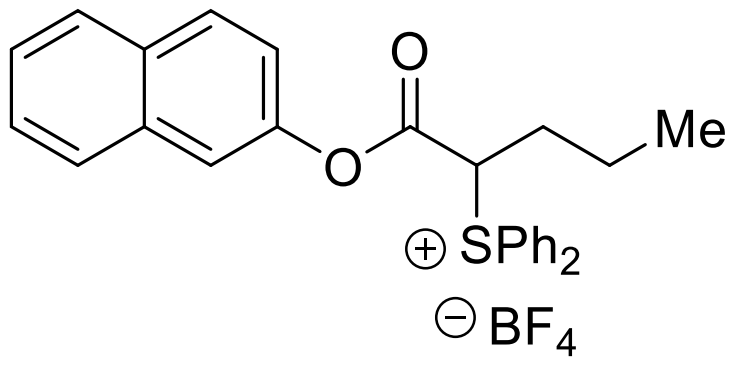

4e

(1-(Naphthalen-2-yloxy)-1-oxopentan-2-yl)diphenylsulfonium tetrafluoroborate (4e) was prepared as a red solid from naphthalen-2-yl 2-bromopentanoate (3.5 g, 11.6 mmol), diphenyl sulfide (4.3 g, 23.2 mmol), and $AgBF_4$ (2.3 g, 11.6 mmol) according to the General Procedure D (eluent: hexanes/ethyl acetate = 10:1 → ethyl acetate) in 7% yield.

^{1}H NMR (400 MHz, $CDCl_3$) δ 8.26 – 8.22 (m, 4H), 7.81 – 7.67 (m, 9H), 7.50 – 7.45 (m, 2H), 7.381 – 7.375 (m, 1H), 7.00 – 6.97 (m, 1H), 6.31 – 6.28 (m, 1H), 2.38 – 2.29 (m, 1H), 2.08 – 1.99 (m, 1H), 1.79 – 1.64 (m, 2H), 0.98 (t, J = 7.3 Hz, 3H).

^{13}C NMR (100 MHz, $CDCl_3$) δ 165.4, 147.2, 135.6, 135.1, 133.3, 132.1, 131.9,

131.8(2C), 131.1, 129.8, 127.8, 127.7, 126.9, 126.3, 123.9, 121.8, 119.6, 118.1, 62.2, 30.7, 19.2, 13.7.

HRMS (ES+) Calcd for $C_{27}H_{25}O_2S$ $[M-BF_4]^+$: 413.1570, found: 413.1575.

III. Catalytic Asymmetric N−H Insertion

General Procedure E

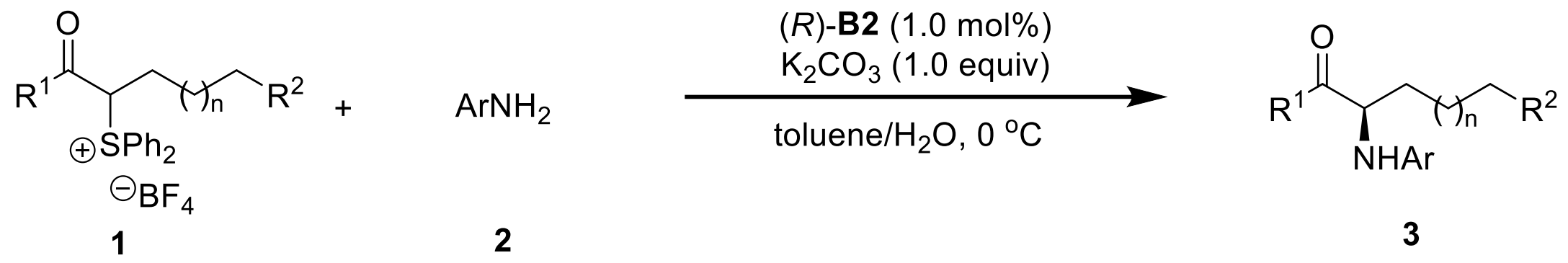

At room temperature, an oven-dried 10-mL vial was charged with K_2CO_3 (55.3 mg, 0.4 mmol), (*R*)-**B2** (2.8 mg, 4.0 μmol, 1.0 mol%), and toluene (4 mL). The mixture was vigorously stirred for 25 min before the sulfonium salt **1** (0.4 mmol) was added. The mixture was kept stirring for another 10 min before it was cooled to 0 °C and the amine **2** (0.44 mmol, 1.1 equiv) was added together with water (4 mL). The mixture was vigorously stirred at 0 °C until full conversion of the sulfonium salt **1**. Then, the organic layer was separated, and the aqueous layer was extracted by DCM (2 mL × 3). The combined organic layers were directly subjected to flash silica gel column chromatography to afford the desired product **3**.

General Procedure F

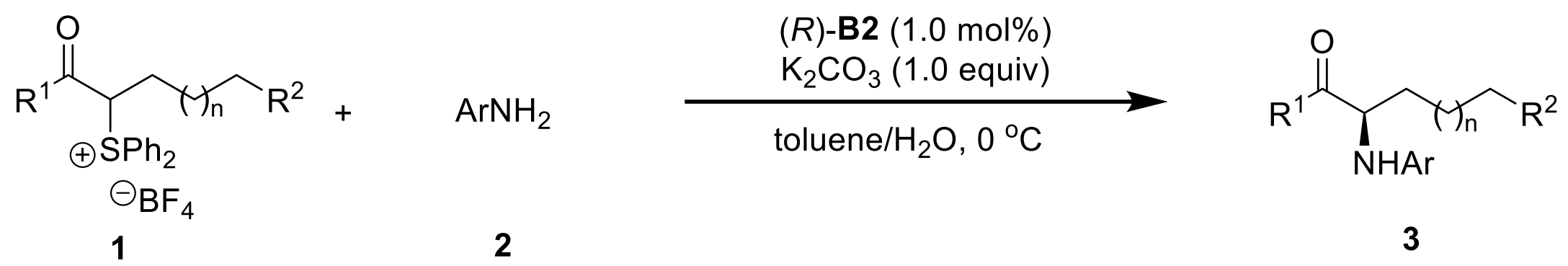

At room temperature, an oven-dried 10-mL vial was charged with K_2CO_3 (55.3 mg, 0.4 mmol, 1.0 equiv), (*R*)-**B2** (2.8 mg, 4.0 μmol, 1.0 mol%), and toluene (4 mL). The mixture was vigorously stirred for 25 min before the sulfonium salt **1** (0.4 mmol) was added. The mixture was kept stirring for another 10 min before it was cooled to –10 °C and the amine **2** (0.44 mmol, 1.1 equiv) was added together with saturated brine (4 mL). The mixture was vigorously stirred at –10 °C until full conversion of the sulfonium salt **1**. Then, the organic layer was

separated, and the aqueous layer was extracted by DCM (2 mL × 3). The combined organic layers were directly subjected to flash silica gel column chromatography to afford the desired product **3**.

General Procedure G

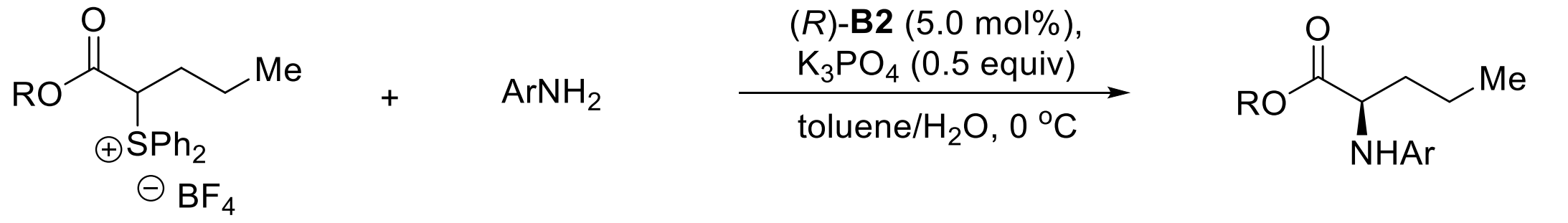

4 **2** **5**

At room temperature, an oven-dried 10-mL vial was charged with K_3PO_4 (42.5 mg, 0.2 mmol, 0.5 equiv), (*R*)-**B2** (14.0 mg, 20 μmol, 5.0 mol%), and toluene (4 mL). The mixture was vigorously stirred for 25 min before the sulfonium salt **4** (0.4 mmol) was added. The mixture was kept stirring for another 10 min before it was cooled to 0 °C and the amine **2** (0.44 mmol, 1.1 equiv) was added together with water (4 mL). The mixture was vigorously stirred at 0 °C until full conversion of the sulfonium salt **4**. Then, the organic layer was separated, and the aqueous layer was extracted by DCM (2 mL × 3). The combined organic layers were directly subjected to flash silica gel column chromatography to afford the desired product **5**.

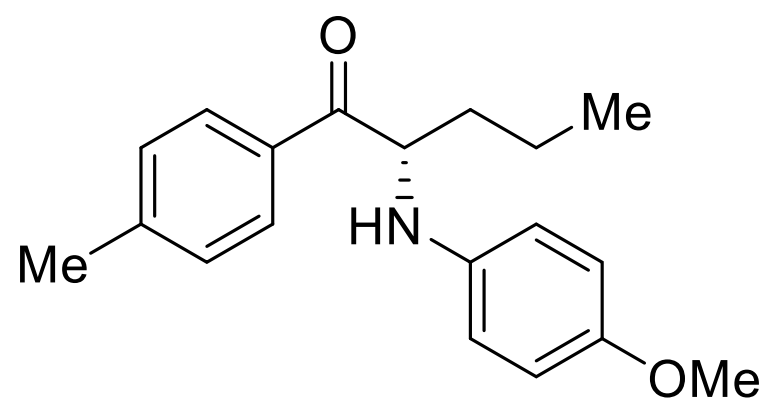

3a

(*S*)-2-((4-Methoxyphenyl)amino)-1-(*p*-tolyl)pentan-1-one (3a) was prepared as a green oil from (1-oxo-1-(*p*-tolyl)pentan-2-yl)diphenylsulfonium tetrafluoroborate (179.2 mg, 0.4 mmol), (*S*)-**B2** (2.8 mg, 1.0 mol%), K_2CO_3 (55.3 mg, 0.4 mmol) and *p*-anisidine (54.2 mg, 0.44 mmol) in toluene (4 mL) and water (4 mL) according to the General Procedure E (4 h, eluent: hexanes/ethyl acetate

= 10:1 → 5:1, 110.1 mg, 93% yield, 95% ee).

$[\alpha]_D^{22}$: +55.7 (*c* = 1.0, CH_2Cl_2). HPLC analysis of the product: Daicel CHIRALPAK IC column; 3% *i*-PrOH in hexanes; 1.0 mL/min; retention times: 29.6 min (major), 35.3 min (minor).

^{1}H NMR (400 MHz, $CDCl_3$) δ 7.91 (d, *J* = 8.2 Hz, 2H), 7.29 (d, *J* = 8.0 Hz, 2H), 6.77 – 6.73 (m, 2H), 6.67 – 6.65 (m, 2H), 4.99 – 4.96 (m, 1H), 4.39 (br, 1H), 3.72 (s, 3H), 2.43 (s, 3H), 1.95 – 1.86 (m, 1H), 1.69 – 1.60 (m, 1H), 1.53 – 1.38 (m, 2H), 0.91 (t, *J* = 7.3 Hz, 3H).

^{13}C NMR (100 MHz, $CDCl_3$) δ 200.9, 152.4, 144.3, 141.5, 132.8, 129.4, 128.3, 115.2, 114.8, 58.8, 55.6, 35.6, 21.6, 18.7, 13.9.

HRMS (ES+) Calcd for $C_{19}H_{24}NO_2$ $[M+H]^+$: 298.1802, found: 298.1815.

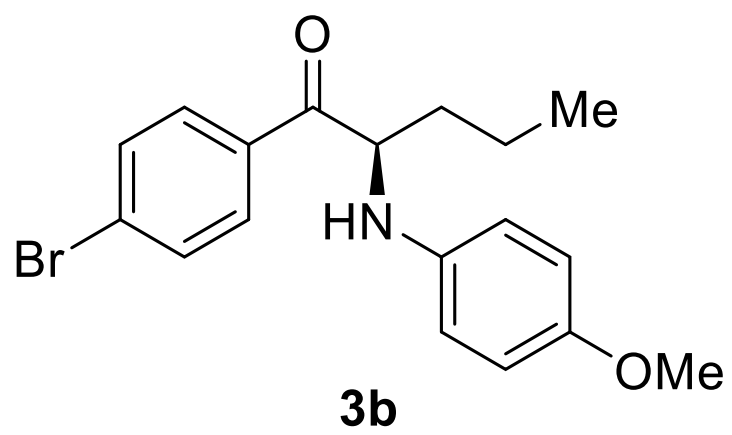

3b

(*R*)-1-(4-Bromophenyl)-2-((4-methoxyphenyl)amino)pentan-1-one (3b) was prepared as a green oil from (1-(4-bromophenyl)-1-oxopentan-2-yl)diphenyl-sulfonium tetrafluoroborate (204.8 mg, 0.4 mmol), (*R*)-**B2** (2.8 mg, 1.0 mol%), K_2CO_3 (55.3 mg, 0.4 mmol) and *p*-anisidine (54.2 mg, 0.44 mmol) in toluene (4 mL) and saturated brine (4 mL) according to the General Procedure F (12 h, eluent: hexanes/ethyl acetate = 10:1 → 5:1, 127.0 mg, 88% yield, 95% ee).

$[\alpha]_D^{20}$: –43.1 (*c* = 1.0, CH_2Cl_2). HPLC analysis of the product: Daicel CHIRALPAK IC column; 3% *i*-PrOH in hexanes; 1.0 mL/min; retention times: 23.7 min (minor), 26.7 min (major).

^{1}H NMR (400 MHz, $CDCl_3$) δ 7.86 – 7.84 (m, 2H), 7.64 – 7.61 (m, 2H), 6.77 – 6.75 (m, 2H), 6.65 – 6.63 (m, 2H), 4.93 – 4.90 (m, 1H), 4.27 (br, 1H), 3.72 (s, 3H), 1.93 – 1.84 (m, 1H), 1.67 – 1.58 (m, 1H), 1.52 – 1.38 (m, 2H), 0.91 (t, *J* = 7.3 Hz, 3H).

^{13}C NMR (100 MHz, $CDCl_3$) δ 200.5, 152.5, 141.2, 134.0, 132.1, 129.7, 128.6, 115.2,

114.9, 59.1, 55.6, 35.3, 18.7, 13.9.

HRMS (ES+) Calcd for $C_{18}H_{21}BrNO_2$ $[M+H]^+$: 362.0750, found: 362.0768.

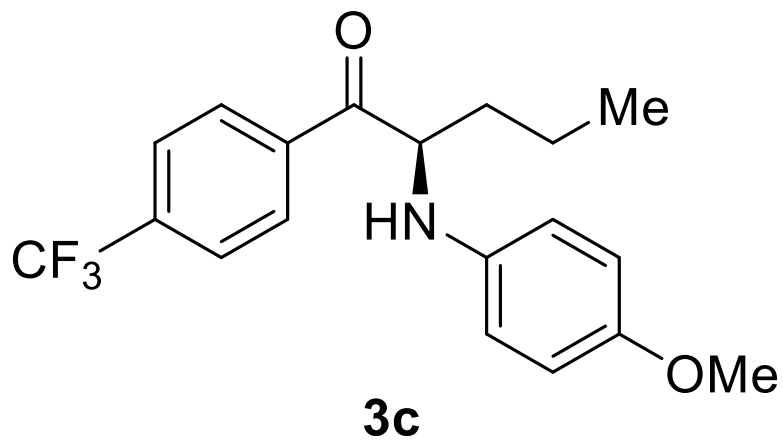

3c

(*R*)-2-((4-Methoxyphenyl)amino)-1-(4-(trifluoromethyl)phenyl)pentan-1-one (3c) was prepared as a green oil from (1-oxo-1-(4-(trifluoromethyl)phenyl)-pentan-2-yl)diphenylsulfonium tetrafluoroborate (200.9 mg, 0.4 mmol), (*R*)-**B2** (2.8 mg, 1.0 mol%), K_2CO_3 (55.3 mg, 0.4 mmol) and *p*-anisidine (54.2 mg, 0.44 mmol) in toluene (4 mL) and water (4 mL) according to the General Procedure E (12 h, eluent: hexanes/ethyl acetate = 10:1 → 5:1, 107.0 mg, 76% yield, 95% ee). $[\alpha]_D^{20}$: –29.5 (*c* = 1.0, CH_2Cl_2). HPLC analysis of the product: Daicel CHIRALPAK IC column; 3% *i*-PrOH in hexanes; 1.0 mL/min; retention times: 13.2 min (minor), 14.5 min (major).

^{1}H NMR (400 MHz, $CDCl_3$) δ 8.08 (d, *J* = 8.1 Hz, 2H), 7.75 (d, *J* = 8.2 Hz, 2H), 6.77 – 6.75 (m, 2H), 6.67 – 6.65 (m, 2H), 4.97 – 4.94 (m, 1H), 4.02 (br, 1H), 3.72 (s, 3H), 1.95 – 1.86 (m, 1H), 1.69 – 1.60 (m, 1H), 1.51 – 1.39 (m, 2H), 0.91 (t, *J* = 7.3 Hz, 3H).

^{13}C NMR (100 MHz, $CDCl_3$) δ 200.7, 152.8, 141.0, 138.1, 134.6 (q, *J* = 32.7 Hz), 128.6, 125.9 (q, *J* = 3.6 Hz), 123.8 (q, *J* = 303.1 Hz), 115.5, 114.9, 59.7, 55.6, 35.2, 18.7, 13.9.

^{19}F NMR (376 MHz, $CDCl_3$) δ –63.2.

HRMS (ES+) Calcd for $C_{19}H_{21}F_3NO_2$ $[M+H]^+$: 352.1519, found: 352.1534.

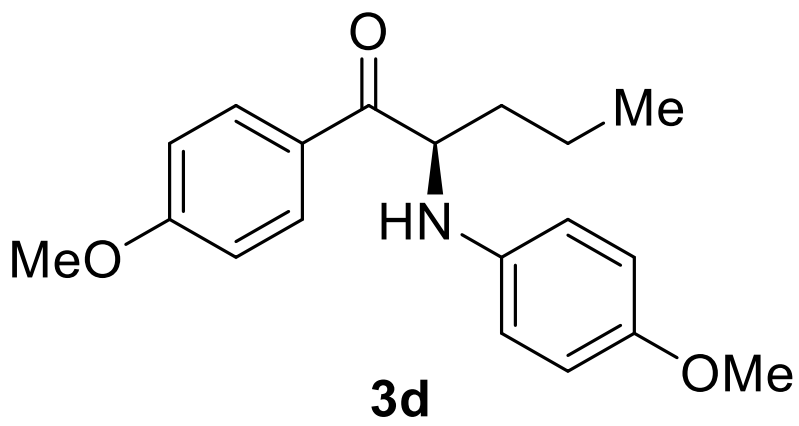

3d

(*R*)-1-(4-Methoxyphenyl)-2-((4-methoxyphenyl)amino)pentan-1-one (3d) was prepared as a green oil from (1-(4-methoxyphenyl)-1-oxopentan-2-yl)diphenylsulfonium tetrafluoroborate (185.7 mg, 0.4 mmol), (*R*)-**B2** (2.8 mg, 1.0 mol%), K_2CO_3 (55.3 mg, 0.4 mmol) and *p*-anisidine (54.2 mg, 0.44 mmol) in toluene (4 mL) and water (4 mL) according to the General Procedure E (4 h, eluent: hexanes/ethyl acetate = 10:1 → 5:1, 116.5 mg, 93% yield, 92% ee).

$[\alpha]_D^{20}$: –66.1 (c = 1.0, CH_2Cl_2). HPLC analysis of the product: Daicel CHIRALCEL OD-H column; 7% *i*-PrOH in hexanes; 1.0 mL/min; retention times: 13.3 min (major), 15.3 min (minor).

^{1}H NMR (400 MHz, $CDCl_3$) δ 8.01 – 7.96 (m, 2H), 6.97 – 6.95 (m, 2H), 6.76 – 6.74 (m, 2H), 6.67 – 6.65 (m, 2H), 4.96 – 4.93 (m, 1H), 4.39 (br, 1H), 3.86 (s, 3H), 3.71 (s, 3H), 1.94 – 1.85 (m, 1H), 1.69 – 1.60 (m, 1H), 1.53 – 1.39 (m, 2H), 0.91 (t, J = 7.3 Hz, 3H).

^{13}C NMR (100 MHz, $CDCl_3$) δ 199.7, 163.7, 152.3, 141.5, 130.5, 128.2, 115.1, 114.8, 113.9, 58.5, 55.5, 55.3, 35.7, 18.7, 13.9.

HRMS (ES+) Calcd for $C_{19}H_{24}NO_3$ $[M+H]^+$: 314.1751, found: 314.1768.

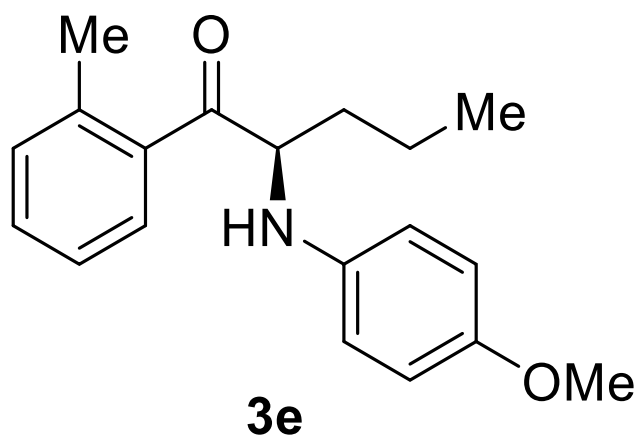

3e

(*R*)-2-((4-Methoxyphenyl)amino)-1-(*o*-tolyl)pentan-1-one (3e) was prepared as a green oil from (1-oxo-1-(*o*-tolyl)pentan-2-yl)diphenylsulfonium tetrafluoroborate (179.2 mg, 0.4 mmol), (*R*)-**B2** (2.8 mg, 1.0 mol%), K_2CO_3 (55.3 mg, 0.4 mmol) and *p*-anisidine (54.2 mg, 0.44 mmol) in toluene (4 mL) and water

(4 mL) according to the General Procedure E (24 h, eluent: hexanes/ethyl acetate = 10:1 → 5:1, 91.0 mg, 76% yield, 94% ee).

$[\alpha]_D^{20}$: –33.4 (c = 1.0, CH_2Cl_2). HPLC analysis of the product: Daicel CHIRALCEL OD-H column; 5% *i*-PrOH in hexanes; 1.0 mL/min; retention times: 7.7 min (minor), 9.6 min (major).

^{1}H NMR (400 MHz, $CDCl_3$) δ 7.64 (d, J = 7.8 Hz, 1H), 7.42 – 7.38 (m, 1H), 7.31 – 7.27 (m, 2H), 6.80 – 6.76 (m, 2H), 6.70 – 6.67 (m, 2H), 4.82 (m, 1H), 4.37 (br, 1H), 3.74 (s, 3H), 2.45 (s, 3H), 1.85 – 1.77 (m, 1H), 1.60 – 1.36 (m, 3H), 0.88 (t, J = 7.3 Hz, 3H).

^{13}C NMR (100 MHz, $CDCl_3$) δ 205.1, 152.5, 141.6, 138.6, 136.4, 132.0, 131.5, 127.9, 125.7, 115.4, 114.9, 61.5, 55.7, 34.8, 20.9, 18.7, 13.9.

HRMS (ES+) Calcd for $C_{19}H_{24}NO_2$ $[M+H]^+$: 298.1802, found: 298.1816.

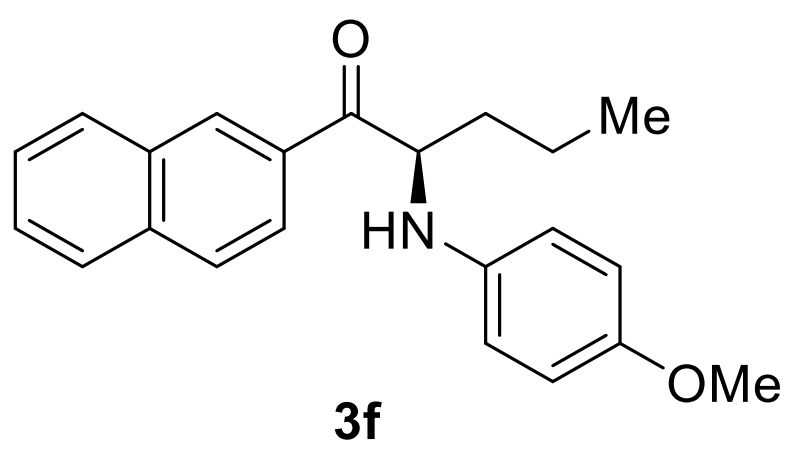

3f

(*R*)-2-((4-Methoxyphenyl)amino)-1-(naphthalen-2-yl)pentan-1-one (3f) was prepared as a green oil from (1-(naphthalen-2-yl)-1-oxopentan-2-yl)diphenylsulfonium tetrafluoroborate (193.7 mg, 0.4 mmol), (*R*)-**B2** (2.8 mg, 1.0 mol%), K_2CO_3 (55.3 mg, 0.4 mmol) and *p*-anisidine (54.2 mg, 0.44 mmol) in toluene (4 mL) and water (4 mL) according to the General Procedure E (4 h, eluent: hexanes/ethyl acetate = 10:1 → 5:1, 118.8 mg, 89% yield, 96% ee).

$[\alpha]_D^{20}$: –112.9 (c = 1.0, CH_2Cl_2). HPLC analysis of the product: Daicel CHIRALPAK IC column; 5% *i*-PrOH in hexanes; 1.0 mL/min; retention times: 19.3 min (minor), 27.0 min (major).

^{1}H NMR (400 MHz, $CDCl_3$) δ 8.53 (s, 1H), 8.06 – 7.99 (m, 2H), 7.94 – 7.89 (m, 2H), 7.65 – 7.56 (m, 2H), 6.79 – 6.71 (m, 4H), 5.16 (m, 1H), 4.27 (br, 1H), 3.73 (s, 3H), 2.03 – 1.94 (m, 1H), 1.77 – 1.68 (m, 1H), 1.58 – 1.44 (m, 2H), 0.92 (t, J = 7.3

Hz, 3H).

^{13}C NMR (100 MHz, $CDCl_3$) δ 201.4, 152.5, 141.5, 135.7, 132.7, 132.5, 129.8, 129.6, 128.72, 128.68, 127.8, 126.9, 124.0, 115.3, 114.9, 59.1, 55.7, 35.7, 18.7, 14.0.

HRMS (ES+) Calcd for $C_{22}H_{24}NO_2$ $[M+H]^+$: 334.1802, found: 334.1816.

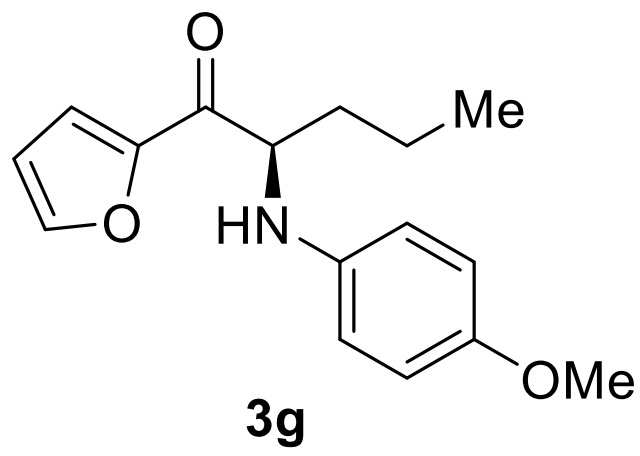

3g

(*R*)-1-(Furan-2-yl)-2-((4-methoxyphenyl)amino)pentan-1-one (3g) was prepared as a green soild from (1-(furan-2-yl)-1-oxopentan-2-yl)diphenylsulfonium tetrafluoroborate (169.7 mg, 0.4 mmol), (*R*)-**B2** (2.8 mg, 1.0 mol%), K_2CO_3 (55.3 mg, 0.4 mmol) and *p*-anisidine (54.2 mg, 0.44 mmol) in toluene (4 mL) and water (4 mL) according to the General Procedure E (4 h, eluent: hexanes/ethyl acetate = 10:1 → 5:1, 95.0 mg, 87% yield, 96% ee).

$[\alpha]_D^{20}$: –50.1 (c = 1.0, CH_2Cl_2). HPLC analysis of the product: Daicel CHIRALPAK IC column; 5% *i*-PrOH in hexanes; 1.0 mL/min; retention times: 34.9 min (minor), 37.9 min (major).

^{1}H NMR (400 MHz, $CDCl_3$) δ 7.62 – 7.61 (m, 1H), 7.29 – 7.28 (m, 1H), 6.75 – 6.72 (m, 2H), 6.64 – 6.62 (m, 2H), 6.56 – 6.55 (m, 1H), 4.75 – 4.72 (m, 1H), 4.20 (br, 1H), 3.71 (s, 3H), 1.92 – 1.83 (m, 1H), 1.74 – 1.65 (m, 1H), 1.53 – 1.42 (m, 2H), 0.94 (t, J = 7.3 Hz, 3H).

^{13}C NMR (100 MHz, $CDCl_3$) δ 191.0, 152.5, 151.7, 146.6, 141.3, 117.9, 115.3, 114.8, 112.4, 59.8, 55.6, 35.5, 18.9, 13.9.

HRMS (ES+) Calcd for $C_{16}H_{20}NO_3$ $[M+H]^+$: 274.1438, found: 274.1455.

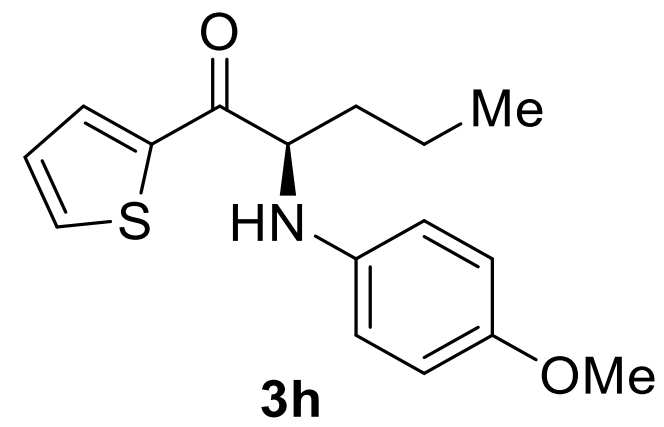

3h

(*R*)-2-((4-Methoxyphenyl)amino)-1-(thiophen-2-yl)pentan-1-one (3h) as prepared as a green oil from (1-oxo-1-(thiophen-2-yl)pentan-2-yl)diphenylsulfonium tetrafluoroborate (176.0 mg, 0.4 mmol), (*R*)-**B2** (2.8 mg, 1.0 mol%), K_2CO_3 (55.3 mg, 0.4 mmol) and *p*-anisidine (54.2 mg, 0.44 mmol) in toluene (4 mL) and water (4 mL) according to the General Procedure E (4 h, eluent: hexanes/ethyl acetate = 10:1 → 5:1, 107.5 mg, 93% yield, 97% ee).

$[\alpha]_D^{20}$: –8.7 (c = 1.0, CH_2Cl_2). HPLC analysis of the product: Daicel CHIRALPAK AD-H column; 7% *i*-PrOH in hexanes; 1.0 mL/min; retention times: 26.7 min (major), 29.8 min (minor).

^{1}H NMR (400 MHz, $CDCl_3$) δ 7.87 – 7.86 (m, 1H), 7.65 – 7.63 (m, 1H), 7.15 – 7.13 (m, 1H), 6.76 – 6.73 (m, 2H), 6.66 – 6.63 (m, 2H), 4.69 – 4.66 (m, 1H), 4.14 (br, 1H), 3.71 (s, 3H), 1.97 – 1.88 (m, 1H), 1.79 – 1.70 (m, 1H), 1.56 – 1.44 (m, 2H), 0.95 (t, J = 7.3 Hz, 3H).

^{13}C NMR (100 MHz, $CDCl_3$) δ 194.8, 152.5, 141.6, 141.1, 134.2, 132.3, 128.1, 115.2, 114.8, 61.1, 55.6, 36.2, 19.0, 13.9.

HRMS (ES+) Calcd for $C_{16}H_{20}NO_2S$ [M+H]$^+$: 290.1209, found: 290.1223.

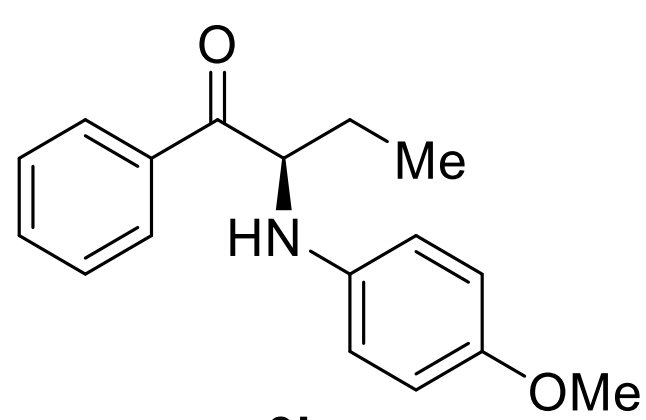

3i

(*R*)-2-((4-Methoxyphenyl)amino)-1-phenylbutan-1-one (3i) was prepared as a green oil from (1-oxo-1-phenylbutan-2-yl)diphenylsulfonium tetrafluoroborate (168.0 mg, 0.4 mmol), (*R*)-**B2** (2.8 mg, 1.0 mol%), K_2CO_3 (55.3 mg, 0.4 mmol) and *p*-anisidine (54.2 mg, 0.44 mmol) in toluene (4 mL) and water (4 mL) according

to the General Procedure E (4 h, eluent: hexanes/ethyl acetate = 10:1 → 5:1, 97.9 mg, 91% yield, 98% ee).

$[\alpha]_D^{20}$: –27.9 (*c* = 1.0, CH_2Cl_2). HPLC analysis of the product: Daicel CHIRALPAK IC column; 3% *i*-PrOH in hexanes; 1.0 mL/min; retention times: 25.0 min (minor), 31.0 min (major).

^{1}H NMR (400 MHz, $CDCl_3$) δ 8.00 (d, *J* = 7.2 Hz, 2H), 7.61 – 7.57 (m, 1H), 7.51 – 7.48 (m, 2H), 6.79 – 6.76 (m, 2H), 6.71 – 6.68 (m, 2H), 5.01 – 4.98 (m, 1H), 4.46 (br, 1H), 3.73 (s, 3H), 2.09 – 2.00 (m, 1H), 1.77 – 1.67 (m, 1H), 0.95 (t, *J* = 7.4 Hz, 3H).

^{13}C NMR (100 MHz, $CDCl_3$) δ 201.0, 152.3, 141.2, 135.3, 133.4, 128.7, 128.2, 115.2, 114.8, 59.9, 55.6, 26.0, 9.3.

HRMS (ES+) Calcd for $C_{17}H_{20}NO_2$ $[M+H]^+$: 270.1489, found: 270.1506.

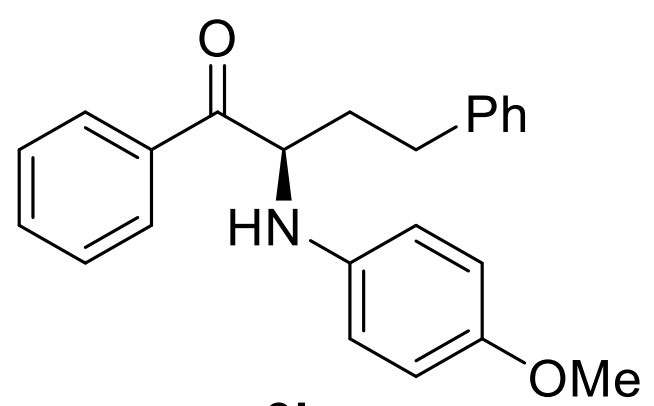

3j

(*R*)-2-((4-Methoxyphenyl)amino)-1,4-diphenylbutan-1-one (3j) was prepared as a green oil from (1-oxo-1,4-diphenylbutan-2-yl)diphenylsulfonium tetrafluoroborate (198.5 mg, 0.4 mmol), (*R*)-**B2** (2.8 mg, 1.0 mol%), K_2CO_3 (55.3 mg, 0.4 mmol) and *p*-anisidine (54.2 mg, 0.44 mmol) in toluene (4 mL) and water (4 mL) according to the General Procedure E (6 h, eluent: hexanes/ethyl acetate = 10:1 → 5:1, 128.3 mg, 93% yield, 95% ee).

$[\alpha]_D^{20}$: –35.0 (*c* = 1.0, CH_2Cl_2). HPLC analysis of the product: Daicel CHIRALPAK IC column; 5% *i*-PrOH in hexanes; 1.0 mL/min; retention times: 17.5 min (minor), 20.4 min (major).

^{1}H NMR (400 MHz, $CDCl_3$) δ 7.86 – 7.84 (m, 2H), 7.60 – 7.56 (m, 1H), 7.47 – 7.43 (m, 2H), 7.33 – 7.29 (m, 2H), 7.26 – 7.22 (m, 1H), 7.18 – 7.16 (m, 2H), 6.79 – 6.76 (m, 2H), 6.70 – 6.66 (m, 2H), 4.99 – 4.96 (m, 1H), 4.21 (br, 1H), 3.74 (s, 3H), 2.91

– 2.76 (m, 2H), 2.30 – 2.22 (m, 1H), 1.99 – 1.90 (m, 1H).

^{13}C NMR (100 MHz, $CDCl_3$) δ 201.2, 152.6, 141.4, 141.0, 135.0, 133.4, 128.7, 128.6, 128.4, 128.2, 126.1, 115.5, 114.9, 58.4, 55.6, 34.9, 31.6.

HRMS (ES+) Calcd for $C_{23}H_{23}NO_2Na$ $[M+Na]^+$: 368.1621, found: 368.1638.

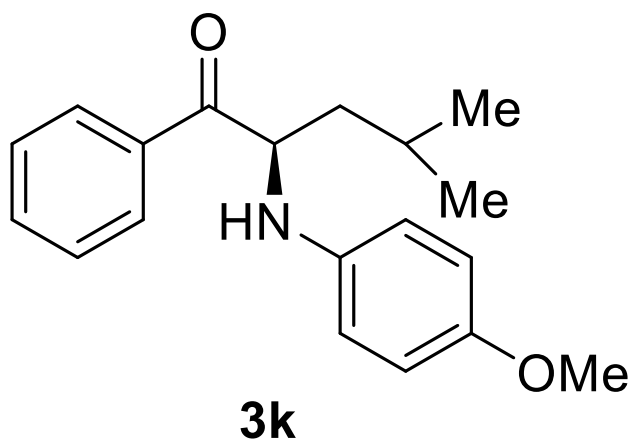

3k

(*R*)-2-((4-Methoxyphenyl)amino)-4-methyl-1-phenylpentan-1-one (3k) was prepared as a green oil from (4-methyl-1-oxo-1-phenylpentan-2-yl)diphenylsulfonium tetrafluoroborate (179.3 mg, 0.4 mmol), (*R*)-**B2** (2.8 mg, 1.0 mol%), K_2CO_3 (55.3 mg, 0.4 mmol) and *p*-anisidine (54.2 mg, 0.44 mmol) in toluene (4 mL) and water (4 mL) according to the General Procedure E (12 h, eluent: hexanes/ethyl acetate = 10:1 → 5:1, 87.0 mg, 73% yield, 97% ee).

$[\alpha]_D^{20}$: –31.3 (c = 1.0, CH_2Cl_2). HPLC analysis of the product: Daicel CHIRALPAK IC column; 3% *i*-PrOH in hexanes; 1.0 mL/min; retention times: 17.1 min (minor), 20.8 min (major).

^{1}H NMR (400 MHz, $CDCl_3$) δ 8.02 – 8.00 (m, 2H), 7.62 – 7.57 (m, 1H), 7.51 – 7.48 (m, 2H), 6.76 – 6.73 (m, 2H), 6.67 – 6.65 (m, 2H), 5.03 – 5.00 (m, 1H), 4.16 (br, 1H), 3.71 (s, 3H), 2.05 – 1.97 (m, 1H), 1.70 – 1.63 (m, 1H), 1.57 – 1.50 (m, 1H), 1.08 (d, J = 6.6 Hz, 3H), 0.96 (d, J = 6.6 Hz, 3H).

^{13}C NMR (100 MHz, $CDCl_3$) δ 202.1, 152.6, 141.7, 135.3, 133.4, 128.8, 128.1, 115.5, 114.8, 57.9, 55.6, 42.7, 25.1, 23.3, 21.8.

HRMS (ES+) Calcd for $C_{19}H_{23}NO_2Na$ $[M+Na]^+$: 320.1621, found: 320.1630.

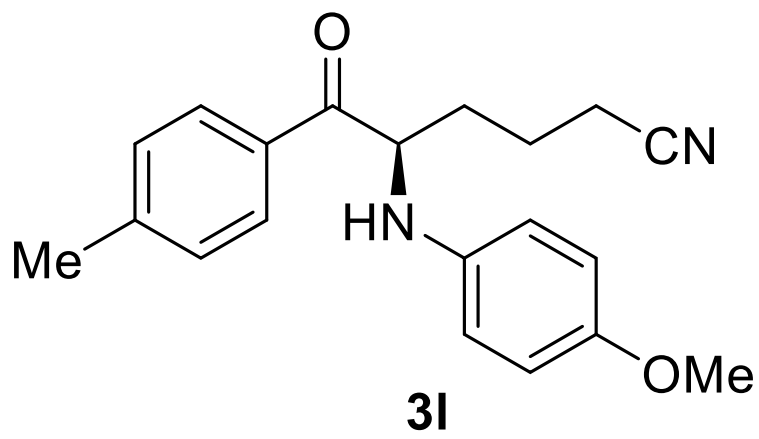

3l

(*R*)-5-((4-Methoxyphenyl)amino)-6-oxo-6-(*p*-tolyl)hexanenitrile (3l) was prepared as a green oil from (4-cyano-1-oxo-1-(*p*-tolyl)butan-2-yl)diphenylsulfonium tetrafluoroborate (194.9 mg, 0.4 mmol), (*R*)-**B2** (2.8 mg, 1.0 mol%), K_2CO_3 (55.3 mg, 0.4 mmol) and *p*-anisidine (54.2 mg, 0.44 mmol) in toluene (4 mL) and saturated brine (4 mL) according to the General Procedure F (–20 °C, 24 h, eluent: hexanes/ethyl acetate = 10:1 → 5:1, 104.0 mg, 81% yield, >99% ee).

$[\alpha]_D^{22}$: –23.6 (c = 1.0, CH_2Cl_2). HPLC analysis of the product: Daicel CHIRALCEL OD-H column; 10% *i*-PrOH in hexanes; 1.0 mL/min; retention times: 36.8 min (major).

^{1}H NMR (400 MHz, $CDCl_3$) δ 7.88 (d, J = 8.2 Hz, 2H), 7.30 (d, J = 8.0 Hz, 2H), 6.77 – 6.75 (m, 2H), 6.67 – 6.65 (m, 2H), 5.00 – 4.97 (m, 1H), 4.42 (s, 1H), 3.72 (s, 3H), 2.43 (s, 3H), 2.33 (t, J = 6.9 Hz, 2H). 2.12 – 2.04 (m, 1H), 1.85 – 1.72 (m, 3H).

^{13}C NMR (100 MHz, $CDCl_3$) δ 199.8, 152.7, 144.9, 140.9, 132.2, 129.6, 128.4, 119.2, 115.4, 114.9, 58.2, 55.6, 32.0, 21.6, 21.3, 17.0.

HRMS (ES+) Calcd for $C_{20}H_{23}N_2O_2$ $[M+H]^+$: 323.1754, found: 323.1768.

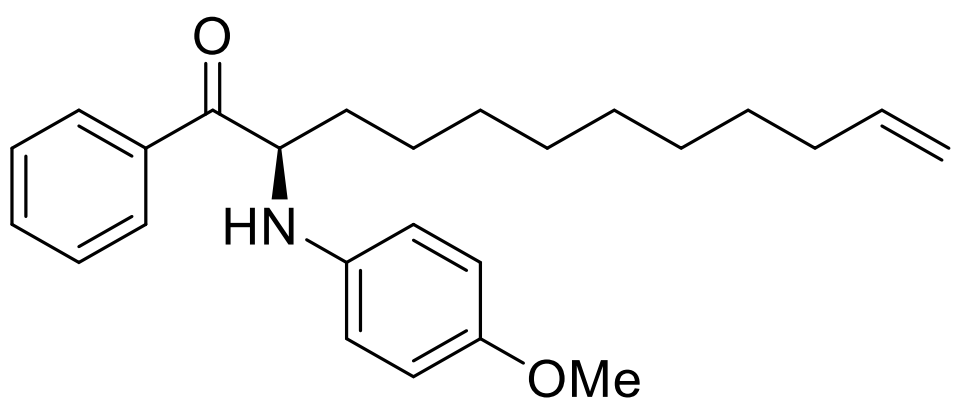

3m

(*R*)-2-((4-Methoxyphenyl)amino)-1-phenyldodec-11-en-1-one (3m) was prepared as a green oil from (1-oxo-1-phenyldodec-11-en-2-yl)diphenyl-sulfonium tetrafluoroborate (212.1 mg, 0.4 mmol), (*R*)-**B2** (2.8 mg, 1.0 mol%),

K_2CO_3 (55.3 mg, 0.4 mmol) and *p*-anisidine (54.2 mg, 0.44 mmol) in toluene (4 mL) and saturated brine (4 mL) according to the General Procedure F (12 h, eluent: hexanes/ethyl acetate = 10:1 → 5:1, 131.9 mg, 87% yield, 91% ee).

$[\alpha]_D^{22}$: –30.7 (c = 1.0, CH_2Cl_2). HPLC analysis of the product: Daicel CHIRALPAK IC column; 3% *i*-PrOH in hexanes; 1.0 mL/min; retention times: 18.3 min (minor), 23.5 min (major).

^{1}H NMR (400 MHz, $CDCl_3$) δ 8.01 – 7.99 (m, 2H), 7.62 – 7.59 (m, 1H), 7.52 – 7.48 (m, 2H), 6.79 – 6.75 (m, 2H), 6.69 – 6.65 (m, 2H), 5.86 – 5.76 (m, 1H), 5.02 – 4.92 (m, 3H), 4.39 (s, 1H), 3.73 (s, 3H), 2.05 – 1.90 (m, 3H), 1.69 – 1.60 (m, 1H), 1.48 – 1.24 (m, 12H).

^{13}C NMR (100 MHz, $CDCl_3$) δ 201.3, 152.4, 141.4, 139.1, 135.3, 133.4, 128.8, 128.2, 115.2, 114.8, 114.1, 59.2, 55.6, 33.7, 33.3, 29.4, 29.2 (2C), 29.0, 28.8, 25.3.

HRMS (ES+) Calcd for $C_{25}H_{34}NO_2$ $[M+H]^+$: 380.2584, found: 380.2595.

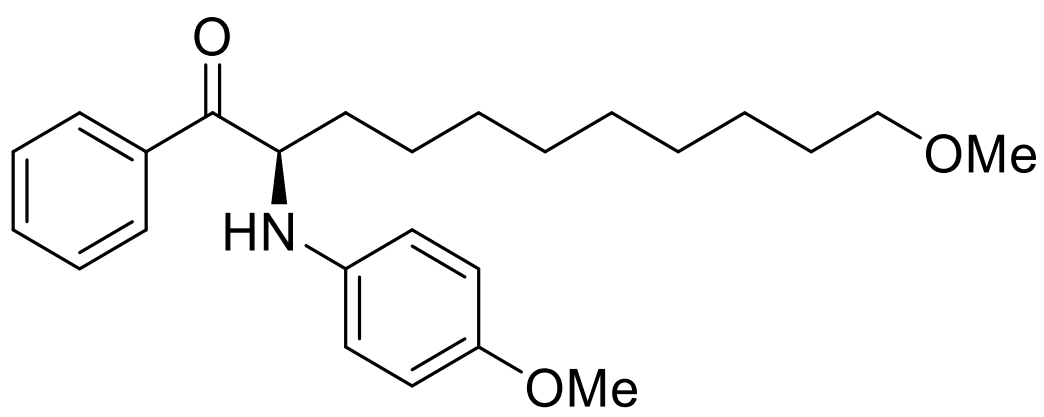

3n

(*R*)-11-Methoxy-2-((4-methoxyphenyl)amino)-1-phenylundecan-1-one (3n) was prepared as a green oil from (11-methoxy-1-oxo-1-phenylundecan-2-yl)diphenylsulfonium tetrafluoroborate (219.3 mg, 0.4 mmol), (*R*)-**B2** (2.8 mg, 1.0 mol%), K_2CO_3 (55.3 mg, 0.4 mmol) and *p*-anisidine (54.2 mg, 0.44 mmol) in toluene (4 mL) and saturated brine (4 mL) according to the General Procedure F (24 h, eluent: hexanes/ethyl acetate = 10:1 → 5:1, 128.6 mg, 81% yield, 97% ee).

$[\alpha]_D^{22}$: –30.6 (c = 1.0, CH_2Cl_2). HPLC analysis of the product: Daicel CHIRALPAK IC column; 10% *i*-PrOH in hexanes; 1.0 mL/min; retention times: 19.8 min (minor), 24.9 min (major).

^{1}H NMR (400 MHz, $CDCl_3$) δ 8.00 – 7.97 (m, 2H), 7.60 – 7.56 (m, 1H), 7.50 – 7.46

(m, 2H), 6.76 – 6.73 (m, 2H), 6.68 – 6.64 (m, 2H), 4.99 – 4.96 (m, 1H), 4.39 (s, 1H), 3.71 (s, 3H), 3.36 – 3.32 (m, 5H), 1.96 – 1.90 (m, 1H), 1.65 – 1.23 (m, 15H).

^{13}C NMR (100 MHz, $CDCl_3$) δ 201.3, 152.3, 141.4, 135.3, 133.3, 128.7, 128.1, 115.2, 114.8, 72.8, 59.1, 58.4, 55.5, 33.2, 29.5, 29.34, 29.27 (2C), 29.2, 26.0, 25.2.

HRMS (ES+) Calcd for $C_{25}H_{36}NO_3$ $[M+H]^+$: 398.2690, found: 398.2695.

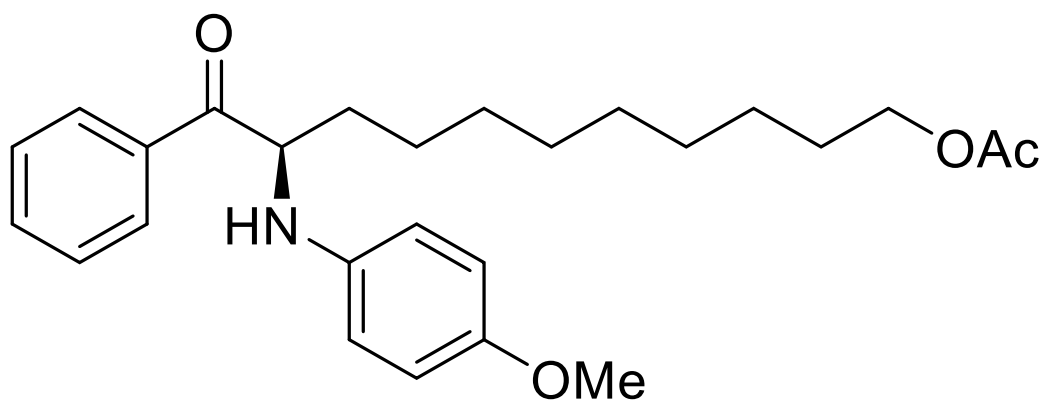

3o

(*R*)-10-((4-Methoxyphenyl)amino)-11-oxo-11-phenylundecyl acetate (3o) was prepared as a green oil from (11-acetoxy-1-oxo-1-phenylundecan-2-yl)diphenylsulfonium tetrafluoroborate (230.5 mg, 0.4 mmol), (*R*)-**B2** (2.8 mg, 1.0 mol%), K_2CO_3 (55.3 mg, 0.4 mmol) and *p*-anisidine (54.2 mg, 0.44 mmol) in toluene (4 mL) and saturated brine (4 mL) according to the General Procedure F (12 h, eluent: hexanes/ethyl acetate = 10:1 → 5:1, 150.0 mg, 88% yield, 90% ee). $[\alpha]_D^{22}$: –19.5 (c = 1.0, CH_2Cl_2). HPLC analysis of the product: Daicel CHIRALCEL OD-H column; 7% *i*-PrOH in hexanes; 1.0 mL/min; retention times: 14.9 min (minor), 18.9 min (major).

^{1}H NMR (400 MHz, $CDCl_3$) δ 7.99 – 7.97 (m, 2H), 7.62 – 7.58 (m, 1H), 7.51 – 7.48 (m, 2H), 6.77 – 6.73 (m, 2H), 6.67 – 6.63 (m, 2H), 4.98 – 4.95 (m, 1H), 4.03 (t, J = 6.7 Hz, 2H), 3.72 (s, 3H), 2.04 (s, 3H), 1.95 – 1.88 (m, 1H), 1.66 – 1.55 (m, 3H), 1.43 – 1.38 (m, 2H), 1.25 – 1.22 (m, 11H).

^{13}C NMR (100 MHz, $CDCl_3$) δ 201.4, 171.2, 152.5, 141.4, 135.4, 133.5, 128.8, 128.3, 115.4, 114.9, 64.6, 59.3, 55.7, 33.4, 29.4, 29.3, 29.2, 29.1, 28.5, 25.8, 25.3, 21.0.

HRMS (ES+) Calcd for $C_{26}H_{36}NO_4$ $[M+H]^+$: 426.2639, found: 426.2647.

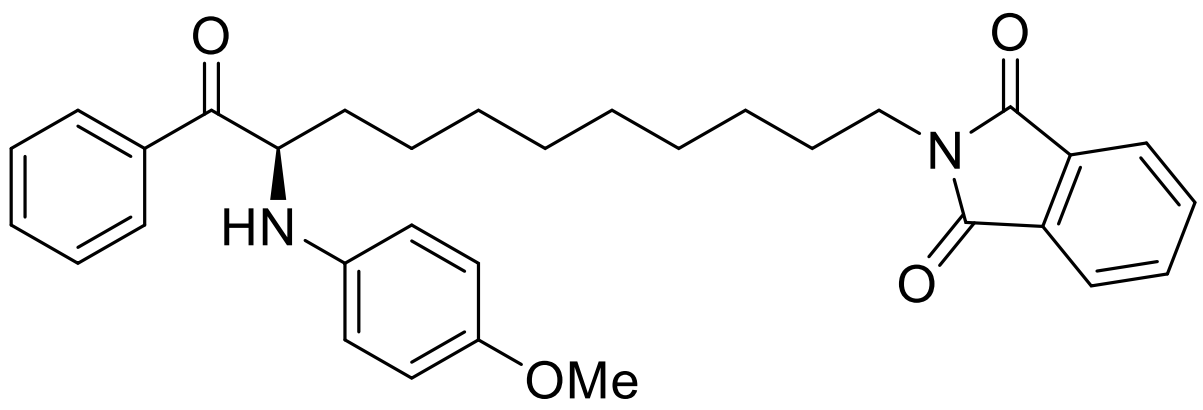

3p

(*R*)-2-(10-((4-Methoxyphenyl)amino)-11-oxo-11-phenylundecyl)isoindoline-1,3-dione (3p) was prepared as a green oil from (11-(1,3-dioxoisoindolin-2-yl)-1-oxo-1-phenylundecan-2-yl)diphenylsulfo-nium tetrafluoroborate (265.3 mg, 0.4 mmol), (*R*)-**B2** (2.8 mg, 1.0 mol%), K_2CO_3 (55.3 mg, 0.4 mmol) and *p*-anisidine (54.2 mg, 0.44 mmol) in toluene (4 mL) and saturated brine (4 mL) according to the General Procedure F (12 h, eluent: hexanes/ethyl acetate = 10:1 → 5:1, 183.0 mg, 89% yield, 96% ee).

$[\alpha]_D^{22}$: –17.9 (c = 1.0, CH_2Cl_2). HPLC analysis of the: Daicel CHIRALPAK AD-H column; 30% *i*-PrOH in hexanes; 1.0 mL/min; retention times: 37.4 min (major), 46.4 min (minor).

^{1}H NMR (400 MHz, $CDCl_3$) δ 7.98 – 7.96 (m, 2H), 7.84 – 7.82 (m, 2H), 7.70 – 7.68 (m, 2H), 7.61 – 7.57 (m, 1H), 7.51 – 7.47 (m, 2H), 6.75 – 6.72 (m, 2H), 6.66 – 6.63 (m, 2H), 4.97 – 4.94 (m, 1H), 4.34 (br, 1H), 3.71 (s, 3H), 3.65 (t, J = 7.3 Hz, 2H), 1.95 – 1.86 (m, 1H), 1.65 – 1.57 (m, 3H), 1.43 – 1.37 (m, 2H), 1.27 – 1.21 (m, 10H).

^{13}C NMR (100 MHz, $CDCl_3$) δ 201.4, 168.4, 152.5, 141.5, 135.4, 133.8, 133.4, 132.1, 128.8, 128.3, 123.1, 115.3, 114.9, 59.3, 55.7, 38.0, 33.3, 29.4, 29.23, 29.21, 29.0, 28.5, 26.7, 25.3.

HRMS (ES+) Calcd for $C_{32}H_{36}N_2O_4$ $[M]^+$: 512.2670, found: 512.2670.

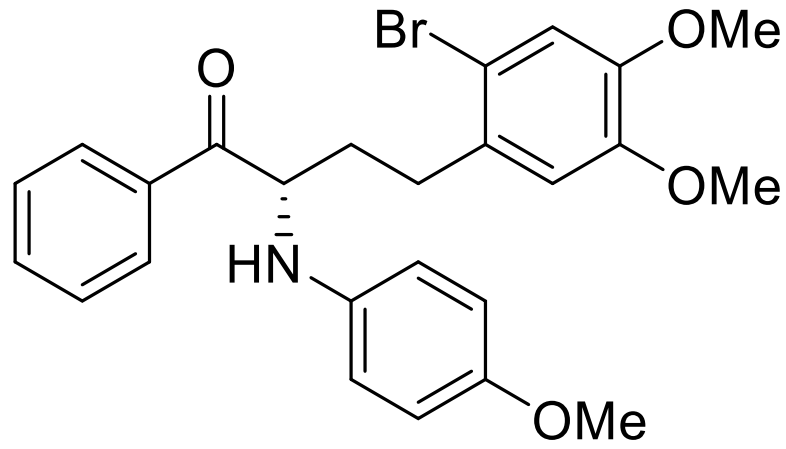

3q

(*S*)-4-(2-Bromo-4,5-dimethoxyphenyl)-2-((4-methoxyphenyl)amino)-1-phen-

ylbutan-1-one (3q) was prepared as a green oil from (4-(2-bromo-4,5-dimethoxyphenyl)-1-oxo-1-phenylbutan-2-yl)diphenyl-sulfonium tetrafluoroborate (1.14 g, 1.8 mmol), (*S*)- **B2** (12.6 mg, 1.0 mol%), K_2CO_3 (250.4 mg, 1.8 mmol) and *p*-anisidine (245.2 mg, 2.0 mmol) in toluene (18 mL) and saturated brine (18 mL) according to the General Procedure F (–20 °C, 24 h, eluent: hexanes/ethyl acetate = 8:1 → 3:1, 739.6 mg, 85% yield, 91% ee).

$[\alpha]_D^{22}$: +8.0 (*c* = 1.0, CH_2Cl_2). HPLC analysis of the product: Daicel CHIRALPAK IC column; 15% *i*-PrOH in hexanes; 1.0 mL/min; retention times: 27.3 min (major), 33.8 min (minor).

^{1}H NMR (400 MHz, $CDCl_3$) δ 7.88 – 7.86 (m, 2H), 7.59 – 7.56 (m, 1H), 7.47 – 7.43 (m, 2H), 6.98 (s, 1H), 6.76 – 6.73 (m, 2H), 6.66 – 6.63 (m, 3H), 4.93 – 4.90 (m, 1H), 4.29 (br, 1H), 3.85 (s, 3H), 3.71 (s, 3H), 3.69 (s, 3H), 2.86 (t, *J* = 7.6 Hz, 2H), 2.32 – 2.28 (m, 1H), 1.87 – 1.82 (m, 1H).

^{13}C NMR (100 MHz, $CDCl_3$) δ 201.0, 152.7, 148.3, 148.0, 141.3, 135.0, 133.5, 132.0, 128.8, 128.3, 115.48, 115.46, 114.9, 113.9, 113.4, 58.4, 56.1, 55.8, 55.7, 33.1, 32.0.

HRMS (ES+) Calcd for $C_{25}H_{26}BrNO_4$ $[M]^+$: 483.1040, found: 483.1041.

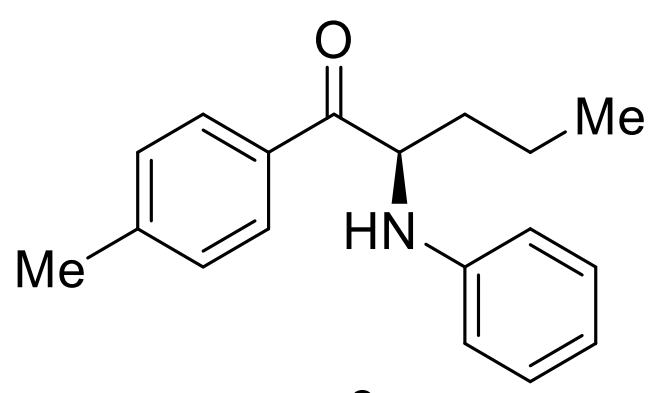

3r

(*R*)-2-(Phenylamino)-1-(*p*-tolyl)pentan-1-one (3r) was prepared as a green oil from (1-oxo-1-(*p*-tolyl)pentan-2-yl)diphenylsulfonium tetrafluoroborate (179.2 mg, 0.4 mmol), (*R*)-**B2** (2.8 mg, 1.0 mol%), K_2CO_3 (55.3 mg, 0.4 mmol) and aniline (40.1 μL, 0.44 mmol) in toluene (4 mL) and water (4 mL) according to the General Procedure E (5 h, eluent: hexanes/ethyl acetate = 10:1 → 5:1, 97.3 mg, 91% yield, 97% ee).

$[\alpha]_D^{20}$: –64.2 (*c* = 1.0, CH_2Cl_2). HPLC analysis of the product: Daicel CHIRALPAK IC column; 3% *i*-PrOH in hexanes; 1.0 mL/min; retention times:

10.2 min (major), 11.7 min (minor).

^{1}H NMR (400 MHz, $CDCl_3$) δ 7.92 (d, J = 8.2 Hz, 2H), 7.31 (d, J = 8.0 Hz, 2H), 7.17 (t, J = 7.9 Hz, 2H), 6.73 – 6.69 (m, 3H), 5.09 – 5.06 (m, 1H), 4.71 (br, 1H), 2.48 (s, 3H), 2.00 – 1.91 (m, 1H), 1.72 – 1.63 (m, 1H), 1.52 – 1.35 (m, 2H), 0.90 (t, J = 7.3 Hz, 3H).

^{13}C NMR (100 MHz, $CDCl_3$) δ 200.3, 147.2, 144.4, 132.7, 129.5, 129.3, 128.4, 117.8, 113.5, 57.6, 35.5, 21.7, 18.5, 14.0.

HRMS (ES+) Calcd for $C_{18}H_{22}NO$ $[M+H]^+$: 268.1696, found: 268.1709.

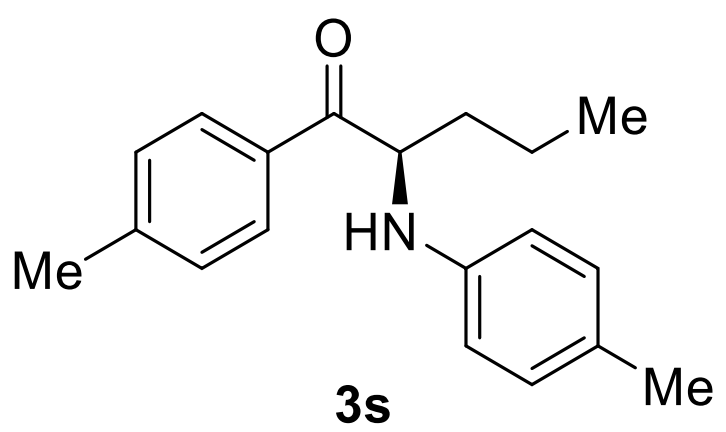

3s

(*R*)-1-(*p*-Tolyl)-2-(*p*-tolylamino)pentan-1-one (3s) was prepared as a green solid from (1-oxo-1-(*p*-tolyl)pentan-2-yl)diphenylsulfonium tetrafluoroborate (179.2 mg, 0.4 mmol), (*R*)-**B2** (2.8 mg, 1.0 mol%), K_2CO_3 (55.3 mg, 0.4 mmol) and *p*-toluidine (47.1 mg, 0.44 mmol) in toluene (4 mL) and water (4 mL) according to the General Procedure E (5 h, eluent: hexanes/ethyl acetate = 10:1 → 5:1, 106.8 mg, 95% yield, 97% ee).

$[\alpha]_D^{20}$: –58.7 (c = 1.0, CH_2Cl_2). HPLC analysis of the product: Daicel CHIRALPAK IC column; 2% *i*-PrOH in hexanes; 1.0 mL/min; retention times: 15.6 min (major), 17.6 min (minor).

^{1}H NMR (400 MHz, $CDCl_3$) δ 7.93 (d, J = 8.2 Hz, 2H), 7.31 (d, J = 8.0 Hz, 2H), 6.99 (d, J = 8.1 Hz, 2H), 6.65 – 6.63 (m, 2H), 5.07 – 5.04 (m, 1H), 4.55 (br, 1H), 2.45 (s, 3H), 2.44 (s, 3H), 1.99 – 1.90 (m, 1H), 1.72 – 1.63 (m, 1H), 1.54 – 1.37 (m, 2H), 0.91 (t, J = 7.3 Hz, 3H).

^{13}C NMR (100 MHz, $CDCl_3$) δ 200.6, 145.0, 144.3, 132.8, 129.7, 129.4, 128.4, 127.0, 113.7, 58.0, 35.5, 21.6, 20.3, 18.6, 14.0.

HRMS (ES+) Calcd for $C_{19}H_{24}NO$ $[M+H]^+$: 282.1852, found: 282.1867.

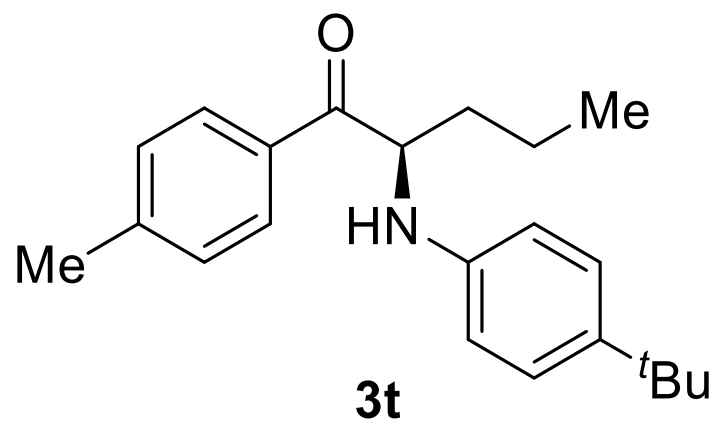

3t

(*R*)-2-((4-(*tert*-Butyl)phenyl)amino)-1-(*p*-tolyl)pentan-1-one (3t) was prepared as a green soild from (1-oxo-1-(*p*-tolyl)pentan-2-yl)diphenylsulfonium tetrafluoroborate (179.2 mg, 0.4 mmol), (*R*)-**B2** (2.8 mg, 1.0 mol%), K_2CO_3 (55.3 mg, 0.4 mmol) and 4-(*tert*-butyl)aniline (70.4 μL, 0.44 mmol) in toluene (4 mL) and water (4 mL) according to the General Procedure E (5 h, eluent: hexanes/ethyl acetate = 10:1 → 5:1, 120.1 mg, 93% yield, 96% ee). $[\alpha]_D^{20}$: –56.1 (*c* = 1.0, CH_2Cl_2). HPLC analysis of the product: Daicel CHIRALPAK IC column; 2% *i*-PrOH in hexanes; 1.0 mL/min; retention times: 10.3 min (minor), 18.4 min (major).

^{1}H NMR (400 MHz, $CDCl_3$) *δ* 7.93 (d, *J* = 8.2 Hz, 2H), 7.31 (d, *J* = 8.0 Hz, 2H), 7.22 – 7.19 (m, 2H), 6.67 – 6.65 (m, 2H), 5.06 – 5.03 (m, 1H), 4.61 (br, 1H), 2.44 (s, 3H), 1.98 – 1.90 (m, 1H), 1.71 – 1.62 (m, 1H), 1.53 – 1.38 (m, 2H), 1.28 (s, 9H), 0.91 (t, *J* = 7.3 Hz, 3H).

^{13}C NMR (100 MHz, $CDCl_3$) *δ* 200.6, 144.9, 144.3, 140.5, 132.7, 129.5, 128.4, 126.1, 113.2, 57.9, 35.7, 33.8, 31.5, 21.7, 18.6, 14.0.

HRMS (ES+) Calcd for $C_{22}H_{30}NO$ $[M+H]^+$: 324.2322, found: 324.2336.

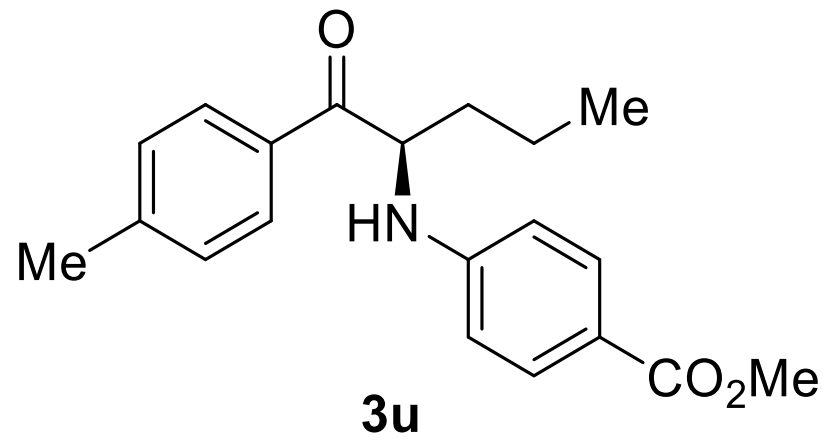

3u

Methyl (*R*)-4-((1-oxo-1-(*p*-tolyl)pentan-2-yl)amino)benzoate (3u) was prepared as a green oil from (1-oxo-1-(*p*-tolyl)pentan-2-yl)diphenylsulfonium tetrafluoroborate (179.2 mg, 0.4 mmol), (*R*)-**B2** (14.0 mg, 5.0 mol%), K_2CO_3 (55.3

mg, 0.4 mmol) and methyl 4-aminobenzoate (66.5 mg, 0.44 mmol) in toluene (4 mL) and water (4 mL) according to the General Procedure E (12 h, eluent: hexanes/ethyl acetate = 10:1 → 3:1, 118.3 mg, 91% yield, 97% ee).

$[\alpha]_D^{20}$: –79.5 (c = 1.0, CH_2Cl_2). HPLC analysis of the product: Daicel CHIRALPAK IC column; 15% *i*-PrOH in hexanes; 1.0 mL/min; retention times: 16.5 min (minor), 28.3 min (major).

^{1}H NMR (400 MHz, $CDCl_3$) δ 7.90 (d, J = 8.2 Hz, 2H), 7.85 (d, J = 8.8 Hz, 2H), 7.31 (d, J = 8.0 Hz, 2H), 6.63 (d, J = 8.8 Hz, 2H), 5.21 (br, 1H), 5.15 – 5.12 (m, 1H), 3.84 (s, 3H), 2.44 (s, 3H), 2.02 – 1.94 (m, 1H), 1.74 – 1.65 (m, 1H), 1.45 – 1.24 (m, 2H), 0.85 (t, J = 7.3 Hz, 3H).

^{13}C NMR (100 MHz, $CDCl_3$) δ 199.0, 167.2, 150.7, 144.8, 132.2, 131.6, 129.6, 128.5, 118.6, 112.0, 56.7, 51.5, 35.1, 21.7, 18.1, 13.9.

HRMS (ES+) Calcd for $C_{20}H_{24}NO_3$ $[M+H]^+$: 326.1751, found: 326.1764.

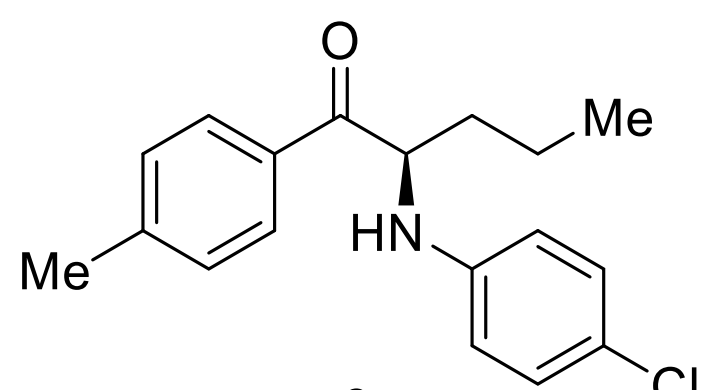

3v

(*R*)-2-((4-Chlorophenyl)amino)-1-(*p*-tolyl)pentan-1-one (3v) was prepared as a green oil from (1-oxo-1-(*p*-tolyl)pentan-2-yl)diphenylsulfonium tetrafluoroborate (179.2 mg, 0.4 mmol), (*R*)-**B2** (2.8 mg, 1.0 mol%), K_2CO_3 (55.3 mg, 0.4 mmol) and 4-chloroaniline (55.9 mg, 0.44 mmol) in toluene (4 mL) and water (4 mL) according to the General Procedure E (8 h, eluent: hexanes/ethyl acetate = 10:1 → 5:1, 114.3 mg, 95% yield, 96% ee).

$[\alpha]_D^{20}$: –67.3 (c = 1.0, CH_2Cl_2). HPLC analysis of the product: Daicel CHIRALPAK IC column; 2% *i*-PrOH in hexanes; 1.0 mL/min; retention times: 9.7 min (major), 10.7 min (minor).

^{1}H NMR (400 MHz, $CDCl_3$) δ 7.90 (d, J = 8.2 Hz, 2H), 7.31 (d, J = 8.0 Hz, 2H), 7.11 – 7.08 (m, 2H), 6.61 – 6.58 (m, 2H), 5.02 – 4.99 (m, 1H), 4.68 (br, 1H), 2.44 (s,

3H), 1.97 – 1.89 (m, 1H), 1.70 – 1.61 (m, 1H), 1.48 – 1.32 (m, 2H), 0.88 (t, *J* = 7.3 Hz, 3H).

^{13}C NMR (100 MHz, $CDCl_3$) δ 199.9, 145.8, 144.7, 132.5, 129.6, 129.1, 128.4, 122.3, 114.6, 57.7, 35.3, 21.7, 18.4, 13.9.

HRMS (ES+) Calcd for $C_{18}H_{21}ClNO$ $[M+H]^+$: 302.1306, found: 302.1324.

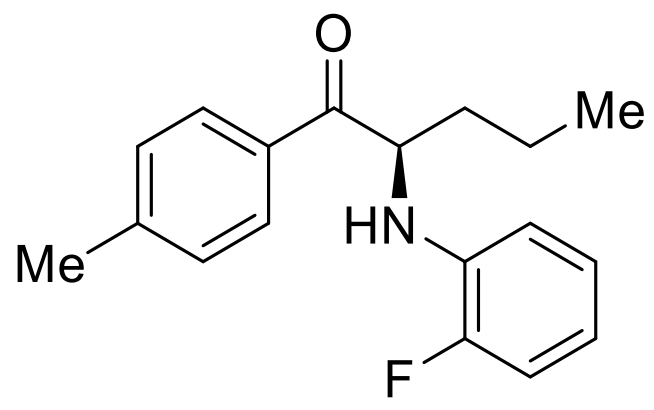

3w

(*R*)-2-((2-Fluorophenyl)amino)-1-(*p*-tolyl)pentan-1-one (3w) was prepared as a green oil from (1-oxo-1-(*p*-tolyl)pentan-2-yl)diphenylsulfonium tetrafluoroborate (179.2 mg, 0.4 mmol), (*R*)-**B2** (14.0 mg, 5.0 mol%), K_2CO_3 (55.3 mg, 0.4 mmol) and 2-fluoroaniline (42.5 μL, 0.44 mmol) in toluene (4 mL) and water (4 mL) according to the General Procedure E (12 h, eluent: hexanes/ethyl acetate = 10:1 → 5:1, 101.5 mg, 89% yield, 93% ee).

$[\alpha]_D^{20}$: –64.1 (*c* = 1.0, CH_2Cl_2). HPLC analysis of the product: Daicel CHIRALCEL OD-H column; 2% *i*-PrOH in hexanes; 1.0 mL/min; retention times: 7.0 min (major), 9.7 min (minor).

^{1}H NMR (400 MHz, $CDCl_3$) δ 7.92 (d, *J* = 8.2 Hz, 2H), 7.31 (d, *J* = 8.0 Hz, 2H), 7.01 – 6.91 (m, 2H), 6.70 – 6.60 (m, 2H), 5.09 – 5.05 (m, 1H), 4.87 – 4.86 (m, 1H), 2.44 (s, 3H), 2,01 – 1.92 (m, 1H), 1.75 – 1.64 (m, 1H), 1.53 – 1.38 (m, 2H), 0.91 (t, *J* = 7.3 Hz, 3H).

^{13}C NMR (100 MHz, $CDCl_3$) δ 199.7, 151.9 (d, *J* = 237.9 Hz), 144.5, 135.7 (d, *J* = 11.5 Hz), 132.6, 129.5, 128.4, 124.4 (d, *J* = 3.5 Hz), 117.2 (d, *J* = 6.9 Hz), 114.8 (d, *J* = 18.5 Hz), 112.7 (d, *J* = 3.1 Hz), 57.4, 35.4, 21.7, 18.5, 13.9.

^{19}F NMR (376 MHz, $CDCl_3$) δ –135.0.

HRMS (ES+) Calcd for $C_{18}H_{21}FNO$ $[M+H]^+$: 286.1602, found: 286.1617.

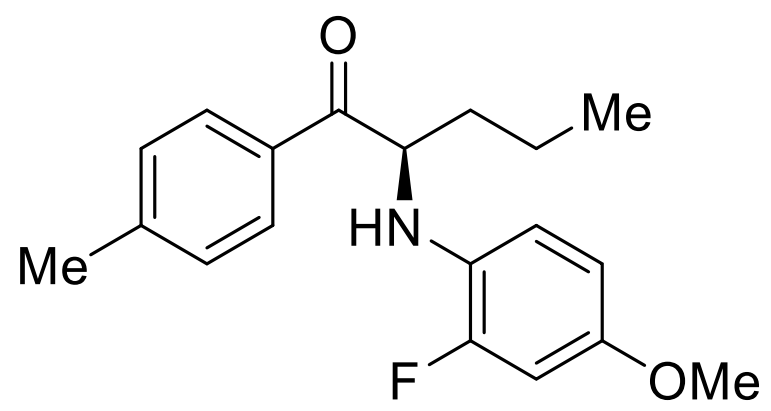

3x

(*R*)-2-((2-Fluoro-4-methoxyphenyl)amino)-1-(*p*-tolyl)pentan-1-one (3x) was prepared as a green soild from (1-oxo-1-(*p*-tolyl)pentan-2-yl)diphenylsulfonium tetrafluoroborate (179.2 mg, 0.4 mmol), (*R*)-**B2** (2.8 mg, 1.0 mol%), K_2CO_3 (55.3 mg, 0.4 mmol) and 2-fluoro-4-methoxyaniline (62.1 mg, 0.44 mmol) in toluene (4 mL) and water (4 mL) according to the General Procedure E (8 h, eluent: hexanes/ethyl acetate = 10:1 → 5:1, 116.0 mg, 92% yield, 95% ee).

$[\alpha]_D^{20}$: –66.3 (*c* = 1.0, CH_2Cl_2). HPLC analysis of the product: Daicel CHIRALPAK IC column; 3% *i*-PrOH in hexanes; 1.0 mL/min; retention times: 15.8 min (minor), 18.1 min (major).

^{1}H NMR (400 MHz, $CDCl_3$) δ 7.90 (d, *J* = 8.2 Hz, 2H), 7.29 (d, *J* = 8.0 Hz, 2H), 6.66 – 6.61 (m, 2H), 6.53 – 6.49 (m, 1H), 5.00 – 4.97 (m, 1H), 4.14 (br, 1H), 3.70 (s, 3H), 2.42 (s, 3H), 1.93 – 1.86 (m, 1H), 1.70 – 1.62 (m, 1H), 1.50 – 1.42 (m, 2H), 0.91 (t, *J* = 7.3 Hz, 3H).

^{13}C NMR (100 MHz, $CDCl_3$) δ 200.4, 152.6 (d, *J* = 234.0 Hz), 152.2 (d, *J* = 9.7 Hz), 144.4, 132.7, 129.54 (d, *J* = 9.9 Hz), 129.49, 128.4, 114.5 (d, *J* = 4.5 Hz), 109.3 (d, *J* = 3.2 Hz), 102.6 (d, *J* = 22.6 Hz), 58.5, 55.8, 35.6, 21.6, 18.7, 13.9.

^{19}F NMR (376 MHz, $CDCl_3$) δ –131.0.

HRMS (ES+) Calcd for $C_{19}H_{23}FNO_2$ $[M+H]^+$: 316.1707, found: 316.1721.

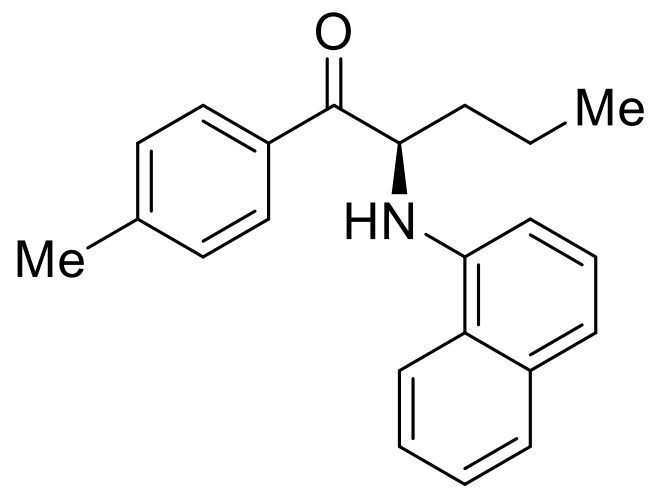

3y

(*R*)-2-(Naphthalen-1-ylamino)-1-(*p*-tolyl)pentan-1-one (3y) was prepared as a black oil from (1-oxo-1-(*p*-tolyl)pentan-2-yl)diphenylsulfonium tetrafluoroborate (179.2 mg, 0.4 mmol), (*R*)-**B2** (2.8 mg, 1.0 mol%), K_2CO_3 (55.3 mg, 0.4 mmol) and naphthalen-1-amine (63.0 mg, 0.44 mmol) in toluene (4 mL) and water (4 mL) according to the General Procedure E (12 h, eluent: hexanes/ethyl acetate = 10:1 → 5:1, 114.1 mg, 90% yield, 99% ee).

$[\alpha]_D^{20}$: –152.2 (*c* = 1.0, CH_2Cl_2). HPLC analysis of the product: Daicel CHIRALPAK IC column; 0.7% *i*-PrOH in hexanes; 1.0 mL/min; retention times: 31.6 min (minor), 33.2 min (major).

^{1}H NMR (400 MHz, $CDCl_3$) δ 8.11 – 8.08 (m, 1H), 8.01 (d, *J* = 8.2 Hz, 2H), 7.84 – 7.82 (m, 1H), 7.54 – 7.48 (m, 2H), 7.36 – 7.27 (m, 4H), 6.66 (d, *J* = 7.1 Hz, 1H), 5.61 – 5.59 (m, 1H), 5.33 – 5.29 (m, 1H), 2.46 (s, 3H), 2.17 – 2.10 (m, 1H), 1.90 – 1.81 (m, 1H), 1.59 – 1.42 (m, 2H), 0.93 (t, *J* = 7.3 Hz, 3H).

^{13}C NMR (100 MHz, $CDCl_3$) δ 200.2, 144.5, 142.3, 134.5, 132.5, 129.5, 128.50, 128.46, 126.3, 125.8, 124.7, 123.8, 120.3, 117.6, 104.8, 57.5, 35.1, 21.6, 18.4, 14.0.

HRMS (ES+) Calcd for $C_{22}H_{24}NO$ $[M+H]^+$: 318.1852, found: 318.1862.

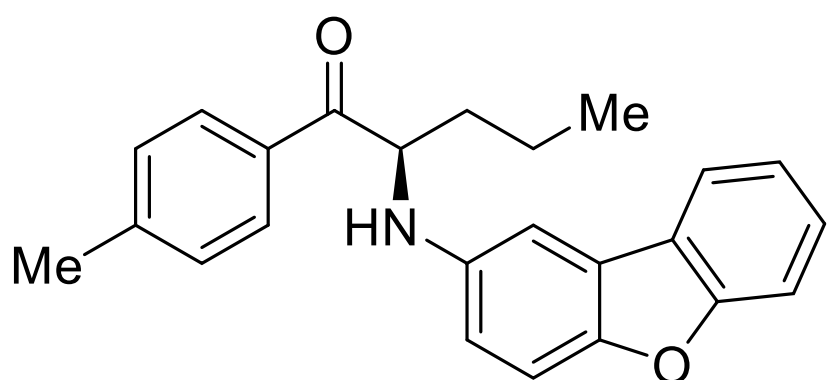

3z

(*R*)-2-(Dibenzo[*b*,*d*]furan-2-ylamino)-1-(*p*-tolyl)pentan-1-one (3z) was prepared as a light yellow solid from (1-oxo-1-(*p*-tolyl)pentan-2-yl)diphenylsulfonium tetrafluoroborate (179.2 mg, 0.4 mmol), (*R*)-**B2** (2.8 mg,

1.0 mol%), K_2CO_3 (55.3 mg, 0.4 mmol) and dibenzo[*b*,*d*]furan-2-amine (81.0 mg, 0.44 mmol) in toluene (4 mL) and water (4 mL) according to the General Procedure E (4 h, eluent: *n*-hexane/ethyl acetate = 20:1, 125.2 mg, 88% yield, 95% ee).

$[\alpha]_D^{24}$: −35.8 (*c* = 2.5, CH_2Cl_2). HPLC analysis of the product: Daicel CHIRALPAK IC column; 5% *i*-PrOH in *n*-hexane; 1.0 mL/min; retention times: 22.2 min (minor), 26.2 min (major).

^{1}H NMR (400 MHz, $CDCl_3$) δ 7.96 – 7.94 (m, 2H), 7.87 – 7.84 (m, 1H), 7.50 (dt, *J* = 8.3, 0.9 Hz, 1H), 7.42 – 7.36 (m, 2H), 7.32 – 7.26 (m, 3H), 7.22 (d, *J* = 2.4 Hz, 1H), 6.87 (dd, *J* = 8.8, 2.5 Hz, 1H), 5.13 (dd, *J* = 6.9, 4.6 Hz, 1H), 4.72 (s, 1H), 2.44 (s, 3H), 2.05 – 1.97 (m, 1H), 1.77 – 1.68 (m, 1H), 1.58 – 1.41 (m, 2H), 0.93 (t, *J* = 7.3 Hz, 3H).

^{13}C NMR (100 MHz, $CDCl_3$) δ 200.8, 156.8, 150.2, 144.7, 143.7, 132.9, 129.7, 128.6, 126.9, 125.0, 124.5, 122.3, 120.6, 115.5, 112.2, 111.7, 104.5, 59.3, 35.7, 21.8, 18.8, 14.2.

HRMS (ES+) Calcd for $C_{24}H_{24}NO_2$ $[M+H]^+$: 358.1802, found: 358.1809.

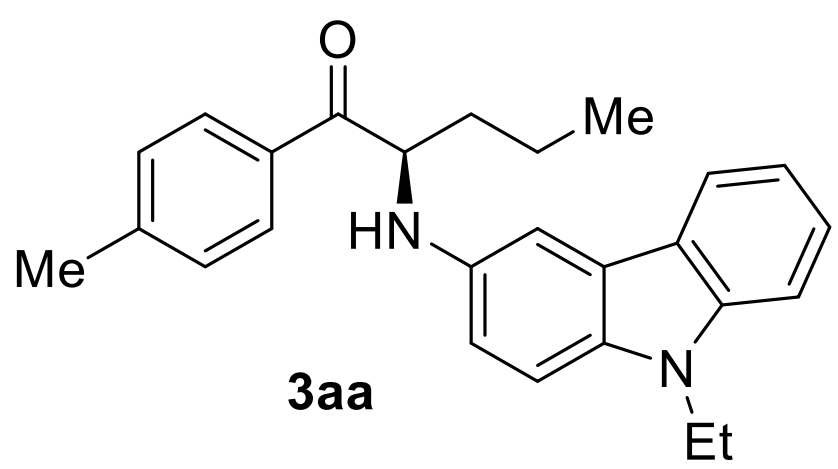

3aa

(*R*)-2-((9-Ethyl-*9H*-carbazol-3-yl)amino)-1-(*p*-tolyl)pentan-1-one (3aa) was prepared as a yellow solid from (1-oxo-1-(*p*-tolyl)pentan-2-yl)diphenylsulfonium tetrafluoroborate (179.2 mg, 0.4 mmol), (*R*)-**B2** (2.8 mg, 1 mol%), K_2CO_3 (55.3 mg, 0.4 mmol) and 9-ethyl-9*H*-carbazol-3-amine (92.9 mg, 0.44 mmol) in toluene (4 mL) and water (4 mL) according to the General Procedure E (4 h, eluent: *n*-hexane/ethyl acetate = 20:1, 139.9 mg, 91% yield, 96% ee).

$[\alpha]_D^{25}$: +3.9 (*c* = 1.4, CH_2Cl_2). HPLC analysis of the product: Daicel CHIRALPAK

IC column; 20% *i*-PrOH in *n*-hexane; 1.0 mL/min; retention times: 14.0 min (minor), 16.6 min (major).

^{1}H NMR (400 MHz, $CDCl_3$) δ 8.01 (dt, *J* = 7.7, 1.0 Hz, 1H), 7.98 – 7.96 (m, 2H), 7.45 – 7.41 (m, 2H), 7.35 – 7.30 (m, 3H), 7.24 (d, *J* = 8.7 Hz, 1H), 7.19 – 7.15 (m, 1H), 7.00 (dd, *J* = 8.6, 2.3 Hz, 1H), 5.17 (dd, *J* = 7.0, 4.6 Hz, 1H), 4.28 (q, *J* = 7.2 Hz, 2H), 2.44 (s, 3H), 2.07 – 1.98 (m, 1H), 1.80 – 1.71 (m, 1H), 1.63 – 1.46 (m, 2H), 1.38 (t, *J* = 7.2 Hz, 3H), 0.96 (t, *J* = 7.3 Hz, 3H).

^{13}C NMR (101 MHz, $CDCl_3$) δ 201.5, 144.4, 140.9, 140.4, 134.5, 133.2, 129.6, 128.6, 125.4, 123.7, 122.7, 120.4, 118.0, 115.8, 109.31 108.4, 105.1, 59.9, 37.6, 35.9, 21.8, 18.9, 14.2, 14.0.

HRMS (ES+) Calcd for $C_{26}H_{29}N_2O$ $[M+H]^+$: 385.2275, found: 385.2272.

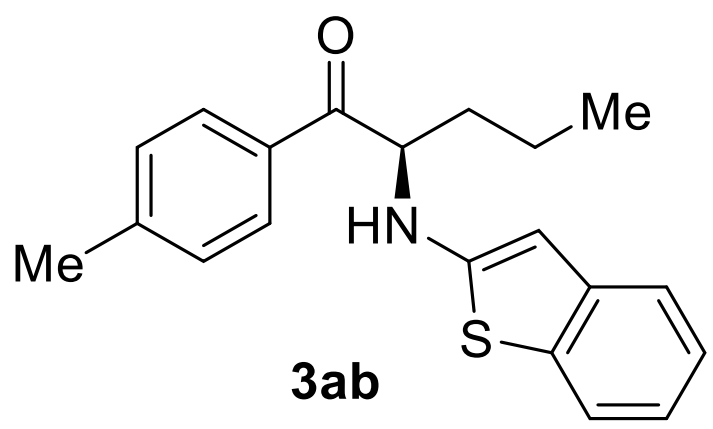

3ab

(*R*)-2-(Benzo[*b*]thiophen-2-ylamino)-1-(*p*-tolyl)pentan-1-one (3ab) was prepared as a yellow oil from (1-oxo-1-(*p*-tolyl)pentan-2-yl)diphenylsulfonium tetrafluoroborate (179.2 mg, 0.4 mmol), (*R*)-**B2** (28.6 mg, 10 mol%), K_2CO_3 (55.3 mg, 0.4 mmol) and benzo[*b*]thiophen-2-amine (69.1 mg, 0.44 mmol) in toluene (4 mL) and water (4 mL) according to the General Procedure E (4 h, eluent: *n*-hexane/ethyl acetate = 40:1, 117.2 mg, 91% yield, 95% ee).

$[\alpha]_D^{25}$: −85.7 (*c* = 1.0, CH_2Cl_2). HPLC analysis of the product: Daicel CHIRALPAK IBN column; 10% *i*-PrOH in *n*-hexane; 1.0 mL/min; retention times: 11.4 min (major), 13.8 min (minor).

^{1}H NMR (400 MHz, $CDCl_3$) δ 7.94 – 7.91 (m, 2H), 7.54 (dd, *J* = 8.0, 1.0 Hz, 1H), 7.40 – 7.37 (m, 1H), 7.32 (d, *J* = 8.0 Hz, 2H), 7.22 – 7.18 (m, 1H), 7.07 – 7.03 (m, 1H), 6.16 (s, 1H), 5.16 (d, *J* = 7.9 Hz, 1H), 5.06 – 5.01 (m, 1H), 2.45 (s, 3H), 2.06 – 1.97 (m, 1H), 1.74 – 1.65 (m, 1H), 1.54 – 1.35 (m, 2H), 0.91 (t, *J* = 7.3 Hz, 3H).

^{13}C NMR (101 MHz, $CDCl_3$) δ 199.4, 152.9, 145.0, 141.0, 132.4, 132.2, 129.8, 128.7, 124.6, 121.6, 121.2, 120.5, 98.4, 61.1, 35.5, 21.9, 18.6, 14.1.

HRMS (ES+) Calcd for $C_{20}H_{21}NNaOS$ $[M+Na]^+$: 346.1237, found: 346.1242.

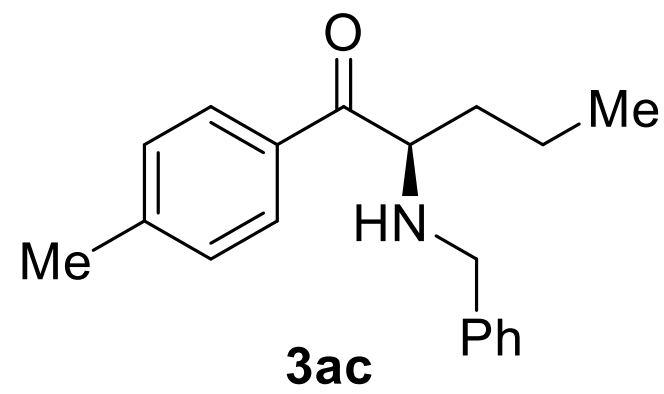

3ac

(*R*)-2-(Benzylamino)-1-(*p*-tolyl)pentan-1-one (3ac) was prepared as a colorless oil from (1-oxo-1-(*p*-tolyl)pentan-2-yl)diphenylsulfonium tetrafluoroborate (179.2 mg, 0.4 mmol), (*R*)-**B2** (28.6 mg, 10 mol%), K_2CO_3 (55.3 mg, 0.4 mmol) and benzylamine (49 μL, 0.44 mmol) in toluene (4 mL) and water (4 mL) according to the General Procedure E (rt for 48 h, eluent: *n*-hexane/ethyl acetate = 40:1 → 20:1 → 10:1, 60.1 mg, 53% yield, 67% ee).

$[\alpha]_D^{25}$: +9.7 (c = 1.2, CH_2Cl_2). HPLC analysis of the product: Daicel CHIRALPAK IC column; 5% *i*-PrOH in *n*-hexane; 1.0 mL/min; retention times: 6.6 min (minor), 7.3 min (major).

^{1}H NMR (400 MHz, $CDCl_3$) δ 7.84 – 7.82 (m, 2H), 7.39 – 7.22 (m, 7H), 4.20 (dd, J = 7.3, 4.6 Hz, 1H), 3.84 (d, J = 13.0 Hz, 1H), 3.58 (d, J = 13.0 Hz, 1H), 2.42 (s, 3H), 2.28 (s, 1H), 1.72 – 1.64 (m, 1H), 1.53 – 1.36 (m, 3H), 0.87 (t, J = 7.2 Hz, 3H).

^{13}C NMR (100 MHz, $CDCl_3$) δ 203.6, 144.3, 140.4, 133.9, 129.5, 128.4, 128.4, 127.1, 61.4, 52.4, 36.4, 21.8, 19.3, 14.1.

HRMS (ES+) Calcd for $C_{19}H_{24}NO$ $[M+H]^+$: 282.1853, found: 282.1854.

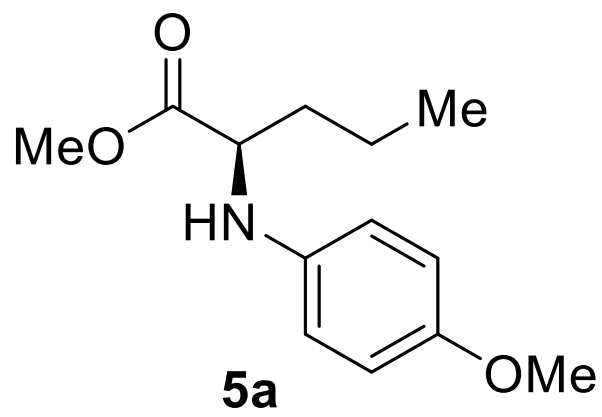

5a

Methyl (*R*)-2-((4-methoxyphenyl)amino)pentanoate (5a) was prepared as a

green oil from (1-methoxy-1-oxopentan-2-yl)diphenylsulfonium tetrafluoroborate (155.3 mg, 0.4 mmol), (*R*)-**B2** (14.0 mg, 5.0 mol%), K_3PO_4 (42.5 mg, 0.2 mmol, 0.5 equiv) and *p*-anisidine (54.2 mg, 0.44 mmol) in toluene (4 mL) and water (4 mL) according to the General Procedure G (18 h, eluent: hexanes/ethyl acetate = 50:1 → 10:1, 86.5 mg, 91% yield, 94% ee).

$[\alpha]_D^{23}$: +52.4 (c = 1.0, CH_2Cl_2). (c = 1.0, CH_2Cl_2). HPLC analysis of the product: Daicel CHIRALCEL OD-H column; 3% *i*-PrOH in hexanes; 1.0 mL/min; retention times: 12.1 min (minor), 12.7 min (major).

^{1}H NMR (400 MHz, $CDCl_3$) δ 6.77 – 6.74 (m, 2H), 6.61 – 6.58 (m, 2H), 3.99 (t, J = 6.6 Hz, 1H), 3.85 (br, 1H), 3.73 (s, 3H), 3.69 (s, 3H), 1.79 – 1.70 (m, 2H), 1.48 – 1.42 (m, 2H), 0.95 (t, J = 7.3 Hz, 3H).

^{13}C NMR (100 MHz, $CDCl_3$) δ 175.0, 152.6, 141.0, 114.9, 114.8, 57.4, 55.5, 51.8, 35.2, 18.9, 13.7.

HRMS (ES+) Calcd for $C_{13}H_{20}NO_3$ $[M+H]^+$: 238.1438, found: 238.1444.

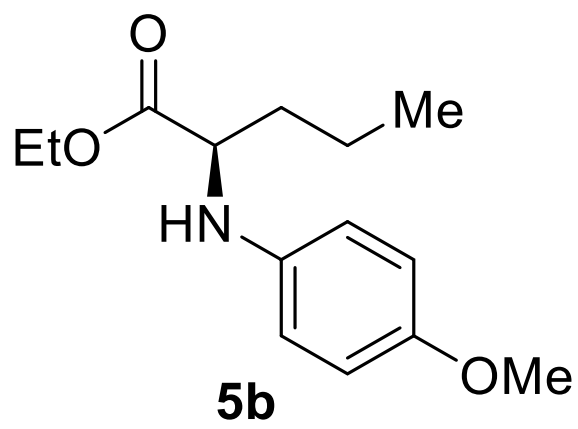

5b

Ethyl (*R*)-2-((4-methoxyphenyl)amino)pentanoate (5b) was prepared as a green oil from (1-ethoxy-1-oxopentan-2-yl)diphenylsulfonium tetrafluoroborate (160.9 mg, 0.4 mmol), (*R*)-**B2** (14.0 mg, 5.0 mol%), K_3PO_4 (42.5 mg, 0.2 mmol, 0.5 equiv) and *p*-anisidine (54.2 mg, 0.44 mmol) in toluene (4 mL) and water (4 mL) according to the General Procedure G (18 h, eluent: hexanes/ethyl acetate = 50:1 → 10:1, 87.5 mg, 87% yield, 96% ee).

$[\alpha]_D^{23}$: +54.9 (c = 1.0, CH_2Cl_2). (c = 1.0, CH_2Cl_2). HPLC analysis of the product: Daicel CHIRALCEL OD-H column; 3% *i*-PrOH in hexanes; 1.0 mL/min; retention times: 9.4 min (minor), 10.7 min (major).

^{1}H NMR (400 MHz, $CDCl_3$) δ 6.78 – 6.74 (m, 2H), 6.62 – 6.58 (m, 2H), 4.16 (q, J

= 7.1 Hz, 2H), 3.97 (t, *J* = 6.6 Hz, 1H), 3.87 (br, 1H), 3.73 (s, 3H), 1.78 – 1.66 (m, 2H), 1.51 – 1.41 (m, 2H), 1.23 (t, *J* = 7.1 Hz, 3H), 0.95 (t, *J* = 7.3 Hz, 3H).

^{13}C NMR (100 MHz, $CDCl_3$) δ 174.5, 152.5, 141.0, 114.9, 114.7, 60.7, 57.5, 55.5, 35.2, 18.9, 14.1, 13.7.

HRMS (ES+) Calcd for $C_{14}H_{22}NO_3$ $[M+H]^+$: 252.1594, found: 252.1603.

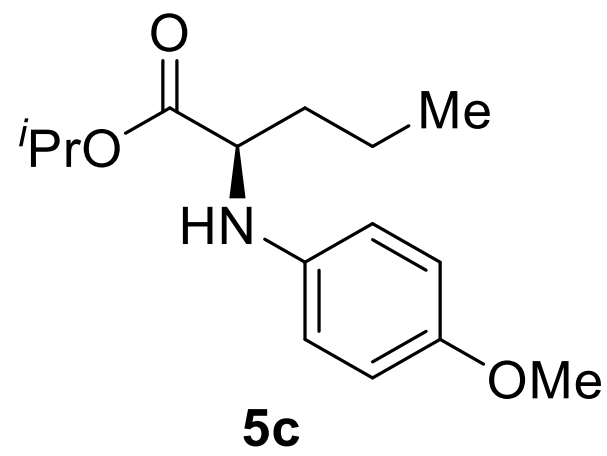

5c

Isopropyl (*R*)-2-((4-methoxyphenyl)amino)pentanoate (5c) was prepared as a green oil from (1-isopropoxy-1-oxopentan-2-yl)diphenylsulfonium tetrafluoroborate (166.5 mg, 0.4 mmol), (*R*)-**B2** (14.0 mg, 5.0 mol%), K_3PO_4 (42.5 mg, 0.2 mmol, 0.5 equiv) and *p*-anisidine (54.2 mg, 0.44 mmol) in toluene (4 mL) and water (4 mL) according to the General Procedure G (18 h, eluent: hexanes/ethyl acetate = 50:1 → 10:1, 90.2 mg, 85% yield, 94% ee).

$[\alpha]_D^{23}$: +47.8 (*c* = 1.0, CH_2Cl_2). HPLC analysis of the product: Daicel CHIRALCEL OD-H column; 3% *i*-PrOH in hexanes; 1.0 mL/min; retention times: 6.9 min (minor), 7.3 min (major).

^{1}H NMR (400 MHz, $CDCl_3$) δ 6.77 – 6.73 (m, 2H), 6.61 – 6.57 (m, 2H), 5.07 – 4.97 (m, 1H), 3.95 (t, *J* = 6.6 Hz, 1H), 3.85 (br, 1H), 3.73 (s, 3H), 1.82 – 1.65 (m, 2H), 1.51 – 1.41 (m, 2H), 1.21 (d, *J* = 6.3 Hz, 3H), 1.18 (d, *J* = 6.3 Hz, 3H), 0.95 (t, *J* = 7.3 Hz, 3H).

^{13}C NMR (100 MHz, $CDCl_3$) δ 174.0, 152.5, 141.1, 115.0, 114.7, 68.2, 57.6, 55.6, 35.2, 21.8, 21.6, 18.8, 13.8.

HRMS (ES+) Calcd for $C_{15}H_{24}NO_3$ $[M+H]^+$: 266.1751, found: 266.1758.

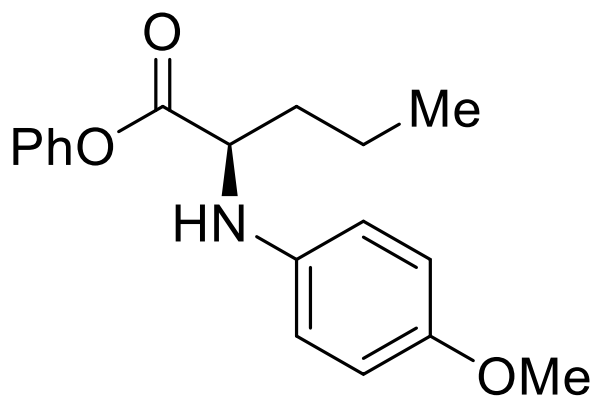

5d

Phenyl (*R*)-2-((4-methoxyphenyl)amino)pentanoate (5d) was prepared as a green oil from (1-oxo-1-phenoxypentan-2-yl)diphenylsulfonium tetrafluoroborate (180.1 mg, 0.4 mmol), (*R*)-**B2** (14.0 mg, 5.0 mol%), K_3PO_4 (42.5 mg, 0.2 mmol, 0.5 equiv) and *p*-anisidine (54.2 mg, 0.44 mmol) in toluene (4 mL) and water (4 mL) according to the General Procedure G (20 h, eluent: hexanes/ethyl acetate = 50:1 → 10:1, 110.8 mg, 93% yield, 92% ee).

$[\alpha]_D^{23}$: +97.6 (c = 1.0, CH_2Cl_2). HPLC analysis of the product: Daicel CHIRALCEL OD-H column; 3% *i*-PrOH in hexanes; 1.0 mL/min; retention times: 15.0 min (major), 17.5 min (minor).

^{1}H NMR (400 MHz, $CDCl_3$) δ 7.39 – 7.35 (m, 2H), 7.27 – 7.22 (m, 1H), 7.01 – 6.99 (m, 2H), 6.85 – 6.82 (m, 2H), 6.75 – 6.72 (m, 2H), 4.26 (t, J = 6.7 Hz, 1H), 3.96 (br, 1H), 3.78 (s, 3H), 1.96 – 1.87 (m, 2H), 1.70 – 1.56 (m, 2H), 1.06 (t, J = 7.3 Hz, 3H).

^{13}C NMR (100 MHz, $CDCl_3$) δ 173.3, 152.9, 150.4, 140.8, 129.4, 125.9, 121.3, 115.2, 114.9, 57.9, 55.6, 35.2, 19.0, 13.8.

HRMS (ES+) Calcd for $C_{18}H_{22}NO_3$ $[M+H]^+$: 300.1594, found: 300.1591.

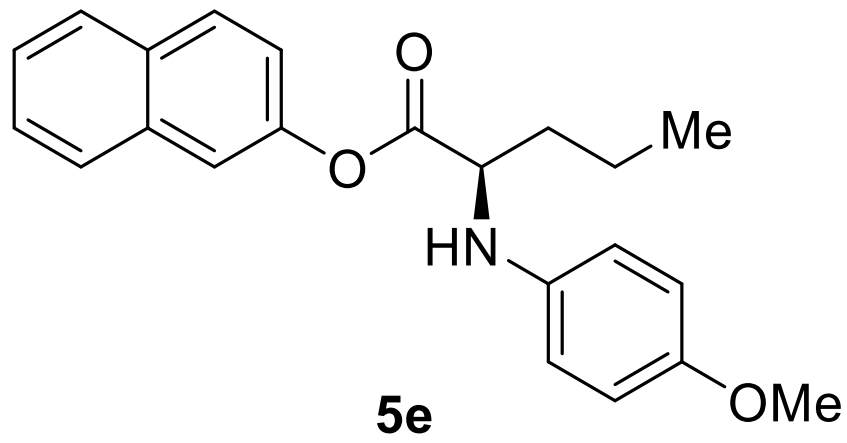

5e

Naphthalen-2-yl (*R*)-2-((4-methoxyphenyl)amino)pentanoate (5e) was prepared as a green oil from (1-(naphthalen-2-yloxy)-1-oxopentan-2-yl)diphenylsulfonium tetrafluoroborate (200.1 mg, 0.4 mmol), (*R*)-**B2** (14.0 mg, 5.0 mol%), K_3PO_4 (42.5 mg, 0.2 mmol, 0.5 equiv) and *p*-anisidine (54.2 mg, 0.44 mmol) in toluene (4 mL) and water (4 mL) according to the General Procedure

G (21 h, eluent: hexanes/ethyl acetate = 50:1 → 10:1, 132.1 mg, 95% yield, 93% ee).

$[\alpha]_D^{23}$: +82.0 (c = 1.0, CH_2Cl_2). HPLC analysis of the product: Daicel CHIRALPAK AS-H column; 5% *i*-PrOH in hexanes; 1.0 mL/min; retention times: 17.3 min (major), 21.3 min (minor).

^{1}H NMR (400 MHz, $CDCl_3$) δ 7.85 – 7.79 (m, 3H), 7.48 – 7.46 (m, 3H), 7.15 – 7.12 (m, 1H), 6.88 – 6.86 (m, 2H), 6.79 – 6.76 (m, 2H), 4.32 (t, *J* = 6.5 Hz, 1H), 4.00 (br, 1H), 3.79 (s, 3H), 2.10 – 1.92 (m, 2H), 1.75 – 1.61 (m, 2H), 1.09 (t, *J* = 7.3 Hz, 3H).

^{13}C NMR (100 MHz, $CDCl_3$) δ 173.4, 152.9, 148.1, 140.8, 133.6, 131.4, 129.4, 127.7, 127.5, 126.5, 125.7, 120.8, 118.3, 115.2, 114.9, 57.9, 55.6, 35.3, 19.1, 13.8.

HRMS (ES+) Calcd for $C_{22}H_{24}NO_3$ $[M+H]^+$: 350.1751, found: 350.1755.

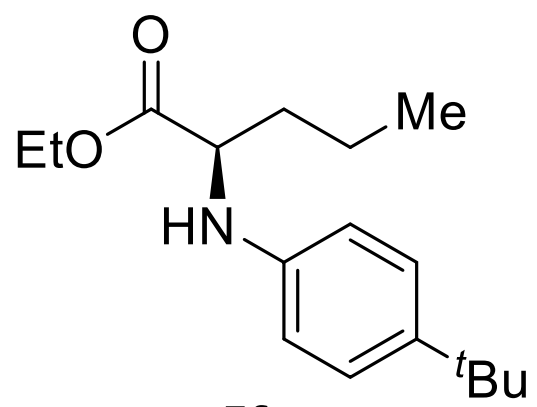

5f

Ethyl (*R*)-2-((4-(*tert*-butyl)phenyl)amino)pentanoate (5f) was prepared as a green oil from (1-ethoxy-1-oxopentan-2-yl)diphenylsulfonium tetrafluoroborate (160.9 mg, 0.4 mmol), (*R*)-**B2** (14.0 mg, 5.0 mol%), K_3PO_4 (42.5 mg, 0.2 mmol, 0.5 equiv) and 4-(*tert*-butyl)aniline (70.4 μL, 0.44 mmol) in toluene (4 mL) and water (4 mL) according to the General Procedure G (19 h, eluent: hexanes/ethyl acetate = 50:1 → 10:1, 104.2 mg, 94% yield, 92% ee).

$[\alpha]_D^{23}$: +41.2 (c = 1.0, CH_2Cl_2). HPLC analysis of the product: Daicel CHIRALPAK AD-H column; 3% *i*-PrOH in hexanes; 1.0 mL/min; retention times: 6.8 min (major), 8.6 min (minor).

^{1}H NMR (400 MHz, $CDCl_3$) δ 7.24 – 7.20 (m, 2H), 6.62 – 6.58 (m, 2H), 4.20 (q, *J* = 7.1 Hz, 2H), 4.06 (br, 2H), 1.88 – 1.74 (m, 2H), 1.53 – 1.44 (m, 2H), 1.30 – 1.26 (m, 12H), 0.98 (t, *J* = 7.3 Hz, 3H).

^{13}C NMR (100 MHz, $CDCl_3$) δ 174.4, 144.5, 140.8, 126.0, 113.0, 60.8, 56.6, 35.2,

33.8, 31.4, 18.8, 14.2, 13.8.

HRMS (ES+) Calcd for $C_{17}H_{28}NO_2$ $[M+H]^+$: 278.2115, found: 278.2121.

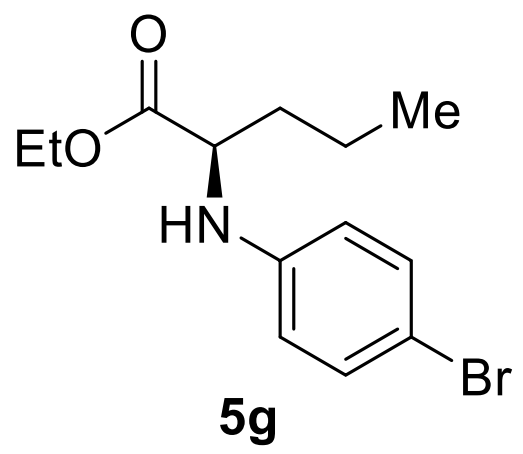

5g

Ethyl (*R*)-2-((4-bromophenyl)amino)pentanoate (5g) was prepared as a green oil from (1-ethoxy-1-oxopentan-2-yl)diphenylsulfonium tetrafluoroborate (160.9 mg, 0.4 mmol), (*R*)-**B2** (14.0 mg, 5.0 mol%), K_3PO_4 (42.5 mg, 0.2 mmol, 0.5 equiv) and 4-bromoaniline (75.2 mg, 0.44 mmol) in toluene (4 mL) and water (4 mL) according to the General Procedure G (20 h, eluent: hexanes/ethyl acetate = 50:1 → 10:1, 111.2 mg, 93% yield, 97% ee).

$[\alpha]_D^{23}$: +43.7 (c = 1.0, CH_2Cl_2). HPLC analysis of the product: Daicel CHIRALPAK IC column; 3% *i*-PrOH in hexanes; 1.0 mL/min; retention times: 7.9 min (major), 8.7 min (minor).

^{1}H NMR (400 MHz, $CDCl_3$) δ 7.25 – 7.22 (m, 2H), 6.50 – 6.47 (m, 2H), 4.20 – 4.15 (m, 3H), 4.00 – 3.97 (m, 1H), 1.85 – 1.67 (m, 2H), 1.49 – 1.39 (m, 2H), 1.24 (t, J = 7.1 Hz, 3H), 0.95 (t, J = 7.3 Hz, 3H).

^{13}C NMR (100 MHz, $CDCl_3$) δ 173.8, 145.9, 131.9, 114.9, 109.7, 61.0, 56.3, 34.9, 18.7, 14.2, 13.7.

HRMS (ES+) Calcd for $C_{13}H_{19}BrNO_2$ $[M+H]^+$: 300.0594, found: 300.0600.

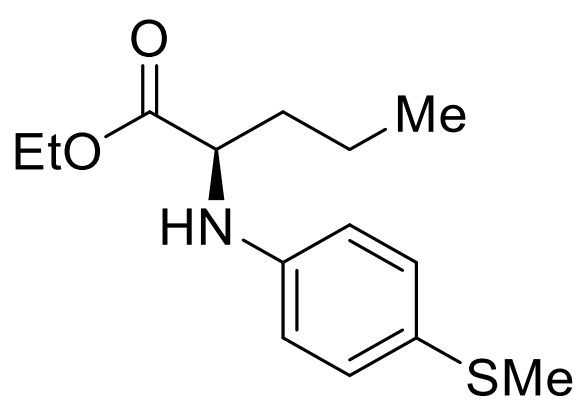

5h

Ethyl (*R*)-2-((4-(methylthio)phenyl)amino)pentanoate (5h) was prepared as a green oil from (1-ethoxy-1-oxopentan-2-yl)diphenylsulfonium

tetrafluoroborate (160.9 mg, 0.4 mmol), (*R*)-**B2** (14.0 mg, 5.0 mol%), K_3PO_4 (42.5 mg, 0.2 mmol, 0.5 equiv) and 4-(methylthio)aniline (54.7 μL, 0.44 mmol) in toluene (4 mL) and water (4 mL) according to the General Procedure G (20 h, eluent: hexanes/ethyl acetate = 50:1 → 10:1, 98.1 mg, 92% yield, 96% ee).

$[\alpha]_D^{23}$: +51.9 (*c* = 1.0, CH_2Cl_2). HPLC analysis of the product: Daicel CHIRALPAK IC column; 3% *i*-PrOH in hexanes; 1.0 mL/min; retention times: 15.6 min (minor), 18.0 min (major).

^{1}H NMR (400 MHz, $CDCl_3$) δ 7.21 – 7.17 (m, 2H), 6.58 – 6.54 (m, 2H), 4.20 – 4.15 (m, 3H), 4.03 – 4.00 (m, 1H), 2.39 (s, 3H), 1.84 – 1.67 (m, 2H), 1.49 – 1.40 (m, 2H), 1.24 (t, *J* = 7.1 Hz, 3H), 0.95 (t, *J* = 7.4 Hz, 3H).

^{13}C NMR (100 MHz, $CDCl_3$) δ 174.0, 145.7, 131.1, 125.2, 113.9, 76.7, 61.0, 56.3, 35.0, 18.8, 14.2, 13.7.

HRMS (ES+) Calcd for $C_{14}H_{22}NO_2S$ $[M+H]^+$: 268.1366, found: 268.1371.

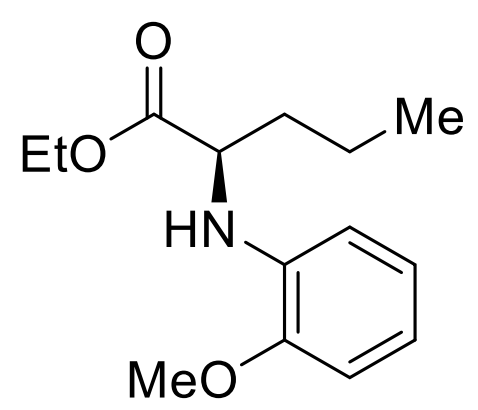

5i

Ethyl (*R*)-2-((2-methoxyphenyl)amino)pentanoate (5i) was prepared as a light green oil from (1-ethoxy-1-oxopentan-2-yl)diphenylsulfonium tetrafluoroborate (160.9 mg, 0.4 mmol), (*R*)-**B2** (14.0 mg, 5.0 mol%), K_3PO_4 (42.5 mg, 0.2 mmol, 0.5 equiv) and *o*-anisidine (49.6 μL, 0.44 mmol) in toluene (4 mL) and water (4 mL) according to the General Procedure G (18 h, eluent: hexanes/ethyl acetate = 50:1 → 10:1, 85.4 mg, 85% yield, 95% ee).

$[\alpha]_D^{23}$: +46.2 (*c* = 1.0, CH_2Cl_2). HPLC analysis of the product: Daicel CHIRALPAK IC column; 3% *i*-PrOH in hexanes; 1.0 mL/min; retention times: 9.4 min (major), 10.6 min (minor).

^{1}H NMR (400 MHz, $CDCl_3$) δ 6.86 – 6.78 (m, 2H), 6.71 – 6.68 (m, 1H), 6.57 – 6.55

(m, 1H), 4.70 – 4.68 (m, 1H), 4.07 (q, *J* = 6.9 Hz, 2H), 4.09 – 4.04 (m, 1H), 3.86 (s, 3H), 1.91 – 1.75 (m, 2H), 1.54 – 1.45 (m, 2H), 1.25 (t, *J* = 7.1 Hz, 3H), 0.97 (t, *J* = 7.3 Hz, 3H).

^{13}C NMR (100 MHz, $CDCl_3$) δ 174.2, 147.0, 136.8, 121.0, 117.2, 110.1, 109.6, 60.8, 56.1, 55.4, 35.1, 18.9, 14.2, 13.8.

HRMS (ES+) Calcd for $C_{14}H_{22}NO_3$ $[M+H]^+$: 252.1594, found: 252.1601.

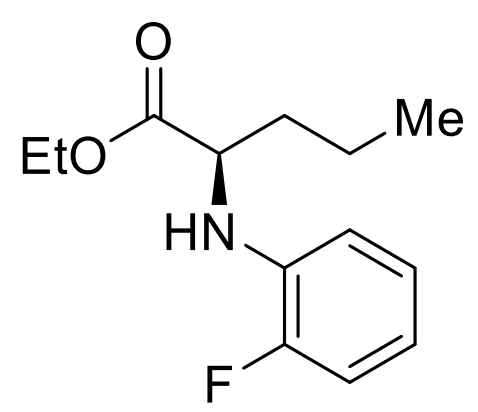

5j

Ethyl (*R*)-2-((2-fluorophenyl)amino)pentanoate (5j) was prepared as a green oil from (1-ethoxy-1-oxopentan-2-yl)diphenylsulfonium tetrafluoroborate (160.9 mg, 0.4 mmol), (*R*)-**B2** (14.0 mg, 5.0 mol%), K_3PO_4 (42.5 mg, 0.2 mmol, 0.5 equiv) and 2-fluoroaniline (42.5 μL, 0.44 mmol) in toluene (4 mL) and water (4 mL) according to the General Procedure G (20 h, eluent: hexanes/ethyl acetate = 50:1 → 10:1, 83.7 mg, 88% yield, 97% ee).

$[\alpha]_D^{23}$: +50.1 (*c* = 1.0, CH_2Cl_2). HPLC analysis of the product: Daicel CHIRALCEL OD-H column; 3% *i*-PrOH in hexanes; 1.0 mL/min; retention times: 5.0 min (minor), 7.9 min (major).

^{1}H NMR (400 MHz, $CDCl_3$) δ 7.01 – 6.95 (m, 2H), 6.68 – 6.62 (m, 2H), 4.35 – 4.33 (m, 1H), 4.18 (q, *J* = 7.1 Hz, 2H), 4.09 – 4.03 (m, 1H), 1.89 – 1.73 (m, 2H), 1.53 – 1.42 (m, 2H), 1.24 (t, *J* = 7.1 Hz, 3H), 0.97 (t, *J* = 7.4 Hz, 3H).

^{13}C NMR (100 MHz, $CDCl_3$) δ 173.7, 151.7 (d, *J* = 237.4 Hz), 135.4 (d, *J* = 11.3 Hz), 124.4 (d, *J* = 3.3 Hz), 117.5 (d, *J* = 6.8 Hz), 114.7 (d, *J* = 18.4 Hz), 112.6 (d, *J* = 3.0 Hz), 61.0, 56.2, 35.0, 18.8, 14.1, 13.7.

^{19}F NMR (376 MHz, $CDCl_3$) δ –135.6.

HRMS (ES+) Calcd for $C_{13}H_{19}FNO_2$ $[M+H]^+$: 240.1394, found: 240.1399.

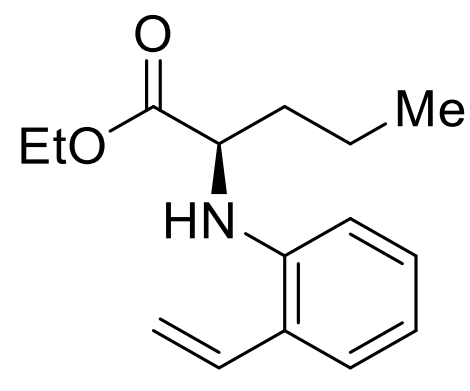

5k

Ethyl (*R*)-2-((2-vinylphenyl)amino)pentanoate (5k) was prepared as a light green oil from (1-ethoxy-1-oxopentan-2-yl)diphenylsulfonium tetrafluoroborate (160.9 mg, 0.4 mmol), (*R*)-**B2** (14.0 mg, 5.0 mol%), K_3PO_4 (42.5 mg, 0.2 mmol, 0.5 equiv) and 2-aminostyrene (51.5 μL, 0.44 mmol) in toluene (4 mL) and water (4 mL) according to the General Procedure G (18 h, eluent: hexanes/ethyl acetate = 50:1 → 10:1, 61.0 mg, 62% yield, 93% ee).

$[\alpha]_D^{23}$: +23.0 (c = 1.0, CH_2Cl_2). HPLC analysis of the product: Daicel CHIRALPAK IC column; 2% *i*-PrOH in hexanes; 1.0 mL/min; retention times: 5.1 min (major), 5.4 min (minor).

^{1}H NMR (400 MHz, $CDCl_3$) δ 7.30 – 7.28 (m, 1H), 7.18 – 7.14 (m, 1H), 6.89 – 6.75 (m, 2H), 6.61 – 6.59 (m, 1H), 5.65 (dd, J_1 = 17.3 Hz, J_2 = 1.6 Hz, 1H), 5.37 (dd, J_1 = 11.0 Hz, J_2 = 1.6 Hz, 1H), 4.33 – 4.31 (m, 1H), 4.20 (q, J = 7.1 Hz, 2H), 4.14 – 4.08 (m, 1H), 1.92 – 1.75 (m, 2H), 1.54 – 1.44 (m, 2H), 1.27 (t, J = 7.1 Hz, 3H), 0.99 (t, J = 7.3 Hz, 3H).

^{13}C NMR (100 MHz, $CDCl_3$) δ 174.2, 143.9, 132.8, 128.8, 127.6, 125.0, 118.1, 116.5, 111.5, 61.0, 56.5, 35.1, 18.8, 14.2, 13.8.

HRMS (ES+) Calcd for $C_{15}H_{22}NO_2$ $[M+H]^+$: 248.1645, found: 248.1651.

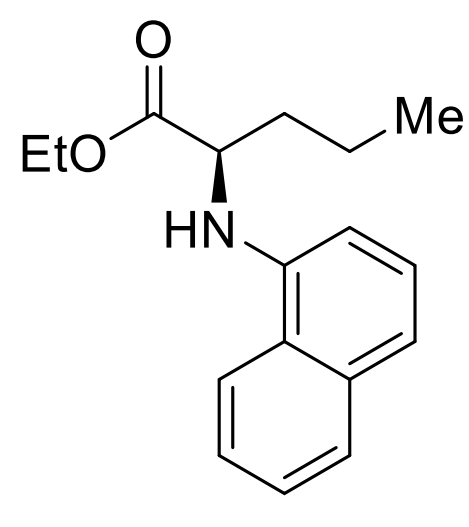

5l

Ethyl (*R*)-2-(naphthalen-1-ylamino)pentanoate (5l) was prepared as a black oil

from (1-ethoxy-1-oxopentan-2-yl)diphenylsulfonium tetrafluoroborate (160.9 mg, 0.4 mmol), (*R*)-**B2** (14.0 mg, 5.0 mol%), K_3PO_4 (42.5 mg, 0.2 mmol, 0.5 equiv) and naphthalen-1-amine (63.0 mg, 0.44 mmol) in toluene (4 mL) and water (4 mL) according to the General Procedure G (20 h, eluent: hexanes/ethyl acetate = 50:1 → 10:1, 97.8 mg, 90% yield, 95% ee).

$[\alpha]_D^{23}$: +1.8 (c = 1.0, CH_2Cl_2). HPLC analysis of the product: Daicel CHIRALCEL OD-H column; 3% *i*-PrOH in hexanes; 1.0 mL/min; retention times: 11.6 min (minor), 24.6 min (major).

^{1}H NMR (400 MHz, $CDCl_3$) δ 7.95 – 7.93 (m, 1H), 7.83 – 7.80 (m, 1H), 7.50 – 7.46 (m, 2H), 7.36 – 7.28 (m, 2H), 6.61 – 6.59 (m, 1H), 4.93 – 4.91 (m, 1H), 4.30 – 4.21 (m, 3H), 2.01 – 1.90 (m, 2H), 1.62 – 1.53 (m, 2H), 1.29 (t, J = 7.1 Hz, 3H), 1.02 (t, J = 7.3 Hz, 3H).

^{13}C NMR (100 MHz, $CDCl_3$) δ 174.3, 142.1, 134.3, 128.6, 126.3, 125.8, 124.8, 123.7, 120.1, 118.1, 105.0, 61.0, 56.4, 35.0, 18.9, 14.2, 13.8.

HRMS (ES+) Calcd for $C_{17}H_{22}NO_2$ $[M+H]^+$: 272.1645, found: 372.1651.

IV. Catalytic Asymmetric H–N_3 Insertion

Table S1. Solvent Effect of Enantioselective Azidation[a]

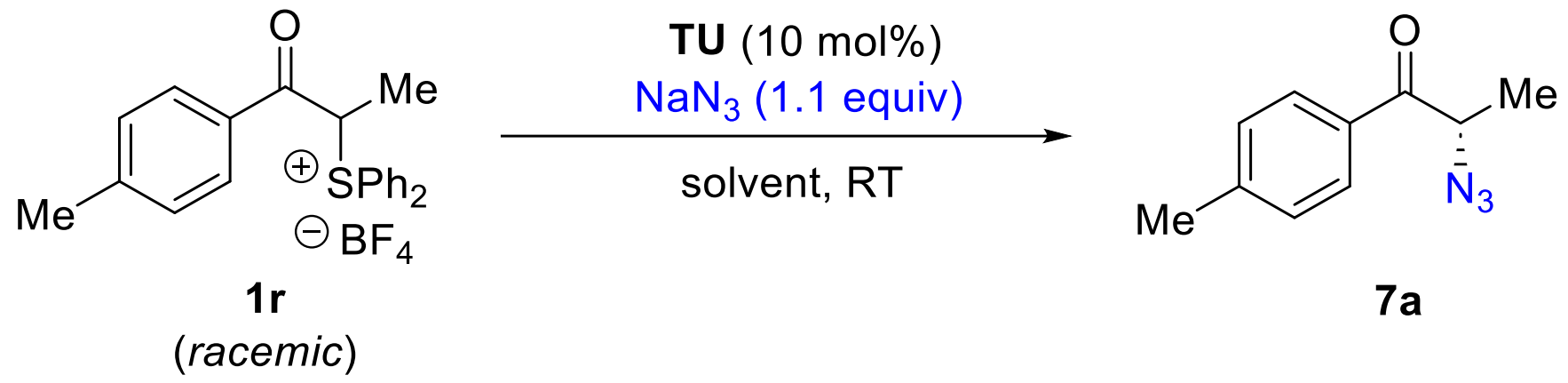

Entry	Solvent	Yield (%)	Ee (%)
1	toluene	>95	92
2[b]	DCM	89	31
3[b]	Et_2O	>95	91
4	EtOAc	>95	61
5	*o*-xylene	>95	93
6	*m*-xylene	>95	93
7	PhCl	>95	80
8	$PhCF_3$	>95	86
9[c]	*o*-xylene	>95	91
10[b,d]	*o*-xylene	74	92
11[e]	*o*-xylene	>95	95
12[f]	*o*-xylene	>95	91

[a]Reaction conditions: **1r** (0.05 mmol), NaN_3 (0.055 mmol), **TU** (10 mol%), solvent (0.5 mL), 4 h. Yield was determined by ^{1}H NMR spectra of the crude mixture using CH_2Br_2 as an internal standard. The ee value was determined by chiral HPLC analysis. [b]Run for 24 h. [c]c = 0.2 M. [d]c = 0.066 M. [e]Run at –15 °C, 48 h. [f]Run at –15 °C with **1r** (0.5 mmol), NaN_3 (0.55 mmol), **TU** (1.0 mol%), *o*-xylene (5 mL), 96 h. Isolated yield is provided.

General Procedure H

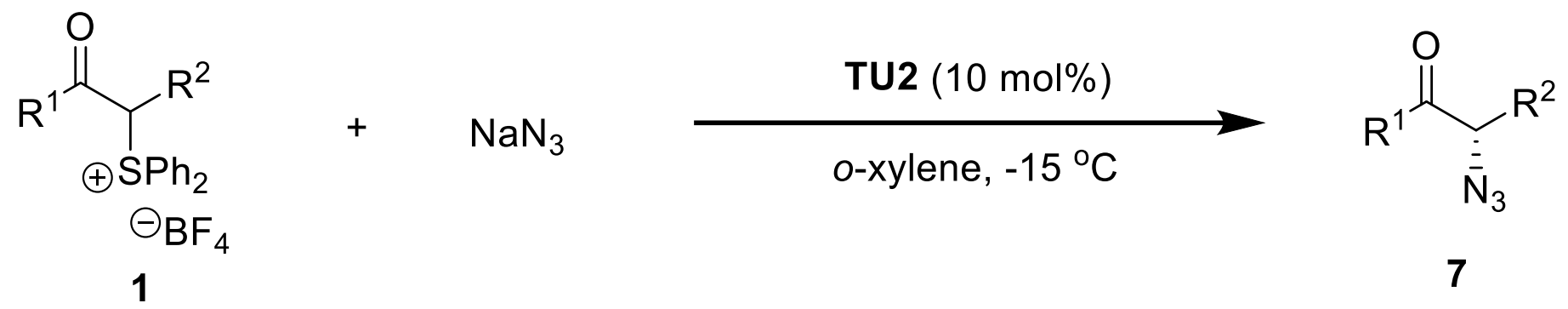

An oven-dried 30-mL vial was charged with sulfonium salt **1** (0.5 mmol, 1.0 equiv) and **TU** (34.1 mg, 0.05 mol, 10 mol%). The vial was carefully sealed with a puncturable screwcap and electrical tape. Then it was cooled to –20 °C, and *o*-xylene (5 mL) was injected into the vial through the cap. The resulted suspension was stirred at –20 °C for 2 min followed by addition of NaN_3 (35.8 mg, 0.55 mmol). The mixture was kept stirring at –15 °C, and the progress was monitored by TLC. Upon completion, the organic layer was separated, and the aqueous phase was extracted by DCM (4 mL × 3). The combined organic layers were directly subjected to silica flash silica gel column chromatography to afford the desired product **7**.

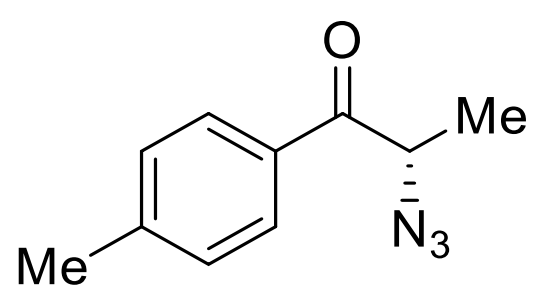

7a

(*S*)-2-Azido-1-(*p*-tolyl)propan-1-one (7a) was prepared as a white solid from (1-oxo-1-(*p*-tolyl)propan-2-yl)diphenylsulfonium tetrafluoroborate (210.1 mg, 0.5 mmol), **TU** (3.4 mg, 1.0 mol%) and NaN_3 (35.8 mg, 0.55 mmol) in *o*-xylene (5 mL) according to the General Procedure H (48 h, eluent: *n*-hexane/DCM = 10:1 → *n*-hexane/ethyl acetate = 10:1, 92.5 mg, 98% yield, 91% ee).

$[\alpha]_D^{21}$: +166.8 (*c* = 1.0, CH_2Cl_2). HPLC analysis of the product: Daicel CHIRALPAK AS-H column; 5% *i*-PrOH in hexanes; 1.0 mL/min; retention times: 7.7 min (major), 13.4 min (minor).

^{1}**H NMR** (400 MHz, $CDCl_3$) δ 7.86 – 7.79 (m, 2H), 7.31 – 7.24 (m, 2H), 4.68 (q, *J* = 7.0 Hz, 1H), 2.40, (s, 3H)1.54 (d, *J* = 7.0 Hz, 3H).

^{13}**C NMR** (100 MHz, $CDCl_3$) δ 196.2, 144.8, 131.5, 129.5, 128.6, 58.1, 21.6, 16.5.
HRMS (CI+) Calcd for $C_{10}H_{11}O$ $[M–N_3]^+$: 147.0804, found: 147.0813.

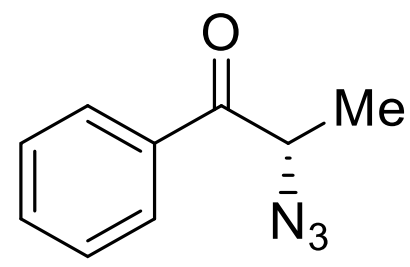

7b

(*S*)-2-Azido-1-phenylpropan-1-one (7b) was prepared as a white solid from (1-oxo-1-phenylpropan-2-yl)diphenylsulfonium tetrafluoroborate (203.1 mg, 0.5 mmol), **TU** (34.1 mg, 10 mol%) and NaN_3 (35.8 mg, 0.55 mmol) in *m*-xylene (5 mL) according to the General Procedure C (–30 °C, 48 h, eluent: *n*-hexane/DCM = 10:1 → *n*-hexane/ethyl acetate = 10:1, 92.5 mg, 82% yield, 94% ee).
$[\alpha]_D^{21}$: +106.7 (*c* = 1.0, CH_2Cl_2). HPLC analysis of the product: Daicel CHIRALPAK AS-H column; 5% *i*-PrOH in hexanes; 1.0 mL/min; retention times: 7.7 min (major), 12.6 min (minor).
^{1}**H NMR** (400 MHz, DMSO-*d*6) δ 8.01 – 7.98 (m, 2H), 7.72 – 7.68 (m, 1H), 7.59 – 7.55 (m, 2H), 5.21 (q, *J* = 7.2 Hz, 1H), 1.42 (d, *J* = 7.0 Hz, 3H).
^{13}**C NMR** (100 MHz, DMSO-*d*6) δ 197.4, 134.0, 133.9, 129.0, 128.6, 58.2, 16.6.
HRMS (CI+) Calcd for $C_9H_{10}N_3O$ $[M+H]^+$: 176.0818, found: 176.0820.

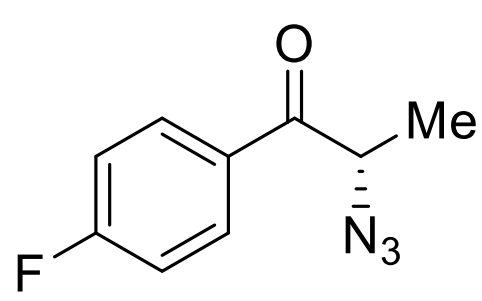

7c

(*S*)-2-Azido-1-(4-fluorophenyl)propan-1-one (7c) was prepared as a green oil from (1-(4-fluorophenyl)-1-oxopropan-2-yl)diphenylsulfonium tetrafluoroborate (212.1 mg, 0.5 mmol), **TU** (68.2 mg, 20 mol%) and NaN_3 (35.8 mg, 0.55 mmol) in *m*-xylene (5 mL) according to the General Procedure H (–25 °C, 48 h, eluent: *n*-hexane/DCM = 10:1 → *n*-hexane/ethyl acetate = 10:1, 92.6 mg, 96% yield, 95% ee).

$[\alpha]_D^{21}$: +141.9 (*c* = 1.0, CH_2Cl_2). HPLC analysis of the product: Daicel CHIRALPAK AS-H column; 5% *i*-PrOH in hexanes; 1.0 mL/min; retention times: 8.9 min (major), 14.5 min (minor).

^{1}H NMR (400 MHz, $CDCl_3$) δ 8.02 – 7.92 (m, 2H), 7.20 – 7.09 (m, 2H), 4.65 (q, *J* = 7.0 Hz, 1H), 1.55 (d, *J* = 7.0 Hz, 3H).

^{13}C NMR (100 MHz, $CDCl_3$) δ 195.0, 166.0 (d, *J* = 256.4 Hz), 131.3 (d, *J* = 9.5 Hz), 130.6 (d, *J* = 3.2 Hz), 116.0 (d, *J* = 22.0 Hz), 58.2, 16.2.

^{19}F NMR (376 MHz, $CDCl_3$) δ –103.3.

HRMS (CI+) Calcd for C_9H_8FO $[M–N_3]^+$: 151.0554, found: 151.0553.

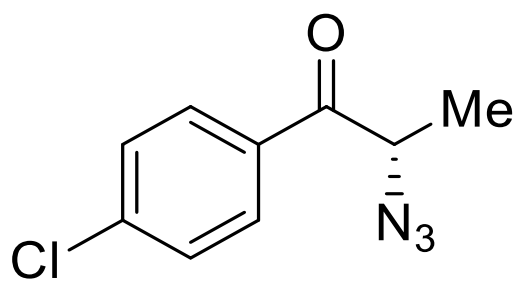

7d

(*S*)-2-Azido-1-(4-chlorophenyl)propan-1-one (7d) was prepared as a green oil from (1-(4-chlorophenyl)-1-oxopropan-2-yl)diphenylsulfonium tetrafluoroborate (220.0 mg, 0.5 mmol), **TU** (34.1 mg, 10 mol%) and NaN_3 (35.8 mg, 0.55 mmol) in *o*-xylene (5 mL) according to the General Procedure H (48 h, eluent: *n*-hexane/DCM = 10:1 → *n*-hexane/ethyl acetate = 10:1, 94.0 mg, 90% yield, 94% ee).

$[\alpha]_D^{21}$: +132.6 (*c* = 1.0, CH_2Cl_2). HPLC analysis of the product: Daicel CHIRALPAK AS-H column; 5% *i*-PrOH in hexanes; 1.0 mL/min; retention times: 9.0 min (major), 11.7 min (minor).

^{1}H NMR (400 MHz, DMSO-d_6) δ 8.02 – 7.99 (m, 2H), 7.65 – 7.62 (m, 2H), 5.20 (q, *J* = 7.0 Hz, 1H), 1.41 (d, *J* = 7.0 Hz, 3H).

^{13}C NMR (100 MHz, DMSO-d_6) δ 196.4, 139.0, 132.6, 130.6, 129.1, 58.3, 16.4.

HRMS (CI+) Calcd for C_9H_8ClO $[M–N_3]^+$: 167.0258, found: 167.0269.

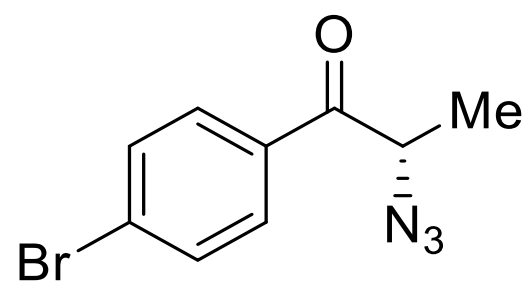

7e

(*S*)-2-Azido-1-(4-bromophenyl)propan-1-one (7e) was prepared as a white solid from (1-(4-bromophenyl)-1-oxopropan-2-yl)diphenylsulfonium tetrafluoroborate (242.0 mg, 0.5 mmol), **TU** (34.1 mg, 10 mol%) and NaN_3 (35.8 mg, 0.55 mmol) in *o*-xylene (5 mL) according to the General Procedure H (48 h, eluent: *n*-hexane/DCM = 10:1 → *n*-hexane/ethyl acetate = 10:1, 116.3 mg, 92% yield, 93% ee).

$[\alpha]_D^{23}$: +122.7 (*c* = 1.0, CH_2Cl_2). HPLC analysis of the product: Daicel CHIRALPAK AS-H column; 5% *i*-PrOH in hexanes; 1.0 mL/min; retention times: 9.3 min (major), 11.1 min (minor).

^{1}H NMR (400 MHz, DMSO-d_6) δ 7.93 – 7.91 (m, 2H), 7.80 – 7.78 (m, 2H), 5.20 (q, *J* = 7.0 Hz, 1H), 1.40 (d, *J* = 7.0 Hz, 3H).

^{13}C NMR (100 MHz, DMSO-d_6) δ 196.6, 133.0, 132.1, 130.6, 128.2, 58.3, 16.4.

HRMS (CI+) Calcd for C_9H_8BrO $[M–N_3]^+$: 210.9753, found: 210.9759.

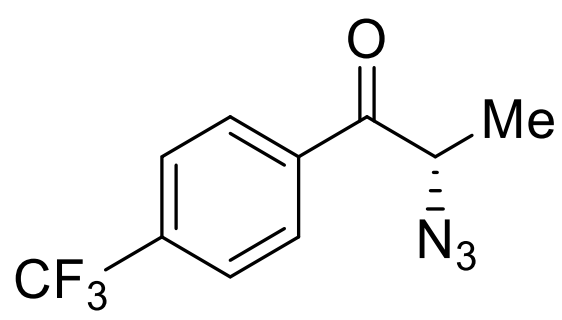

7f

(*S*)-2-Azido-1-(4-(trifluoromethyl)phenyl)propan-1-one (7f) was prepared as a white solid from (1-oxo-1-(4-(trifluoromethyl)phenyl)propan-2-yl)diphenylsulfonium tetrafluoroborate (237.1 mg, 0.5 mmol), **TU** (68.2 mg, 20 mol%) and NaN_3 (35.8 mg, 0.55 mmol) in *m*-xylene (5 mL) according to the General Procedure H (–25 °C, 72 h, eluent: *n*-hexane/DCM = 10:1 → *n*-hexane/ethyl acetate = 10:1, 82.6 mg, 68% yield, 92% ee).

$[\alpha]_D^{21}$: +114.8 (*c* = 1.0, CH_2Cl_2). HPLC analysis of the product: Daicel CHIRALPAK IC column; 3% *i*-PrOH in hexanes; 1.0 mL/min; retention times:

5.6 min (major), 6.2 min (minor).

^{1}H NMR (400 MHz, DMSO-d_6) δ 8.18 (d, *J* = 8.2 Hz, 2H), 7.94 (d, *J* = 8.4 Hz, 2H), 5.29 (q, *J* = 7.1 Hz, 1H), 1.42 (d, *J* = 7.0 Hz, 3H).

^{13}C NMR (100 MHz, DMSO-d_6) δ 196.8, 137.4, 133.1 (q, *J* = 32.0 Hz), 129.5, 125.9 (q, *J* = 3.7 Hz), 123.5 (q, *J* = 311.0 Hz), 58.8, 16.2.

^{19}F NMR (376 MHz, DMSO-d_6) δ –61.7.

HRMS (CI+) Calcd for $C_{10}H_8F_3O$ [M–N_3]$^+$: 201.0522, found: 201.0522.

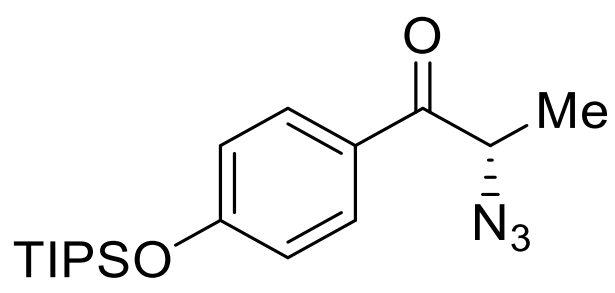

7g

(*S*)-2-Azido-1-(4-((triisopropylsilyl)oxy)phenyl)propan-1-one (7g) was prepared as a light green oil from (1-oxo-1-(4-((triisopropylsilyl)oxy)phenyl)-propan-2-yl)diphenyl-sulfonium tetrafluoroborate (289.1 mg, 0.5 mmol), **TU** (68.2 mg, 20 mol%) and NaN_3 (35.8 mg, 0.55 mmol) in *m*-xylene (5 mL) according to the General Procedure H (–25 °C, 96 h, eluent: *n*-hexane/DCM = 10:1 → *n*-hexane/ethyl acetate = 10:1, 168.6 mg, 97% yield, 87% ee).

$[\alpha]_D^{23}$: +97.0 (*c* = 1.0, CH_2Cl_2). HPLC analysis of the product: Daicel CHIRALPAK IC column; 5% *i*-PrOH in hexanes; 1.0 mL/min; retention times: 5.5 min (major), 6.5 min (minor).

^{1}H NMR (400 MHz, $CDCl_3$) δ 7.86 (d, *J* = 8.5 Hz, 2H), 6.92 (d, *J* = 8.5 Hz, 2H), 4.64 (q, *J* = 6.9 Hz, 1H), 1.54 (d, *J* = 7.0 Hz, 3H), 1.29 – 1.24 (m, 3H), 1.10 – 1.08 (m, 18H).

^{13}C NMR (100 MHz, $CDCl_3$) δ 195.1, 161.3, 130.9, 127.3, 120.0, 57.7, 17.7, 16.5, 12.6.

HRMS (ES+) Calcd for $C_{18}H_{29}N_3NaO_2Si$ [M+Na]$^+$: 370.1921, found: 370.1926.

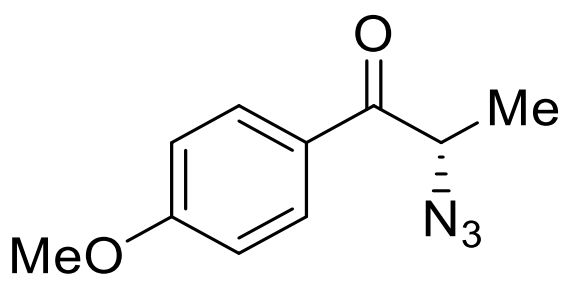

7h

(*S*)-2-Azido-1-(4-methoxyphenyl)propan-1-one (7h) was prepared as a white solid from (1-(4-methoxyphenyl)-1-oxopropan-2-yl)diphenylsulfonium tetrafluoroborate (218.1 mg, 0.5 mmol), **TU** (34.1 mg, 10 mol%) and NaN_3 (35.8 mg, 0.55 mmol) in *o*-xylene (5 mL) according to the General Procedure H (48 h, eluent: *n*-hexane/DCM = 10:1 → *n*-hexane/ethyl acetate = 10:1, 95.1 mg, 93% yield, 96% ee).

$[\alpha]_D^{23}$: +189.0 (*c* = 1.0, CH_2Cl_2). HPLC analysis of the product: Daicel CHIRALPAK IC column; 10% *i*-PrOH in hexanes; 1.0 mL/min; retention times: 9.8 min (major), 12.3 min (minor).

^{1}H NMR (400 MHz, $CDCl_3$) δ 7.96 – 7.87 (m, 2H), 6.99 – 6.90 (m, 2H), 4.65 (q, *J* = 7.0 Hz, 1H), 3.86 (s, 3H), 1.54 (d, *J* = 7.0 Hz, 3H).

^{13}C NMR (100 MHz, $CDCl_3$) δ 195.1, 164.0, 130.9, 126.9, 114.0, 57.9, 55.5, 16.6.

HRMS (CI+) Calcd for $C_{10}H_{12}N_3O_2$ $[M+H]^+$: 206.0924, found: 206.0936.

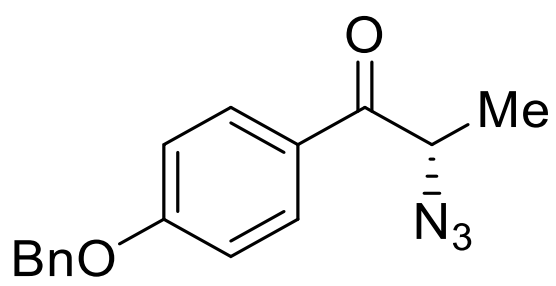

7i

(*S*)-2-Azido-1-(4-(benzyloxy)phenyl)propan-1-one (7i) was prepared as a light green oil from (1-(4-(benzyloxy)phenyl)-1-oxopropan-2-yl)diphenylsulfonium tetrafluoroborate (256.1 mg, 0.5 mmol), **TU** (68.2 mg, 20 mol%) and NaN_3 (35.8 mg, 0.55 mmol) in *o*-xylene (5 mL) according to the General Procedure H (48 h, eluent: *n*-hexane/DCM = 10:1 → *n*-hexane/ethyl acetate = 10:1, 132.2 mg, 94% yield, 95% ee).

$[\alpha]_D^{22}$: +139.4 (*c* = 1.0, CH_2Cl_2). HPLC analysis of the product: Daicel CHIRALPAK IC column; 7% *i*-PrOH in hexanes; 1.0 mL/min; retention times:

11.9 min (major), 16.2 min (minor).

^{1}H NMR (400 MHz, $CDCl_3$) δ 7.94 (d, *J* = 8.7 Hz, 2H), 7.45 – 7.34 (m, 5H), 7.05 (d, *J* = 8.7 Hz, 2H), 5.14 (s, 2H), 4.66 (q, *J* = 7.0 Hz, 1H), 1.56 (d, *J* = 7.0 Hz, 3H).

^{13}C NMR (100 MHz, $CDCl_3$) δ 195.0, 163.1, 135.8, 130.9, 128.6, 128.2, 127.3, 127.1, 114.8, 70.1, 57.9, 16.5.

HRMS (CI+) Calcd for $C_{16}H_{16}NO_2$ $[M–N_2+H]^+$: 254.1176, found: 254.1188.

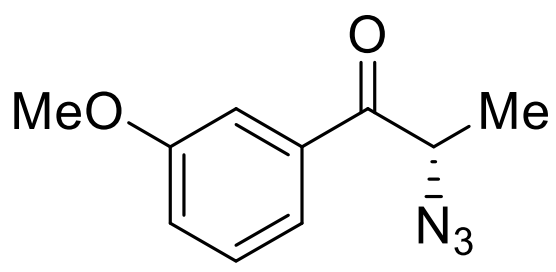

7j

(*S*)-2-Azido-1-(3-methoxyphenyl)propan-1-one (7j) was prepared as a green oil from (1-(3-methoxyphenyl)-1-oxopropan-2-yl)diphenylsulfonium tetrafluoroborate (218.1 mg, 0.5 mmol), **TU** (34.1 mg, 10 mol%) and NaN_3 (35.8 mg, 0.55 mmol) in *o*-xylene (5 mL) according to the General Procedure H (48 h, eluent: *n*-hexane/DCM = 10:1 → *n*-hexane/ethyl acetate = 10:1, 99.1 mg, 97% yield, 92% ee).

$[\alpha]_D^{21}$: +108.4 (*c* = 1.0, CH_2Cl_2). HPLC analysis of the product: Daicel CHIRALPAK IC column; 5% *i*-PrOH in hexanes; 1.0 mL/min; retention times: 8.5 min (major), 9.7 min (minor).

^{1}H NMR (400 MHz, $CDCl_3$) δ 7.52 – 7.44 (m, 2H), 7.44 – 7.35 (m, 1H), 7.15 (dd, *J* = 7.3, 2.1 Hz, 1H), 4.69 (q, *J* = 7.0 Hz, 1H), 3.86 (s, 3H), 1.56 (d, *J* = 7.0 Hz, 3H).

^{13}C NMR (100 MHz, $CDCl_3$) δ 196.6, 159.9, 135.4, 129.8, 120.9, 120.2, 112.9, 58.3, 55.3, 16.5.

HRMS (CI+) Calcd for $C_{10}H_{11}N_3O_2$ $[M]^+$: 205.0851, found: 205.0853.

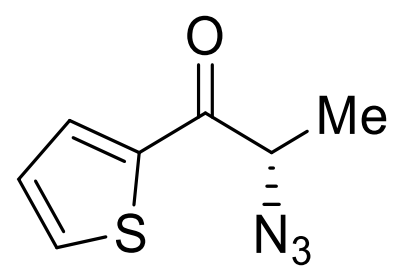

7k

(*S*)-2-Azido-1-(thiophen-2-yl)propan-1-one (7k) was prepared as a light green oil from (1-oxo-1-(thiophen-2-yl)propan-2-yl)diphenylsulfonium tetrafluoroborate (206.0 mg, 0.5 mmol), **TU** (68.2 mg, 20 mol%) and NaN_3 (35.8 mg, 0.55 mmol) in *o*-xylene (5 mL) according to the General Procedure H (48 h, eluent: *n*-hexane/DCM = 10:1 → *n*-hexane/ethyl acetate = 10:1, 84.0 mg, 92% yield, 94% ee).

$[\alpha]_D^{23}$: +168.4 (*c* = 1.0, CH_2Cl_2). HPLC analysis of the product: Daicel CHIRALPAK AS-H column; 5% *i*-PrOH in hexanes; 1.0 mL/min; retention times: 10.3 min (major), 13.5 min (minor).

^{1}H NMR (400 MHz, DMSO-d_6) δ 8.17 – 8.07 (m, 2H), 7.34 – 7.27 (m, 1H), 5.02 (q, *J* = 7.0 Hz, 1H), 1.47 (d, *J* = 7.0 Hz, 3H).

^{13}C NMR (100 MHz, DMSO-d_6) δ 190.3, 140.4, 136.7, 134.9, 129.1, 58.6, 17.0.

HRMS (CI+) Calcd for $C_7H_8N_3OS$ $[M+H]^+$: 182.0383, found: 182.0390.

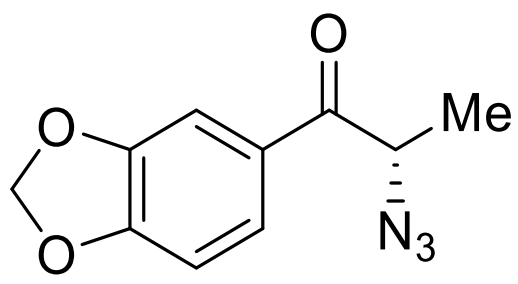

7l

(*S*)-2-Azido-1-(benzo[d][1,3]dioxol-5-yl)propan-1-one (7l) was prepared as a green oil from (1-(benzo[d][1,3]dioxol-5-yl)-1-oxopropan-2-yl)diphenylsulfonium tetrafluoroborate (225.1 mg, 0.5 mmol), **TU** (34.1 mg, 20 mol%) mol and NaN_3 (35.8 mg, 0.55 mmol) in *o*-xylene (5 mL) according to the General Procedure H (48 h, eluent: *n*-hexane/DCM = 10:1 → *n*-hexane/ethyl acetate = 10:1, 87.7 mg, 80% yield, 95% ee).

$[\alpha]_D^{22}$: +168.2 (*c* = 1.0, CH_2Cl_2). HPLC analysis of the product: Daicel CHIRALPAK IC column; 7% *i*-PrOH in hexanes; 1.0 mL/min; retention times:

13.3 min (major), 17.5 min (minor).

[1]**H NMR** (400 MHz, $CDCl_3$) δ 7.50 (dd, *J* = 8.2, 1.8 Hz, 1H), 7.38 (d, *J* = 1.8 Hz, 1H), 6.84 (d, *J* = 8.2 Hz, 1H), 6.03 (s, 2H), 4.59 (q, *J* = 7.0 Hz, 1H), 1.52 (d, *J* = 7.0 Hz, 3H).

[13]**C NMR** (100 MHz, $CDCl_3$) δ 194.7, 152.4, 148.4, 128.7, 124.9, 108.2, 108.0, 102.0, 57.9, 16.5.

HRMS (CI+) Calcd for $C_{10}H_9N_3O_3$ $[M]^+$: 219.0644, found: 219.0642.

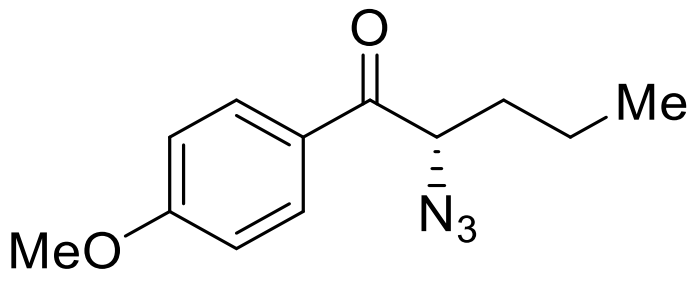

7m

(*S*)-2-Azido-1-(4-methoxyphenyl)pentan-1-one (7m) was prepared as a green solid from (1-(4-methoxyphenyl)-1-oxopentan-2-yl)diphenylsulfonium tetrafluoroborate (232.1 mg, 0.5 mmol), **TU′** (29.1 mg, 10 mol%) and NaN_3 (35.8 mg, 0.55 mmol) in *o*-xylene (5 mL) according to the General Procedure H (48 h, eluent: *n*-hexane/DCM = 10:1 → *n*-hexane/ethyl acetate = 10:1, 108.3 mg, 93% yield, 93% ee).

$[\alpha]_D^{23}$: +152.3 (*c* = 1.0, CH_2Cl_2). HPLC analysis of the product: Daicel CHIRALPAK AD-H column; 10% *i*-PrOH in hexanes; 1.0 mL/min; retention times: 7.2 min (minor), 7.7 min (major).

[1]**H NMR** (400 MHz, $CDCl_3$) δ 7.94 – 7.85 (m, 2H), 6.99 – 6.90 (m, 2H), 4.53 (dd, *J* = 8.6, 5.2 Hz, 1H), 3.86 (s, 3H), 1.93 – 1.73 (m, 2H), 1.61 – 1.39 (m, 2H), 0.96 (t, *J* = 7.4 Hz, 3H).

[13]**C NMR** (100 MHz, $CDCl_3$) δ 195.2, 164.0, 130.8, 127.4, 114.0, 62.3, 55.5, 33.4, 19.4, 13.6.

HRMS (ES+) Calcd for $C_{12}H_{15}N_3NaO_2$ $[M+Na]^+$: 256.1056, found: 256.1066.

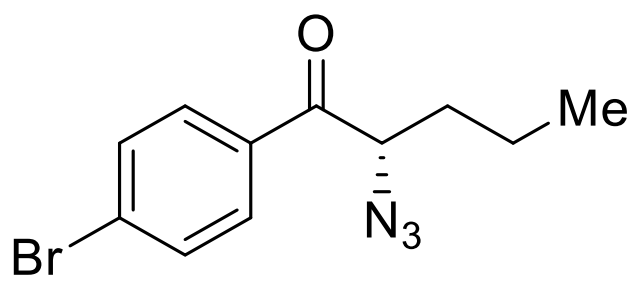

7n

(*S*)-2-Azido-1-(4-bromophenyl)pentan-1-one (7n) was prepared as a light green solid from (1-(4-bromophenyl)-1-oxopentan-2-yl)diphenylsulfonium tetrafluoroborate (256.0 mg, 0.5 mmol), **TU′** (58.2 mg, 20 mol%) and NaN_3 (35.8 mg, 0.55 mmol) in *m*-xylene (5 mL) according to the General Procedure H (–40 to –30 °C, 96 h, eluent: *n*-hexane/DCM = 10:1 → *n*-hexane/ethyl acetate = 10:1, 136.5 mg, 97% yield, 91% ee).

$[\alpha]_D^{22}$: +114.8 (*c* = 1.0, CH_2Cl_2). HPLC analysis of the product: Daicel CHIRALPAK AD-H column; 3% *i*-PrOH in hexanes; 1.0 mL/min; retention times: 7.3 min (minor), 7.7 min (major).

^{1}H NMR (400 MHz, $CDCl_3$) δ 7.83 – 7.74 (m, 2H), 7.66 – 7.58 (m, 2H), 4.51 (dd, *J* = 8.6, 5.2 Hz, 1H), 1.92 – 1.72 (m, 2H), 1.61 – 1.39 (m, 2H), 0.96 (t, *J* = 7.3 Hz, 3H).

^{13}C NMR (100 MHz, $CDCl_3$) δ 195.7, 133.2, 132.1, 129.9, 129.1, 62.8, 33.0, 19.3, 13.6.

HRMS (CI+) Calcd for $C_{11}H_{12}BrO$ $[M–N_3]^+$: 239.0066, found: 239.0078.

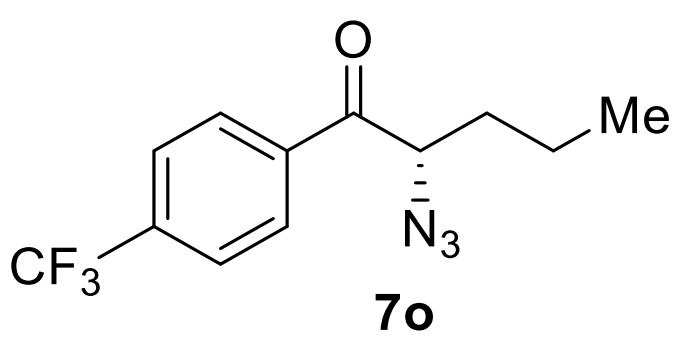

7o

(*S*)-2-Azido-1-(4-(trifluoromethyl)phenyl)pentan-1-one (7o) was prepared as a green oil from (1-oxo-1-(4-(trifluoromethyl)phenyl)pentan-2-yl)diphenylsulfonium tetrafluoroborate (251.1 mg, 0.5 mmol), **TU′** (58.2 mg, 20 mol%) and NaN_3 (35.8 mg, 0.55 mmol) in *m*-xylene (5 mL) according to the General Procedure H (–40 to –30 °C, 96 h, eluent: *n*-hexane/DCM = 10:1 → *n*-hexane/ethyl acetate = 10:1, 122.6 mg, 90% yield, 86% ee).

$[\alpha]_D^{22}$: +79.3 (c = 1.0, CH_2Cl_2). HPLC analysis of the product: Daicel CHIRALPAK AD-H column; 3% *i*-PrOH in hexanes; 1.0 mL/min; retention times: 5.8 min (minor), 6.2 min (major).

^{1}H NMR (400 MHz, $CDCl_3$) δ 8.03 (d, J = 8.3 Hz, 2H), 7.76 (d, J = 8.2 Hz, 2H), 4.57 (q, J = 4.3 Hz, 1H), 1.92 – 1.78 (m, 2H), 1.61 – 1.43 (m, 2H), 0.98 (t, J = 7.4 Hz, 3H).

^{13}C NMR (100 MHz, $CDCl_3$) δ 195.9, 137.4, 135.0 (q, J = 32.8 Hz), 128.9, 125.9 (q, J = 3.7 Hz), 123.4 (q, J = 272.9 Hz), 63.2, 32.9, 19.4, 13.5.

^{19}F NMR (376 MHz, $CDCl_3$) δ –63.3.

HRMS (CI+) Calcd for $C_{12}H_{12}F_3NO$ $[M–N_2]^+$: 243.0866, found: 243.0868.

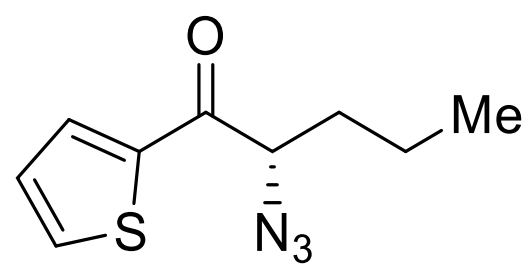

7p

(*S*)-2-Azido-1-(thiophen-2-yl)pentan-1-one (7p) was prepared as a light green solid from (1-oxo-1-(thiophen-2-yl)pentan-2-yl)diphenylsulfonium tetrafluoroborate (220.1 mg, 0.5 mmol), **TU'** (58.2 mg, 20 mol%) and NaN_3 (35.8 mg, 0.55 mmol) in *m*-xylene (5 mL) according to the General Procedure H (–40 to –30 °C, 48 h, eluent: *n*-hexane/DCM = 10:1 → *n*-hexane/ethyl acetate = 10:1, 101.4 mg, 97% yield, 92% ee).

$[\alpha]_D^{22}$: +128.9 (c = 1.0, CH_2Cl_2). HPLC analysis of the product: Daicel CHIRALPAK AS-H column; 5% *i*-PrOH in hexanes; 1.0 mL/min; retention times: 6.9 min (major), 11.2 min (minor).

^{1}H NMR (400 MHz, $CDCl_3$) δ 7.81 – 7.70 (m, 2H), 7.21 – 7.14 (m, 1H), 4.38 (dd, J = 8.3, 5.7 Hz, 1H), 1.99 – 1.81 (m, 2H), 1.64 – 1.52 (m, 2H), 0.99 (t, J = 7.3 Hz, 3H).

^{13}C NMR (100 MHz, $CDCl_3$) δ 189.7, 141.2, 135.1, 132.9, 128.4, 64.1, 33.8, 19.3, 13.5.

HRMS (CI+) Calcd for $C_9H_{11}N_3OS$ [M]$^+$: 209.0623, found: 209.0627.

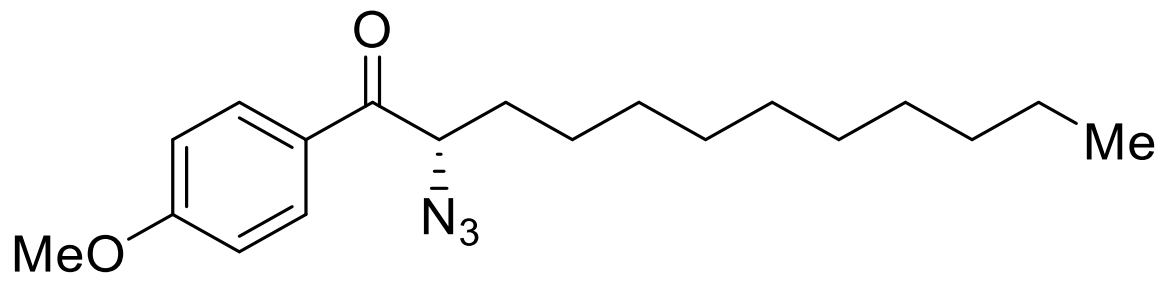

7q

(*S*)-2-Azido-1-(4-methoxyphenyl)dodecan-1-one (7q) was prepared as a light green solid from (1-(4-methoxyphenyl)-1-oxododecan-2-yl)diphenylsulfonium tetrafluoroborate (281.1 mg, 0.5 mmol), **TU′** (58.2 mg, 20 mol%) and NaN_3 (35.8 mg, 0.55 mmol) in *o*-xylene (5 mL) according to the General Procedure H (–40 to –30 °C, 36 h, eluent: *n*-hexane/DCM = 10:1 → *n*-hexane/ethyl acetate = 10:1, 162.2 mg, 98% yield, 90% ee).

$[\alpha]_D^{23}$: +87.5 (*c* = 1.0, CH_2Cl_2). HPLC analysis of the product: Daicel CHIRALPAK AD-3 column; 1% *i*-PrOH in hexanes; 1.0 mL/min; retention times: 10.5 min (minor), 11.5 min (major).

^{1}H NMR (400 MHz, $CDCl_3$) δ 7.91 (d, *J* = 8.9 Hz, 2H), 6.95 (d, *J* = 8.9 Hz, 2H), 4.51 (dd, *J* = 8.7, 5.0 Hz, 1H), 3.87 (s, 3H), 1.93 – 1.78 (m, 2H), 1.60 – 1.22 (m, 16H), 0.86 (t, *J* = 6.7 Hz, 3H).

^{13}C NMR (100 MHz, $CDCl_3$) δ 195.2, 164.0, 130.8, 127.5, 114.0, 62.7, 55.5, 31.8, 31.4, 29.5, 29.4, 29.3, 29.2, 29.1, 26.1, 22.6, 14.0.

HRMS (CI+) Calcd for $C_{19}H_{30}N_3O_2$ [M+H]$^+$: 332.2333, found: 332.2344.

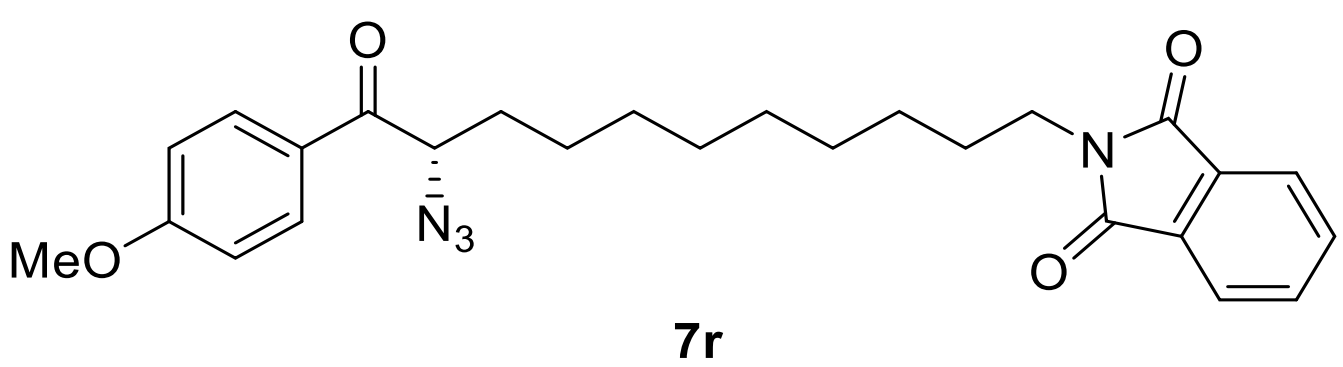

7r

(*S*)-2-(10-Azido-11-(4-methoxyphenyl)-11-oxoundecyl)isoindoline-1,3-dione (7r) was prepared as a light green solid from (11-(1,3-Dioxoisoindolin-2-yl)-1-(4-methoxyphenyl)-1-oxoundecan-2-yl)diphenylsulfonium tetrafluoroborate (346.6 mg, 0.5 mmol), **TU′** (58.2 mg, 20 mol%) and NaN_3 (35.8 mg, 0.55 mmol)

in *m*-xylene (5 mL) according to the General Procedure H (–40 to –30 °C, 36 h, eluent: *n*-hexane/DCM = 10:1 → toluene/ethyl acetate = 30:1, 224.1 mg, 97% yield, 94% ee).

$[\alpha]_D^{22}$: +66.5 (*c* = 1.0, CH_2Cl_2). HPLC analysis of the product: Daicel CHIRALPAK AD-H column; 15% *i*-PrOH in hexanes; 1.0 mL/min; retention times: 27.7 min (minor), 36.4 min (major).

^{1}H NMR (400 MHz, $CDCl_3$) δ 7.93 – 7.85 (m, 2H), 7.79 (dd, *J* = 5.5, 3.0 Hz, 2H), 7.67 (dd, *J* = 5.5, 3.1 Hz, 2H), 6.97 – 6.89 (m, 2H), 4.50 (dd, *J* = 8.8, 5.0 Hz, 1H), 3.85 (s, 3H), 3.63 (t, *J* = 7.2 Hz, 2H), 1.93 – 1.71 (m, 2H), 1.67 – 1.58 (m, 2H), 1.55 – 1.12 (m, 12H).

^{13}C NMR (100 MHz, $CDCl_3$) δ 195.1, 168.3, 164.0, 133.7, 132.0, 130.8, 127.4, 123.0, 114.0, 62.6, 55.4, 37.9, 31.4, 29.2, 29.1, 29.0, 28.9, 28.4, 26.7, 26.0.

HRMS (ES+) Calcd for $C_{26}H_{30}N_4NaO_4$ $[M+Na]^+$: 485.2159, found: 485.2164.

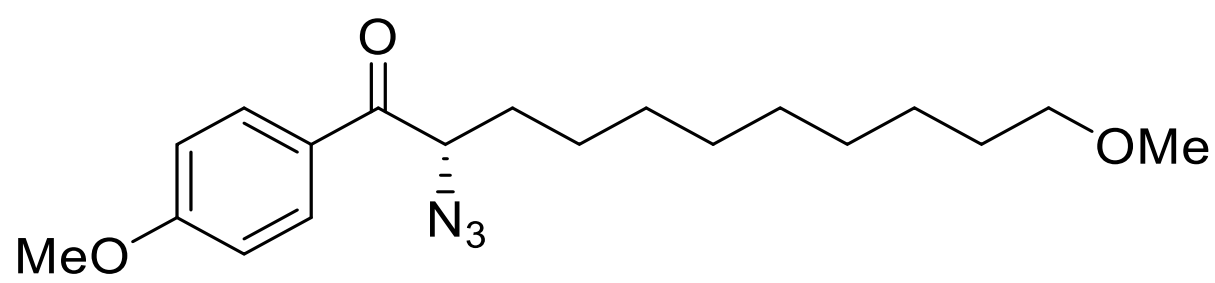

7s

(*S*)-2-Azido-11-methoxy-1-(4-methoxyphenyl)undecan-1-one (7s) was prepared as a red viscous oil from (11-methoxy-1-(4-methoxyphenyl)-1-oxoundecan-2-yl)diphenylsulfonium tetrafluoroborate (289.1 mg, 0.5 mmol), **TU'** (58.2 mg, 20 mol%) and NaN_3 (35.8 mg, 0.55 mmol) in *m*-xylene (5 mL) according to the General Procedure H (–40 to –30 °C, 48 h, eluent: *n*-hexane/DCM = 10:1 → *n*-hexane/ethyl acetate = 10:1, 149.8 mg, 86% yield, 88% ee).

$[\alpha]_D^{22}$: +96.6 (*c* = 1.0, CH_2Cl_2). HPLC analysis of the product: Daicel CHIRALPAK AD-H column; 15% *i*-PrOH in hexanes; 1.0 mL/min; retention times: 6.8 min (minor), 8.2 min (major).

^{1}H NMR (400 MHz, $CDCl_3$) δ 7.91 – 7.83 (m, 2H), 6.96 – 6.87 (m, 2H), 4.49 (dd, *J* = 8.7, 5.0 Hz, 1H), 3.83 (s, 3H), 3.33 – 3.25 (m, 5H), 1.91 – 1.70 (m, 2H), 1.66 –

1.11 (m, 14H).

^{13}C NMR (100 MHz, $CDCl_3$) δ 195.1, 164.0, 130.7, 127.3, 114.0, 72.7, 62.5, 58.3, 55.4, 31.3, 29.9, 29.2(2C), 29.1, 29.0, 26.0, 25.9.

HRMS (CI+) Calcd for $C_{19}H_{30}N_3O_3$ $[M+H]^+$: 348.2282, found: 348.2295.

V. Mechanistic Studies

(1) Time-dependence of product enantiopurity

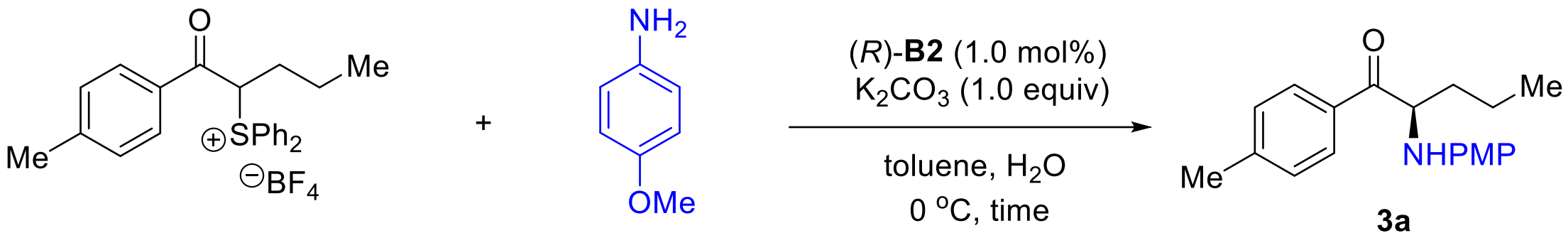

At room temperature, an oven-dried 4-mL vial was charged with K_2CO_3 (13.8 mg, 0.1 mmol), (*R*)-**B2** (0.7 mg, 1.0 μmol, 1.0 mol%), and toluene (1 mL). The mixture was vigorously stirred for 25 min before the sulfonium salt **1a** (44.8 mg, 0.1 mmol) was added. The mixture was kept stirring for another 10 min before it was cooled to 0 °C and the amine **2a** (13.6 mg, 0.11 mmol, 1.1 equiv) was added together with water (1 mL). The mixture was stirred vigorously at 0 °C, and the enantiomeric excess (ee) of product **3a** was determined at 30 min intervals until complete consumption of starting material **1a**.

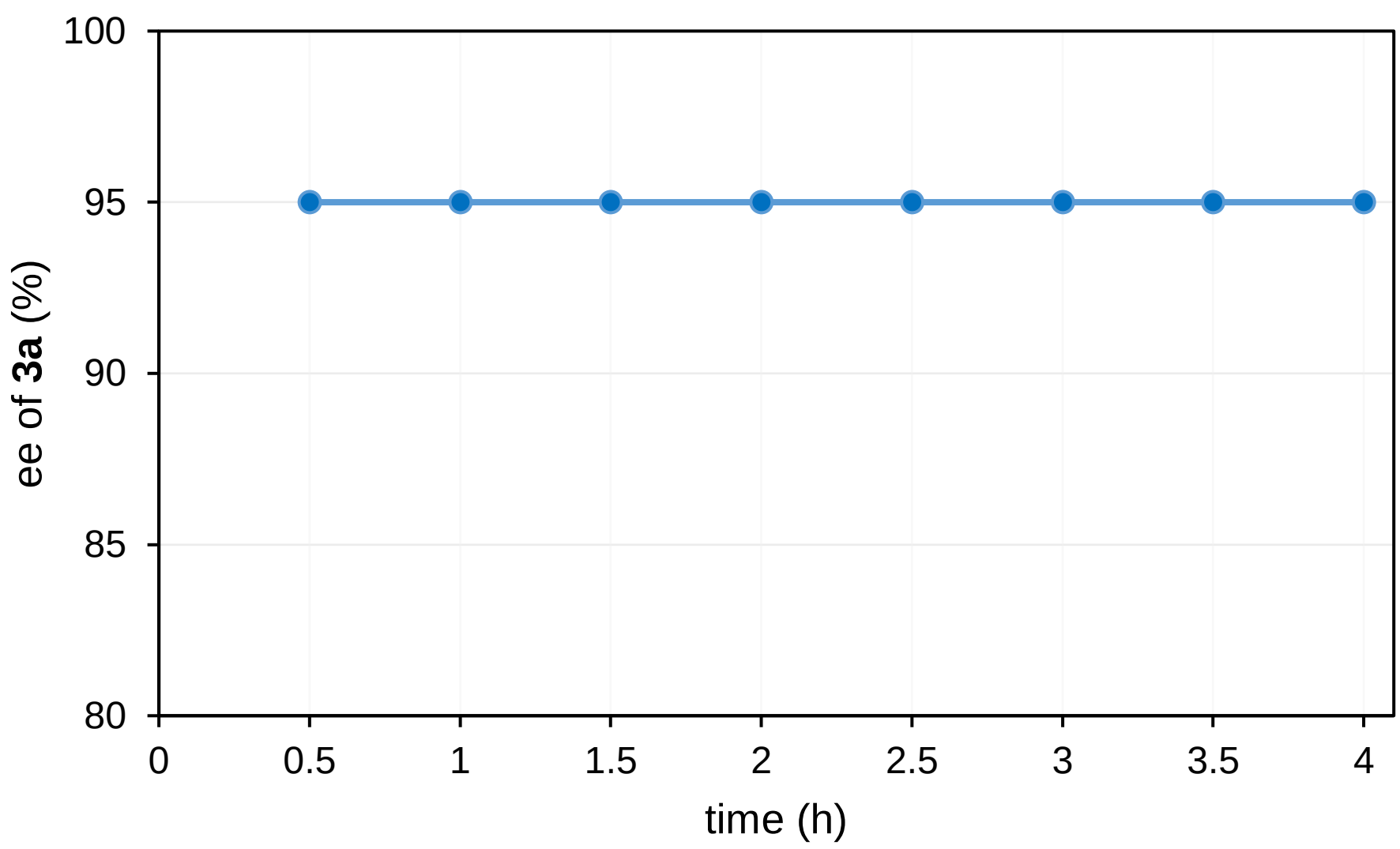

(2) H/D-exchange at the α-position of the sulfonium salt

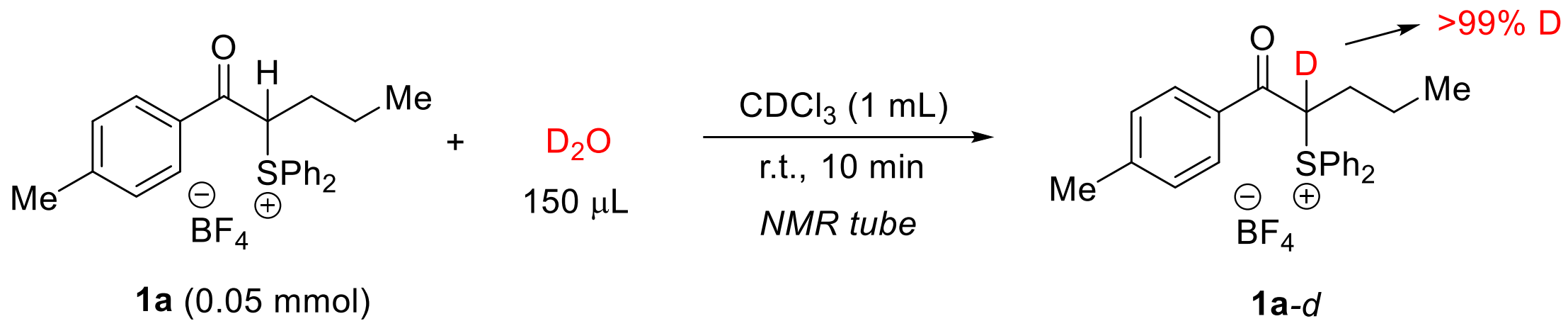

At room temperature, an NMR tube was charged with sulfonium salt **1a** (22.4 mg, 0.05 mmol), D_2O (150 μL), and $CDCl_3$ (1 mL). The mixture was vigorously shaken and allowed to stand for 10 min prior to ^1H NMR measurement.

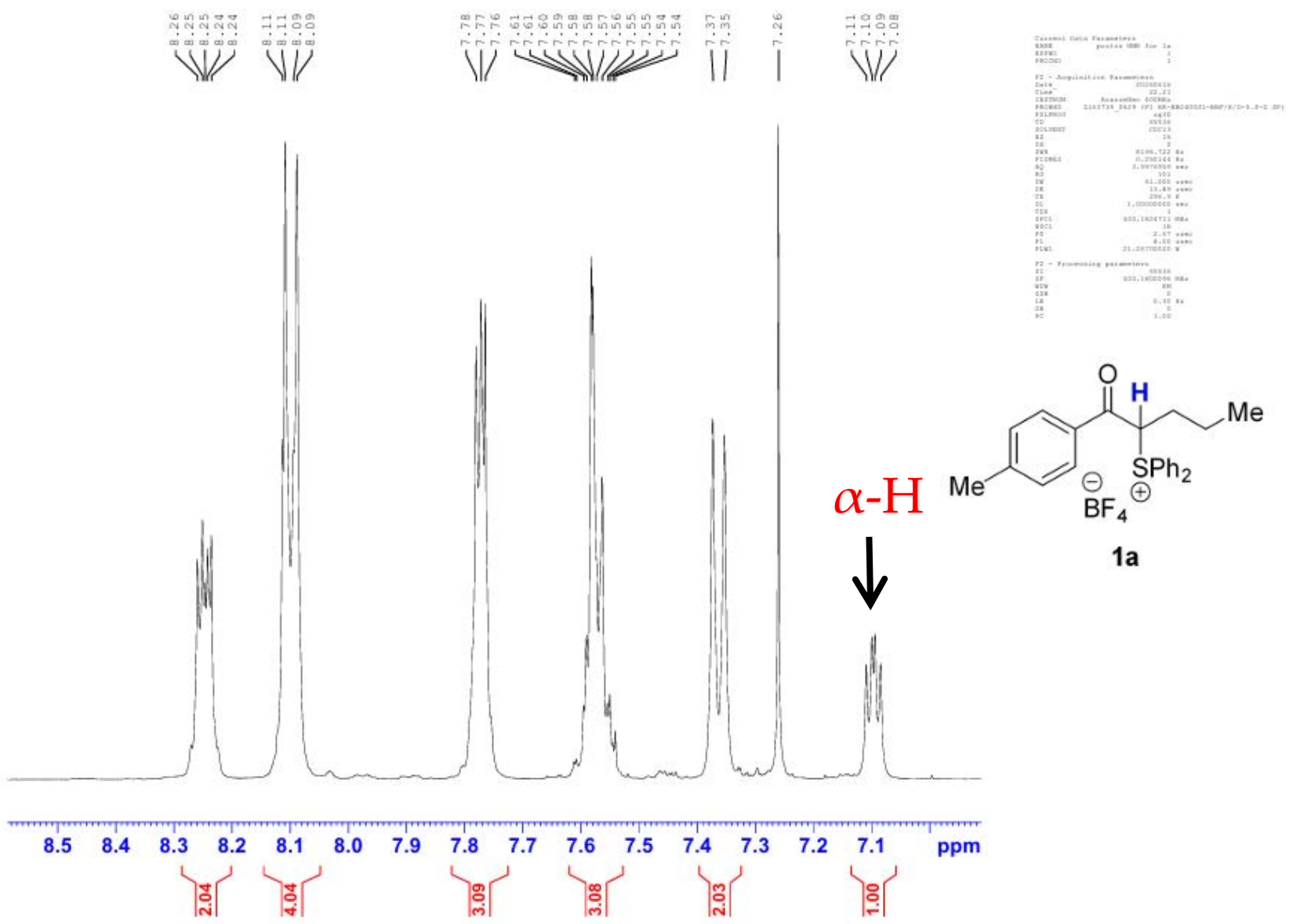

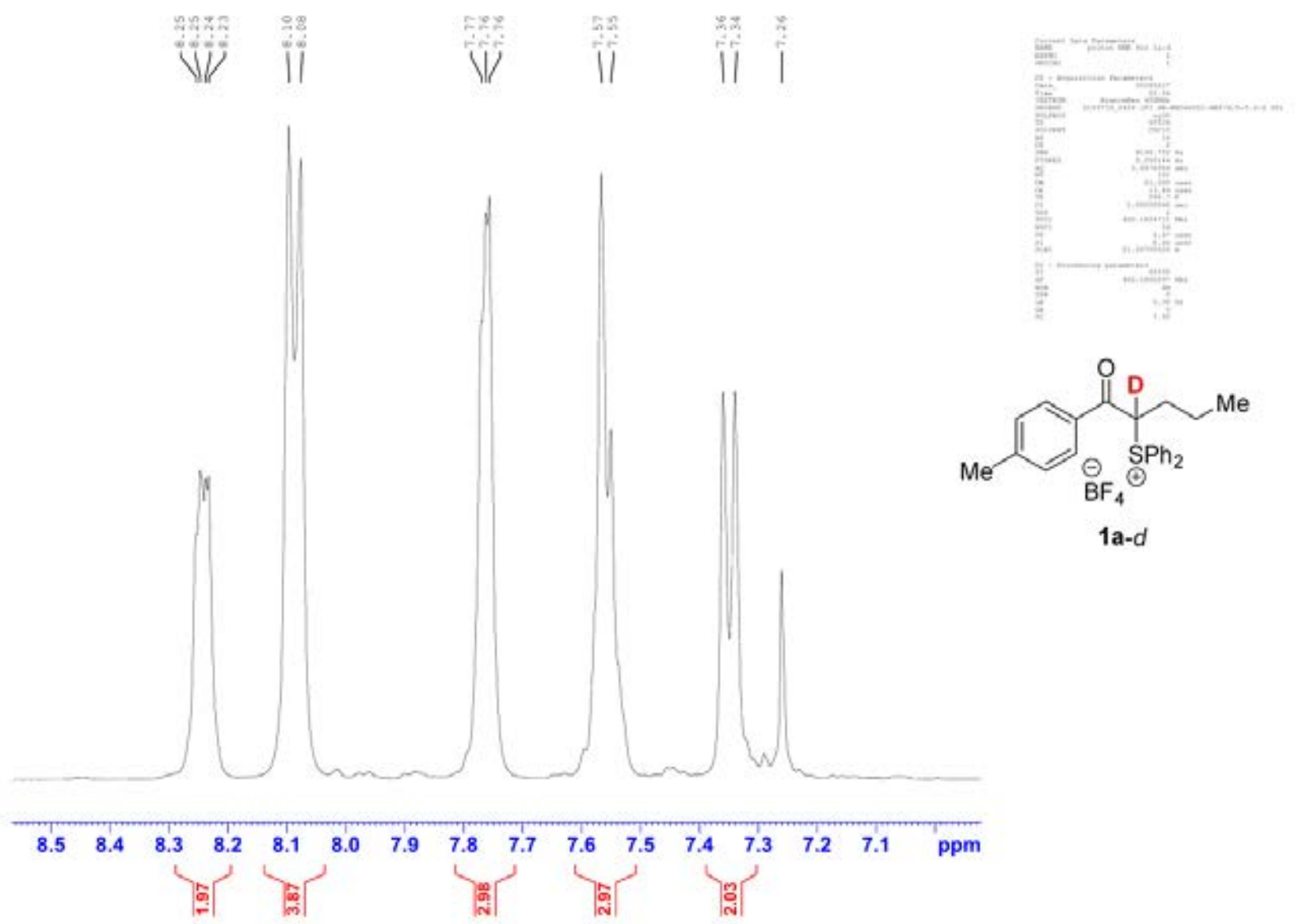

VI. Deprotection of *p*-Methoxyphenyl Group

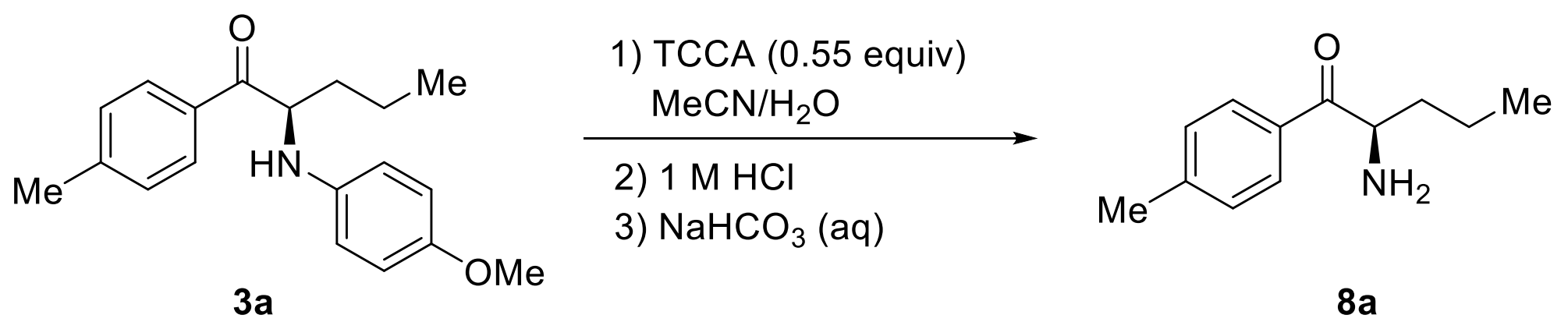

(*R*)-2-Amino-1-(*p*-tolyl)pentan-1-one (8a). To a stirred solution of (*R*)-**3a** (104.1 mg, 0.35 mmol, 1.0 equiv, 95% ee) in CH_3CN/H_2O (8 mL, v/v = 1:1) were sequentially added trichloroisocyanuric acid (TCCA, 45.4 mg, 0.19 mmol, 0.55 equiv) and an aqueous H_2SO_4 solution (0.35 mL, 1 M). Upon addition of TCCA, the reaction mixture turned dark purple. The reaction solution was stirred at ambient temperature, and its color slowly faded from dark purple to red as the reaction proceeded. After stirring for 16 h, an aqueous HCl solution (18 mL, 1 M) was poured into the mixture. The aqueous layer was extracted with dichloromethane, then basified with a saturated aqueous $NaHCO_3$ solution, followed by extraction with dichloromethane. The combined organic layers were concentrated under reduced pressure to afford product **8a** (39.2 mg, 59% yield, 95% ee).

$[\alpha]_D^{25}$: −24.0 (c = 0.8, CH_2Cl_2). HPLC analysis of the product: Daicel CHIRALPAK IBN column; 10% *i*-PrOH in *n*-hexane; 1.0 mL/min; retention times: 7.9 min (minor), 9.3 min (major).

^{1}H NMR (400 MHz, $CDCl_3$) δ 7.85 – 7.81 (m, 2H), 7.28 – 7.26 (m, 2H), 4.39 (dd, *J* = 7.3, 3.9 Hz, 1H), 2.41 (s, 3H), 1.80 (s, 2H), 1.78 – 1.69 (m, 1H), 1.50 – 1.39 (m, 3H), 0.91 (t, *J* = 6.8 Hz,1H).

^{13}C NMR (101 MHz, $CDCl_3$) δ 202.9, 144.3, 132.9, 129.6, 128.5, 55.7, 38.0, 21.8, 19.1, 14.1.

HRMS (ES+) Calcd for $C_{12}H_{18}NO$ [M+H]$^+$: 192.1383, found: 192.1394.

VII. Determination of Absolute Stereochemistry

The product stereochemistry was determined by comparing the optical rotation values of the representative known products with the literature data.

(1) Products **3a**, **3d**, and **7b** are known compounds.[1-3]

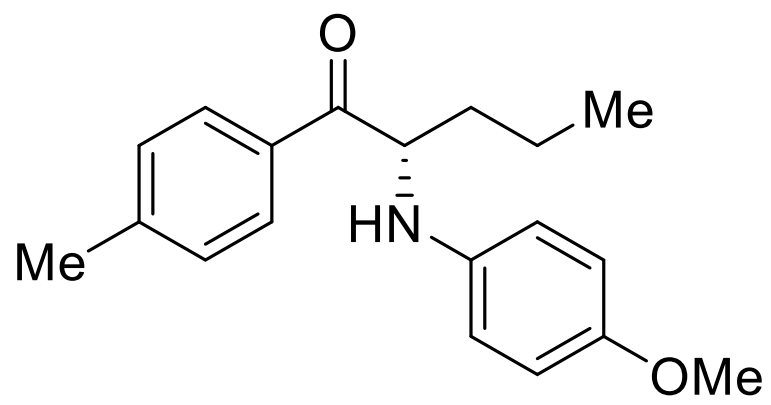

3a

(*S*)-2-((4-Methoxyphenyl)amino)-1-(*p*-tolyl)pentan-1-one (3a). The optical rotation value for (*S*)-**3a** in 95% ee was reported to be $[\alpha]_D^{25}$= +42.2 (c = 1.0, CH_2Cl_2).[1] The measured value of our product in 95% ee is $[\alpha]_D^{22}$: +55.7 (*c* = 1.0, CH_2Cl_2). Thus, the absolute configuration of our product was assigned to be *R*.

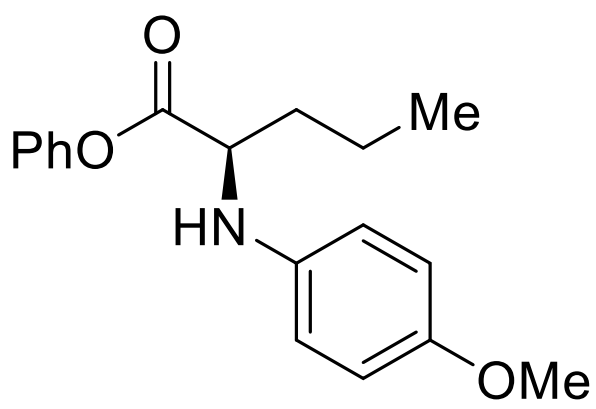

5d

Phenyl (*R*)-2-((4-methoxyphenyl)amino)pentanoate (5d) The optical rotation value for (*R*)-**5d** in 90% ee was reported to be $[\alpha]_D^{25}$ = +95.3 (c = 1.0, $CHCl_3$).[2] The measured value of our product in 92% ee is $[\alpha]_D^{23}$: +97.6 (*c* = 1.0, CH_2Cl_2). Thus, the absolute configuration of our product was assigned to be *R*.

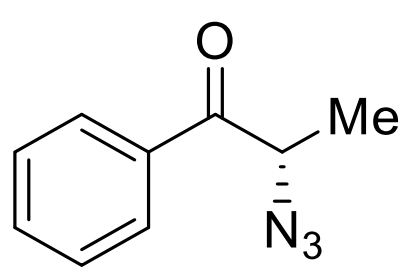

7b

(*S*)-2-Azido-1-phenylpropan-1-one (7b) The optical rotation value for (*R*)-**7b** in

85% ee was reported to be $[\alpha]_D^{25}$ = +138.0 (*c* = 0.95, $CHCl_3$).[3] The measured value of our product in 94% ee is $[\alpha]_D^{21}$: +106.7 (*c* = 1.0, CH_2Cl_2). Thus, the absolute configuration of our product was assigned to be *S*.

(2) Compound **7m** is an unknown compound, but the absolute configuration of its derivative **S5** is known. Therefore, the absolute stereochemistry of **7m** was also deduced by comparison of the optical rotation value of its derivative with the literature data.

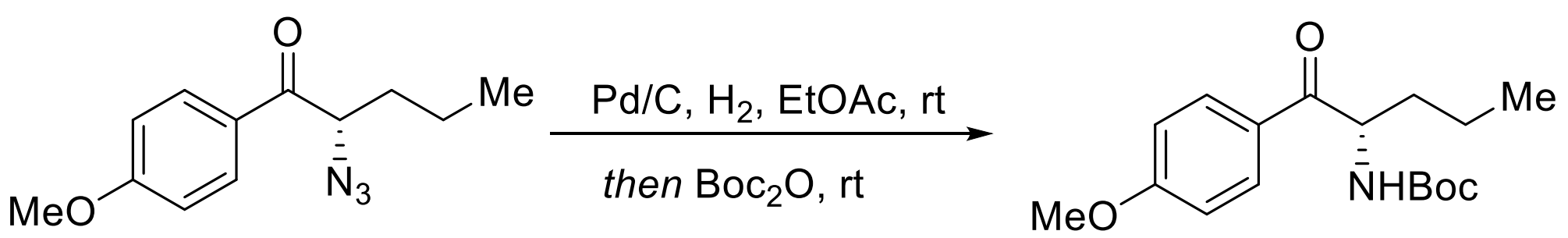

7m **S5**

***tert*-Butyl (*S*)-(1-(4-methoxyphenyl)-1-oxopentan-2-yl)carbamate (S5)** was synthesized according to a modified literature procedure.[1] To a stirred solution of **7m** (11.7 mg, 0.05 mmol, 93% ee) in ethyl acetate (1 mL) was added Pd/C (1.2 mg, 40 mol%). The vial was capped by a septum and filled with H_2 with balloon. Next, Boc_2O (13.0 mg, 1.2 equiv) was injected. The mixture was stirred at room temperature for 12 h, and the reaction progress was monitored by TLC. Upon completion, the mixture was filtered. The filtrate was concentrated, and the residue was purified by preparative TLC to give the desired product **S5** (49% yield, 90% ee).

$[\alpha]_D^{22}$: +23.3 (*c* = 1.0, CH_2Cl_2). HPLC analysis of the product: Daicel CHIRALPAK IC column; 5% *i*-PrOH in hexanes; 1.0 mL/min; retention times: 12.4 min (minor), 19.4 min (major).

^{1}H NMR (400 MHz, $CDCl_3$) δ 8.00 – 7.92 (m, 2H), 7.00 – 6.91 (m, 2H), 5.52 – 5.44 (m, 1H), 5.29 – 5.19 (m, 1H), 3.87 (s, 3H), 1.88 – 1.75 (m, 1H), 1.60 – 1.26 (m, 12H), 0.89 (t, *J* = 7.3 Hz, 3H).

^{13}C NMR (100 MHz, $CDCl_3$) δ 197.9, 163.9, 155.6, 130.9, 127.6, 114.0, 79.5, 55.50, 54.45, 36.1, 28.4, 18.5, 13.9.

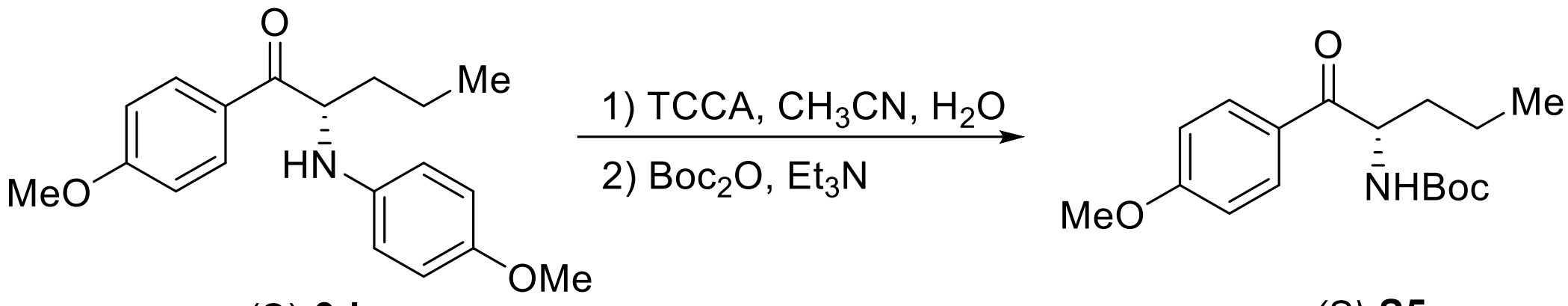

(S)-**3d** (S)-**S5**

***tert*-Butyl (*S*)-(1-(4-methoxyphenyl)-1-oxopentan-2-yl)carbamate** was synthesized from (*S*)-1-(4-methoxyphenyl)-2-((4-methoxyphenyl)amino) pentan-1-one (46.5 mg, 76% yield, 94% ee) according to the literature.[2]

$[\alpha]_D^{22}$: +24.3 (c = 0.5, CH_2Cl_2). HPLC analysis of the product: Daicel CHIRALPAK IC column; 5% *i*-PrOH in hexanes; 1.0 mL/min; retention times: 12.4 min (minor), 19.2 min (major).

By comparing the optical rotation values the configuration of product **7m** was determined to be *S*.

VIII. References

(1) Z. Li, B. Wang, S. Fan, C. Zhang and J. Sun, *J. Am. Chem. Soc.*, 2025, **147**, 576–584.

(2) W. Guo, Y. Luo, H. H. Y. Sung, I. D. Williams, P. Li and J. Sun, *J. Am. Chem. Soc.*, 2020, **142**, 14384–14390.

(3) K. Shibatomi, K. Kitahara, N. Sasaki, Y. Kawasaki, I. Fujisawa and S. Iwasa, *Nat. Commun.*, 2017, **8**, 15600–15607.

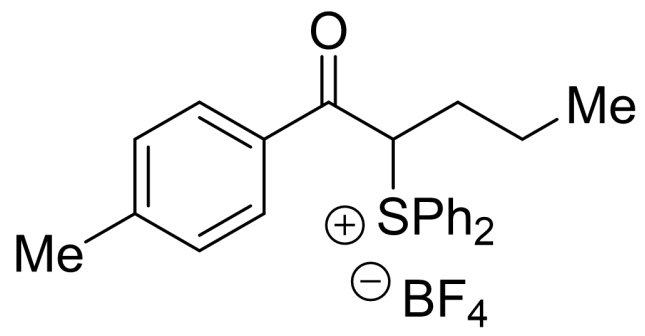

1a

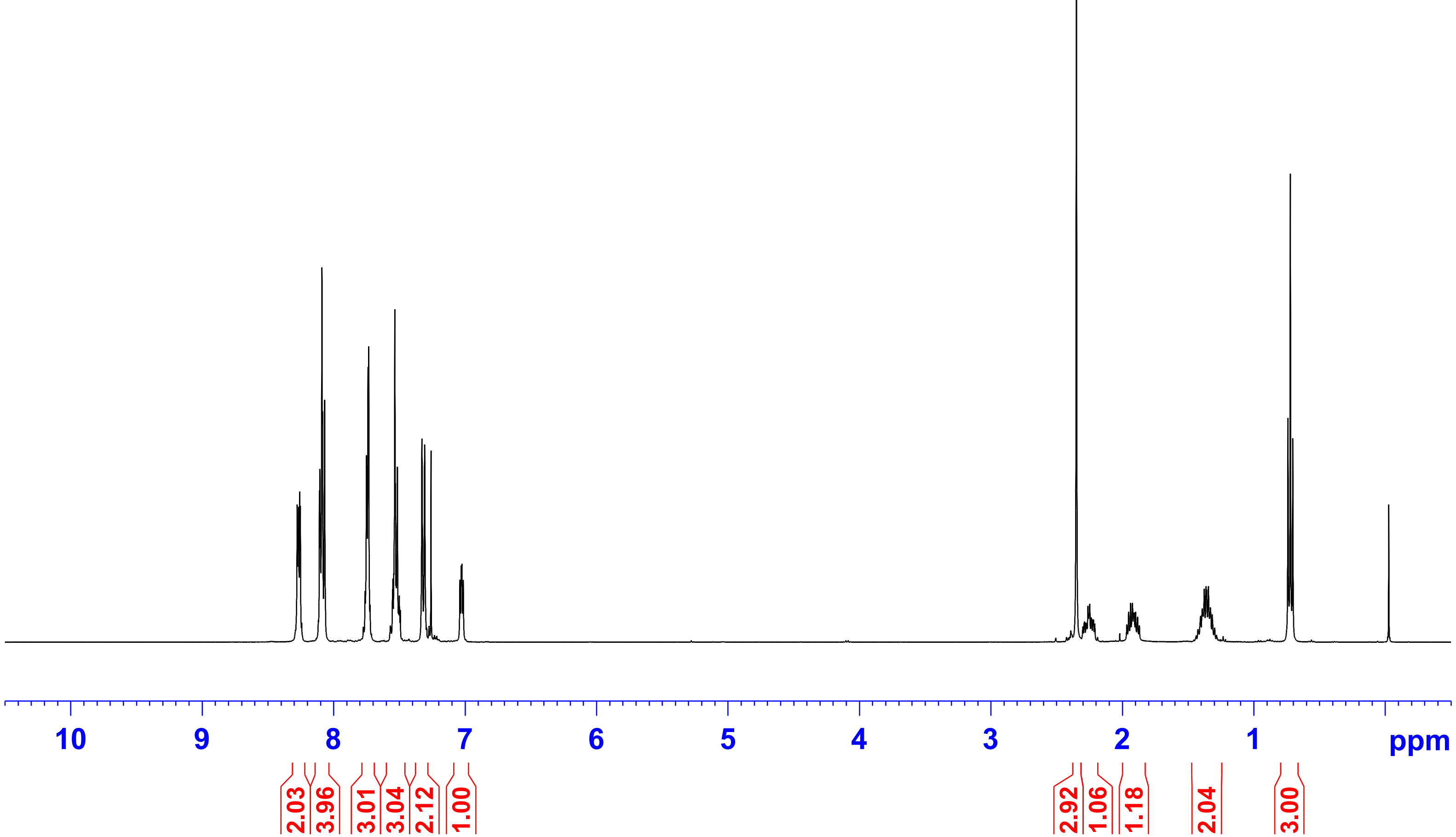

NAME	lzy-04-185-A-3
EXPNO	1
PROCNO	1
Date_	20201102
Time_	22.08
INSTRUM	spect
PROBHD	5 mm PABBO BB/
PULPROG	zg30
TD	65536
SOLVENT	CDCl3
NS	4
DS	0
SWH	8012.820 Hz
FIDRES	0.122266 Hz
AQ	4.0894966 sec
RG	39.46
DW	62.400 usec
DE	6.50 usec
TE	297.2 K
D1	1.00000000 sec
TD0	1

======== CHANNEL f1 ========

SFO1	400.1324710 MHz
NUC1	1H
P1	14.50 usec
SI	65536
SF	400.1300097 MHz
WDW	EM
SSB	0
LB	0.30 Hz
GB	0
PC	1.00

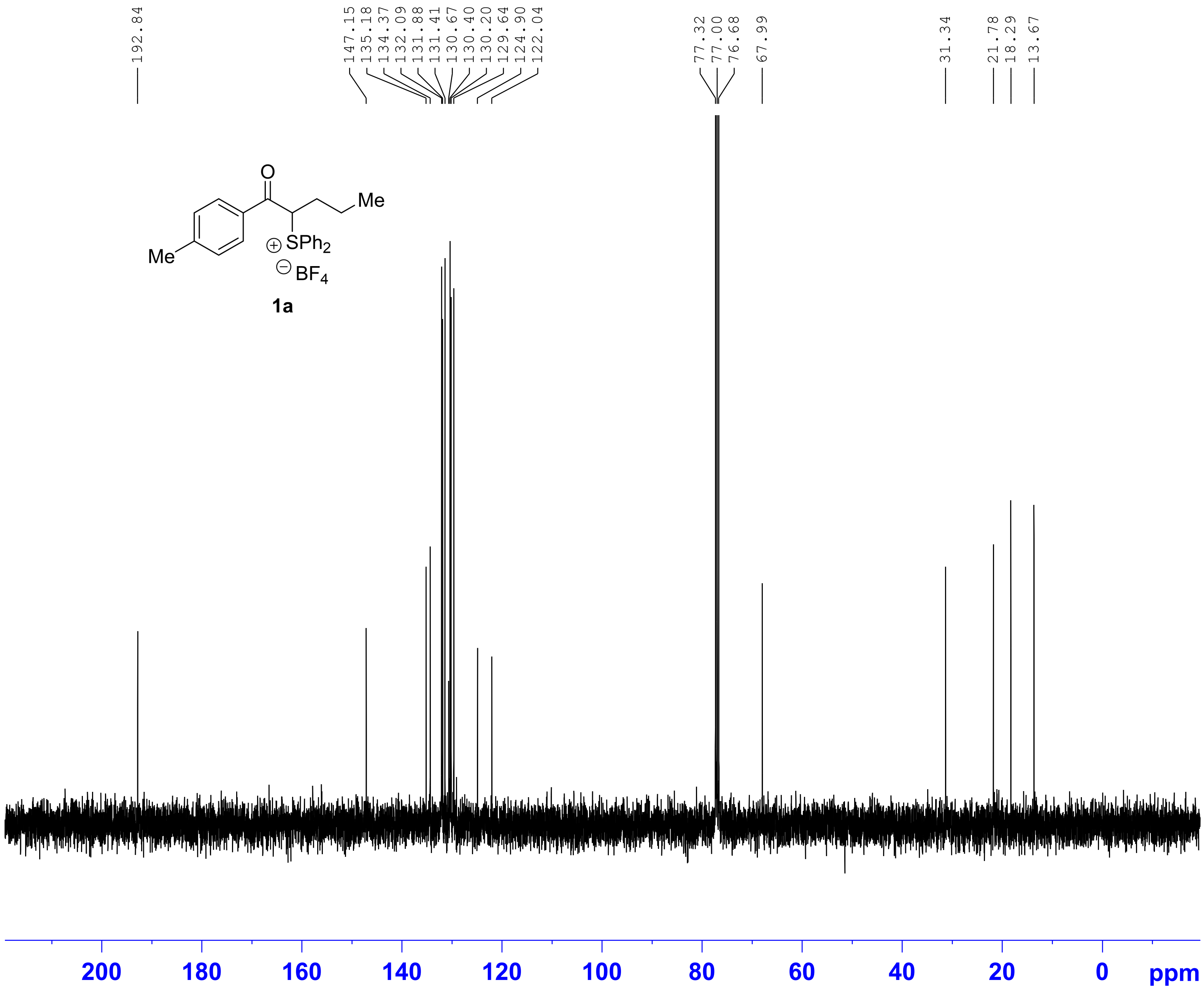

1a

NAME	lzy-04-185-A-3-C
EXPNO	1
PROCNO	1
Date_	20201102
Time	22.10
INSTRUM	spect
PROBHD	5 mm PABBO BB/
PULPROG	zgpg30
TD	65536
SOLVENT	CDCl3
NS	24
DS	0
SWH	24038.461 Hz
FIDRES	0.366798 Hz
AQ	1.3631988 sec
RG	196.92
DW	20.800 usec
DE	6.50 usec
TE	297.8 K
D1	2.00000000 sec
D11	0.03000000 sec
TD0	1

======== CHANNEL f1 ========

SFO1	100.6228298 MHz
NUC1	13C
P1	9.70 usec
SI	32768
SF	100.6127787 MHz
WDW	EM
SSB	0
LB	1.00 Hz
GB	0
PC	1.40

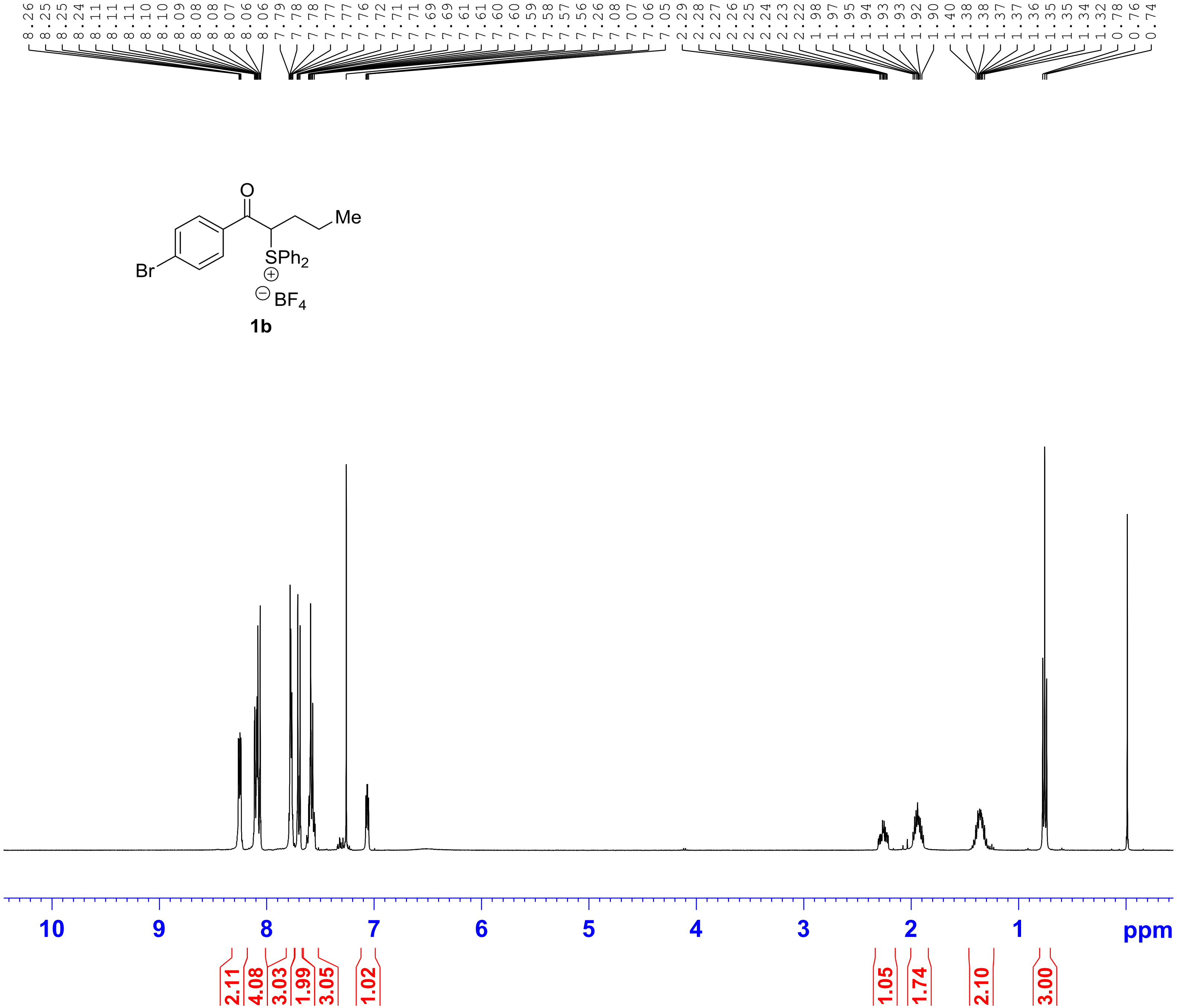

NAME	lzy-04-185-B
EXPNO	1
PROCNO	1
Date_	20201030
Time_	11.06
INSTRUM	spect
PROBHD	5 mm PABBO BB/
PULPROG	zg30
TD	65536
SOLVENT	CDCl3
NS	4
DS	0
SWH	8012.820 Hz
FIDRES	0.122266 Hz
AQ	4.0894966 sec
RG	112.31
DW	62.400 usec
DE	6.50 usec
TE	297.2 K
D1	1.00000000 sec
TD0	1

======== CHANNEL f1 ========

SFO1	400.1324710 MHz
NUC1	1H
P1	14.50 usec
SI	65536
SF	400.1300098 MHz
WDW	EM
SSB	0
LB	0.30 Hz
GB	0
PC	1.00

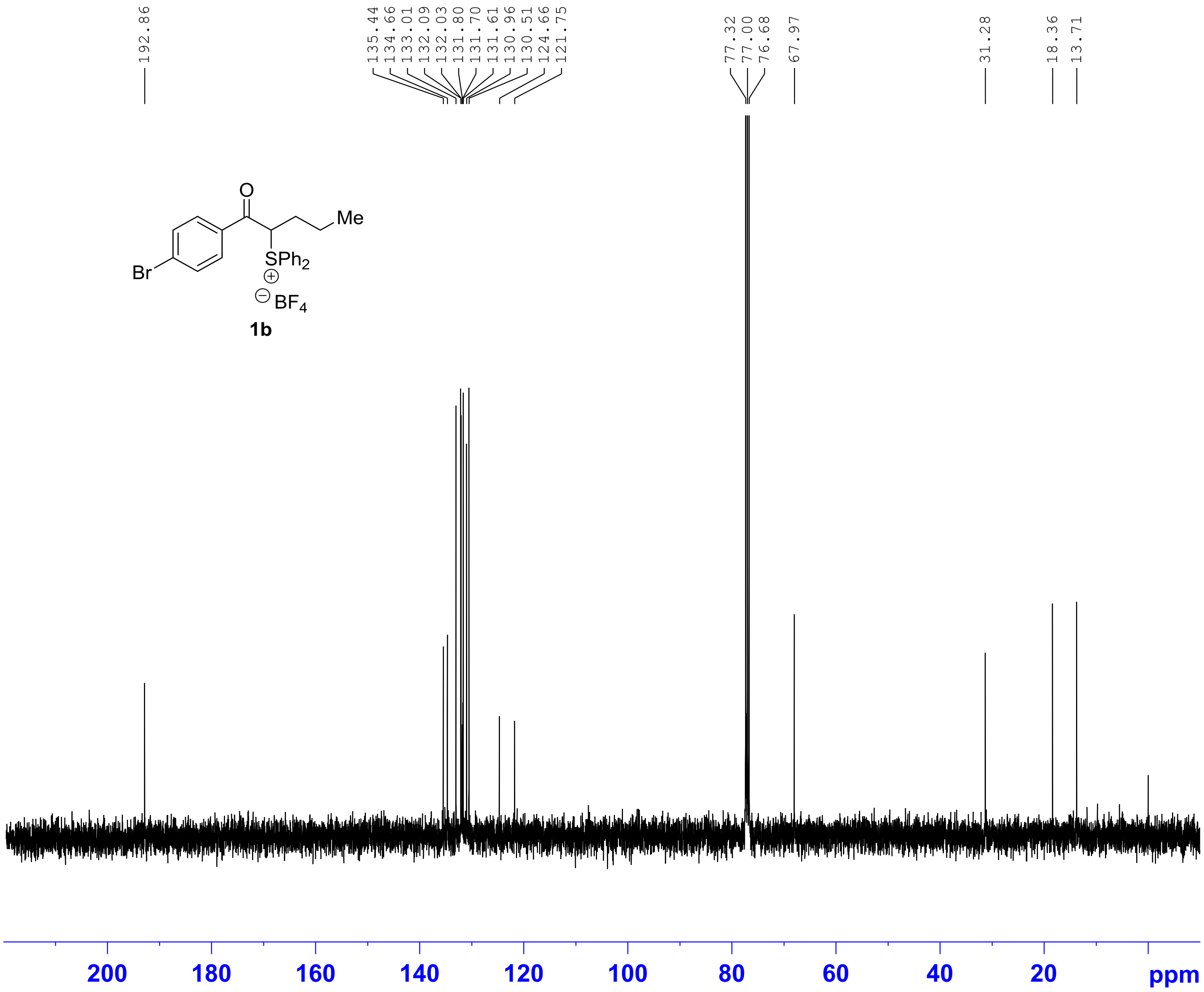

NAME	lzy-04-185-B-C
EXPNO	1
PROCNO	1
Date_	20201030
Time	11.08
INSTRUM	spect
PROBHD	5 mm PABBO BB/
PULPROG	zgpg30
TD	65536
SOLVENT	CDCl3
NS	69
DS	0
SWH	24038.461 Hz
FIDRES	0.366798 Hz
AQ	1.3631988 sec
RG	196.92
DW	20.800 usec
DE	6.50 usec
TE	297.6 K
D1	2.00000000 sec
D11	0.03000000 sec
TD0	1

======== CHANNEL f1 ========

SFO1	100.6228298 MHz
NUC1	13C
P1	9.70 usec
SI	32768
SF	100.6127736 MHz
WDW	EM
SSB	0
LB	1.00 Hz
GB	0
PC	1.40

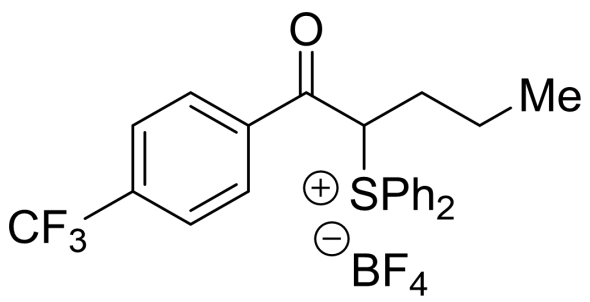

1c

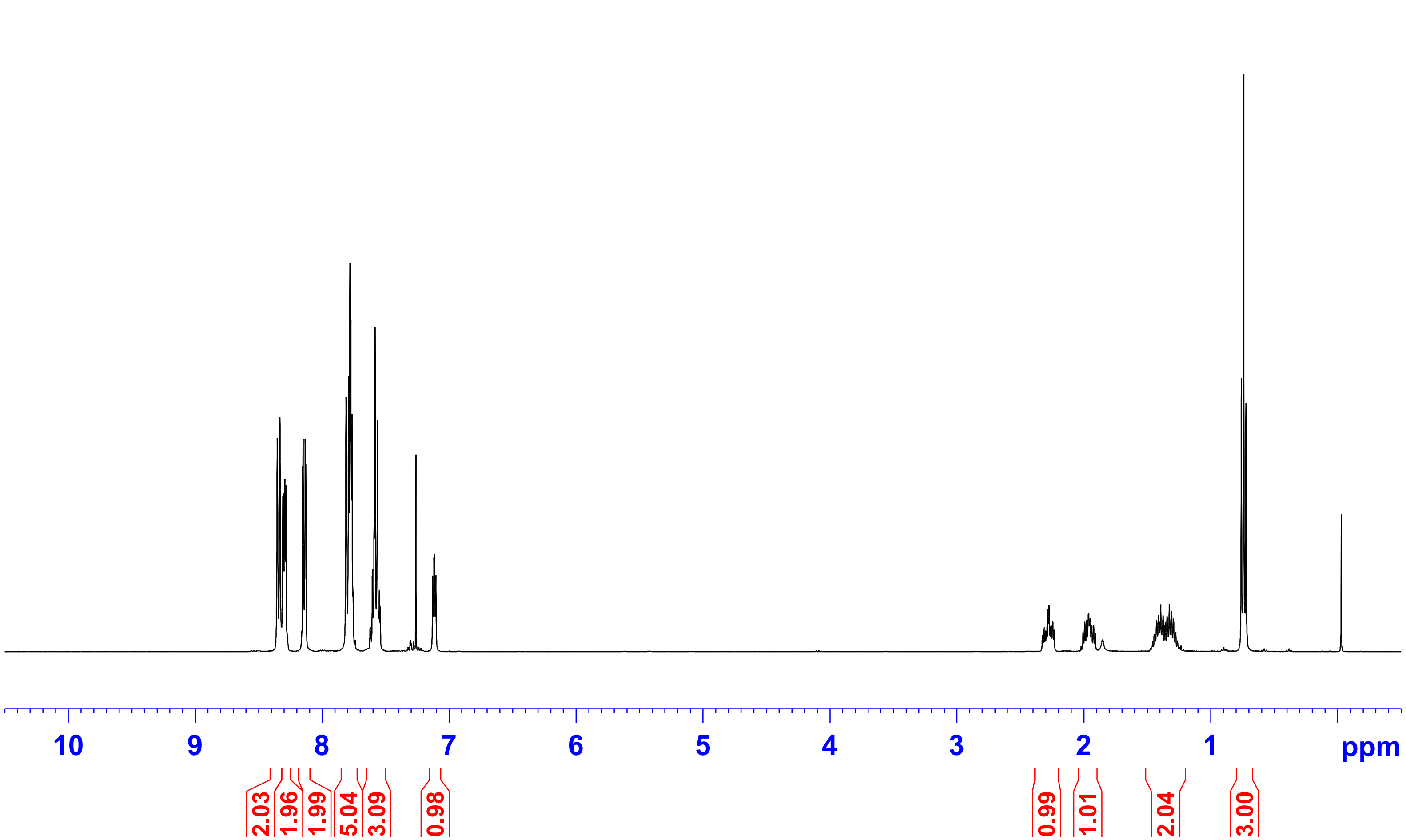

NAME	lzy-05-14	
EXPNO	1	
PROCNO	1	
Date_	20210313	
Time	10.54	
INSTRUM	spect	
PROBHD	5 mm DUL 13C-1	
PULPROG	zg30	
TD	65536	
SOLVENT	CDCl3	
NS	4	
DS	0	
SWH	8223.685	Hz
FIDRES	0.125483	Hz
AQ	3.9846387	sec
RG	161	
DW	60.800	usec
DE	6.00	usec
TE	294.2	K
D1	1.00000000	sec
TD0	1	

======== CHANNEL f1 ========

NUC1	1H	
P1	15.80	usec
PL1	-1.00	dB
PL1W	12.17476940	W
SFO1	400.1324710	MHz
SI	32768	
SF	400.1300093	MHz
WDW	EM	
SSB	0	
LB	0.30	Hz
GB	0	
PC	1.00	

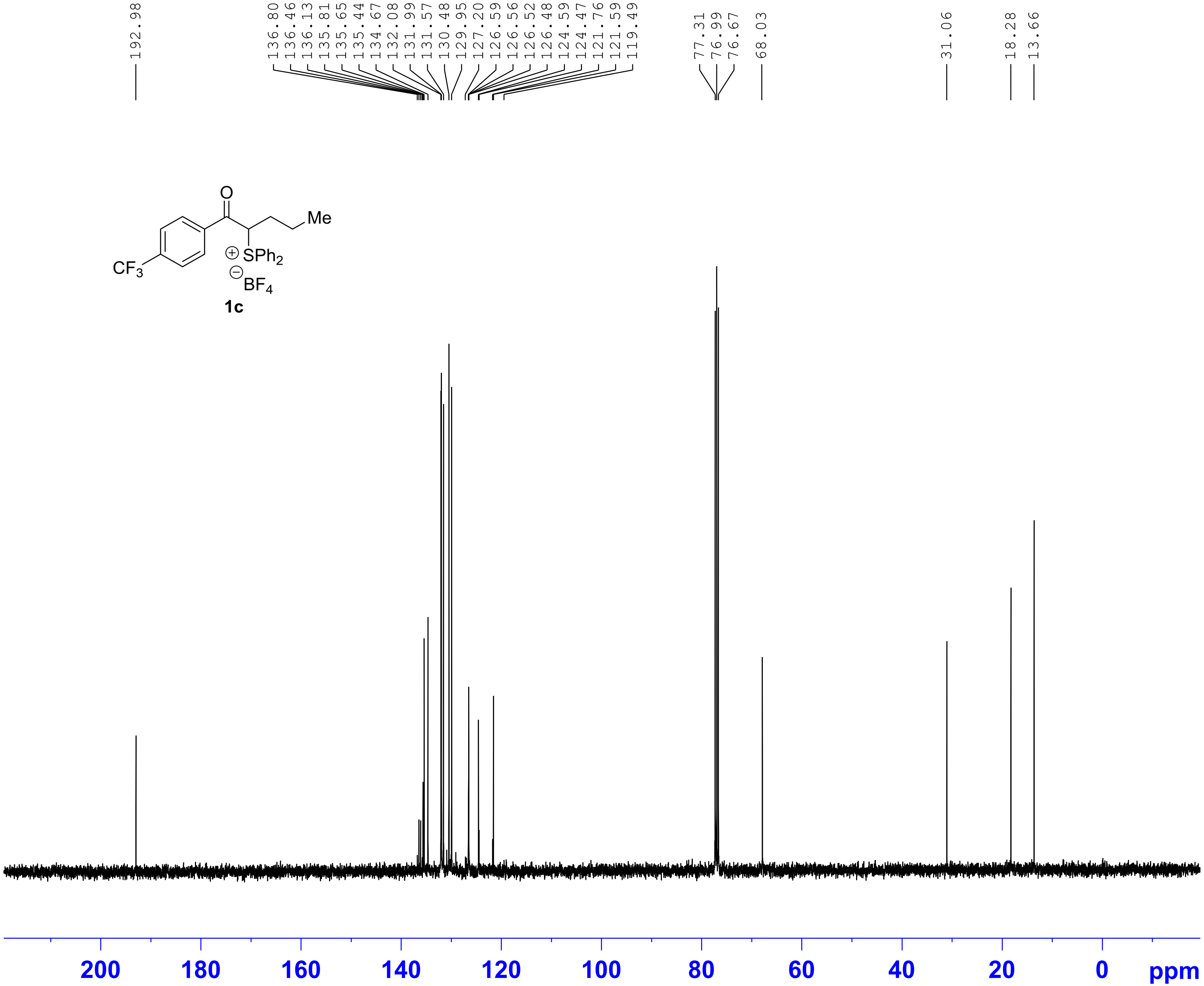

1c

NAME	lzy-05-14-C
EXPNO	1
PROCNO	1
Date_	20210313
Time	10.58
INSTRUM	spect
PROBHD	5 mm DUL 13C-1
PULPROG	zgpg30
TD	65536
SOLVENT	CDCl3
NS	400
DS	0
SWH	24038.461 Hz
FIDRES	0.366798 Hz
AQ	1.3631988 sec
RG	2050
DW	20.800 usec
DE	6.00 usec
TE	294.5 K
D1	2.00000000 sec
D11	0.03000000 sec
TD0	1

======== CHANNEL f1 ========

NUC1	13C
P1	40.00 usec
PL1	-3.00 dB
PL1W	60.64365387 W
SFO1	100.6228298 MHz

======== CHANNEL f2 ========

CPDPRG2	waltz16
NUC2	1H
PCPD2	80.00 usec
PL2	-1.00 dB
PL12	14.39 dB
PL13	18.00 dB
PL2W	12.17476940 W
PL12W	0.35193357 W
PL13W	0.15327126 W
SFO2	400.1316005 MHz
SI	32768
SF	100.6127782 MHz
WDW	EM
SSB	0
LB	1.00 Hz
GB	0
PC	1.40

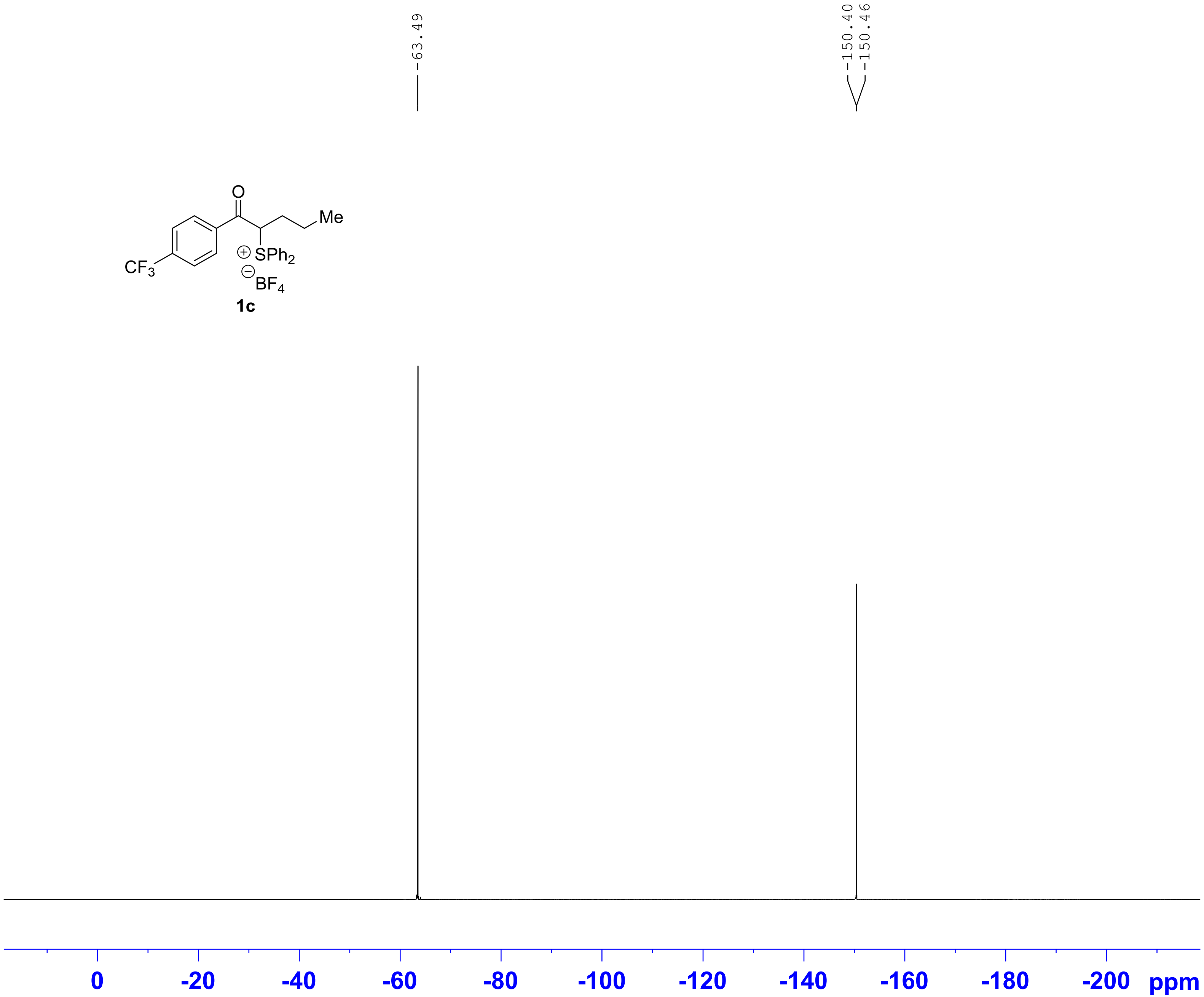

NAME	lzy-04-185-L-1112-F
EXPNO	1
PROCNO	1
Date_	20201112
Time	11.16
INSTRUM	spect
PROBHD	5 mm PABBO BB/
PULPROG	zgflqn
TD	131072
SOLVENT	CDCl3
NS	16
DS	0
SWH	89285.711 Hz
FIDRES	0.681196 Hz
AQ	0.7340532 sec
RG	196.92
DW	5.600 usec
DE	6.50 usec
TE	297.5 K
D1	1.00000000 sec
TD0	1

======== CHANNEL f1 ========

SFO1	376.4607164 MHz
NUC1	19F
P1	14.70 usec
SI	65536
SF	376.4983660 MHz
WDW	EM
SSB	0
LB	0.30 Hz
GB	0
PC	1.00

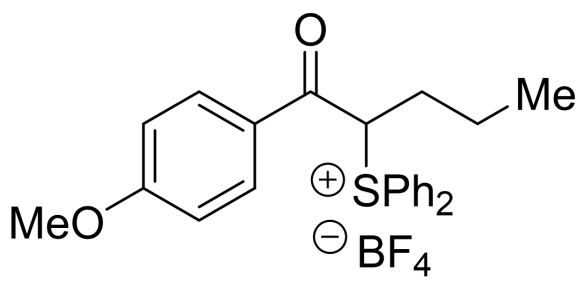

1d

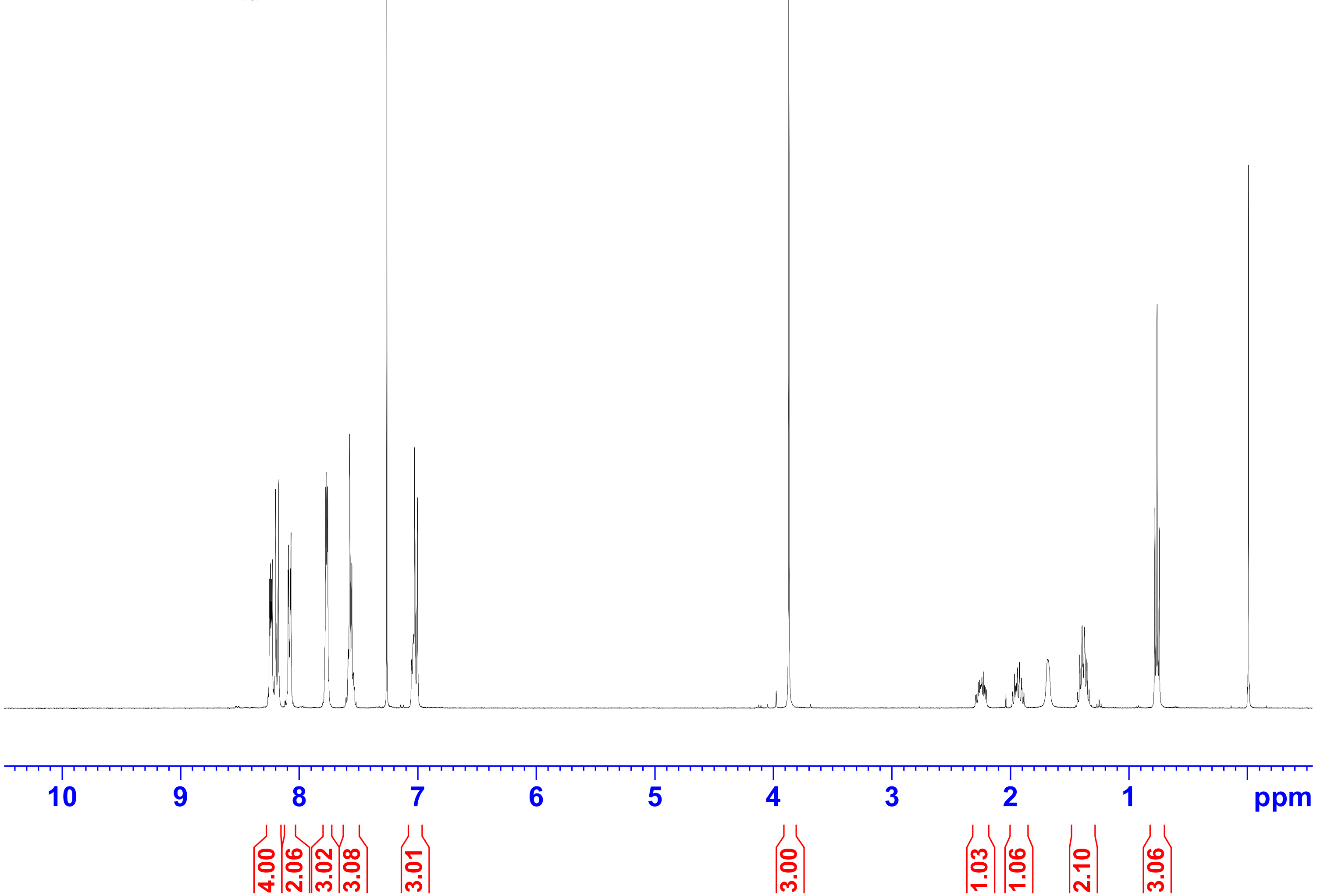


```
Current Data Parameters
NAME              lzy-04-185-C
EXPNO                        1
PROCNO                       1

F2 - Acquisition Parameters
Date_                 20201031
Time                     18.58
INSTRUM                  spect
PROBHD     5 mm PABBO BB/19F-1H/D Z-GRD Z108618/0582
PULPROG                   zg30
TD                       65536
SOLVENT                  CDCl3
NS                           4
DS                           0
SWH                   8012.820 Hz
FIDRES                0.122266 Hz
AQ                   4.0894465 sec
RG                      126.97
DW                      62.400 usec
DE                        6.50 usec
TE                       297.5 K
D1                  1.00000000 sec
TD0                          1

======== CHANNEL f1 ========
SFO1               400.1324710 MHz
NUC1                        1H
P1                       14.50 usec
PLW1               11.99499989 W

F2 - Processing parameters
SI                       65536
SF                 400.1300099 MHz
WDW                         EM
SSB                          0
LB                        0.30 Hz
GB                           0
PC                        1.00
```

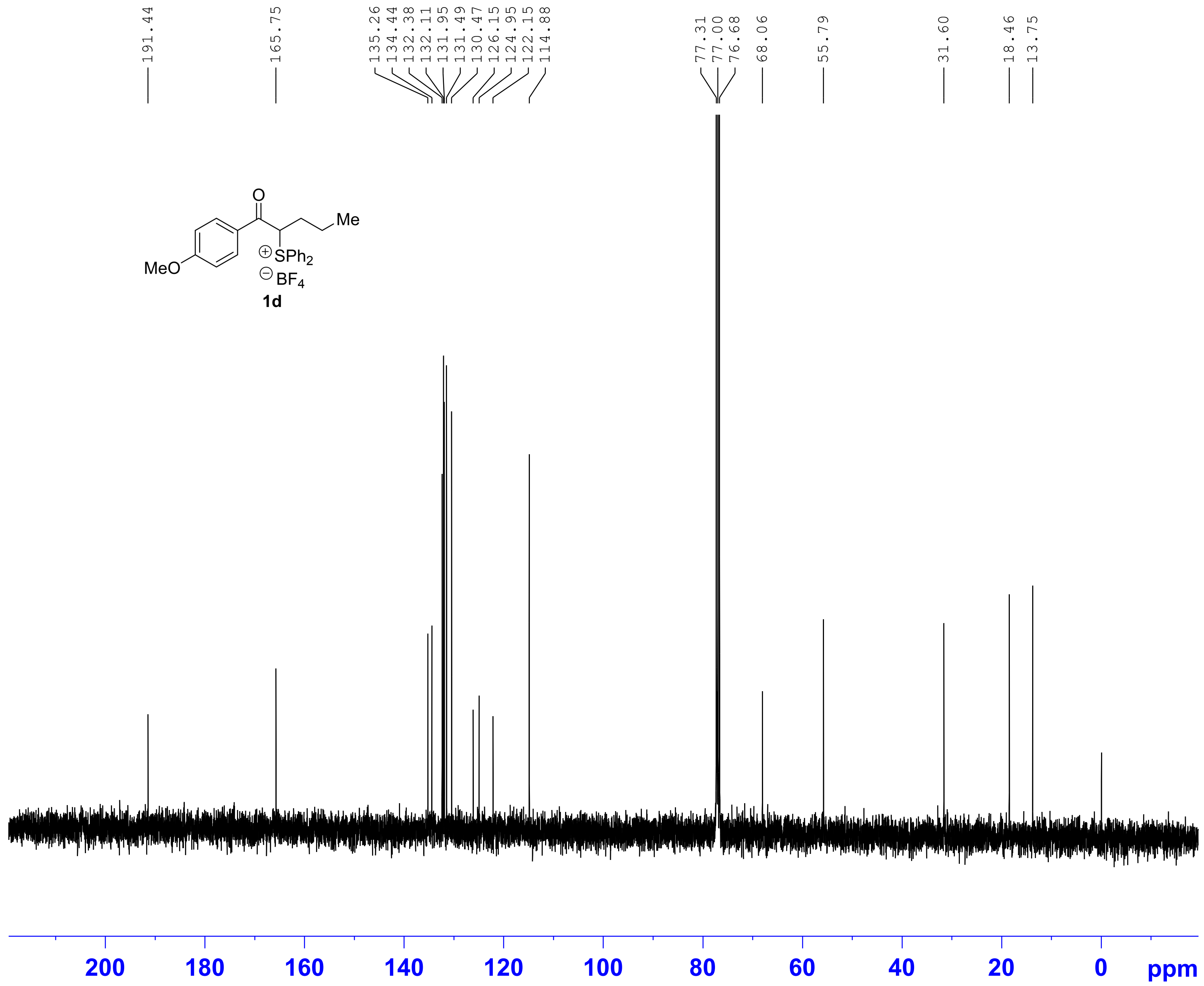

NAME	lzy-04-185-C-C	
EXPNO	1	
PROCNO	1	
Date_	20201031	
Time	19.01	
INSTRUM	spect	
PROBHD	5 mm PABBO BB/	
PULPROG	zgpg30	
TD	65536	
SOLVENT	CDCl3	
NS	230	
DS	0	
SWH	24038.461	Hz
FIDRES	0.366798	Hz
AQ	1.3631988	sec
RG	196.92	
DW	20.800	usec
DE	6.50	usec
TE	298.3	K
D1	2.00000000	sec
D11	0.03000000	sec
TD0	1	

======== CHANNEL f1 ========

SFO1	100.6228298	MHz
NUC1	13C	
P1	9.70	usec
SI	32768	
SF	100.6127728	MHz
WDW	EM	
SSB	0	
LB	1.00	Hz
GB	0	
PC	1.40	

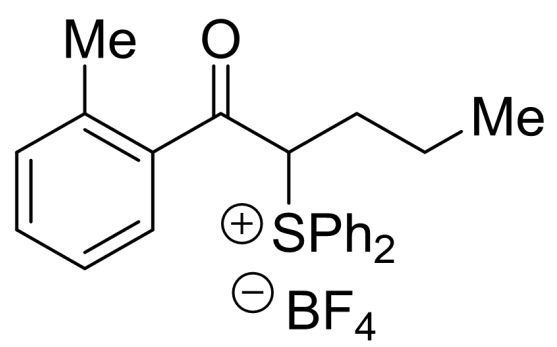

1e

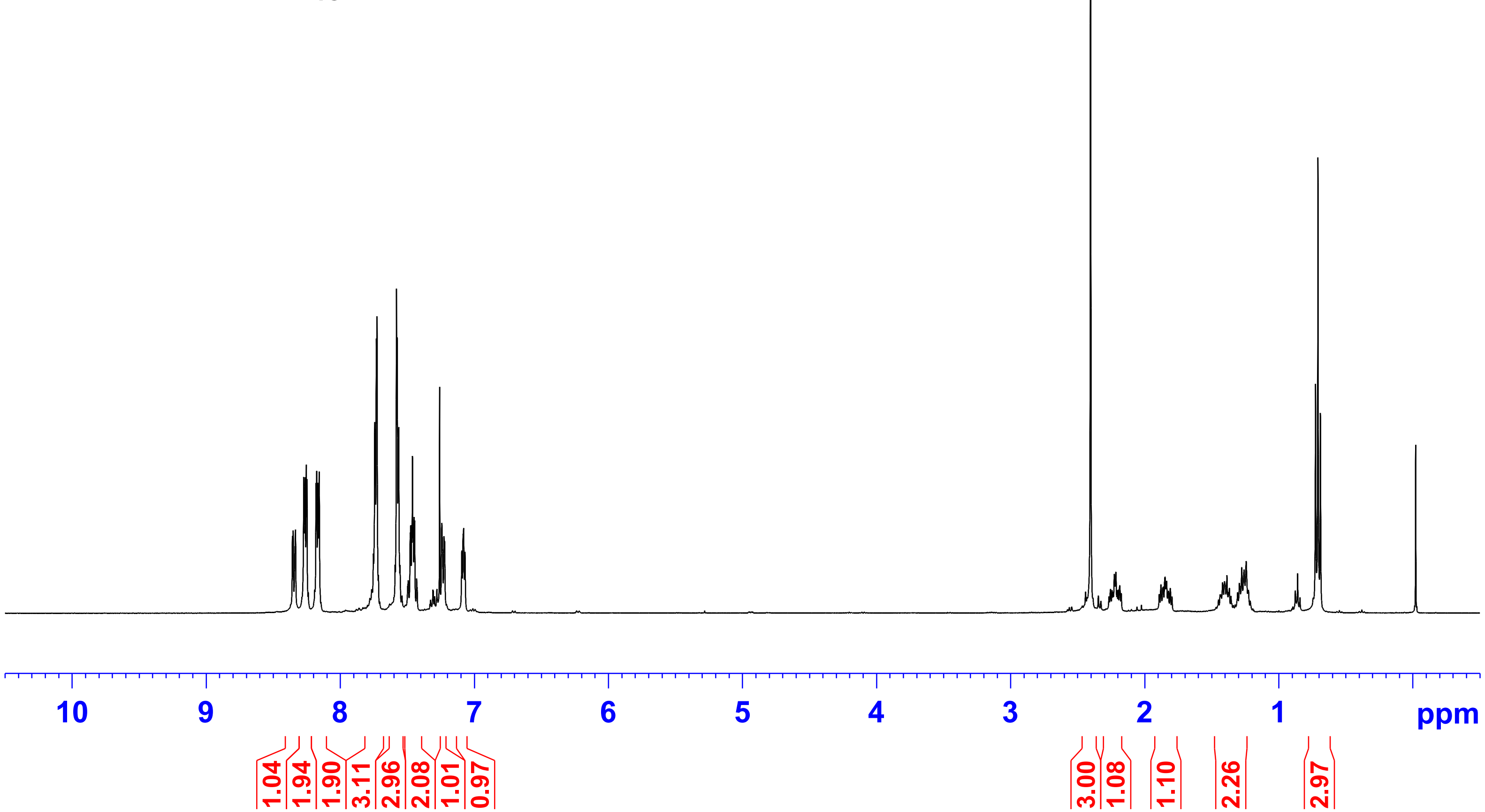

NAME	lzy-04-185-D
EXPNO	1
PROCNO	1
Date_	20201031
Time	18.34
INSTRUM	spect
PROBHD	5 mm PABBO BB/
PULPROG	zg30
TD	65536
SOLVENT	CDCl3
NS	4
DS	0
SWH	8012.820 Hz
FIDRES	0.122266 Hz
AQ	4.0894966 sec
RG	39.46
DW	62.400 usec
DE	6.50 usec
TE	297.6 K
D1	1.00000000 sec
TD0	1

======== CHANNEL f1 ========

SFO1	400.1324710 MHz
NUC1	1H
P1	14.50 usec
SI	65536
SF	400.1300098 MHz
WDW	EM
SSB	0
LB	0.30 Hz
GB	0
PC	1.00

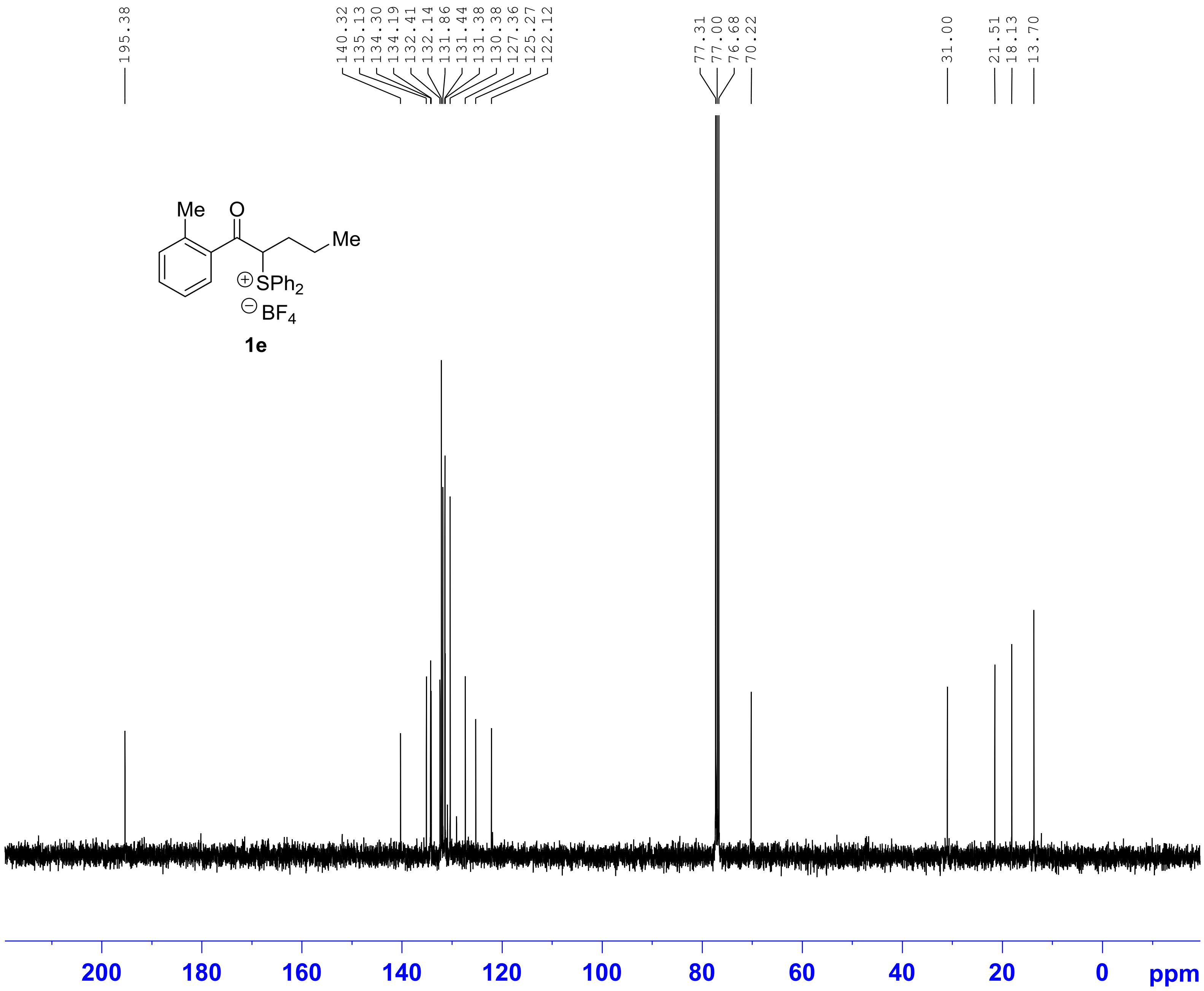


```
NAME       lzy-04-185-D-C
EXPNO                   1
PROCNO                  1
Date_            20201031
Time                18.36
INSTRUM             spect
PROBHD   5 mm PABBO BB/
PULPROG           zgpg30
TD                  65536
SOLVENT             CDCl3
NS                     75
DS                      0
SWH             24038.461 Hz
FIDRES           0.366798 Hz
AQ              1.3631988 sec
RG                 196.92
DW                 20.800 usec
DE                   6.50 usec
TE                  298.3 K
D1             2.00000000 sec
D11            0.03000000 sec
TD0                     1

======== CHANNEL f1 ========
SFO1          100.6228298 MHz
NUC1                  13C
P1                   9.70 usec
SI                  32768
SF            100.6127780 MHz
WDW                    EM
SSB                     0
LB                   1.00 Hz
GB                      0
PC                   1.40
```

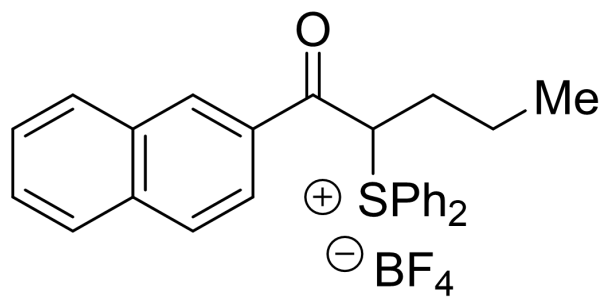

1f

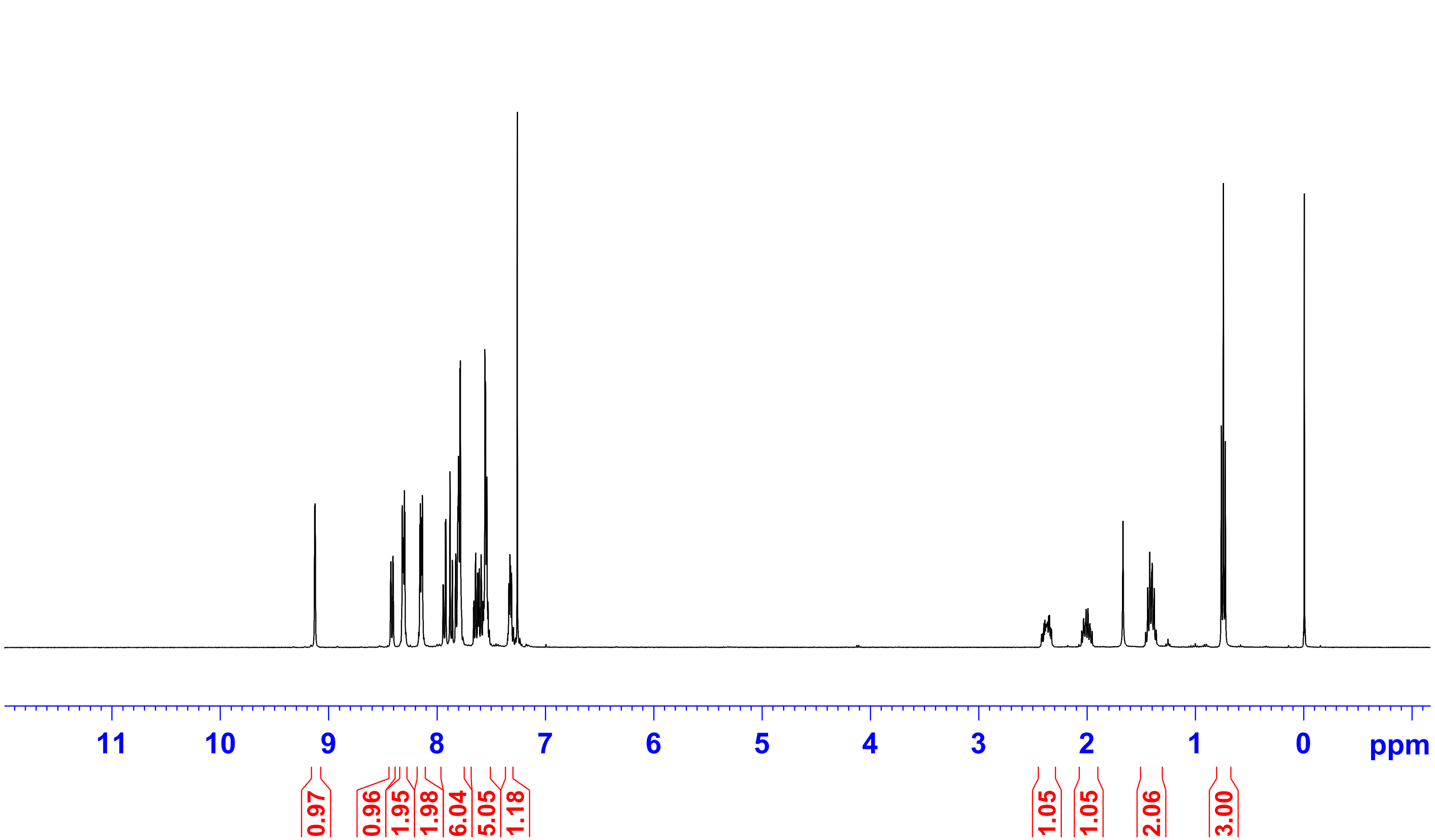


```
NAME          lzy-04-185-E
EXPNO                    1
PROCNO                   1
Date_             20201031
Time                 21.57
INSTRUM              spect
PROBHD     5 mm PABBO BB/
PULPROG               zg30
TD                   65536
SOLVENT              CDCl3
NS                       4
DS                       0
SWH               8012.820 Hz
FIDRES            0.122266 Hz
AQ               4.0894966 sec
RG                  112.31
DW                  62.400 usec
DE                    6.50 usec
TE                   297.9 K
D1              1.00000000 sec
TD0                      1

======== CHANNEL f1 ========
SFO1           400.1324710 MHz
NUC1                    1H
P1                   14.50 usec
SI                   65536
SF             400.1300100 MHz
WDW                     EM
SSB                      0
LB                    0.30 Hz
GB                       0
PC                    1.00
```

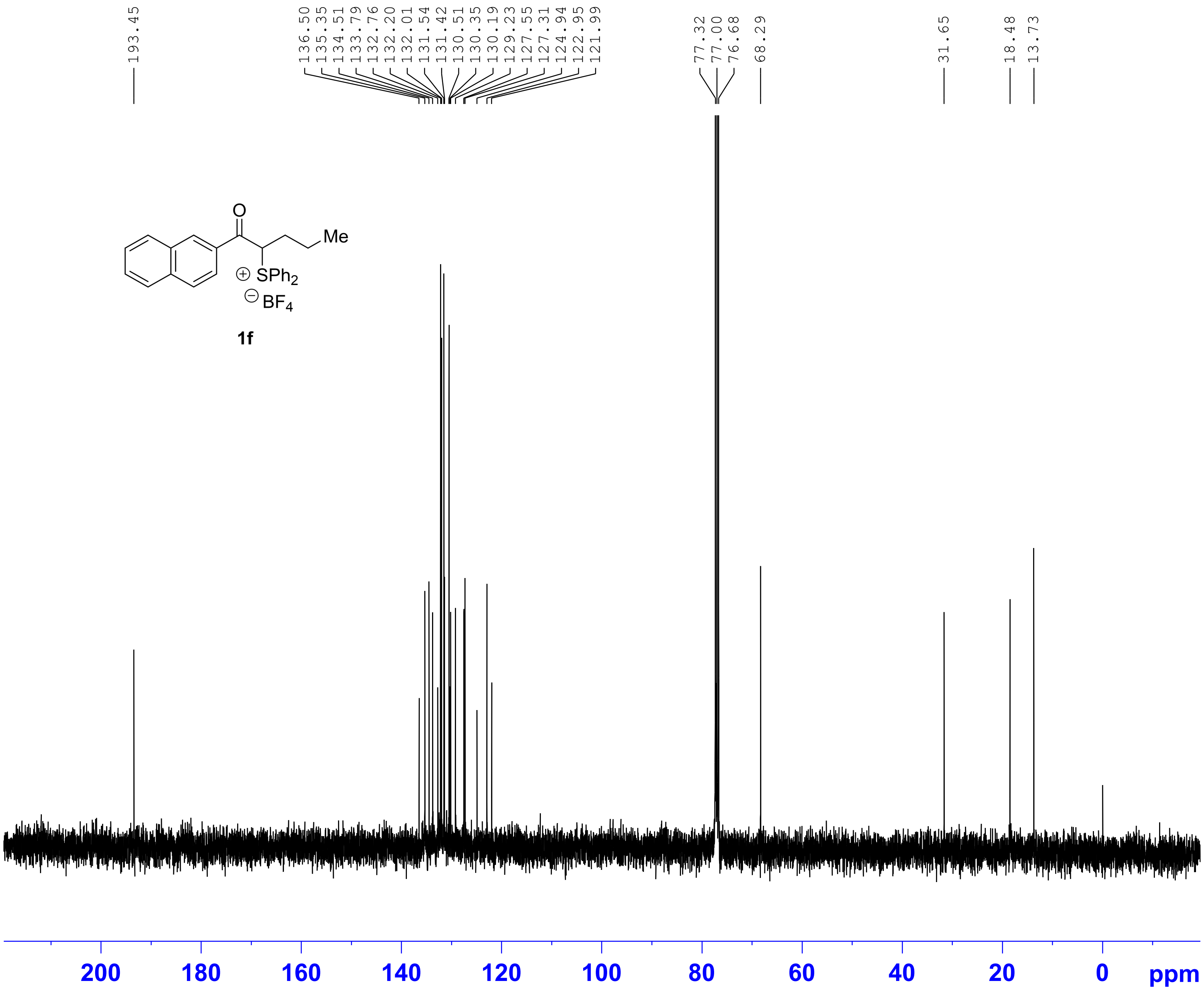

NAME	lzy-04-185-E-C	
EXPNO	1	
PROCNO	1	
Date_	20201031	
Time	21.59	
INSTRUM	spect	
PROBHD	5 mm PABBO BB/	
PULPROG	zgpg30	
TD	65536	
SOLVENT	CDCl3	
NS	150	
DS	0	
SWH	24038.461	Hz
FIDRES	0.366798	Hz
AQ	1.3631988	sec
RG	196.92	
DW	20.800	usec
DE	6.50	usec
TE	298.7	K
D1	2.00000000	sec
D11	0.03000000	sec
TD0	1	

======== CHANNEL f1 ========

SFO1	100.6228298	MHz
NUC1	13C	
P1	9.70	usec
SI	32768	
SF	100.6127729	MHz
WDW	EM	
SSB	0	
LB	1.00	Hz
GB	0	
PC	1.40	

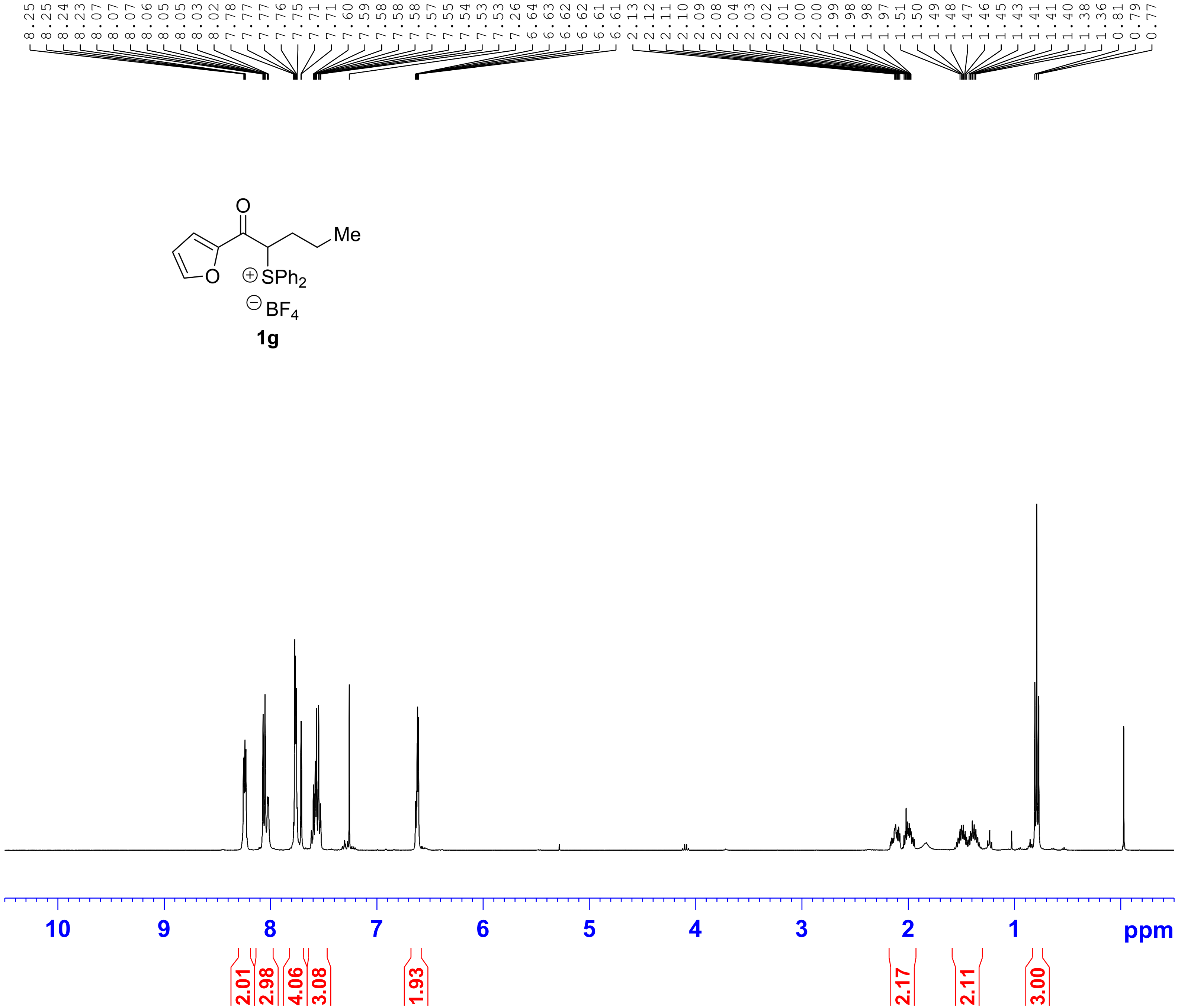

NAME	lzy-04-185-F
EXPNO	1
PROCNO	1
Date_	20201030
Time_	10.43
INSTRUM	spect
PROBHD	5 mm PABBO BB/
PULPROG	zg30
TD	65536
SOLVENT	CDCl3
NS	4
DS	0
SWH	8012.820 Hz
FIDRES	0.122266 Hz
AQ	4.0894966 sec
RG	45.67
DW	62.400 usec
DE	6.50 usec
TE	297.2 K
D1	1.00000000 sec
TD0	1

======== CHANNEL f1 ========

SFO1	400.1324710 MHz
NUC1	1H
P1	14.50 usec
SI	65536
SF	400.1300098 MHz
WDW	EM
SSB	0
LB	0.30 Hz
GB	0
PC	1.00

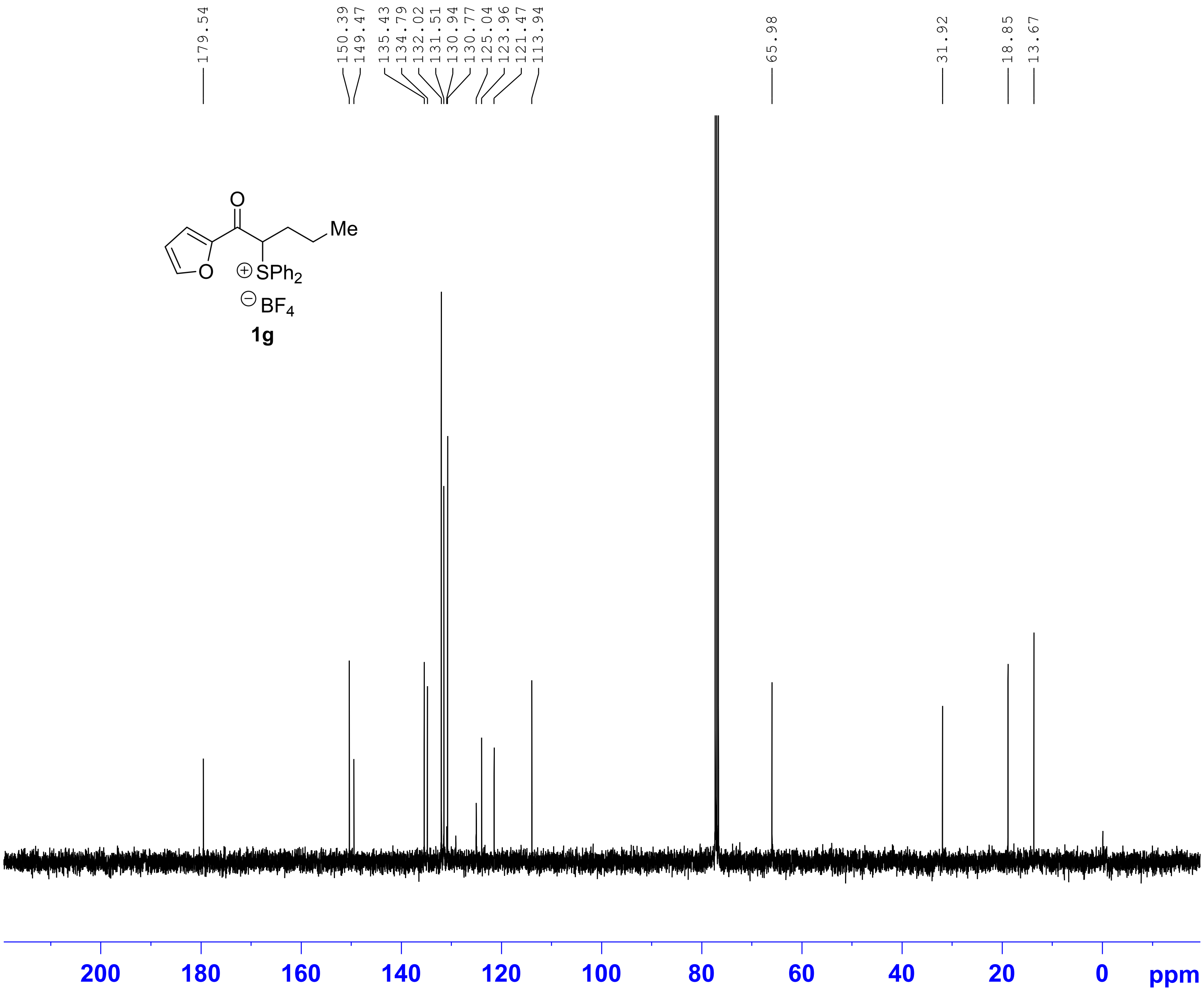

NAME	lzy-04-185-F-C	
EXPNO	1	
PROCNO	1	
Date_	20201030	
Time	10.46	
INSTRUM	spect	
PROBHD	5 mm PABBO BB/	
PULPROG	zgpg30	
TD	65536	
SOLVENT	CDCl3	
NS	61	
DS	0	
SWH	24038.461	Hz
FIDRES	0.366798	Hz
AQ	1.3631988	sec
RG	196.92	
DW	20.800	usec
DE	6.50	usec
TE	298.0	K
D1	2.00000000	sec
D11	0.03000000	sec
TD0	1	

======== CHANNEL f1 ========

SFO1	100.6228298	MHz
NUC1	13C	
P1	9.70	usec
SI	32768	
SF	100.6127765	MHz
WDW	EM	
SSB	0	
LB	1.00	Hz
GB	0	
PC	1.40	

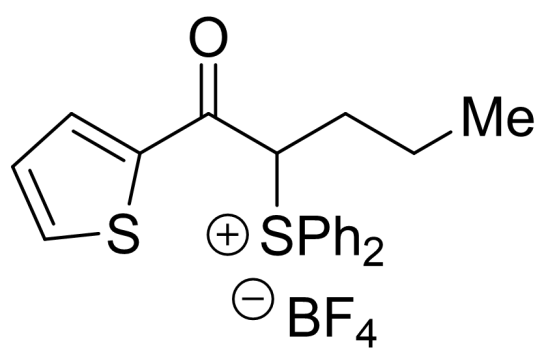

1h

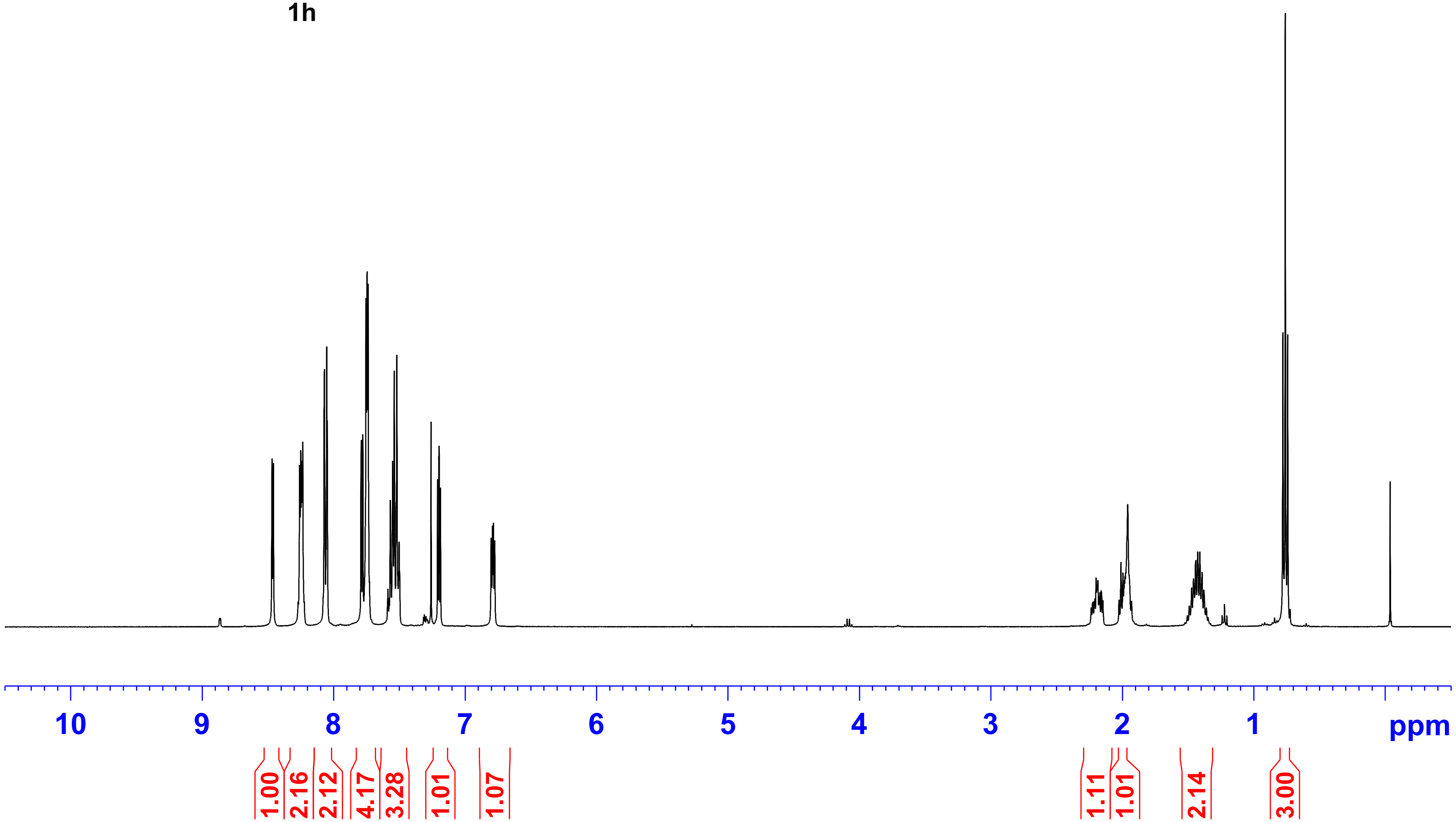

NAME	lzy-04-185-g-02
EXPNO	1
PROCNO	1
Date_	20201030
Time	15.04
INSTRUM	spect
PROBHD	5 mm PABBO BB/
PULPROG	zg30
TD	65536
SOLVENT	CDCl3
NS	4
DS	0
SWH	8012.820 Hz
FIDRES	0.122266 Hz
AQ	4.0894966 sec
RG	39.46
DW	62.400 usec
DE	6.50 usec
TE	296.7 K
D1	1.00000000 sec
TD0	1

======== CHANNEL f1 ========

SFO1	400.1324710 MHz
NUC1	1H
P1	14.50 usec
SI	65536
SF	400.1300099 MHz
WDW	EM
SSB	0
LB	0.30 Hz
GB	0
PC	1.00

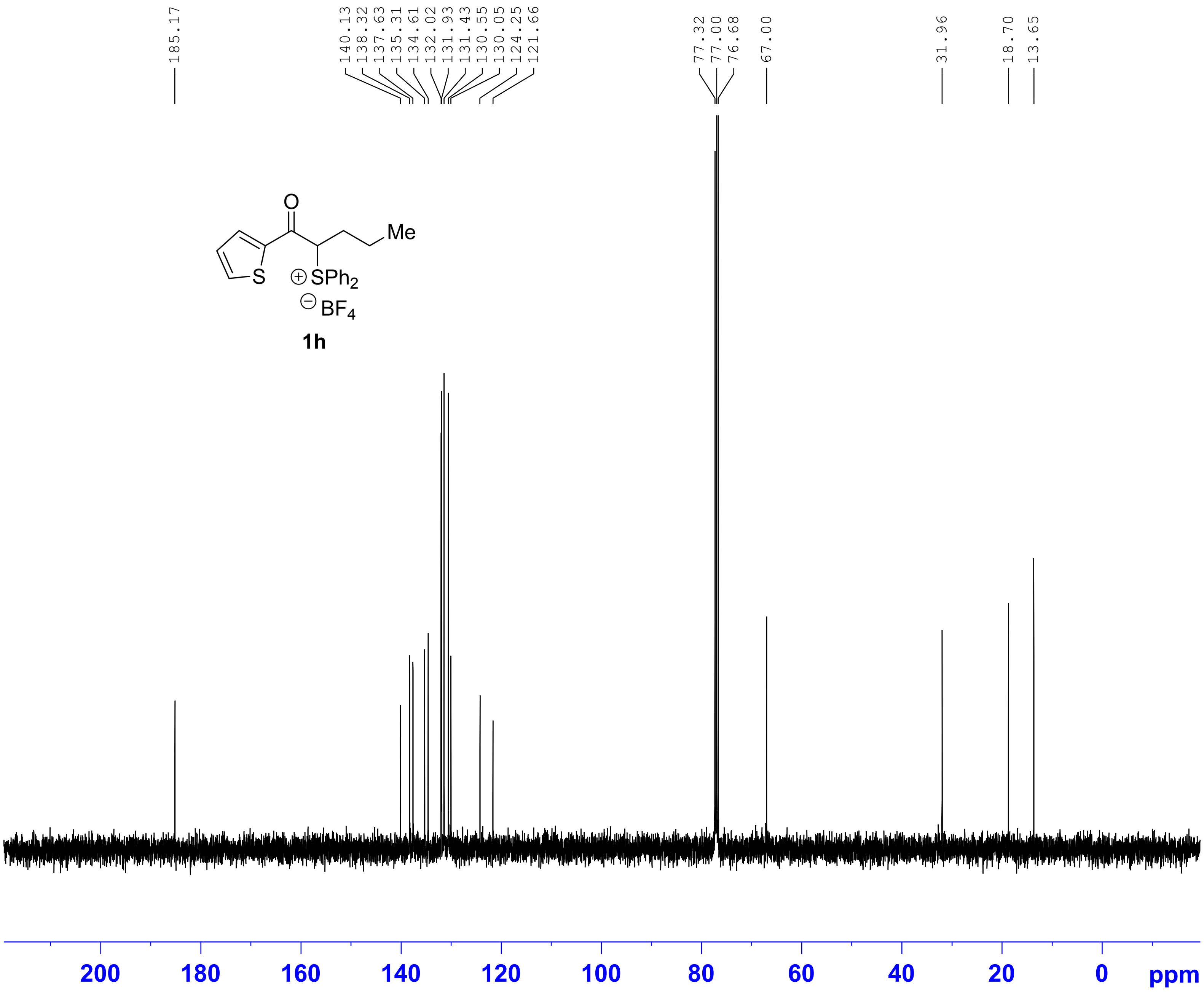

NAME	lzy-04-185-g-02-C	
EXPNO	1	
PROCNO	1	
Date_	20201030	
Time	15.05	
INSTRUM	spect	
PROBHD	5 mm PABBO BB/	
PULPROG	zgpg30	
TD	65536	
SOLVENT	CDCl3	
NS	32	
DS	0	
SWH	24038.461	Hz
FIDRES	0.366798	Hz
AQ	1.3631988	sec
RG	196.92	
DW	20.800	usec
DE	6.50	usec
TE	297.0	K
D1	2.00000000	sec
D11	0.03000000	sec
TD0	1	

======== CHANNEL f1 ========

SFO1	100.6228298	MHz
NUC1	13C	
P1	9.70	usec
SI	32768	
SF	100.6127810	MHz
WDW	EM	
SSB	0	
LB	1.00	Hz
GB	0	
PC	1.40	

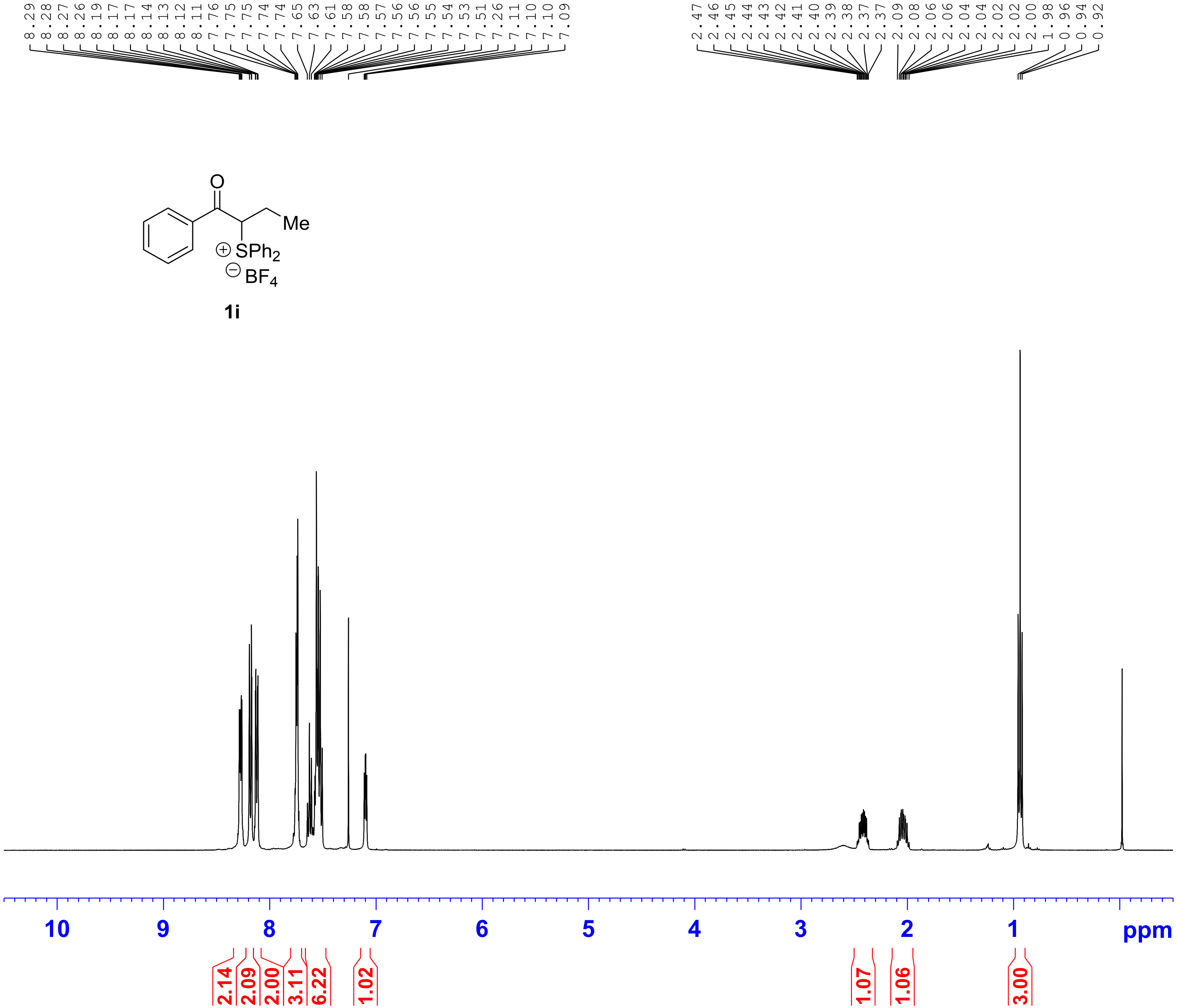

NAME	lzy-04-185-H
EXPNO	1
PROCNO	1
Date_	20201031
Time_	18.28
INSTRUM	spect
PROBHD	5 mm PABBO BB/
PULPROG	zg30
TD	65536
SOLVENT	CDCl3
NS	4
DS	0
SWH	8012.820 Hz
FIDRES	0.122266 Hz
AQ	4.0894966 sec
RG	45.67
DW	62.400 usec
DE	6.50 usec
TE	297.5 K
D1	1.00000000 sec
TD0	1

======== CHANNEL f1 ========

SFO1	400.1324710 MHz
NUC1	1H
P1	14.50 usec
SI	65536
SF	400.1300098 MHz
WDW	EM
SSB	0
LB	0.30 Hz
GB	0
PC	1.00

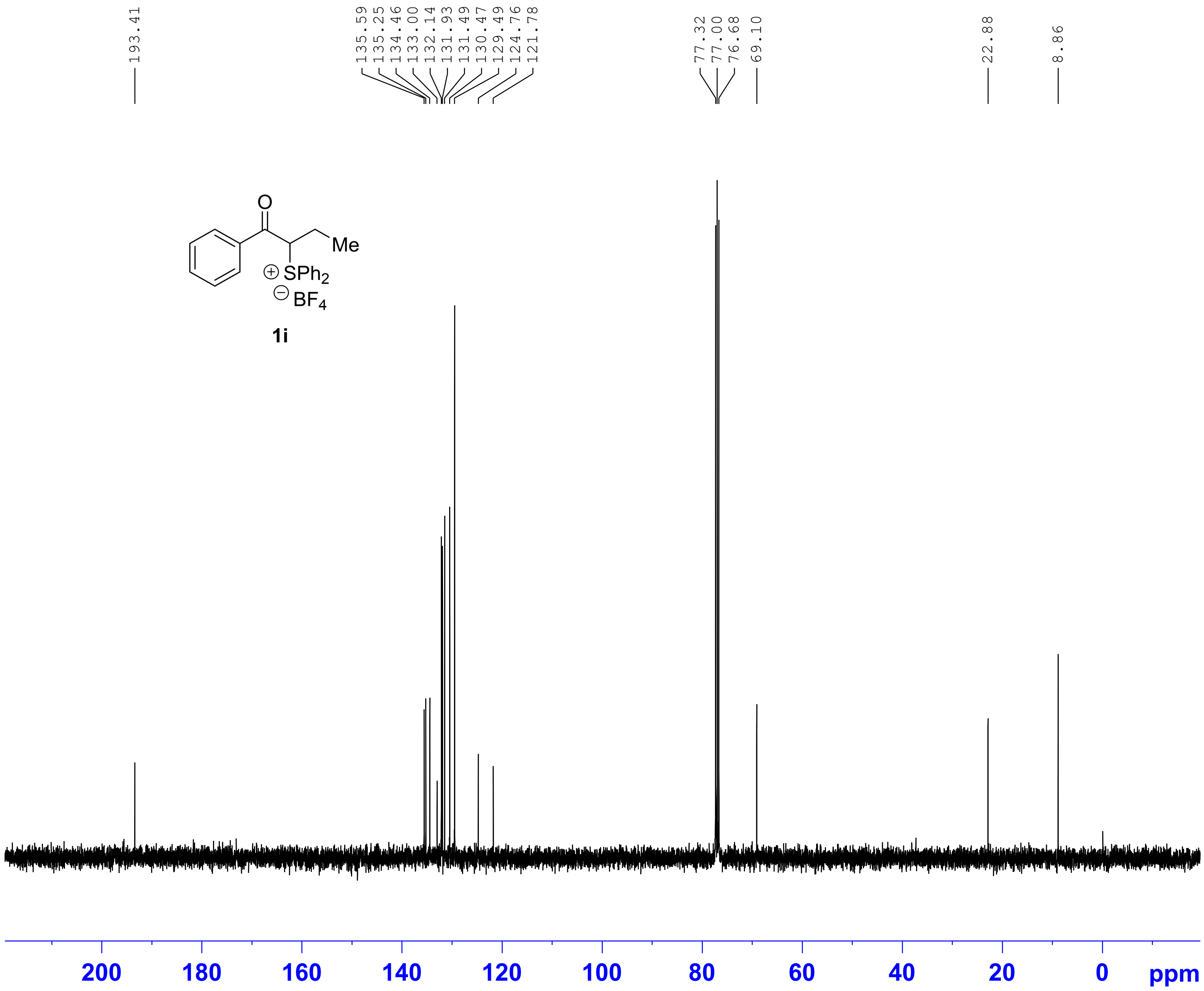

1i

NAME lzy-04-185-H-C
EXPNO 1
PROCNO 1
Date_ 20201031
Time 18.31
INSTRUM spect
PROBHD 5 mm PABBO BB/
PULPROG zgpg30
TD 65536
SOLVENT CDCl3
NS 44
DS 0
SWH 24038.461 Hz
FIDRES 0.366798 Hz
AQ 1.3631988 sec
RG 196.92
DW 20.800 usec
DE 6.50 usec
TE 298.3 K
D1 2.00000000 sec
D11 0.03000000 sec
TD0 1

======== CHANNEL f1 ========
SFO1 100.6228298 MHz
NUC1 13C
P1 9.70 usec
SI 32768
SF 100.6127773 MHz
WDW EM
SSB 0
LB 1.00 Hz
GB 0
PC 1.40

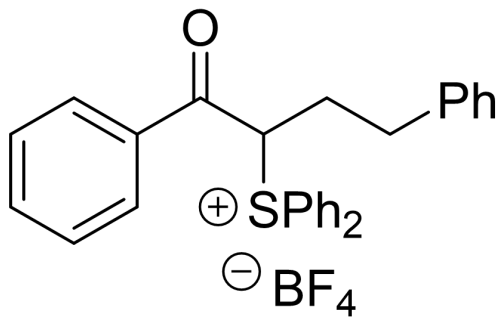

1j

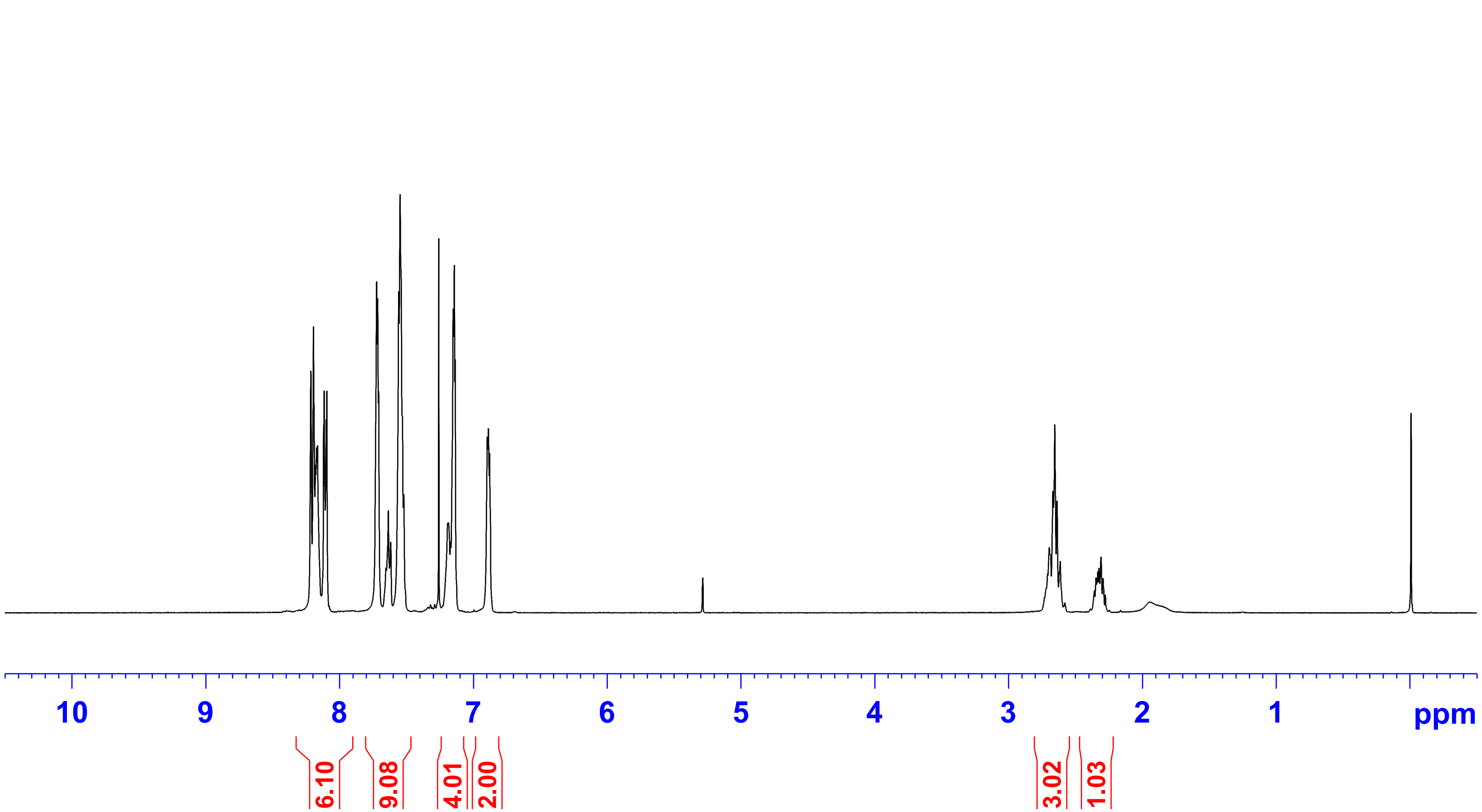

NAME	lzy-04-185-i
EXPNO	1
PROCNO	1
Date_	20201030
Time	10.33
INSTRUM	spect
PROBHD	5 mm PABBO BB/
PULPROG	zg30
TD	65536
SOLVENT	CDCl3
NS	4
DS	0
SWH	8012.820 Hz
FIDRES	0.122266 Hz
AQ	4.0894966 sec
RG	45.67
DW	62.400 usec
DE	6.50 usec
TE	297.2 K
D1	1.00000000 sec
TD0	1

======== CHANNEL f1 ========

SFO1	400.1324710 MHz
NUC1	1H
P1	14.50 usec
SI	65536
SF	400.1300099 MHz
WDW	EM
SSB	0
LB	0.30 Hz
GB	0
PC	1.00

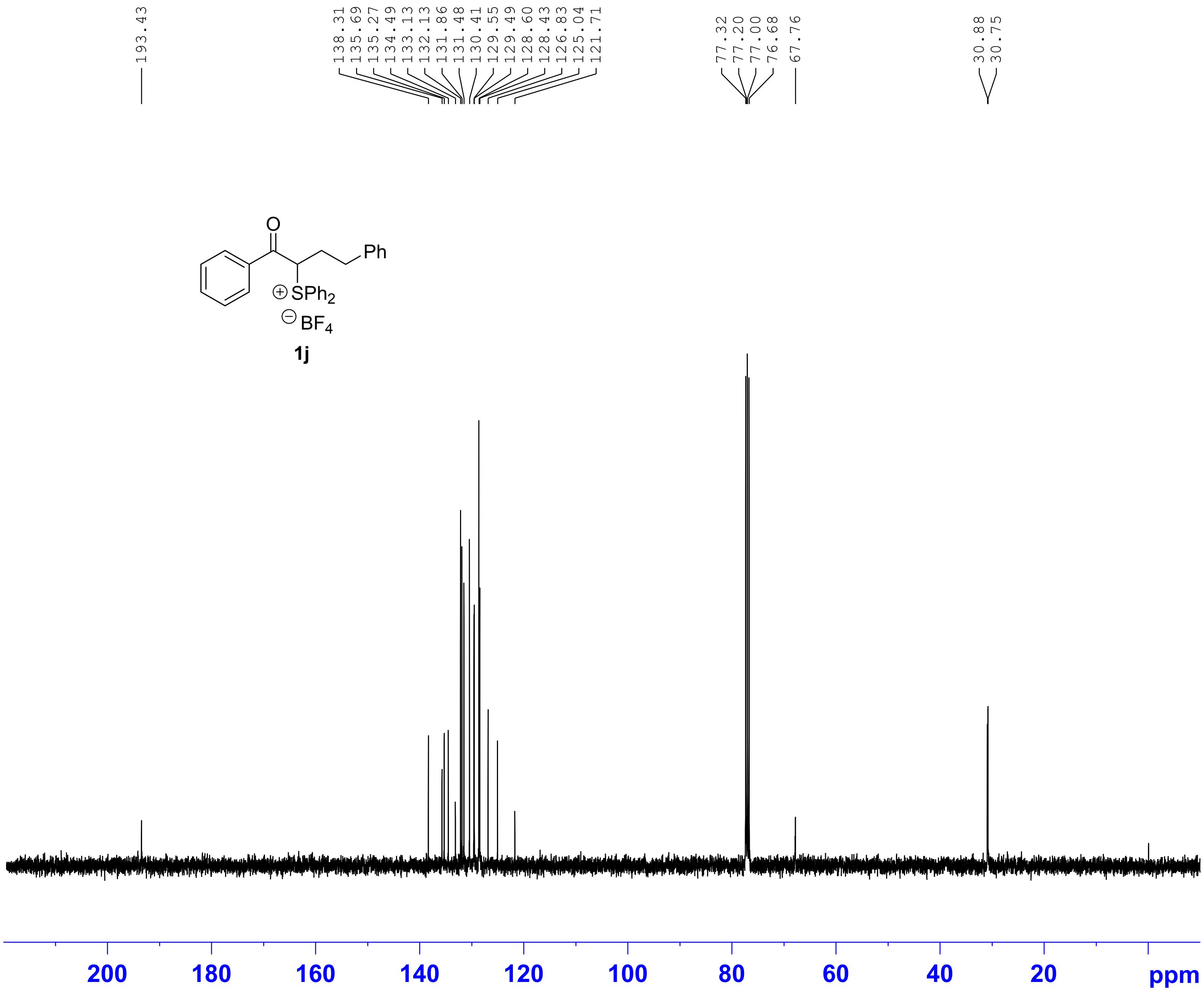

1j

NAME	lzy-04-185-i-C	
EXPNO	1	
PROCNO	1	
Date_	20201030	
Time	10.35	
INSTRUM	spect	
PROBHD	5 mm PABBO BB/	
PULPROG	zgpg30	
TD	65536	
SOLVENT	CDCl3	
NS	104	
DS	0	
SWH	24038.461	Hz
FIDRES	0.366798	Hz
AQ	1.3631988	sec
RG	196.92	
DW	20.800	usec
DE	6.50	usec
TE	297.7	K
D1	2.00000000	sec
D11	0.03000000	sec
TD0	1	

======== CHANNEL f1 ========

SFO1	100.6228298	MHz
NUC1	13C	
P1	9.70	usec
SI	32768	
SF	100.6127781	MHz
WDW	EM	
SSB	0	
LB	1.00	Hz
GB	0	
PC	1.40	

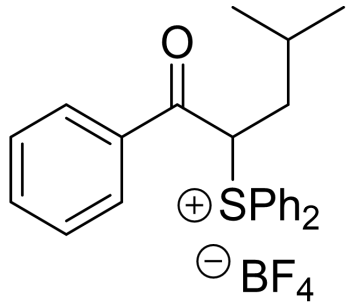

1k

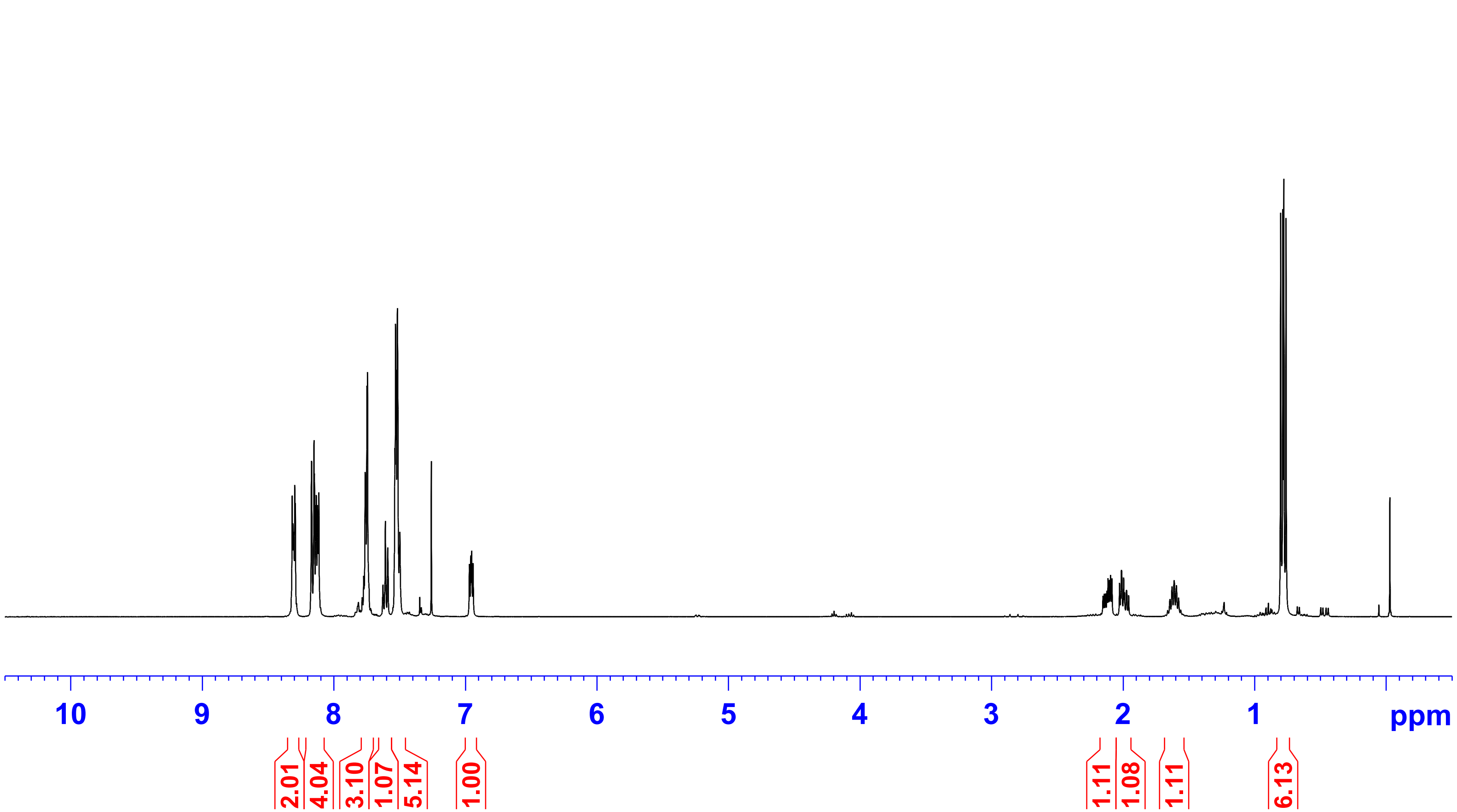

NAME	lzy-04-185-J-1103
EXPNO	1
PROCNO	1
Date_	20201103
Time	14.11
INSTRUM	spect
PROBHD	5 mm PABBO BB/
PULPROG	zg30
TD	65536
SOLVENT	CDCl3
NS	4
DS	0
SWH	8012.820 Hz
FIDRES	0.122266 Hz
AQ	4.0894966 sec
RG	34.77
DW	62.400 usec
DE	6.50 usec
TE	296.8 K
D1	1.00000000 sec
TD0	1

======== CHANNEL f1 ========

SFO1	400.1324710 MHz
NUC1	1H
P1	14.50 usec
SI	65536
SF	400.1300098 MHz
WDW	EM
SSB	0
LB	0.30 Hz
GB	0
PC	1.00

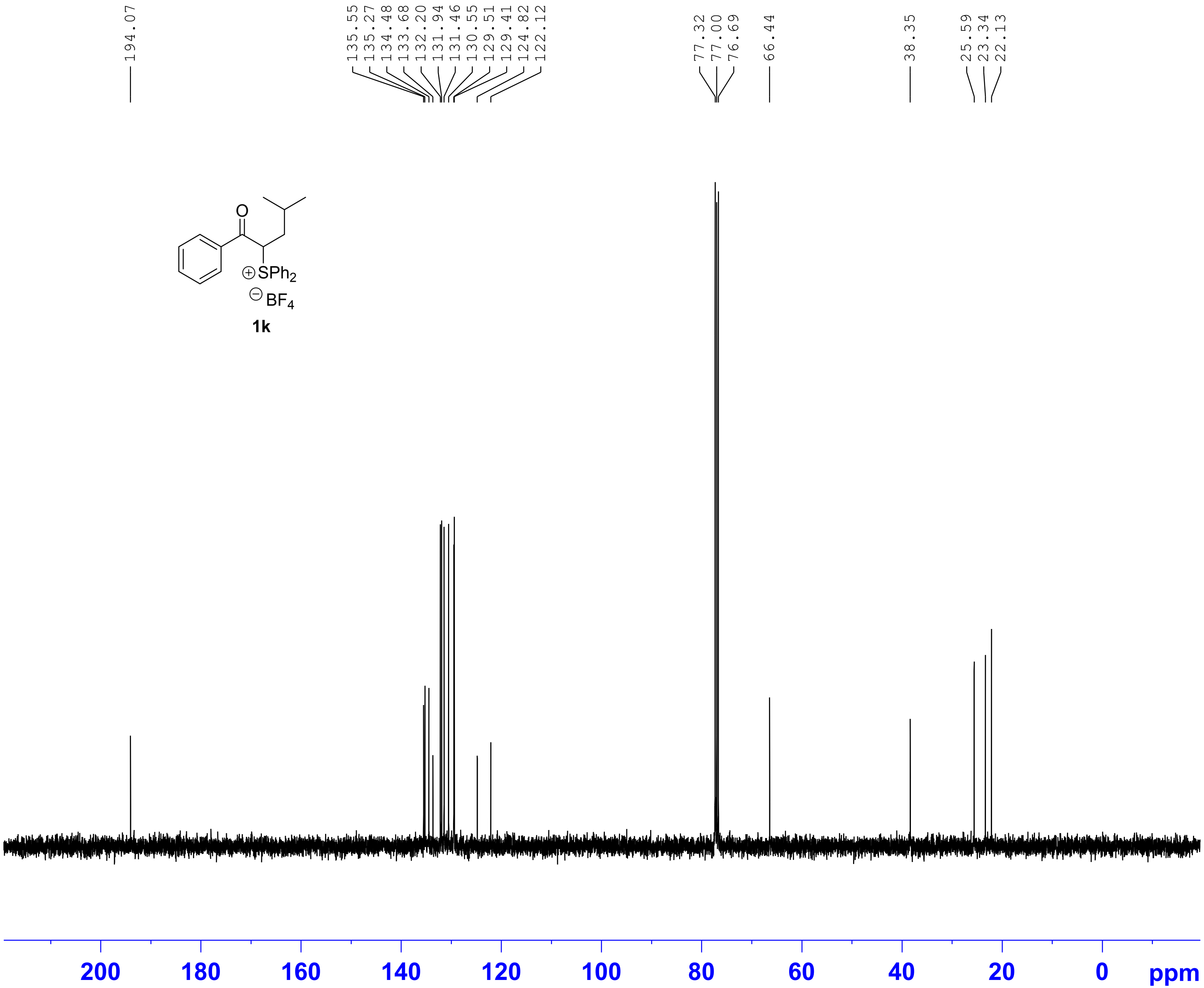

NAME	lzy-04-185-J-1103-C	
EXPNO	1	
PROCNO	1	
Date_	20201103	
Time	14.14	
INSTRUM	spect	
PROBHD	5 mm PABBO BB/	
PULPROG	zgpg30	
TD	65536	
SOLVENT	CDCl3	
NS	36	
DS	2	
SWH	24038.461	Hz
FIDRES	0.366798	Hz
AQ	1.3631988	sec
RG	196.92	
DW	20.800	usec
DE	6.50	usec
TE	297.6	K
D1	2.00000000	sec
D11	0.03000000	sec
TD0	1	

======== CHANNEL f1 ========

SFO1	100.6228298	MHz
NUC1	13C	
P1	9.70	usec
SI	32768	
SF	100.6127780	MHz
WDW	EM	
SSB	0	
LB	1.00	Hz
GB	0	
PC	1.40	

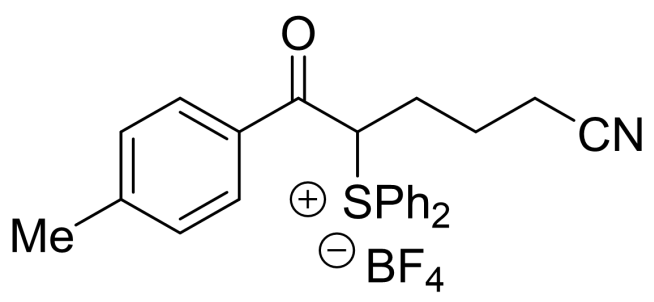

1l

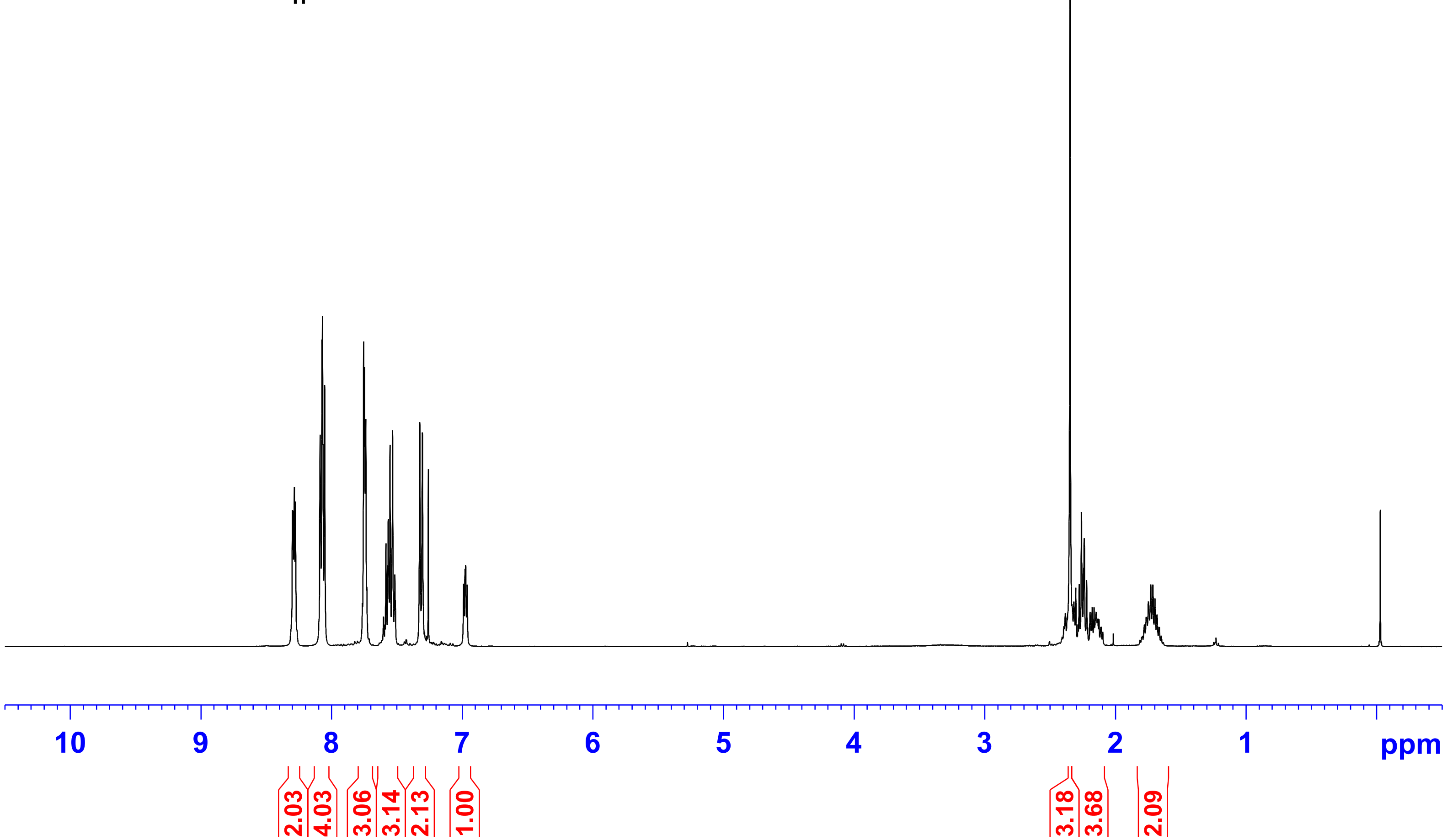

NAME	lzy-04-185-K-3
EXPNO	1
PROCNO	1
Date_	20201031
Time_	20.57
INSTRUM	spect
PROBHD	5 mm PABBO BB/
PULPROG	zg30
TD	65536
SOLVENT	CDCl3
NS	13
DS	2
SWH	8012.820 Hz
FIDRES	0.122266 Hz
AQ	4.0894966 sec
RG	27.78
DW	62.400 usec
DE	6.50 usec
TE	297.8 K
D1	1.00000000 sec
TD0	1

======== CHANNEL f1 ========

SFO1	400.1324710 MHz
NUC1	1H
P1	14.50 usec
SI	65536
SF	400.1300097 MHz
WDW	EM
SSB	0
LB	0.30 Hz
GB	0
PC	1.00

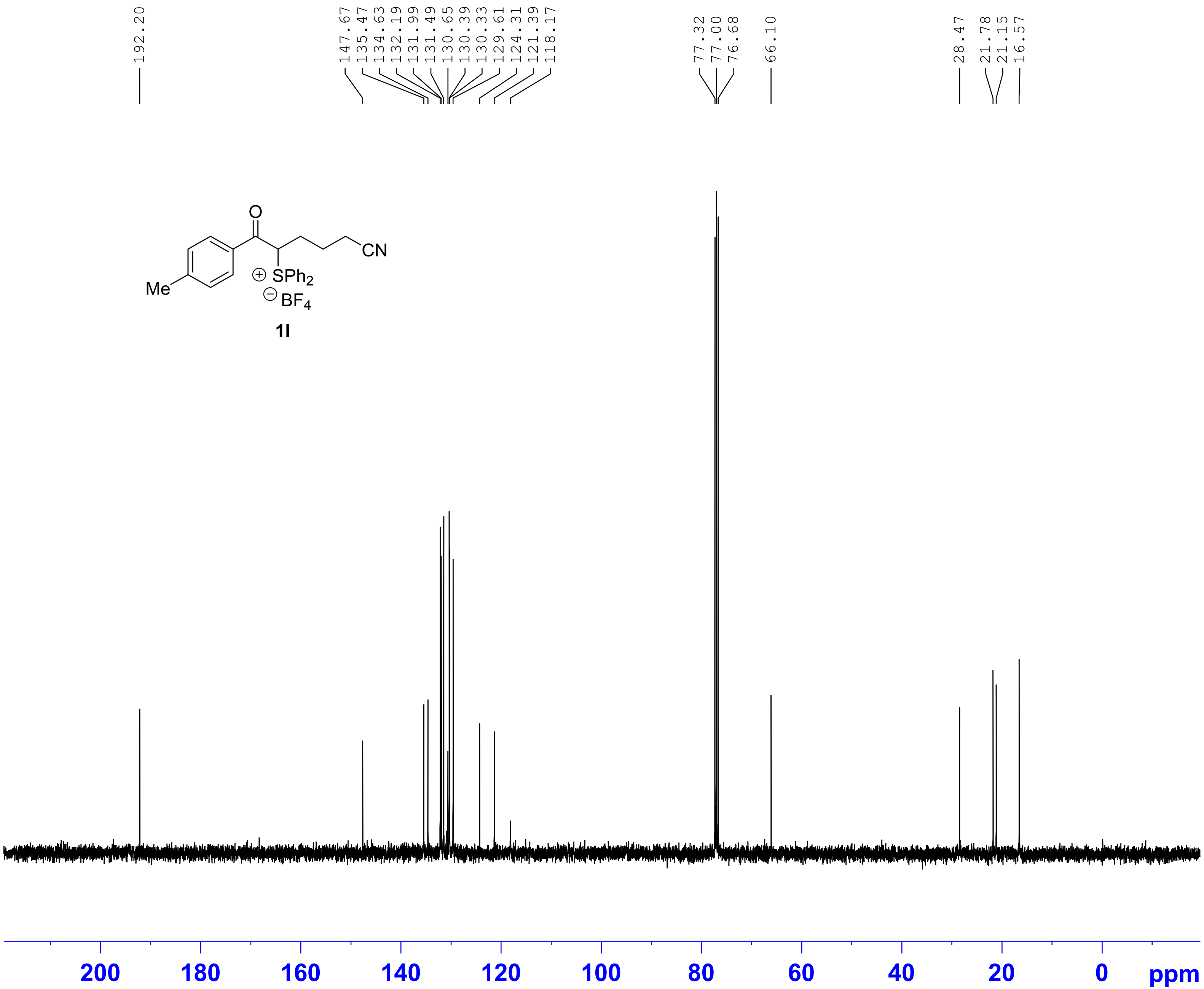

1l

NAME	lzy-04-185-K-4-C
EXPNO	1
PROCNO	1
Date_	20201031
Time	21.05
INSTRUM	spect
PROBHD	5 mm PABBO BB/
PULPROG	zgpg30
TD	65536
SOLVENT	CDCl3
NS	74
DS	0
SWH	24038.461 Hz
FIDRES	0.366798 Hz
AQ	1.3631988 sec
RG	196.92
DW	20.800 usec
DE	6.50 usec
TE	298.7 K
D1	2.00000000 sec
D11	0.03000000 sec
TD0	1

======== CHANNEL f1 ========	
SFO1	100.6228298 MHz
NUC1	13C
P1	9.70 usec
SI	32768
SF	100.6127824 MHz
WDW	EM
SSB	0
LB	1.00 Hz
GB	0
PC	1.40

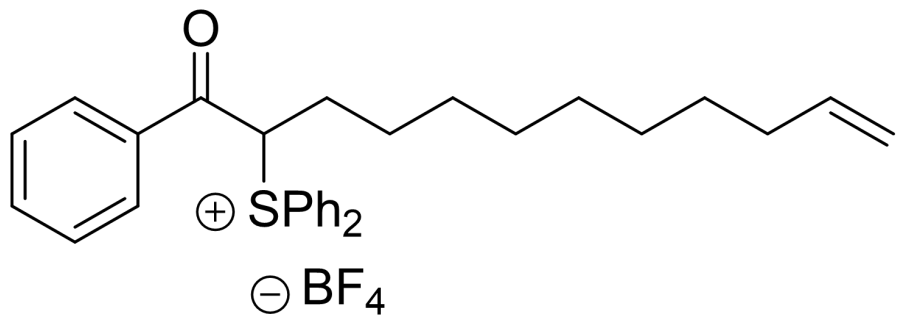

1m

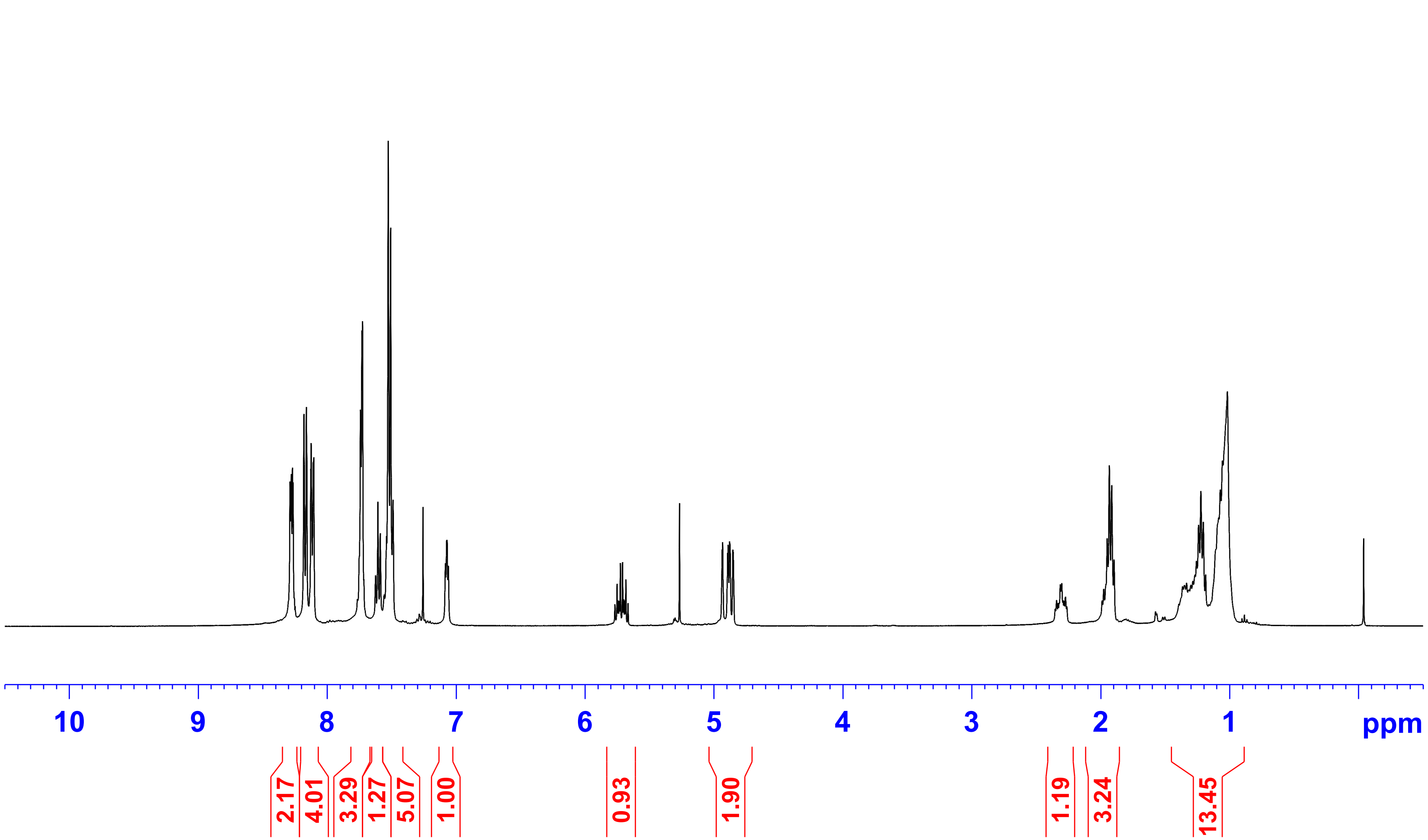

NAME	lzy-05-159-J-1	
EXPNO	1	
PROCNO	1	
Date_	20210210	
Time	10.58	
INSTRUM	spect	
PROBHD	5 mm PABBO BB/	
PULPROG	zg30	
TD	65536	
SOLVENT	CDCl3	
NS	4	
DS	0	
SWH	8012.820	Hz
FIDRES	0.122266	Hz
AQ	4.0894966	sec
RG	25.32	
DW	62.400	usec
DE	6.50	usec
TE	296.0	K
D1	1.00000000	sec
TD0	1	

======== CHANNEL f1 ========

SFO1	400.1324710	MHz
NUC1	1H	
P1	14.50	usec
SI	65536	
SF	400.1300103	MHz
WDW	EM	
SSB	0	
LB	0.30	Hz
GB	0	
PC	1.00	

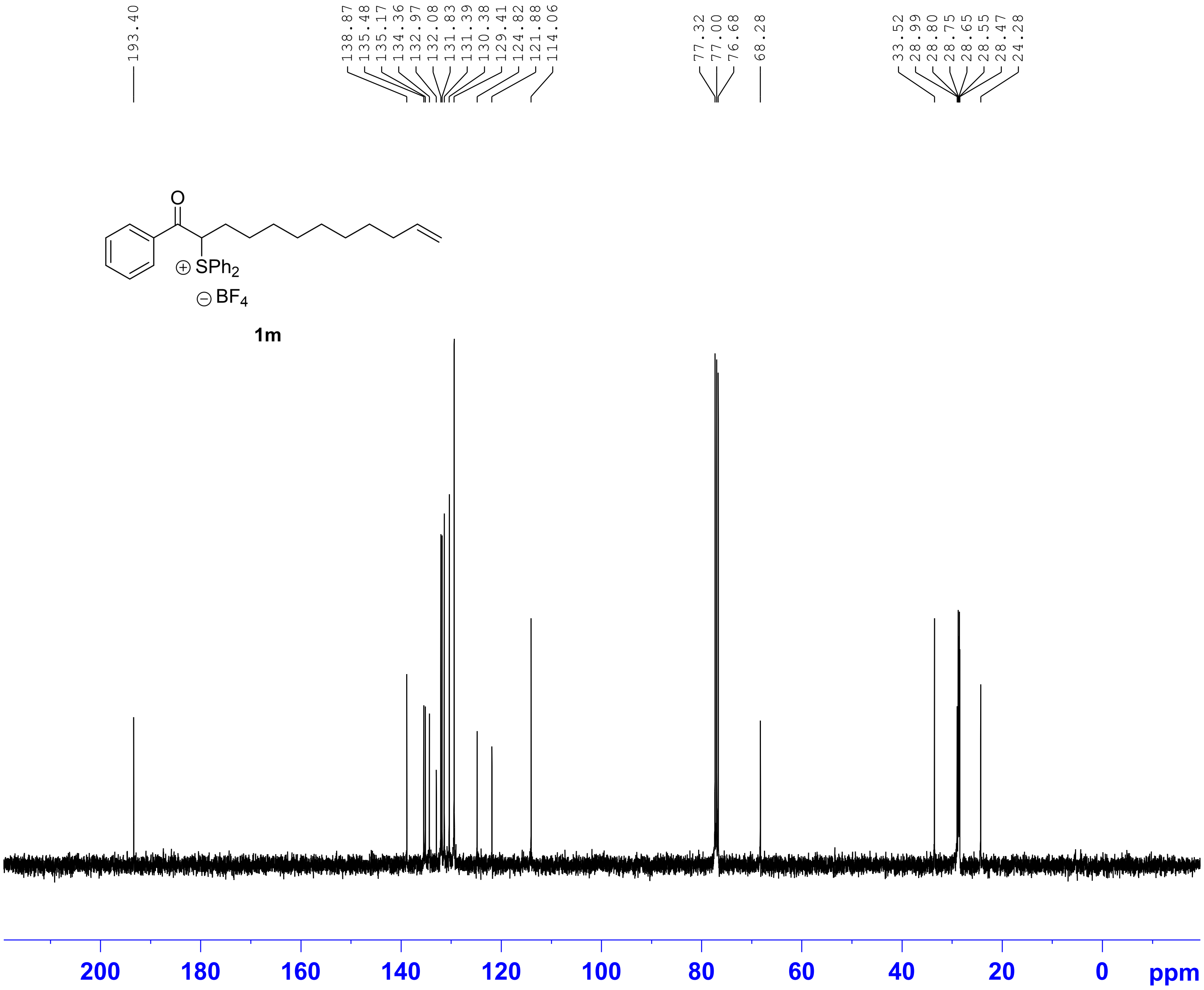

NAME	lzy-05-159-J-1-C
EXPNO	1
PROCNO	1
Date_	20210210
Time	10.59
INSTRUM	spect
PROBHD	5 mm PABBO BB/
PULPROG	zgpg30
TD	65536
SOLVENT	CDCl3
NS	63
DS	0
SWH	24038.461 Hz
FIDRES	0.366798 Hz
AQ	1.3631988 sec
RG	196.92
DW	20.800 usec
DE	6.50 usec
TE	296.4 K
D1	2.00000000 sec
D11	0.03000000 sec
TD0	1

======== CHANNEL f1 ========

SFO1	100.6228298 MHz
NUC1	13C
P1	9.70 usec
SI	32768
SF	100.6127831 MHz
WDW	EM
SSB	0
LB	1.00 Hz
GB	0
PC	1.40

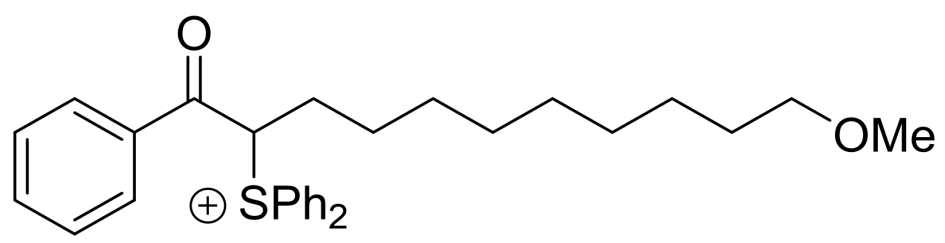

1n

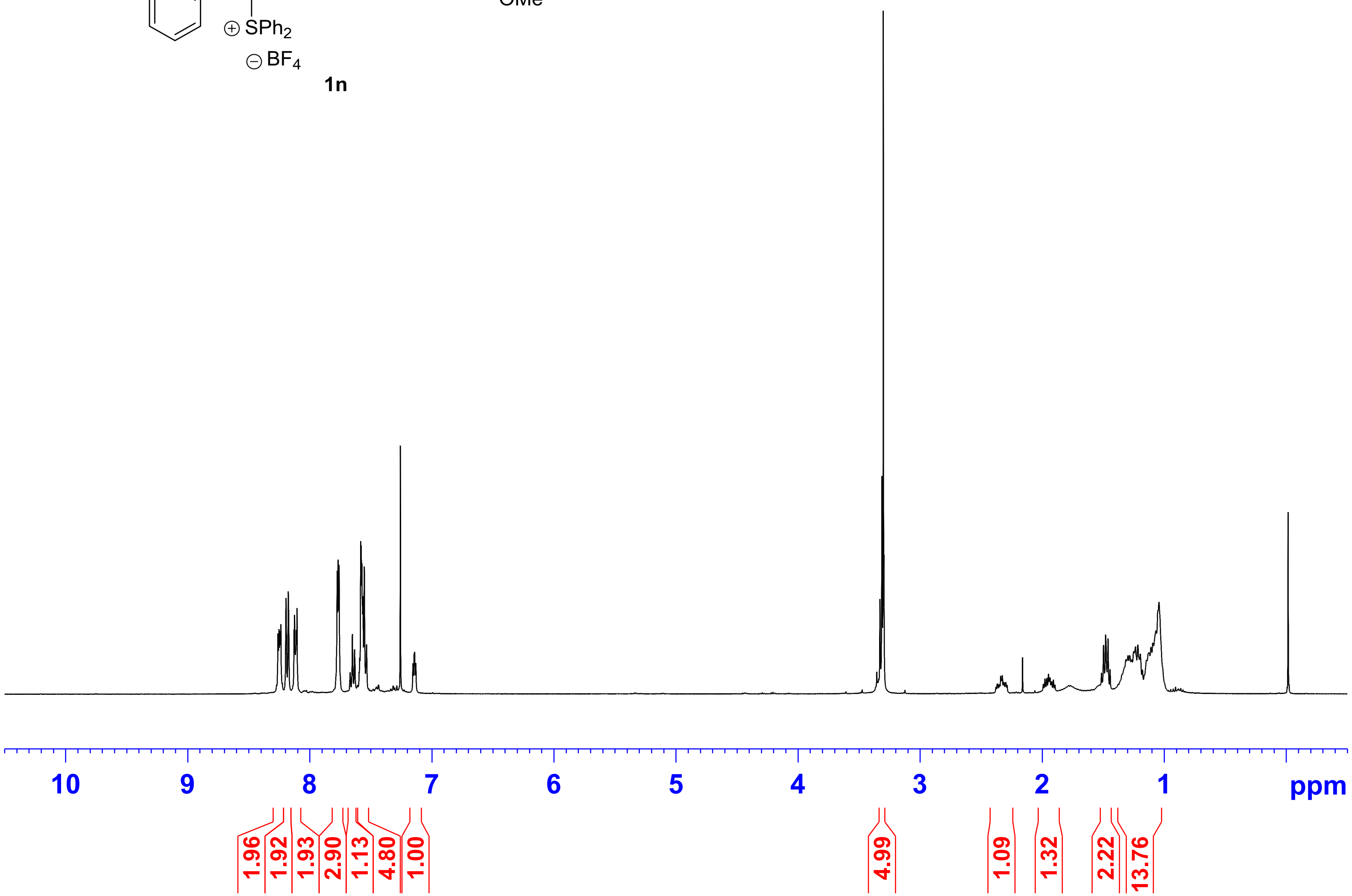


```
NAME          lzy-05-169-sm
EXPNO                     1
PROCNO                    1
Date_              20210210
Time                  11.18
INSTRUM               spect
PROBHD     5 mm PABBO BB/
PULPROG                zg30
TD                    65536
SOLVENT               CDCl3
NS                        4
DS                        0
SWH              8012.820 Hz
FIDRES           0.122266 Hz
AQ              4.0894966 sec
RG                    82.92
DW                   62.400 usec
DE                     6.50 usec
TE                    296.1 K
D1               1.00000000 sec
TD0                       1

======== CHANNEL f1 ========
SFO1          400.1324710 MHz
NUC1                     1H
P1                    14.50 usec
SI                    65536
SF            400.1300102 MHz
WDW                      EM
SSB                       0
LB                     0.30 Hz
GB                        0
PC                     1.00
```

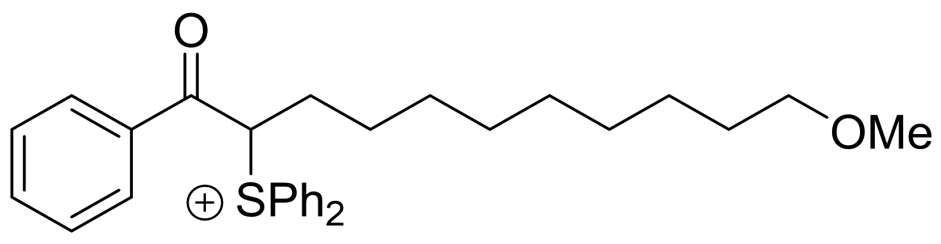

1n

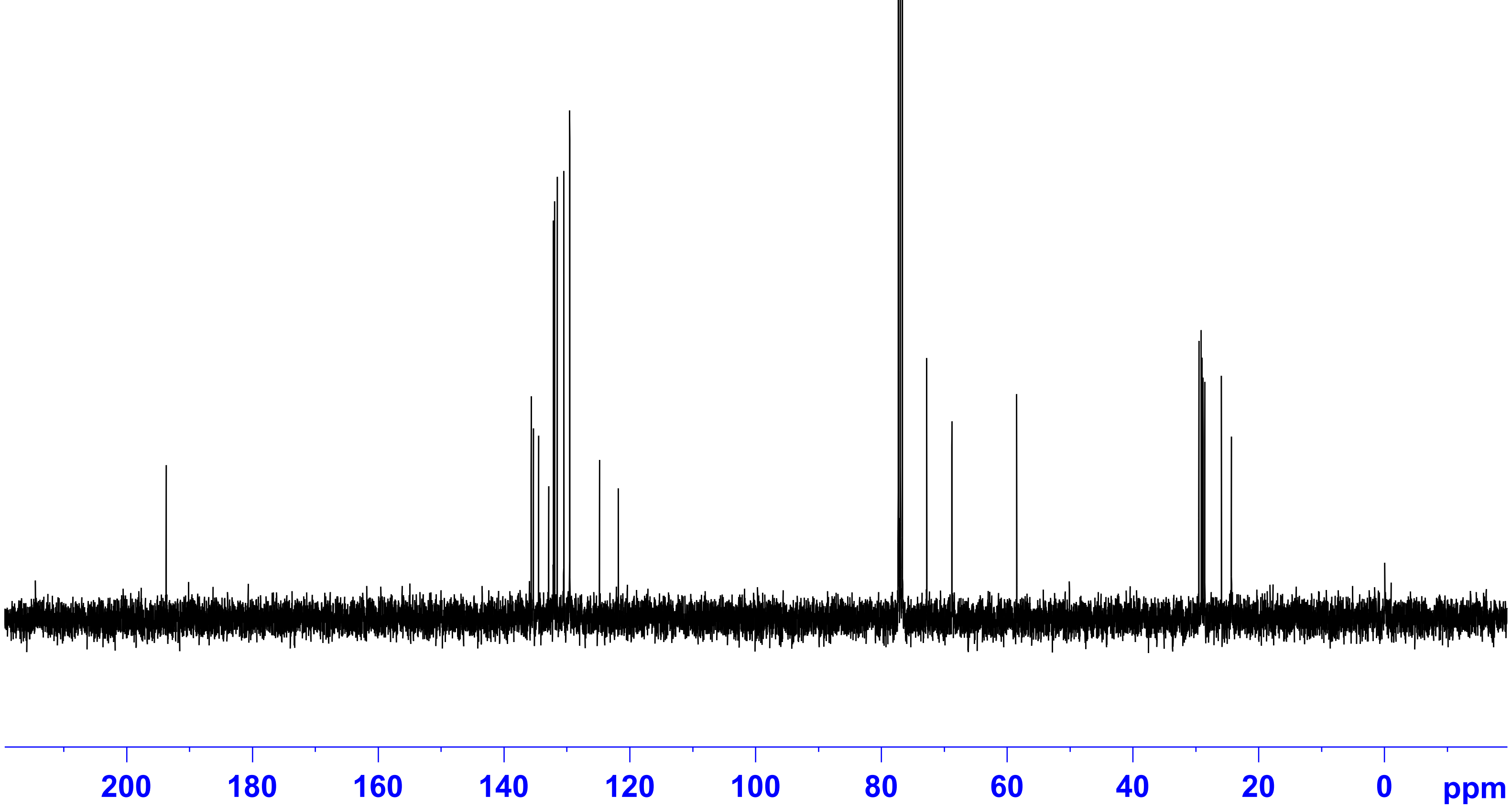

NAME	lzy-05-169-C	
EXPNO	1	
PROCNO	1	
Date_	20210202	
Time	18.45	
INSTRUM	spect	
PROBHD	5 mm DUL 13C-1	
PULPROG	zgpg30	
TD	65536	
SOLVENT	CDCl3	
NS	223	
DS	1	
SWH	24038.461	Hz
FIDRES	0.366798	Hz
AQ	1.3631988	sec
RG	2050	
DW	20.800	usec
DE	6.00	usec
TE	293.9	K
D1	2.00000000	sec
D11	0.03000000	sec
TD0	1	

======== CHANNEL f1 ========

NUC1	13C	
P1	40.00	usec
PL1	-3.00	dB
PL1W	60.64365387	W
SFO1	100.6228298	MHz

======== CHANNEL f2 ========

CPDPRG2	waltz16	
NUC2	1H	
PCPD2	80.00	usec
PL2	-1.00	dB
PL12	14.39	dB
PL13	18.00	dB
PL2W	12.17476940	W
PL12W	0.35193357	W
PL13W	0.15327126	W
SFO2	400.1316005	MHz
SI	32768	
SF	100.6127743	MHz
WDW	EM	
SSB	0	
LB	1.00	Hz
GB	0	
PC	1.40	

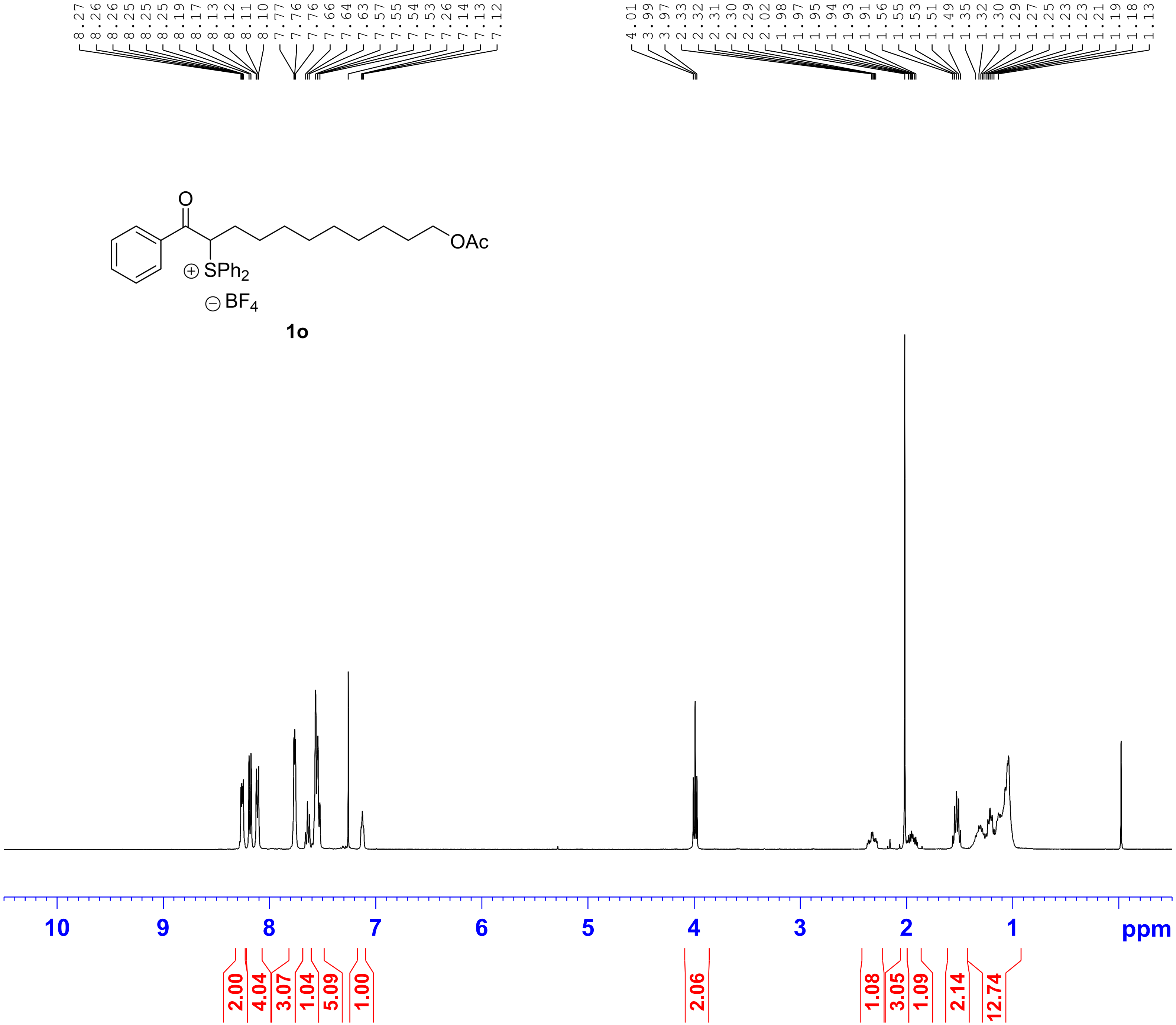

NAME	lzy-05-159-K
EXPNO	1
PROCNO	1
Date_	20210210
Time_	10.46
INSTRUM	spect
PROBHD	5 mm PABBO BB/
PULPROG	zg30
TD	65536
SOLVENT	CDCl3
NS	4
DS	0
SWH	8012.820 Hz
FIDRES	0.122266 Hz
AQ	4.0894966 sec
RG	70.97
DW	62.400 usec
DE	6.50 usec
TE	295.9 K
D1	1.00000000 sec
TD0	1

======== CHANNEL f1 ========

SFO1	400.1324710 MHz
NUC1	1H
P1	14.50 usec
SI	65536
SF	400.1300103 MHz
WDW	EM
SSB	0
LB	0.30 Hz
GB	0
PC	1.00

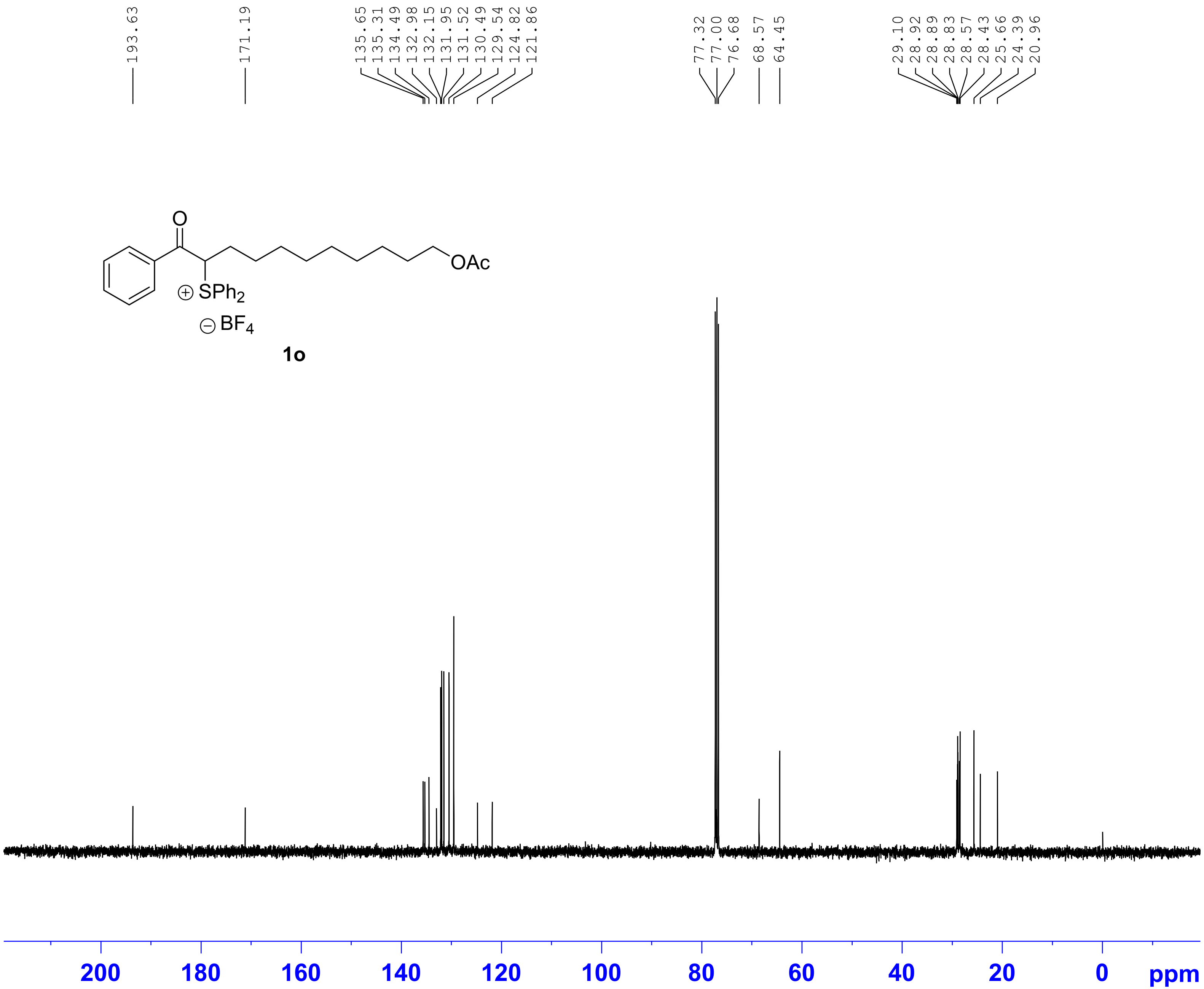

NAME	lzy-05-159-K-C	
EXPNO	1	
PROCNO	1	
Date_	20210210	
Time_	10.48	
INSTRUM	spect	
PROBHD	5 mm PABBO BB/	
PULPROG	zgpg30	
TD	65536	
SOLVENT	CDCl3	
NS	138	
DS	0	
SWH	24038.461	Hz
FIDRES	0.366798	Hz
AQ	1.3631988	sec
RG	196.92	
DW	20.800	usec
DE	6.50	usec
TE	296.4	K
D1	2.00000000	sec
D11	0.03000000	sec
TD0	1	

======== CHANNEL f1 ========

SFO1	100.6228298	MHz
NUC1	13C	
P1	9.70	usec
SI	32768	
SF	100.6127757	MHz
WDW	EM	
SSB	0	
LB	1.00	Hz
GB	0	
PC	1.40	

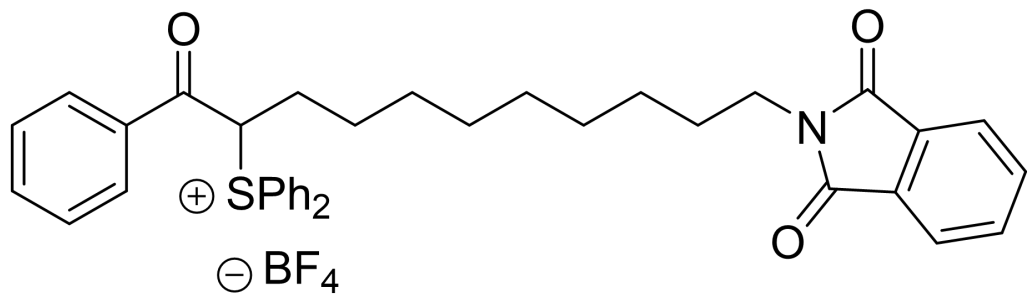

1p

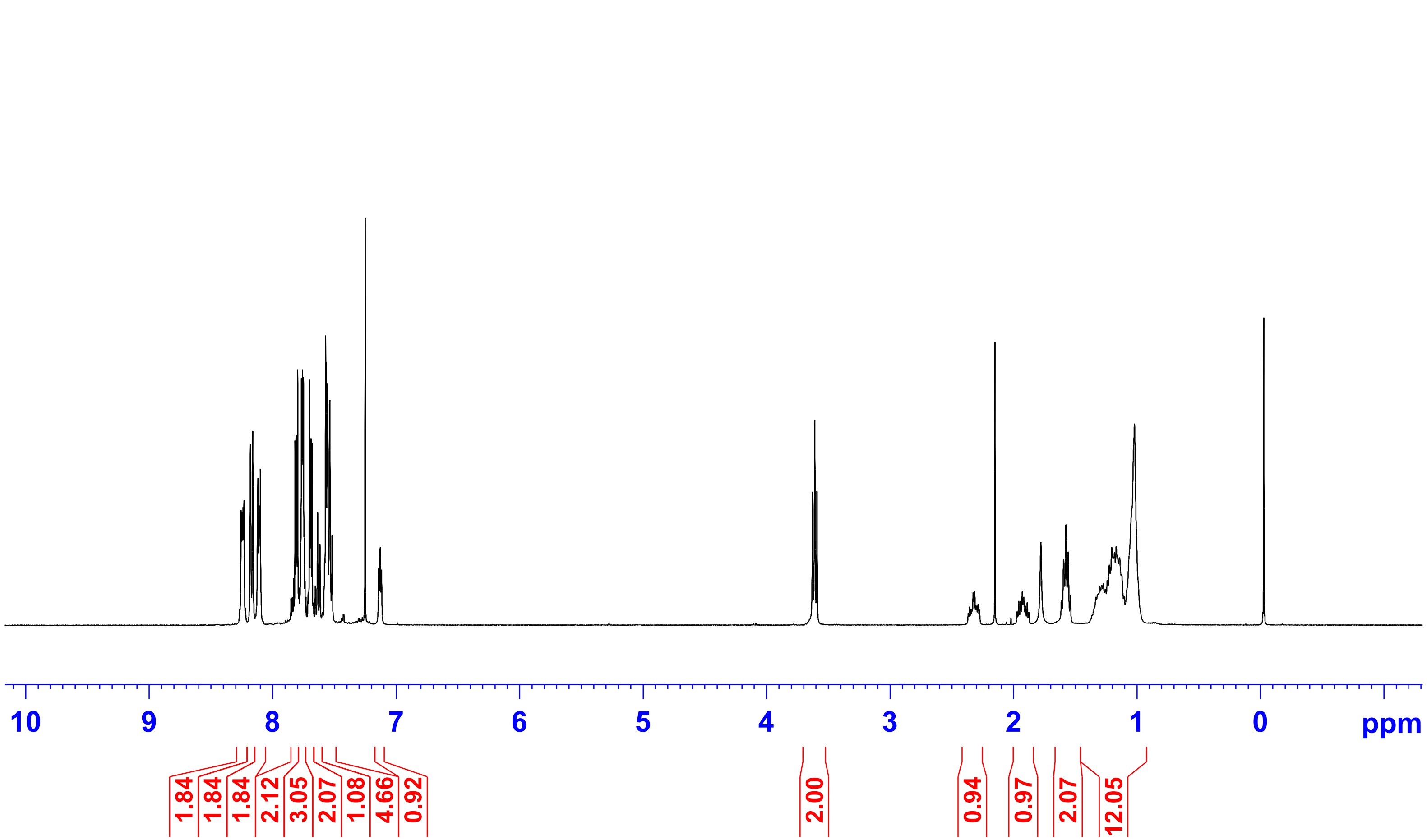

NAME	lzy-05-159-G
EXPNO	1
PROCNO	1
Date_	20210210
Time	11.05
INSTRUM	spect
PROBHD	5 mm PABBO BB/
PULPROG	zg30
TD	65536
SOLVENT	CDCl3
NS	4
DS	0
SWH	8012.820 Hz
FIDRES	0.122266 Hz
AQ	4.0894966 sec
RG	82.92
DW	62.400 usec
DE	6.50 usec
TE	296.0 K
D1	1.00000000 sec
TD0	1

======== CHANNEL f1 ========

SFO1	400.1324710 MHz
NUC1	1H
P1	14.50 usec
SI	65536
SF	400.1300129 MHz
WDW	EM
SSB	0
LB	0.30 Hz
GB	0
PC	1.00

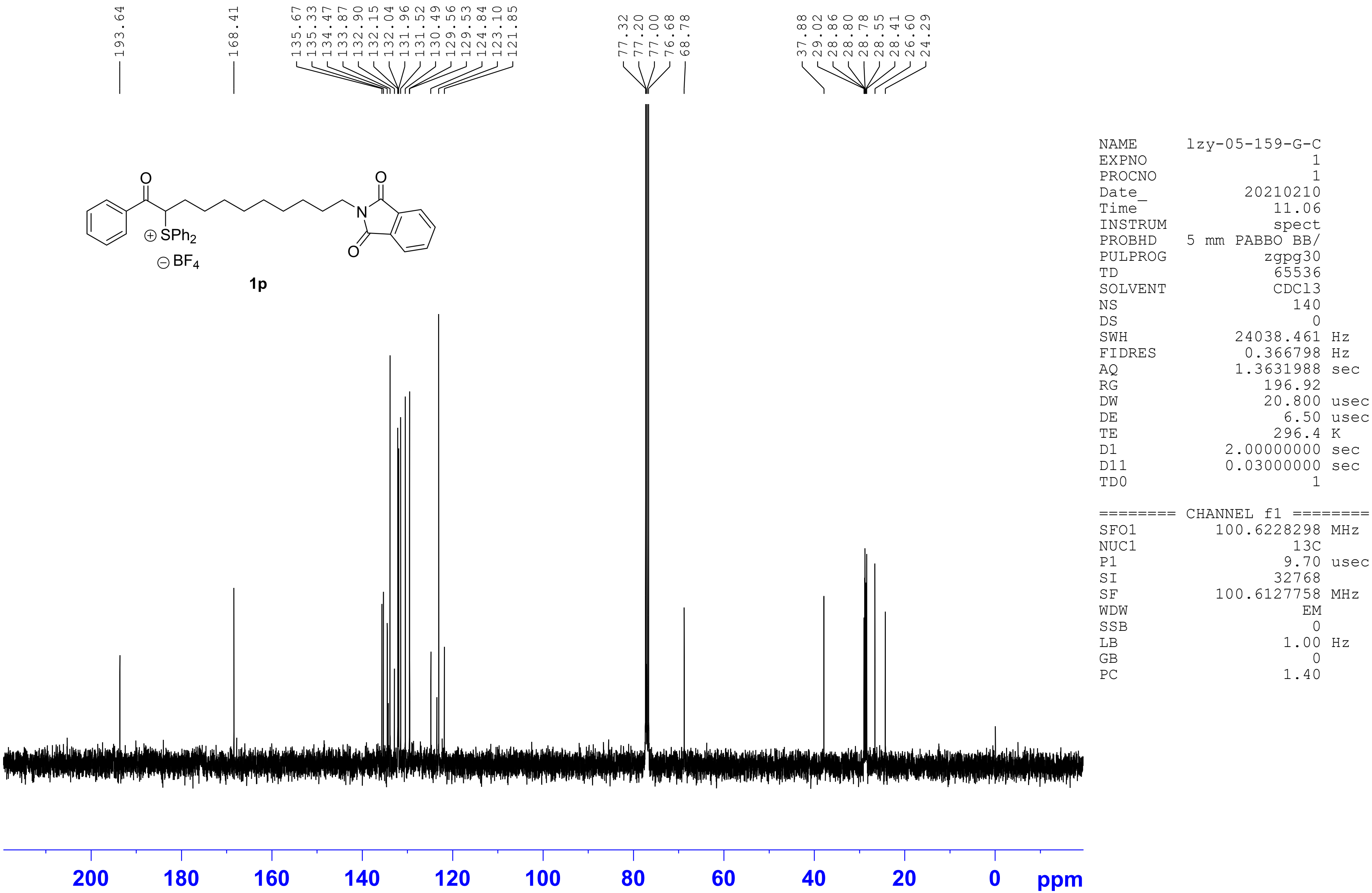

193.64
168.41
135.67
135.33
134.47
133.87
132.90
132.15
132.04
131.96
131.52
130.49
129.56
129.53
124.84
123.10
121.85
77.32
77.20
77.00
76.68
68.78
37.88
29.02
28.86
28.80
28.78
28.55
28.41
26.60
24.29
O
O
N
O
⊕ SPh2
⊖ BF4
1p
NAME lzy-05-159-G-C
EXPNO 1
PROCNO 1
Date_ 20210210
Time 11.06
INSTRUM spect
PROBHD 5 mm PABBO BB/
PULPROG zgpg30
TD 65536
SOLVENT CDCl3
NS 140
DS 0
SWH 24038.461 Hz
FIDRES 0.366798 Hz
AQ 1.3631988 sec
RG 196.92
DW 20.800 usec
DE 6.50 usec
TE 296.4 K
D1 2.00000000 sec
D11 0.03000000 sec
TD0 1
======== CHANNEL f1 ========
SFO1 100.6228298 MHz
NUC1 13C
P1 9.70 usec
SI 32768
SF 100.6127758 MHz
WDW EM
SSB 0
LB 1.00 Hz
GB 0
PC 1.40
200
180
160
140
120
100
80
60
40
20
0
ppm

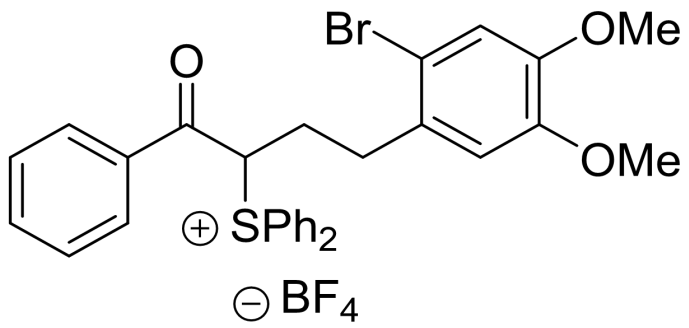

1q

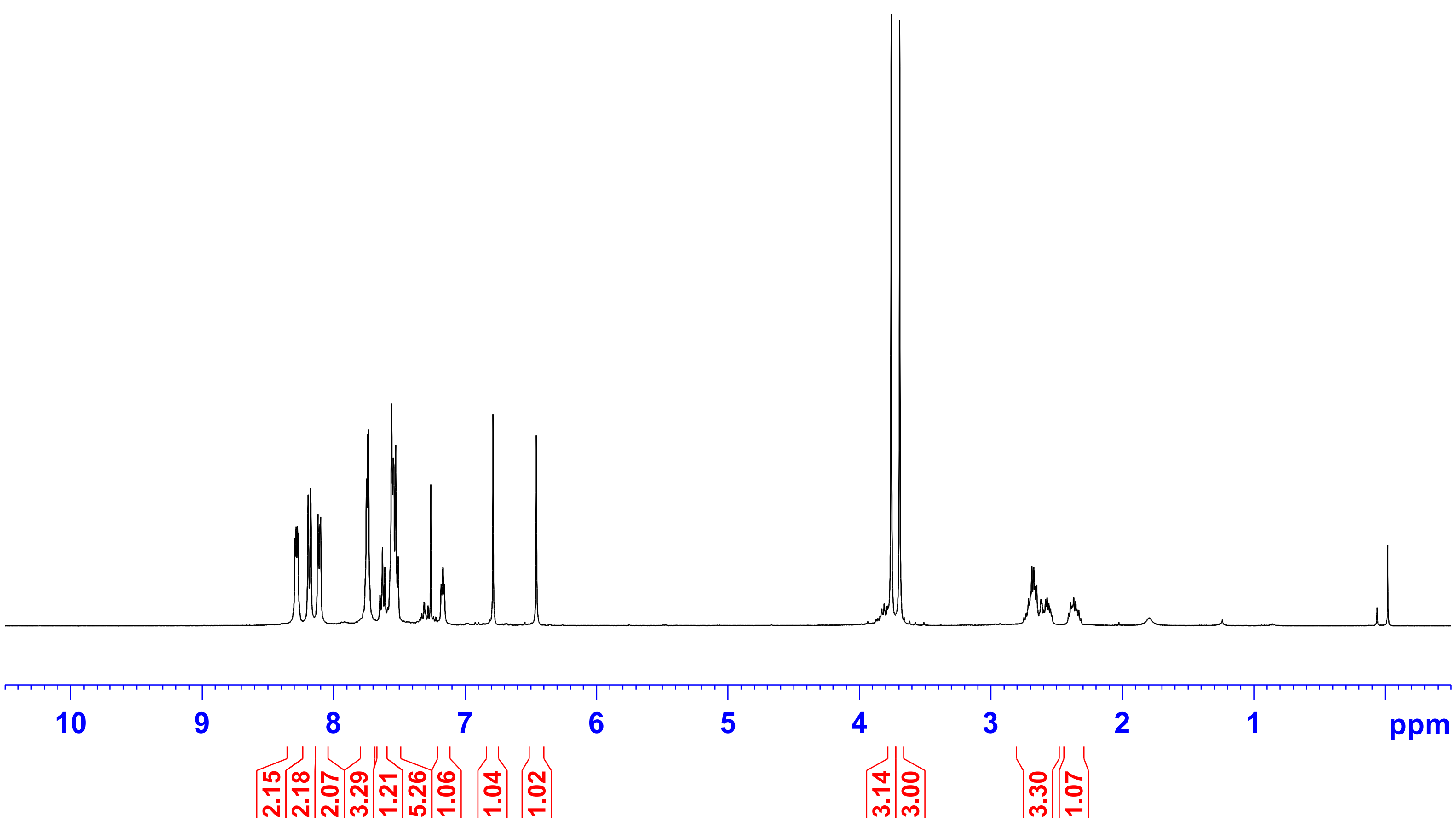

NAME	lzy-05-159-C-2
EXPNO	1
PROCNO	1
Date_	20210312
Time	16.02
INSTRUM	spect
PROBHD	5 mm DUL 13C-1
PULPROG	zg30
TD	65536
SOLVENT	CDCl3
NS	4
DS	0
SWH	8223.685 Hz
FIDRES	0.125483 Hz
AQ	3.9846387 sec
RG	228
DW	60.800 usec
DE	6.00 usec
TE	293.6 K
D1	1.00000000 sec
TD0	1

======== CHANNEL f1 ========

NUC1	1H
P1	15.80 usec
PL1	-1.00 dB
PL1W	12.17476940 W
SFO1	400.1324710 MHz
SI	32768
SF	400.1300096 MHz
WDW	EM
SSB	0
LB	0.30 Hz
GB	0
PC	1.00

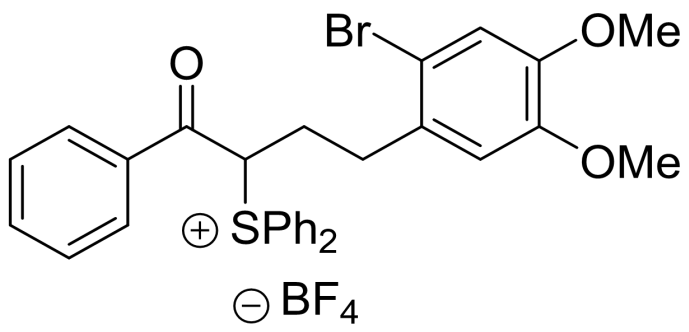

1q

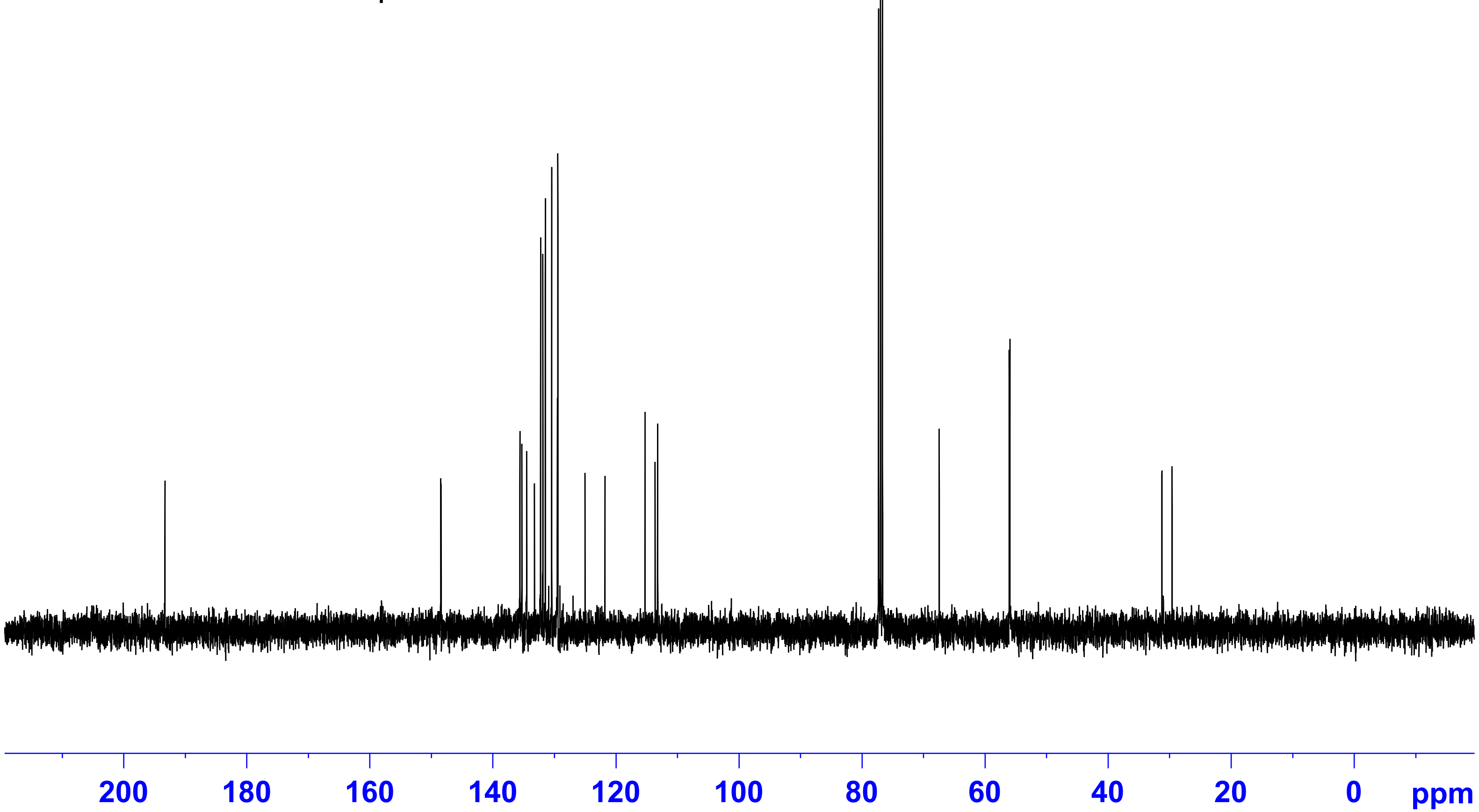


```
NAME          lzy-05-159-C-2-C
EXPNO                        3
PROCNO                       1
Date_                 20210312
Time                     16.27
INSTRUM                  spect
PROBHD        5 mm DUL 13C-1
PULPROG                zgpg30
TD                       65536
SOLVENT                  CDCl3
NS                         150
DS                           0
SWH                  24038.461 Hz
FIDRES                0.366798 Hz
AQ                   1.3631988 sec
RG                        2050
DW                      20.800 usec
DE                        6.00 usec
TE                       294.4 K
D1                  2.00000000 sec
D11                 0.03000000 sec
TD0                          1

======== CHANNEL f1 ========
NUC1                       13C
P1                       40.00 usec
PL1                      -3.00 dB
PL1W               60.64365387 W
SFO1               100.6228298 MHz

======== CHANNEL f2 ========
CPDPRG2                waltz16
NUC2                        1H
PCPD2                    80.00 usec
PL2                      -1.00 dB
PL12                     14.39 dB
PL13                     18.00 dB
PL2W               12.17476940 W
PL12W               0.35193357 W
PL13W               0.15327126 W
SFO2               400.1316005 MHz
SI                       32768
SF                 100.6127780 MHz
WDW                         EM
SSB                          0
LB                        1.00 Hz
GB                           0
PC                        1.40
```

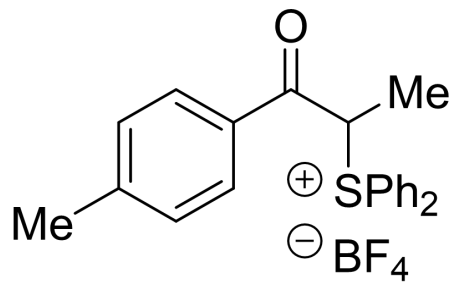

1r

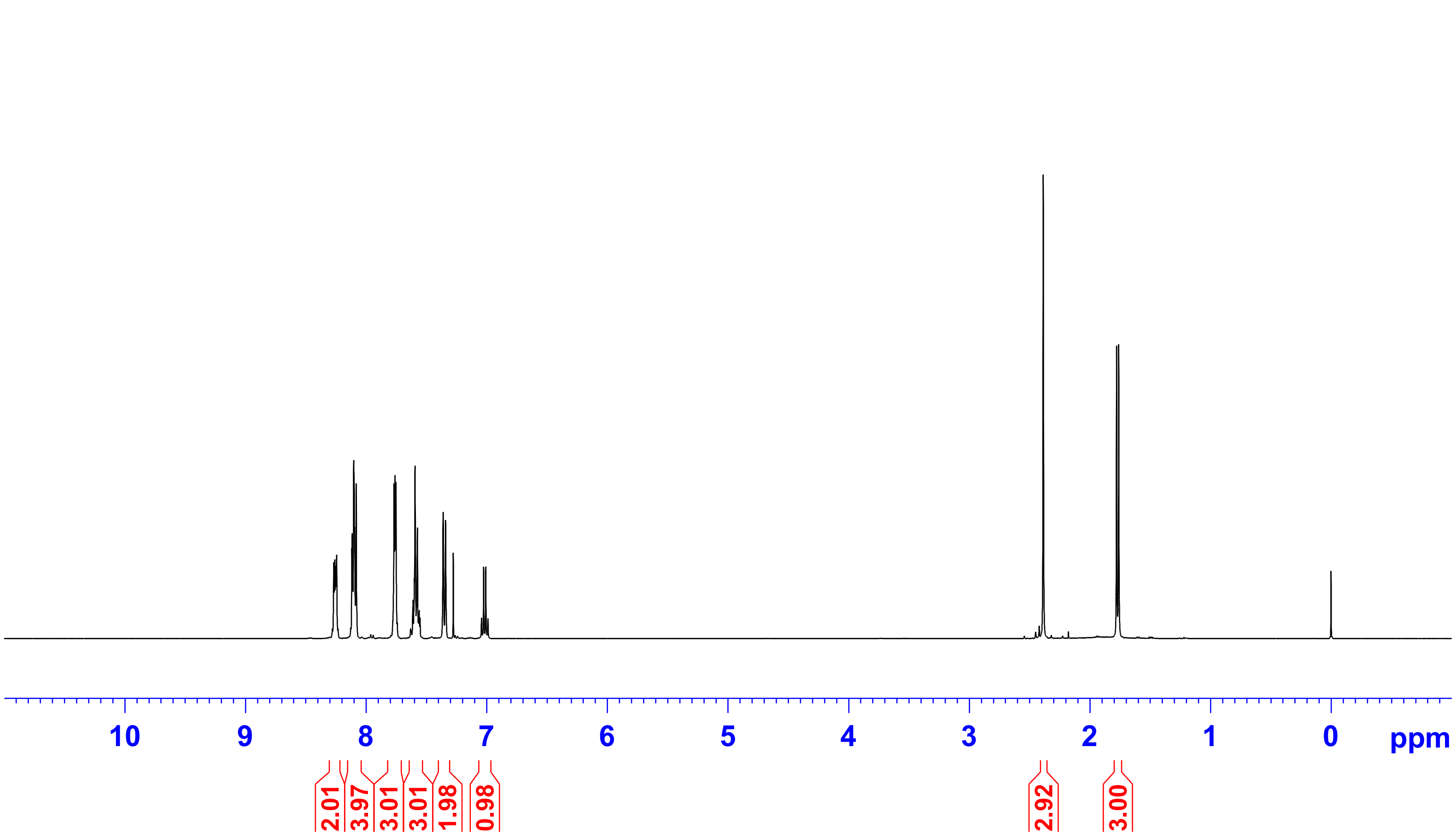


```
NAME       HNMR-gwg-3-87-1
EXPNO                    1
PROCNO                   1
Date_             20190826
Time                 13.06
INSTRUM              spect
PROBHD    5 mm PABBO BB-
PULPROG               zg30
TD                   65536
SOLVENT              CDCl3
NS                       4
DS                       0
SWH               8223.685 Hz
FIDRES            0.125483 Hz
AQ               3.9846387 sec
RG                     228
DW                  60.800 usec
DE                    6.00 usec
TE                   293.9 K
D1              1.00000000 sec
TD0                      1

======== CHANNEL f1 ========
NUC1                    1H
P1                   15.80 usec
PL1                  -1.00 dB
PL1W           12.17476940 W
SFO1           400.1324710 MHz
SI                   32768
SF             400.1300026 MHz
WDW                     EM
SSB                      0
LB                    0.30 Hz
GB                       0
PC                    1.00
```

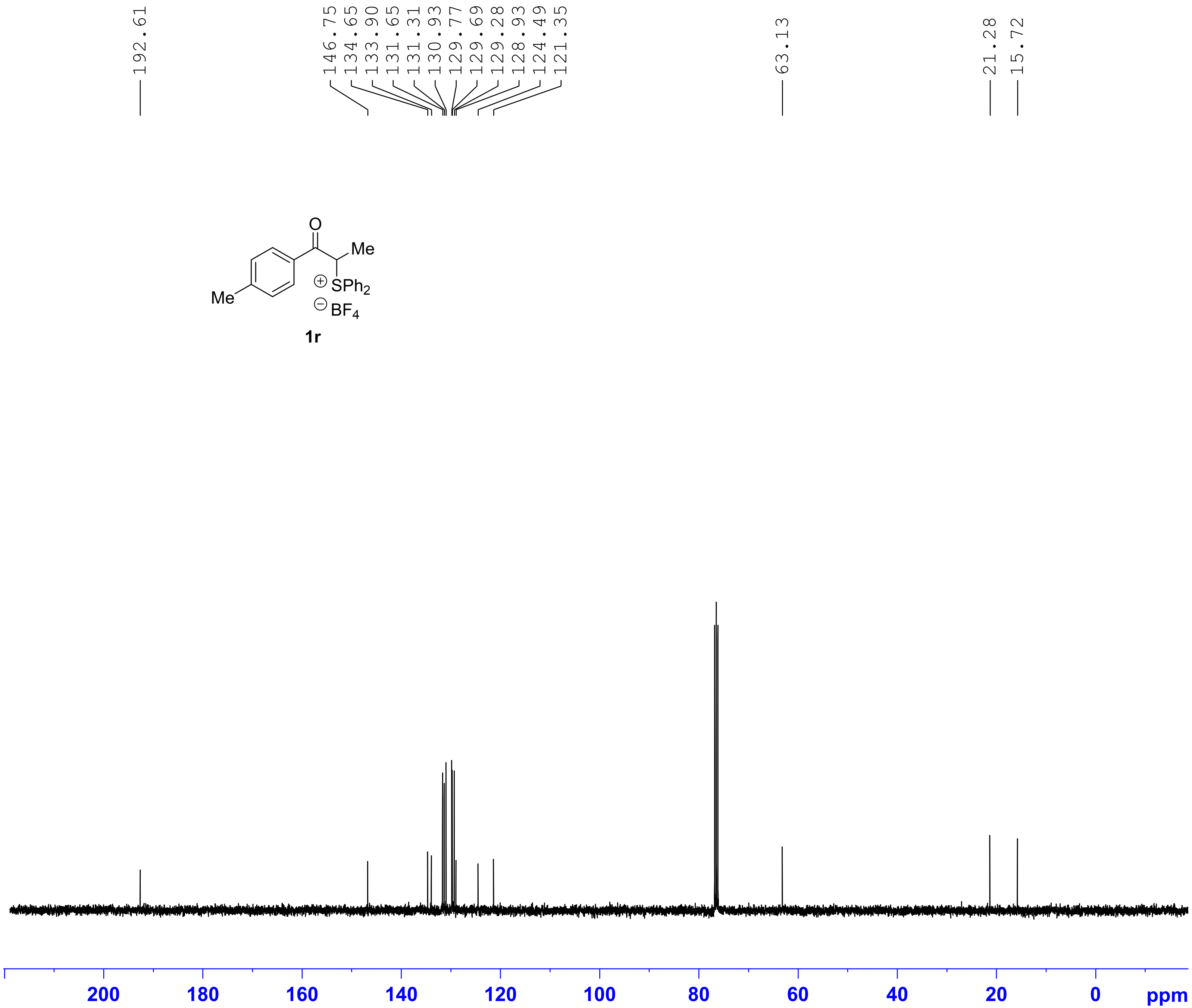

1r

NAME	CNMR-gwg-3-87-1
EXPNO	1
PROCNO	1
Date_	20190826
Time	13.13
INSTRUM	spect
PROBHD	5 mm PABBO BB-
PULPROG	zgpg30
TD	65536
SOLVENT	CDCl3
NS	47
DS	0
SWH	24038.461 Hz
FIDRES	0.366798 Hz
AQ	1.3631988 sec
RG	181
DW	20.800 usec
DE	6.00 usec
TE	294.5 K
D1	2.00000000 sec
D11	0.03000000 sec
TD0	1
======== CHANNEL f1 ========	
NUC1	13C
P1	8.60 usec
PL1	-3.00 dB
PL1W	60.64365387 W
SFO1	100.6228298 MHz
======== CHANNEL f2 ========	
CPDPRG2	waltz16
NUC2	1H
PCPD2	80.00 usec
PL2	-1.00 dB
PL12	14.39 dB
PL13	18.00 dB
PL2W	12.17476940 W
PL12W	0.35193357 W
PL13W	0.15327126 W
SFO2	400.1316005 MHz
SI	32768
SF	100.6128330 MHz
WDW	EM
SSB	0
LB	1.00 Hz
GB	0
PC	1.40

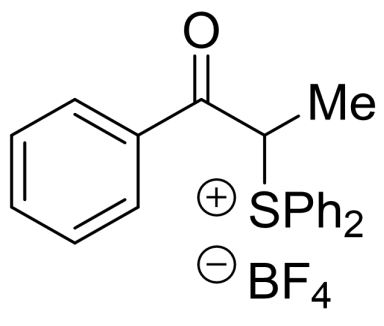

1s

```
NAME       HNMR-gwg-2-20-model substrate
EXPNO                    1
PROCNO                   1
Date_             20190814
Time                 16.38
INSTRUM              spect
PROBHD     5 mm PABBO BB-
PULPROG               zg30
TD                   65536
SOLVENT              CDCl3
NS                       4
DS                       0
SWH               8223.685 Hz
FIDRES            0.125483 Hz
AQ               3.9846387 sec
RG                      64
DW                  60.800 usec
DE                    6.00 usec
TE                   294.5 K
D1              1.00000000 sec
TD0                      1

======== CHANNEL f1 ========
NUC1                    1H
P1                   15.80 usec
PL1                  -1.00 dB
PL1W           12.17476940 W
SFO1           400.1324710 MHz
SI                   32768
SF             400.1300048 MHz
WDW                     EM
SSB                      0
LB                    0.30 Hz
GB                       0
PC                    1.00
```

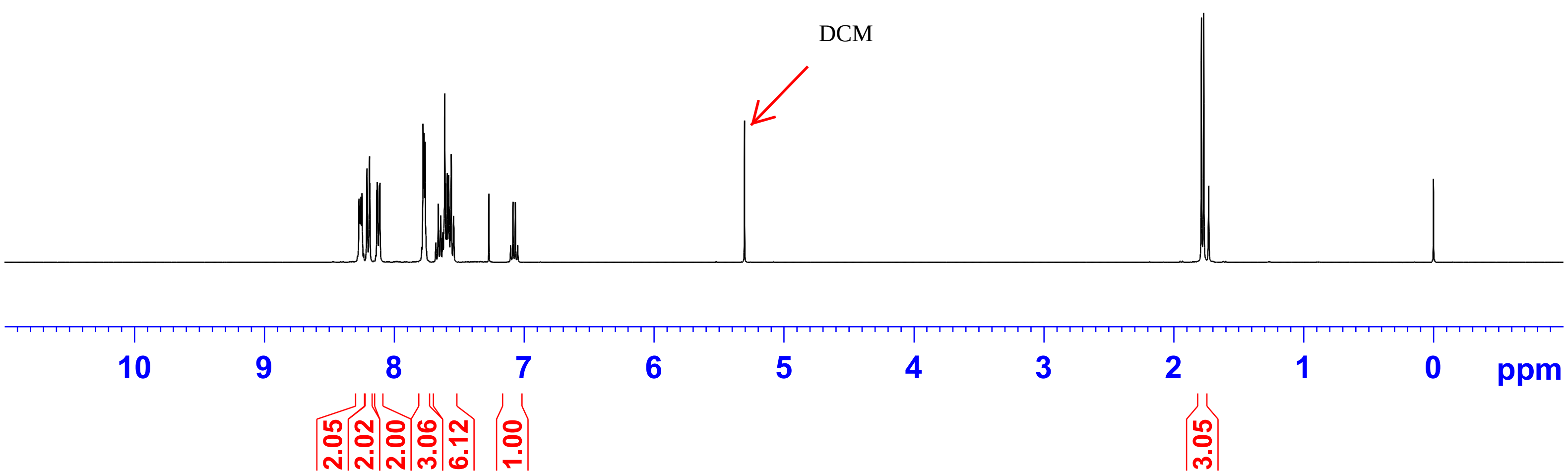

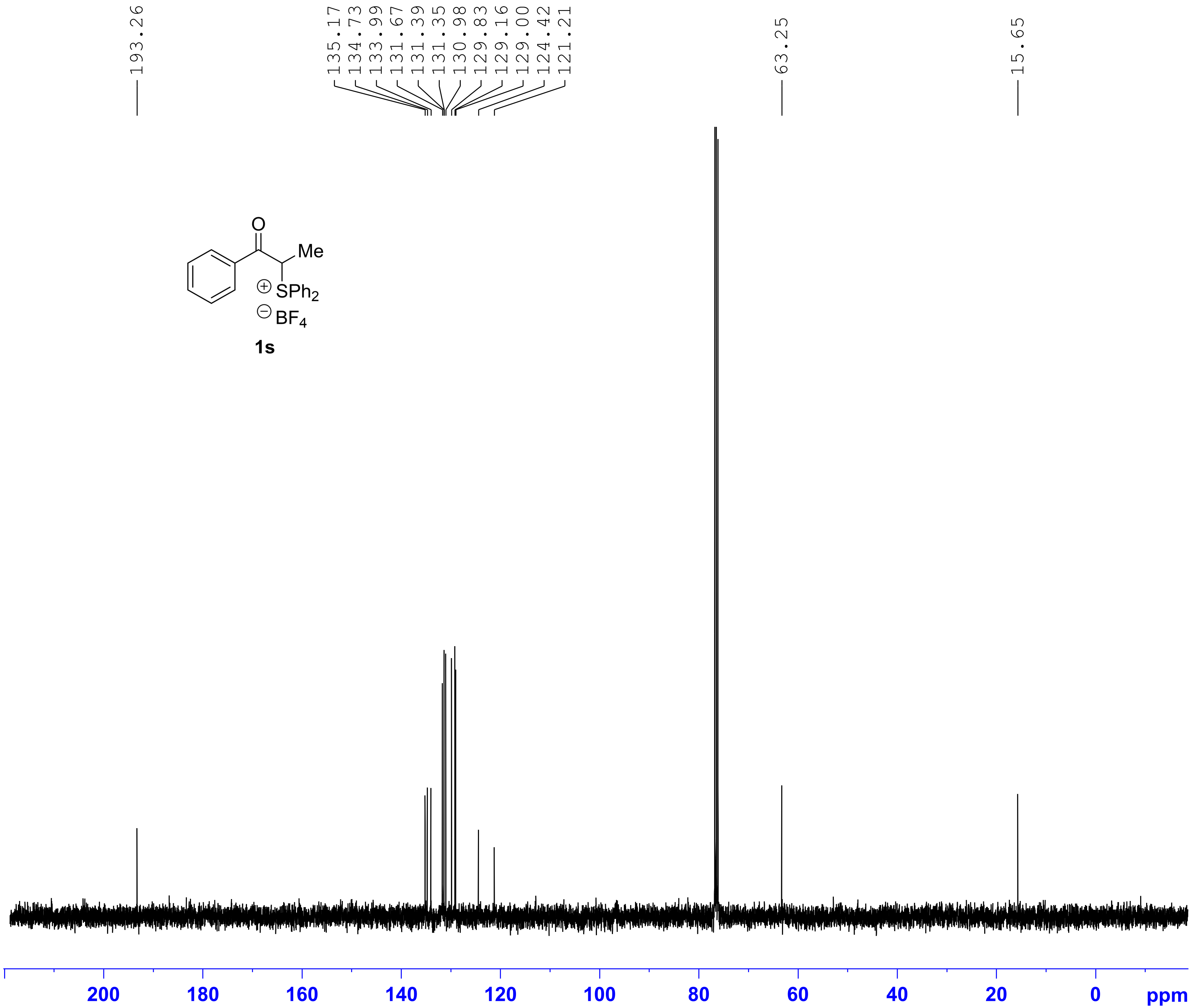

1s

NAME	CNMR-gwg-2-20-model substrate
EXPNO	1
PROCNO	1
Date_	20190814
Time	16.46
INSTRUM	spect
PROBHD	5 mm PABBO BB-
PULPROG	zgpg30
TD	65536
SOLVENT	CDCl3
NS	65
DS	0
SWH	24038.461 Hz
FIDRES	0.366798 Hz
AQ	1.3631988 sec
RG	90.5
DW	20.800 usec
DE	6.00 usec
TE	295.1 K
D1	2.00000000 sec
D11	0.03000000 sec
TD0	1

======== CHANNEL f1 ========

NUC1	13C
P1	8.60 usec
PL1	-3.00 dB
PL1W	60.64365387 W
SFO1	100.6228298 MHz

======== CHANNEL f2 ========

CPDPRG2	waltz16
NUC2	1H
PCPD2	80.00 usec
PL2	-1.00 dB
PL12	14.39 dB
PL13	18.00 dB
PL2W	12.17476940 W
PL12W	0.35193357 W
PL13W	0.15327126 W
SFO2	400.1316005 MHz
SI	32768
SF	100.6128330 MHz
WDW	EM
SSB	0
LB	1.00 Hz
GB	0
PC	1.40

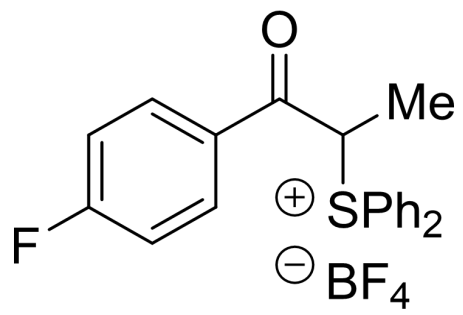

1t

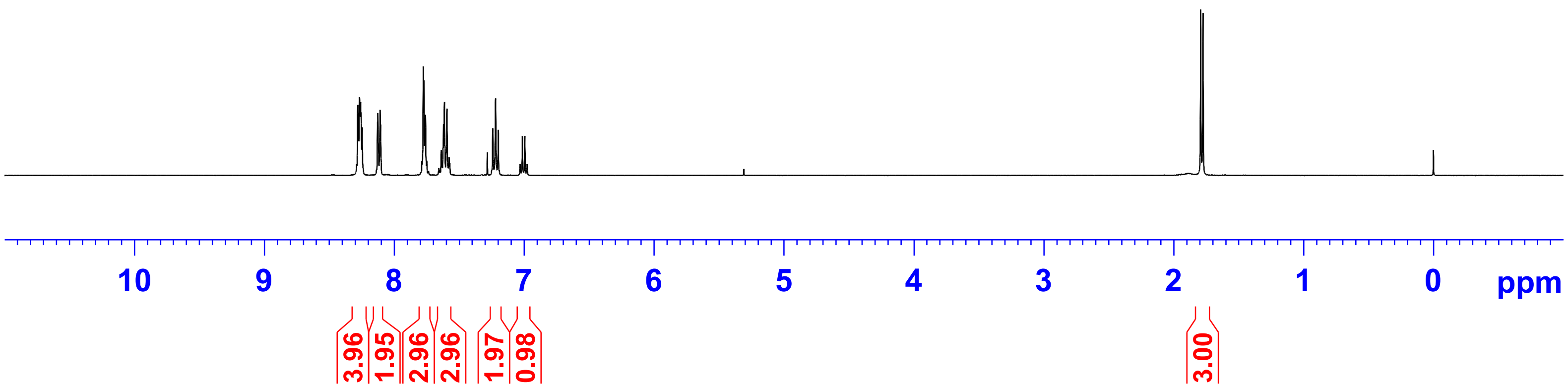


```
NAME       HNMR-gwg-3-61-1
EXPNO                    1
PROCNO                   1
Date_             20190815
Time                 16.36
INSTRUM              spect
PROBHD     5 mm PABBO BB-
PULPROG               zg30
TD                   65536
SOLVENT              CDCl3
NS                       4
DS                       0
SWH               8223.685 Hz
FIDRES            0.125483 Hz
AQ               3.9846387 sec
RG                      64
DW                  60.800 usec
DE                    6.00 usec
TE                   294.3 K
D1              1.00000000 sec
TD0                      1

======== CHANNEL f1 ========
NUC1                    1H
P1                   15.80 usec
PL1                  -1.00 dB
PL1W           12.17476940 W
SFO1           400.1324710 MHz
SI                   32768
SF             400.1299999 MHz
WDW                     EM
SSB                      0
LB                    0.30 Hz
GB                       0
PC                    1.00
```

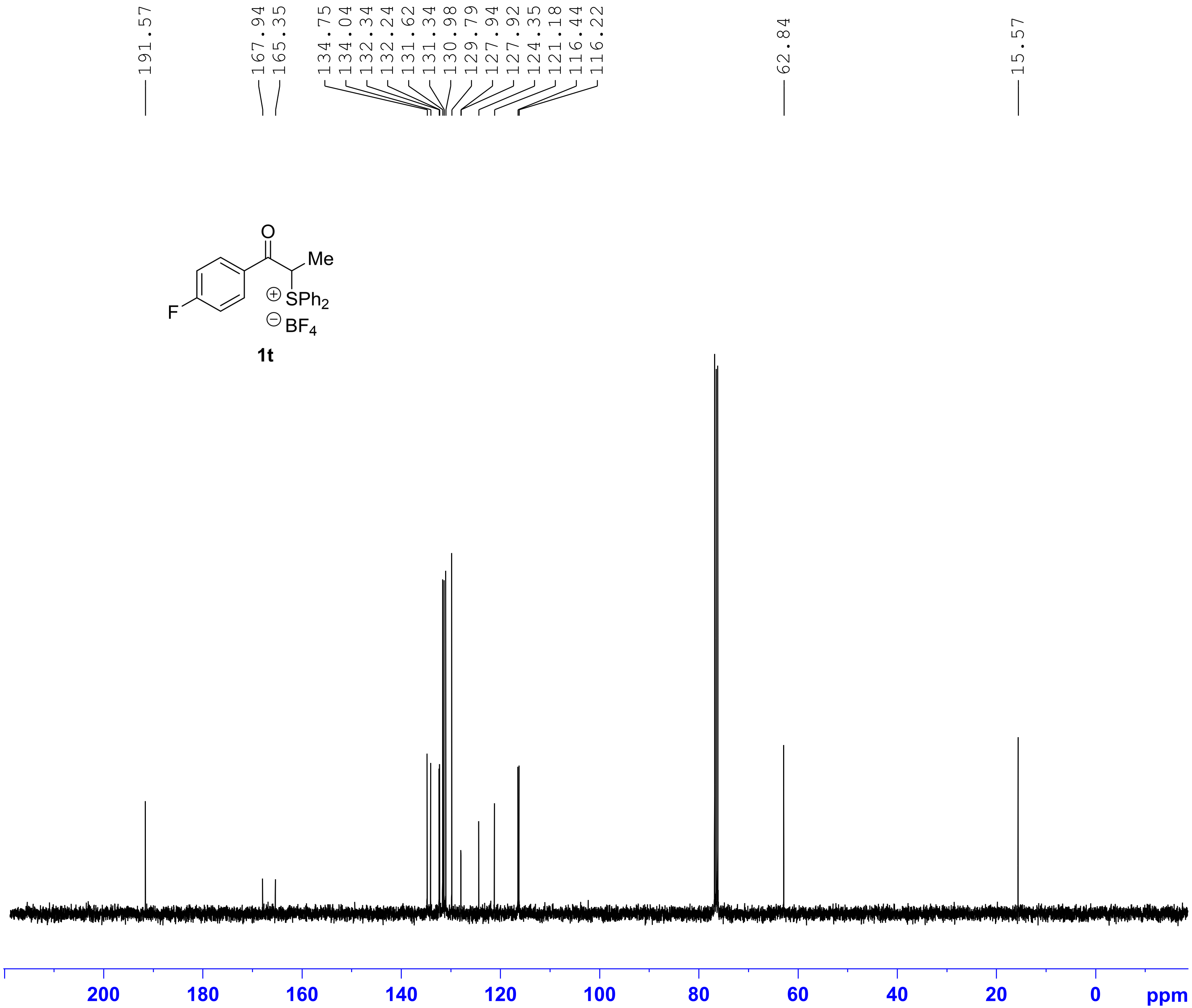

1t

```
NAME       CNMR-gwg-3-61-1
EXPNO                    1
PROCNO                   1
Date_             20190815
Time                 16.44
INSTRUM              spect
PROBHD   5 mm PABBO BB-
PULPROG            zgpg30
TD                   65536
SOLVENT              CDCl3
NS                      74
DS                       0
SWH              24038.461 Hz
FIDRES            0.366798 Hz
AQ               1.3631988 sec
RG                     228
DW                  20.800 usec
DE                    6.00 usec
TE                   294.7 K
D1              2.00000000 sec
D11             0.03000000 sec
TD0                      1

======== CHANNEL f1 ========
NUC1                   13C
P1                    8.60 usec
PL1                  -3.00 dB
PL1W           60.64365387 W
SFO1           100.6228298 MHz

======== CHANNEL f2 ========
CPDPRG2            waltz16
NUC2                    1H
PCPD2                80.00 usec
PL2                  -1.00 dB
PL12                 14.39 dB
PL13                 18.00 dB
PL2W           12.17476940 W
PL12W           0.35193357 W
PL13W           0.15327126 W
SFO2           400.1316005 MHz
SI                   32768
SF             100.6128330 MHz
WDW                     EM
SSB                      0
LB                    1.00 Hz
GB                       0
PC                    1.40
```

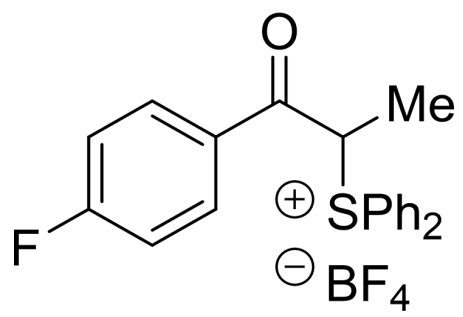

1t

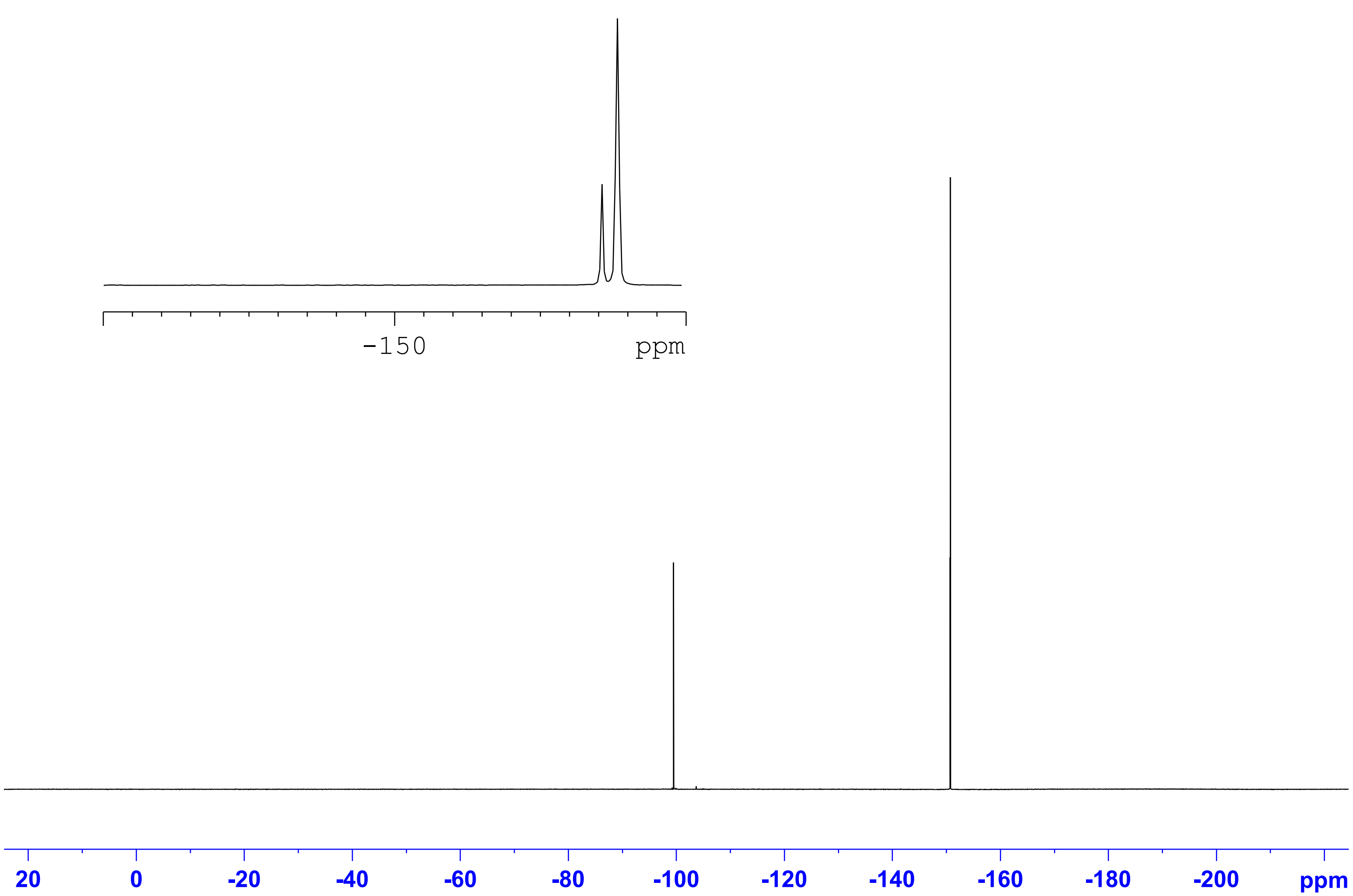


```
NAME       FNMR-gwg-S1i-for published
EXPNO                    1
PROCNO                   1
Date_             20200530
Time                 22.46
INSTRUM              spect
PROBHD    5 mm PABBO BB/
PULPROG            zgpg30
TD                   65536
SOLVENT              CDCl3
NS                       4
DS                       0
SWH              93750.000 Hz
FIDRES            1.430511 Hz
AQ               0.3495753 sec
RG                  196.92
DW                   5.333 usec
DE                    6.50 usec
TE                   297.8 K
D1              2.00000000 sec
D11             0.03000000 sec
TD0                      1

======== CHANNEL f1 ========
SFO1           376.4607162 MHz
NUC1                   19F
P1                   14.70 usec
SI                   32768
SF             376.4983660 MHz
WDW                     EM
SSB                      0
LB                    1.00 Hz
GB                       0
PC                    1.40
```

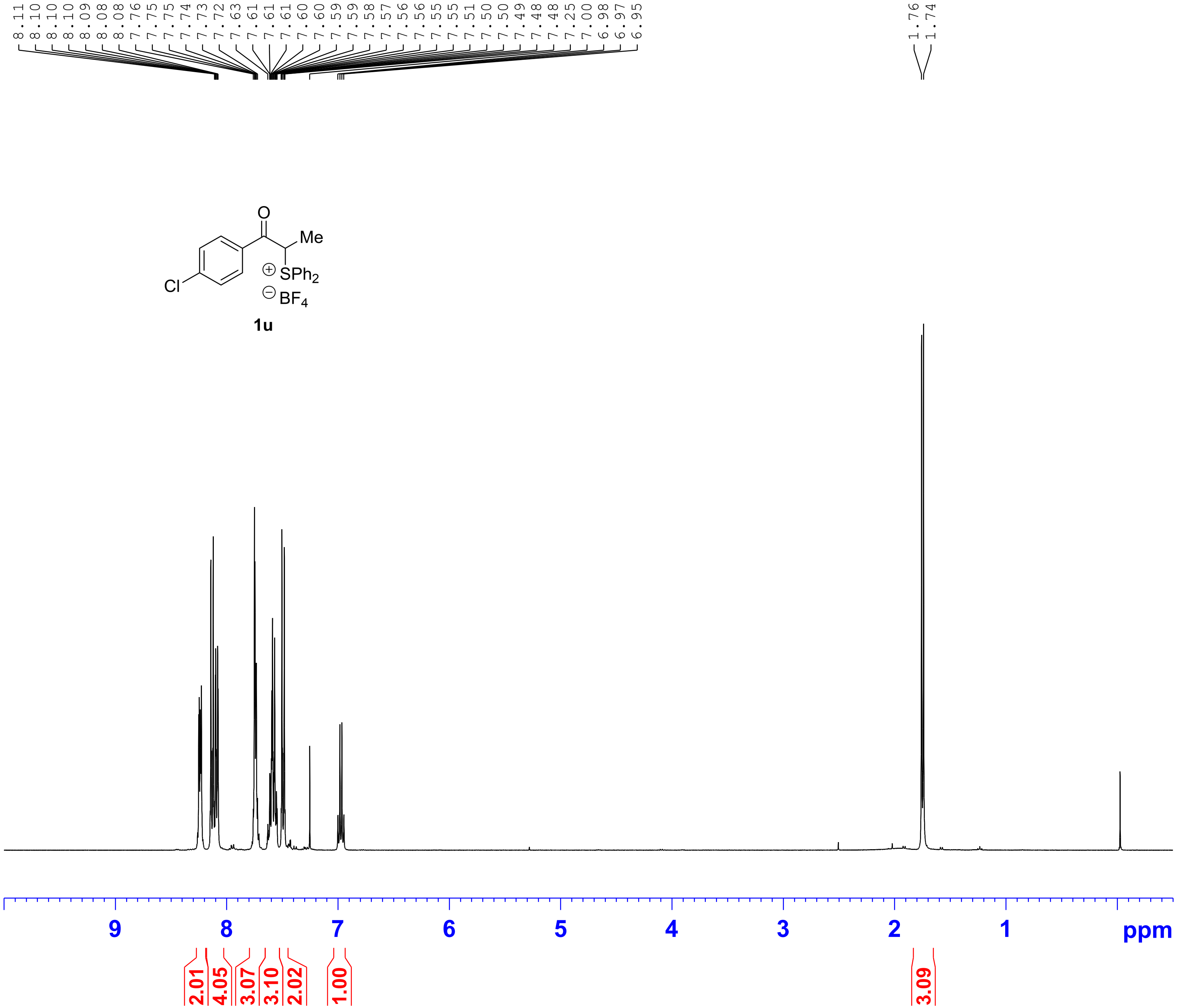


```
NAME      lzy-11-42-G-SM-good-topshim
EXPNO                     1
PROCNO                    1
Date_              20221105
Time                  22.13
INSTRUM               spect
PROBHD    5 mm DUL 13C-1
PULPROG                zg30
TD                    65536
SOLVENT               CDCl3
NS                        4
DS                        0
SWH                8223.685 Hz
FIDRES             0.125483 Hz
AQ                3.9846387 sec
RG                      287
DW                   60.800 usec
DE                     6.00 usec
TE                    295.4 K
D1               1.00000000 sec
TD0                       1

======== CHANNEL f1 ========
NUC1                     1H
P1                    15.80 usec
PL1                   -1.00 dB
PL1W            12.17476940 W
SFO1            400.1324710 MHz
SI                    32768
SF              400.1300118 MHz
WDW                      EM
SSB                       0
LB                     0.30 Hz
GB                        0
PC                     1.00
```

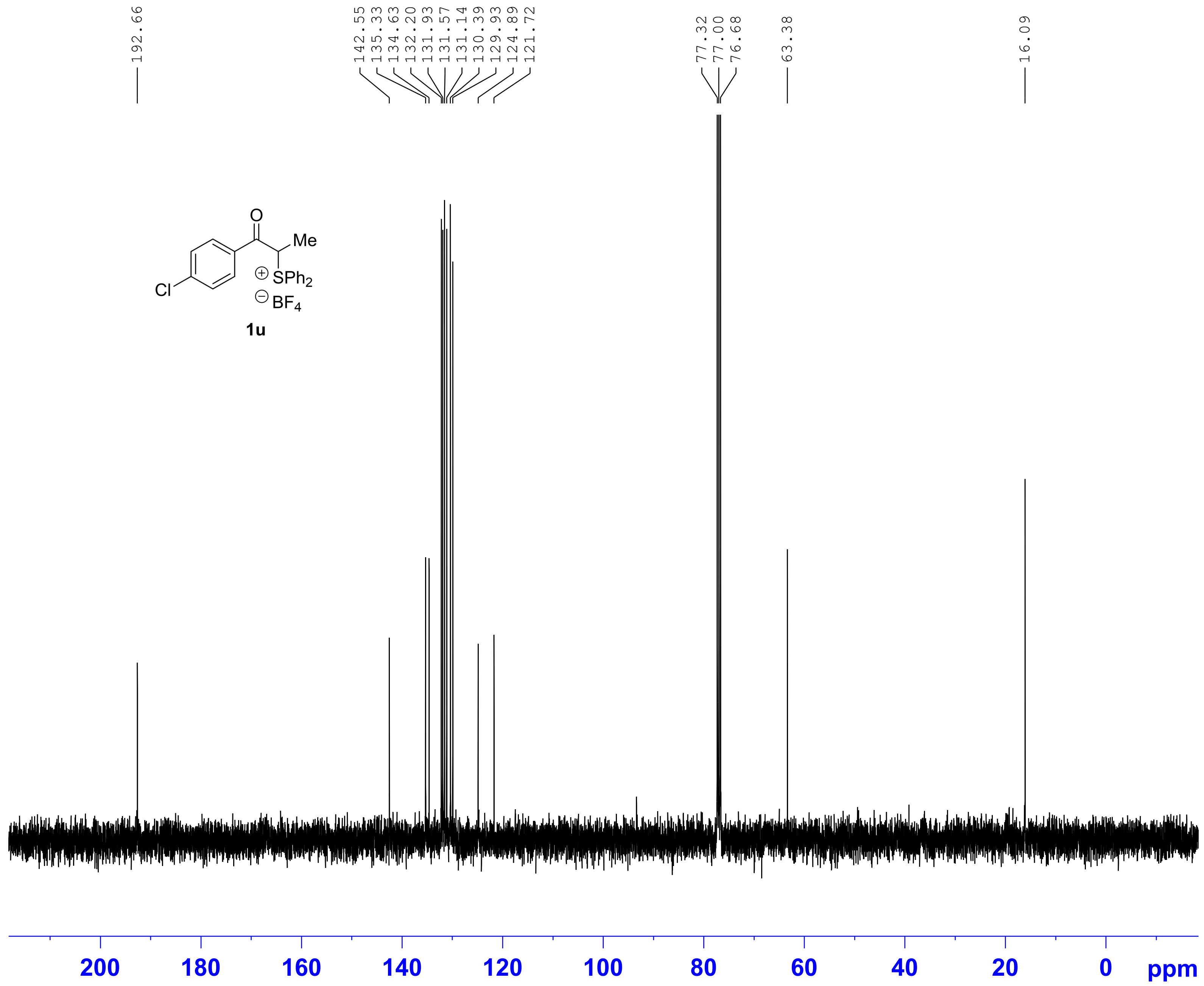

NAME	lzy-11-42-G-SM-C
EXPNO	3
PROCNO	1
Date_	20221105
Time_	22.03 h
INSTRUM	AvanceNeo 400MHz
PROBHD	Z163739_0629 (
PULPROG	zgpg30
TD	65536
SOLVENT	CDCl3
NS	40
DS	4
SWH	23809.523 Hz
FIDRES	0.726609 Hz
AQ	1.3763061 sec
RG	22.6
DW	21.000 usec
DE	6.50 usec
TE	302.2 K
D1	2.00000000 sec
D11	0.03000000 sec
TD0	1
SFO1	100.6354036 MHz
NUC1	13C
P0	2.67 usec
P1	8.00 usec
SI	32768
SF	100.6253473 MHz
WDW	EM
SSB	0
LB	1.00 Hz
GB	0
PC	1.40

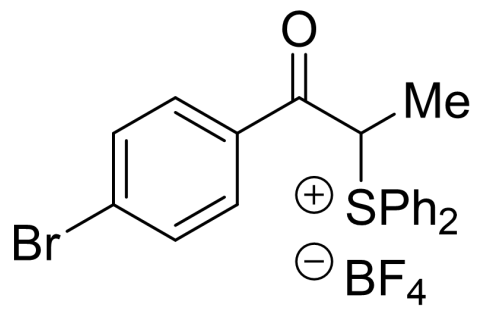

1v

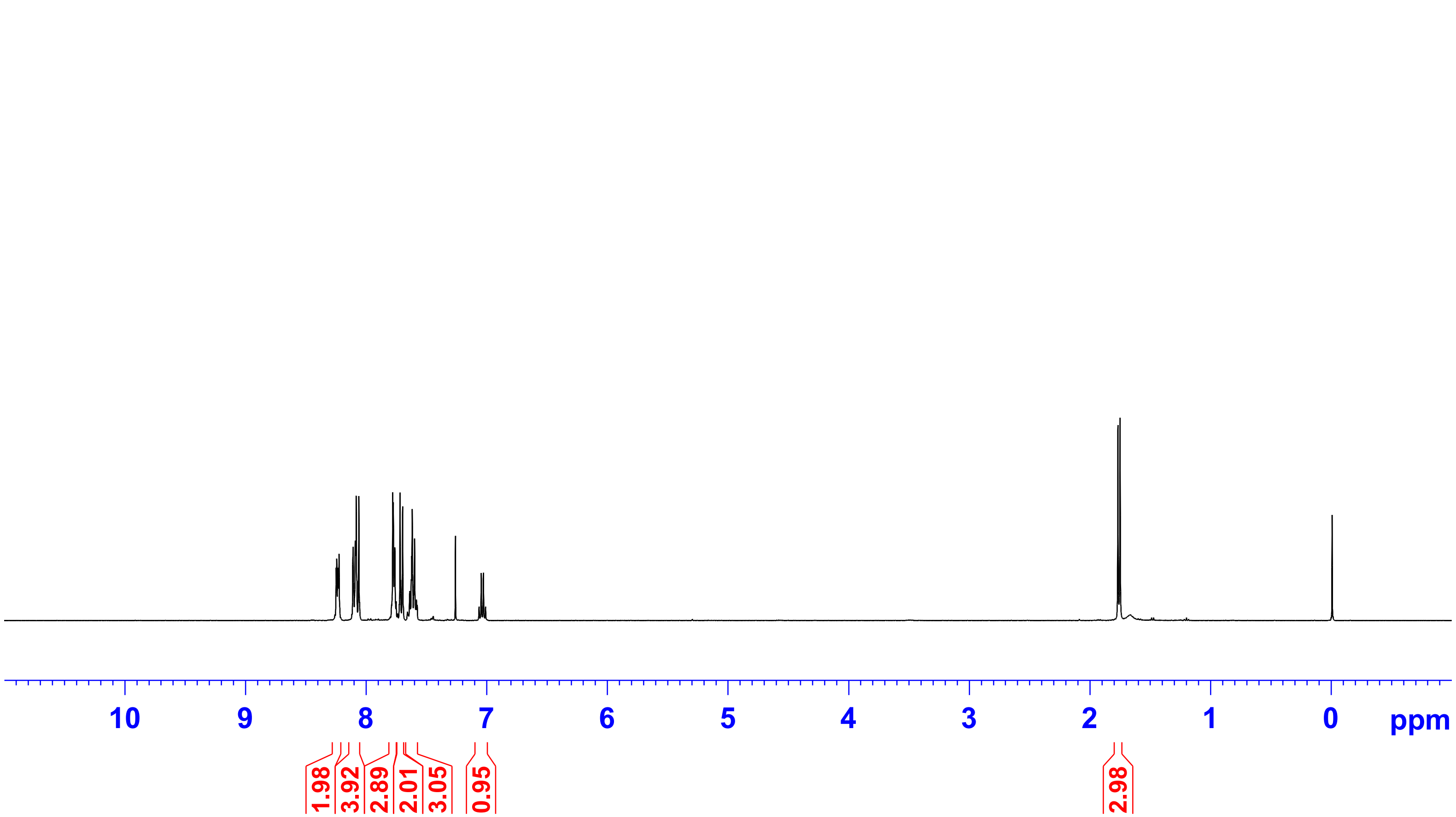

NAME	HNMR-gwg-3-31
EXPNO	1
PROCNO	1
Date_	20190801
Time_	22.00
INSTRUM	spect
PROBHD	5 mm PABBO BB-
PULPROG	zg30
TD	65536
SOLVENT	CDCl3
NS	3
DS	0
SWH	8223.685 Hz
FIDRES	0.125483 Hz
AQ	3.9846387 sec
RG	64
DW	60.800 usec
DE	6.00 usec
TE	294.7 K
D1	1.00000000 sec
TD0	1

======== CHANNEL f1 ========

NUC1	1H
P1	15.80 usec
PL1	-1.00 dB
PL1W	12.17476940 W
SFO1	400.1324710 MHz
SI	32768
SF	400.1300096 MHz
WDW	EM
SSB	0
LB	0.30 Hz
GB	0
PC	1.00

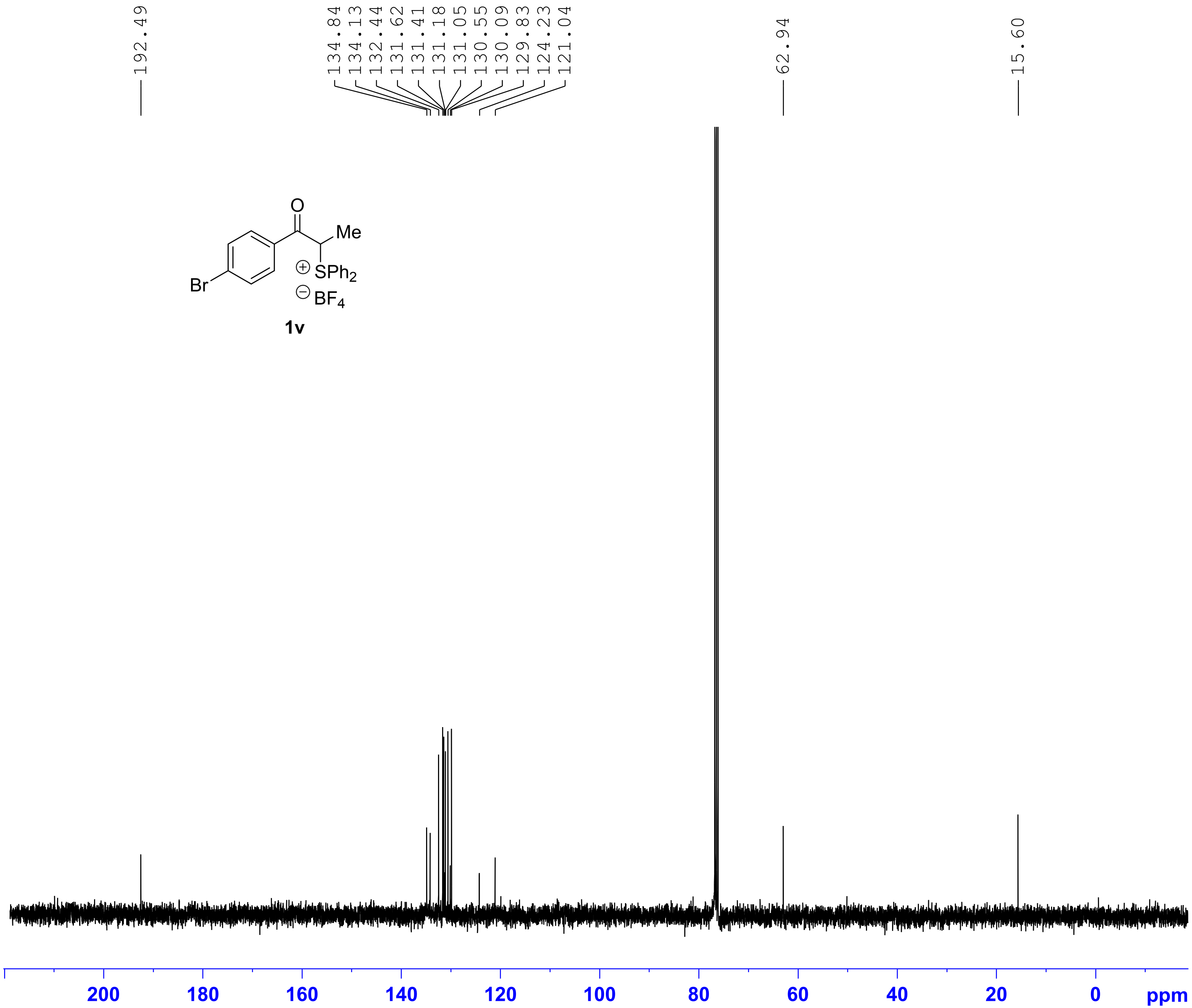


```
NAME       CNMR-gwg-3-31
EXPNO                  1
PROCNO                 1
Date_           20190801
Time               22.09
INSTRUM            spect
PROBHD    5 mm PABBO BB-
PULPROG          zgpg30
TD                 65536
SOLVENT            CDCl3
NS                    73
DS                     0
SWH            24038.461 Hz
FIDRES          0.366798 Hz
AQ             1.3631988 sec
RG                   161
DW                20.800 usec
DE                  6.00 usec
TE                 295.4 K
D1            2.00000000 sec
D11           0.03000000 sec
TD0                    1

======== CHANNEL f1 ========
NUC1                 13C
P1                  8.60 usec
PL1                -3.00 dB
PL1W         60.64365387 W
SFO1         100.6228298 MHz

======== CHANNEL f2 ========
CPDPRG2          waltz16
NUC2                  1H
PCPD2              80.00 usec
PL2                -1.00 dB
PL12               14.39 dB
PL13               18.00 dB
PL2W         12.17476940 W
PL12W         0.35193357 W
PL13W         0.15327126 W
SFO2         400.1316005 MHz
SI                 32768
SF           100.6128330 MHz
WDW                   EM
SSB                    0
LB                  1.00 Hz
GB                     0
PC                  1.40
```

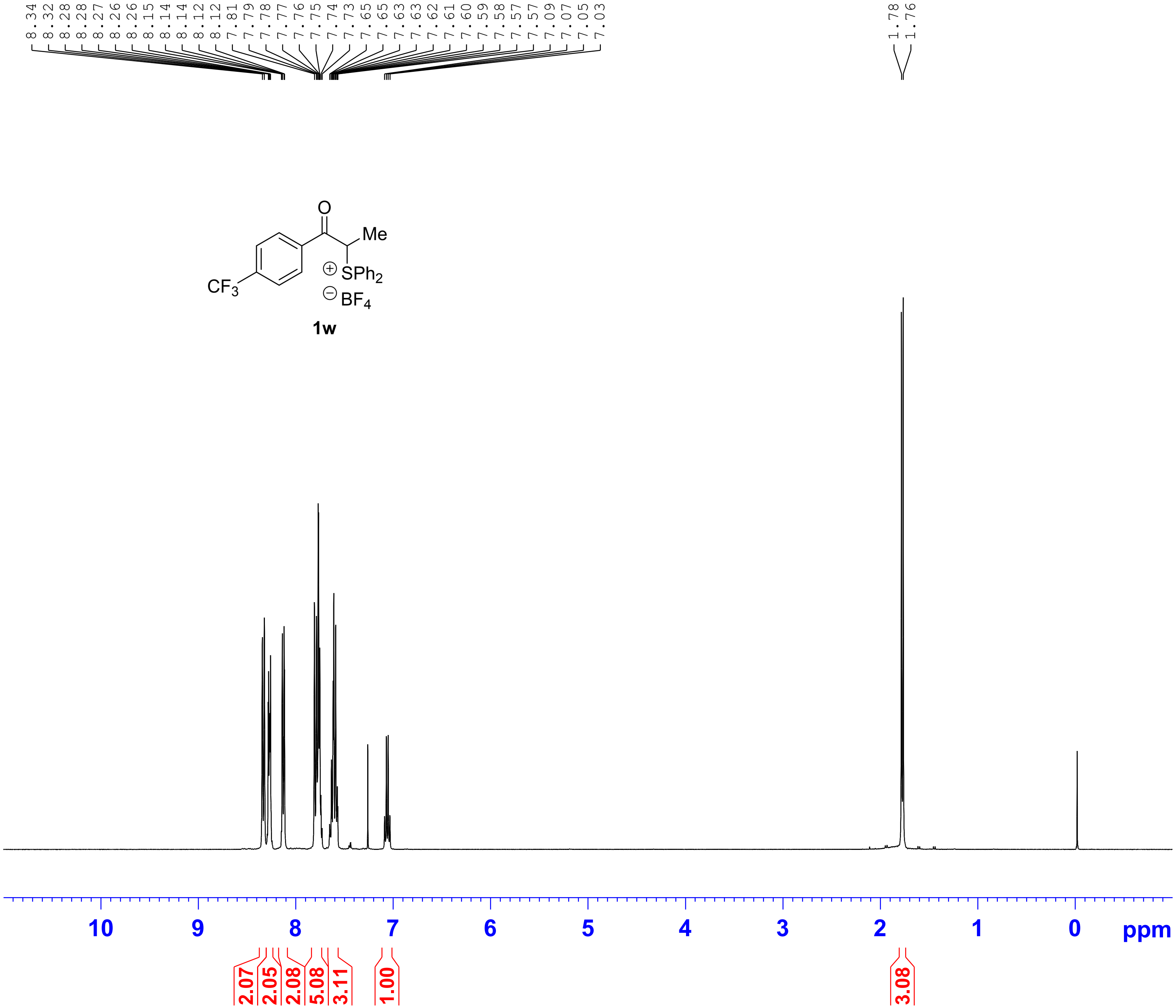

NAME	HNMR-gwg-3-93-1
EXPNO	1
PROCNO	1
Date_	20190828
Time_	20.57
INSTRUM	spect
PROBHD	5 mm PABBO BB-
PULPROG	zg30
TD	65536
SOLVENT	CDCl3
NS	4
DS	0
SWH	8223.685 Hz
FIDRES	0.125483 Hz
AQ	3.9846387 sec
RG	228
DW	60.800 usec
DE	6.00 usec
TE	293.9 K
D1	1.00000000 sec
TD0	1

======== CHANNEL f1 ========

NUC1	1H
P1	15.80 usec
PL1	-1.00 dB
PL1W	12.17476940 W
SFO1	400.1324710 MHz
SI	32768
SF	400.1300095 MHz
WDW	EM
SSB	0
LB	0.30 Hz
GB	0
PC	1.00

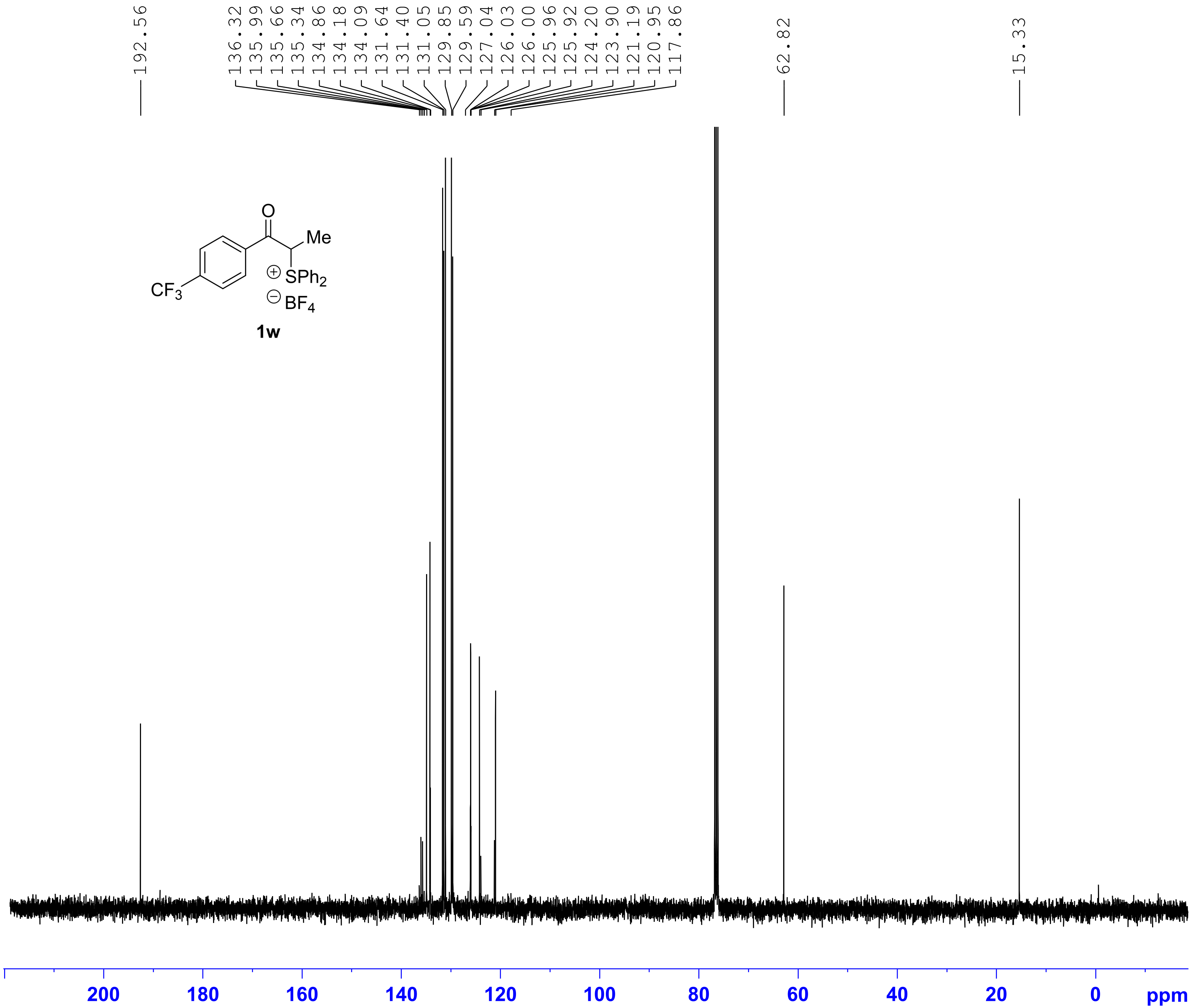


```
NAME       CNMR-gwg-3-93-1
EXPNO                    1
PROCNO                   1
Date_             20190828
Time                 21.15
INSTRUM              spect
PROBHD     5 mm PABBO BB-
PULPROG            zgpg30
TD                   65536
SOLVENT              CDCl3
NS                     235
DS                       0
SWH              24038.461 Hz
FIDRES            0.366798 Hz
AQ               1.3631988 sec
RG                     362
DW                  20.800 usec
DE                    6.00 usec
TE                   294.9 K
D1              2.00000000 sec
D11             0.03000000 sec
TD0                      1

======== CHANNEL f1 ========
NUC1                   13C
P1                    8.60 usec
PL1                  -3.00 dB
PL1W           60.64365387 W
SFO1           100.6228298 MHz

======== CHANNEL f2 ========
CPDPRG2            waltz16
NUC2                    1H
PCPD2                80.00 usec
PL2                  -1.00 dB
PL12                 14.39 dB
PL13                 18.00 dB
PL2W           12.17476940 W
PL12W           0.35193357 W
PL13W           0.15327126 W
SFO2           400.1316005 MHz
SI                   32768
SF             100.6128330 MHz
WDW                     EM
SSB                      0
LB                    1.00 Hz
GB                       0
PC                    1.40
```

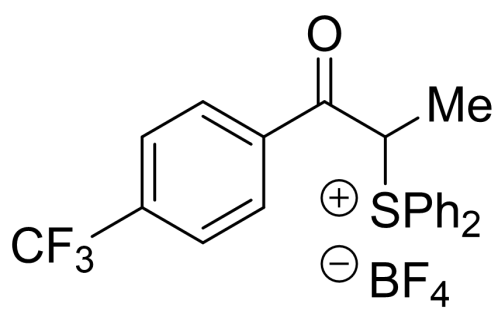

1w

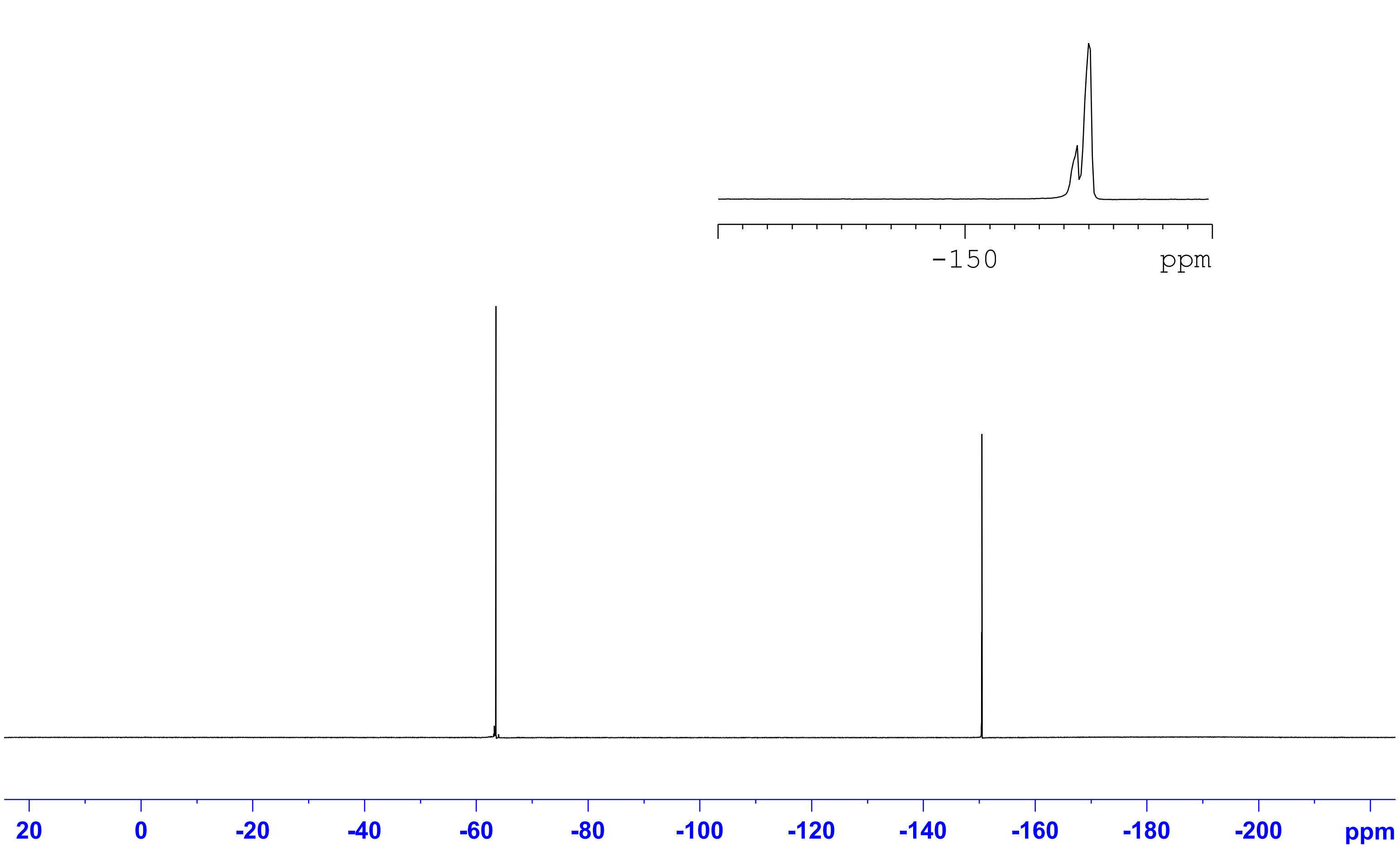


```
NAME      FNMR-gwg-S1j-for published
EXPNO                  1
PROCNO                 1
Date_           20200530
Time               22.40
INSTRUM            spect
PROBHD    5 mm PABBO BB/
PULPROG          zgpg30
TD                65536
SOLVENT            CDCl3
NS                     4
DS                     0
SWH           93750.000 Hz
FIDRES         1.430511 Hz
AQ            0.3495753 sec
RG               196.92
DW                5.333 usec
DE                 6.50 usec
TE                297.9 K
D1           2.00000000 sec
D11          0.03000000 sec
TD0                    1

======== CHANNEL f1 ========
SFO1        376.4607162 MHz
NUC1                 19F
P1                14.70 usec
SI                32768
SF          376.4983660 MHz
WDW                   EM
SSB                    0
LB                 1.00 Hz
GB                     0
PC                 1.40
```

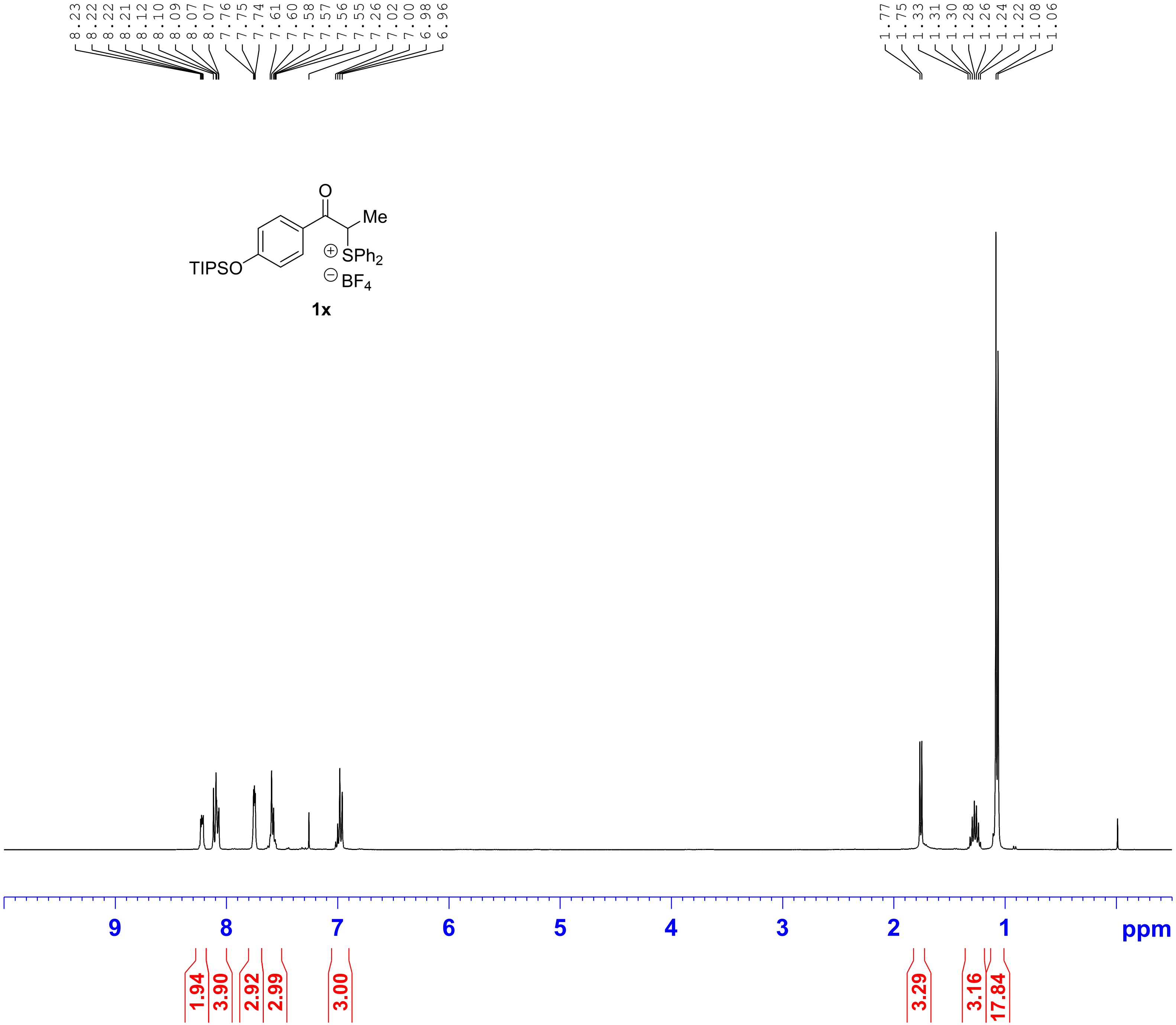

NAME	lzy-12-151-D-SM
EXPNO	1
PROCNO	1
Date_	20230317
Time	9.44 h
INSTRUM	AvanceNeo 400MHz
PROBHD	Z163739_0629 (
PULPROG	zg30
TD	65536
SOLVENT	CDCl3
NS	16
DS	2
SWH	8196.722 Hz
FIDRES	0.250144 Hz
AQ	3.9977460 sec
RG	101
DW	61.000 usec
DE	13.89 usec
TE	296.8 K
D1	1.00000000 sec
TD0	1
SFO1	400.1824711 MHz
NUC1	1H
P0	2.67 usec
P1	8.00 usec
SI	65536
SF	400.1800094 MHz
WDW	EM
SSB	0
LB	0.30 Hz
GB	0
PC	1.00

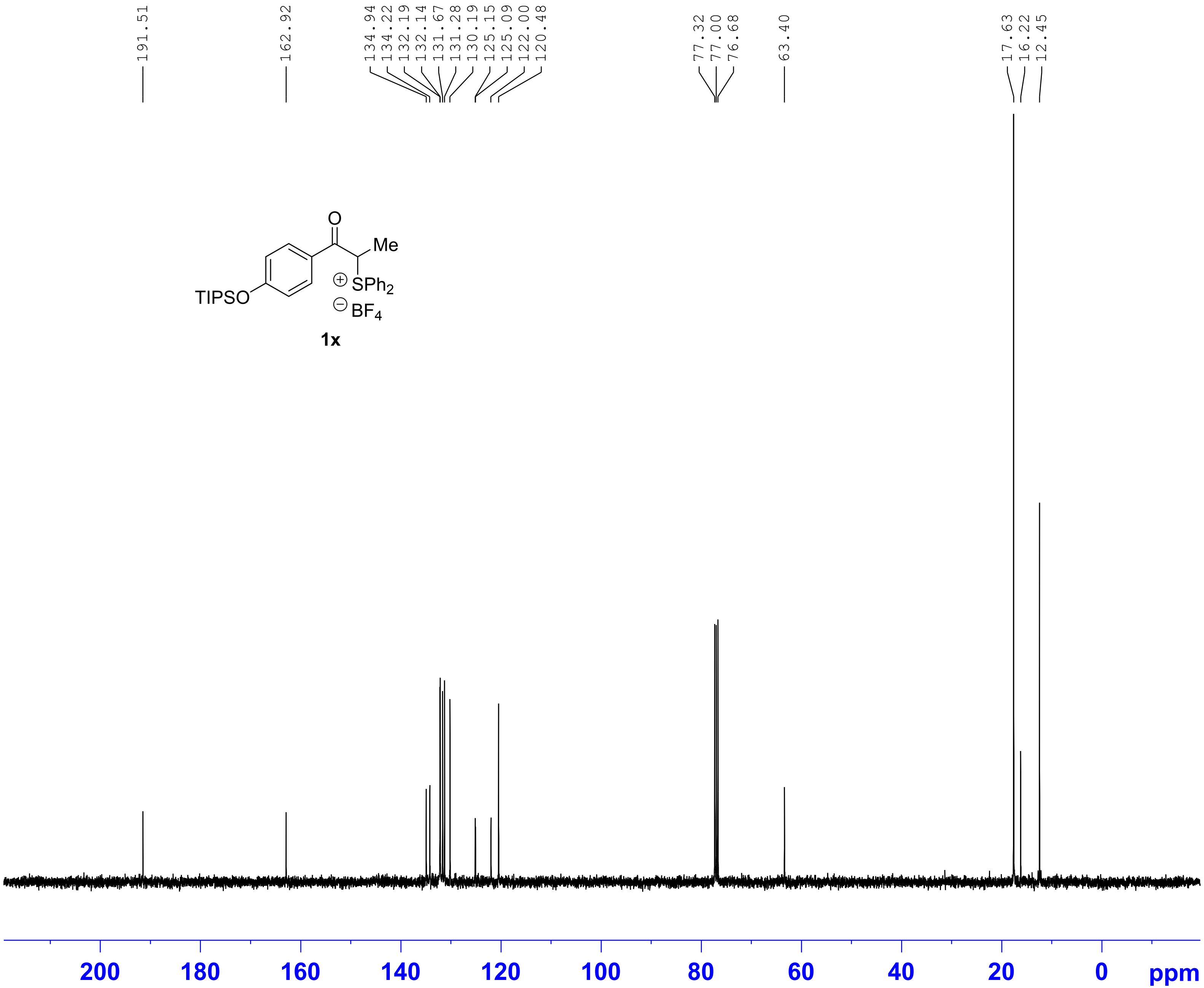

NAME	lzy-11-42-A-SM-C
EXPNO	1
PROCNO	1
Date_	20221104
Time	22.32
INSTRUM	spect
PROBHD	5 mm PABBO BB/
PULPROG	zgpg30
TD	65536
SOLVENT	CDCl3
NS	61
DS	2
SWH	24038.461 Hz
FIDRES	0.366798 Hz
AQ	1.3631988 sec
RG	196.92
DW	20.800 usec
DE	6.50 usec
TE	296.6 K
D1	2.00000000 sec
D11	0.03000000 sec
TD0	1

======== CHANNEL f1 ========

SFO1	100.6228298 MHz
NUC1	13C
P1	9.70 usec
SI	32768
SF	100.6127865 MHz
WDW	EM
SSB	0
LB	1.00 Hz
GB	0
PC	1.40

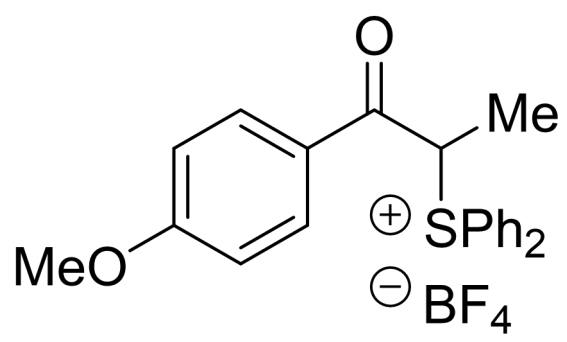

1y

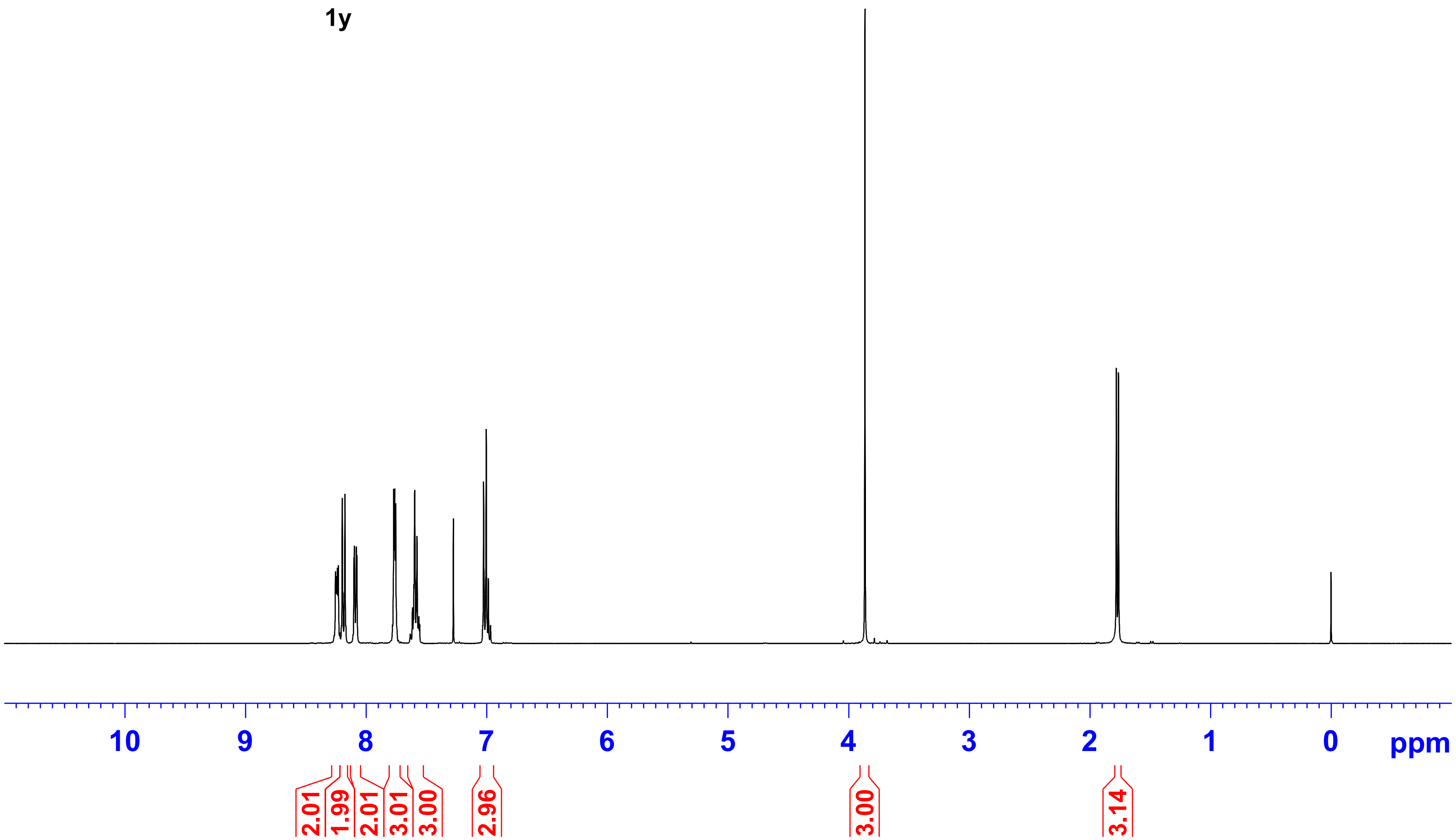

NAME	HNMR-gwg-3-88-1
EXPNO	1
PROCNO	1
Date_	20190826
Time_	13.18
INSTRUM	spect
PROBHD	5 mm PABBO BB-
PULPROG	zg30
TD	65536
SOLVENT	CDCl3
NS	4
DS	0
SWH	8223.685 Hz
FIDRES	0.125483 Hz
AQ	3.9846387 sec
RG	256
DW	60.800 usec
DE	6.00 usec
TE	294.0 K
D1	1.00000000 sec
TD0	1

======== CHANNEL f1 ========

NUC1	1H
P1	15.80 usec
PL1	-1.00 dB
PL1W	12.17476940 W
SFO1	400.1324710 MHz
SI	32768
SF	400.1300028 MHz
WDW	EM
SSB	0
LB	0.30 Hz
GB	0
PC	1.00

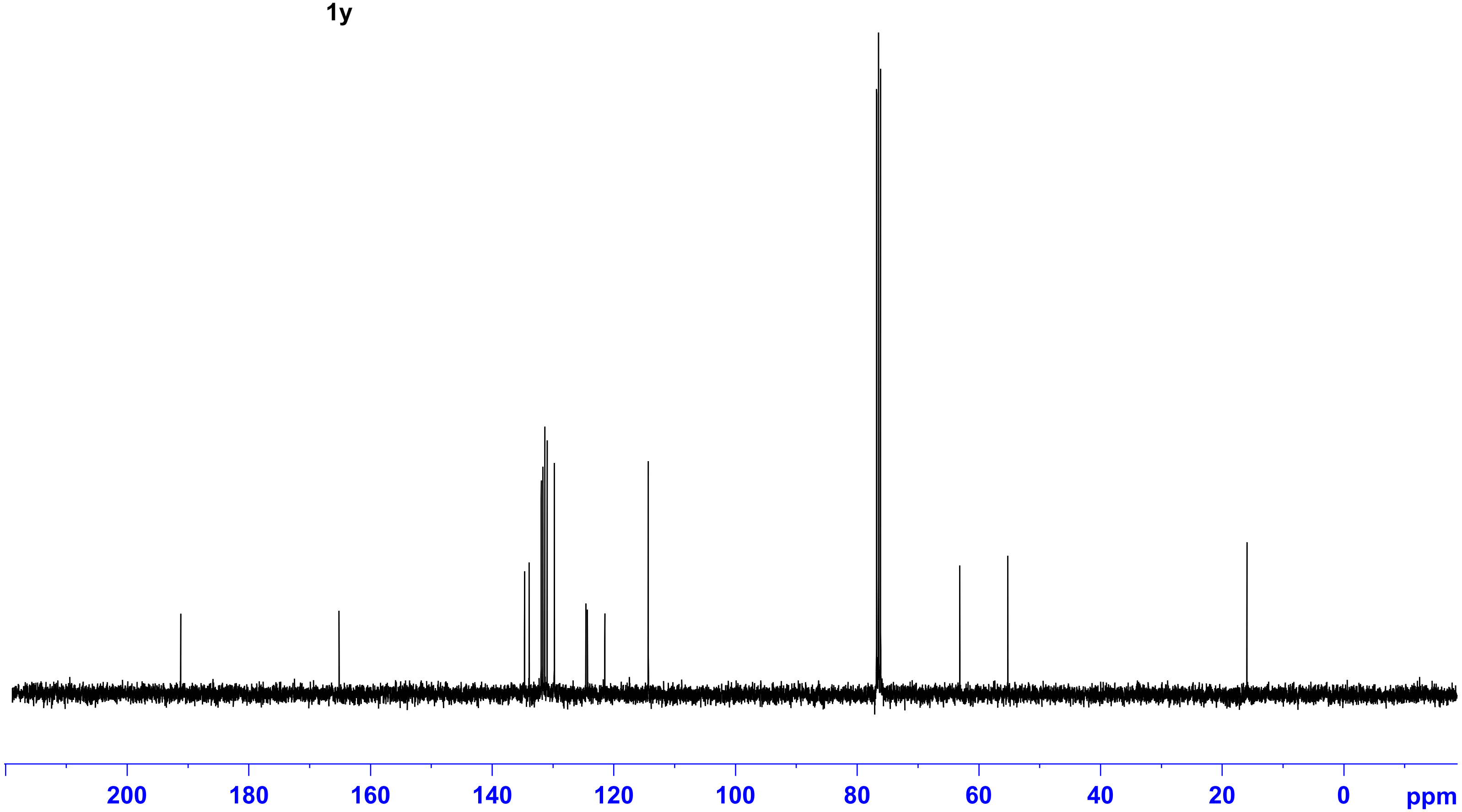

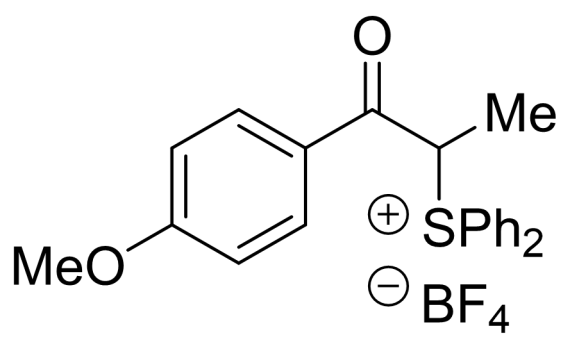

1y

NAME	CNMR-gwg-3-88-1
EXPNO	1
PROCNO	1
Date_	20190826
Time	13.26
INSTRUM	spect
PROBHD	5 mm PABBO BB-
PULPROG	zgpg30
TD	65536
SOLVENT	CDCl3
NS	59
DS	0
SWH	24038.461 Hz
FIDRES	0.366798 Hz
AQ	1.3631988 sec
RG	128
DW	20.800 usec
DE	6.00 usec
TE	294.6 K
D1	2.00000000 sec
D11	0.03000000 sec
TD0	1

======== CHANNEL f1 ========

NUC1	13C
P1	8.60 usec
PL1	-3.00 dB
PL1W	60.64365387 W
SFO1	100.6228298 MHz

======== CHANNEL f2 ========

CPDPRG2	waltz16
NUC2	1H
PCPD2	80.00 usec
PL2	-1.00 dB
PL12	14.39 dB
PL13	18.00 dB
PL2W	12.17476940 W
PL12W	0.35193357 W
PL13W	0.15327126 W
SFO2	400.1316005 MHz
SI	32768
SF	100.6128330 MHz
WDW	EM
SSB	0
LB	1.00 Hz
GB	0
PC	1.40

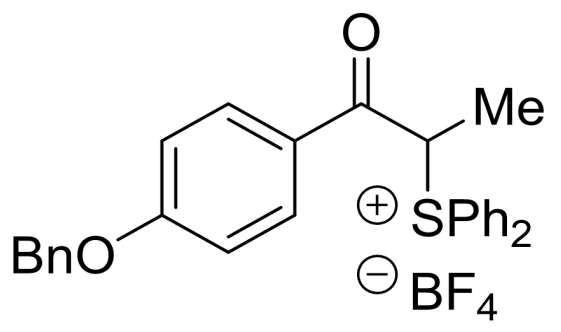

1z

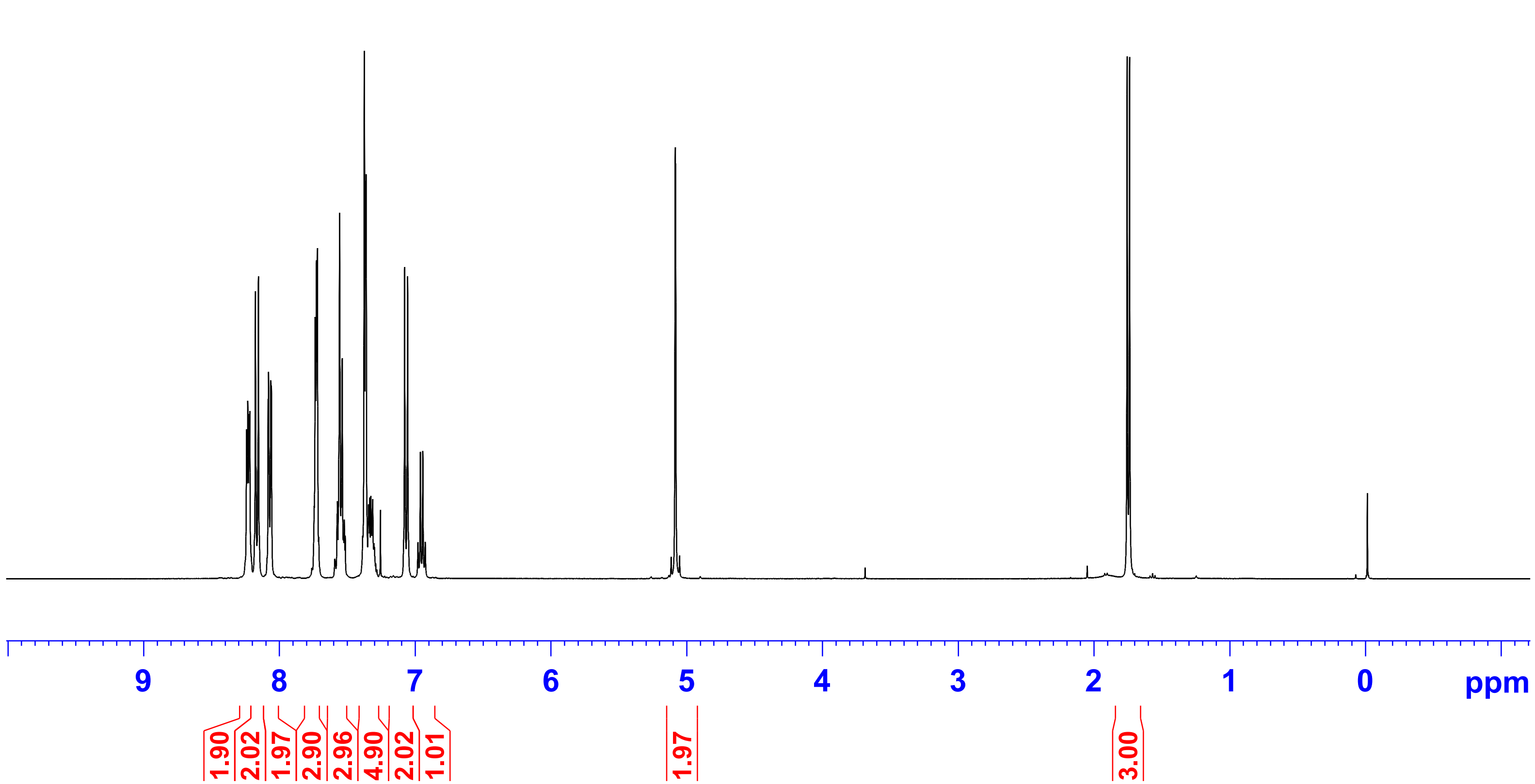

NAME	lzy-11-42-OBN-SM
EXPNO	1
PROCNO	1
Date_	20230420
Time	11.04
INSTRUM	spect
PROBHD	5 mm PABBO BB/
PULPROG	zg30
TD	65536
SOLVENT	CDCl3
NS	16
DS	2
SWH	8012.820 Hz
FIDRES	0.122266 Hz
AQ	4.0894966 sec
RG	31.55
DW	62.400 usec
DE	6.50 usec
TE	296.2 K
D1	1.00000000 sec
TD0	1

======== CHANNEL f1 ========

SFO1	400.1324710 MHz
NUC1	1H
P1	14.50 usec
SI	65536
SF	400.1300112 MHz
WDW	EM
SSB	0
LB	0.30 Hz
GB	0
PC	1.00

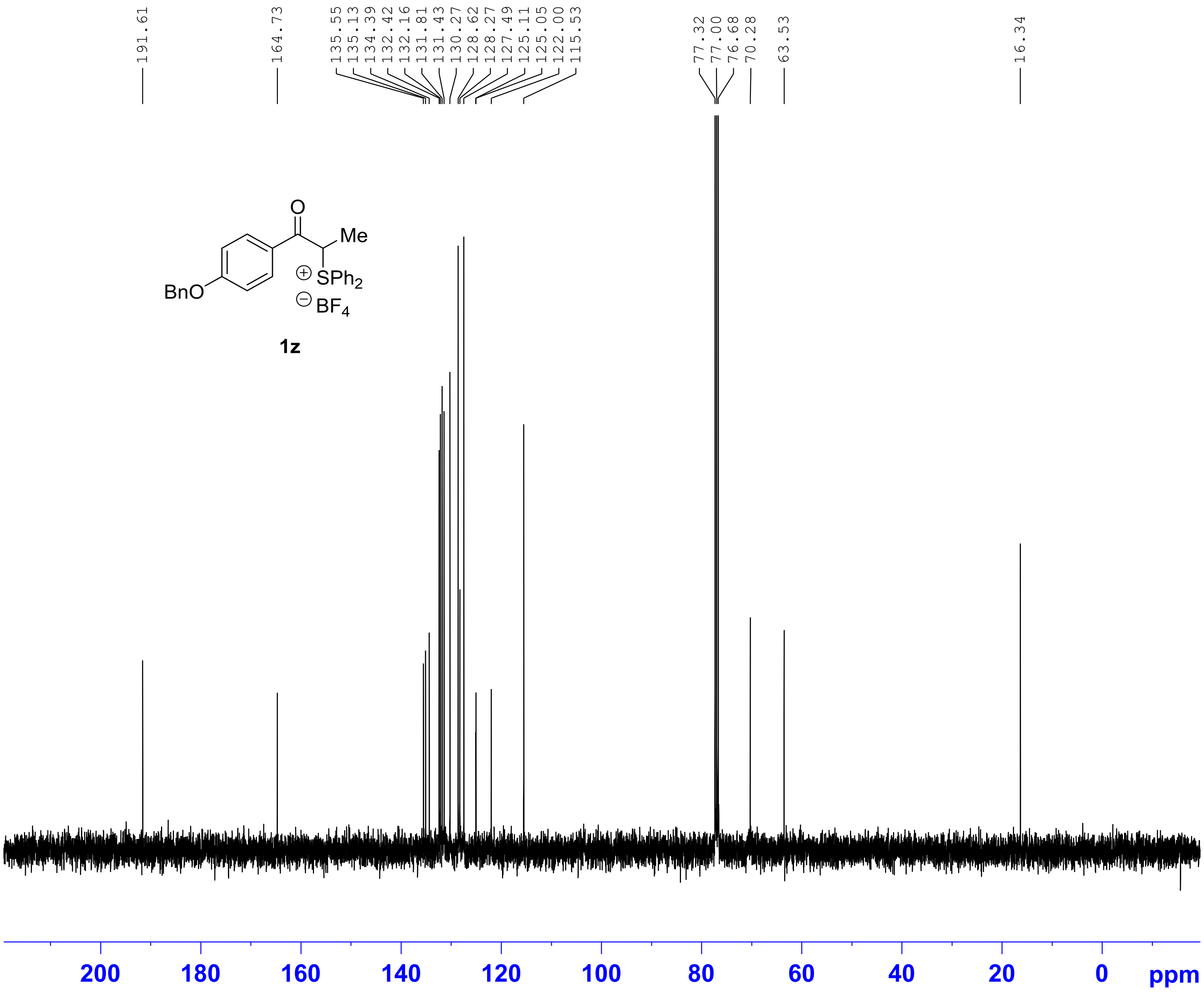

NAME	lzy-11-42-OBN-SM-C
EXPNO	1
PROCNO	1
Date_	20230420
Time	11.07
INSTRUM	spect
PROBHD	5 mm PABBO BB/
PULPROG	zgpg30
TD	65536
SOLVENT	CDCl3
NS	76
DS	2
SWH	24038.461 Hz
FIDRES	0.366798 Hz
AQ	1.3631988 sec
RG	196.92
DW	20.800 usec
DE	6.50 usec
TE	296.9 K
D1	2.00000000 sec
D11	0.03000000 sec
TD0	1

======== CHANNEL f1 ========

SFO1	100.6228298 MHz
NUC1	13C
P1	9.70 usec
SI	32768
SF	100.6127800 MHz
WDW	EM
SSB	0
LB	1.00 Hz
GB	0
PC	1.40

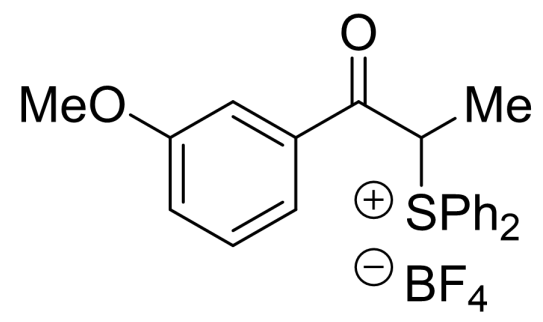

1aa

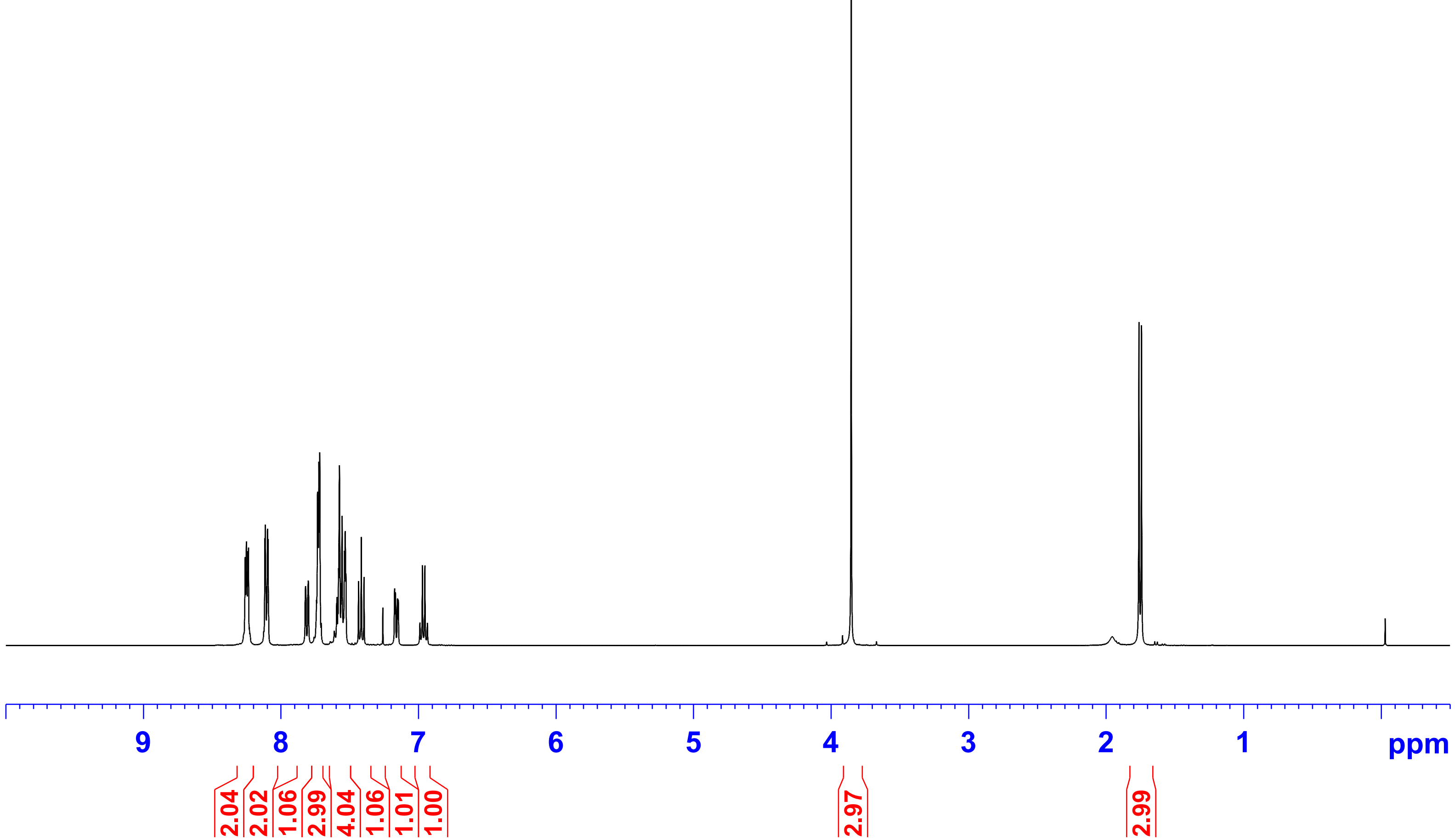

NAME	lzy-11-42-m-OMe-SM
EXPNO	1
PROCNO	1
Date_	20230317
Time	10.07
INSTRUM	spect
PROBHD	5 mm PABBO BB/
PULPROG	zg30
TD	65536
SOLVENT	CDCl3
NS	4
DS	2
SWH	8012.820 Hz
FIDRES	0.122266 Hz
AQ	4.0894966 sec
RG	31.55
DW	62.400 usec
DE	6.50 usec
TE	296.3 K
D1	1.00000000 sec
TD0	1

======== CHANNEL f1 ========

SFO1	400.1324710 MHz
NUC1	1H
P1	14.50 usec
SI	65536
SF	400.1300097 MHz
WDW	EM
SSB	0
LB	0.30 Hz
GB	0
PC	1.00

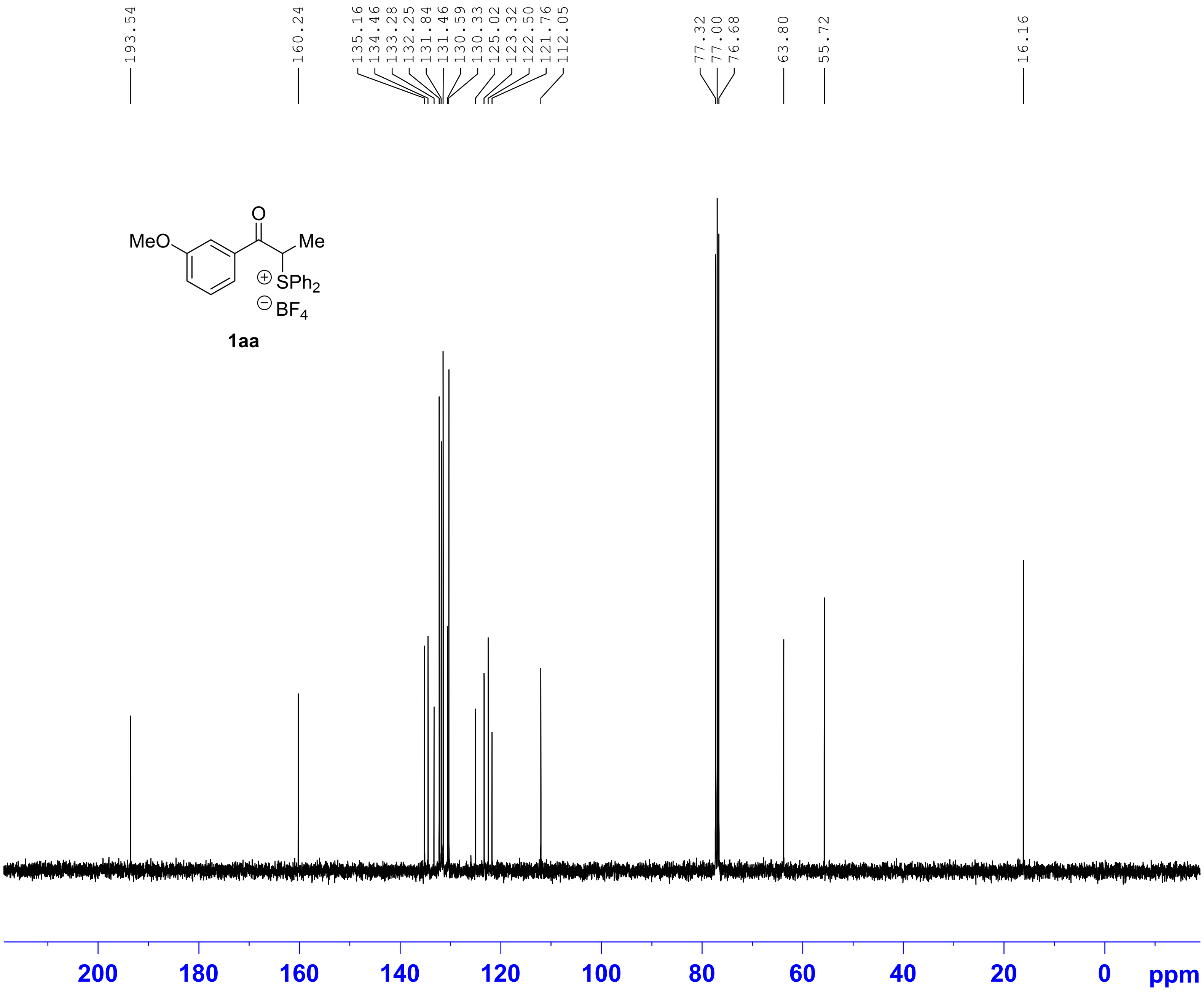

NAME	lzy-11-42-m-OMe-SM-C
EXPNO	1
PROCNO	1
Date_	20230317
Time	10.08
INSTRUM	spect
PROBHD	5 mm PABBO BB/
PULPROG	zgpg30
TD	65536
SOLVENT	CDCl3
NS	100
DS	2
SWH	24038.461 Hz
FIDRES	0.366798 Hz
AQ	1.3631988 sec
RG	196.92
DW	20.800 usec
DE	6.50 usec
TE	296.4 K
D1	2.00000000 sec
D11	0.03000000 sec
TD0	1

======== CHANNEL f1 ========

SFO1	100.6228298 MHz
NUC1	13C
P1	9.70 usec
SI	32768
SF	100.6127801 MHz
WDW	EM
SSB	0
LB	1.00 Hz
GB	0
PC	1.40

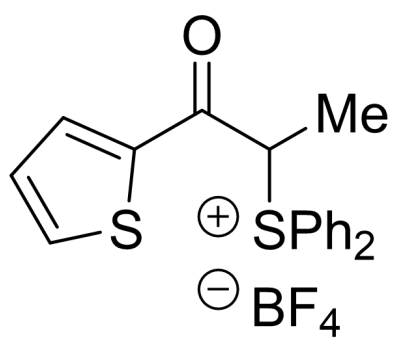

1ab

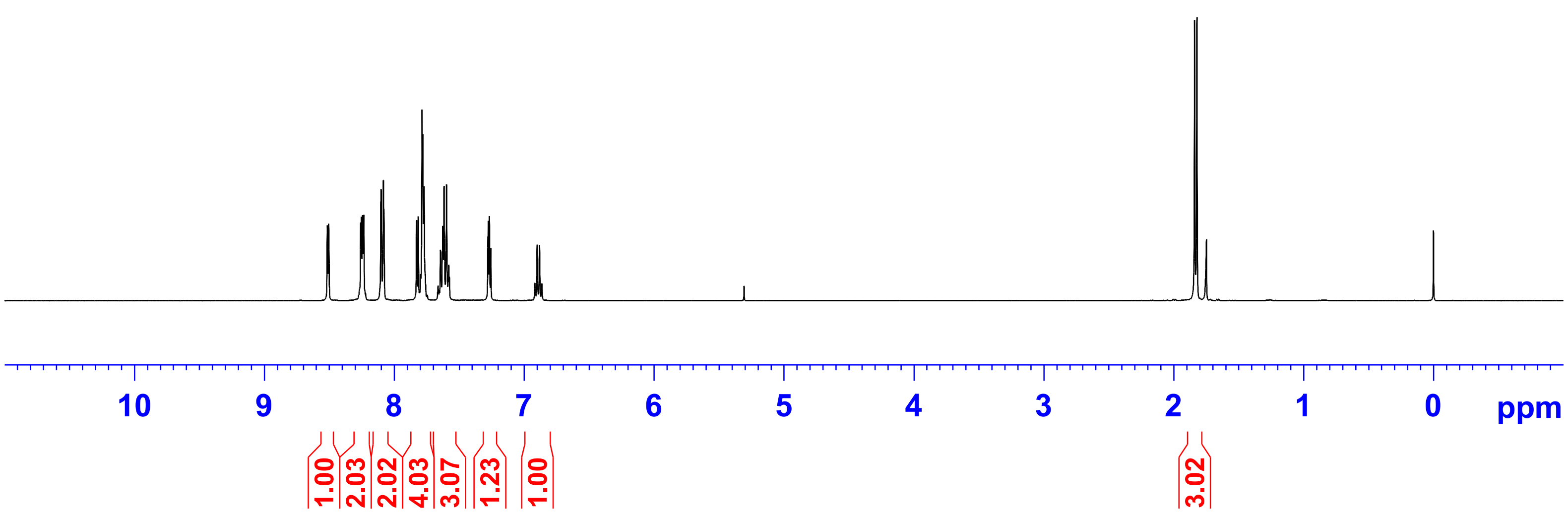

NAME HNMR-gwg-3-134-1
EXPNO 1
PROCNO 1
Date_ 20190907
Time_ 16.35
INSTRUM spect
PROBHD 5 mm PABBO BB-
PULPROG zg30
TD 65536
SOLVENT CDCl3
NS 4
DS 0
SWH 8223.685 Hz
FIDRES 0.125483 Hz
AQ 3.9846387 sec
RG 322
DW 60.800 usec
DE 6.00 usec
TE 294.1 K
D1 1.00000000 sec
TD0 1

======== CHANNEL f1 ========
NUC1 1H
P1 15.80 usec
PL1 -1.00 dB
PL1W 12.17476940 W
SFO1 400.1324710 MHz
SI 32768
SF 400.1300033 MHz
WDW EM
SSB 0
LB 0.30 Hz
GB 0
PC 1.00

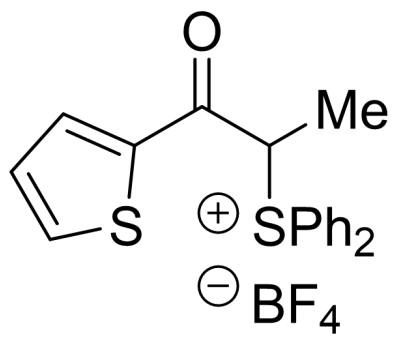

1ab

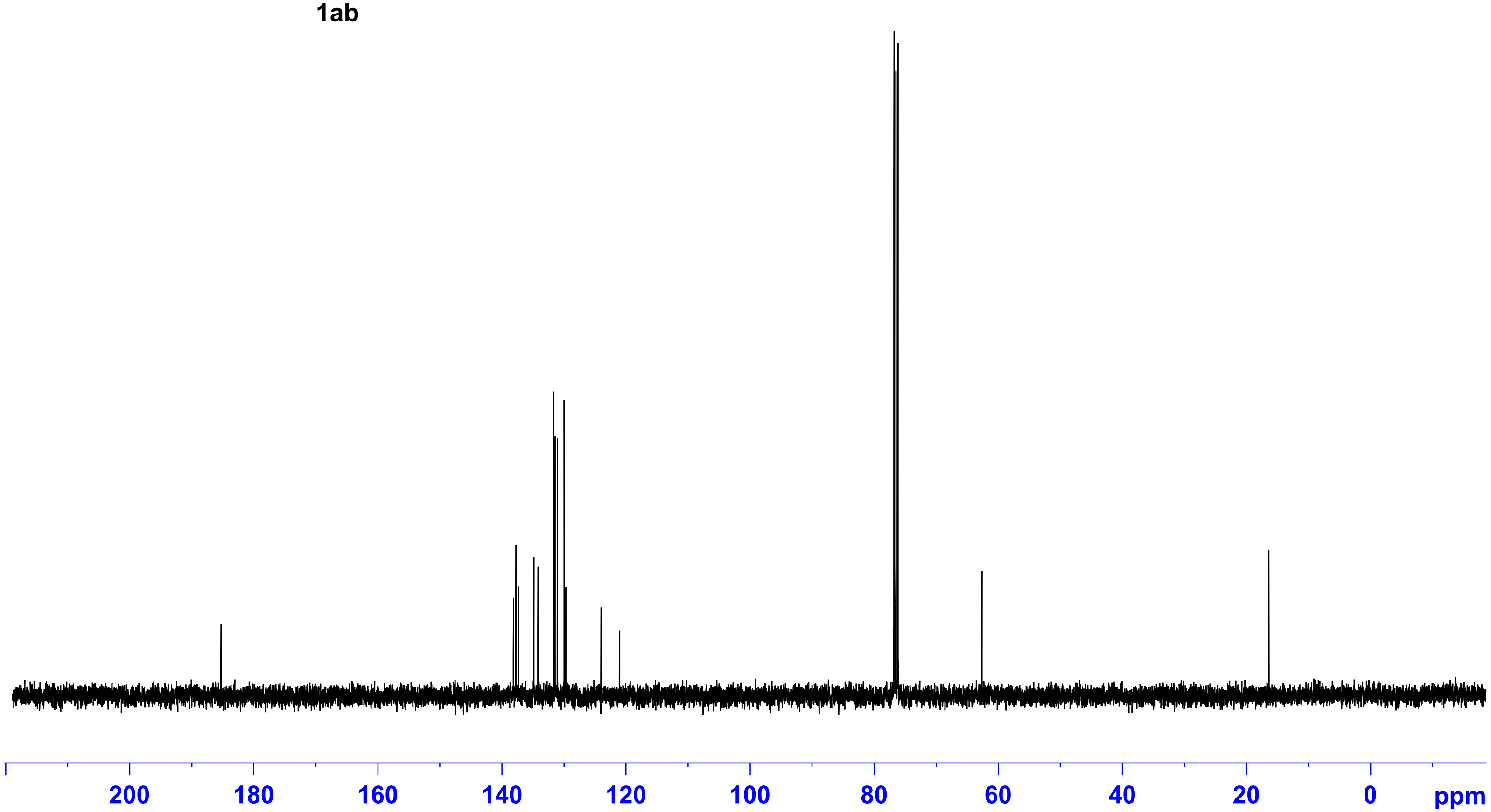

NAME	CNMR-gwg-3-134-1
EXPNO	1
PROCNO	1
Date_	20190907
Time	16.43
INSTRUM	spect
PROBHD	5 mm PABBO BB-
PULPROG	zgpg30
TD	65536
SOLVENT	CDCl3
NS	62
DS	0
SWH	24038.461 Hz
FIDRES	0.366798 Hz
AQ	1.3631988 sec
RG	144
DW	20.800 usec
DE	6.00 usec
TE	294.6 K
D1	2.00000000 sec
D11	0.03000000 sec
TD0	1
======== CHANNEL f1 ========	
NUC1	13C
P1	8.60 usec
PL1	-3.00 dB
PL1W	60.64365387 W
SFO1	100.6228298 MHz
======== CHANNEL f2 ========	
CPDPRG2	waltz16
NUC2	1H
PCPD2	80.00 usec
PL2	-1.00 dB
PL12	14.39 dB
PL13	18.00 dB
PL2W	12.17476940 W
PL12W	0.35193357 W
PL13W	0.15327126 W
SFO2	400.1316005 MHz
SI	32768
SF	100.6128330 MHz
WDW	EM
SSB	0
LB	1.00 Hz
GB	0
PC	1.40

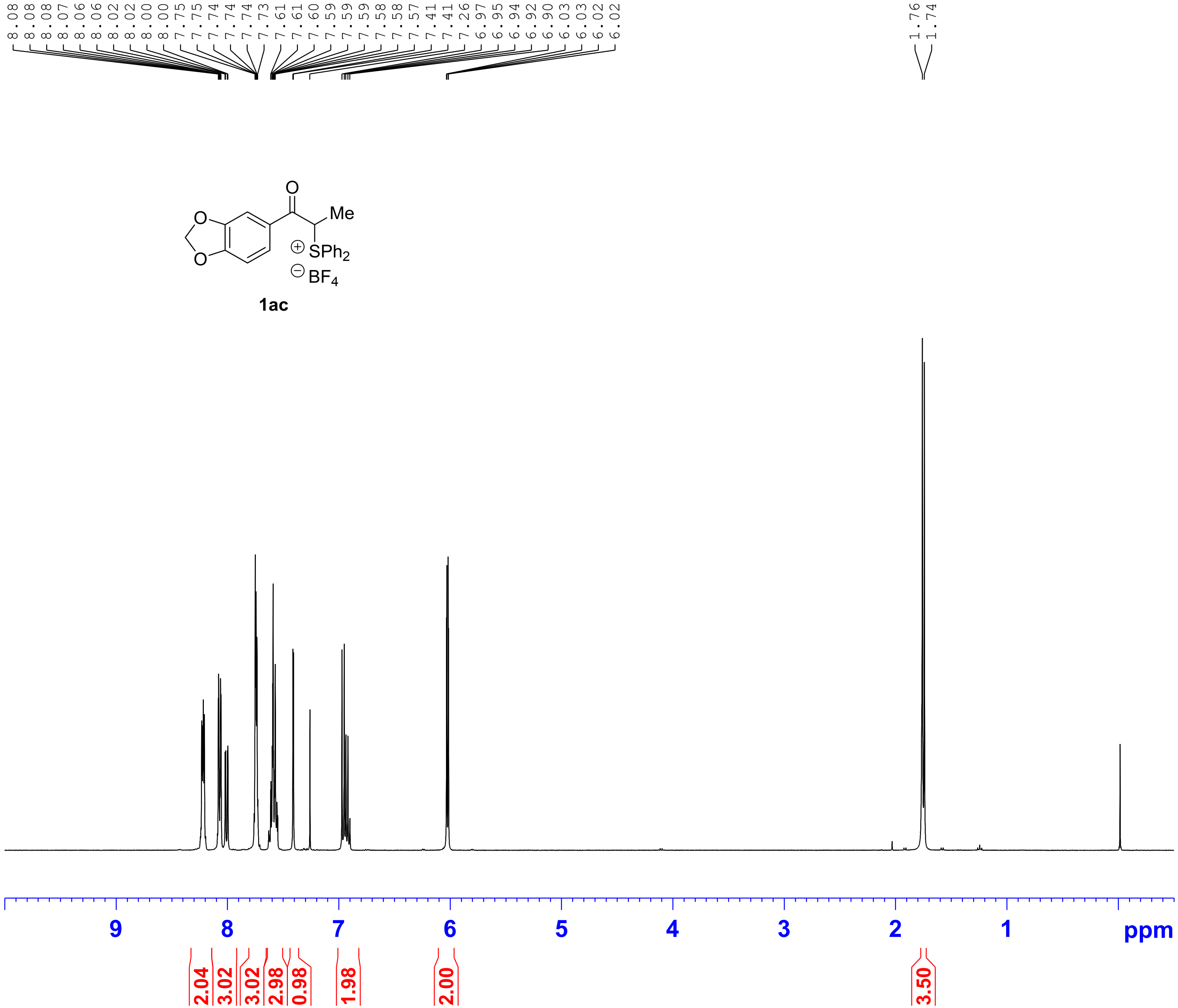

1ac

NAME	lzy-12-175-C-SM
EXPNO	1
PROCNO	1
Date_	20230322
Time	9.03
INSTRUM	spect
PROBHD	5 mm DUL 13C-1
PULPROG	zg30
TD	65536
SOLVENT	CDCl3
NS	4
DS	0
SWH	8223.685 Hz
FIDRES	0.125483 Hz
AQ	3.9846387 sec
RG	322
DW	60.800 usec
DE	6.00 usec
TE	294.5 K
D1	1.00000000 sec
TD0	1
======== CHANNEL f1 ========	
NUC1	1H
P1	15.80 usec
PL1	-1.00 dB
PL1W	12.17476940 W
SFO1	400.1324710 MHz
SI	32768
SF	400.1300095 MHz
WDW	EM
SSB	0
LB	0.30 Hz
GB	0
PC	1.00

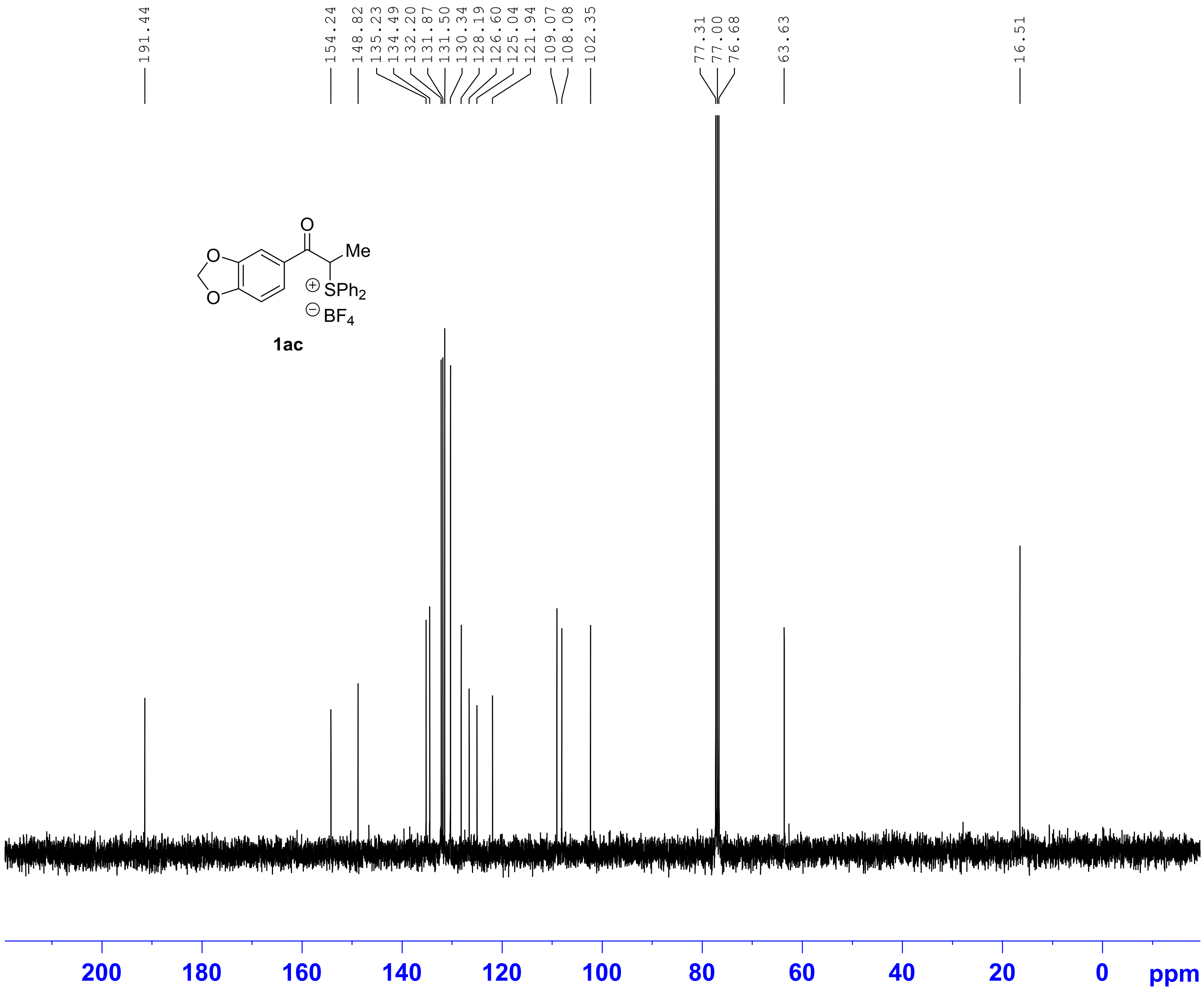


```
NAME       lzy-12-175-C-SM-C
EXPNO                      1
PROCNO                     1
Date_               20230322
Time                    9.06
INSTRUM                spect
PROBHD     5 mm DUL 13C-1
PULPROG              zgpg30
TD                     65536
SOLVENT                CDCl3
NS                       241
DS                         0
SWH                24038.461 Hz
FIDRES              0.366798 Hz
AQ                 1.3631988 sec
RG                      2050
DW                    20.800 usec
DE                      6.00 usec
TE                     294.7 K
D1                2.00000000 sec
D11               0.03000000 sec
TD0                        1

======== CHANNEL f1 ========
NUC1                     13C
P1                     40.00 usec
PL1                    -3.00 dB
PL1W             60.64365387 W
SFO1             100.6228298 MHz

======== CHANNEL f2 ========
CPDPRG2              waltz16
NUC2                      1H
PCPD2                  80.00 usec
PL2                    -1.00 dB
PL12                   14.39 dB
PL13                   18.00 dB
PL2W             12.17476940 W
PL12W             0.35193357 W
PL13W             0.15327126 W
SFO2             400.1316005 MHz
SI                     32768
SF               100.6127757 MHz
WDW                       EM
SSB                        0
LB                      1.00 Hz
GB                         0
PC                      1.40
```

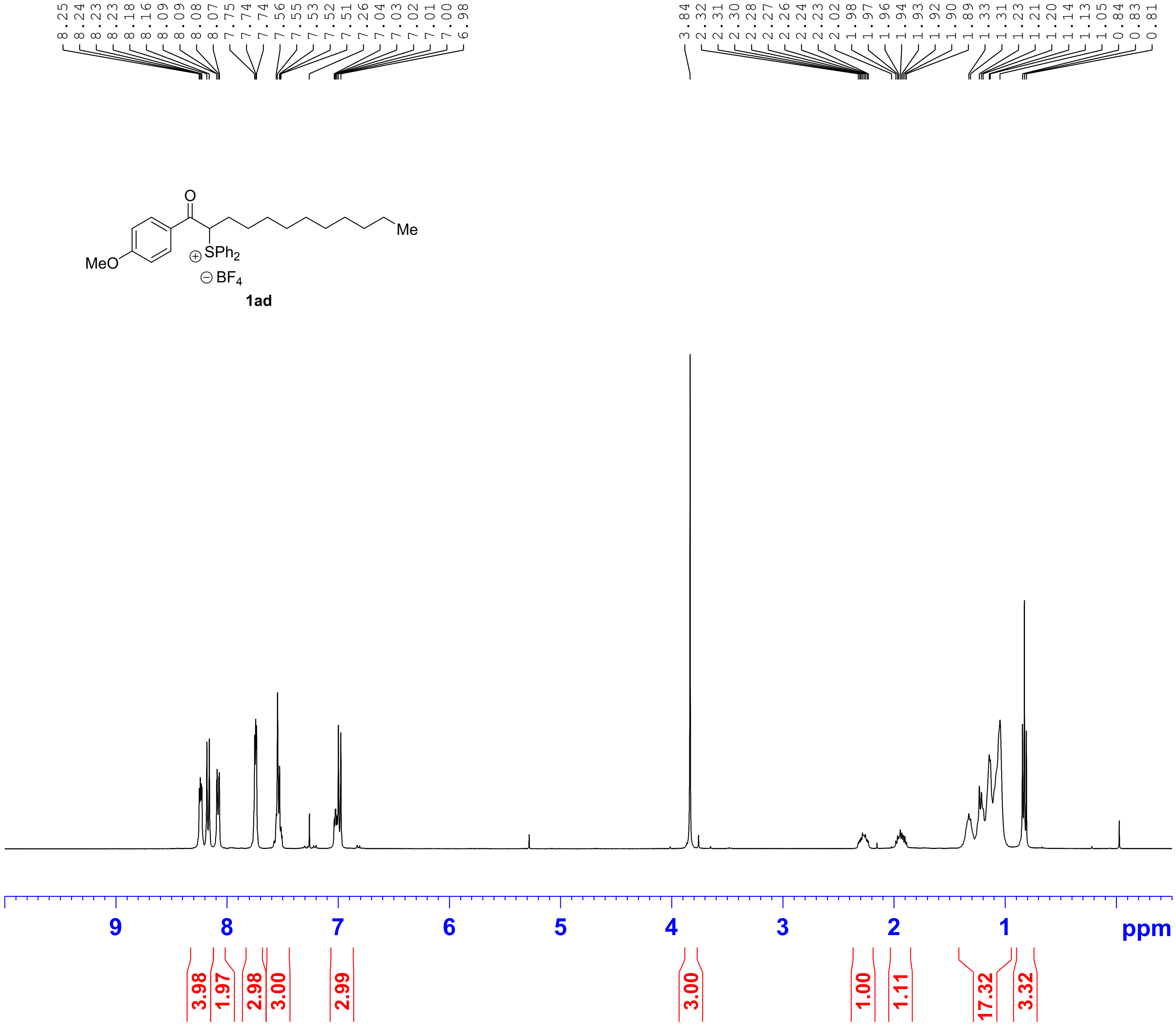

NAME	lzy-12-165-F	
EXPNO	1	
PROCNO	1	
Date_	20230222	
Time	19.41	
INSTRUM	spect	
PROBHD	5 mm PABBO BB/	
PULPROG	zg30	
TD	65536	
SOLVENT	CDCl3	
NS	5	
DS	2	
SWH	8012.820	Hz
FIDRES	0.122266	Hz
AQ	4.0894966	sec
RG	45.67	
DW	62.400	usec
DE	6.50	usec
TE	293.6	K
D1	1.00000000	sec
TD0	1	

======== CHANNEL f1 ========

SFO1	400.1324710	MHz
NUC1	1H	
P1	14.50	usec
SI	65536	
SF	400.1300095	MHz
WDW	EM	
SSB	0	
LB	0.30	Hz
GB	0	
PC	1.00	

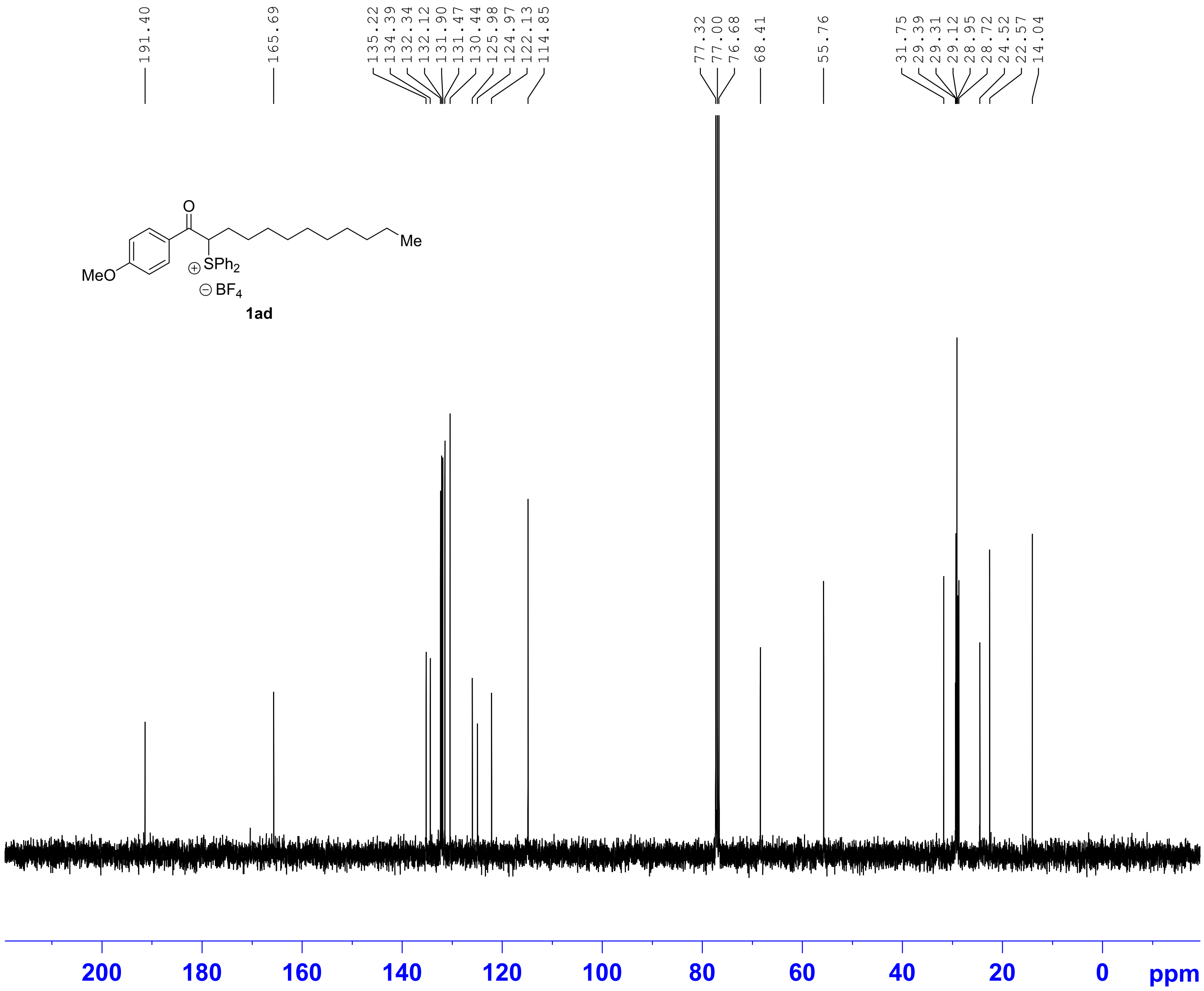


```
NAME        lzy-12-165-E-C
EXPNO                    1
PROCNO                   1
Date_             20230222
Time                  8.39
INSTRUM              spect
PROBHD     5 mm DUL 13C-1
PULPROG            zgpg30
TD                  65536
SOLVENT              CDCl3
NS                     380
DS                       0
SWH             24038.461 Hz
FIDRES           0.366798 Hz
AQ              1.3631988 sec
RG                    2050
DW                  20.800 usec
DE                    6.00 usec
TE                   295.0 K
D1              2.00000000 sec
D11             0.03000000 sec
TD0                      1

======== CHANNEL f1 ========
NUC1                   13C
P1                   40.00 usec
PL1                  -3.00 dB
PL1W           60.64365387 W
SFO1           100.6228298 MHz

======== CHANNEL f2 ========
CPDPRG2            waltz16
NUC2                    1H
PCPD2                80.00 usec
PL2                  -1.00 dB
PL12                 14.39 dB
PL13                 18.00 dB
PL2W           12.17476940 W
PL12W           0.35193357 W
PL13W           0.15327126 W
SFO2           400.1316005 MHz
SI                   32768
SF             100.6127743 MHz
WDW                     EM
SSB                      0
LB                    1.00 Hz
GB                       0
PC                    1.40
```

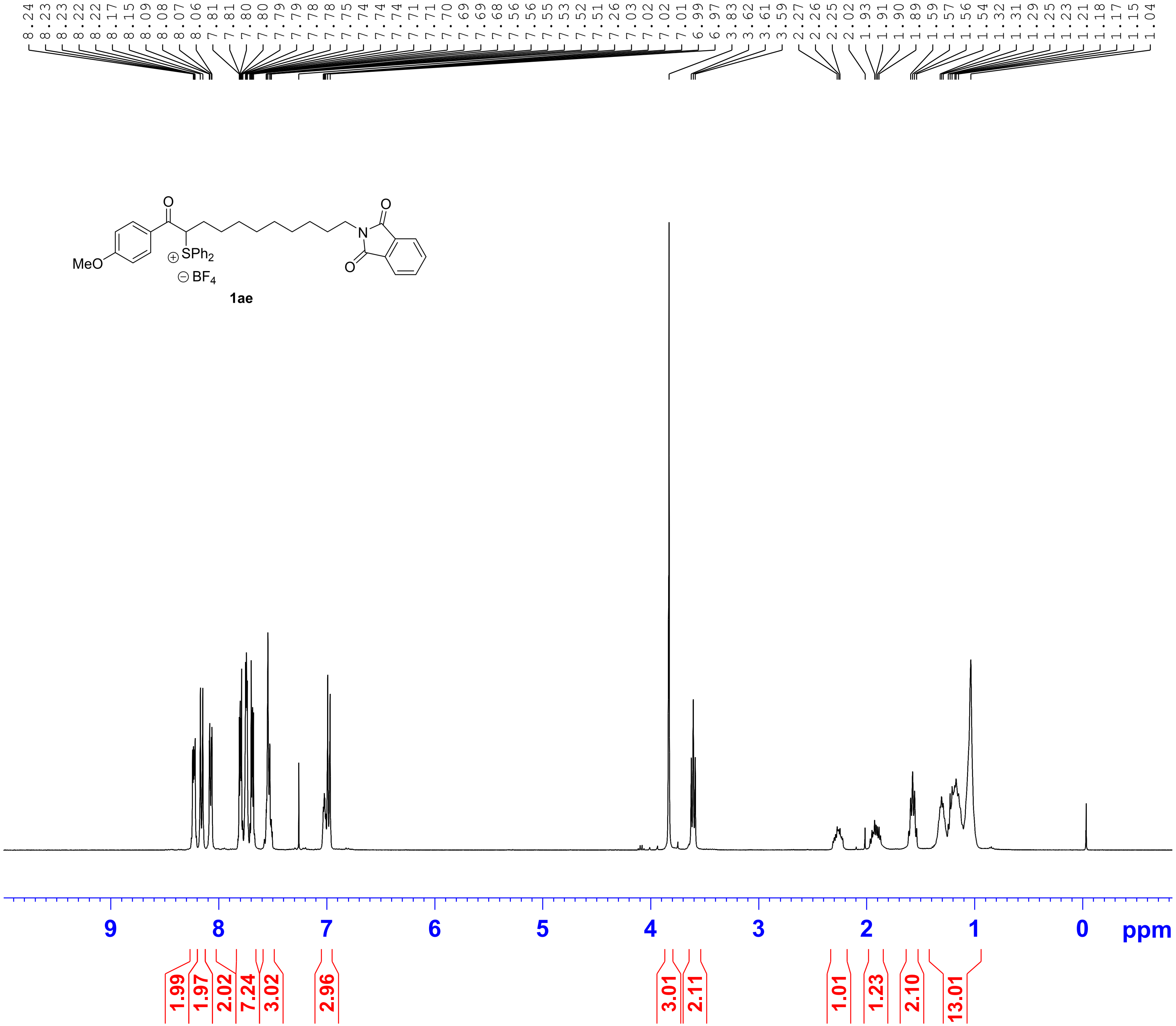

NAME lzy-13-7
EXPNO 1
PROCNO 1
Date_ 20230314
Time_ 20.05
INSTRUM spect
PROBHD 5 mm PABBO BB/
PULPROG zg30
TD 65536
SOLVENT CDCl3
NS 4
DS 2
SWH 8012.820 Hz
FIDRES 0.122266 Hz
AQ 4.0894966 sec
RG 27.78
DW 62.400 usec
DE 6.50 usec
TE 295.1 K
D1 1.00000000 sec
TD0 1

======== CHANNEL f1 ========
SFO1 400.1324710 MHz
NUC1 1H
P1 14.50 usec
SI 65536
SF 400.1300098 MHz
WDW EM
SSB 0
LB 0.30 Hz
GB 0
PC 1.00

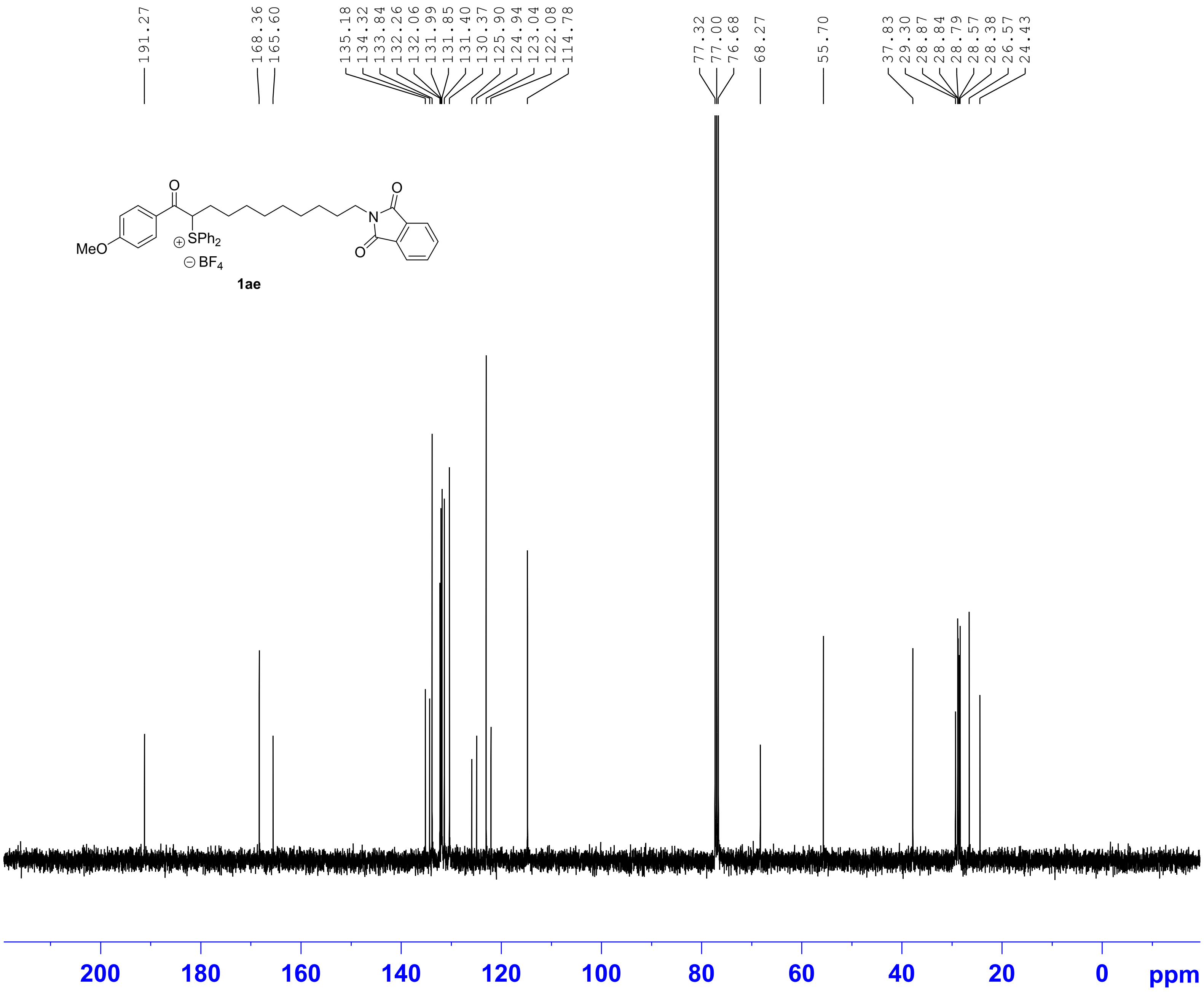

1ae

NAME	lzy-13-7-C
EXPNO	1
PROCNO	1
Date_	20230314
Time	20.54
INSTRUM	spect
PROBHD	5 mm PABBO BB/
PULPROG	zgpg30
TD	65536
SOLVENT	CDCl3
NS	99
DS	2
SWH	24038.461 Hz
FIDRES	0.366798 Hz
AQ	1.3631988 sec
RG	196.92
DW	20.800 usec
DE	6.50 usec
TE	295.8 K
D1	2.00000000 sec
D11	0.03000000 sec
TD0	1

======== CHANNEL f1 ========

SFO1	100.6228298 MHz
NUC1	13C
P1	9.70 usec
SI	32768
SF	100.6127794 MHz
WDW	EM
SSB	0
LB	1.00 Hz
GB	0
PC	1.40

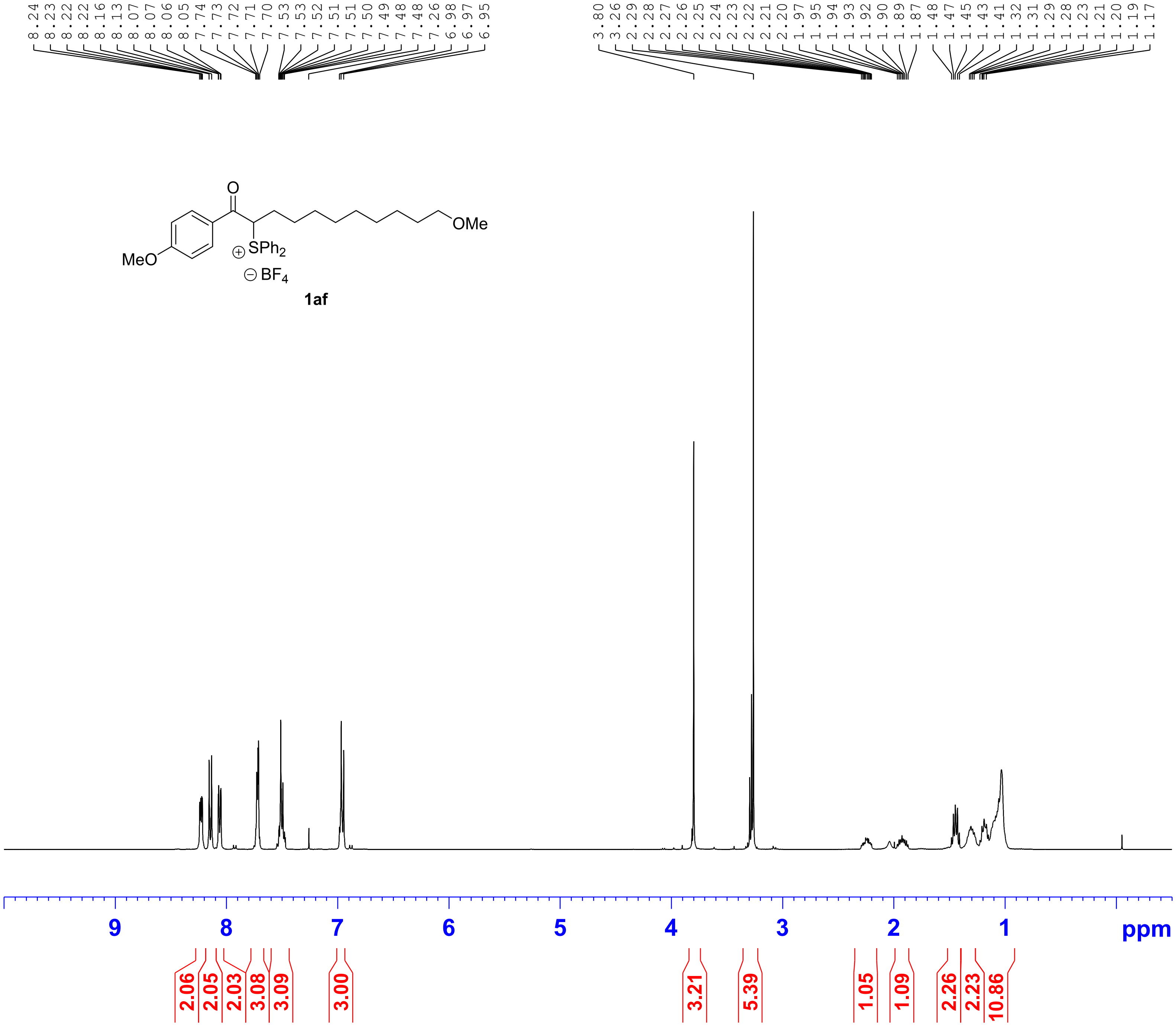

NAME	lzy-12-65-B-SM
EXPNO	1
PROCNO	1
Date_	20230321
Time_	10.19
INSTRUM	spect
PROBHD	5 mm PABBO BB/
PULPROG	zg30
TD	65536
SOLVENT	CDCl3
NS	4
DS	2
SWH	8012.820 Hz
FIDRES	0.122266 Hz
AQ	4.0894966 sec
RG	19.7
DW	62.400 usec
DE	6.50 usec
TE	296.6 K
D1	1.00000000 sec
TD0	1

======== CHANNEL f1 ========

SFO1	400.1324710 MHz
NUC1	1H
P1	14.50 usec
SI	65536
SF	400.1300097 MHz
WDW	EM
SSB	0
LB	0.30 Hz
GB	0
PC	1.00

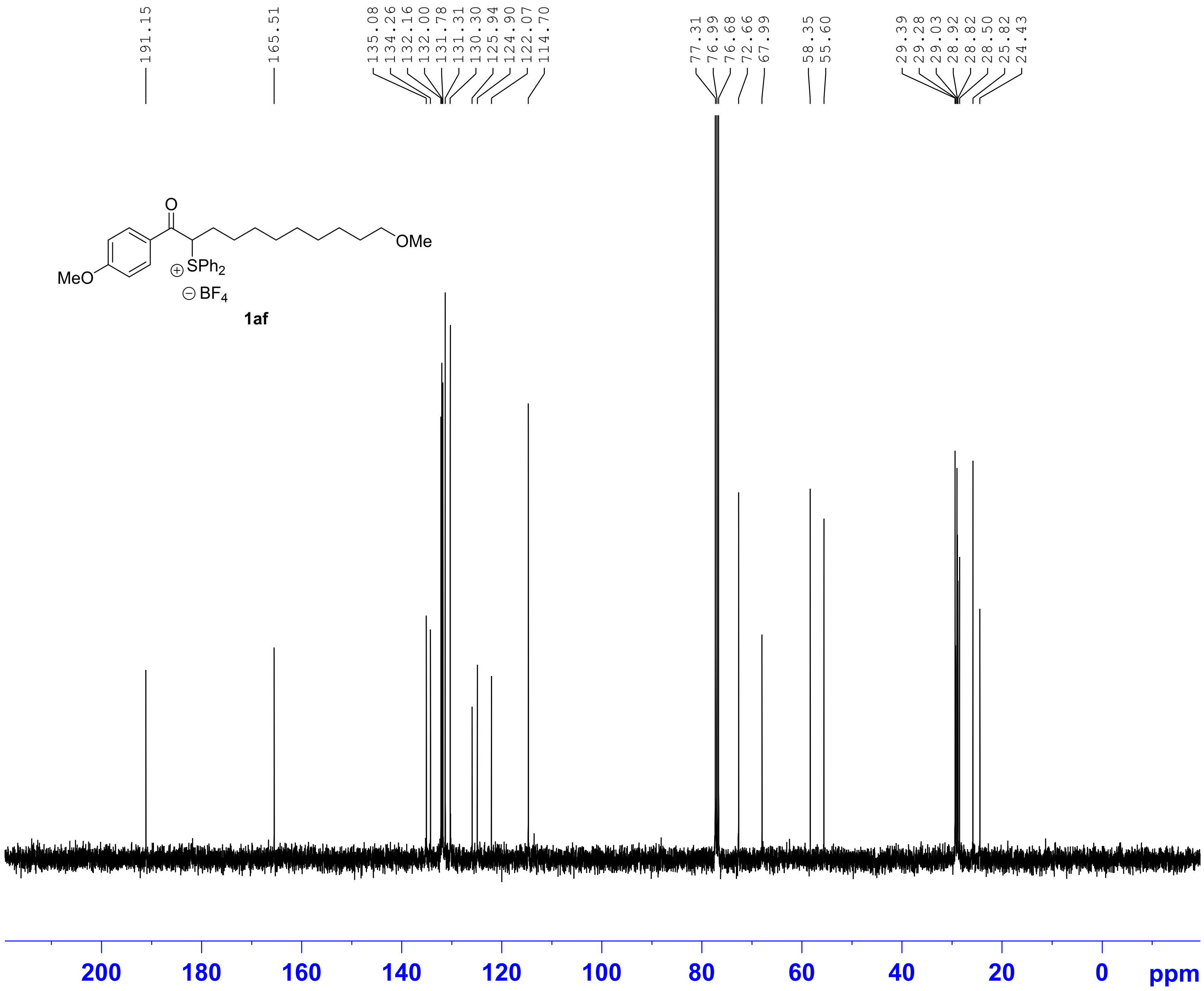

1af

NAME	lzy-12-65-B-SM-C
EXPNO	1
PROCNO	1
Date_	20230321
Time	10.20
INSTRUM	spect
PROBHD	5 mm PABBO BB/
PULPROG	zgpg30
TD	65536
SOLVENT	CDCl3
NS	65
DS	2
SWH	24038.461 Hz
FIDRES	0.366798 Hz
AQ	1.3631988 sec
RG	196.92
DW	20.800 usec
DE	6.50 usec
TE	296.8 K
D1	2.00000000 sec
D11	0.03000000 sec
TD0	1

======== CHANNEL f1 ========

SFO1	100.6228298 MHz
NUC1	13C
P1	9.70 usec
SI	32768
SF	100.6127838 MHz
WDW	EM
SSB	0
LB	1.00 Hz
GB	0
PC	1.40

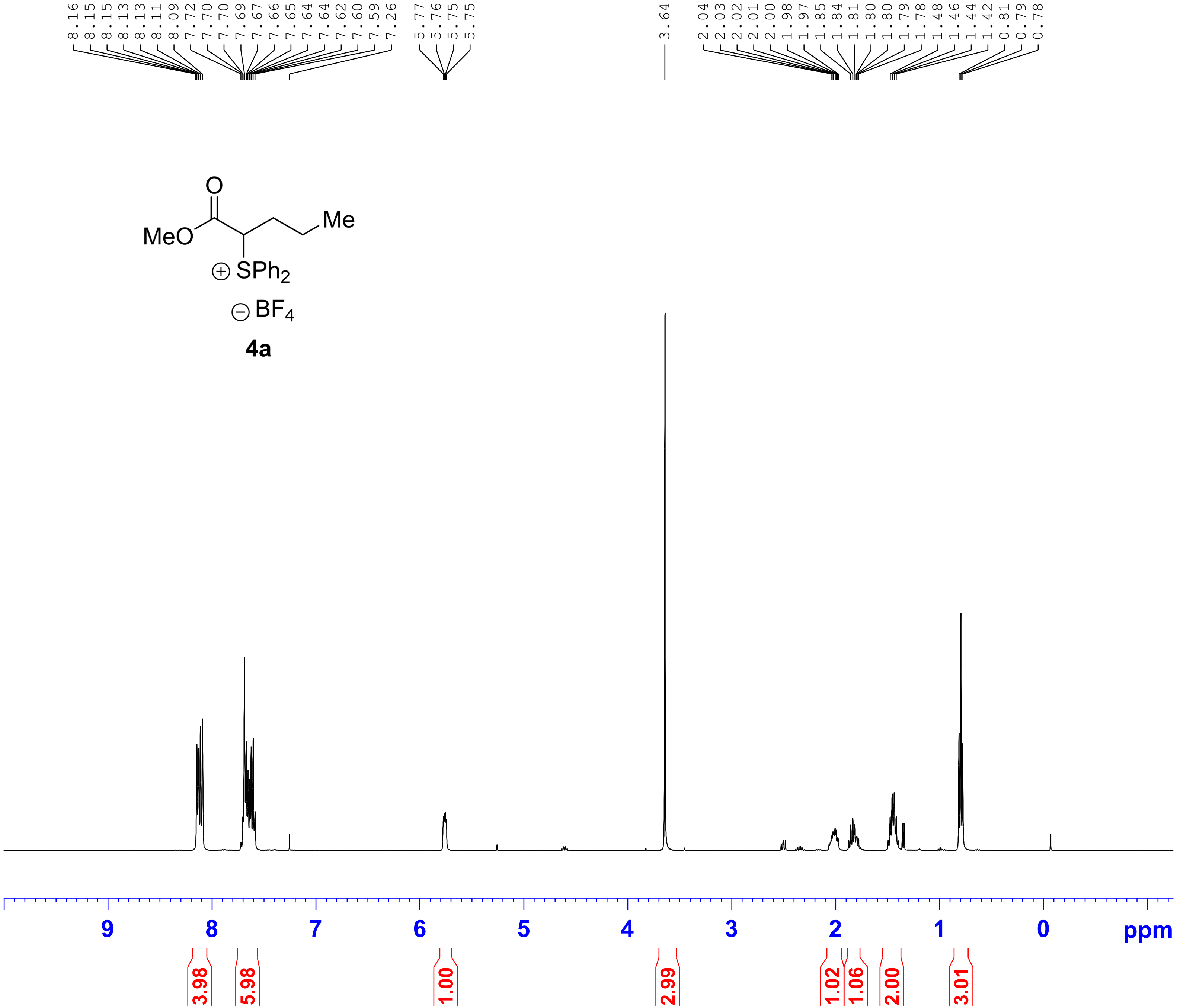

NAME	lzy-08-OMe-SM	
EXPNO	1	
PROCNO	1	
Date_	20220301	
Time	12.07	
INSTRUM	spect	
PROBHD	5 mm DUL 13C-1	
PULPROG	zg30	
TD	65536	
SOLVENT	CDCl3	
NS	4	
DS	0	
SWH	8223.685	Hz
FIDRES	0.125483	Hz
AQ	3.9846387	sec
RG	114	
DW	60.800	usec
DE	6.00	usec
TE	293.9	K
D1	1.00000000	sec
TD0	1	

======== CHANNEL f1 ========

NUC1	1H	
P1	15.80	usec
PL1	-1.00	dB
PL1W	12.17476940	W
SFO1	400.1324710	MHz
SI	32768	
SF	400.1300111	MHz
WDW	EM	
SSB	0	
LB	0.30	Hz
GB	0	
PC	1.00	

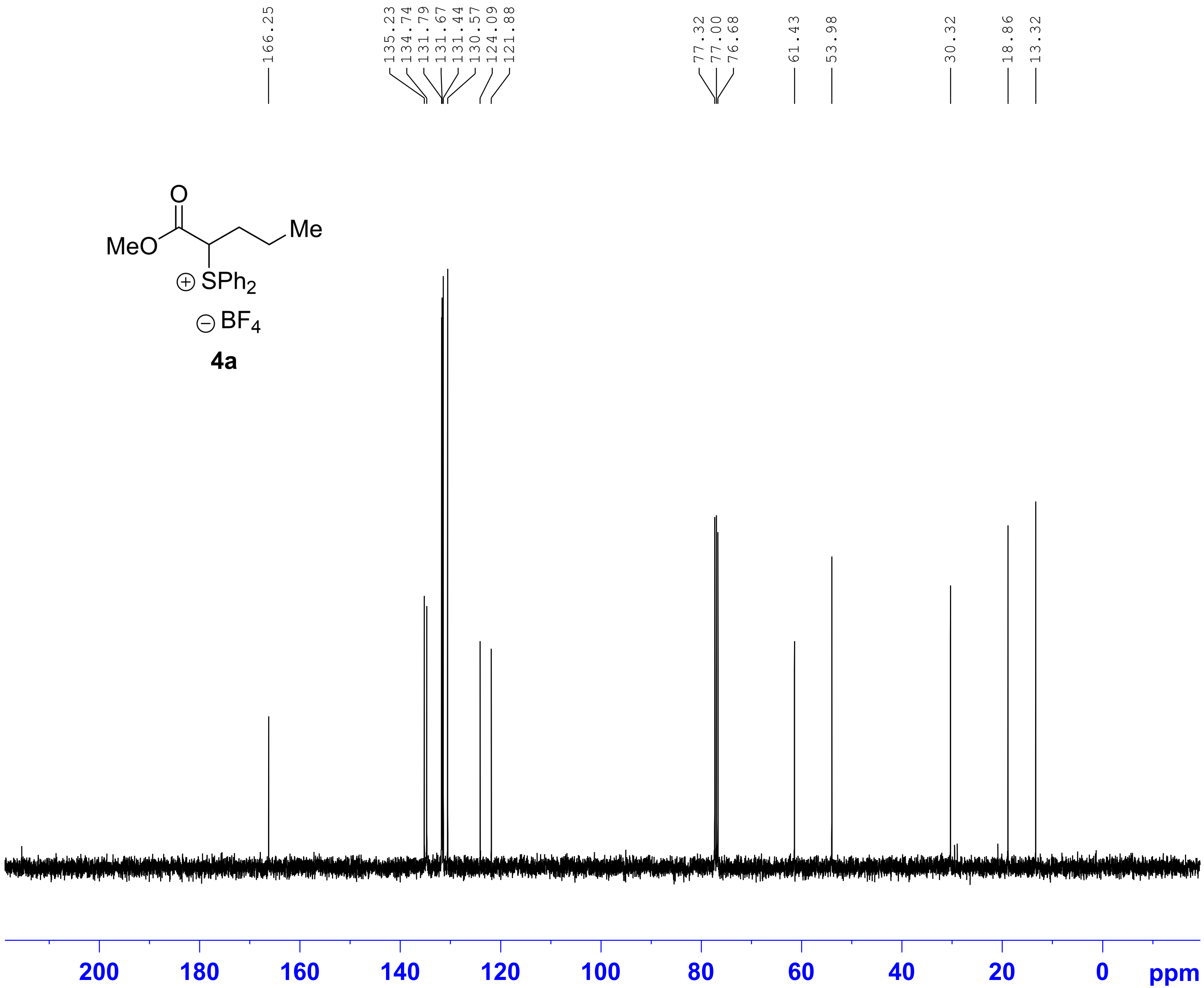

NAME	lzy-08-OMe-SM-C	
EXPNO	1	
PROCNO	1	
Date_	20220301	
Time	12.09	
INSTRUM	spect	
PROBHD	5 mm DUL 13C-1	
PULPROG	zgpg30	
TD	65536	
SOLVENT	CDCl3	
NS	93	
DS	0	
SWH	24038.461	Hz
FIDRES	0.366798	Hz
AQ	1.3631988	sec
RG	2050	
DW	20.800	usec
DE	6.00	usec
TE	294.0	K
D1	2.00000000	sec
D11	0.03000000	sec
TD0	1	

======== CHANNEL f1 ========

NUC1	13C	
P1	40.00	usec
PL1	-3.00	dB
PL1W	60.64365387	W
SFO1	100.6228298	MHz

======== CHANNEL f2 ========

CPDPRG2	waltz16	
NUC2	1H	
PCPD2	80.00	usec
PL2	-1.00	dB
PL12	14.39	dB
PL13	18.00	dB
PL2W	12.17476940	W
PL12W	0.35193357	W
PL13W	0.15327126	W
SFO2	400.1316005	MHz
SI	32768	
SF	100.6127863	MHz
WDW	EM	
SSB	0	
LB	1.00	Hz
GB	0	
PC	1.40	

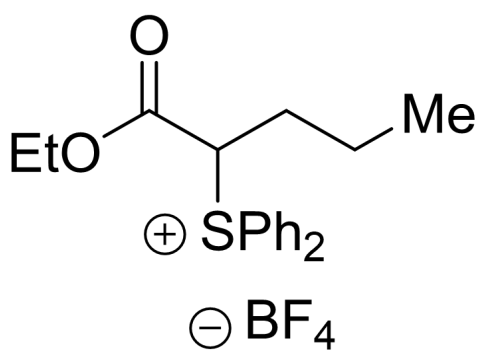

4b

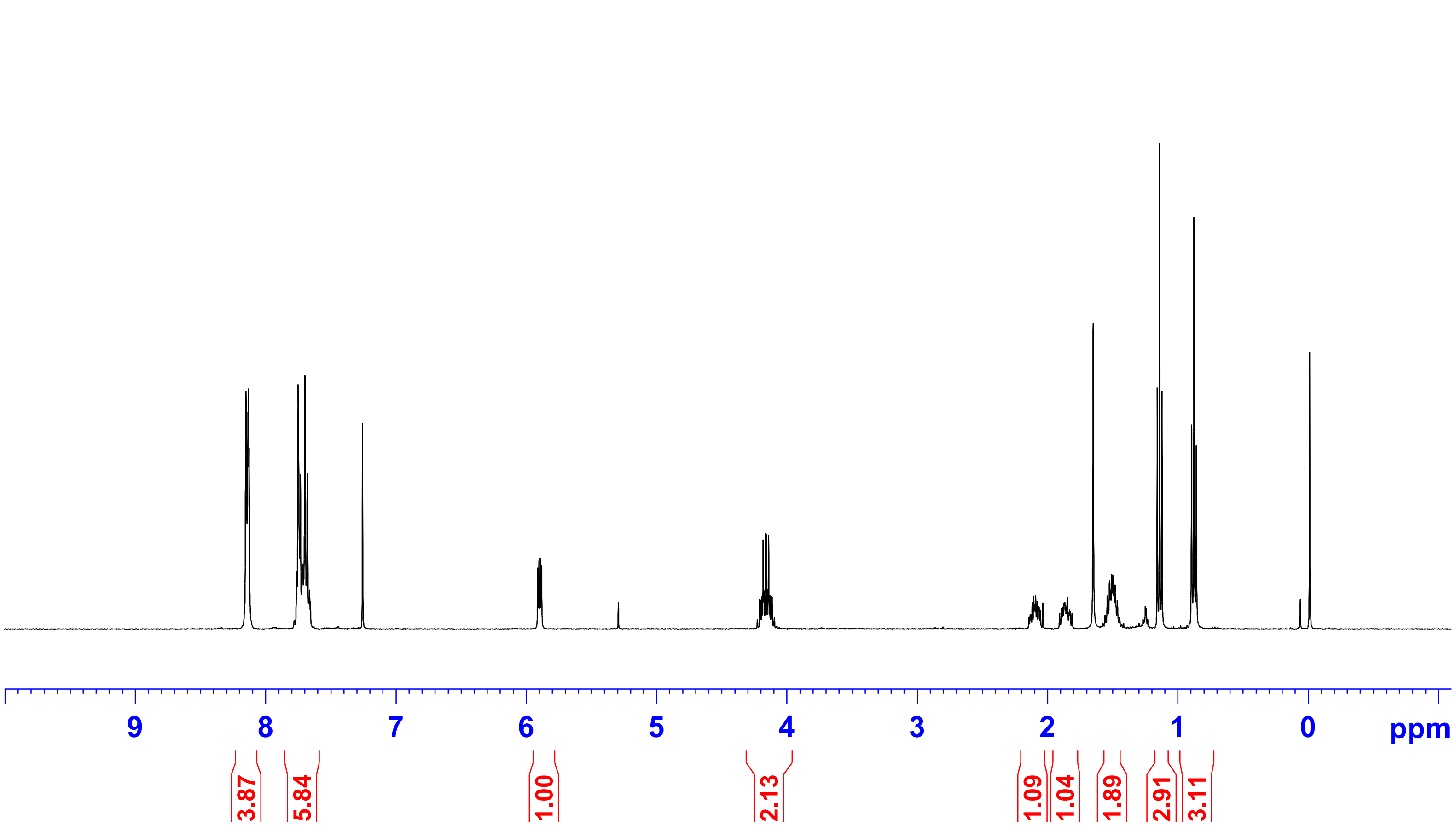

NAME	lzy-08-Et-SM	
EXPNO	1	
PROCNO	1	
Date_	20220301	
Time	10.47	
INSTRUM	spect	
PROBHD	5 mm DUL 13C-1	
PULPROG	zg30	
TD	65536	
SOLVENT	CDCl3	
NS	3	
DS	0	
SWH	8223.685	Hz
FIDRES	0.125483	Hz
AQ	3.9846387	sec
RG	512	
DW	60.800	usec
DE	6.00	usec
TE	294.1	K
D1	1.00000000	sec
TD0	1	

======== CHANNEL f1 ========

NUC1	1H	
P1	15.80	usec
PL1	-1.00	dB
PL1W	12.17476940	W
SFO1	400.1324710	MHz
SI	32768	
SF	400.1300108	MHz
WDW	EM	
SSB	0	
LB	0.30	Hz
GB	0	
PC	1.00	

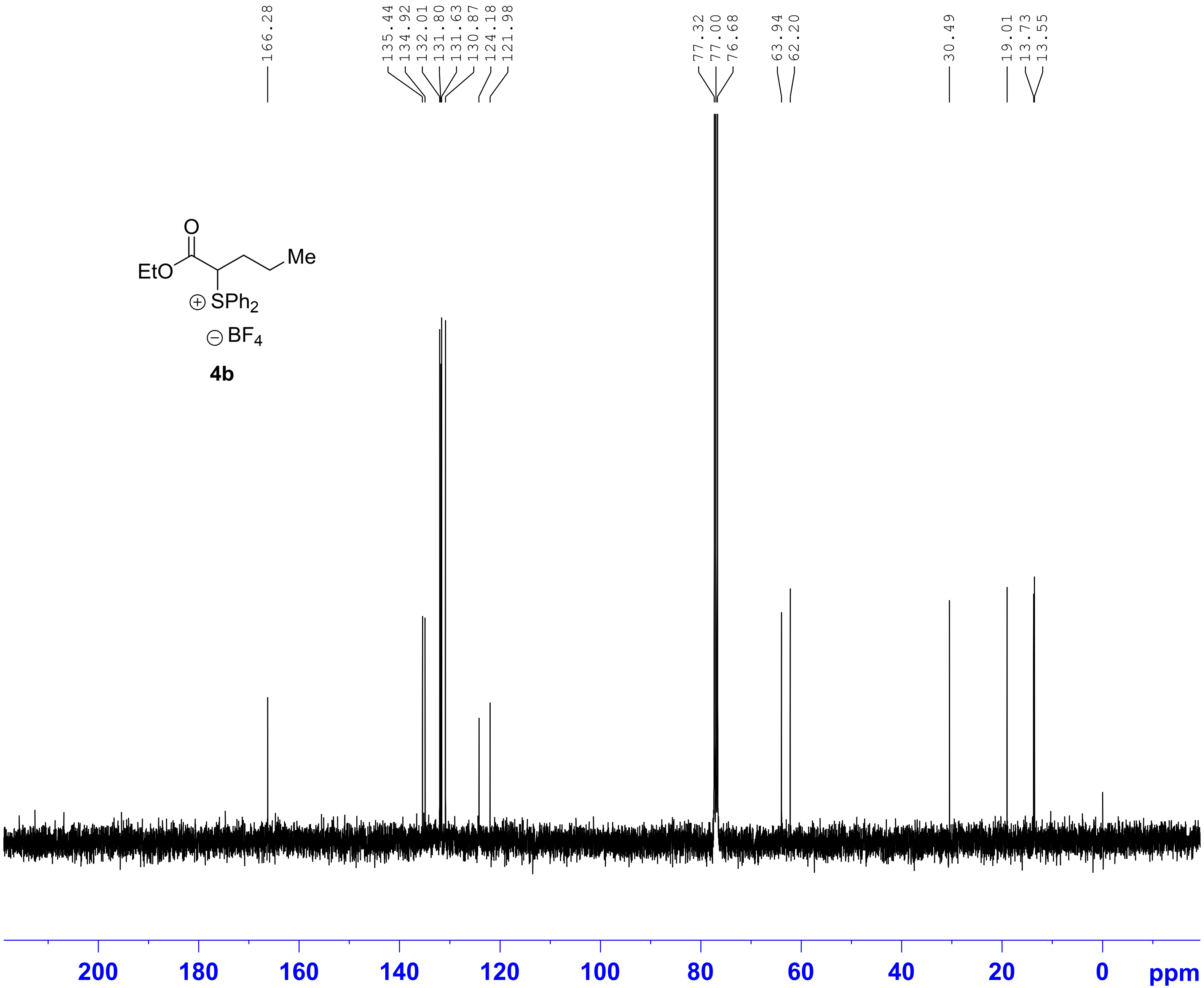

NAME	lzy-08-Et-SM-C	
EXPNO	3	
PROCNO	1	
Date_	20220301	
Time	12.33	
INSTRUM	spect	
PROBHD	5 mm DUL 13C-1	
PULPROG	zgpg30	
TD	65536	
SOLVENT	CDCl3	
NS	1321	
DS	0	
SWH	24038.461	Hz
FIDRES	0.366798	Hz
AQ	1.3631988	sec
RG	2050	
DW	20.800	usec
DE	6.00	usec
TE	294.1	K
D1	2.00000000	sec
D11	0.03000000	sec
TD0	1	
======== CHANNEL f1 ========		
NUC1	13C	
P1	40.00	usec
PL1	-3.00	dB
PL1W	60.64365387	W
SFO1	100.6228298	MHz
======== CHANNEL f2 ========		
CPDPRG2	waltz16	
NUC2	1H	
PCPD2	80.00	usec
PL2	-1.00	dB
PL12	14.39	dB
PL13	18.00	dB
PL2W	12.17476940	W
PL12W	0.35193357	W
PL13W	0.15327126	W
SFO2	400.1316005	MHz
SI	32768	
SF	100.6127731	MHz
WDW	EM	
SSB	0	
LB	1.00	Hz
GB	0	
PC	1.40	

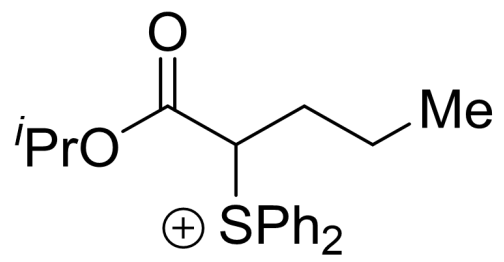

4c

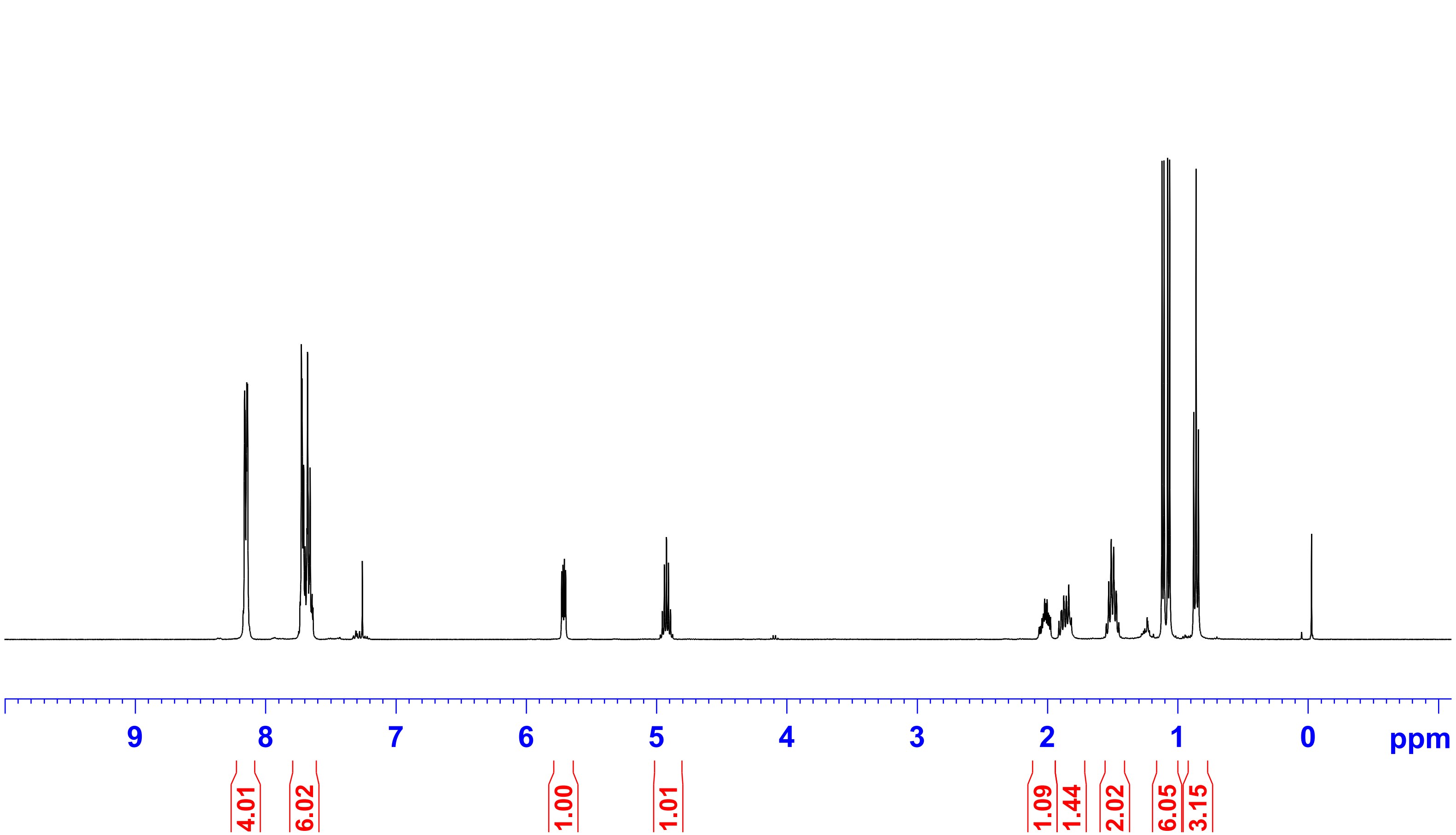

NAME	lzy-08-iPr-SM	
EXPNO	1	
PROCNO	1	
Date_	20220301	
Time	10.34	
INSTRUM	spect	
PROBHD	5 mm DUL 13C-1	
PULPROG	zg30	
TD	65536	
SOLVENT	CDCl3	
NS	2	
DS	0	
SWH	8223.685	Hz
FIDRES	0.125483	Hz
AQ	3.9846387	sec
RG	228	
DW	60.800	usec
DE	6.00	usec
TE	294.1	K
D1	1.00000000	sec
TD0	1	
======== CHANNEL f1 ========		
NUC1	1H	
P1	15.80	usec
PL1	-1.00	dB
PL1W	12.17476940	W
SFO1	400.1324710	MHz
SI	32768	
SF	400.1300101	MHz
WDW	EM	
SSB	0	
LB	0.30	Hz
GB	0	
PC	1.00	

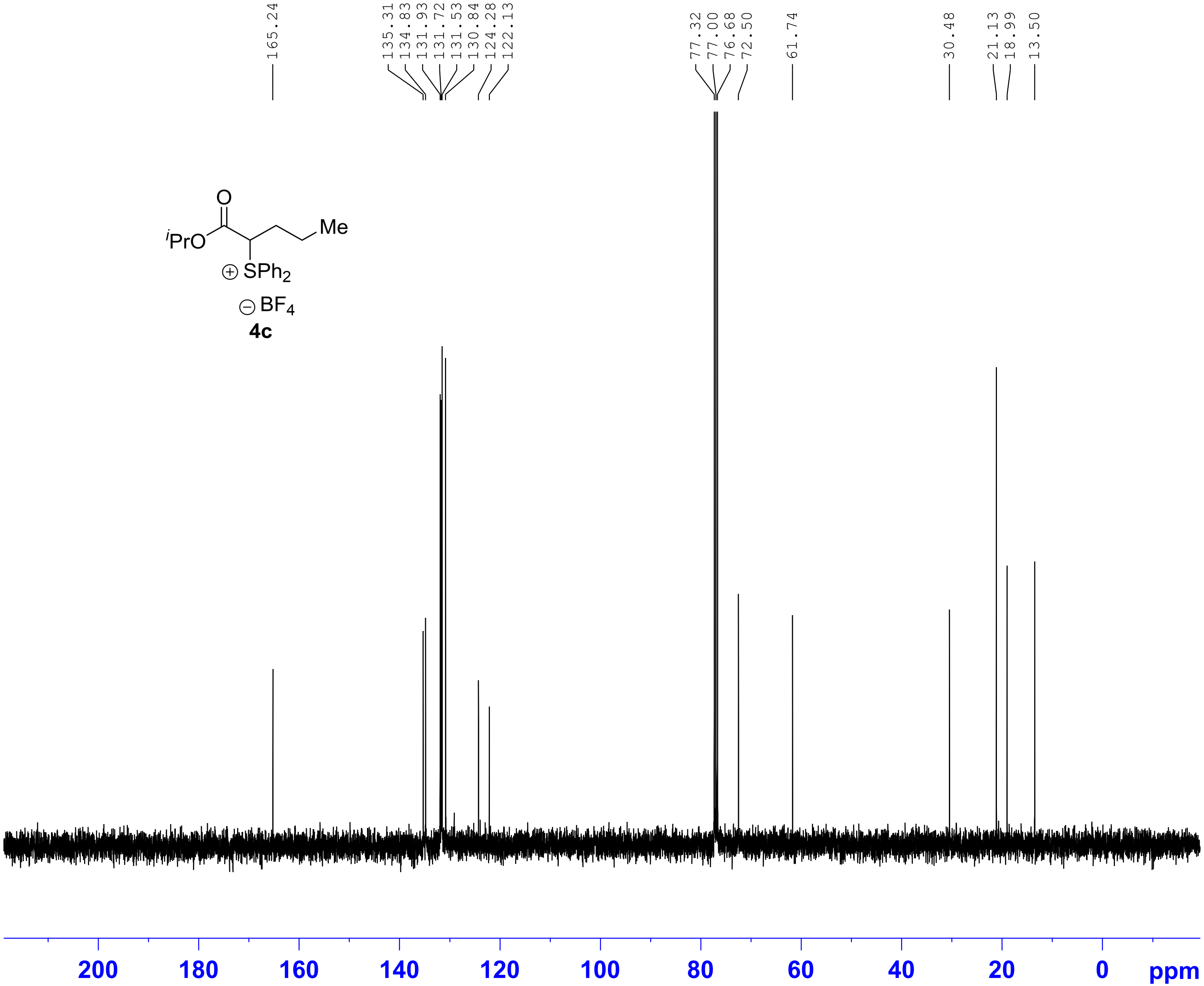

4c

NAME	lzy-08-iPr-SM-C
EXPNO	1
PROCNO	1
Date_	20220301
Time_	10.36
INSTRUM	spect
PROBHD	5 mm DUL 13C-1
PULPROG	zgpg30
TD	65536
SOLVENT	CDCl3
NS	166
DS	0
SWH	24038.461 Hz
FIDRES	0.366798 Hz
AQ	1.3631988 sec
RG	2050
DW	20.800 usec
DE	6.00 usec
TE	294.4 K
D1	2.00000000 sec
D11	0.03000000 sec
TD0	1

======== CHANNEL f1 ========

NUC1	13C
P1	40.00 usec
PL1	-3.00 dB
PL1W	60.64365387 W
SFO1	100.6228298 MHz

======== CHANNEL f2 ========

CPDPRG2	waltz16
NUC2	1H
PCPD2	80.00 usec
PL2	-1.00 dB
PL12	14.39 dB
PL13	18.00 dB
PL2W	12.17476940 W
PL12W	0.35193357 W
PL13W	0.15327126 W
SFO2	400.1316005 MHz
SI	32768
SF	100.6127763 MHz
WDW	EM
SSB	0
LB	1.00 Hz
GB	0
PC	1.40

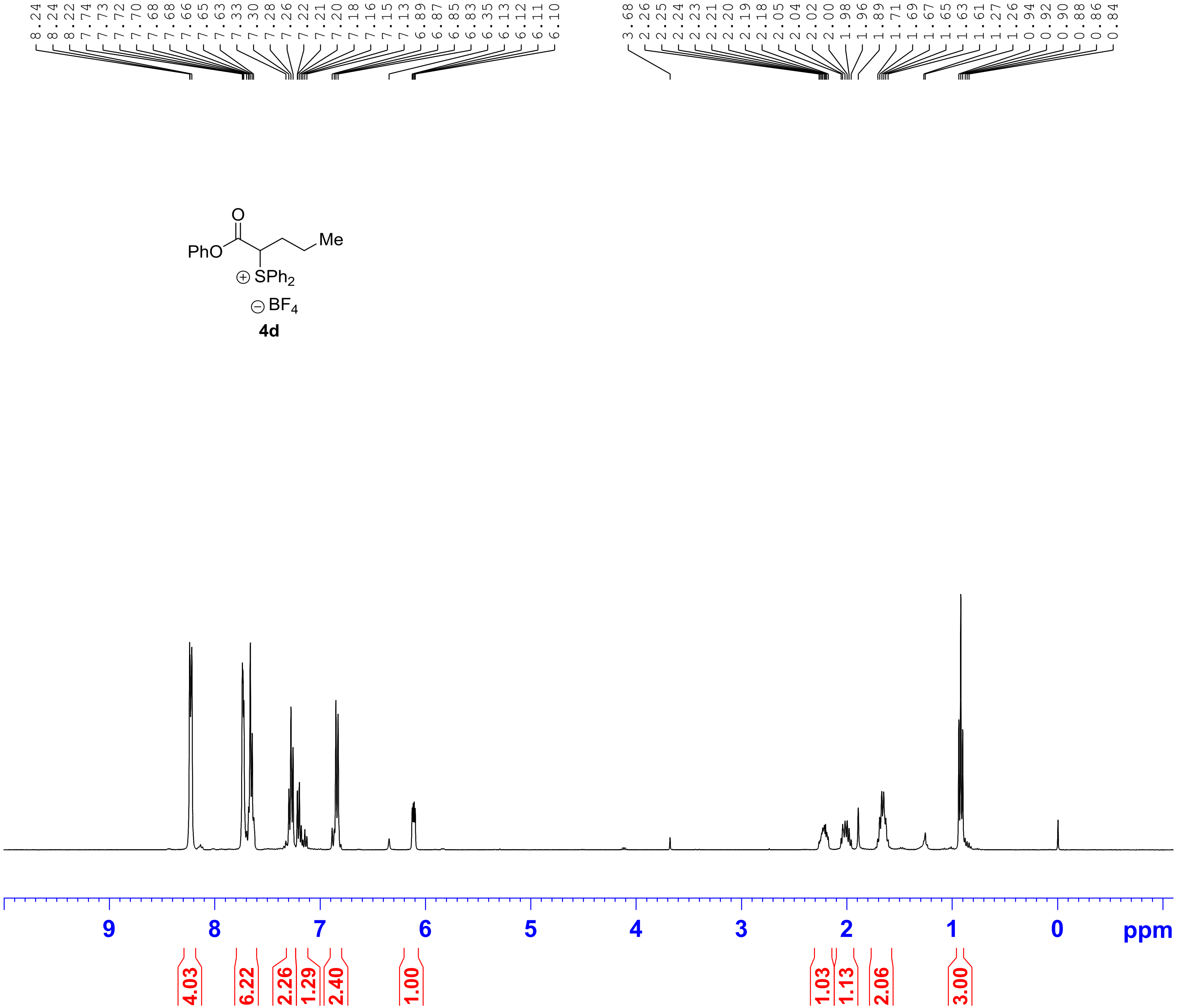

NAME	lzy-08-182-A-SM
EXPNO	2
PROCNO	1
Date_	20220228
Time	20.42
INSTRUM	spect
PROBHD	5 mm DUL 13C-1
PULPROG	zg30
TD	65536
SOLVENT	CDCl3
NS	4
DS	0
SWH	8223.685 Hz
FIDRES	0.125483 Hz
AQ	3.9846387 sec
RG	181
DW	60.800 usec
DE	6.00 usec
TE	293.7 K
D1	1.00000000 sec
TD0	1

======== CHANNEL f1 ========

NUC1	1H
P1	15.80 usec
PL1	-1.00 dB
PL1W	12.17476940 W
SFO1	400.1324710 MHz
SI	32768
SF	400.1300054 MHz
WDW	EM
SSB	0
LB	0.30 Hz
GB	0
PC	1.00

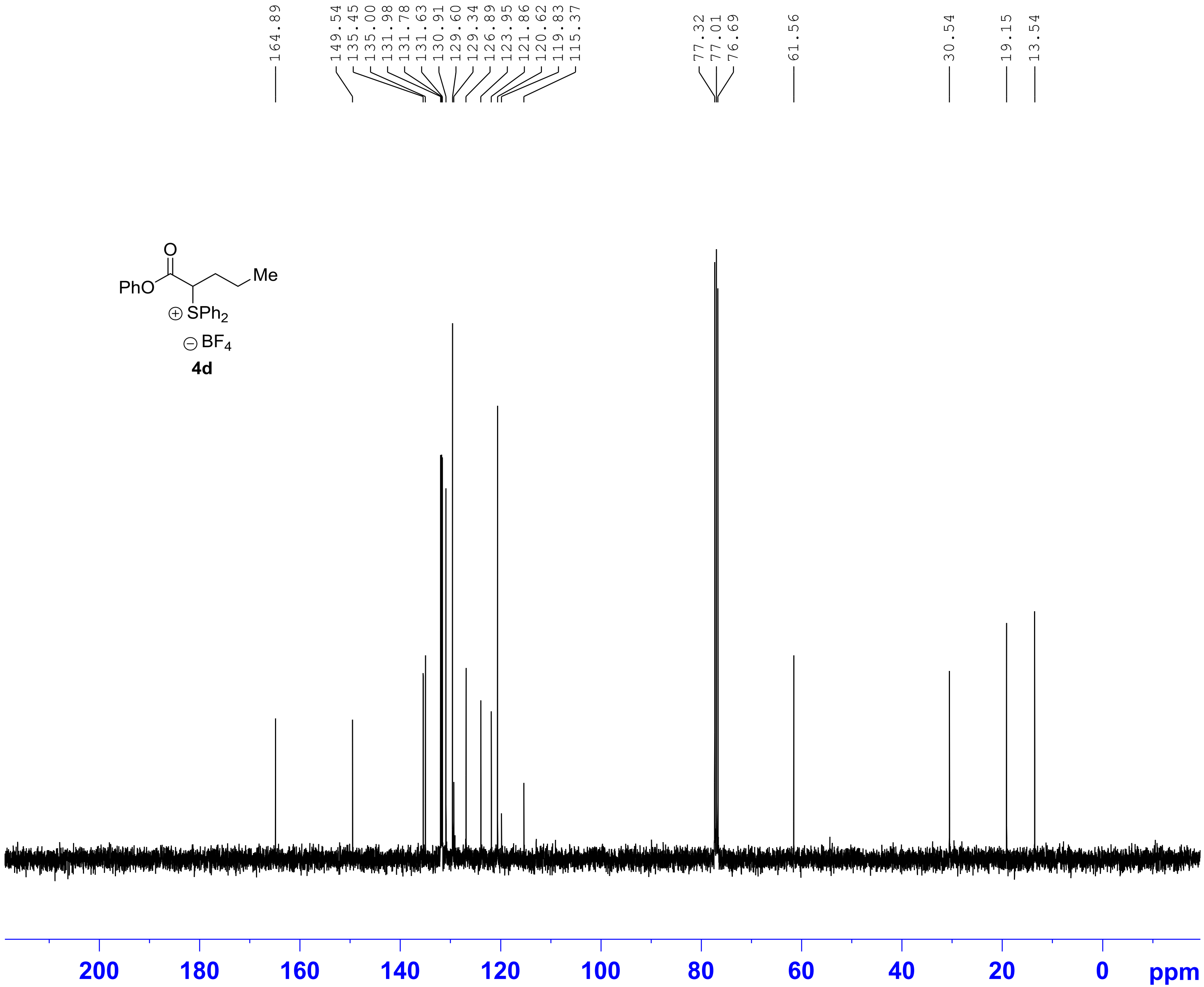

4d

```
NAME        lzy-08-182-A-SM-C
EXPNO                       2
PROCNO                      1
Date_                20220228
Time                    20.45
INSTRUM                 spect
PROBHD       5 mm DUL 13C-1
PULPROG               zgpg30
TD                      65536
SOLVENT                 CDCl3
NS                        166
DS                          0
SWH                 24038.461 Hz
FIDRES               0.366798 Hz
AQ                  1.3631988 sec
RG                       2050
DW                     20.800 usec
DE                       6.00 usec
TE                      293.7 K
D1                 2.00000000 sec
D11                0.03000000 sec
TD0                         1

======== CHANNEL f1 ========
NUC1                      13C
P1                      40.00 usec
PL1                     -3.00 dB
PL1W              60.64365387 W
SFO1              100.6228298 MHz

======== CHANNEL f2 ========
CPDPRG2               waltz16
NUC2                       1H
PCPD2                   80.00 usec
PL2                     -1.00 dB
PL12                    14.39 dB
PL13                    18.00 dB
PL2W              12.17476940 W
PL12W              0.35193357 W
PL13W              0.15327126 W
SFO2              400.1316005 MHz
SI                      32768
SF                100.6127783 MHz
WDW                        EM
SSB                         0
LB                       1.00 Hz
GB                          0
PC                       1.40
```

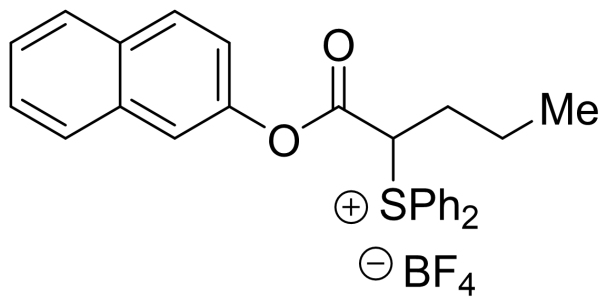

4e

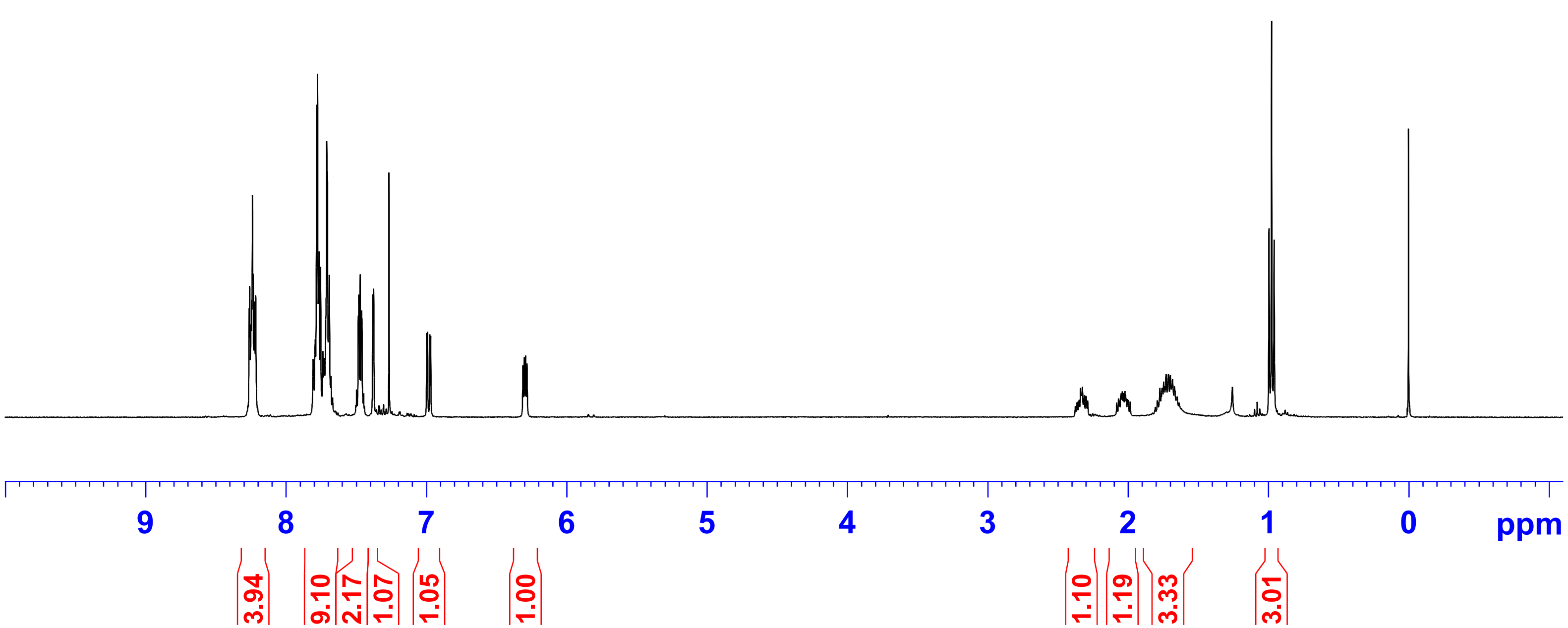

NAME	lzy-08-182-B-SM
EXPNO	2
PROCNO	1
Date_	20220227
Time	17.05
INSTRUM	spect
PROBHD	5 mm DUL 13C-1
PULPROG	zg30
TD	65536
SOLVENT	CDCl3
NS	4
DS	0
SWH	8223.685 Hz
FIDRES	0.125483 Hz
AQ	3.9846387 sec
RG	456
DW	60.800 usec
DE	6.00 usec
TE	293.9 K
D1	1.00000000 sec
TD0	1

======== CHANNEL f1 ========

NUC1	1H
P1	15.80 usec
PL1	-1.00 dB
PL1W	12.17476940 W
SFO1	400.1324710 MHz
SI	32768
SF	400.1300076 MHz
WDW	EM
SSB	0
LB	0.30 Hz
GB	0
PC	1.00

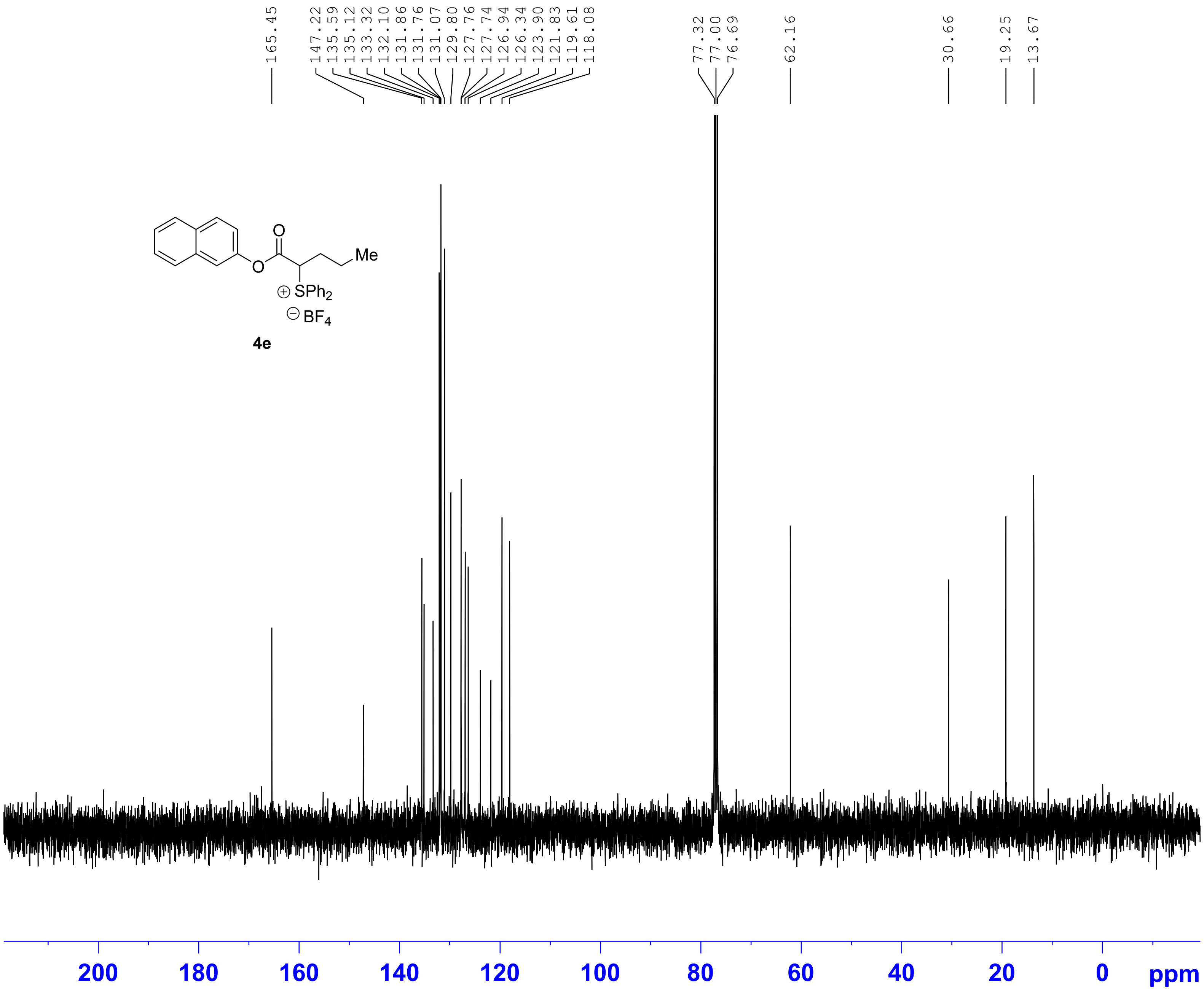

4e

NAME	lzy-08-182-B-SM-C	
EXPNO	3	
PROCNO	1	
Date_	20220227	
Time	17.11	
INSTRUM	spect	
PROBHD	5 mm DUL 13C-1	
PULPROG	zgpg30	
TD	65536	
SOLVENT	CDCl3	
NS	1000	
DS	0	
SWH	24038.461	Hz
FIDRES	0.366798	Hz
AQ	1.3631988	sec
RG	2050	
DW	20.800	usec
DE	6.00	usec
TE	294.2	K
D1	2.00000000	sec
D11	0.03000000	sec
TD0	1	
======== CHANNEL f1 ========		
NUC1	13C	
P1	40.00	usec
PL1	-3.00	dB
PL1W	60.64365387	W
SFO1	100.6228298	MHz
======== CHANNEL f2 ========		
CPDPRG2	waltz16	
NUC2	1H	
PCPD2	80.00	usec
PL2	-1.00	dB
PL12	14.39	dB
PL13	18.00	dB
PL2W	12.17476940	W
PL12W	0.35193357	W
PL13W	0.15327126	W
SFO2	400.1316005	MHz
SI	32768	
SF	100.6127728	MHz
WDW	EM	
SSB	0	
LB	1.00	Hz
GB	0	
PC	1.40	

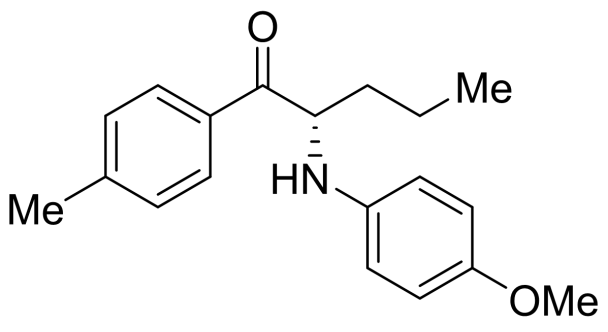

3a

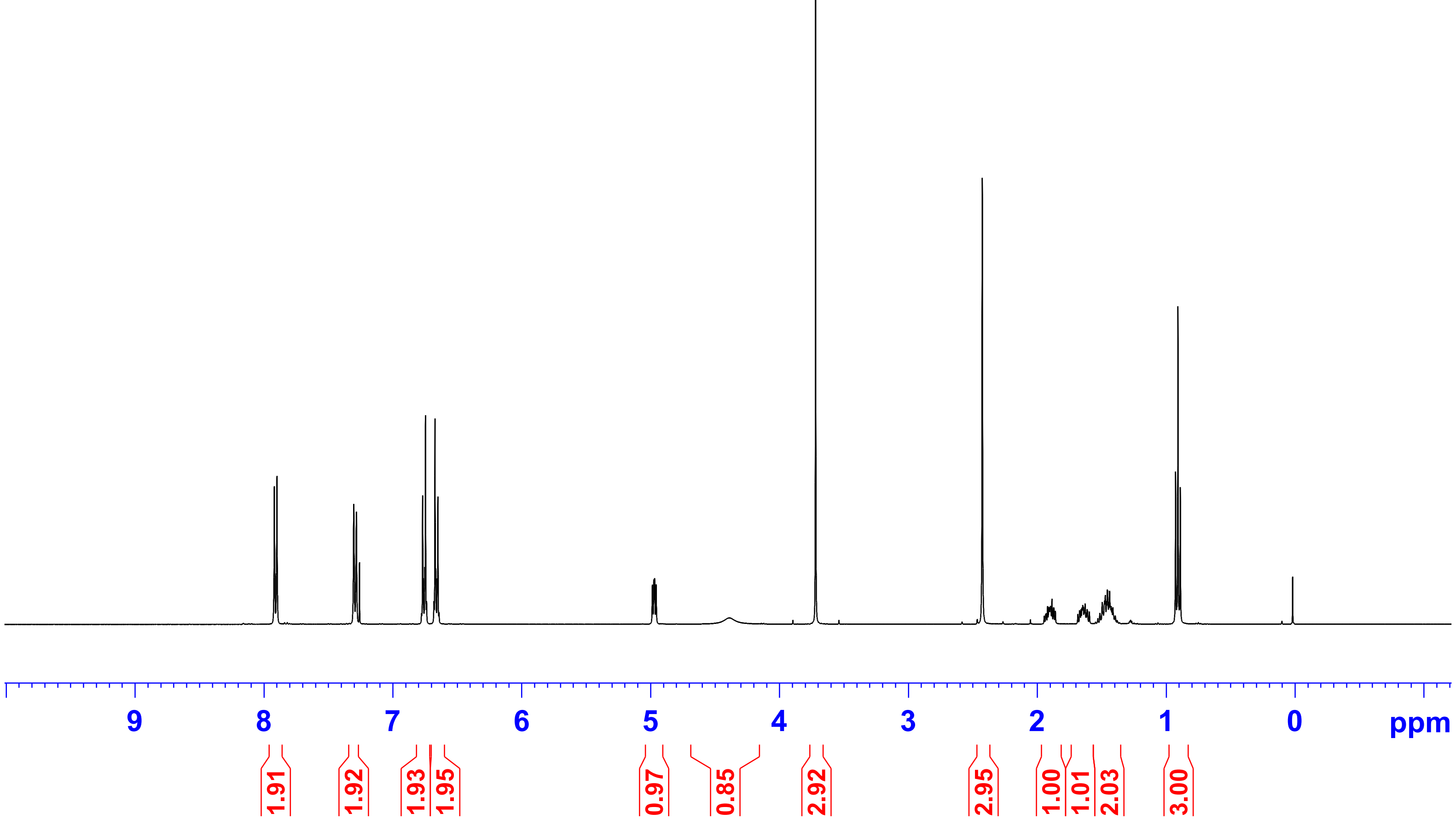

NAME	lzy-04-31-B-2
EXPNO	1
PROCNO	1
Date_	20201031
Time	10.47
INSTRUM	spect
PROBHD	5 mm PABBO BB/
PULPROG	zg30
TD	65536
SOLVENT	CDCl3
NS	4
DS	0
SWH	8012.820 Hz
FIDRES	0.122266 Hz
AQ	4.0894966 sec
RG	27.78
DW	62.400 usec
DE	6.50 usec
TE	297.1 K
D1	1.00000000 sec
TD0	1

======== CHANNEL f1 ========	
SFO1	400.1324710 MHz
NUC1	1H
P1	14.50 usec
SI	65536
SF	400.1300097 MHz
WDW	EM
SSB	0
LB	0.30 Hz
GB	0
PC	1.00

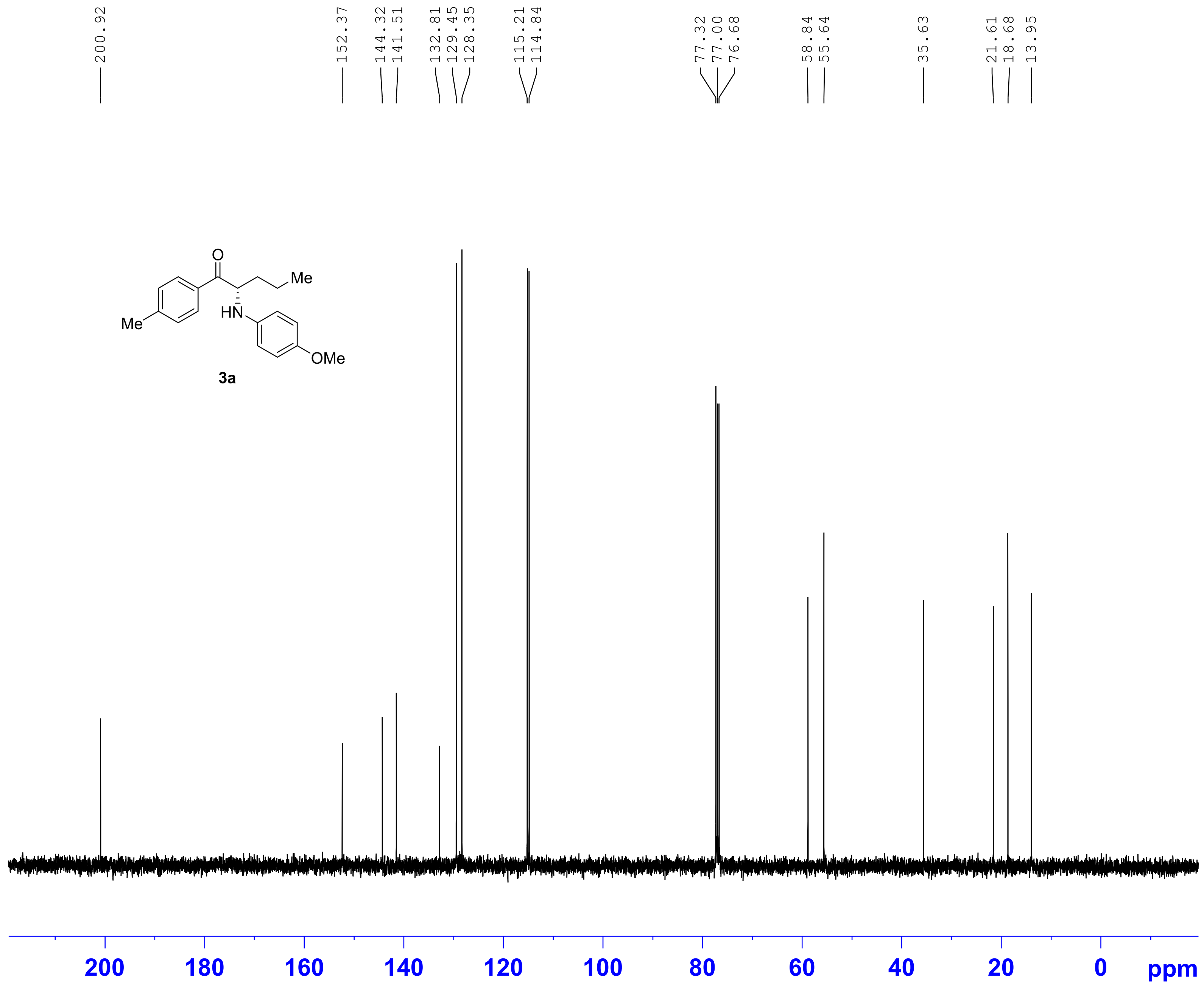

NAME	lzy-04-31-B-C	
EXPNO	1	
PROCNO	1	
Date_	20201031	
Time_	10.48	
INSTRUM	spect	
PROBHD	5 mm PABBO BB/	
PULPROG	zgpg30	
TD	65536	
SOLVENT	CDCl3	
NS	20	
DS	0	
SWH	24038.461	Hz
FIDRES	0.366798	Hz
AQ	1.3631988	sec
RG	196.92	
DW	20.800	usec
DE	6.50	usec
TE	297.7	K
D1	2.00000000	sec
D11	0.03000000	sec
TD0	1	

======== CHANNEL f1 ========

SFO1	100.6228298	MHz
NUC1	13C	
P1	9.70	usec
SI	32768	
SF	100.6127795	MHz
WDW	EM	
SSB	0	
LB	1.00	Hz
GB	0	
PC	1.40	

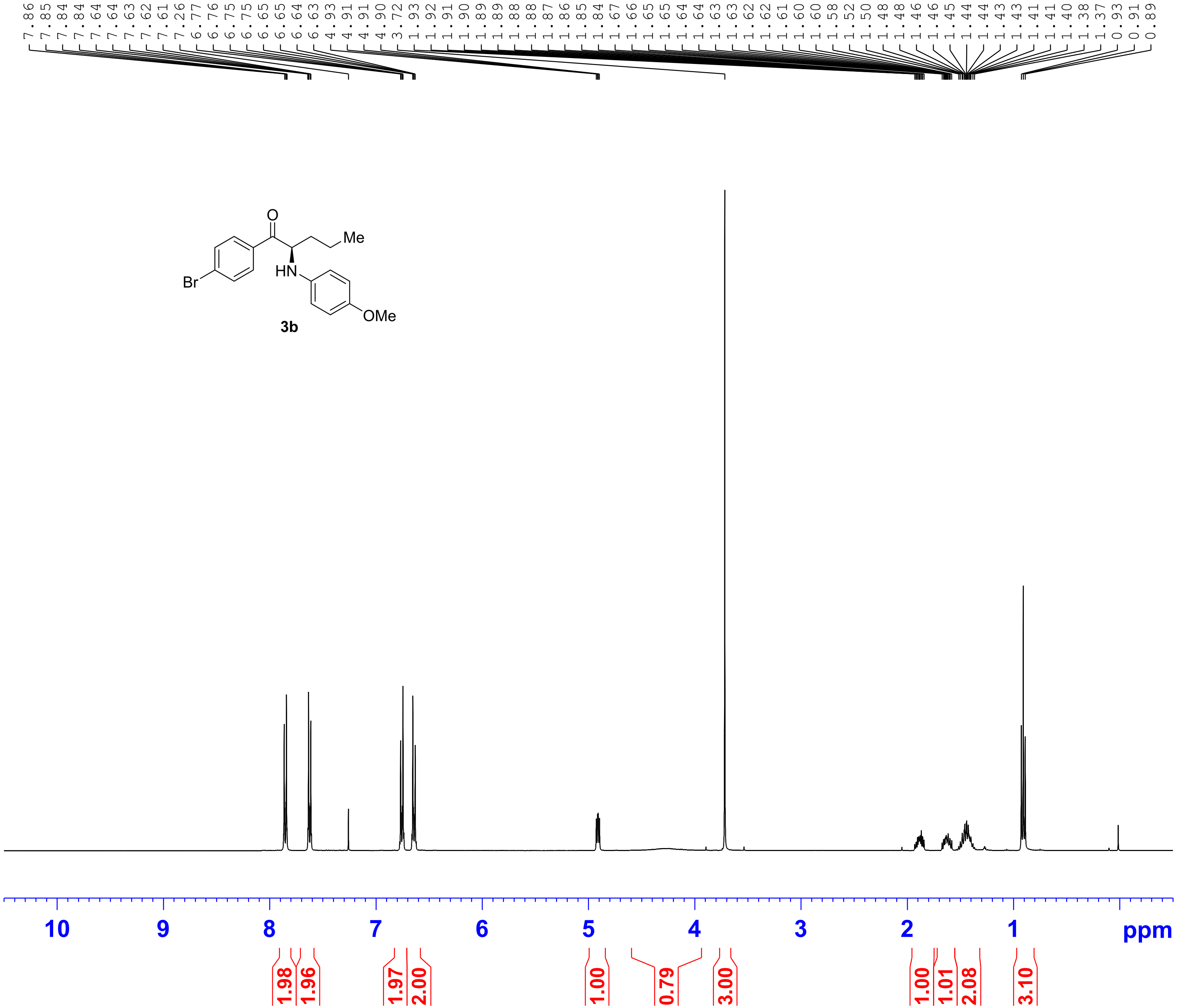

3b

NAME	lzy-04-174-A
EXPNO	1
PROCNO	1
Date_	20201031
Time_	10.36
INSTRUM	spect
PROBHD	5 mm PABBO BB/
PULPROG	zg30
TD	65536
SOLVENT	CDCl3
NS	4
DS	0
SWH	8012.820 Hz
FIDRES	0.122266 Hz
AQ	4.0894966 sec
RG	27.78
DW	62.400 usec
DE	6.50 usec
TE	297.2 K
D1	1.00000000 sec
TD0	1

======== CHANNEL f1 ========

SFO1	400.1324710 MHz
NUC1	1H
P1	14.50 usec
SI	65536
SF	400.1300097 MHz
WDW	EM
SSB	0
LB	0.30 Hz
GB	0
PC	1.00

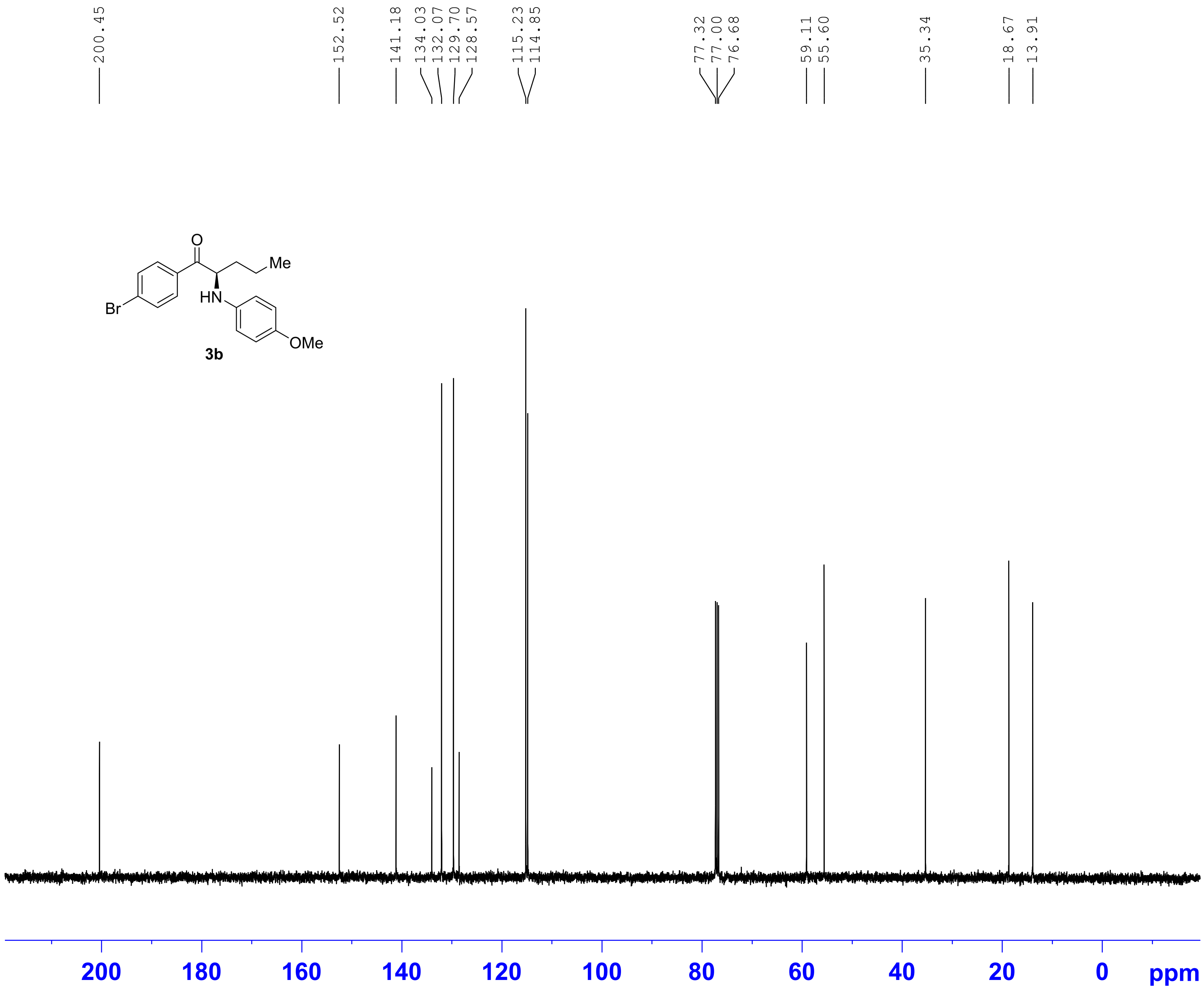

3b

NAME	lzy-04-174-A-C
EXPNO	1
PROCNO	1
Date_	20201031
Time	10.38
INSTRUM	spect
PROBHD	5 mm PABBO BB/
PULPROG	zgpg30
TD	65536
SOLVENT	CDCl3
NS	27
DS	0
SWH	24038.461 Hz
FIDRES	0.366798 Hz
AQ	1.3631988 sec
RG	196.92
DW	20.800 usec
DE	6.50 usec
TE	297.8 K
D1	2.00000000 sec
D11	0.03000000 sec
TD0	1

======== CHANNEL f1 ========

SFO1	100.6228298 MHz
NUC1	13C
P1	9.70 usec
SI	32768
SF	100.6127817 MHz
WDW	EM
SSB	0
LB	1.00 Hz
GB	0
PC	1.40

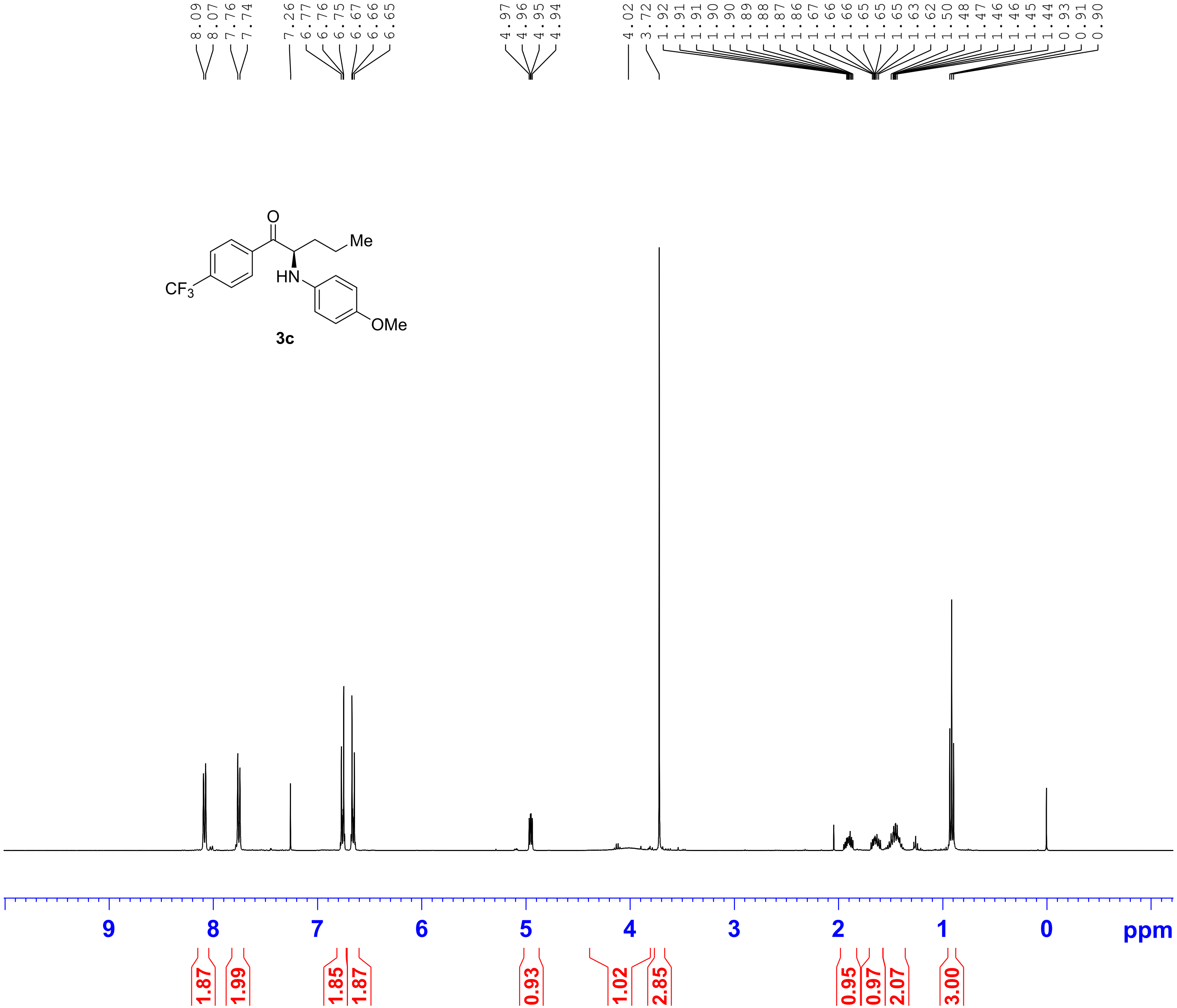

NAME	lzy-04-107
EXPNO	1
PROCNO	1
Date_	20200919
Time	21.50
INSTRUM	spect
PROBHD	5 mm PABBO BB-
PULPROG	zg30
TD	65536
SOLVENT	CDCl3
NS	4
DS	0
SWH	8223.685 Hz
FIDRES	0.125483 Hz
AQ	3.9846387 sec
RG	287
DW	60.800 usec
DE	6.00 usec
TE	296.4 K
D1	1.00000000 sec
TD0	1

======== CHANNEL f1 ========

NUC1	1H
P1	15.80 usec
PL1	-1.00 dB
PL1W	12.17476940 W
SFO1	400.1324710 MHz
SI	32768
SF	400.1300099 MHz
WDW	EM
SSB	0
LB	0.30 Hz
GB	0
PC	1.00

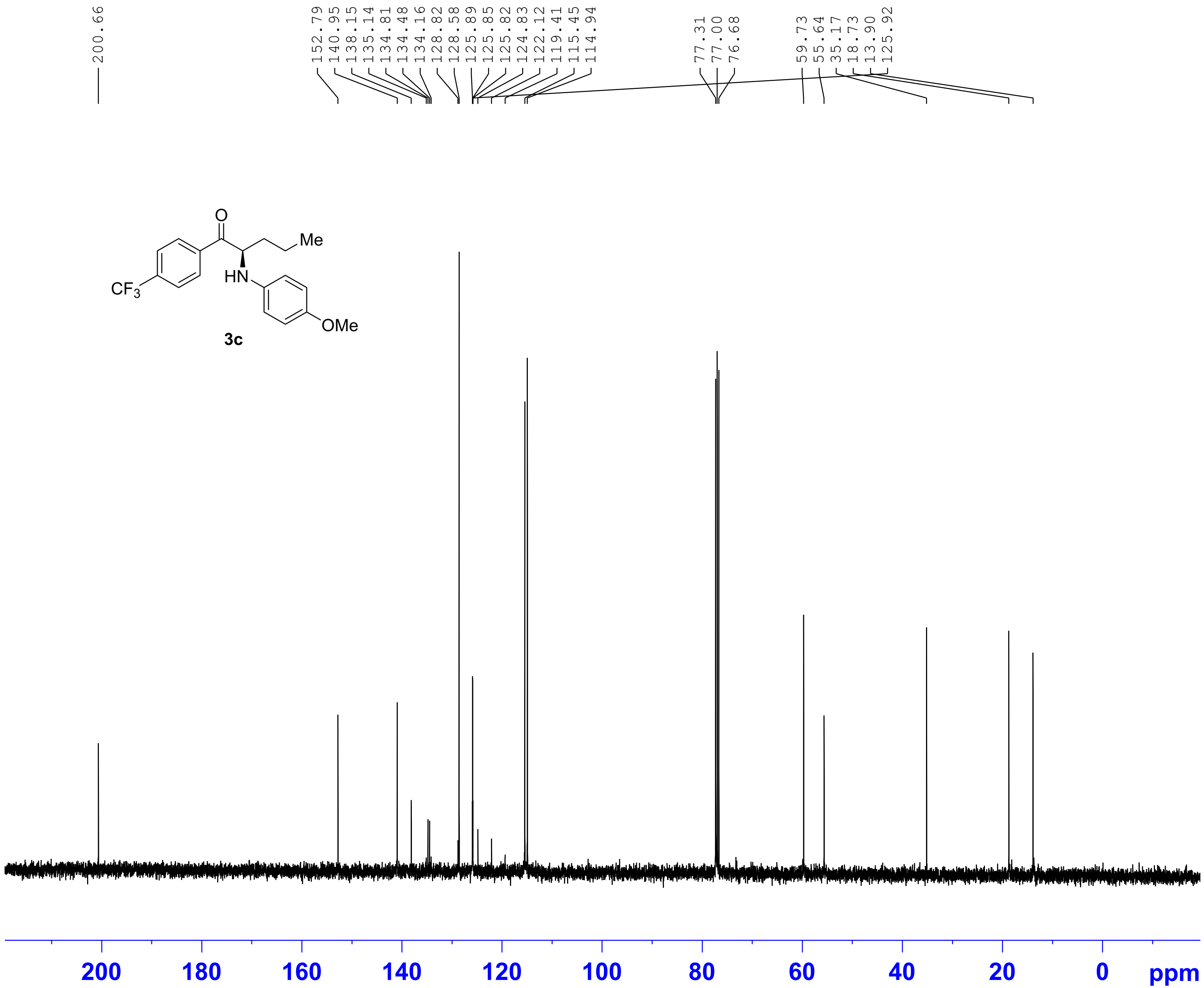

NAME	lzy-04-107-C	
EXPNO	1	
PROCNO	1	
Date_	20200919	
Time	21.52	
INSTRUM	spect	
PROBHD	5 mm PABBO BB-	
PULPROG	zgpg30	
TD	65536	
SOLVENT	CDCl3	
NS	88	
DS	0	
SWH	24038.461	Hz
FIDRES	0.366798	Hz
AQ	1.3631988	sec
RG	90.5	
DW	20.800	usec
DE	6.00	usec
TE	296.4	K
D1	2.00000000	sec
D11	0.03000000	sec
TD0	1	

======== CHANNEL f1 ========

NUC1	13C	
P1	8.60	usec
PL1	-3.00	dB
PL1W	60.64365387	W
SFO1	100.6228298	MHz

======== CHANNEL f2 ========

CPDPRG2	waltz16	
NUC2	1H	
PCPD2	80.00	usec
PL2	-1.00	dB
PL12	14.39	dB
PL13	18.00	dB
PL2W	12.17476940	W
PL12W	0.35193357	W
PL13W	0.15327126	W
SFO2	400.1316005	MHz
SI	32768	
SF	100.6127737	MHz
WDW	EM	
SSB	0	
LB	1.00	Hz
GB	0	
PC	1.40	

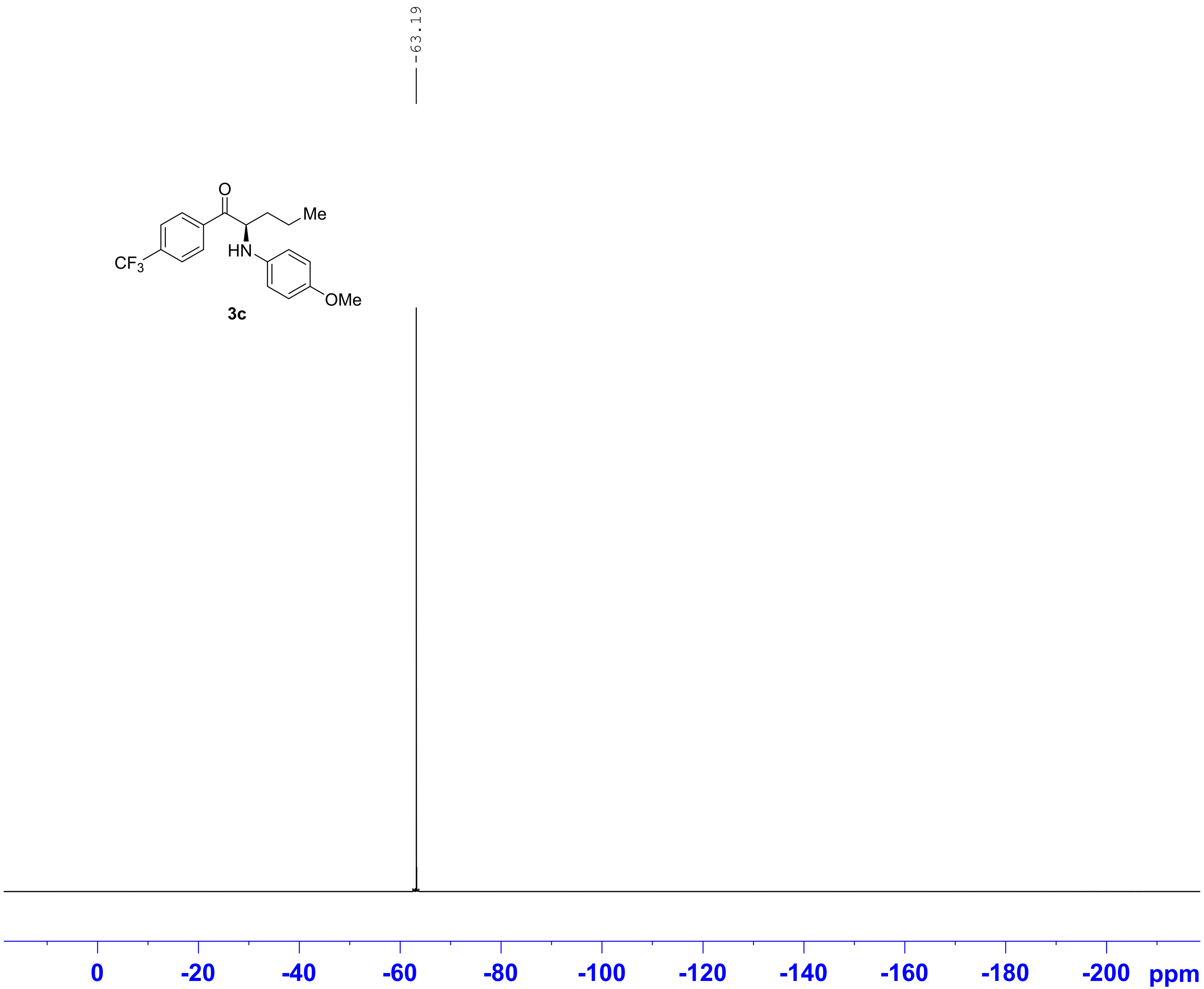

3c

NAME	lzy-04-107-F	
EXPNO	1	
PROCNO	1	
Date_	20200919	
Time	21.59	
INSTRUM	spect	
PROBHD	5 mm PABBO BB-	
PULPROG	zgflqn	
TD	131072	
SOLVENT	CDCl3	
NS	8	
DS	4	
SWH	89285.711	Hz
FIDRES	0.681196	Hz
AQ	0.7340532	sec
RG	1290	
DW	5.600	usec
DE	6.00	usec
TE	296.5	K
D1	1.00000000	sec
TD0	1	

======== CHANNEL f1 ========

NUC1	19F	
P1	19.50	usec
PL1	-4.00	dB
PL1W	16.97275162	W
SFO1	376.4607164	MHz
SI	65536	
SF	376.4983660	MHz
WDW	EM	
SSB	0	
LB	0.30	Hz
GB	0	
PC	1.00	

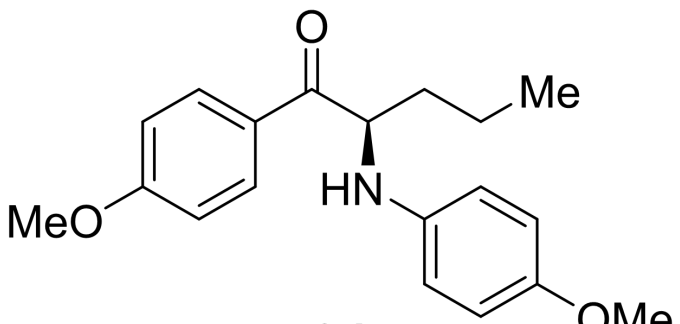

3d

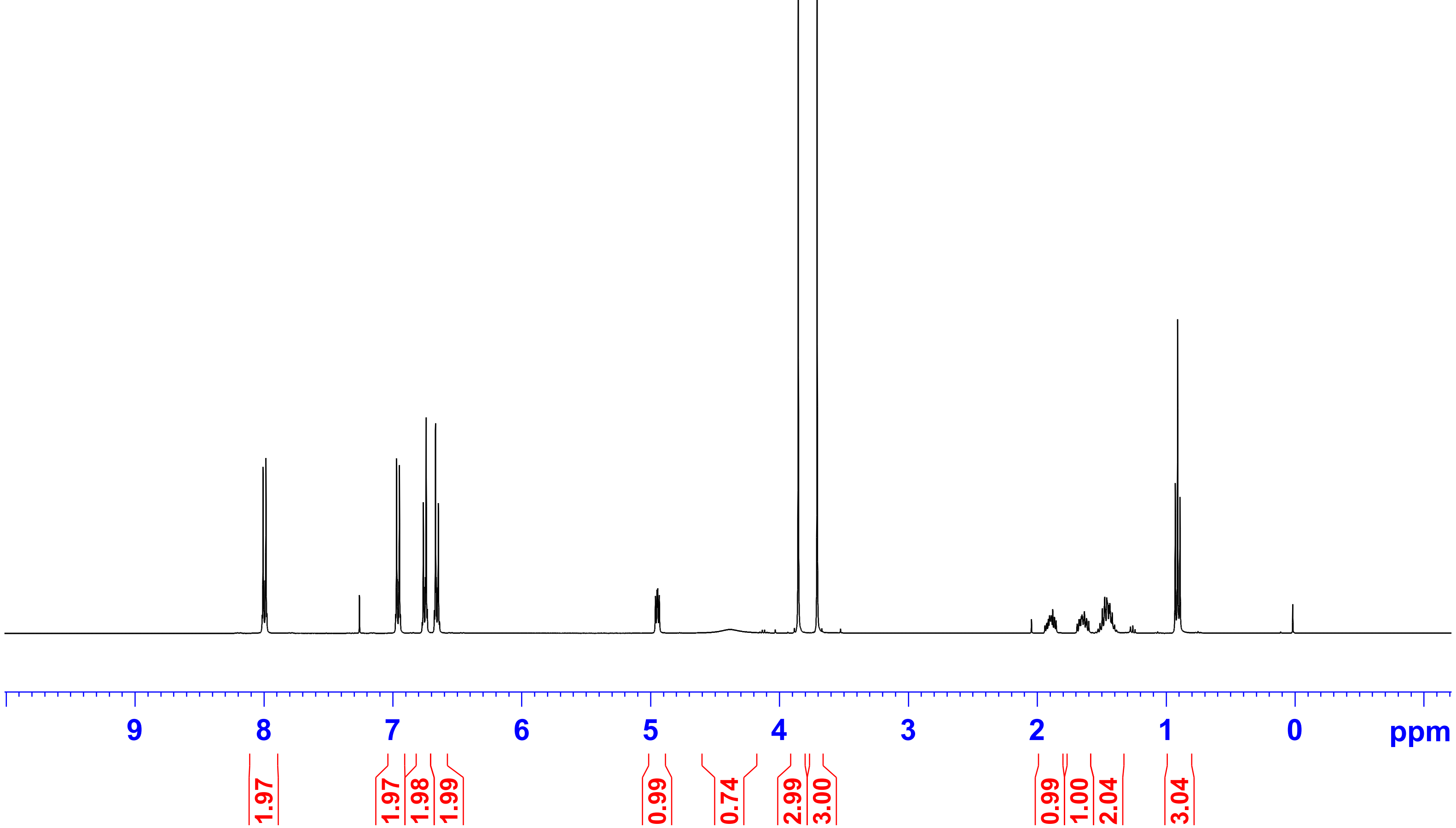

NAME	lzy-04-174-B
EXPNO	1
PROCNO	1
Date_	20201031
Time	10.21
INSTRUM	spect
PROBHD	5 mm PABBO BB/
PULPROG	zg30
TD	65536
SOLVENT	CDCl3
NS	4
DS	0
SWH	8012.820 Hz
FIDRES	0.122266 Hz
AQ	4.0894966 sec
RG	22.47
DW	62.400 usec
DE	6.50 usec
TE	297.1 K
D1	1.00000000 sec
TD0	1

======== CHANNEL f1 ========

SFO1	400.1324710 MHz
NUC1	1H
P1	14.50 usec
SI	65536
SF	400.1300095 MHz
WDW	EM
SSB	0
LB	0.30 Hz
GB	0
PC	1.00

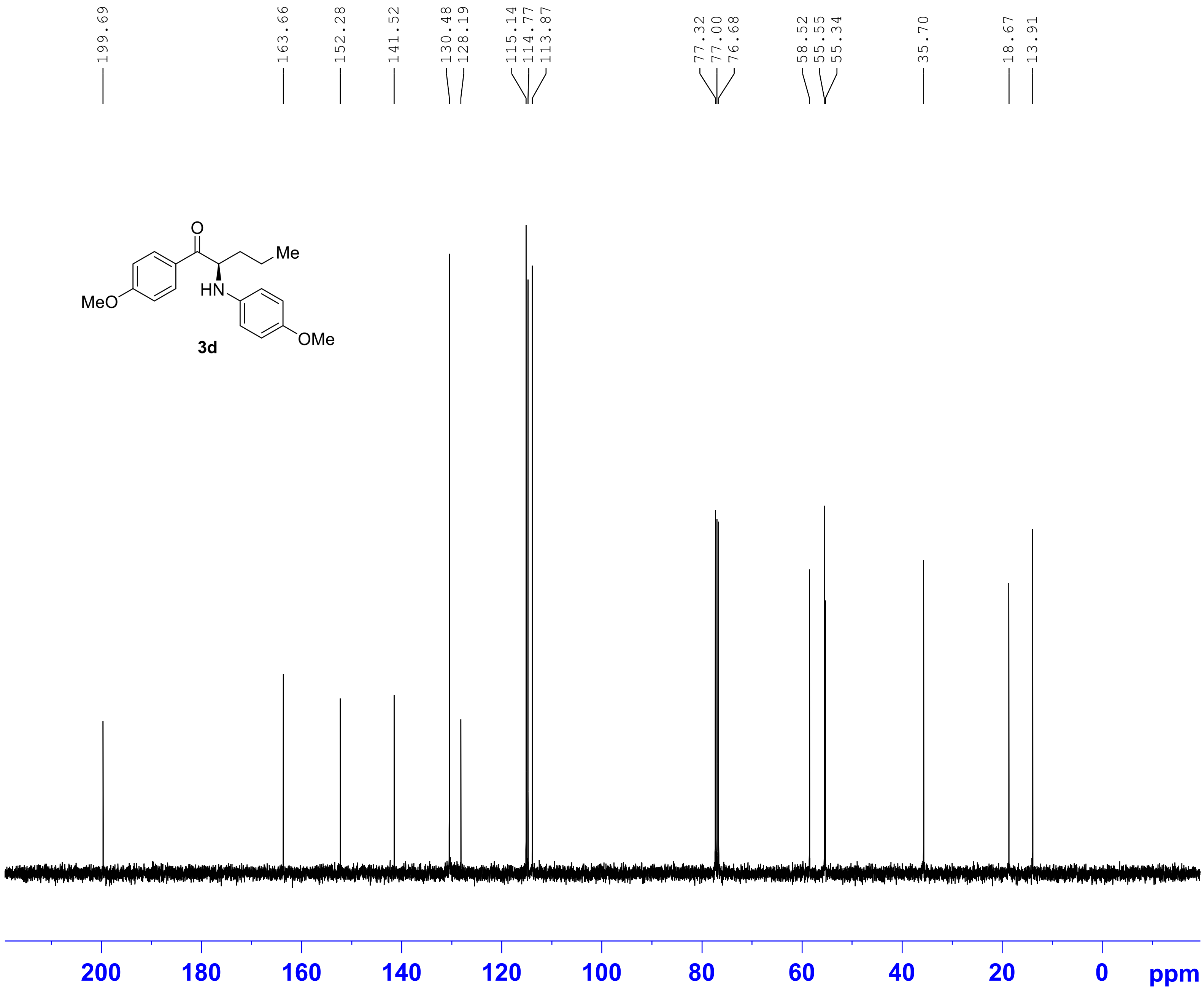

NAME	lzy-04-174-B-C
EXPNO	1
PROCNO	1
Date_	20201031
Time	10.23
INSTRUM	spect
PROBHD	5 mm PABBO BB/
PULPROG	zgpg30
TD	65536
SOLVENT	CDCl3
NS	12
DS	0
SWH	24038.461 Hz
FIDRES	0.366798 Hz
AQ	1.3631988 sec
RG	196.92
DW	20.800 usec
DE	6.50 usec
TE	297.3 K
D1	2.00000000 sec
D11	0.03000000 sec
TD0	1

======== CHANNEL f1 ========

SFO1	100.6228298 MHz
NUC1	13C
P1	9.70 usec
SI	32768
SF	100.6127861 MHz
WDW	EM
SSB	0
LB	1.00 Hz
GB	0
PC	1.40

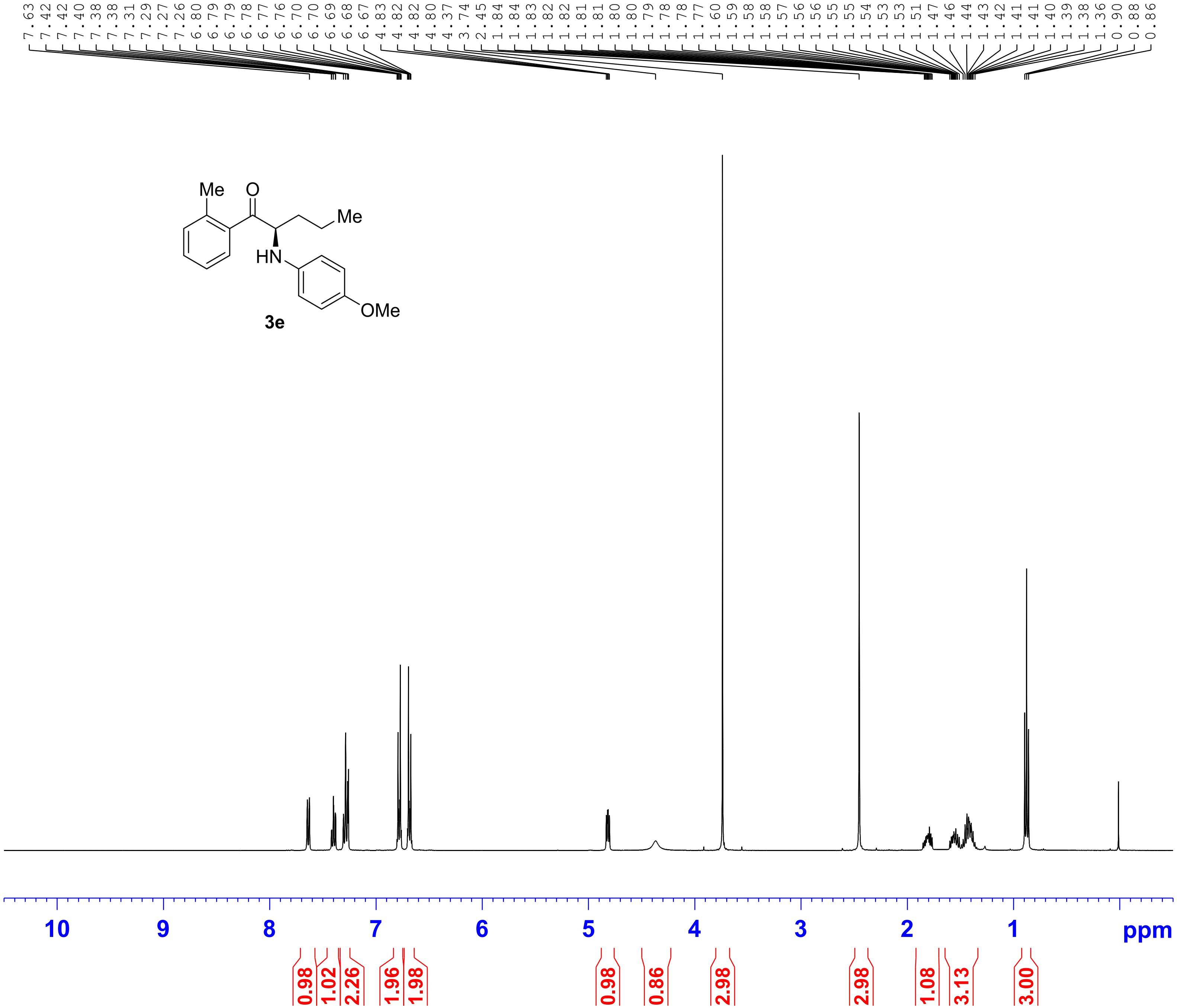

NAME	lzy-04-125-C
EXPNO	1
PROCNO	1
Date_	20200928
Time	19.37
INSTRUM	spect
PROBHD	5 mm PABBO BB-
PULPROG	zg30
TD	65536
SOLVENT	CDCl3
NS	4
DS	0
SWH	8223.685 Hz
FIDRES	0.125483 Hz
AQ	3.9846387 sec
RG	228
DW	60.800 usec
DE	6.00 usec
TE	294.5 K
D1	1.00000000 sec
TD0	1

======== CHANNEL f1 ========

NUC1	1H
P1	15.80 usec
PL1	-1.00 dB
PL1W	12.17476940 W
SFO1	400.1324710 MHz
SI	32768
SF	400.1300096 MHz
WDW	EM
SSB	0
LB	0.30 Hz
GB	0
PC	1.00

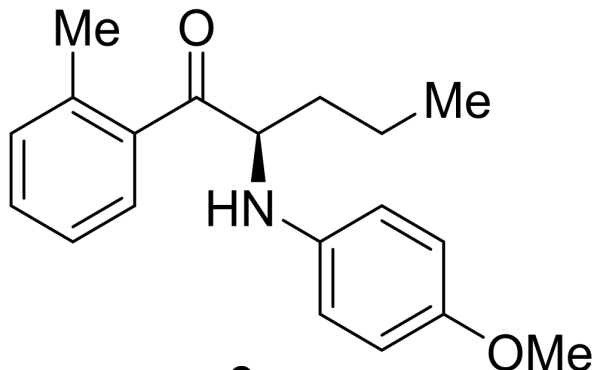

3e

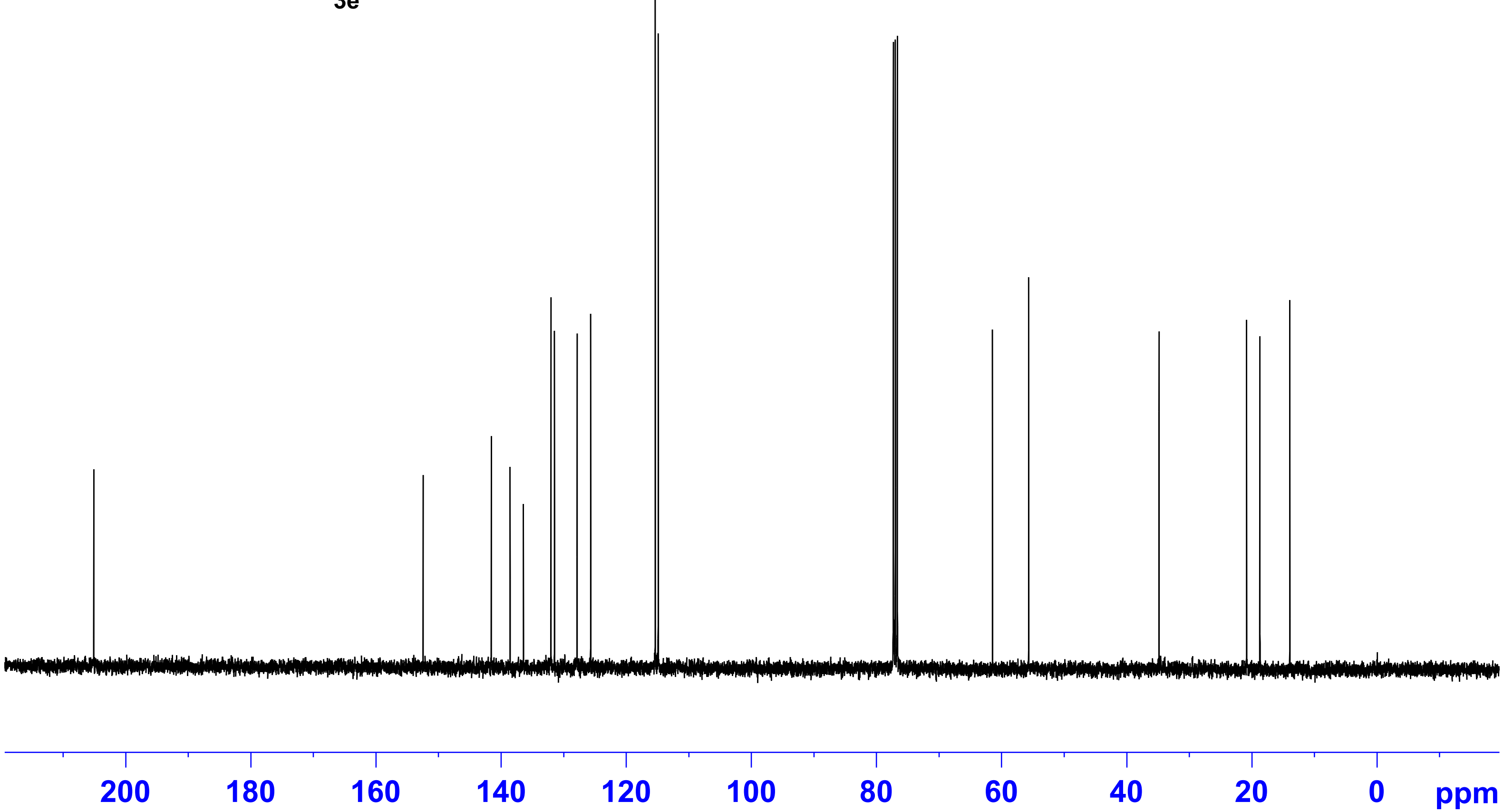

NAME	lzy-04-125-C-C	
EXPNO	2	
PROCNO	1	
Date_	20200928	
Time_	19.48	
INSTRUM	spect	
PROBHD	5 mm PABBO BB-	
PULPROG	zgpg30	
TD	65536	
SOLVENT	CDCl3	
NS	105	
DS	2	
SWH	24038.461	Hz
FIDRES	0.366798	Hz
AQ	1.3631988	sec
RG	90.5	
DW	20.800	usec
DE	6.00	usec
TE	295.1	K
D1	2.00000000	sec
D11	0.03000000	sec
TD0	1	
======== CHANNEL f1 ========		
NUC1	13C	
P1	8.60	usec
PL1	-3.00	dB
PL1W	60.64365387	W
SFO1	100.6228298	MHz
======== CHANNEL f2 ========		
CPDPRG2	waltz16	
NUC2	1H	
PCPD2	80.00	usec
PL2	-1.00	dB
PL12	14.39	dB
PL13	18.00	dB
PL2W	12.17476940	W
PL12W	0.35193357	W
PL13W	0.15327126	W
SFO2	400.1316005	MHz
SI	32768	
SF	100.6127766	MHz
WDW	EM	
SSB	0	
LB	1.00	Hz
GB	0	
PC	1.40	

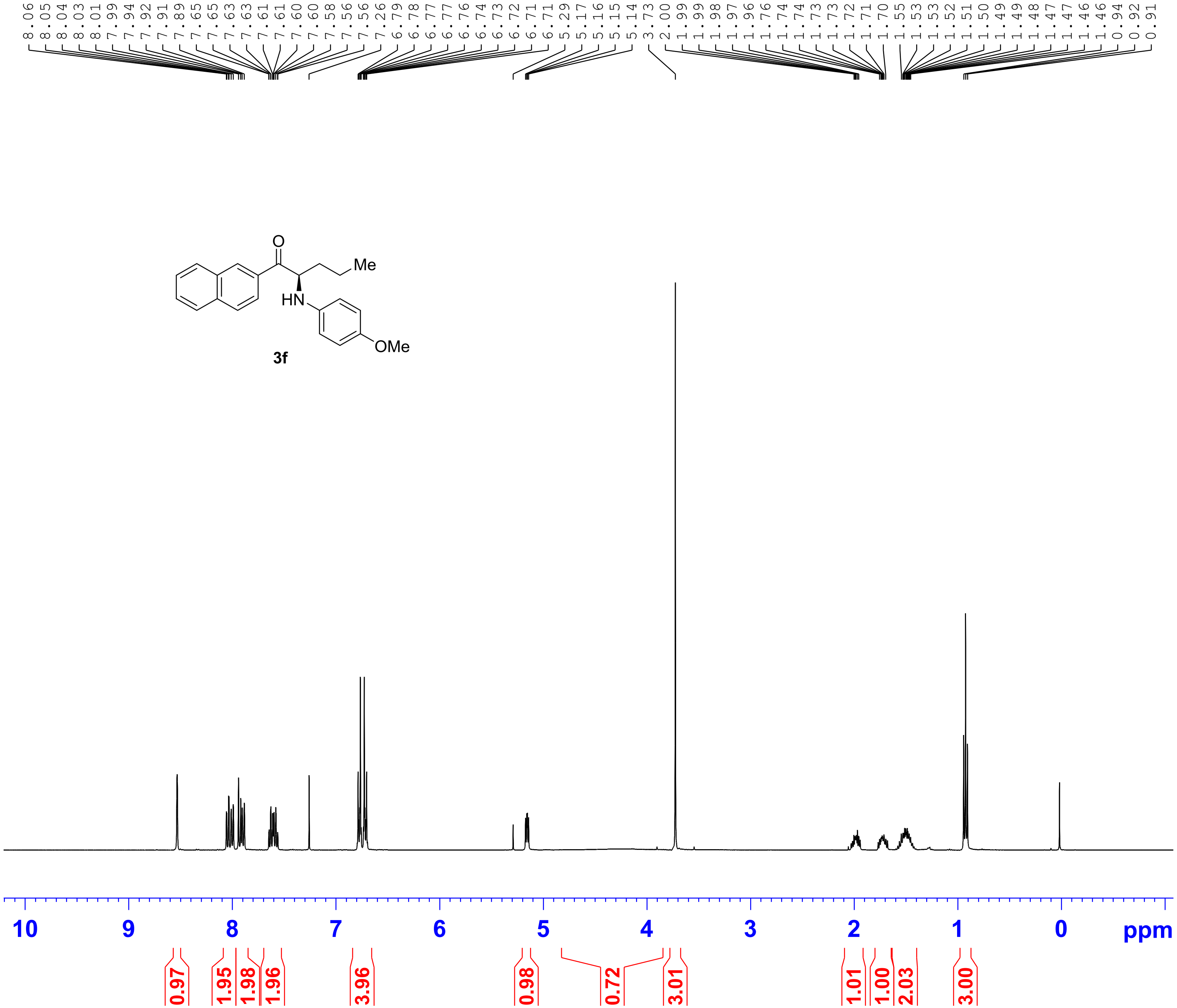

NAME	lzy-04-125-B
EXPNO	1
PROCNO	1
Date_	20200926
Time	21.29
INSTRUM	spect
PROBHD	5 mm PABBO BB-
PULPROG	zg30
TD	65536
SOLVENT	CDCl3
NS	4
DS	0
SWH	8223.685 Hz
FIDRES	0.125483 Hz
AQ	3.9846387 sec
RG	203
DW	60.800 usec
DE	6.00 usec
TE	296.1 K
D1	1.00000000 sec
TD0	1

======== CHANNEL f1 ========

NUC1	1H
P1	15.80 usec
PL1	-1.00 dB
PL1W	12.17476940 W
SFO1	400.1324710 MHz
SI	32768
SF	400.1300095 MHz
WDW	EM
SSB	0
LB	0.30 Hz
GB	0
PC	1.00

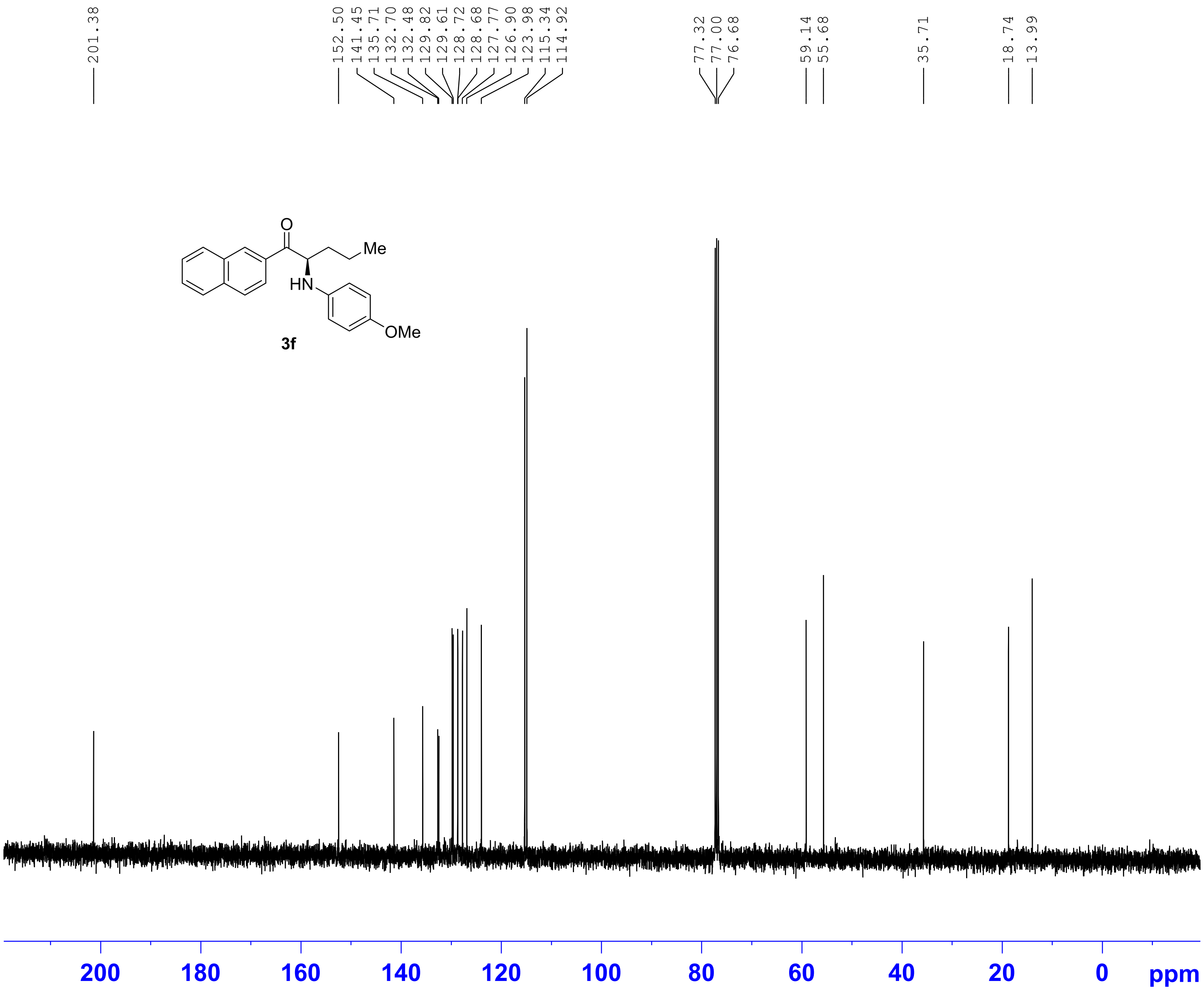

NAME lzy-04-125-B-C
EXPNO 1
PROCNO 1
Date_ 20200926
Time 21.33
INSTRUM spect
PROBHD 5 mm PABBO BB-
PULPROG zgpg30
TD 65536
SOLVENT CDCl3
NS 40
DS 2
SWH 24038.461 Hz
FIDRES 0.366798 Hz
AQ 1.3631988 sec
RG 80.6
DW 20.800 usec
DE 6.00 usec
TE 296.5 K
D1 2.00000000 sec
D11 0.03000000 sec
TD0 1

======== CHANNEL f1 ========
NUC1 13C
P1 8.60 usec
PL1 -3.00 dB
PL1W 60.64365387 W
SFO1 100.6228298 MHz

======== CHANNEL f2 ========
CPDPRG2 waltz16
NUC2 1H
PCPD2 80.00 usec
PL2 -1.00 dB
PL12 14.39 dB
PL13 18.00 dB
PL2W 12.17476940 W
PL12W 0.35193357 W
PL13W 0.15327126 W
SFO2 400.1316005 MHz
SI 32768
SF 100.6127765 MHz
WDW EM
SSB 0
LB 1.00 Hz
GB 0
PC 1.40

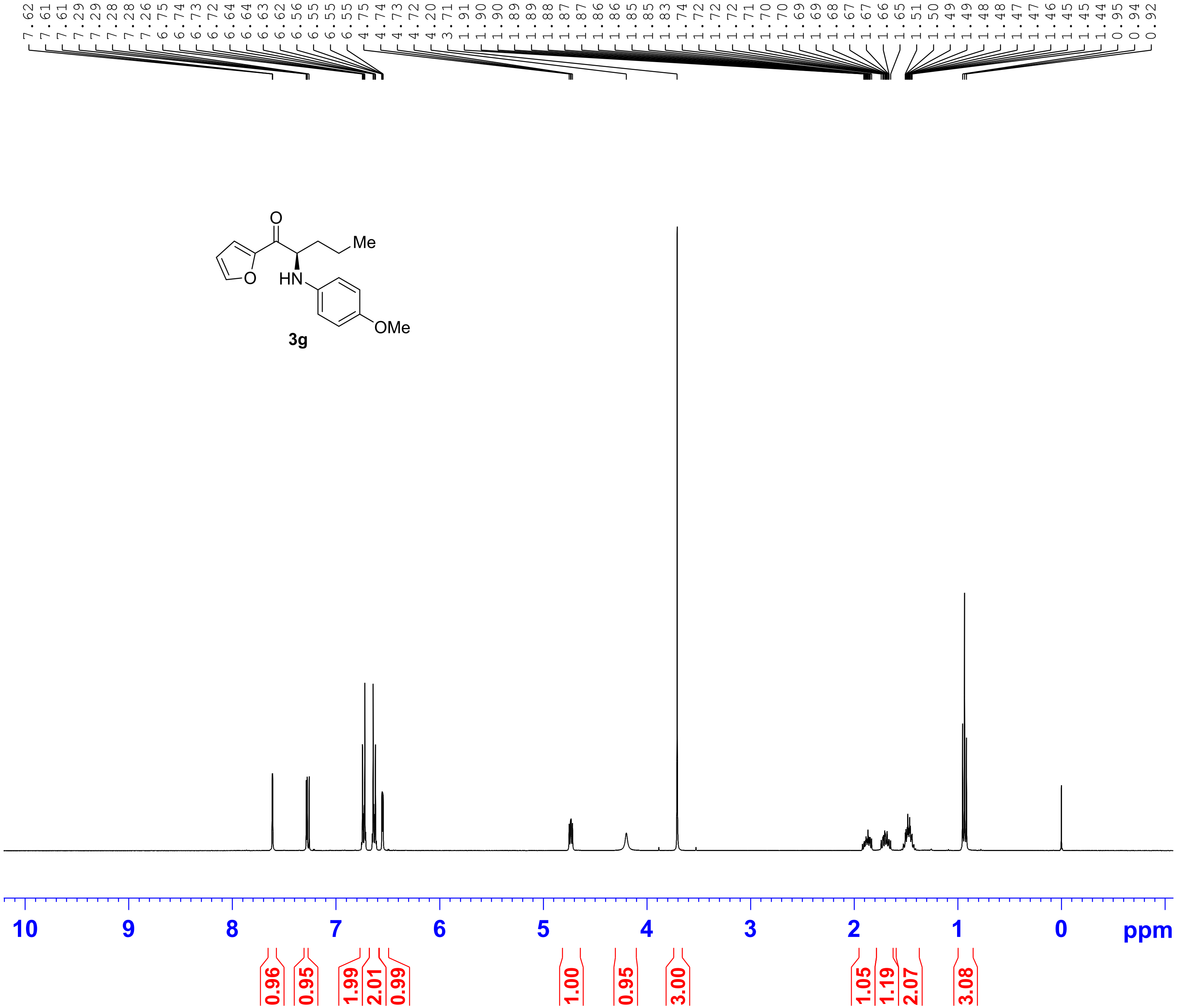

3g

NAME	lzy-04-140-2
EXPNO	1
PROCNO	1
Date_	20201006
Time_	19.38
INSTRUM	spect
PROBHD	5 mm PABBO BB/
PULPROG	zg30
TD	65536
SOLVENT	CDCl3
NS	4
DS	0
SWH	8012.820 Hz
FIDRES	0.122266 Hz
AQ	4.0894966 sec
RG	39.46
DW	62.400 usec
DE	6.50 usec
TE	296.6 K
D1	1.00000000 sec
TD0	1

======== CHANNEL f1 ========

SFO1	400.1324710 MHz
NUC1	1H
P1	14.50 usec
SI	65536
SF	400.1300098 MHz
WDW	EM
SSB	0
LB	0.30 Hz
GB	0
PC	1.00

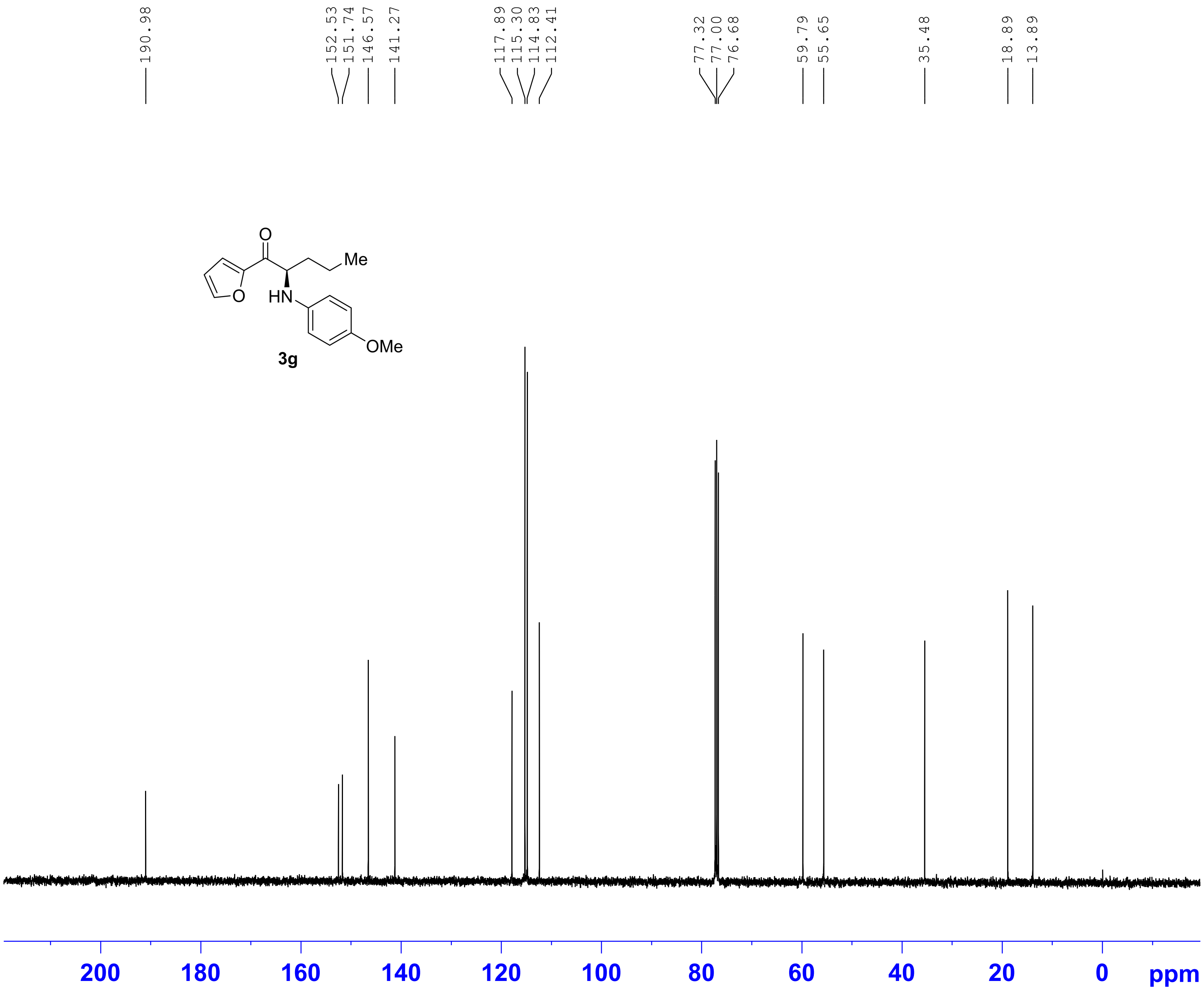

3g

NAME	lzy-04-140-2-C
EXPNO	1
PROCNO	1
Date_	20201006
Time	19.44
INSTRUM	spect
PROBHD	5 mm PABBO BB/
PULPROG	zgpg30
TD	65536
SOLVENT	CDCl3
NS	134
DS	0
SWH	24038.461 Hz
FIDRES	0.366798 Hz
AQ	1.3631988 sec
RG	196.92
DW	20.800 usec
DE	6.50 usec
TE	297.6 K
D1	2.00000000 sec
D11	0.03000000 sec
TD0	1

======== CHANNEL f1 ========

SFO1	100.6228298 MHz
NUC1	13C
P1	9.70 usec
SI	32768
SF	100.6127759 MHz
WDW	EM
SSB	0
LB	1.00 Hz
GB	0
PC	1.40

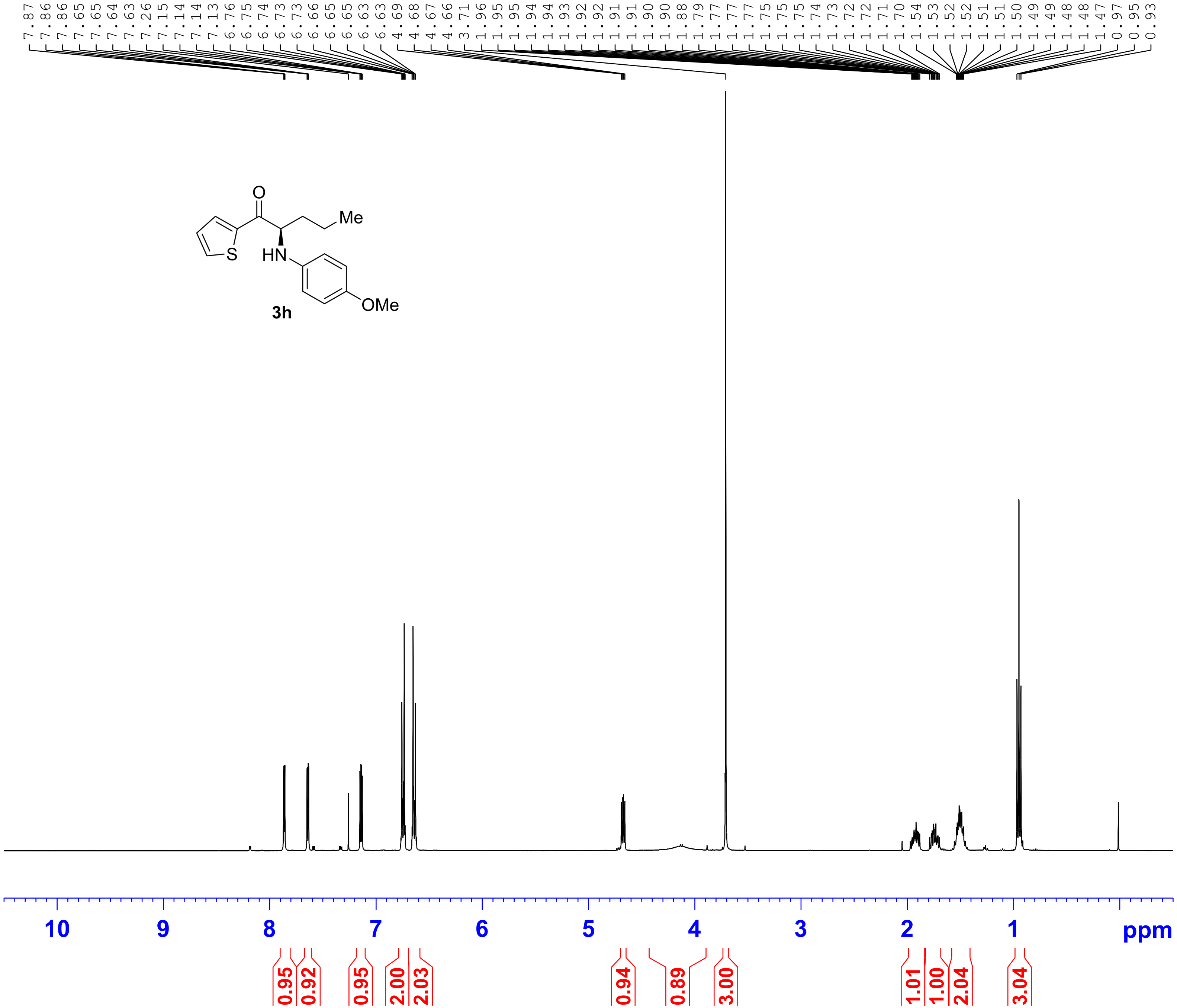

NAME	lzy-04-174-H-2
EXPNO	1
PROCNO	1
Date_	20201031
Time	11.04
INSTRUM	spect
PROBHD	5 mm PABBO BB/
PULPROG	zg30
TD	65536
SOLVENT	CDCl3
NS	3
DS	0
SWH	8012.820 Hz
FIDRES	0.122266 Hz
AQ	4.0894966 sec
RG	27.78
DW	62.400 usec
DE	6.50 usec
TE	297.0 K
D1	1.00000000 sec
TD0	1

======== CHANNEL f1 ========

SFO1	400.1324710 MHz
NUC1	1H
P1	14.50 usec
SI	65536
SF	400.1300098 MHz
WDW	EM
SSB	0
LB	0.30 Hz
GB	0
PC	1.00

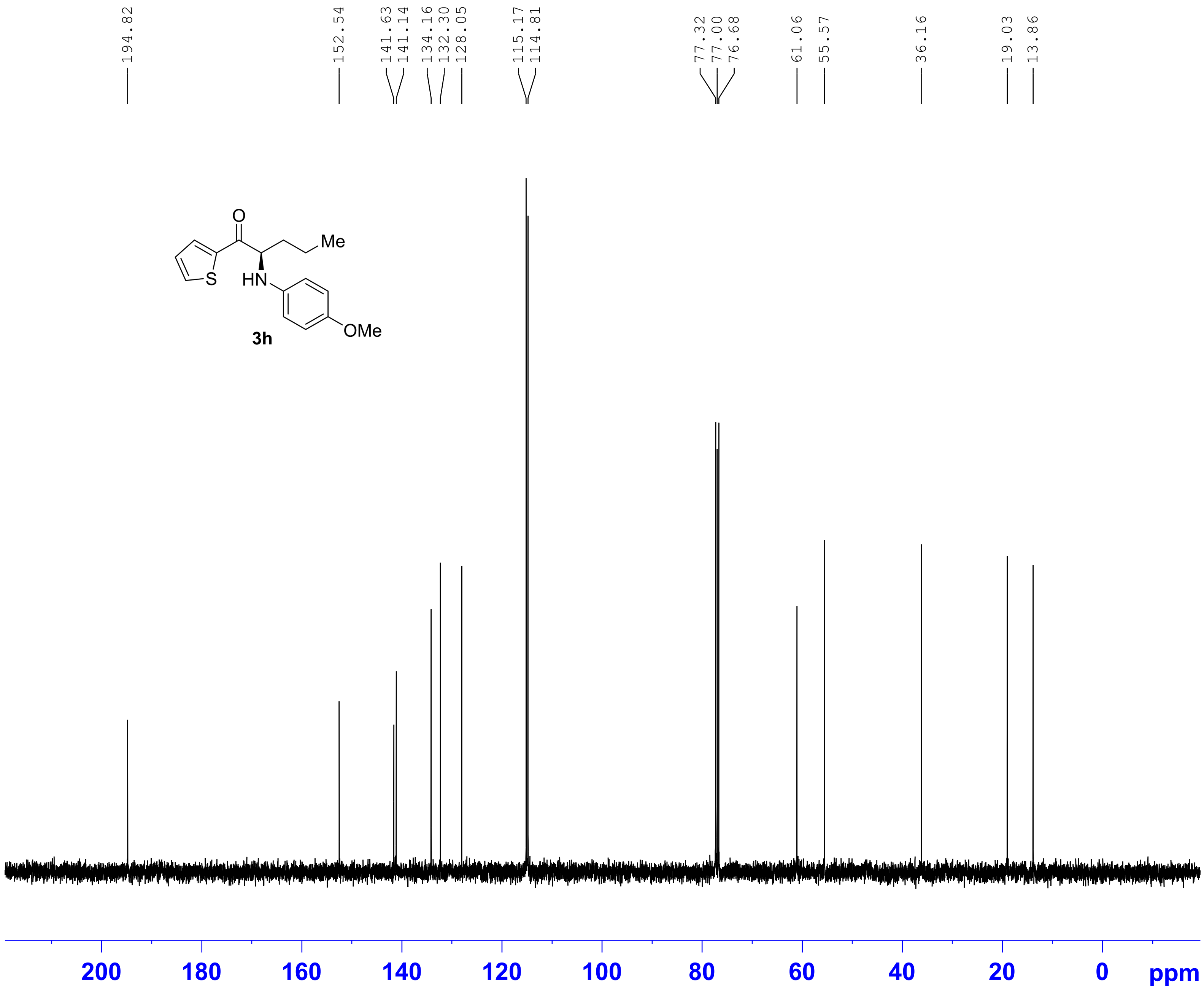

NAME	lzy-04-174-H-2-C
EXPNO	1
PROCNO	1
Date_	20201031
Time_	11.05
INSTRUM	spect
PROBHD	5 mm PABBO BB/
PULPROG	zgpg30
TD	65536
SOLVENT	CDCl3
NS	20
DS	0
SWH	24038.461 Hz
FIDRES	0.366798 Hz
AQ	1.3631988 sec
RG	196.92
DW	20.800 usec
DE	6.50 usec
TE	297.4 K
D1	2.00000000 sec
D11	0.03000000 sec
TD0	1

======== CHANNEL f1 ========

SFO1	100.6228298 MHz
NUC1	13C
P1	9.70 usec
SI	32768
SF	100.6127820 MHz
WDW	EM
SSB	0
LB	1.00 Hz
GB	0
PC	1.40

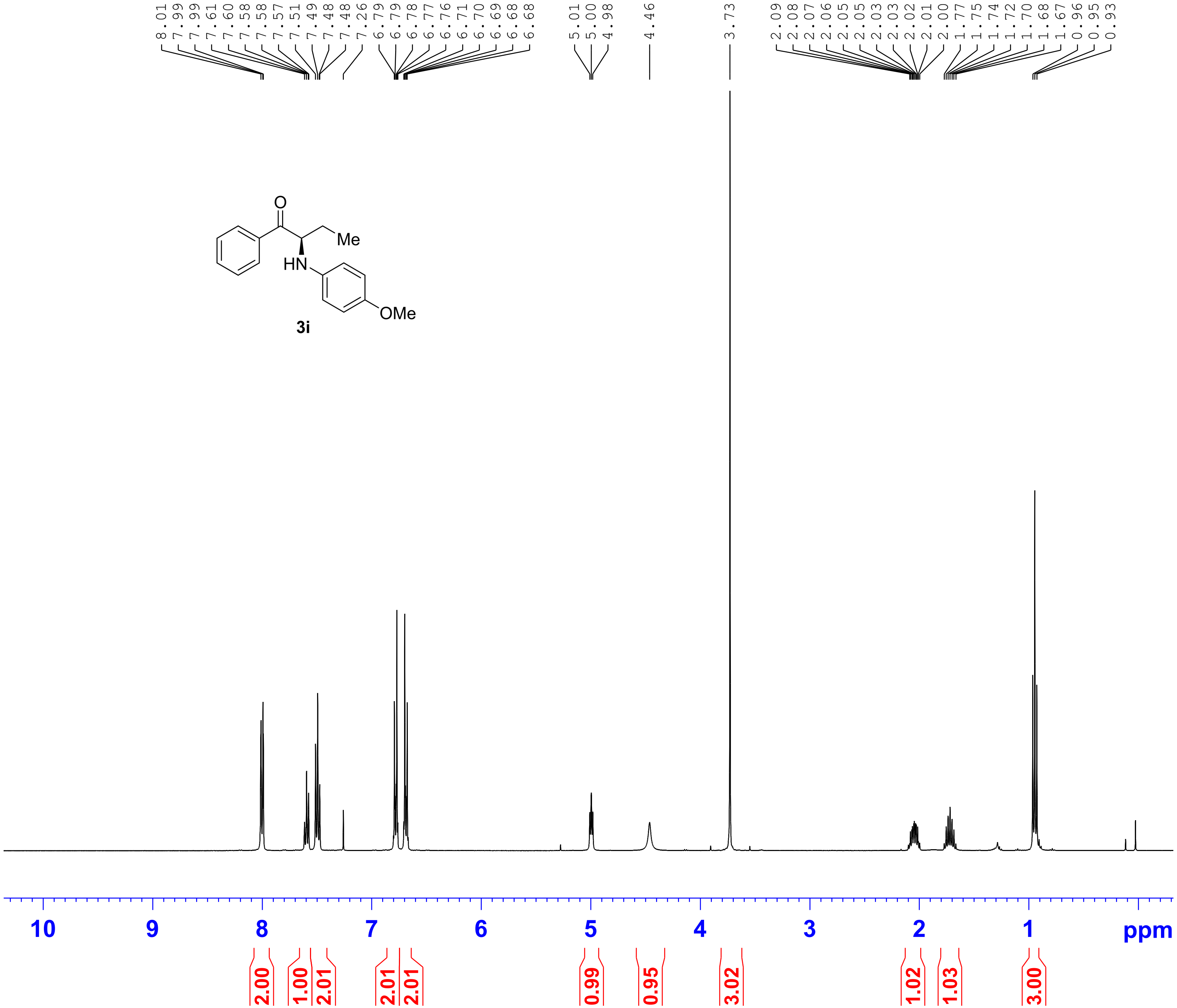

NAME	lzy-04-101
EXPNO	1
PROCNO	1
Date_	20200917
Time	20.47
INSTRUM	spect
PROBHD	5 mm PABBO BB-
PULPROG	zg30
TD	65536
SOLVENT	CDCl3
NS	4
DS	0
SWH	8223.685 Hz
FIDRES	0.125483 Hz
AQ	3.9846387 sec
RG	161
DW	60.800 usec
DE	6.00 usec
TE	294.5 K
D1	1.00000000 sec
TD0	1

======== CHANNEL f1 ========

NUC1	1H
P1	15.80 usec
PL1	-1.00 dB
PL1W	12.17476940 W
SFO1	400.1324710 MHz
SI	32768
SF	400.1300093 MHz
WDW	EM
SSB	0
LB	0.30 Hz
GB	0
PC	1.00

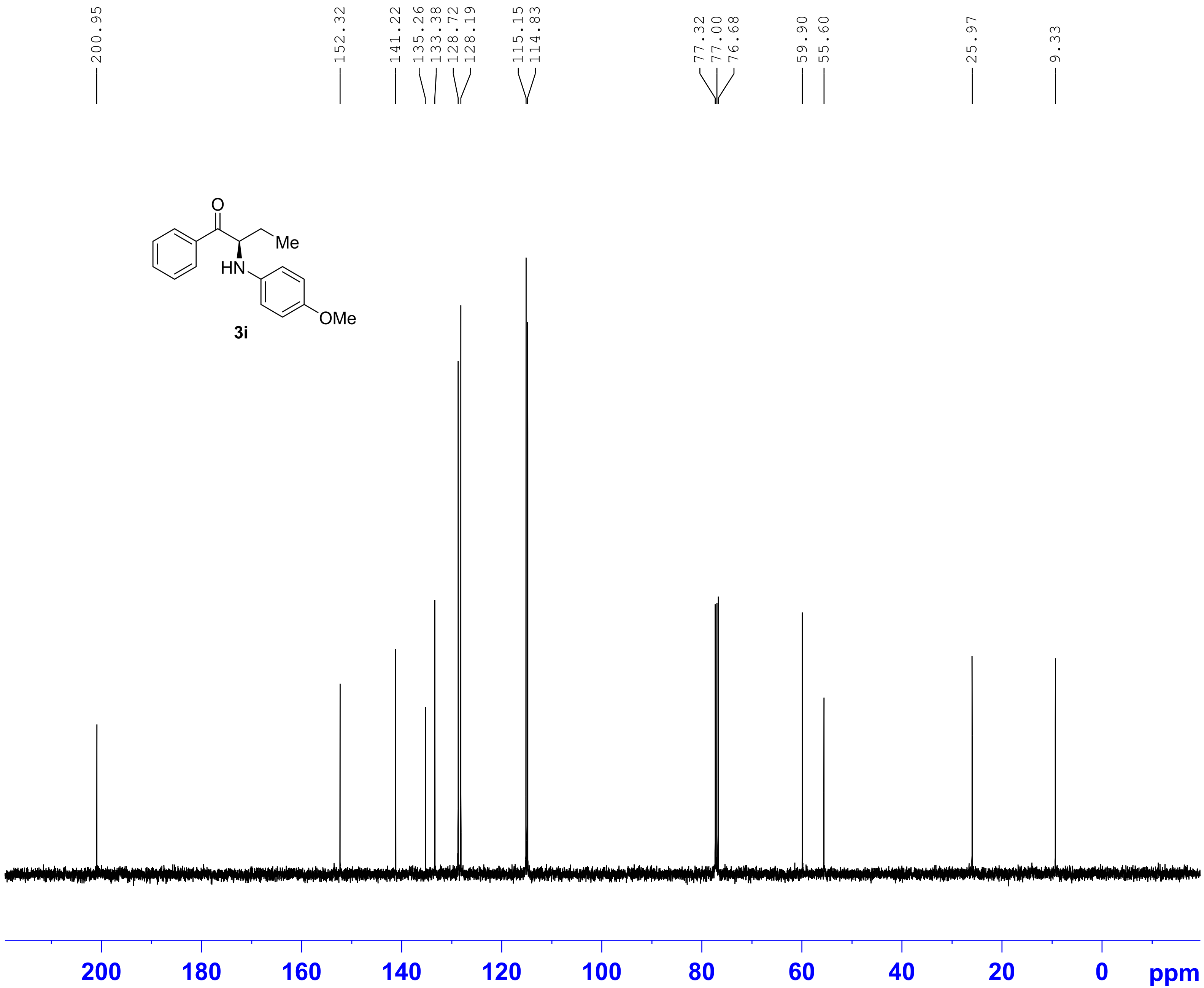

NAME	lzy-04-101-C	
EXPNO	1	
PROCNO	1	
Date_	20200917	
Time	20.49	
INSTRUM	spect	
PROBHD	5 mm PABBO BB-	
PULPROG	zgpg30	
TD	65536	
SOLVENT	CDCl3	
NS	39	
DS	2	
SWH	24038.461	Hz
FIDRES	0.366798	Hz
AQ	1.3631988	sec
RG	114	
DW	20.800	usec
DE	6.00	usec
TE	294.7	K
D1	2.00000000	sec
D11	0.03000000	sec
TD0	1	

======== CHANNEL f1 ========

NUC1	13C	
P1	8.60	usec
PL1	-3.00	dB
PL1W	60.64365387	W
SFO1	100.6228298	MHz

======== CHANNEL f2 ========

CPDPRG2	waltz16	
NUC2	1H	
PCPD2	80.00	usec
PL2	-1.00	dB
PL12	14.39	dB
PL13	18.00	dB
PL2W	12.17476940	W
PL12W	0.35193357	W
PL13W	0.15327126	W
SFO2	400.1316005	MHz
SI	32768	
SF	100.6127841	MHz
WDW	EM	
SSB	0	
LB	1.00	Hz
GB	0	
PC	1.40	

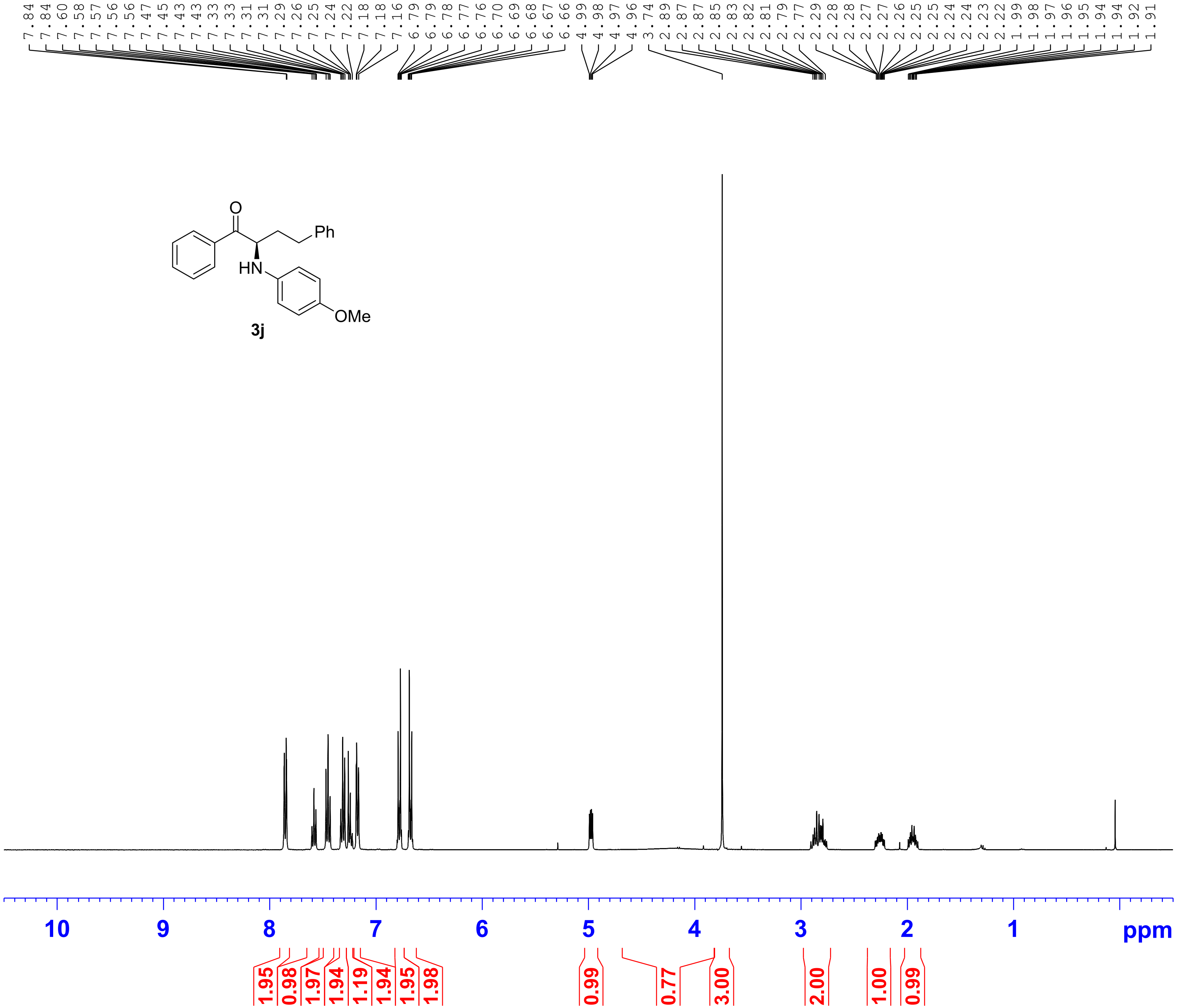

3j

NAME	lzy-04-125-A
EXPNO	2
PROCNO	1
Date_	20200926
Time	21.36
INSTRUM	spect
PROBHD	5 mm PABBO BB-
PULPROG	zg30
TD	65536
SOLVENT	CDCl3
NS	4
DS	0
SWH	8223.685 Hz
FIDRES	0.125483 Hz
AQ	3.9846387 sec
RG	128
DW	60.800 usec
DE	6.00 usec
TE	296.3 K
D1	1.00000000 sec
TD0	1

======== CHANNEL f1 ========

NUC1	1H
P1	15.80 usec
PL1	-1.00 dB
PL1W	12.17476940 W
SFO1	400.1324710 MHz
SI	32768
SF	400.1300096 MHz
WDW	EM
SSB	0
LB	0.30 Hz
GB	0
PC	1.00

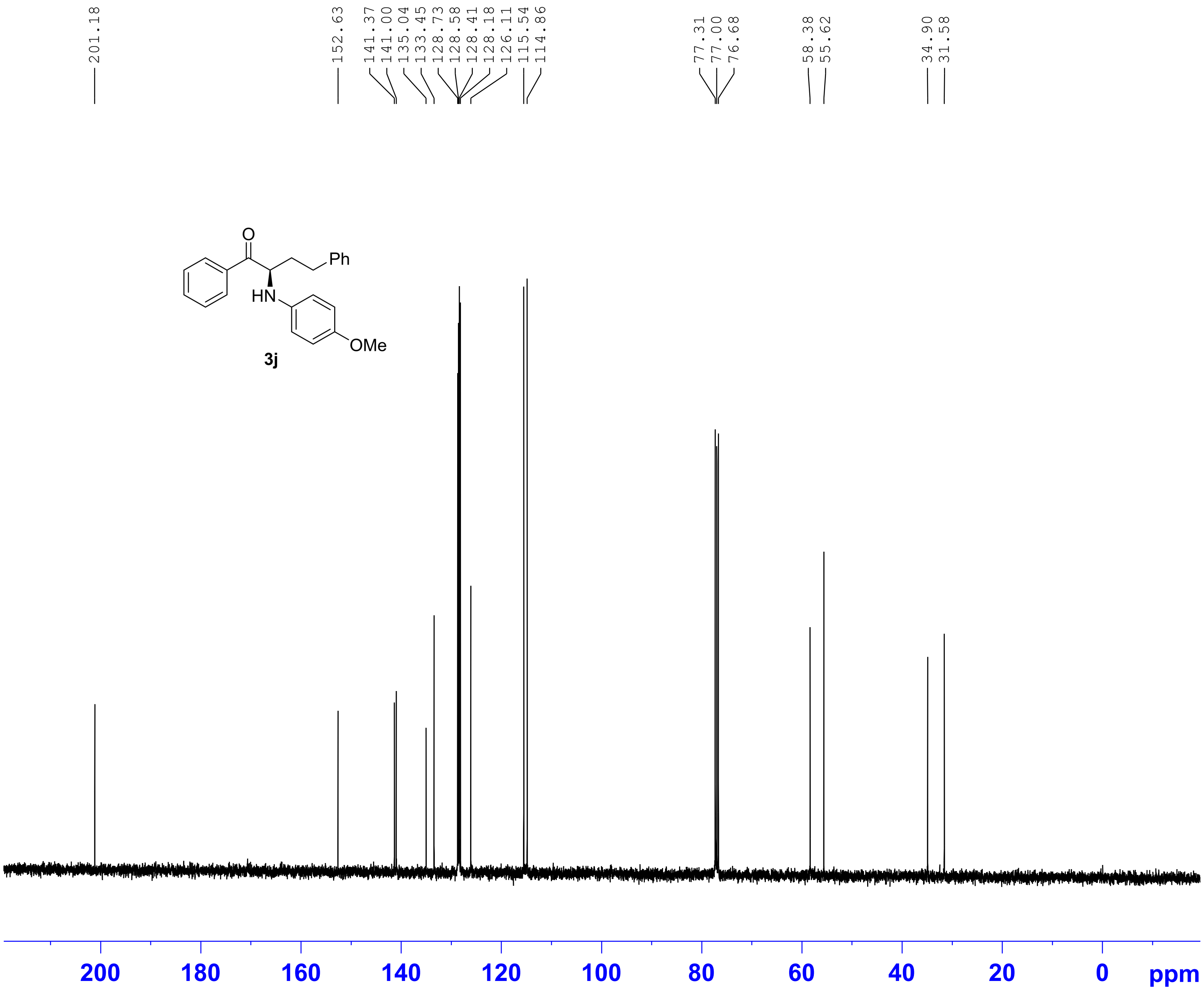

NAME	lzy-04-125-A-C
EXPNO	2
PROCNO	1
Date_	20200926
Time	21.38
INSTRUM	spect
PROBHD	5 mm PABBO BB-
PULPROG	zgpg30
TD	65536
SOLVENT	CDCl3
NS	62
DS	2
SWH	24038.461 Hz
FIDRES	0.366798 Hz
AQ	1.3631988 sec
RG	90.5
DW	20.800 usec
DE	6.00 usec
TE	296.5 K
D1	2.00000000 sec
D11	0.03000000 sec
TD0	1
======== CHANNEL f1 ========	
NUC1	13C
P1	8.60 usec
PL1	-3.00 dB
PL1W	60.64365387 W
SFO1	100.6228298 MHz
======== CHANNEL f2 ========	
CPDPRG2	waltz16
NUC2	1H
PCPD2	80.00 usec
PL2	-1.00 dB
PL12	14.39 dB
PL13	18.00 dB
PL2W	12.17476940 W
PL12W	0.35193357 W
PL13W	0.15327126 W
SFO2	400.1316005 MHz
SI	32768
SF	100.6127817 MHz
WDW	EM
SSB	0
LB	1.00 Hz
GB	0
PC	1.40

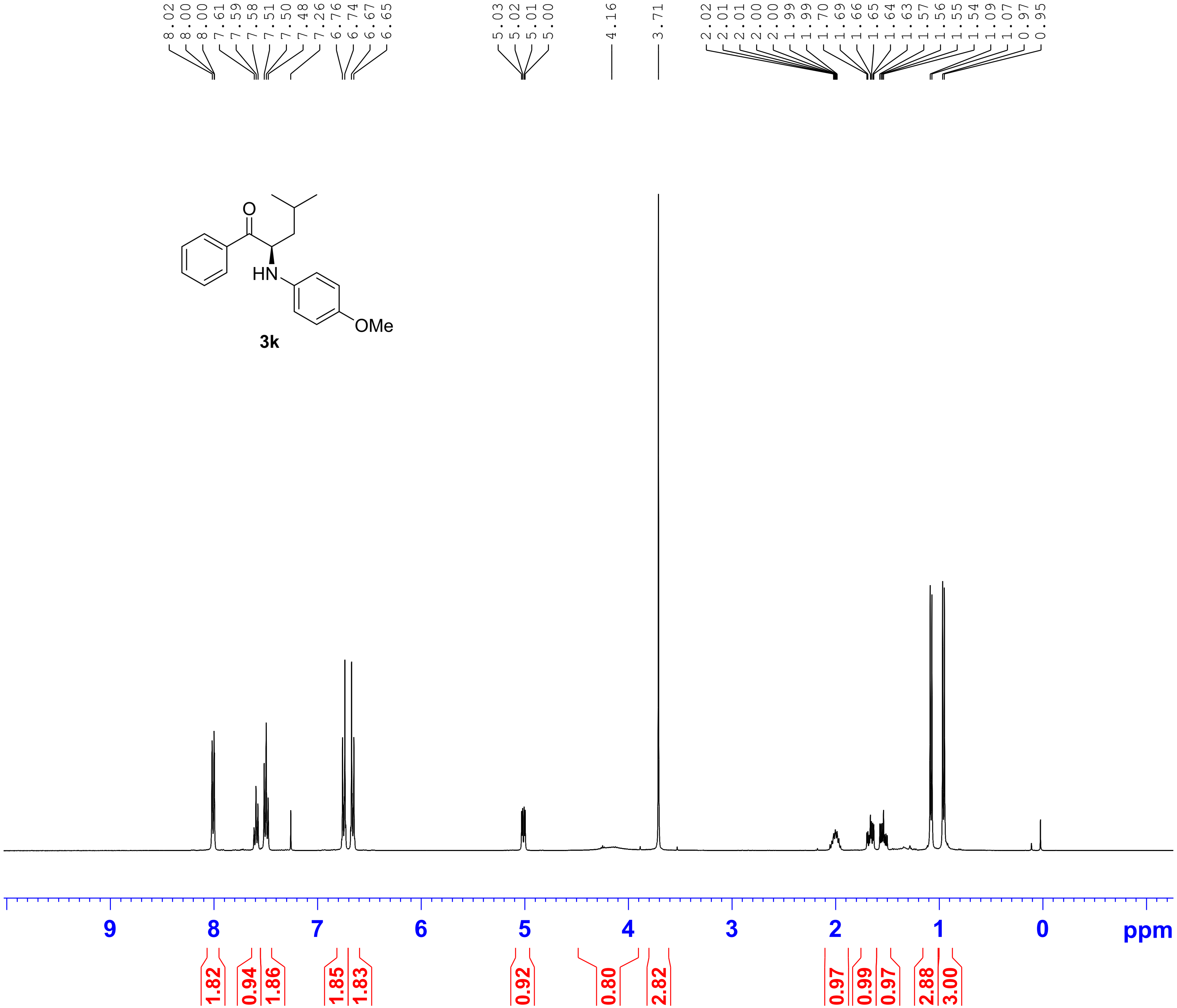


```
NAME          lzy-04-174-R
EXPNO                    1
PROCNO                   1
Date_             20201031
Time                 10.41
INSTRUM              spect
PROBHD   5 mm PABBO BB/
PULPROG               zg30
TD                   65536
SOLVENT              CDCl3
NS                       4
DS                       0
SWH            8012.820 Hz
FIDRES         0.122266 Hz
AQ            4.0894966 sec
RG                   25.32
DW               62.400 usec
DE                 6.50 usec
TE                 297.2 K
D1           1.00000000 sec
TD0                      1

======== CHANNEL f1 ========
SFO1        400.1324710 MHz
NUC1                    1H
P1                14.50 usec
SI                   65536
SF          400.1300098 MHz
WDW                     EM
SSB                      0
LB                 0.30 Hz
GB                       0
PC                    1.00
```

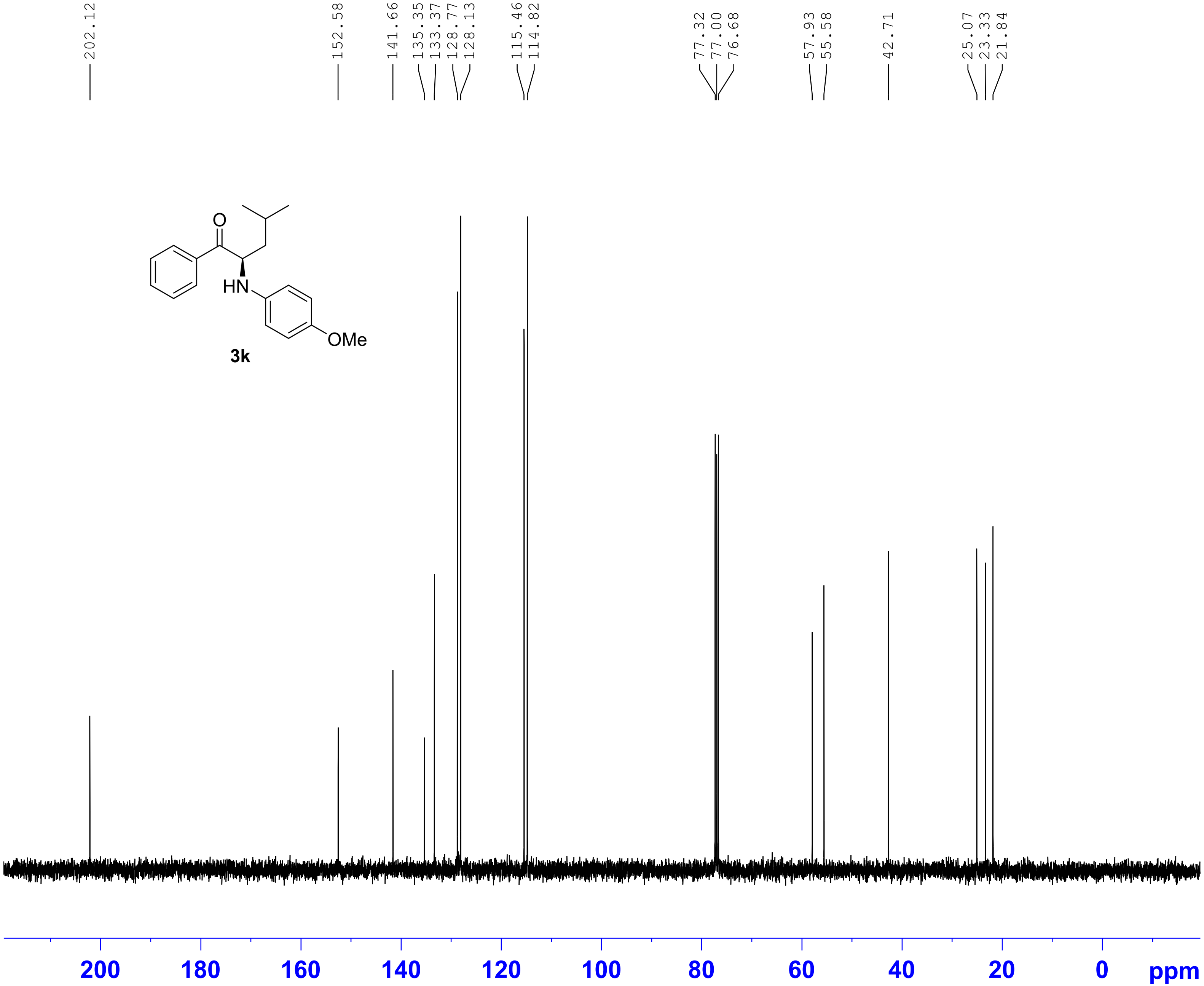

3k

NAME	lzy-04-174-R-C
EXPNO	1
PROCNO	1
Date_	20201031
Time	10.43
INSTRUM	spect
PROBHD	5 mm PABBO BB/
PULPROG	zgpg30
TD	65536
SOLVENT	CDCl3
NS	13
DS	0
SWH	24038.461 Hz
FIDRES	0.366798 Hz
AQ	1.3631988 sec
RG	196.92
DW	20.800 usec
DE	6.50 usec
TE	297.6 K
D1	2.00000000 sec
D11	0.03000000 sec
TD0	1

======== CHANNEL f1 ========

SFO1	100.6228298 MHz
NUC1	13C
P1	9.70 usec
SI	32768
SF	100.6127813 MHz
WDW	EM
SSB	0
LB	1.00 Hz
GB	0
PC	1.40

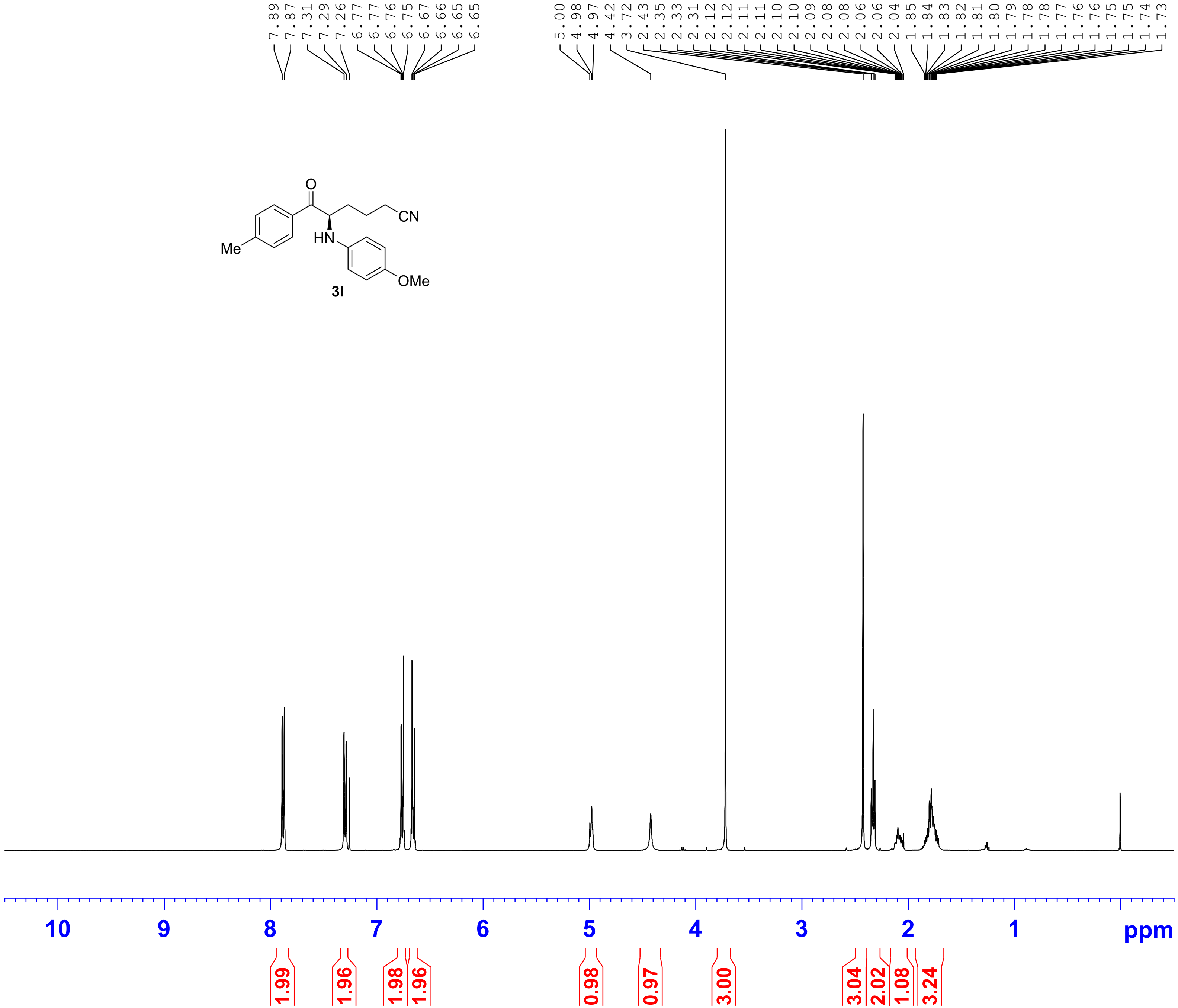

3l

NAME	lzy-04-172-1023
EXPNO	1
PROCNO	1
Date_	20201023
Time	11.03
INSTRUM	spect
PROBHD	5 mm PABBO BB/
PULPROG	zg30
TD	65536
SOLVENT	CDCl3
NS	4
DS	0
SWH	8012.820 Hz
FIDRES	0.122266 Hz
AQ	4.0894966 sec
RG	34.77
DW	62.400 usec
DE	6.50 usec
TE	296.9 K
D1	1.00000000 sec
TD0	1

======== CHANNEL f1 ========

SFO1	400.1324710 MHz
NUC1	1H
P1	14.50 usec
SI	65536
SF	400.1300101 MHz
WDW	EM
SSB	0
LB	0.30 Hz
GB	0
PC	1.00

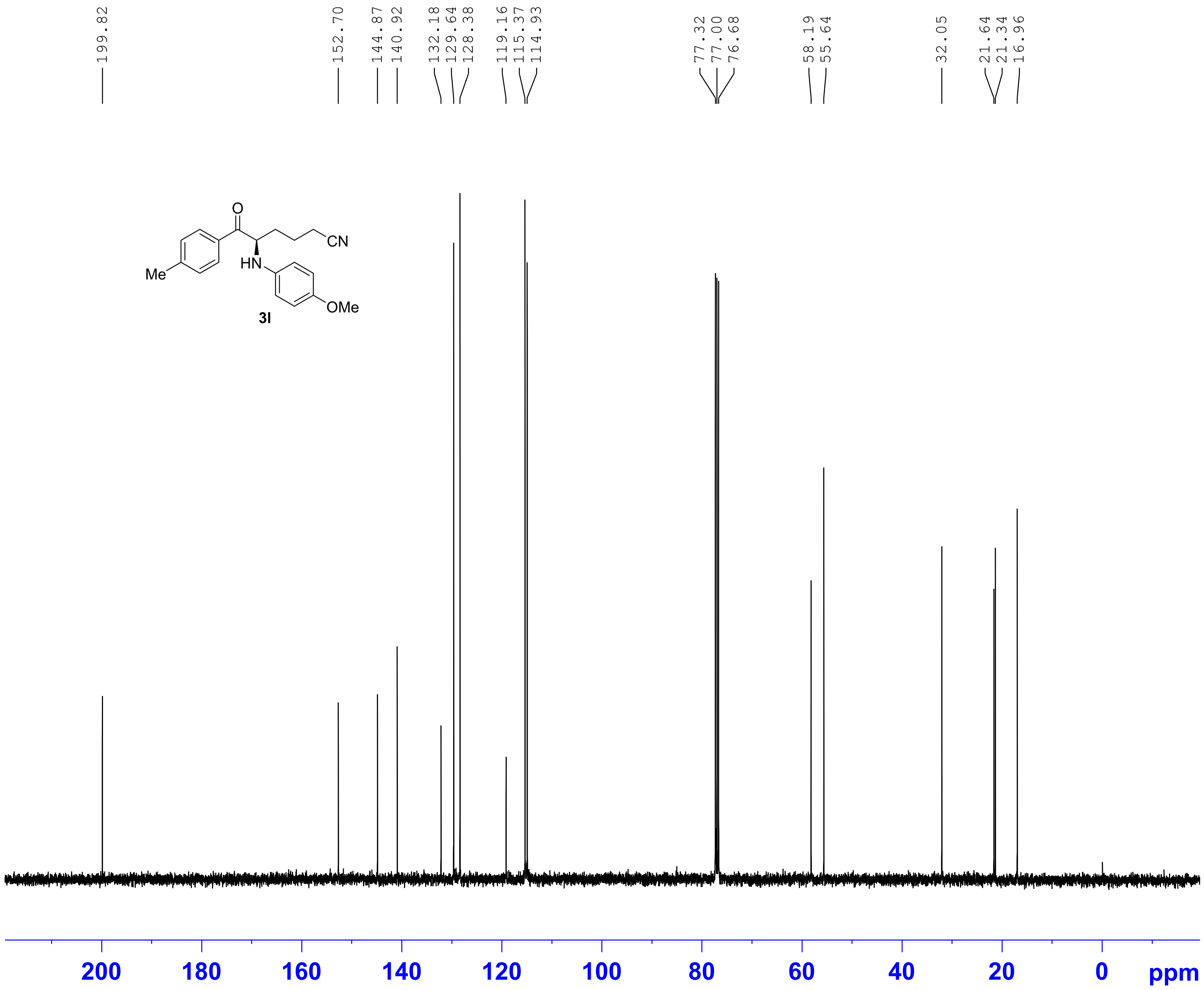

NAME	lzy-04-172-1023-C
EXPNO	1
PROCNO	1
Date_	20201023
Time_	11.11
INSTRUM	spect
PROBHD	5 mm PABBO BB/
PULPROG	zgpg30
TD	65536
SOLVENT	CDCl3
NS	112
DS	2
SWH	24038.461 Hz
FIDRES	0.366798 Hz
AQ	1.3631988 sec
RG	196.92
DW	20.800 usec
DE	6.50 usec
TE	297.9 K
D1	2.00000000 sec
D11	0.03000000 sec
TD0	1

======== CHANNEL f1 ========

SFO1	100.6228298 MHz
NUC1	13C
P1	9.70 usec
SI	32768
SF	100.6127795 MHz
WDW	EM
SSB	0
LB	1.00 Hz
GB	0
PC	1.40

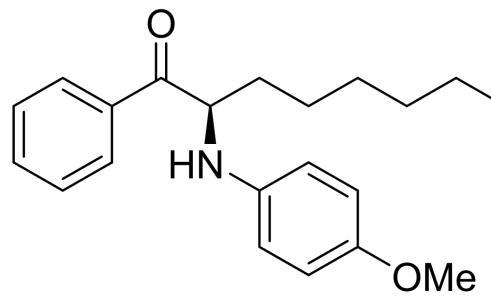

3m

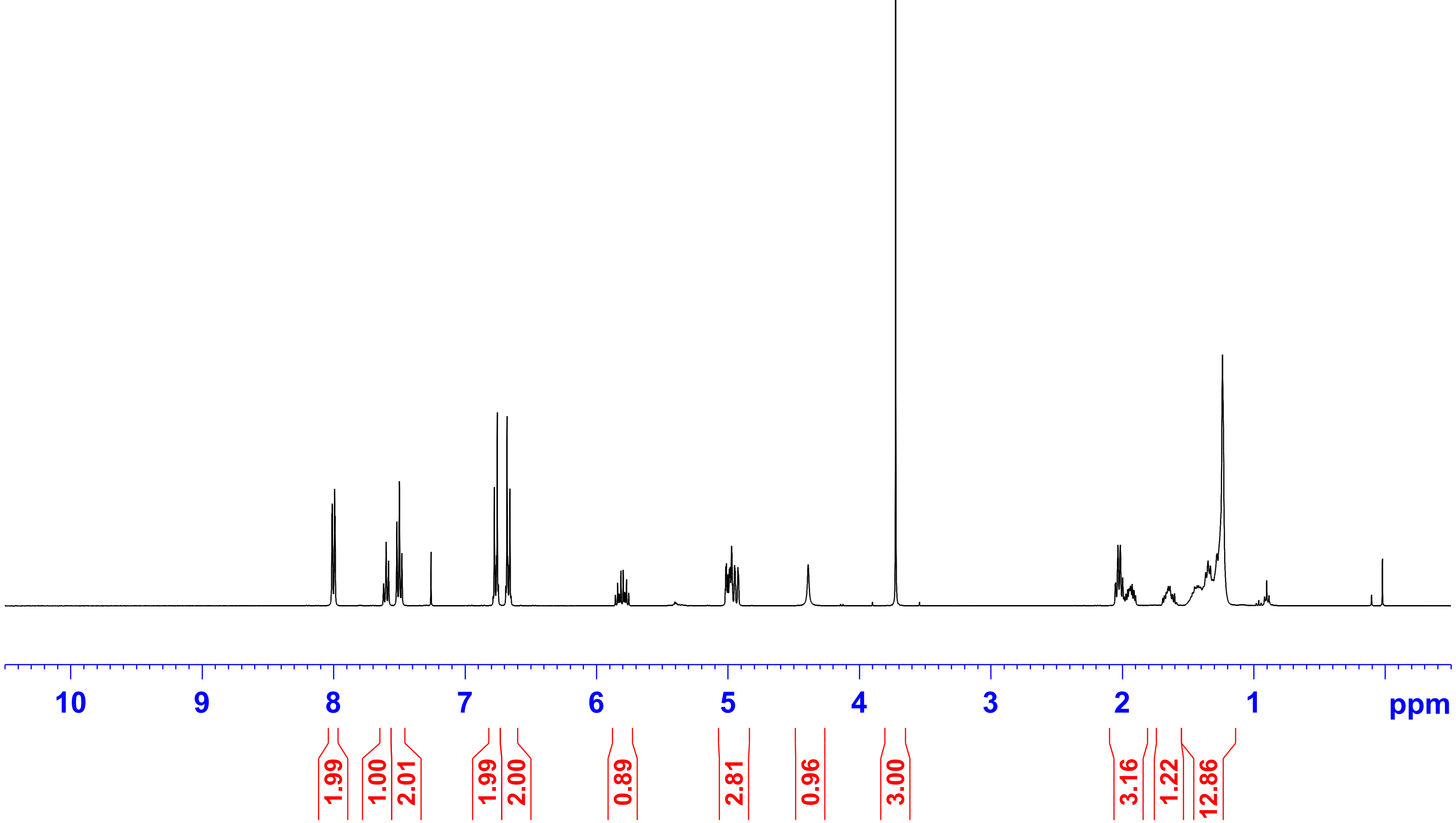

NAME	lzy-05-159-E
EXPNO	1
PROCNO	1
Date_	20210203
Time	19.20
INSTRUM	spect
PROBHD	5 mm DUL 13C-1
PULPROG	zg30
TD	65536
SOLVENT	CDCl3
NS	4
DS	0
SWH	8223.685 Hz
FIDRES	0.125483 Hz
AQ	3.9846387 sec
RG	64
DW	60.800 usec
DE	6.00 usec
TE	293.7 K
D1	1.00000000 sec
TD0	1

======== CHANNEL f1 ========

NUC1	1H
P1	15.80 usec
PL1	-1.00 dB
PL1W	12.17476940 W
SFO1	400.1324710 MHz
SI	32768
SF	400.1300094 MHz
WDW	EM
SSB	0
LB	0.30 Hz
GB	0
PC	1.00

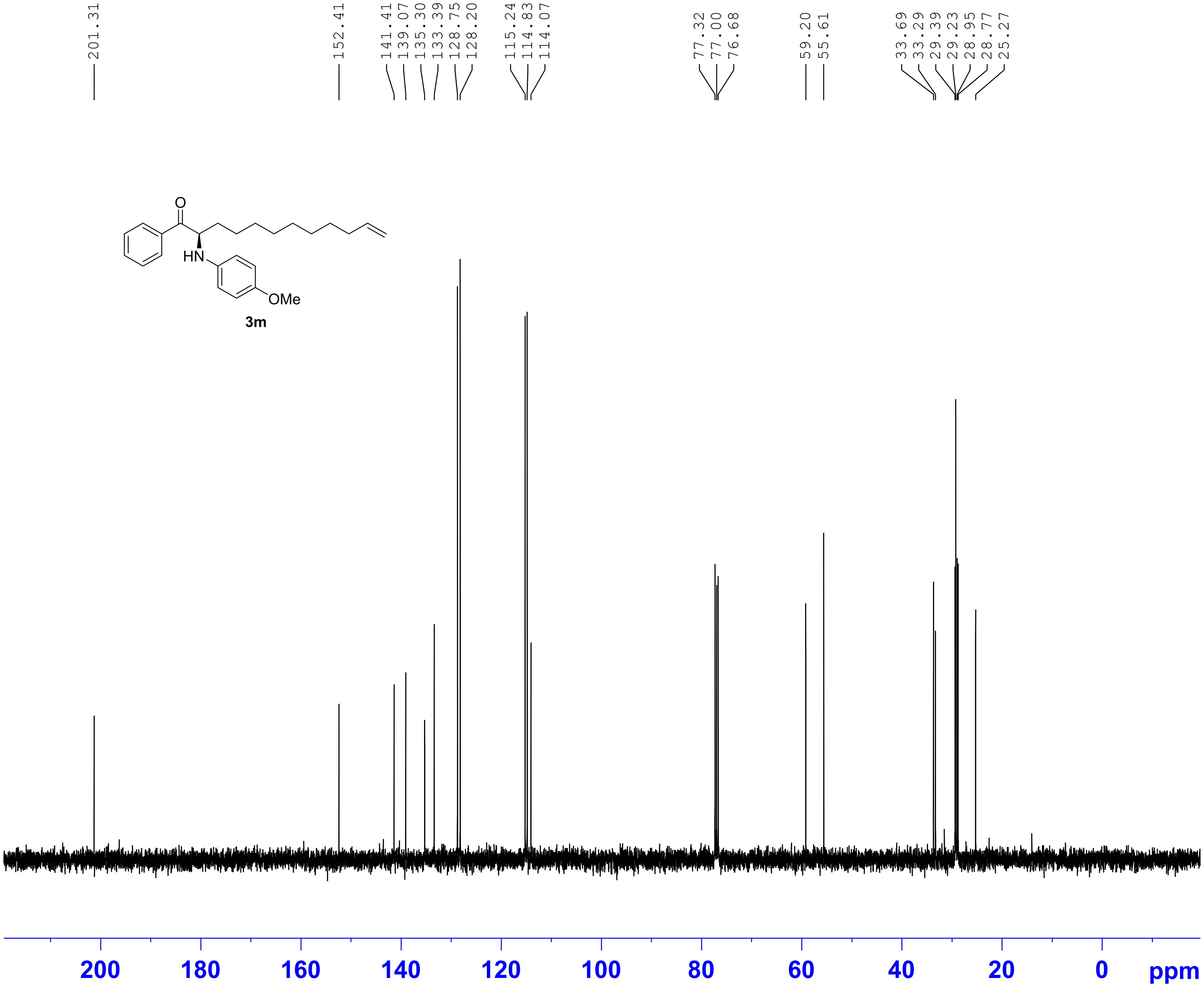


```
NAME        lzy-05-159-E-C
EXPNO                    1
PROCNO                   1
Date_             20210203
Time                 19.22
INSTRUM              spect
PROBHD     5 mm DUL 13C-1
PULPROG            zgpg30
TD                  65536
SOLVENT             CDCl3
NS                     43
DS                      0
SWH             24038.461 Hz
FIDRES           0.366798 Hz
AQ              1.3631988 sec
RG                   2050
DW                 20.800 usec
DE                   6.00 usec
TE                  293.8 K
D1             2.00000000 sec
D11            0.03000000 sec
TD0                     1

======== CHANNEL f1 ========
NUC1                  13C
P1                  40.00 usec
PL1                 -3.00 dB
PL1W          60.64365387 W
SFO1          100.6228298 MHz

======== CHANNEL f2 ========
CPDPRG2           waltz16
NUC2                   1H
PCPD2               80.00 usec
PL2                 -1.00 dB
PL12                14.39 dB
PL13                18.00 dB
PL2W          12.17476940 W
PL12W          0.35193357 W
PL13W          0.15327126 W
SFO2          400.1316005 MHz
SI                  32768
SF            100.6127807 MHz
WDW                    EM
SSB                     0
LB                   1.00 Hz
GB                      0
PC                   1.40
```

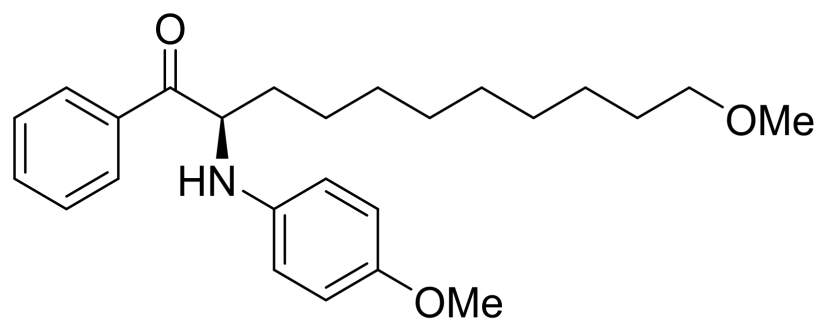

3n

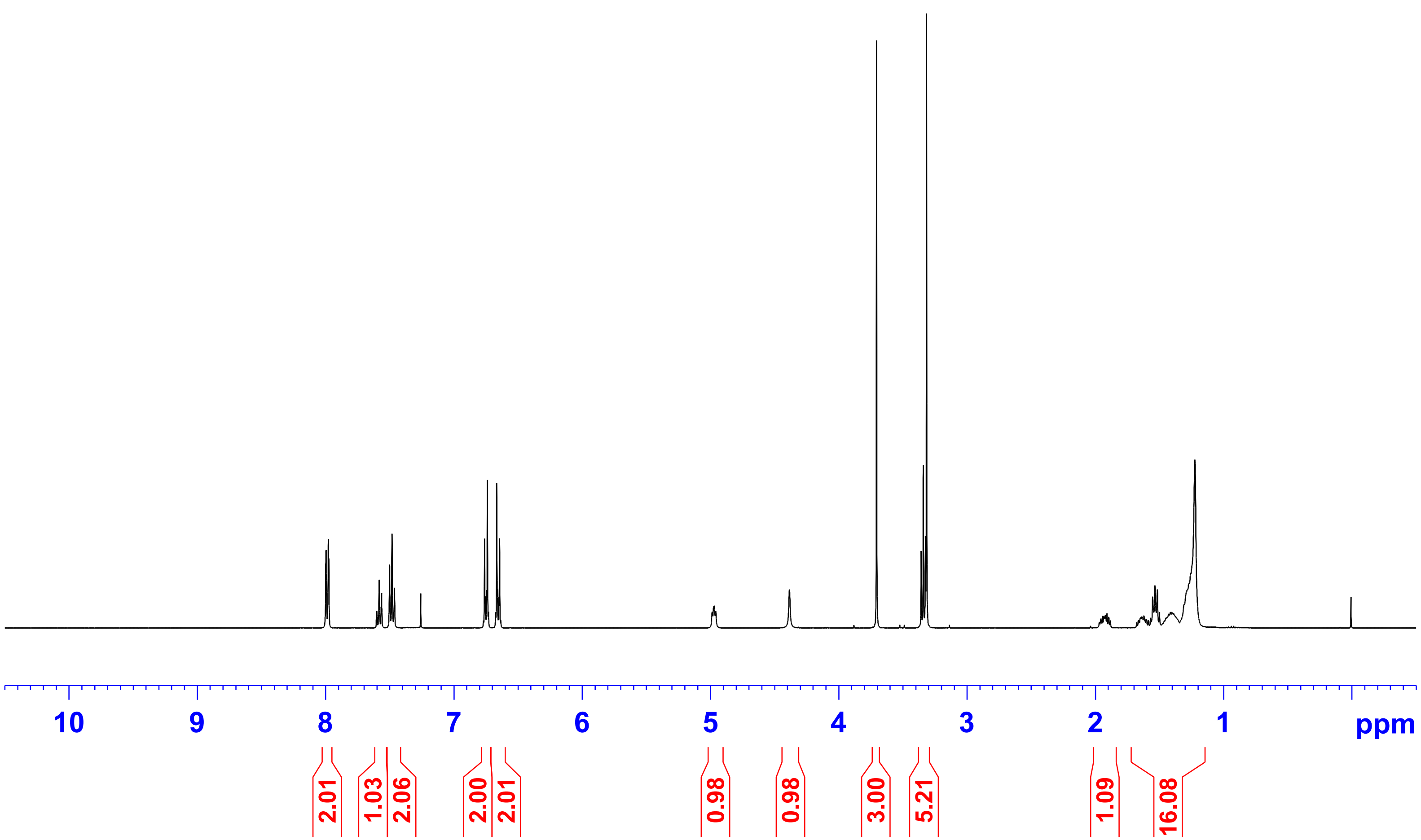

NAME	lzy-05-159-I
EXPNO	1
PROCNO	1
Date_	20210203
Time	19.13
INSTRUM	spect
PROBHD	5 mm DUL 13C-1
PULPROG	zg30
TD	65536
SOLVENT	CDCl3
NS	4
DS	0
SWH	8223.685 Hz
FIDRES	0.125483 Hz
AQ	3.9846387 sec
RG	64
DW	60.800 usec
DE	6.00 usec
TE	293.5 K
D1	1.00000000 sec
TD0	1

======== CHANNEL f1 ========

NUC1	1H
P1	15.80 usec
PL1	-1.00 dB
PL1W	12.17476940 W
SFO1	400.1324710 MHz
SI	32768
SF	400.1300094 MHz
WDW	EM
SSB	0
LB	0.30 Hz
GB	0
PC	1.00

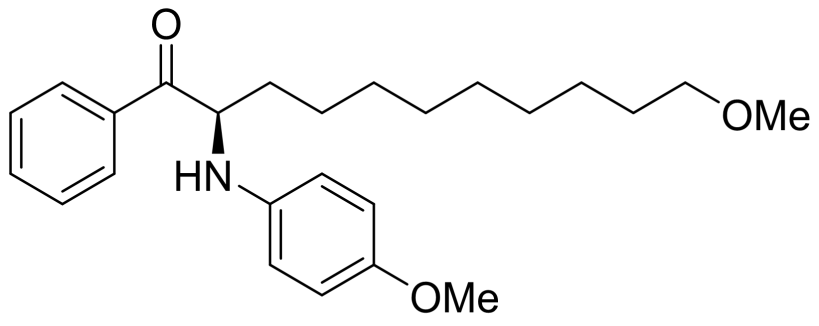

3n

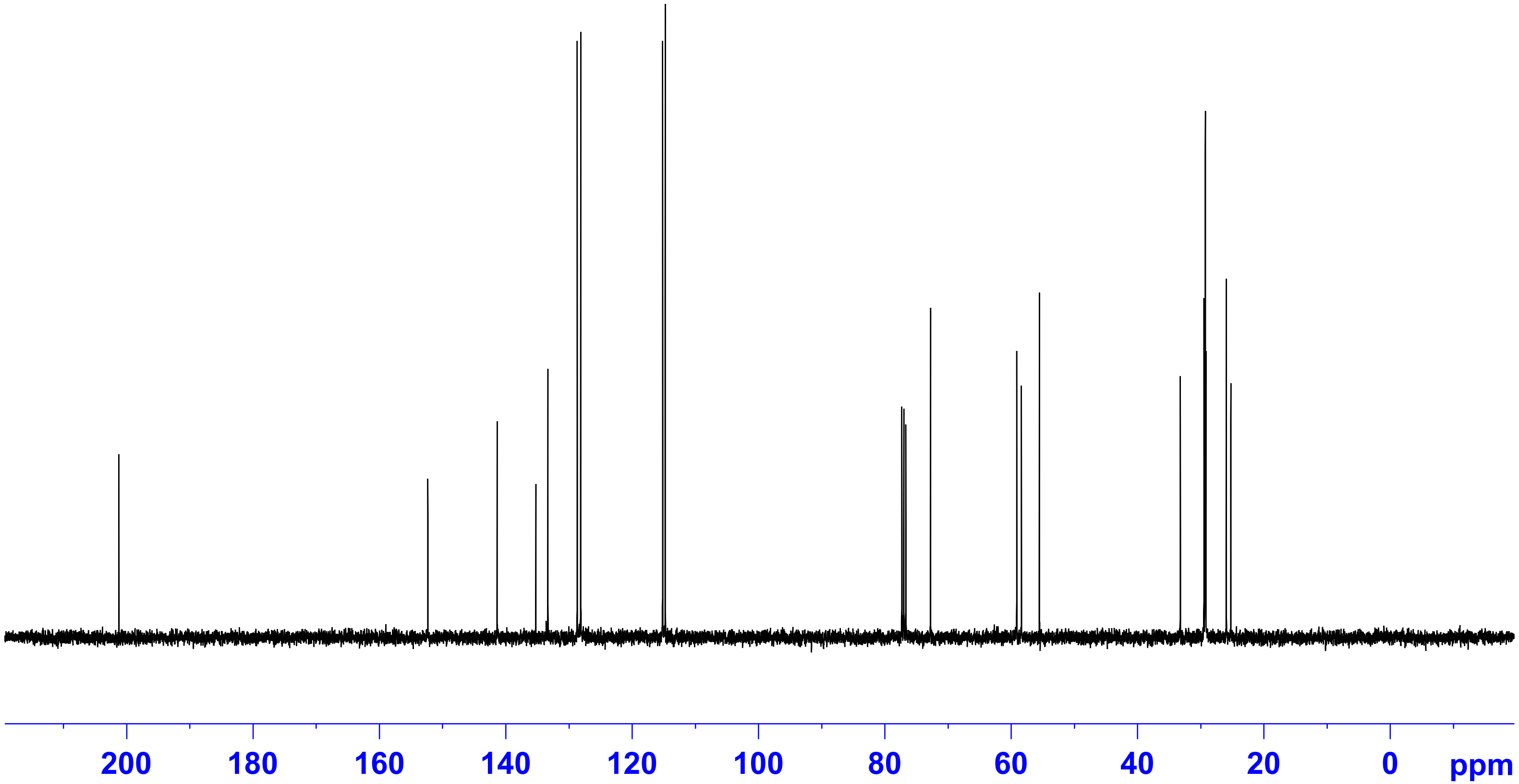

NAME	lzy-05-159-I-C
EXPNO	1
PROCNO	1
Date_	20210203
Time_	19.17
INSTRUM	spect
PROBHD	5 mm DUL 13C-1
PULPROG	zgpg30
TD	65536
SOLVENT	CDCl3
NS	62
DS	0
SWH	24038.461 Hz
FIDRES	0.366798 Hz
AQ	1.3631988 sec
RG	2050
DW	20.800 usec
DE	6.00 usec
TE	293.9 K
D1	2.00000000 sec
D11	0.03000000 sec
TD0	1
======== CHANNEL f1 ========	
NUC1	13C
P1	40.00 usec
PL1	-3.00 dB
PL1W	60.64365387 W
SFO1	100.6228298 MHz
======== CHANNEL f2 ========	
CPDPRG2	waltz16
NUC2	1H
PCPD2	80.00 usec
PL2	-1.00 dB
PL12	14.39 dB
PL13	18.00 dB
PL2W	12.17476940 W
PL12W	0.35193357 W
PL13W	0.15327126 W
SFO2	400.1316005 MHz
SI	32768
SF	100.6127846 MHz
WDW	EM
SSB	0
LB	1.00 Hz
GB	0
PC	1.40

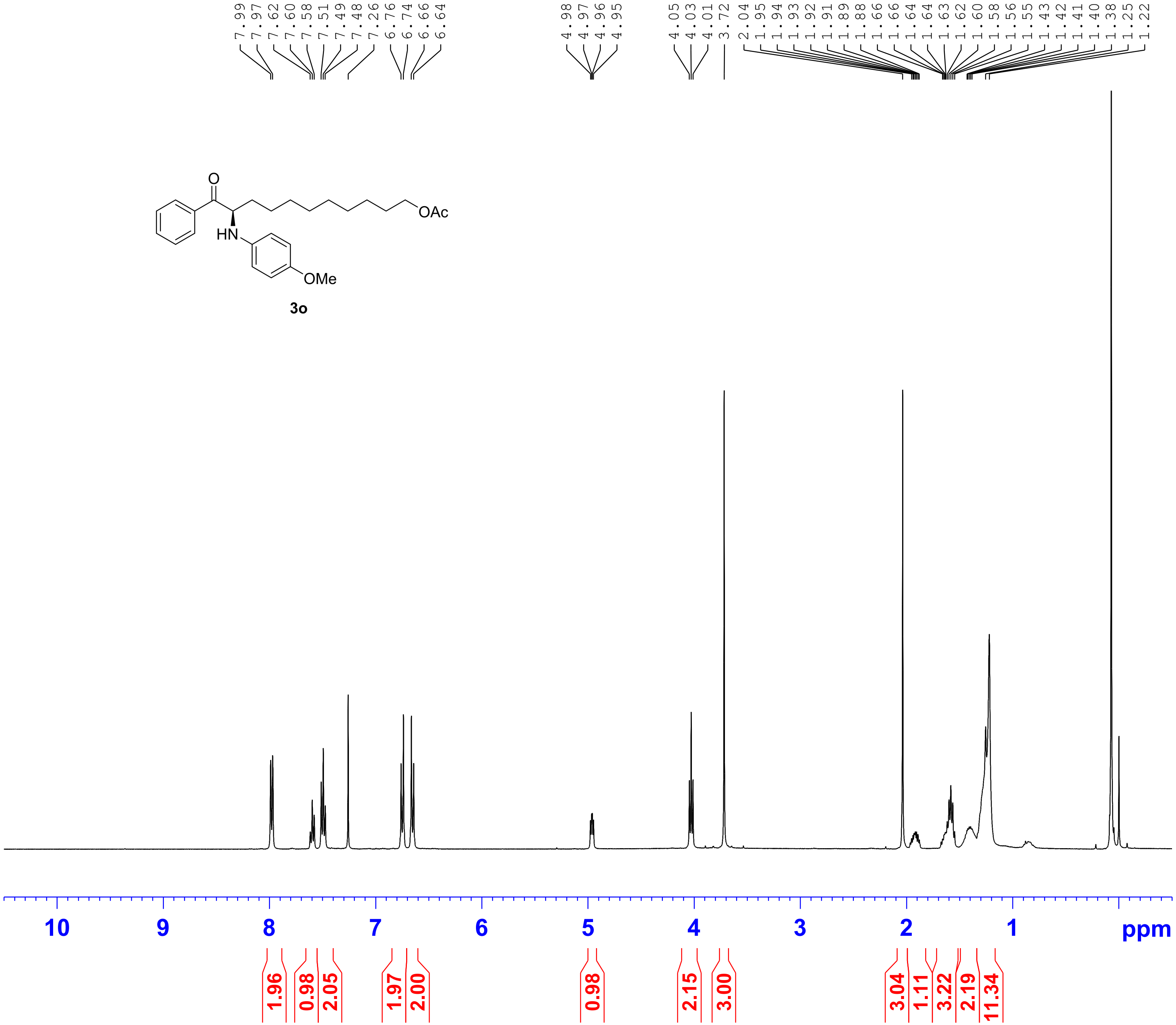

NAME	lzy-05-159-OAc
EXPNO	1
PROCNO	1
Date_	20210311
Time_	19.52
INSTRUM	spect
PROBHD	5 mm PABBO BB/
PULPROG	zg30
TD	65536
SOLVENT	CDCl3
NS	4
DS	0
SWH	8012.820 Hz
FIDRES	0.122266 Hz
AQ	4.0894966 sec
RG	49.32
DW	62.400 usec
DE	6.50 usec
TE	296.2 K
D1	1.00000000 sec
TD0	1

======== CHANNEL f1 ========

SFO1	400.1324710 MHz
NUC1	1H
P1	14.50 usec
SI	65536
SF	400.1300100 MHz
WDW	EM
SSB	0
LB	0.30 Hz
GB	0
PC	1.00

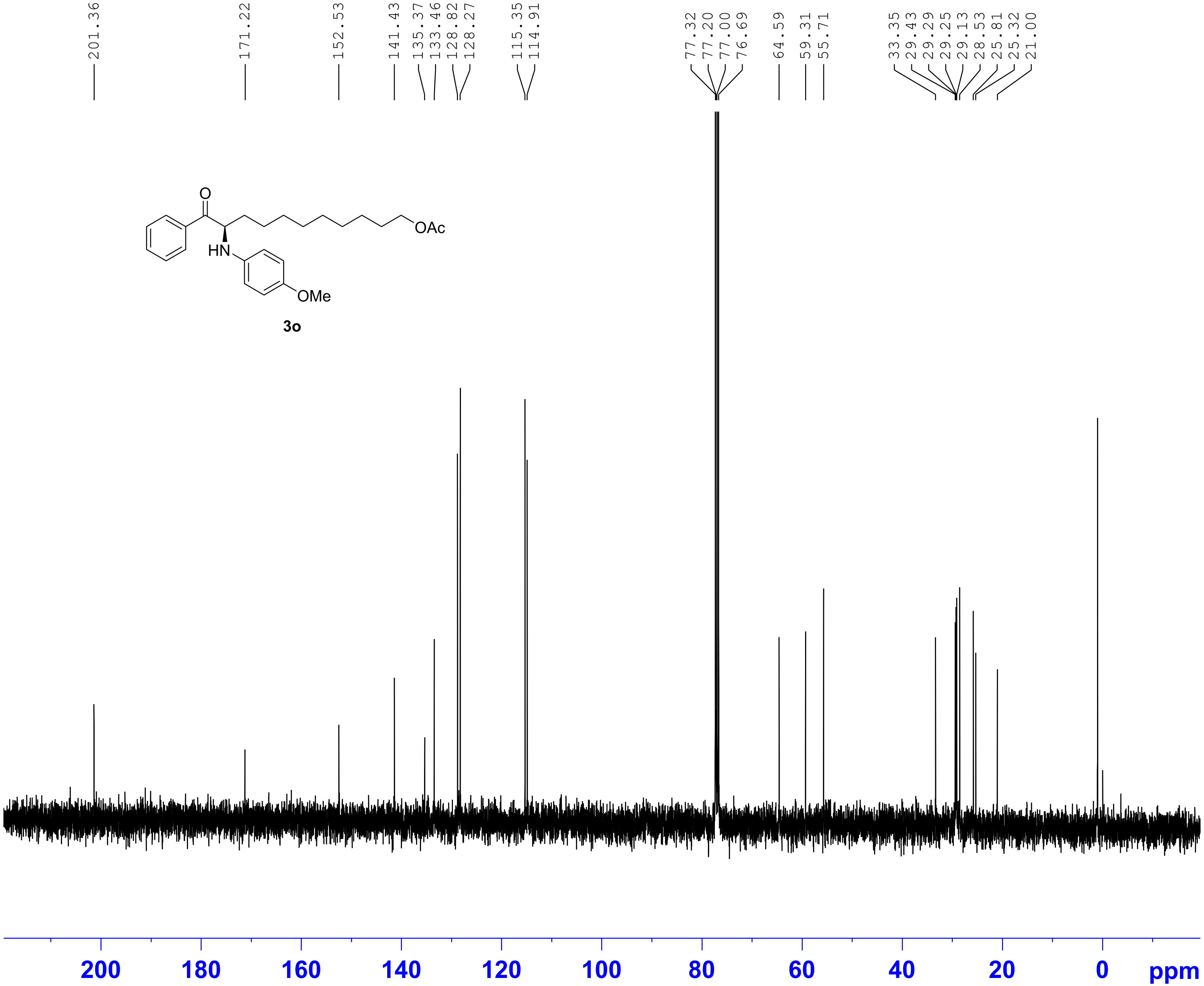

NAME	lzy-05-159-OAc-C
EXPNO	1
PROCNO	1
Date_	20210311
Time	19.59
INSTRUM	spect
PROBHD	5 mm PABBO BB/
PULPROG	zgpg30
TD	65536
SOLVENT	CDCl3
NS	112
DS	0
SWH	24038.461 Hz
FIDRES	0.366798 Hz
AQ	1.3631988 sec
RG	196.92
DW	20.800 usec
DE	6.50 usec
TE	297.3 K
D1	2.00000000 sec
D11	0.03000000 sec
TD0	1

======== CHANNEL f1 ========

SFO1	100.6228298 MHz
NUC1	13C
P1	9.70 usec
SI	32768
SF	100.6127721 MHz
WDW	EM
SSB	0
LB	1.00 Hz
GB	0
PC	1.40

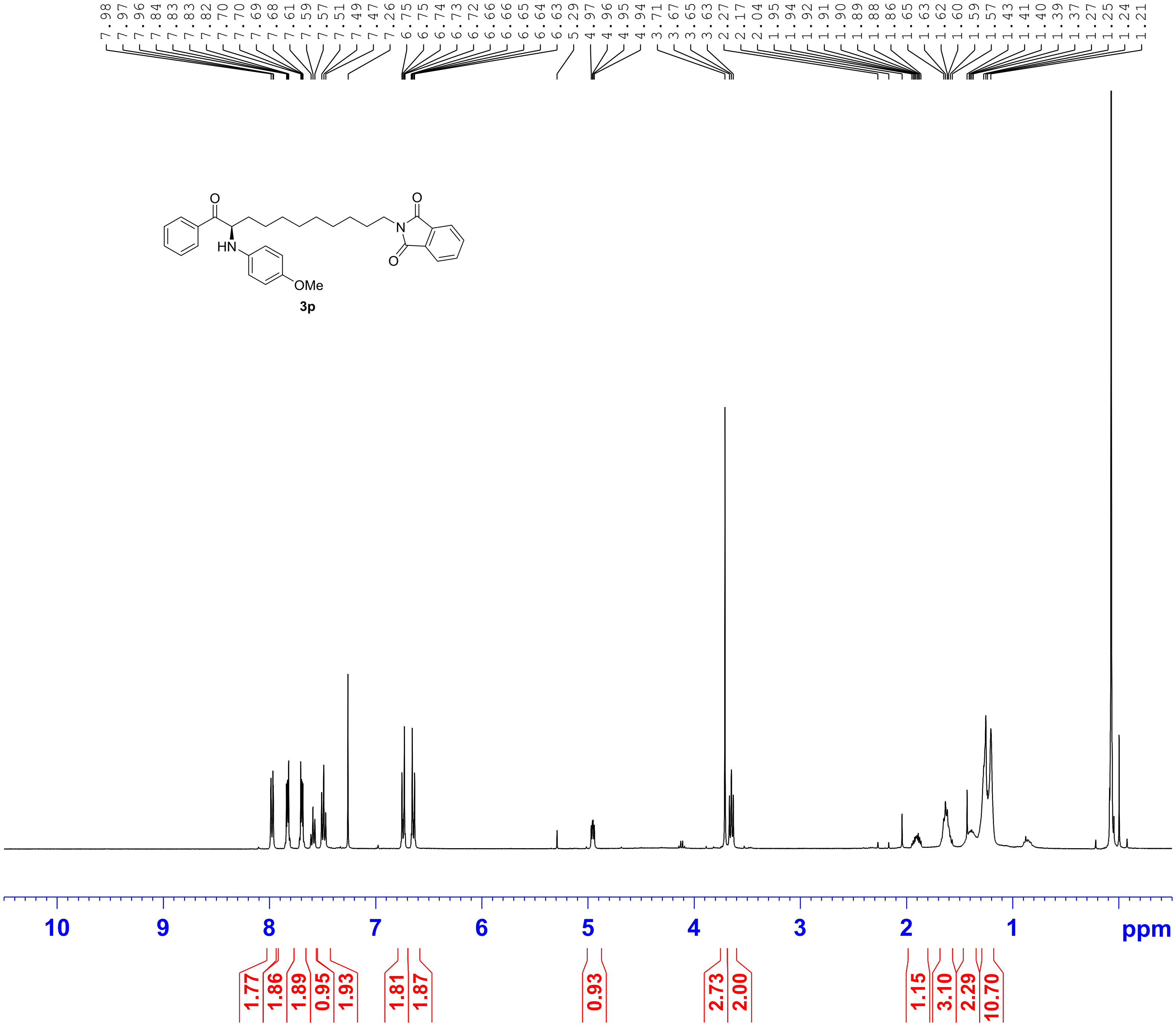

3p

NAME	lzy-05-159-H-0221
EXPNO	1
PROCNO	1
Date_	20210221
Time_	19.10
INSTRUM	spect
PROBHD	5 mm DUL 13C-1
PULPROG	zg30
TD	65536
SOLVENT	CDCl3
NS	4
DS	0
SWH	8223.685 Hz
FIDRES	0.125483 Hz
AQ	3.9846387 sec
RG	203
DW	60.800 usec
DE	6.00 usec
TE	294.7 K
D1	1.00000000 sec
TD0	1

======== CHANNEL f1 ========

NUC1	1H
P1	15.80 usec
PL1	-1.00 dB
PL1W	12.17476940 W
SFO1	400.1324710 MHz
SI	32768
SF	400.1300096 MHz
WDW	EM
SSB	0
LB	0.30 Hz
GB	0
PC	1.00

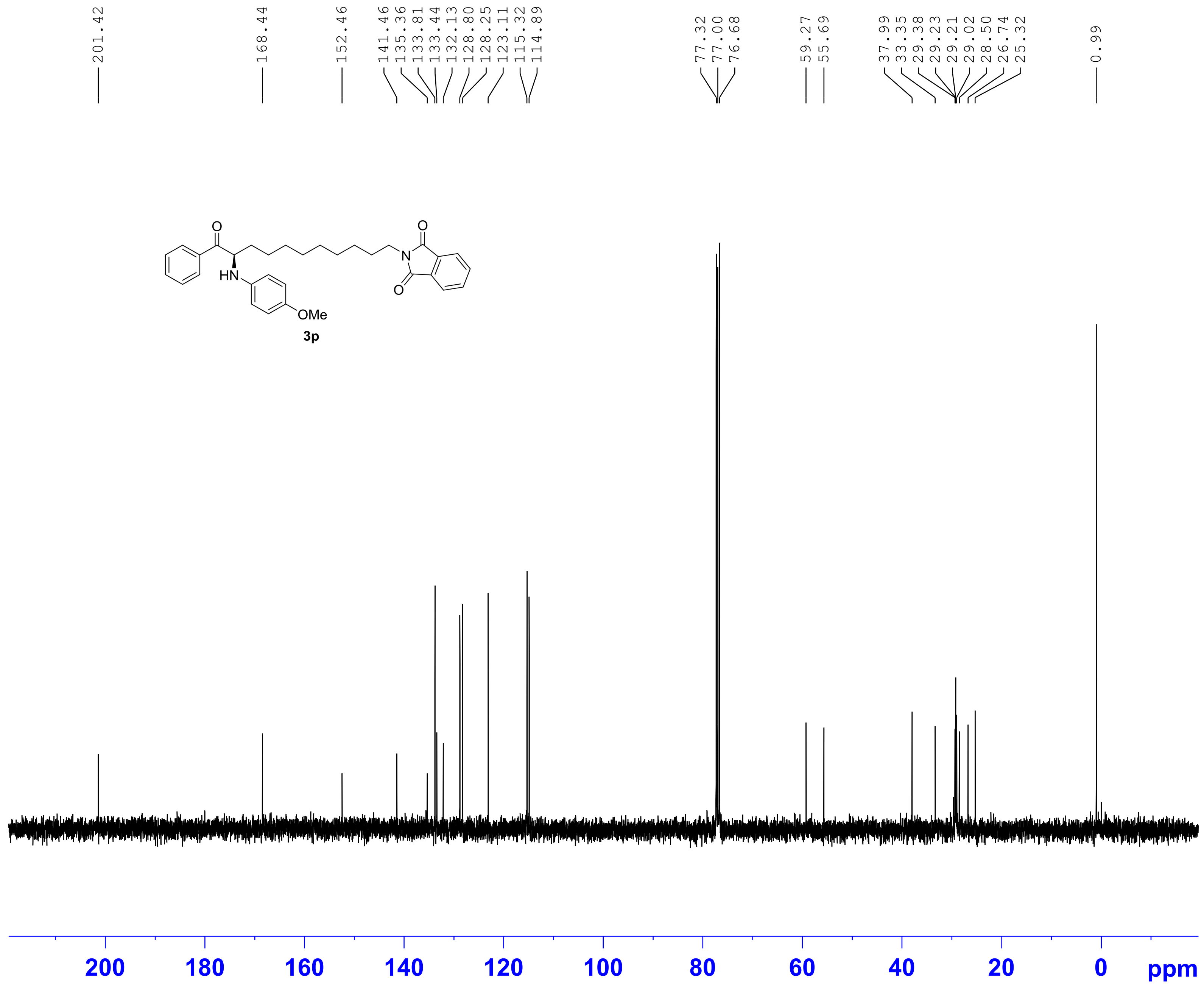

3p

NAME	lzy-05-159-H-0221-C
EXPNO	1
PROCNO	1
Date_	20210221
Time	19.13
INSTRUM	spect
PROBHD	5 mm DUL 13C-1
PULPROG	zgpg30
TD	65536
SOLVENT	CDCl3
NS	200
DS	1
SWH	24038.461 Hz
FIDRES	0.366798 Hz
AQ	1.3631988 sec
RG	2050
DW	20.800 usec
DE	6.00 usec
TE	294.9 K
D1	2.00000000 sec
D11	0.03000000 sec
TD0	1

======== CHANNEL f1 ========

NUC1	13C
P1	40.00 usec
PL1	-3.00 dB
PL1W	60.64365387 W
SFO1	100.6228298 MHz

======== CHANNEL f2 ========

CPDPRG2	waltz16
NUC2	1H
PCPD2	80.00 usec
PL2	-1.00 dB
PL12	14.39 dB
PL13	18.00 dB
PL2W	12.17476940 W
PL12W	0.35193357 W
PL13W	0.15327126 W
SFO2	400.1316005 MHz
SI	32768
SF	100.6127735 MHz
WDW	EM
SSB	0
LB	1.00 Hz
GB	0
PC	1.40

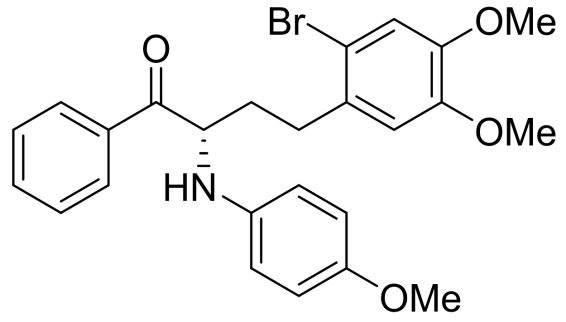

3q

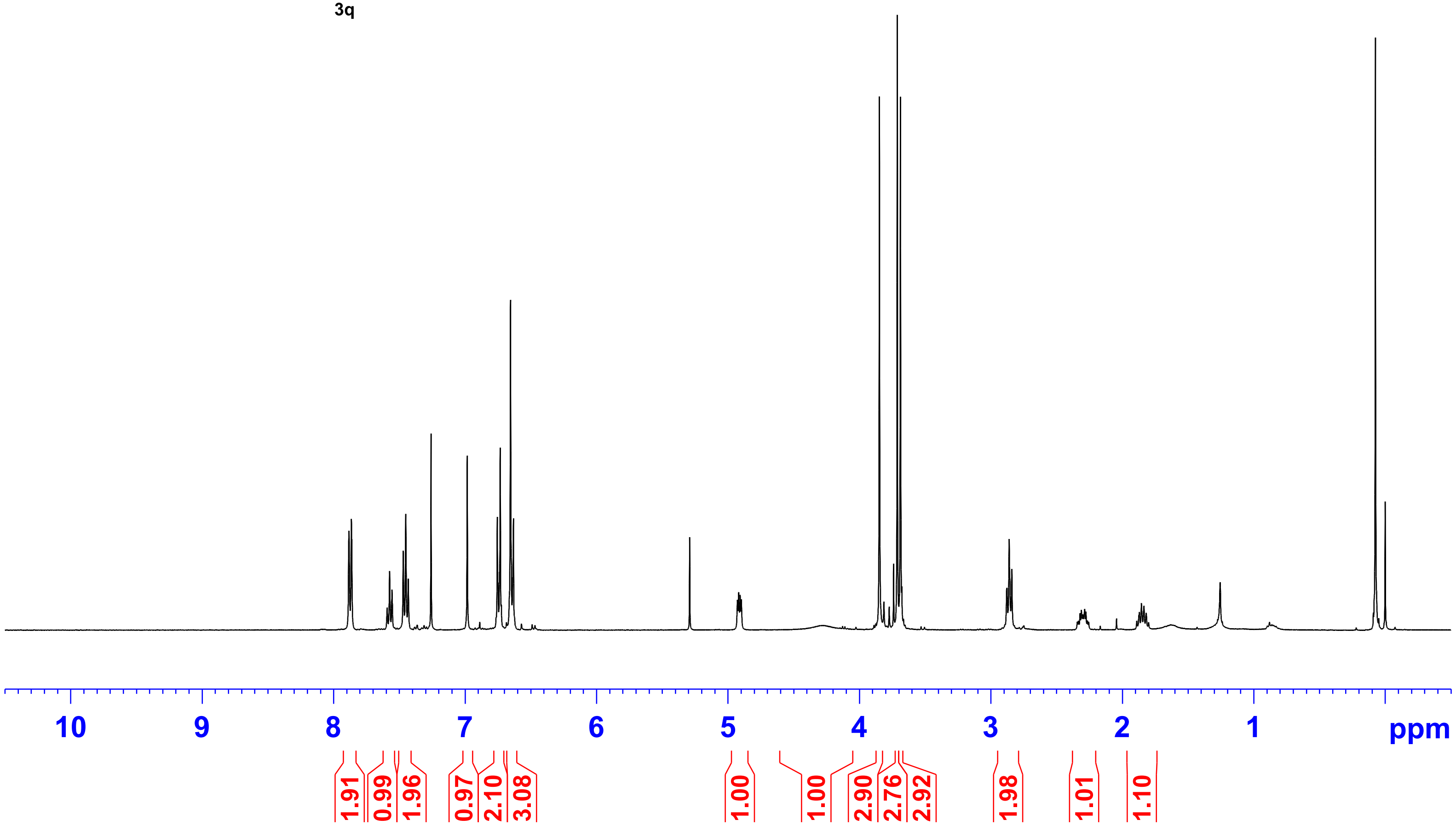

NAME	lzy-06-159-D-0310
EXPNO	1
PROCNO	1
Date_	20210310
Time	9.54
INSTRUM	spect
PROBHD	5 mm DUL 13C-1
PULPROG	zg30
TD	65536
SOLVENT	CDCl3
NS	4
DS	0
SWH	8223.685 Hz
FIDRES	0.125483 Hz
AQ	3.9846387 sec
RG	228
DW	60.800 usec
DE	6.00 usec
TE	294.3 K
D1	1.00000000 sec
TD0	1

======== CHANNEL f1 ========

NUC1	1H
P1	15.80 usec
PL1	-1.00 dB
PL1W	12.17476940 W
SFO1	400.1324710 MHz
SI	32768
SF	400.1300096 MHz
WDW	EM
SSB	0
LB	0.30 Hz
GB	0
PC	1.00

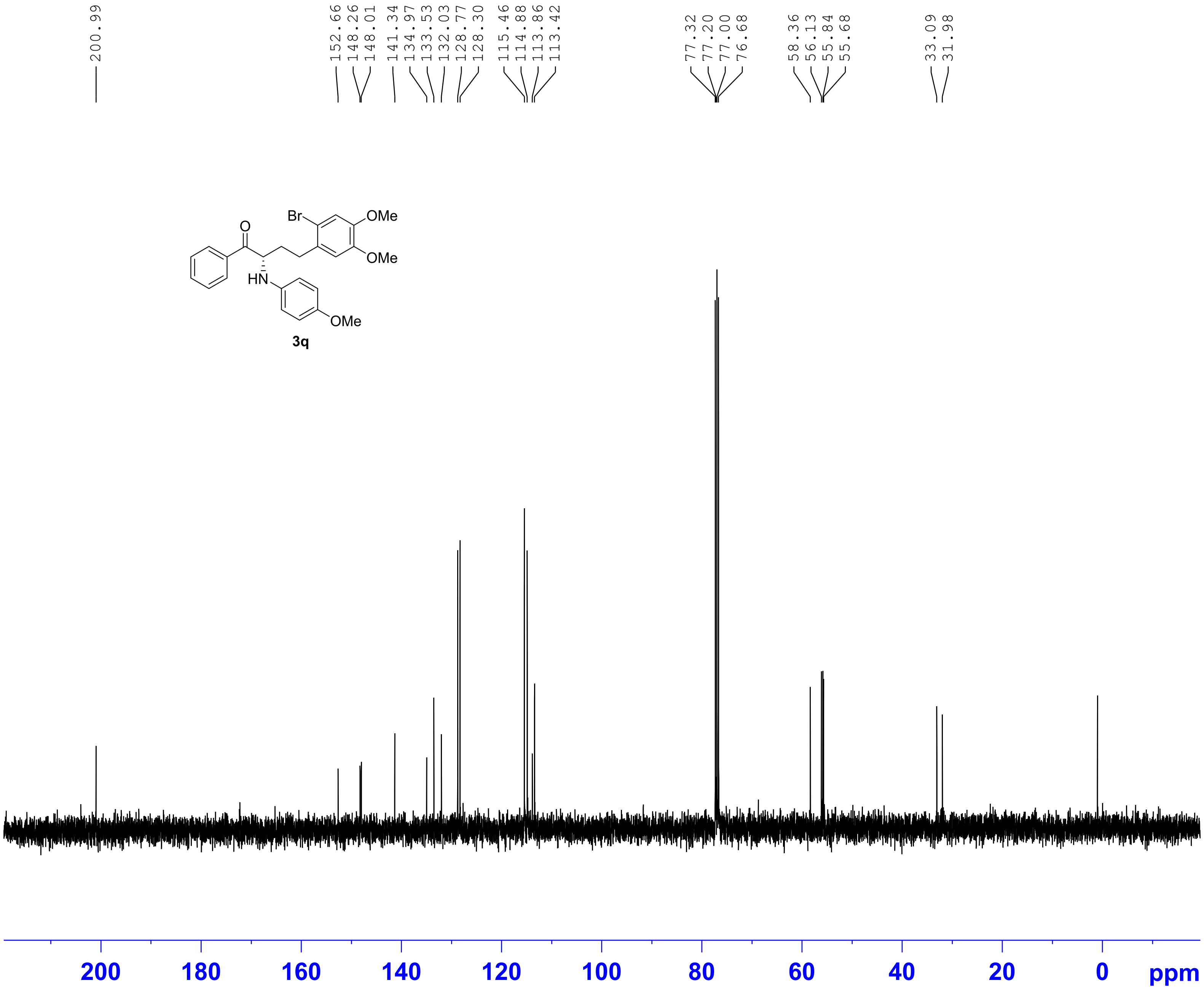

3q

NAME	lzy-06-159-D-0310-C-2	
EXPNO	1	
PROCNO	1	
Date_	20210310	
Time	10.15	
INSTRUM	spect	
PROBHD	5 mm DUL 13C-1	
PULPROG	zgpg30	
TD	65536	
SOLVENT	CDCl3	
NS	170	
DS	0	
SWH	24038.461	Hz
FIDRES	0.366798	Hz
AQ	1.3631988	sec
RG	2050	
DW	20.800	usec
DE	6.00	usec
TE	294.5	K
D1	2.00000000	sec
D11	0.03000000	sec
TD0	1	
======== CHANNEL f1 ========		
NUC1	13C	
P1	40.00	usec
PL1	-3.00	dB
PL1W	60.64365387	W
SFO1	100.6228298	MHz
======== CHANNEL f2 ========		
CPDPRG2	waltz16	
NUC2	1H	
PCPD2	80.00	usec
PL2	-1.00	dB
PL12	14.39	dB
PL13	18.00	dB
PL2W	12.17476940	W
PL12W	0.35193357	W
PL13W	0.15327126	W
SFO2	400.1316005	MHz
SI	32768	
SF	100.6127742	MHz
WDW	EM	
SSB	0	
LB	1.00	Hz
GB	0	
PC	1.40	

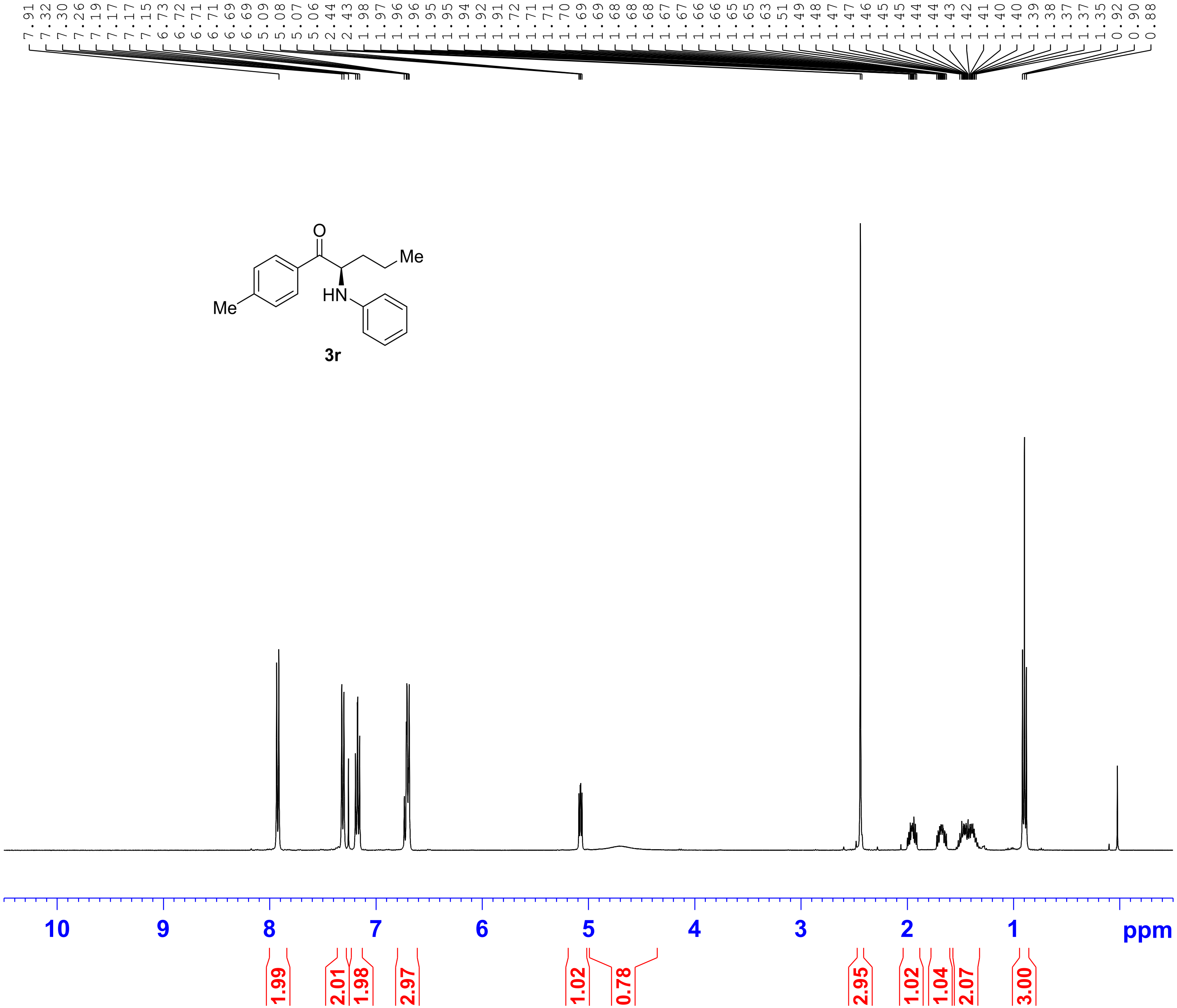

NAME	lzy-04-106-A
EXPNO	1
PROCNO	1
Date_	20200921
Time	21.01
INSTRUM	spect
PROBHD	5 mm PABBO BB-
PULPROG	zg30
TD	65536
SOLVENT	CDCl3
NS	4
DS	0
SWH	8223.685 Hz
FIDRES	0.125483 Hz
AQ	3.9846387 sec
RG	287
DW	60.800 usec
DE	6.00 usec
TE	294.6 K
D1	1.00000000 sec
TD0	1

======== CHANNEL f1 ========

NUC1	1H
P1	15.80 usec
PL1	-1.00 dB
PL1W	12.17476940 W
SFO1	400.1324710 MHz
SI	32768
SF	400.1300103 MHz
WDW	EM
SSB	0
LB	0.30 Hz
GB	0
PC	1.00

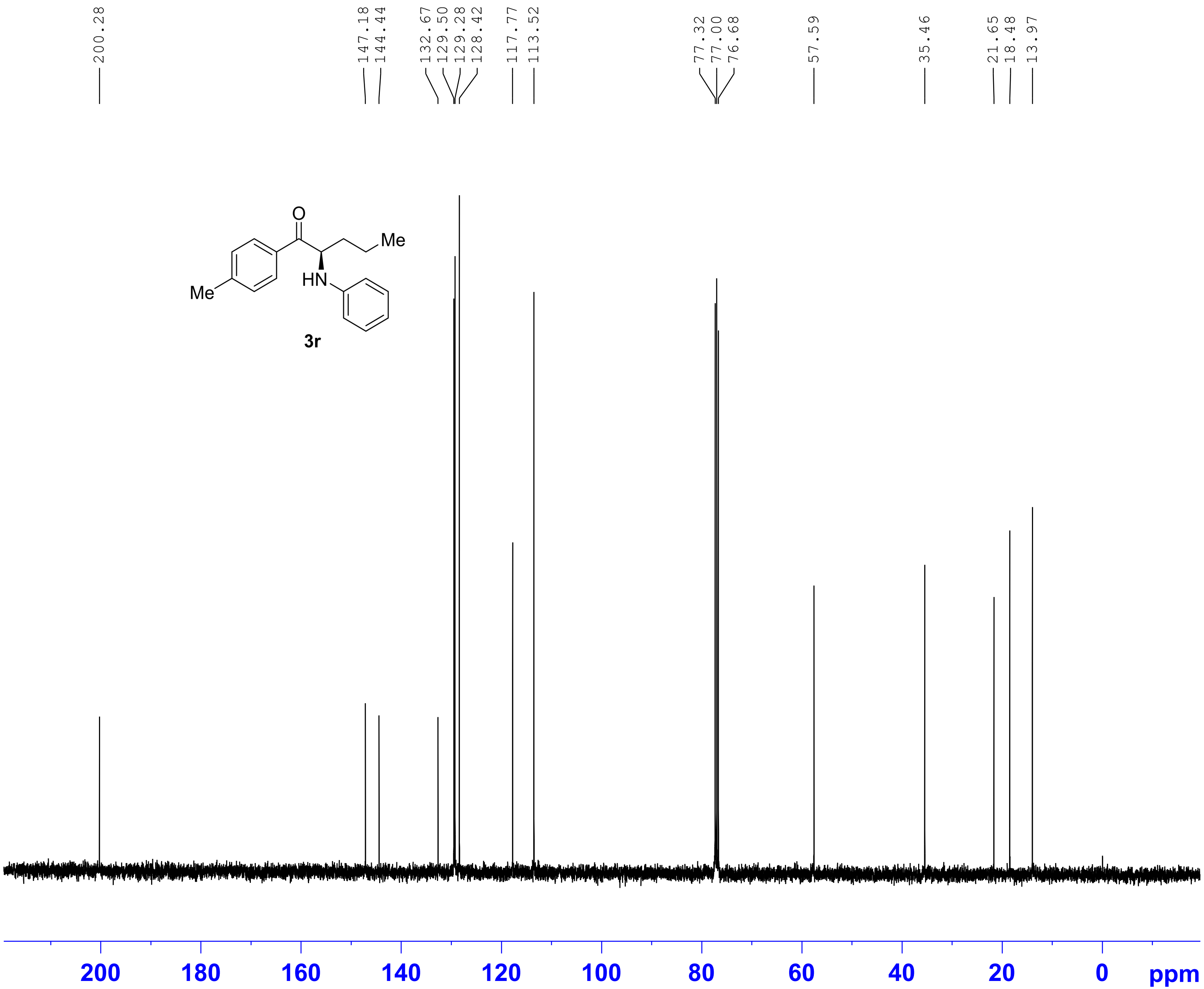

NAME	lzy-04-106-A-C	
EXPNO	1	
PROCNO	1	
Date_	20200921	
Time_	21.10	
INSTRUM	spect	
PROBHD	5 mm PABBO BB-	
PULPROG	zgpg30	
TD	65536	
SOLVENT	CDCl3	
NS	82	
DS	0	
SWH	24038.461	Hz
FIDRES	0.366798	Hz
AQ	1.3631988	sec
RG	80.6	
DW	20.800	usec
DE	6.00	usec
TE	295.4	K
D1	2.00000000	sec
D11	0.03000000	sec
TD0	1	
======== CHANNEL f1 ========		
NUC1	13C	
P1	8.60	usec
PL1	-3.00	dB
PL1W	60.64365387	W
SFO1	100.6228298	MHz
======== CHANNEL f2 ========		
CPDPRG2	waltz16	
NUC2	1H	
PCPD2	80.00	usec
PL2	-1.00	dB
PL12	14.39	dB
PL13	18.00	dB
PL2W	12.17476940	W
PL12W	0.35193357	W
PL13W	0.15327126	W
SFO2	400.1316005	MHz
SI	32768	
SF	100.6127773	MHz
WDW	EM	
SSB	0	
LB	1.00	Hz
GB	0	
PC	1.40	

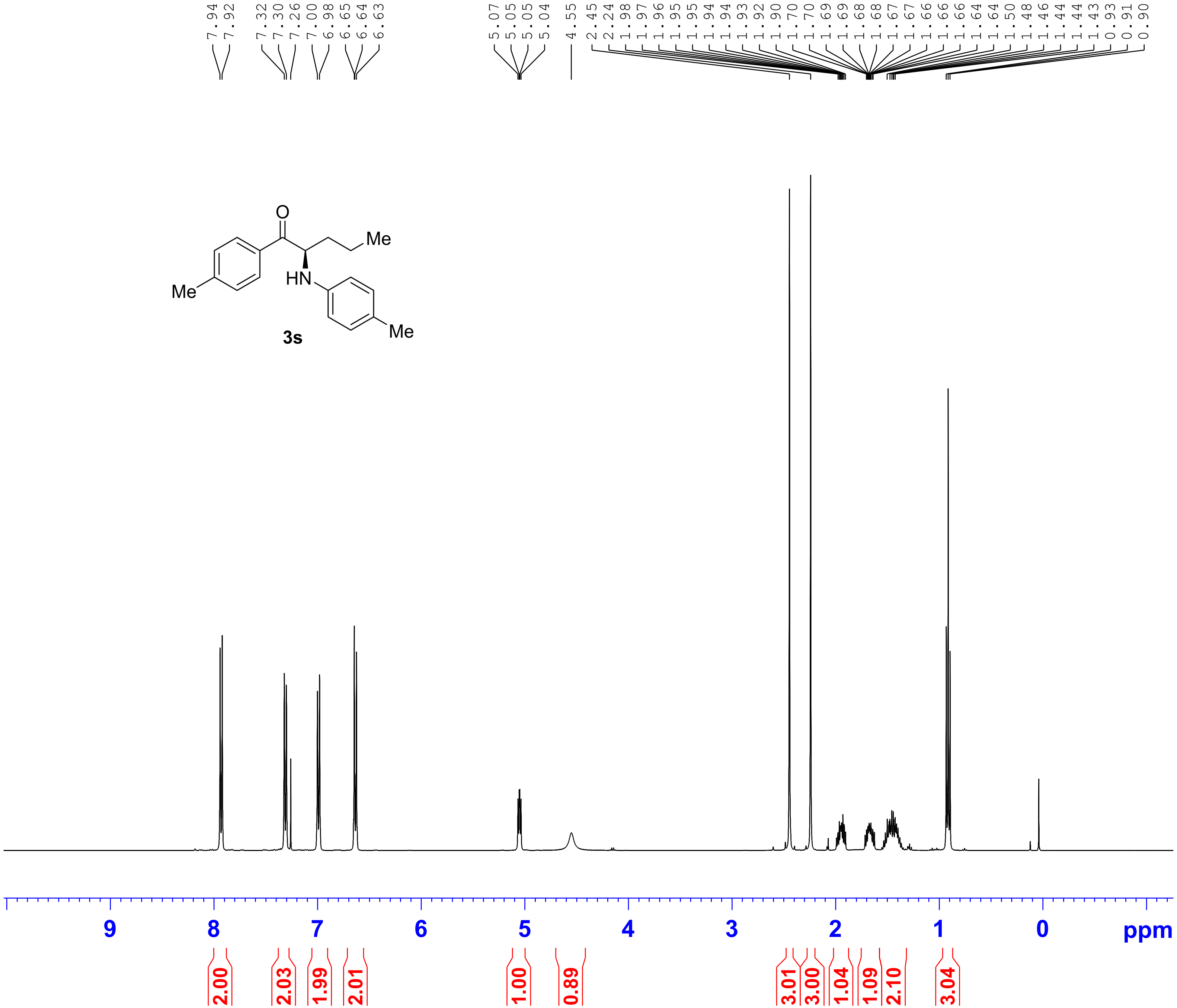


```
NAME          lzy-04-174-J
EXPNO                    1
PROCNO                   1
Date_             20201031
Time                 10.32
INSTRUM              spect
PROBHD    5 mm PABBO BB/
PULPROG               zg30
TD                   65536
SOLVENT              CDCl3
NS                       4
DS                       0
SWH               8012.820 Hz
FIDRES            0.122266 Hz
AQ               4.0894966 sec
RG                   27.78
DW                  62.400 usec
DE                    6.50 usec
TE                   297.2 K
D1              1.00000000 sec
TD0                      1

======== CHANNEL f1 ========
SFO1           400.1324710 MHz
NUC1                    1H
P1                   14.50 usec
SI                   65536
SF             400.1300097 MHz
WDW                     EM
SSB                      0
LB                    0.30 Hz
GB                       0
PC                    1.00
```

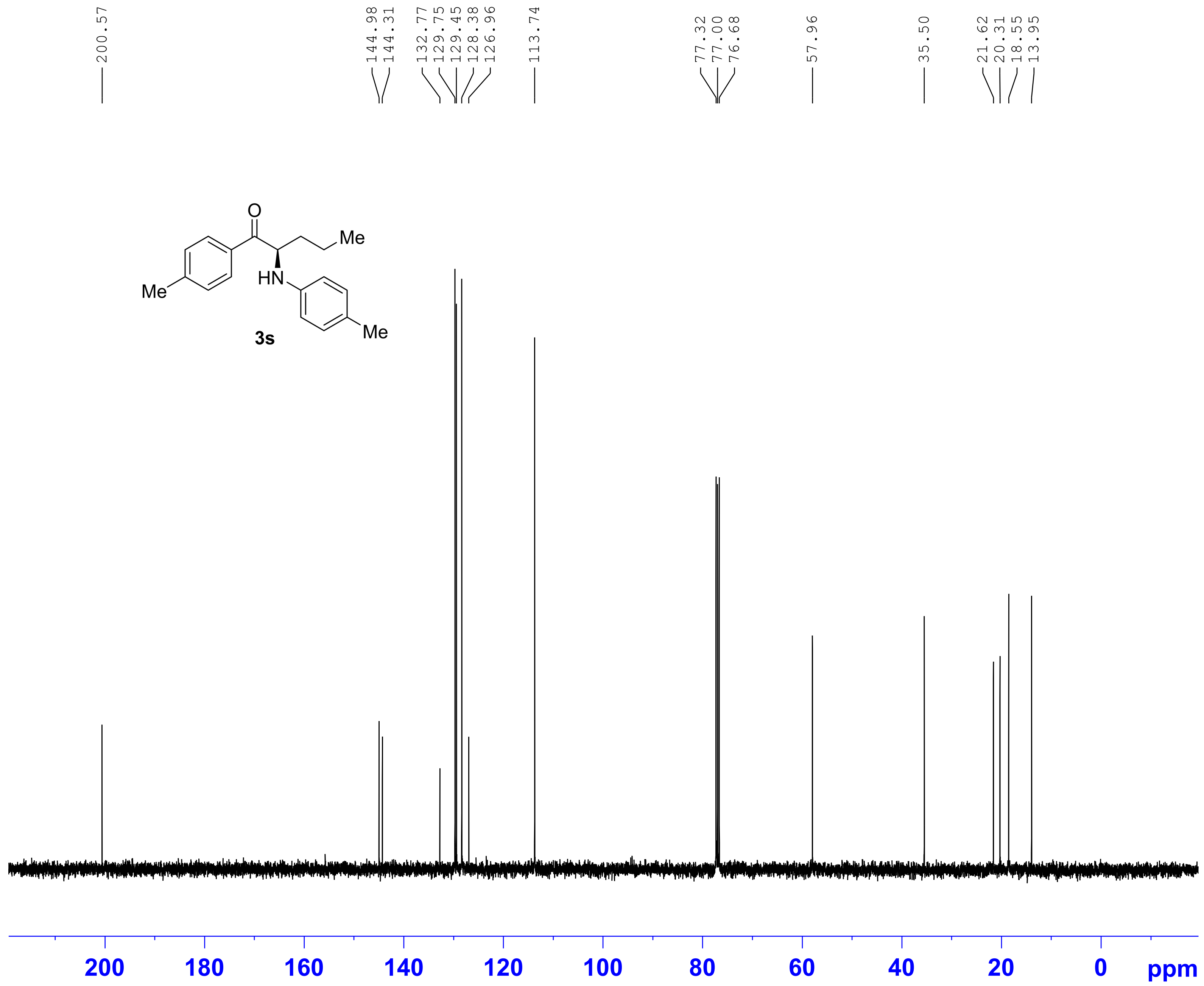


```
NAME       lzy-04-174-J-C
EXPNO                   1
PROCNO                  1
Date_            20201031
Time                10.33
INSTRUM             spect
PROBHD   5 mm PABBO BB/
PULPROG            zgpg30
TD                  65536
SOLVENT             CDCl3
NS                     21
DS                      0
SWH             24038.461 Hz
FIDRES           0.366798 Hz
AQ              1.3631988 sec
RG                 196.92
DW                 20.800 usec
DE                   6.50 usec
TE                  297.7 K
D1             2.00000000 sec
D11            0.03000000 sec
TD0                     1

======== CHANNEL f1 ========
SFO1          100.6228298 MHz
NUC1                  13C
P1                   9.70 usec
SI                  32768
SF            100.6127802 MHz
WDW                    EM
SSB                     0
LB                   1.00 Hz
GB                      0
PC                   1.40
```

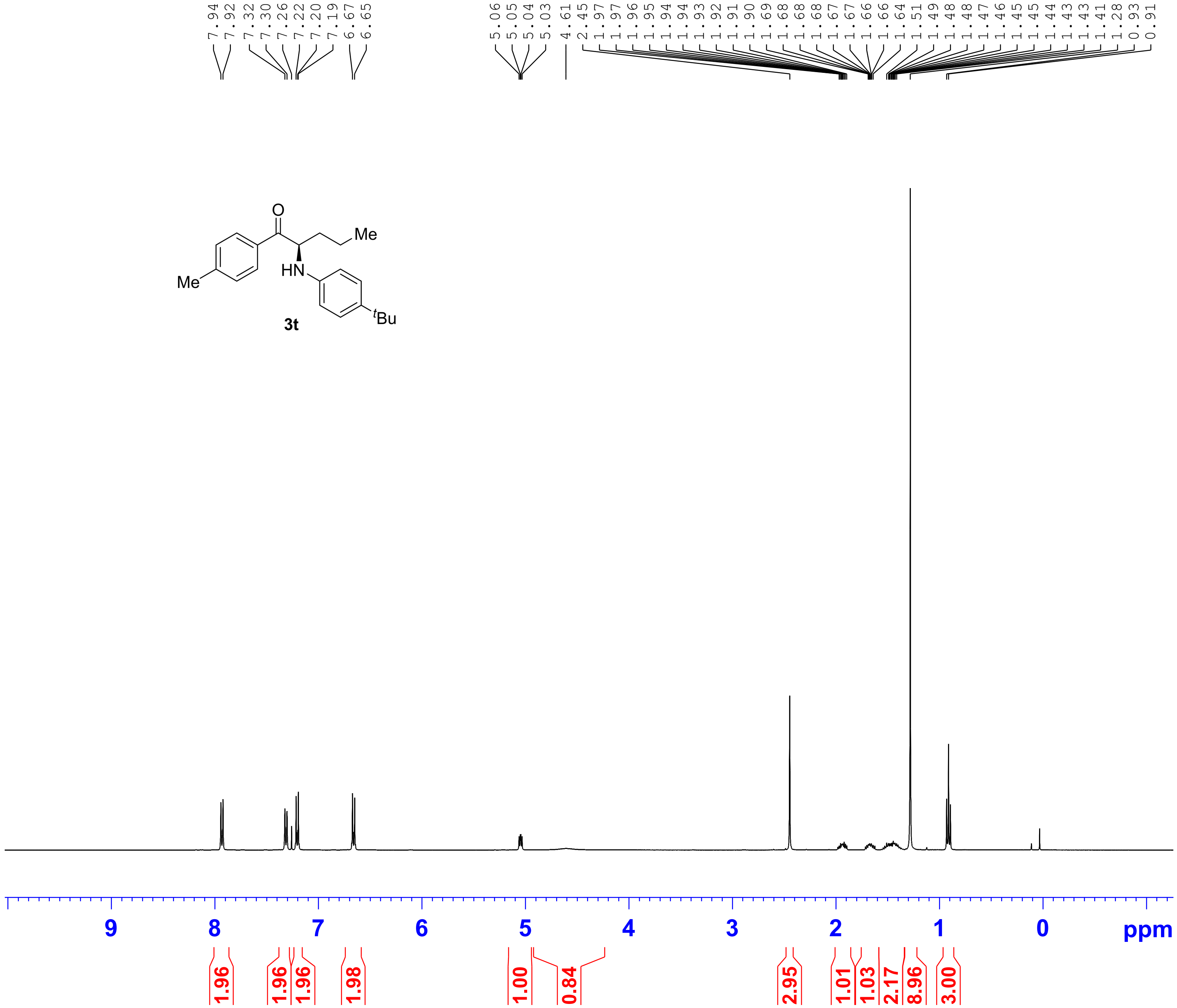

3t

NAME	lzy-04-105-C
EXPNO	1
PROCNO	1
Date_	20200919
Time	14.47
INSTRUM	spect
PROBHD	5 mm PABBO BB/
PULPROG	zg30
TD	65536
SOLVENT	CDCl3
NS	4
DS	0
SWH	8012.820 Hz
FIDRES	0.122266 Hz
AQ	4.0894966 sec
RG	31.55
DW	62.400 usec
DE	6.50 usec
TE	296.8 K
D1	1.00000000 sec
TD0	1

======== CHANNEL f1 ========

SFO1	400.1324710 MHz
NUC1	1H
P1	14.50 usec
SI	65536
SF	400.1300098 MHz
WDW	EM
SSB	0
LB	0.30 Hz
GB	0
PC	1.00

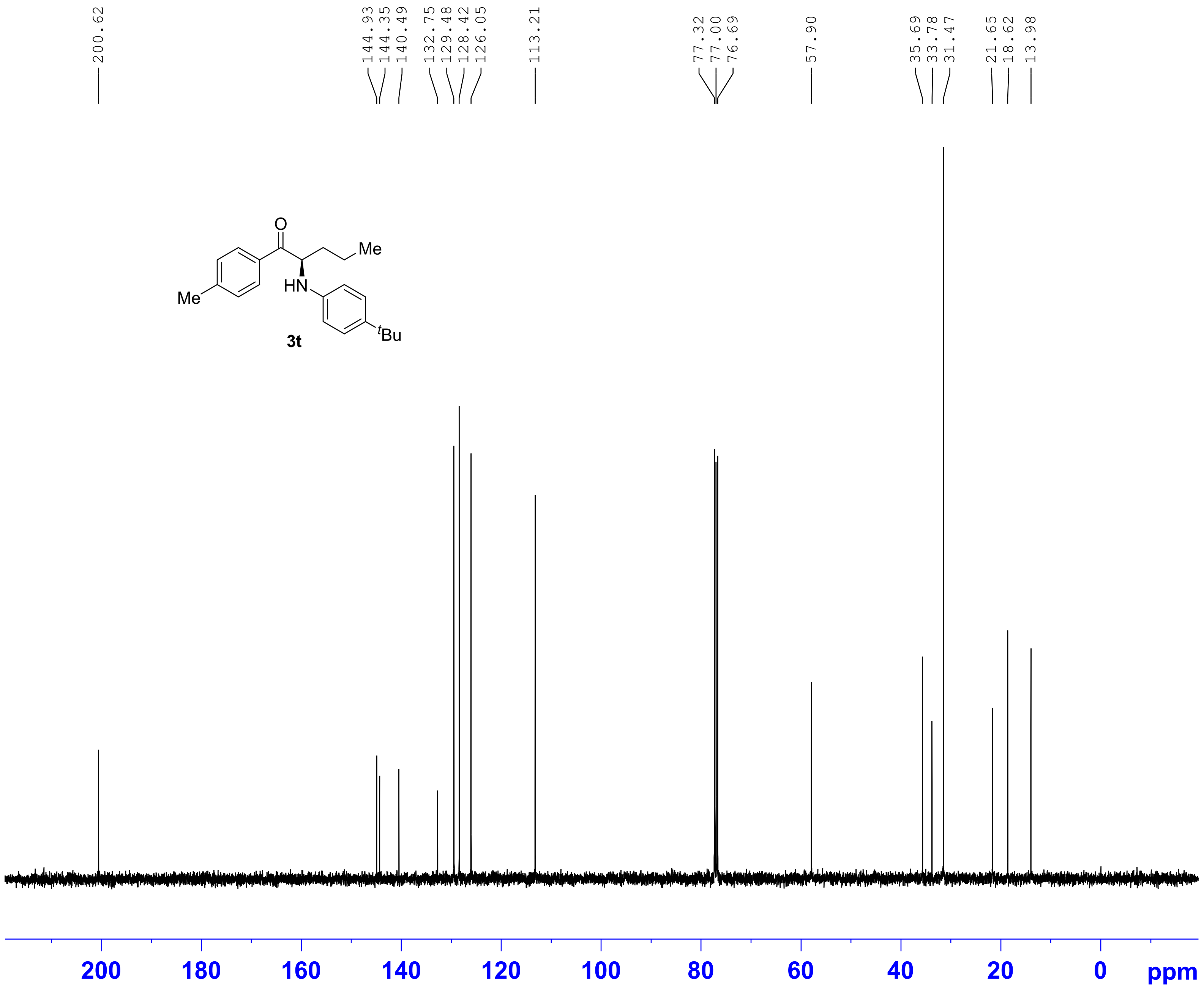

3t

NAME	lzy-04-105-C-C
EXPNO	1
PROCNO	1
Date_	20200919
Time	14.50
INSTRUM	spect
PROBHD	5 mm PABBO BB/
PULPROG	zgpg30
TD	65536
SOLVENT	CDCl3
NS	75
DS	0
SWH	24038.461 Hz
FIDRES	0.366798 Hz
AQ	1.3631988 sec
RG	196.92
DW	20.800 usec
DE	6.50 usec
TE	297.4 K
D1	2.00000000 sec
D11	0.03000000 sec
TD0	1

======== CHANNEL f1 ========

SFO1	100.6228298 MHz
NUC1	13C
P1	9.70 usec
SI	32768
SF	100.6127773 MHz
WDW	EM
SSB	0
LB	1.00 Hz
GB	0
PC	1.40

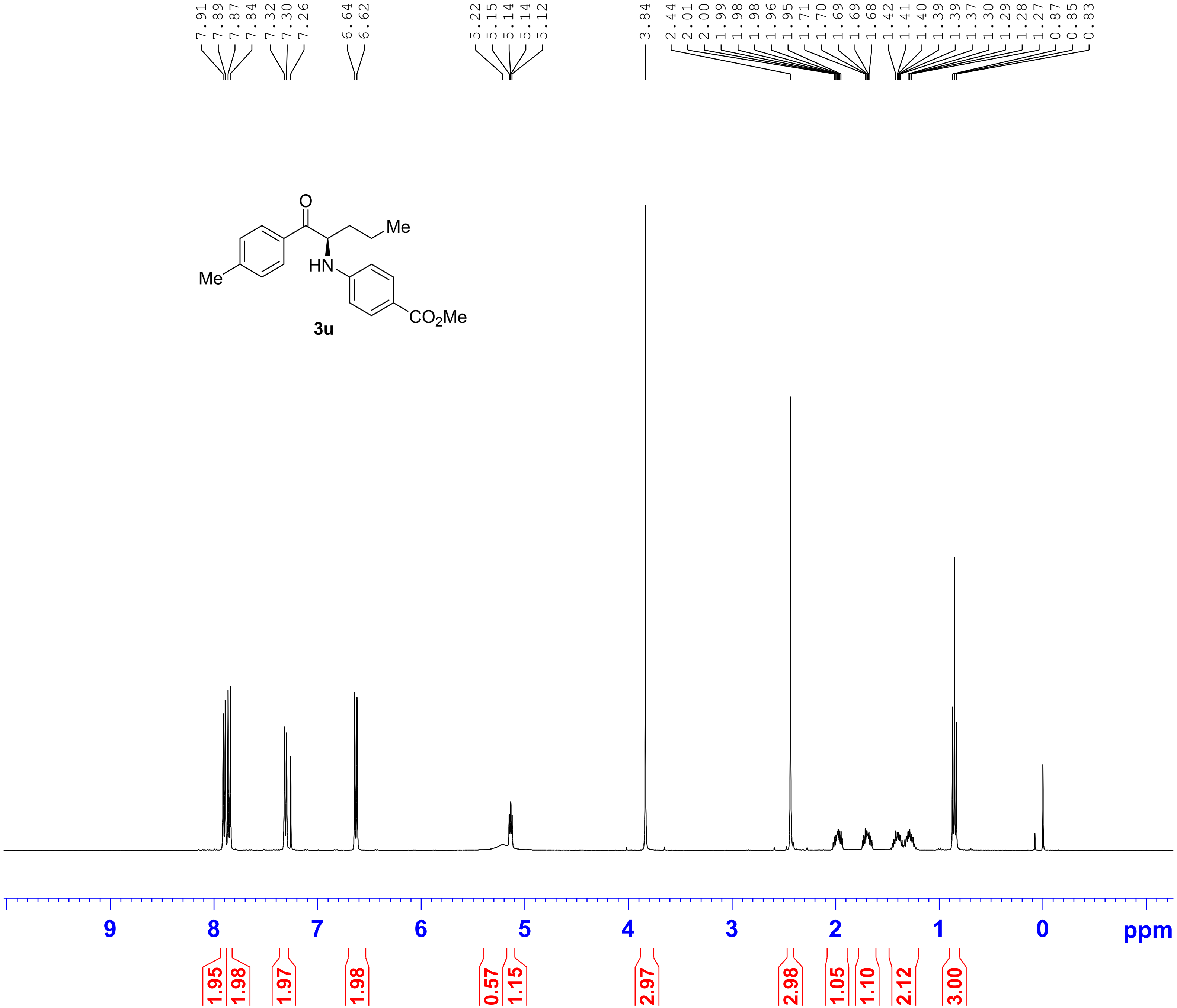

NAME	lzy-04-116
EXPNO	1
PROCNO	1
Date_	20200925
Time_	14.43
INSTRUM	spect
PROBHD	5 mm PABBO BB-
PULPROG	zg30
TD	65536
SOLVENT	CDCl3
NS	3
DS	0
SWH	8223.685 Hz
FIDRES	0.125483 Hz
AQ	3.9846387 sec
RG	203
DW	60.800 usec
DE	6.00 usec
TE	294.6 K
D1	1.00000000 sec
TD0	1

======== CHANNEL f1 ========

NUC1	1H
P1	15.80 usec
PL1	-1.00 dB
PL1W	12.17476940 W
SFO1	400.1324710 MHz
SI	32768
SF	400.1300096 MHz
WDW	EM
SSB	0
LB	0.30 Hz
GB	0
PC	1.00

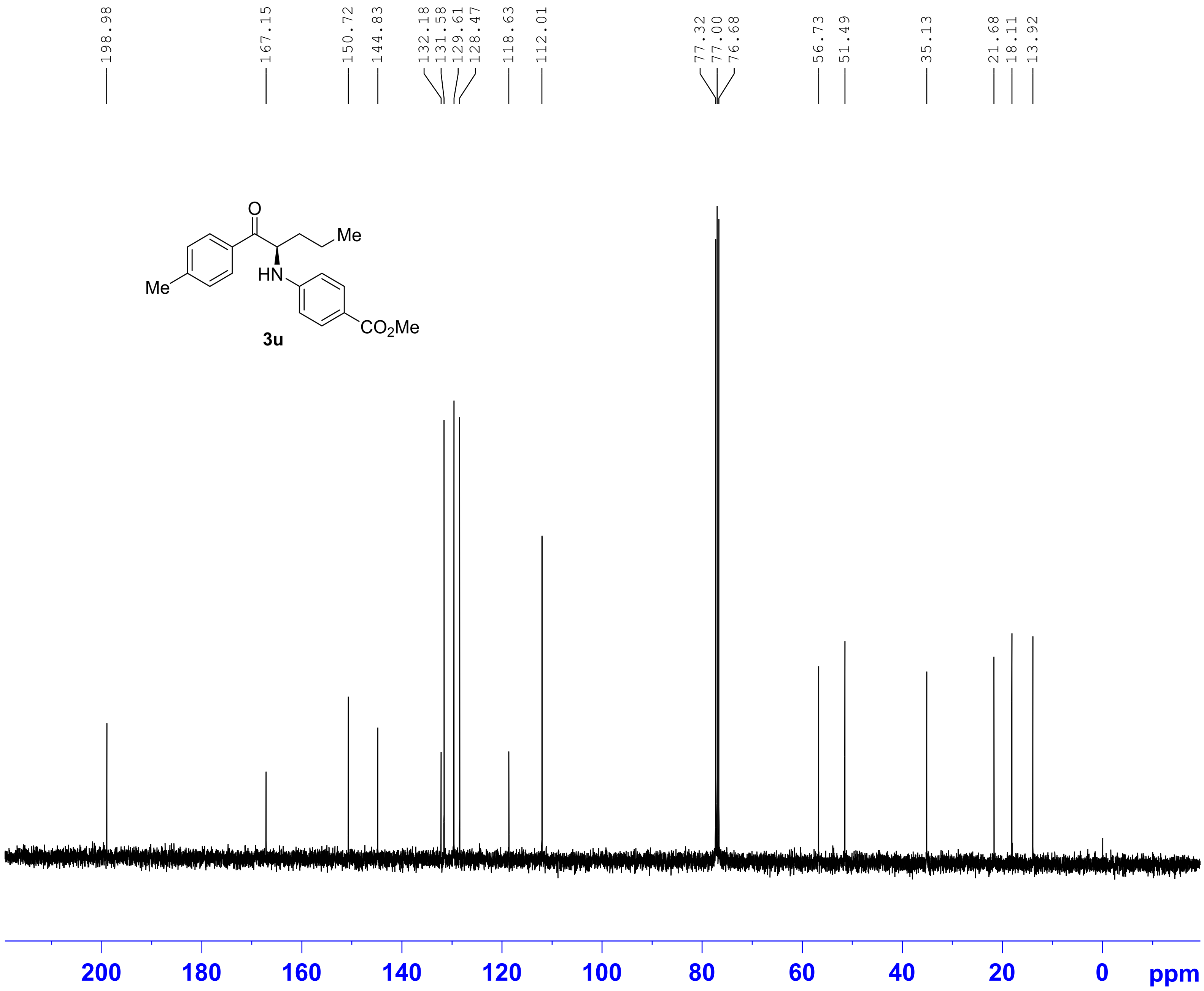

NAME	lzy-04-116-C
EXPNO	1
PROCNO	1
Date_	20200925
Time	14.48
INSTRUM	spect
PROBHD	5 mm PABBO BB-
PULPROG	zgpg30
TD	65536
SOLVENT	CDCl3
NS	50
DS	0
SWH	24038.461 Hz
FIDRES	0.366798 Hz
AQ	1.3631988 sec
RG	90.5
DW	20.800 usec
DE	6.00 usec
TE	295.1 K
D1	2.00000000 sec
D11	0.03000000 sec
TD0	1
======== CHANNEL f1 ========	
NUC1	13C
P1	8.60 usec
PL1	-3.00 dB
PL1W	60.64365387 W
SFO1	100.6228298 MHz
======== CHANNEL f2 ========	
CPDPRG2	waltz16
NUC2	1H
PCPD2	80.00 usec
PL2	-1.00 dB
PL12	14.39 dB
PL13	18.00 dB
PL2W	12.17476940 W
PL12W	0.35193357 W
PL13W	0.15327126 W
SFO2	400.1316005 MHz
SI	32768
SF	100.6127758 MHz
WDW	EM
SSB	0
LB	1.00 Hz
GB	0
PC	1.40

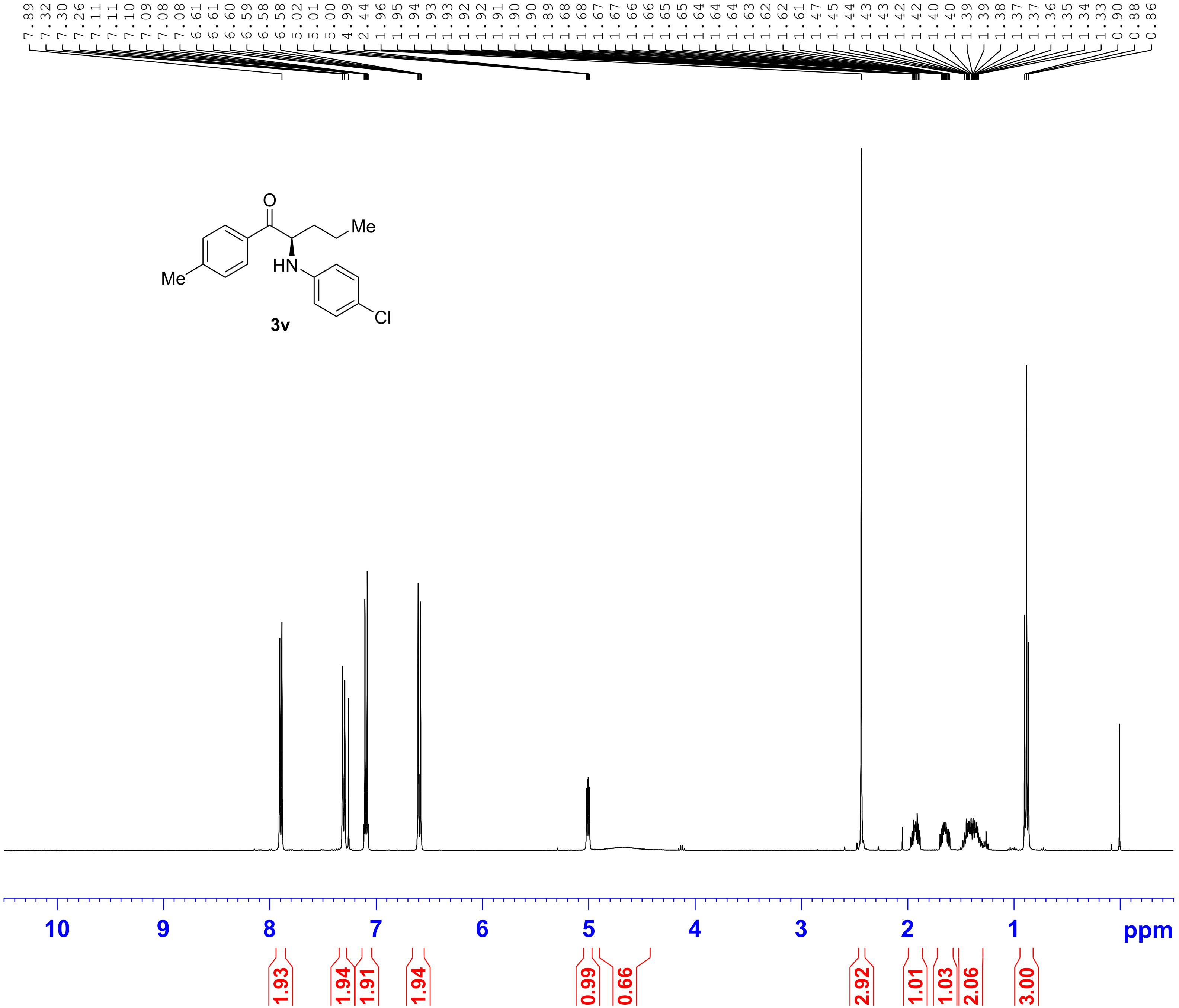

NAME	lzy-04-105-A
EXPNO	1
PROCNO	1
Date_	20200919
Time	16.34
INSTRUM	spect
PROBHD	5 mm PABBO BB/
PULPROG	zg30
TD	65536
SOLVENT	CDCl3
NS	4
DS	0
SWH	8012.820 Hz
FIDRES	0.122266 Hz
AQ	4.0894966 sec
RG	70.97
DW	62.400 usec
DE	6.50 usec
TE	296.3 K
D1	1.00000000 sec
TD0	1

======== CHANNEL f1 ========

SFO1	400.1324710 MHz
NUC1	1H
P1	14.50 usec
SI	65536
SF	400.1300100 MHz
WDW	EM
SSB	0
LB	0.30 Hz
GB	0
PC	1.00

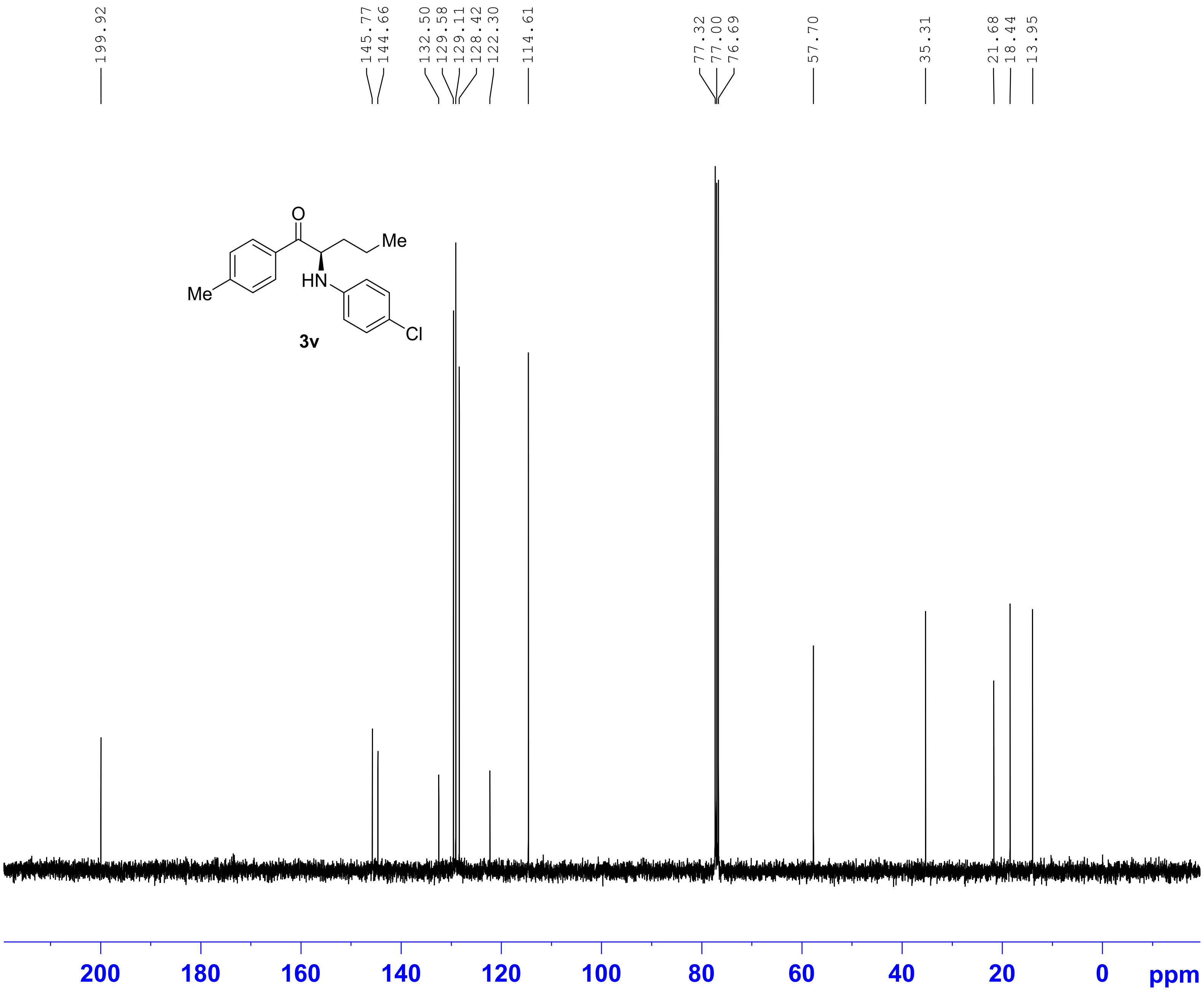


```
NAME       lzy-04-105-A-C
EXPNO                   1
PROCNO                  1
Date_            20200919
Time                16.37
INSTRUM             spect
PROBHD    5 mm PABBO BB/
PULPROG            zgpg30
TD                  65536
SOLVENT             CDCl3
NS                     51
DS                      0
SWH             24038.461 Hz
FIDRES           0.366798 Hz
AQ              1.3631988 sec
RG                 196.92
DW                 20.800 usec
DE                   6.50 usec
TE                  297.2 K
D1             2.00000000 sec
D11            0.03000000 sec
TD0                     1

======== CHANNEL f1 ========
SFO1          100.6228298 MHz
NUC1                  13C
P1                   9.70 usec
SI                  32768
SF            100.6127744 MHz
WDW                    EM
SSB                     0
LB                   1.00 Hz
GB                      0
PC                   1.40
```

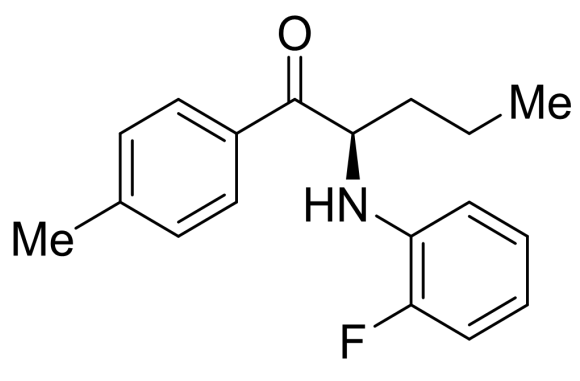

3w

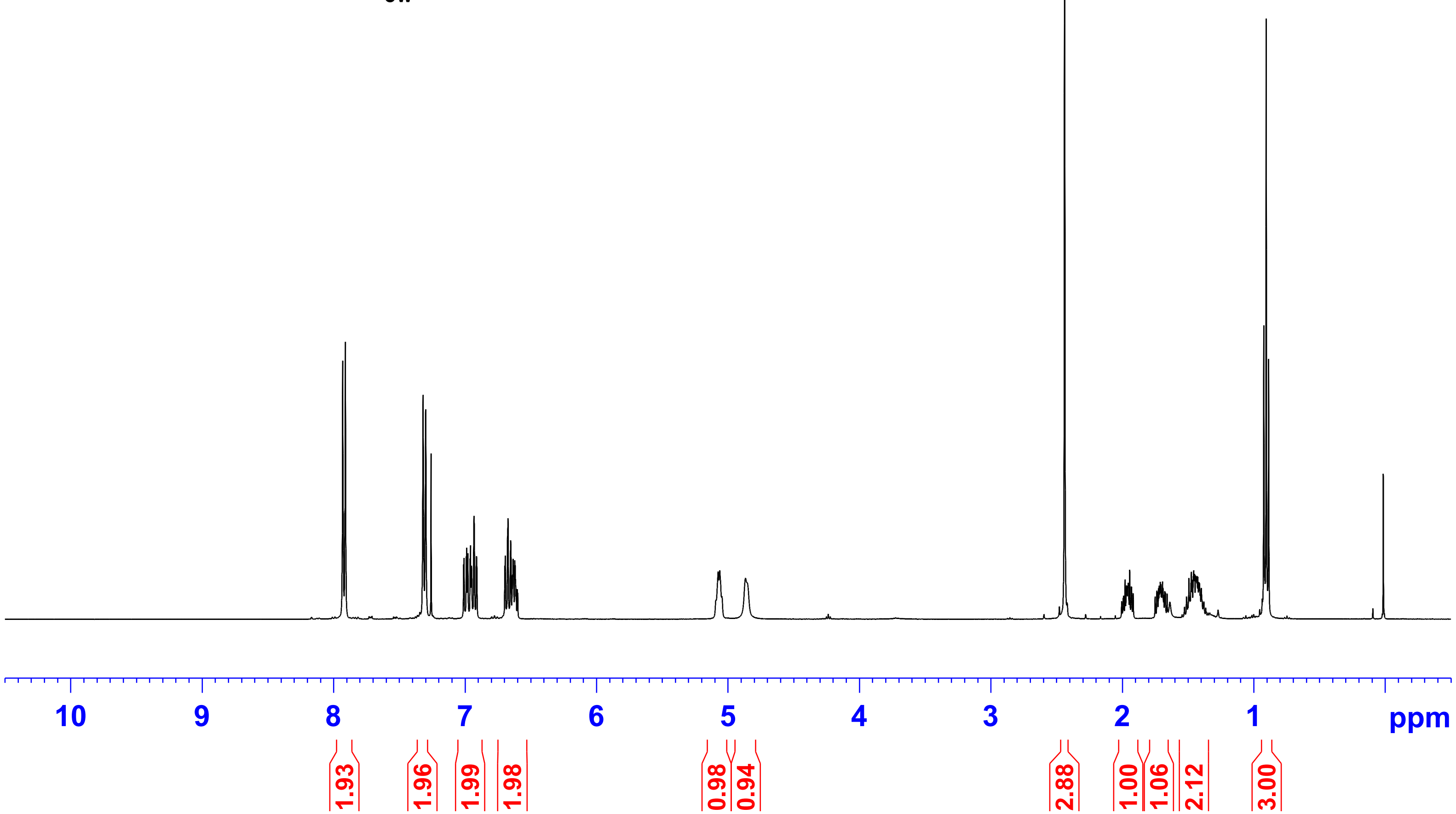

NAME	lzy-04-127
EXPNO	1
PROCNO	1
Date_	20200927
Time	19.43
INSTRUM	spect
PROBHD	5 mm PABBO BB/
PULPROG	zg30
TD	65536
SOLVENT	CDCl3
NS	4
DS	0
SWH	8012.820 Hz
FIDRES	0.122266 Hz
AQ	4.0894966 sec
RG	39.46
DW	62.400 usec
DE	6.50 usec
TE	297.8 K
D1	1.00000000 sec
TD0	1

======== CHANNEL f1 ========

SFO1	400.1324710 MHz
NUC1	1H
P1	14.50 usec
SI	65536
SF	400.1300100 MHz
WDW	EM
SSB	0
LB	0.30 Hz
GB	0
PC	1.00

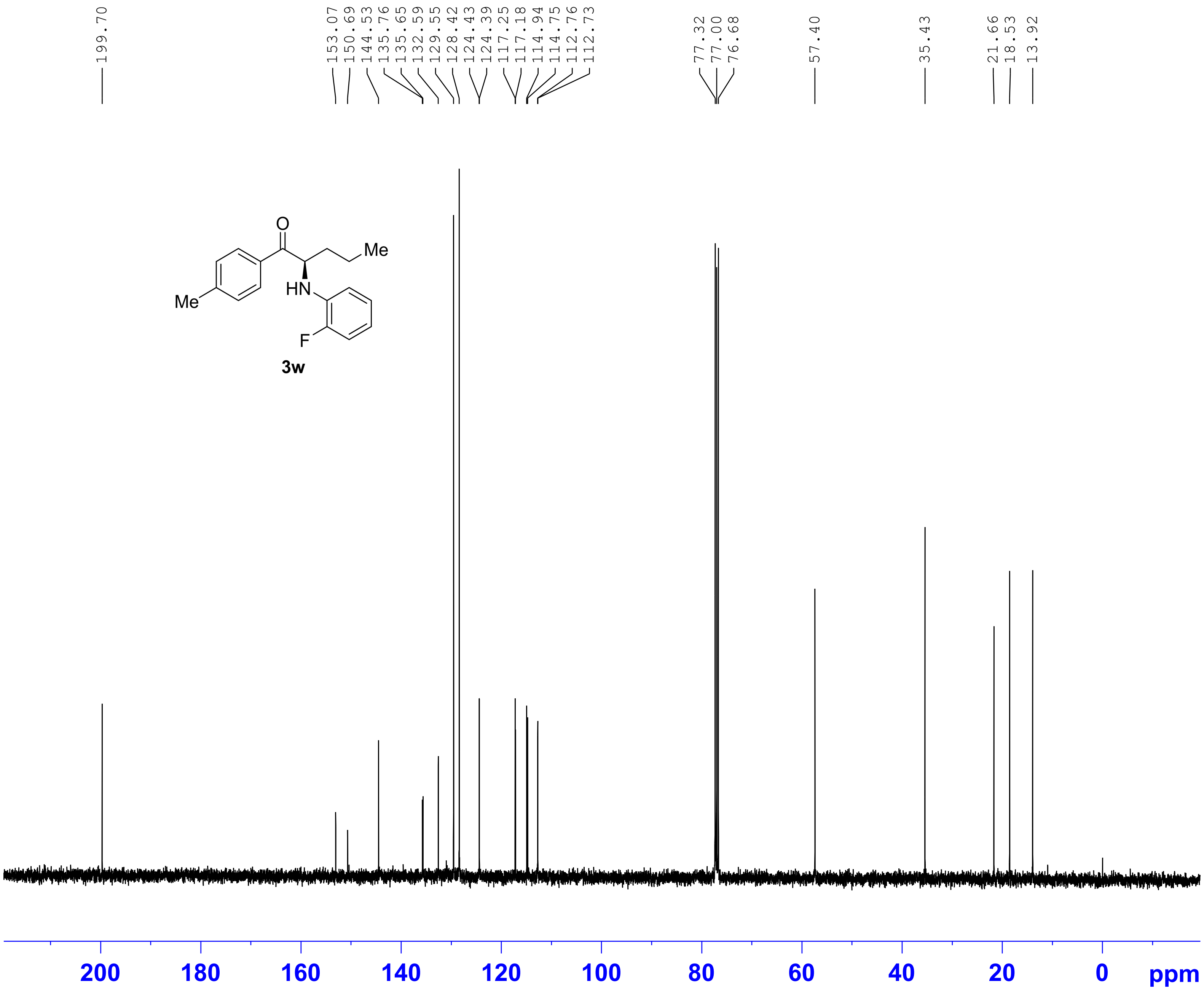

NAME	lzy-04-127-C
EXPNO	1
PROCNO	1
Date_	20200927
Time_	19.46
INSTRUM	spect
PROBHD	5 mm PABBO BB/
PULPROG	zgpg30
TD	65536
SOLVENT	CDCl3
NS	99
DS	0
SWH	24038.461 Hz
FIDRES	0.366798 Hz
AQ	1.3631988 sec
RG	196.92
DW	20.800 usec
DE	6.50 usec
TE	298.5 K
D1	2.00000000 sec
D11	0.03000000 sec
TD0	1

======== CHANNEL f1 ========

SFO1	100.6228298 MHz
NUC1	13C
P1	9.70 usec
SI	32768
SF	100.6127745 MHz
WDW	EM
SSB	0
LB	1.00 Hz
GB	0
PC	1.40

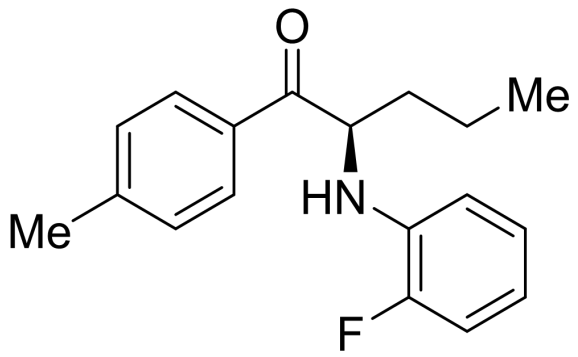

3w

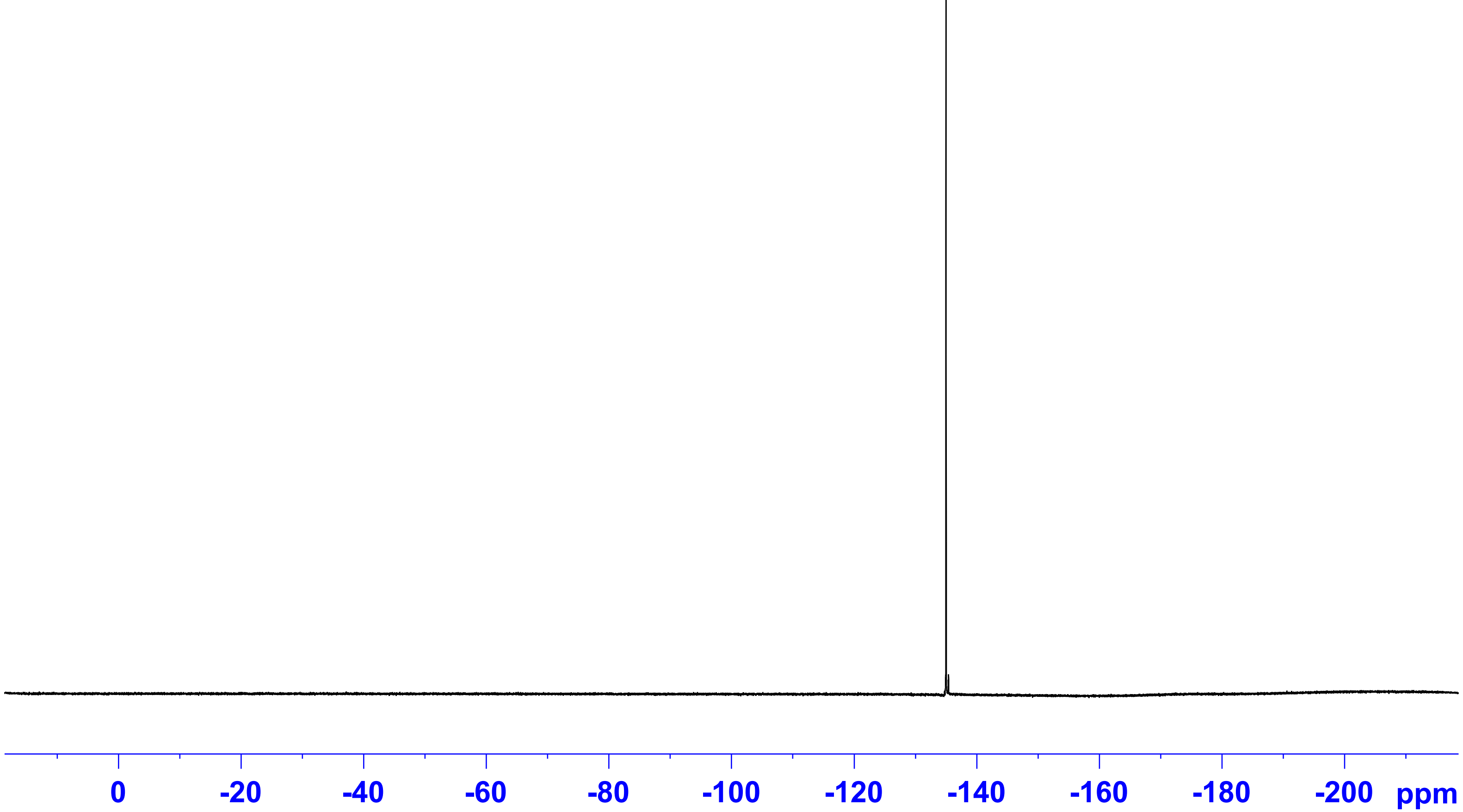

NAME	lzy-04-127-F	
EXPNO	1	
PROCNO	1	
Date_	20200927	
Time	19.53	
INSTRUM	spect	
PROBHD	5 mm PABBO BB/	
PULPROG	zgflqn	
TD	131072	
SOLVENT	CDCl3	
NS	7	
DS	4	
SWH	89285.711	Hz
FIDRES	0.681196	Hz
AQ	0.7340532	sec
RG	196.92	
DW	5.600	usec
DE	6.50	usec
TE	297.9	K
D1	1.00000000	sec
TD0	1	
======== CHANNEL f1 ========		
SFO1	376.4607164	MHz
NUC1	19F	
P1	14.70	usec
SI	65536	
SF	376.4983660	MHz
WDW	EM	
SSB	0	
LB	0.30	Hz
GB	0	
PC	1.00	

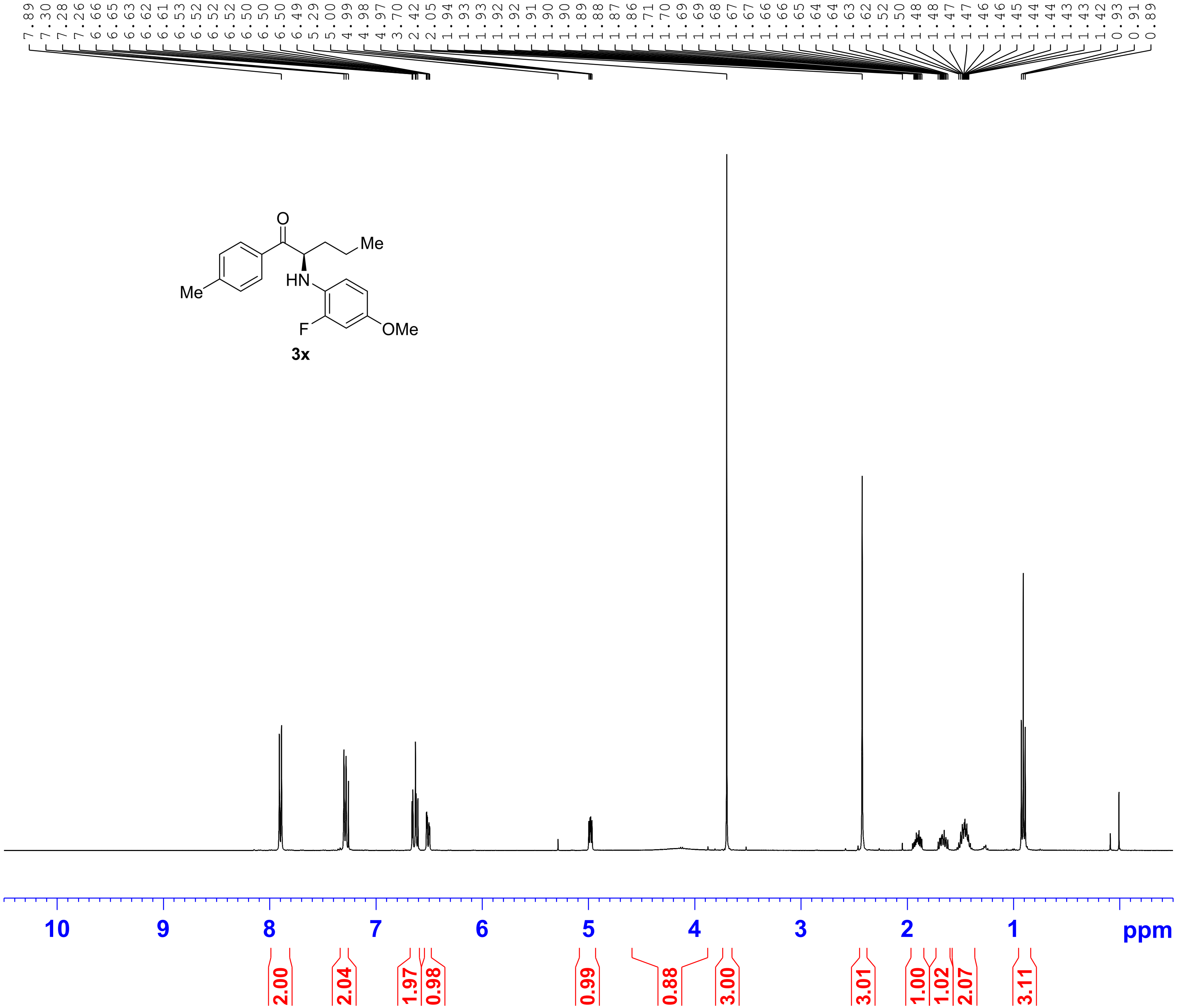

NAME lzy-04-106-B
EXPNO 1
PROCNO 1
Date_ 20200921
Time_ 21.45
INSTRUM spect
PROBHD 5 mm PABBO BB/
PULPROG zg30
TD 65536
SOLVENT CDCl3
NS 1
DS 0
SWH 8012.820 Hz
FIDRES 0.122266 Hz
AQ 4.0894966 sec
RG 34.77
DW 62.400 usec
DE 6.50 usec
TE 297.4 K
D1 1.00000000 sec
TD0 1

======== CHANNEL f1 ========
SFO1 400.1324710 MHz
NUC1 1H
P1 14.50 usec
SI 65536
SF 400.1300100 MHz
WDW EM
SSB 0
LB 0.30 Hz
GB 0
PC 1.00

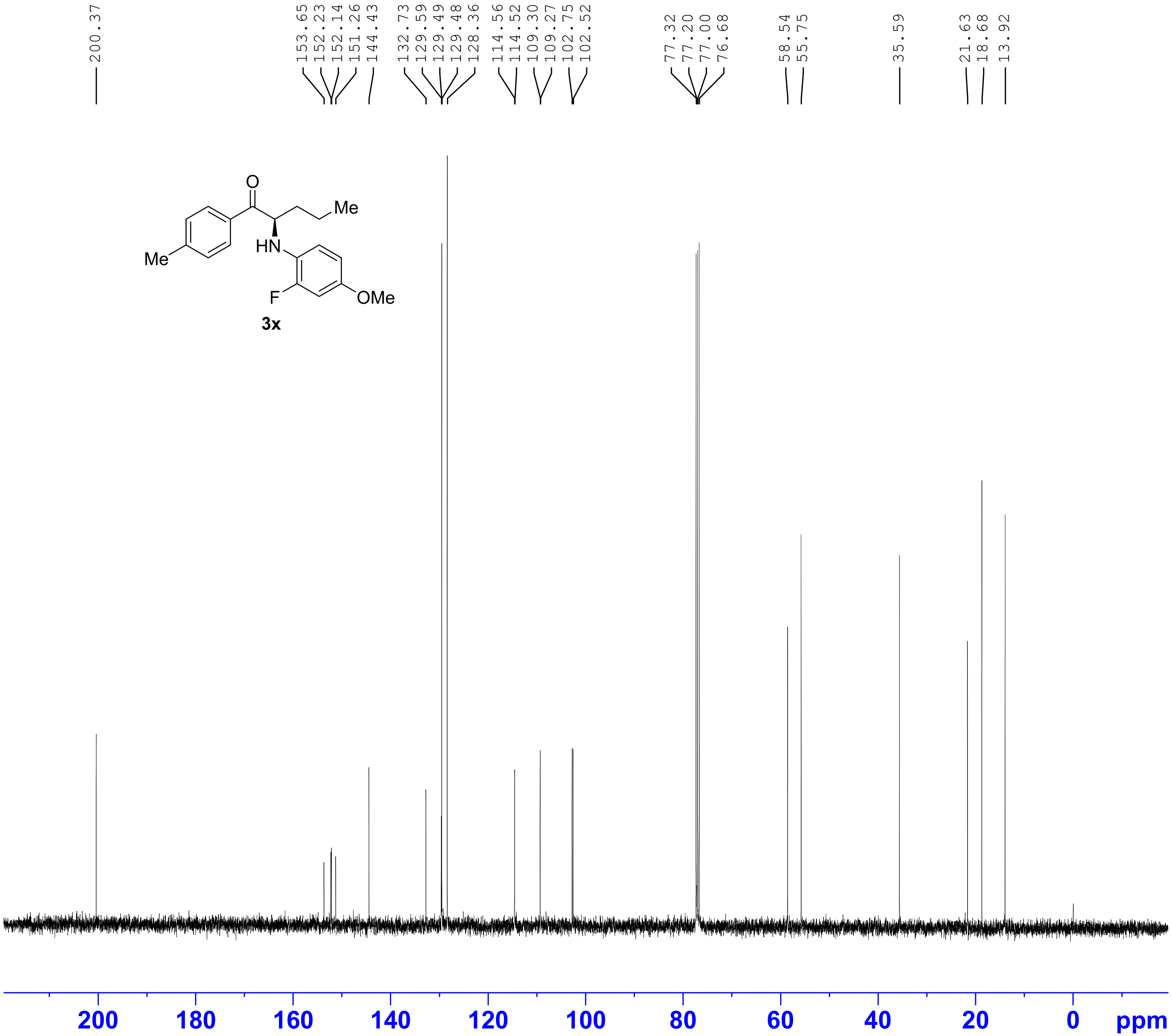


```
Current Data Parameters
NAME              lzy-04-106-B-C
EXPNO                          1
PROCNO                         1

F2 - Acquisition Parameters
Date_                   20200921
Time                       21.53
INSTRUM                    spect
PROBHD     5 mm PABBO BB/19F-1H/D Z-GRD Z108618/0582
PULPROG                  zgpg30
TD                         65536
SOLVENT                    CDCl3
NS                            63
DS                             0
SWH                    24038.461 Hz
FIDRES                  0.366798 Hz
AQ                     1.3631488 sec
RG                        196.92
DW                        20.800 usec
DE                          6.50 usec
TE                         298.3 K
D1                    2.00000000 sec
D11                   0.03000000 sec
TD0                            1

======== CHANNEL f1 ========
SFO1                 100.6228298 MHz
NUC1                         13C
P1                          9.70 usec
PLW1                 46.98899841 W

======== CHANNEL f2 ========
SFO2                 400.1316005 MHz
NUC2                          1H
CPDPRG[2]                 waltz16
PCPD2                      90.00 usec
PLW2                 11.99499989 W
PLW12                 0.34213999 W
PLW13                 0.27713001 W

F2 - Processing parameters
SI                         32768
SF                   100.6127758 MHz
WDW                           EM
SSB                            0
LB                          1.00 Hz
GB                             0
PC                          1.40
```

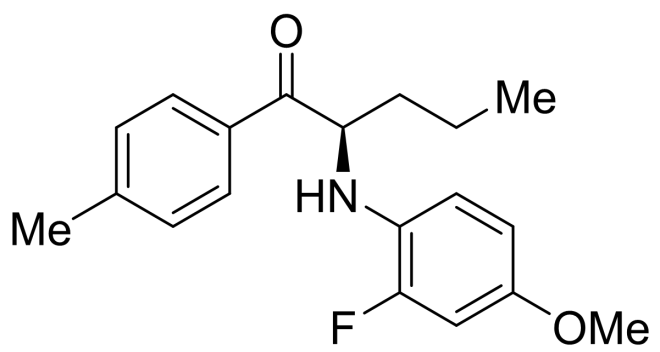

3x

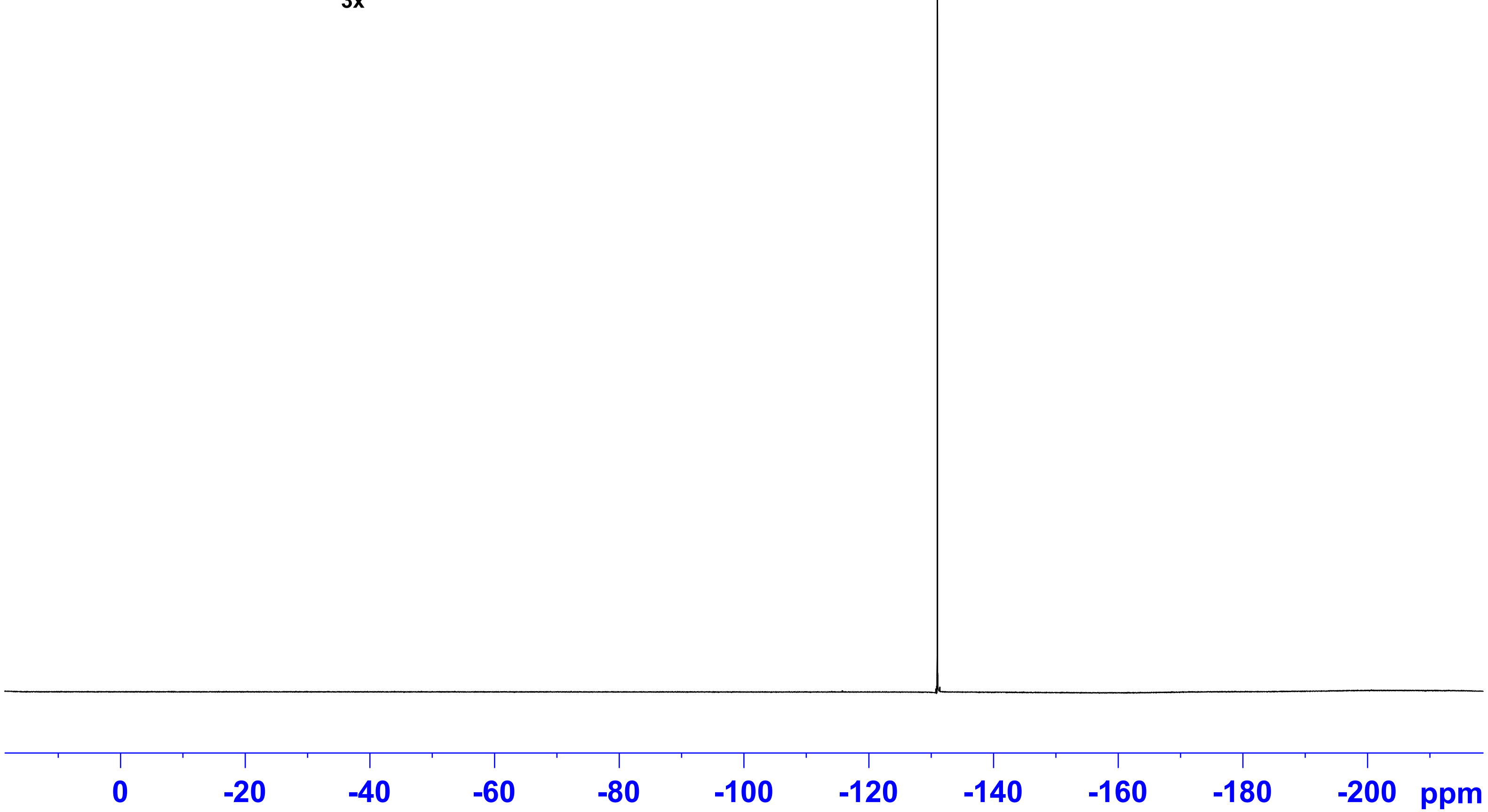

NAME	lzy-04-106-B-F	
EXPNO	1	
PROCNO	1	
Date_	20200921	
Time_	21.54	
INSTRUM	spect	
PROBHD	5 mm PABBO BB/	
PULPROG	zgflqn	
TD	131072	
SOLVENT	CDCl3	
NS	16	
DS	4	
SWH	89285.711	Hz
FIDRES	0.681196	Hz
AQ	0.7340532	sec
RG	196.92	
DW	5.600	usec
DE	6.50	usec
TE	297.7	K
D1	1.00000000	sec
TD0	1	

======== CHANNEL f1 ========

SFO1	376.4607164	MHz
NUC1	19F	
P1	14.70	usec
SI	65536	
SF	376.4983660	MHz
WDW	EM	
SSB	0	
LB	0.30	Hz
GB	0	
PC	1.00	

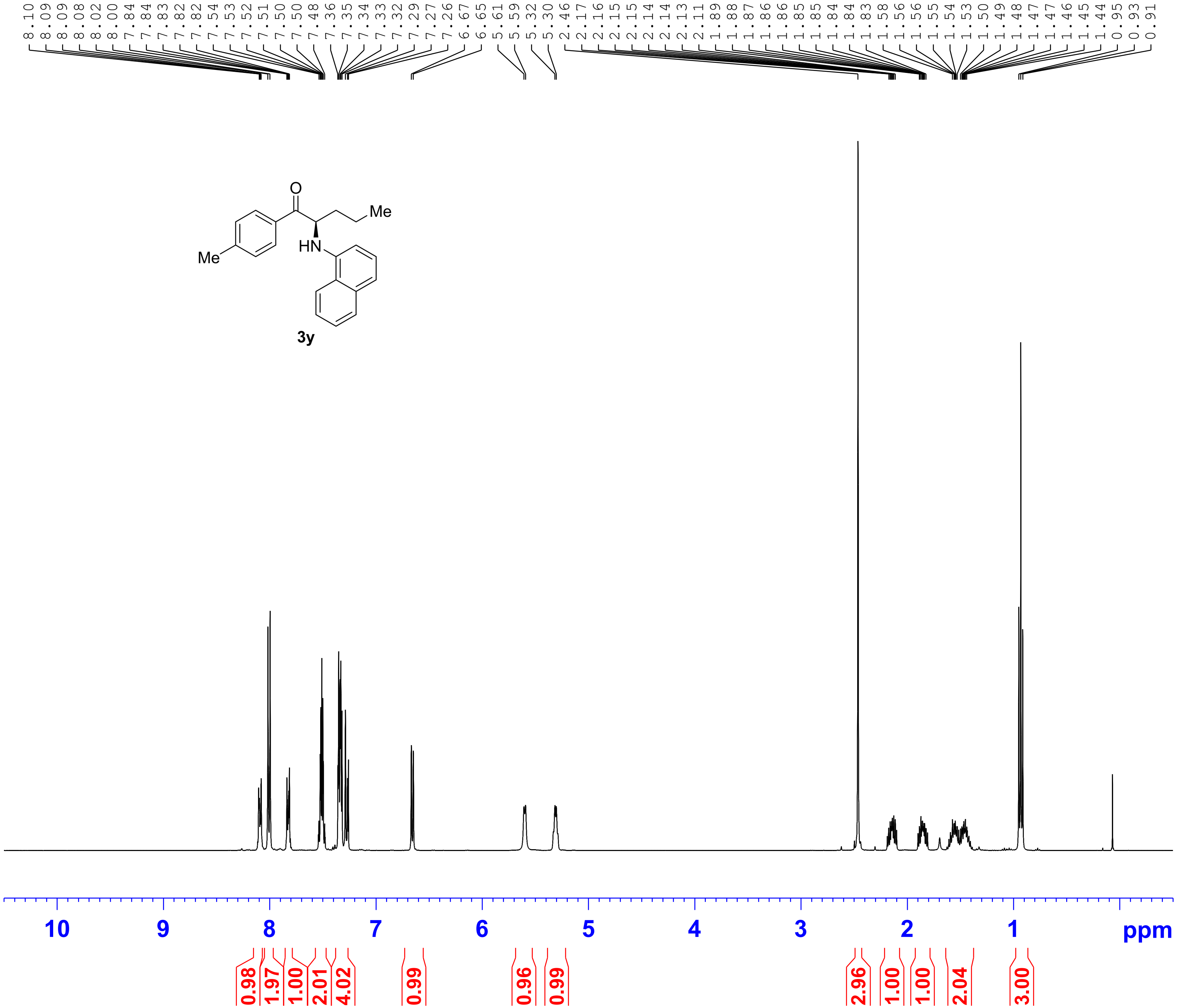

NAME	lzy-04-125-D
EXPNO	1
PROCNO	1
Date_	20200927
Time	19.36
INSTRUM	spect
PROBHD	5 mm PABBO BB/
PULPROG	zg30
TD	65536
SOLVENT	CDCl3
NS	4
DS	0
SWH	8012.820 Hz
FIDRES	0.122266 Hz
AQ	4.0894966 sec
RG	27.78
DW	62.400 usec
DE	6.50 usec
TE	297.6 K
D1	1.00000000 sec
TD0	1

======== CHANNEL f1 ========

SFO1	400.1324710 MHz
NUC1	1H
P1	14.50 usec
SI	65536
SF	400.1300096 MHz
WDW	EM
SSB	0
LB	0.30 Hz
GB	0
PC	1.00

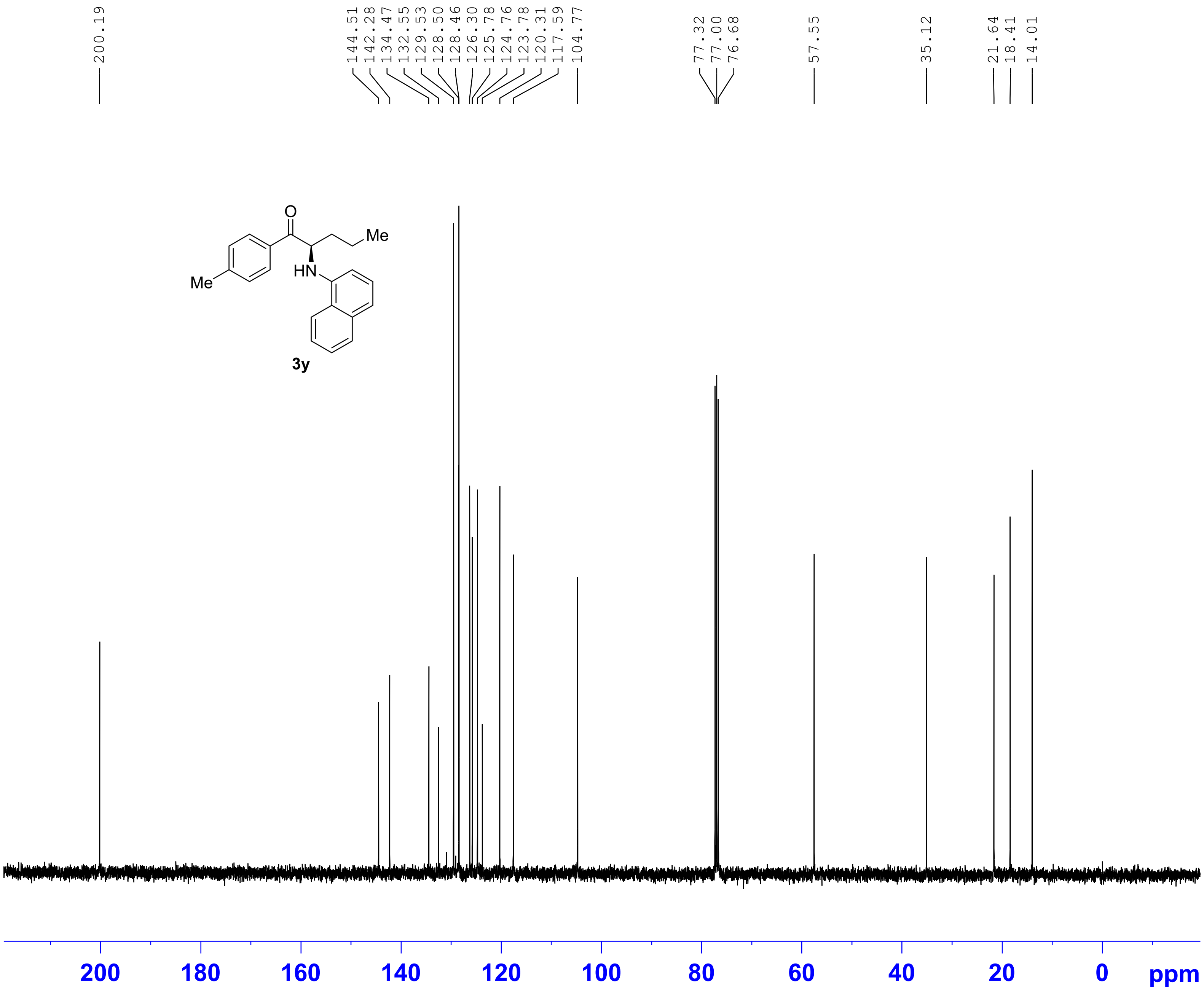


```
NAME      lzy-04-125-D-C
EXPNO                  1
PROCNO                 1
Date_           20200927
Time               19.40
INSTRUM            spect
PROBHD    5 mm PABBO BB/
PULPROG          zgpg30
TD                65536
SOLVENT            CDCl3
NS                    49
DS                     0
SWH           24038.461 Hz
FIDRES         0.366798 Hz
AQ            1.3631988 sec
RG                196.92
DW                20.800 usec
DE                  6.50 usec
TE                 298.4 K
D1            2.00000000 sec
D11           0.03000000 sec
TD0                    1

======== CHANNEL f1 ========
SFO1        100.6228298 MHz
NUC1                 13C
P1                  9.70 usec
SI                 32768
SF          100.6127824 MHz
WDW                   EM
SSB                    0
LB                  1.00 Hz
GB                     0
PC                  1.40
```

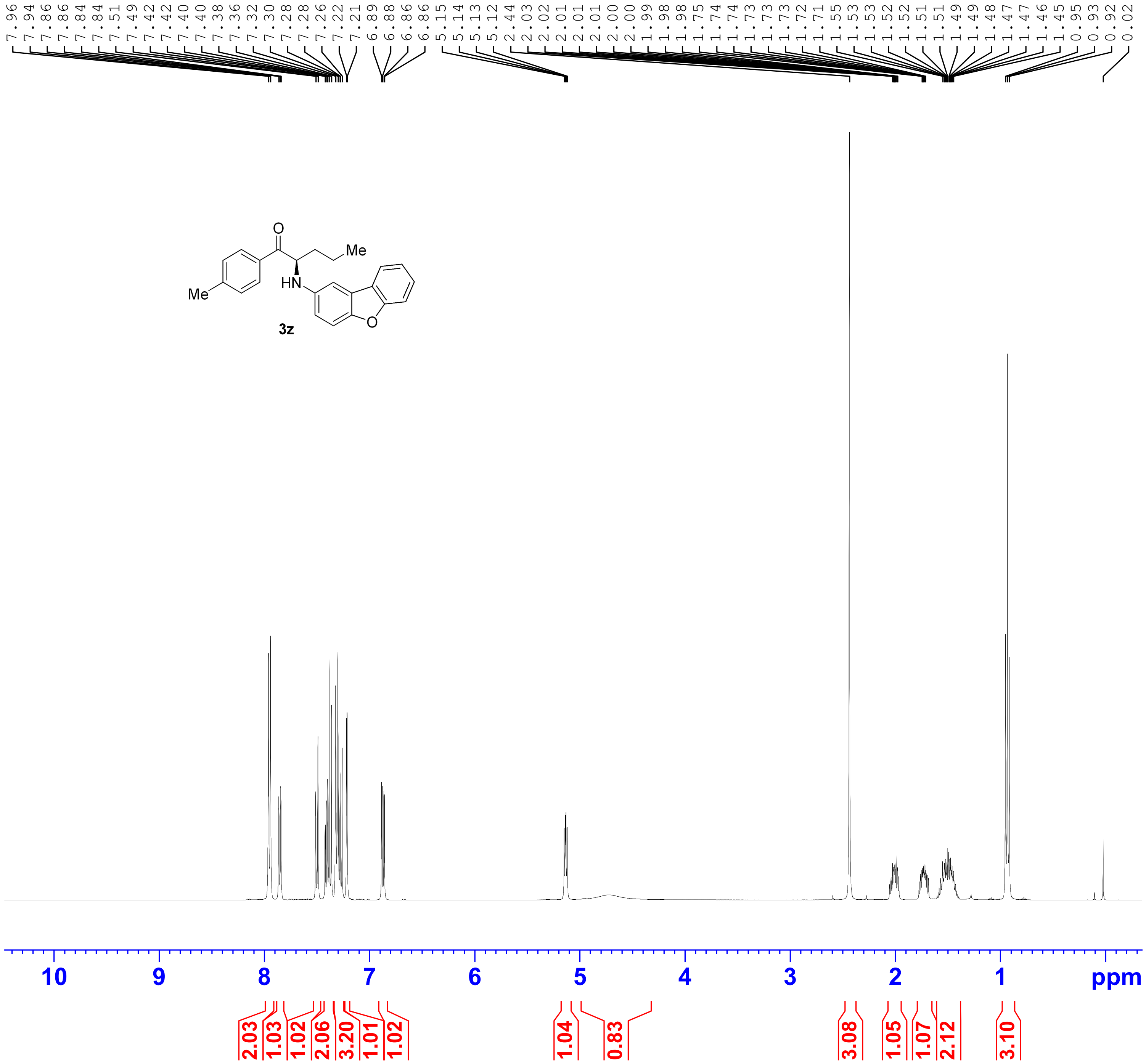

Current Data Parameters
NAME luoy-2-129a
EXPNO 1
PROCNO 1

F2 - Acquisition Parameters
Date_ 20260628
Time 16.55
INSTRUM AvanceNeo 400MHz
PROBHD Z163739_0629 (PI HR-BBO400S1-BBF/H/D-5.0-Z SP)
PULPROG zg30
TD 65536
SOLVENT CDCl3
NS 16
DS 2
SWH 8196.722 Hz
FIDRES 0.250144 Hz
AQ 3.9976959 sec
RG 101
DW 61.000 usec
DE 13.89 usec
TE 297.9 K
D1 1.00000000 sec
TD0 1
SFO1 400.1824711 MHz
NUC1 1H
P0 2.67 usec
P1 8.00 usec
PLW1 21.26700020 W

F2 - Processing parameters
SI 65536
SF 400.1800095 MHz
WDW EM
SSB 0
LB 0.30 Hz
GB 0
PC 1.00

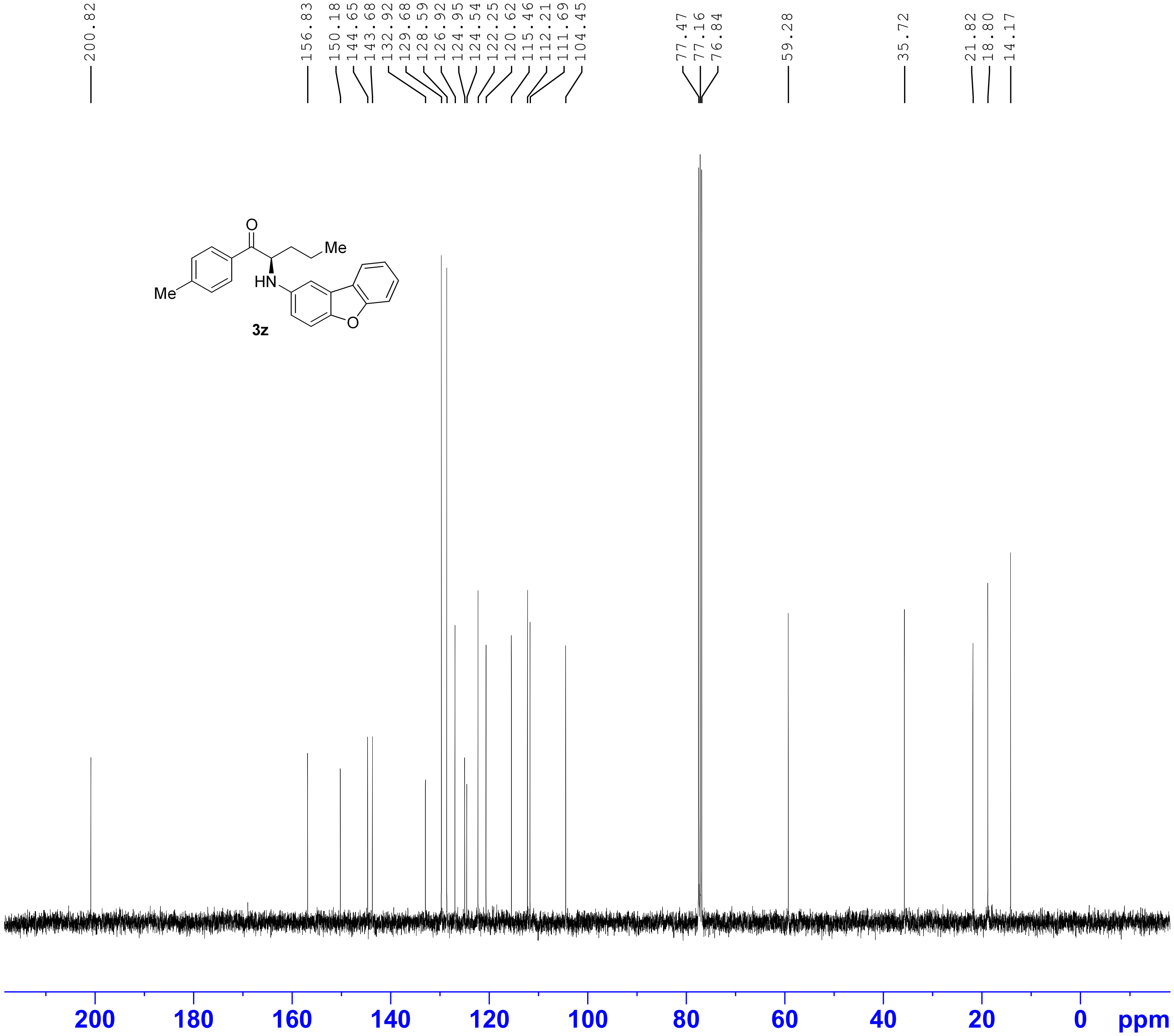


```
Current Data Parameters
NAME              luoy-2-129a
EXPNO                       2
PROCNO                      1

F2 - Acquisition Parameters
Date_                20260628
Time                    17.03
INSTRUM        AvanceNeo 400MHz
PROBHD     Z163739_0629 (PI HR-BBO400S1-BBF/H/D-5.0-Z SP)
PULPROG                zgpg30
TD                      65536
SOLVENT                 CDCl3
NS                        103
DS                          4
SWH                 23809.523 Hz
FIDRES               0.726609 Hz
AQ                  1.3762560 sec
RG                         10
DW                     21.000 usec
DE                       6.50 usec
TE                      298.4 K
D1                 2.00000000 sec
D11                0.03000000 sec
TD0                         1
SFO1              100.6354036 MHz
NUC1                      13C
P0                       2.67 usec
P1                       8.00 usec
PLW1              85.25399780 W
SFO2              400.1816007 MHz
NUC2                       1H
CPDPRG[2]             waltz65
PCPD2                   90.00 usec
PLW2              21.26700020 W
PLW12              0.16802999 W
PLW13              0.08452000 W

F2 - Processing parameters
SI                      32768
SF                100.6253329 MHz
WDW                        EM
SSB                         0
LB                       1.00 Hz
GB                          0
PC                       1.40
```

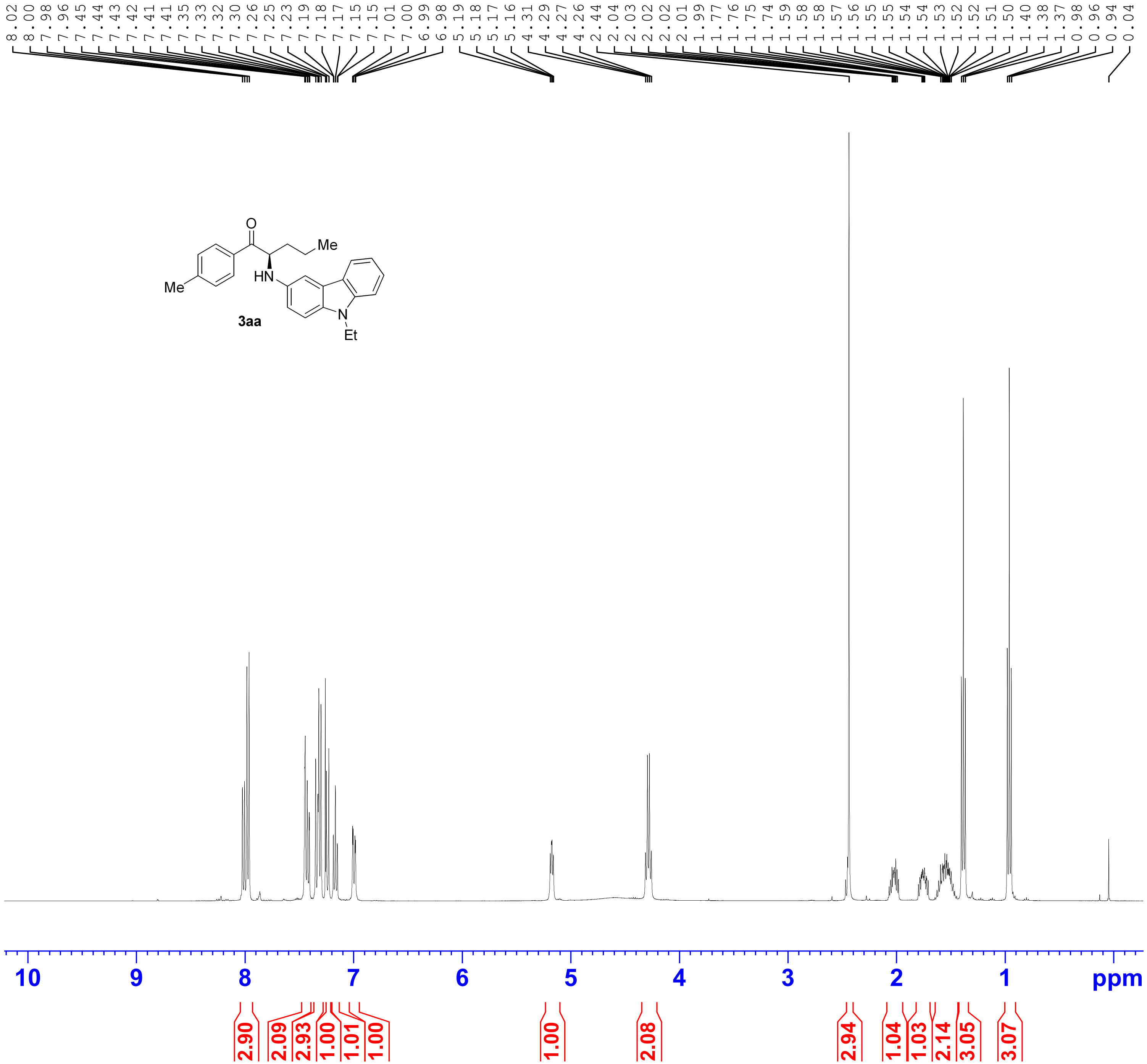

3aa

```
Current Data Parameters
NAME              luoy-2-141a
EXPNO                       1
PROCNO                      1

F2 - Acquisition Parameters
Date_                20260707
Time                    20.02
INSTRUM                 spect
PROBHD     5 mm PABBO BB/19F-1H/D Z-GRD Z108618/0582
PULPROG                  zg30
TD                      65536
SOLVENT                 CDCl3
NS                         16
DS                          2
SWH                  8012.820 Hz
FIDRES               0.122266 Hz
AQ                  4.0894465 sec
RG                      27.78
DW                     62.400 usec
DE                       6.50 usec
TE                      296.0 K
D1                 1.00000000 sec
TD0                         1

======== CHANNEL f1 ========
SFO1              400.1324710 MHz
NUC1                       1H
P1                      14.50 usec
PLW1              11.99499989 W

F2 - Processing parameters
SI                      65536
SF                400.1300096 MHz
WDW                        EM
SSB                         0
LB                       0.30 Hz
GB                          0
PC                       1.00
```

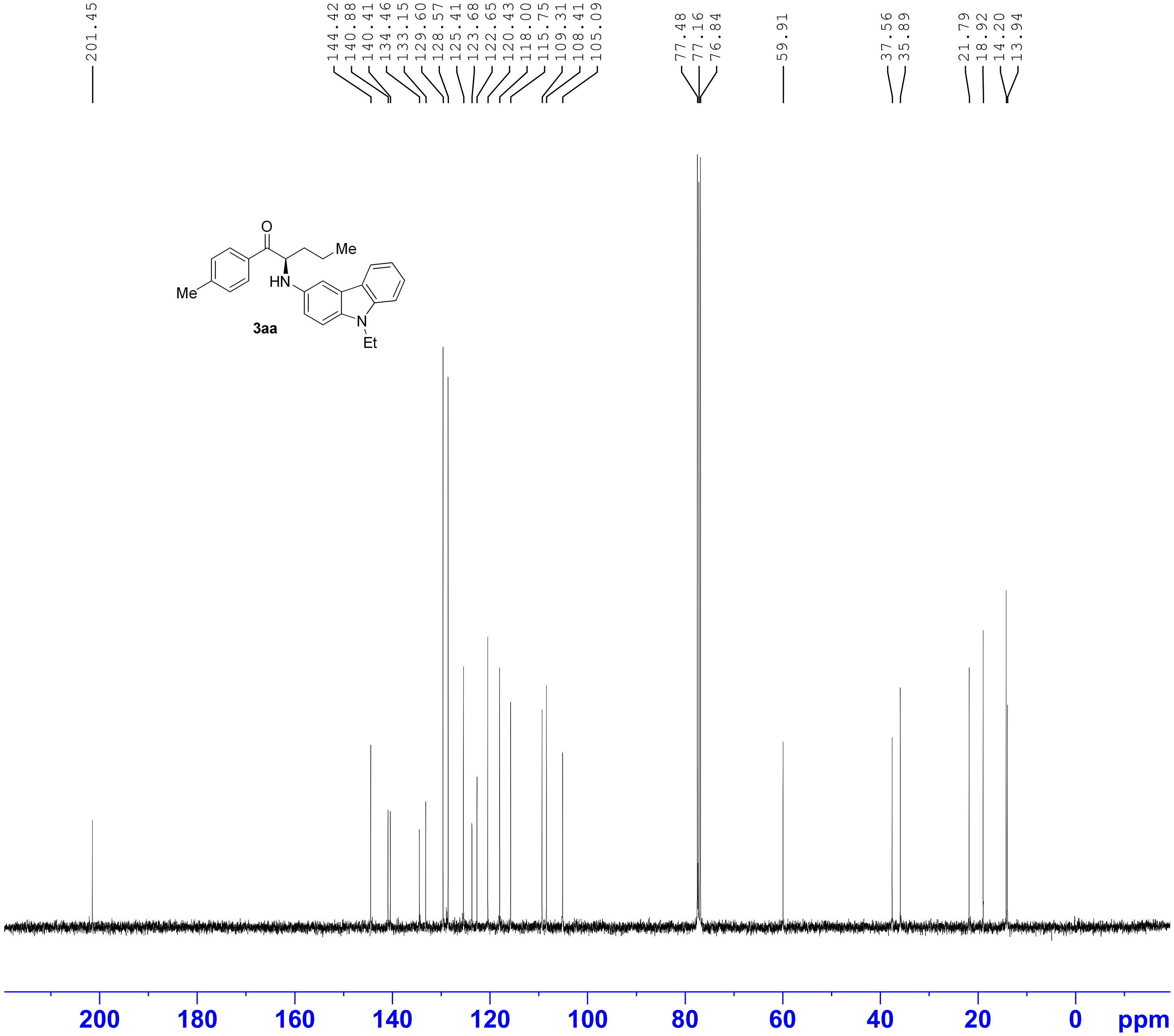

Current Data Parameters
NAME luoy-2-141a
EXPNO 2
PROCNO 1

F2 - Acquisition Parameters
Date_ 20260707
Time 20.11
INSTRUM spect
PROBHD 5 mm PABBO BB/19F-1H/D Z-GRD Z108618/0582
PULPROG zgpg30
TD 65536
SOLVENT CDCl3
NS 190
DS 2
SWH 24038.461 Hz
FIDRES 0.366798 Hz
AQ 1.3631488 sec
RG 196.92
DW 20.800 usec
DE 6.50 usec
TE 297.0 K
D1 2.00000000 sec
D11 0.03000000 sec
TD0 1

======== CHANNEL f1 ========
SFO1 100.6228298 MHz
NUC1 13C
P1 9.70 usec
PLW1 46.98899841 W

======== CHANNEL f2 ========
SFO2 400.1316005 MHz
NUC2 1H
CPDPRG[2] waltz16
PCPD2 90.00 usec
PLW2 11.99499989 W
PLW12 0.34213999 W
PLW13 0.27713001 W

F2 - Processing parameters
SI 32768
SF 100.6127633 MHz
WDW EM
SSB 0
LB 1.00 Hz
GB 0
PC 1.40

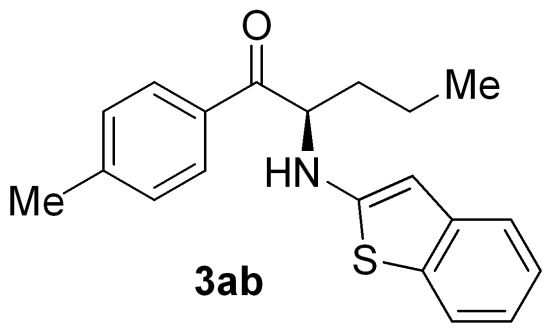

3ab

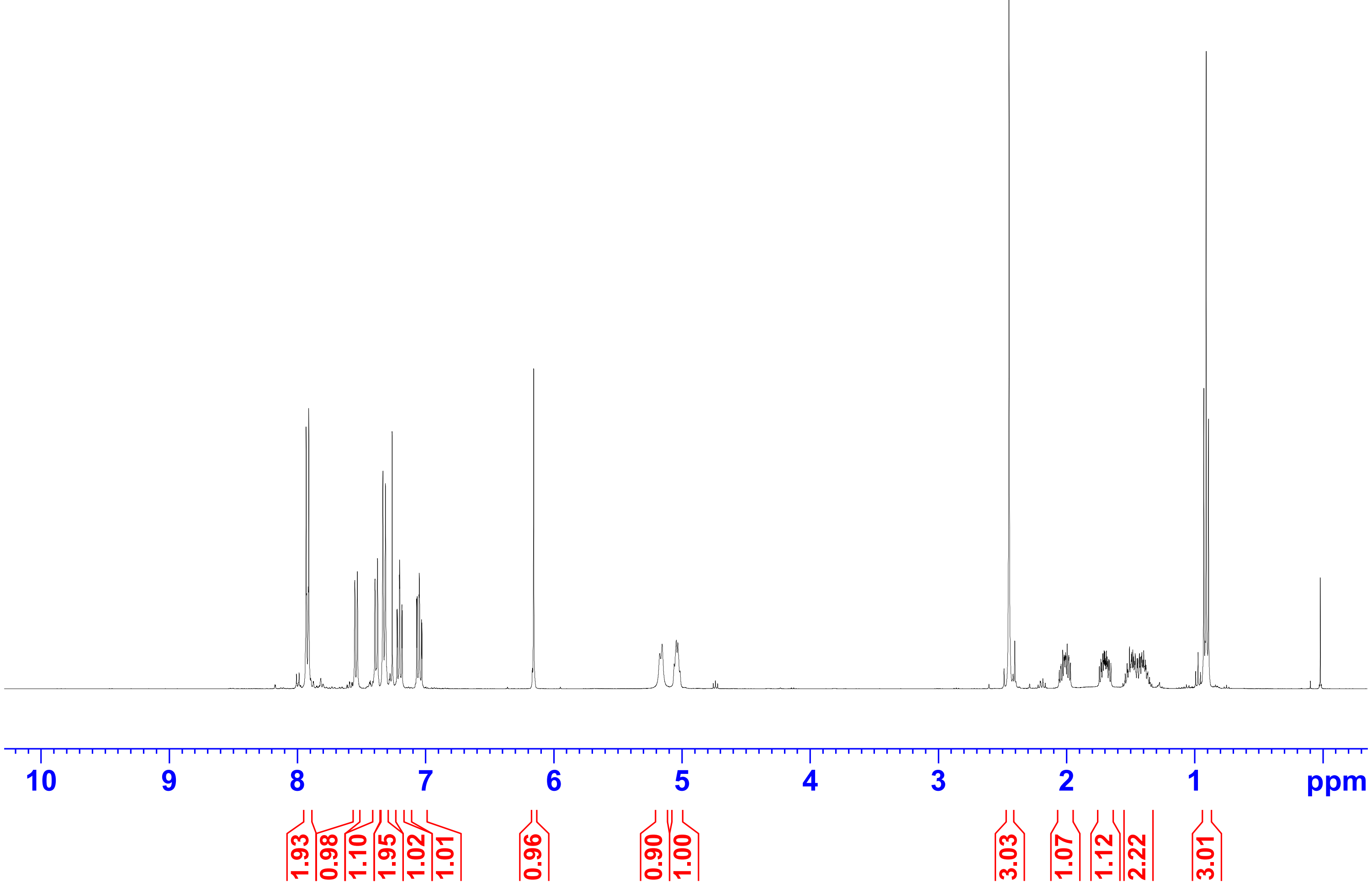

Current Data Parameters
NAME luoy-2-138a
EXPNO 5
PROCNO 1

F2 - Acquisition Parameters
Date_ 20260705
Time 20.40
INSTRUM AvanceNeo 400MHz
PROBHD Z163739_0629 (PI HR-BBO400S1-BBF/H/D-5.0-Z SP)
PULPROG zg30
TD 65536
SOLVENT CDCl3
NS 16
DS 2
SWH 8196.722 Hz
FIDRES 0.250144 Hz
AQ 3.9976959 sec
RG 101
DW 61.000 usec
DE 13.89 usec
TE 296.7 K
D1 1.00000000 sec
TD0 1
SFO1 400.1824711 MHz
NUC1 1H
P0 2.67 usec
P1 8.00 usec
PLW1 21.26700020 W

F2 - Processing parameters
SI 65536
SF 400.1800096 MHz
WDW no
SSB 0
LB 0 Hz
GB 0
PC 1.00

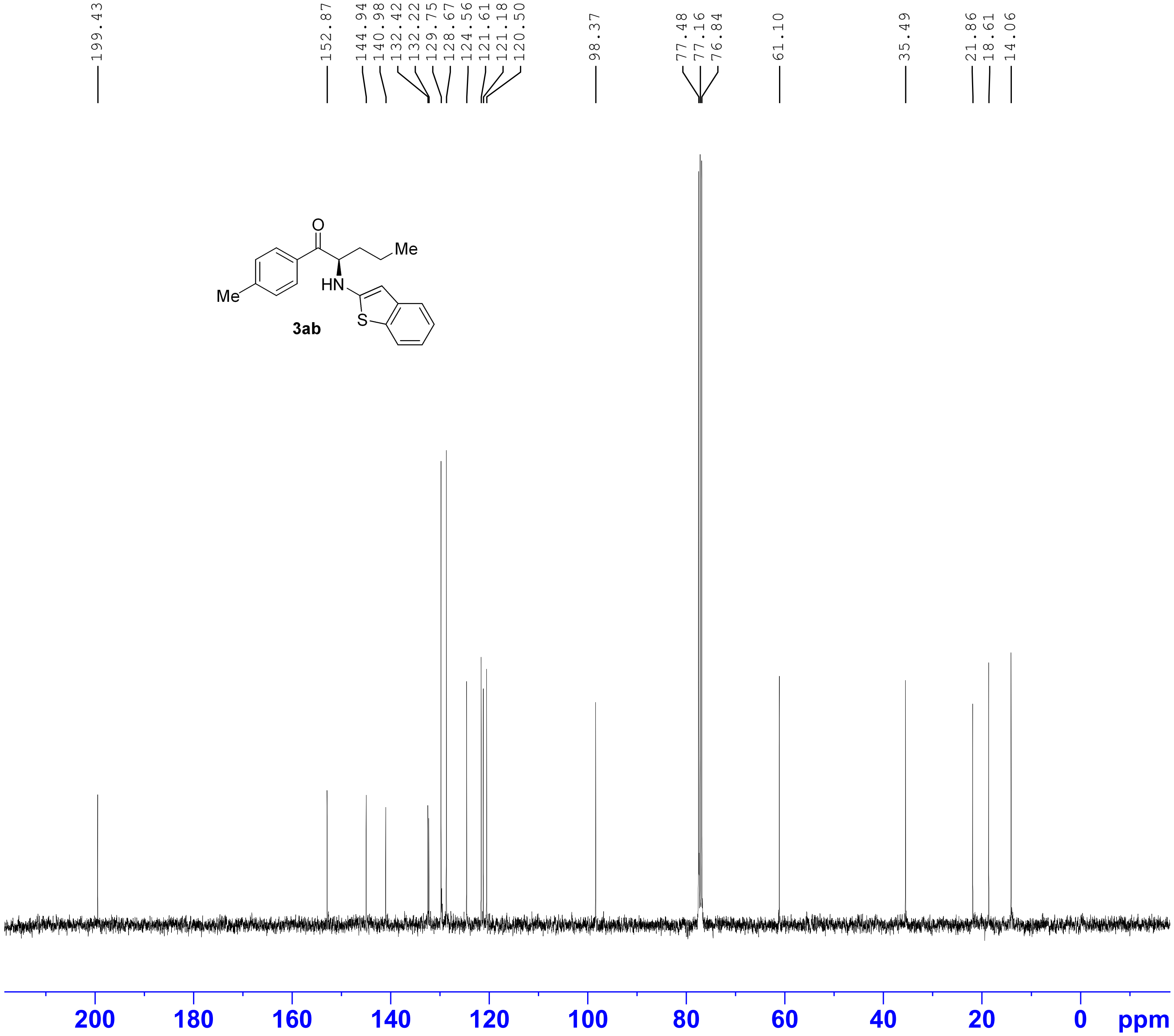


```
Current Data Parameters
NAME              luoy-2-138a
EXPNO                       6
PROCNO                      1

F2 - Acquisition Parameters
Date_                20260705
Time                    20.47
INSTRUM       AvanceNeo 400MHz
PROBHD     Z163739_0629 (PI HR-BBO400S1-BBF/H/D-5.0-Z SP)
PULPROG                zgpg30
TD                      65536
SOLVENT                 CDCl3
NS                        101
DS                          4
SWH                 23809.523 Hz
FIDRES               0.726609 Hz
AQ                  1.3762560 sec
RG                         16
DW                     21.000 usec
DE                       6.50 usec
TE                      297.4 K
D1                 2.00000000 sec
D11                0.03000000 sec
TD0                         1
SFO1              100.6354036 MHz
NUC1                      13C
P0                       2.67 usec
P1                       8.00 usec
PLW1              85.25399780 W
SFO2              400.1816007 MHz
NUC2                       1H
CPDPRG[2]             waltz65
PCPD2                   90.00 usec
PLW2              21.26700020 W
PLW12              0.16802999 W
PLW13              0.08452000 W

F2 - Processing parameters
SI                      32768
SF                100.6253326 MHz
WDW                        EM
SSB                         0
LB                       2.00 Hz
GB                          0
PC                       1.40
```

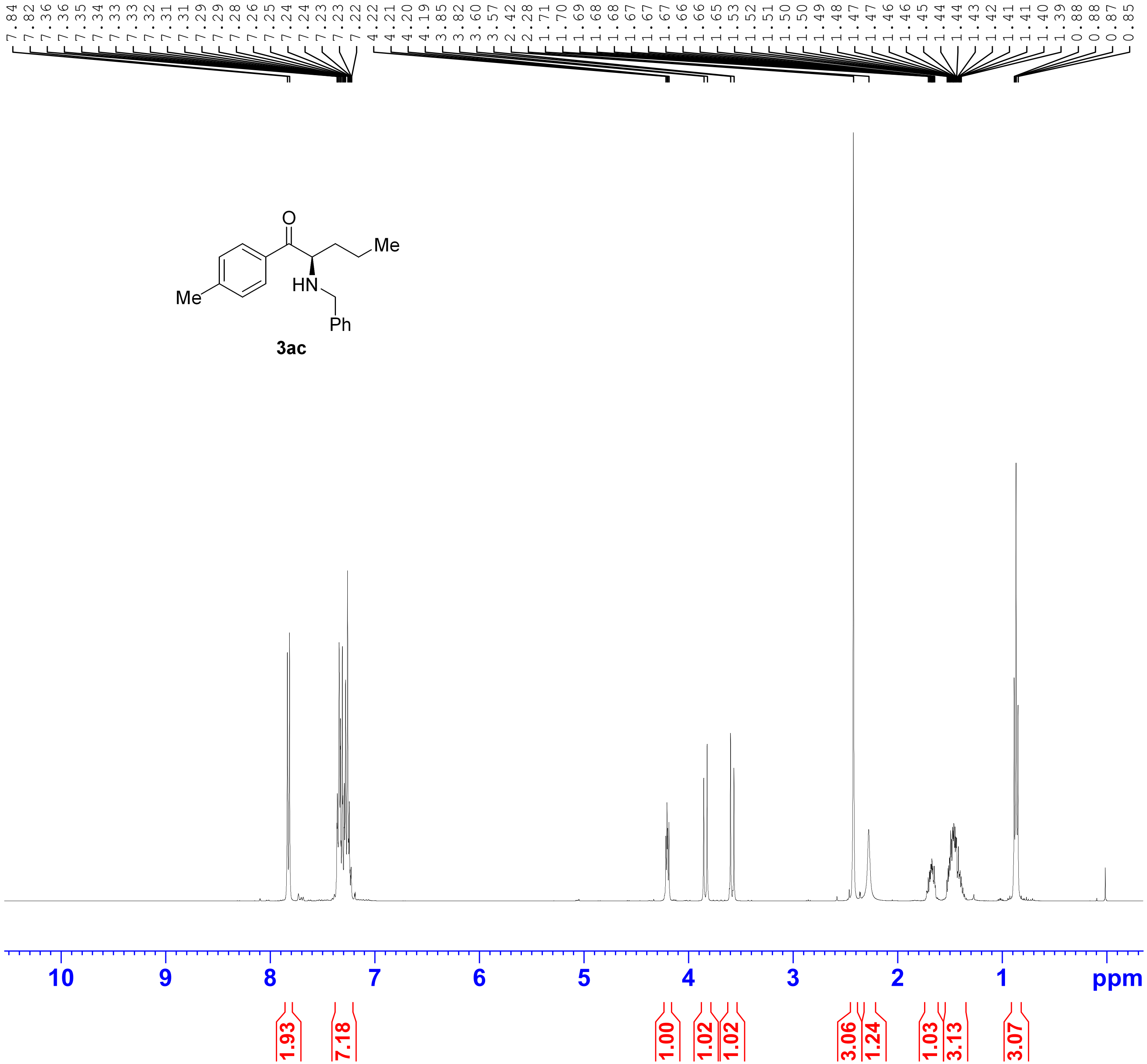

3ac


```
Current Data Parameters
NAME               luoy-2-137b
EXPNO                        7
PROCNO                       1

F2 - Acquisition Parameters
Date_                 20260705
Time                     20.27
INSTRUM        AvanceNeo 400MHz
PROBHD     Z163739_0629 (PI HR-BBO400S1-BBF/H/D-5.0-Z SP)
PULPROG                   zg30
TD                       65536
SOLVENT                  CDCl3
NS                          16
DS                           2
SWH                   8196.722 Hz
FIDRES                0.250144 Hz
AQ                   3.9976959 sec
RG                        71.8
DW                      61.000 usec
DE                       13.89 usec
TE                       296.7 K
D1                  1.00000000 sec
TD0                          1
SFO1               400.1824711 MHz
NUC1                        1H
P0                        2.67 usec
P1                        8.00 usec
PLW1               21.26700020 W

F2 - Processing parameters
SI                       65536
SF                 400.1800095 MHz
WDW                         EM
SSB                          0
LB                        0.30 Hz
GB                           0
PC                        1.00
```

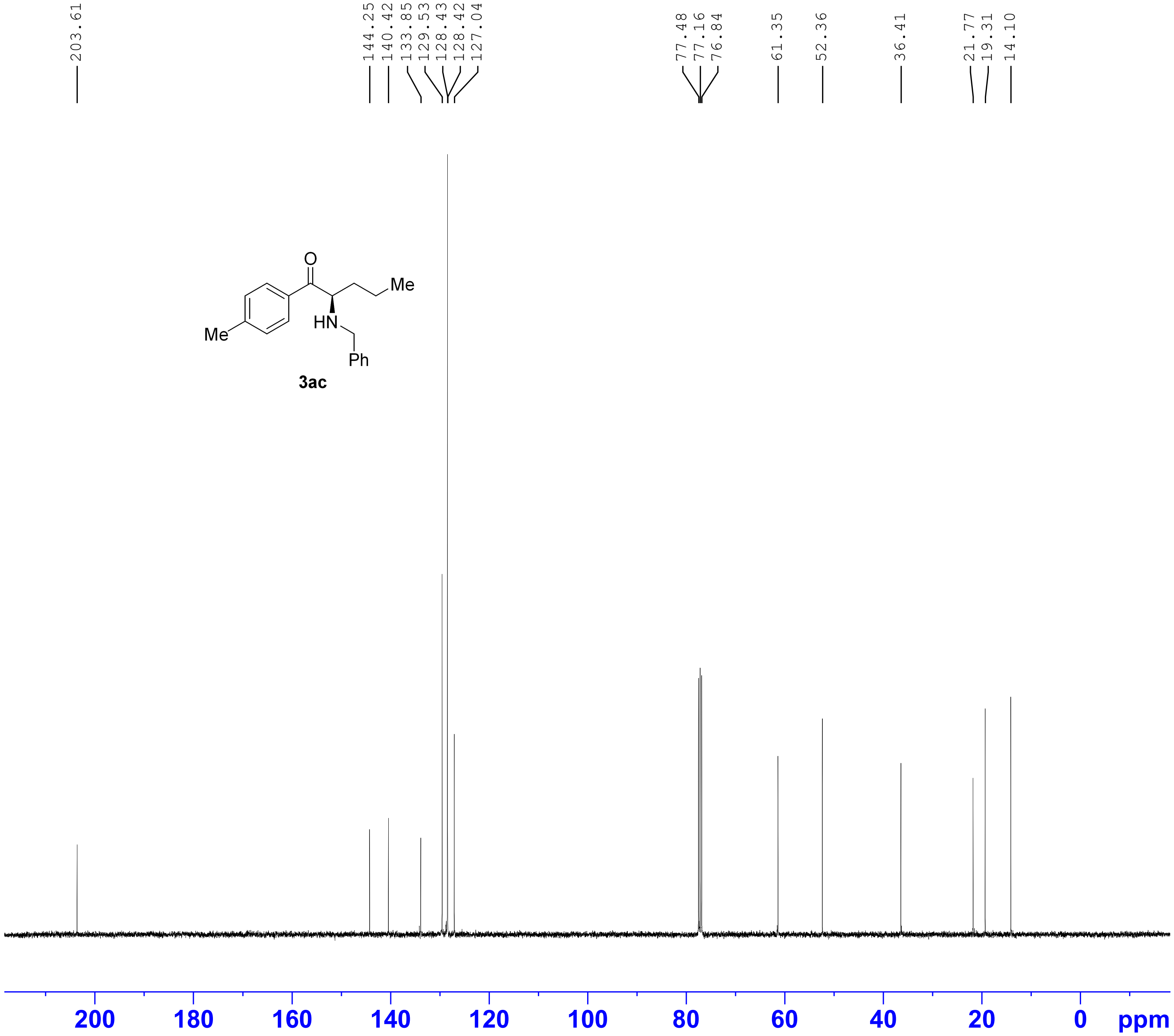


```
Current Data Parameters
NAME                luoy-2-137b
EXPNO                         6
PROCNO                        1

F2 - Acquisition Parameters
Date_                  20260705
Time                      20.35
INSTRUM        AvanceNeo 400MHz
PROBHD     Z163739_0629 (PI HR-BBO400S1-BBF/H/D-5.0-Z SP)
PULPROG                 zgpg30
TD                        65536
SOLVENT                   CDCl3
NS                          119
DS                            4
SWH                   23809.523 Hz
FIDRES                 0.726609 Hz
AQ                    1.3762560 sec
RG                           16
DW                       21.000 usec
DE                         6.50 usec
TE                        297.4 K
D1                   2.00000000 sec
D11                  0.03000000 sec
TD0                           1
SFO1                100.6354036 MHz
NUC1                        13C
P0                         2.67 usec
P1                         8.00 usec
PLW1                85.25399780 W
SFO2                400.1816007 MHz
NUC2                         1H
CPDPRG[2]               waltz65
PCPD2                     90.00 usec
PLW2                21.26700020 W
PLW12                0.16802999 W
PLW13                0.08452000 W

F2 - Processing parameters
SI                        32768
SF                  100.6253339 MHz
WDW                          EM
SSB                           0
LB                         1.00 Hz
GB                            0
PC                         1.40
```

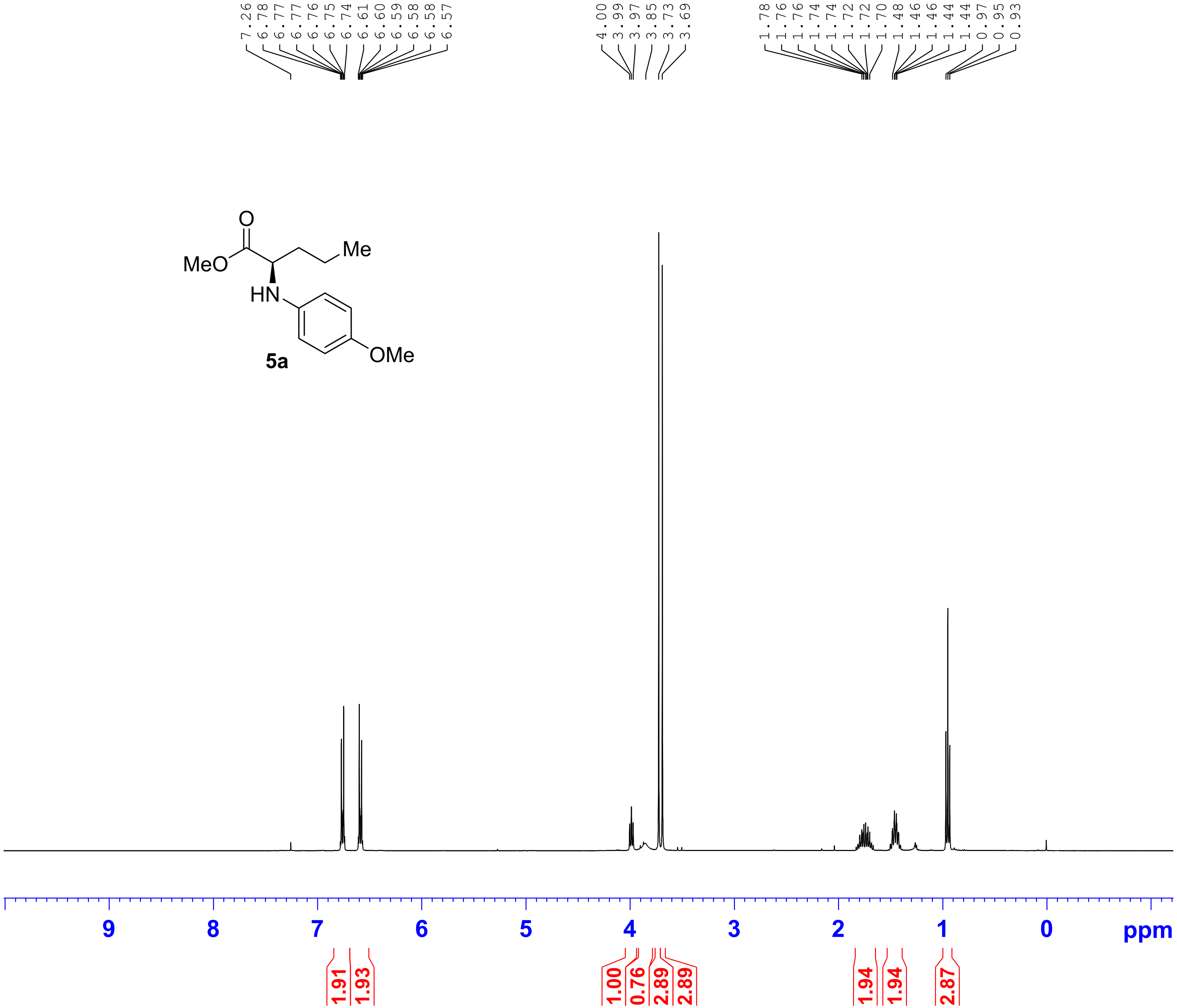

NAME	lzy-09-08-A
EXPNO	1
PROCNO	1
Date_	20220127
Time_	21.56
INSTRUM	spect
PROBHD	5 mm DUL 13C-1
PULPROG	zg30
TD	65536
SOLVENT	CDCl3
NS	3
DS	0
SWH	8223.685 Hz
FIDRES	0.125483 Hz
AQ	3.9846387 sec
RG	64
DW	60.800 usec
DE	6.00 usec
TE	294.0 K
D1	1.00000000 sec
TD0	1

======== CHANNEL f1 ========

NUC1	1H
P1	15.80 usec
PL1	-1.00 dB
PL1W	12.17476940 W
SFO1	400.1324710 MHz
SI	32768
SF	400.1300102 MHz
WDW	EM
SSB	0
LB	0.30 Hz
GB	0
PC	1.00

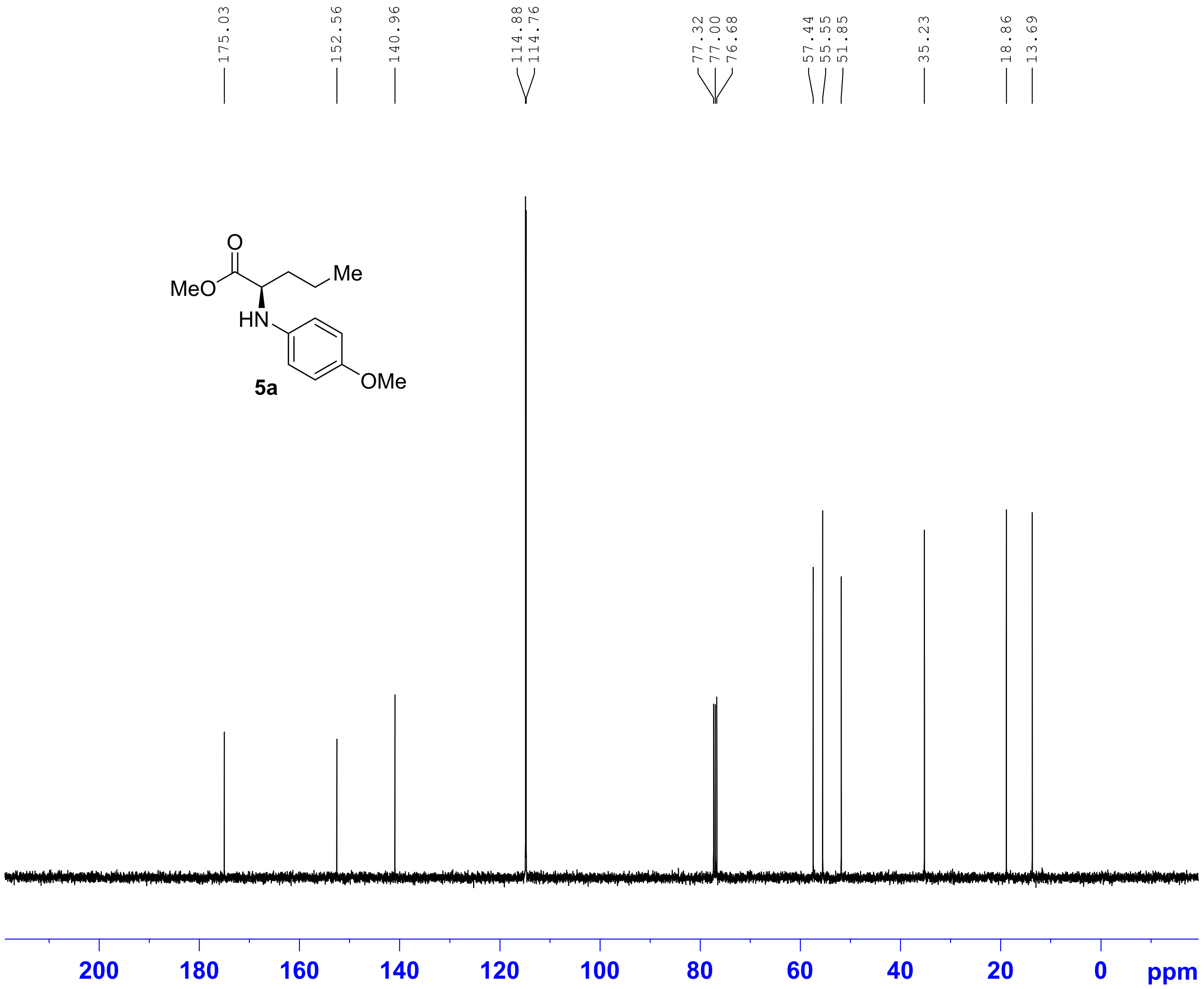

NAME	lzy-09-08-A-C	
EXPNO	1	
PROCNO	1	
Date_	20220127	
Time	21.57	
INSTRUM	spect	
PROBHD	5 mm DUL 13C-1	
PULPROG	zgpg30	
TD	65536	
SOLVENT	CDCl3	
NS	91	
DS	1	
SWH	24038.461	Hz
FIDRES	0.366798	Hz
AQ	1.3631988	sec
RG	2050	
DW	20.800	usec
DE	6.00	usec
TE	294.3	K
D1	2.00000000	sec
D11	0.03000000	sec
TD0	1	
======== CHANNEL f1 ========		
NUC1	13C	
P1	40.00	usec
PL1	-3.00	dB
PL1W	60.64365387	W
SFO1	100.6228298	MHz
======== CHANNEL f2 ========		
CPDPRG2	waltz16	
NUC2	1H	
PCPD2	80.00	usec
PL2	-1.00	dB
PL12	14.39	dB
PL13	18.00	dB
PL2W	12.17476940	W
PL12W	0.35193357	W
PL13W	0.15327126	W
SFO2	400.1316005	MHz
SI	32768	
SF	100.6127813	MHz
WDW	EM	
SSB	0	
LB	1.00	Hz
GB	0	
PC	1.40	

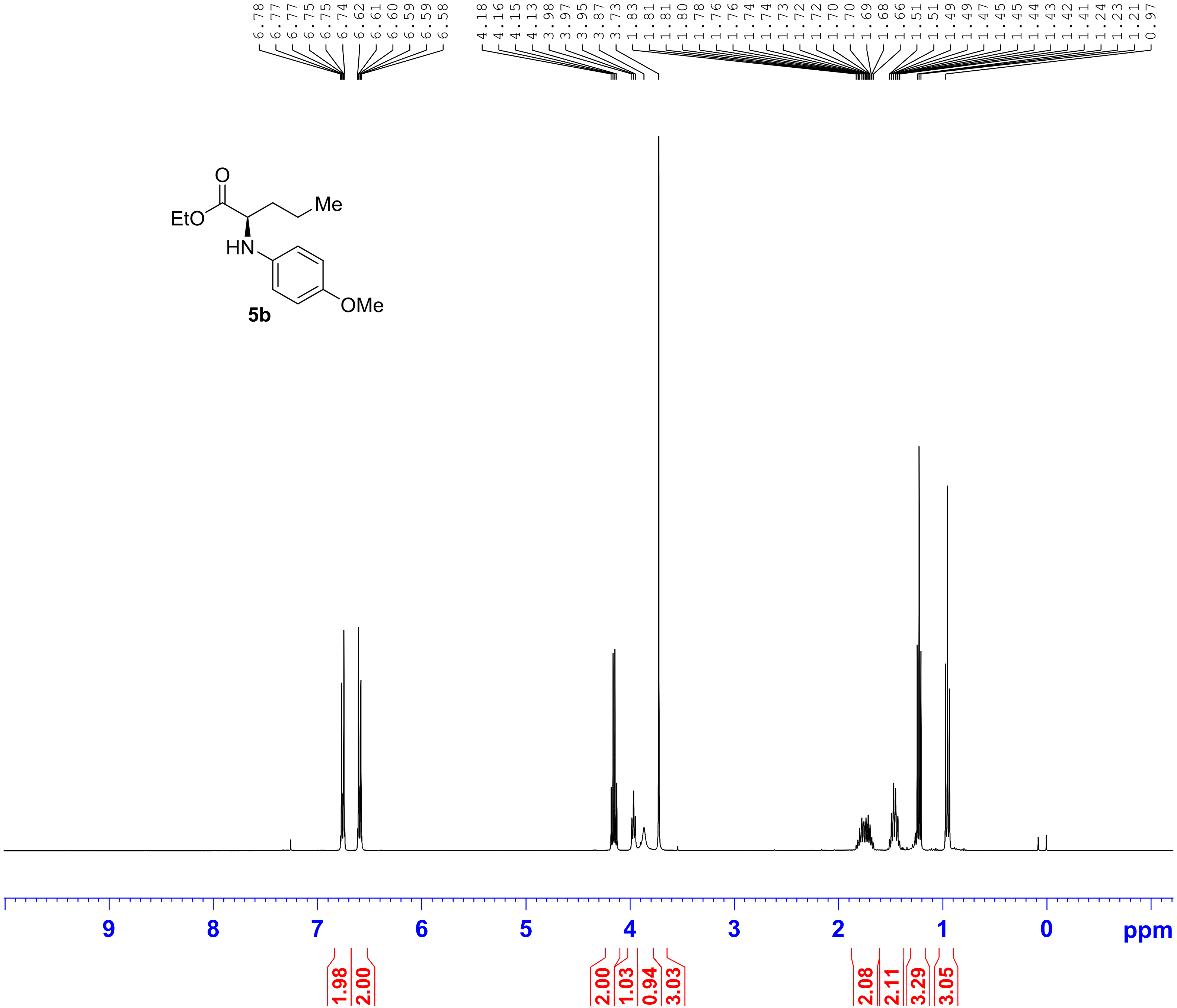

NAME	lzy-09-9-A-p-OMe
EXPNO	2
PROCNO	1
Date_	20220303
Time_	15.15
INSTRUM	spect
PROBHD	5 mm PABBO BB/
PULPROG	zg30
TD	65536
SOLVENT	CDCl3
NS	4
DS	0
SWH	8012.820 Hz
FIDRES	0.122266 Hz
AQ	4.0894966 sec
RG	22.47
DW	62.400 usec
DE	6.50 usec
TE	294.6 K
D1	1.00000000 sec
TD0	1

======== CHANNEL f1 ========

SFO1	400.1324710 MHz
NUC1	1H
P1	14.50 usec
SI	65536
SF	400.1300100 MHz
WDW	EM
SSB	0
LB	0.30 Hz
GB	0
PC	1.00

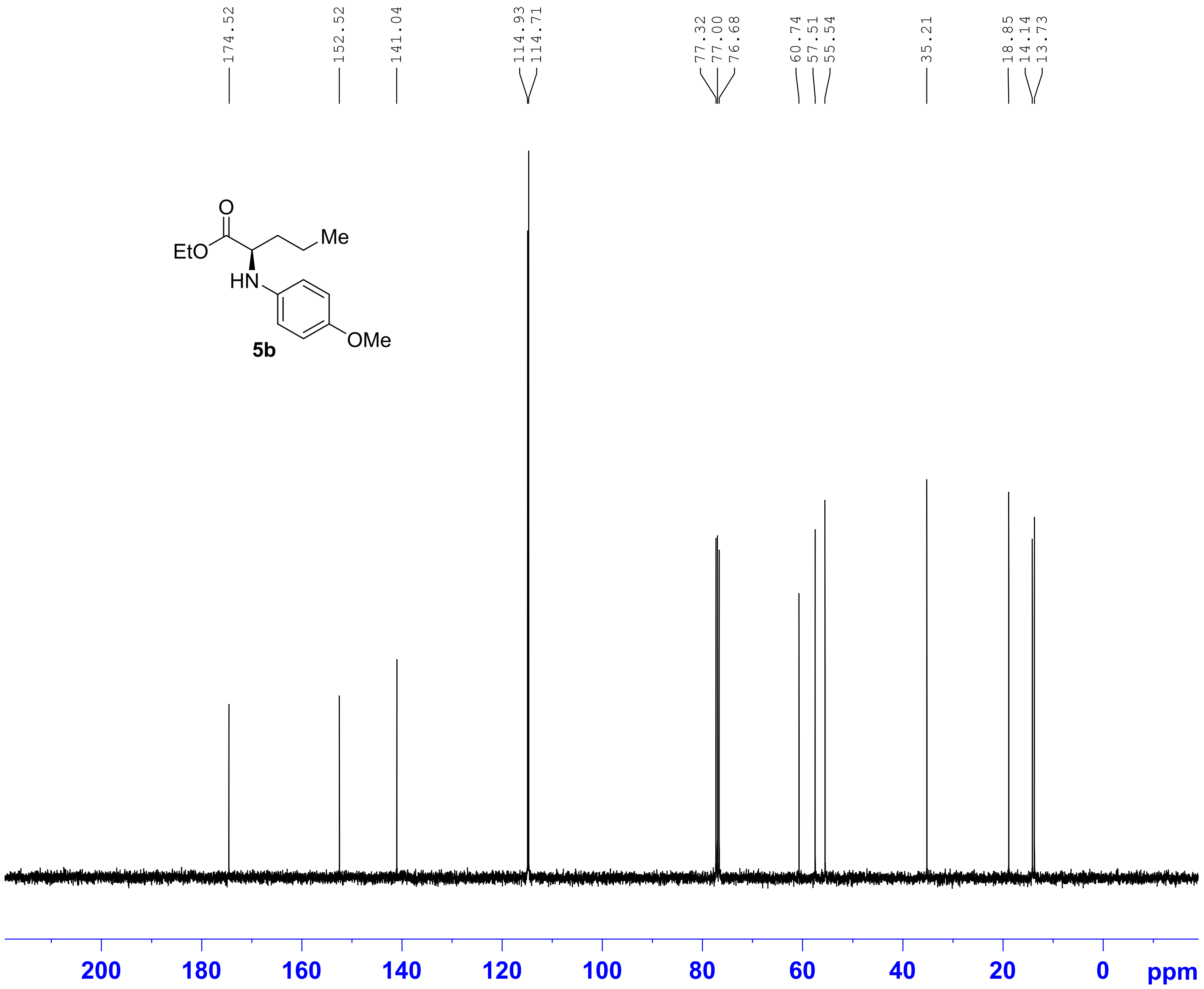

NAME	lzy-09-9-A-p-OMe-C
EXPNO	2
PROCNO	1
Date_	20220303
Time	15.20
INSTRUM	spect
PROBHD	5 mm PABBO BB/
PULPROG	zgpg30
TD	65536
SOLVENT	CDCl3
NS	20
DS	0
SWH	24038.461 Hz
FIDRES	0.366798 Hz
AQ	1.3631988 sec
RG	196.92
DW	20.800 usec
DE	6.50 usec
TE	295.2 K
D1	2.00000000 sec
D11	0.03000000 sec
TD0	1

======== CHANNEL f1 ========

SFO1	100.6228298 MHz
NUC1	13C
P1	9.70 usec
SI	32768
SF	100.6127816 MHz
WDW	EM
SSB	0
LB	1.00 Hz
GB	0
PC	1.40

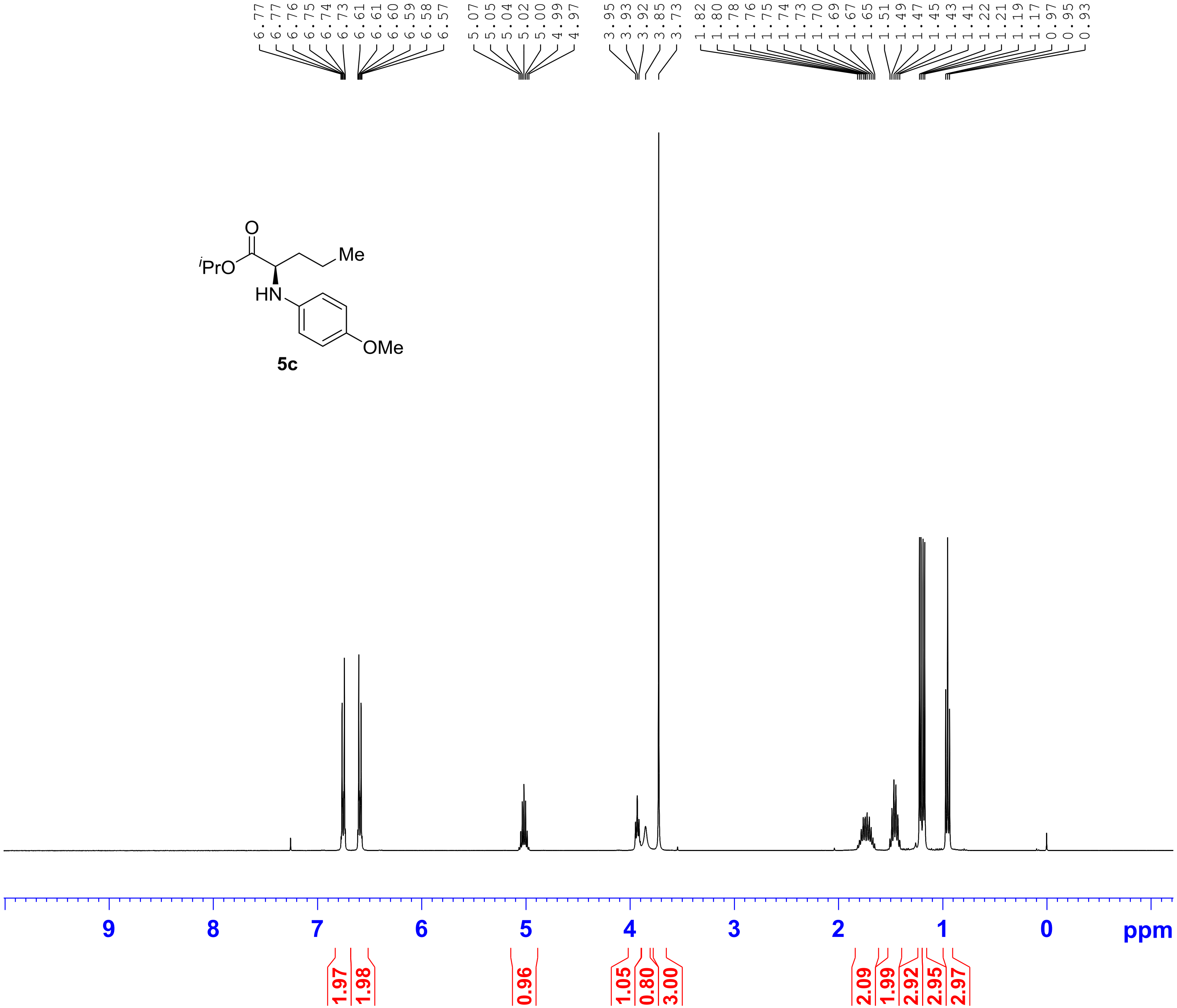

NAME	lzy-09-36
EXPNO	1
PROCNO	1
Date_	20220301
Time	12.00
INSTRUM	spect
PROBHD	5 mm DUL 13C-1
PULPROG	zg30
TD	65536
SOLVENT	CDCl3
NS	4
DS	0
SWH	8223.685 Hz
FIDRES	0.125483 Hz
AQ	3.9846387 sec
RG	64
DW	60.800 usec
DE	6.00 usec
TE	293.8 K
D1	1.00000000 sec
TD0	1

======== CHANNEL f1 ========

NUC1	1H
P1	15.80 usec
PL1	-1.00 dB
PL1W	12.17476940 W
SFO1	400.1324710 MHz
SI	32768
SF	400.1300097 MHz
WDW	EM
SSB	0
LB	0.30 Hz
GB	0
PC	1.00

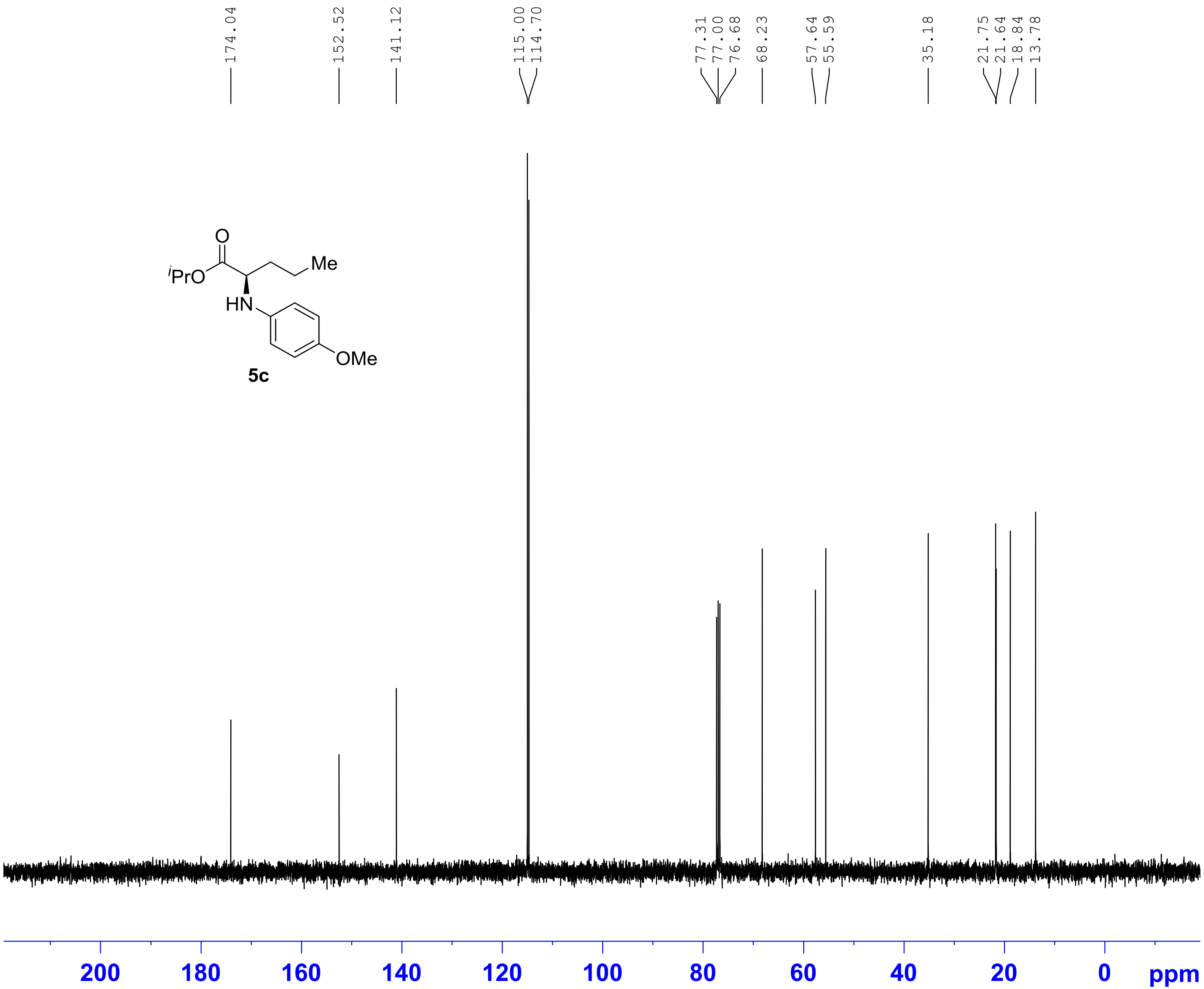

NAME	lzy-09-36-C
EXPNO	1
PROCNO	1
Date_	20220301
Time_	12.04
INSTRUM	spect
PROBHD	5 mm DUL 13C-1
PULPROG	zgpg30
TD	65536
SOLVENT	CDCl3
NS	46
DS	0
SWH	24038.461 Hz
FIDRES	0.366798 Hz
AQ	1.3631988 sec
RG	2050
DW	20.800 usec
DE	6.00 usec
TE	294.1 K
D1	2.00000000 sec
D11	0.03000000 sec
TD0	1
======== CHANNEL f1 ========	
NUC1	13C
P1	40.00 usec
PL1	-3.00 dB
PL1W	60.64365387 W
SFO1	100.6228298 MHz
======== CHANNEL f2 ========	
CPDPRG2	waltz16
NUC2	1H
PCPD2	80.00 usec
PL2	-1.00 dB
PL12	14.39 dB
PL13	18.00 dB
PL2W	12.17476940 W
PL12W	0.35193357 W
PL13W	0.15327126 W
SFO2	400.1316005 MHz
SI	32768
SF	100.6127789 MHz
WDW	EM
SSB	0
LB	1.00 Hz
GB	0
PC	1.40

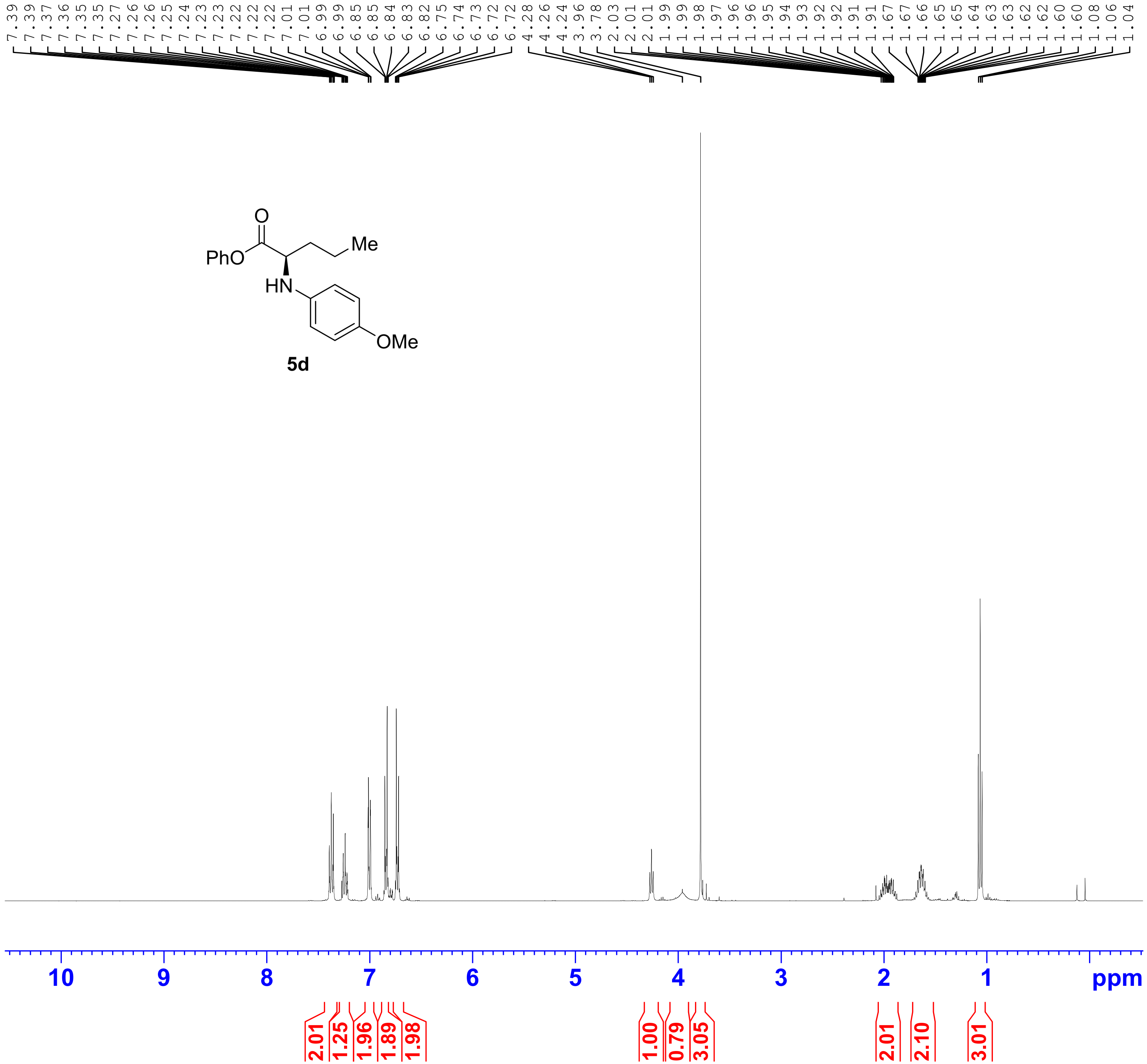

Current Data Parameters
NAME lzy-08-182-A
EXPNO 1
PROCNO 1

F2 - Acquisition Parameters
Date_ 20220215
Time 22.06
INSTRUM spect
PROBHD 5 mm DUL 13C-1H/D Z-GRD Z8246/0122
PULPROG zg30
TD 65536
SOLVENT CDCl3
NS 4
DS 0
SWH 8223.685 Hz
FIDRES 0.125483 Hz
AQ 3.9845889 sec
RG 128
DW 60.800 usec
DE 6.00 usec
TE 294.3 K
D1 1.00000000 sec
TD0 1

======== CHANNEL f1 ========
NUC1 1H
P1 15.80 usec
PL1 -1.00 dB
PL1W 12.17476940 no
SFO1 400.1324710 MHz

F2 - Processing parameters
SI 32768
SF 400.1300054 MHz
WDW EM
SSB 0
LB 0.30 Hz
GB 0
PC 1.00

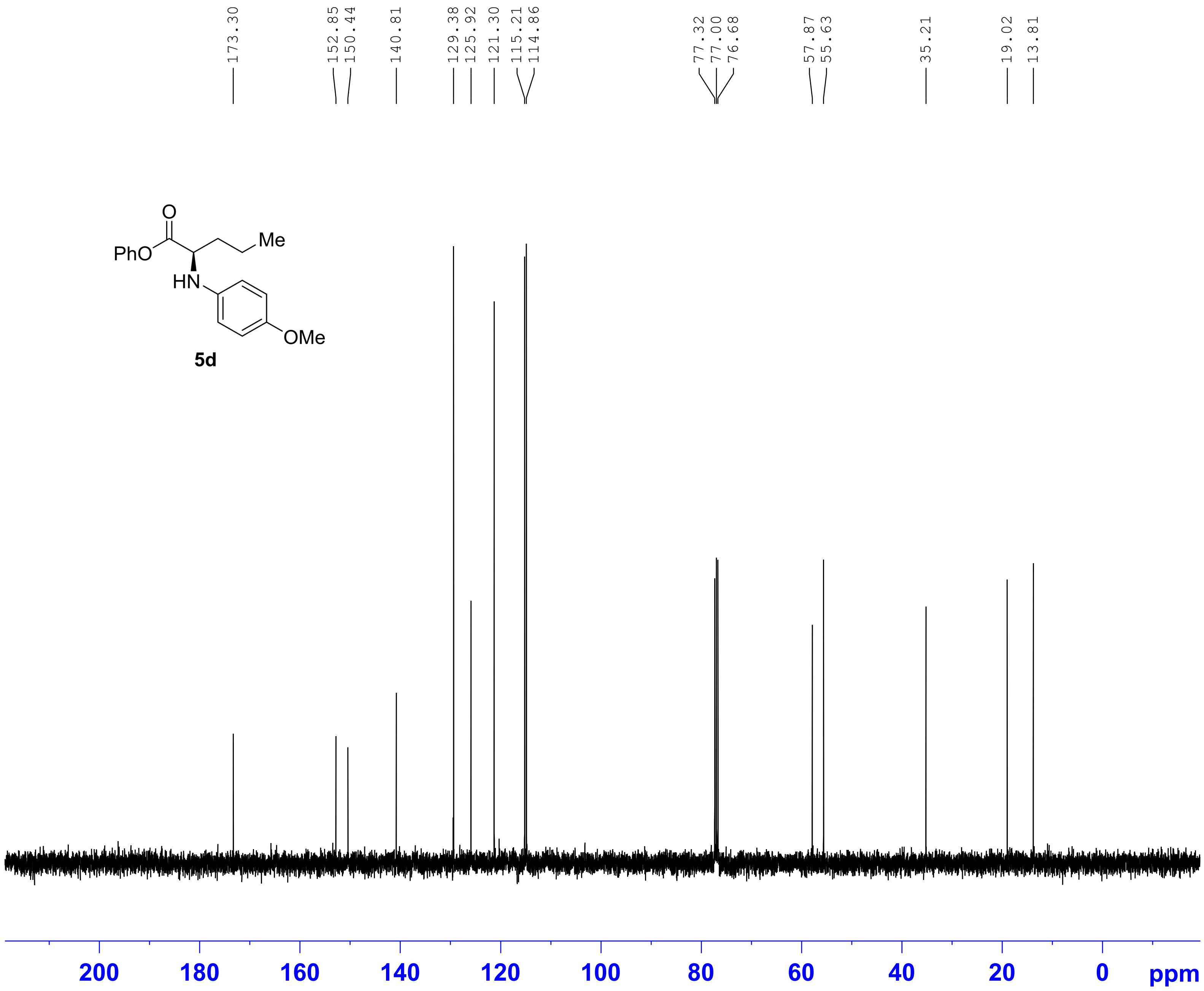

NAME	lzy-08-182-A-C
EXPNO	1
PROCNO	1
Date_	20220215
Time_	22.08
INSTRUM	spect
PROBHD	5 mm DUL 13C-1
PULPROG	zgpg30
TD	65536
SOLVENT	CDCl3
NS	35
DS	1
SWH	24038.461 Hz
FIDRES	0.366798 Hz
AQ	1.3631988 sec
RG	2050
DW	20.800 usec
DE	6.00 usec
TE	294.6 K
D1	2.00000000 sec
D11	0.03000000 sec
TD0	1
======== CHANNEL f1 ========	
NUC1	13C
P1	40.00 usec
PL1	-3.00 dB
PL1W	60.64365387 W
SFO1	100.6228298 MHz
======== CHANNEL f2 ========	
CPDPRG2	waltz16
NUC2	1H
PCPD2	80.00 usec
PL2	-1.00 dB
PL12	14.39 dB
PL13	18.00 dB
PL2W	12.17476940 W
PL12W	0.35193357 W
PL13W	0.15327126 W
SFO2	400.1316005 MHz
SI	32768
SF	100.6127803 MHz
WDW	EM
SSB	0
LB	1.00 Hz
GB	0
PC	1.40

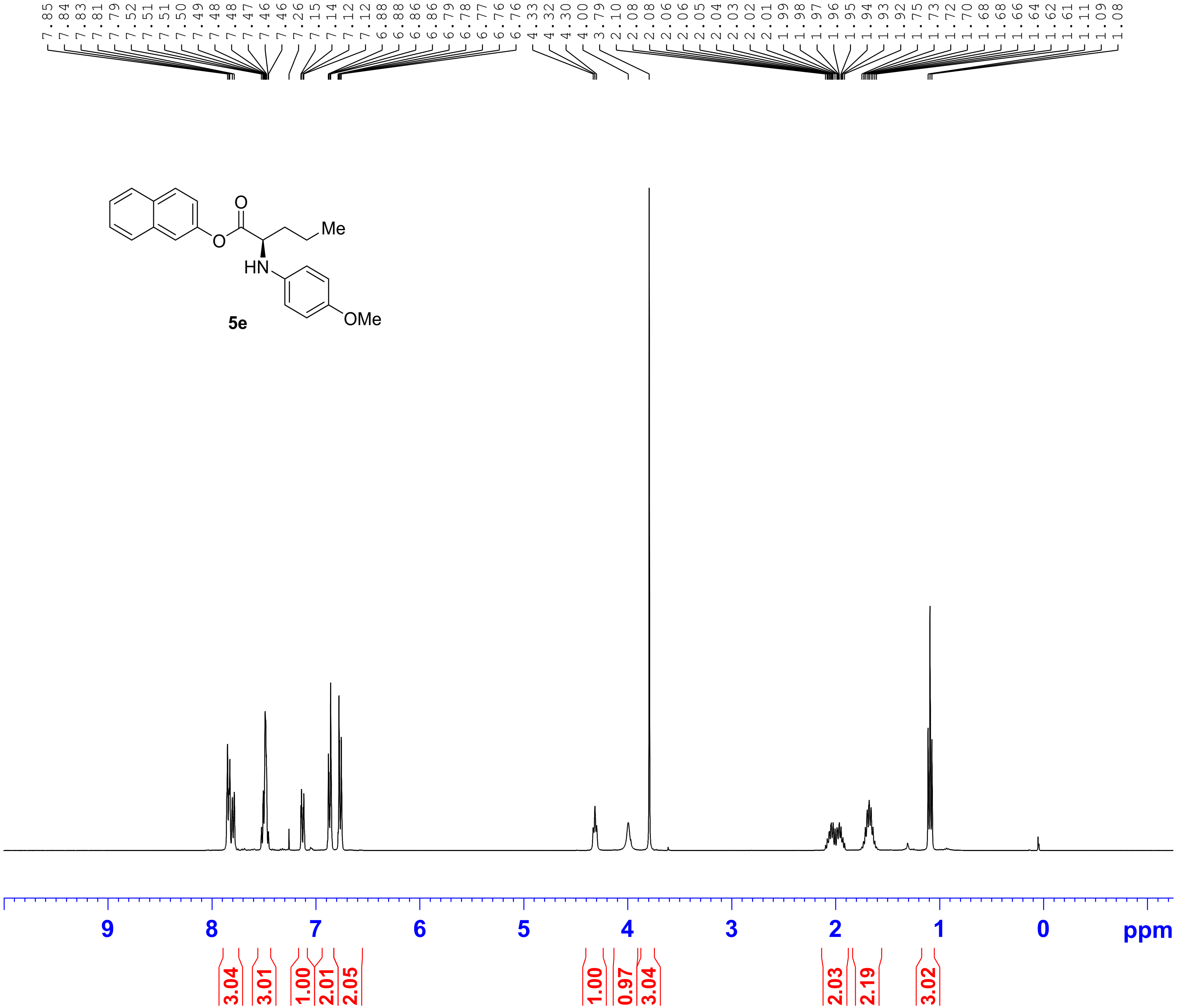

NAME	lzy-08-182-B-3
EXPNO	2
PROCNO	1
Date_	20220227
Time_	11.05
INSTRUM	spect
PROBHD	5 mm DUL 13C-1
PULPROG	zg30
TD	65536
SOLVENT	CDCl3
NS	4
DS	0
SWH	8223.685 Hz
FIDRES	0.125483 Hz
AQ	3.9846387 sec
RG	101
DW	60.800 usec
DE	6.00 usec
TE	294.2 K
D1	1.00000000 sec
TD0	1

======== CHANNEL f1 ========

NUC1	1H
P1	15.80 usec
PL1	-1.00 dB
PL1W	12.17476940 W
SFO1	400.1324710 MHz
SI	32768
SF	400.1300097 MHz
WDW	EM
SSB	0
LB	0.30 Hz
GB	0
PC	1.00

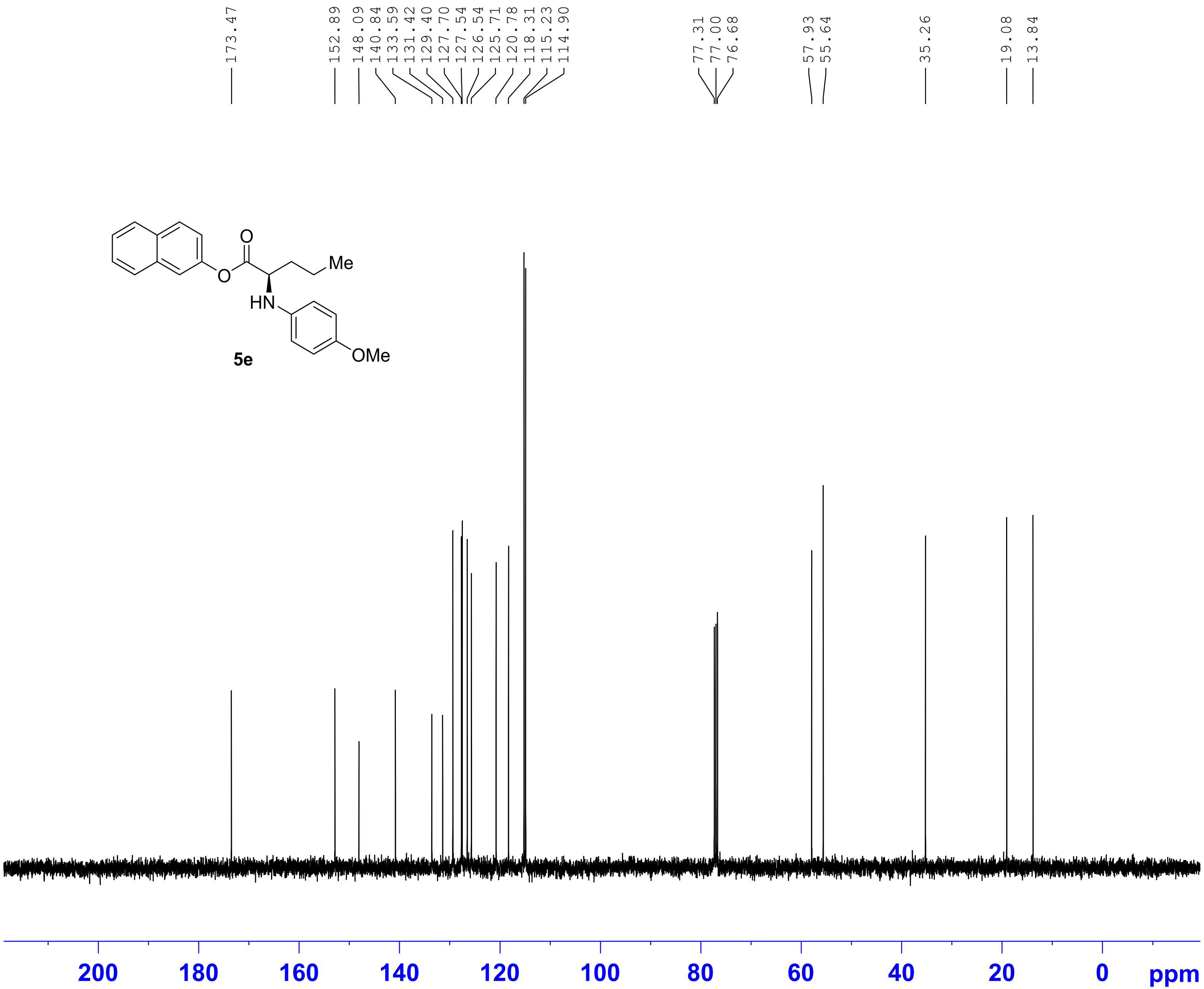

5e

NAME	lzy-08-182-B-3-C	
EXPNO	1	
PROCNO	1	
Date_	20220227	
Time	11.16	
INSTRUM	spect	
PROBHD	5 mm DUL 13C-1	
PULPROG	zgpg30	
TD	65536	
SOLVENT	CDCl3	
NS	100	
DS	0	
SWH	24038.461	Hz
FIDRES	0.366798	Hz
AQ	1.3631988	sec
RG	2050	
DW	20.800	usec
DE	6.00	usec
TE	294.5	K
D1	2.00000000	sec
D11	0.03000000	sec
TD0	1	
======== CHANNEL f1 ========		
NUC1	13C	
P1	40.00	usec
PL1	-3.00	dB
PL1W	60.64365387	W
SFO1	100.6228298	MHz
======== CHANNEL f2 ========		
CPDPRG2	waltz16	
NUC2	1H	
PCPD2	80.00	usec
PL2	-1.00	dB
PL12	14.39	dB
PL13	18.00	dB
PL2W	12.17476940	W
PL12W	0.35193357	W
PL13W	0.15327126	W
SFO2	400.1316005	MHz
SI	32768	
SF	100.6127824	MHz
WDW	EM	
SSB	0	
LB	1.00	Hz
GB	0	
PC	1.40	

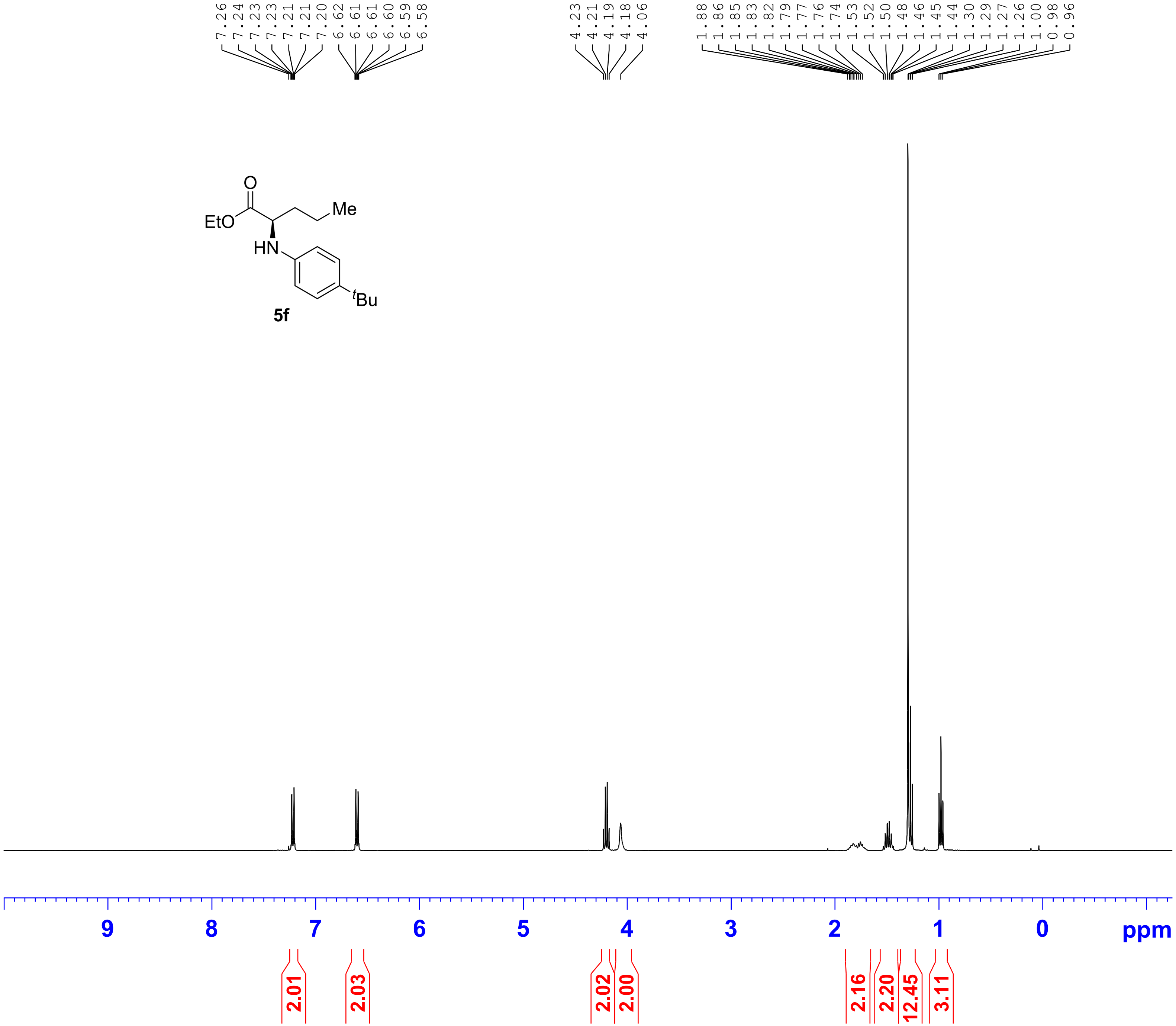

5f

NAME	lzy-08-189-A
EXPNO	2
PROCNO	1
Date_	20220228
Time_	20.37
INSTRUM	spect
PROBHD	5 mm DUL 13C-1
PULPROG	zg30
TD	65536
SOLVENT	CDCl3
NS	4
DS	0
SWH	8223.685 Hz
FIDRES	0.125483 Hz
AQ	3.9846387 sec
RG	64
DW	60.800 usec
DE	6.00 usec
TE	293.7 K
D1	1.00000000 sec
TD0	1

======== CHANNEL f1 ========

NUC1	1H
P1	15.80 usec
PL1	-1.00 dB
PL1W	12.17476940 W
SFO1	400.1324710 MHz
SI	32768
SF	400.1300097 MHz
WDW	EM
SSB	0
LB	0.30 Hz
GB	0
PC	1.00

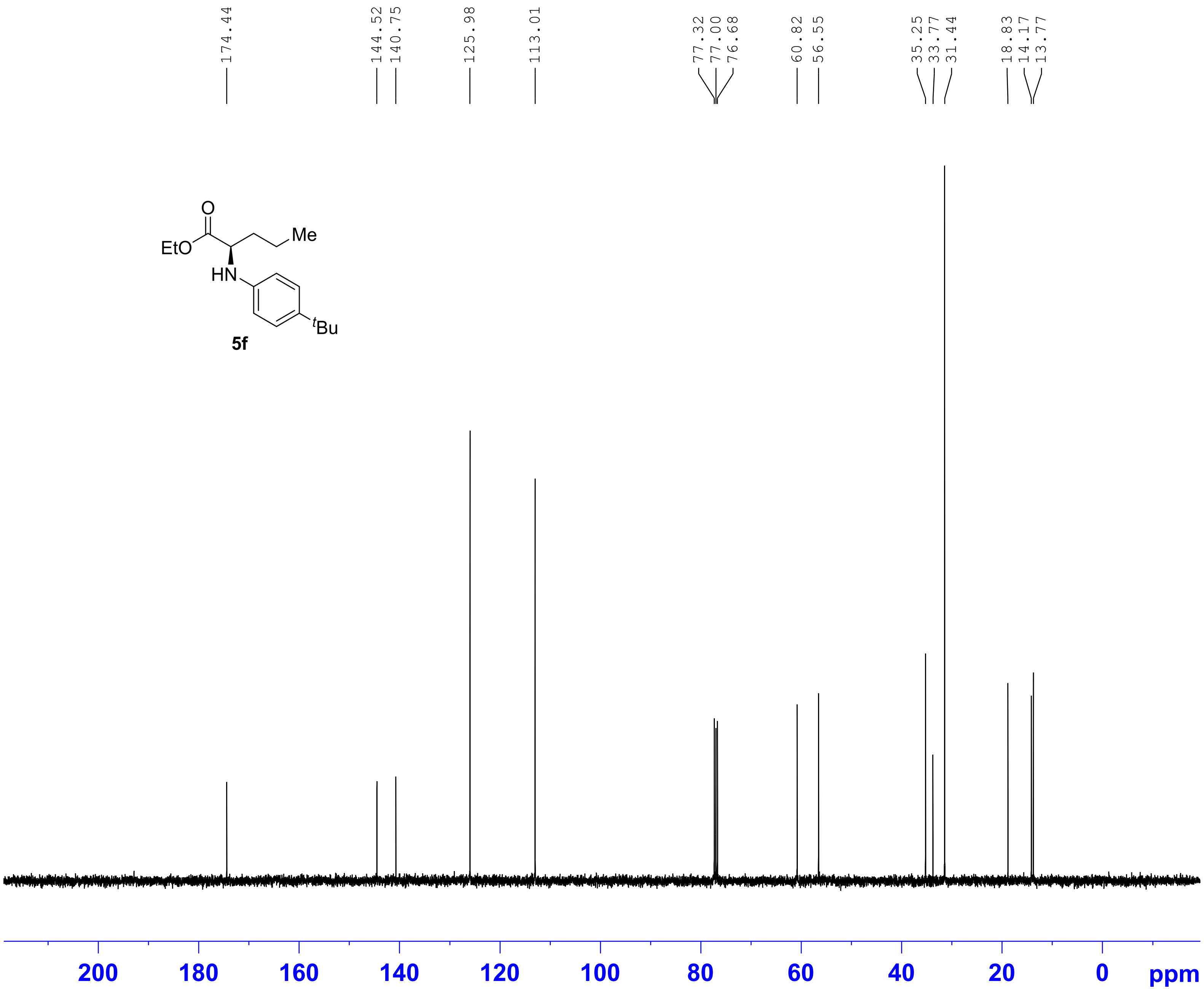

5f

NAME	lzy-08-189-A-C
EXPNO	2
PROCNO	1
Date_	20220228
Time_	20.38
INSTRUM	spect
PROBHD	5 mm DUL 13C-1
PULPROG	zgpg30
TD	65536
SOLVENT	CDCl3
NS	47
DS	0
SWH	24038.461 Hz
FIDRES	0.366798 Hz
AQ	1.3631988 sec
RG	2050
DW	20.800 usec
DE	6.00 usec
TE	293.8 K
D1	2.00000000 sec
D11	0.03000000 sec
TD0	1
======== CHANNEL f1 ========	
NUC1	13C
P1	40.00 usec
PL1	-3.00 dB
PL1W	60.64365387 W
SFO1	100.6228298 MHz
======== CHANNEL f2 ========	
CPDPRG2	waltz16
NUC2	1H
PCPD2	80.00 usec
PL2	-1.00 dB
PL12	14.39 dB
PL13	18.00 dB
PL2W	12.17476940 W
PL12W	0.35193357 W
PL13W	0.15327126 W
SFO2	400.1316005 MHz
SI	32768
SF	100.6127806 MHz
WDW	EM
SSB	0
LB	1.00 Hz
GB	0
PC	1.40

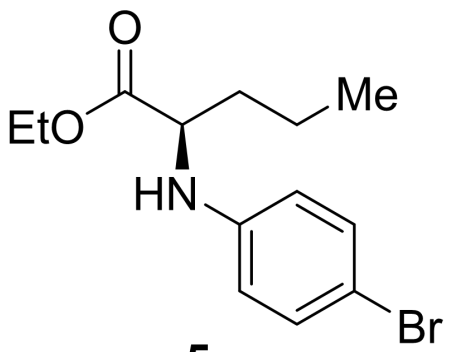

5g

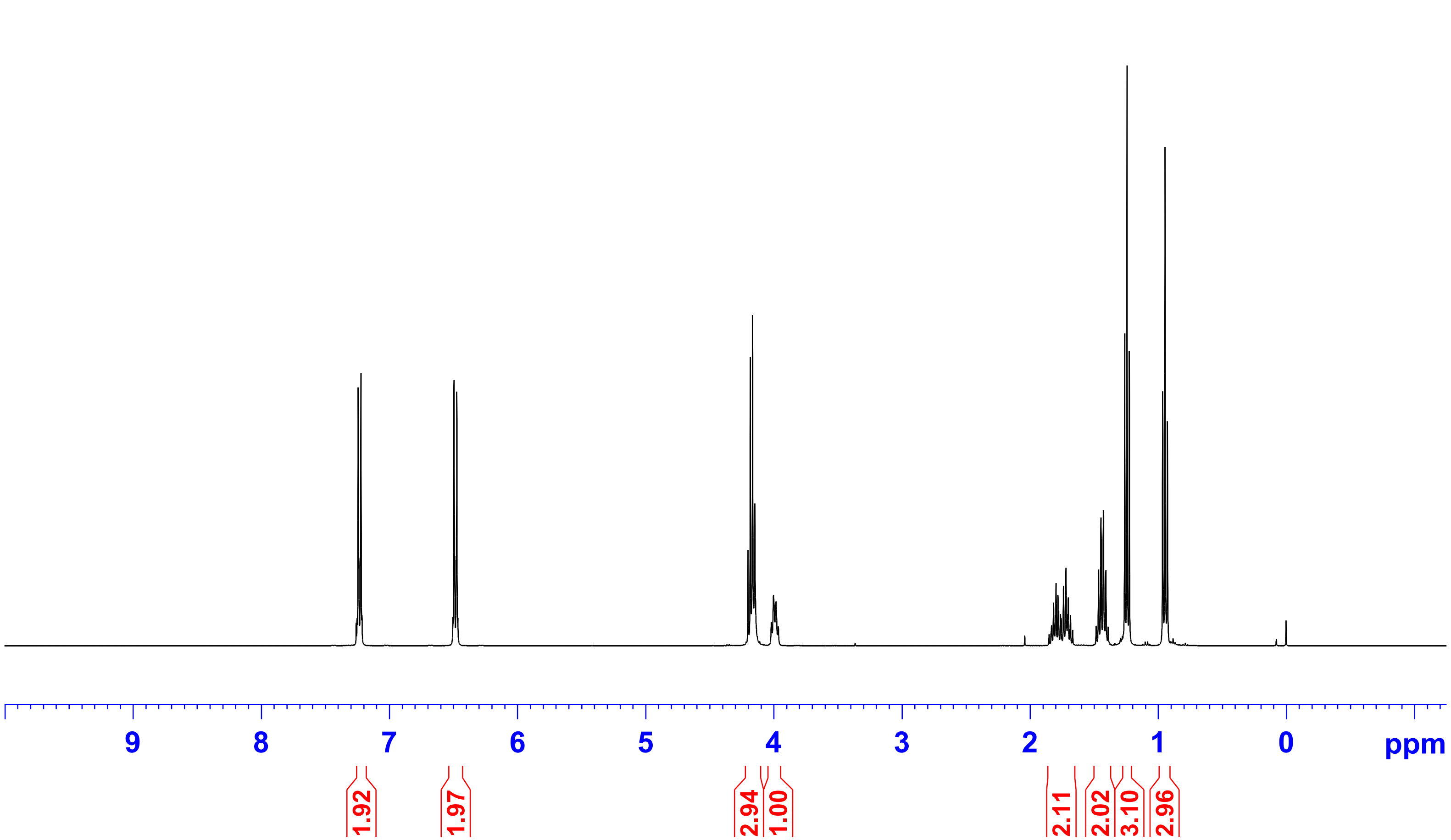

NAME	lzy-08-189-B-Br
EXPNO	1
PROCNO	1
Date_	20220227
Time	18.39
INSTRUM	spect
PROBHD	5 mm DUL 13C-1
PULPROG	zg30
TD	65536
SOLVENT	CDCl3
NS	4
DS	0
SWH	8223.685 Hz
FIDRES	0.125483 Hz
AQ	3.9846387 sec
RG	114
DW	60.800 usec
DE	6.00 usec
TE	294.5 K
D1	1.00000000 sec
TD0	1

======== CHANNEL f1 ========

NUC1	1H
P1	15.80 usec
PL1	-1.00 dB
PL1W	12.17476940 W
SFO1	400.1324710 MHz
SI	32768
SF	400.1300096 MHz
WDW	EM
SSB	0
LB	0.30 Hz
GB	0
PC	1.00

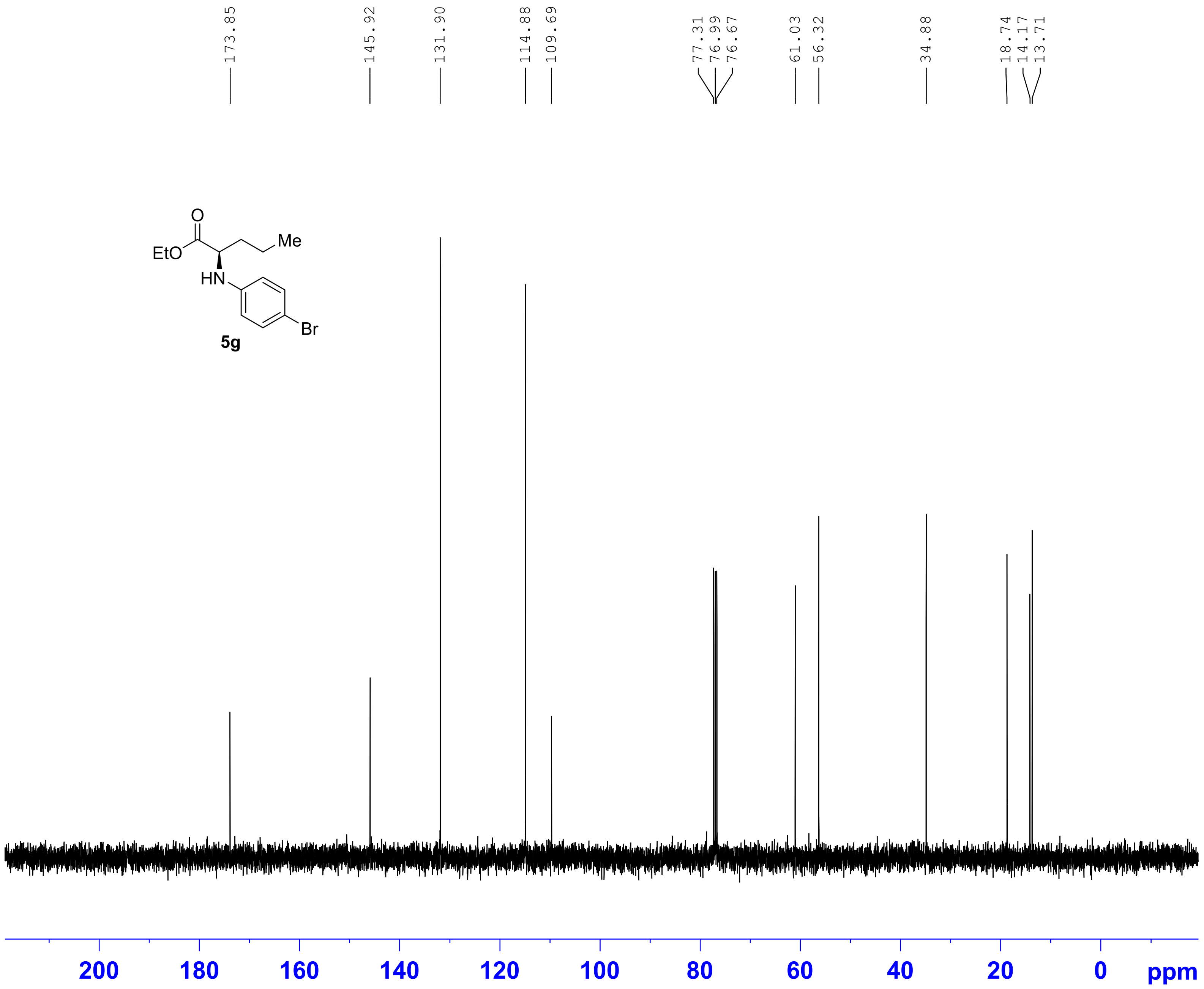

NAME	lzy-08-189-B-Br-C	
EXPNO	1	
PROCNO	1	
Date_	20220227	
Time	18.41	
INSTRUM	spect	
PROBHD	5 mm DUL 13C-1	
PULPROG	zgpg30	
TD	65536	
SOLVENT	CDCl3	
NS	22	
DS	0	
SWH	24038.461	Hz
FIDRES	0.366798	Hz
AQ	1.3631988	sec
RG	2050	
DW	20.800	usec
DE	6.00	usec
TE	294.8	K
D1	2.00000000	sec
D11	0.03000000	sec
TD0	1	
========	CHANNEL f1	========
NUC1	13C	
P1	40.00	usec
PL1	-3.00	dB
PL1W	60.64365387	W
SFO1	100.6228298	MHz
========	CHANNEL f2	========
CPDPRG2	waltz16	
NUC2	1H	
PCPD2	80.00	usec
PL2	-1.00	dB
PL12	14.39	dB
PL13	18.00	dB
PL2W	12.17476940	W
PL12W	0.35193357	W
PL13W	0.15327126	W
SFO2	400.1316005	MHz
SI	32768	
SF	100.6127794	MHz
WDW	EM	
SSB	0	
LB	1.00	Hz
GB	0	
PC	1.40	

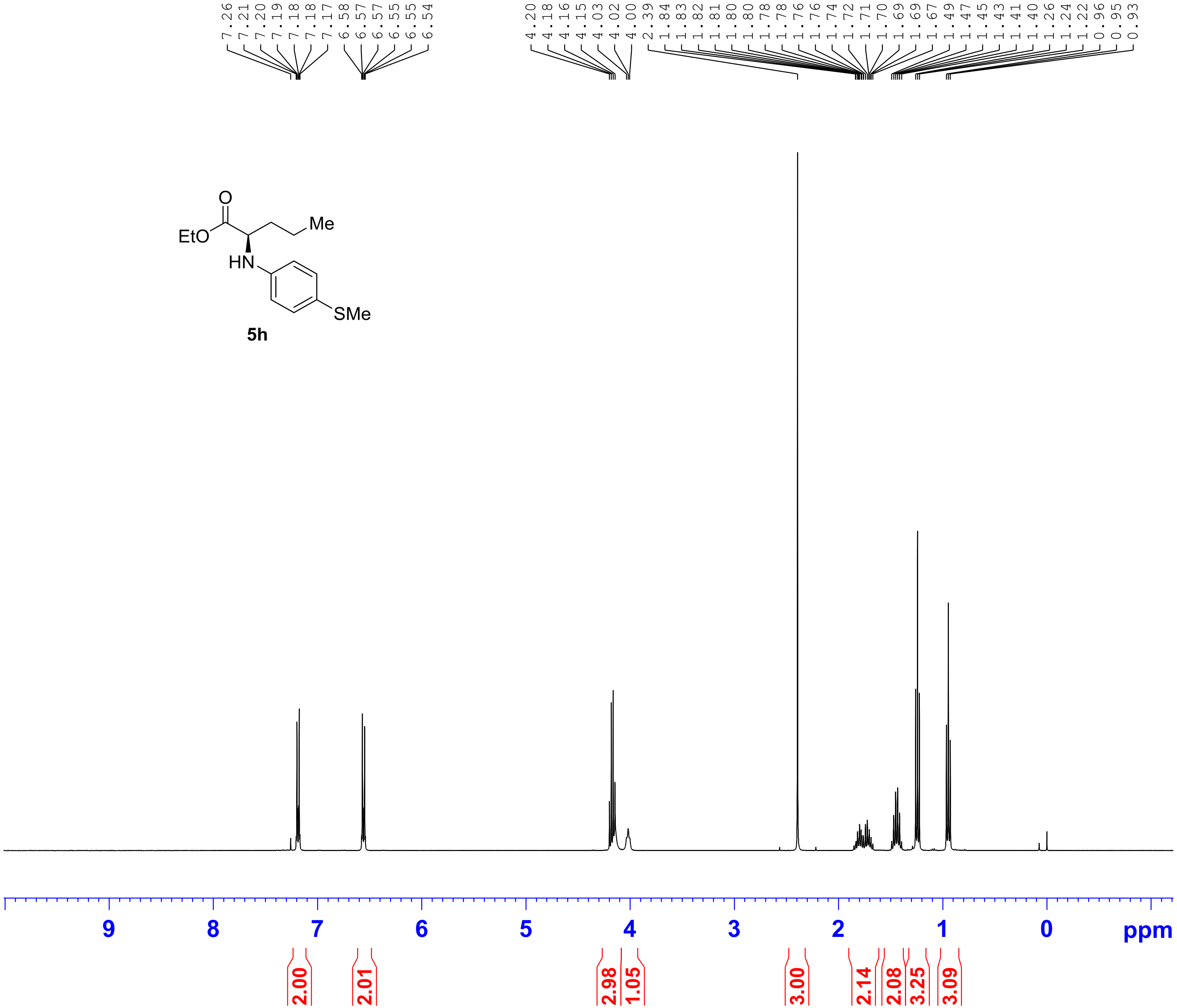

NAME	lzy-09-08-B
EXPNO	1
PROCNO	1
Date_	20220204
Time	10.15
INSTRUM	spect
PROBHD	5 mm DUL 13C-1
PULPROG	zg30
TD	65536
SOLVENT	CDCl3
NS	4
DS	0
SWH	8223.685 Hz
FIDRES	0.125483 Hz
AQ	3.9846387 sec
RG	114
DW	60.800 usec
DE	6.00 usec
TE	294.1 K
D1	1.00000000 sec
TD0	1

======== CHANNEL f1 ========

NUC1	1H
P1	15.80 usec
PL1	-1.00 dB
PL1W	12.17476940 W
SFO1	400.1324710 MHz
SI	32768
SF	400.1300102 MHz
WDW	EM
SSB	0
LB	0.30 Hz
GB	0
PC	1.00

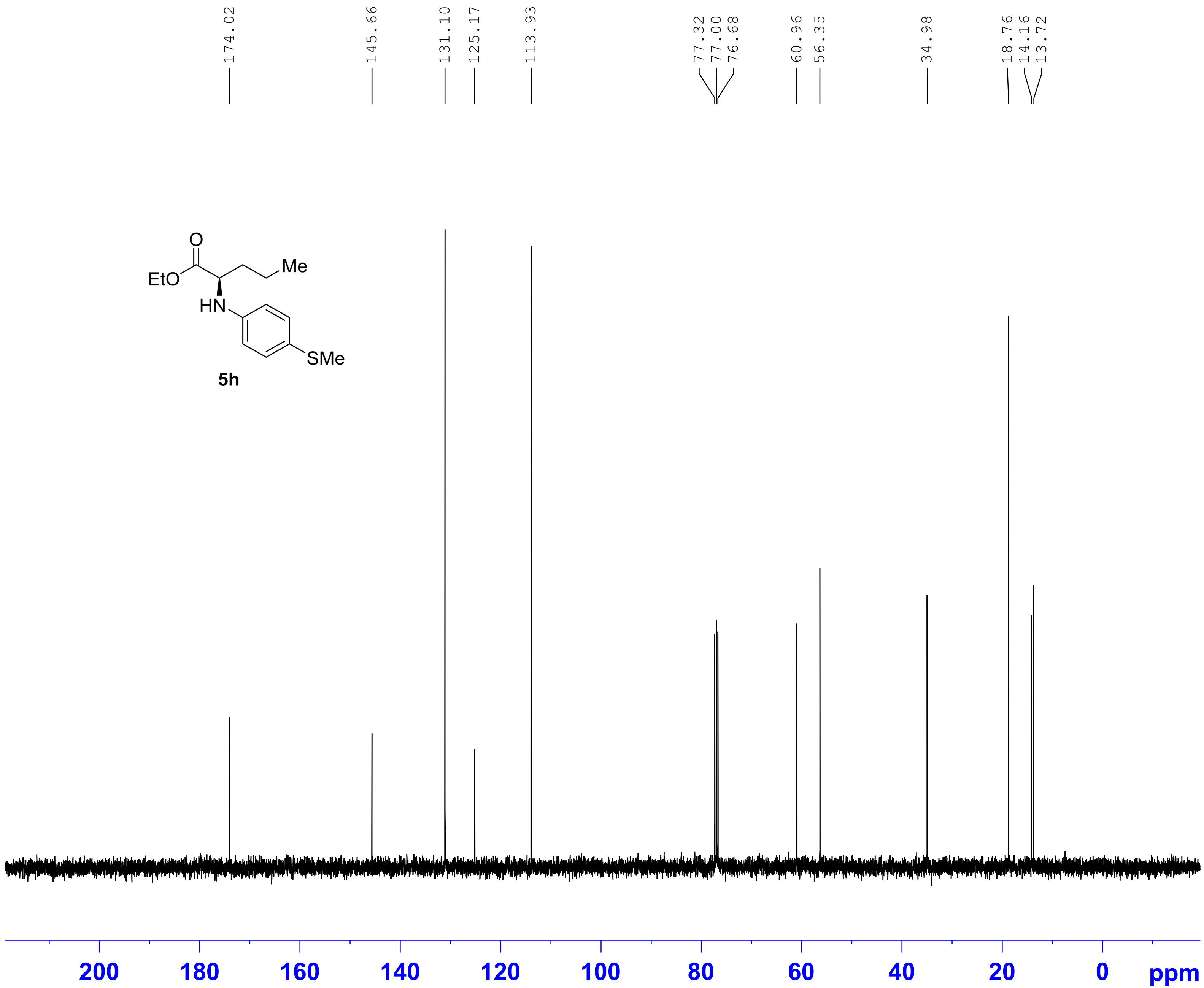

NAME	lzy-09-08-B-C
EXPNO	1
PROCNO	1
Date_	20220204
Time	10.19
INSTRUM	spect
PROBHD	5 mm DUL 13C-1
PULPROG	zgpg30
TD	65536
SOLVENT	CDCl3
NS	48
DS	1
SWH	24038.461 Hz
FIDRES	0.366798 Hz
AQ	1.3631988 sec
RG	2050
DW	20.800 usec
DE	6.00 usec
TE	294.4 K
D1	2.00000000 sec
D11	0.03000000 sec
TD0	1
======== CHANNEL f1 ========	
NUC1	13C
P1	40.00 usec
PL1	-3.00 dB
PL1W	60.64365387 W
SFO1	100.6228298 MHz
======== CHANNEL f2 ========	
CPDPRG2	waltz16
NUC2	1H
PCPD2	80.00 usec
PL2	-1.00 dB
PL12	14.39 dB
PL13	18.00 dB
PL2W	12.17476940 W
PL12W	0.35193357 W
PL13W	0.15327126 W
SFO2	400.1316005 MHz
SI	32768
SF	100.6127796 MHz
WDW	EM
SSB	0
LB	1.00 Hz
GB	0
PC	1.40

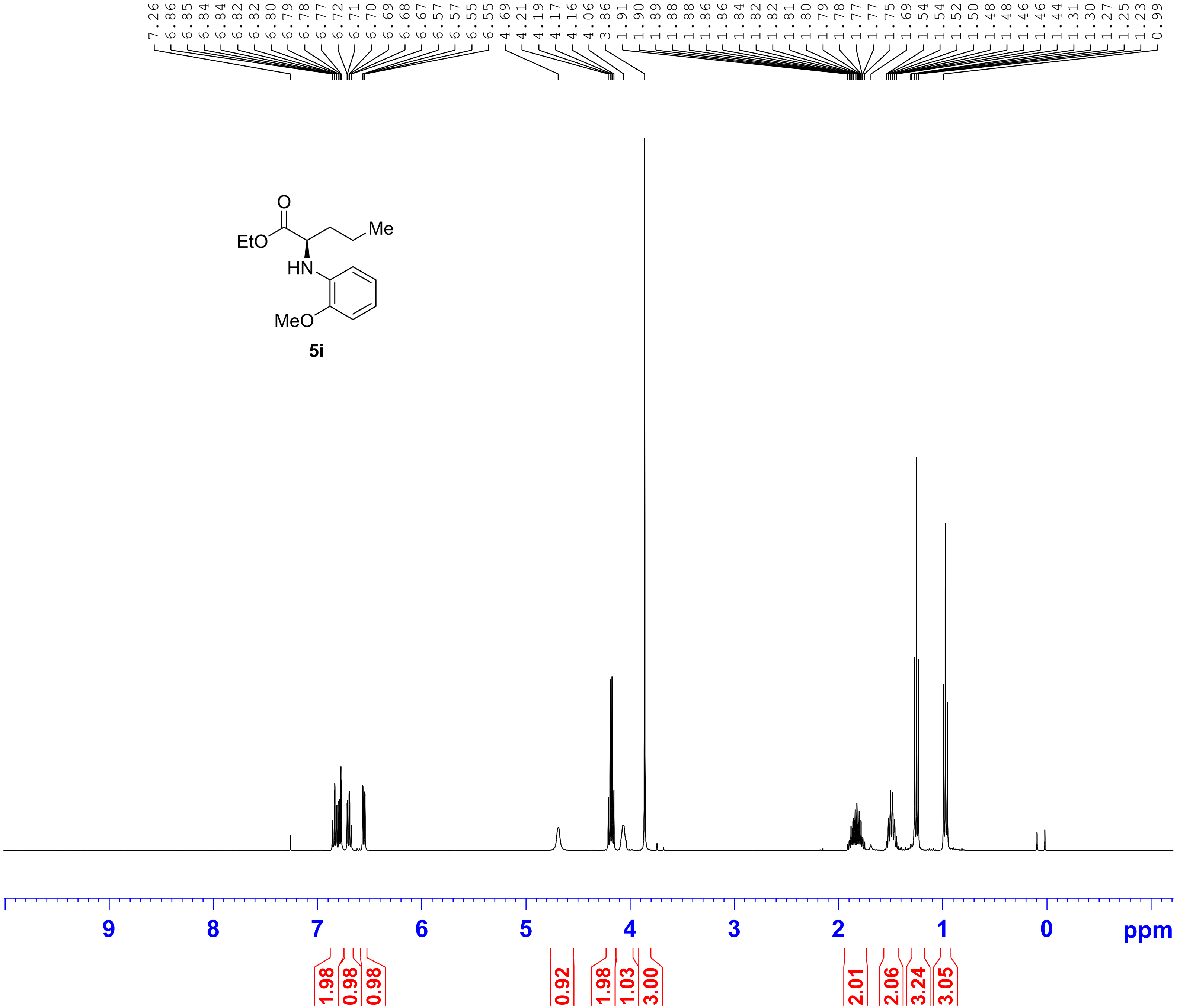

NAME	lzy-09-09-A	
EXPNO	2	
PROCNO	1	
Date_	20220227	
Time	16.57	
INSTRUM	spect	
PROBHD	5 mm DUL 13C-1	
PULPROG	zg30	
TD	65536	
SOLVENT	CDCl3	
NS	3	
DS	0	
SWH	8223.685	Hz
FIDRES	0.125483	Hz
AQ	3.9846387	sec
RG	101	
DW	60.800	usec
DE	6.00	usec
TE	293.7	K
D1	1.00000000	sec
TD0	1	

======== CHANNEL f1 ========

NUC1	1H	
P1	15.80	usec
PL1	-1.00	dB
PL1W	12.17476940	W
SFO1	400.1324710	MHz
SI	32768	
SF	400.1300096	MHz
WDW	EM	
SSB	0	
LB	0.30	Hz
GB	0	
PC	1.00	

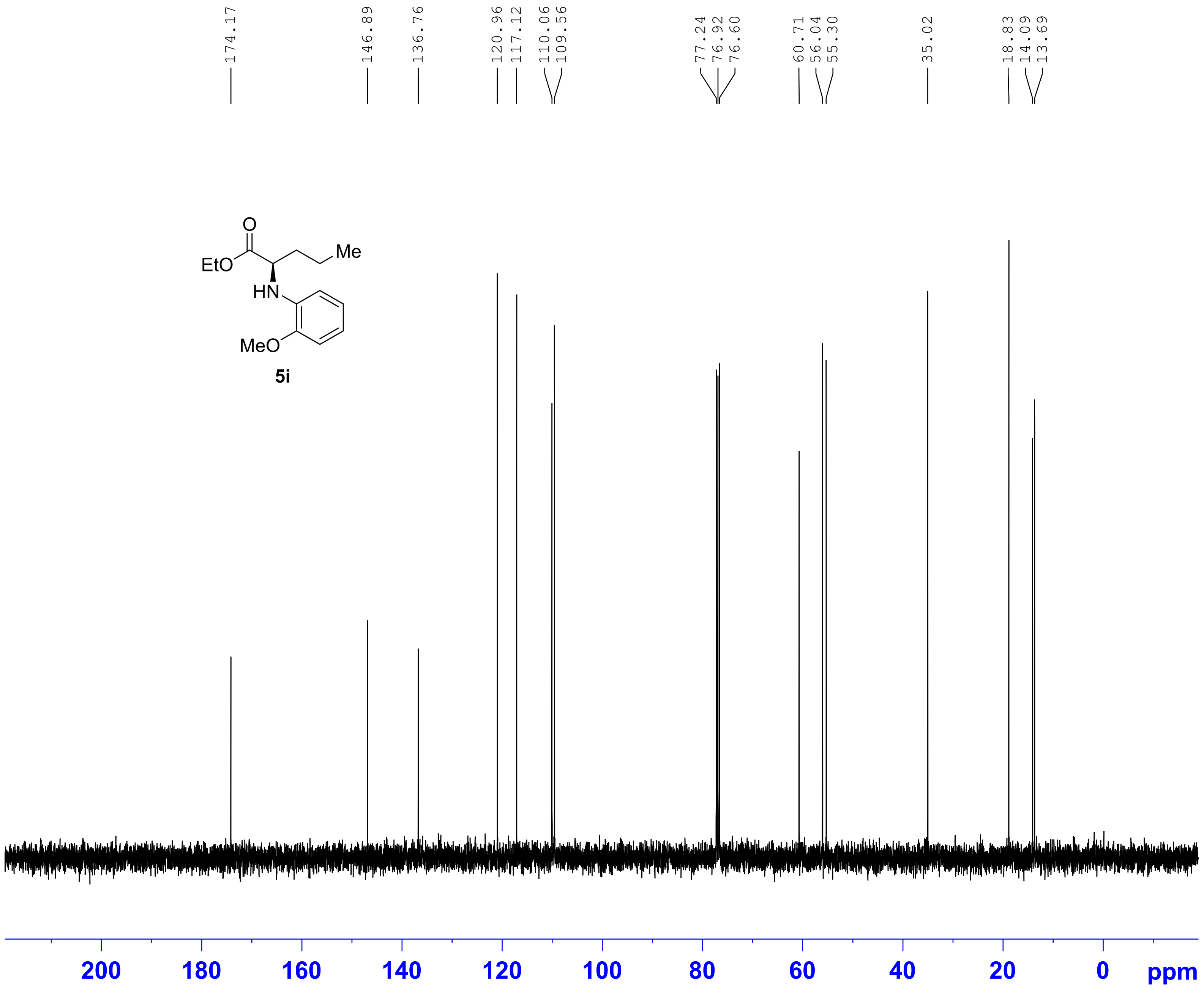

5i

```
NAME        lzy-09-09-A-C
EXPNO                   2
PROCNO                  1
Date_            20220227
Time                17.02
INSTRUM             spect
PROBHD     5 mm DUL 13C-1
PULPROG            zgpg30
TD                  65536
SOLVENT             CDCl3
NS                     68
DS                      0
SWH             24038.461 Hz
FIDRES           0.366798 Hz
AQ              1.3631988 sec
RG                   2050
DW                 20.800 usec
DE                   6.00 usec
TE                  294.1 K
D1             2.00000000 sec
D11            0.03000000 sec
TD0                     1

======== CHANNEL f1 ========
NUC1                  13C
P1                  40.00 usec
PL1                 -3.00 dB
PL1W          60.64365387 W
SFO1          100.6228298 MHz

======== CHANNEL f2 ========
CPDPRG2           waltz16
NUC2                   1H
PCPD2               80.00 usec
PL2                 -1.00 dB
PL12                14.39 dB
PL13                18.00 dB
PL2W          12.17476940 W
PL12W          0.35193357 W
PL13W          0.15327126 W
SFO2          400.1316005 MHz
SI                  32768
SF            100.6127868 MHz
WDW                    EM
SSB                     0
LB                   1.00 Hz
GB                      0
PC                   1.40
```

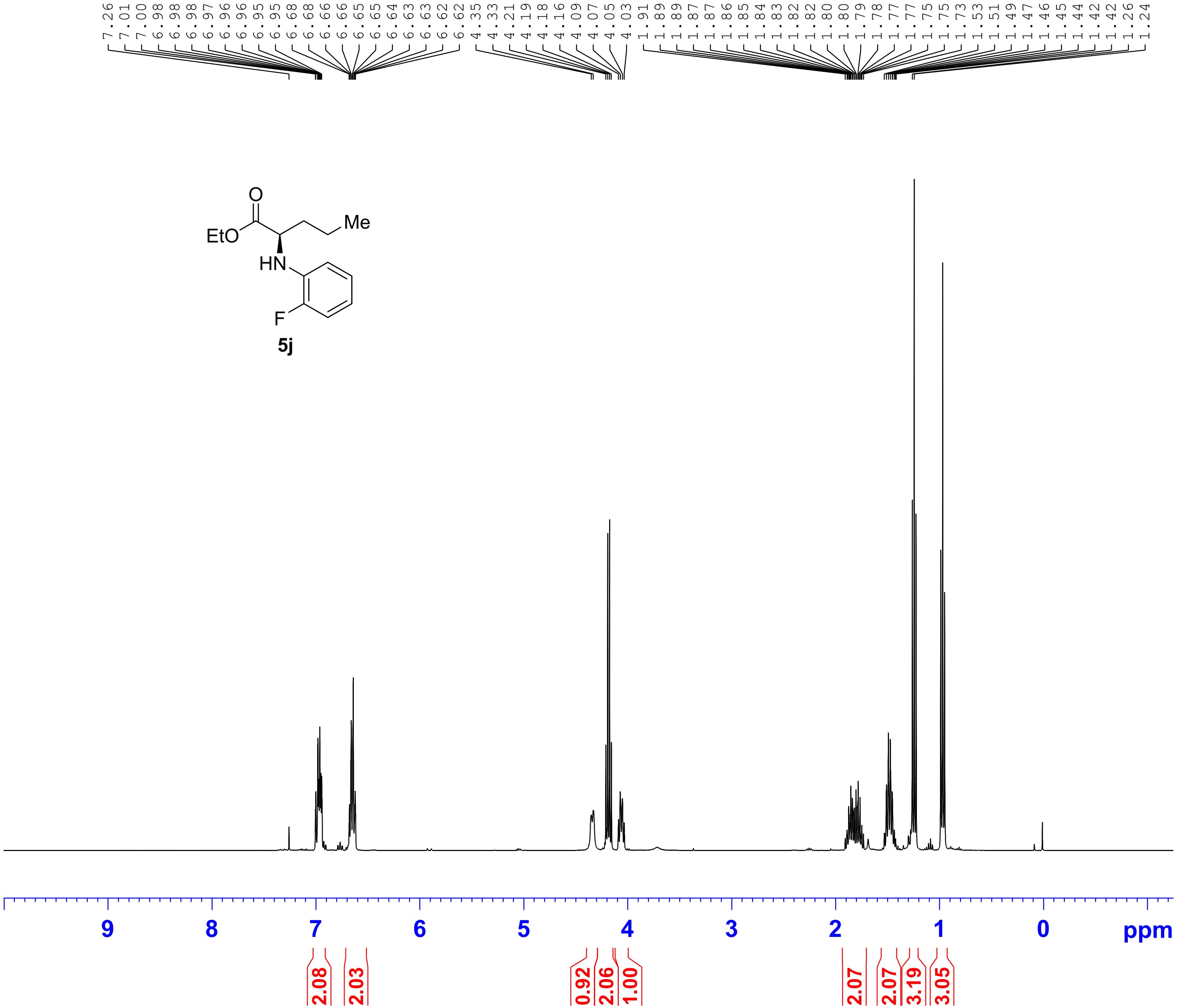

5j

NAME	lzy-08-189-C-F
EXPNO	1
PROCNO	1
Date_	20220227
Time	18.43
INSTRUM	spect
PROBHD	5 mm DUL 13C-1
PULPROG	zg30
TD	65536
SOLVENT	CDCl3
NS	3
DS	0
SWH	8223.685 Hz
FIDRES	0.125483 Hz
AQ	3.9846387 sec
RG	114
DW	60.800 usec
DE	6.00 usec
TE	294.6 K
D1	1.00000000 sec
TD0	1

======== CHANNEL f1 ========

NUC1	1H
P1	15.80 usec
PL1	-1.00 dB
PL1W	12.17476940 W
SFO1	400.1324710 MHz
SI	32768
SF	400.1300098 MHz
WDW	EM
SSB	0
LB	0.30 Hz
GB	0
PC	1.00

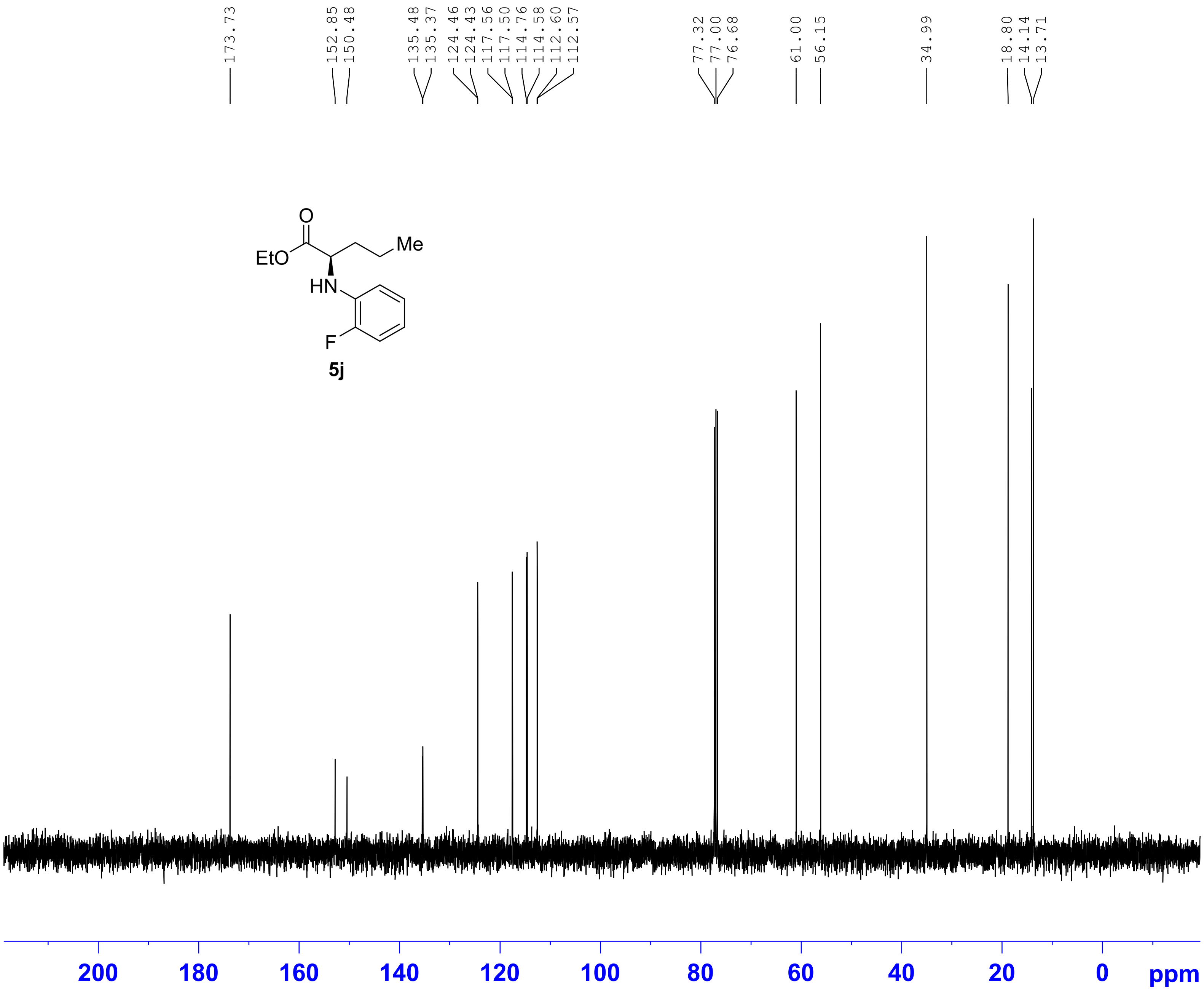

NAME	lzy-08-189-C-F-C	
EXPNO	1	
PROCNO	1	
Date_	20220227	
Time	18.47	
INSTRUM	spect	
PROBHD	5 mm DUL 13C-1	
PULPROG	zgpg30	
TD	65536	
SOLVENT	CDCl3	
NS	64	
DS	0	
SWH	24038.461	Hz
FIDRES	0.366798	Hz
AQ	1.3631988	sec
RG	2050	
DW	20.800	usec
DE	6.00	usec
TE	294.9	K
D1	2.00000000	sec
D11	0.03000000	sec
TD0	1	
======== CHANNEL f1 ========		
NUC1	13C	
P1	40.00	usec
PL1	-3.00	dB
PL1W	60.64365387	W
SFO1	100.6228298	MHz
======== CHANNEL f2 ========		
CPDPRG2	waltz16	
NUC2	1H	
PCPD2	80.00	usec
PL2	-1.00	dB
PL12	14.39	dB
PL13	18.00	dB
PL2W	12.17476940	W
PL12W	0.35193357	W
PL13W	0.15327126	W
SFO2	400.1316005	MHz
SI	32768	
SF	100.6127765	MHz
WDW	EM	
SSB	0	
LB	1.00	Hz
GB	0	
PC	1.40	

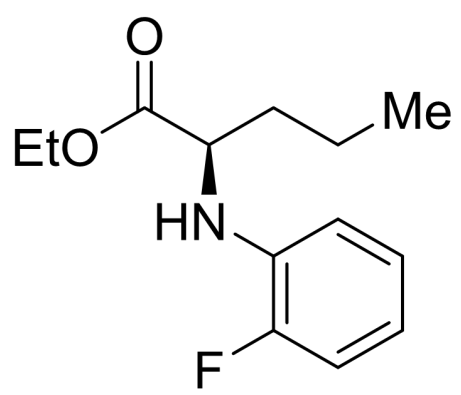

5j

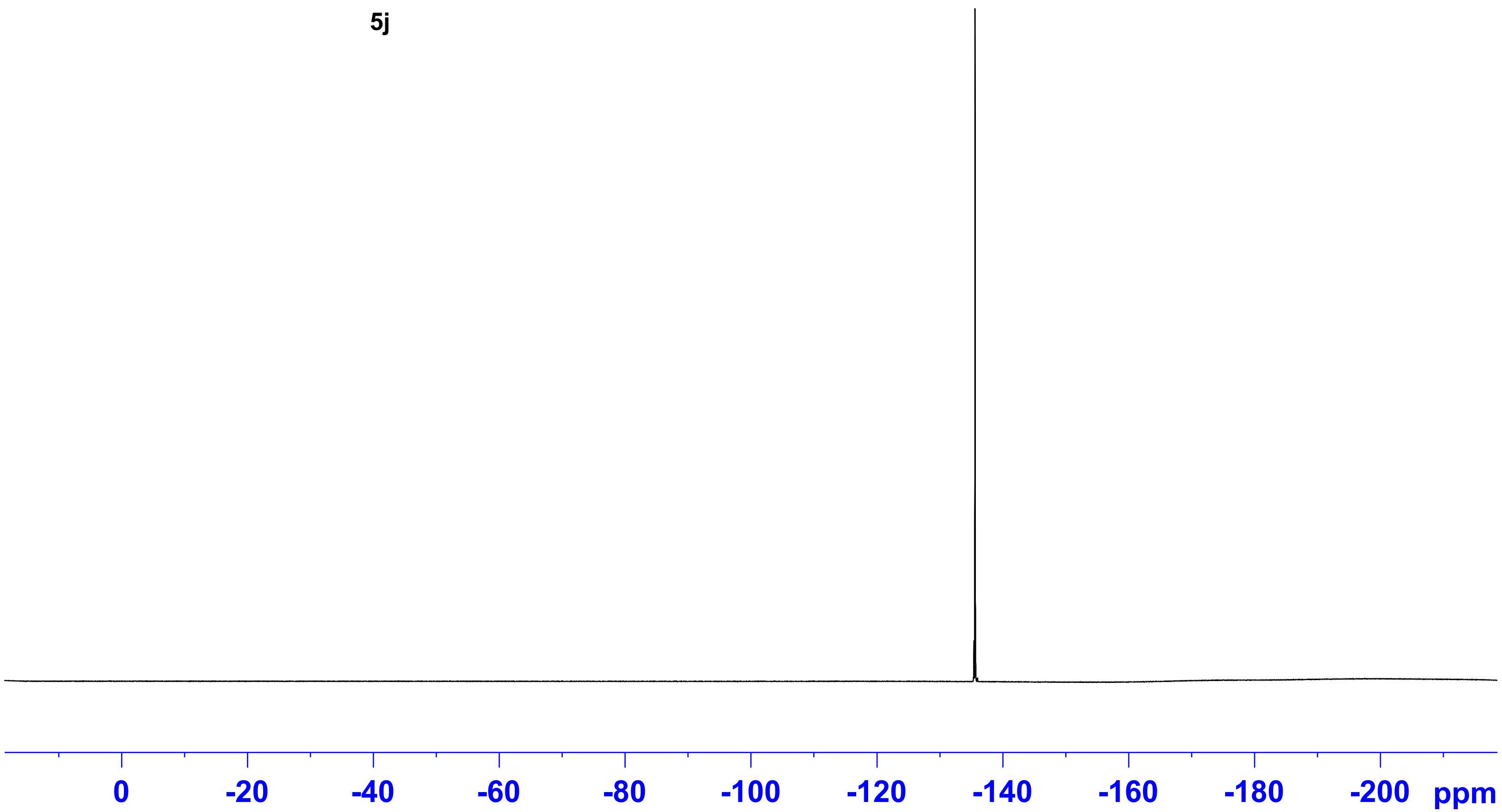

NAME	lzy-08-189-C-F-F
EXPNO	5
PROCNO	1
Date_	20220227
Time	19.09
INSTRUM	spect
PROBHD	5 mm PABBO BB/
PULPROG	zgflqn
TD	131072
SOLVENT	CDCl3
NS	16
DS	0
SWH	89285.711 Hz
FIDRES	0.681196 Hz
AQ	0.7340532 sec
RG	196.92
DW	5.600 usec
DE	6.50 usec
TE	294.7 K
D1	1.00000000 sec
TD0	1

======== CHANNEL f1 ========

SFO1	376.4607164 MHz
NUC1	19F
P1	14.70 usec
SI	65536
SF	376.4983660 MHz
WDW	EM
SSB	0
LB	0.30 Hz
GB	0
PC	1.00

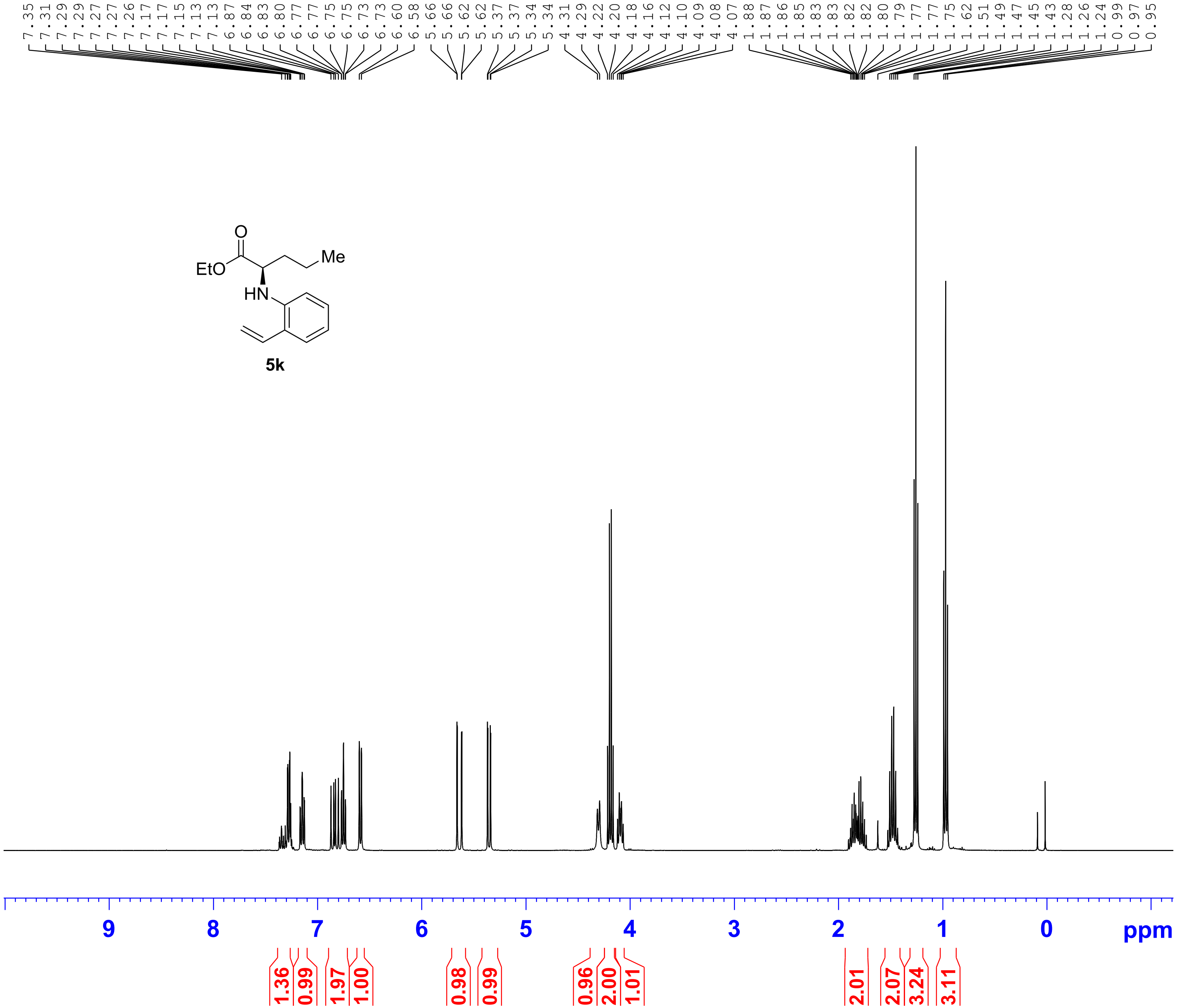

NAME	lzy-09-09-C
EXPNO	1
PROCNO	1
Date_	20220204
Time	10.22
INSTRUM	spect
PROBHD	5 mm DUL 13C-1
PULPROG	zg30
TD	65536
SOLVENT	CDCl3
NS	4
DS	0
SWH	8223.685 Hz
FIDRES	0.125483 Hz
AQ	3.9846387 sec
RG	144
DW	60.800 usec
DE	6.00 usec
TE	294.1 K
D1	1.00000000 sec
TD0	1

======== CHANNEL f1 ========

NUC1	1H
P1	15.80 usec
PL1	-1.00 dB
PL1W	12.17476940 W
SFO1	400.1324710 MHz
SI	32768
SF	400.1300103 MHz
WDW	EM
SSB	0
LB	0.30 Hz
GB	0
PC	1.00

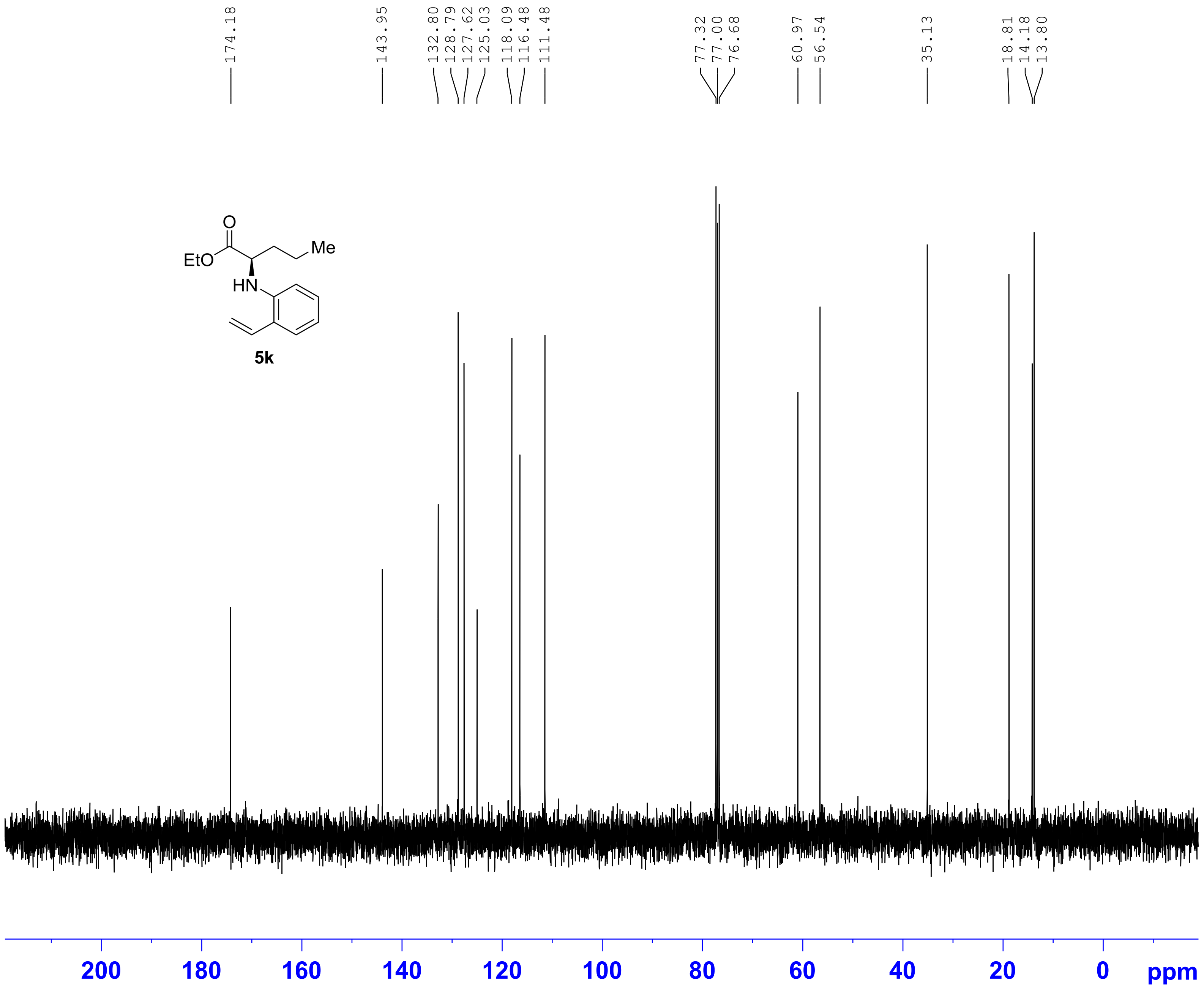

NAME	lzy-09-09-C-C	
EXPNO	2	
PROCNO	1	
Date_	20220204	
Time	21.38	
INSTRUM	spect	
PROBHD	5 mm DUL 13C-1	
PULPROG	zgpg30	
TD	65536	
SOLVENT	CDCl3	
NS	53	
DS	0	
SWH	24038.461	Hz
FIDRES	0.366798	Hz
AQ	1.3631988	sec
RG	2050	
DW	20.800	usec
DE	6.00	usec
TE	294.0	K
D1	2.00000000	sec
D11	0.03000000	sec
TD0	1	

======== CHANNEL f1 ========

NUC1	13C	
P1	40.00	usec
PL1	-3.00	dB
PL1W	60.64365387	W
SFO1	100.6228298	MHz

======== CHANNEL f2 ========

CPDPRG2	waltz16	
NUC2	1H	
PCPD2	80.00	usec
PL2	-1.00	dB
PL12	14.39	dB
PL13	18.00	dB
PL2W	12.17476940	W
PL12W	0.35193357	W
PL13W	0.15327126	W
SFO2	400.1316005	MHz
SI	32768	
SF	100.6127769	MHz
WDW	EM	
SSB	0	
LB	1.00	Hz
GB	0	
PC	1.40	

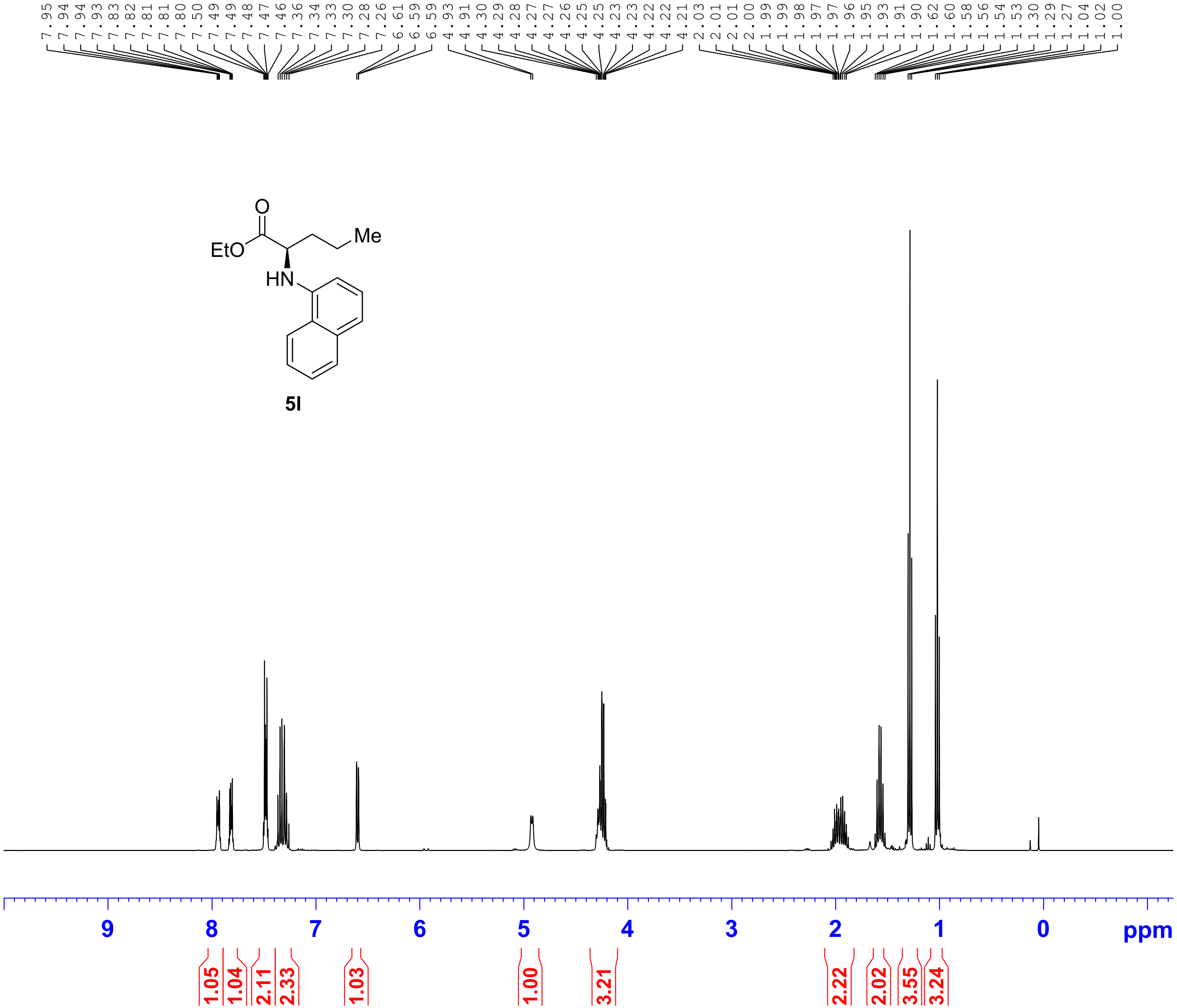

NAME	lzy-08-189-D	
EXPNO	1	
PROCNO	1	
Date_	20220215	
Time	22.11	
INSTRUM	spect	
PROBHD	5 mm DUL 13C-1	
PULPROG	zg30	
TD	65536	
SOLVENT	CDCl3	
NS	4	
DS	0	
SWH	8223.685	Hz
FIDRES	0.125483	Hz
AQ	3.9846387	sec
RG	114	
DW	60.800	usec
DE	6.00	usec
TE	294.3	K
D1	1.00000000	sec
TD0	1	

======== CHANNEL f1 ========

NUC1	1H	
P1	15.80	usec
PL1	-1.00	dB
PL1W	12.17476940	W
SFO1	400.1324710	MHz
SI	32768	
SF	400.1300098	MHz
WDW	EM	
SSB	0	
LB	0.30	Hz
GB	0	
PC	1.00	

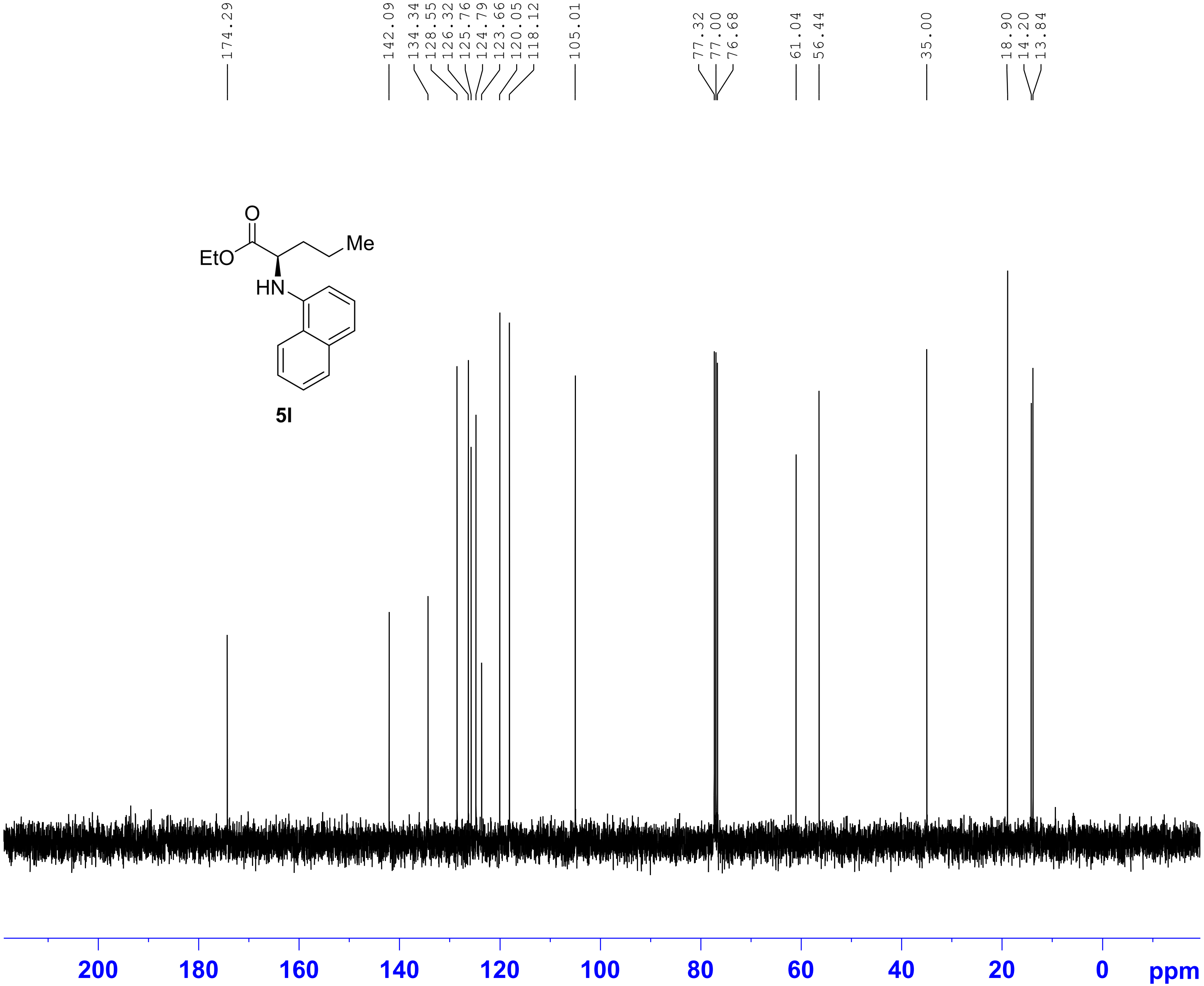

5l

NAME	lzy-08-189-D-C-2
EXPNO	1
PROCNO	1
Date_	20220215
Time	21.54
INSTRUM	spect
PROBHD	5 mm DUL 13C-1
PULPROG	zgpg30
TD	65536
SOLVENT	CDCl3
NS	37
DS	0
SWH	24038.461 Hz
FIDRES	0.366798 Hz
AQ	1.3631988 sec
RG	2050
DW	20.800 usec
DE	6.00 usec
TE	294.4 K
D1	2.00000000 sec
D11	0.03000000 sec
TD0	1
======== CHANNEL f1 ========	
NUC1	13C
P1	40.00 usec
PL1	-3.00 dB
PL1W	60.64365387 W
SFO1	100.6228298 MHz
======== CHANNEL f2 ========	
CPDPRG2	waltz16
NUC2	1H
PCPD2	80.00 usec
PL2	-1.00 dB
PL12	14.39 dB
PL13	18.00 dB
PL2W	12.17476940 W
PL12W	0.35193357 W
PL13W	0.15327126 W
SFO2	400.1316005 MHz
SI	32768
SF	100.6127798 MHz
WDW	EM
SSB	0
LB	1.00 Hz
GB	0
PC	1.40

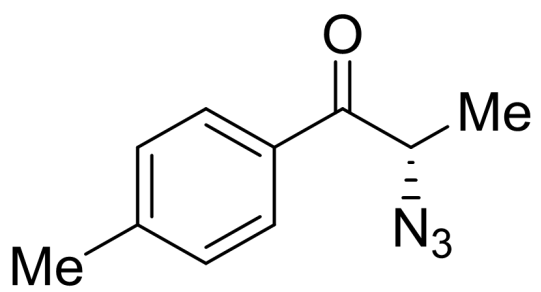

7a

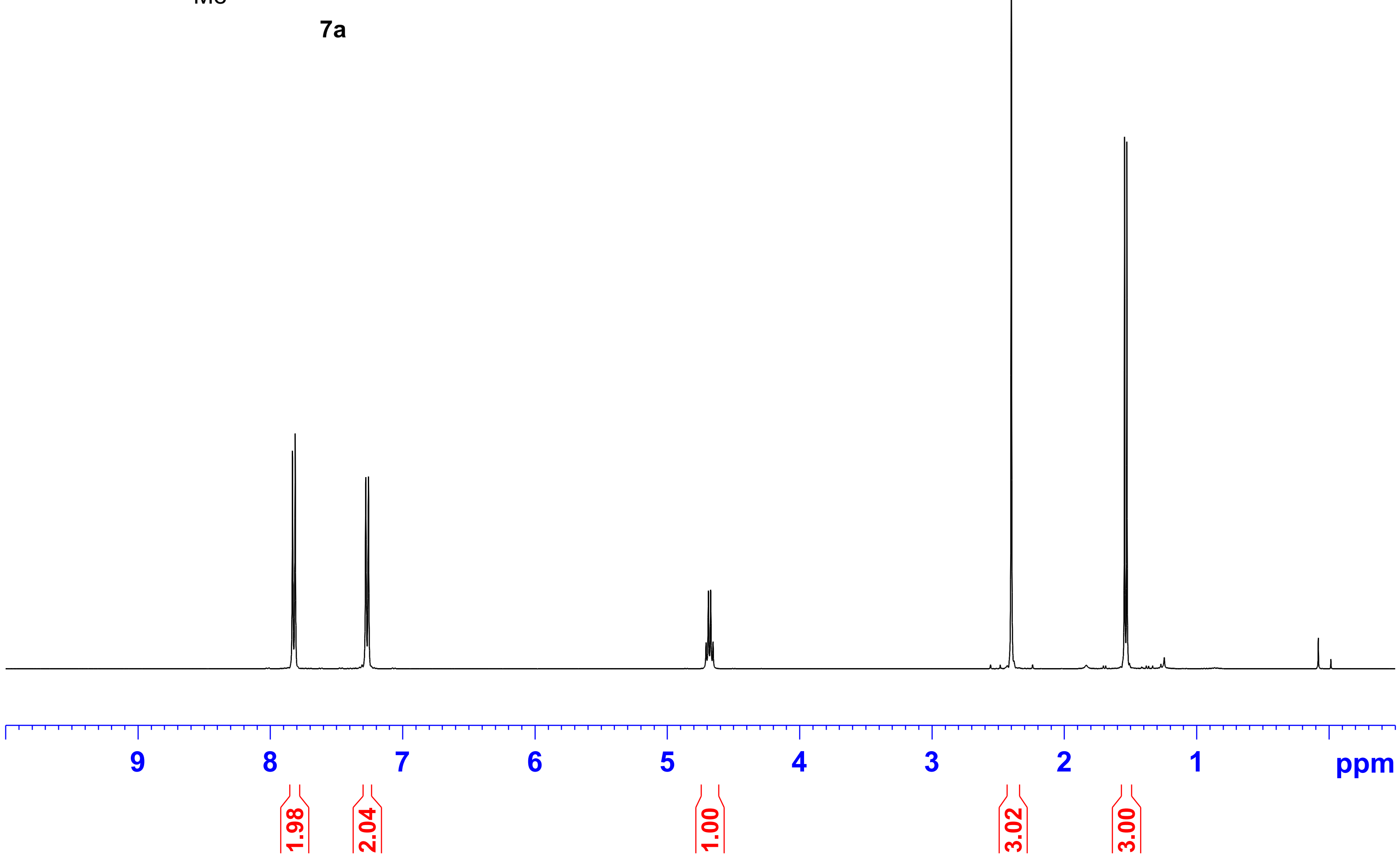

NAME	lzy-11-42-AG
EXPNO	1
PROCNO	1
Date_	20220716
Time_	18.48
INSTRUM	spect
PROBHD	5 mm DUL 13C-1
PULPROG	zg30
TD	65536
SOLVENT	CDCl3
NS	1
DS	0
SWH	8223.685 Hz
FIDRES	0.125483 Hz
AQ	3.9846387 sec
RG	64
DW	60.800 usec
DE	6.00 usec
TE	294.1 K
D1	1.00000000 sec
TD0	1

======== CHANNEL f1 ========

NUC1	1H
P1	15.80 usec
PL1	-1.00 dB
PL1W	12.17476940 W
SFO1	400.1324710 MHz
SI	32768
SF	400.1300097 MHz
WDW	EM
SSB	0
LB	0.30 Hz
GB	0
PC	1.00

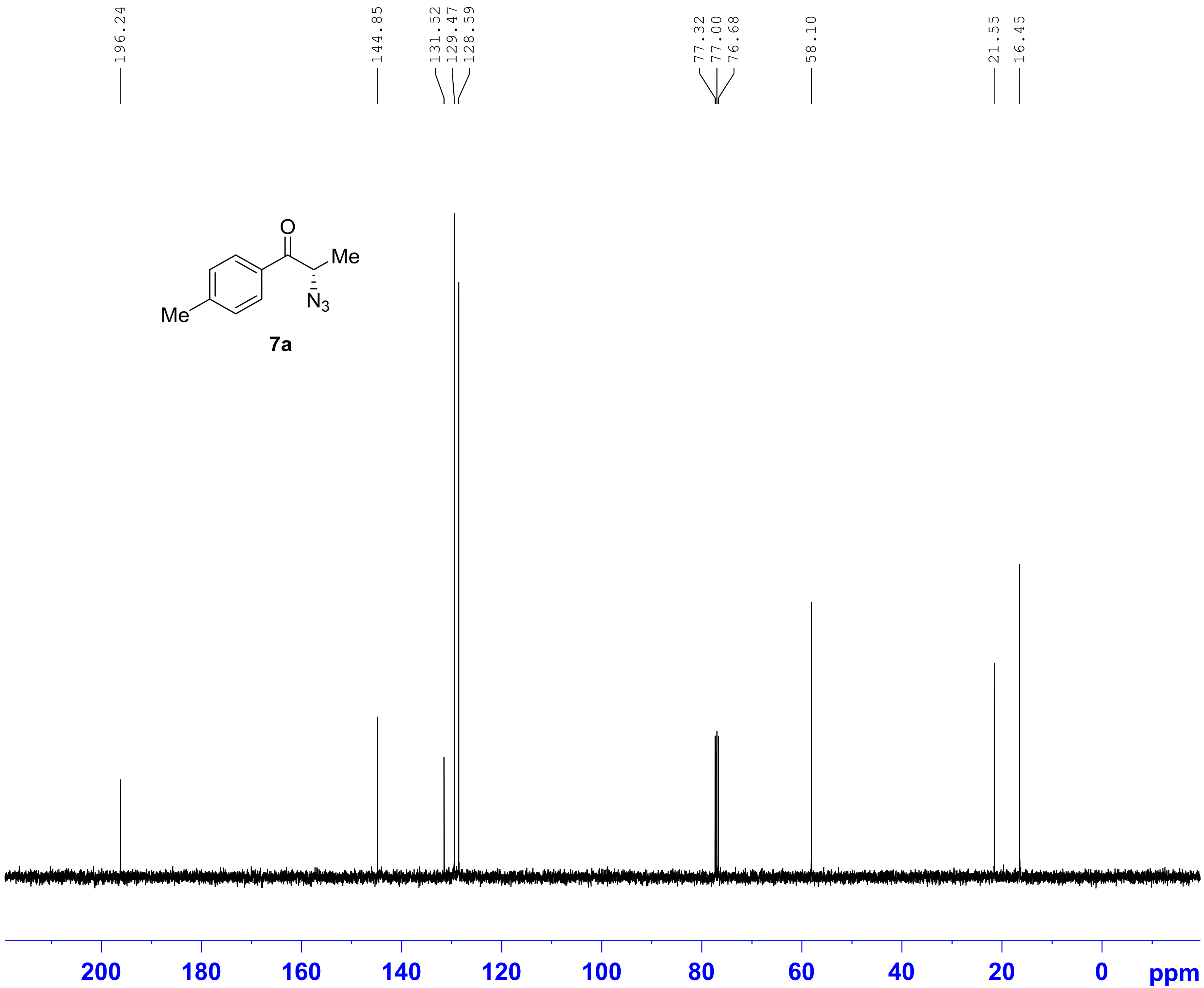

NAME	lzy-11-42-AG-C	
EXPNO	1	
PROCNO	1	
Date_	20220716	
Time	18.50	
INSTRUM	spect	
PROBHD	5 mm DUL 13C-1	
PULPROG	zgpg30	
TD	65536	
SOLVENT	CDCl3	
NS	34	
DS	0	
SWH	24038.461	Hz
FIDRES	0.366798	Hz
AQ	1.3631988	sec
RG	2050	
DW	20.800	usec
DE	6.00	usec
TE	294.3	K
D1	2.00000000	sec
D11	0.03000000	sec
TD0	1	

======== CHANNEL f1 ========

NUC1	13C	
P1	40.00	usec
PL1	-3.00	dB
PL1W	60.64365387	W
SFO1	100.6228298	MHz

======== CHANNEL f2 ========

CPDPRG2	waltz16	
NUC2	1H	
PCPD2	80.00	usec
PL2	-1.00	dB
PL12	14.39	dB
PL13	18.00	dB
PL2W	12.17476940	W
PL12W	0.35193357	W
PL13W	0.15327126	W
SFO2	400.1316005	MHz
SI	32768	
SF	100.6127854	MHz
WDW	EM	
SSB	0	
LB	1.00	Hz
GB	0	
PC	1.40	

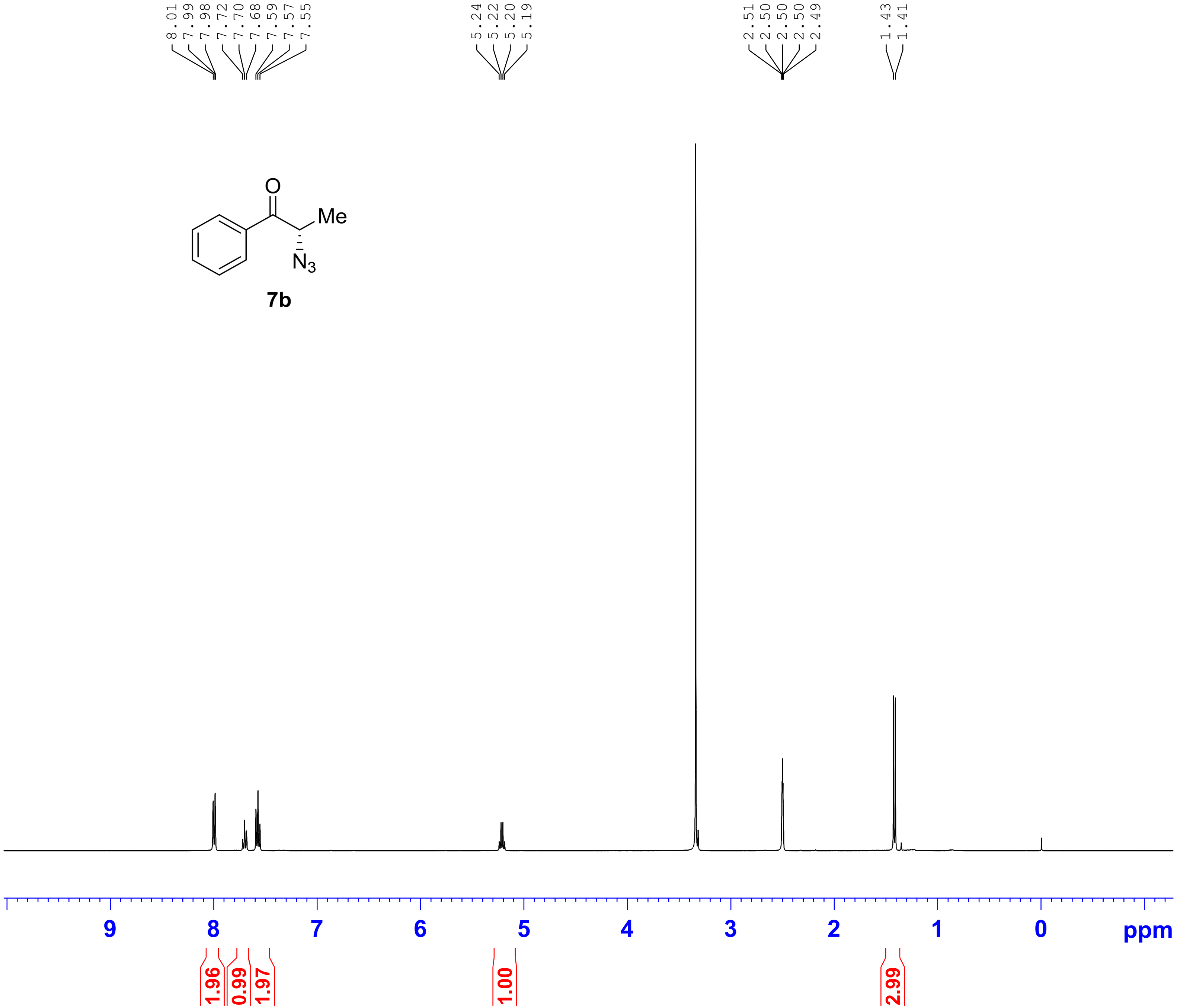

NAME	lzy-11-42-Ph-N3-DMSO
EXPNO	1
PROCNO	1
Date_	20230417
Time	9.39 h
INSTRUM	AvanceNeo 400MHz
PROBHD	Z163739_0629 (
PULPROG	zg30
TD	65536
SOLVENT	DMSO
NS	16
DS	2
SWH	8196.722 Hz
FIDRES	0.250144 Hz
AQ	3.9977460 sec
RG	101
DW	61.000 usec
DE	13.89 usec
TE	297.0 K
D1	1.00000000 sec
TD0	1
SFO1	400.1824711 MHz
NUC1	1H
P0	2.67 usec
P1	8.00 usec
SI	65536
SF	400.1800027 MHz
WDW	EM
SSB	0
LB	0.30 Hz
GB	0
PC	1.00

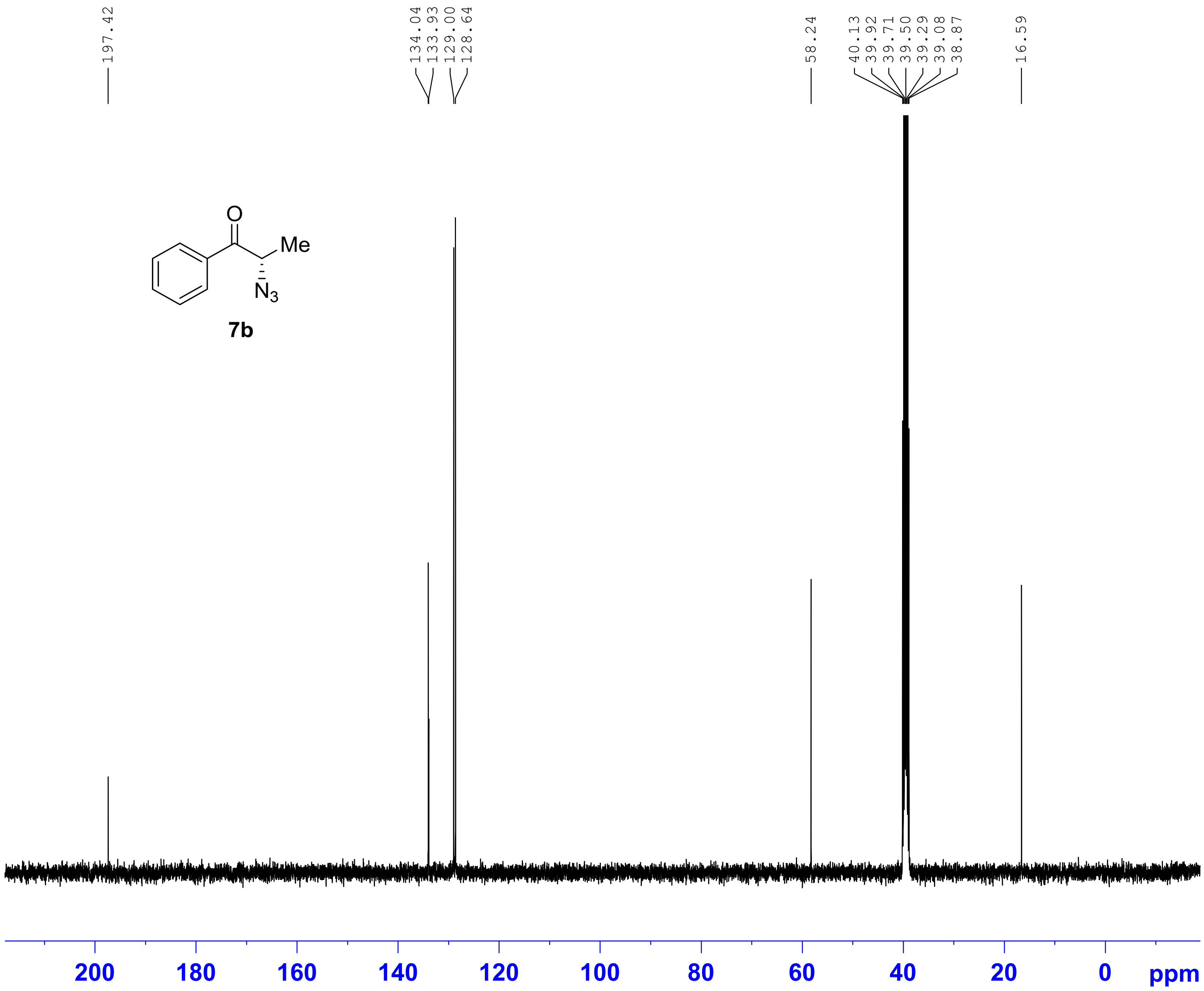

NAME	lzy-11-42-Ph-N3-DMSO-C
EXPNO	3
PROCNO	1
Date_	20230417
Time	10.08 h
INSTRUM	AvanceNeo 400MHz
PROBHD	Z163739_0629 (
PULPROG	zgpg30
TD	65536
SOLVENT	DMSO
NS	465
DS	4
SWH	23809.523 Hz
FIDRES	0.726609 Hz
AQ	1.3763061 sec
RG	11.3
DW	21.000 usec
DE	6.50 usec
TE	297.7 K
D1	2.00000000 sec
D11	0.03000000 sec
TD0	1
SFO1	100.6354036 MHz
NUC1	13C
P0	2.67 usec
P1	8.00 usec
SI	32768
SF	100.6253891 MHz
WDW	EM
SSB	0
LB	1.00 Hz
GB	0
PC	1.40

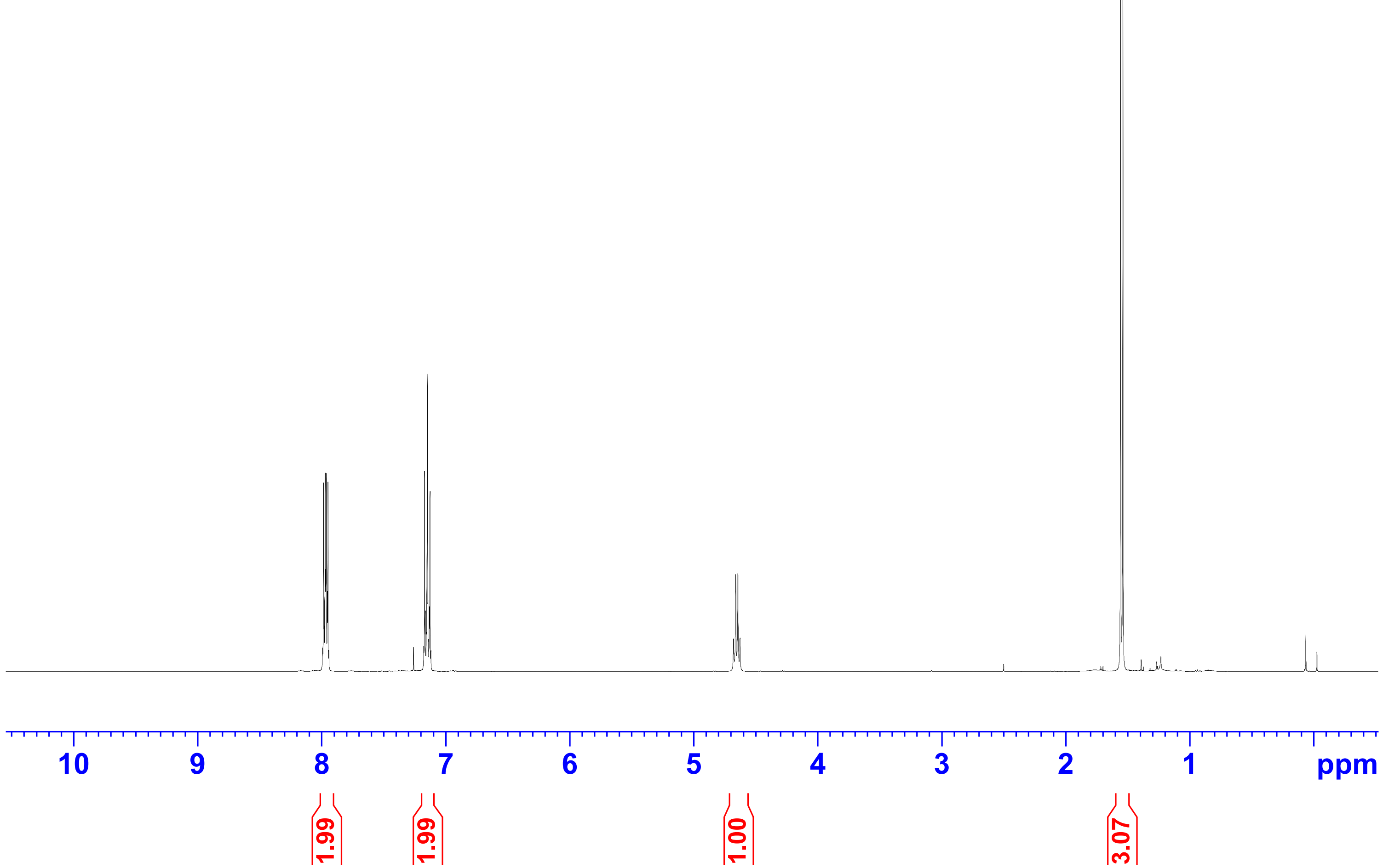

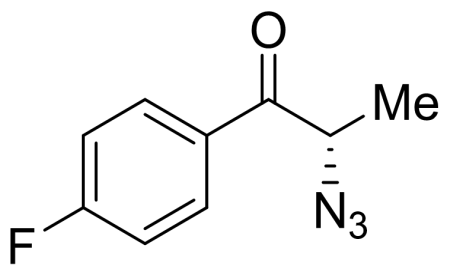

7c

```
Current Data Parameters
NAME                lzy-11-149
EXPNO                        1
PROCNO                       1

F2 - Acquisition Parameters
Date_                 20221115
Time                     21.47
INSTRUM                  spect
PROBHD     5 mm PABBO BB/19F-1H/D Z-GRD Z108618/0582
PULPROG                   zg30
TD                       65536
SOLVENT                  CDCl3
NS                           8
DS                           2
SWH                   8012.820 Hz
FIDRES                0.122266 Hz
AQ                   4.0894465 sec
RG                       31.55
DW                      62.400 usec
DE                        6.50 usec
TE                       296.2 K
D1                  1.00000000 sec
TD0                          1

======== CHANNEL f1 ========
SFO1               400.1324710 MHz
NUC1                        1H
P1                       14.50 usec
PLW1               11.99499989 W

F2 - Processing parameters
SI                       65536
SF                 400.1300104 MHz
WDW                         EM
SSB                          0
LB                        0.30 Hz
GB                           0
PC                        1.00
```

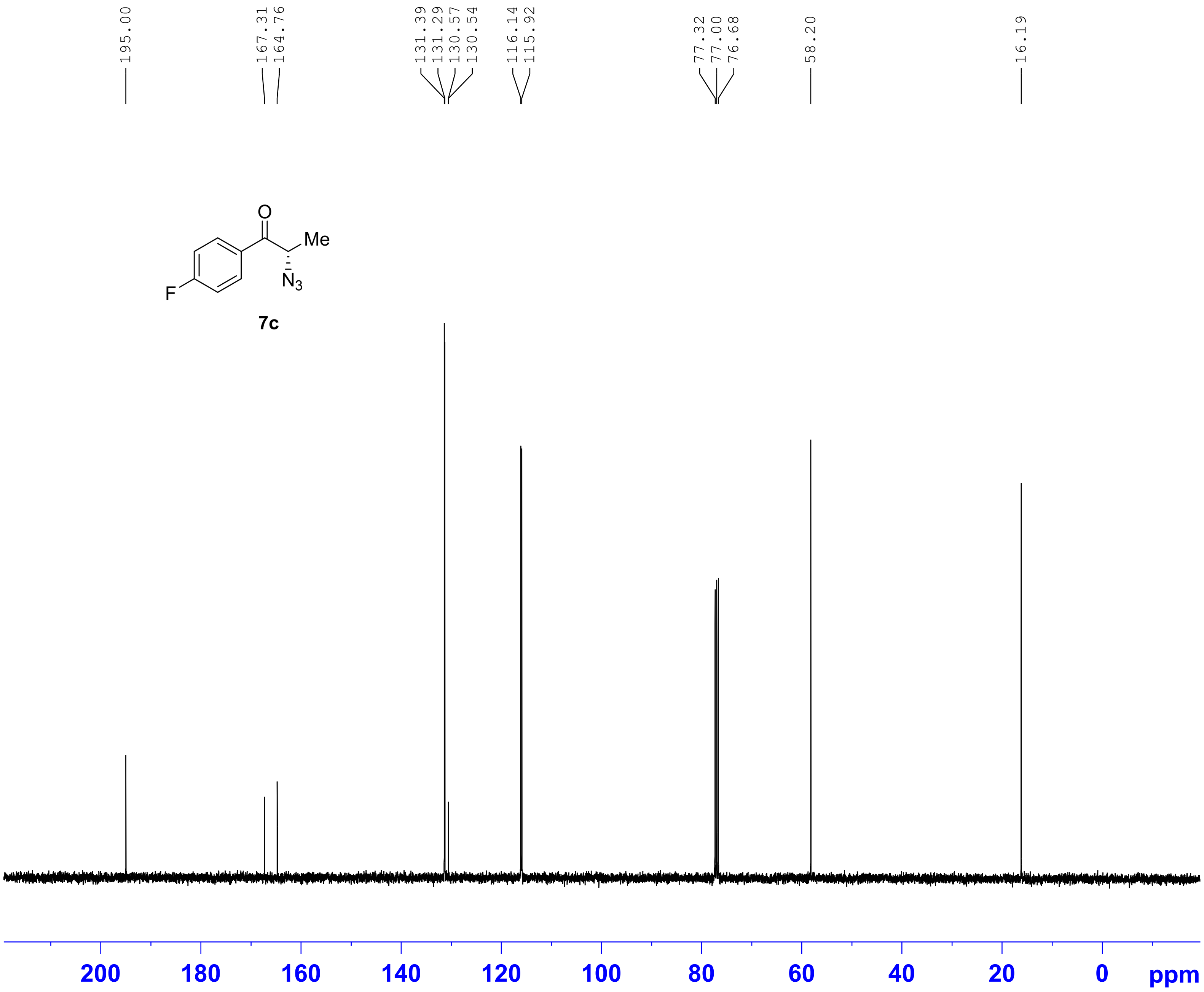

NAME	lzy-11-149-C	
EXPNO	1	
PROCNO	1	
Date_	20221115	
Time	21.51	
INSTRUM	spect	
PROBHD	5 mm PABBO BB/	
PULPROG	zgpg30	
TD	65536	
SOLVENT	CDCl3	
NS	41	
DS	2	
SWH	24038.461	Hz
FIDRES	0.366798	Hz
AQ	1.3631988	sec
RG	196.92	
DW	20.800	usec
DE	6.50	usec
TE	297.0	K
D1	2.00000000	sec
D11	0.03000000	sec
TD0	1	

======== CHANNEL f1 ========

SFO1	100.6228298	MHz
NUC1	13C	
P1	9.70	usec
SI	32768	
SF	100.6127788	MHz
WDW	EM	
SSB	0	
LB	1.00	Hz
GB	0	
PC	1.40	

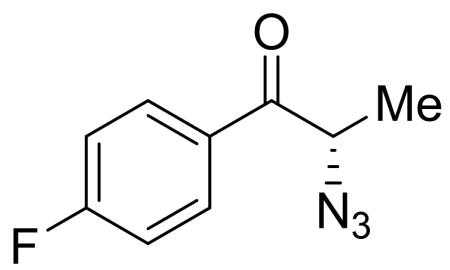

7c

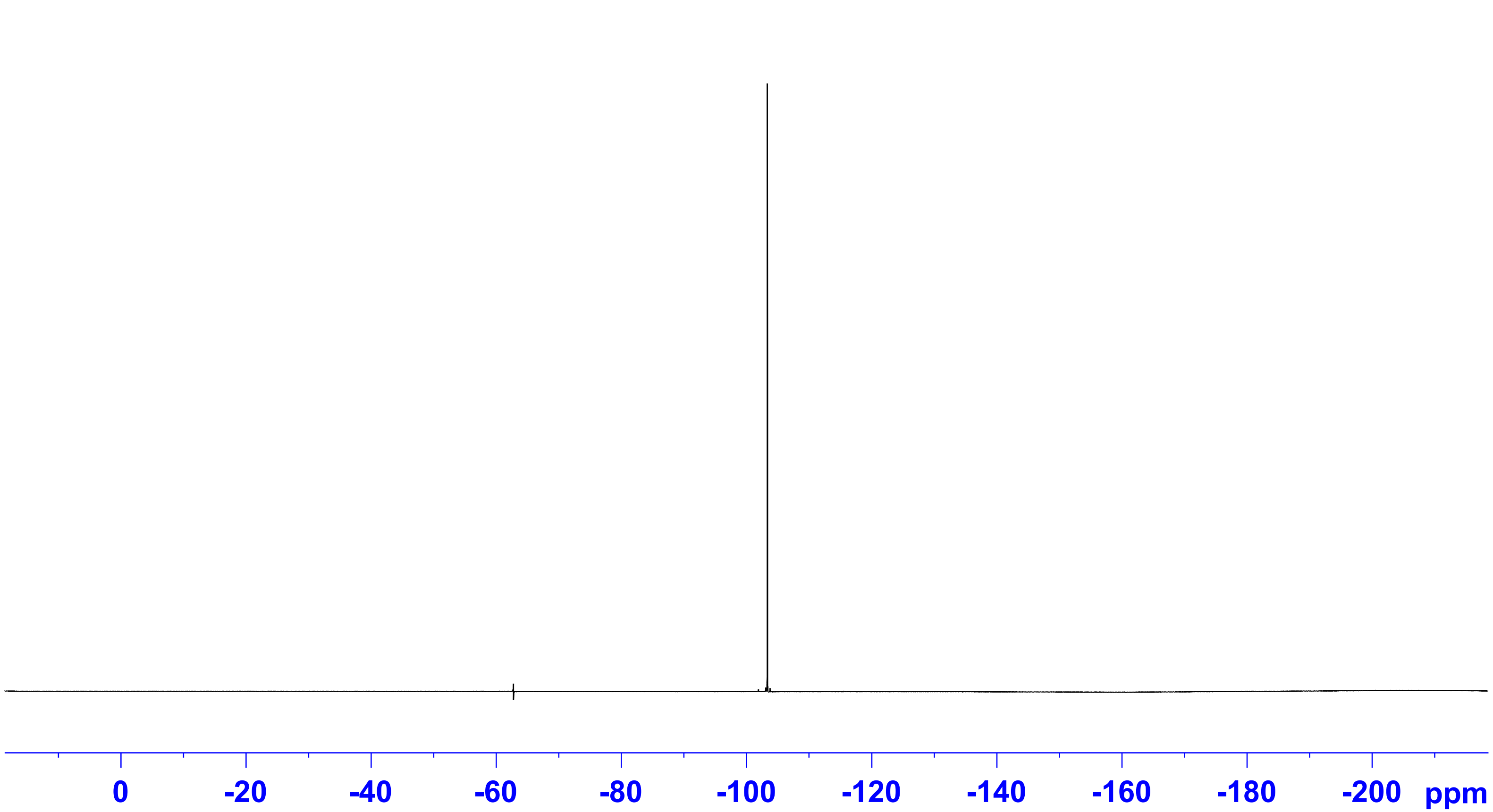

NAME	lzy-11-149-F	
EXPNO	1	
PROCNO	1	
Date_	20221115	
Time	21.53	
INSTRUM	spect	
PROBHD	5 mm PABBO BB/	
PULPROG	zgflqn	
TD	131072	
SOLVENT	CDCl3	
NS	16	
DS	4	
SWH	89285.711	Hz
FIDRES	0.681196	Hz
AQ	0.7340532	sec
RG	196.92	
DW	5.600	usec
DE	6.50	usec
TE	296.4	K
D1	1.00000000	sec
TD0	1	

======== CHANNEL f1 ========

SFO1	376.4607164	MHz
NUC1	19F	
P1	14.70	usec
SI	65536	
SF	376.4983660	MHz
WDW	EM	
SSB	0	
LB	0.30	Hz
GB	0	
PC	1.00	

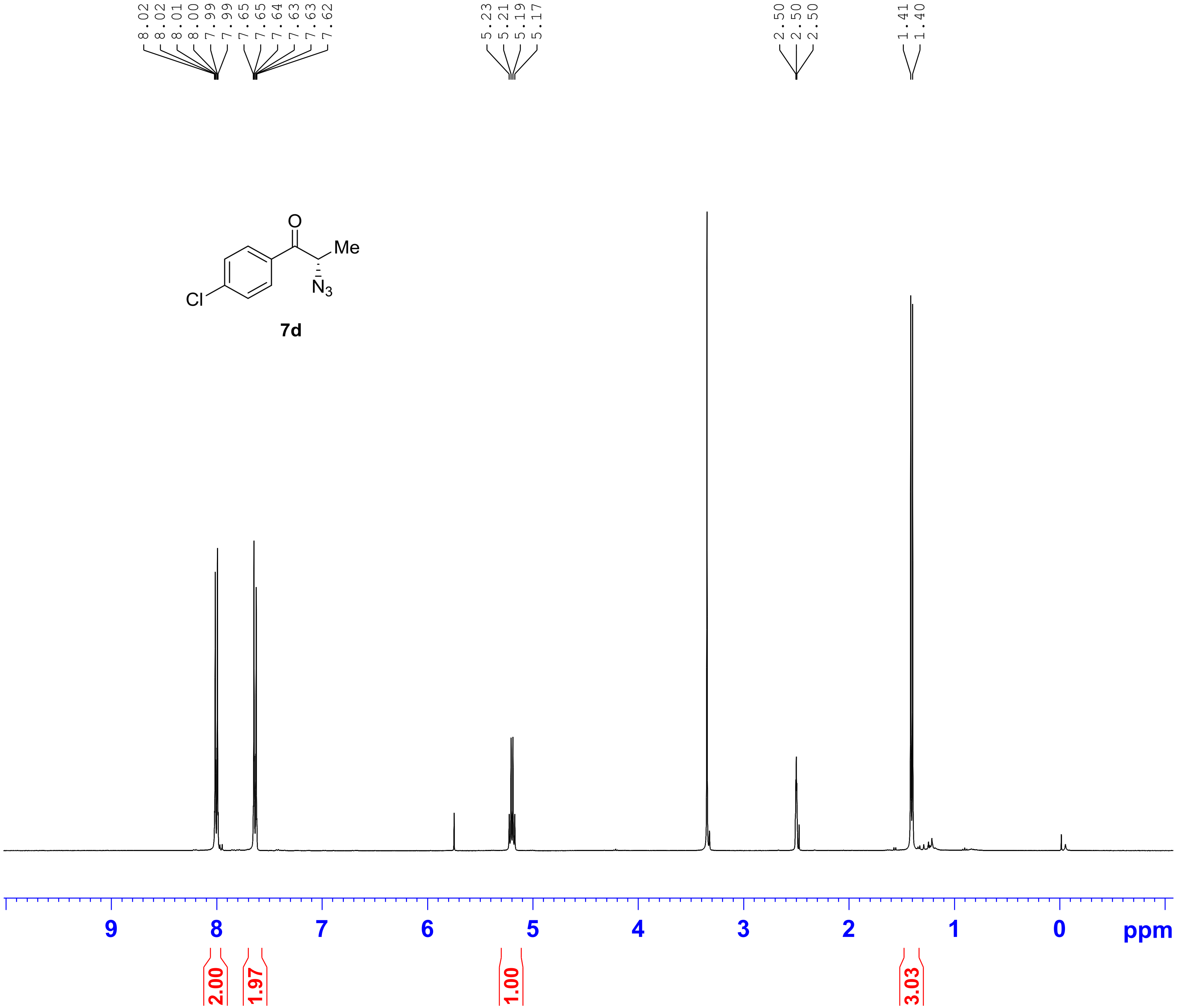

NAME	lzy-11-42-K-DMSO
EXPNO	1
PROCNO	1
Date_	20230411
Time_	19.08 h
INSTRUM	AvanceNeo 400MHz
PROBHD	Z163739_0629 (
PULPROG	zg30
TD	65536
SOLVENT	DMSO
NS	13
DS	2
SWH	8196.722 Hz
FIDRES	0.250144 Hz
AQ	3.9977460 sec
RG	101
DW	61.000 usec
DE	13.89 usec
TE	296.3 K
D1	1.00000000 sec
TD0	1
SFO1	400.1824711 MHz
NUC1	1H
P0	2.67 usec
P1	8.00 usec
SI	65536
SF	400.1800025 MHz
WDW	EM
SSB	0
LB	0.30 Hz
GB	0
PC	1.00

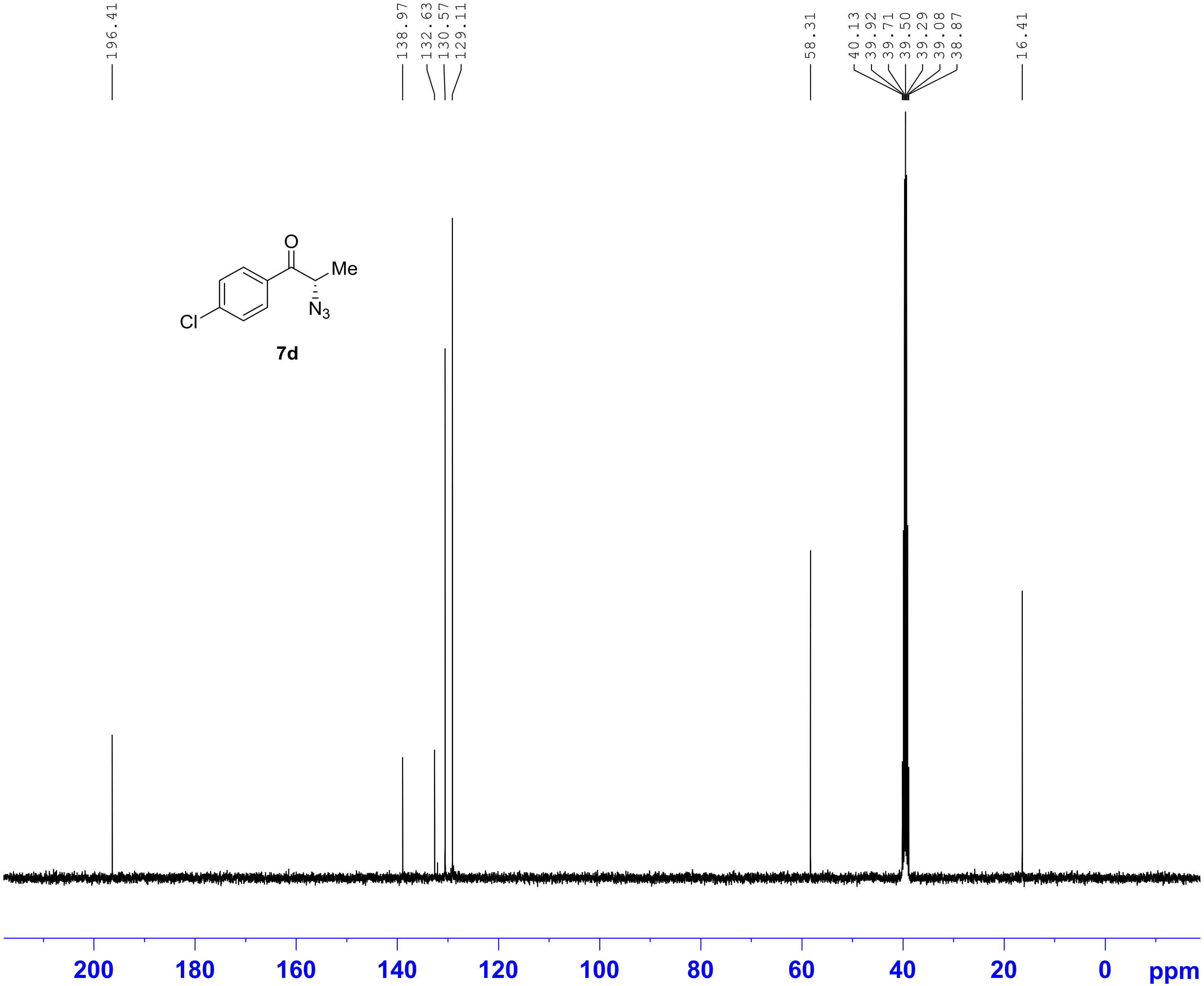

7d

NAME	lzy-11-42-K-DMSO-C
EXPNO	3
PROCNO	1
Date_	20230411
Time_	19.18 h
INSTRUM	AvanceNeo 400MHz
PROBHD	Z163739_0629 (
PULPROG	zgpg30
TD	65536
SOLVENT	DMSO
NS	147
DS	4
SWH	23809.523 Hz
FIDRES	0.726609 Hz
AQ	1.3763061 sec
RG	10
DW	21.000 usec
DE	6.50 usec
TE	297.1 K
D1	2.00000000 sec
D11	0.03000000 sec
TD0	1
SFO1	100.6354036 MHz
NUC1	13C
P0	2.67 usec
P1	8.00 usec
SI	32768
SF	100.6253886 MHz
WDW	EM
SSB	0
LB	1.00 Hz
GB	0
PC	1.40

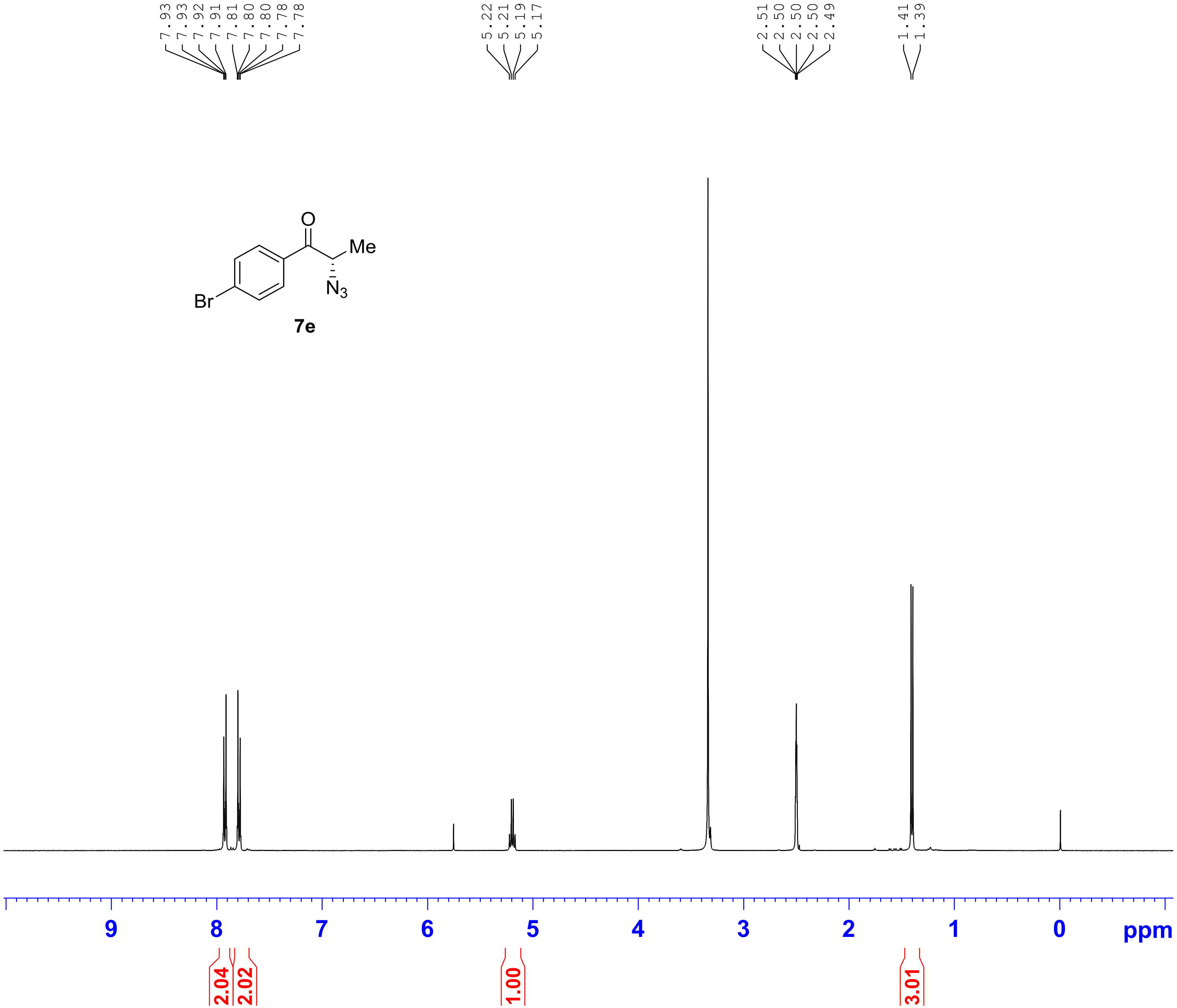

NAME	lzy-11-42-E-DMSO
EXPNO	1
PROCNO	1
Date_	20230411
Time	17.41 h
INSTRUM	AvanceNeo 400MHz
PROBHD	Z163739_0629 (
PULPROG	zg30
TD	65536
SOLVENT	DMSO
NS	16
DS	2
SWH	8196.722 Hz
FIDRES	0.250144 Hz
AQ	3.9977460 sec
RG	101
DW	61.000 usec
DE	13.89 usec
TE	296.6 K
D1	1.00000000 sec
TD0	1
SFO1	400.1824711 MHz
NUC1	1H
P0	2.67 usec
P1	8.00 usec
SI	65536
SF	400.1800027 MHz
WDW	EM
SSB	0
LB	0.30 Hz
GB	0
PC	1.00

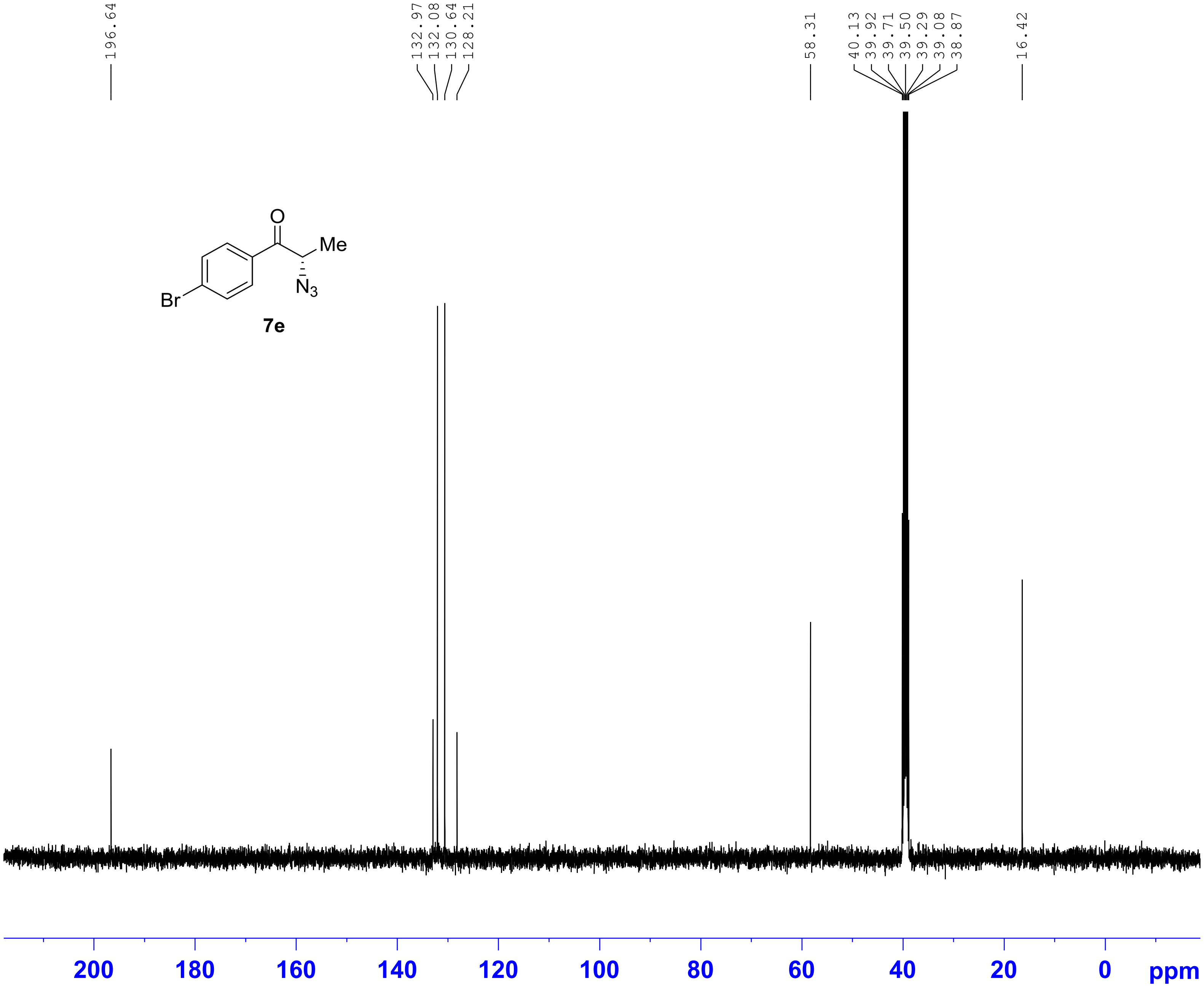

NAME	lzy-11-42-E-DMSO-C
EXPNO	3
PROCNO	1
Date_	20230411
Time	17.55 h
INSTRUM	AvanceNeo 400MHz
PROBHD	Z163739_0629 (
PULPROG	zgpg30
TD	65536
SOLVENT	DMSO
NS	206
DS	4
SWH	23809.523 Hz
FIDRES	0.726609 Hz
AQ	1.3763061 sec
RG	11.3
DW	21.000 usec
DE	6.50 usec
TE	297.1 K
D1	2.00000000 sec
D11	0.03000000 sec
TD0	1
SFO1	100.6354036 MHz
NUC1	13C
P0	2.67 usec
P1	8.00 usec
SI	32768
SF	100.6253893 MHz
WDW	EM
SSB	0
LB	1.00 Hz
GB	0
PC	1.40

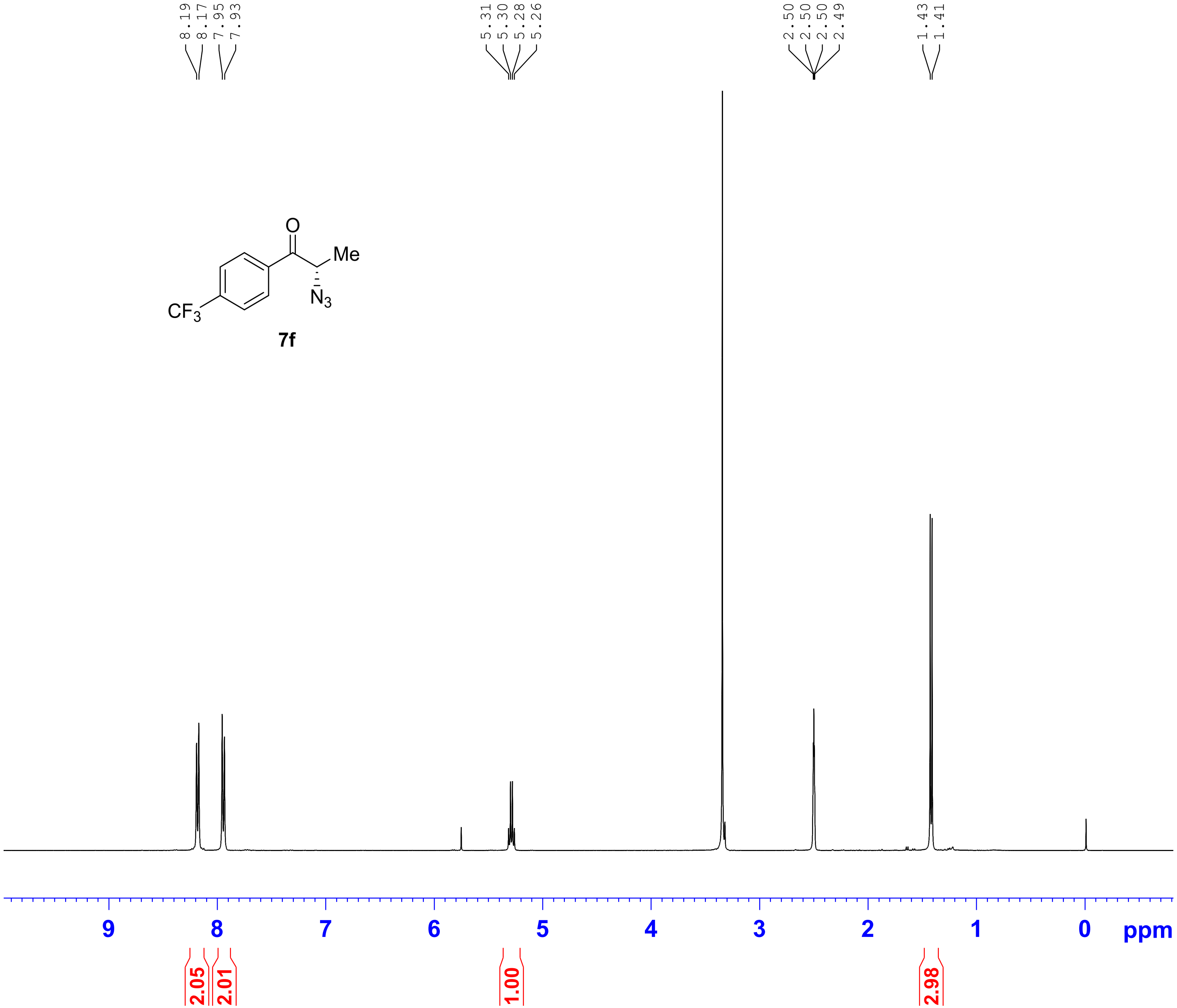

NAME	lzy-11-42-p-cf3-DMSO
EXPNO	1
PROCNO	1
Date_	20230413
Time	19.00 h
INSTRUM	AvanceNeo 400MHz
PROBHD	Z163739_0629 (
PULPROG	zg30
TD	65536
SOLVENT	DMSO
NS	4
DS	2
SWH	8196.722 Hz
FIDRES	0.250144 Hz
AQ	3.9977460 sec
RG	101
DW	61.000 usec
DE	13.89 usec
TE	296.4 K
D1	1.00000000 sec
TD0	1
SFO1	400.1824711 MHz
NUC1	1H
P0	2.67 usec
P1	8.00 usec
SI	65536
SF	400.1800027 MHz
WDW	EM
SSB	0
LB	0.30 Hz
GB	0
PC	1.00

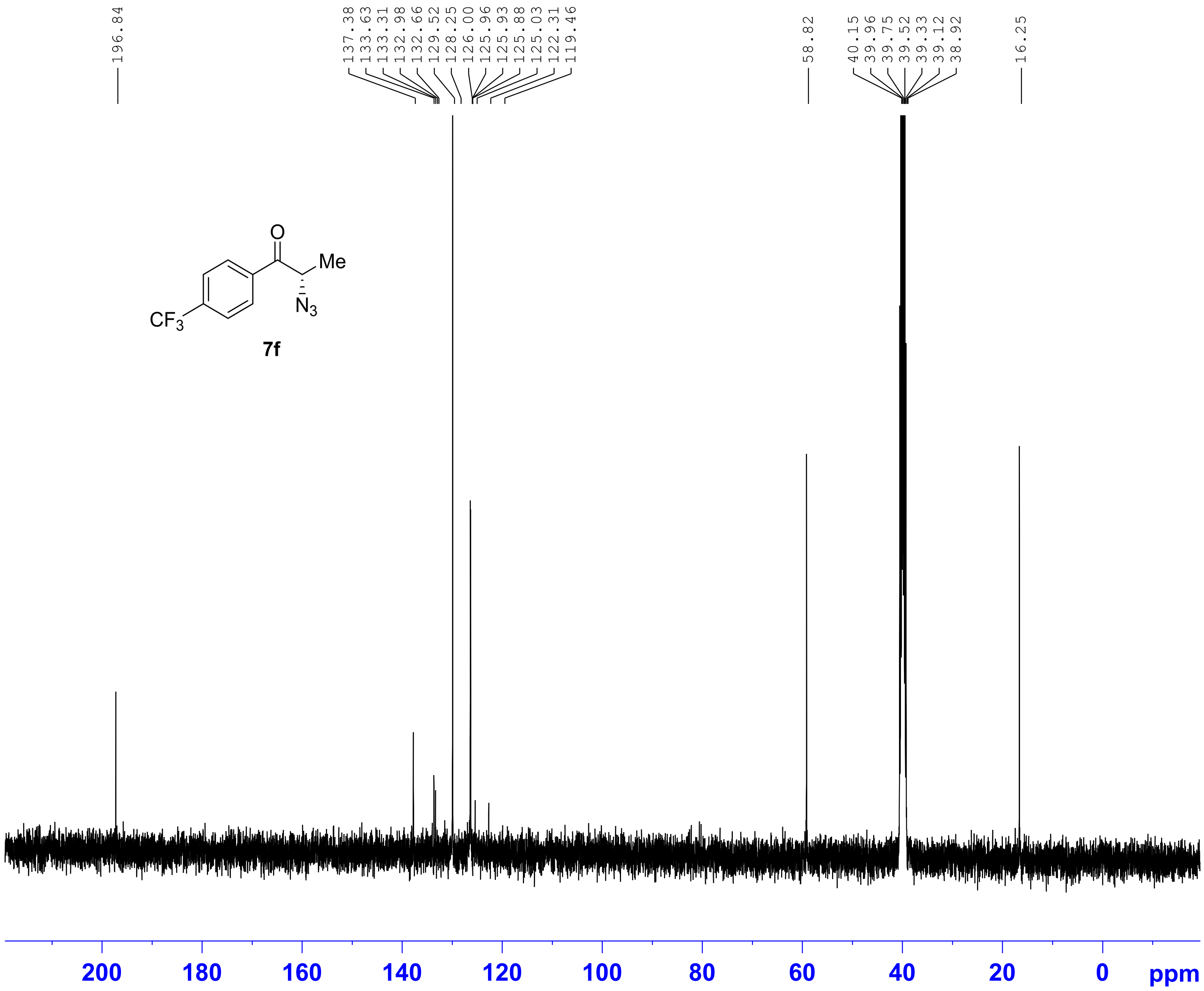

NAME	lzy-11-42-p-cf3-DMSO-C
EXPNO	1
PROCNO	1
Date_	20230413
Time	20.25
INSTRUM	spect
PROBHD	5 mm PABBO BB/
PULPROG	zgpg30
TD	65536
SOLVENT	DMSO
NS	340
DS	2
SWH	24038.461 Hz
FIDRES	0.366798 Hz
AQ	1.3631988 sec
RG	196.92
DW	20.800 usec
DE	6.50 usec
TE	296.9 K
D1	2.00000000 sec
D11	0.03000000 sec
TD0	1

======== CHANNEL f1 ========

SFO1	100.6228298 MHz
NUC1	13C
P1	9.70 usec
SI	32768
SF	100.6127739 MHz
WDW	EM
SSB	0
LB	1.00 Hz
GB	0
PC	1.40

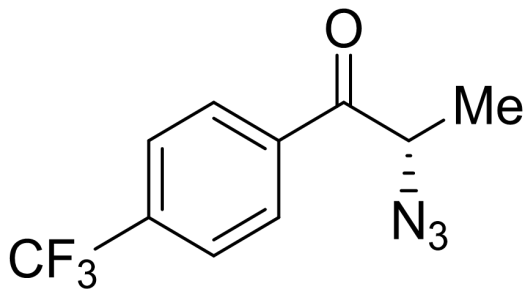

7f

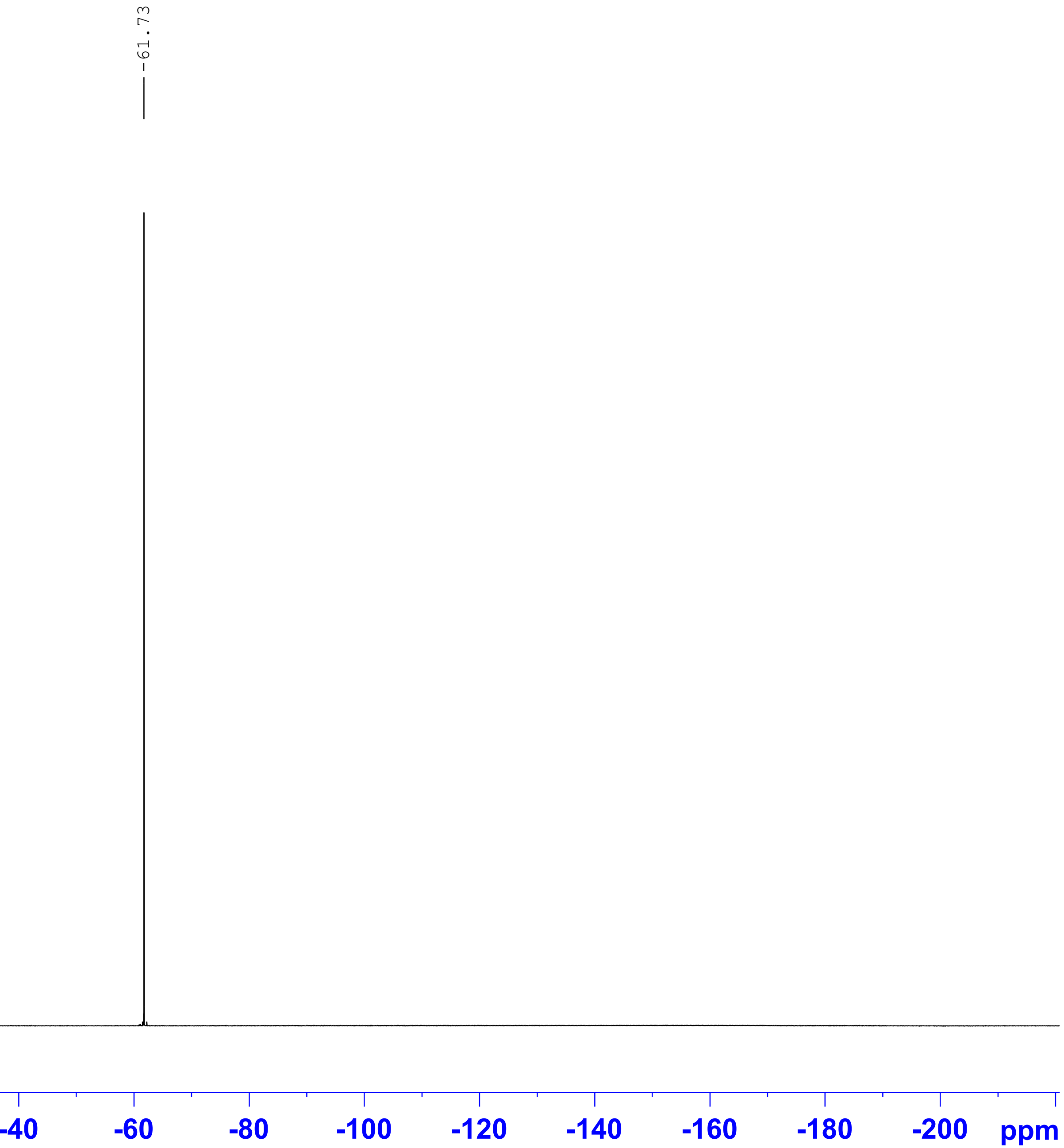

NAME	lzy-11-42-p-cf3-DMSO-F
EXPNO	5
PROCNO	1
Date_	20230413
Time	19.08 h
INSTRUM	AvanceNeo 400MHz
PROBHD	Z163739_0629 (
PULPROG	zg
TD	131072
SOLVENT	DMSO
NS	3
DS	4
SWH	90909.094 Hz
FIDRES	1.387163 Hz
AQ	0.7209460 sec
RG	101
DW	5.500 usec
DE	6.50 usec
TE	296.6 K
D1	1.00000000 sec
TD0	1
SFO1	376.5077587 MHz
NUC1	19F
P1	12.00 usec
SI	65536
SF	376.5454132 MHz
WDW	EM
SSB	0
LB	0.30 Hz
GB	0
PC	1.00

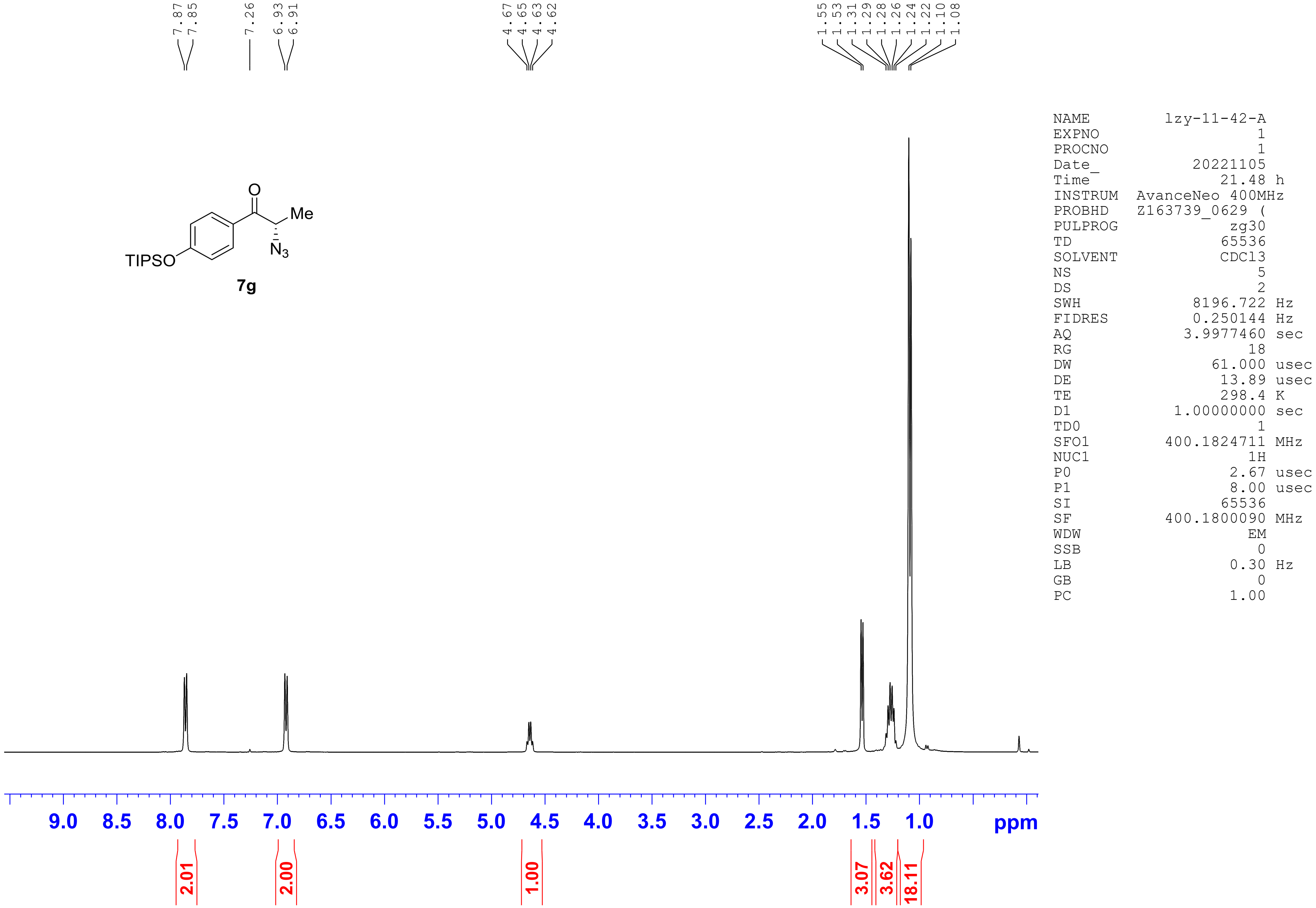

7g
7.87
7.85
7.26
6.93
6.91
4.67
4.65
4.63
4.62
1.55
1.53
1.31
1.29
1.28
1.26
1.24
1.22
1.10
1.08
NAME lzy-11-42-A
EXPNO 1
PROCNO 1
Date_ 20221105
Time_ 21.48 h
INSTRUM AvanceNeo 400MHz
PROBHD Z163739_0629 (
PULPROG zg30
TD 65536
SOLVENT CDCl3
NS 5
DS 2
SWH 8196.722 Hz
FIDRES 0.250144 Hz
AQ 3.9977460 sec
RG 18
DW 61.000 usec
DE 13.89 usec
TE 298.4 K
D1 1.00000000 sec
TD0 1
SFO1 400.1824711 MHz
NUC1 1H
P0 2.67 usec
P1 8.00 usec
SI 65536
SF 400.1800090 MHz
WDW EM
SSB 0
LB 0.30 Hz
GB 0
PC 1.00
9.0 8.5 8.0 7.5 7.0 6.5 6.0 5.5 5.0 4.5 4.0 3.5 3.0 2.5 2.0 1.5 1.0 ppm
2.01
2.00
1.00
3.07
3.62
18.11

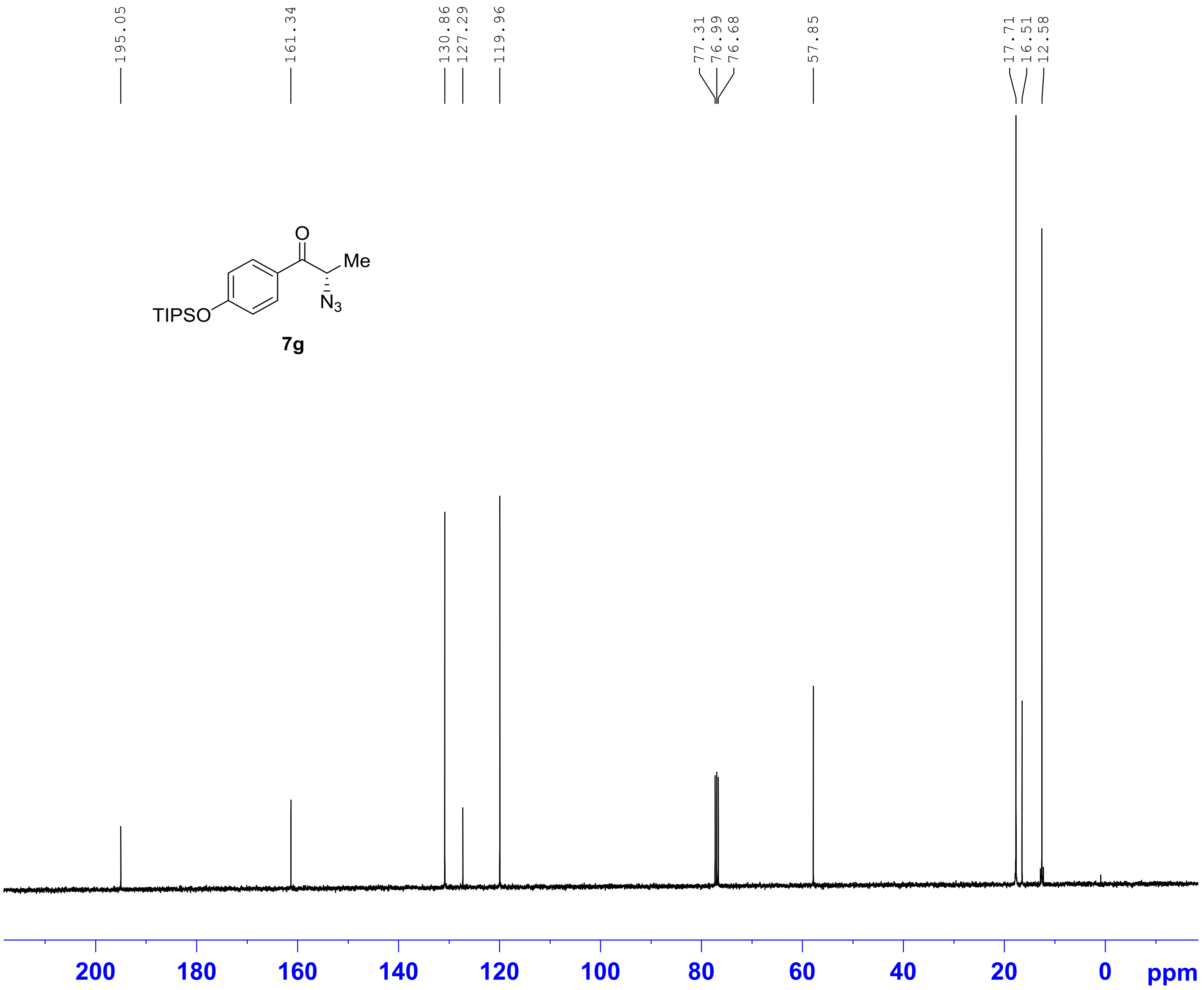

7g

NAME	lzy-11-42-A-C
EXPNO	3
PROCNO	1
Date_	20221105
Time	21.53 h
INSTRUM	AvanceNeo 400MHz
PROBHD	Z163739_0629 (
PULPROG	zgpg30
TD	65536
SOLVENT	CDCl3
NS	34
DS	4
SWH	23809.523 Hz
FIDRES	0.726609 Hz
AQ	1.3763061 sec
RG	10
DW	21.000 usec
DE	6.50 usec
TE	300.9 K
D1	2.00000000 sec
D11	0.03000000 sec
TD0	1
SFO1	100.6354036 MHz
NUC1	13C
P0	2.67 usec
P1	8.00 usec
SI	32768
SF	100.6253516 MHz
WDW	EM
SSB	0
LB	1.00 Hz
GB	0
PC	1.40

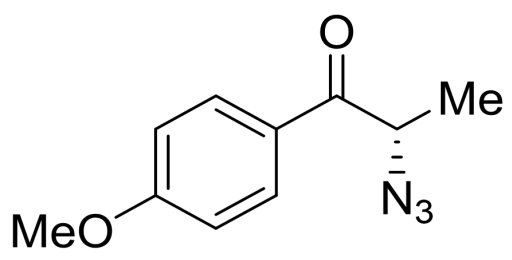

7h

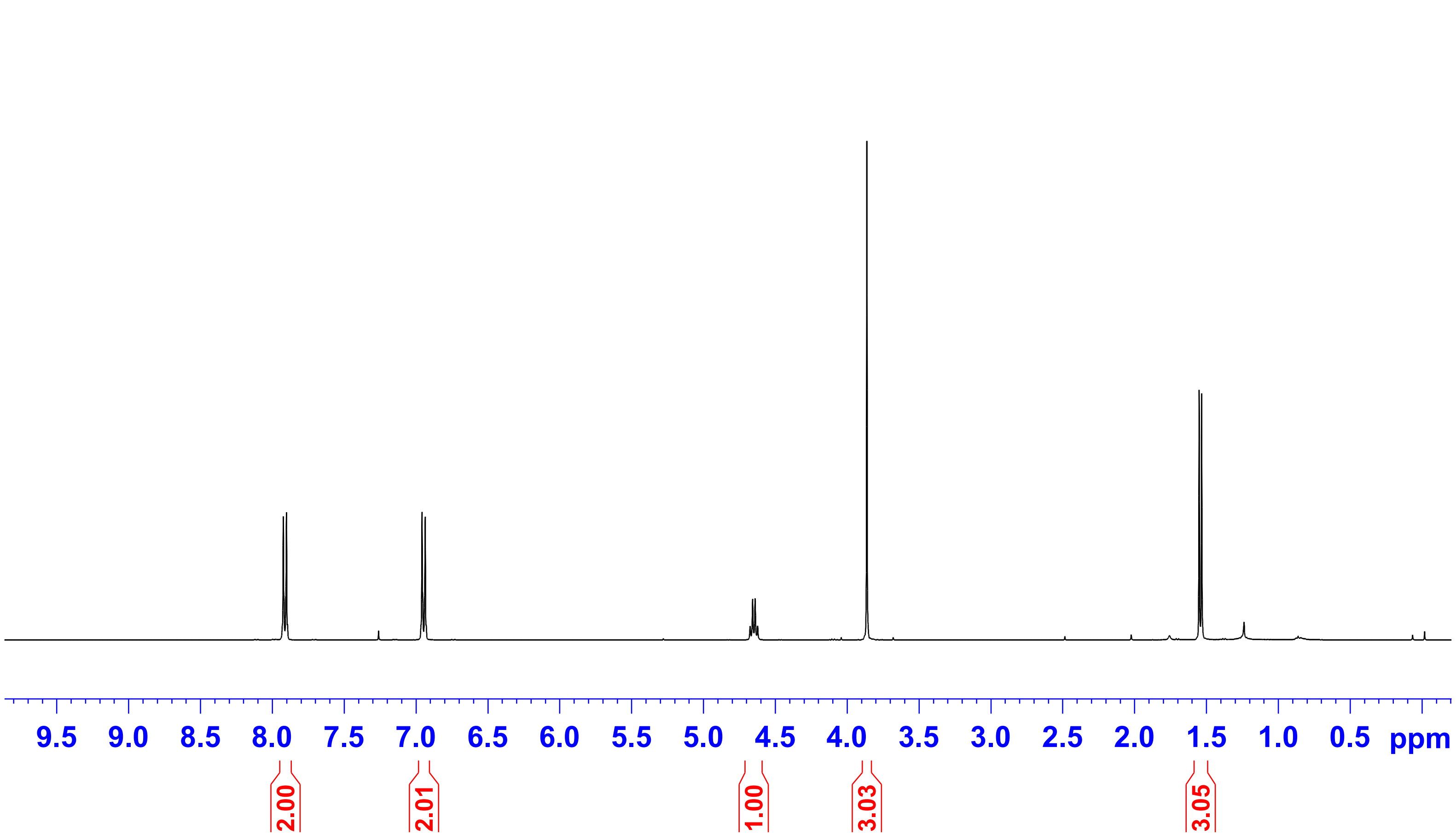

NAME	lzy-11-19
EXPNO	1
PROCNO	1
Date_	20220829
Time	21.22
INSTRUM	spect
PROBHD	5 mm DUL 13C-1
PULPROG	zg30
TD	65536
SOLVENT	CDCl3
NS	1
DS	0
SWH	8223.685 Hz
FIDRES	0.125483 Hz
AQ	3.9846387 sec
RG	144
DW	60.800 usec
DE	6.00 usec
TE	293.0 K
D1	1.00000000 sec
TD0	1

======== CHANNEL f1 ========

NUC1	1H
P1	15.80 usec
PL1	-1.00 dB
PL1W	12.17476940 W
SFO1	400.1324710 MHz
SI	32768
SF	400.1300098 MHz
WDW	EM
SSB	0
LB	0.30 Hz
GB	0
PC	1.00

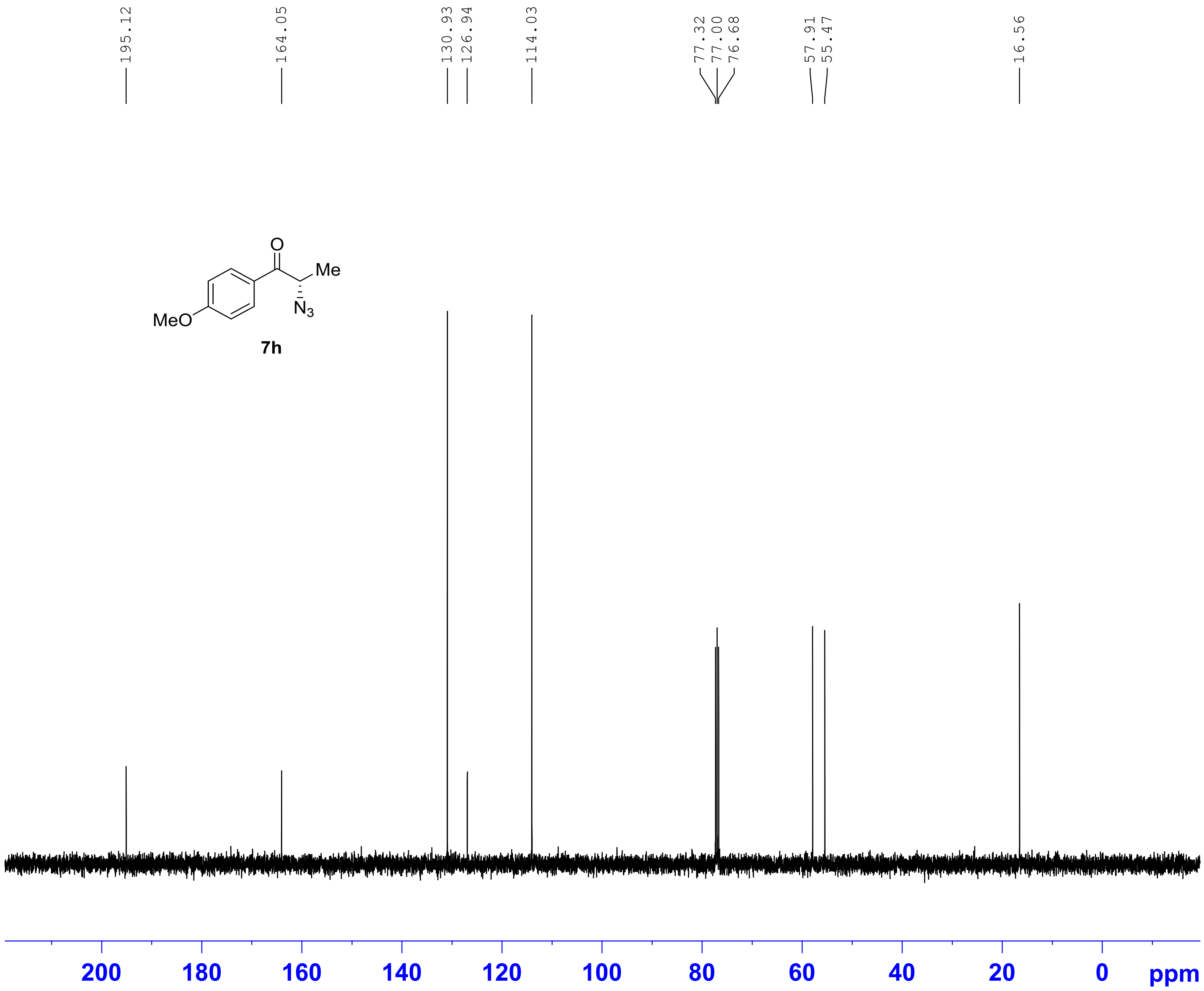

NAME	lzy-11-19-C
EXPNO	1
PROCNO	1
Date_	20220829
Time_	21.23
INSTRUM	spect
PROBHD	5 mm DUL 13C-1
PULPROG	zgpg30
TD	65536
SOLVENT	CDCl3
NS	23
DS	0
SWH	24038.461 Hz
FIDRES	0.366798 Hz
AQ	1.3631988 sec
RG	2050
DW	20.800 usec
DE	6.00 usec
TE	293.3 K
D1	2.00000000 sec
D11	0.03000000 sec
TD0	1

======== CHANNEL f1 ========

NUC1	13C
P1	40.00 usec
PL1	-3.00 dB
PL1W	60.64365387 W
SFO1	100.6228298 MHz

======== CHANNEL f2 ========

CPDPRG2	waltz16
NUC2	1H
PCPD2	80.00 usec
PL2	-1.00 dB
PL12	14.39 dB
PL13	18.00 dB
PL2W	12.17476940 W
PL12W	0.35193357 W
PL13W	0.15327126 W
SFO2	400.1316005 MHz
SI	32768
SF	100.6127795 MHz
WDW	EM
SSB	0
LB	1.00 Hz
GB	0
PC	1.40

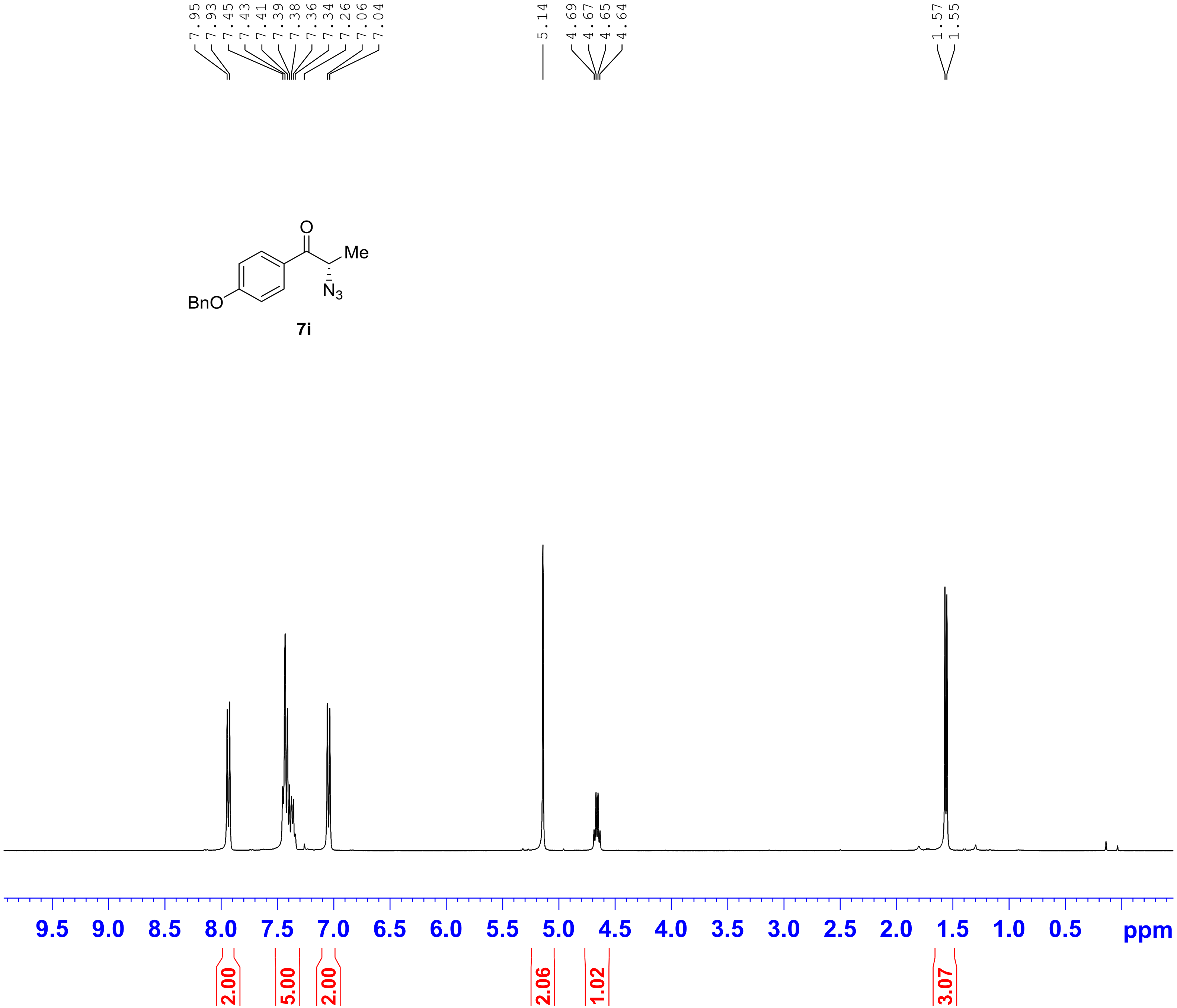

NAME	lzy-11-191
EXPNO	1
PROCNO	1
Date_	20221107
Time	21.25 h
INSTRUM	AvanceNeo 400MHz
PROBHD	Z163739_0629 (
PULPROG	zg30
TD	65536
SOLVENT	CDCl3
NS	4
DS	2
SWH	8196.722 Hz
FIDRES	0.250144 Hz
AQ	3.9977460 sec
RG	36
DW	61.000 usec
DE	13.89 usec
TE	300.3 K
D1	1.00000000 sec
TD0	1
SFO1	400.1824711 MHz
NUC1	1H
P0	2.67 usec
P1	8.00 usec
SI	65536
SF	400.1800081 MHz
WDW	EM
SSB	0
LB	0.30 Hz
GB	0
PC	1.00

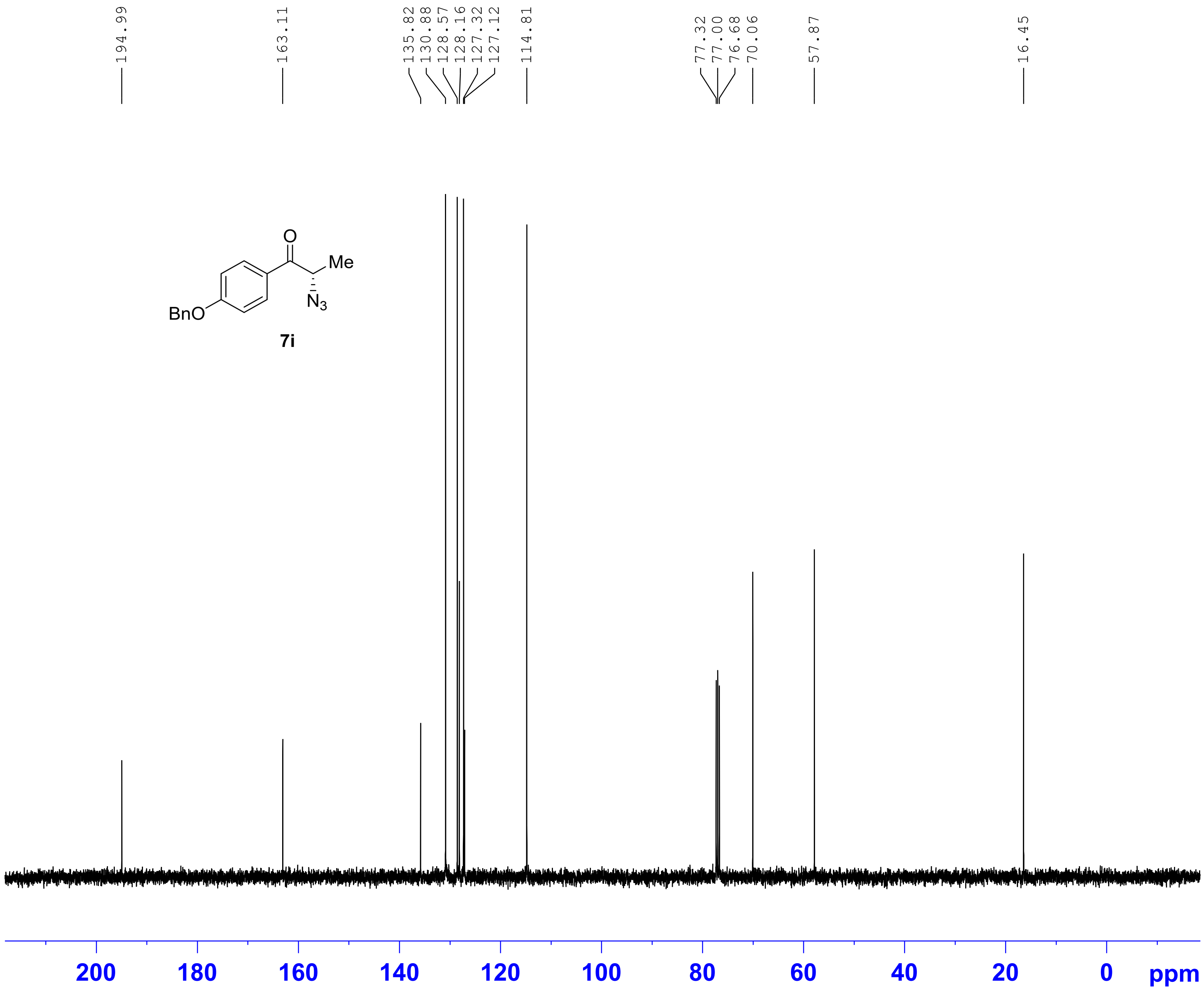

7i

NAME	lzy-11-191-C
EXPNO	3
PROCNO	1
Date_	20221107
Time_	21.28 h
INSTRUM	AvanceNeo 400MHz
PROBHD	Z163739_0629 (
PULPROG	zgpg30
TD	65536
SOLVENT	CDCl3
NS	10
DS	4
SWH	23809.523 Hz
FIDRES	0.726609 Hz
AQ	1.3763061 sec
RG	10
DW	21.000 usec
DE	6.50 usec
TE	300.6 K
D1	2.00000000 sec
D11	0.03000000 sec
TD0	1
SFO1	100.6354036 MHz
NUC1	13C
P0	2.67 usec
P1	8.00 usec
SI	32768
SF	100.6253616 MHz
WDW	EM
SSB	0
LB	1.00 Hz
GB	0
PC	1.40

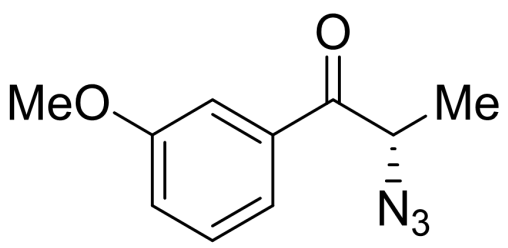

7j

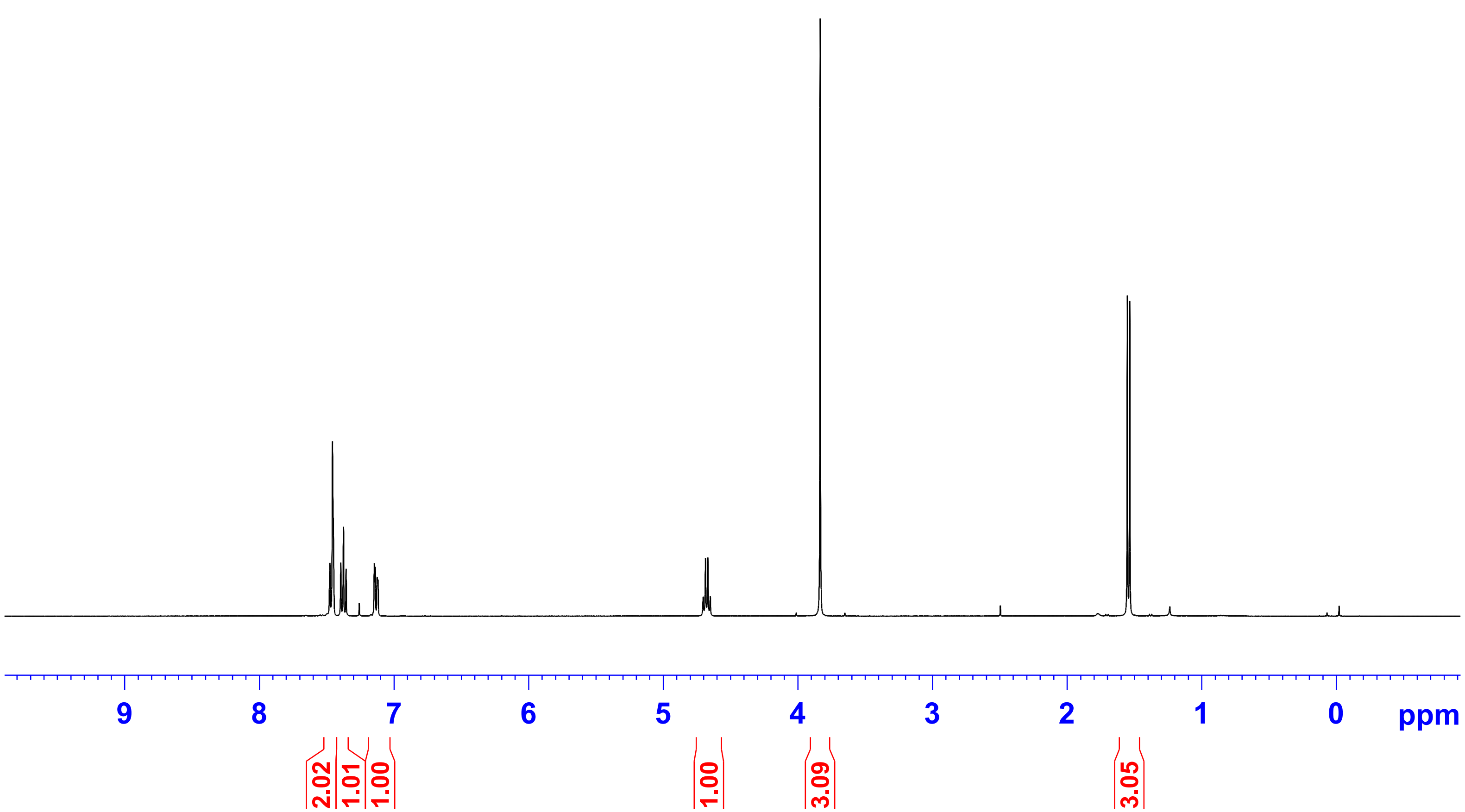


```
NAME         lzy-10-115-B
EXPNO                   3
PROCNO                  1
Date_            20220716
Time                10.17
INSTRUM             spect
PROBHD     5 mm PABBO BB/
PULPROG              zg30
TD                  65536
SOLVENT             CDCl3
NS                      4
DS                      0
SWH              8012.820 Hz
FIDRES           0.122266 Hz
AQ              4.0894966 sec
RG                  31.55
DW                 62.400 usec
DE                   6.50 usec
TE                  295.7 K
D1             1.00000000 sec
TD0                     1

======== CHANNEL f1 ========
SFO1          400.1324710 MHz
NUC1                   1H
P1                  14.50 usec
SI                  65536
SF            400.1300102 MHz
WDW                    EM
SSB                     0
LB                   0.30 Hz
GB                      0
PC                   1.00
```

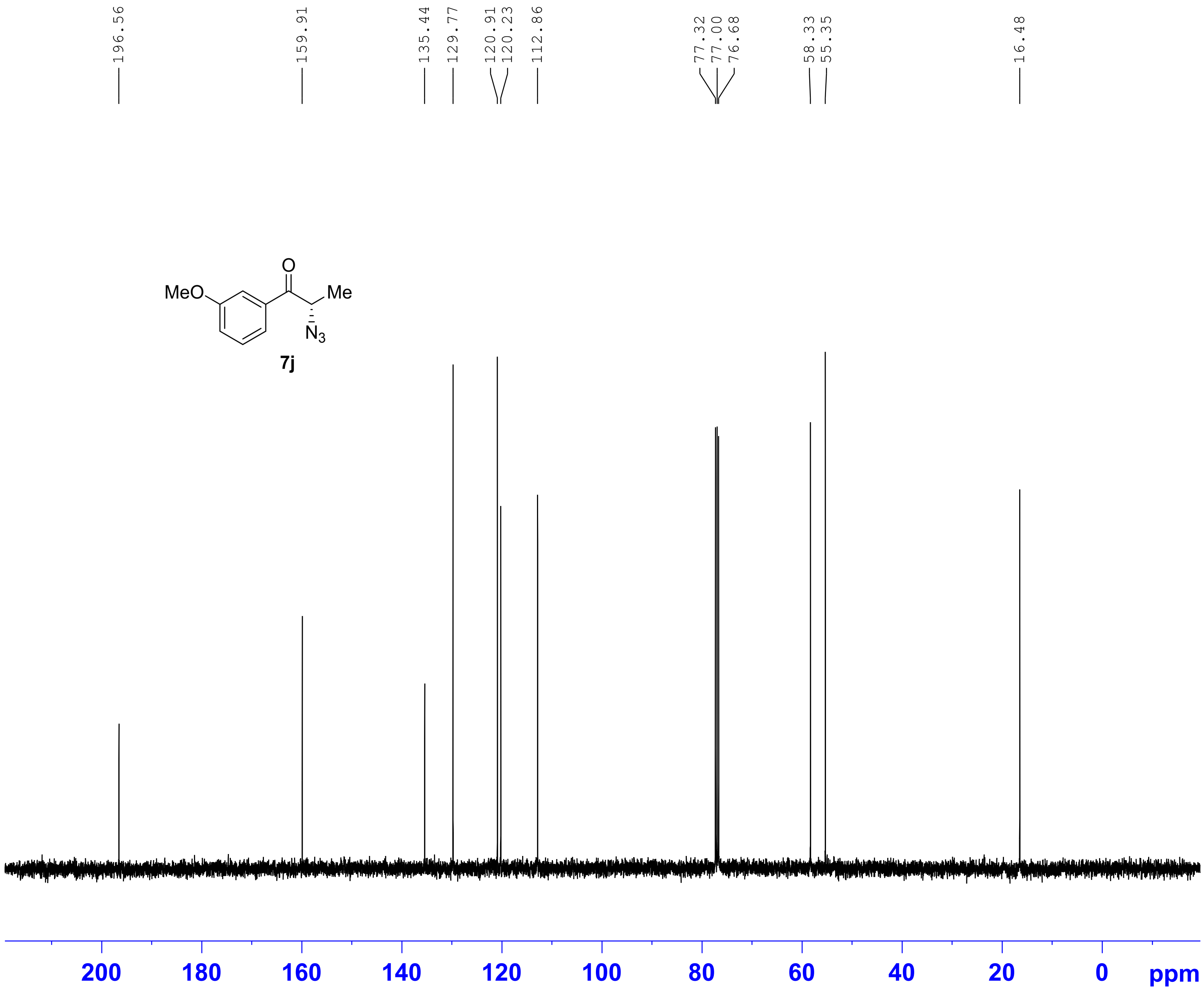

7j

NAME	lzy-10-115-B-C	
EXPNO	3	
PROCNO	1	
Date_	20220716	
Time_	10.20	
INSTRUM	spect	
PROBHD	5 mm PABBO BB/	
PULPROG	zgpg30	
TD	65536	
SOLVENT	CDCl3	
NS	38	
DS	0	
SWH	24038.461	Hz
FIDRES	0.366798	Hz
AQ	1.3631988	sec
RG	196.92	
DW	20.800	usec
DE	6.50	usec
TE	296.4	K
D1	2.00000000	sec
D11	0.03000000	sec
TD0	1	

======== CHANNEL f1 ========

SFO1	100.6228298	MHz
NUC1	13C	
P1	9.70	usec
SI	32768	
SF	100.6127813	MHz
WDW	EM	
SSB	0	
LB	1.00	Hz
GB	0	
PC	1.40	

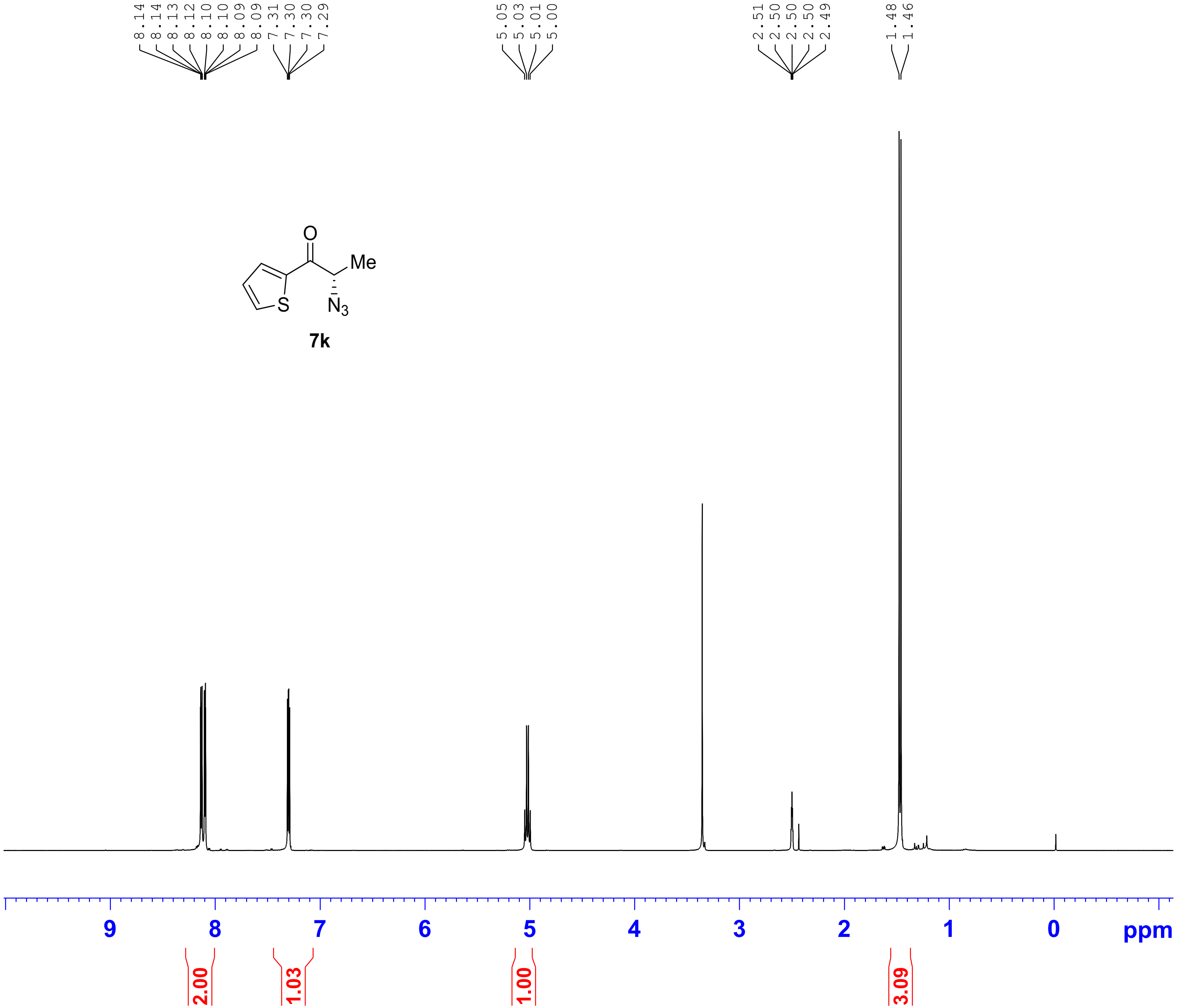

7k

```
NAME       lzy-11-42-D-DMSO
EXPNO                     1
PROCNO                    1
Date_              20230411
Time                  17.47
INSTRUM               spect
PROBHD     5 mm PABBO BB/
PULPROG                zg30
TD                    65536
SOLVENT                DMSO
NS                       16
DS                        2
SWH                8012.820 Hz
FIDRES             0.122266 Hz
AQ                4.0894966 sec
RG                    62.93
DW                   62.400 usec
DE                     6.50 usec
TE                    296.0 K
D1               1.00000000 sec
TD0                       1

======== CHANNEL f1 ========
SFO1            400.1324710 MHz
NUC1                     1H
P1                    14.50 usec
SI                    65536
SF              400.1300037 MHz
WDW                      EM
SSB                       0
LB                     0.30 Hz
GB                        0
PC                     1.00
```

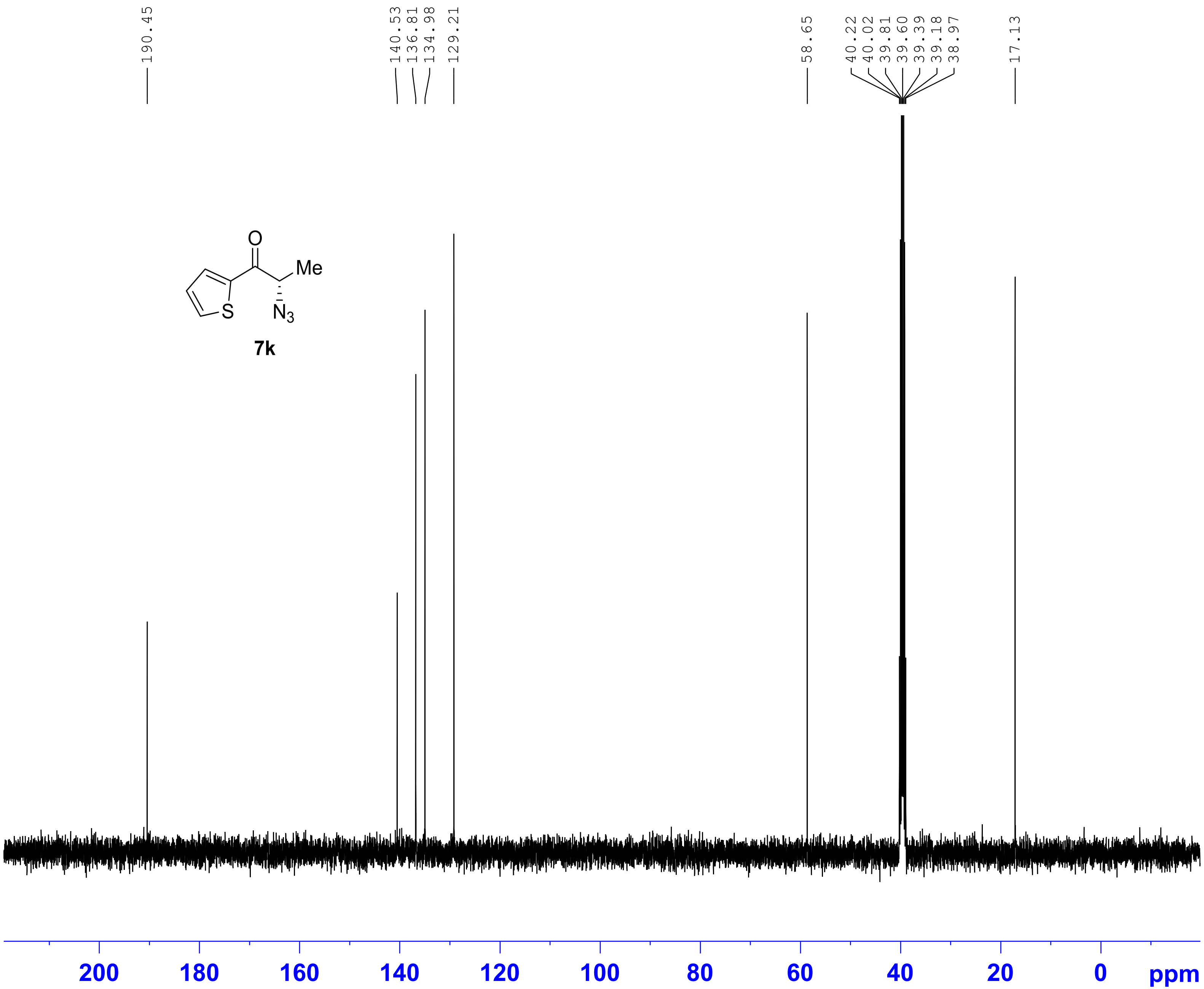

7k

NAME	lzy-11-42-D-DMSO-C
EXPNO	1
PROCNO	1
Date_	20230411
Time	17.50
INSTRUM	spect
PROBHD	5 mm PABBO BB/
PULPROG	zgpg30
TD	65536
SOLVENT	DMSO
NS	60
DS	2
SWH	24038.461 Hz
FIDRES	0.366798 Hz
AQ	1.3631988 sec
RG	196.92
DW	20.800 usec
DE	6.50 usec
TE	296.8 K
D1	2.00000000 sec
D11	0.03000000 sec
TD0	1

======== CHANNEL f1 ========

SFO1	100.6228298 MHz
NUC1	13C
P1	9.70 usec
SI	32768
SF	100.6128067 MHz
WDW	EM
SSB	0
LB	1.00 Hz
GB	0
PC	1.40

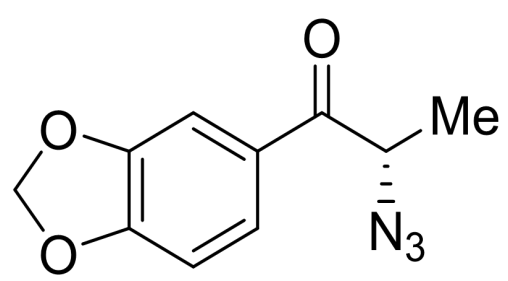

7l

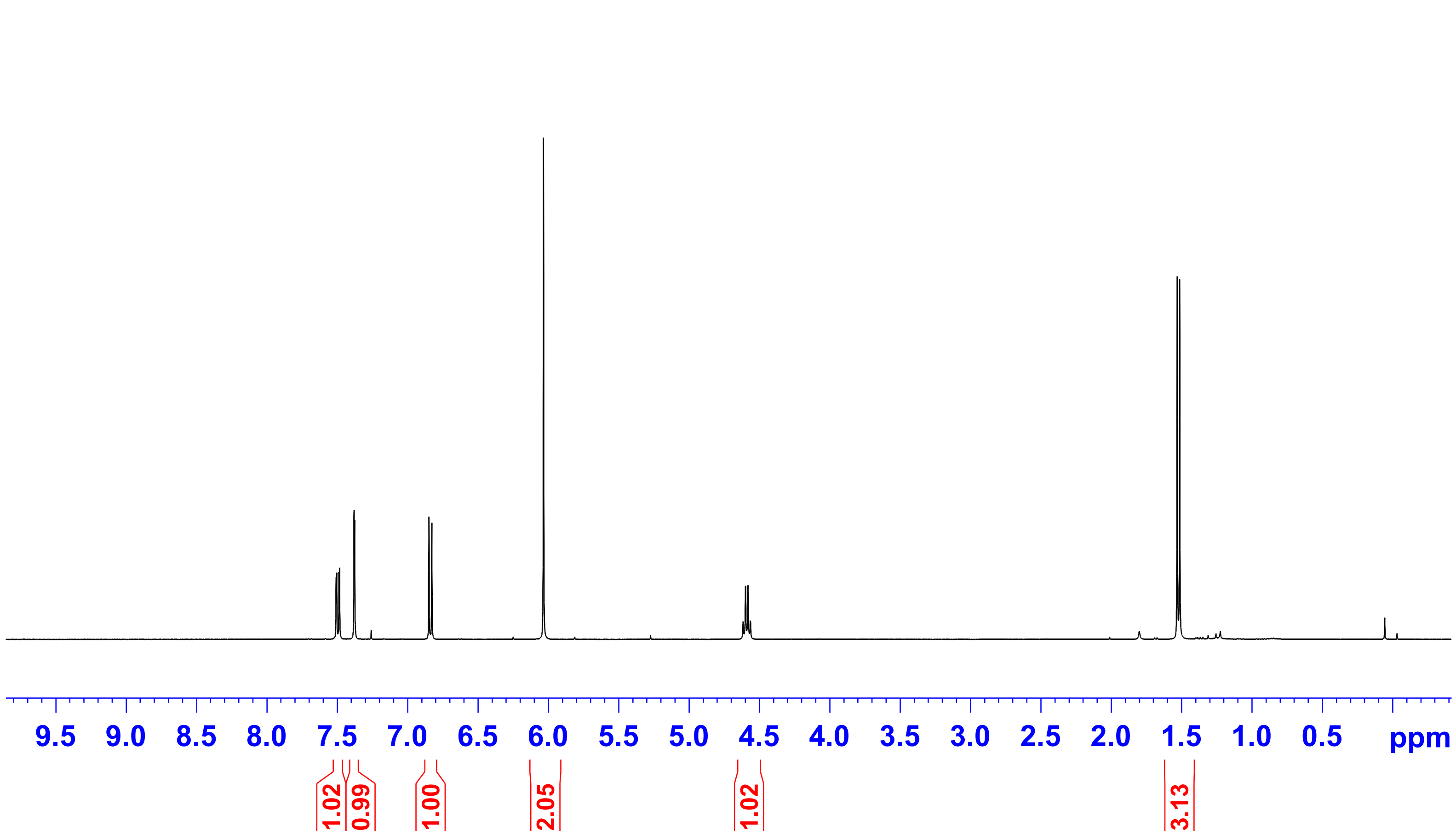

NAME	lzy-11-42-Z
EXPNO	1
PROCNO	1
Date_	20221108
Time	22.01
INSTRUM	spect
PROBHD	5 mm DUL 13C-1
PULPROG	zg30
TD	65536
SOLVENT	CDCl3
NS	4
DS	0
SWH	8223.685 Hz
FIDRES	0.125483 Hz
AQ	3.9846387 sec
RG	128
DW	60.800 usec
DE	6.00 usec
TE	293.6 K
D1	1.00000000 sec
TD0	1

======== CHANNEL f1 ========

NUC1	1H
P1	15.80 usec
PL1	-1.00 dB
PL1W	12.17476940 W
SFO1	400.1324710 MHz
SI	32768
SF	400.1300099 MHz
WDW	EM
SSB	0
LB	0.30 Hz
GB	0
PC	1.00

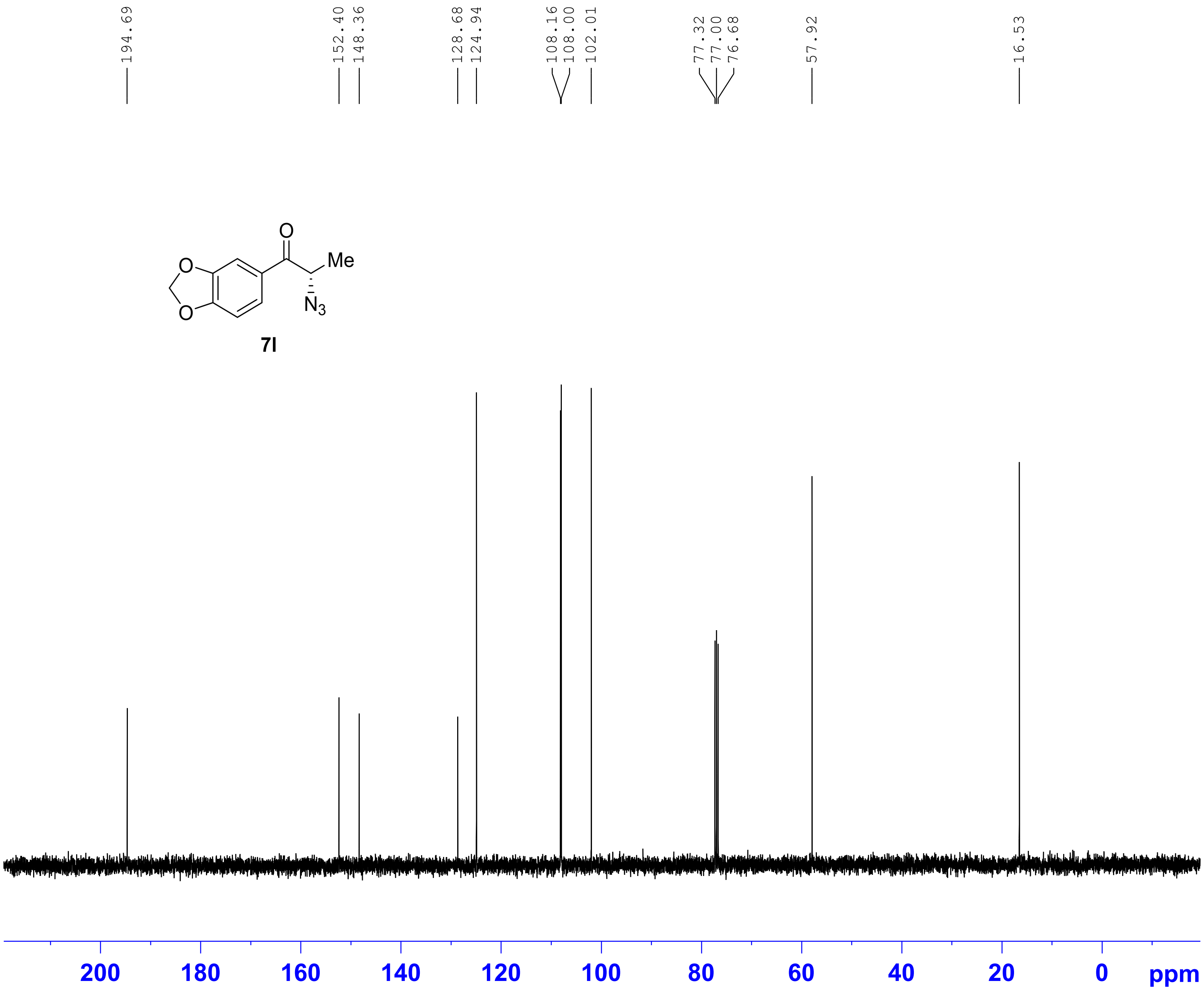

NAME	lzy-11-42-Z-C
EXPNO	1
PROCNO	1
Date_	20221108
Time_	22.04
INSTRUM	spect
PROBHD	5 mm DUL 13C-1
PULPROG	zgpg30
TD	65536
SOLVENT	CDCl3
NS	35
DS	0
SWH	24038.461 Hz
FIDRES	0.366798 Hz
AQ	1.3631988 sec
RG	2050
DW	20.800 usec
DE	6.00 usec
TE	293.9 K
D1	2.00000000 sec
D11	0.03000000 sec
TD0	1

======== CHANNEL f1 ========

NUC1	13C
P1	40.00 usec
PL1	-3.00 dB
PL1W	60.64365387 W
SFO1	100.6228298 MHz

======== CHANNEL f2 ========

CPDPRG2	waltz16
NUC2	1H
PCPD2	80.00 usec
PL2	-1.00 dB
PL12	14.39 dB
PL13	18.00 dB
PL2W	12.17476940 W
PL12W	0.35193357 W
PL13W	0.15327126 W
SFO2	400.1316005 MHz
SI	32768
SF	100.6127824 MHz
WDW	EM
SSB	0
LB	1.00 Hz
GB	0
PC	1.40

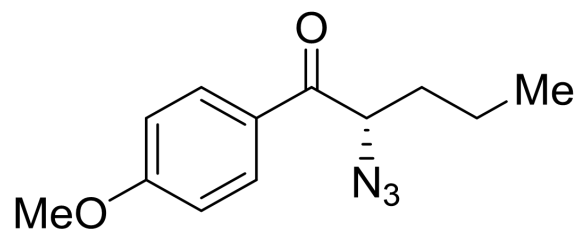

7m

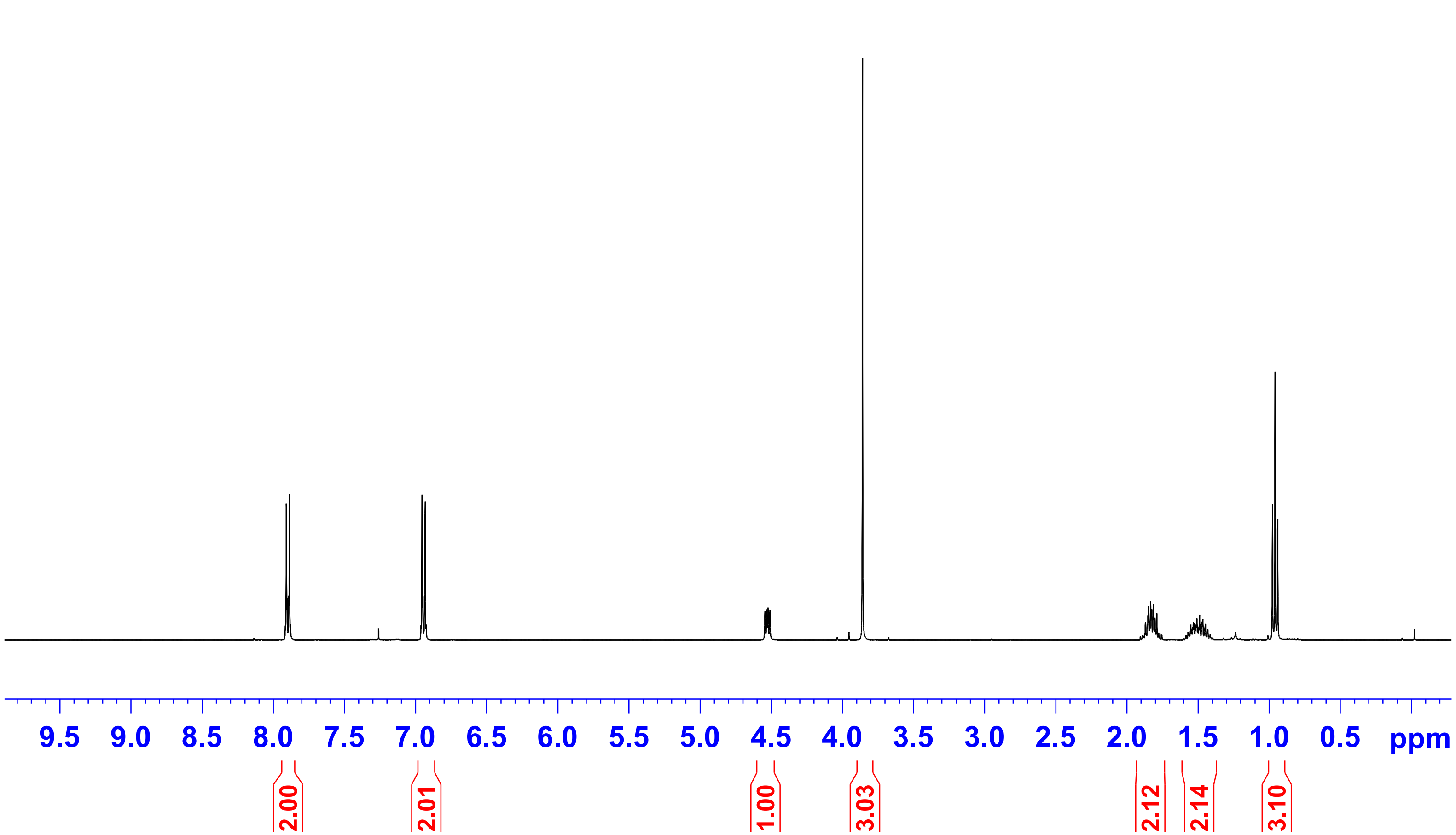

NAME	lzy-11-58
EXPNO	1
PROCNO	1
Date_	20220910
Time	14.39
INSTRUM	spect
PROBHD	5 mm DUL 13C-1
PULPROG	zg30
TD	65536
SOLVENT	CDCl3
NS	3
DS	0
SWH	8223.685 Hz
FIDRES	0.125483 Hz
AQ	3.9846387 sec
RG	64
DW	60.800 usec
DE	6.00 usec
TE	292.6 K
D1	1.00000000 sec
TD0	1

======== CHANNEL f1 ========

NUC1	1H
P1	15.80 usec
PL1	-1.00 dB
PL1W	12.17476940 W
SFO1	400.1324710 MHz
SI	32768
SF	400.1300095 MHz
WDW	EM
SSB	0
LB	0.30 Hz
GB	0
PC	1.00

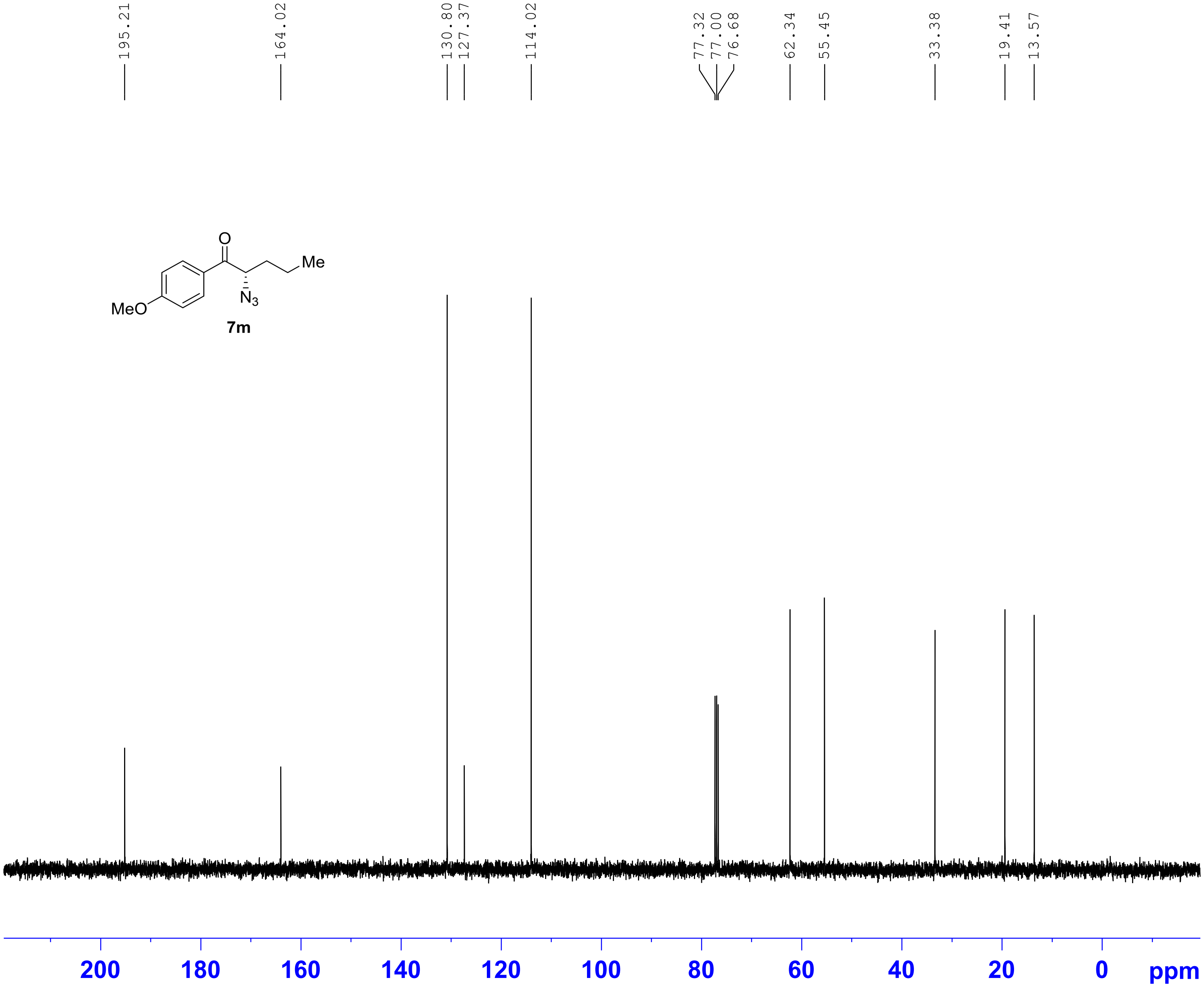

NAME	lzy-11-58-C	
EXPNO	1	
PROCNO	1	
Date_	20220910	
Time	14.41	
INSTRUM	spect	
PROBHD	5 mm DUL 13C-1	
PULPROG	zgpg30	
TD	65536	
SOLVENT	CDCl3	
NS	19	
DS	0	
SWH	24038.461	Hz
FIDRES	0.366798	Hz
AQ	1.3631988	sec
RG	2050	
DW	20.800	usec
DE	6.00	usec
TE	292.8	K
D1	2.00000000	sec
D11	0.03000000	sec
TD0	1	
======== CHANNEL f1 ========		
NUC1	13C	
P1	40.00	usec
PL1	-3.00	dB
PL1W	60.64365387	W
SFO1	100.6228298	MHz
======== CHANNEL f2 ========		
CPDPRG2	waltz16	
NUC2	1H	
PCPD2	80.00	usec
PL2	-1.00	dB
PL12	14.39	dB
PL13	18.00	dB
PL2W	12.17476940	W
PL12W	0.35193357	W
PL13W	0.15327126	W
SFO2	400.1316005	MHz
SI	32768	
SF	100.6127810	MHz
WDW	EM	
SSB	0	
LB	1.00	Hz
GB	0	
PC	1.40	

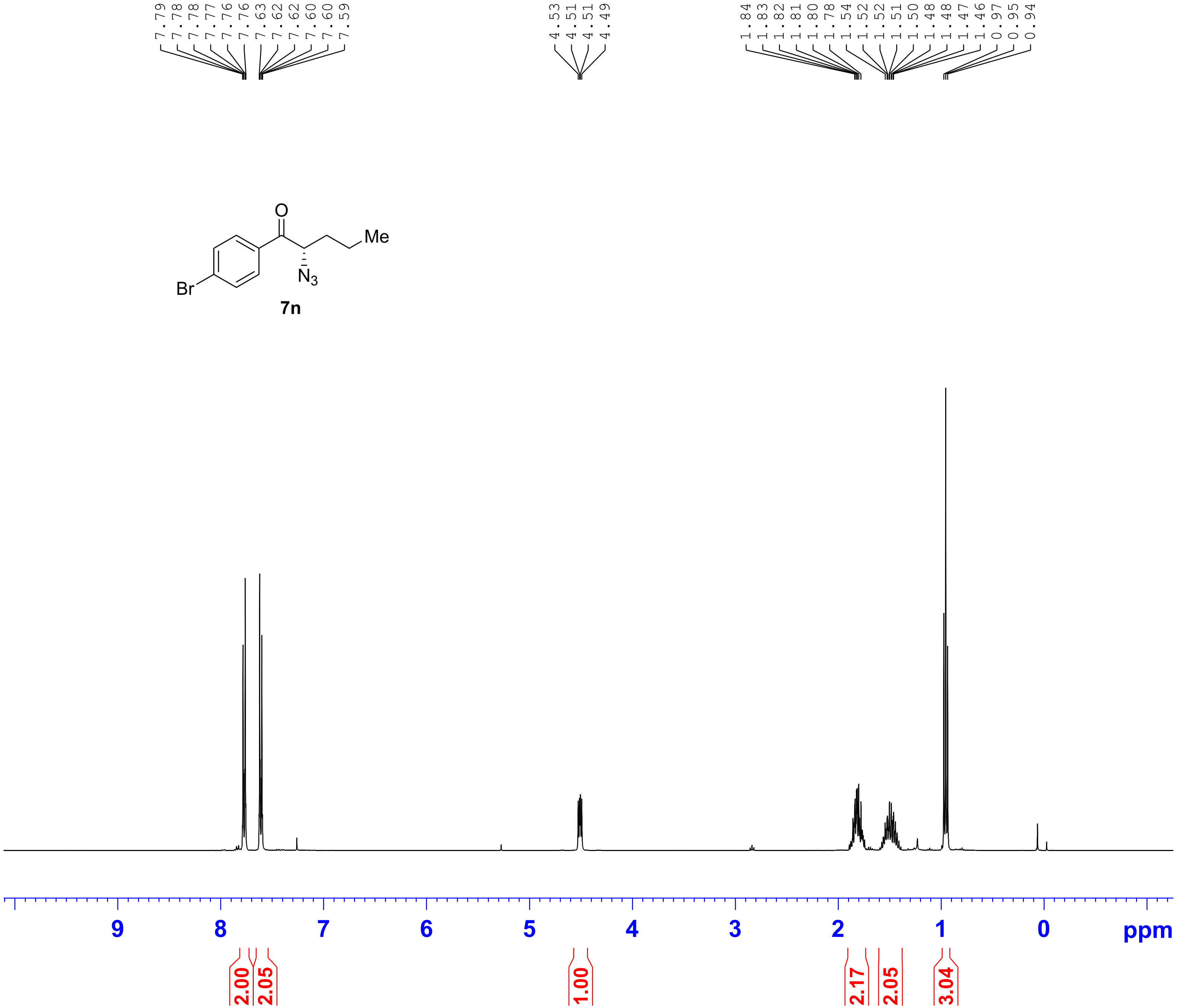

NAME	lzy-11-42-V
EXPNO	1
PROCNO	1
Date_	20221109
Time	10.11
INSTRUM	spect
PROBHD	5 mm DUL 13C-1
PULPROG	zg30
TD	65536
SOLVENT	CDCl3
NS	1
DS	0
SWH	8223.685 Hz
FIDRES	0.125483 Hz
AQ	3.9846387 sec
RG	64
DW	60.800 usec
DE	6.00 usec
TE	293.8 K
D1	1.00000000 sec
TD0	1

======== CHANNEL f1 ========

NUC1	1H
P1	15.80 usec
PL1	-1.00 dB
PL1W	12.17476940 W
SFO1	400.1324710 MHz
SI	32768
SF	400.1300100 MHz
WDW	EM
SSB	0
LB	0.30 Hz
GB	0
PC	1.00

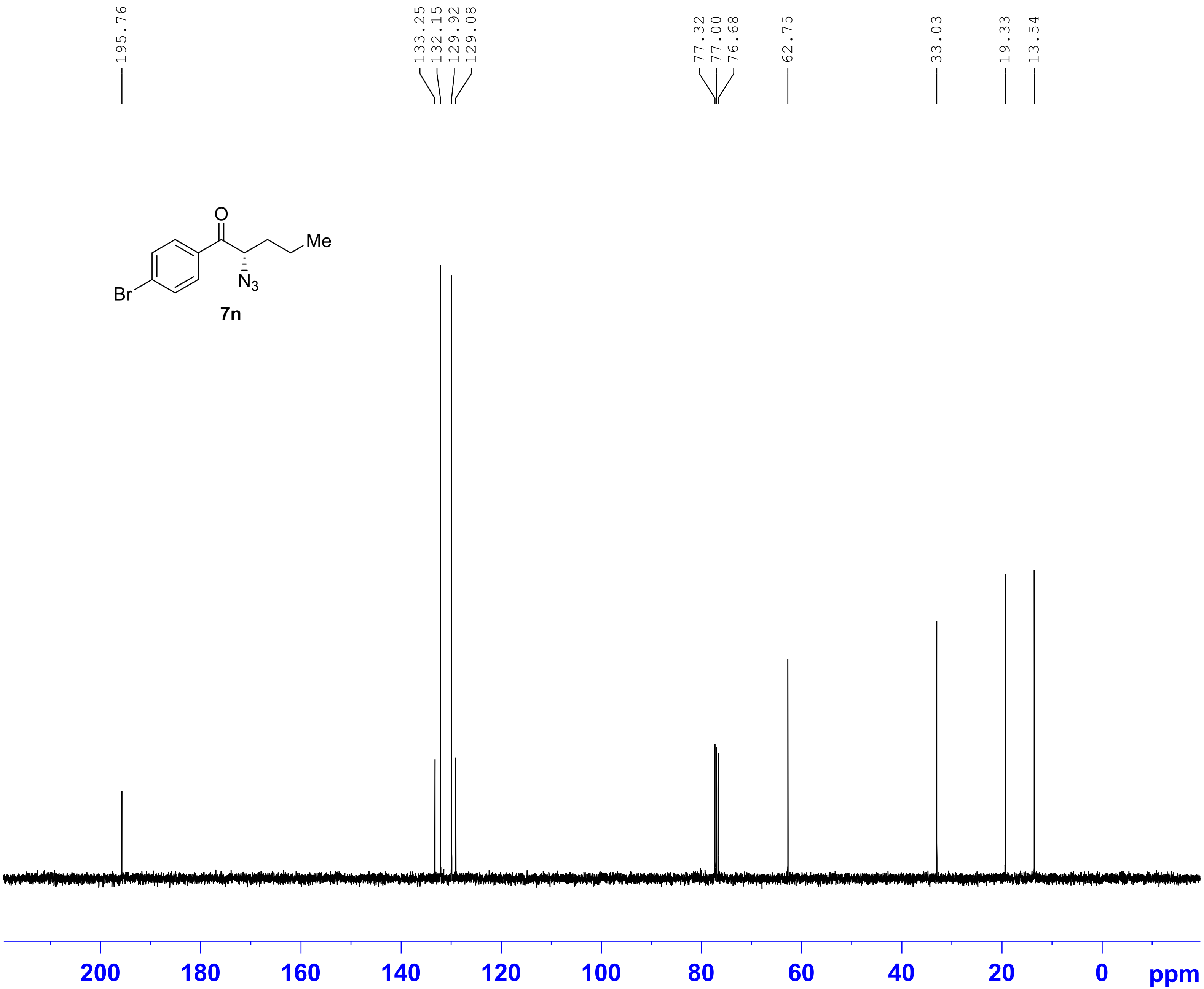

NAME lzy-11-42-V-C
EXPNO 1
PROCNO 1
Date_ 20221109
Time_ 10.13
INSTRUM spect
PROBHD 5 mm DUL 13C-1
PULPROG zgpg30
TD 65536
SOLVENT CDCl3
NS 30
DS 0
SWH 24038.461 Hz
FIDRES 0.366798 Hz
AQ 1.3631988 sec
RG 2050
DW 20.800 usec
DE 6.00 usec
TE 294.1 K
D1 2.00000000 sec
D11 0.03000000 sec
TD0 1

======== CHANNEL f1 ========
NUC1 13C
P1 40.00 usec
PL1 -3.00 dB
PL1W 60.64365387 W
SFO1 100.6228298 MHz

======== CHANNEL f2 ========
CPDPRG2 waltz16
NUC2 1H
PCPD2 80.00 usec
PL2 -1.00 dB
PL12 14.39 dB
PL13 18.00 dB
PL2W 12.17476940 W
PL12W 0.35193357 W
PL13W 0.15327126 W
SFO2 400.1316005 MHz
SI 32768
SF 100.6127816 MHz
WDW EM
SSB 0
LB 1.00 Hz
GB 0
PC 1.40

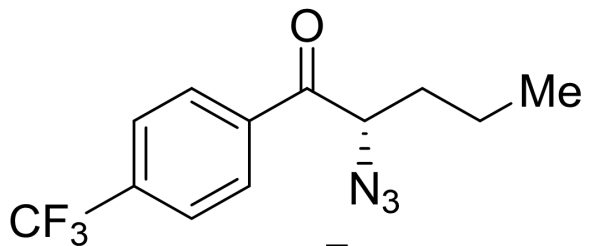

7o

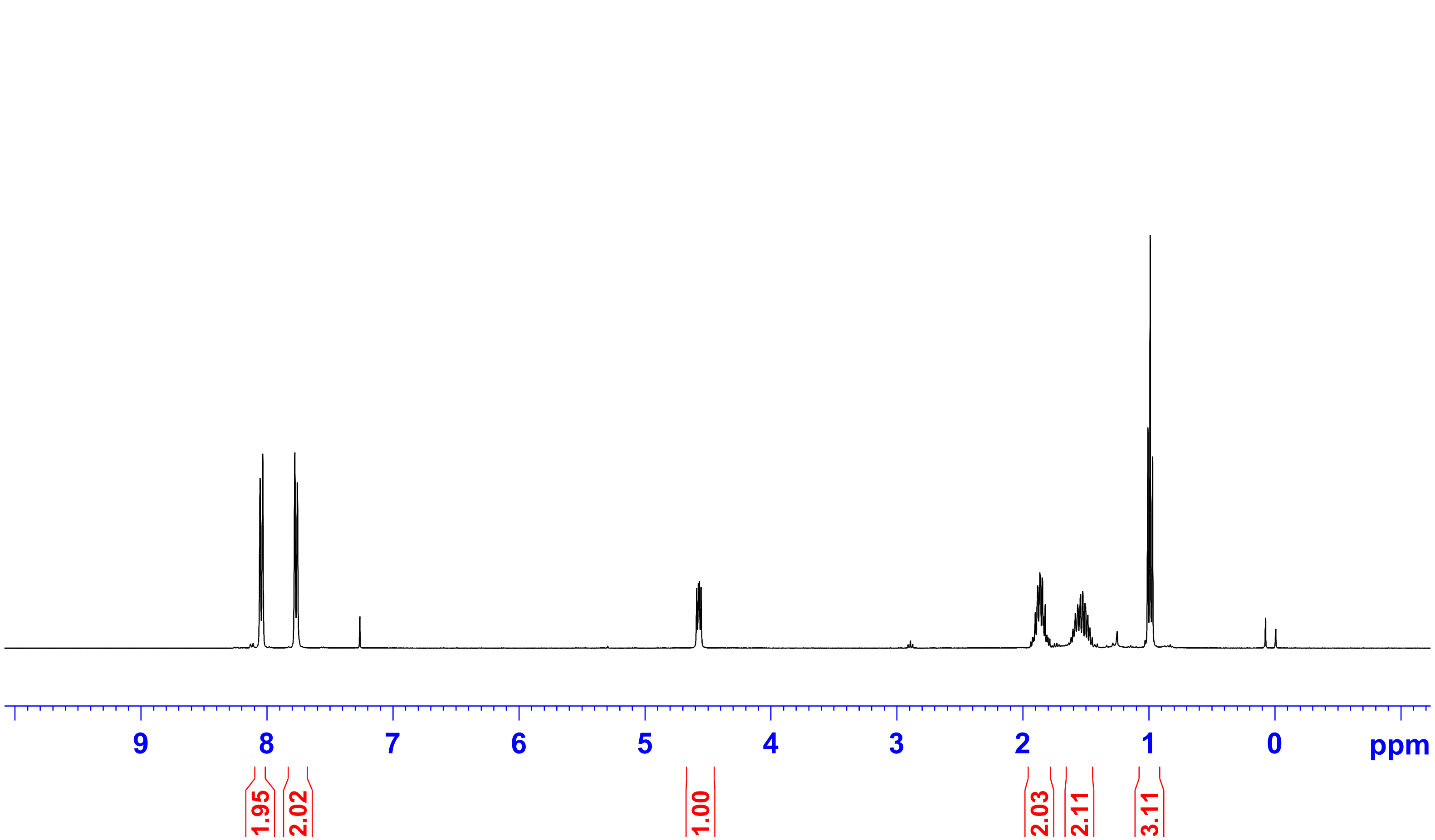

NAME	lzy-11--42-AA-Re
EXPNO	1
PROCNO	1
Date_	20221028
Time	21.14 h
INSTRUM	AvanceNeo 400MHz
PROBHD	Z163739_0629 (
PULPROG	zg30
TD	65536
SOLVENT	CDCl3
NS	6
DS	2
SWH	8196.722 Hz
FIDRES	0.250144 Hz
AQ	3.9977460 sec
RG	101
DW	61.000 usec
DE	13.89 usec
TE	296.6 K
D1	1.00000000 sec
TD0	1
SFO1	400.1824711 MHz
NUC1	1H
P0	2.67 usec
P1	8.00 usec
SI	65536
SF	400.1800073 MHz
WDW	EM
SSB	0
LB	0.30 Hz
GB	0
PC	1.00

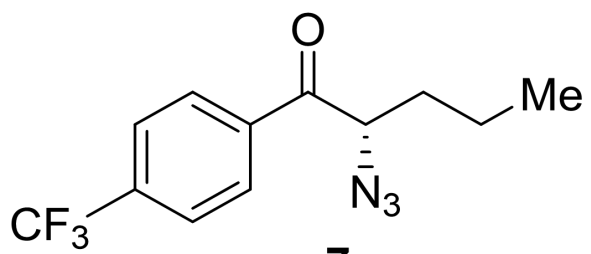

7o

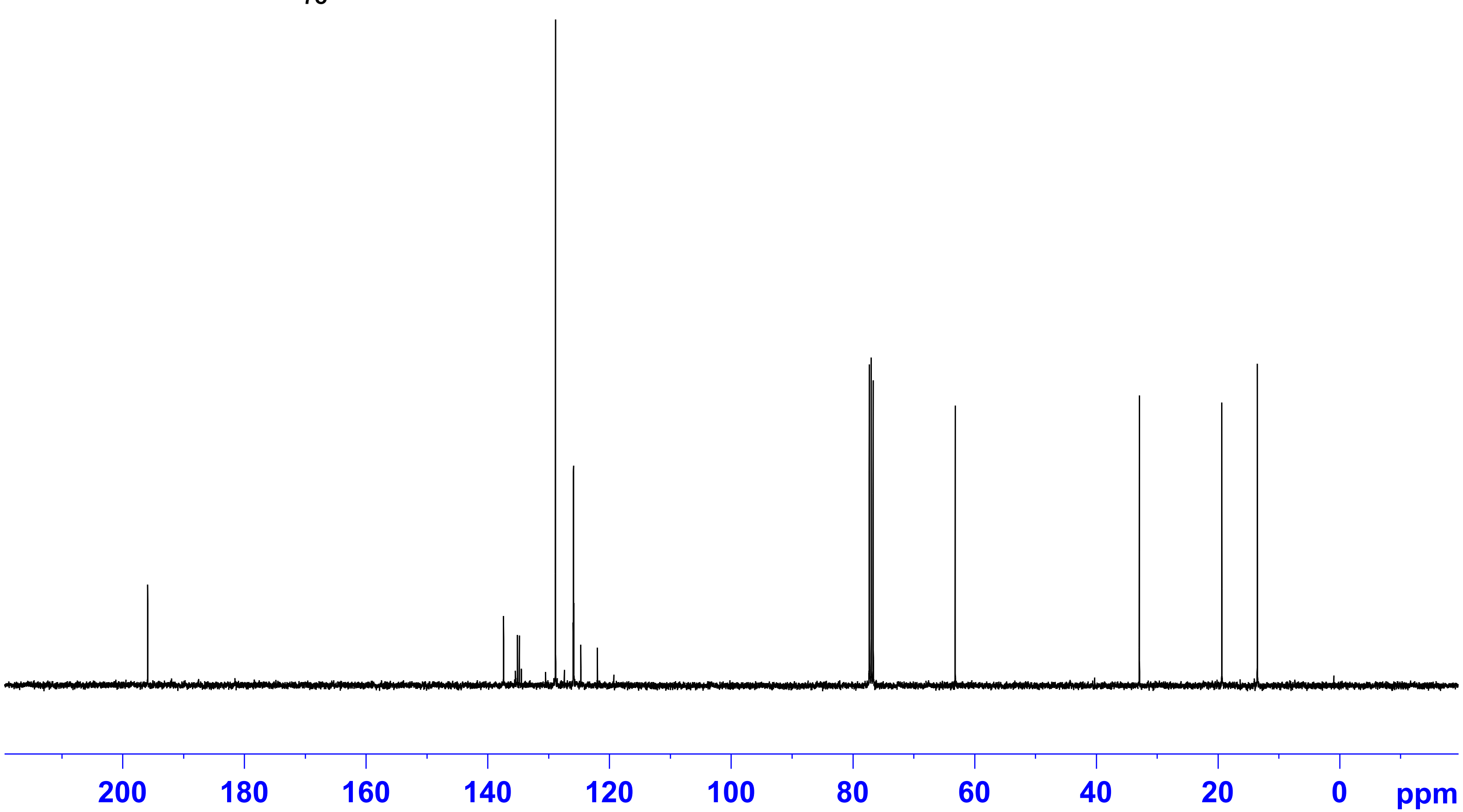

NAME	lzy-11-42-AA-C	
EXPNO	1	
PROCNO	1	
Date_	20221028	
Time	19.36	
INSTRUM	spect	
PROBHD	5 mm PABBO BB/	
PULPROG	zgpg30	
TD	65536	
SOLVENT	CDCl3	
NS	96	
DS	2	
SWH	24038.461	Hz
FIDRES	0.366798	Hz
AQ	1.3631988	sec
RG	196.92	
DW	20.800	usec
DE	6.50	usec
TE	296.4	K
D1	2.00000000	sec
D11	0.03000000	sec
TD0	1	

======== CHANNEL f1 ========

SFO1	100.6228298	MHz
NUC1	13C	
P1	9.70	usec
SI	32768	
SF	100.6127729	MHz
WDW	EM	
SSB	0	
LB	1.00	Hz
GB	0	
PC	1.40	

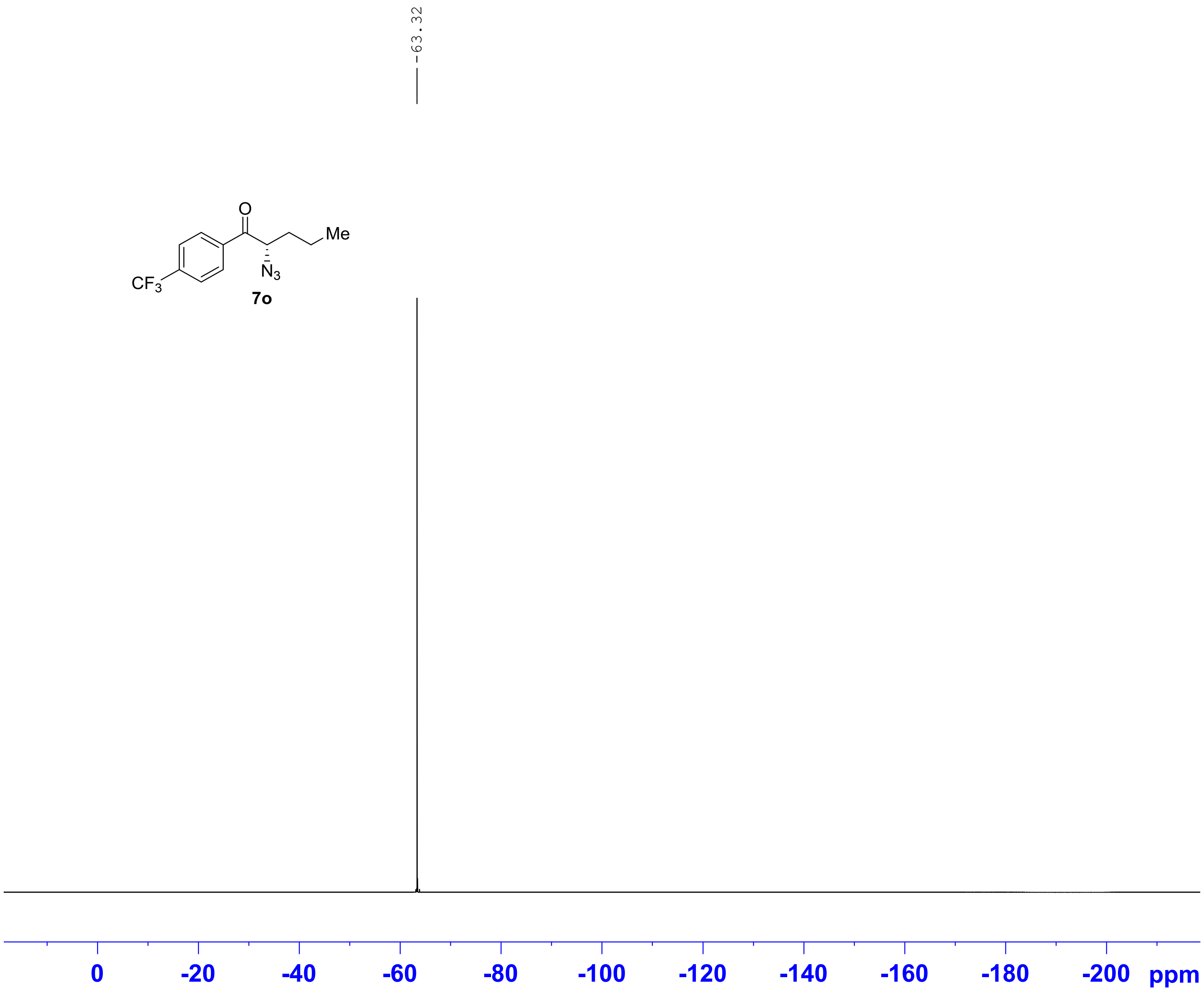

NAME	lzy-11-42-AA-F
EXPNO	1
PROCNO	1
Date_	20221028
Time_	19.43
INSTRUM	spect
PROBHD	5 mm PABBO BB/
PULPROG	zgflqn
TD	131072
SOLVENT	CDCl3
NS	9
DS	4
SWH	89285.711 Hz
FIDRES	0.681196 Hz
AQ	0.7340532 sec
RG	196.92
DW	5.600 usec
DE	6.50 usec
TE	296.5 K
D1	1.00000000 sec
TD0	1
======== CHANNEL f1 ========	
SFO1	376.4607164 MHz
NUC1	19F
P1	14.70 usec
SI	65536
SF	376.4983660 MHz
WDW	EM
SSB	0
LB	0.30 Hz
GB	0
PC	1.00

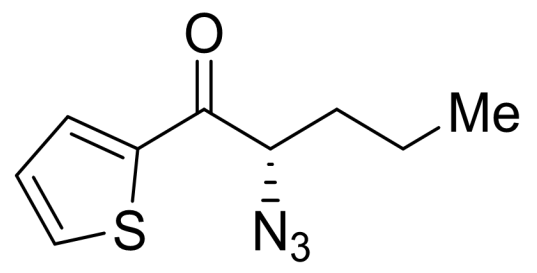

7p

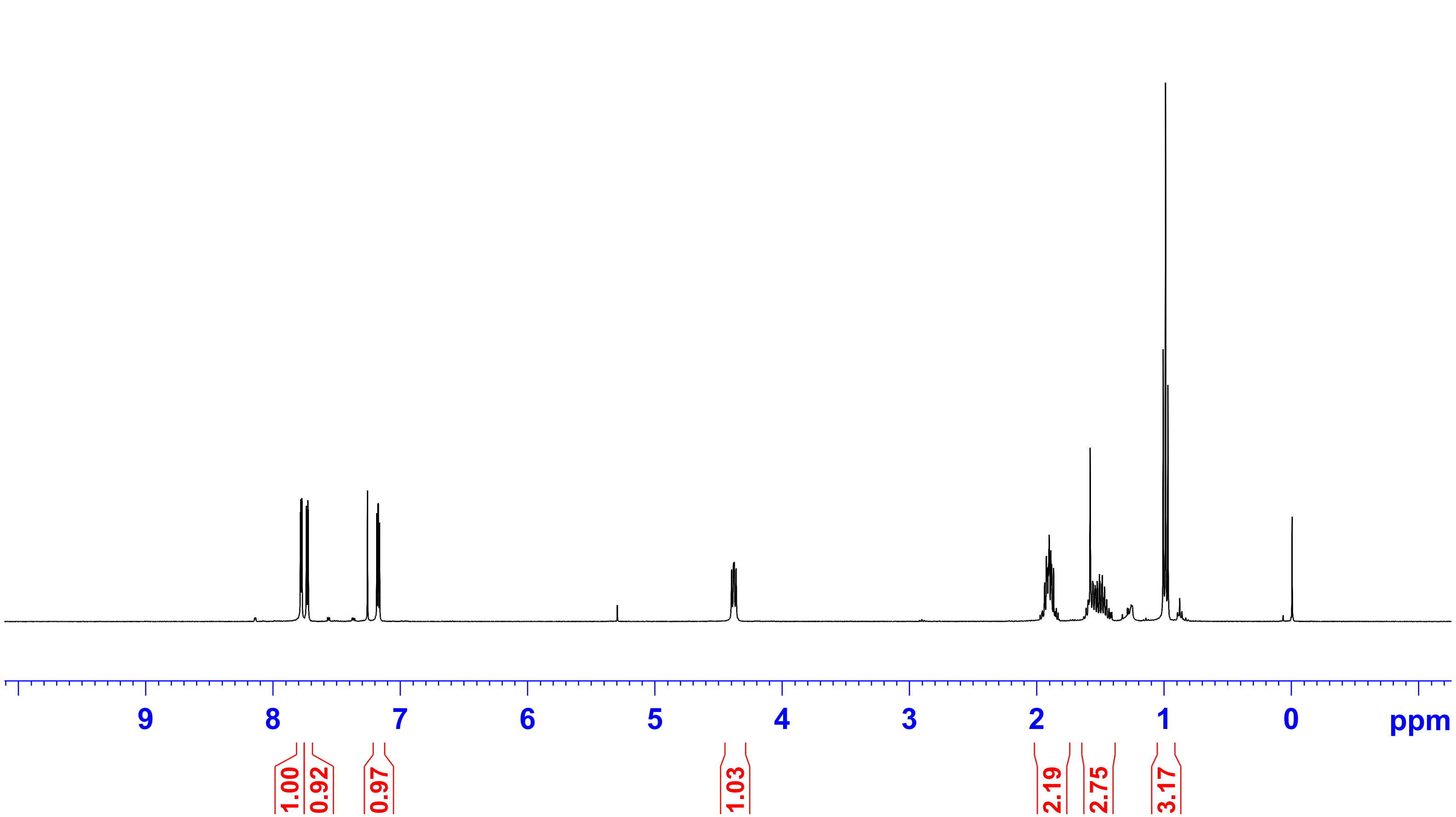

NAME	lzy-11-42-S
EXPNO	1
PROCNO	1
Date_	20221102
Time_	11.14
INSTRUM	spect
PROBHD	5 mm PABBO BB/
PULPROG	zg30
TD	65536
SOLVENT	CDCl3
NS	6
DS	2
SWH	8012.820 Hz
FIDRES	0.122266 Hz
AQ	4.0894966 sec
RG	126.97
DW	62.400 usec
DE	6.50 usec
TE	296.1 K
D1	1.00000000 sec
TD0	1

======== CHANNEL f1 ========

SFO1	400.1324710 MHz
NUC1	1H
P1	14.50 usec
SI	65536
SF	400.1300107 MHz
WDW	EM
SSB	0
LB	0.30 Hz
GB	0
PC	1.00

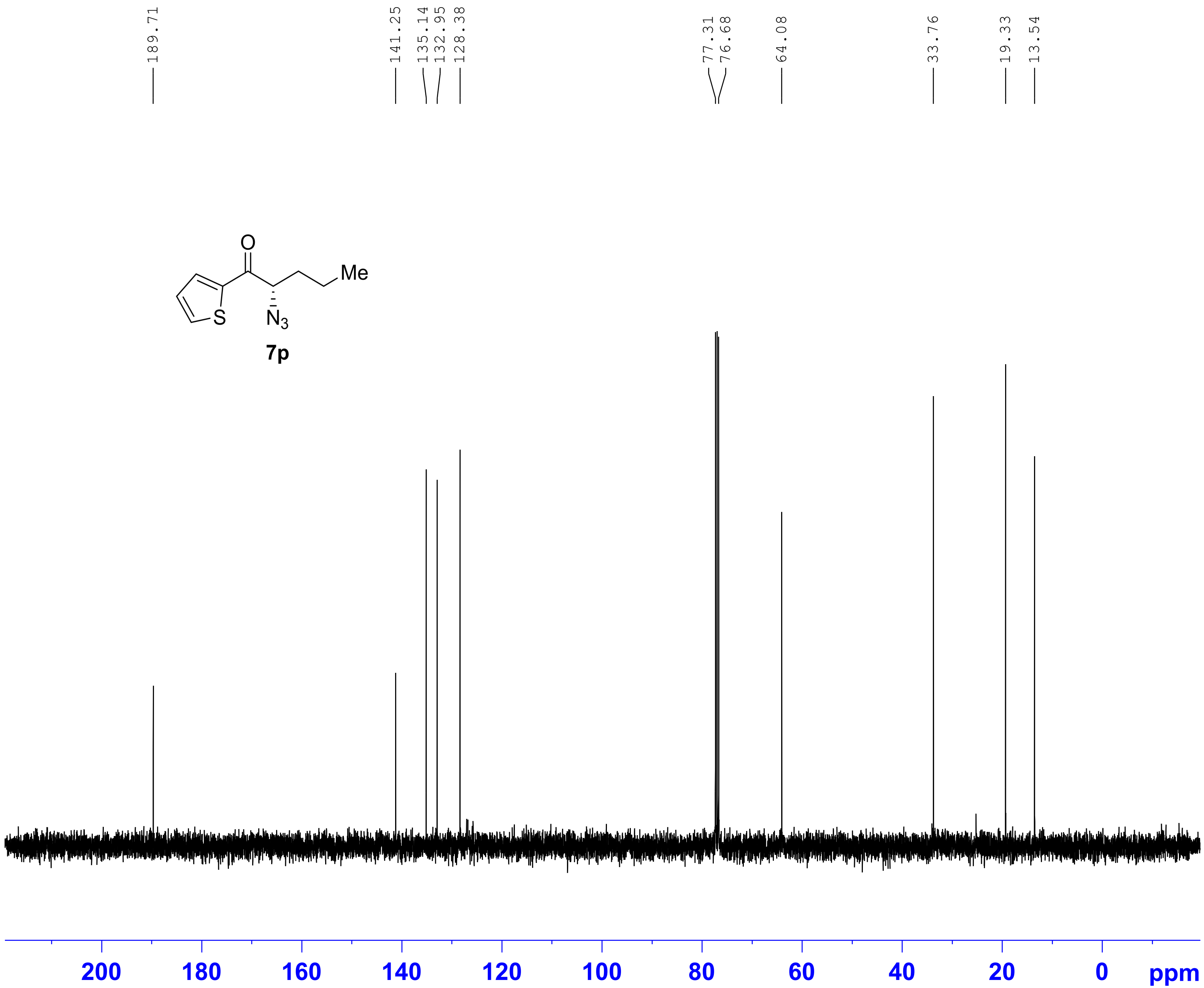

NAME	lzy-11-42-S-C	
EXPNO	1	
PROCNO	1	
Date_	20221027	
Time	22.47	
INSTRUM	spect	
PROBHD	5 mm PABBO BB/	
PULPROG	zgpg30	
TD	65536	
SOLVENT	CDCl3	
NS	12	
DS	2	
SWH	24038.461	Hz
FIDRES	0.366798	Hz
AQ	1.3631988	sec
RG	196.92	
DW	20.800	usec
DE	6.50	usec
TE	296.7	K
D1	2.00000000	sec
D11	0.03000000	sec
TD0	1	

======== CHANNEL f1 ========

SFO1	100.6228298	MHz
NUC1	13C	
P1	9.70	usec
SI	32768	
SF	100.6127803	MHz
WDW	EM	
SSB	0	
LB	1.00	Hz
GB	0	
PC	1.40	

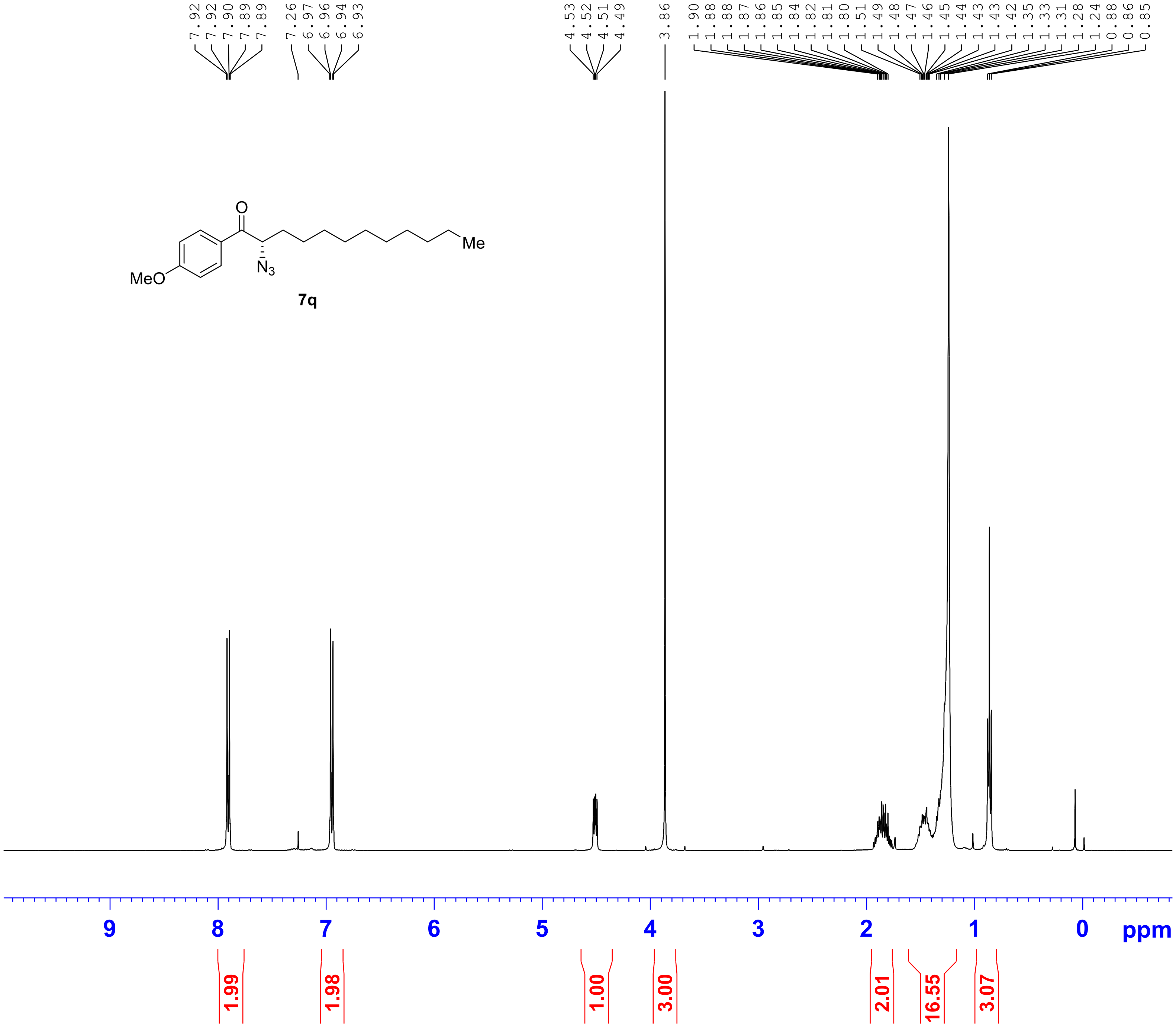

7q

NAME	lzy-11-42-P
EXPNO	1
PROCNO	1
Date_	20221102
Time	22.47
INSTRUM	spect
PROBHD	5 mm PABBO BB/
PULPROG	zg30
TD	65536
SOLVENT	CDCl3
NS	7
DS	2
SWH	8012.820 Hz
FIDRES	0.122266 Hz
AQ	4.0894966 sec
RG	17.38
DW	62.400 usec
DE	6.50 usec
TE	296.4 K
D1	1.00000000 sec
TD0	1

======== CHANNEL f1 ========

SFO1	400.1324710 MHz
NUC1	1H
P1	14.50 usec
SI	65536
SF	400.1300100 MHz
WDW	EM
SSB	0
LB	0.30 Hz
GB	0
PC	1.00

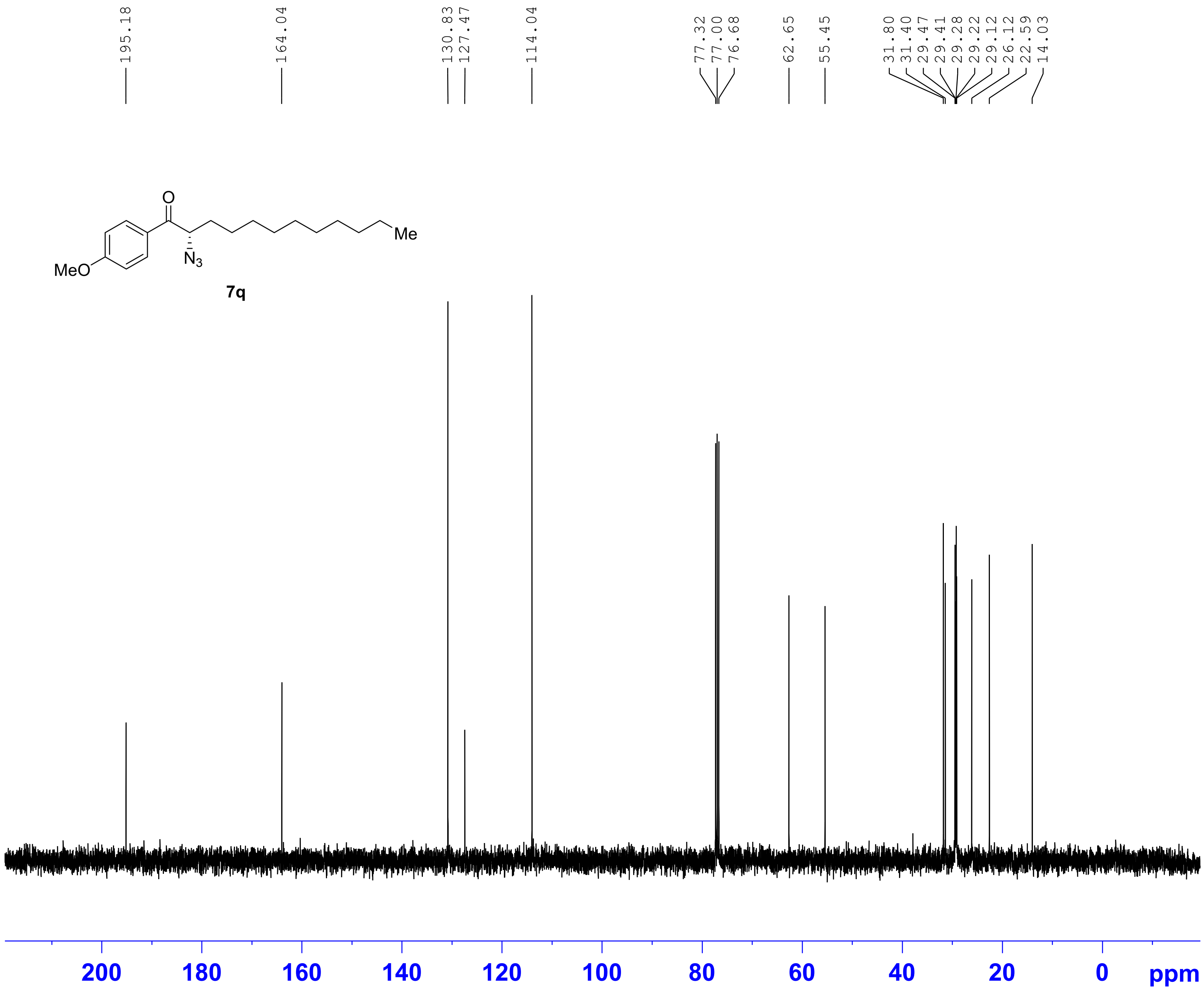

NAME	lzy-11-42-P-C
EXPNO	1
PROCNO	1
Date_	20221102
Time_	22.49
INSTRUM	spect
PROBHD	5 mm PABBO BB/
PULPROG	zgpg30
TD	65536
SOLVENT	CDCl3
NS	10
DS	2
SWH	24038.461 Hz
FIDRES	0.366798 Hz
AQ	1.3631988 sec
RG	196.92
DW	20.800 usec
DE	6.50 usec
TE	296.7 K
D1	2.00000000 sec
D11	0.03000000 sec
TD0	1

======== CHANNEL f1 ========

SFO1	100.6228298 MHz
NUC1	13C
P1	9.70 usec
SI	32768
SF	100.6127778 MHz
WDW	EM
SSB	0
LB	1.00 Hz
GB	0
PC	1.40

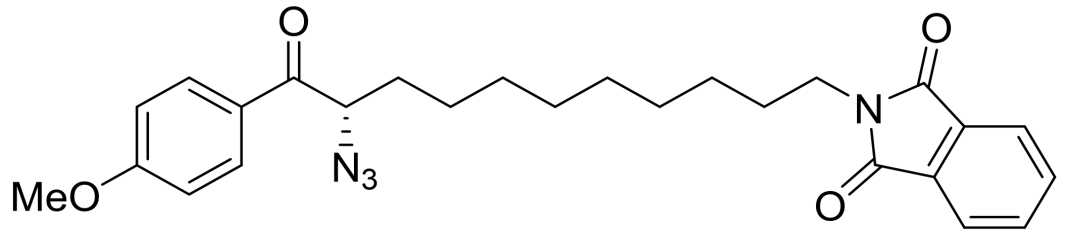

7r

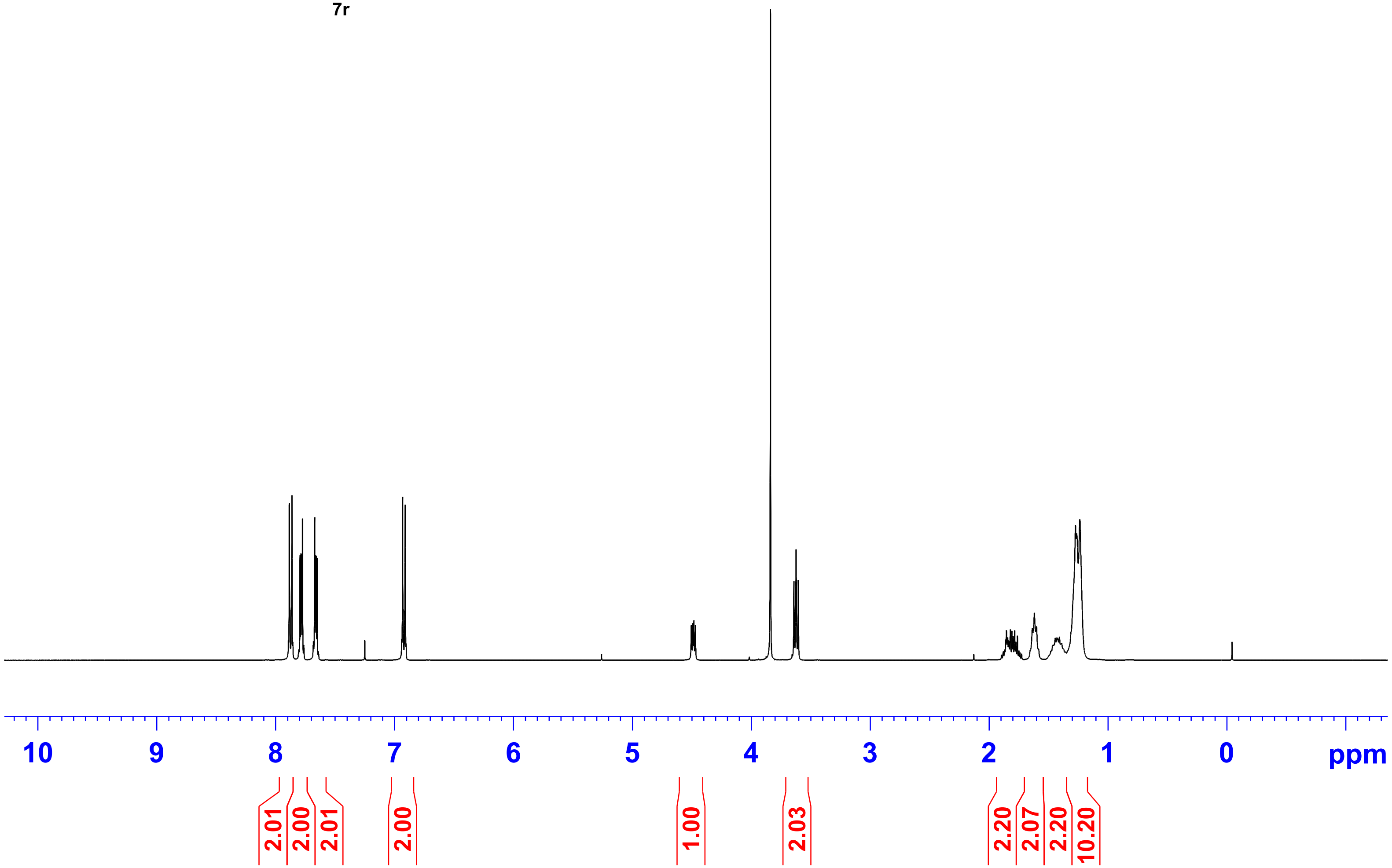

NAME	lzy-11-157
EXPNO	1
PROCNO	1
Date_	20221020
Time_	17.46
INSTRUM	spect
PROBHD	5 mm PABBO BB/
PULPROG	zg30
TD	65536
SOLVENT	CDCl3
NS	16
DS	2
SWH	8012.820 Hz
FIDRES	0.122266 Hz
AQ	4.0894966 sec
RG	22.47
DW	62.400 usec
DE	6.50 usec
TE	296.2 K
D1	1.00000000 sec
TD0	1

======== CHANNEL f1 ========

SFO1	400.1324710 MHz
NUC1	1H
P1	14.50 usec
SI	65536
SF	400.1300131 MHz
WDW	EM
SSB	0
LB	0.30 Hz
GB	0
PC	1.00

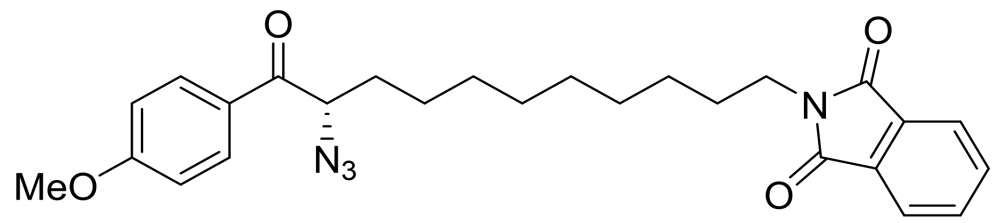

7r

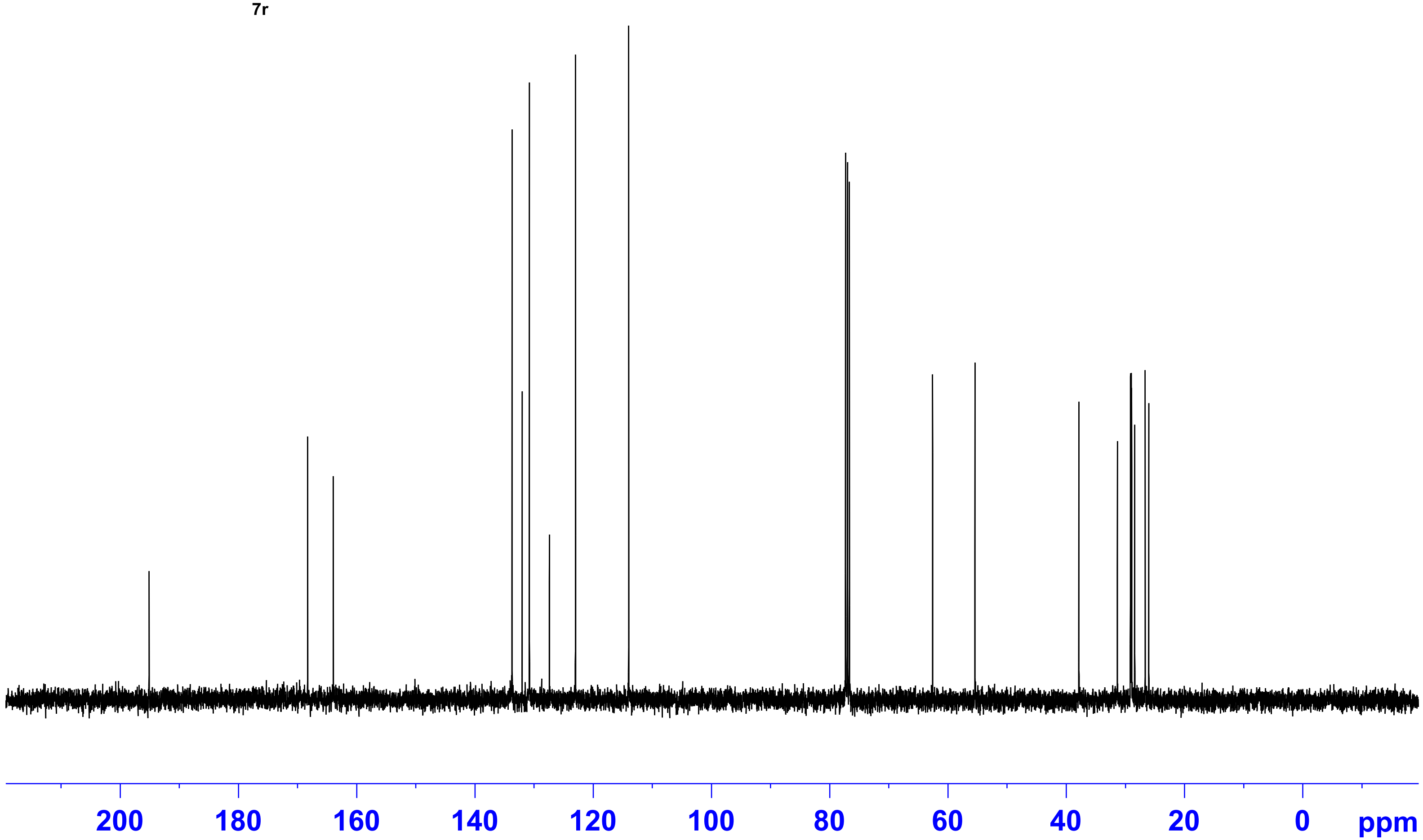

NAME	lzy-11-157-C
EXPNO	1
PROCNO	1
Date_	20221020
Time	17.49
INSTRUM	spect
PROBHD	5 mm PABBO BB/
PULPROG	zgpg30
TD	65536
SOLVENT	CDCl3
NS	32
DS	2
SWH	24038.461 Hz
FIDRES	0.366798 Hz
AQ	1.3631988 sec
RG	196.92
DW	20.800 usec
DE	6.50 usec
TE	296.9 K
D1	2.00000000 sec
D11	0.03000000 sec
TD0	1

======== CHANNEL f1 ========

SFO1	100.6228298 MHz
NUC1	13C
P1	9.70 usec
SI	32768
SF	100.6127808 MHz
WDW	EM
SSB	0
LB	1.00 Hz
GB	0
PC	1.40

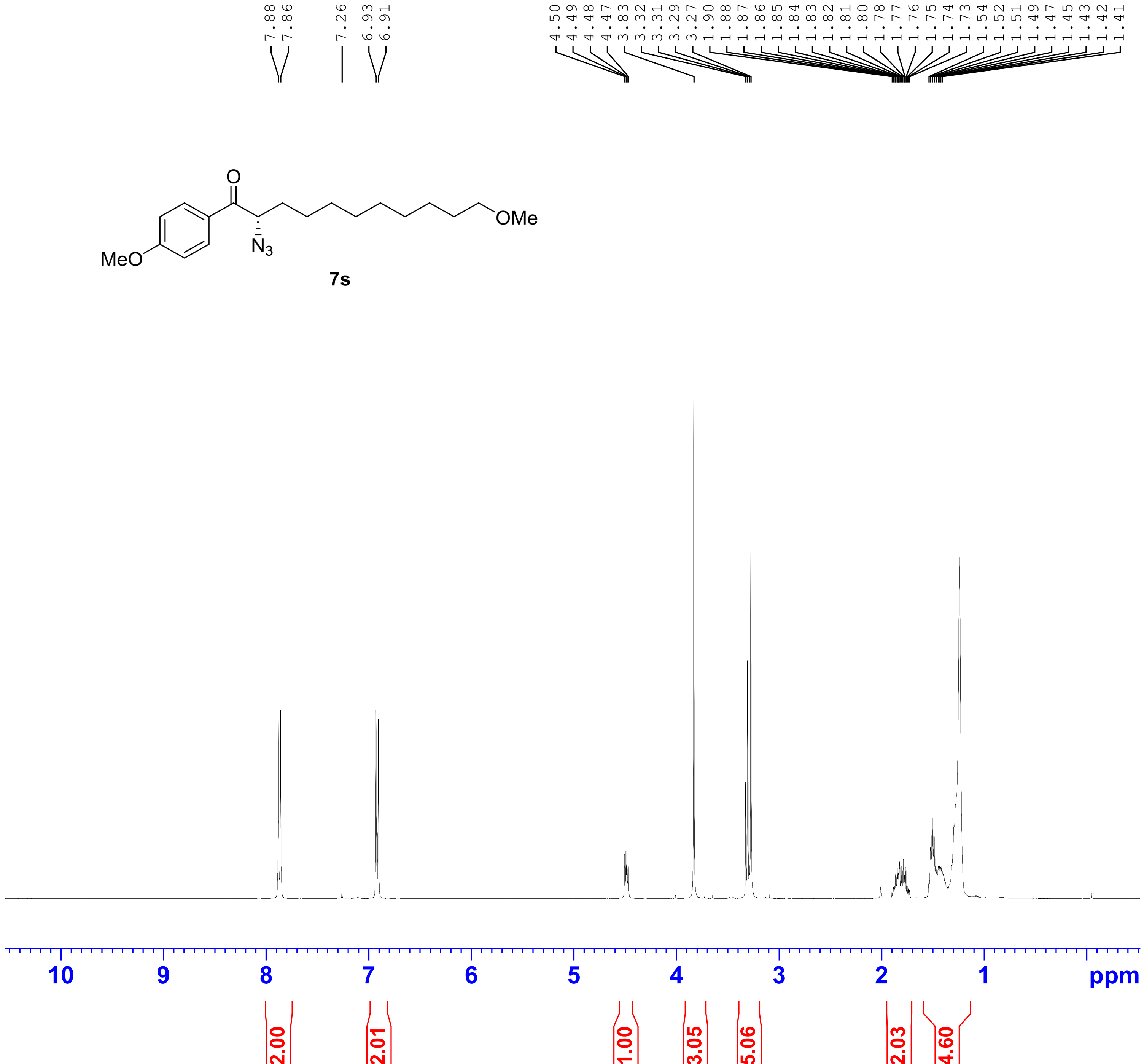

Current Data Parameters
NAME lzy-11-174
EXPNO 1
PROCNO 1

F2 - Acquisition Parameters
Date_ 20221104
Time 10.29
INSTRUM spect
PROBHD 5 mm PABBO BB/19F-1H/D Z-GRD Z108618/0582
PULPROG zg30
TD 65536
SOLVENT CDCl3
NS 8
DS 2
SWH 8012.820 Hz
FIDRES 0.122266 Hz
AQ 4.0894465 sec
RG 12.56
DW 62.400 usec
DE 6.50 usec
TE 296.3 K
D1 1.00000000 sec
TD0 1

======== CHANNEL f1 ========
SFO1 400.1324710 MHz
NUC1 1H
P1 14.50 usec
PLW1 11.99499989 W

F2 - Processing parameters
SI 65536
SF 400.1300097 MHz
WDW EM
SSB 0
LB 0.30 Hz
GB 0
PC 1.00

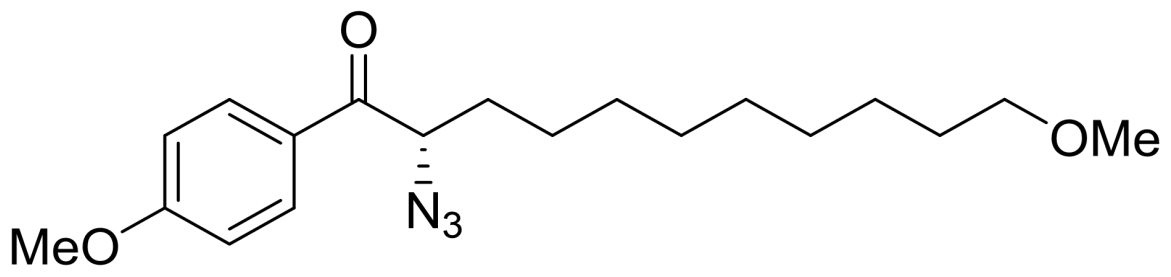

7s

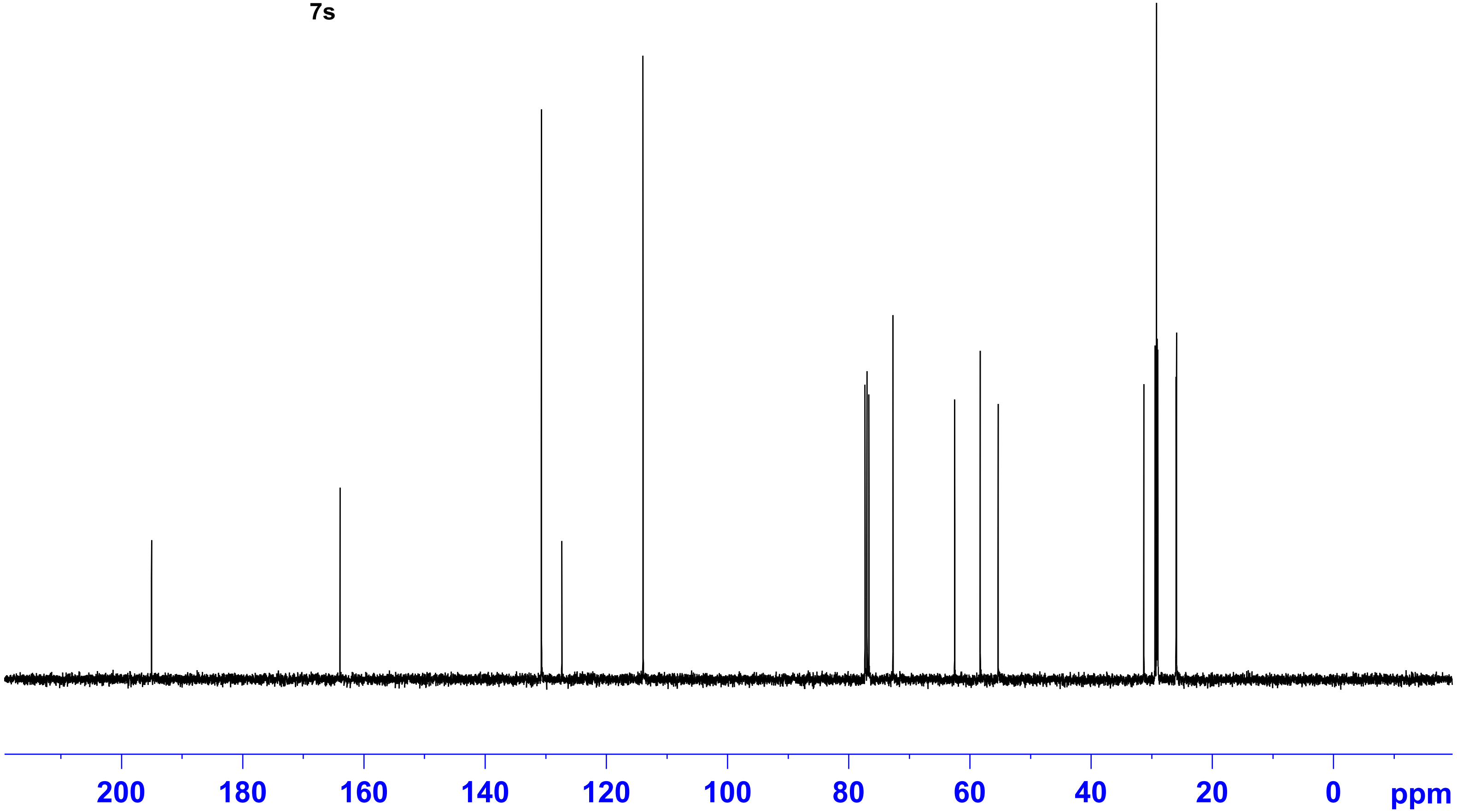

NAME	lzy-11-174-C
EXPNO	1
PROCNO	1
Date_	20221104
Time	10.33
INSTRUM	spect
PROBHD	5 mm PABBO BB/
PULPROG	zgpg30
TD	65536
SOLVENT	CDCl3
NS	36
DS	2
SWH	24038.461 Hz
FIDRES	0.366798 Hz
AQ	1.3631988 sec
RG	196.92
DW	20.800 usec
DE	6.50 usec
TE	297.1 K
D1	2.00000000 sec
D11	0.03000000 sec
TD0	1

======== CHANNEL f1 ========

SFO1	100.6228298 MHz
NUC1	13C
P1	9.70 usec
SI	32768
SF	100.6127845 MHz
WDW	EM
SSB	0
LB	1.00 Hz
GB	0
PC	1.40

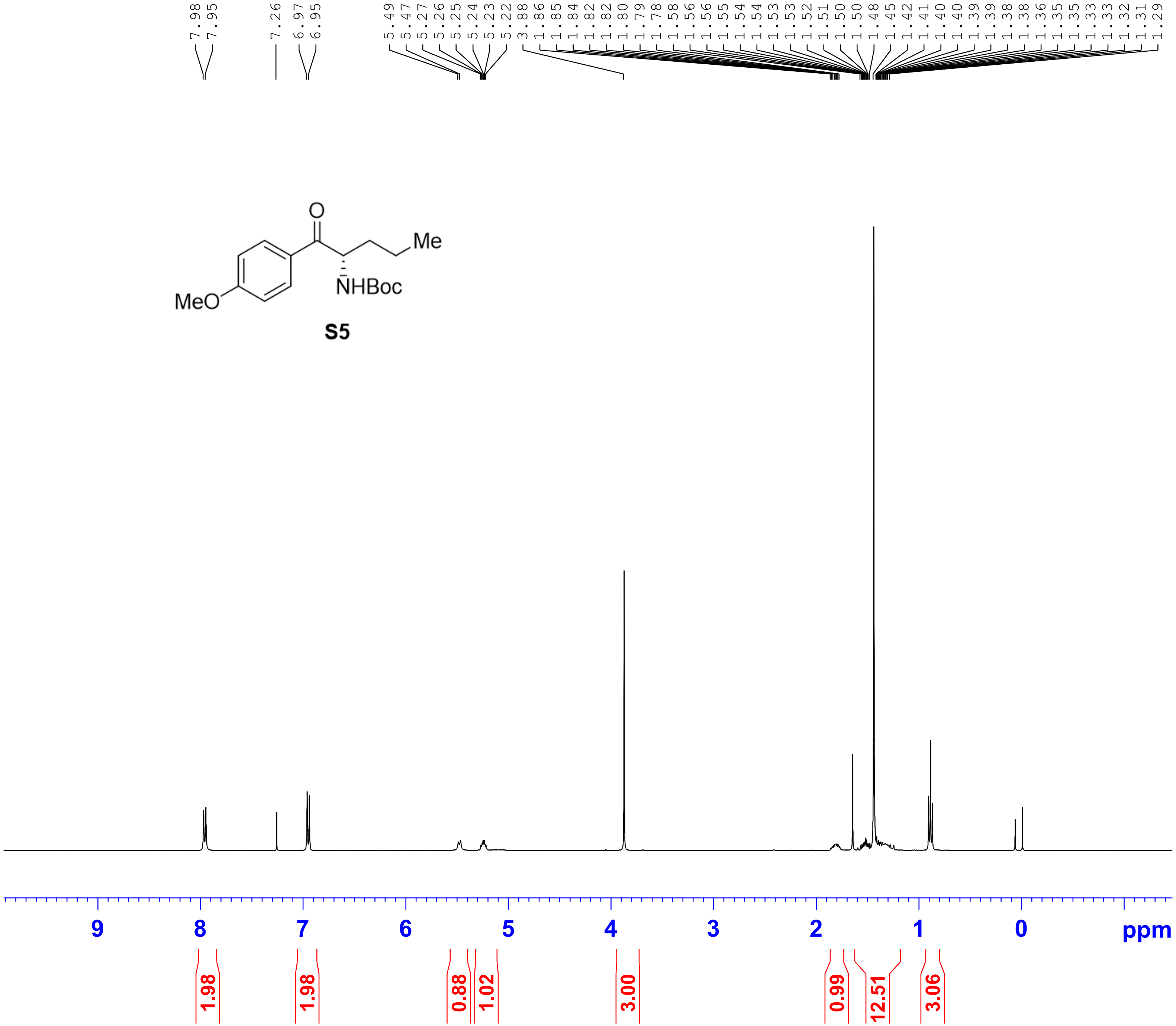

S5

NAME	lzy-13-19-T
EXPNO	2
PROCNO	1
Date_	20230503
Time	10.07
INSTRUM	spect
PROBHD	5 mm PABBO BB/
PULPROG	zg30
TD	65536
SOLVENT	CDCl3
NS	12
DS	2
SWH	8012.820 Hz
FIDRES	0.122266 Hz
AQ	4.0894966 sec
RG	82.92
DW	62.400 usec
DE	6.50 usec
TE	296.4 K
D1	1.00000000 sec
TD0	1

======== CHANNEL f1 ========

SFO1	400.1324710 MHz
NUC1	1H
P1	14.50 usec
SI	65536
SF	400.1300109 MHz
WDW	EM
SSB	0
LB	0.30 Hz
GB	0
PC	1.00

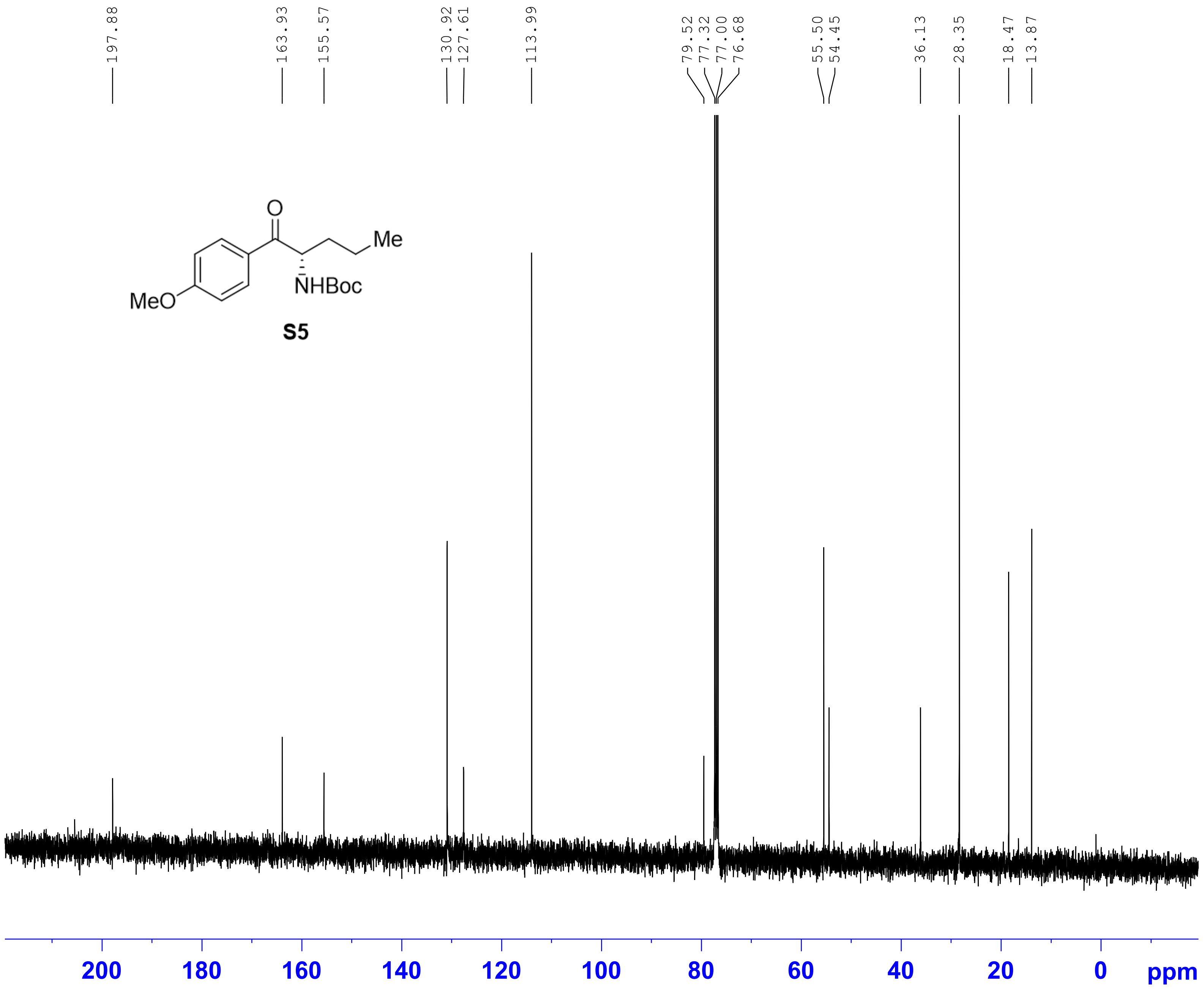

NAME	lzy-13-19-T-C
EXPNO	2
PROCNO	1
Date_	20230503
Time	10.08
INSTRUM	spect
PROBHD	5 mm PABBO BB/
PULPROG	zgpg30
TD	65536
SOLVENT	CDCl3
NS	400
DS	2
SWH	24038.461 Hz
FIDRES	0.366798 Hz
AQ	1.3631988 sec
RG	196.92
DW	20.800 usec
DE	6.50 usec
TE	296.8 K
D1	2.00000000 sec
D11	0.03000000 sec
TD0	1

======== CHANNEL f1 ========

SFO1	100.6228298 MHz
NUC1	13C
P1	9.70 usec
SI	32768
SF	100.6127714 MHz
WDW	EM
SSB	0
LB	1.00 Hz
GB	0
PC	1.40

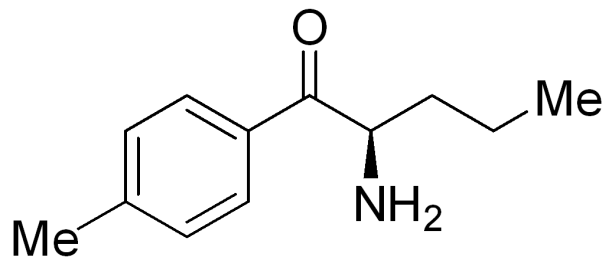

8a

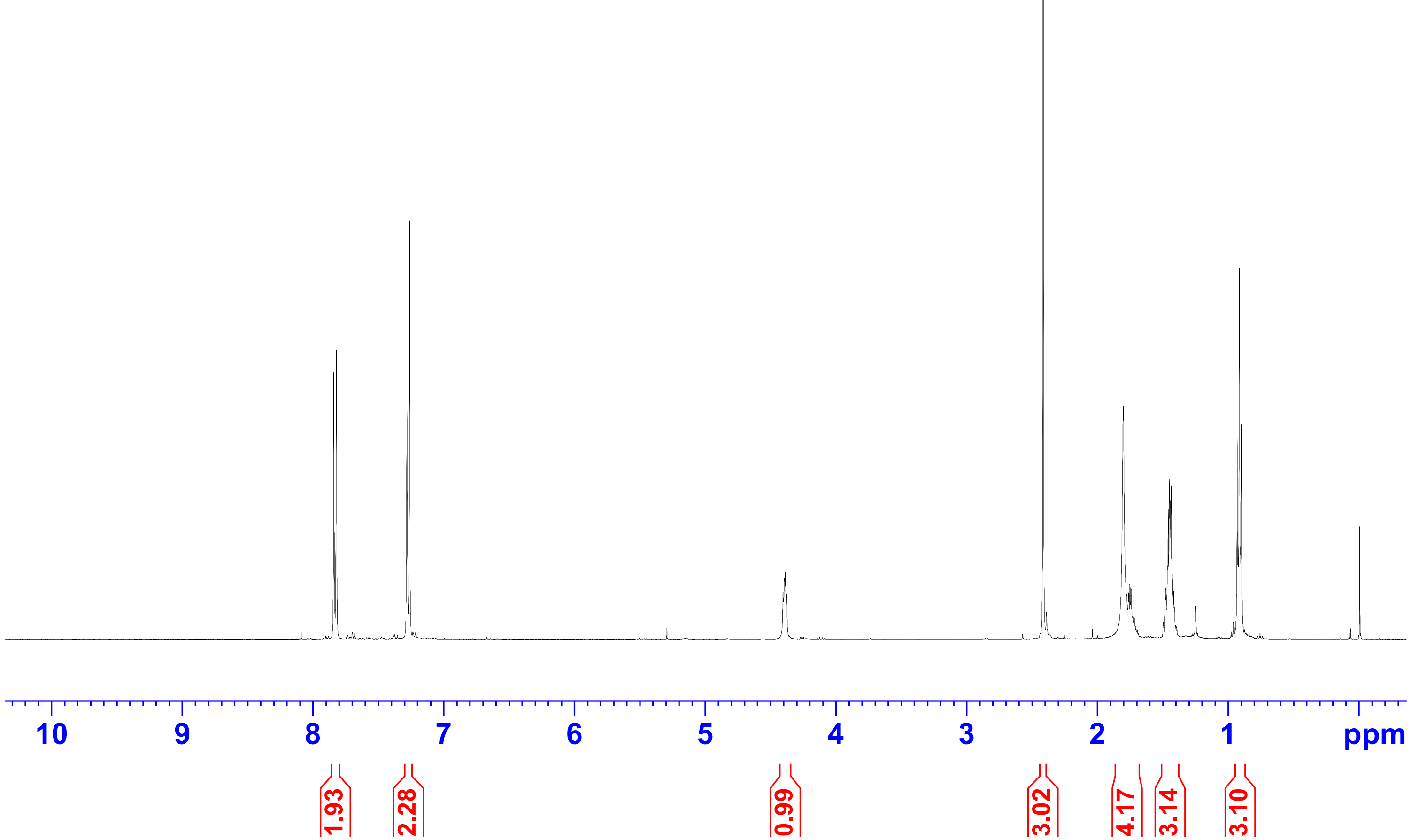


```
Current Data Parameters
NAME              luoy-2-136a
EXPNO                       1
PROCNO                      1

F2 - Acquisition Parameters
Date_                20260703
Time                    20.44
INSTRUM                 spect
PROBHD     5 mm PABBO BB/19F-1H/D Z-GRD Z108618/0582
PULPROG                  zg30
TD                      65536
SOLVENT                 CDCl3
NS                         16
DS                          2
SWH               8012.820 Hz
FIDRES            0.122266 Hz
AQ               4.0894465 sec
RG                      88.84
DW                 62.400 usec
DE                   6.50 usec
TE                    296.0 K
D1             1.00000000 sec
TD0                         1

======== CHANNEL f1 ========
SFO1           400.1324710 MHz
NUC1                       1H
P1                  14.50 usec
PLW1          11.99499989 W

F2 - Processing parameters
SI                      65536
SF             400.1300095 MHz
WDW                        EM
SSB                         0
LB                    0.30 Hz
GB                          0
PC                       1.00
```

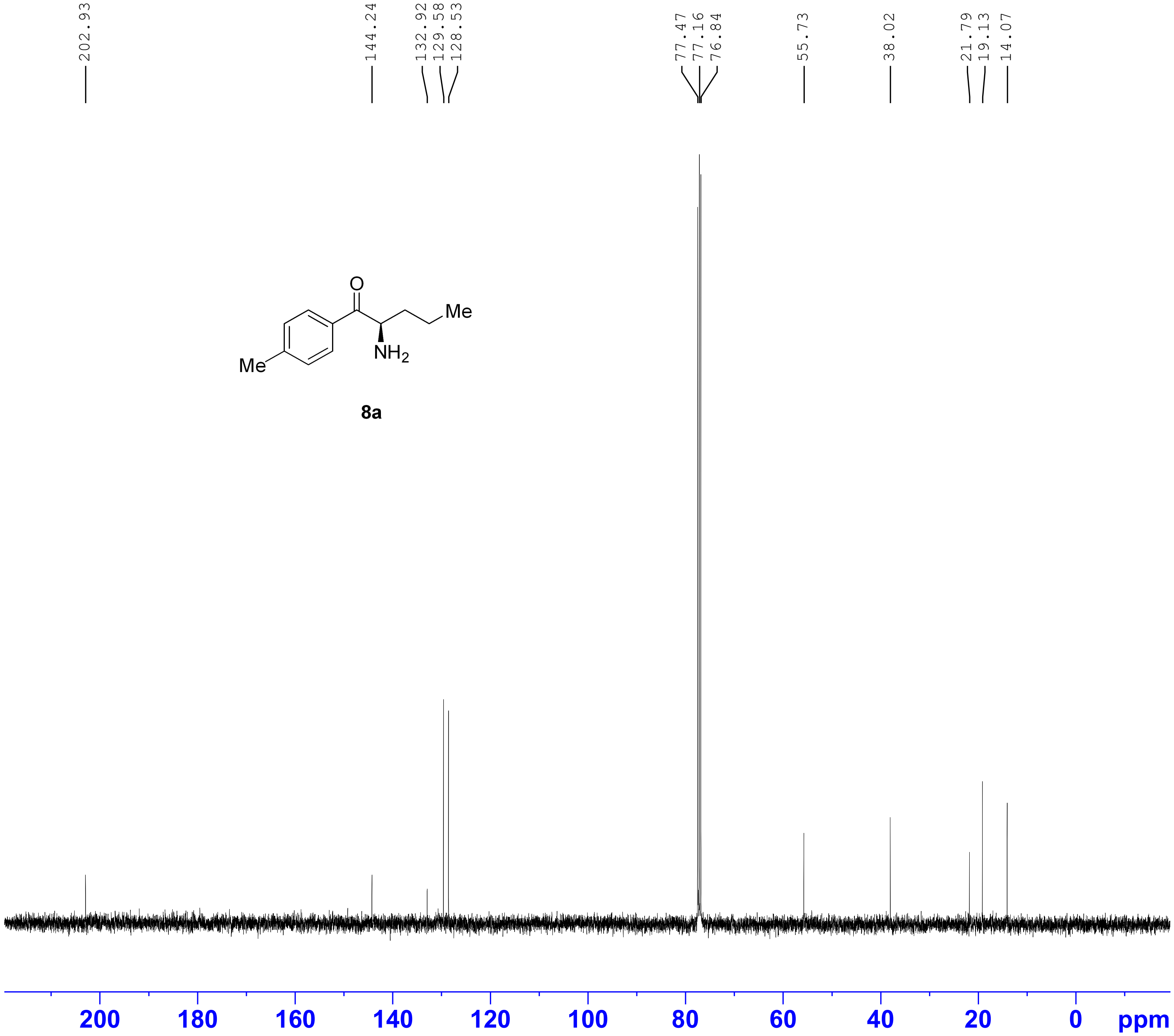

Current Data Parameters
NAME luoy-2-136a
EXPNO 2
PROCNO 1

F2 - Acquisition Parameters
Date_ 20260703
Time 20.49
INSTRUM spect
PROBHD 5 mm PABBO BB/19F-1H/D Z-GRD Z108618/0582
PULPROG zgpg30
TD 65536
SOLVENT CDCl3
NS 68
DS 2
SWH 24038.461 Hz
FIDRES 0.366798 Hz
AQ 1.3631488 sec
RG 196.92
DW 20.800 usec
DE 6.50 usec
TE 296.8 K
D1 2.00000000 sec
D11 0.03000000 sec
TD0 1

======== CHANNEL f1 ========
SFO1 100.6228298 MHz
NUC1 13C
P1 9.70 usec
PLW1 46.98899841 W

======== CHANNEL f2 ========
SFO2 400.1316005 MHz
NUC2 1H
CPDPRG[2] waltz16
PCPD2 90.00 usec
PLW2 11.99499989 W
PLW12 0.34213999 W
PLW13 0.27713001 W

F2 - Processing parameters
SI 32768
SF 100.6127564 MHz
WDW EM
SSB 0
LB 1.00 Hz
GB 0
PC 1.40

===

Acq. Operator : Seq. Line : 25
Acq. Instrument : Instrument 1 Location : Vial 54
Injection Date : 10/23/2019 1:16:39 AM Inj : 1
Inj Volume : 5.000 µl
Different Inj Volume from Sequence ! Actual Inj Volume : 3.000 µl
Acq. Method : C:\CHEM32\1\DATA\QDY 2019-10-22 12-42-40\IC-03-60.M
Last changed : 5/7/2019 10:27:30 AM
Analysis Method : C:\CHEM32\1\METHODS\IC-007-90.M
Last changed : 11/26/2020 2:57:59 PM
(modified after loading)
Additional Info : Peak(s) manually integrated

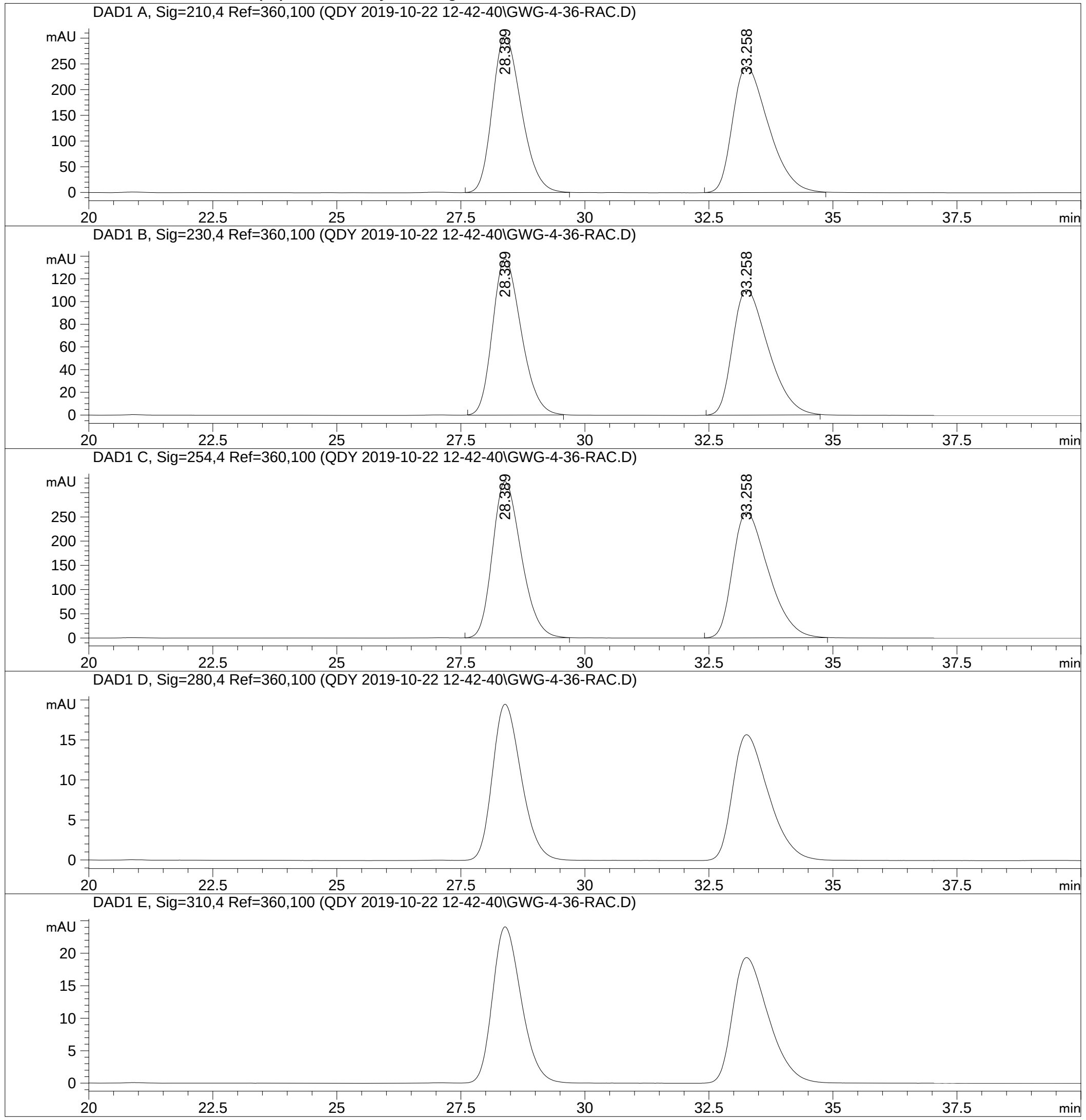

Data File C:\CHEM32\1\DATA\QDY 2019-10-22 12-42-40\GWG-4-36-RAC.D
Sample Name:

===

Area Percent Report

===

Sorted By : Signal
Multiplier : 1.0000
Dilution : 1.0000
Use Multiplier & Dilution Factor with ISTDs

Signal 1: DAD1 A, Sig=210,4 Ref=360,100

Peak #	RetTime [min]	Type	Width [min]	Area [mAU*s]	Height [mAU]	Area %
1	28.389	BB	0.6289	1.23089e4	303.66568	50.0181
2	33.258	BB	0.7691	1.23000e4	245.24716	49.9819
Totals :				2.46088e4	548.91284	

Signal 2: DAD1 B, Sig=230,4 Ref=360,100

Peak #	RetTime [min]	Type	Width [min]	Area [mAU*s]	Height [mAU]	Area %
1	28.389	BB	0.6227	5526.88525	137.57271	50.0739
2	33.258	BB	0.7689	5510.57422	110.68127	49.9261
Totals :				1.10375e4	248.25398	

Signal 3: DAD1 C, Sig=254,4 Ref=360,100

Peak #	RetTime [min]	Type	Width [min]	Area [mAU*s]	Height [mAU]	Area %
1	28.389	BB	0.6234	1.29816e4	322.67422	50.0331
2	33.258	BB	0.7685	1.29644e4	259.66385	49.9669
Totals :				2.59459e4	582.33807	

Signal 4: DAD1 D, Sig=280,4 Ref=360,100

Signal 5: DAD1 E, Sig=310,4 Ref=360,100

===

*** End of Report ***

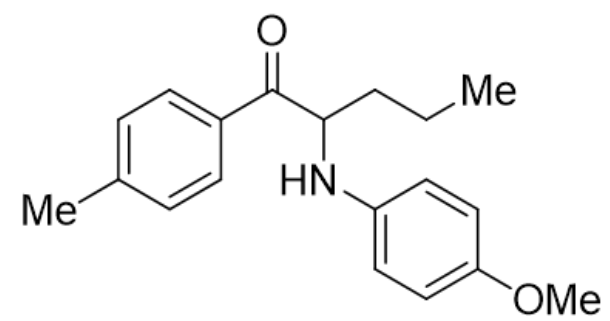

3a-Rac

```
=====================================================================
Acq. Operator   :                               Seq. Line :   2
Acq. Instrument : Instrument 1                   Location : Vial 83
Injection Date  : 11/4/2020 5:12:42 PM                Inj :   1
                                               Inj Volume : 5.000 µl
Different Inj Volume from Sequence !     Actual Inj Volume : 2.000 µl
Acq. Method     : C:\CHEM32\1\DATA\QDY 2020-11-04 16-59-53\IC-03-40.M
Last changed    : 11/4/2020 5:11:49 PM
                  (modified after loading)
Analysis Method : C:\CHEM32\1\DATA\QDY 2020-11-26 19-45-55\OD-01-20.M
Last changed    : 11/26/2020 10:09:08 PM
                  (modified after loading)
Additional Info : Peak(s) manually integrated
```

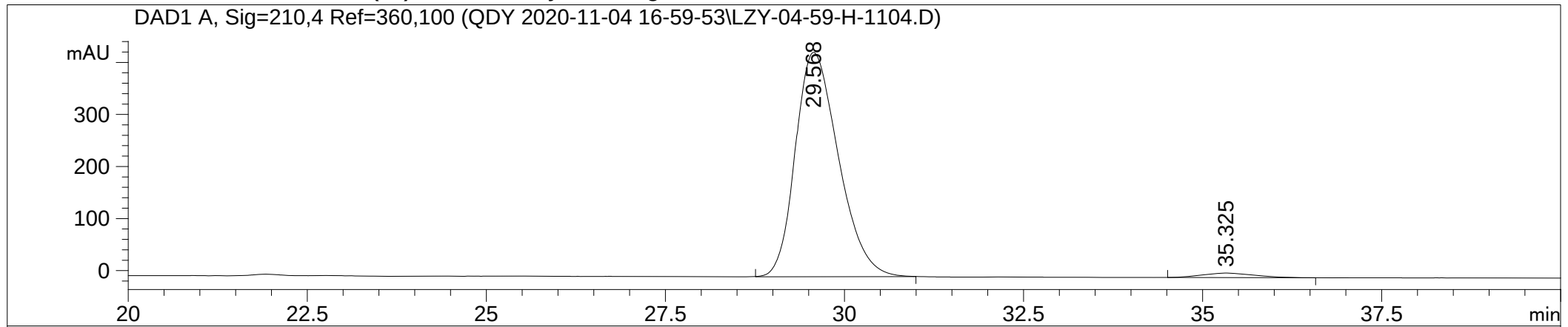

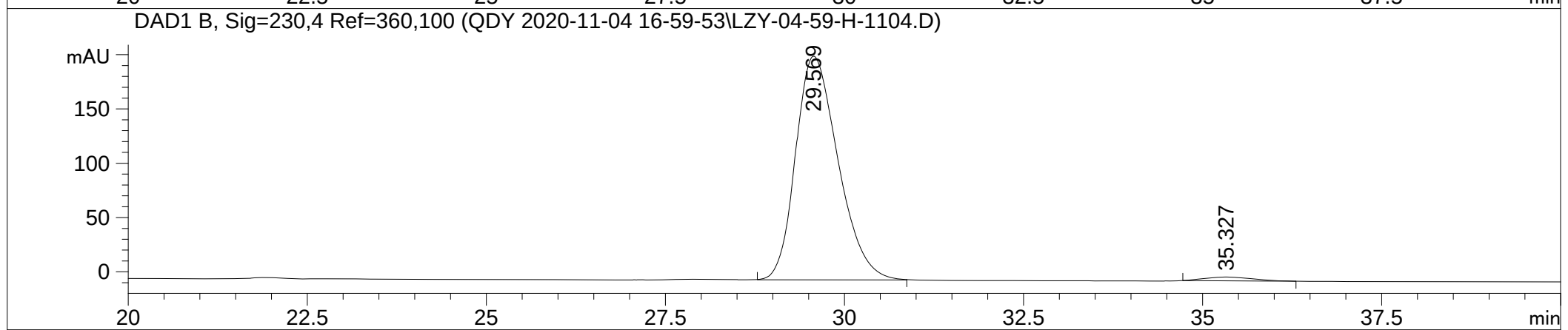

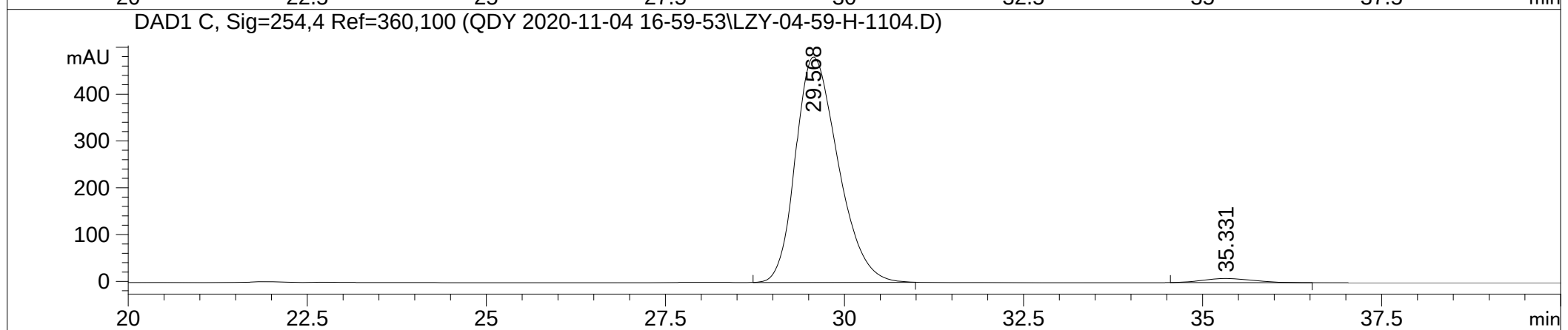

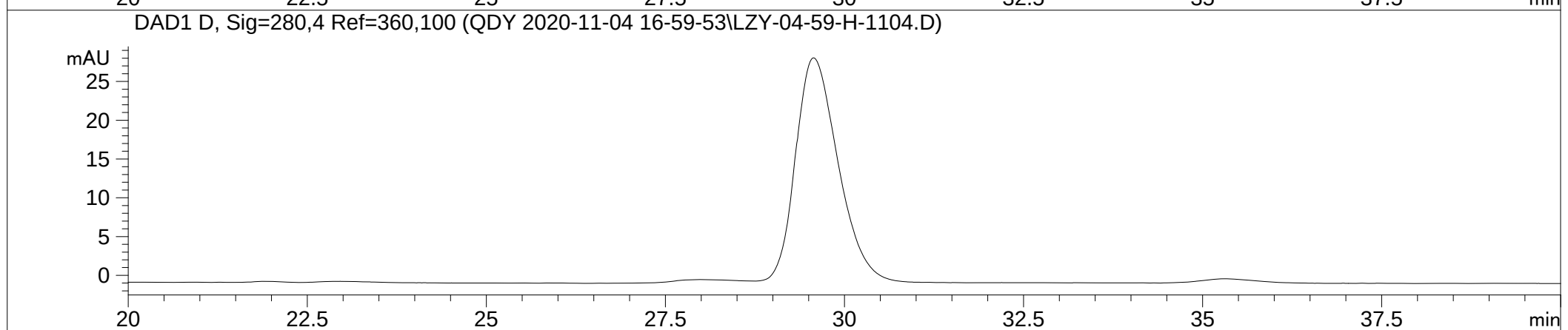

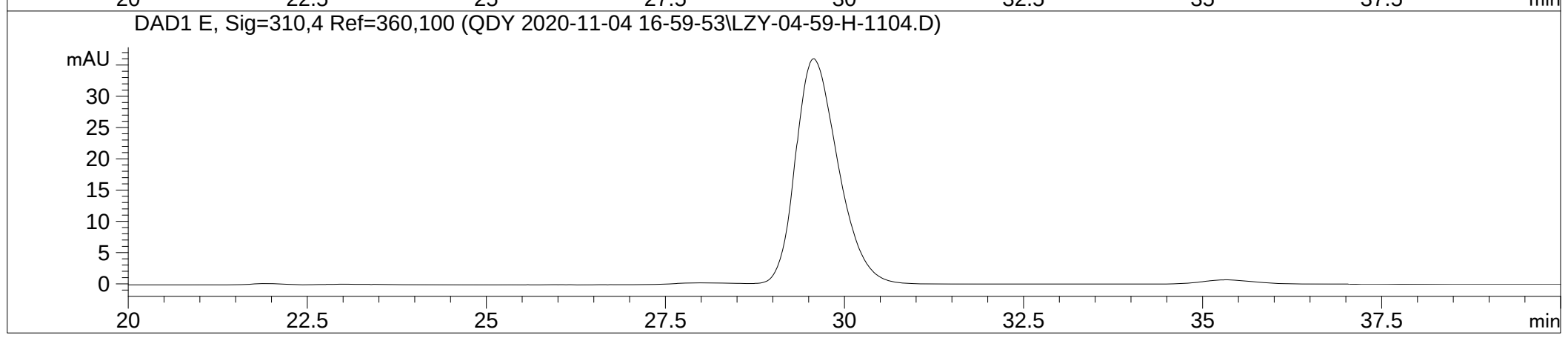

Data File C:\CHEM32\1\DATA\QDY 2020-11-04 16-59-53\LZY-04-59-H-1104.D
Sample Name:

```
=====================================================================
                         Area Percent Report
=====================================================================

Sorted By             :      Signal
Multiplier            :      1.0000
Dilution              :      1.0000
Use Multiplier & Dilution Factor with ISTDs


Signal 1: DAD1 A, Sig=210,4 Ref=360,100

Peak RetTime Type  Width     Area      Height     Area
  #   [min]        [min]   [mAU*s]     [mAU]        %
----|-------|----|-------|----------|----------|--------|
   1  29.568 BB    0.6453 1.79922e4  430.77173 97.6243
   2  35.325 BB    0.6036  437.83377    8.66370  2.3757

Totals :                  1.84300e4  439.43543


Signal 2: DAD1 B, Sig=230,4 Ref=360,100

Peak RetTime Type  Width     Area      Height     Area
  #   [min]        [min]   [mAU*s]     [mAU]        %
----|-------|----|-------|----------|----------|--------|
   1  29.569 BB    0.6345 8482.98730 205.95845 98.1088
   2  35.327 BB    0.6351  163.52400   3.59231  1.8912

Totals :                  8646.51131  209.55076


Signal 3: DAD1 C, Sig=254,4 Ref=360,100

Peak RetTime Type  Width     Area      Height     Area
  #   [min]        [min]   [mAU*s]     [mAU]        %
----|-------|----|-------|----------|----------|--------|
   1  29.568 BB    0.6352 1.98614e4  481.49551 97.8799
   2  35.331 BB    0.7387  430.19412    8.91880  2.1201

Totals :                  2.02916e4  490.41431


Signal 4: DAD1 D, Sig=280,4 Ref=360,100


Signal 5: DAD1 E, Sig=310,4 Ref=360,100

=====================================================================
                         *** End of Report ***
```

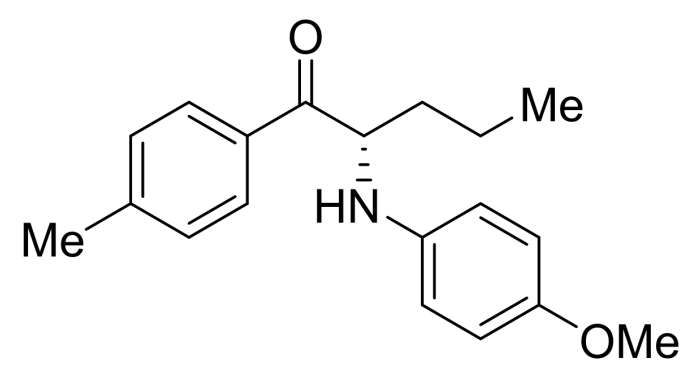

3a

Data File C:\CHEM32\1\DATA\QDY 2020-11-06 10-02-06\LZY-04-82-A-RAC.D
Sample Name:

```
=====================================================================
Acq. Operator   :                            Seq. Line :   2
Acq. Instrument : Instrument 1                Location : Vial 83
Injection Date  : 11/6/2020 10:15:14 AM            Inj :   1
                                            Inj Volume : 5.000 µl
Different Inj Volume from Sequence !     Actual Inj Volume : 2.000 µl
Acq. Method     : C:\CHEM32\1\DATA\QDY 2020-11-06 10-02-06\IC-03-40.M
Last changed    : 11/6/2020 10:14:22 AM
                  (modified after loading)
Analysis Method : C:\CHEM32\1\DATA\QDY 2020-11-05 11-21-39\OD-20-10.M
Last changed    : 11/6/2020 11:12:41 AM
                  (modified after loading)
Additional Info : Peak(s) manually integrated
```

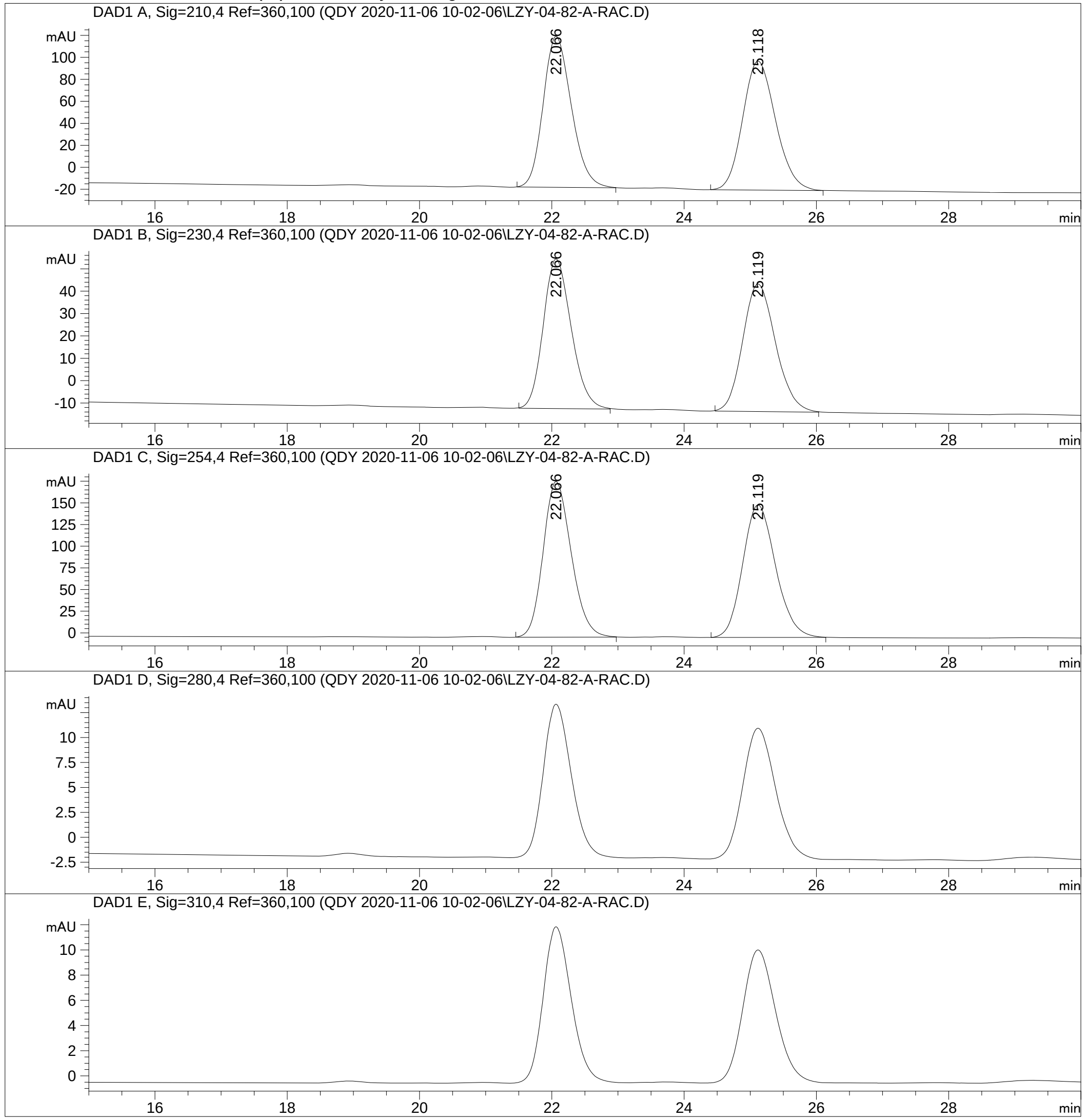

Data File C:\CHEM32\1\DATA\QDY 2020-11-06 10-02-06\LZY-04-82-A-RAC.D
Sample Name:

===
Area Percent Report
===

Sorted By : Signal
Multiplier : 1.0000
Dilution : 1.0000
Use Multiplier & Dilution Factor with ISTDs

Signal 1: DAD1 A, Sig=210,4 Ref=360,100

Peak #	RetTime [min]	Type	Width [min]	Area [mAU*s]	Height [mAU]	Area %
1	22.066	BB	0.4618	4069.55493	137.30174	50.0336
2	25.118	BB	0.5381	4064.08325	117.32632	49.9664

Totals : 8133.63818 254.62807

Signal 2: DAD1 B, Sig=230,4 Ref=360,100

Peak #	RetTime [min]	Type	Width [min]	Area [mAU*s]	Height [mAU]	Area %
1	22.066	BB	0.4578	1972.09277	66.91785	50.0293
2	25.119	BB	0.5348	1969.78528	57.05464	49.9707

Totals : 3941.87805 123.97249

Signal 3: DAD1 C, Sig=254,4 Ref=360,100

Peak #	RetTime [min]	Type	Width [min]	Area [mAU*s]	Height [mAU]	Area %
1	22.066	BB	0.4583	5296.60889	179.47356	50.0323
2	25.119	BB	0.5356	5289.76367	152.91937	49.9677

Totals : 1.05864e4 332.39293

Signal 4: DAD1 D, Sig=280,4 Ref=360,100

Signal 5: DAD1 E, Sig=310,4 Ref=360,100

===
*** End of Report ***

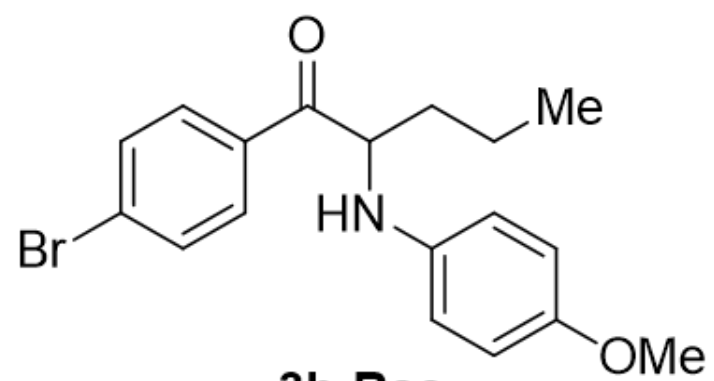

3b-Rac

```
Data File C:\CHEM32\1\DATA\QDY 2021-01-13 22-34-23\LZY-05-123-A.D
Sample Name:

=====================================================================
Acq. Operator   :                          Seq. Line :   6
Acq. Instrument : Instrument 1              Location : Vial 83
Injection Date  : 1/13/2021 11:31:21 PM          Inj :   1
                                          Inj Volume : 5.000 µl
Different Inj Volume from Sequence !     Actual Inj Volume : 2.000 µl
Acq. Method     : C:\CHEM32\1\DATA\QDY 2021-01-13 22-34-23\IC-03-40.M
Last changed    : 1/13/2021 11:30:31 PM
                  (modified after loading)
Analysis Method : C:\CHEM32\1\METHODS\OD-20-10.M
Last changed    : 3/13/2021 9:25:22 PM
                  (modified after loading)
Additional Info : Peak(s) manually integrated
```

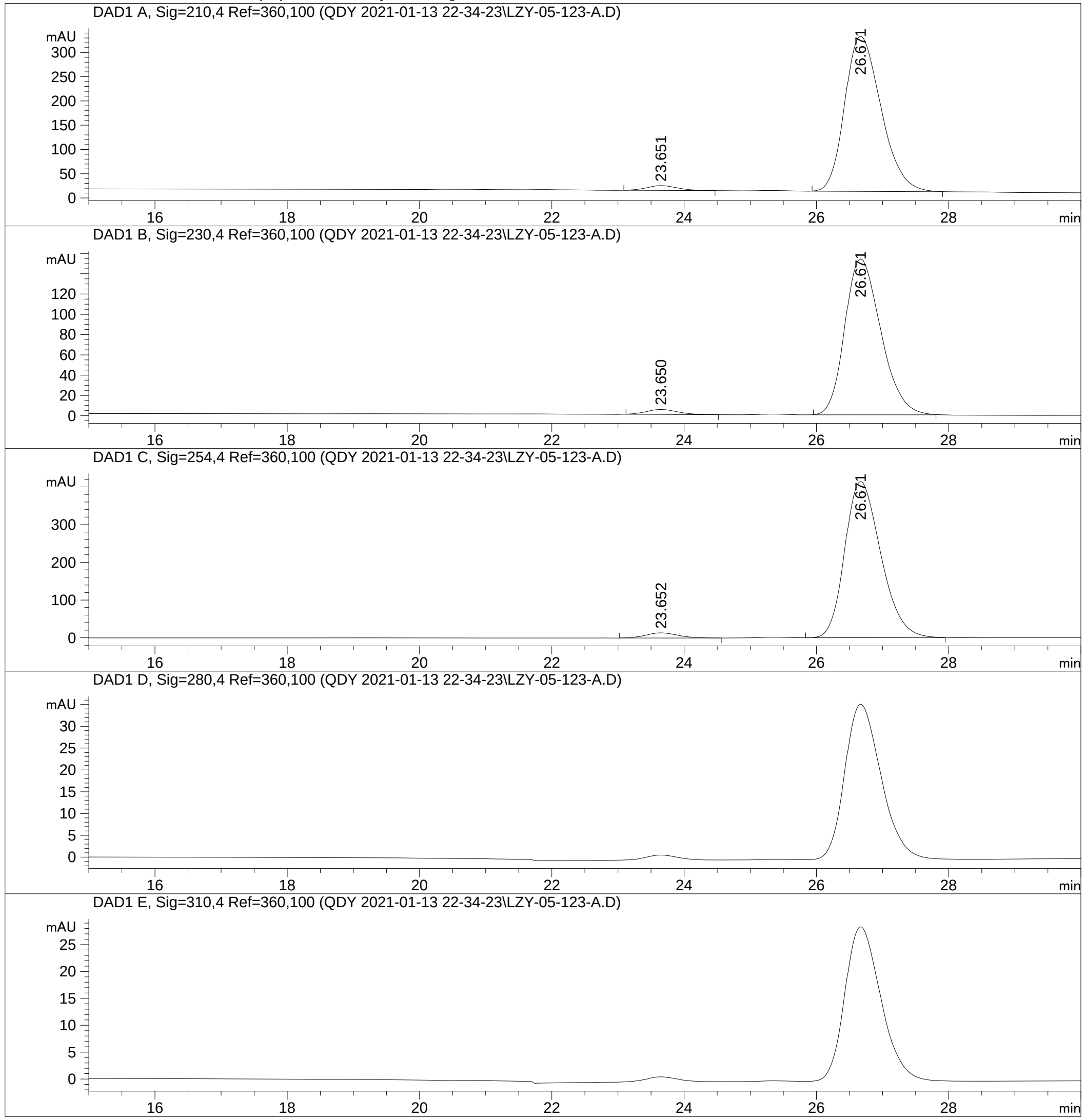

Data File C:\CHEM32\1\DATA\QDY 2021-01-13 22-34-23\LZY-05-123-A.D
Sample Name:

===

Area Percent Report

===

```
Sorted By             :      Signal
Multiplier            :      1.0000
Dilution              :      1.0000
Use Multiplier & Dilution Factor with ISTDs
```

Signal 1: DAD1 A, Sig=210,4 Ref=360,100

Peak #	RetTime [min]	Type	Width [min]	Area [mAU*s]	Height [mAU]	Area %
1	23.651	BB	0.4738	321.62668	9.93273	2.5723
2	26.671	BB	0.5905	1.21821e4	319.71472	97.4277

Totals : 1.25037e4 329.64745

Signal 2: DAD1 B, Sig=230,4 Ref=360,100

Peak #	RetTime [min]	Type	Width [min]	Area [mAU*s]	Height [mAU]	Area %
1	23.650	BB	0.5092	158.49904	4.87698	2.6546
2	26.671	BB	0.5849	5812.25049	153.80045	97.3454

Totals : 5970.74953 158.67743

Signal 3: DAD1 C, Sig=254,4 Ref=360,100

Peak #	RetTime [min]	Type	Width [min]	Area [mAU*s]	Height [mAU]	Area %
1	23.652	BB	0.5256	454.06857	13.59840	2.8169
2	26.671	VB	0.5853	1.56654e4	414.17831	97.1831

Totals : 1.61194e4 427.77672

Signal 4: DAD1 D, Sig=280,4 Ref=360,100

Signal 5: DAD1 E, Sig=310,4 Ref=360,100

===

*** End of Report ***

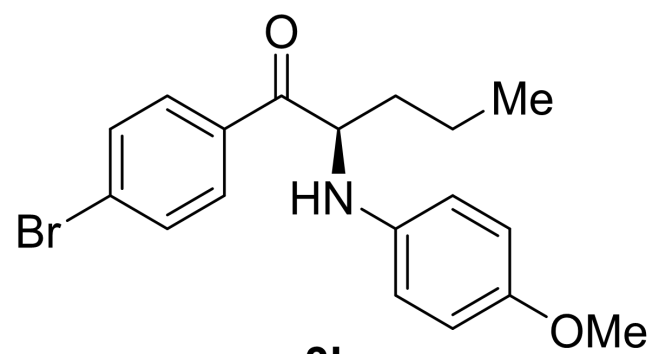

3b

Data File C:\CHEM32\1\DATA\QDY 2020-09-19 22-37-37\LZY-04-107-RAC-IC03.D
Sample Name:

```
=====================================================================
Acq. Operator   :                              Seq. Line :  14
Acq. Instrument : Instrument 1                  Location : Vial 91
Injection Date  : 9/20/2020 3:45:57 AM               Inj :   1
                                              Inj Volume : 5.000 µl
Different Inj Volume from Sequence !     Actual Inj Volume : 3.000 µl
Acq. Method     : C:\CHEM32\1\DATA\QDY 2020-09-19 22-37-37\IC-03-20.M
Last changed    : 9/20/2020 3:45:05 AM
                  (modified after loading)
Analysis Method : C:\CHEM32\1\METHODS\IC-007-90.M
Last changed    : 11/25/2020 8:19:49 PM
                  (modified after loading)
Additional Info : Peak(s) manually integrated
```

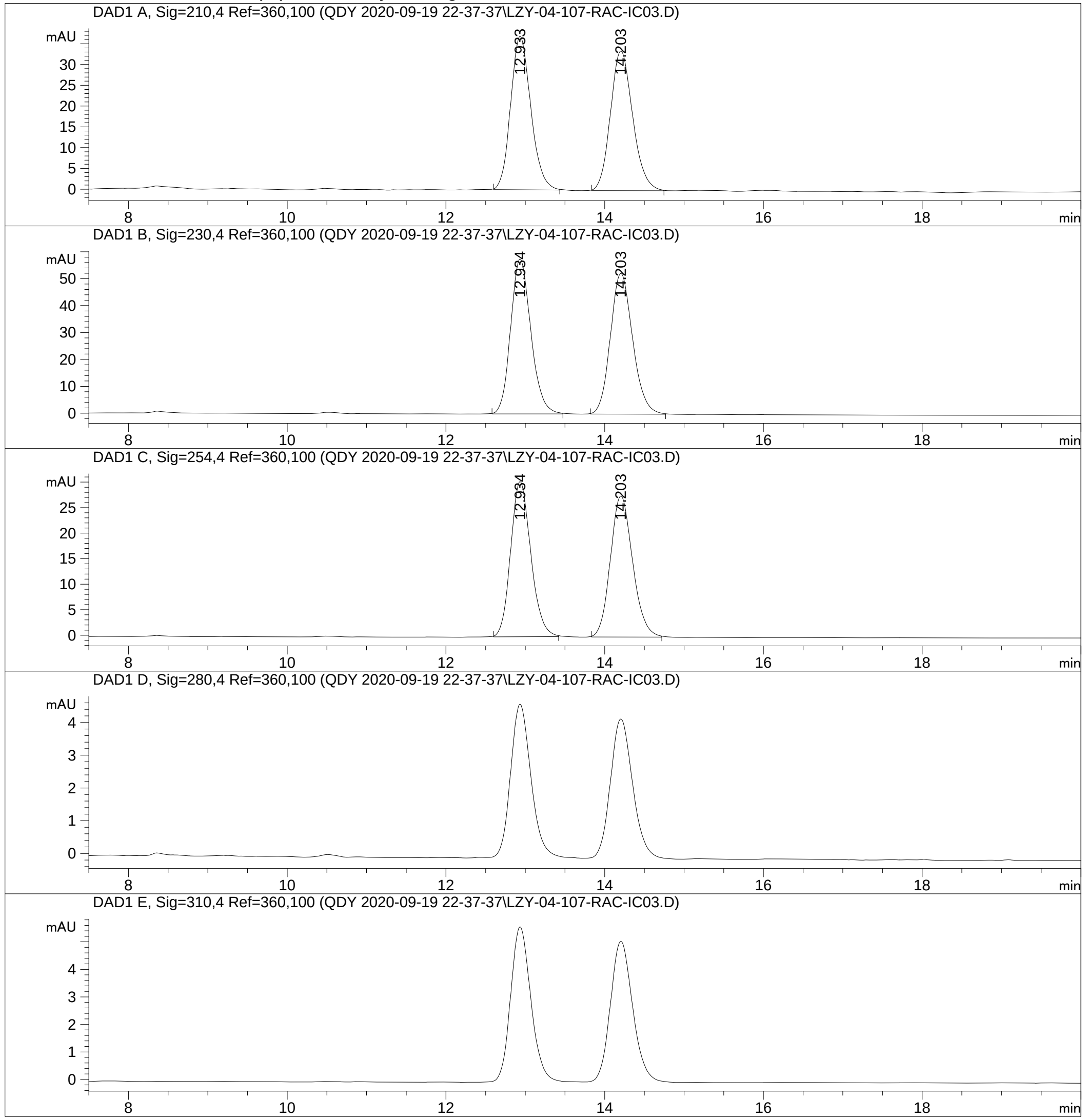

Data File C:\CHEM32\1\DATA\QDY 2020-09-19 22-37-37\LZY-04-107-RAC-IC03.D
Sample Name:

```
=====================================================================
                         Area Percent Report
=====================================================================

Sorted By             :      Signal
Multiplier            :      1.0000
Dilution              :      1.0000
Use Multiplier & Dilution Factor with ISTDs
```

Signal 1: DAD1 A, Sig=210,4 Ref=360,100

Peak #	RetTime [min]	Type	Width [min]	Area [mAU*s]	Height [mAU]	Area %
1	12.933	BB	0.2723	652.77069	36.91661	49.9467
2	14.203	BB	0.3011	654.16364	33.61052	50.0533

Totals : 1306.93433 70.52713

Signal 2: DAD1 B, Sig=230,4 Ref=360,100

Peak #	RetTime [min]	Type	Width [min]	Area [mAU*s]	Height [mAU]	Area %
1	12.934	BB	0.2728	1020.20844	57.56078	50.0765
2	14.203	BB	0.2989	1017.09259	52.28540	49.9235

Totals : 2037.30103 109.84617

Signal 3: DAD1 C, Sig=254,4 Ref=360,100

Peak #	RetTime [min]	Type	Width [min]	Area [mAU*s]	Height [mAU]	Area %
1	12.934	BB	0.2719	536.71246	30.40441	49.9803
2	14.203	BB	0.3007	537.13519	27.64681	50.0197

Totals : 1073.84766 58.05122

Signal 4: DAD1 D, Sig=280,4 Ref=360,100

Signal 5: DAD1 E, Sig=310,4 Ref=360,100

```
=====================================================================
                         *** End of Report ***
```

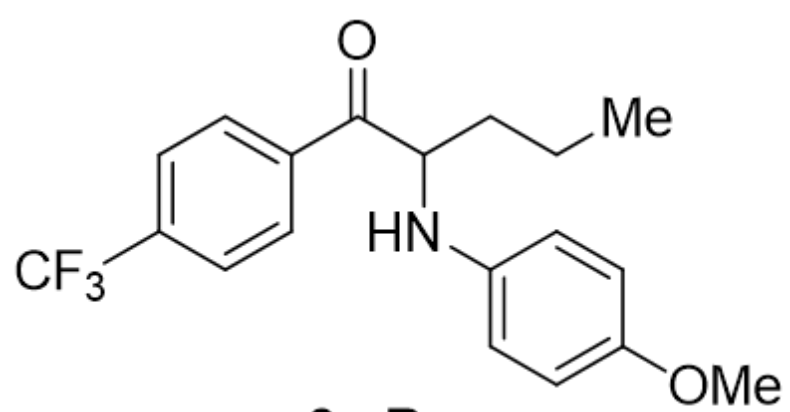

3c-Rac

===

Acq. Operator : Seq. Line : 2
Acq. Instrument : Instrument 1 Location : Vial 84
Injection Date : 11/18/2020 10:29:50 PM Inj : 1
Inj Volume : 5.000 µl
Different Inj Volume from Sequence ! Actual Inj Volume : 2.000 µl
Acq. Method : C:\CHEM32\1\DATA\QDY 2020-11-18 22-17-02\IC-03-20.M
Last changed : 11/18/2020 10:29:00 PM
(modified after loading)
Analysis Method : C:\CHEM32\1\METHODS\IC-007-90.M
Last changed : 11/25/2020 8:48:54 PM
(modified after loading)
Additional Info : Peak(s) manually integrated

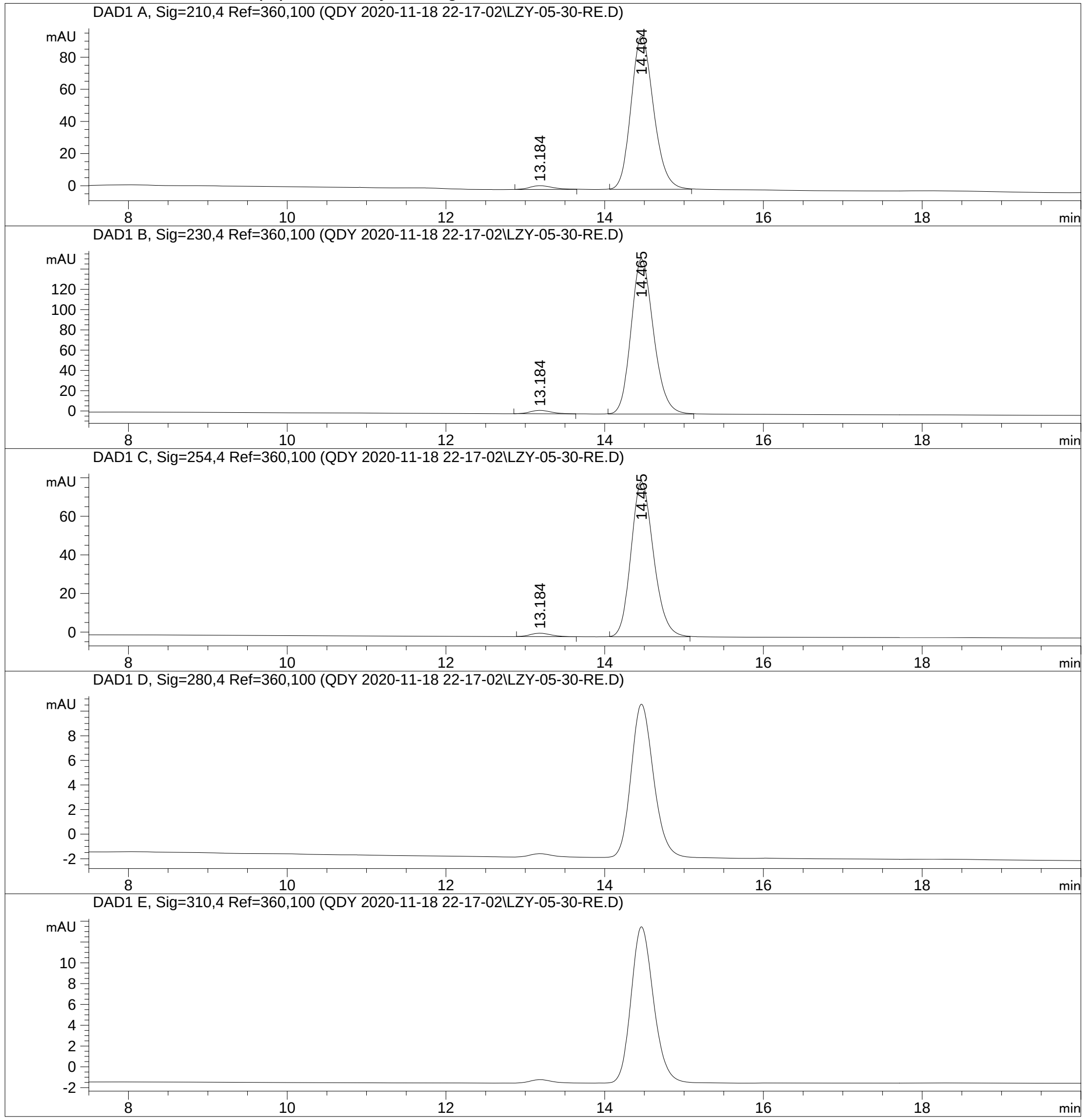

Data File C:\CHEM32\1\DATA\QDY 2020-11-18 22-17-02\LZY-05-30-RE.D
Sample Name:

===

Area Percent Report

===

Sorted By : Signal
Multiplier : 1.0000
Dilution : 1.0000
Use Multiplier & Dilution Factor with ISTDs

Signal 1: DAD1 A, Sig=210,4 Ref=360,100

Peak #	RetTime [min]	Type	Width [min]	Area [mAU*s]	Height [mAU]	Area %
1	13.184	BB	0.2820	46.22804	2.34505	2.4039
2	14.464	BB	0.3040	1876.78442	95.20882	97.5961

Totals : 1923.01246 97.55388

Signal 2: DAD1 B, Sig=230,4 Ref=360,100

Peak #	RetTime [min]	Type	Width [min]	Area [mAU*s]	Height [mAU]	Area %
1	13.184	BB	0.2775	62.21390	3.43116	2.0278
2	14.465	BB	0.3052	3005.84717	153.00552	97.9722

Totals : 3068.06107 156.43668

Signal 3: DAD1 C, Sig=254,4 Ref=360,100

Peak #	RetTime [min]	Type	Width [min]	Area [mAU*s]	Height [mAU]	Area %
1	13.184	BB	0.2758	31.80793	1.78572	1.9711
2	14.465	BB	0.3054	1581.93652	80.45036	98.0289

Totals : 1613.74445 82.23608

Signal 4: DAD1 D, Sig=280,4 Ref=360,100

Signal 5: DAD1 E, Sig=310,4 Ref=360,100

===

*** End of Report ***

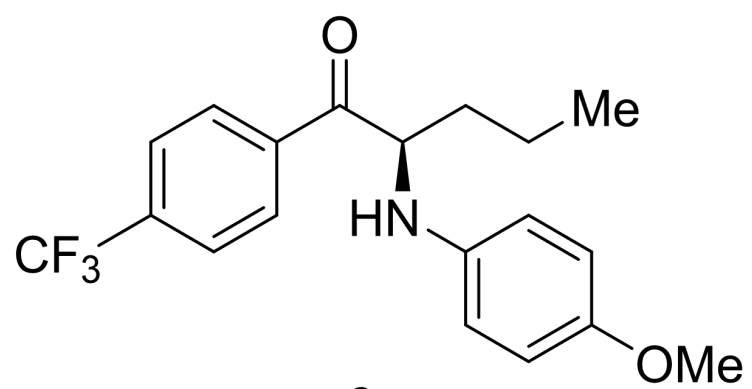

3c

Data File C:\CHEM32\1\DATA\QDY 2020-09-09 16-35-55\LZY-04-65-B-OD07.D
Sample Name:

===

```
Acq. Operator   :                              Seq. Line :  27
Acq. Instrument : Instrument 1                  Location : Vial 92
Injection Date  : 9/10/2020 12:49:23 AM              Inj :   1
                                              Inj Volume : 5.000 µl
Different Inj Volume from Sequence !     Actual Inj Volume : 3.000 µl
Acq. Method     : C:\CHEM32\1\DATA\QDY 2020-09-09 16-35-55\OD-07-60.M
Last changed    : 1/9/2019 4:37:12 PM
Analysis Method : C:\CHEM32\1\METHODS\IC-007-90.M
Last changed    : 11/25/2020 7:36:28 PM
                  (modified after loading)
Additional Info : Peak(s) manually integrated
```

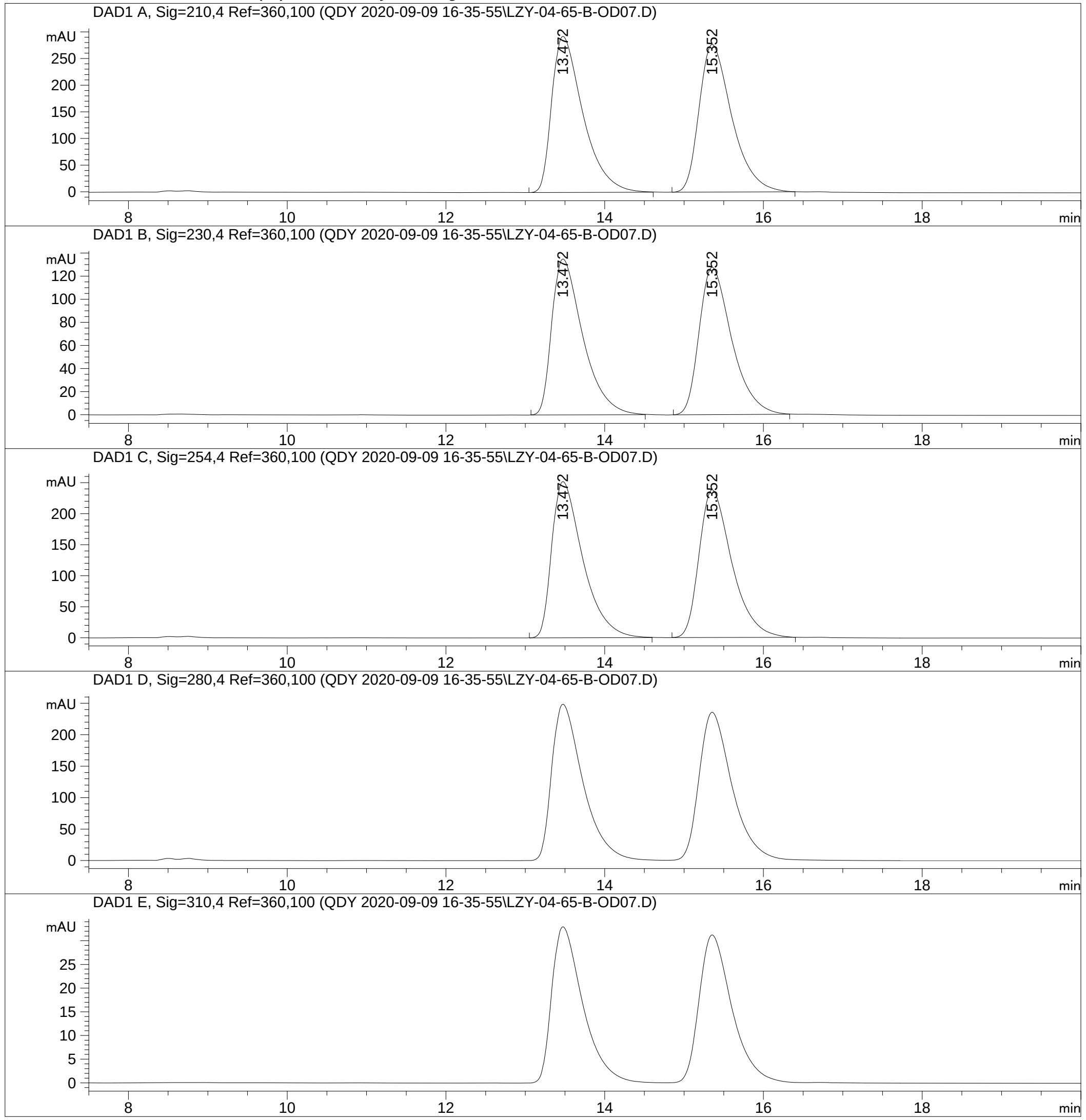

Data File C:\CHEM32\1\DATA\QDY 2020-09-09 16-35-55\LZY-04-65-B-OD07.D
Sample Name:

===
Area Percent Report
===

```
Sorted By             :      Signal
Multiplier            :      1.0000
Dilution              :      1.0000
Use Multiplier & Dilution Factor with ISTDs
```

Signal 1: DAD1 A, Sig=210,4 Ref=360,100

Peak #	RetTime [min]	Type	Width [min]	Area [mAU*s]	Height [mAU]	Area %
1	13.472	BB	0.4153	8013.05225	292.81342	50.2652
2	15.352	BB	0.4380	7928.50684	276.93594	49.7348

Totals : 1.59416e4 569.74936

Signal 2: DAD1 B, Sig=230,4 Ref=360,100

Peak #	RetTime [min]	Type	Width [min]	Area [mAU*s]	Height [mAU]	Area %
1	13.472	BB	0.4133	3672.99390	135.05763	50.2480
2	15.352	BB	0.4344	3636.73145	127.64735	49.7520

Totals : 7309.72534 262.70498

Signal 3: DAD1 C, Sig=254,4 Ref=360,100

Peak #	RetTime [min]	Type	Width [min]	Area [mAU*s]	Height [mAU]	Area %
1	13.472	BB	0.4118	6857.42432	251.71680	50.2307
2	15.352	BB	0.4352	6794.43311	237.93533	49.7693

Totals : 1.36519e4 489.65213

Signal 4: DAD1 D, Sig=280,4 Ref=360,100

Signal 5: DAD1 E, Sig=310,4 Ref=360,100

===
*** End of Report ***

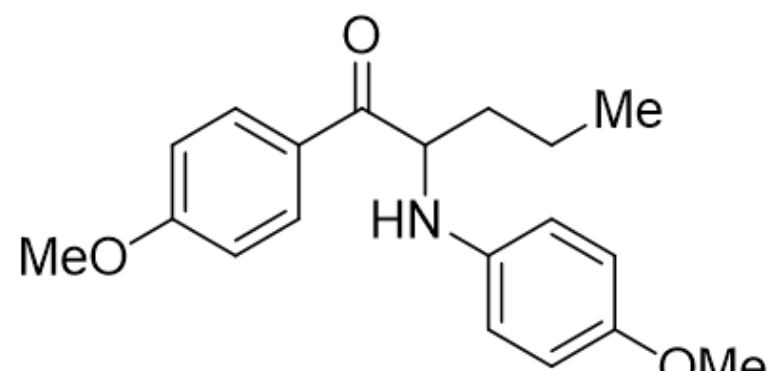

3d-Rac

Data File C:\CHEM32\1\DATA\QDY 2020-09-10 09-22-21\LZY-04-82-B.D
Sample Name:

===

Acq. Operator   :   Seq. Line :   2
Acq. Instrument : Instrument 1   Location : Vial 83
Injection Date : 9/10/2020 9:35:37 AM   Inj :   1
Inj Volume : 5.000 µl
Different Inj Volume from Sequence !   Actual Inj Volume : 3.000 µl
Acq. Method   : C:\CHEM32\1\DATA\QDY 2020-09-10 09-22-21\OD-07-60.M
Last changed   : 1/9/2019 4:37:12 PM
Analysis Method : C:\CHEM32\1\METHODS\IC-007-90.M
Last changed   : 11/25/2020 7:36:28 PM
(modified after loading)
Additional Info : Peak(s) manually integrated

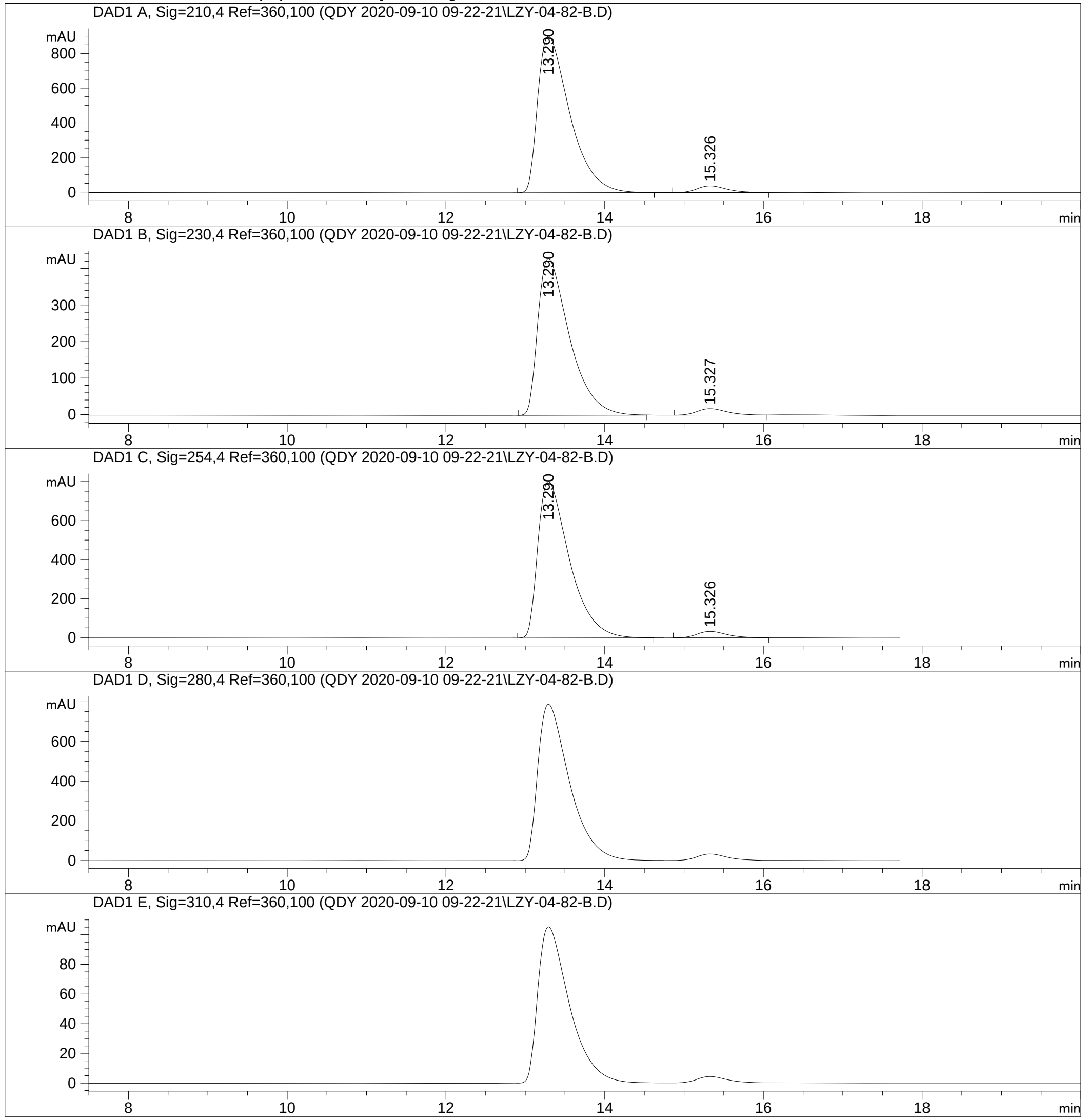

Data File C:\CHEM32\1\DATA\QDY 2020-09-10 09-22-21\LZY-04-82-B.D
Sample Name:

===

Area Percent Report

===

Sorted By   :   Signal
Multiplier   :   1.0000
Dilution   :   1.0000
Use Multiplier & Dilution Factor with ISTDs

Signal 1: DAD1 A, Sig=210,4 Ref=360,100

Peak #	RetTime [min]	Type	Width [min]	Area [mAU*s]	Height [mAU]	Area %
1	13.290	BB	0.4270	2.53005e4	902.68622	96.1791
2	15.326	BB	0.4007	1005.10974	38.74205	3.8209
Totals :				2.63056e4	941.42827	

Signal 2: DAD1 B, Sig=230,4 Ref=360,100

Peak #	RetTime [min]	Type	Width [min]	Area [mAU*s]	Height [mAU]	Area %
1	13.290	BB	0.4184	1.18216e4	427.84180	96.2821
2	15.327	BB	0.3968	456.48822	17.70475	3.7179
Totals :				1.22781e4	445.54655	

Signal 3: DAD1 C, Sig=254,4 Ref=360,100

Peak #	RetTime [min]	Type	Width [min]	Area [mAU*s]	Height [mAU]	Area %
1	13.290	BB	0.4178	2.21146e4	801.75116	96.2367
2	15.326	BB	0.4012	864.78418	33.05760	3.7633
Totals :				2.29794e4	834.80876	

Signal 4: DAD1 D, Sig=280,4 Ref=360,100

Signal 5: DAD1 E, Sig=310,4 Ref=360,100

===

*** End of Report ***

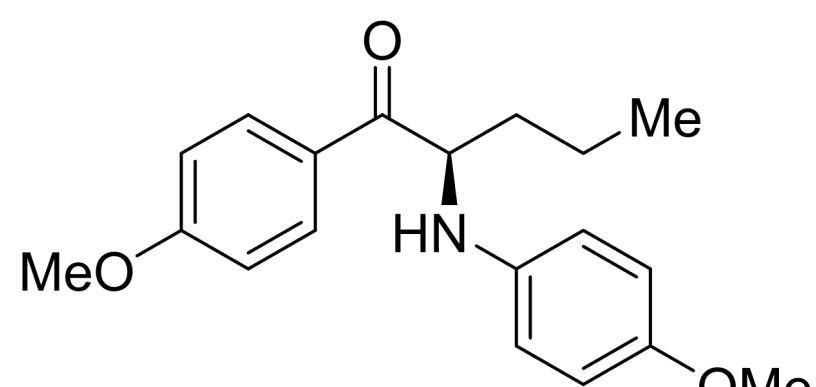

3d

Data File C:\CHEM32\1\DATA\QDY 2020-09-29 13-20-52\LZY-04-125-C-RAC-OD05.D
Sample Name:

===

```
Acq. Operator   :                                Seq. Line :   9
Acq. Instrument : Instrument 1                    Location : Vial 82
Injection Date  : 9/29/2020 4:01:19 PM                  Inj :   1
                                               Inj Volume : 5.000 µl
Acq. Method     : C:\CHEM32\1\DATA\QDY 2020-09-29 13-20-52\OD-05-30.M
Last changed    : 9/29/2020 4:00:25 PM
                  (modified after loading)
Analysis Method : C:\CHEM32\1\METHODS\IC-007-90.M
Last changed    : 11/25/2020 8:38:10 PM
                  (modified after loading)
Additional Info : Peak(s) manually integrated
```

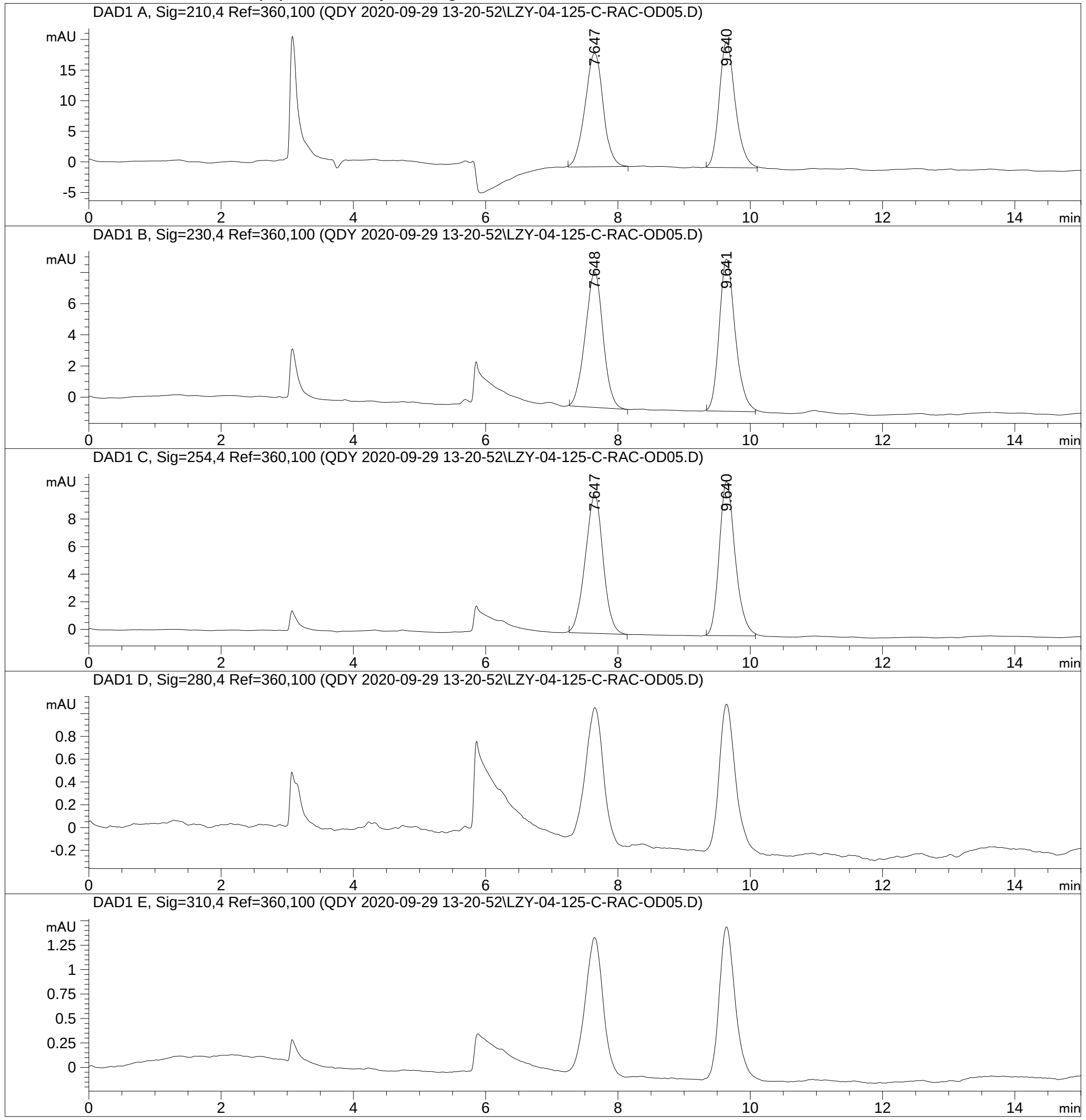

Data File C:\CHEM32\1\DATA\QDY 2020-09-29 13-20-52\LZY-04-125-C-RAC-OD05.D
Sample Name:

===
Area Percent Report
===

```
Sorted By             :      Signal
Multiplier            :      1.0000
Dilution              :      1.0000
Use Multiplier & Dilution Factor with ISTDs
```

Signal 1: DAD1 A, Sig=210,4 Ref=360,100

Peak #	RetTime [min]	Type	Width [min]	Area [mAU*s]	Height [mAU]	Area %
1	7.647	BB	0.2907	355.20343	18.60508	50.5154
2	9.640	BB	0.2592	347.95468	20.58146	49.4846

Totals : 703.15811 39.18654

Signal 2: DAD1 B, Sig=230,4 Ref=360,100

Peak #	RetTime [min]	Type	Width [min]	Area [mAU*s]	Height [mAU]	Area %
1	7.648	BB	0.2840	162.51901	8.69926	49.7207
2	9.641	BB	0.2563	164.34489	9.76746	50.2793

Totals : 326.86391 18.46672

Signal 3: DAD1 C, Sig=254,4 Ref=360,100

Peak #	RetTime [min]	Type	Width [min]	Area [mAU*s]	Height [mAU]	Area %
1	7.647	BB	0.2854	188.51672	10.02276	50.1658
2	9.640	BB	0.2557	187.27080	11.16361	49.8342

Totals : 375.78752 21.18637

Signal 4: DAD1 D, Sig=280,4 Ref=360,100

Signal 5: DAD1 E, Sig=310,4 Ref=360,100

===
*** End of Report ***

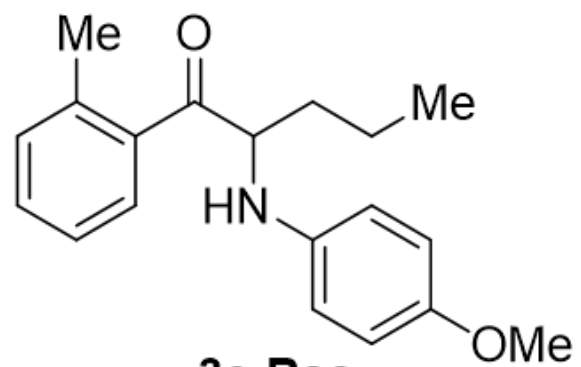

3e-Rac

Data File C:\CHEM32\1\DATA\QDY 2020-09-29 13-20-52\LZY-04-125-C-OD05.D
Sample Name:

===

Acq. Operator :   Seq. Line : 10
Acq. Instrument : Instrument 1   Location : Vial 81
Injection Date : 9/29/2020 4:32:17 PM   Inj : 1
                                                 Inj Volume : 5.000 µl
Different Inj Volume from Sequence !   Actual Inj Volume : 2.000 µl
Acq. Method : C:\CHEM32\1\DATA\QDY 2020-09-29 13-20-52\OD-05-30.M
Last changed : 9/29/2020 4:00:25 PM
(modified after loading)
Analysis Method : C:\CHEM32\1\METHODS\IC-007-90.M
Last changed : 11/25/2020 8:38:10 PM
(modified after loading)
Additional Info : Peak(s) manually integrated

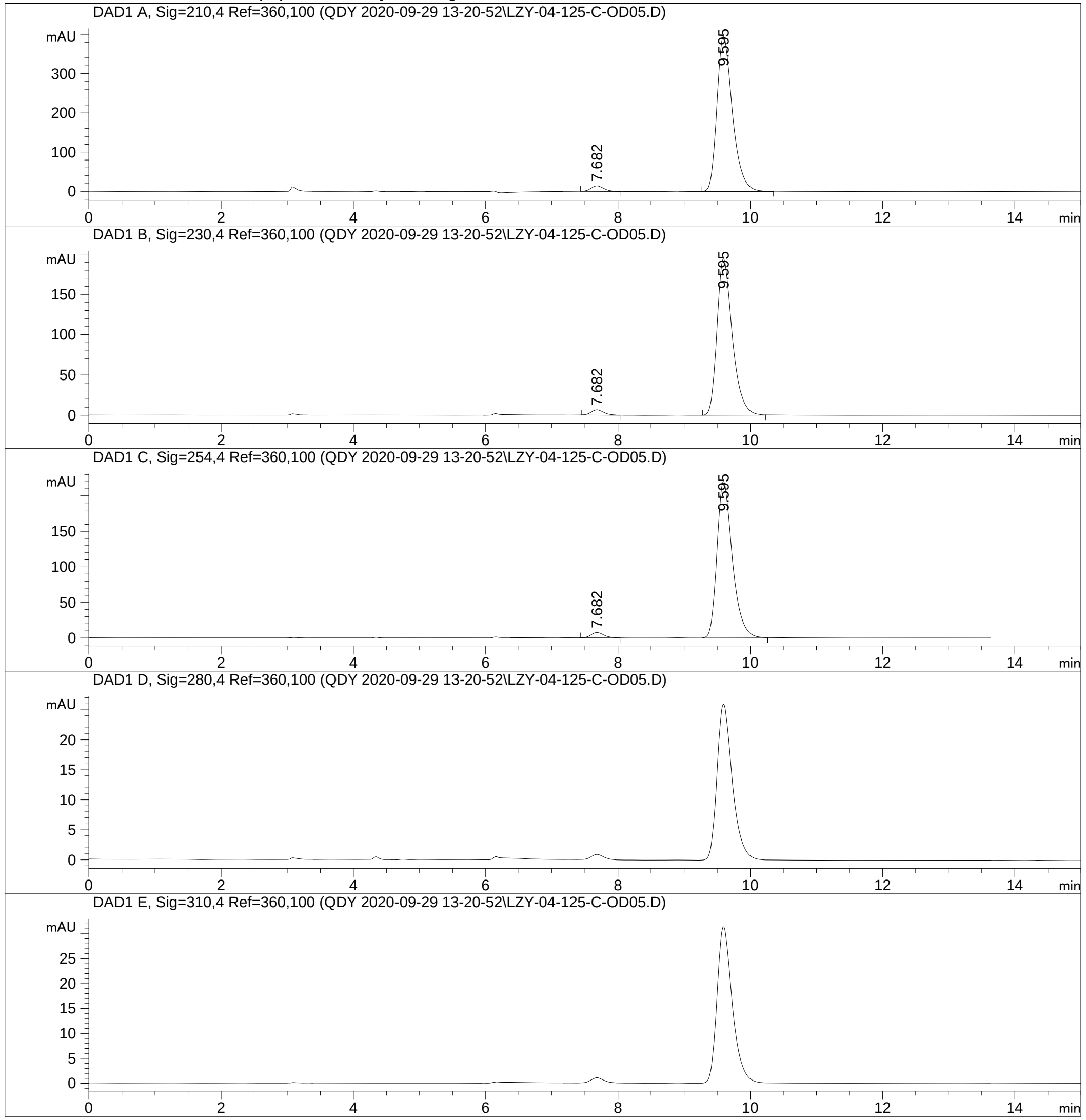

Data File C:\CHEM32\1\DATA\QDY 2020-09-29 13-20-52\LZY-04-125-C-OD05.D
Sample Name:

===

Area Percent Report

===

Sorted By : Signal
Multiplier : 1.0000
Dilution : 1.0000
Use Multiplier & Dilution Factor with ISTDs

Signal 1: DAD1 A, Sig=210,4 Ref=360,100

Peak #	RetTime [min]	Type	Width [min]	Area [mAU*s]	Height [mAU]	Area %
1	7.682	BB	0.2079	190.60579	14.08597	2.8649
2	9.595	BB	0.2488	6462.47705	395.21231	97.1351

Totals : 6653.08284 409.29828

Signal 2: DAD1 B, Sig=230,4 Ref=360,100

Peak #	RetTime [min]	Type	Width [min]	Area [mAU*s]	Height [mAU]	Area %
1	7.682	BB	0.2071	88.86763	6.59775	2.7620
2	9.595	BB	0.2441	3128.63867	194.08778	97.2380

Totals : 3217.50630 200.68553

Signal 3: DAD1 C, Sig=254,4 Ref=360,100

Peak #	RetTime [min]	Type	Width [min]	Area [mAU*s]	Height [mAU]	Area %
1	7.682	BB	0.2081	102.89380	7.59356	2.8056
2	9.595	BB	0.2470	3564.55273	220.07793	97.1944

Totals : 3667.44653 227.67148

Signal 4: DAD1 D, Sig=280,4 Ref=360,100

Signal 5: DAD1 E, Sig=310,4 Ref=360,100

===

*** End of Report ***

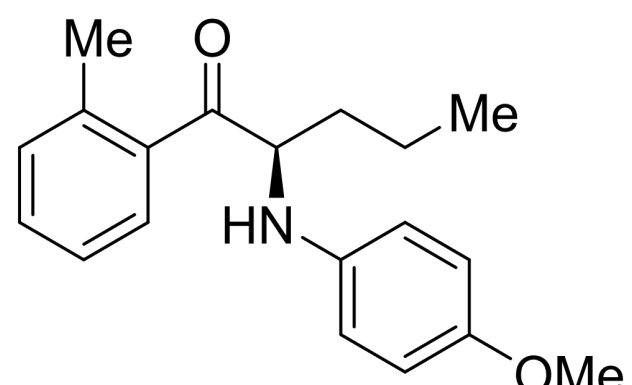

3e

Data File C:\CHEM32\1\DATA\QDY 2020-09-24 11-48-22\LZY-04-115-A-RAC-IC05.D
Sample Name:

===

Acq. Operator :   Seq. Line : 17
Acq. Instrument : Instrument 1   Location : Vial 81
Injection Date : 9/24/2020 5:47:24 PM   Inj : 1
                                               Inj Volume : 5.000 µl
Different Inj Volume from Sequence !   Actual Inj Volume : 2.000 µl
Acq. Method : C:\CHEM32\1\DATA\QDY 2020-09-24 11-48-22\IC-05-30.M
Last changed : 8/23/2012 1:59:23 PM
Analysis Method : C:\CHEM32\1\METHODS\IC-007-90.M
Last changed : 11/25/2020 8:27:45 PM
(modified after loading)
Additional Info : Peak(s) manually integrated

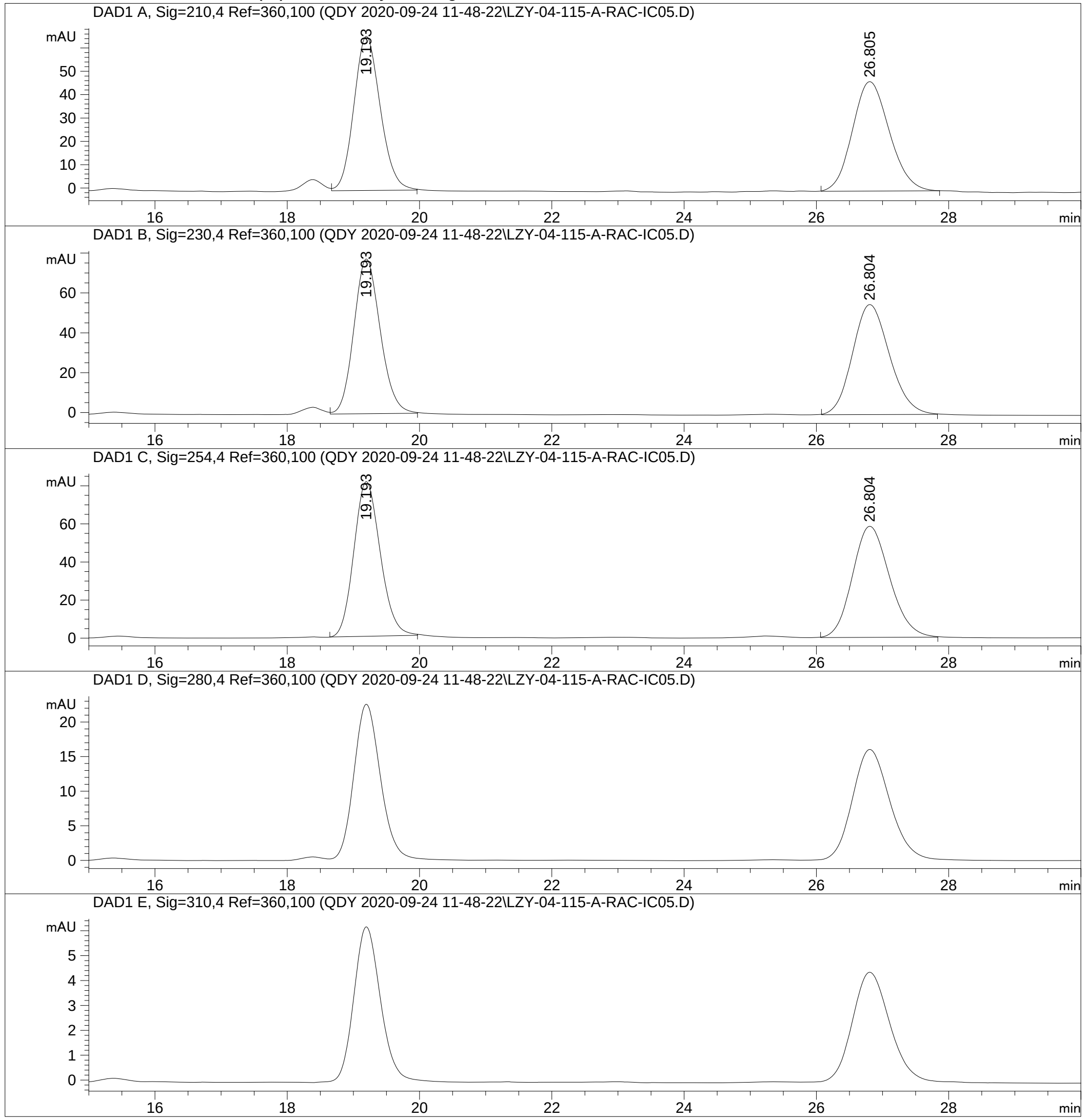

Data File C:\CHEM32\1\DATA\QDY 2020-09-24 11-48-22\LZY-04-115-A-RAC-IC05.D
Sample Name:

===

Area Percent Report

===

Sorted By : Signal
Multiplier : 1.0000
Dilution : 1.0000
Use Multiplier & Dilution Factor with ISTDs

Signal 1: DAD1 A, Sig=210,4 Ref=360,100

Peak #	RetTime [min]	Type	Width [min]	Area [mAU*s]	Height [mAU]	Area %
1	19.193	VB	0.4293	1829.74316	66.03419	50.1180
2	26.805	BB	0.5851	1821.12524	46.88972	49.8820

Totals : 3650.86841 112.92391

Signal 2: DAD1 B, Sig=230,4 Ref=360,100

Peak #	RetTime [min]	Type	Width [min]	Area [mAU*s]	Height [mAU]	Area %
1	19.193	VB	0.4291	2148.81372	77.59110	50.0175
2	26.804	BB	0.6040	2147.31348	55.18215	49.9825

Totals : 4296.12720 132.77325

Signal 3: DAD1 C, Sig=254,4 Ref=360,100

Peak #	RetTime [min]	Type	Width [min]	Area [mAU*s]	Height [mAU]	Area %
1	19.193	BB	0.4309	2249.20020	81.27332	49.6665
2	26.804	BB	0.6077	2279.40991	58.36015	50.3335

Totals : 4528.61011 139.63347

Signal 4: DAD1 D, Sig=280,4 Ref=360,100

Signal 5: DAD1 E, Sig=310,4 Ref=360,100

===

*** End of Report ***

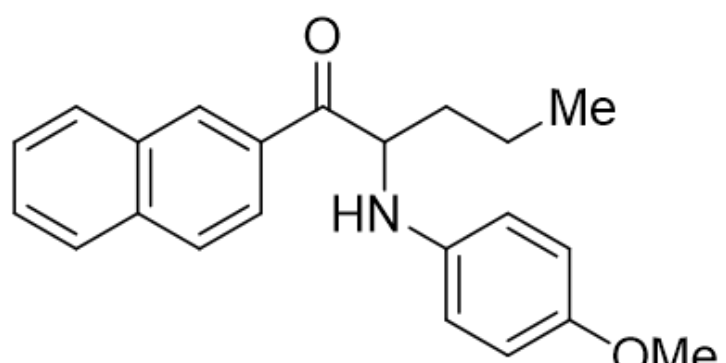

3f-Rac

===

Acq. Operator : Seq. Line : 65
Acq. Instrument : Instrument 1 Location : Vial 82
Injection Date : 9/27/2020 10:54:22 AM Inj : 1
Inj Volume : 5.000 µl
Different Inj Volume from Sequence ! Actual Inj Volume : 2.000 µl
Acq. Method : C:\CHEM32\1\DATA\QDY 2020-09-26 09-35-58\IC-05-30.M
Last changed : 8/23/2012 1:59:23 PM
Analysis Method : C:\CHEM32\1\METHODS\IC-007-90.M
Last changed : 11/25/2020 8:27:45 PM
(modified after loading)
Additional Info : Peak(s) manually integrated

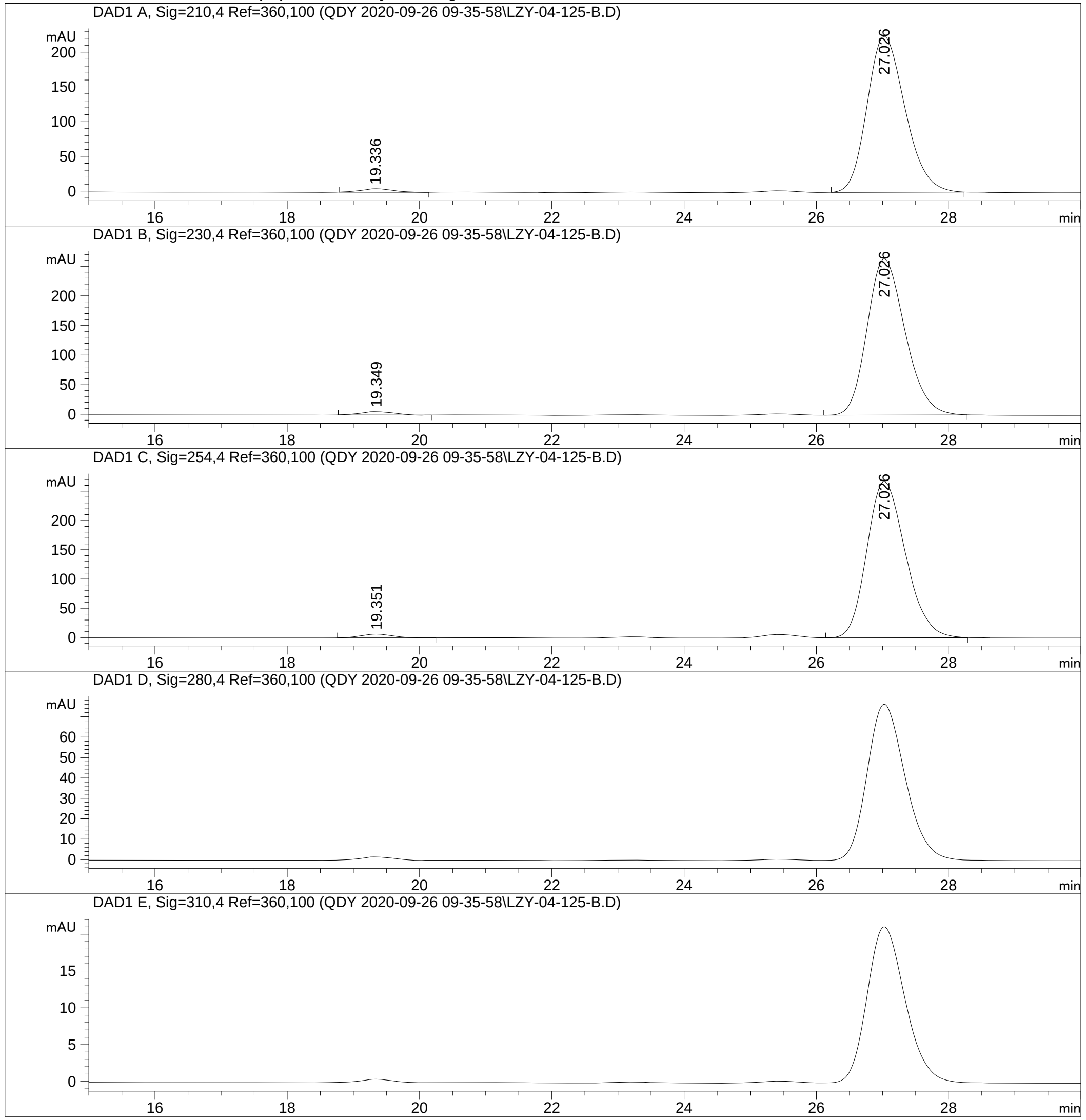

===

Area Percent Report

===

Sorted By : Signal
Multiplier : 1.0000
Dilution : 1.0000
Use Multiplier & Dilution Factor with ISTDs

Signal 1: DAD1 A, Sig=210,4 Ref=360,100

Peak #	RetTime [min]	Type	Width [min]	Area [mAU*s]	Height [mAU]	Area %
1	19.336	BB	0.4478	168.32915	5.06451	1.8439
2	27.026	BB	0.6197	8960.61621	224.50299	98.1561

Totals : 9128.94536 229.56750

Signal 2: DAD1 B, Sig=230,4 Ref=360,100

Peak #	RetTime [min]	Type	Width [min]	Area [mAU*s]	Height [mAU]	Area %
1	19.349	BB	0.5060	193.36757	5.90618	1.8029
2	27.026	VB	0.6178	1.05320e4	263.79053	98.1971

Totals : 1.07254e4 269.69671

Signal 3: DAD1 C, Sig=254,4 Ref=360,100

Peak #	RetTime [min]	Type	Width [min]	Area [mAU*s]	Height [mAU]	Area %
1	19.351	BB	0.5115	203.66541	6.29437	1.8459
2	27.026	VB	0.6296	1.08297e4	266.76236	98.1541

Totals : 1.10333e4 273.05673

Signal 4: DAD1 D, Sig=280,4 Ref=360,100

Signal 5: DAD1 E, Sig=310,4 Ref=360,100

===

*** End of Report ***

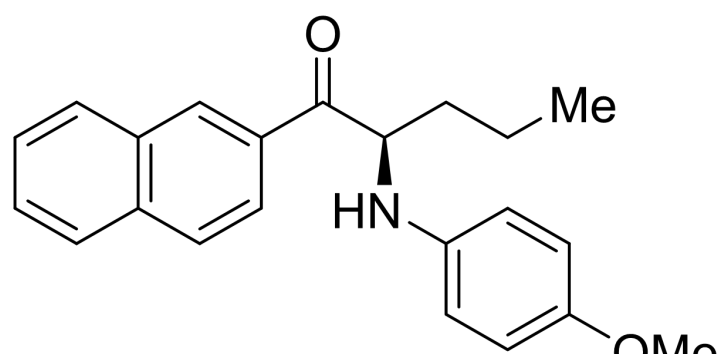

3f

Data File C:\CHEM32\1\DATA\QDY 2020-10-05 14-11-40\LZY-04-136-C-RAC-IC05-1.D
Sample Name:

===

Acq. Operator   :   Seq. Line :   4
Acq. Instrument : Instrument 1   Location : Vial 81
Injection Date : 10/5/2020 3:46:29 PM   Inj :   1
                                                   Inj Volume : 5.000 µl
Different Inj Volume from Sequence !   Actual Inj Volume : 8.000 µl
Acq. Method   : C:\CHEM32\1\DATA\QDY 2020-10-05 14-11-40\IC-05-40.M
Last changed   : 10/5/2020 2:23:37 PM
                  (modified after loading)
Analysis Method : C:\CHEM32\1\METHODS\OD-015-40.M
Last changed   : 11/4/2020 9:21:37 PM
                  (modified after loading)
Additional Info : Peak(s) manually integrated

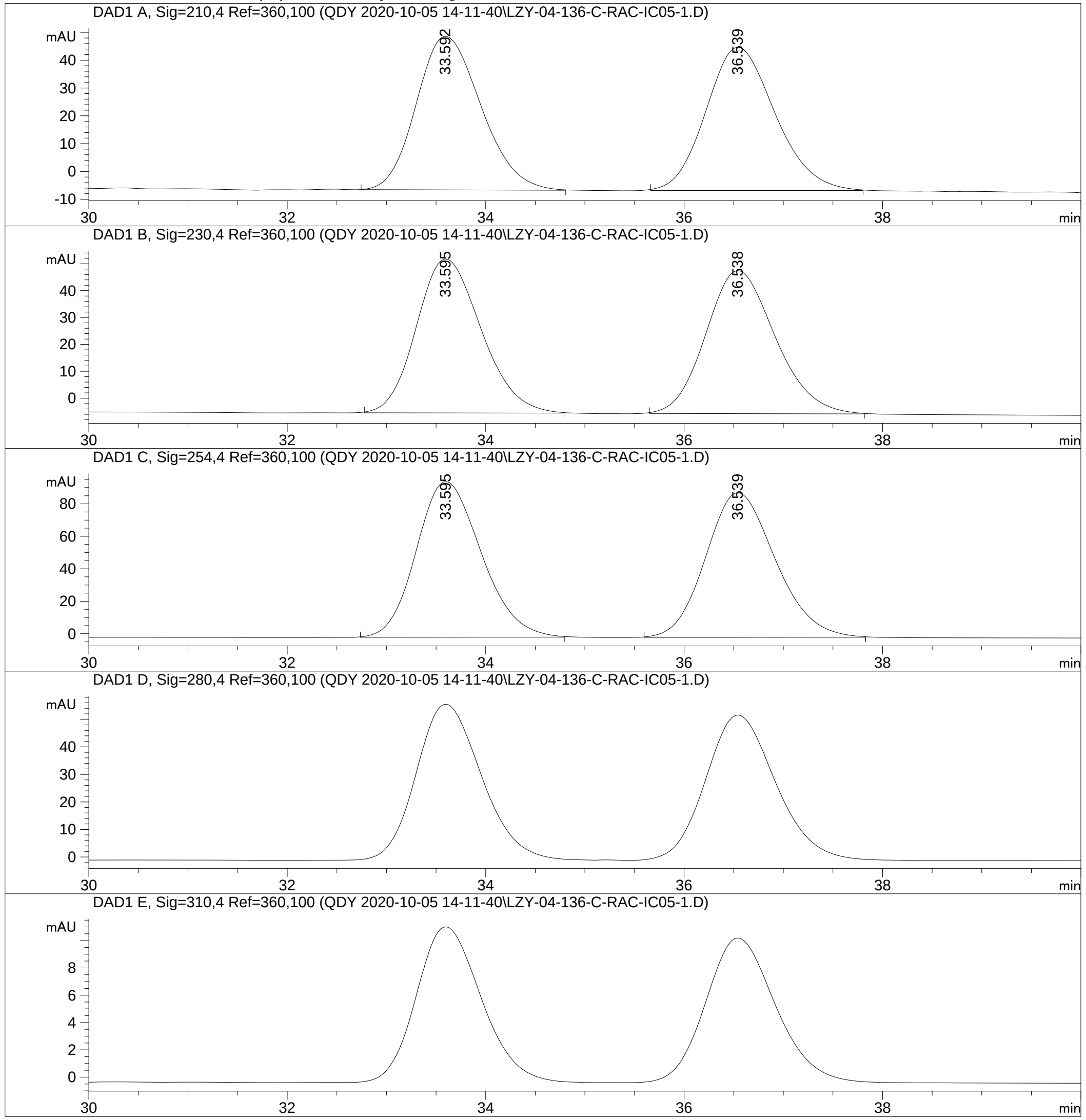

Data File C:\CHEM32\1\DATA\QDY 2020-10-05 14-11-40\LZY-04-136-C-RAC-IC05-1.D
Sample Name:

===
Area Percent Report
===

Sorted By   :   Signal
Multiplier   :   1.0000
Dilution   :   1.0000
Use Multiplier & Dilution Factor with ISTDs

Signal 1: DAD1 A, Sig=210,4 Ref=360,100

Peak #	RetTime [min]	Type	Width [min]	Area [mAU*s]	Height [mAU]	Area %
1	33.592	BB	0.7064	2499.83350	55.19099	50.0623
2	36.539	BB	0.7406	2493.61401	51.52316	49.9377

Totals :   4993.44751 106.71415

Signal 2: DAD1 B, Sig=230,4 Ref=360,100

Peak #	RetTime [min]	Type	Width [min]	Area [mAU*s]	Height [mAU]	Area %
1	33.595	BB	0.7006	2594.34106	57.26112	50.0698
2	36.538	BB	0.7453	2587.10742	53.20451	49.9302

Totals :   5181.44849 110.46564

Signal 3: DAD1 C, Sig=254,4 Ref=360,100

Peak #	RetTime [min]	Type	Width [min]	Area [mAU*s]	Height [mAU]	Area %
1	33.595	BB	0.7053	4348.84619	95.85885	50.0407
2	36.539	BB	0.7528	4341.76563	89.05508	49.9593

Totals :   8690.61182 184.91393

Signal 4: DAD1 D, Sig=280,4 Ref=360,100

Signal 5: DAD1 E, Sig=310,4 Ref=360,100

===
*** End of Report ***

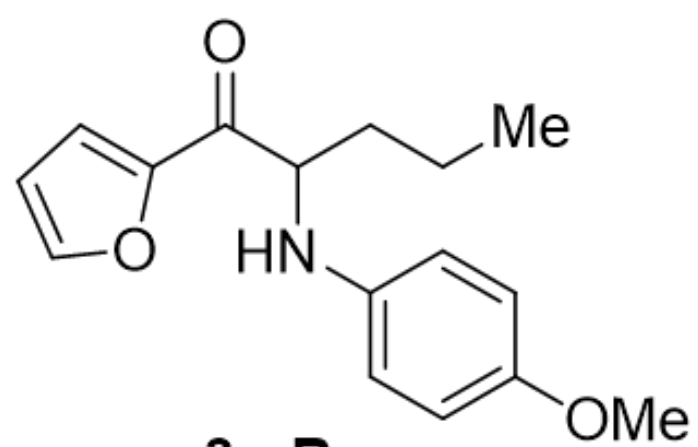

3g-Rac

```
=====================================================================
Acq. Operator   :                          Seq. Line :   5
Acq. Instrument : Instrument 1              Location : Vial 81
Injection Date  : 10/6/2020 8:10:03 PM           Inj :   1
                                          Inj Volume : 5.000 µl
Different Inj Volume from Sequence !   Actual Inj Volume : 4.000 µl
Acq. Method     : C:\CHEM32\1\DATA\QDY 2020-10-06 19-14-03\IC-05-40.M
Last changed    : 10/6/2020 8:09:10 PM
                  (modified after loading)
Analysis Method : C:\CHEM32\1\METHODS\OD-015-40.M
Last changed    : 11/4/2020 9:21:37 PM
                  (modified after loading)
Additional Info : Peak(s) manually integrated
```

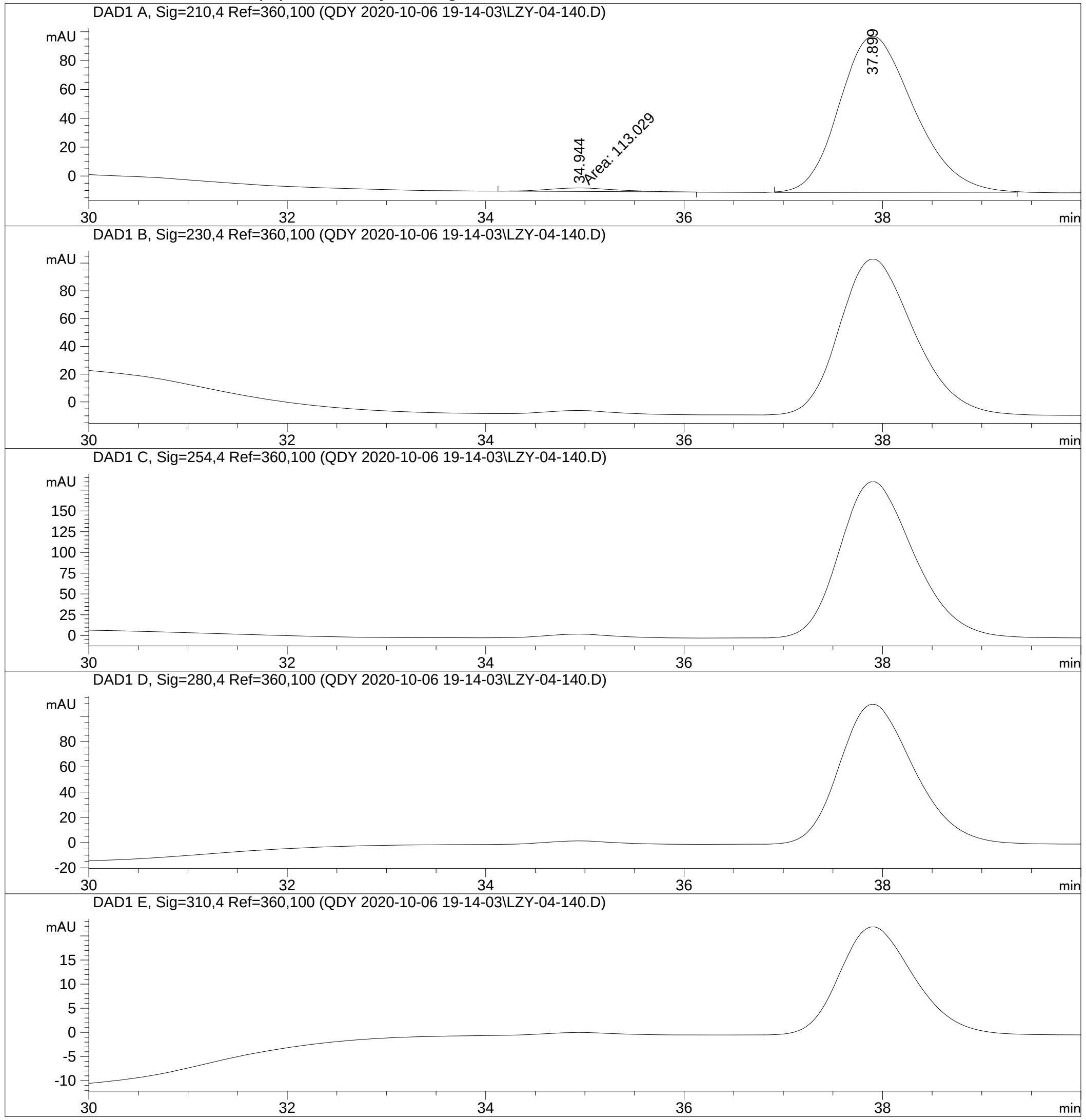

Data File C:\CHEM32\1\DATA\QDY 2020-10-06 19-14-03\LZY-04-140.D
Sample Name:

```
=====================================================================
                         Area Percent Report
=====================================================================

Sorted By             :      Signal
Multiplier            :      1.0000
Dilution              :      1.0000
Use Multiplier & Dilution Factor with ISTDs


Signal 1: DAD1 A, Sig=210,4 Ref=360,100

Peak RetTime Type  Width     Area      Height     Area
  #   [min]        [min]   [mAU*s]     [mAU]        %
----|-------|----|-------|----------|----------|--------|
   1  34.944 MM    0.7574  113.02866    2.48717   1.9548
   2  37.899 BB    0.8039 5668.94629  108.08791  98.0452

Totals :                  5781.97495  110.57508



Signal 2: DAD1 B, Sig=230,4 Ref=360,100


Signal 3: DAD1 C, Sig=254,4 Ref=360,100


Signal 4: DAD1 D, Sig=280,4 Ref=360,100


Signal 5: DAD1 E, Sig=310,4 Ref=360,100

=====================================================================
                          *** End of Report ***
```

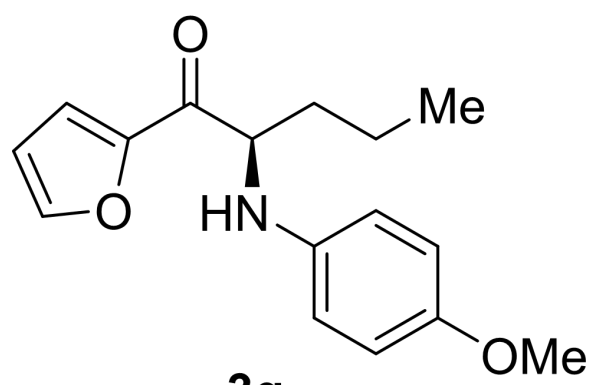

3g

```
=====================================================================
Acq. Operator   :                               Seq. Line :  18
Acq. Instrument : Instrument 1                   Location : Vial 83
Injection Date  : 10/9/2020 4:01:56 PM                Inj :   1
                                               Inj Volume : 5.000 µl
Different Inj Volume from Sequence !     Actual Inj Volume : 2.000 µl
Acq. Method     : C:\CHEM32\1\DATA\QDY 2020-10-09 09-27-26\AD-07-35.M
Last changed    : 7/14/2016 2:19:30 PM
Analysis Method : C:\CHEM32\1\METHODS\IC-007-90.M
Last changed    : 11/25/2020 7:39:16 PM
                  (modified after loading)
Additional Info : Peak(s) manually integrated
```

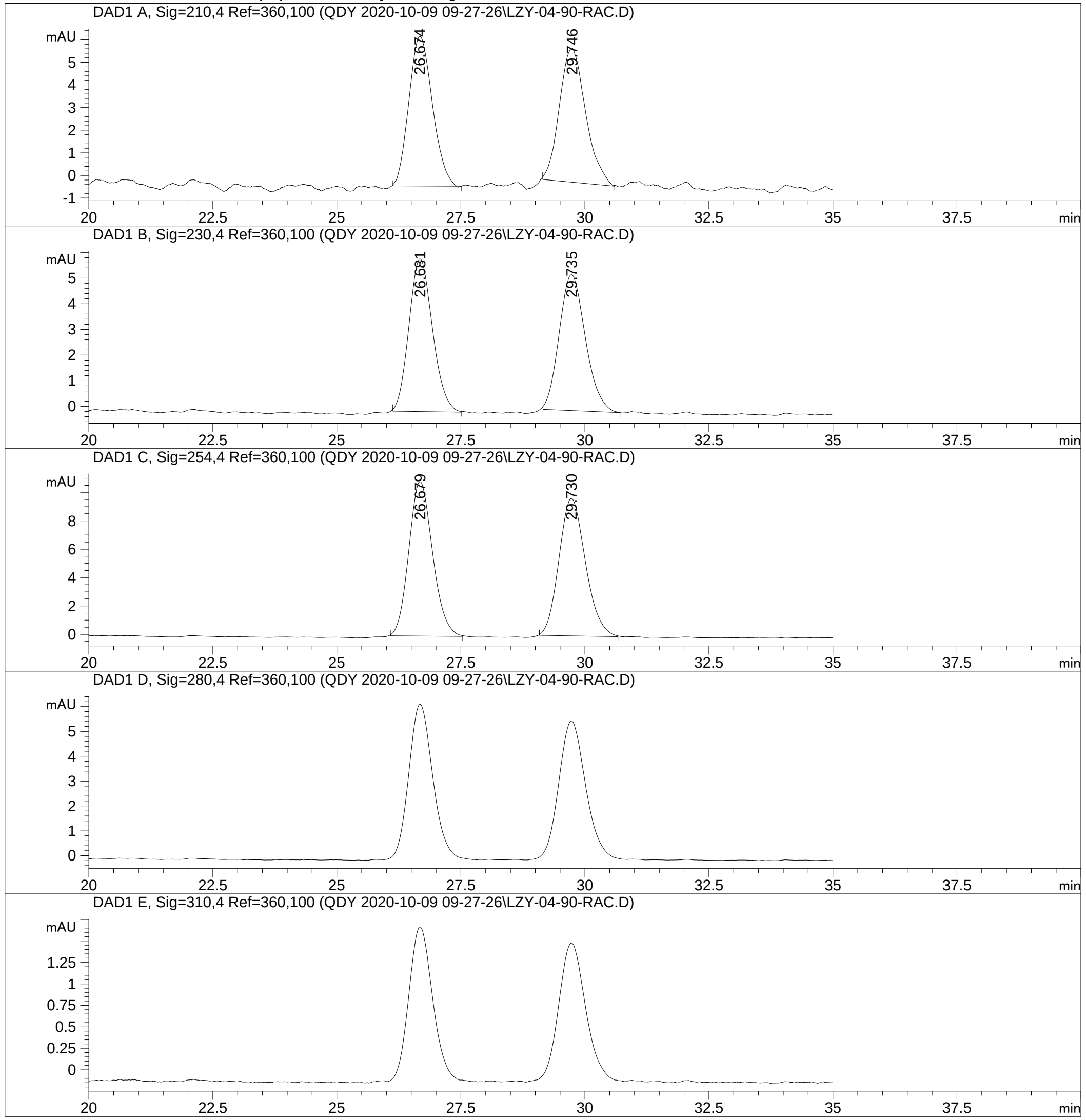

Data File C:\CHEM32\1\DATA\QDY 2020-10-09 09-27-26\LZY-04-90-RAC.D
Sample Name:

```
=====================================================================
                         Area Percent Report
=====================================================================

Sorted By             :      Signal
Multiplier            :      1.0000
Dilution              :      1.0000
Use Multiplier & Dilution Factor with ISTDs
```

Signal 1: DAD1 A, Sig=210,4 Ref=360,100

Peak #	RetTime [min]	Type	Width [min]	Area [mAU*s]	Height [mAU]	Area %
1	26.674	BB	0.4946	218.13307	6.61689	50.1290
2	29.746	BB	0.5047	217.01060	5.93290	49.8710

Totals : 435.14368 12.54979

Signal 2: DAD1 B, Sig=230,4 Ref=360,100

Peak #	RetTime [min]	Type	Width [min]	Area [mAU*s]	Height [mAU]	Area %
1	26.681	BB	0.5009	196.34564	5.95139	50.2654
2	29.735	BB	0.5740	194.27249	5.29573	49.7346

Totals : 390.61813 11.24711

Signal 3: DAD1 C, Sig=254,4 Ref=360,100

Peak #	RetTime [min]	Type	Width [min]	Area [mAU*s]	Height [mAU]	Area %
1	26.679	BB	0.5078	361.79019	10.88294	50.1858
2	29.730	BB	0.5728	359.11096	9.67953	49.8142

Totals : 720.90115 20.56247

Signal 4: DAD1 D, Sig=280,4 Ref=360,100

Signal 5: DAD1 E, Sig=310,4 Ref=360,100

```
=====================================================================
                         *** End of Report ***
```

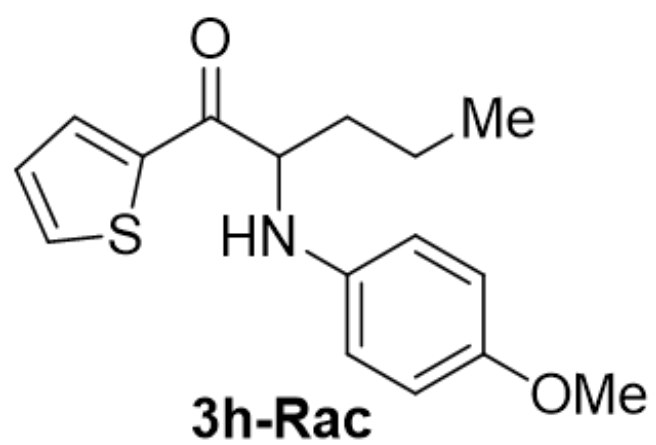

3h-Rac

===

Acq. Operator :
Seq. Line : 17
Acq. Instrument : Instrument 1
Location : Vial 82
Injection Date : 10/9/2020 3:25:58 PM
Inj : 1
Inj Volume : 5.000 µl
Different Inj Volume from Sequence ! Actual Inj Volume : 2.000 µl
Acq. Method : C:\CHEM32\1\DATA\QDY 2020-10-09 09-27-26\AD-07-35.M
Last changed : 7/14/2016 2:19:30 PM
Analysis Method : C:\CHEM32\1\METHODS\IC-007-90.M
Last changed : 11/25/2020 7:39:16 PM
(modified after loading)
Additional Info : Peak(s) manually integrated

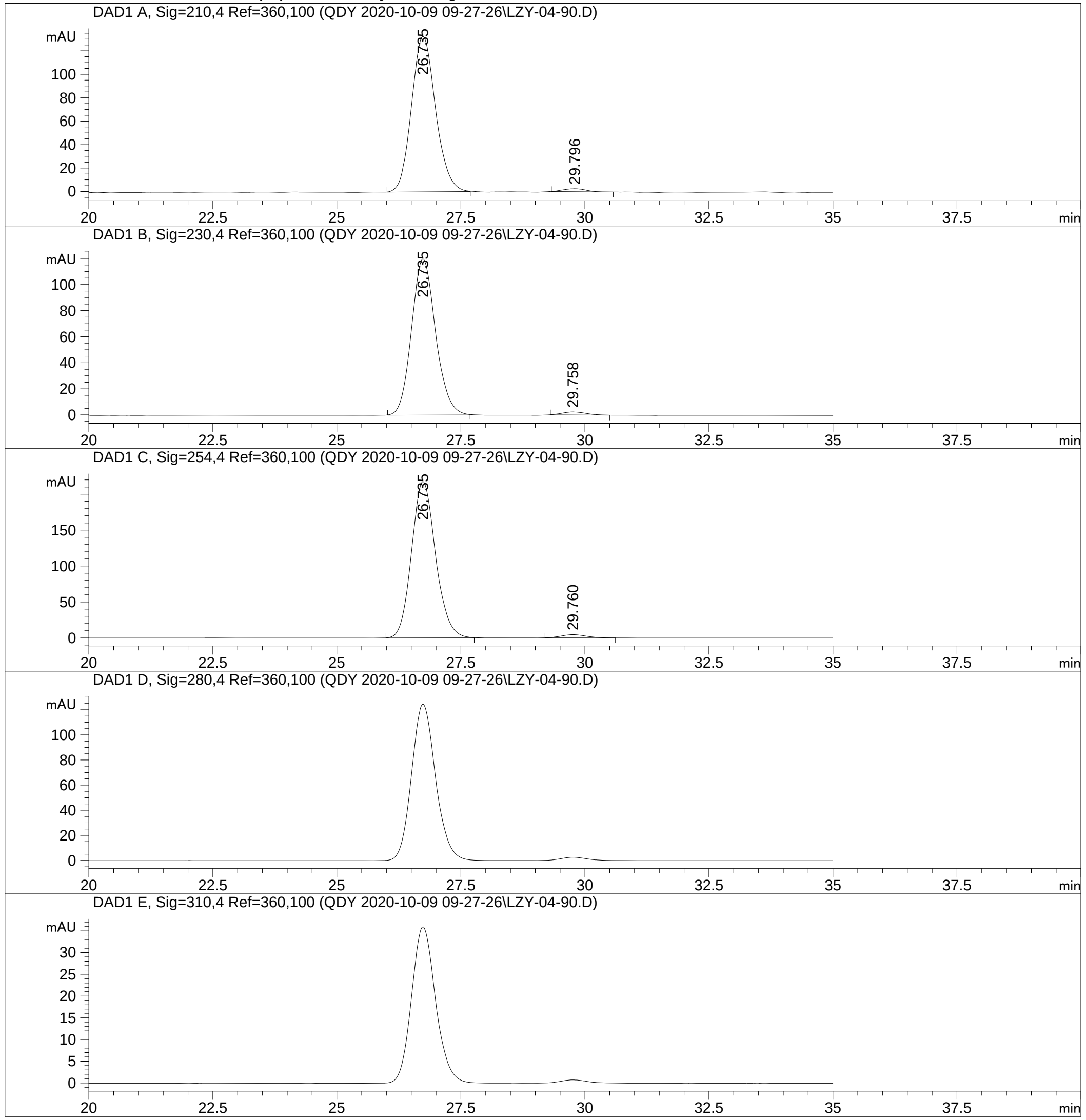

Data File C:\CHEM32\1\DATA\QDY 2020-10-09 09-27-26\LZY-04-90.D
Sample Name:

===

Area Percent Report

===

Sorted By : Signal
Multiplier : 1.0000
Dilution : 1.0000
Use Multiplier & Dilution Factor with ISTDs

Signal 1: DAD1 A, Sig=210,4 Ref=360,100

Peak #	RetTime [min]	Type	Width [min]	Area [mAU*s]	Height [mAU]	Area %
1	26.735	BB	0.5194	4472.19922	132.63557	98.2737
2	29.796	BB	0.3898	78.55873	2.53348	1.7263

Totals : 4550.75795 135.16905

Signal 2: DAD1 B, Sig=230,4 Ref=360,100

Peak #	RetTime [min]	Type	Width [min]	Area [mAU*s]	Height [mAU]	Area %
1	26.735	BB	0.5179	4022.17114	119.74784	98.0852
2	29.758	BB	0.4882	78.51913	2.34764	1.9148

Totals : 4100.69028 122.09548

Signal 3: DAD1 C, Sig=254,4 Ref=360,100

Peak #	RetTime [min]	Type	Width [min]	Area [mAU*s]	Height [mAU]	Area %
1	26.735	BB	0.5182	7315.55371	217.60095	97.8514
2	29.760	BB	0.5450	160.63394	4.49434	2.1486

Totals : 7476.18765 222.09530

Signal 4: DAD1 D, Sig=280,4 Ref=360,100

Signal 5: DAD1 E, Sig=310,4 Ref=360,100

===

*** End of Report ***

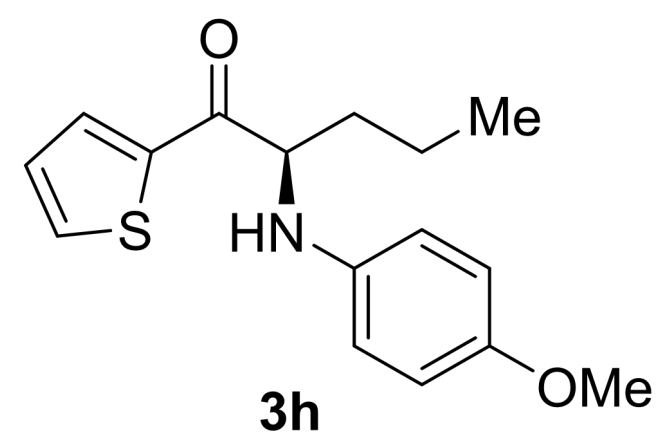

3h

```
Data File C:\CHEM32\1\DATA\QDY 2020-11-06 10-02-06\LZY-04-101-RAC.D
Sample Name:

    =====================================================================
    Acq. Operator   :                             Seq. Line :   3
    Acq. Instrument : Instrument 1                  Location : Vial 84
    Injection Date  : 11/6/2020 10:56:13 AM              Inj :   1
                                                  Inj Volume : 5.000 µl
    Different Inj Volume from Sequence !     Actual Inj Volume : 2.000 µl
    Acq. Method     : C:\CHEM32\1\DATA\QDY 2020-11-06 10-02-06\IC-03-40.M
    Last changed    : 11/6/2020 10:14:22 AM
                      (modified after loading)
    Analysis Method : C:\CHEM32\1\METHODS\IC-007-90.M
    Last changed    : 11/25/2020 7:43:25 PM
                      (modified after loading)
    Additional Info : Peak(s) manually integrated
```

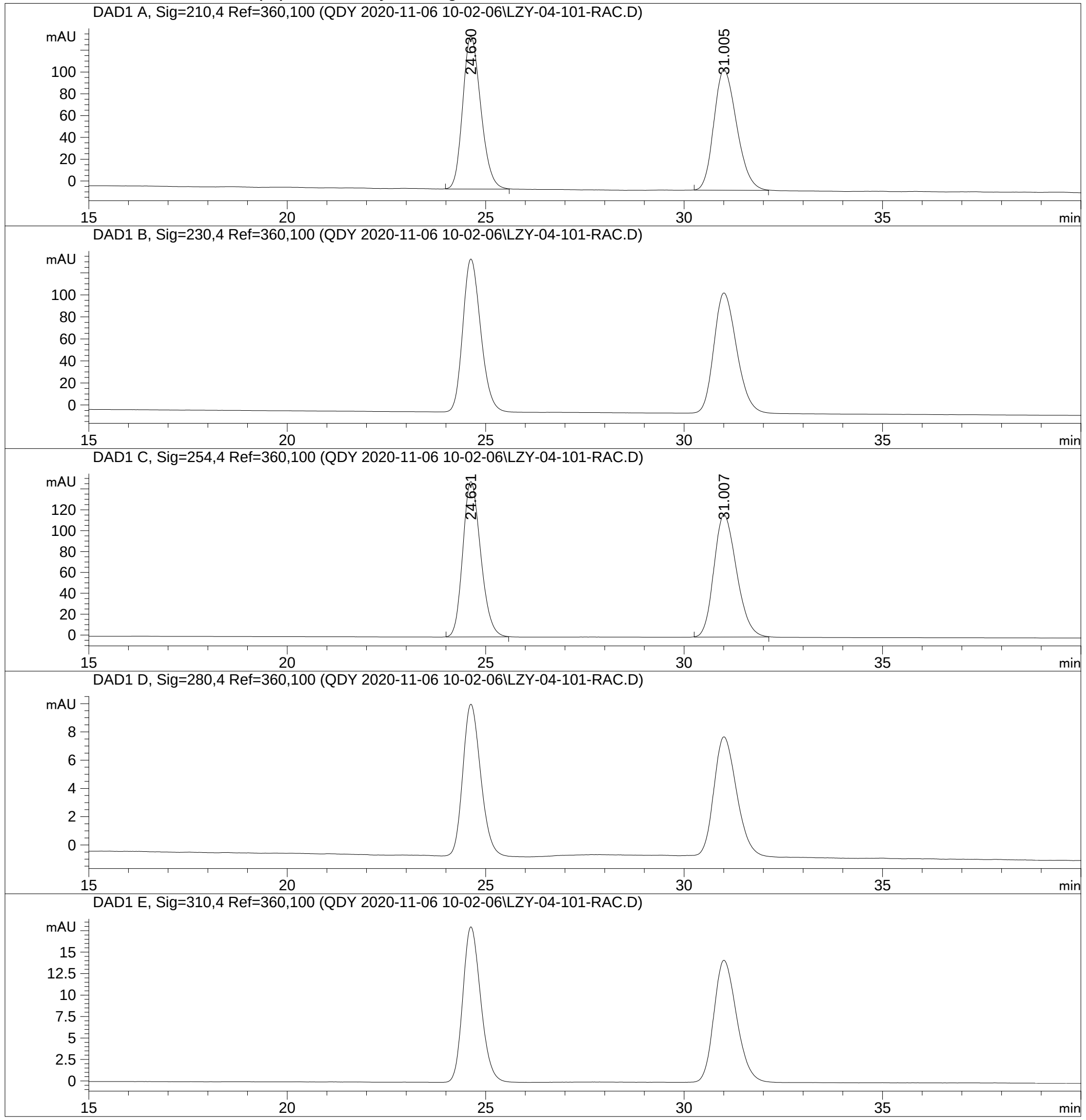


```
Data File C:\CHEM32\1\DATA\QDY 2020-11-06 10-02-06\LZY-04-101-RAC.D
Sample Name:

    =====================================================================
                              Area Percent Report
    =====================================================================

    Sorted By             :      Signal
    Multiplier            :      1.0000
    Dilution              :      1.0000
    Use Multiplier & Dilution Factor with ISTDs


    Signal 1: DAD1 A, Sig=210,4 Ref=360,100

    Peak RetTime Type  Width     Area      Height     Area
      #   [min]        [min]   [mAU*s]     [mAU]        %
    ----|-------|----|-------|----------|----------|--------|
       1  24.630 BB    0.4931 4433.40723  140.11790  50.0508
       2  31.005 BB    0.6247 4424.40723  110.60483  49.9492

    Totals :                  8857.81445  250.72273



    Signal 2: DAD1 B, Sig=230,4 Ref=360,100


    Signal 3: DAD1 C, Sig=254,4 Ref=360,100

    Peak RetTime Type  Width     Area      Height     Area
      #   [min]        [min]   [mAU*s]     [mAU]        %
    ----|-------|----|-------|----------|----------|--------|
       1  24.631 BB    0.4861 4669.93555  148.81953  50.0454
       2  31.007 BB    0.6183 4661.45605  117.12613  49.9546

    Totals :                  9331.39160  265.94566



    Signal 4: DAD1 D, Sig=280,4 Ref=360,100


    Signal 5: DAD1 E, Sig=310,4 Ref=360,100

    =====================================================================
                             *** End of Report ***
```

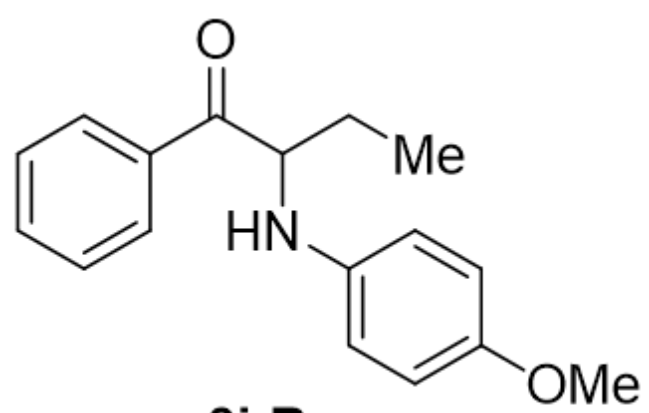

3i-Rac

Data File C:\CHEM32\1\DATA\QDY 2020-09-17 21-53-42\LZY-04-101.D
Sample Name:

```
=====================================================================
Acq. Operator   :                               Seq. Line :   2
Acq. Instrument : Instrument 1                   Location : Vial 81
Injection Date  : 9/17/2020 10:06:34 PM               Inj :   1
                                               Inj Volume : 5.000 µl
Different Inj Volume from Sequence !     Actual Inj Volume : 2.000 µl
Acq. Method     : C:\CHEM32\1\DATA\QDY 2020-09-17 21-53-42\IC-03-40.M
Last changed    : 9/17/2020 10:05:42 PM
                  (modified after loading)
Analysis Method : C:\CHEM32\1\DATA\QDY 2020-11-05 11-21-39\OD-20-10.M
Last changed    : 11/6/2020 2:53:59 PM
                  (modified after loading)
Additional Info : Peak(s) manually integrated
```

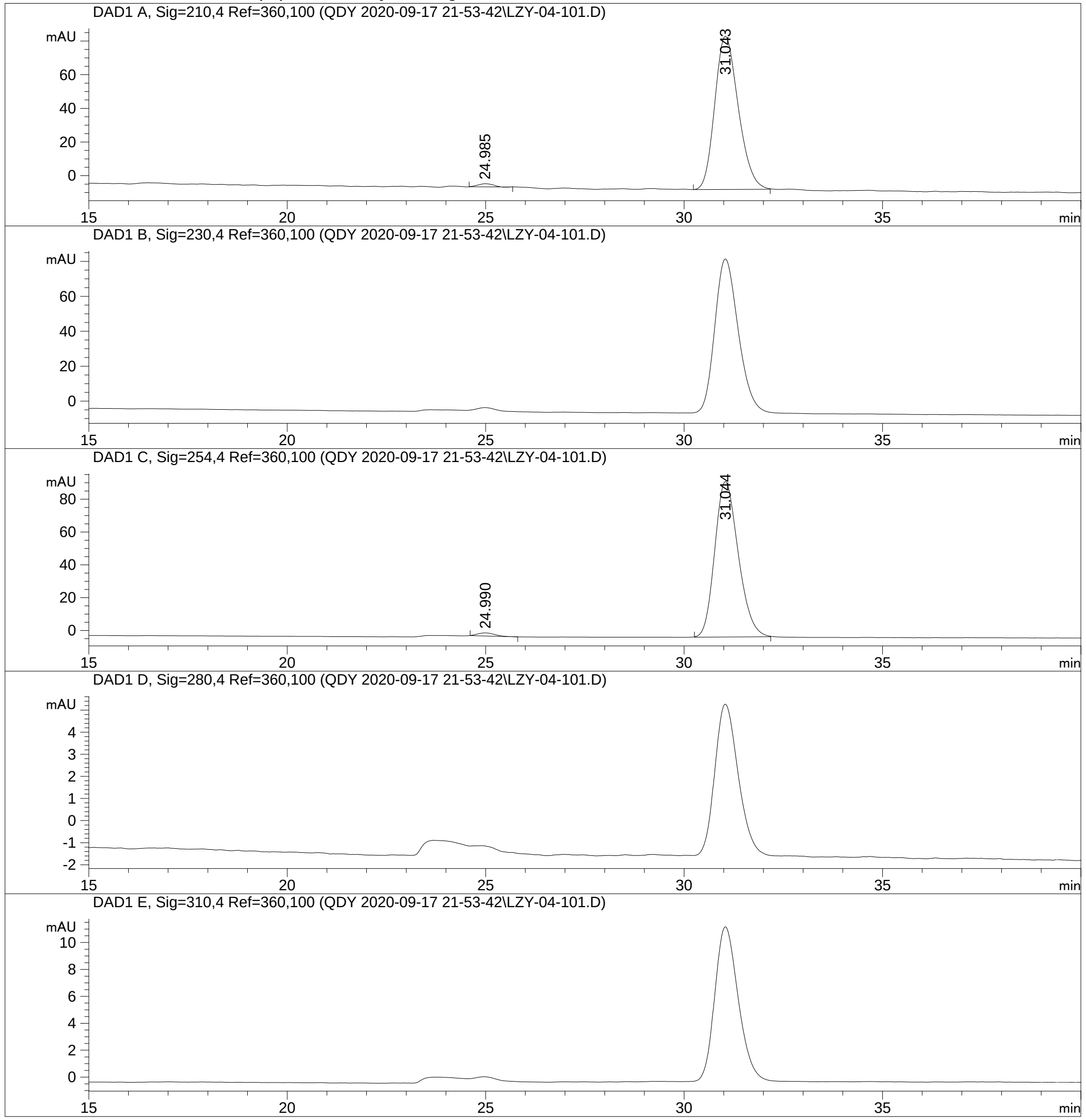

Data File C:\CHEM32\1\DATA\QDY 2020-09-17 21-53-42\LZY-04-101.D
Sample Name:

===

Area Percent Report

===

Sorted By : Signal
Multiplier : 1.0000
Dilution : 1.0000
Use Multiplier & Dilution Factor with ISTDs

Signal 1: DAD1 A, Sig=210,4 Ref=360,100

Peak #	RetTime [min]	Type	Width [min]	Area [mAU*s]	Height [mAU]	Area %
1	24.985	BB	0.3141	47.09527	1.83078	1.2415
2	31.043	BB	0.6386	3746.42212	90.95081	98.7585

Totals : 3793.51739 92.78158

Signal 2: DAD1 B, Sig=230,4 Ref=360,100

Signal 3: DAD1 C, Sig=254,4 Ref=360,100

Peak #	RetTime [min]	Type	Width [min]	Area [mAU*s]	Height [mAU]	Area %
1	24.990	BB	0.4622	56.00177	1.92060	1.4178
2	31.044	BB	0.6358	3893.80688	94.67651	98.5822

Totals : 3949.80865 96.59711

Signal 4: DAD1 D, Sig=280,4 Ref=360,100

Signal 5: DAD1 E, Sig=310,4 Ref=360,100

===

*** End of Report ***

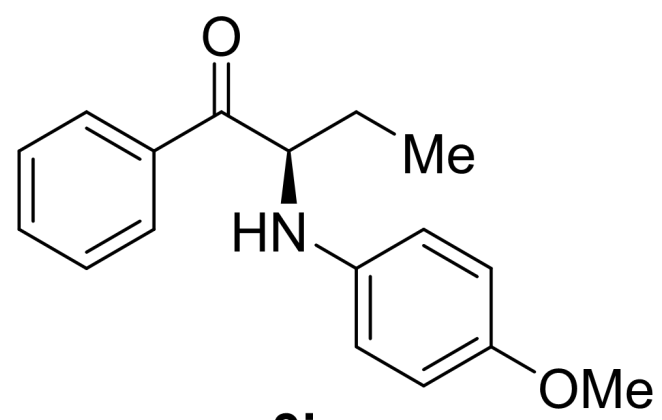

3i

===

Acq. Operator : Seq. Line : 24
Acq. Instrument : Instrument 1 Location : Vial 55
Injection Date : 4/4/2020 9:21:30 PM Inj : 1
Inj Volume : 5.000 µl
Different Inj Volume from Sequence ! Actual Inj Volume : 3.000 µl
Acq. Method : C:\CHEM32\1\DATA\QDY 2020-04-04 12-37-36\IC-05-40.M
Last changed : 4/4/2020 9:47:54 PM
(modified after loading)
Analysis Method : C:\CHEM32\1\METHODS\IC-007-90.M
Last changed : 11/26/2020 2:59:57 PM
(modified after loading)
Additional Info : Peak(s) manually integrated

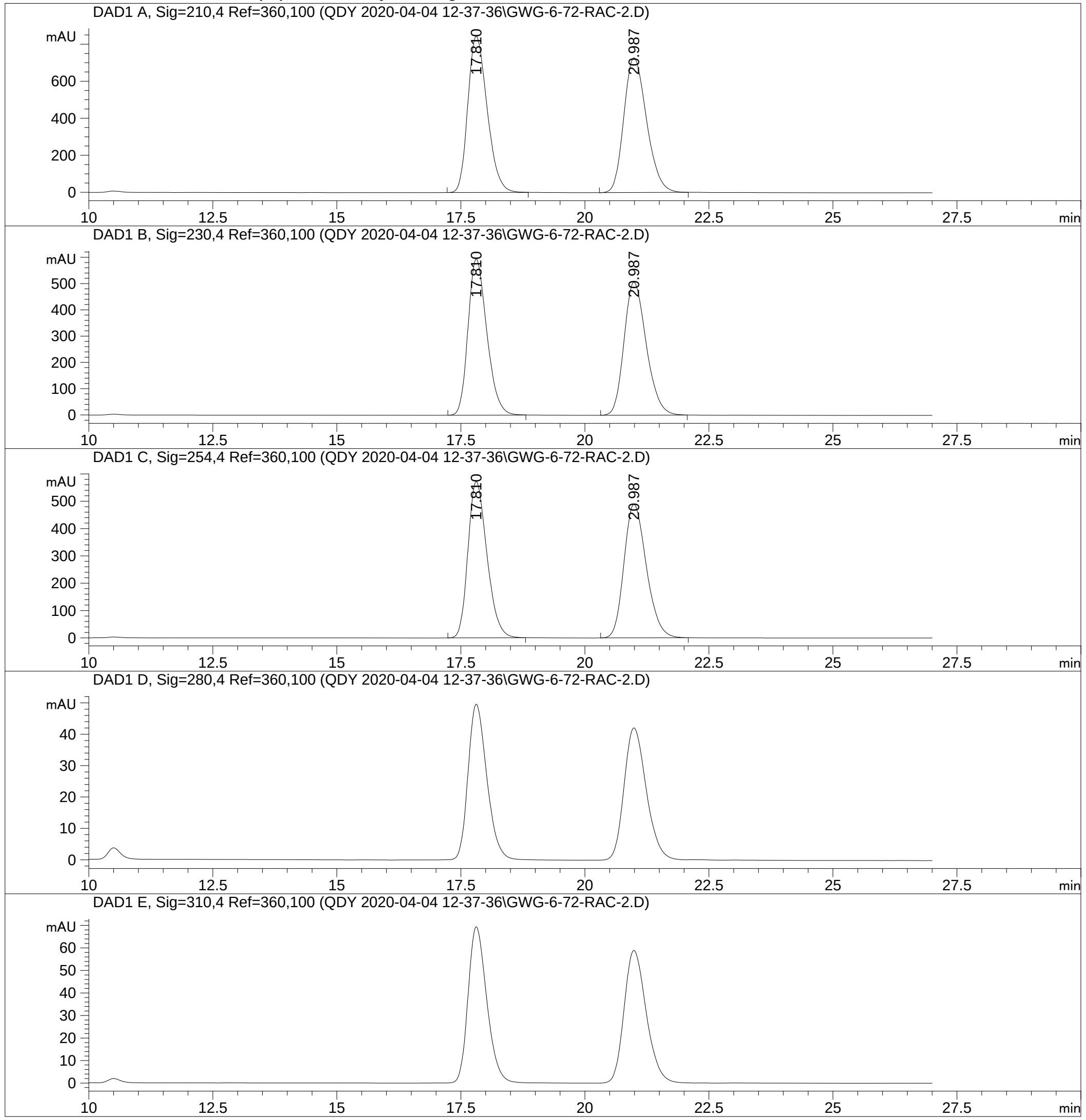

Data File C:\CHEM32\1\DATA\QDY 2020-04-04 12-37-36\GWG-6-72-RAC-2.D
Sample Name:

===

Area Percent Report

===

Sorted By : Signal
Multiplier : 1.0000
Dilution : 1.0000
Use Multiplier & Dilution Factor with ISTDs

Signal 1: DAD1 A, Sig=210,4 Ref=360,100

Peak #	RetTime [min]	Type	Width [min]	Area [mAU*s]	Height [mAU]	Area %
1	17.810	BB	0.4198	2.28343e4	843.63538	49.6495
2	20.987	BB	0.4973	2.31568e4	727.70294	50.3505

Totals : 4.59911e4 1571.33832

Signal 2: DAD1 B, Sig=230,4 Ref=360,100

Peak #	RetTime [min]	Type	Width [min]	Area [mAU*s]	Height [mAU]	Area %
1	17.810	BB	0.4070	1.56405e4	594.43054	49.8395
2	20.987	BB	0.4849	1.57412e4	506.00049	50.1605

Totals : 3.13817e4 1100.43103

Signal 3: DAD1 C, Sig=254,4 Ref=360,100

Peak #	RetTime [min]	Type	Width [min]	Area [mAU*s]	Height [mAU]	Area %
1	17.810	BB	0.4117	1.51822e4	572.00317	49.7486
2	20.987	BB	0.4878	1.53357e4	489.04858	50.2514

Totals : 3.05179e4 1061.05176

Signal 4: DAD1 D, Sig=280,4 Ref=360,100

Signal 5: DAD1 E, Sig=310,4 Ref=360,100

===

*** End of Report ***

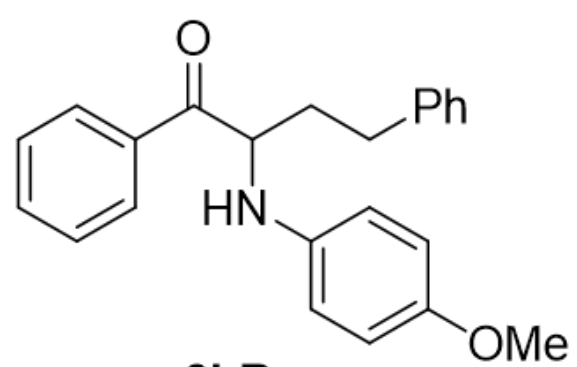

3j-Rac

Data File C:\CHEM32\1\DATA\QDY 2020-09-26 09-35-58\LZY-04-125-A.D
Sample Name:

===

Acq. Operator : Seq. Line : 64
Acq. Instrument : Instrument 1 Location : Vial 81
Injection Date : 9/27/2020 10:23:21 AM Inj : 1
Inj Volume : 5.000 µl
Different Inj Volume from Sequence ! Actual Inj Volume : 2.000 µl
Acq. Method : C:\CHEM32\1\DATA\QDY 2020-09-26 09-35-58\IC-05-30.M
Last changed : 8/23/2012 1:59:23 PM
Analysis Method : C:\CHEM32\1\METHODS\IC-007-90.M
Last changed : 11/25/2020 8:27:08 PM
(modified after loading)
Additional Info : Peak(s) manually integrated

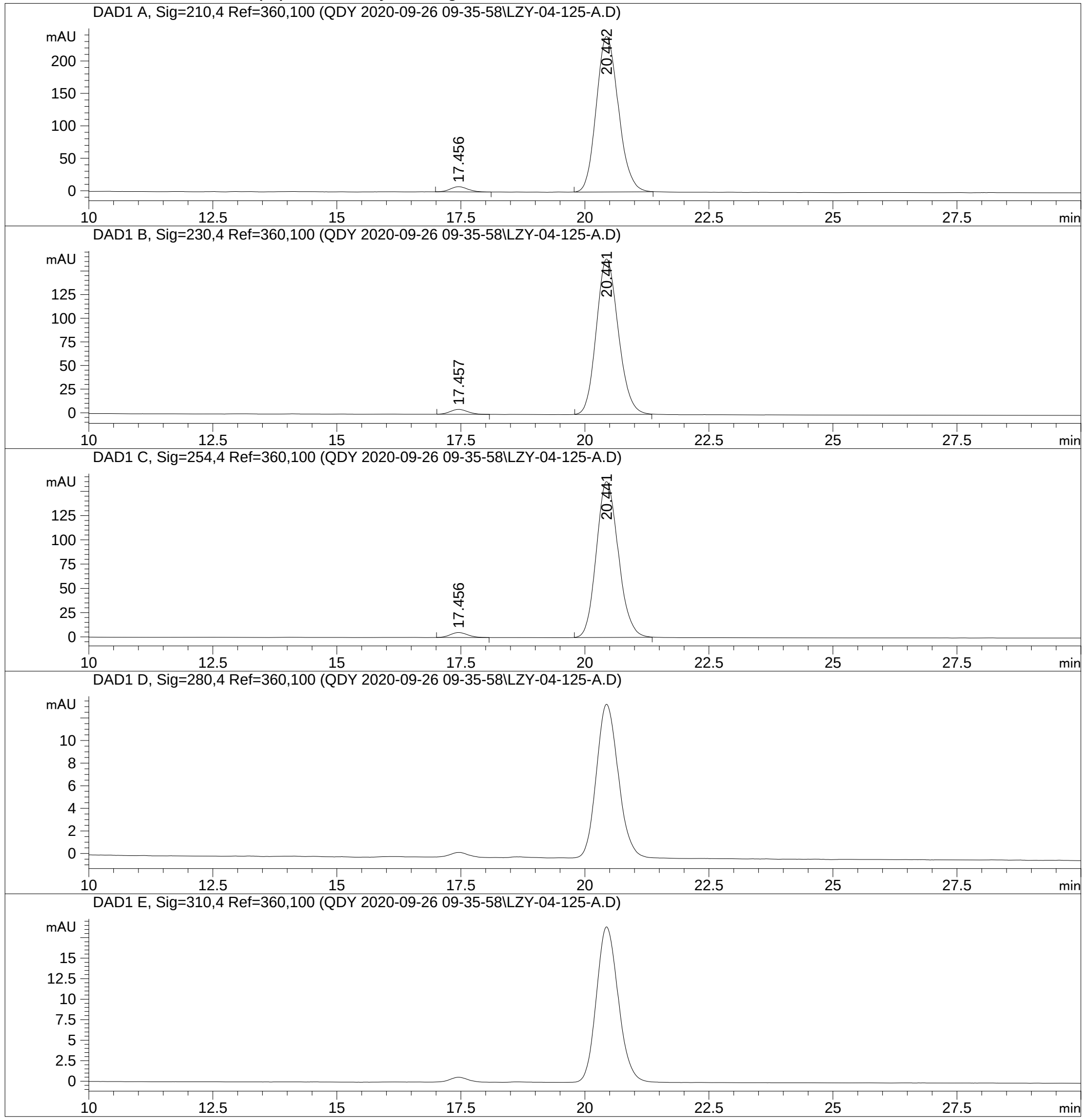

Data File C:\CHEM32\1\DATA\QDY 2020-09-26 09-35-58\LZY-04-125-A.D
Sample Name:

===

Area Percent Report

===

Sorted By : Signal
Multiplier : 1.0000
Dilution : 1.0000
Use Multiplier & Dilution Factor with ISTDs

Signal 1: DAD1 A, Sig=210,4 Ref=360,100

Peak #	RetTime [min]	Type	Width [min]	Area [mAU*s]	Height [mAU]	Area %
1	17.456	BB	0.3784	193.53168	8.05340	2.5470
2	20.442	BB	0.4804	7404.91406	239.70705	97.4530

Totals : 7598.44574 247.76045

Signal 2: DAD1 B, Sig=230,4 Ref=360,100

Peak #	RetTime [min]	Type	Width [min]	Area [mAU*s]	Height [mAU]	Area %
1	17.457	BB	0.3684	126.56236	5.22781	2.4489
2	20.441	BB	0.4756	5041.55469	164.49963	97.5511

Totals : 5168.11705 169.72744

Signal 3: DAD1 C, Sig=254,4 Ref=360,100

Peak #	RetTime [min]	Type	Width [min]	Area [mAU*s]	Height [mAU]	Area %
1	17.456	BB	0.3747	124.53020	5.13860	2.4607
2	20.441	BB	0.4765	4936.32861	160.64256	97.5393

Totals : 5060.85882 165.78117

Signal 4: DAD1 D, Sig=280,4 Ref=360,100

Signal 5: DAD1 E, Sig=310,4 Ref=360,100

===

*** End of Report ***

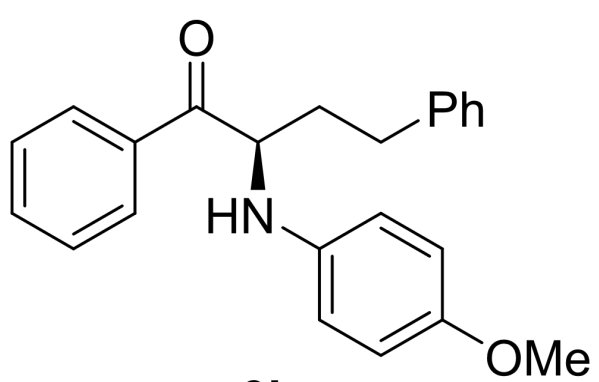

3j

Data File C:\CHEM32\1\DATA\QDY 2020-10-11 14-09-08\LZY-04-153-A-IC03-RAC.D
Sample Name:

===

Acq. Operator   :   Seq. Line :   2
Acq. Instrument : Instrument 1   Location : Vial 81
Injection Date : 10/11/2020 2:21:56 PM   Inj :   1
                                        Inj Volume : 5.000 µl
Different Inj Volume from Sequence !   Actual Inj Volume : 2.000 µl
Acq. Method   : C:\CHEM32\1\DATA\QDY 2020-10-11 14-09-08\IC-03-40.M
Last changed   : 10/11/2020 2:21:04 PM
                  (modified after loading)
Analysis Method : C:\CHEM32\1\METHODS\OD-015-40.M
Last changed   : 11/4/2020 10:47:42 PM
                  (modified after loading)
Additional Info : Peak(s) manually integrated

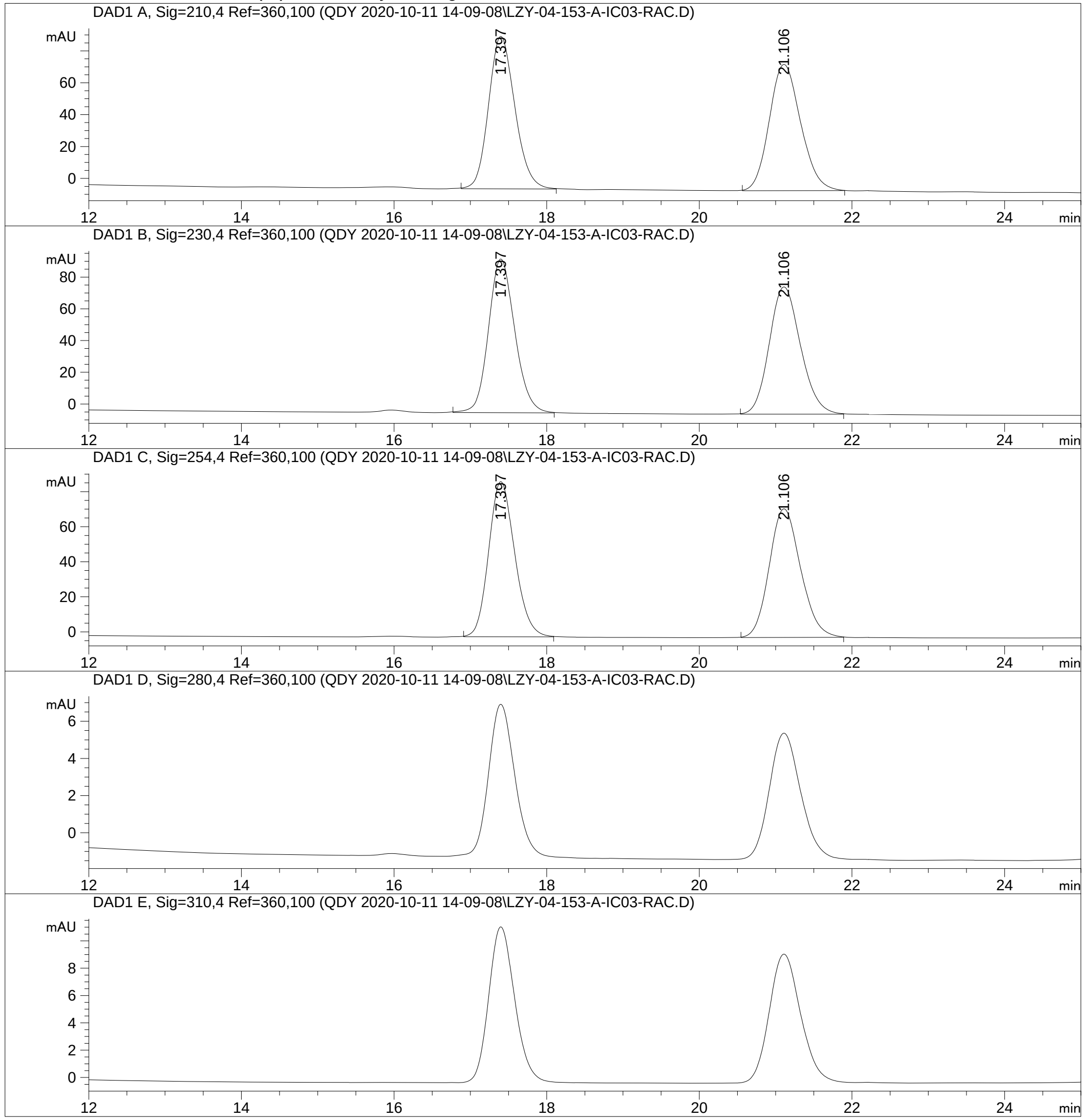

Data File C:\CHEM32\1\DATA\QDY 2020-10-11 14-09-08\LZY-04-153-A-IC03-RAC.D
Sample Name:

===
Area Percent Report
===

Sorted By   :   Signal
Multiplier   :   1.0000
Dilution   :   1.0000
Use Multiplier & Dilution Factor with ISTDs

Signal 1: DAD1 A, Sig=210,4 Ref=360,100

Peak #	RetTime [min]	Type	Width [min]	Area [mAU*s]	Height [mAU]	Area %
1	17.397	BB	0.3713	2290.18384	95.66989	50.2985
2	21.106	BB	0.4430	2263.00000	79.29379	49.7015

Totals :   4553.18384 174.96368

Signal 2: DAD1 B, Sig=230,4 Ref=360,100

Peak #	RetTime [min]	Type	Width [min]	Area [mAU*s]	Height [mAU]	Area %
1	17.397	BB	0.3685	2315.39551	96.98219	50.3006
2	21.106	BB	0.4423	2287.72559	80.31842	49.6994

Totals :   4603.12109 177.30061

Signal 3: DAD1 C, Sig=254,4 Ref=360,100

Peak #	RetTime [min]	Type	Width [min]	Area [mAU*s]	Height [mAU]	Area %
1	17.397	BB	0.3663	2097.34082	88.54610	50.0306
2	21.106	BB	0.4427	2094.77588	73.46728	49.9694

Totals :   4192.11670 162.01338

Signal 4: DAD1 D, Sig=280,4 Ref=360,100

Signal 5: DAD1 E, Sig=310,4 Ref=360,100

===
*** End of Report ***

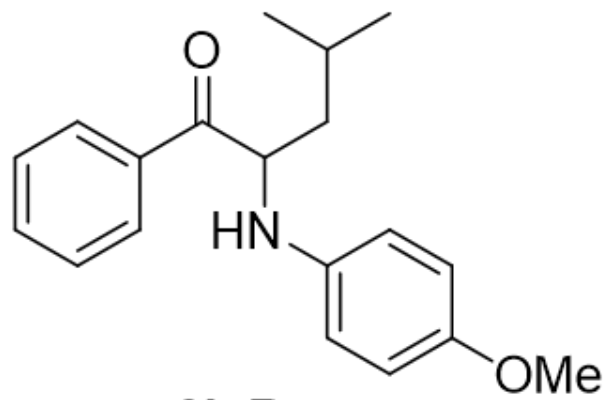

3k-Rac

Data File C:\CHEM32\1\DATA\QDY 2020-10-12 22-15-38\LZY-04-155.D
Sample Name:

===

Acq. Operator   :   Seq. Line :   5
Acq. Instrument : Instrument 1   Location : Vial 81
Injection Date : 10/12/2020 11:21:33 PM   Inj :   1
Inj Volume : 5.000 µl
Different Inj Volume from Sequence !   Actual Inj Volume : 2.000 µl
Acq. Method   : C:\CHEM32\1\DATA\QDY 2020-10-12 22-15-38\IC-03-30.M
Last changed   : 10/12/2020 11:20:40 PM
(modified after loading)
Analysis Method : C:\CHEM32\1\METHODS\OD-015-40.M
Last changed   : 11/4/2020 10:47:42 PM
(modified after loading)
Additional Info : Peak(s) manually integrated

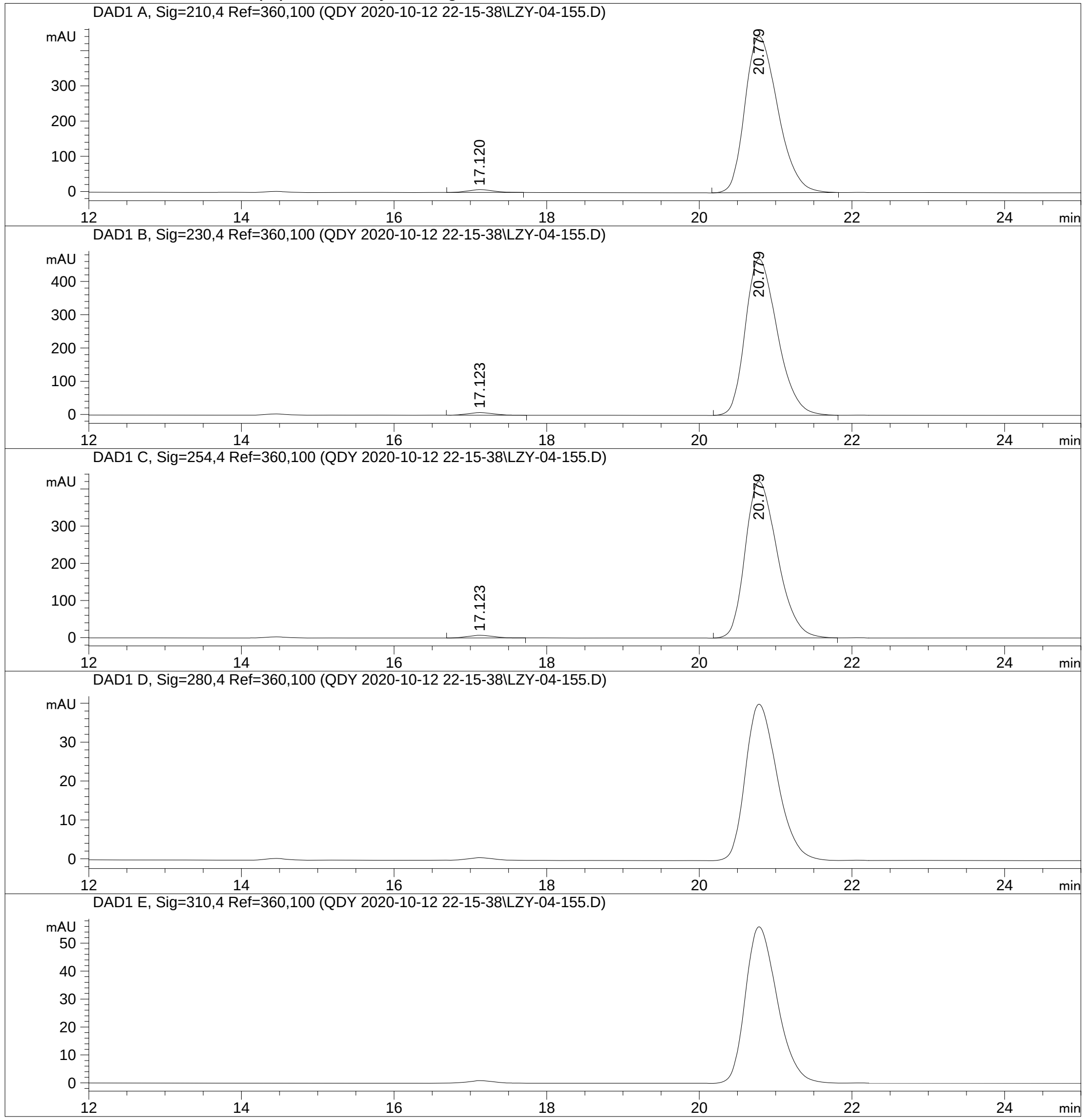

Data File C:\CHEM32\1\DATA\QDY 2020-10-12 22-15-38\LZY-04-155.D
Sample Name:

===

Area Percent Report

===

Sorted By   :   Signal
Multiplier   :   1.0000
Dilution   :   1.0000
Use Multiplier & Dilution Factor with ISTDs

Signal 1: DAD1 A, Sig=210,4 Ref=360,100

Peak #	RetTime [min]	Type	Width [min]	Area [mAU*s]	Height [mAU]	Area %
1	17.120	BB	0.3522	182.02367	7.97730	1.3589
2	20.779	BB	0.4633	1.32130e4	443.83066	98.6411

Totals :   1.33950e4   451.80795

Signal 2: DAD1 B, Sig=230,4 Ref=360,100

Peak #	RetTime [min]	Type	Width [min]	Area [mAU*s]	Height [mAU]	Area %
1	17.123	BB	0.3571	178.09796	7.83871	1.2870
2	20.779	BB	0.4511	1.36600e4	469.98758	98.7130

Totals :   1.38381e4   477.82629

Signal 3: DAD1 C, Sig=254,4 Ref=360,100

Peak #	RetTime [min]	Type	Width [min]	Area [mAU*s]	Height [mAU]	Area %
1	17.123	BB	0.3536	162.76181	7.20489	1.3032
2	20.779	BB	0.4539	1.23263e4	420.61856	98.6968

Totals :   1.24890e4   427.82345

Signal 4: DAD1 D, Sig=280,4 Ref=360,100

Signal 5: DAD1 E, Sig=310,4 Ref=360,100

===

*** End of Report ***

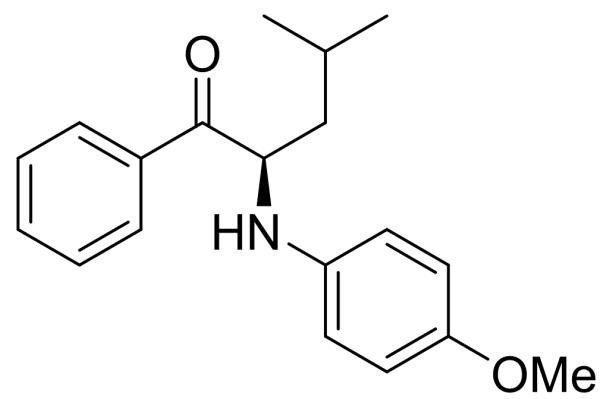

3k

Data File C:\CHEM32\1\DATA\QDY 2019-11-21 13-16-53\GWG-4-149-RACEMIC-2.D
Sample Name:

===

Acq. Operator : Seq. Line : 10
Acq. Instrument : Instrument 1 Location : Vial 51
Injection Date : 11/21/2019 4:39:30 PM Inj : 1
Inj Volume : 5.000 µl
Different Inj Volume from Sequence ! Actual Inj Volume : 3.000 µl
Acq. Method : C:\CHEM32\1\DATA\QDY 2019-11-21 13-16-53\OD-10-50.M
Last changed : 11/21/2019 6:02:19 PM
(modified after loading)
Analysis Method : C:\CHEM32\1\METHODS\IC-009-10-1.0.M
Last changed : 12/3/2019 2:03:28 PM
(modified after loading)
Additional Info : Peak(s) manually integrated

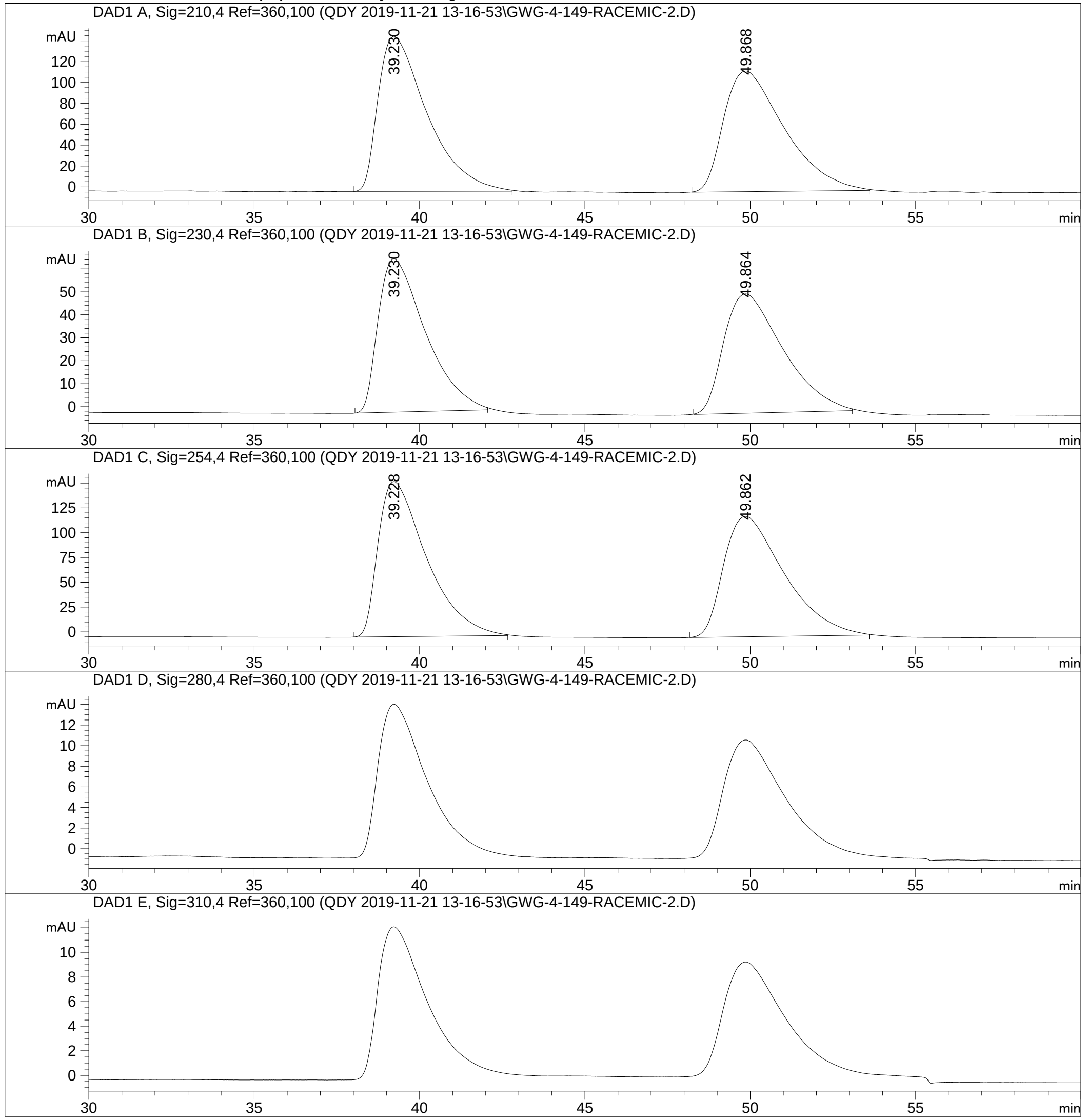

Data File C:\CHEM32\1\DATA\QDY 2019-11-21 13-16-53\GWG-4-149-RACEMIC-2.D
Sample Name:

===
Area Percent Report
===

Sorted By : Signal
Multiplier : 1.0000
Dilution : 1.0000
Use Multiplier & Dilution Factor with ISTDs

Signal 1: DAD1 A, Sig=210,4 Ref=360,100

Peak #	RetTime [min]	Type	Width [min]	Area [mAU*s]	Height [mAU]	Area %
1	39.230	BB	1.5388	1.52809e4	148.47108	50.8203
2	49.868	BB	1.7846	1.47876e4	115.58118	49.1797

Totals : 3.00685e4 264.05226

Signal 2: DAD1 B, Sig=230,4 Ref=360,100

Peak #	RetTime [min]	Type	Width [min]	Area [mAU*s]	Height [mAU]	Area %
1	39.230	BB	1.4501	6622.34912	66.71263	50.5784
2	49.864	BB	1.7513	6470.89795	51.94483	49.4216

Totals : 1.30932e4 118.65746

Signal 3: DAD1 C, Sig=254,4 Ref=360,100

Peak #	RetTime [min]	Type	Width [min]	Area [mAU*s]	Height [mAU]	Area %
1	39.228	BB	1.5160	1.59239e4	156.38461	50.7189
2	49.862	BB	1.9256	1.54725e4	121.34386	49.2811

Totals : 3.13963e4 277.72848

Signal 4: DAD1 D, Sig=280,4 Ref=360,100

Signal 5: DAD1 E, Sig=310,4 Ref=360,100

===
*** End of Report ***

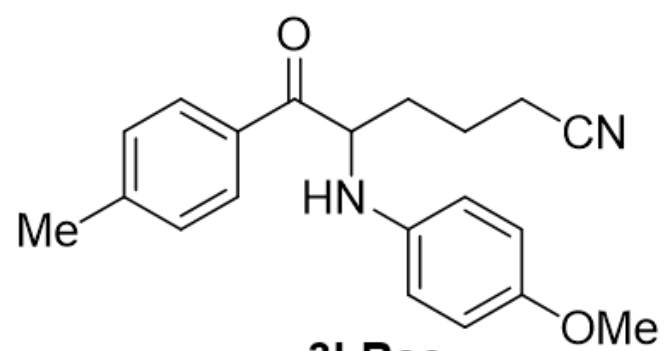

3l-Rac

===

Acq. Operator : Seq. Line : 2
Acq. Instrument : Instrument 1 Location : Vial 81
Injection Date : 3/13/2021 9:44:13 PM Inj : 1
Inj Volume : 5.000 µl
Different Inj Volume from Sequence ! Actual Inj Volume : 4.000 µl
Acq. Method : C:\CHEM32\1\DATA\QDY 2021-03-13 21-30-39\OD-10-60.M
Last changed : 3/13/2021 9:43:20 PM
(modified after loading)
Analysis Method : C:\CHEM32\1\METHODS\OD-20-10.M
Last changed : 3/13/2021 10:45:53 PM
(modified after loading)
Additional Info : Peak(s) manually integrated

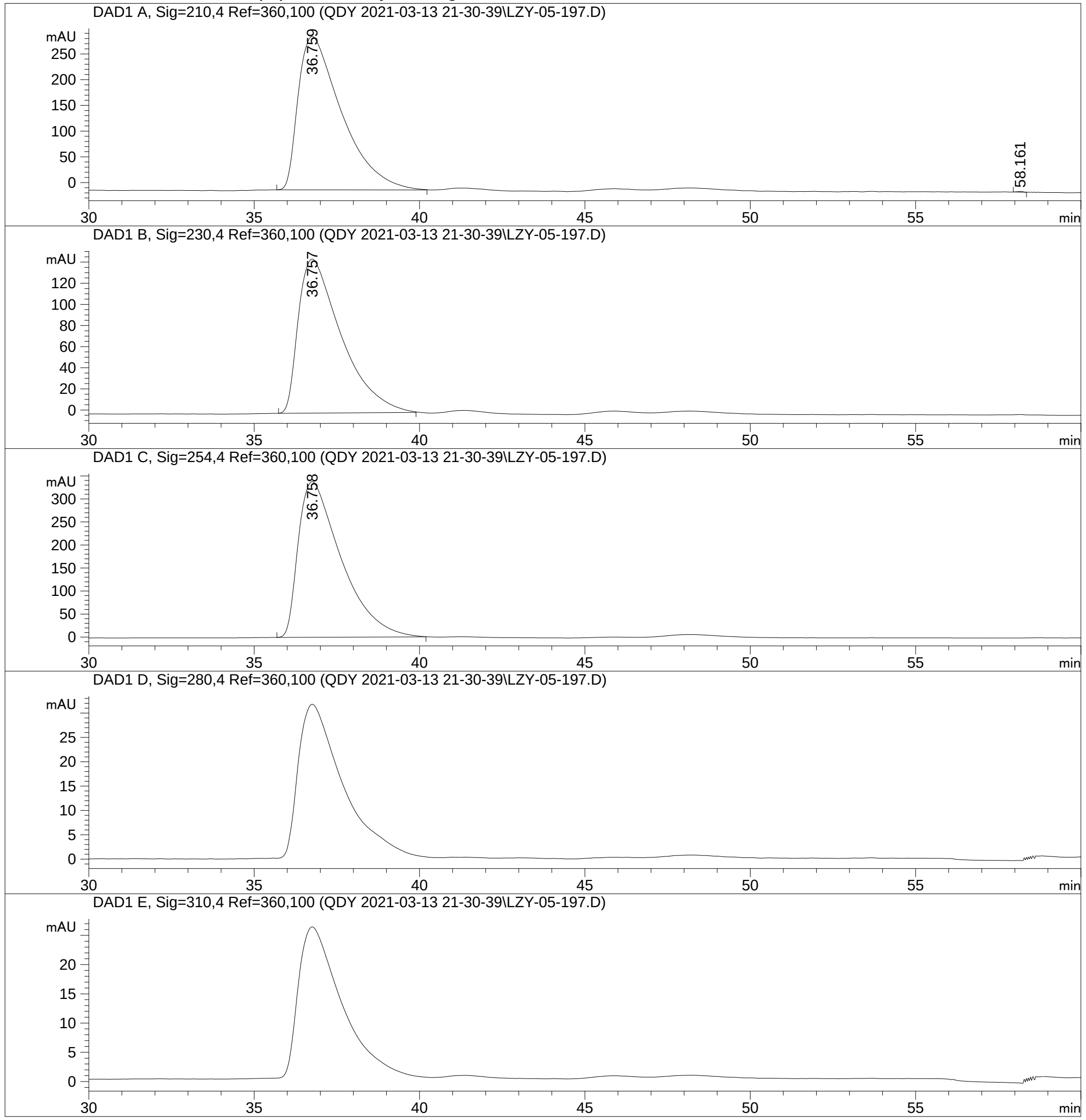

Data File C:\CHEM32\1\DATA\QDY 2021-03-13 21-30-39\LZY-05-197.D
Sample Name:

===

Area Percent Report

===

Sorted By : Signal
Multiplier : 1.0000
Dilution : 1.0000
Use Multiplier & Dilution Factor with ISTDs

Signal 1: DAD1 A, Sig=210,4 Ref=360,100

Peak #	RetTime [min]	Type	Width [min]	Area [mAU*s]	Height [mAU]	Area %
1	36.759	BB	1.2950	2.71875e4	298.38062	99.9399
2	58.161	BV	0.1900	16.34378	1.08334	0.0601

Totals : 2.72038e4 299.46395

Signal 2: DAD1 B, Sig=230,4 Ref=360,100

Peak #	RetTime [min]	Type	Width [min]	Area [mAU*s]	Height [mAU]	Area %
1	36.757	BB	1.3260	1.31306e4	145.89760	100.0000

Totals : 1.31306e4 145.89760

Signal 3: DAD1 C, Sig=254,4 Ref=360,100

Peak #	RetTime [min]	Type	Width [min]	Area [mAU*s]	Height [mAU]	Area %
1	36.758	BB	1.3487	3.04494e4	338.09262	100.0000

Totals : 3.04494e4 338.09262

Signal 4: DAD1 D, Sig=280,4 Ref=360,100

Signal 5: DAD1 E, Sig=310,4 Ref=360,100

===

*** End of Report ***

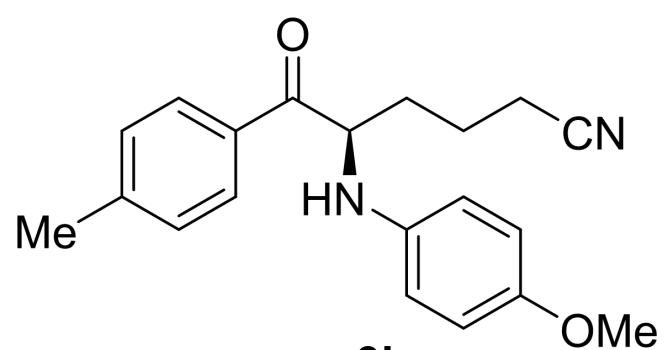

3l

Data File C:\CHEM32\1\DATA\QDY 2021-01-15 10-00-40\LZY-05-129-RAC-IC03.D
Sample Name:

===

Acq. Operator : Seq. Line : 2
Acq. Instrument : Instrument 1 Location : Vial 83
Injection Date : 1/15/2021 10:13:34 AM Inj : 1
Inj Volume : 5.000 µl
Different Inj Volume from Sequence ! Actual Inj Volume : 3.000 µl
Acq. Method : C:\CHEM32\1\DATA\QDY 2021-01-15 10-00-40\IC-03-40.M
Last changed : 1/15/2021 10:12:42 AM
(modified after loading)
Analysis Method : C:\CHEM32\1\METHODS\OD-20-10.M
Last changed : 3/12/2021 4:20:20 PM
(modified after loading)
Additional Info : Peak(s) manually integrated

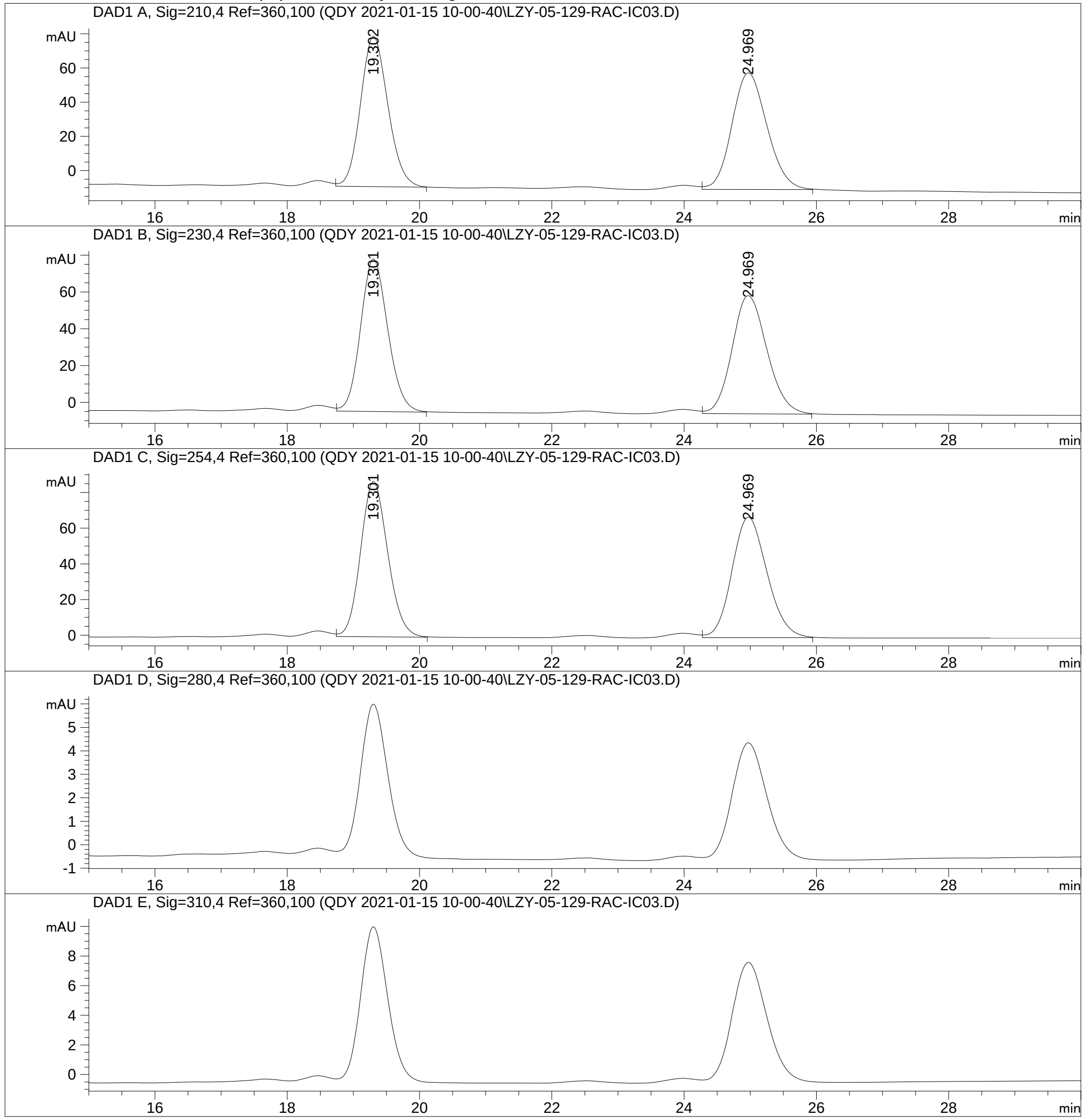

Data File C:\CHEM32\1\DATA\QDY 2021-01-15 10-00-40\LZY-05-129-RAC-IC03.D
Sample Name:

===

Area Percent Report

===

Sorted By : Signal
Multiplier : 1.0000
Dilution : 1.0000
Use Multiplier & Dilution Factor with ISTDs

Signal 1: DAD1 A, Sig=210,4 Ref=360,100

Peak #	RetTime [min]	Type	Width [min]	Area [mAU*s]	Height [mAU]	Area %
1	19.302	VB	0.4461	2532.49097	87.90887	50.1844
2	24.969	VB	0.5764	2513.87573	68.14234	49.8156

Totals : 5046.36670 156.05122

Signal 2: DAD1 B, Sig=230,4 Ref=360,100

Peak #	RetTime [min]	Type	Width [min]	Area [mAU*s]	Height [mAU]	Area %
1	19.301	VB	0.4436	2369.48804	82.85910	50.1162
2	24.969	VB	0.5687	2358.49927	64.18577	49.8838

Totals : 4727.98730 147.04487

Signal 3: DAD1 C, Sig=254,4 Ref=360,100

Peak #	RetTime [min]	Type	Width [min]	Area [mAU*s]	Height [mAU]	Area %
1	19.301	VB	0.4457	2488.93359	87.02237	50.0184
2	24.969	VB	0.5737	2487.10083	67.53403	49.9816

Totals : 4976.03442 154.55640

Signal 4: DAD1 D, Sig=280,4 Ref=360,100

Signal 5: DAD1 E, Sig=310,4 Ref=360,100

===

*** End of Report ***

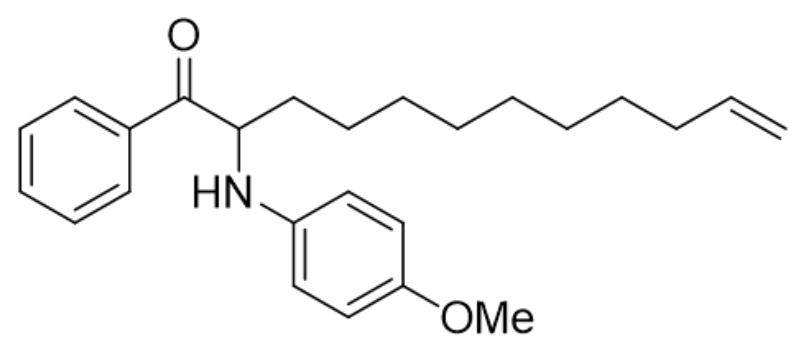

3m-Rac

```
=====================================================================
Acq. Operator   :                               Seq. Line :  12
                                                Location : Vial 81
Injection Date  : 3/12/2021 6:01:15 PM               Inj :   1
Acq. Method     : IC-03-40.M
Analysis Method : C:\CHEM32\1\METHODS\OD-20-10.M
Last changed    : 3/12/2021 5:34:21 PM
                  (modified after loading)
Additional Info : Peak(s) manually integrated
```

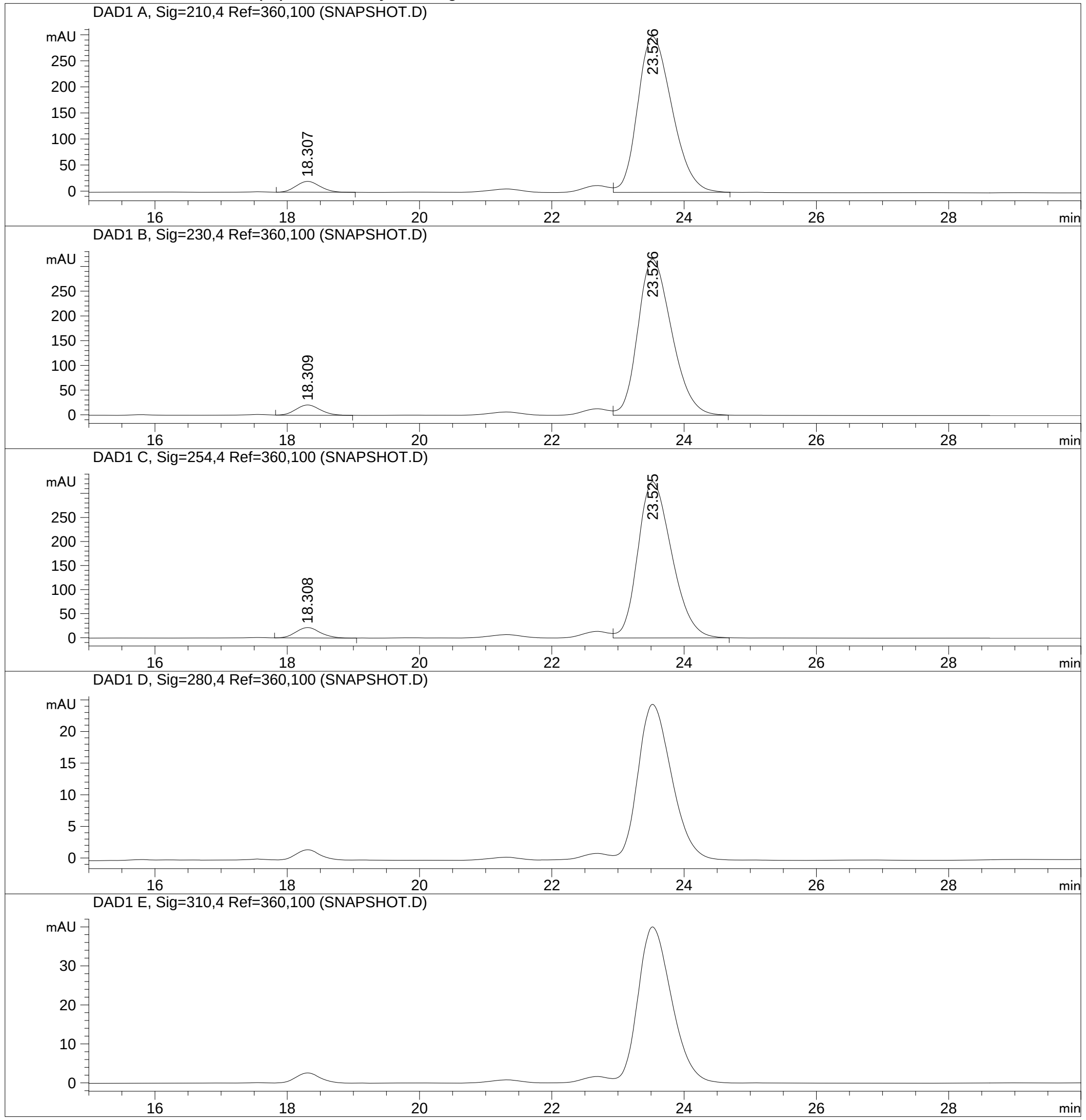


```
=====================================================================
                         Area Percent Report
=====================================================================

Sorted By             :      Signal
Multiplier            :      1.0000
Dilution              :      1.0000
Use Multiplier & Dilution Factor with ISTDs


Signal 1: DAD1 A, Sig=210,4 Ref=360,100

Peak RetTime Type  Width     Area      Height     Area
  #   [min]        [min]   [mAU*s]     [mAU]        %
----|-------|----|-------|----------|----------|--------|
   1  18.307 BB    0.3919  529.99329   20.89439   4.6819
   2  23.526 VB    0.5605 1.07901e4  299.34000  95.3181

Totals :                  1.13201e4  320.23439


Signal 2: DAD1 B, Sig=230,4 Ref=360,100

Peak RetTime Type  Width     Area      Height     Area
  #   [min]        [min]   [mAU*s]     [mAU]        %
----|-------|----|-------|----------|----------|--------|
   1  18.309 VB    0.4023  537.98956   20.90210   4.5896
   2  23.526 VB    0.5511 1.11839e4  315.84521  95.4104

Totals :                  1.17219e4  336.74732


Signal 3: DAD1 C, Sig=254,4 Ref=360,100

Peak RetTime Type  Width     Area      Height     Area
  #   [min]        [min]   [mAU*s]     [mAU]        %
----|-------|----|-------|----------|----------|--------|
   1  18.308 BB    0.3973  551.44269   21.63947   4.5526
   2  23.525 VB    0.5531 1.15613e4  324.91861  95.4474

Totals :                  1.21127e4  346.55808


Signal 4: DAD1 D, Sig=280,4 Ref=360,100


Signal 5: DAD1 E, Sig=310,4 Ref=360,100

=====================================================================
                          *** End of Report ***
```

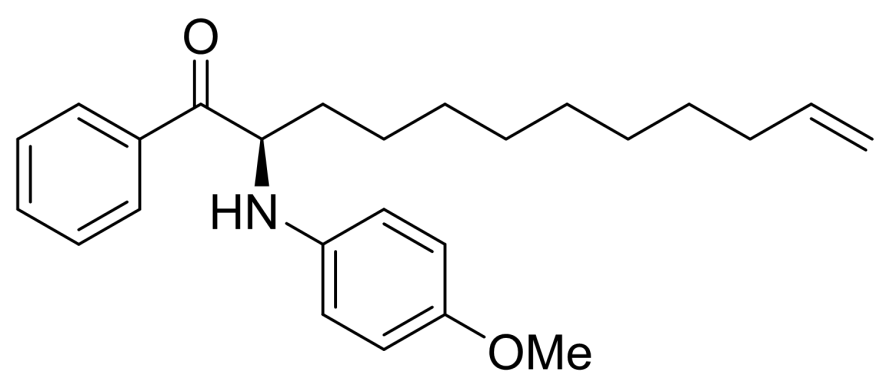

3m

Data File C:\CHEM32\1\DATA\QDY 2021-02-04 14-44-09\LZY-05-175-RAC-IC10.D
Sample Name:

===

Acq. Operator : Seq. Line : 2
Acq. Instrument : Instrument 1 Location : Vial 85
Injection Date : 2/4/2021 2:57:04 PM Inj : 1
Inj Volume : 5.000 µl
Different Inj Volume from Sequence ! Actual Inj Volume : 2.000 µl
Acq. Method : C:\CHEM32\1\DATA\QDY 2021-02-04 14-44-09\IC-10-40.M
Last changed : 2/4/2021 2:56:11 PM
(modified after loading)
Analysis Method : C:\CHEM32\1\METHODS\OD-20-10.M
Last changed : 3/12/2021 4:43:26 PM
(modified after loading)
Additional Info : Peak(s) manually integrated

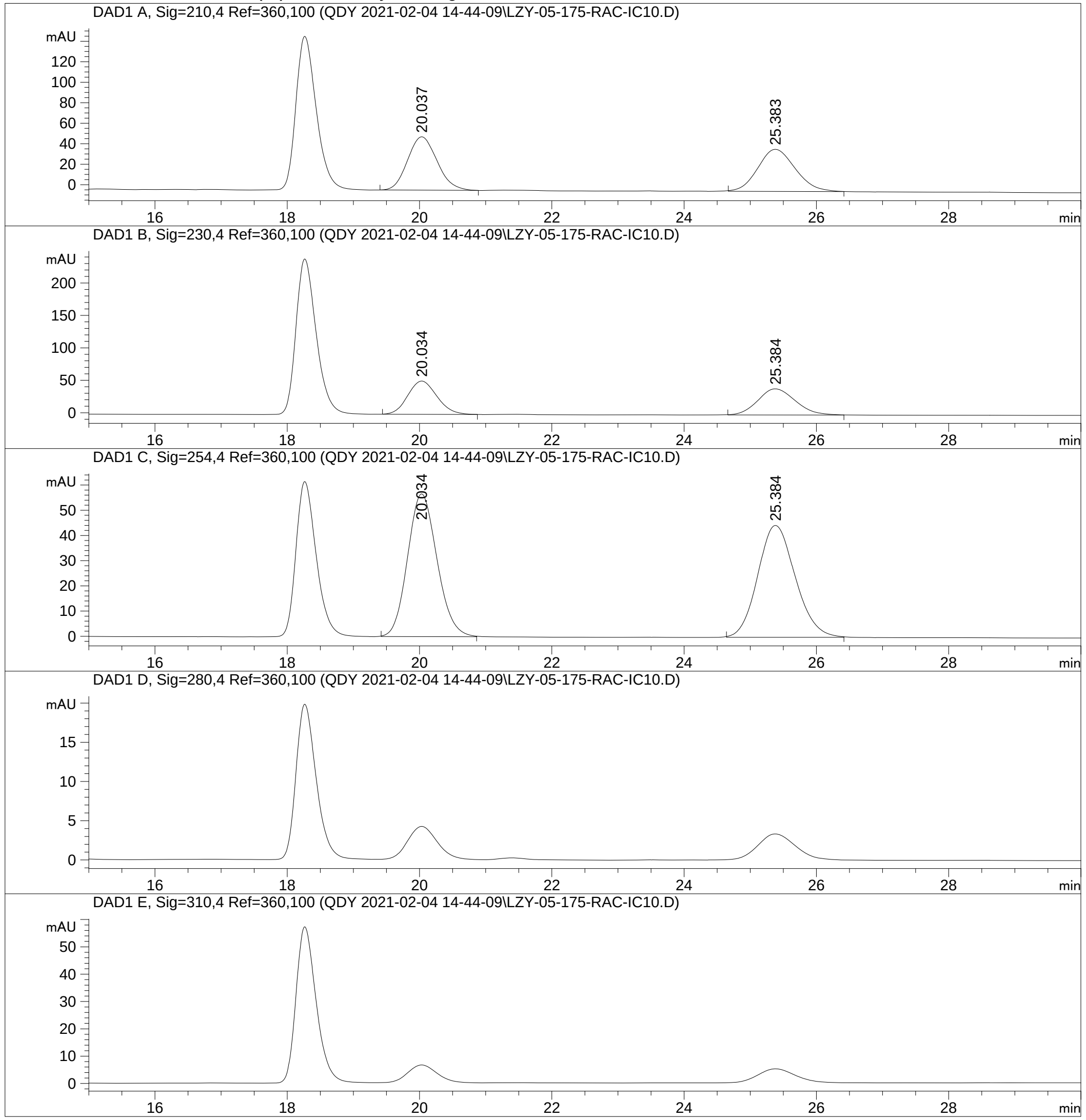

Data File C:\CHEM32\1\DATA\QDY 2021-02-04 14-44-09\LZY-05-175-RAC-IC10.D
Sample Name:

===

Area Percent Report

===

Sorted By : Signal
Multiplier : 1.0000
Dilution : 1.0000
Use Multiplier & Dilution Factor with ISTDs

Signal 1: DAD1 A, Sig=210,4 Ref=360,100

Peak #	RetTime [min]	Type	Width [min]	Area [mAU*s]	Height [mAU]	Area %
1	20.037	BB	0.4852	1612.55542	52.08304	49.9339
2	25.383	BB	0.5827	1616.82593	41.12543	50.0661

Totals : 3229.38135 93.20847

Signal 2: DAD1 B, Sig=230,4 Ref=360,100

Peak #	RetTime [min]	Type	Width [min]	Area [mAU*s]	Height [mAU]	Area %
1	20.034	BB	0.4780	1574.35706	51.29890	49.7862
2	25.384	BB	0.6045	1587.87646	40.40758	50.2138

Totals : 3162.23352 91.70648

Signal 3: DAD1 C, Sig=254,4 Ref=360,100

Peak #	RetTime [min]	Type	Width [min]	Area [mAU*s]	Height [mAU]	Area %
1	20.034	BB	0.4821	1747.21472	56.60837	49.9970
2	25.384	BB	0.6090	1747.42493	44.41662	50.0030

Totals : 3494.63965 101.02499

Signal 4: DAD1 D, Sig=280,4 Ref=360,100

Signal 5: DAD1 E, Sig=310,4 Ref=360,100

===

*** End of Report ***

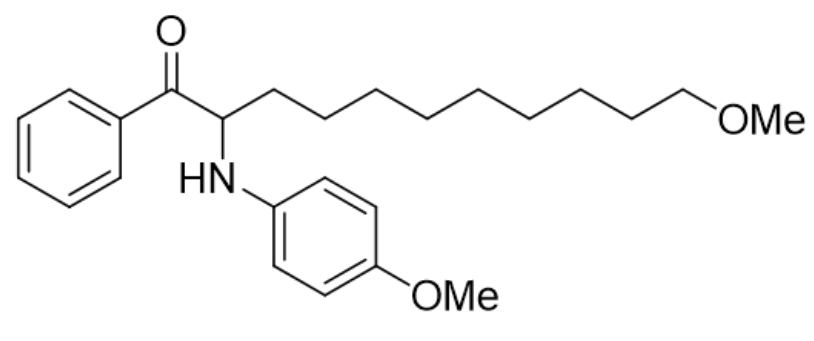

3n-Rac

Data File C:\CHEM32\1\DATA\QDY 2021-02-04 14-44-09\LZY-05-175-IC10.D
Sample Name:

===

```
Acq. Operator   :                        Seq. Line :   5
Acq. Instrument : Instrument 1            Location : Vial 86
Injection Date  : 2/4/2021 4:00:15 PM          Inj :   1
                                        Inj Volume : 5.000 µl
Different Inj Volume from Sequence !   Actual Inj Volume : 2.000 µl
Acq. Method     : C:\CHEM32\1\DATA\QDY 2021-02-04 14-44-09\IC-10-40.M
Last changed    : 11/19/2015 9:28:20 PM
Analysis Method : C:\CHEM32\1\METHODS\OD-20-10.M
Last changed    : 3/12/2021 5:34:21 PM
                  (modified after loading)
Additional Info : Peak(s) manually integrated
```

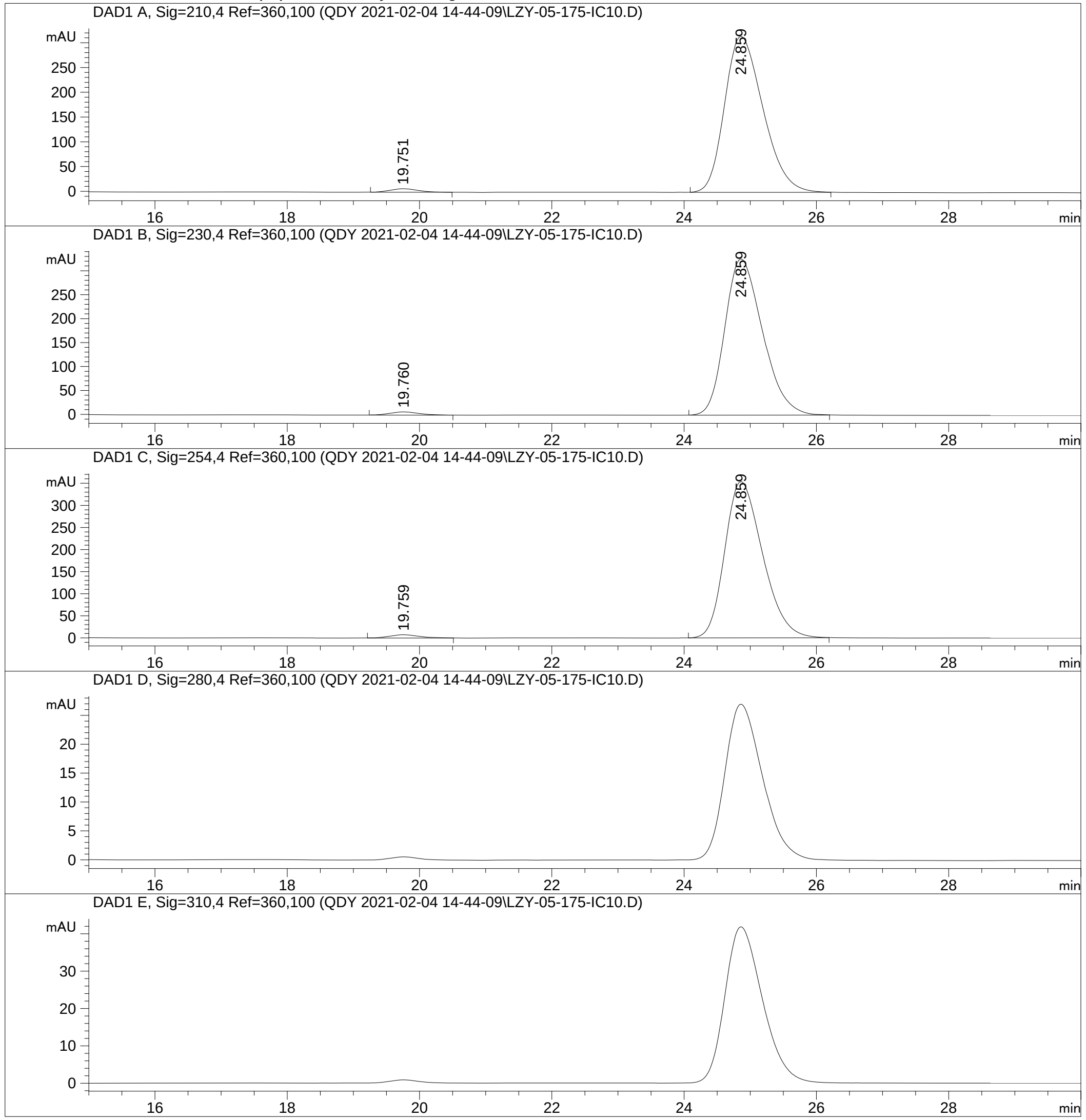

Data File C:\CHEM32\1\DATA\QDY 2021-02-04 14-44-09\LZY-05-175-IC10.D
Sample Name:

===

Area Percent Report

===

```
Sorted By             :      Signal
Multiplier            :      1.0000
Dilution              :      1.0000
Use Multiplier & Dilution Factor with ISTDs
```

Signal 1: DAD1 A, Sig=210,4 Ref=360,100

Peak #	RetTime [min]	Type	Width [min]	Area [mAU*s]	Height [mAU]	Area %
1	19.751	BB	0.4378	206.50334	7.00719	1.6000
2	24.859	BB	0.6264	1.27000e4	314.98502	98.4000

Totals : 1.29065e4 321.99220

Signal 2: DAD1 B, Sig=230,4 Ref=360,100

Peak #	RetTime [min]	Type	Width [min]	Area [mAU*s]	Height [mAU]	Area %
1	19.760	BB	0.4546	199.58362	6.64067	1.5226
2	24.859	BB	0.6137	1.29081e4	326.20282	98.4774

Totals : 1.31077e4 332.84349

Signal 3: DAD1 C, Sig=254,4 Ref=360,100

Peak #	RetTime [min]	Type	Width [min]	Area [mAU*s]	Height [mAU]	Area %
1	19.759	BB	0.4602	219.30855	7.30515	1.5363
2	24.859	BB	0.6156	1.40556e4	353.72433	98.4637

Totals : 1.42749e4 361.02949

Signal 4: DAD1 D, Sig=280,4 Ref=360,100

Signal 5: DAD1 E, Sig=310,4 Ref=360,100

===

*** End of Report ***

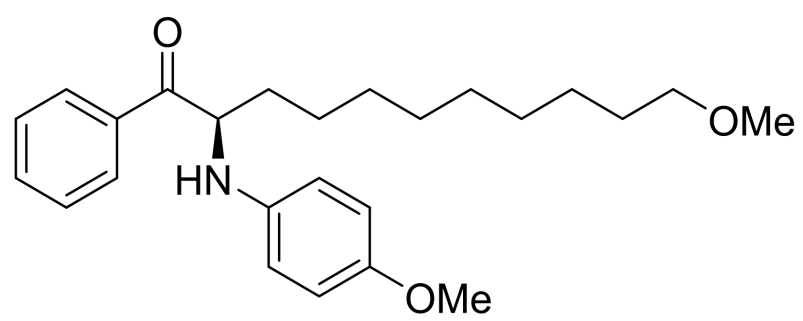

3n

Data File C:\CHEM32\1\DATA\QDY 2021-01-20 10-14-10\LZY-05-139-RAC-OD07.D
Sample Name:

===

Acq. Operator   :   Seq. Line :   8
Acq. Instrument : Instrument 1   Location : Vial 91
Injection Date : 1/20/2021 12:23:34 PM   Inj :   1
                                        Inj Volume : 5.000 µl
Different Inj Volume from Sequence !   Actual Inj Volume : 4.000 µl
Acq. Method   : C:\CHEM32\1\DATA\QDY 2021-01-20 10-14-10\OD-07-60.M
Last changed   : 1/9/2019 4:37:12 PM
Analysis Method : C:\CHEM32\1\METHODS\OD-20-10.M
Last changed   : 3/12/2021 4:25:36 PM
                  (modified after loading)
Additional Info : Peak(s) manually integrated

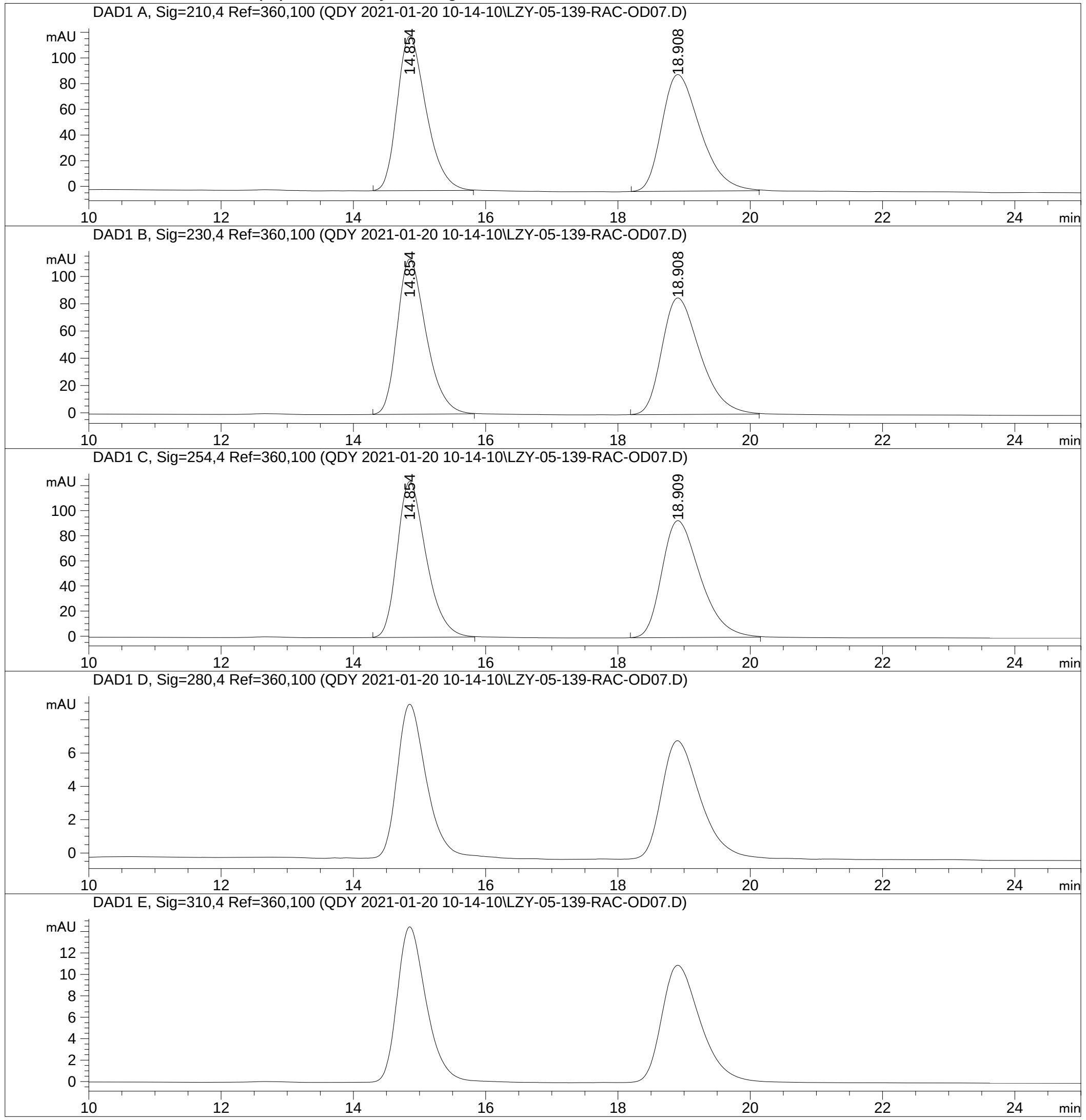

Data File C:\CHEM32\1\DATA\QDY 2021-01-20 10-14-10\LZY-05-139-RAC-OD07.D
Sample Name:

===

Area Percent Report

===

Sorted By   :   Signal
Multiplier   :   1.0000
Dilution   :   1.0000
Use Multiplier & Dilution Factor with ISTDs

Signal 1: DAD1 A, Sig=210,4 Ref=360,100

Peak #	RetTime [min]	Type	Width [min]	Area [mAU*s]	Height [mAU]	Area %
1	14.854	BB	0.4671	3656.54419	120.13018	49.8367
2	18.908	BB	0.6208	3680.50903	90.82384	50.1633

Totals :   7337.05322 210.95402

Signal 2: DAD1 B, Sig=230,4 Ref=360,100

Peak #	RetTime [min]	Type	Width [min]	Area [mAU*s]	Height [mAU]	Area %
1	14.854	BB	0.4633	3451.15942	113.96552	50.0305
2	18.908	BB	0.6182	3446.95728	85.52669	49.9695

Totals :   6898.11670 199.49220

Signal 3: DAD1 C, Sig=254,4 Ref=360,100

Peak #	RetTime [min]	Type	Width [min]	Area [mAU*s]	Height [mAU]	Area %
1	14.854	BB	0.4640	3765.15381	124.08714	50.0318
2	18.909	BB	0.6191	3760.36597	93.12852	49.9682

Totals :   7525.51978 217.21566

Signal 4: DAD1 D, Sig=280,4 Ref=360,100

Signal 5: DAD1 E, Sig=310,4 Ref=360,100

===

*** End of Report ***

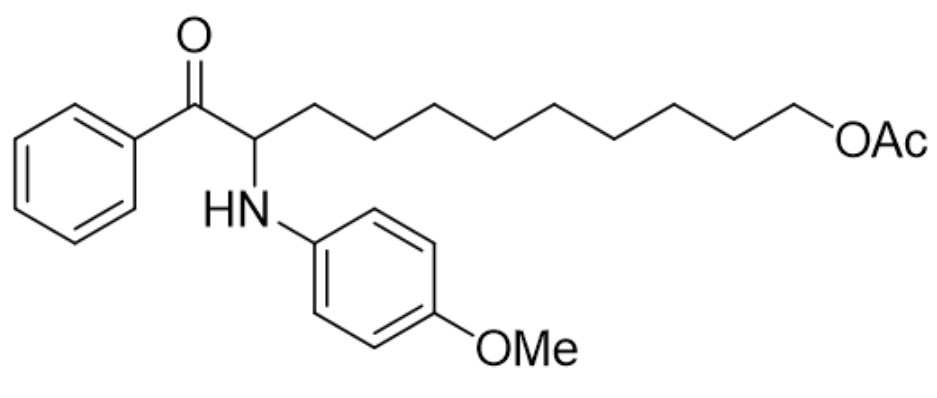

3o-Rac

Data File C:\CHEM32\1\DATA\QDY 2021-01-24 19-32-15\LZY-05-154-B.D
Sample Name:

```
=====================================================================
Acq. Operator   :                              Seq. Line :   5
Acq. Instrument : Instrument 1                  Location : Vial 83
Injection Date  : 1/24/2021 8:28:20 PM               Inj :   1
                                              Inj Volume : 5.000 µl
Different Inj Volume from Sequence !     Actual Inj Volume : 3.000 µl
Acq. Method     : C:\CHEM32\1\DATA\QDY 2021-01-24 19-32-15\OD-07-60.M
Last changed    : 1/9/2019 4:37:12 PM
Analysis Method : C:\CHEM32\1\METHODS\OD-20-10.M
Last changed    : 3/12/2021 8:22:12 PM
                  (modified after loading)
Additional Info : Peak(s) manually integrated
```

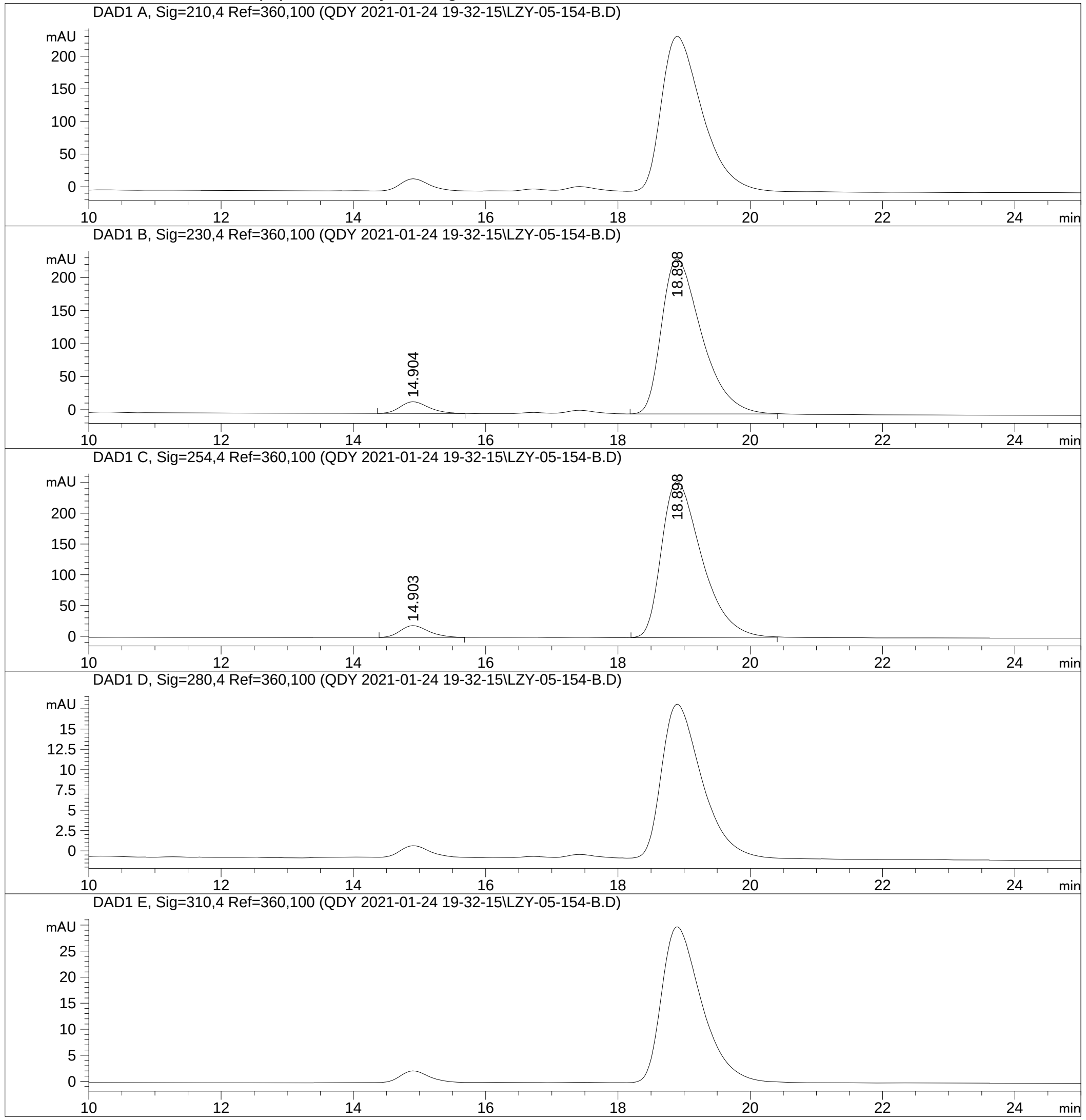

Data File C:\CHEM32\1\DATA\QDY 2021-01-24 19-32-15\LZY-05-154-B.D
Sample Name:

Area Percent Report

Sorted By : Signal
Multiplier : 1.0000
Dilution : 1.0000
Use Multiplier & Dilution Factor with ISTDs

Signal 1: DAD1 A, Sig=210,4 Ref=360,100

Signal 2: DAD1 B, Sig=230,4 Ref=360,100

Peak #	RetTime [min]	Type	Width [min]	Area [mAU*s]	Height [mAU]	Area %
1	14.904	BB	0.4595	529.07953	17.66152	5.0924
2	18.898	BB	0.6426	9860.41992	234.47581	94.9076

Totals : 1.03895e4 252.13733

Signal 3: DAD1 C, Sig=254,4 Ref=360,100

Peak #	RetTime [min]	Type	Width [min]	Area [mAU*s]	Height [mAU]	Area %
1	14.903	BB	0.4586	575.13324	19.24746	5.1013
2	18.898	BB	0.6444	1.06991e4	253.49432	94.8987

Totals : 1.12742e4 272.74178

Signal 4: DAD1 D, Sig=280,4 Ref=360,100

Peak #	RetTime [min]	Type	Width [min]	Area [mAU*s]	Height [mAU]	Area %
1	3.234	BB	0.1918	21.04720	1.63870	63.2295
2	4.351	BB	0.0804	12.23979	2.28894	36.7705

Totals : 33.28700 3.92764

Signal 5: DAD1 E, Sig=310,4 Ref=360,100

*** End of Report ***

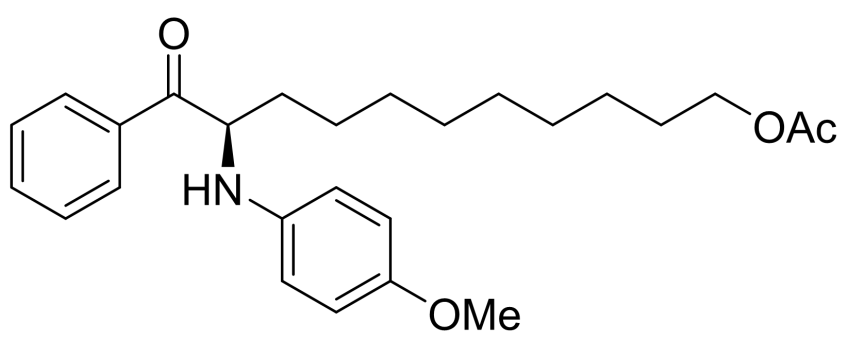

3o

Data File C:\CHEM32\1\DATA\QDY 2021-01-29 09-23-56\LZY-05-156-RAC-AD30-ALL.D
Sample Name:

===

Acq. Operator : Seq. Line : 2
Acq. Instrument : Instrument 1 Location : Vial 83
Injection Date : 1/29/2021 9:36:58 AM Inj : 1
Inj Volume : 5.000 µl
Acq. Method : C:\CHEM32\1\DATA\QDY 2021-01-29 09-23-56\AD-30-60.M
Last changed : 5/7/2016 9:30:19 AM
Analysis Method : C:\CHEM32\1\METHODS\OD-20-10.M
Last changed : 3/12/2021 4:31:32 PM
(modified after loading)
Additional Info : Peak(s) manually integrated

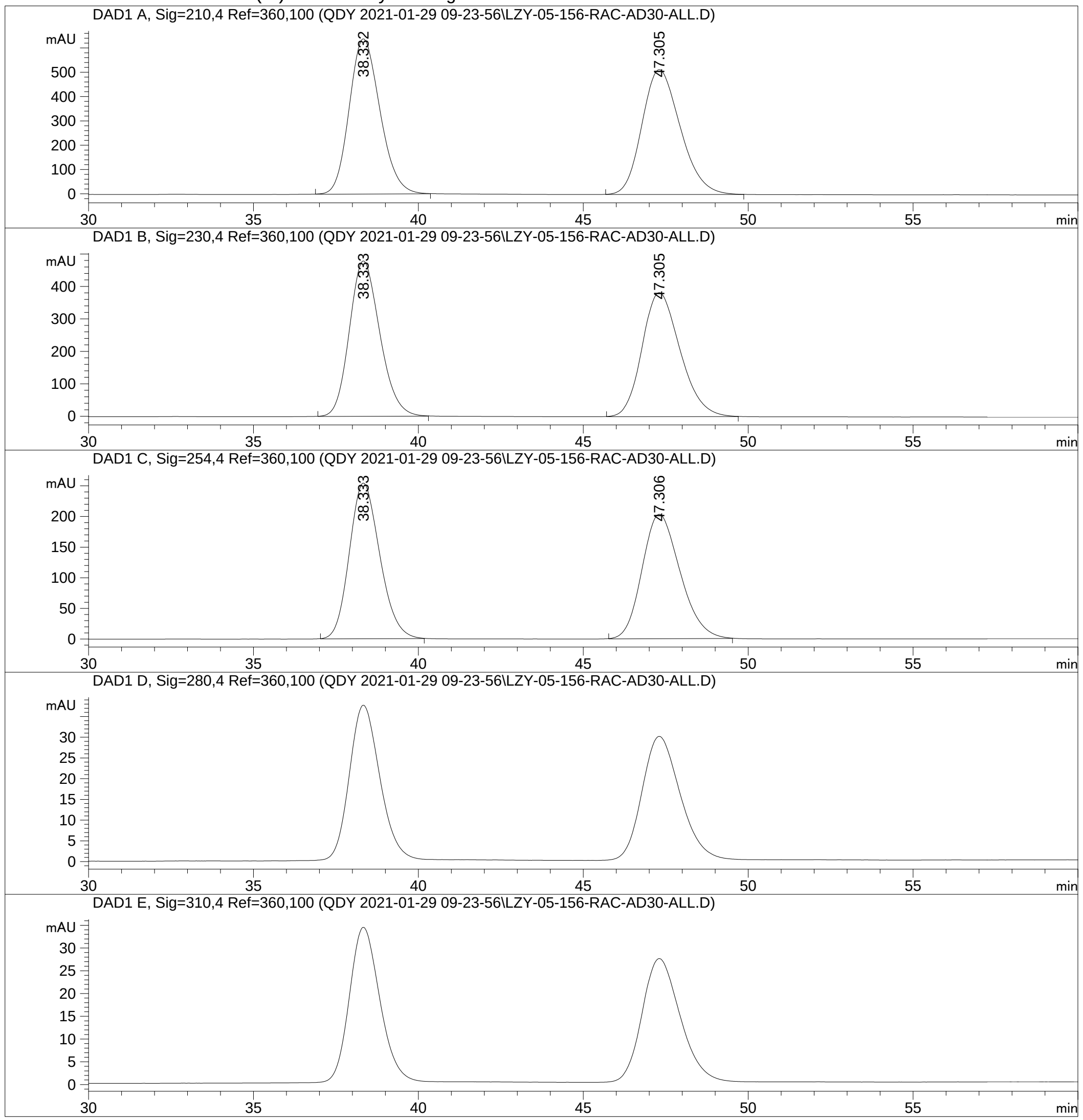

Data File C:\CHEM32\1\DATA\QDY 2021-01-29 09-23-56\LZY-05-156-RAC-AD30-ALL.D
Sample Name:

===

Area Percent Report

===

Sorted By : Signal
Multiplier : 1.0000
Dilution : 1.0000
Use Multiplier & Dilution Factor with ISTDs

Signal 1: DAD1 A, Sig=210,4 Ref=360,100

Peak #	RetTime [min]	Type	Width [min]	Area [mAU*s]	Height [mAU]	Area %
1	38.332	BB	1.0061	4.13549e4	638.09406	49.9161
2	47.305	BB	1.2572	4.14940e4	513.26154	50.0839

Totals : 8.28489e4 1151.35559

Signal 2: DAD1 B, Sig=230,4 Ref=360,100

Peak #	RetTime [min]	Type	Width [min]	Area [mAU*s]	Height [mAU]	Area %
1	38.333	BB	0.9914	3.06587e4	478.55048	50.0290
2	47.305	BB	1.2408	3.06232e4	382.16910	49.9710

Totals : 6.12819e4 860.71957

Signal 3: DAD1 C, Sig=254,4 Ref=360,100

Peak #	RetTime [min]	Type	Width [min]	Area [mAU*s]	Height [mAU]	Area %
1	38.333	BB	0.9977	1.62864e4	254.11452	50.0375
2	47.306	BB	1.2399	1.62620e4	203.14082	49.9625

Totals : 3.25483e4 457.25534

Signal 4: DAD1 D, Sig=280,4 Ref=360,100

Signal 5: DAD1 E, Sig=310,4 Ref=360,100

===

*** End of Report ***

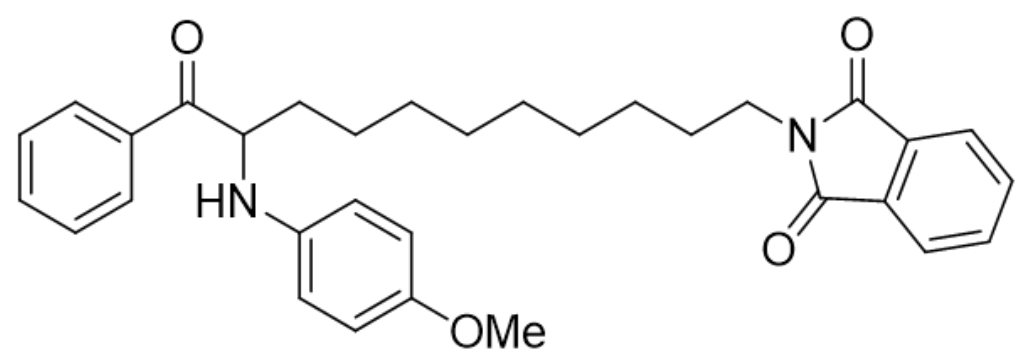

3p-Rac

Data File C:\CHEM32\1\DATA\QDY 2021-01-29 09-23-56\LZY-05-156-AD30.D
Sample Name:

===

Acq. Operator : Seq. Line : 3
Acq. Instrument : Instrument 1 Location : Vial 84
Injection Date : 1/29/2021 10:37:58 AM Inj : 1
Inj Volume : 5.000 µl
Different Inj Volume from Sequence ! Actual Inj Volume : 3.000 µl
Acq. Method : C:\CHEM32\1\DATA\QDY 2021-01-29 09-23-56\AD-30-60.M
Last changed : 5/7/2016 9:30:19 AM
Analysis Method : C:\CHEM32\1\METHODS\OD-20-10.M
Last changed : 3/12/2021 4:31:32 PM
(modified after loading)
Additional Info : Peak(s) manually integrated

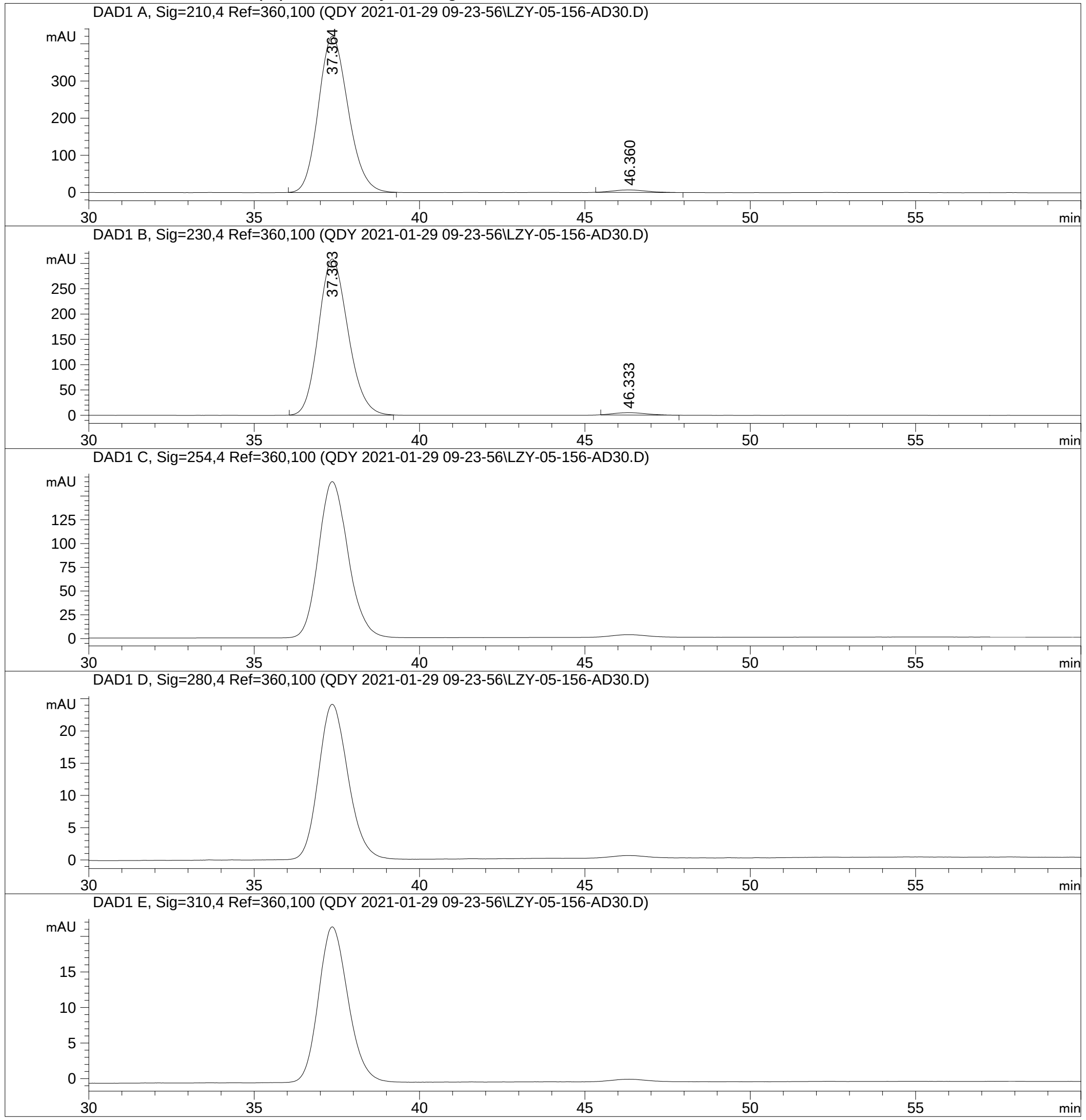

Data File C:\CHEM32\1\DATA\QDY 2021-01-29 09-23-56\LZY-05-156-AD30.D
Sample Name:

===

Area Percent Report

===

Sorted By : Signal
Multiplier : 1.0000
Dilution : 1.0000
Use Multiplier & Dilution Factor with ISTDs

Signal 1: DAD1 A, Sig=210,4 Ref=360,100

Peak #	RetTime [min]	Type	Width [min]	Area [mAU*s]	Height [mAU]	Area %
1	37.364	BB	0.9699	2.62022e4	419.97513	98.1963
2	46.360	BB	0.8449	481.29813	6.75823	1.8037

Totals : 2.66835e4 426.73335

Signal 2: DAD1 B, Sig=230,4 Ref=360,100

Peak #	RetTime [min]	Type	Width [min]	Area [mAU*s]	Height [mAU]	Area %
1	37.363	BB	0.9640	1.91543e4	308.65674	98.3656
2	46.333	BB	0.8537	318.25906	4.68985	1.6344

Totals : 1.94726e4 313.34659

Signal 3: DAD1 C, Sig=254,4 Ref=360,100

Signal 4: DAD1 D, Sig=280,4 Ref=360,100

Signal 5: DAD1 E, Sig=310,4 Ref=360,100

===

*** End of Report ***

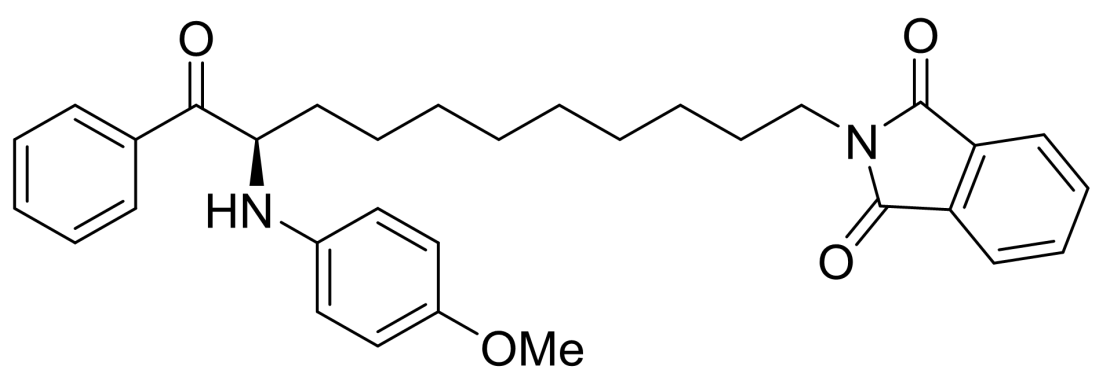

3p

Data File C:\CHEM32\1\DATA\QDY 2020-12-30 16-25-27\LZY-05-95-A-IC-15.D
Sample Name:

===

Acq. Operator   :   Seq. Line :   8
Acq. Instrument : Instrument 1   Location : Vial 83
Injection Date : 12/30/2020 6:59:40 PM   Inj :   1
                                          Inj Volume : 5.000 µl
Different Inj Volume from Sequence !   Actual Inj Volume : 4.000 µl
Acq. Method   : C:\CHEM32\1\DATA\QDY 2020-12-30 16-25-27\IC-15-40.M
Last changed   : 12/30/2020 6:58:48 PM
                  (modified after loading)
Analysis Method : C:\CHEM32\1\METHODS\OD-20-10.M
Last changed   : 3/12/2021 4:38:40 PM
                  (modified after loading)
Additional Info : Peak(s) manually integrated

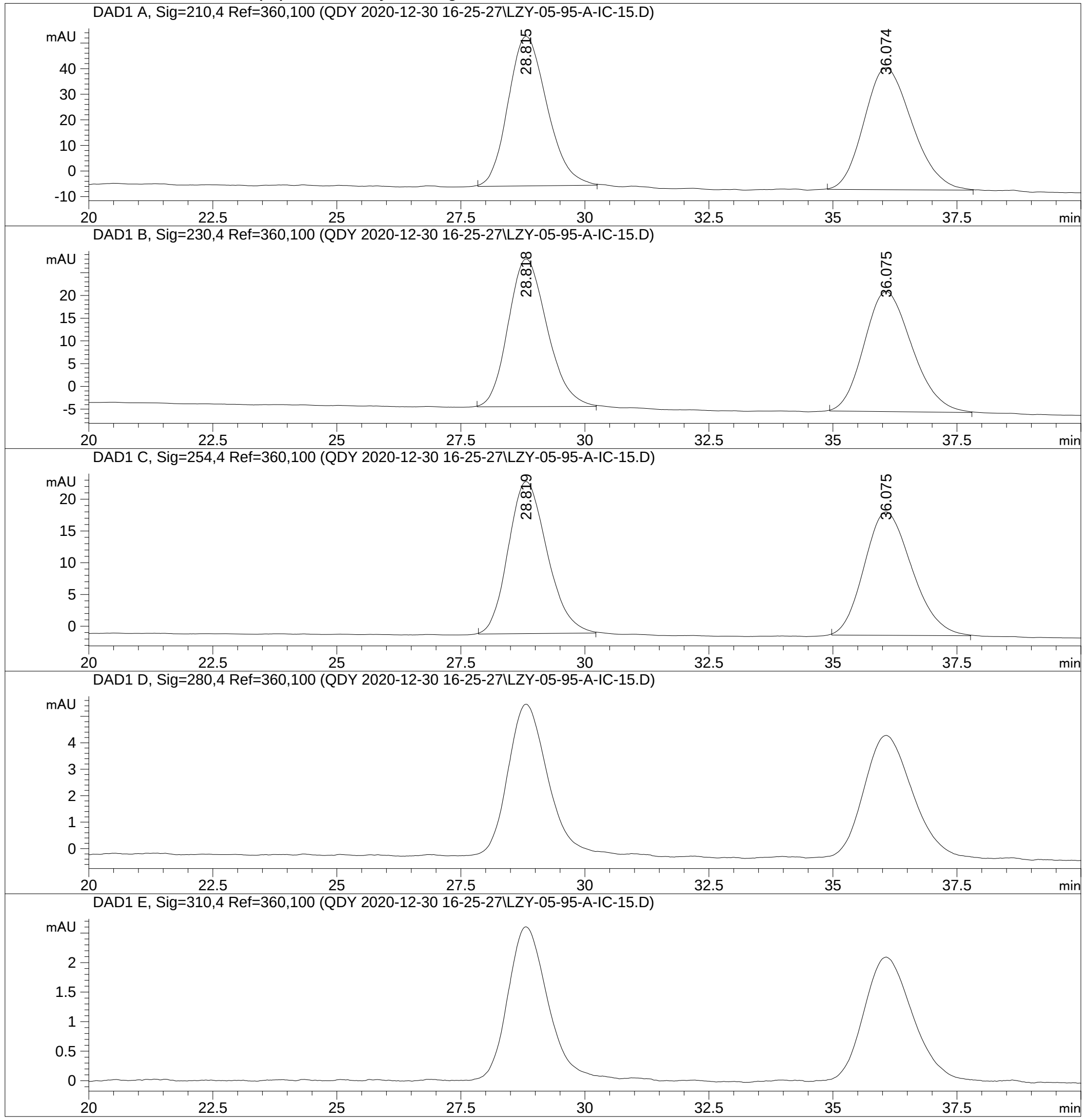

Data File C:\CHEM32\1\DATA\QDY 2020-12-30 16-25-27\LZY-05-95-A-IC-15.D
Sample Name:

===

Area Percent Report

===

Sorted By   :   Signal
Multiplier   :   1.0000
Dilution   :   1.0000
Use Multiplier & Dilution Factor with ISTDs

Signal 1: DAD1 A, Sig=210,4 Ref=360,100

Peak #	RetTime [min]	Type	Width [min]	Area [mAU*s]	Height [mAU]	Area %
1	28.815	BB	0.8180	3185.37646	58.43152	49.7890
2	36.074	BB	0.9707	3212.37720	47.63633	50.2110
Totals :				6397.75366	106.06786	

Signal 2: DAD1 B, Sig=230,4 Ref=360,100

Peak #	RetTime [min]	Type	Width [min]	Area [mAU*s]	Height [mAU]	Area %
1	28.818	BB	0.8314	1768.83325	32.47530	49.9192
2	36.075	BB	1.0134	1774.56262	26.42696	50.0808
Totals :				3543.39587	58.90226	

Signal 3: DAD1 C, Sig=254,4 Ref=360,100

Peak #	RetTime [min]	Type	Width [min]	Area [mAU*s]	Height [mAU]	Area %
1	28.819	BB	0.8371	1302.32251	23.92321	50.0654
2	36.075	BB	1.0222	1298.91809	19.42309	49.9346
Totals :				2601.24060	43.34630	

Signal 4: DAD1 D, Sig=280,4 Ref=360,100

Signal 5: DAD1 E, Sig=310,4 Ref=360,100

===

*** End of Report ***

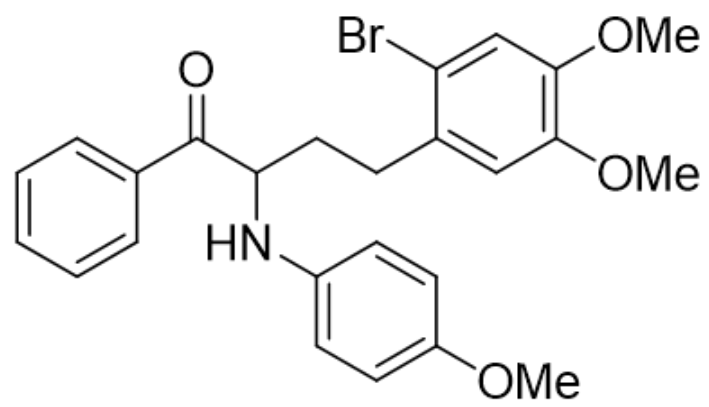

3q-Rac

Data File C:\CHEM32\1\DATA\QDY 2021-02-06 21-40-24\LZY-05-182.D
Sample Name:

===

Acq. Operator : Seq. Line : 5
Acq. Instrument : Instrument 1 Location : Vial 95
Injection Date : 2/6/2021 10:36:29 PM Inj : 1
Inj Volume : 5.000 µl
Different Inj Volume from Sequence ! Actual Inj Volume : 4.000 µl
Acq. Method : C:\CHEM32\1\DATA\QDY 2021-02-06 21-40-24\IC-15-40.M
Last changed : 2/6/2021 10:35:36 PM
(modified after loading)
Analysis Method : C:\CHEM32\1\METHODS\OD-20-10.M
Last changed : 3/12/2021 4:38:40 PM
(modified after loading)
Additional Info : Peak(s) manually integrated

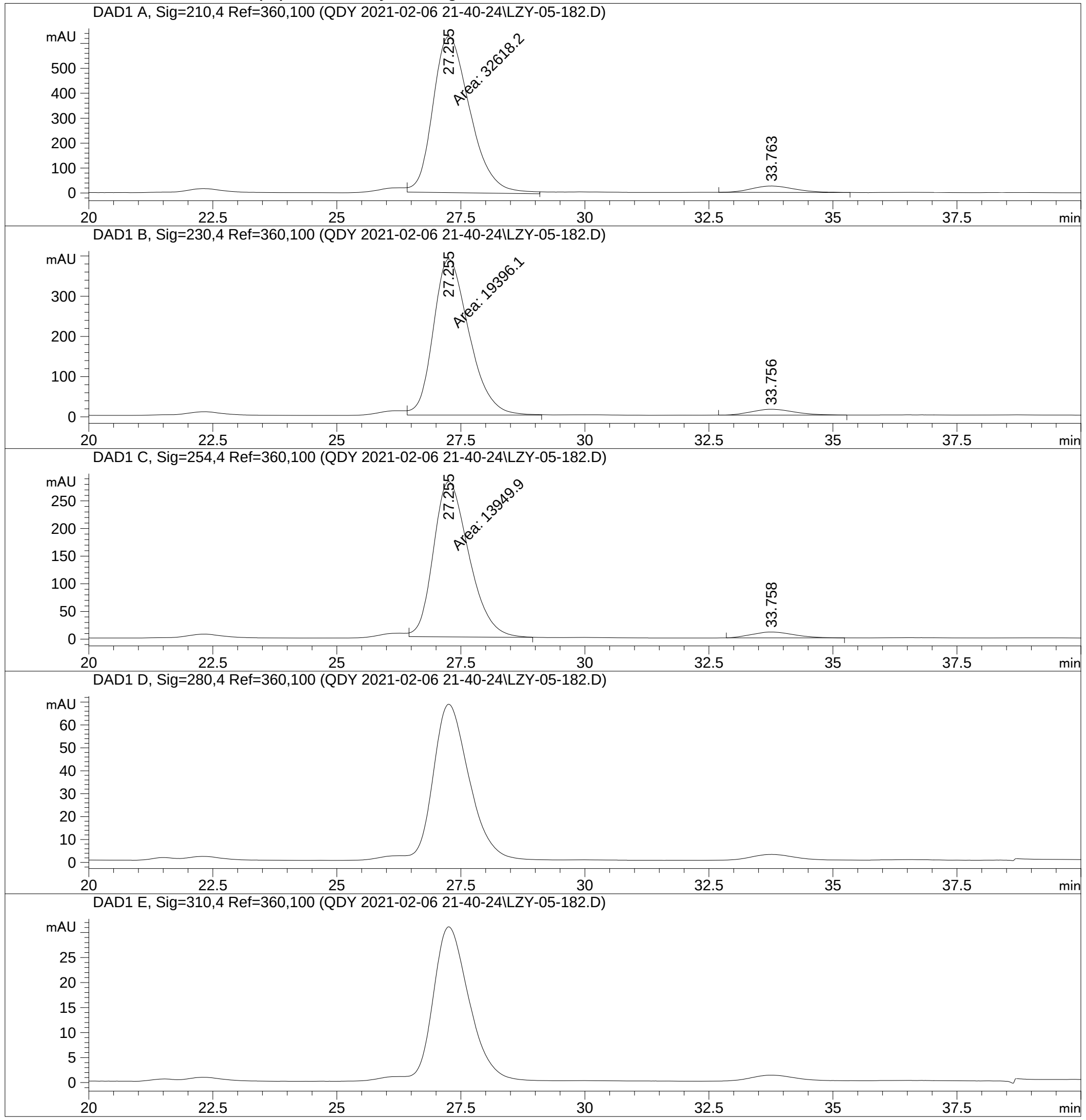

Data File C:\CHEM32\1\DATA\QDY 2021-02-06 21-40-24\LZY-05-182.D
Sample Name:

===
Area Percent Report
===

Sorted By : Signal
Multiplier : 1.0000
Dilution : 1.0000
Use Multiplier & Dilution Factor with ISTDs

Signal 1: DAD1 A, Sig=210,4 Ref=360,100

Peak #	RetTime [min]	Type	Width [min]	Area [mAU*s]	Height [mAU]	Area %
1	27.255	MM	0.8674	3.26182e4	626.73627	95.4329
2	33.763	BB	0.9118	1561.01001	25.65900	4.5671

Totals : 3.41792e4 652.39527

Signal 2: DAD1 B, Sig=230,4 Ref=360,100

Peak #	RetTime [min]	Type	Width [min]	Area [mAU*s]	Height [mAU]	Area %
1	27.255	MM	0.8330	1.93961e4	388.08975	95.6958
2	33.756	BB	0.8662	872.40448	14.57737	4.3042

Totals : 2.02685e4 402.66712

Signal 3: DAD1 C, Sig=254,4 Ref=360,100

Peak #	RetTime [min]	Type	Width [min]	Area [mAU*s]	Height [mAU]	Area %
1	27.255	MM	0.8273	1.39499e4	281.03311	95.6763
2	33.758	BB	0.9051	630.40576	10.64193	4.3237

Totals : 1.45803e4 291.67504

Signal 4: DAD1 D, Sig=280,4 Ref=360,100

Signal 5: DAD1 E, Sig=310,4 Ref=360,100

===
*** End of Report ***

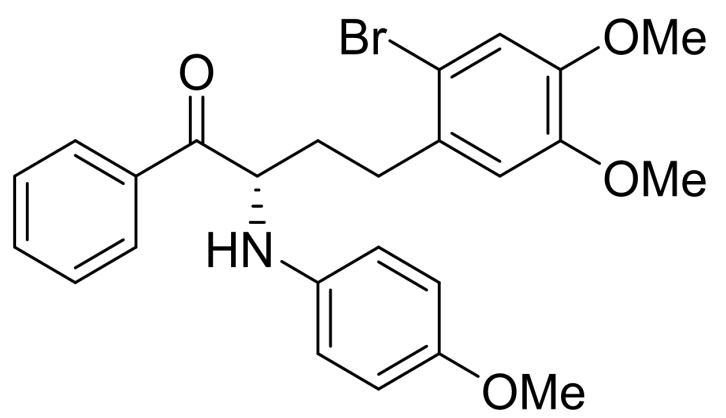

3q

===

```
Acq. Operator   :                               Seq. Line :   2
Acq. Instrument : Instrument 1                   Location : Vial 83
Injection Date  : 11/26/2020 11:06:53 AM              Inj :   1
                                               Inj Volume : 5.000 µl
Different Inj Volume from Sequence !     Actual Inj Volume : 2.000 µl
Acq. Method     : C:\CHEM32\1\DATA\QDY 2020-11-26 10-54-04\IC-03-30.M
Last changed    : 11/26/2020 11:06:01 AM
                  (modified after loading)
Analysis Method : C:\CHEM32\1\METHODS\IC-007-90.M
Last changed    : 11/26/2020 2:48:13 PM
                  (modified after loading)
Additional Info : Peak(s) manually integrated
```

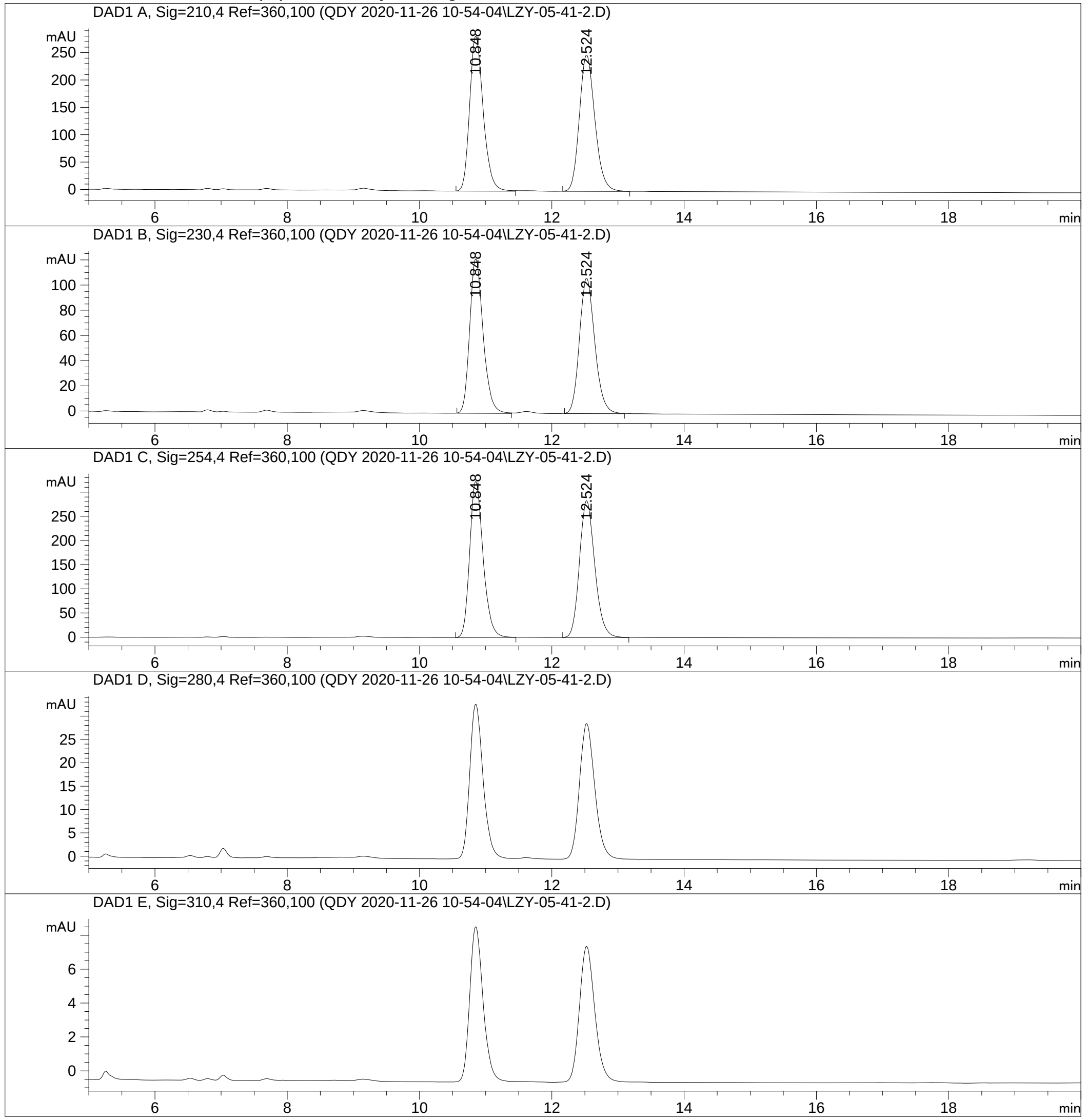

===

Area Percent Report

===

```
Sorted By             :      Signal
Multiplier            :      1.0000
Dilution              :      1.0000
Use Multiplier & Dilution Factor with ISTDs
```

Signal 1: DAD1 A, Sig=210,4 Ref=360,100

Peak #	RetTime [min]	Type	Width [min]	Area [mAU*s]	Height [mAU]	Area %
1	10.848	BB	0.2185	4037.58618	282.85693	49.8512
2	12.524	BB	0.2523	4061.69580	249.00786	50.1488

Totals : 8099.28198 531.86479

Signal 2: DAD1 B, Sig=230,4 Ref=360,100

Peak #	RetTime [min]	Type	Width [min]	Area [mAU*s]	Height [mAU]	Area %
1	10.848	BB	0.2168	1732.85193	122.67387	49.9435
2	12.524	BB	0.2484	1736.77454	107.54414	50.0565

Totals : 3469.62646 230.21801

Signal 3: DAD1 C, Sig=254,4 Ref=360,100

Peak #	RetTime [min]	Type	Width [min]	Area [mAU*s]	Height [mAU]	Area %
1	10.848	BB	0.2165	4548.43896	322.59827	49.9279
2	12.524	BB	0.2481	4561.58154	282.85327	50.0721

Totals : 9110.02051 605.45154

Signal 4: DAD1 D, Sig=280,4 Ref=360,100

Signal 5: DAD1 E, Sig=310,4 Ref=360,100

===

*** End of Report ***

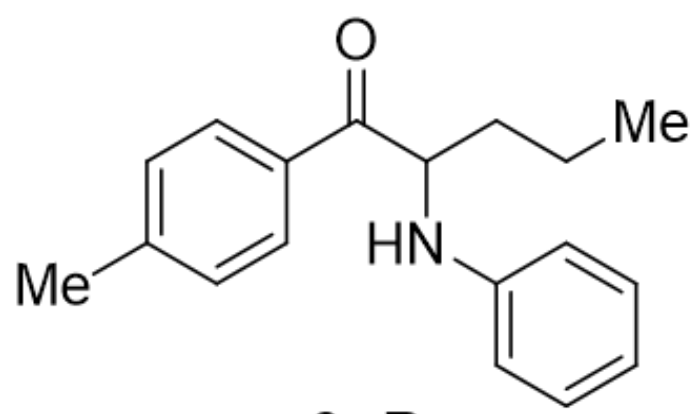

3r-Rac

===

Acq. Operator : Seq. Line : 3
Acq. Instrument : Instrument 1 Location : Vial 92
Injection Date : 9/21/2020 11:51:57 AM Inj : 1
Inj Volume : 5.000 µl
Different Inj Volume from Sequence ! Actual Inj Volume : 3.000 µl
Acq. Method : C:\CHEM32\1\DATA\QDY 2020-09-21 10-19-21\IC-03-20.M
Last changed : 9/21/2020 11:51:06 AM
(modified after loading)
Analysis Method : C:\CHEM32\1\METHODS\IC-007-90.M
Last changed : 11/25/2020 8:08:45 PM
(modified after loading)
Additional Info : Peak(s) manually integrated

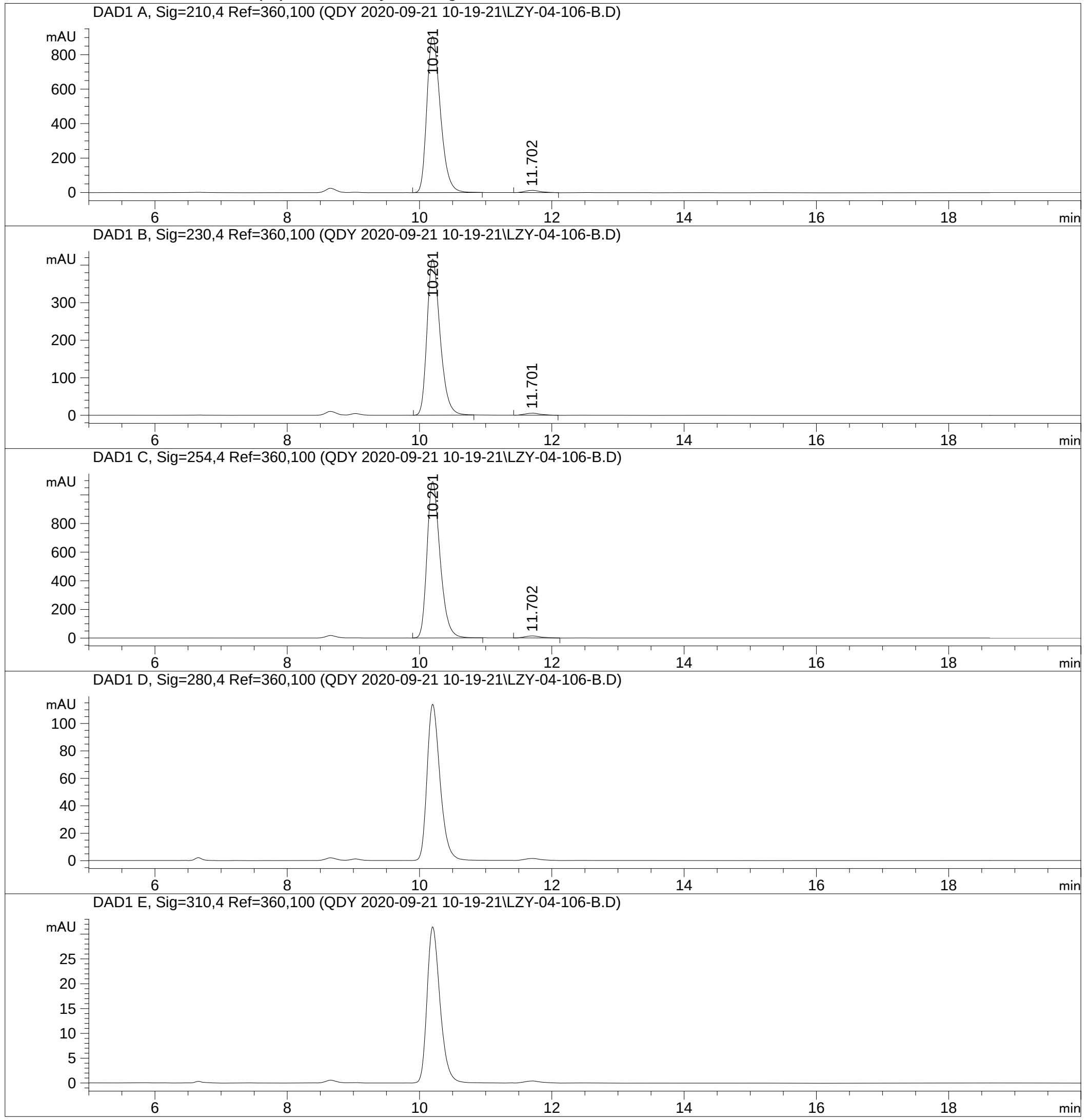

Data File C:\CHEM32\1\DATA\QDY 2020-09-21 10-19-21\LZY-04-106-B.D
Sample Name:

===

Area Percent Report

===

Sorted By : Signal
Multiplier : 1.0000
Dilution : 1.0000
Use Multiplier & Dilution Factor with ISTDs

Signal 1: DAD1 A, Sig=210,4 Ref=360,100

Peak #	RetTime [min]	Type	Width [min]	Area [mAU*s]	Height [mAU]	Area %
1	10.201	BB	0.2198	1.28897e4	907.06024	98.4501
2	11.702	BB	0.2360	202.91675	13.30284	1.5499

Totals : 1.30926e4 920.36308

Signal 2: DAD1 B, Sig=230,4 Ref=360,100

Peak #	RetTime [min]	Type	Width [min]	Area [mAU*s]	Height [mAU]	Area %
1	10.201	BB	0.2095	5692.63965	416.45081	98.5413
2	11.701	BB	0.2350	84.26539	5.49107	1.4587

Totals : 5776.90504 421.94187

Signal 3: DAD1 C, Sig=254,4 Ref=360,100

Peak #	RetTime [min]	Type	Width [min]	Area [mAU*s]	Height [mAU]	Area %
1	10.201	BB	0.2098	1.49581e4	1092.09290	98.5378
2	11.702	BB	0.2374	221.96500	14.43331	1.4622

Totals : 1.51801e4 1106.52621

Signal 4: DAD1 D, Sig=280,4 Ref=360,100

Signal 5: DAD1 E, Sig=310,4 Ref=360,100

===

*** End of Report ***

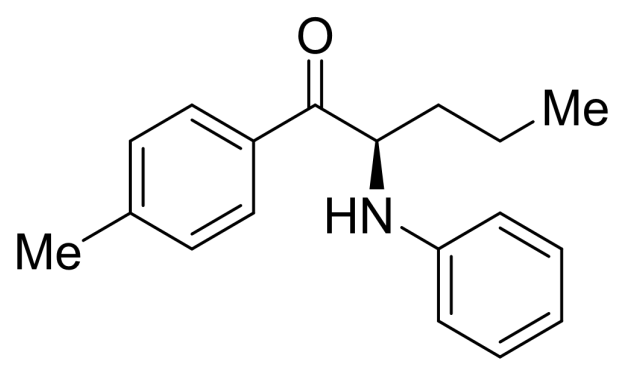

3r

Data File C:\CHEM32\1\DATA\QDY 2020-09-18 22-02-20\LZY-04-103-B-RAC.D
Sample Name:

===

```
Acq. Operator   :                              Seq. Line :   4
Acq. Instrument : Instrument 1                  Location : Vial 83
Injection Date  : 9/18/2020 11:17:24 PM              Inj :   1
                                              Inj Volume : 5.000 µl
Different Inj Volume from Sequence !     Actual Inj Volume : 2.000 µl
Acq. Method     : C:\CHEM32\1\DATA\QDY 2020-09-18 22-02-20\IC-02-30.M
Last changed    : 9/18/2020 10:14:20 PM
                  (modified after loading)
Analysis Method : C:\CHEM32\1\METHODS\IC-007-90.M
Last changed    : 11/25/2020 8:01:49 PM
                  (modified after loading)
Additional Info : Peak(s) manually integrated
```

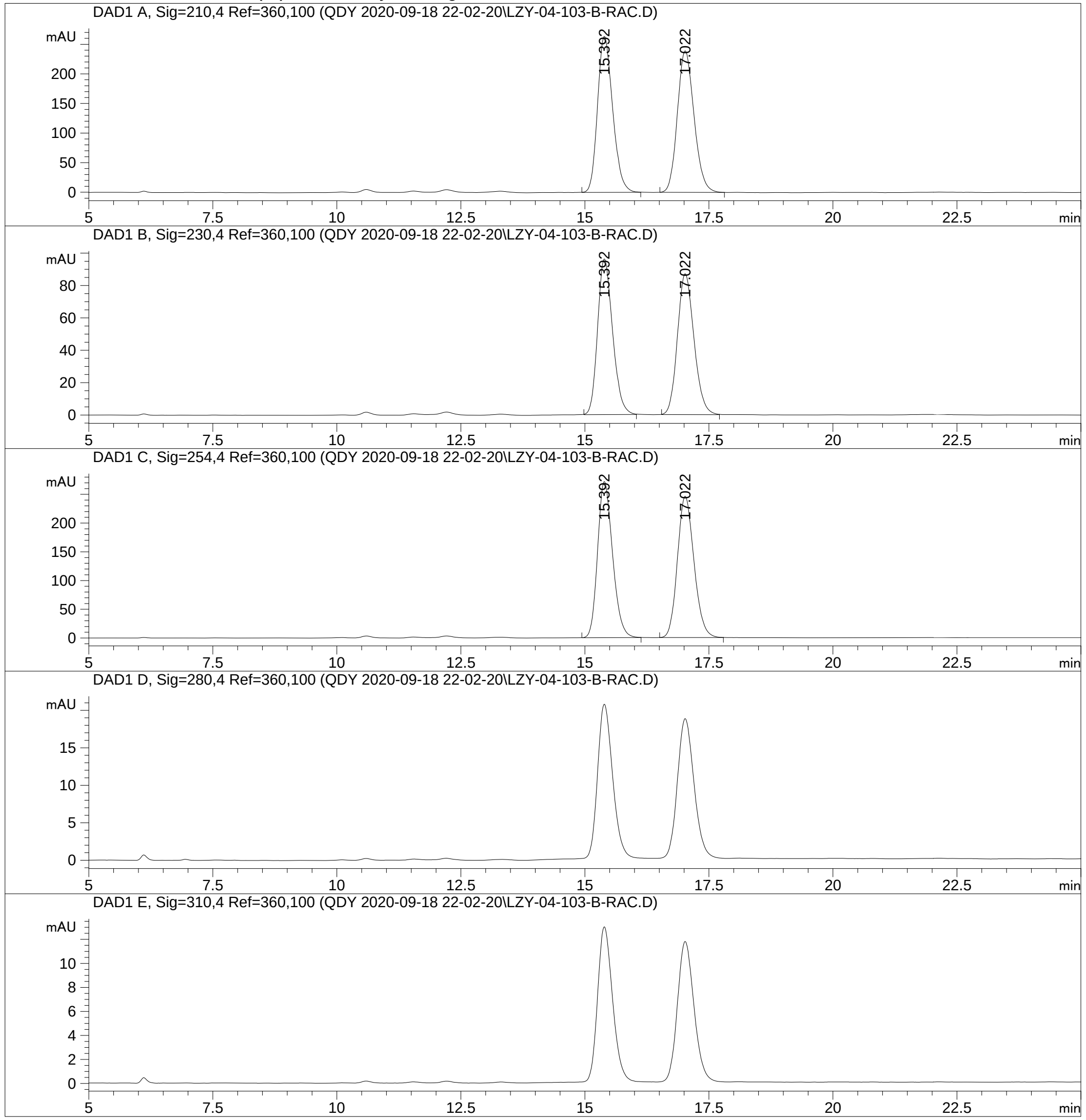

Data File C:\CHEM32\1\DATA\QDY 2020-09-18 22-02-20\LZY-04-103-B-RAC.D
Sample Name:

===

Area Percent Report

===

```
Sorted By             :      Signal
Multiplier            :      1.0000
Dilution              :      1.0000
Use Multiplier & Dilution Factor with ISTDs
```

Signal 1: DAD1 A, Sig=210,4 Ref=360,100

Peak #	RetTime [min]	Type	Width [min]	Area [mAU*s]	Height [mAU]	Area %
1	15.392	BB	0.3295	5591.97754	263.63858	50.0151
2	17.022	BB	0.3626	5588.60107	239.14082	49.9849

Totals : 1.11806e4 502.77940

Signal 2: DAD1 B, Sig=230,4 Ref=360,100

Peak #	RetTime [min]	Type	Width [min]	Area [mAU*s]	Height [mAU]	Area %
1	15.392	BB	0.3250	2018.95398	96.15652	50.0185
2	17.022	BB	0.3584	2017.45984	87.04462	49.9815

Totals : 4036.41382 183.20114

Signal 3: DAD1 C, Sig=254,4 Ref=360,100

Peak #	RetTime [min]	Type	Width [min]	Area [mAU*s]	Height [mAU]	Area %
1	15.392	BB	0.3250	5704.01074	271.66528	50.0220
2	17.022	BB	0.3584	5698.99219	245.91647	49.9780

Totals : 1.14030e4 517.58176

Signal 4: DAD1 D, Sig=280,4 Ref=360,100

Signal 5: DAD1 E, Sig=310,4 Ref=360,100

===

*** End of Report ***

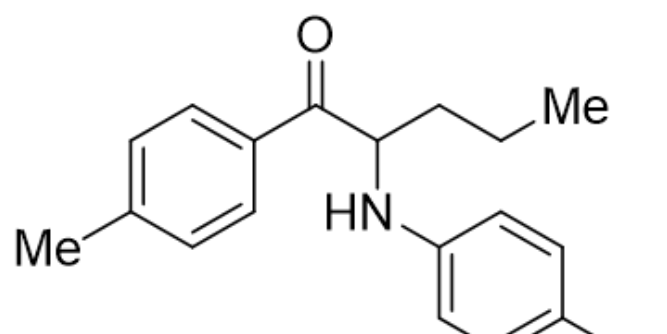

3s-Rac

Data File C:\CHEM32\1\DATA\QDY 2020-09-18 22-02-20\LZY-04-105-B.D
Sample Name:

```
=====================================================================
Acq. Operator   :                               Seq. Line :   6
Acq. Instrument : Instrument 1                   Location : Vial 85
Injection Date  : 9/19/2020 12:19:23 AM               Inj :   1
                                               Inj Volume : 5.000 µl
Different Inj Volume from Sequence !     Actual Inj Volume : 2.000 µl
Acq. Method     : C:\CHEM32\1\DATA\QDY 2020-09-18 22-02-20\IC-02-30.M
Last changed    : 9/18/2020 10:14:20 PM
                  (modified after loading)
Analysis Method : C:\CHEM32\1\METHODS\IC-007-90.M
Last changed    : 11/25/2020 8:01:49 PM
                  (modified after loading)
Additional Info : Peak(s) manually integrated
```

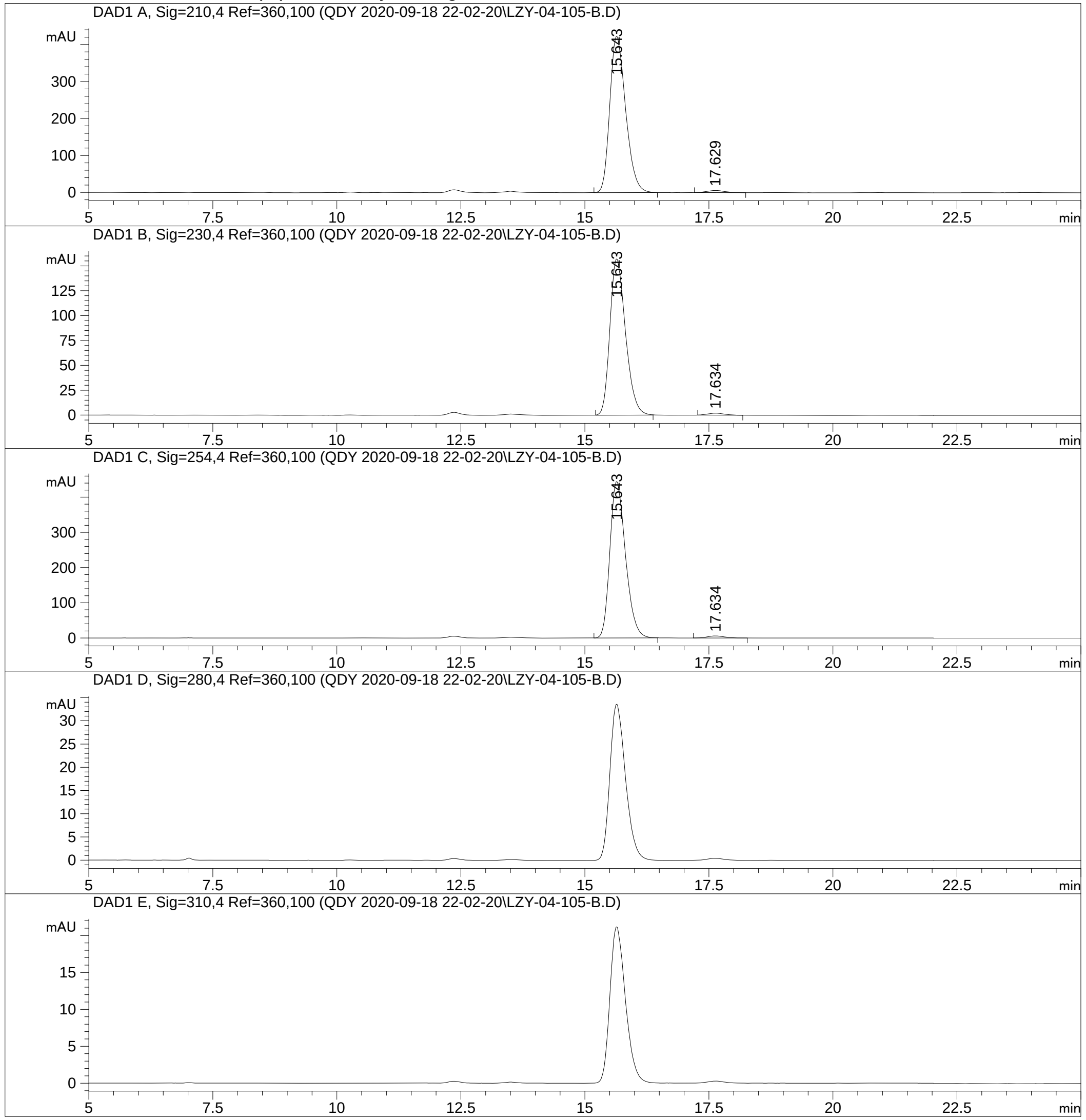

Data File C:\CHEM32\1\DATA\QDY 2020-09-18 22-02-20\LZY-04-105-B.D
Sample Name:

===

Area Percent Report

===

```
Sorted By             :      Signal
Multiplier            :      1.0000
Dilution              :      1.0000
Use Multiplier & Dilution Factor with ISTDs
```

Signal 1: DAD1 A, Sig=210,4 Ref=360,100

Peak #	RetTime [min]	Type	Width [min]	Area [mAU*s]	Height [mAU]	Area %
1	15.643	BB	0.3433	9392.12305	422.62610	98.5102
2	17.629	BB	0.3593	142.03673	5.80788	1.4898

Totals : 9534.15977 428.43398

Signal 2: DAD1 B, Sig=230,4 Ref=360,100

Peak #	RetTime [min]	Type	Width [min]	Area [mAU*s]	Height [mAU]	Area %
1	15.643	BB	0.3395	3439.79761	157.07193	98.6596
2	17.634	BB	0.3550	46.73392	1.99694	1.3404

Totals : 3486.53153 159.06887

Signal 3: DAD1 C, Sig=254,4 Ref=360,100

Peak #	RetTime [min]	Type	Width [min]	Area [mAU*s]	Height [mAU]	Area %
1	15.643	BB	0.3395	9726.89453	444.18155	98.6048
2	17.634	BB	0.3731	137.62654	5.79367	1.3952

Totals : 9864.52107 449.97522

Signal 4: DAD1 D, Sig=280,4 Ref=360,100

Signal 5: DAD1 E, Sig=310,4 Ref=360,100

===

*** End of Report ***

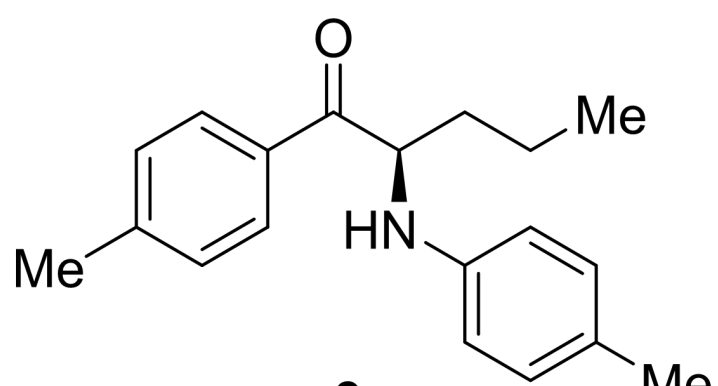

3s

Data File C:\CHEM32\1\DATA\QDY 2020-09-18 22-02-20\LZY-04-103-C-RAC.D
Sample Name:

===

Acq. Operator	:	Seq. Line :	5
Acq. Instrument	: Instrument 1	Location :	Vial 84
Injection Date	: 9/18/2020 11:48:24 PM	Inj :	1
		Inj Volume :	5.000 µl
Different Inj Volume from Sequence !		Actual Inj Volume :	2.000 µl

Acq. Method : C:\CHEM32\1\DATA\QDY 2020-09-18 22-02-20\IC-02-30.M
Last changed : 9/18/2020 10:14:20 PM
(modified after loading)
Analysis Method : C:\CHEM32\1\METHODS\IC-007-90.M
Last changed : 11/25/2020 7:56:45 PM
(modified after loading)
Additional Info : Peak(s) manually integrated

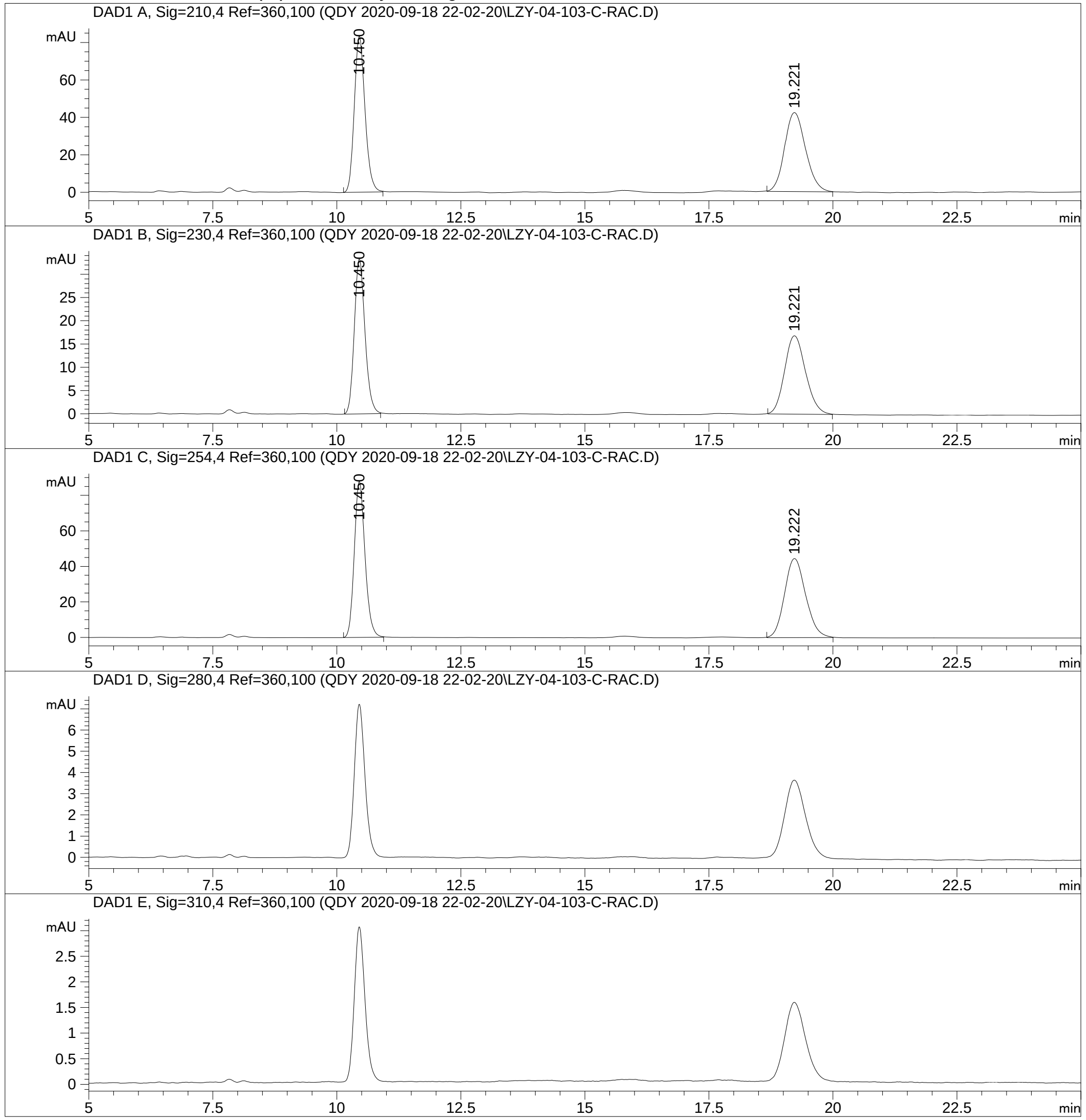

===
Area Percent Report
===

Sorted By : Signal
Multiplier : 1.0000
Dilution : 1.0000
Use Multiplier & Dilution Factor with ISTDs

Signal 1: DAD1 A, Sig=210,4 Ref=360,100

Peak #	RetTime [min]	Type	Width [min]	Area [mAU*s]	Height [mAU]	Area %
1	10.450	BB	0.2314	1250.42358	83.18699	50.1542
2	19.221	BB	0.4552	1242.73523	42.24672	49.8458

Totals : 2493.15881 125.43371

Signal 2: DAD1 B, Sig=230,4 Ref=360,100

Peak #	RetTime [min]	Type	Width [min]	Area [mAU*s]	Height [mAU]	Area %
1	10.450	BB	0.2307	499.02341	33.31635	50.1754
2	19.221	BB	0.4526	495.53482	16.87384	49.8246

Totals : 994.55823 50.19019

Signal 3: DAD1 C, Sig=254,4 Ref=360,100

Peak #	RetTime [min]	Type	Width [min]	Area [mAU*s]	Height [mAU]	Area %
1	10.450	BB	0.2312	1318.08057	87.75509	50.0593
2	19.222	BB	0.4585	1314.95801	44.53448	49.9407

Totals : 2633.03857 132.28957

Signal 4: DAD1 D, Sig=280,4 Ref=360,100

Signal 5: DAD1 E, Sig=310,4 Ref=360,100

===
*** End of Report ***

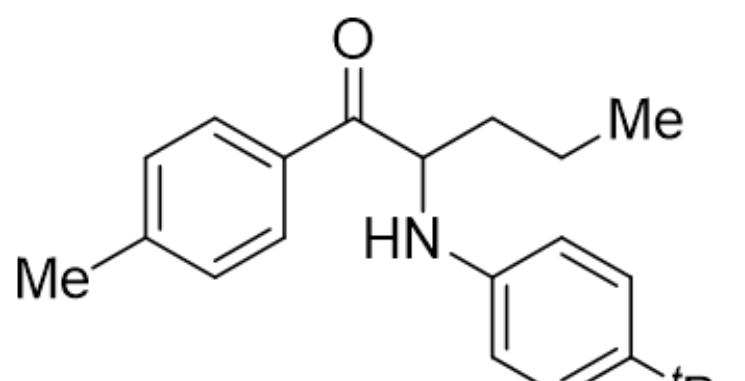

3t-Rac

Data File C:\CHEM32\1\DATA\QDY 2020-09-19 11-05-30\LZY-04-105-C.D
Sample Name:

===

Acq. Operator : Seq. Line : 3
Acq. Instrument : Instrument 1 Location : Vial 83
Injection Date : 9/19/2020 11:49:23 AM Inj : 1
Inj Volume : 5.000 µl
Different Inj Volume from Sequence ! Actual Inj Volume : 3.000 µl
Acq. Method : C:\CHEM32\1\DATA\QDY 2020-09-19 11-05-30\IC-02-30.M
Last changed : 9/19/2020 11:17:30 AM
(modified after loading)
Analysis Method : C:\CHEM32\1\METHODS\IC-007-90.M
Last changed : 11/25/2020 7:52:24 PM
(modified after loading)
Additional Info : Peak(s) manually integrated

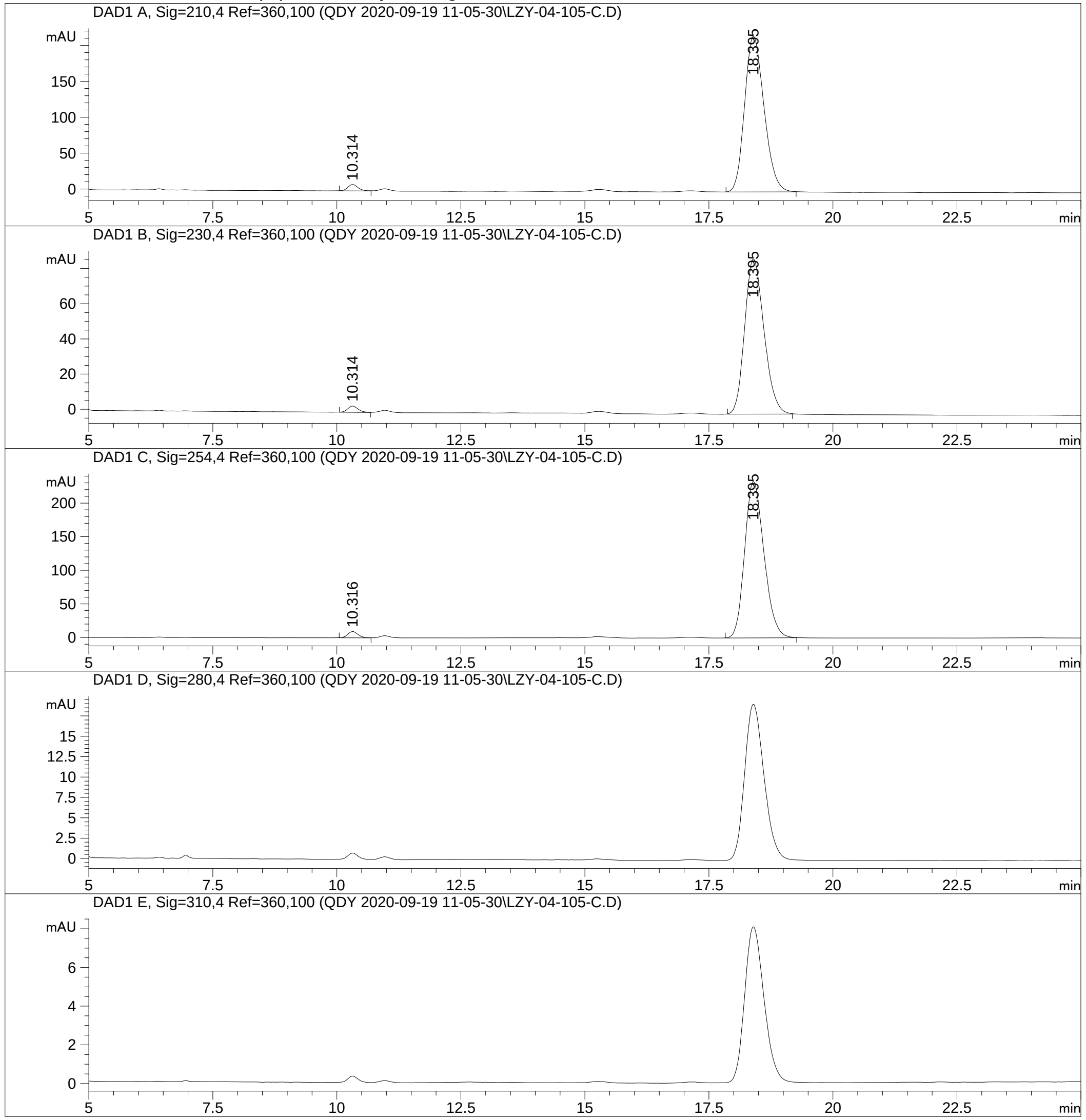

Data File C:\CHEM32\1\DATA\QDY 2020-09-19 11-05-30\LZY-04-105-C.D
Sample Name:

===

Area Percent Report

===

Sorted By : Signal
Multiplier : 1.0000
Dilution : 1.0000
Use Multiplier & Dilution Factor with ISTDs

Signal 1: DAD1 A, Sig=210,4 Ref=360,100

Peak #	RetTime [min]	Type	Width [min]	Area [mAU*s]	Height [mAU]	Area %
1	10.314	BB	0.2245	127.97258	8.86261	2.1159
2	18.395	BB	0.4267	5920.09619	216.75072	97.8841

Totals : 6048.06877 225.61333

Signal 2: DAD1 B, Sig=230,4 Ref=360,100

Peak #	RetTime [min]	Type	Width [min]	Area [mAU*s]	Height [mAU]	Area %
1	10.314	BB	0.2208	50.67800	3.54493	2.0749
2	18.395	BB	0.4223	2391.78662	88.21107	97.9251

Totals : 2442.46462 91.75600

Signal 3: DAD1 C, Sig=254,4 Ref=360,100

Peak #	RetTime [min]	Type	Width [min]	Area [mAU*s]	Height [mAU]	Area %
1	10.316	BB	0.2216	134.98700	9.39843	2.0934
2	18.395	BB	0.4227	6313.21094	232.53741	97.9066

Totals : 6448.19794 241.93584

Signal 4: DAD1 D, Sig=280,4 Ref=360,100

Signal 5: DAD1 E, Sig=310,4 Ref=360,100

===

*** End of Report ***

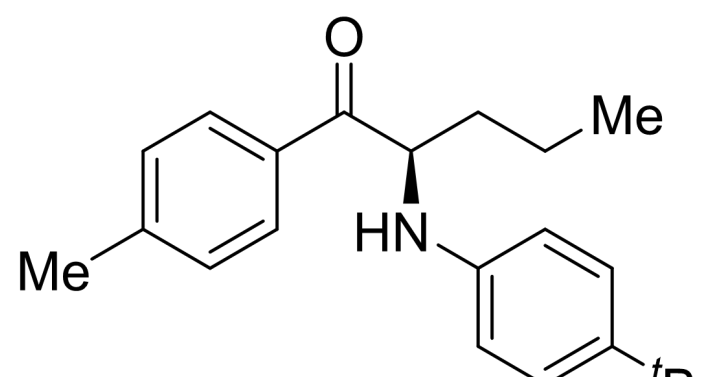

3t

Data File C:\CHEM32\1\DATA\QDY 2020-09-16 19-36-50\LZY-04-91-G-RAC-IC15.D
Sample Name:

```
=====================================================================
Acq. Operator   :                        Seq. Line :   2
Acq. Instrument : Instrument 1            Location : Vial 86
Injection Date  : 9/16/2020 7:49:45 PM         Inj :   1
                                        Inj Volume : 5.000 µl
Different Inj Volume from Sequence !   Actual Inj Volume : 2.000 µl
Acq. Method     : C:\CHEM32\1\DATA\QDY 2020-09-16 19-36-50\IC-15-60.M
Last changed    : 9/16/2020 7:48:52 PM
                  (modified after loading)
Analysis Method : C:\CHEM32\1\METHODS\IC-007-90.M
Last changed    : 11/25/2020 8:24:15 PM
                  (modified after loading)
Additional Info : Peak(s) manually integrated
```

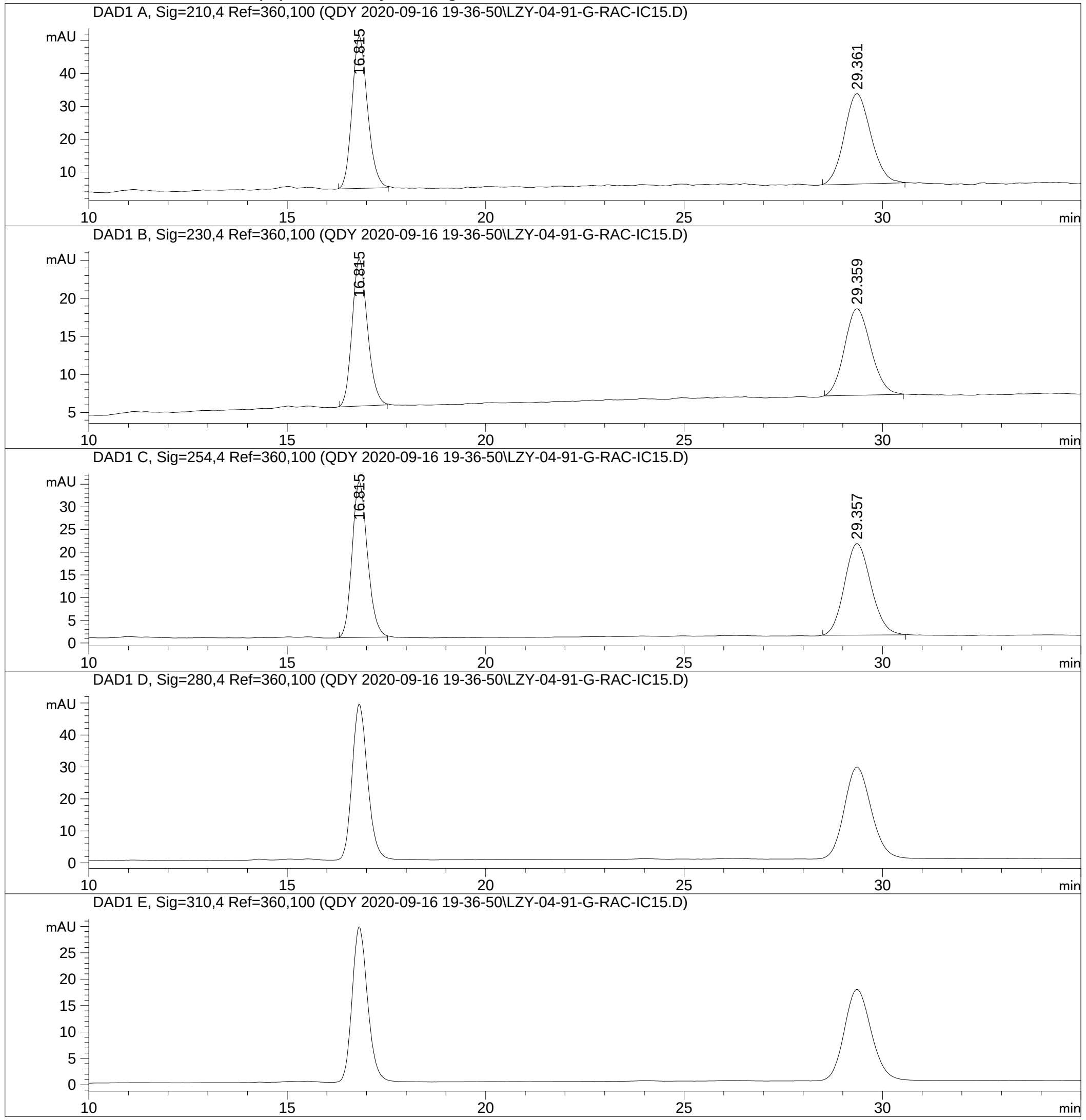

Data File C:\CHEM32\1\DATA\QDY 2020-09-16 19-36-50\LZY-04-91-G-RAC-IC15.D
Sample Name:

===

Area Percent Report

===

```
Sorted By             :      Signal
Multiplier            :      1.0000
Dilution              :      1.0000
Use Multiplier & Dilution Factor with ISTDs
```

Signal 1: DAD1 A, Sig=210,4 Ref=360,100

Peak #	RetTime [min]	Type	Width [min]	Area [mAU*s]	Height [mAU]	Area %
1	16.815	BB	0.4179	1254.22107	46.31514	49.4469
2	29.361	BB	0.6837	1282.28210	27.51607	50.5531

Totals : 2536.50317 73.83121

Signal 2: DAD1 B, Sig=230,4 Ref=360,100

Peak #	RetTime [min]	Type	Width [min]	Area [mAU*s]	Height [mAU]	Area %
1	16.815	BB	0.4163	521.22308	19.34535	49.8113
2	29.359	BB	0.7083	525.17181	11.38282	50.1887

Totals : 1046.39490 30.72817

Signal 3: DAD1 C, Sig=254,4 Ref=360,100

Peak #	RetTime [min]	Type	Width [min]	Area [mAU*s]	Height [mAU]	Area %
1	16.815	BB	0.4162	925.27979	34.36230	49.6196
2	29.357	BB	0.7266	939.46582	20.19982	50.3804

Totals : 1864.74561 54.56211

Signal 4: DAD1 D, Sig=280,4 Ref=360,100

Signal 5: DAD1 E, Sig=310,4 Ref=360,100

===

*** End of Report ***

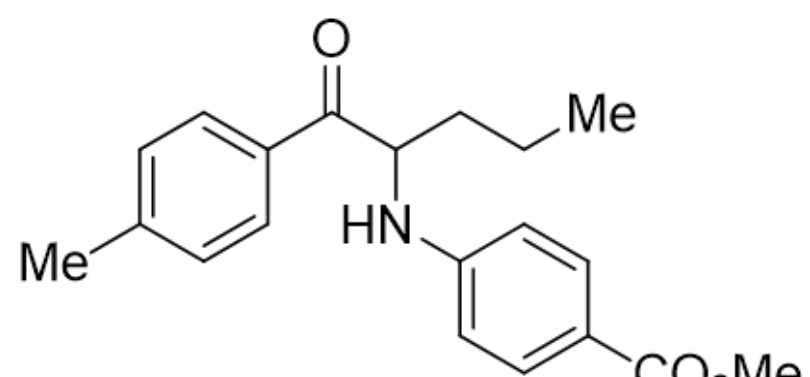

3u-Rac

Data File C:\CHEM32\1\DATA\QDY 2020-09-25 15-21-10\LZY-04-116-1.D
Sample Name:

===

Acq. Operator : Seq. Line : 3
Acq. Instrument : Instrument 1 Location : Vial 90
Injection Date : 9/25/2020 4:15:04 PM Inj : 1
Inj Volume : 5.000 µl
Different Inj Volume from Sequence ! Actual Inj Volume : 3.000 µl
Acq. Method : C:\CHEM32\1\DATA\QDY 2020-09-25 15-21-10\IC-15-40.M
Last changed : 9/25/2020 3:33:09 PM
(modified after loading)
Analysis Method : C:\CHEM32\1\METHODS\IC-007-90.M
Last changed : 11/25/2020 8:24:15 PM
(modified after loading)
Additional Info : Peak(s) manually integrated

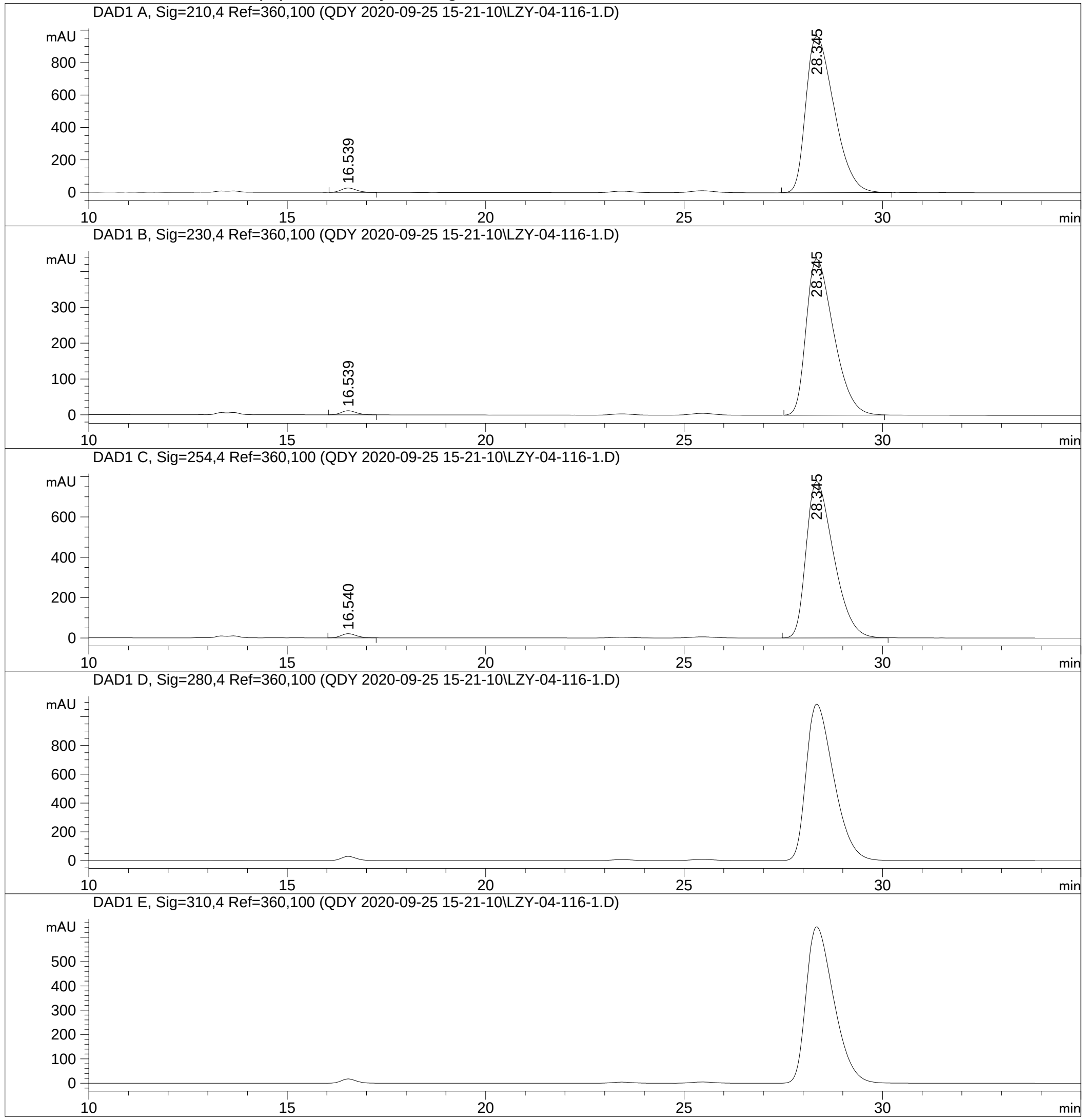

Data File C:\CHEM32\1\DATA\QDY 2020-09-25 15-21-10\LZY-04-116-1.D
Sample Name:

===

Area Percent Report

===

Sorted By : Signal
Multiplier : 1.0000
Dilution : 1.0000
Use Multiplier & Dilution Factor with ISTDs

Signal 1: DAD1 A, Sig=210,4 Ref=360,100

Peak #	RetTime [min]	Type	Width [min]	Area [mAU*s]	Height [mAU]	Area %
1	16.539	BB	0.4049	719.92401	27.18765	1.4887
2	28.345	BB	0.7665	4.76381e4	964.03302	98.5113

Totals : 4.83580e4 991.22067

Signal 2: DAD1 B, Sig=230,4 Ref=360,100

Peak #	RetTime [min]	Type	Width [min]	Area [mAU*s]	Height [mAU]	Area %
1	16.539	BB	0.4148	303.70020	11.47564	1.4290
2	28.345	BB	0.7366	2.09490e4	435.98843	98.5710

Totals : 2.12527e4 447.46407

Signal 3: DAD1 C, Sig=254,4 Ref=360,100

Peak #	RetTime [min]	Type	Width [min]	Area [mAU*s]	Height [mAU]	Area %
1	16.540	BB	0.4119	546.31757	20.56779	1.4450
2	28.345	BB	0.7368	3.72606e4	775.10583	98.5550

Totals : 3.78069e4 795.67362

Signal 4: DAD1 D, Sig=280,4 Ref=360,100

Signal 5: DAD1 E, Sig=310,4 Ref=360,100

===

*** End of Report ***

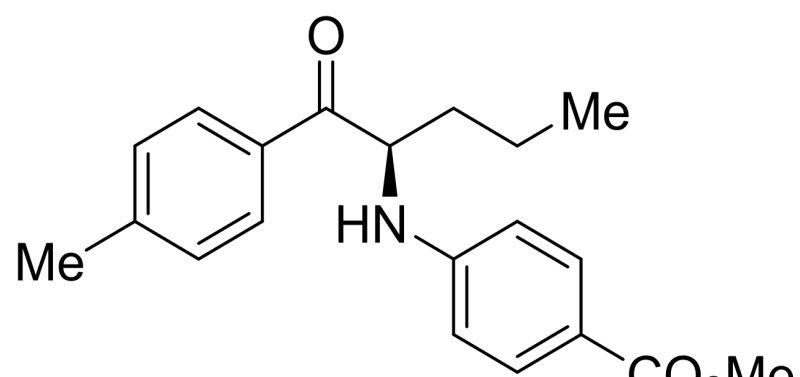

3u

Data File C:\CHEM32\1\DATA\QDY 2020-09-16 19-36-50\LZY-04-91-H-RAC-IC02.D
Sample Name:

===

```
Acq. Operator   :                              Seq. Line :   7
Acq. Instrument : Instrument 1                  Location : Vial 85
Injection Date  : 9/16/2020 10:44:59 PM              Inj :   1
                                              Inj Volume : 5.000 µl
Different Inj Volume from Sequence !     Actual Inj Volume : 2.000 µl
Acq. Method     : C:\CHEM32\1\DATA\QDY 2020-09-16 19-36-50\IC-02-30.M
Last changed    : 9/16/2020 10:13:07 PM
                  (modified after loading)
Analysis Method : C:\CHEM32\1\METHODS\IC-007-90.M
Last changed    : 11/25/2020 7:54:16 PM
                  (modified after loading)
Additional Info : Peak(s) manually integrated
```

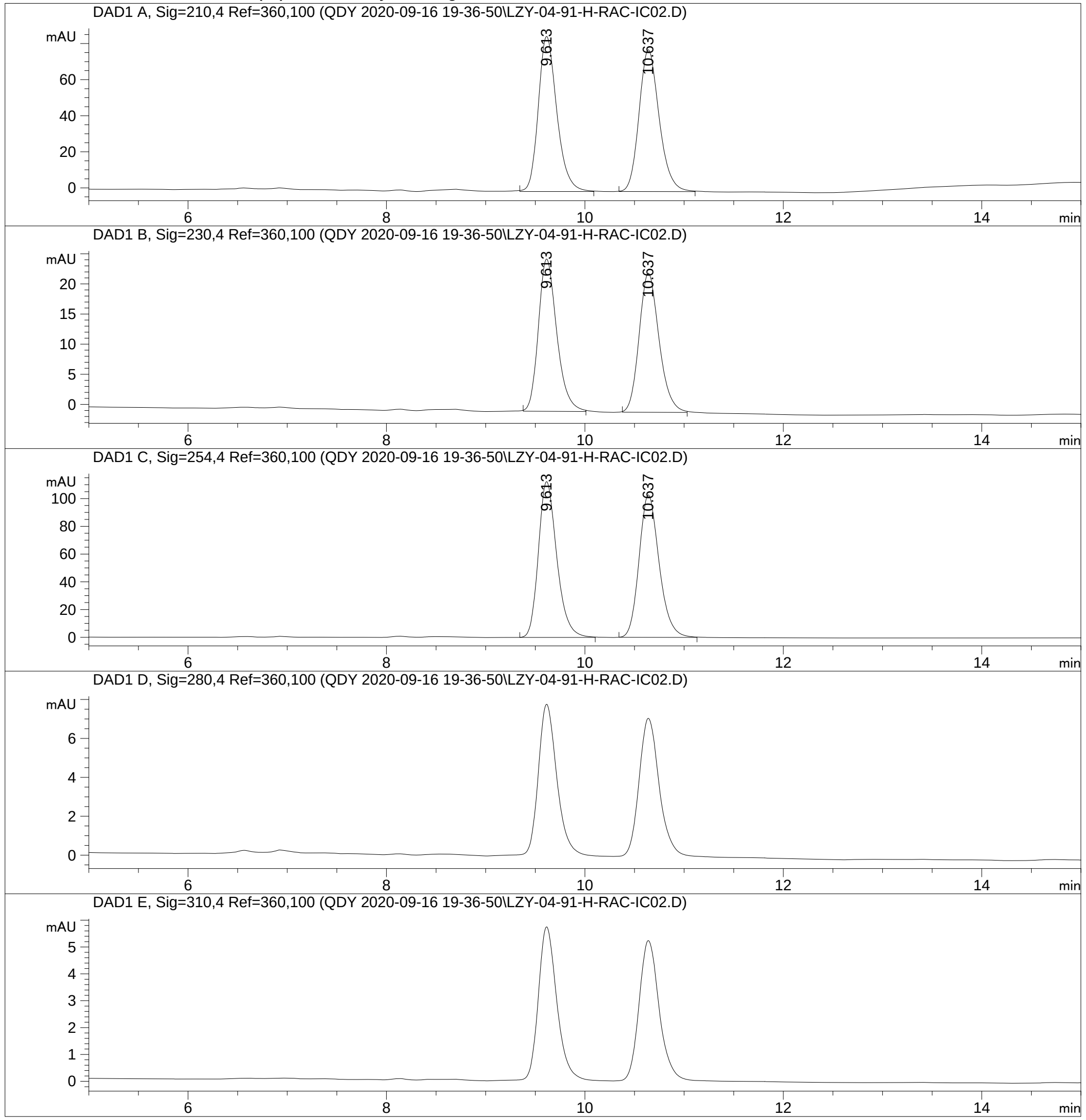

Data File C:\CHEM32\1\DATA\QDY 2020-09-16 19-36-50\LZY-04-91-H-RAC-IC02.D
Sample Name:

===
Area Percent Report
===

```
Sorted By             :      Signal
Multiplier            :      1.0000
Dilution              :      1.0000
Use Multiplier & Dilution Factor with ISTDs
```

Signal 1: DAD1 A, Sig=210,4 Ref=360,100

Peak #	RetTime [min]	Type	Width [min]	Area [mAU*s]	Height [mAU]	Area %
1	9.613	BB	0.1975	1117.19263	86.03838	50.2073
2	10.637	BB	0.2164	1107.96826	78.60401	49.7927

Totals : 2225.16089 164.64239

Signal 2: DAD1 B, Sig=230,4 Ref=360,100

Peak #	RetTime [min]	Type	Width [min]	Area [mAU*s]	Height [mAU]	Area %
1	9.613	BB	0.1964	325.97302	25.29374	50.2445
2	10.637	BB	0.2150	322.80103	23.09854	49.7555

Totals : 648.77405 48.39228

Signal 3: DAD1 C, Sig=254,4 Ref=360,100

Peak #	RetTime [min]	Type	Width [min]	Area [mAU*s]	Height [mAU]	Area %
1	9.613	BB	0.1964	1449.64172	112.41344	50.0818
2	10.637	BB	0.2160	1444.90576	102.77868	49.9182

Totals : 2894.54749 215.19212

Signal 4: DAD1 D, Sig=280,4 Ref=360,100

Signal 5: DAD1 E, Sig=310,4 Ref=360,100

===
*** End of Report ***

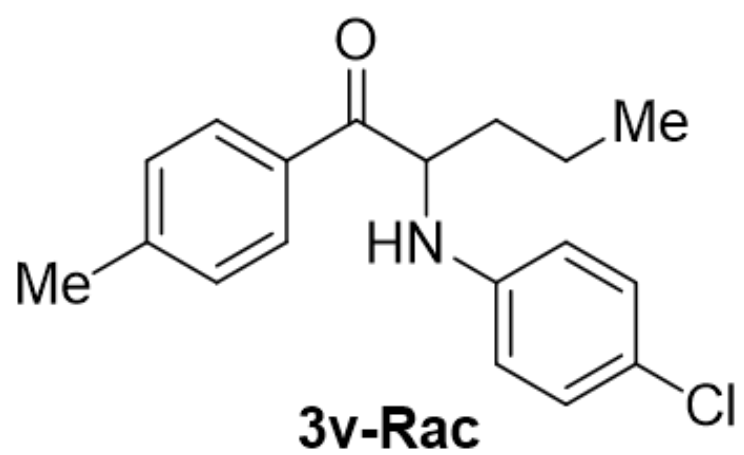

3v-Rac

Acq. Operator : Seq. Line : 2
Acq. Instrument : Instrument 1 Location : Vial 82
Injection Date : 9/19/2020 11:18:22 AM Inj : 1
Inj Volume : 5.000 µl
Different Inj Volume from Sequence ! Actual Inj Volume : 3.000 µl
Acq. Method : C:\CHEM32\1\DATA\QDY 2020-09-19 11-05-30\IC-02-30.M
Last changed : 9/19/2020 11:17:30 AM
(modified after loading)
Analysis Method : C:\CHEM32\1\METHODS\IC-007-90.M
Last changed : 11/25/2020 7:51:48 PM
(modified after loading)
Additional Info : Peak(s) manually integrated

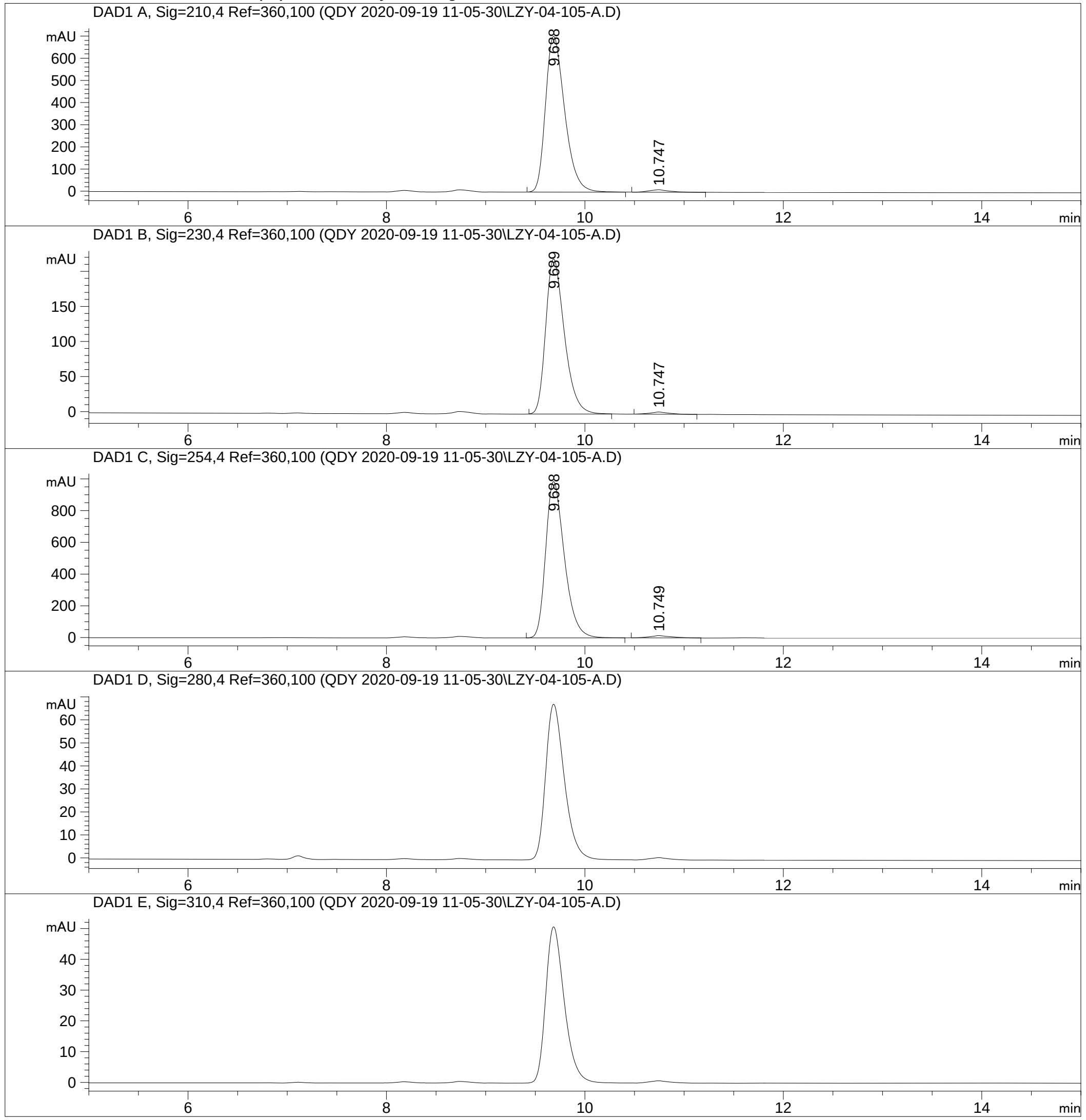

Data File C:\CHEM32\1\DATA\QDY 2020-09-19 11-05-30\LZY-04-105-A.D
Sample Name:

Area Percent Report

Sorted By : Signal
Multiplier : 1.0000
Dilution : 1.0000
Use Multiplier & Dilution Factor with ISTDs

Signal 1: DAD1 A, Sig=210,4 Ref=360,100

Peak #	RetTime [min]	Type	Width [min]	Area [mAU*s]	Height [mAU]	Area %
1	9.688	BB	0.2072	9474.05664	702.91724	98.2325
2	10.747	BB	0.2259	170.46451	11.43823	1.7675

Totals : 9644.52115 714.35546

Signal 2: DAD1 B, Sig=230,4 Ref=360,100

Peak #	RetTime [min]	Type	Width [min]	Area [mAU*s]	Height [mAU]	Area %
1	9.689	BB	0.2032	2904.46436	221.17552	98.5749
2	10.747	BB	0.2096	41.99089	3.14886	1.4251

Totals : 2946.45524 224.32438

Signal 3: DAD1 C, Sig=254,4 Ref=360,100

Peak #	RetTime [min]	Type	Width [min]	Area [mAU*s]	Height [mAU]	Area %
1	9.688	BB	0.2033	1.29498e4	985.35712	98.4360
2	10.749	BB	0.2165	205.75432	14.59243	1.5640

Totals : 1.31556e4 999.94954

Signal 4: DAD1 D, Sig=280,4 Ref=360,100

Signal 5: DAD1 E, Sig=310,4 Ref=360,100

*** End of Report ***

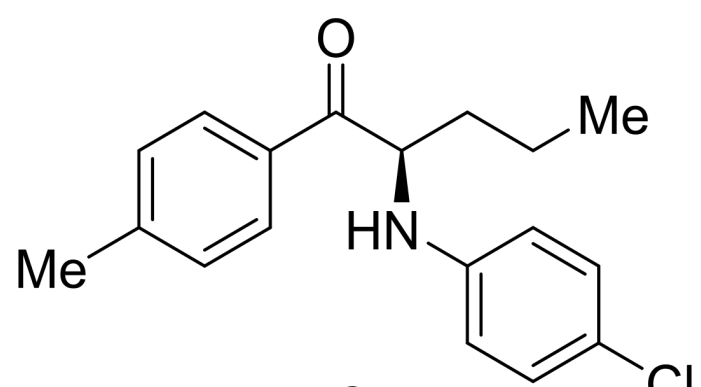

3v

===

Acq. Operator : Seq. Line : 10
Acq. Instrument : Instrument 1 Location : Vial 84
Injection Date : 9/16/2020 11:38:14 PM Inj : 1
Inj Volume : 5.000 µl
Different Inj Volume from Sequence ! Actual Inj Volume : 2.000 µl
Acq. Method : C:\CHEM32\1\DATA\QDY 2020-09-16 19-36-50\OD-02-30.M
Last changed : 9/16/2020 11:37:22 PM
(modified after loading)
Analysis Method : C:\CHEM32\1\METHODS\IC-007-90.M
Last changed : 11/26/2020 3:06:22 PM
(modified after loading)
Additional Info : Peak(s) manually integrated

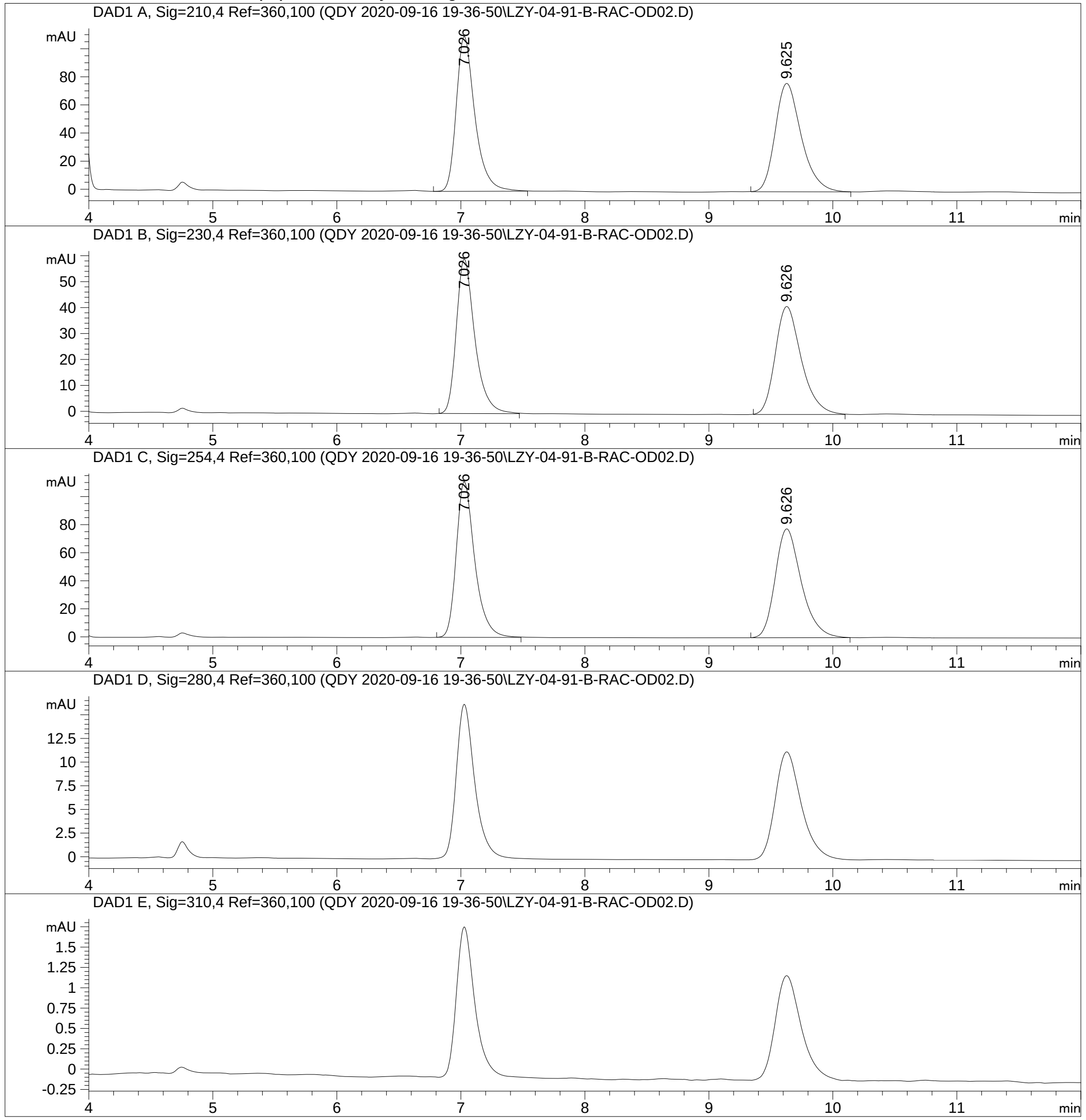

Data File C:\CHEM32\1\DATA\QDY 2020-09-16 19-36-50\LZY-04-91-B-RAC-OD02.D
Sample Name:

===
Area Percent Report
===

Sorted By : Signal
Multiplier : 1.0000
Dilution : 1.0000
Use Multiplier & Dilution Factor with ISTDs

Signal 1: DAD1 A, Sig=210,4 Ref=360,100

Peak #	RetTime [min]	Type	Width [min]	Area [mAU*s]	Height [mAU]	Area %
1	7.026	VB	0.1580	1153.11218	110.27843	50.4922
2	9.625	BB	0.2251	1130.63147	77.09918	49.5078

Totals : 2283.74365 187.37762

Signal 2: DAD1 B, Sig=230,4 Ref=360,100

Peak #	RetTime [min]	Type	Width [min]	Area [mAU*s]	Height [mAU]	Area %
1	7.026	BB	0.1572	619.20068	59.59966	50.3006
2	9.626	BB	0.2234	611.79974	41.65316	49.6994

Totals : 1231.00043 101.25282

Signal 3: DAD1 C, Sig=254,4 Ref=360,100

Peak #	RetTime [min]	Type	Width [min]	Area [mAU*s]	Height [mAU]	Area %
1	7.026	BB	0.1567	1149.13672	111.04838	50.2283
2	9.626	BB	0.2232	1138.68835	77.58112	49.7717

Totals : 2287.82507 188.62949

Signal 4: DAD1 D, Sig=280,4 Ref=360,100

Signal 5: DAD1 E, Sig=310,4 Ref=360,100

===
*** End of Report ***

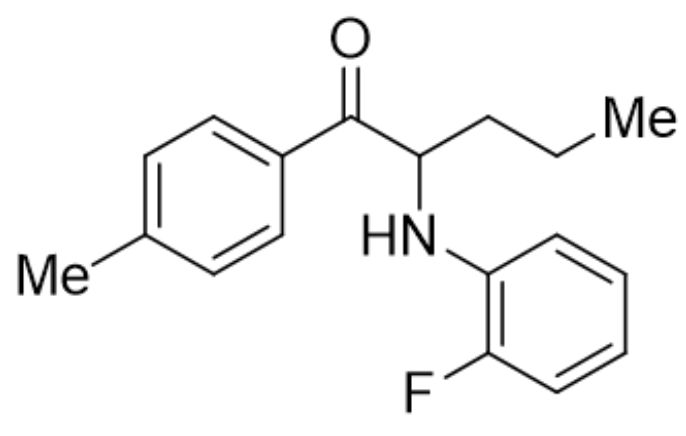

3w-Rac

===

Acq. Operator : Seq. Line : 33
Acq. Instrument : Instrument 1 Location : Vial 92
Injection Date : 9/27/2020 11:31:05 PM Inj : 1
Inj Volume : 5.000 µl
Different Inj Volume from Sequence ! Actual Inj Volume : 2.000 µl
Acq. Method : C:\CHEM32\1\DATA\QDY 2020-09-27 13-49-22\OD-02-20.M
Last changed : 2/16/2019 2:07:27 PM
Analysis Method : C:\CHEM32\1\METHODS\IC-007-90.M
Last changed : 11/26/2020 3:06:22 PM
(modified after loading)
Additional Info : Peak(s) manually integrated

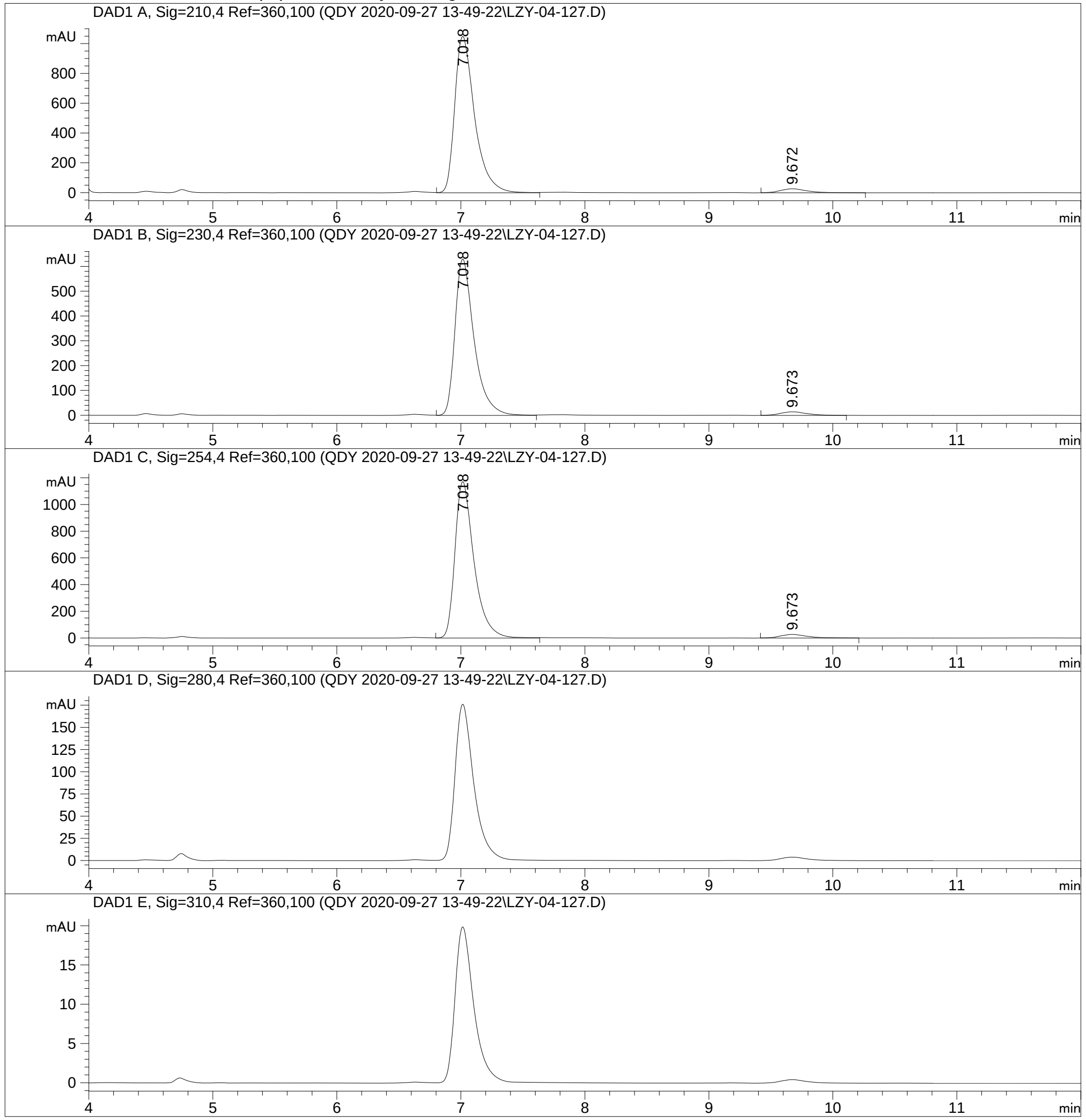

===
Area Percent Report
===

Sorted By : Signal
Multiplier : 1.0000
Dilution : 1.0000
Use Multiplier & Dilution Factor with ISTDs

Signal 1: DAD1 A, Sig=210,4 Ref=360,100

Peak #	RetTime [min]	Type	Width [min]	Area [mAU*s]	Height [mAU]	Area %
1	7.018	VB	0.1701	1.17065e4	1049.74023	96.6731
2	9.672	BB	0.2257	402.86438	26.74663	3.3269

Totals : 1.21094e4 1076.48686

Signal 2: DAD1 B, Sig=230,4 Ref=360,100

Peak #	RetTime [min]	Type	Width [min]	Area [mAU*s]	Height [mAU]	Area %
1	7.018	VB	0.1630	6754.87158	630.64807	96.9563
2	9.673	BB	0.2239	212.04956	14.38777	3.0437

Totals : 6966.92114 645.03584

Signal 3: DAD1 C, Sig=254,4 Ref=360,100

Peak #	RetTime [min]	Type	Width [min]	Area [mAU*s]	Height [mAU]	Area %
1	7.018	VB	0.1624	1.24957e4	1171.90833	96.8857
2	9.673	BB	0.2268	401.66373	26.80191	3.1143

Totals : 1.28973e4 1198.71023

Signal 4: DAD1 D, Sig=280,4 Ref=360,100

Signal 5: DAD1 E, Sig=310,4 Ref=360,100

===
*** End of Report ***

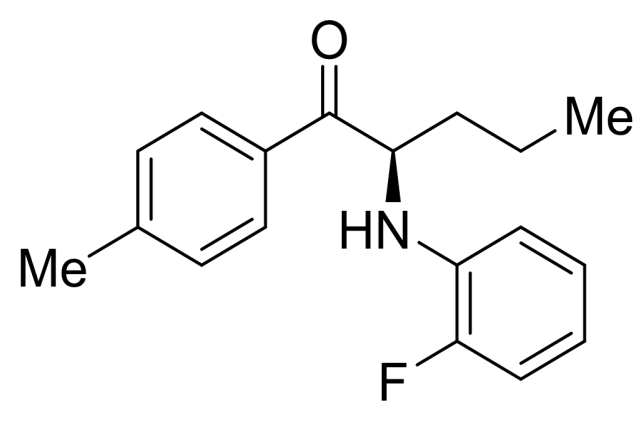

3w

===

Acq. Operator	:	Seq. Line :	4
Acq. Instrument	: Instrument 1	Location :	Vial 84
Injection Date	: 9/15/2020 4:24:55 PM	Inj :	1
		Inj Volume :	5.000 µl

Different Inj Volume from Sequence ! Actual Inj Volume : 2.000 µl
Acq. Method : C:\CHEM32\1\DATA\QDY 2020-09-15 15-29-52\IC-03-30.M
Last changed : 9/15/2020 4:24:02 PM
(modified after loading)
Analysis Method : C:\CHEM32\1\METHODS\IC-007-90.M
Last changed : 11/26/2020 2:54:09 PM
(modified after loading)
Additional Info : Peak(s) manually integrated

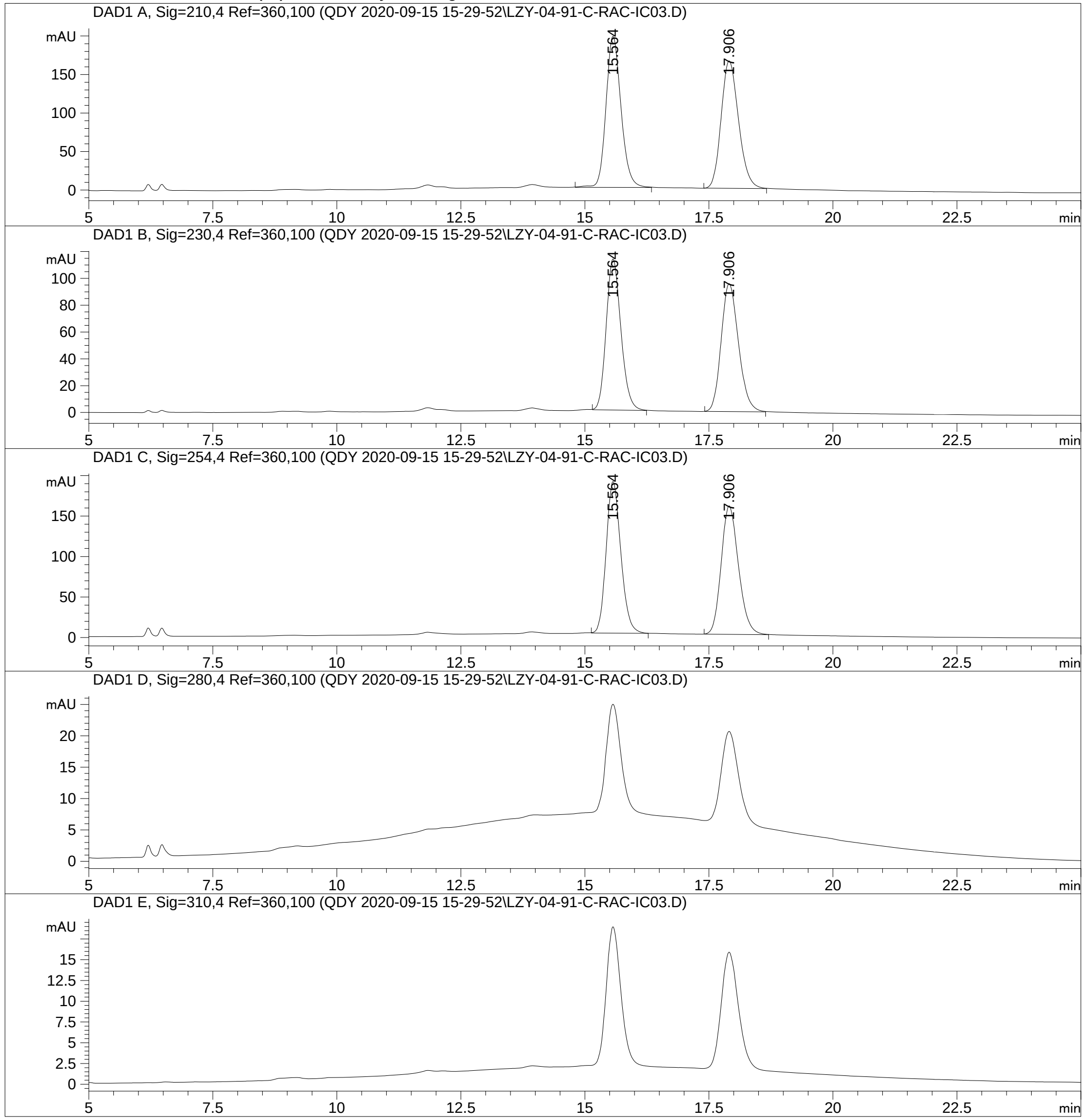

Data File C:\CHEM32\1\DATA\QDY 2020-09-15 15-29-52\LZY-04-91-C-RAC-IC03.D
Sample Name:

===

Area Percent Report

===

Sorted By : Signal
Multiplier : 1.0000
Dilution : 1.0000
Use Multiplier & Dilution Factor with ISTDs

Signal 1: DAD1 A, Sig=210,4 Ref=360,100

Peak #	RetTime [min]	Type	Width [min]	Area [mAU*s]	Height [mAU]	Area %
1	15.564	BB	0.3234	4124.68018	196.10448	50.5390
2	17.906	BB	0.3785	4036.70801	165.53046	49.4610

Totals : 8161.38818 361.63493

Signal 2: DAD1 B, Sig=230,4 Ref=360,100

Peak #	RetTime [min]	Type	Width [min]	Area [mAU*s]	Height [mAU]	Area %
1	15.564	BB	0.3187	2324.27734	112.66708	50.0512
2	17.906	BB	0.3755	2319.52246	95.44273	49.9488

Totals : 4643.79980 208.10981

Signal 3: DAD1 C, Sig=254,4 Ref=360,100

Peak #	RetTime [min]	Type	Width [min]	Area [mAU*s]	Height [mAU]	Area %
1	15.564	BB	0.3181	3851.75708	187.19525	49.9349
2	17.906	BB	0.3758	3861.79419	158.75554	50.0651

Totals : 7713.55127 345.95079

Signal 4: DAD1 D, Sig=280,4 Ref=360,100

Signal 5: DAD1 E, Sig=310,4 Ref=360,100

===

*** End of Report ***

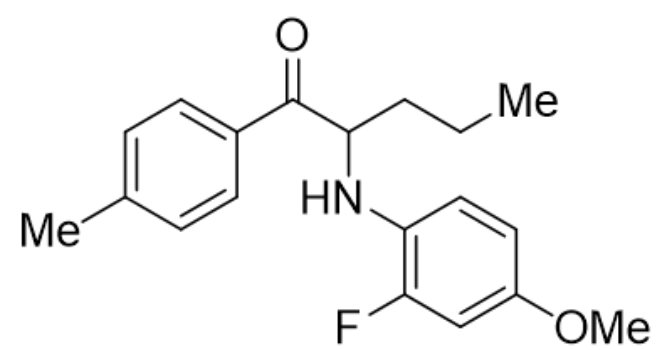

3x-Rac

Data File C:\CHEM32\1\DATA\QDY 2020-09-21 10-19-21\LZY-04-106-A.D
Sample Name:

===

Acq. Operator	:	Seq. Line :	2
Acq. Instrument	: Instrument 1	Location :	Vial 91
Injection Date	: 9/21/2020 11:10:54 AM	Inj :	1
		Inj Volume :	5.000 µl

Different Inj Volume from Sequence ! Actual Inj Volume : 3.000 µl
Acq. Method : C:\CHEM32\1\DATA\QDY 2020-09-21 10-19-21\IC-03-40.M
Last changed : 9/21/2020 11:10:01 AM
(modified after loading)
Analysis Method : C:\CHEM32\1\METHODS\IC-007-90.M
Last changed : 11/25/2020 8:01:49 PM
(modified after loading)
Additional Info : Peak(s) manually integrated

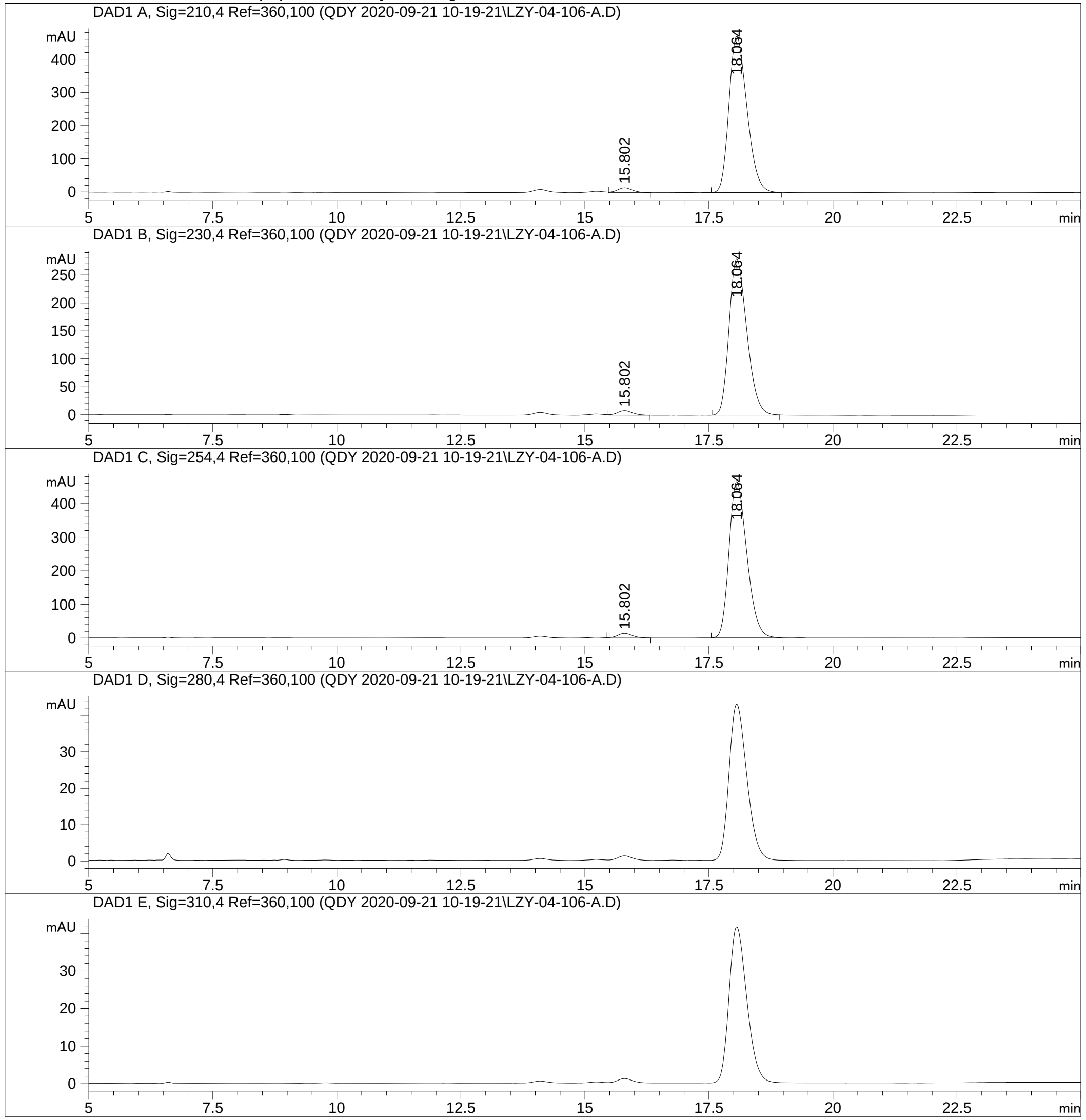

Data File C:\CHEM32\1\DATA\QDY 2020-09-21 10-19-21\LZY-04-106-A.D
Sample Name:

===
Area Percent Report
===

Sorted By : Signal
Multiplier : 1.0000
Dilution : 1.0000
Use Multiplier & Dilution Factor with ISTDs

Signal 1: DAD1 A, Sig=210,4 Ref=360,100

Peak #	RetTime [min]	Type	Width [min]	Area [mAU*s]	Height [mAU]	Area %
1	15.802	VB	0.3209	305.72061	14.56484	2.5071
2	18.064	BB	0.3927	1.18886e4	470.71051	97.4929

Totals : 1.21943e4 485.27535

Signal 2: DAD1 B, Sig=230,4 Ref=360,100

Peak #	RetTime [min]	Type	Width [min]	Area [mAU*s]	Height [mAU]	Area %
1	15.802	VB	0.3179	171.69669	8.21223	2.4013
2	18.064	BB	0.3856	6978.53564	279.22427	97.5987

Totals : 7150.23233 287.43650

Signal 3: DAD1 C, Sig=254,4 Ref=360,100

Peak #	RetTime [min]	Type	Width [min]	Area [mAU*s]	Height [mAU]	Area %
1	15.802	VB	0.3195	283.56161	13.58589	2.3832
2	18.064	BB	0.3854	1.16146e4	465.03595	97.6168

Totals : 1.18982e4 478.62184

Signal 4: DAD1 D, Sig=280,4 Ref=360,100

Signal 5: DAD1 E, Sig=310,4 Ref=360,100

===
*** End of Report ***

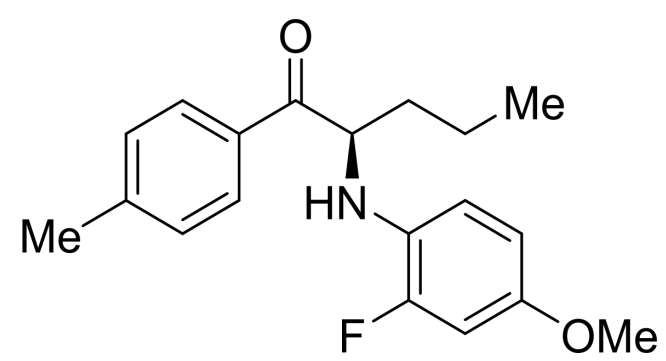

3x

Data File C:\CHEM32\1\DATA\QDY 2020-09-24 11-48-22\LZY-04-115-C-RAC-IC007.D
Sample Name:

Acq. Operator :	Seq. Line : 38
Acq. Instrument : Instrument 1	Location : Vial 85
Injection Date : 9/25/2020 12:25:24 AM	Inj : 1
	Inj Volume : 5.000 µl
Different Inj Volume from Sequence !	Actual Inj Volume : 2.000 µl

Acq. Method : C:\CHEM32\1\DATA\QDY 2020-09-24 11-48-22\IC-007-60.M
Last changed : 9/4/2018 2:53:16 PM
Analysis Method : C:\CHEM32\1\METHODS\IC-007-90.M
Last changed : 11/25/2020 8:33:07 PM
(modified after loading)
Additional Info : Peak(s) manually integrated

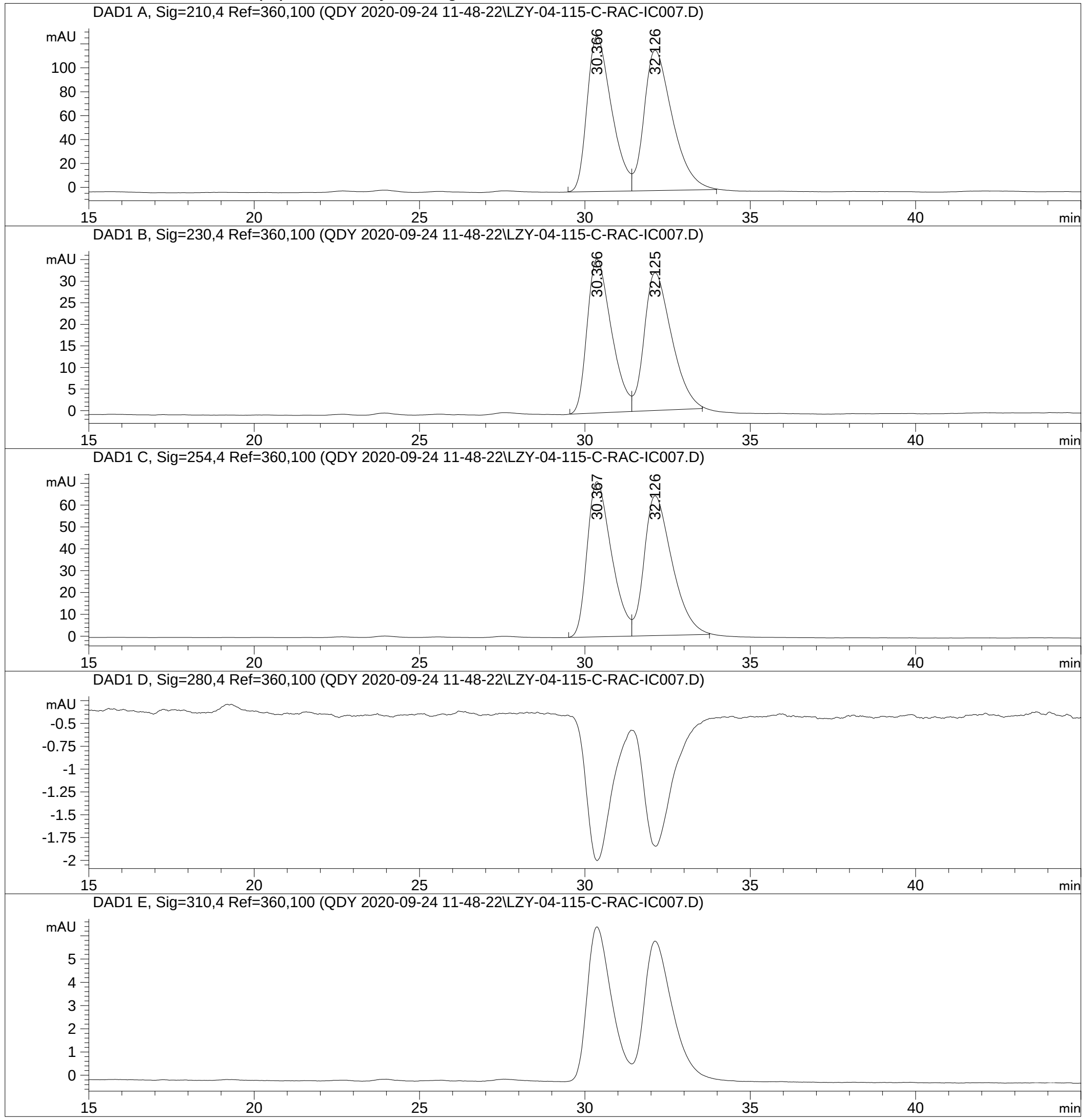

Data File C:\CHEM32\1\DATA\QDY 2020-09-24 11-48-22\LZY-04-115-C-RAC-IC007.D
Sample Name:

Area Percent Report

Sorted By : Signal
Multiplier : 1.0000
Dilution : 1.0000
Use Multiplier & Dilution Factor with ISTDs

Signal 1: DAD1 A, Sig=210,4 Ref=360,100

Peak #	RetTime [min]	Type	Width [min]	Area [mAU*s]	Height [mAU]	Area %
1	30.366	BV	0.7788	6661.35986	129.79579	48.8182
2	32.126	VB	0.9063	6983.88965	117.34990	51.1818

Totals : 1.36452e4 247.14569

Signal 2: DAD1 B, Sig=230,4 Ref=360,100

Peak #	RetTime [min]	Type	Width [min]	Area [mAU*s]	Height [mAU]	Area %
1	30.366	BV	0.7769	1813.59265	35.68384	49.7094
2	32.125	VB	0.8624	1834.79968	31.93094	50.2906

Totals : 3648.39233 67.61478

Signal 3: DAD1 C, Sig=254,4 Ref=360,100

Peak #	RetTime [min]	Type	Width [min]	Area [mAU*s]	Height [mAU]	Area %
1	30.367	BV	0.7829	3635.35254	71.05975	49.2203
2	32.126	VB	0.8958	3750.52979	63.98836	50.7797

Totals : 7385.88232 135.04811

Signal 4: DAD1 D, Sig=280,4 Ref=360,100

Signal 5: DAD1 E, Sig=310,4 Ref=360,100

*** End of Report ***

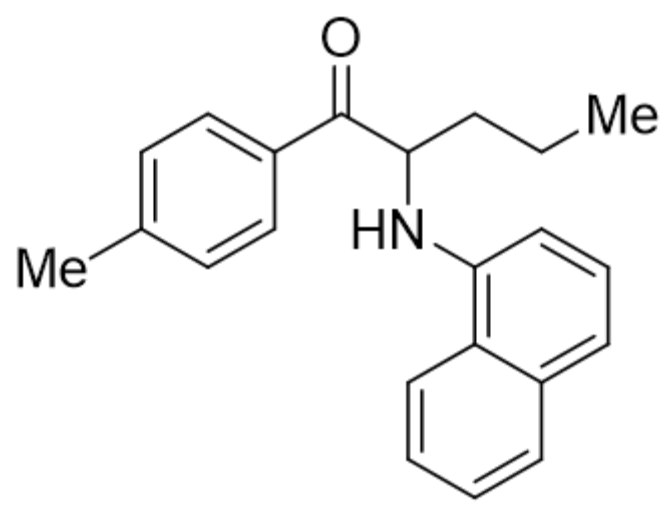

3y-Rac

Data File C:\CHEM32\1\DATA\QDY 2020-09-27 13-49-22\LZY-04-125-D.D
Sample Name:

===

Acq. Operator : Seq. Line : 36
Acq. Instrument : Instrument 1 Location : Vial 91
Injection Date : 9/28/2020 12:14:21 AM Inj : 1
Inj Volume : 5.000 µl
Different Inj Volume from Sequence ! Actual Inj Volume : 2.000 µl
Acq. Method : C:\CHEM32\1\DATA\QDY 2020-09-27 13-49-22\IC-007-60.M
Last changed : 9/4/2018 2:53:16 PM
Analysis Method : C:\CHEM32\1\METHODS\IC-007-90.M
Last changed : 11/25/2020 8:33:07 PM
(modified after loading)
Additional Info : Peak(s) manually integrated

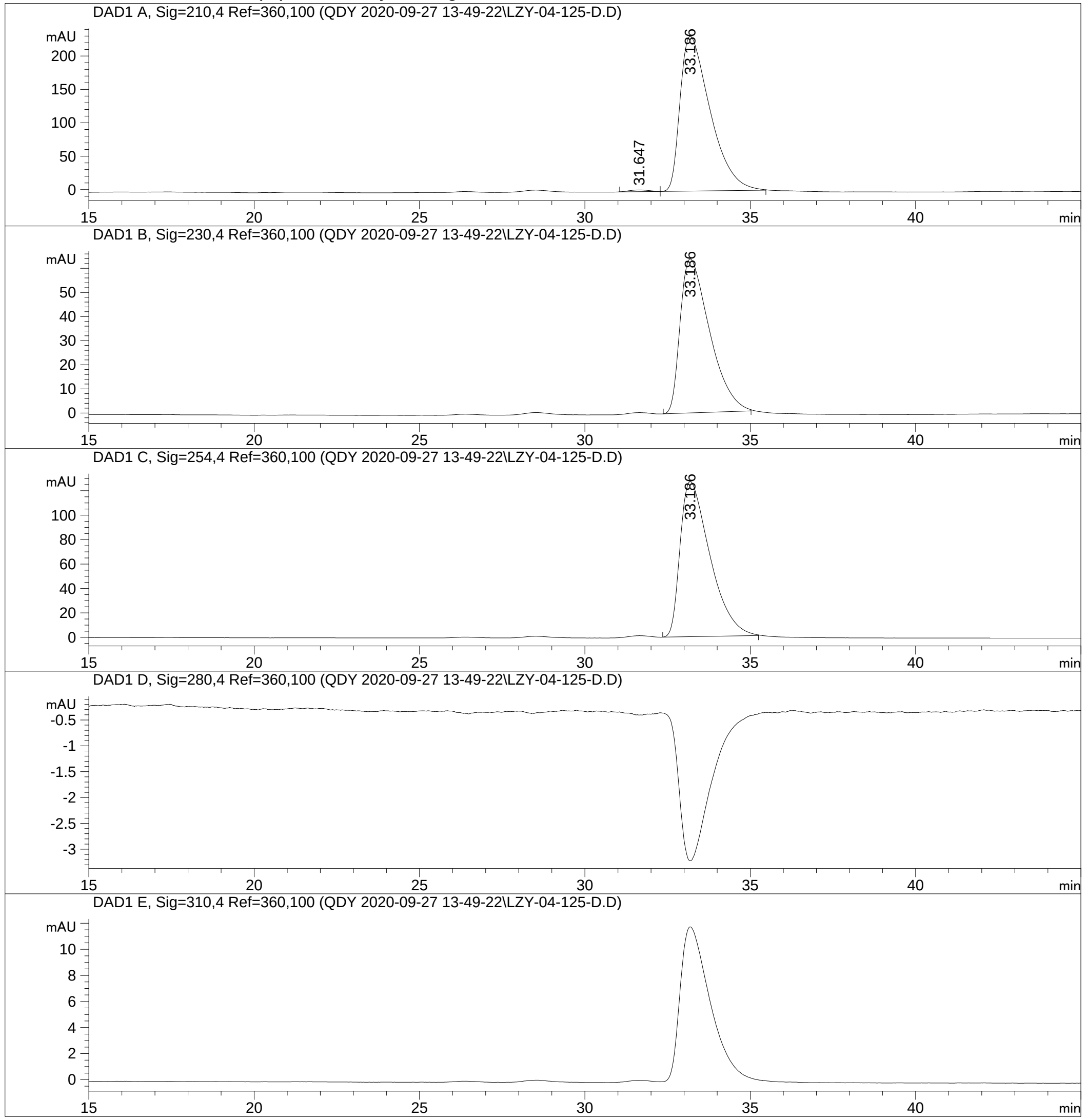

Data File C:\CHEM32\1\DATA\QDY 2020-09-27 13-49-22\LZY-04-125-D.D
Sample Name:

===

Area Percent Report

===

Sorted By : Signal
Multiplier : 1.0000
Dilution : 1.0000
Use Multiplier & Dilution Factor with ISTDs

Signal 1: DAD1 A, Sig=210,4 Ref=360,100

Peak #	RetTime [min]	Type	Width [min]	Area [mAU*s]	Height [mAU]	Area %
1	31.647	BV	0.4976	106.13826	2.61488	0.7208
2	33.186	VB	0.9464	1.46191e4	231.62132	99.2792

Totals : 1.47252e4 234.23620

Signal 2: DAD1 B, Sig=230,4 Ref=360,100

Peak #	RetTime [min]	Type	Width [min]	Area [mAU*s]	Height [mAU]	Area %
1	33.186	BB	0.9316	3950.03491	63.86611	100.0000

Totals : 3950.03491 63.86611

Signal 3: DAD1 C, Sig=254,4 Ref=360,100

Peak #	RetTime [min]	Type	Width [min]	Area [mAU*s]	Height [mAU]	Area %
1	33.186	BB	0.9515	7935.16162	126.90465	100.0000

Totals : 7935.16162 126.90465

Signal 4: DAD1 D, Sig=280,4 Ref=360,100

Signal 5: DAD1 E, Sig=310,4 Ref=360,100

===

*** End of Report ***

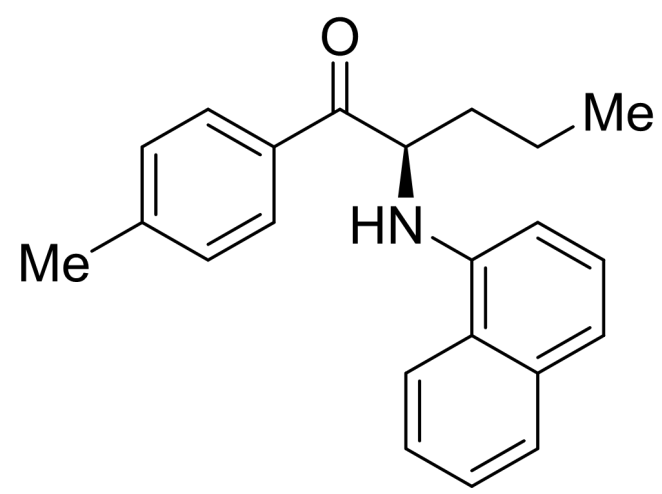

3y

Data File C:\Users\P...on\1\Data\SUN\SUN 2026-06-23 22-04-00\002-P1-C1-luoy-2-124d-IC-95.D
Sample Name: luoy-2-124d-IC-95

===

```
Acq. Operator   : SYSTEM                      Seq. Line :    2
Acq. Instrument : LC1260                       Location :   P1-C-01
Injection Date  : 6/23/2026 10:17:18 PM             Inj :    1
                                             Inj Volume : 5.000 µl
Different Inj Volume from Sample Entry!  Actual Inj Volume : 10.000 µl
Acq. Method     : C:\Users\Public\Documents\ChemStation\1\Data\SUN\SUN 2026-06-23 22-04-00
                  \IC3-05-30.M
Last changed    : 6/23/2026 10:48:34 PM by SYSTEM
                  (modified after loading)
Analysis Method : C:\Users\Public\Documents\ChemStation\1\Data\SUN\SUN 2026-06-23 22-04-00
                  \IC3-05-30.M (Sequence Method)
Last changed    : 7/7/2026 6:55:45 PM by SYSTEM
                  (modified after loading)
Additional Info : Peak(s) manually integrated
```

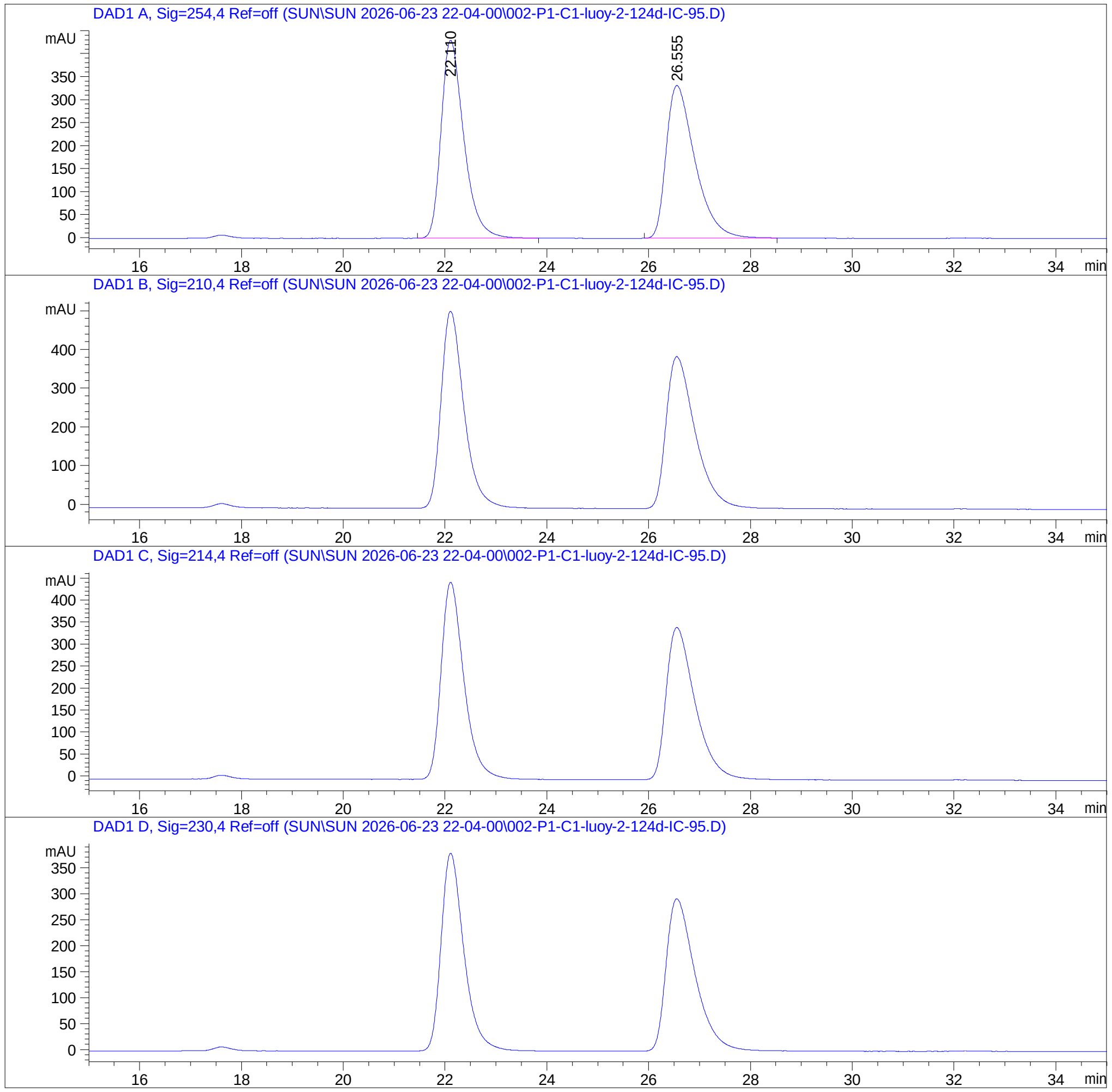

Data File C:\Users\P...on\1\Data\SUN\SUN 2026-06-23 22-04-00\002-P1-C1-luoy-2-124d-IC-95.D
Sample Name: luoy-2-124d-IC-95

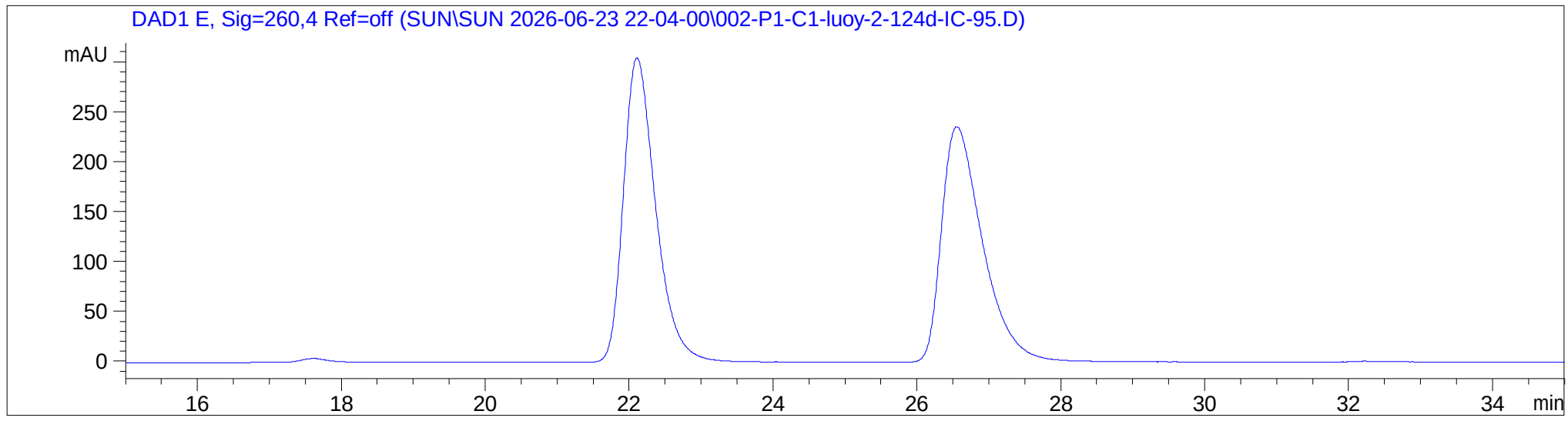

===

Area Percent Report

===

```
Sorted By            :      Signal
Multiplier           :      1.0000
Dilution             :      1.0000
Use Multiplier & Dilution Factor with ISTDs
```

Signal 1: DAD1 A, Sig=254,4 Ref=off

Peak #	RetTime [min]	Type	Width [min]	Area [mAU*s]	Height [mAU]	Area %
1	22.110	BB	0.4781	1.33578e4	430.38504	50.5957
2	26.555	BB	0.5943	1.30432e4	332.06732	49.4043
Totals :				2.64010e4	762.45236	

Signal 2: DAD1 B, Sig=210,4 Ref=off

Signal 3: DAD1 C, Sig=214,4 Ref=off

Signal 4: DAD1 D, Sig=230,4 Ref=off

Signal 5: DAD1 E, Sig=260,4 Ref=off

===

*** End of Report ***

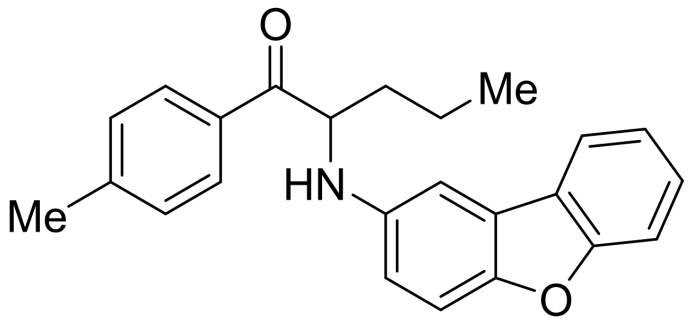

3z-Rac

===

Acq. Operator : SYSTEM   Seq. Line : 2
Acq. Instrument : LC1260   Location : P1-C-01
Injection Date : 6/28/2026 1:38:30 PM   Inj : 1
Inj Volume : 5.000 µl
Different Inj Volume from Sample Entry! Actual Inj Volume : 15.000 µl
Acq. Method : C:\Users\Public\Documents\ChemStation\1\Data\SUN\SUN 2026-06-28 13-25-52 \IC3-05-30.M
Last changed : 6/28/2026 2:07:00 PM by SYSTEM (modified after loading)
Analysis Method : C:\Users\Public\Documents\ChemStation\1\Data\SUN\SUN 2026-06-28 13-25-52 \IC3-05-30.M (Sequence Method)
Last changed : 7/7/2026 7:00:05 PM by SYSTEM (modified after loading)
Additional Info : Peak(s) manually integrated

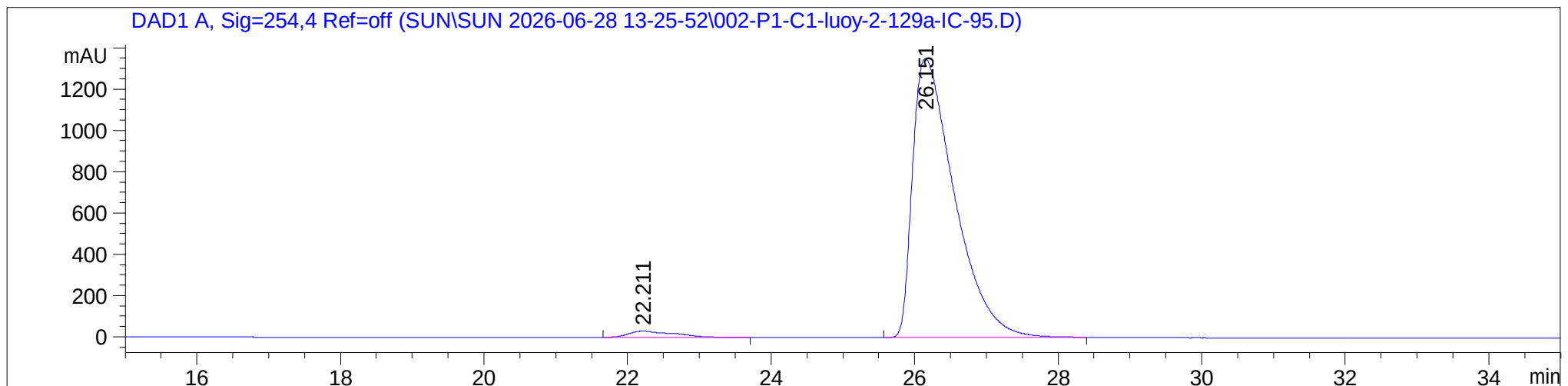

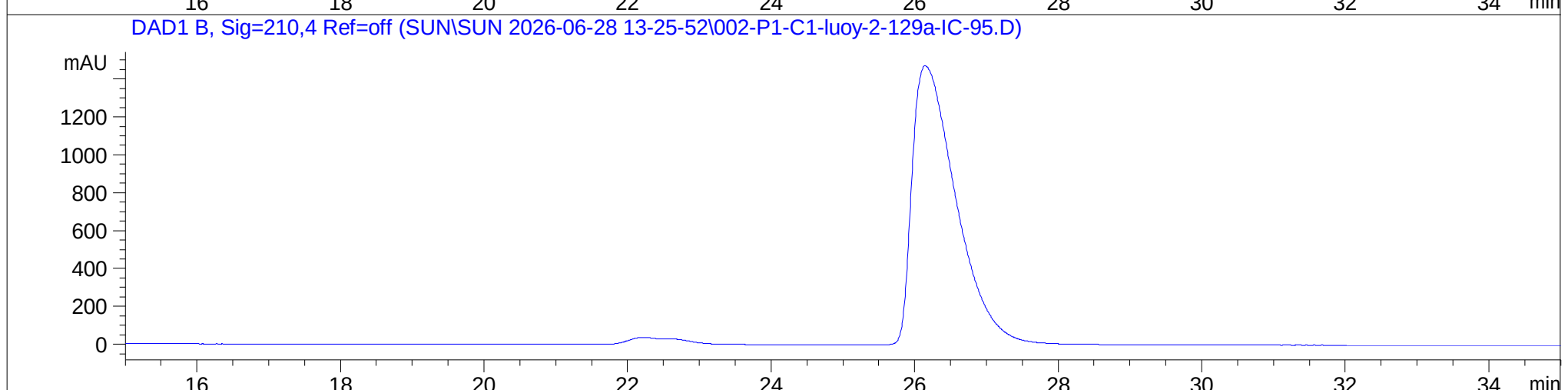

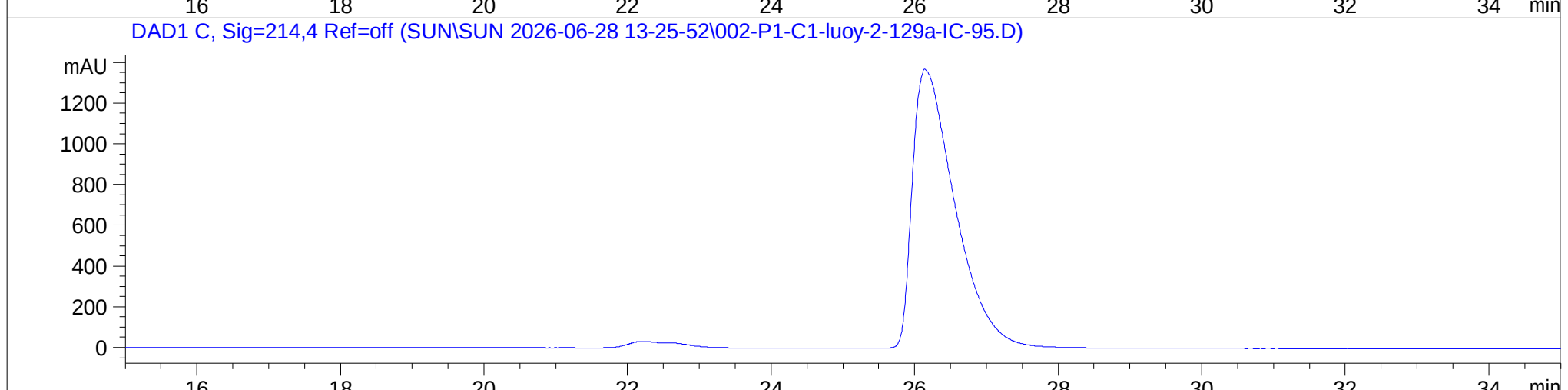

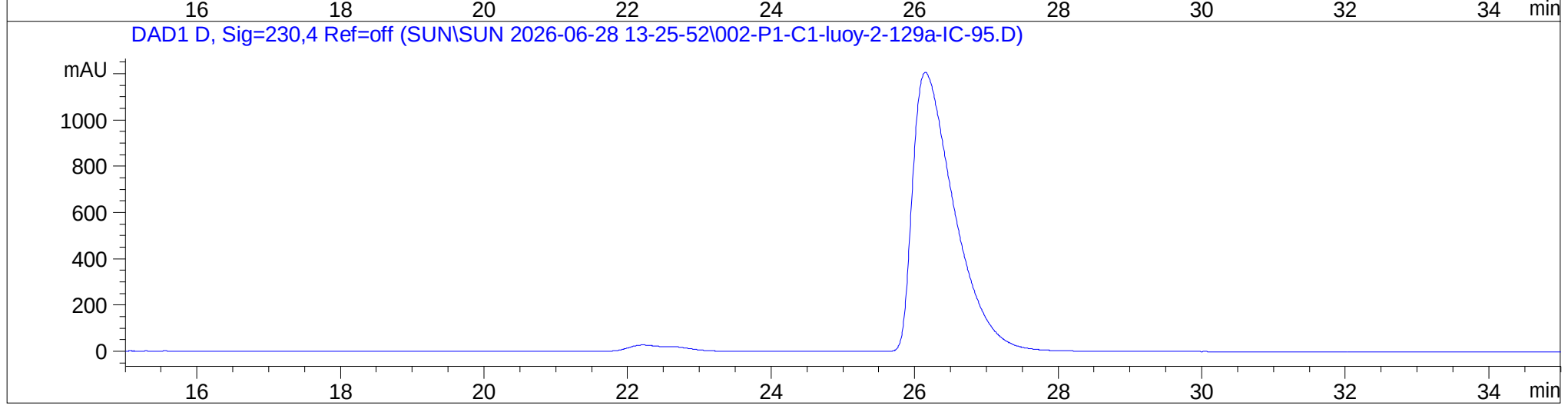

Data File C:\Users\P...on\1\Data\SUN\SUN 2026-06-28 13-25-52\002-P1-C1-luoy-2-129a-IC-95.D
Sample Name: luoy-2-129a-IC-95

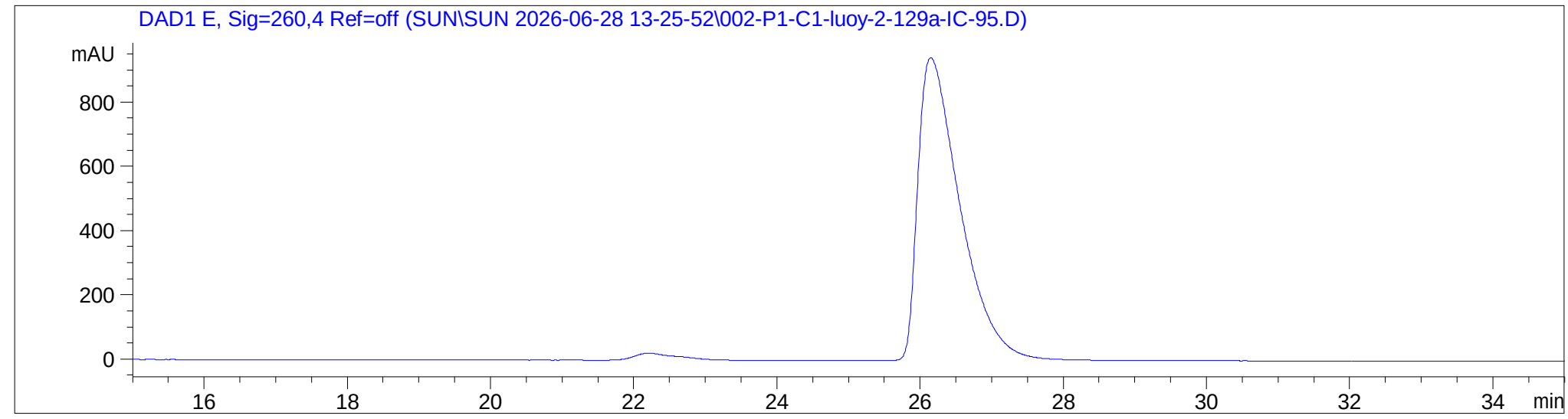

===

Area Percent Report

===

Sorted By : Signal
Multiplier : 1.0000
Dilution : 1.0000
Use Multiplier & Dilution Factor with ISTDs

Signal 1: DAD1 A, Sig=254,4 Ref=off

Peak #	RetTime [min]	Type	Width [min]	Area [mAU*s]	Height [mAU]	Area %
1	22.211	BB R	0.6000	1378.54785	30.69104	2.4523
2	26.151	BB	0.5573	5.48358e4	1351.80383	97.5477

Totals : 5.62143e4 1382.49488

Signal 2: DAD1 B, Sig=210,4 Ref=off

Signal 3: DAD1 C, Sig=214,4 Ref=off

Signal 4: DAD1 D, Sig=230,4 Ref=off

Signal 5: DAD1 E, Sig=260,4 Ref=off

===

*** End of Report ***

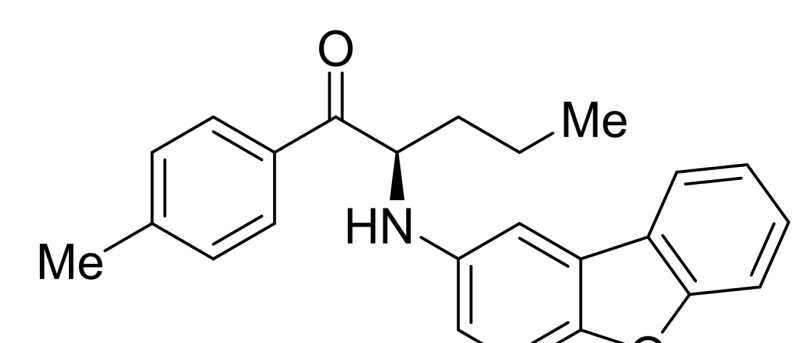

3z

Data File C:\Users\P...on\1\Data\SUN\SUN 2026-07-05 14-29-53\006-P1-C1-luoy-2-139c-IC-80.D
Sample Name: luoy-2-139c-IC-80

===

Acq. Operator : SYSTEM Seq. Line : 6
Sample Operator : SYSTEM
Acq. Instrument : HPLC Location : P1-C-01
Injection Date : 5/7/2026 4:51:16 pm Inj : 1
Inj Volume : 2.000 µl
Different Inj Volume from Sample Entry! Actual Inj Volume : 20.000 µl
Acq. Method : C:\Users\Public\Documents\ChemStation\1\Data\SUN\SUN 2026-07-05 14-29-53\IC3-20-40.M
Last changed : 5/7/2026 5:16:19 pm by SYSTEM
(modified after loading)
Analysis Method : C:\Users\Public\Documents\ChemStation\1\Data\SUN\SUN 2026-07-05 14-29-53\IC3-20-40.M (Sequence Method)
Last changed : 7/7/2026 7:48:45 pm by SYSTEM
(modified after loading)
Additional Info : Peak(s) manually integrated

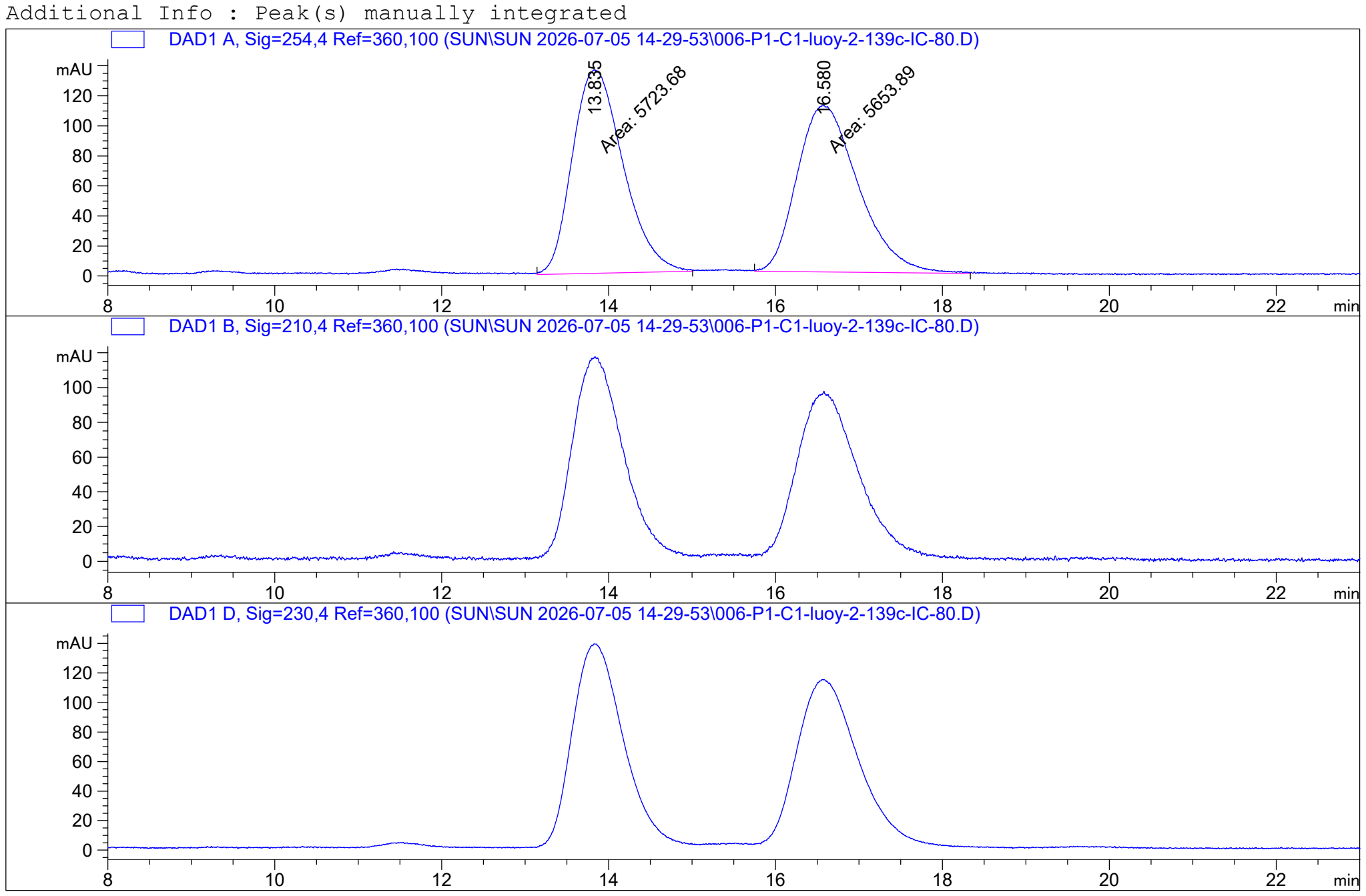

===

Area Percent Report

===

Sorted By : Signal
Multiplier : 1.0000
Dilution : 1.0000
Use Multiplier & Dilution Factor with ISTDs

Data File C:\Users\P...on\1\Data\SUN\SUN 2026-07-05 14-29-53\006-P1-C1-luoy-2-139c-IC-80.D
Sample Name: luoy-2-139c-IC-80

Signal 1: DAD1 A, Sig=254,4 Ref=360,100

Peak #	RetTime [min]	Type	Width [min]	Area [mAU*s]	Height [mAU]	Area %
1	13.835	MM	0.7037	5723.67920	135.55324	50.3067
2	16.580	MM	0.8502	5653.88623	110.84061	49.6933

Totals : 1.13776e4 246.39385

Signal 2: DAD1 B, Sig=210,4 Ref=360,100

Signal 3: DAD1 D, Sig=230,4 Ref=360,100

===

*** End of Report ***

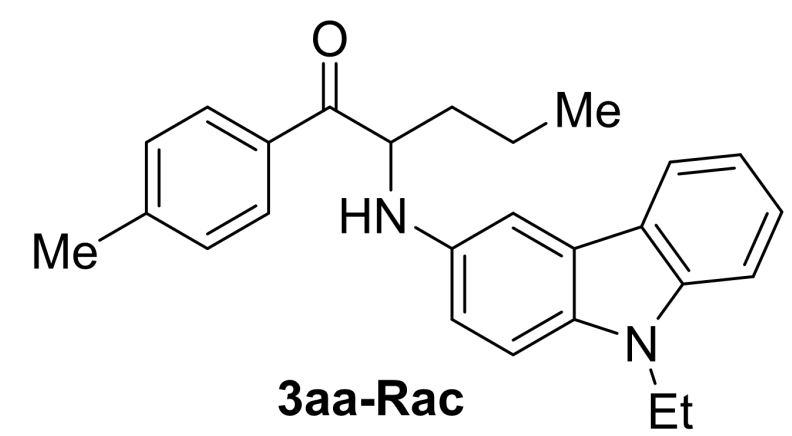

3aa-Rac

Data File C:\Users\P...on\1\Data\SUN\SUN 2026-07-07 11-15-38\022-P1-C1-luoy-2-141a-IC-80.D
Sample Name: luoy-2-141a-IC-80

===

Acq. Operator : SYSTEM   Seq. Line : 22
Sample Operator : SYSTEM
Acq. Instrument : HPLC   Location : P1-C-01
Injection Date : 7/7/2026 7:31:53 pm   Inj : 1
Inj Volume : 2.000 µl
Different Inj Volume from Sample Entry! Actual Inj Volume : 10.000 µl
Acq. Method : C:\Users\Public\Documents\ChemStation\1\Data\SUN\SUN 2026-07-07 11-15-38\IC3-20-30.M
Last changed : 7/7/2026 7:58:18 pm by SYSTEM
(modified after loading)
Analysis Method : C:\Users\Public\Documents\ChemStation\1\Data\SUN\SUN 2026-07-07 11-15-38\IC3-20-30.M (Sequence Method)
Last changed : 7/7/2026 8:28:56 pm by SYSTEM
(modified after loading)
Additional Info : Peak(s) manually integrated

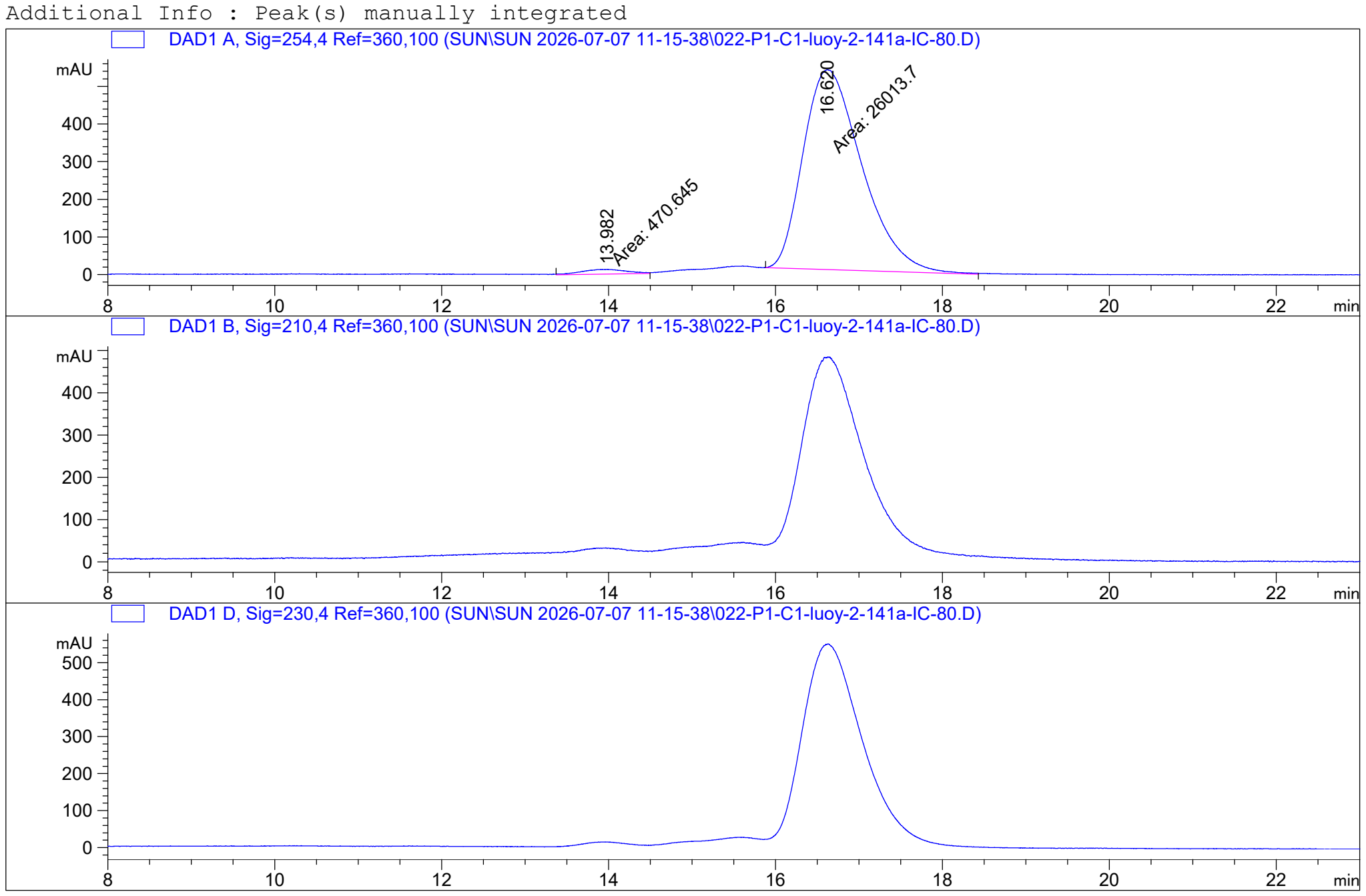

===

Area Percent Report

===

Sorted By : Signal
Multiplier : 1.0000
Dilution : 1.0000
Use Multiplier & Dilution Factor with ISTDs

Data File C:\Users\P...on\1\Data\SUN\SUN 2026-07-07 11-15-38\022-P1-C1-luoy-2-141a-IC-80.D
Sample Name: luoy-2-141a-IC-80

Signal 1: DAD1 A, Sig=254,4 Ref=360,100

Peak #	RetTime [min]	Type	Width [min]	Area [mAU*s]	Height [mAU]	Area %
1	13.982	MM	0.6239	470.64517	12.57199	1.7771
2	16.620	MM	0.8168	2.60137e4	530.79657	98.2229
Totals :				2.64844e4	543.36856	

Signal 2: DAD1 B, Sig=210,4 Ref=360,100

Signal 3: DAD1 D, Sig=230,4 Ref=360,100

===

*** End of Report ***

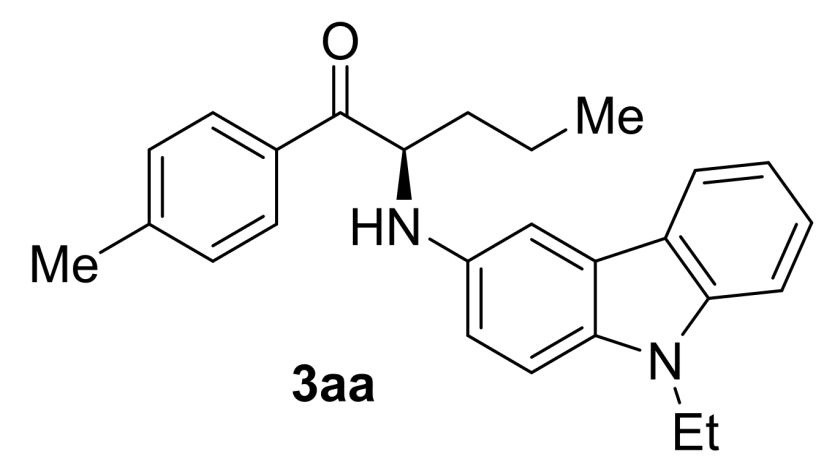

3aa

Data File C:\Users\P...1\Data\SUN\SUN 2026-06-21 15-15-25\007-P1-C4-luoy-2-120b-8-IBN-90.D
Sample Name: luoy-2-120b-8-IBN-90

===

```
Acq. Operator   : SYSTEM                        Seq. Line :    7
Sample Operator : SYSTEM
Acq. Instrument : HPLC                           Location :    P1-C-04
Injection Date  : 21/6/2026 4:39:10 pm                Inj :    1
                                               Inj Volume : 2.000 µl
Different Inj Volume from Sample Entry!  Actual Inj Volume : 20.000 µl
Acq. Method     : C:\Users\Public\Documents\ChemStation\1\Data\SUN\SUN 2026-06-21 15-15-25\IBN3-
                  10-20.M
Last changed    : 21/6/2026 4:17:49 pm by SYSTEM
Analysis Method : C:\Users\Public\Documents\ChemStation\1\Data\SUN\SUN 2026-06-21 15-15-25\IBN3-
                  10-20.M (Sequence Method)
Last changed    : 7/7/2026 7:44:42 pm by SYSTEM
                  (modified after loading)
Additional Info : Peak(s) manually integrated
```

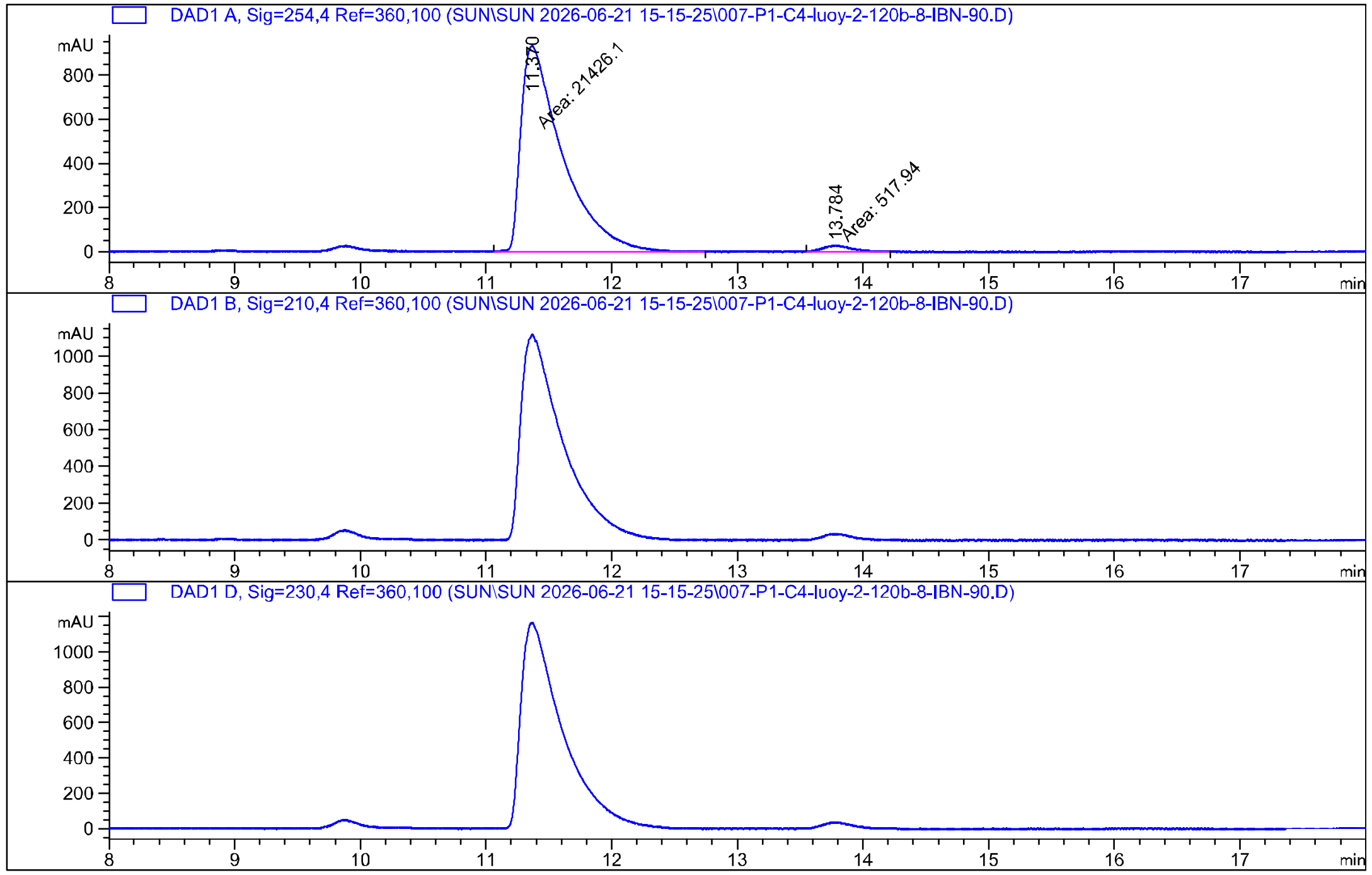

===
Area Percent Report
===

```
Sorted By             :      Signal
Multiplier            :      1.0000
Dilution              :      1.0000
Use Multiplier & Dilution Factor with ISTDs
```


Data File C:\Users\P...1\Data\SUN\SUN 2026-06-21 15-15-25\007-P1-C4-luoy-2-120b-8-IBN-90.D
Sample Name: luoy-2-120b-8-IBN-90

Signal 1: DAD1 A, Sig=254,4 Ref=360,100

Peak #	RetTime [min]	Type	Width [min]	Area [mAU*s]	Height [mAU]	Area %
1	11.370	MM	0.3821	2.14261e4	934.66693	97.6397
2	13.784	MM	0.2939	517.93970	29.36808	2.3603

Totals : 2.19441e4 964.03501

Signal 2: DAD1 B, Sig=210,4 Ref=360,100

Signal 3: DAD1 D, Sig=230,4 Ref=360,100

===
*** End of Report ***

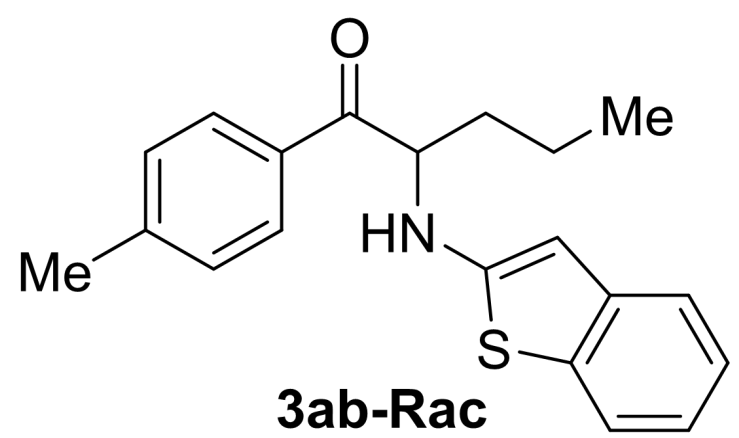

3ab-Rac

Data File C:\Users\P...on\1\Data\SUN\SUN 2026-06-21 15-15-25\006-P1-C2-luoy-2-122b-IB-90.D
Sample Name: luoy-2-122b-IB-90

===

Acq. Operator : SYSTEM   Seq. Line : 6
Sample Operator : SYSTEM
Acq. Instrument : HPLC   Location : P1-C-02
Injection Date : 21/6/2026 4:17:34 pm   Inj : 1
Inj Volume : 2.000 µl
Different Inj Volume from Sample Entry! Actual Inj Volume : 20.000 µl
Acq. Method : C:\Users\Public\Documents\ChemStation\1\Data\SUN\SUN 2026-06-21 15-15-25\IBN3-10-20.M
Last changed : 21/6/2026 4:17:49 pm by SYSTEM
(modified after loading)
Analysis Method : C:\Users\Public\Documents\ChemStation\1\Data\SUN\SUN 2026-06-21 15-15-25\IBN3-10-20.M (Sequence Method)
Last changed : 7/7/2026 7:42:27 pm by SYSTEM
(modified after loading)
Additional Info : Peak(s) manually integrated

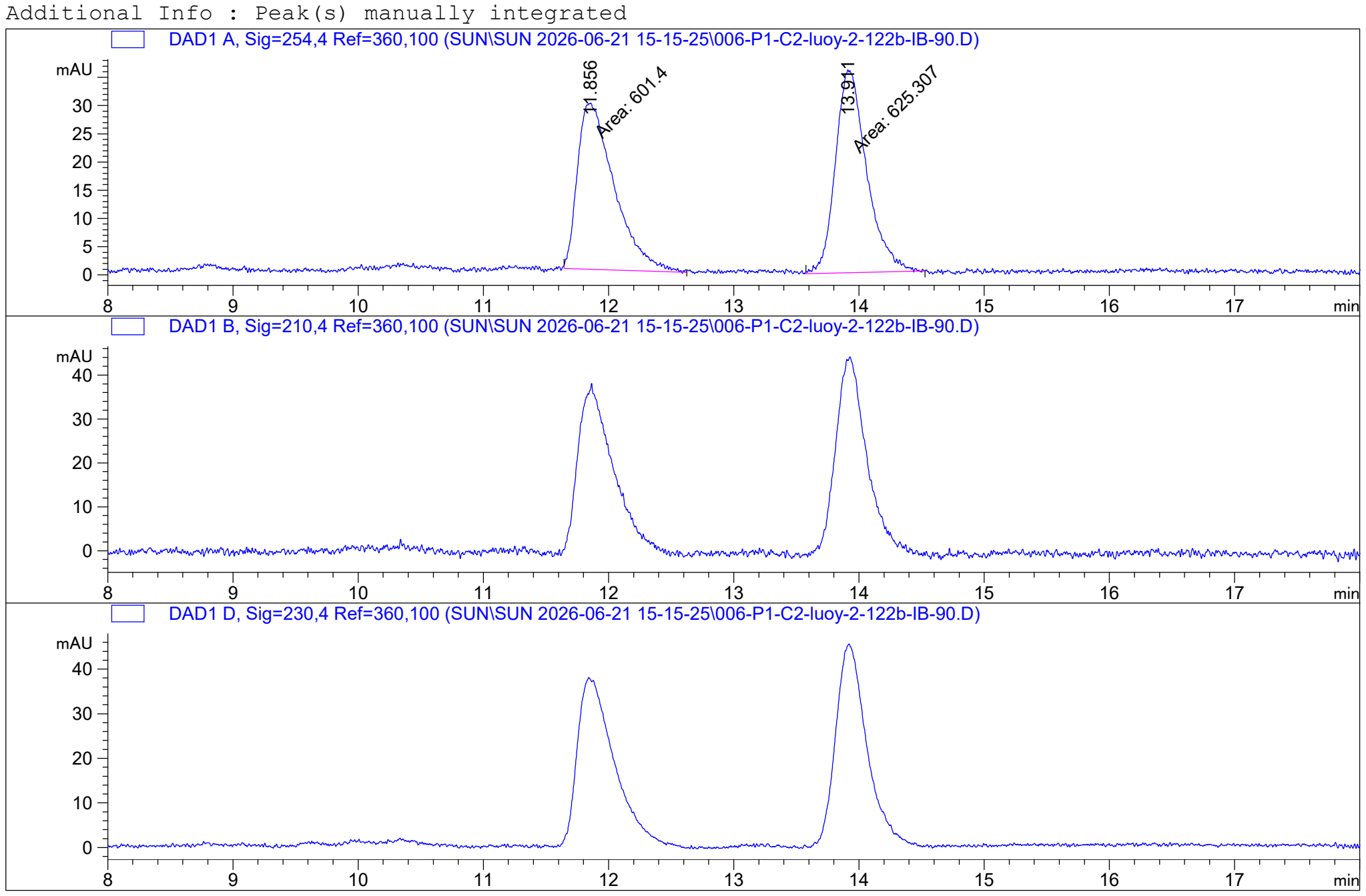

===

Area Percent Report

===

Sorted By : Signal
Multiplier : 1.0000
Dilution : 1.0000
Use Multiplier & Dilution Factor with ISTDs

Data File C:\Users\P...on\1\Data\SUN\SUN 2026-06-21 15-15-25\006-P1-C2-luoy-2-122b-IB-90.D
Sample Name: luoy-2-122b-IB-90

Signal 1: DAD1 A, Sig=254,4 Ref=360,100

Peak #	RetTime [min]	Type	Width [min]	Area [mAU*s]	Height [mAU]	Area %
1	11.856	MM	0.3402	601.39966	29.46213	49.0255
2	13.911	MM	0.2896	625.30707	35.98746	50.9745
Totals :				1226.70673	65.44959	

Signal 2: DAD1 B, Sig=210,4 Ref=360,100

Signal 3: DAD1 D, Sig=230,4 Ref=360,100

===

*** End of Report ***

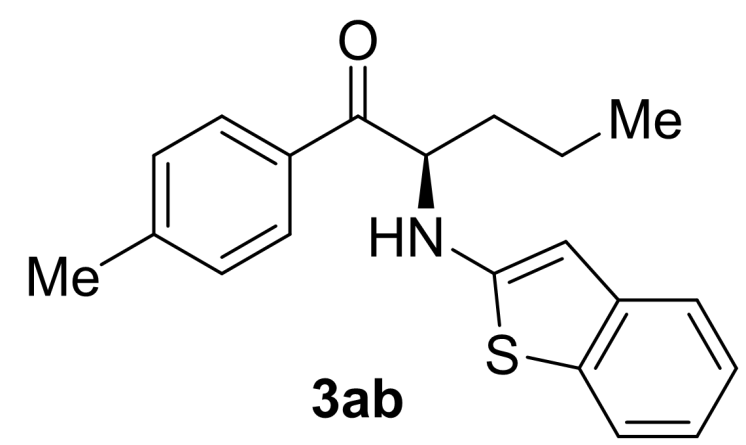

3ab

Data File C:\Users\P...on\1\Data\SUN\SUN 2026-06-21 15-15-25\009-P1-C1-luoy-2-122a-IC-95.D
Sample Name: luoy-2-122a-IC-95

===

Acq. Operator : SYSTEM Seq. Line : 9
Sample Operator : SYSTEM
Acq. Instrument : HPLC Location : P1-C-01
Injection Date : 21/6/2026 5:12:10 pm Inj : 1
Inj Volume : 2.000 µl
Different Inj Volume from Sample Entry! Actual Inj Volume : 10.000 µl
Acq. Method : C:\Users\Public\Documents\ChemStation\1\Data\SUN\SUN 2026-06-21 15-15-25\IC3-05-20.M
Last changed : 21/6/2026 5:21:19 pm by SYSTEM
(modified after loading)
Analysis Method : C:\Users\Public\Documents\ChemStation\1\Data\SUN\SUN 2026-06-21 15-15-25\IC3-05-20.M (Sequence Method)
Last changed : 7/7/2026 7:35:51 pm by SYSTEM
(modified after loading)
Additional Info : Peak(s) manually integrated

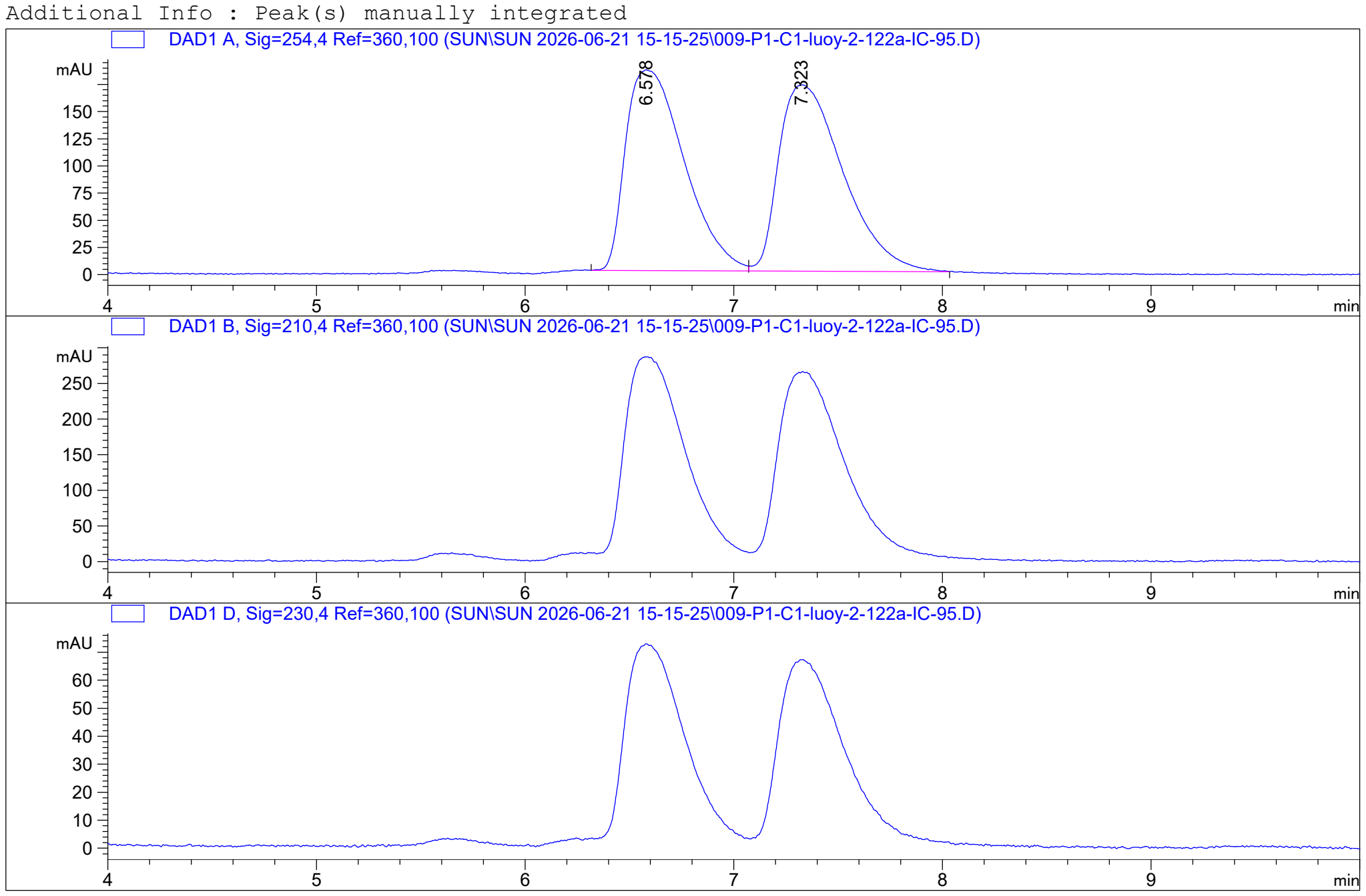

===

Area Percent Report

===

Sorted By : Signal
Multiplier : 1.0000
Dilution : 1.0000
Use Multiplier & Dilution Factor with ISTDs

Data File C:\Users\P...on\1\Data\SUN\SUN 2026-06-21 15-15-25\009-P1-C1-luoy-2-122a-IC-95.D
Sample Name: luoy-2-122a-IC-95

Signal 1: DAD1 A, Sig=254,4 Ref=360,100

Peak #	RetTime [min]	Type	Width [min]	Area [mAU*s]	Height [mAU]	Area %
1	6.578	VV R	0.2310	3612.33838	184.84456	49.5754
2	7.323	VV R	0.2539	3674.21460	171.54654	50.4246
Totals :				7286.55298	356.39110	

Signal 2: DAD1 B, Sig=210,4 Ref=360,100

Signal 3: DAD1 D, Sig=230,4 Ref=360,100

===

*** End of Report ***

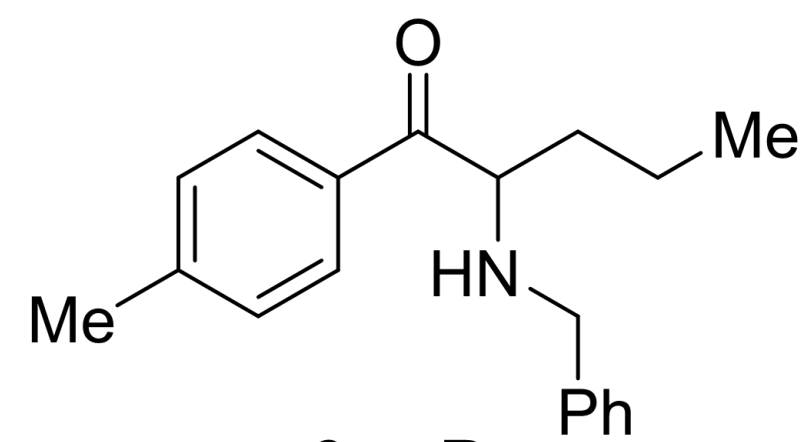

3ac-Rac

===

```
Acq. Operator   : SYSTEM                        Seq. Line :   4
Sample Operator : SYSTEM
Acq. Instrument : HPLC                           Location :   P1-C-04
Injection Date  : 5/7/2026 6:56:23 pm                 Inj :   1
                                               Inj Volume : 2.000 µl
Different Inj Volume from Sample Entry!  Actual Inj Volume : 10.000 µl
Acq. Method     : C:\Users\Public\Documents\ChemStation\1\Data\SUN\SUN 2026-07-05 18-08-51\IC3-
                  05-20.M
Last changed    : 18/8/2022 3:58:26 pm by SYSTEM
Analysis Method : C:\Users\Public\Documents\ChemStation\1\Data\SUN\SUN 2026-07-05 18-08-51\IC3-
                  05-20.M (Sequence Method)
Last changed    : 7/7/2026 7:37:57 pm by SYSTEM
                  (modified after loading)
Additional Info : Peak(s) manually integrated
```

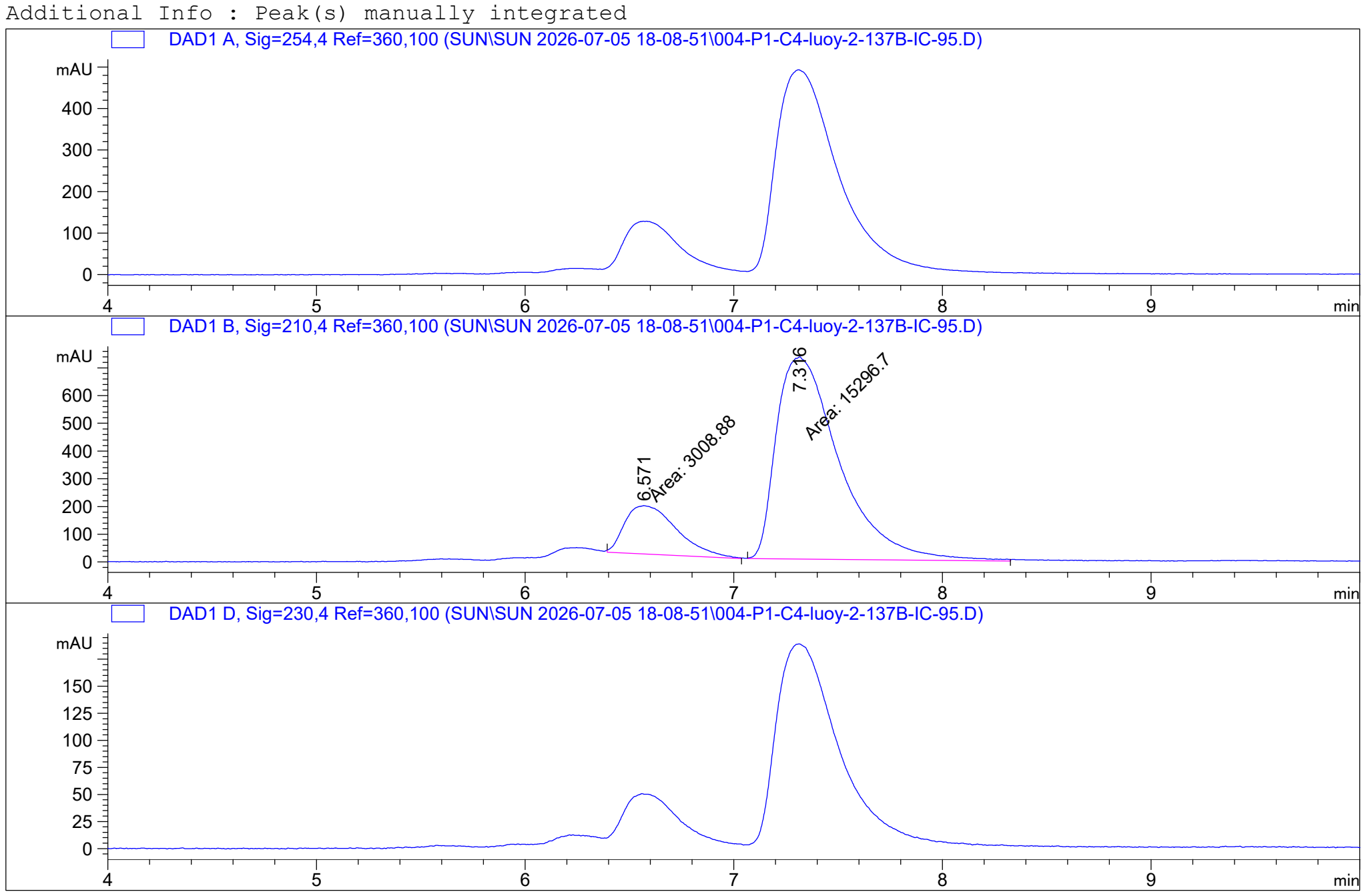

===

Area Percent Report

===

```
Sorted By             :      Signal
Multiplier            :      1.0000
Dilution              :      1.0000
Use Multiplier & Dilution Factor with ISTDs
```


Signal 1: DAD1 A, Sig=254,4 Ref=360,100

Signal 2: DAD1 B, Sig=210,4 Ref=360,100

Peak #	RetTime [min]	Type	Width [min]	Area [mAU*s]	Height [mAU]	Area %
1	6.571	MM	0.2887	3008.87939	173.71938	16.4369
2	7.316	MM	0.3496	1.52967e4	729.21179	83.5631

Totals : 1.83056e4   902.93117

Signal 3: DAD1 D, Sig=230,4 Ref=360,100

===

*** End of Report ***

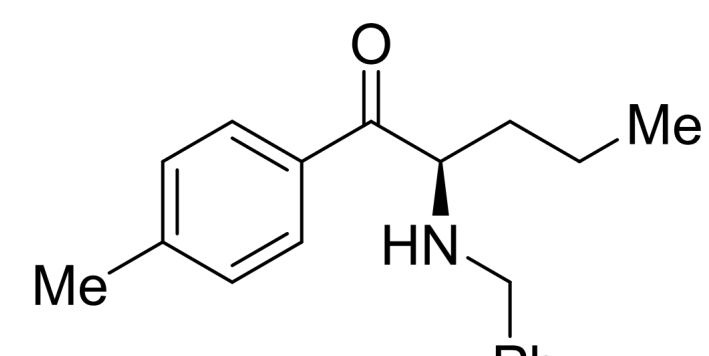

3ac

Data File C:\CHEM32\1\DATA\COLUMNWASHING 2022-03-09 10-24-17\LZY-09-46-E-RAC.D
Sample Name:

===

```
Acq. Operator   :                              Seq. Line :  24
Acq. Instrument : Instrument 1                  Location : Vial 23
Injection Date  : 3/9/2022 6:10:46 PM                Inj :   1
                                              Inj Volume : 5.000 µl
Different Inj Volume from Sequence !     Actual Inj Volume : 8.000 µl
Acq. Method     : C:\CHEM32\1\DATA\COLUMNWASHING 2022-03-09 10-24-17\OD-03-30.M
Last changed    : 1/31/2015 11:39:09 AM
Analysis Method : C:\CHEM32\1\METHODS\OD-03-60-0.6.M
Last changed    : 3/11/2022 12:15:55 PM
                  (modified after loading)
Additional Info : Peak(s) manually integrated
```

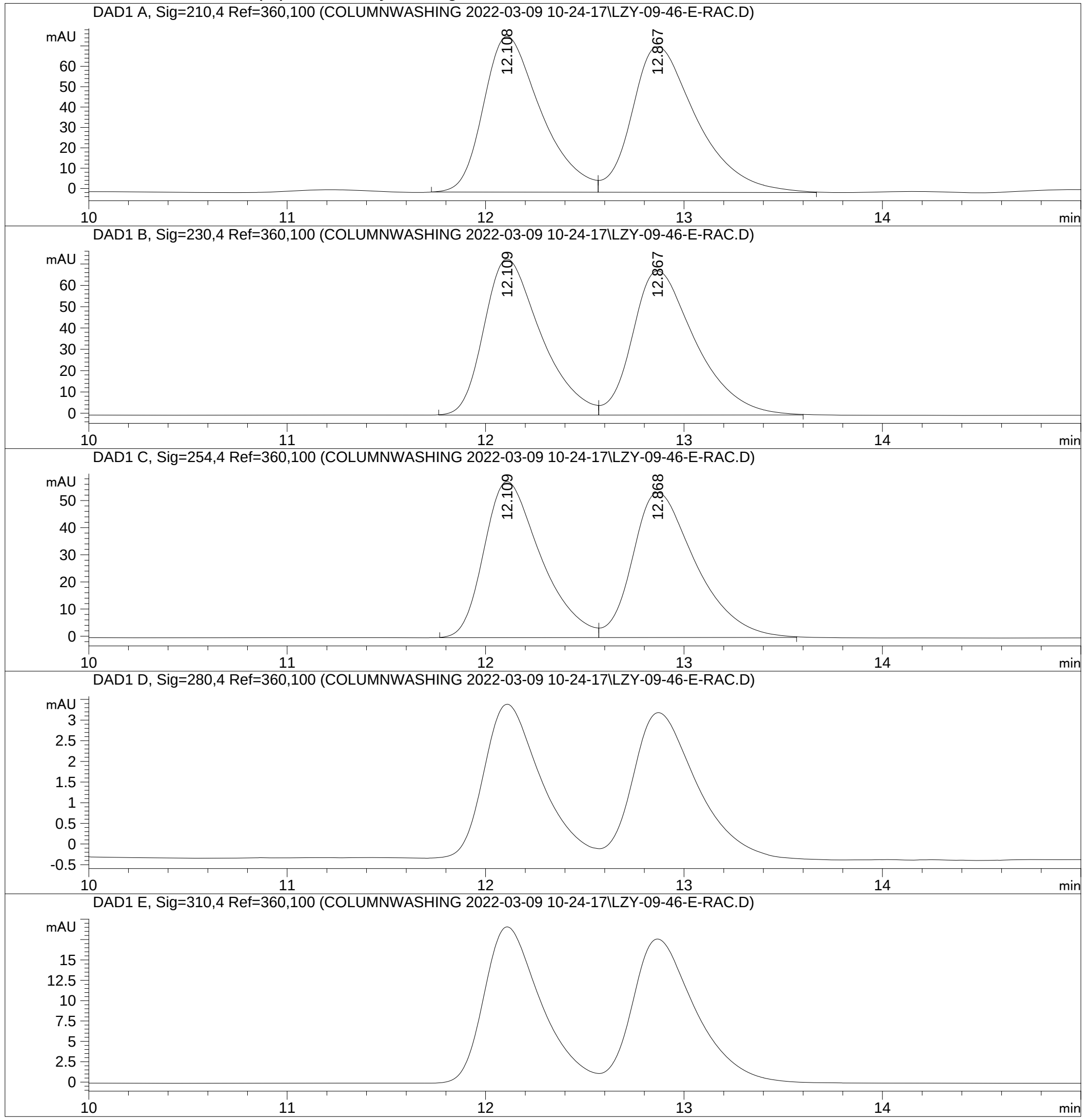

Data File C:\CHEM32\1\DATA\COLUMNWASHING 2022-03-09 10-24-17\LZY-09-46-E-RAC.D
Sample Name:

===

Area Percent Report

===

```
Sorted By             :      Signal
Multiplier            :      1.0000
Dilution              :      1.0000
Use Multiplier & Dilution Factor with ISTDs
```

Signal 1: DAD1 A, Sig=210,4 Ref=360,100

Peak #	RetTime [min]	Type	Width [min]	Area [mAU*s]	Height [mAU]	Area %
1	12.108	BV	0.3056	1544.89001	76.49505	48.6466
2	12.867	VB	0.3416	1630.85034	71.62533	51.3534
Totals :				3175.74036	148.12038	

Signal 2: DAD1 B, Sig=230,4 Ref=360,100

Peak #	RetTime [min]	Type	Width [min]	Area [mAU*s]	Height [mAU]	Area %
1	12.109	BV	0.3021	1456.22266	73.19842	49.3765
2	12.867	VB	0.3314	1493.00037	67.67896	50.6235
Totals :				2949.22302	140.87737	

Signal 3: DAD1 C, Sig=254,4 Ref=360,100

Peak #	RetTime [min]	Type	Width [min]	Area [mAU*s]	Height [mAU]	Area %
1	12.109	BV	0.3021	1144.50073	57.53035	49.1981
2	12.868	VB	0.3337	1181.81055	53.50034	50.8019
Totals :				2326.31128	111.03069	

Signal 4: DAD1 D, Sig=280,4 Ref=360,100

Signal 5: DAD1 E, Sig=310,4 Ref=360,100

===

*** End of Report ***

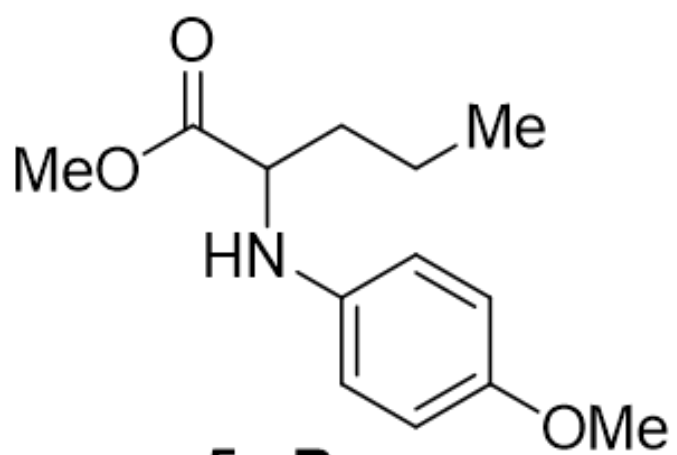

5a-Rac

```
=====================================================================
Acq. Operator   :                              Seq. Line :  25
Acq. Instrument : Instrument 1                  Location : Vial 24
Injection Date  : 3/9/2022 6:41:46 PM                Inj :   1
                                              Inj Volume : 5.000 µl
Different Inj Volume from Sequence !     Actual Inj Volume : 3.000 µl
Acq. Method     : C:\CHEM32\1\DATA\COLUMNWASHING 2022-03-09 10-24-17\OD-03-30.M
Last changed    : 1/31/2015 11:39:09 AM
Analysis Method : C:\CHEM32\1\METHODS\OD-03-60-0.6.M
Last changed    : 3/11/2022 12:15:55 PM
                  (modified after loading)
Additional Info : Peak(s) manually integrated
```

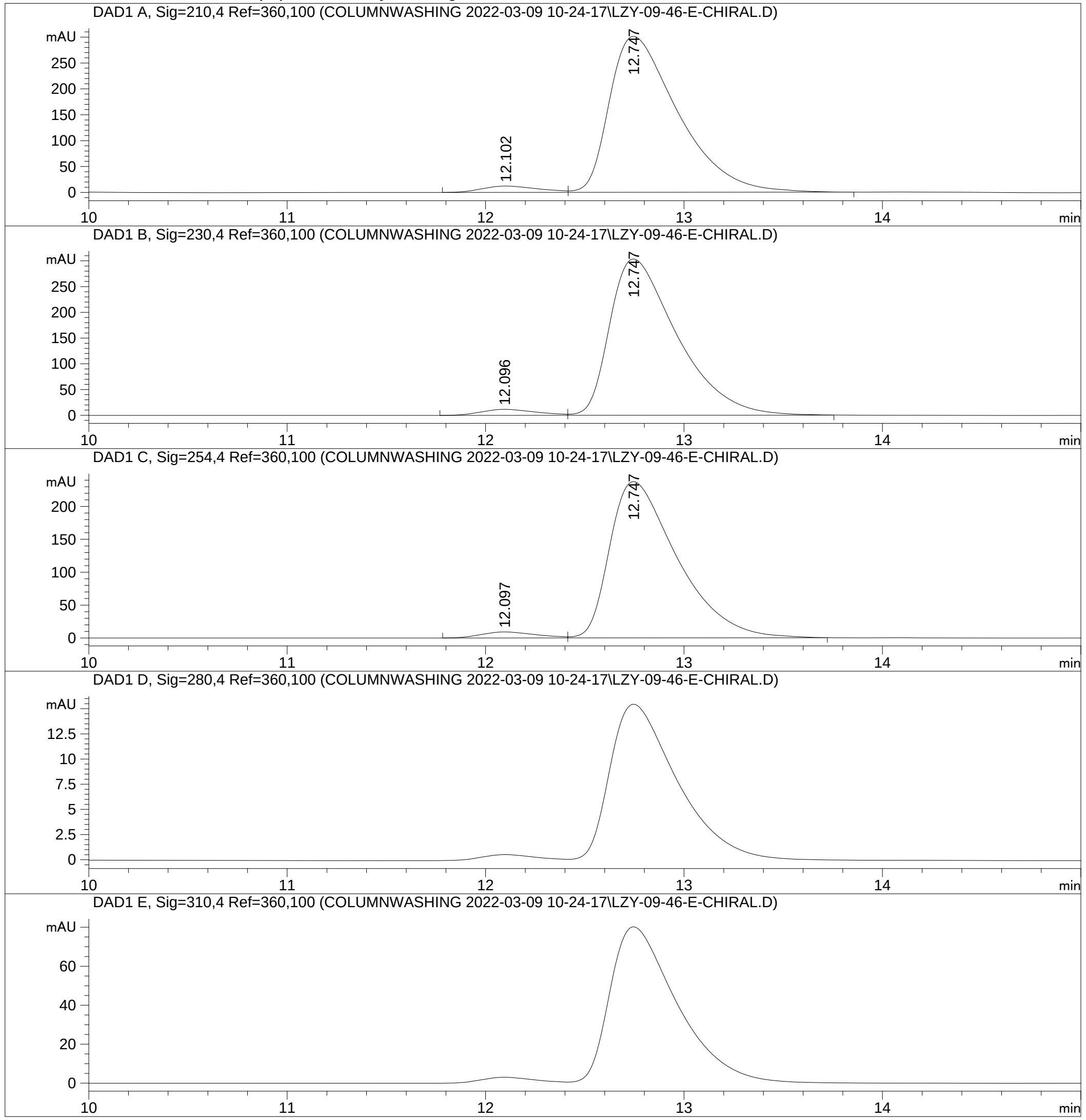

Data File C:\CHEM32\1\DATA\COLUMNWASHING 2022-03-09 10-24-17\LZY-09-46-E-CHIRAL.D
Sample Name:

Area Percent Report

Sorted By : Signal
Multiplier : 1.0000
Dilution : 1.0000
Use Multiplier & Dilution Factor with ISTDs

Signal 1: DAD1 A, Sig=210,4 Ref=360,100

Peak #	RetTime [min]	Type	Width [min]	Area [mAU*s]	Height [mAU]	Area %
1	12.102	BV	0.2945	237.12456	12.21174	3.1704
2	12.747	VB	0.3666	7242.24805	301.16357	96.8296

Totals : 7479.37260 313.37531

Signal 2: DAD1 B, Sig=230,4 Ref=360,100

Peak #	RetTime [min]	Type	Width [min]	Area [mAU*s]	Height [mAU]	Area %
1	12.096	BV	0.2911	221.13583	11.66804	2.9942
2	12.747	VB	0.3552	7164.43408	303.76596	97.0058

Totals : 7385.56992 315.43400

Signal 3: DAD1 C, Sig=254,4 Ref=360,100

Peak #	RetTime [min]	Type	Width [min]	Area [mAU*s]	Height [mAU]	Area %
1	12.097	BV	0.2908	173.49239	9.16894	2.9970
2	12.747	VB	0.3578	5615.46680	237.53308	97.0030

Totals : 5788.95918 246.70202

Signal 4: DAD1 D, Sig=280,4 Ref=360,100

Signal 5: DAD1 E, Sig=310,4 Ref=360,100

*** End of Report ***

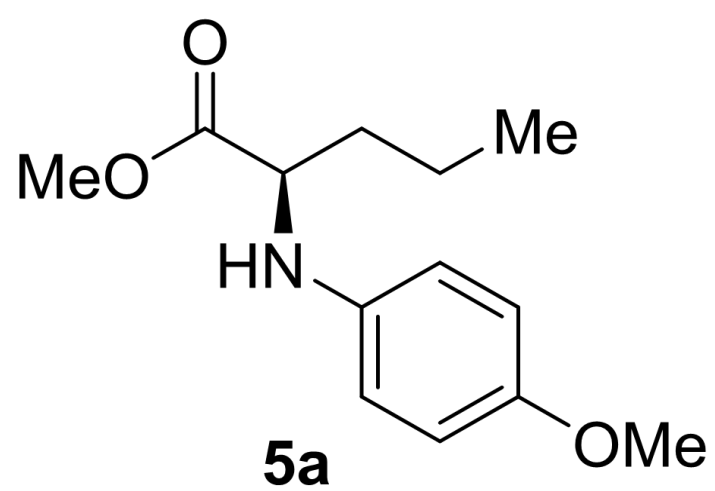

5a

Data File C:\CHEM32\1\DATA\COLUMNWASHING 2022-03-09 10-24-17\LZY-09-46-A-RAC.D
Sample Name:

===

Acq. Operator : Seq. Line : 22
Acq. Instrument : Instrument 1 Location : Vial 21
Injection Date : 3/9/2022 5:08:44 PM Inj : 1
Inj Volume : 5.000 µl
Different Inj Volume from Sequence ! Actual Inj Volume : 8.000 µl
Acq. Method : C:\CHEM32\1\DATA\COLUMNWASHING 2022-03-09 10-24-17\OD-03-30.M
Last changed : 1/31/2015 11:39:09 AM
Analysis Method : C:\CHEM32\1\METHODS\OD-03-60-0.6.M
Last changed : 3/11/2022 12:07:30 PM
(modified after loading)
Additional Info : Peak(s) manually integrated

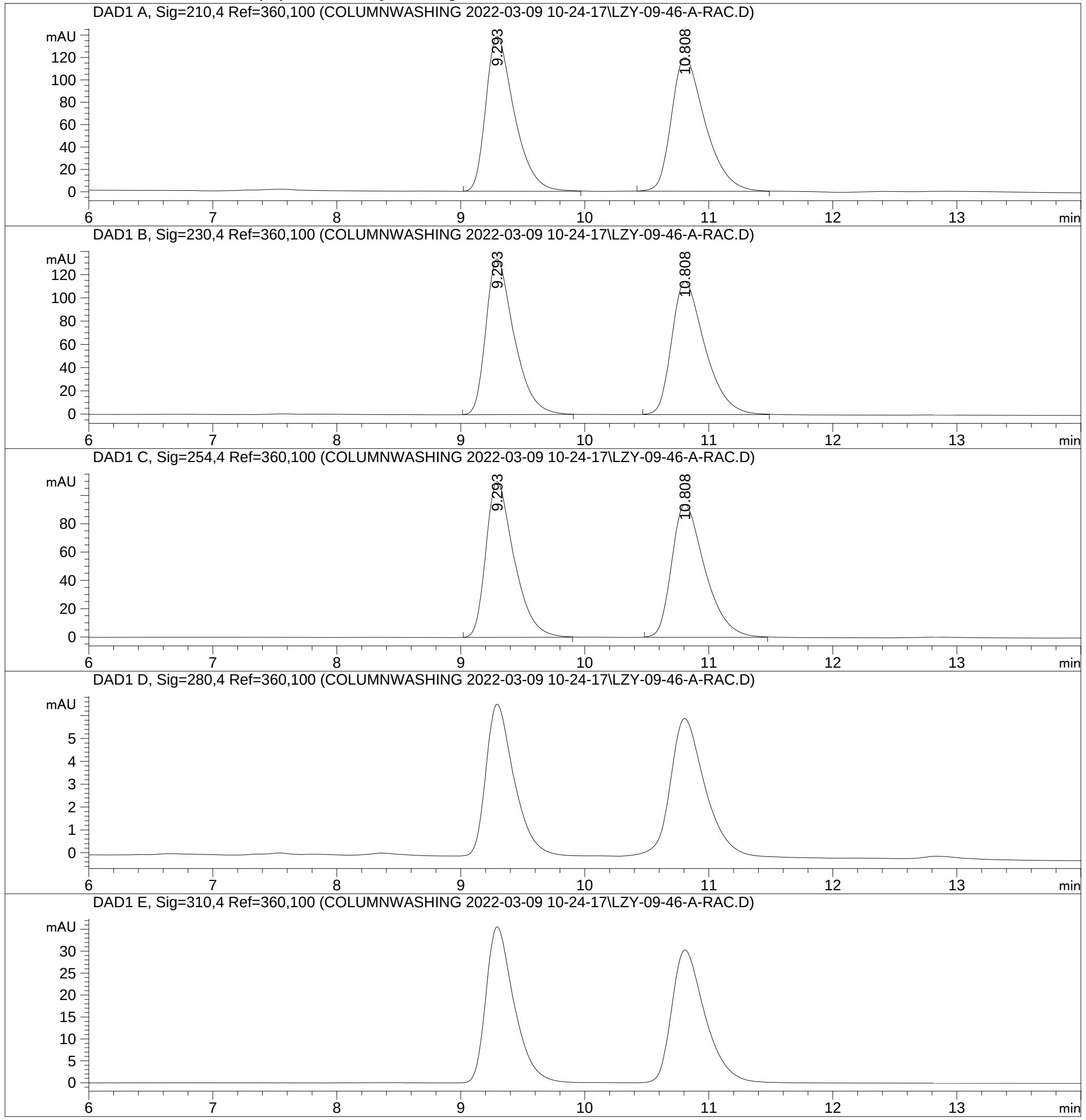

Data File C:\CHEM32\1\DATA\COLUMNWASHING 2022-03-09 10-24-17\LZY-09-46-A-RAC.D
Sample Name:

===

Area Percent Report

===

Sorted By : Signal
Multiplier : 1.0000
Dilution : 1.0000
Use Multiplier & Dilution Factor with ISTDs

Signal 1: DAD1 A, Sig=210,4 Ref=360,100

Peak #	RetTime [min]	Type	Width [min]	Area [mAU*s]	Height [mAU]	Area %
1	9.293	BB	0.2398	2204.13867	138.43321	49.3824
2	10.808	BB	0.2865	2259.27319	119.55634	50.6176

Totals : 4463.41187 257.98955

Signal 2: DAD1 B, Sig=230,4 Ref=360,100

Peak #	RetTime [min]	Type	Width [min]	Area [mAU*s]	Height [mAU]	Area %
1	9.293	BB	0.2377	2108.02075	133.90906	49.7529
2	10.808	BB	0.2833	2128.95630	114.31828	50.2471

Totals : 4236.97705 248.22733

Signal 3: DAD1 C, Sig=254,4 Ref=360,100

Peak #	RetTime [min]	Type	Width [min]	Area [mAU*s]	Height [mAU]	Area %
1	9.293	BB	0.2379	1734.25208	110.03436	49.7690
2	10.808	BB	0.2833	1750.34900	93.97971	50.2310

Totals : 3484.60107 204.01407

Signal 4: DAD1 D, Sig=280,4 Ref=360,100

Signal 5: DAD1 E, Sig=310,4 Ref=360,100

===

*** End of Report ***

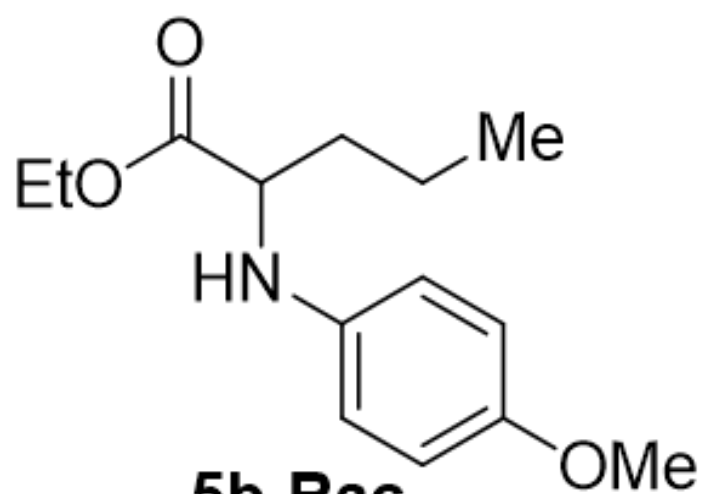

5b-Rac

Data File C:\CHEM32\1\DATA\COLUMNWASHING 2022-03-09 10-24-17\LZY-09-46-A-CHIRAL.D
Sample Name:

```
=====================================================================
Acq. Operator   :                           Seq. Line :  23
Acq. Instrument : Instrument 1               Location : Vial 22
Injection Date  : 3/9/2022 5:39:44 PM              Inj :   1
                                           Inj Volume : 5.000 µl
Different Inj Volume from Sequence !     Actual Inj Volume : 3.000 µl
Acq. Method     : C:\CHEM32\1\DATA\COLUMNWASHING 2022-03-09 10-24-17\OD-03-30.M
Last changed    : 1/31/2015 11:39:09 AM
Analysis Method : C:\CHEM32\1\METHODS\OD-03-60-0.6.M
Last changed    : 3/11/2022 12:15:18 PM
                  (modified after loading)
Additional Info : Peak(s) manually integrated
```

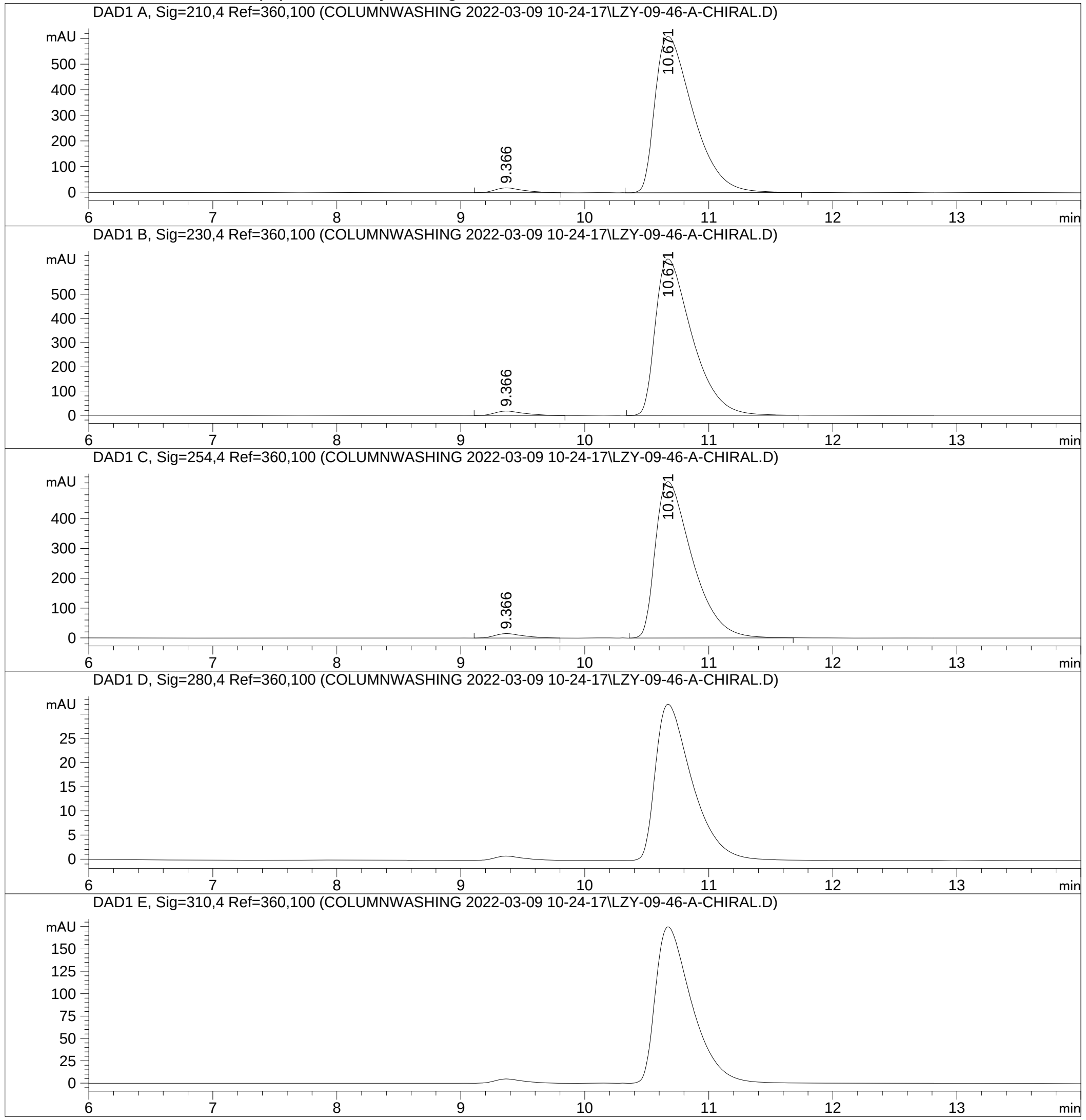

Data File C:\CHEM32\1\DATA\COLUMNWASHING 2022-03-09 10-24-17\LZY-09-46-A-CHIRAL.D
Sample Name:


```
=====================================================================
                         Area Percent Report
=====================================================================

Sorted By             :      Signal
Multiplier            :      1.0000
Dilution              :      1.0000
Use Multiplier & Dilution Factor with ISTDs
```

Signal 1: DAD1 A, Sig=210,4 Ref=360,100

Peak #	RetTime [min]	Type	Width [min]	Area [mAU*s]	Height [mAU]	Area %
1	9.366	BB	0.2359	288.21674	18.69175	2.1647
2	10.671	BB	0.3267	1.30261e4	611.16339	97.8353

Totals : 1.33144e4   629.85514

Signal 2: DAD1 B, Sig=230,4 Ref=360,100

Peak #	RetTime [min]	Type	Width [min]	Area [mAU*s]	Height [mAU]	Area %
1	9.366	BB	0.2356	283.77573	18.03401	2.0963
2	10.671	BB	0.3112	1.32533e4	646.49701	97.9037

Totals : 1.35371e4   664.53102

Signal 3: DAD1 C, Sig=254,4 Ref=360,100

Peak #	RetTime [min]	Type	Width [min]	Area [mAU*s]	Height [mAU]	Area %
1	9.366	BB	0.2335	230.35760	14.81012	2.0858
2	10.671	BB	0.3124	1.08135e4	524.70917	97.9142

Totals : 1.10439e4   539.51929

Signal 4: DAD1 D, Sig=280,4 Ref=360,100

Signal 5: DAD1 E, Sig=310,4 Ref=360,100

```
=====================================================================
                        *** End of Report ***
```

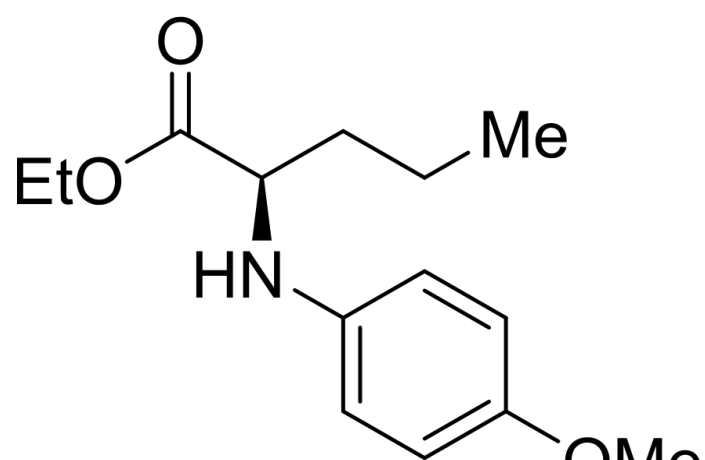

5b

Data File C:\CHEM32\1\DATA\COLUMNWASHING 2022-03-09 10-24-17\LZY-09-46-F-RAC.D
Sample Name:

===

Acq. Operator : Seq. Line : 26
Acq. Instrument : Instrument 1 Location : Vial 25
Injection Date : 3/9/2022 7:12:49 PM Inj : 1
Inj Volume : 5.000 µl
Different Inj Volume from Sequence ! Actual Inj Volume : 8.000 µl
Acq. Method : C:\CHEM32\1\DATA\COLUMNWASHING 2022-03-09 10-24-17\OD-03-20.M
Last changed : 12/21/2012 3:53:52 PM
Analysis Method : C:\CHEM32\1\METHODS\OD-03-60-0.6.M
Last changed : 3/11/2022 12:17:32 PM
(modified after loading)
Additional Info : Peak(s) manually integrated

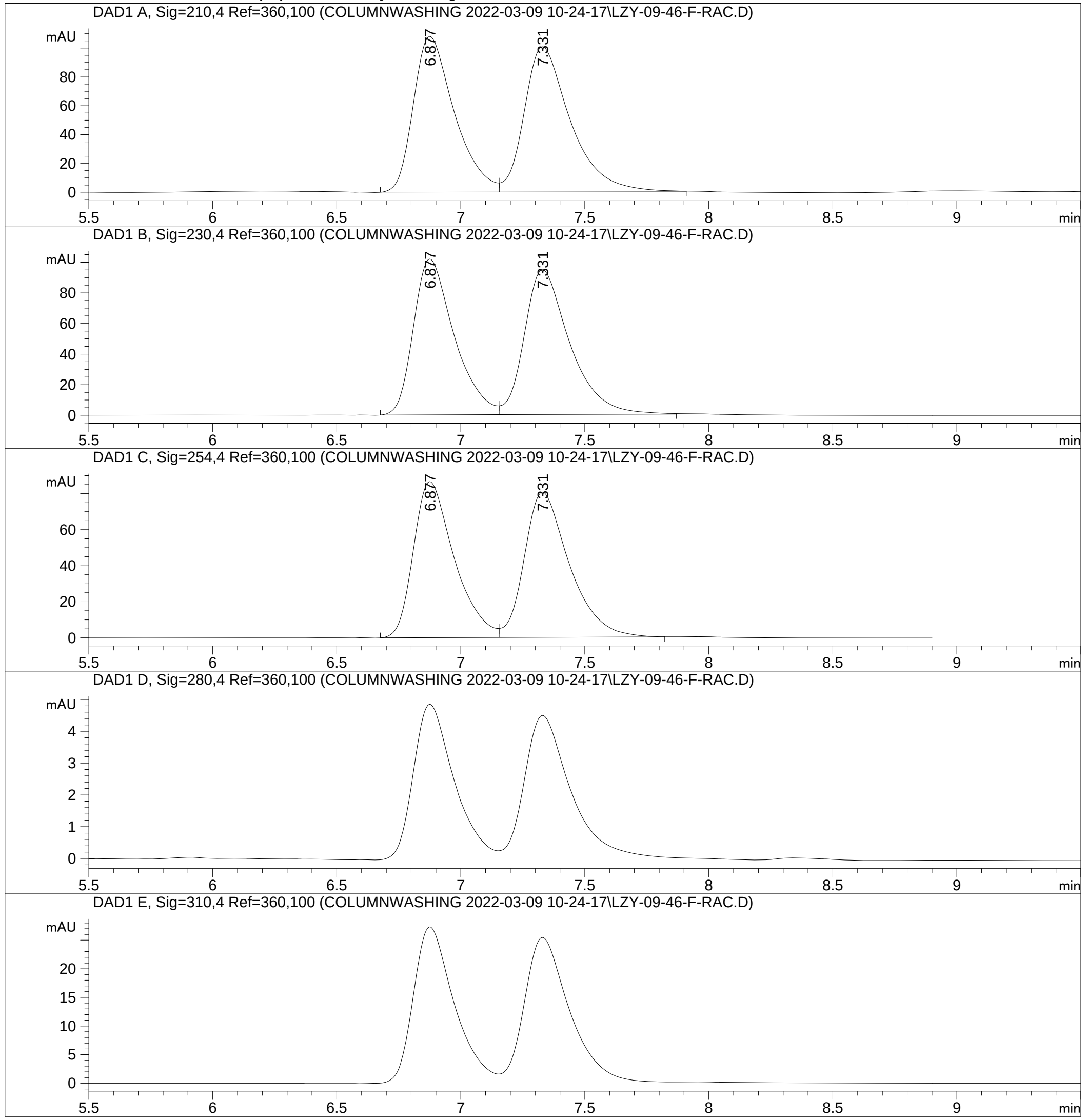

Data File C:\CHEM32\1\DATA\COLUMNWASHING 2022-03-09 10-24-17\LZY-09-46-F-RAC.D
Sample Name:

===

Area Percent Report

===

Sorted By : Signal
Multiplier : 1.0000
Dilution : 1.0000
Use Multiplier & Dilution Factor with ISTDs

Signal 1: DAD1 A, Sig=210,4 Ref=360,100

Peak #	RetTime [min]	Type	Width [min]	Area [mAU*s]	Height [mAU]	Area %
1	6.877	BV	0.1719	1239.53210	108.03699	48.5736
2	7.331	VB	0.1938	1312.32947	100.82731	51.4264

Totals : 2551.86157 208.86430

Signal 2: DAD1 B, Sig=230,4 Ref=360,100

Peak #	RetTime [min]	Type	Width [min]	Area [mAU*s]	Height [mAU]	Area %
1	6.877	BV	0.1707	1158.65027	101.86252	49.0469
2	7.331	VB	0.1903	1203.68091	94.69456	50.9531

Totals : 2362.33118 196.55708

Signal 3: DAD1 C, Sig=254,4 Ref=360,100

Peak #	RetTime [min]	Type	Width [min]	Area [mAU*s]	Height [mAU]	Area %
1	6.877	BV	0.1710	987.07556	86.61629	49.2710
2	7.331	VB	0.1891	1016.28424	80.61058	50.7290

Totals : 2003.35980 167.22688

Signal 4: DAD1 D, Sig=280,4 Ref=360,100

Signal 5: DAD1 E, Sig=310,4 Ref=360,100

===

*** End of Report ***

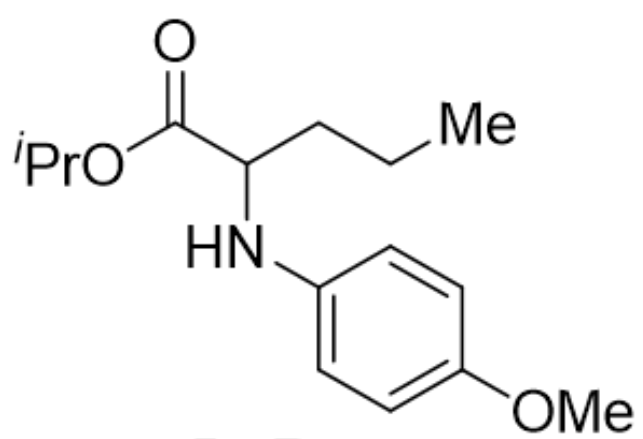

5c-Rac

```
=====================================================================
Acq. Operator   :                            Seq. Line :  27
Acq. Instrument : Instrument 1                Location : Vial 26
Injection Date  : 3/9/2022 7:33:53 PM              Inj :   1
                                            Inj Volume : 5.000 µl
Different Inj Volume from Sequence !     Actual Inj Volume : 3.000 µl
Acq. Method     : C:\CHEM32\1\DATA\COLUMNWASHING 2022-03-09 10-24-17\OD-03-30.M
Last changed    : 1/31/2015 11:39:09 AM
Analysis Method : C:\CHEM32\1\METHODS\OD-03-60-0.6.M
Last changed    : 3/11/2022 12:17:32 PM
                  (modified after loading)
Additional Info : Peak(s) manually integrated
```

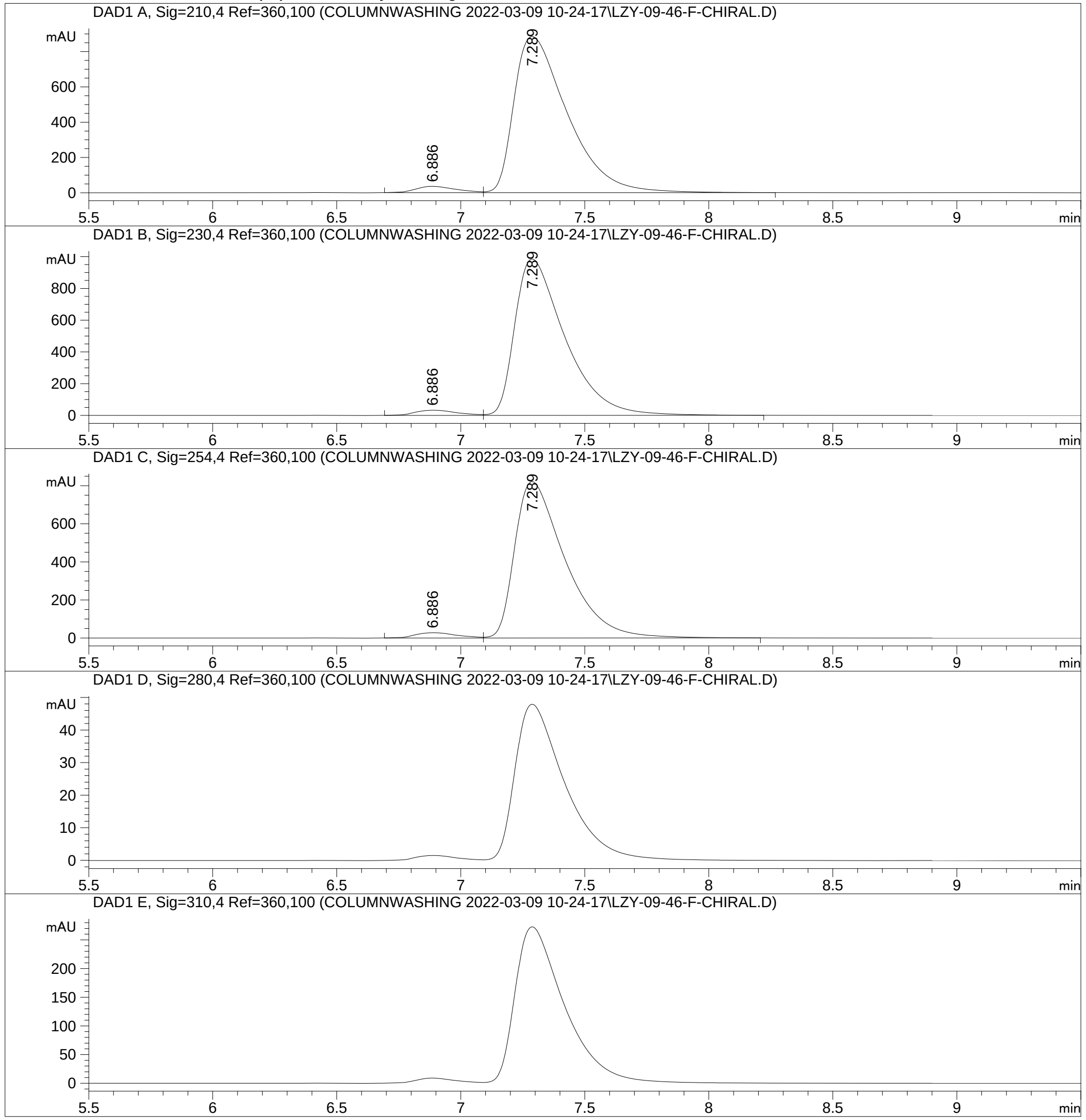

Data File C:\CHEM32\1\DATA\COLUMNWASHING 2022-03-09 10-24-17\LZY-09-46-F-CHIRAL.D
Sample Name:

```
=====================================================================
                         Area Percent Report
=====================================================================

Sorted By             :      Signal
Multiplier            :      1.0000
Dilution              :      1.0000
Use Multiplier & Dilution Factor with ISTDs


Signal 1: DAD1 A, Sig=210,4 Ref=360,100

Peak RetTime Type  Width     Area      Height     Area
  #   [min]        [min]   [mAU*s]     [mAU]        %
----|-------|----|-------|----------|----------|--------|
   1   6.886 BV    0.1675  404.56268   36.43761   2.9617
   2   7.289 VB    0.2266 1.32554e4   885.49792  97.0383

Totals :                  1.36600e4   921.93553


Signal 2: DAD1 B, Sig=230,4 Ref=360,100

Peak RetTime Type  Width     Area      Height     Area
  #   [min]        [min]   [mAU*s]     [mAU]        %
----|-------|----|-------|----------|----------|--------|
   1   6.886 BV    0.1667  372.60223   33.77703   2.6149
   2   7.289 VB    0.2123 1.38769e4   985.09552  97.3851

Totals :                  1.42495e4  1018.87255


Signal 3: DAD1 C, Sig=254,4 Ref=360,100

Peak RetTime Type  Width     Area      Height     Area
  #   [min]        [min]   [mAU*s]     [mAU]        %
----|-------|----|-------|----------|----------|--------|
   1   6.886 BV    0.1671  319.29178   28.85996   2.6703
   2   7.289 VB    0.2133 1.16378e4   821.51093  97.3297

Totals :                  1.19571e4   850.37089


Signal 4: DAD1 D, Sig=280,4 Ref=360,100


Signal 5: DAD1 E, Sig=310,4 Ref=360,100

=====================================================================
                          *** End of Report ***
```

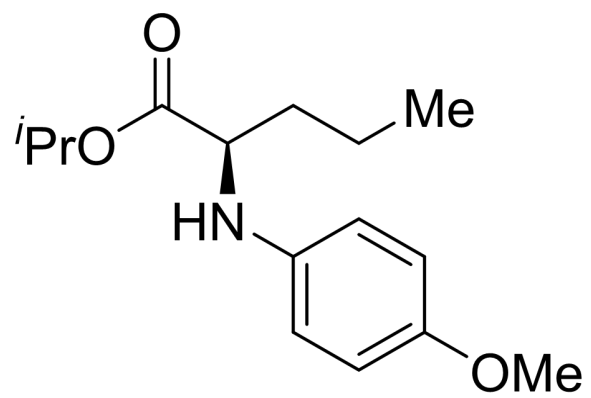

5c

Data File C:\CHEM32\1\DATA\SUN_12 2022-01-15 10-17-35\LZY-08-174-C-RAC-OD03.D
Sample Name:

```
=====================================================================
Acq. Operator   :                        Seq. Line :   4
Acq. Instrument : Instrument 1            Location : Vial 23
Injection Date  : 1/15/2022 11:02:27 AM        Inj :   1
                                        Inj Volume : 5.000 µl
Different Inj Volume from Sequence !   Actual Inj Volume : 7.000 µl
Acq. Method     : C:\CHEM32\1\DATA\SUN_12 2022-01-15 10-17-35\OD-03-30.M
Last changed    : 1/15/2022 11:02:49 AM
                  (modified after loading)
Analysis Method : C:\CHEM32\1\METHODS\OD-03-60-0.6.M
Last changed    : 3/8/2022 3:04:37 PM
                  (modified after loading)
Additional Info : Peak(s) manually integrated
```

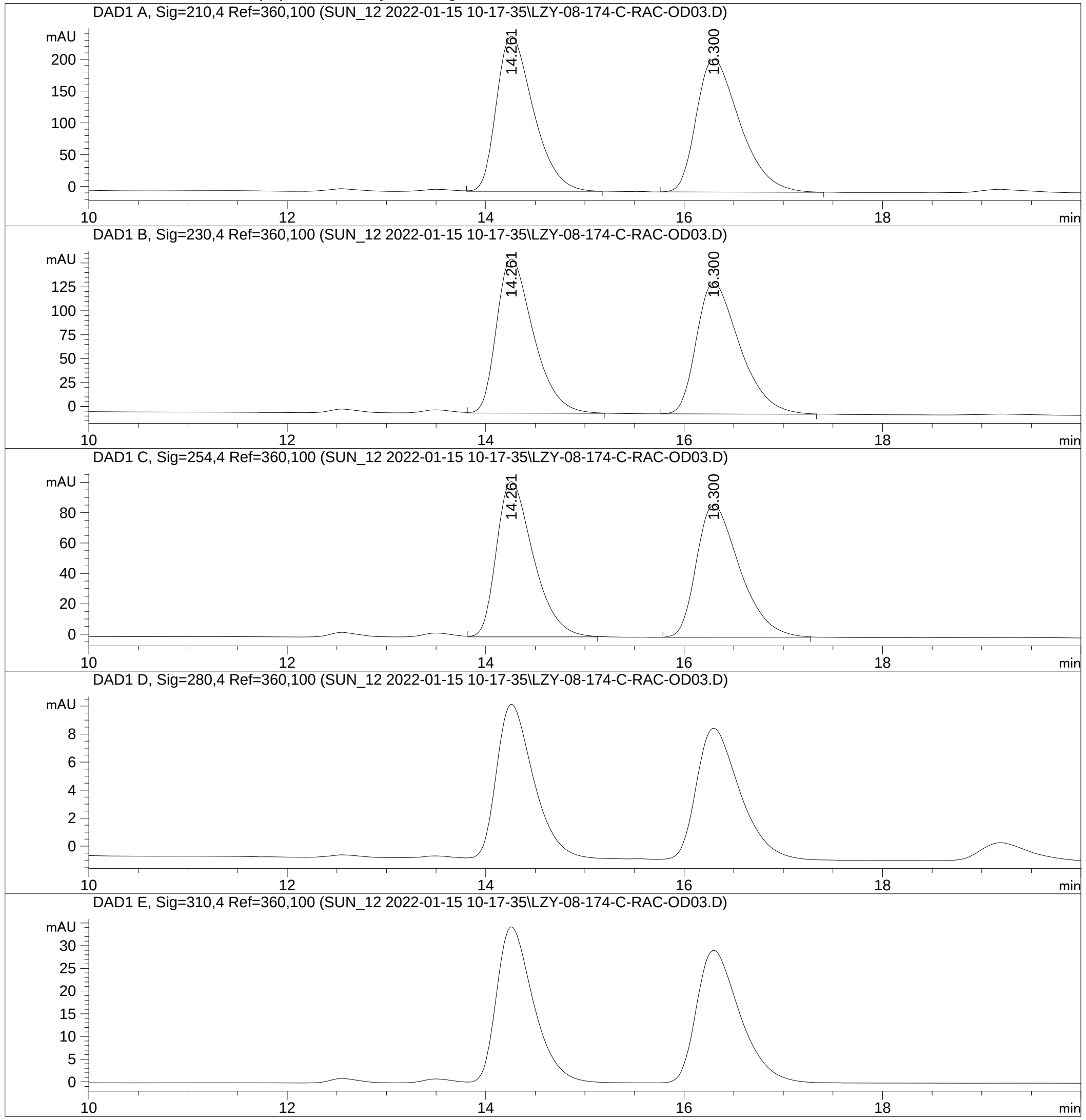

Data File C:\CHEM32\1\DATA\SUN_12 2022-01-15 10-17-35\LZY-08-174-C-RAC-OD03.D
Sample Name:

===
Area Percent Report
===

```
Sorted By             :      Signal
Multiplier            :      1.0000
Dilution              :      1.0000
Use Multiplier & Dilution Factor with ISTDs
```

Signal 1: DAD1 A, Sig=210,4 Ref=360,100

Peak #	RetTime [min]	Type	Width [min]	Area [mAU*s]	Height [mAU]	Area %
1	14.261	VB	0.3981	6306.70654	243.54063	49.7357
2	16.300	BB	0.4704	6373.72656	208.61557	50.2643

Totals : 1.26804e4 452.15620

Signal 2: DAD1 B, Sig=230,4 Ref=360,100

Peak #	RetTime [min]	Type	Width [min]	Area [mAU*s]	Height [mAU]	Area %
1	14.261	VB	0.3946	4153.25146	161.17517	49.9923
2	16.300	BB	0.4637	4154.53906	137.04636	50.0077

Totals : 8307.79053 298.22153

Signal 3: DAD1 C, Sig=254,4 Ref=360,100

Peak #	RetTime [min]	Type	Width [min]	Area [mAU*s]	Height [mAU]	Area %
1	14.261	VB	0.3946	2634.28931	102.22730	49.9688
2	16.300	BB	0.4656	2637.57520	87.03481	50.0312

Totals : 5271.86450 189.26211

Signal 4: DAD1 D, Sig=280,4 Ref=360,100

Signal 5: DAD1 E, Sig=310,4 Ref=360,100

===
*** End of Report ***

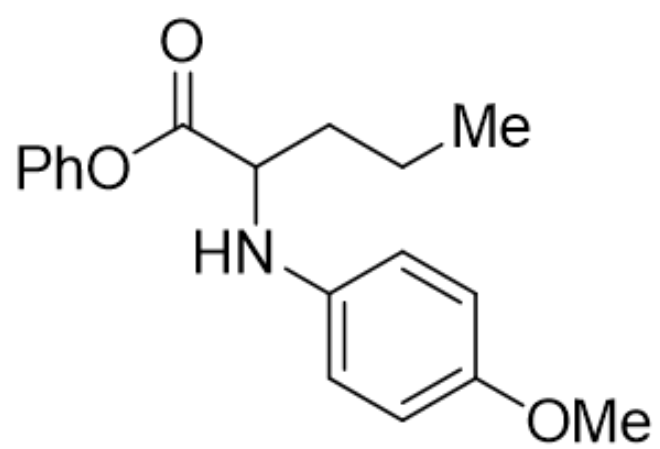

5d-Rac

Data File C:\CHEM32\1\DATA\SUN_12 2022-01-19 22-54-10\LZY-08-182-A.D
Sample Name:

===

Acq. Operator : Seq. Line : 2
Acq. Instrument : Instrument 1 Location : Vial 21
Injection Date : 1/19/2022 11:07:02 PM Inj : 1
Inj Volume : 5.000 µl
Acq. Method : C:\CHEM32\1\DATA\SUN_12 2022-01-19 22-54-10\OD-03-30.M
Last changed : 1/19/2022 11:06:10 PM
(modified after loading)
Analysis Method : C:\CHEM32\1\METHODS\OD-03-60-0.6.M
Last changed : 3/8/2022 3:04:37 PM
(modified after loading)
Additional Info : Peak(s) manually integrated

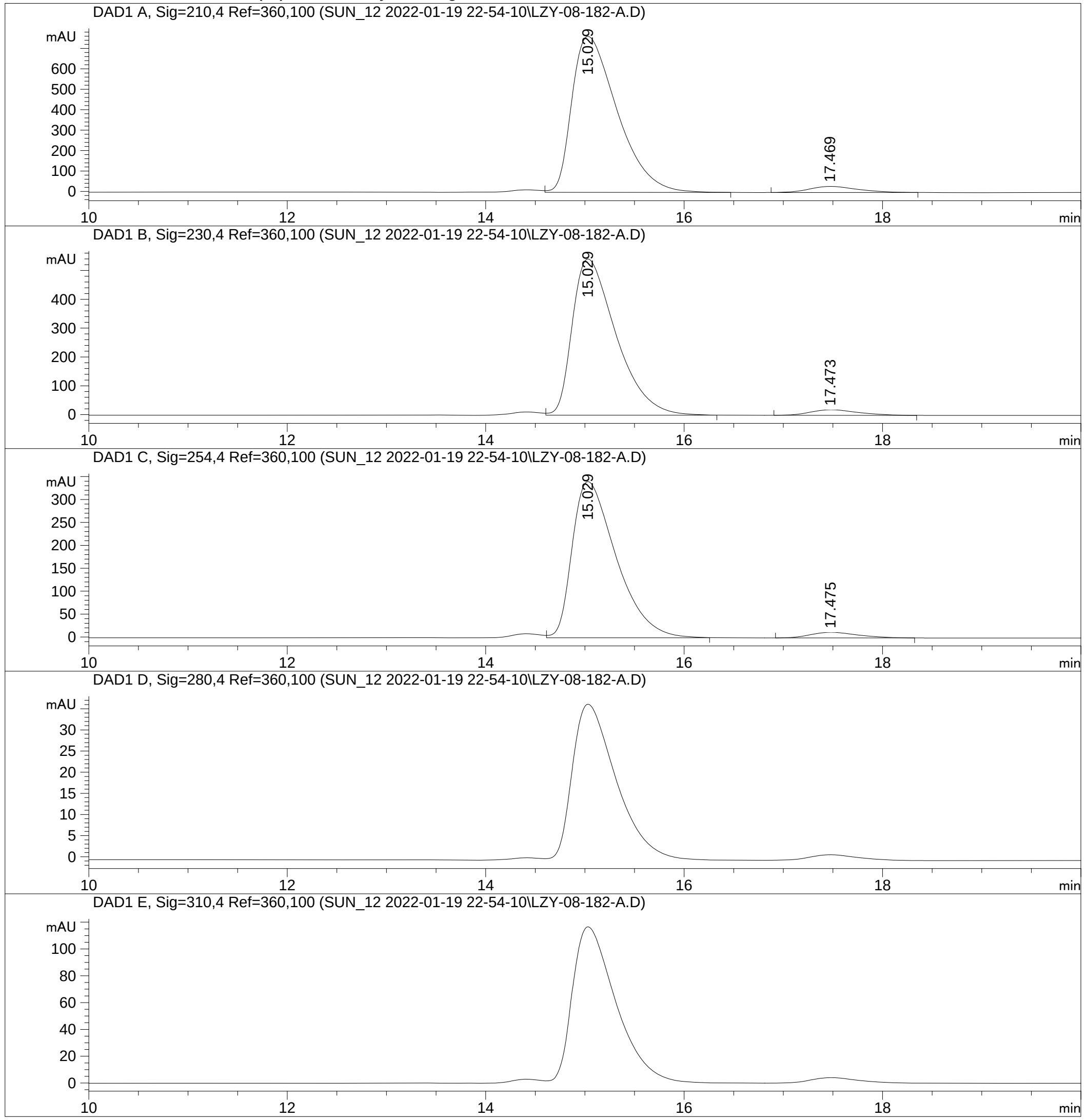

Data File C:\CHEM32\1\DATA\SUN_12 2022-01-19 22-54-10\LZY-08-182-A.D
Sample Name:

===
Area Percent Report
===

Sorted By : Signal
Multiplier : 1.0000
Dilution : 1.0000
Use Multiplier & Dilution Factor with ISTDs

Signal 1: DAD1 A, Sig=210,4 Ref=360,100

Peak #	RetTime [min]	Type	Width [min]	Area [mAU*s]	Height [mAU]	Area %
1	15.029	VB	0.4944	2.42386e4	763.55347	95.9983
2	17.469	BB	0.5037	1010.39374	29.79202	4.0017

Totals : 2.52490e4 793.34549

Signal 2: DAD1 B, Sig=230,4 Ref=360,100

Peak #	RetTime [min]	Type	Width [min]	Area [mAU*s]	Height [mAU]	Area %
1	15.029	VB	0.4689	1.65763e4	541.85181	96.2794
2	17.473	BB	0.5089	640.57288	19.21629	3.7206

Totals : 1.72168e4 561.06809

Signal 3: DAD1 C, Sig=254,4 Ref=360,100

Peak #	RetTime [min]	Type	Width [min]	Area [mAU*s]	Height [mAU]	Area %
1	15.029	VB	0.4705	1.04696e4	340.72995	96.2781
2	17.475	BB	0.5036	404.72852	12.24485	3.7219

Totals : 1.08744e4 352.97480

Signal 4: DAD1 D, Sig=280,4 Ref=360,100

Signal 5: DAD1 E, Sig=310,4 Ref=360,100

===
*** End of Report ***

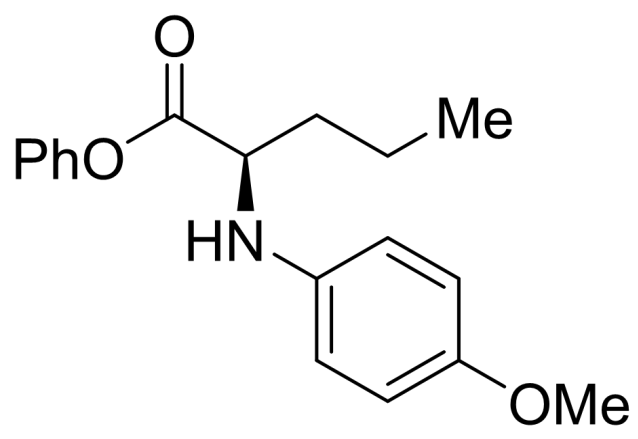

5d

===

Acq. Operator : Seq. Line : 17
Acq. Instrument : Instrument 1 Location : Vial 24
Injection Date : 1/15/2022 3:22:17 PM Inj : 1
Inj Volume : 3.000 µl
Different Inj Volume from Sequence ! Actual Inj Volume : 7.000 µl
Acq. Method : C:\CHEM32\1\DATA\SUN_12 2022-01-15 10-17-35\AS-05-30.M
Last changed : 1/15/2022 3:21:25 PM
(modified after loading)
Analysis Method : C:\CHEM32\1\METHODS\OD-03-60-0.6.M
Last changed : 3/8/2022 3:07:06 PM
(modified after loading)
Additional Info : Peak(s) manually integrated

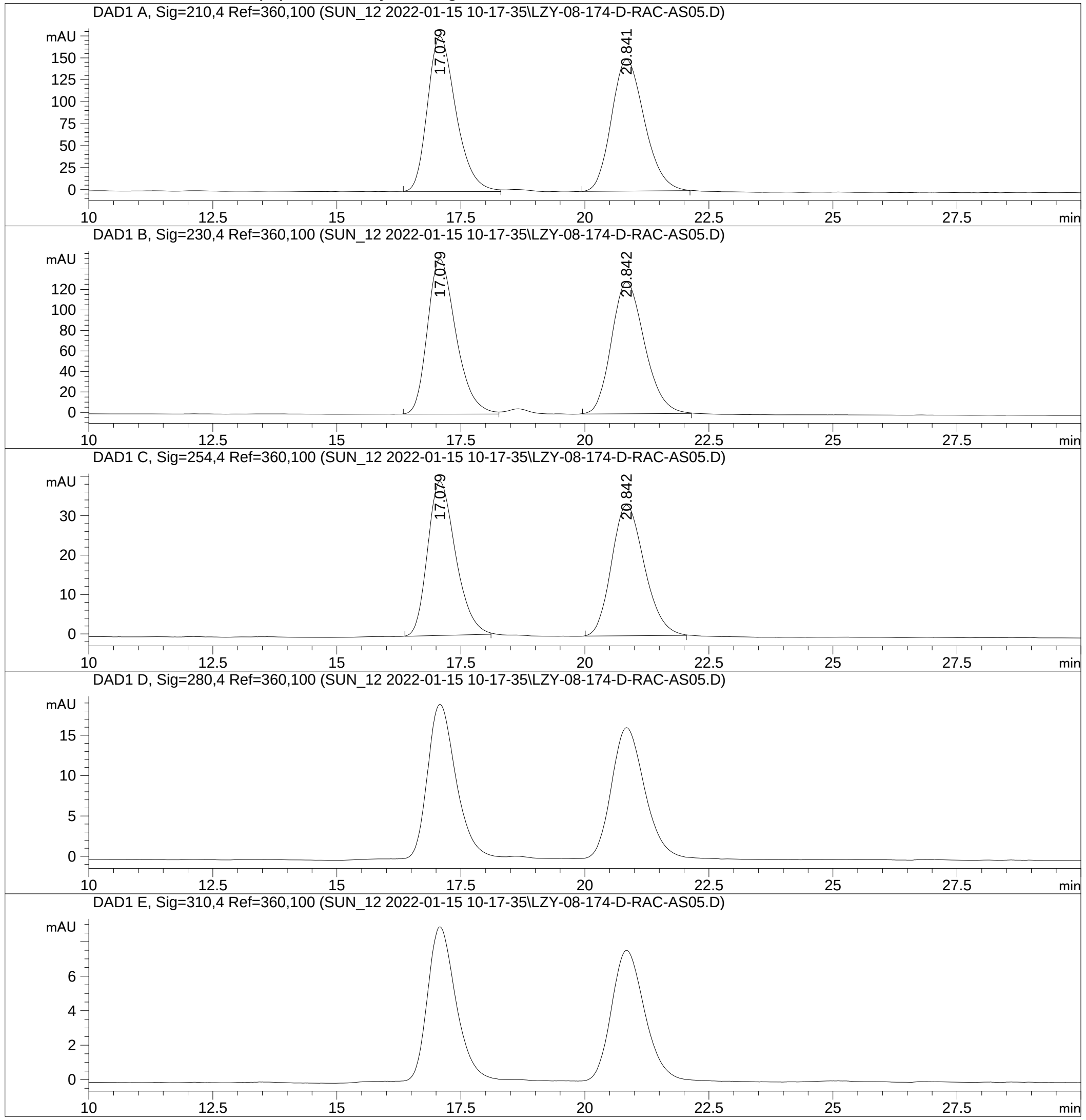

Data File C:\CHEM32\1\DATA\SUN_12 2022-01-15 10-17-35\LZY-08-174-D-RAC-AS05.D
Sample Name:

===

Area Percent Report

===

Sorted By : Signal
Multiplier : 1.0000
Dilution : 1.0000
Use Multiplier & Dilution Factor with ISTDs

Signal 1: DAD1 A, Sig=210,4 Ref=360,100

Peak #	RetTime [min]	Type	Width [min]	Area [mAU*s]	Height [mAU]	Area %
1	17.079	BB	0.6105	7033.29492	176.64159	50.4393
2	20.841	BB	0.7082	6910.78857	149.81155	49.5607

Totals : 1.39441e4 326.45314

Signal 2: DAD1 B, Sig=230,4 Ref=360,100

Peak #	RetTime [min]	Type	Width [min]	Area [mAU*s]	Height [mAU]	Area %
1	17.079	BB	0.6111	6034.91016	151.39333	50.3486
2	20.842	BB	0.7164	5951.34619	128.47522	49.6514

Totals : 1.19863e4 279.86855

Signal 3: DAD1 C, Sig=254,4 Ref=360,100

Peak #	RetTime [min]	Type	Width [min]	Area [mAU*s]	Height [mAU]	Area %
1	17.079	BB	0.6001	1526.86145	39.05182	50.0445
2	20.842	BB	0.7078	1524.14832	33.18859	49.9555

Totals : 3051.00977 72.24041

Signal 4: DAD1 D, Sig=280,4 Ref=360,100

Signal 5: DAD1 E, Sig=310,4 Ref=360,100

===

*** End of Report ***

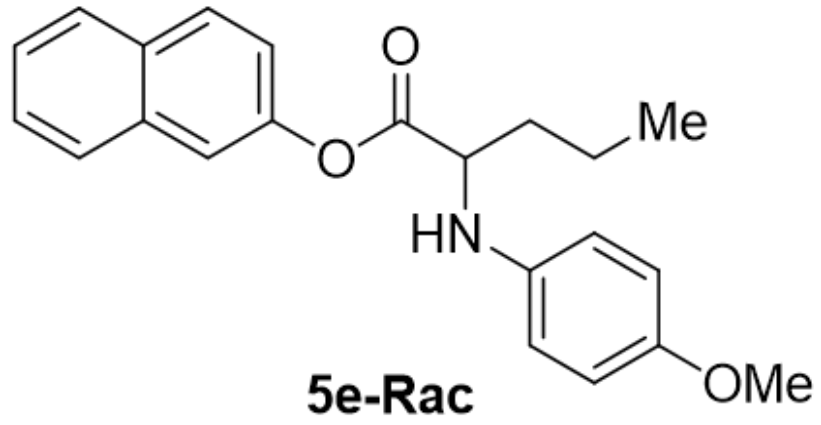

5e-Rac

Data File C:\CHEM32\1\DATA\SUN_12 2022-01-19 22-54-10\LZY-08-182-B.D
Sample Name:

===

Acq. Operator : Seq. Line : 5
Acq. Instrument : Instrument 1 Location : Vial 22
Injection Date : 1/20/2022 12:00:08 AM Inj : 1
Inj Volume : 3.000 µl
Different Inj Volume from Sequence ! Actual Inj Volume : 4.000 µl
Acq. Method : C:\CHEM32\1\DATA\SUN_12 2022-01-19 22-54-10\AS-05-30.M
Last changed : 1/19/2022 11:59:17 PM
(modified after loading)
Analysis Method : C:\CHEM32\1\METHODS\OD-03-60-0.6.M
Last changed : 3/8/2022 3:07:06 PM
(modified after loading)
Additional Info : Peak(s) manually integrated

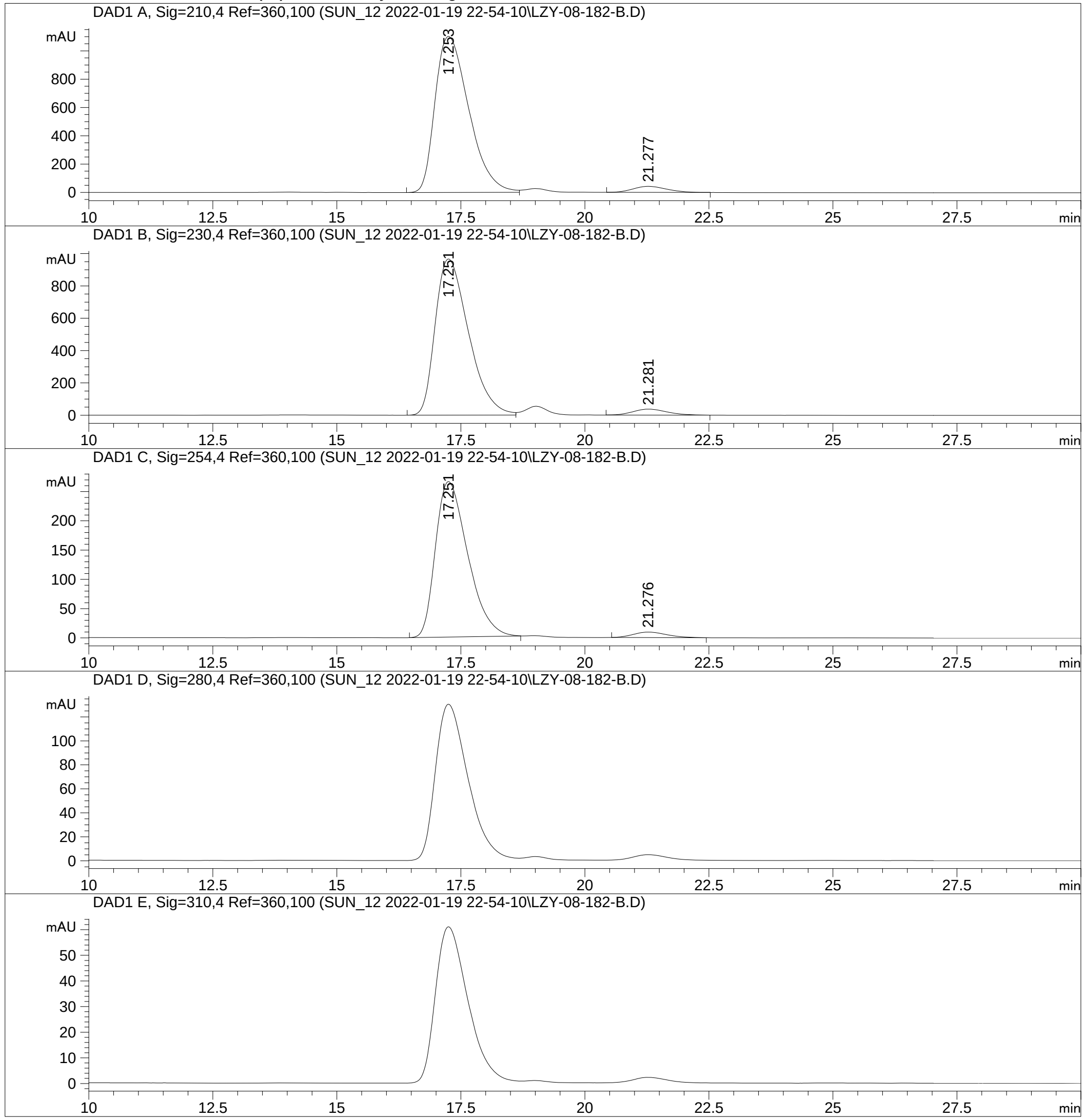

Data File C:\CHEM32\1\DATA\SUN_12 2022-01-19 22-54-10\LZY-08-182-B.D
Sample Name:

===
Area Percent Report
===

Sorted By : Signal
Multiplier : 1.0000
Dilution : 1.0000
Use Multiplier & Dilution Factor with ISTDs

Signal 1: DAD1 A, Sig=210,4 Ref=360,100

Peak #	RetTime [min]	Type	Width [min]	Area [mAU*s]	Height [mAU]	Area %
1	17.253	BV	0.7339	5.21732e4	1102.98828	96.2732
2	21.277	BB	0.7018	2019.64819	42.70162	3.7268

Totals : 5.41928e4 1145.68990

Signal 2: DAD1 B, Sig=230,4 Ref=360,100

Peak #	RetTime [min]	Type	Width [min]	Area [mAU*s]	Height [mAU]	Area %
1	17.251	BV	0.7221	4.50694e4	966.35632	96.2615
2	21.281	BB	0.7297	1750.33521	36.87900	3.7385

Totals : 4.68198e4 1003.23532

Signal 3: DAD1 C, Sig=254,4 Ref=360,100

Peak #	RetTime [min]	Type	Width [min]	Area [mAU*s]	Height [mAU]	Area %
1	17.251	BB	0.7008	1.19957e4	265.67215	96.4929
2	21.276	BB	0.7108	435.98560	9.37082	3.5071

Totals : 1.24317e4 275.04297

Signal 4: DAD1 D, Sig=280,4 Ref=360,100

Signal 5: DAD1 E, Sig=310,4 Ref=360,100

===
*** End of Report ***

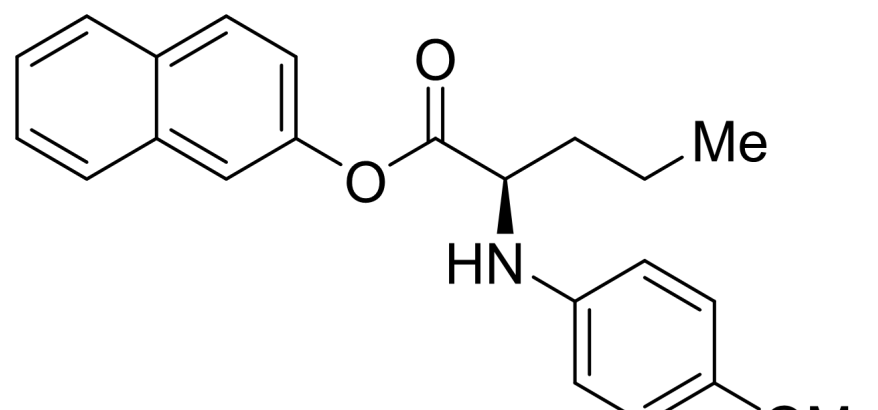

5e

Data File C:\CHEM32\1\DATA\SUN_12 2022-01-22 19-06-15\LWY-01-25-A.D
Sample Name:

===

Acq. Operator   :   Seq. Line : 18
Acq. Instrument : Instrument 1   Location : Vial 91
Injection Date : 1/22/2022 11:45:08 PM   Inj :   1
                                                Inj Volume : 5.000 µl
Different Inj Volume from Sequence !   Actual Inj Volume : 7.000 µl
Acq. Method   : C:\CHEM32\1\DATA\SUN_12 2022-01-22 19-06-15\AD-03-20.M
Last changed   : 7/2/2016 3:26:02 PM
Analysis Method : C:\CHEM32\1\METHODS\OD-03-60-0.6.M
Last changed   : 3/8/2022 3:10:58 PM
                  (modified after loading)
Additional Info : Peak(s) manually integrated

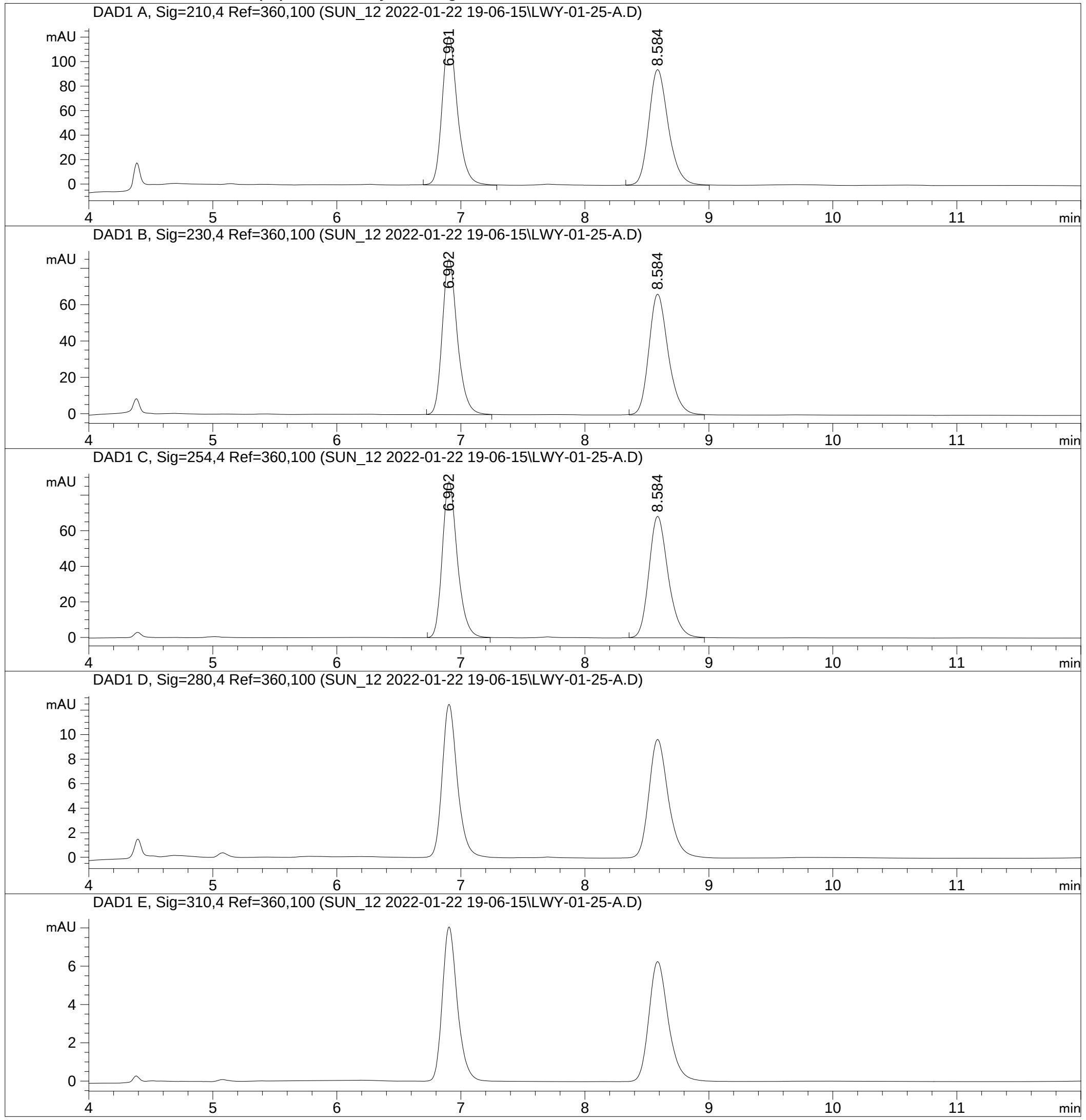

Data File C:\CHEM32\1\DATA\SUN_12 2022-01-22 19-06-15\LWY-01-25-A.D
Sample Name:

===

Area Percent Report

===

Sorted By   :   Signal
Multiplier   :   1.0000
Dilution   :   1.0000
Use Multiplier & Dilution Factor with ISTDs

Signal 1: DAD1 A, Sig=210,4 Ref=360,100

Peak #	RetTime [min]	Type	Width [min]	Area [mAU*s]	Height [mAU]	Area %
1	6.901	BB	0.1323	1043.74231	121.44309	50.1592
2	8.584	BB	0.1660	1037.11499	94.50972	49.8408

Totals :   2080.85730 215.95281

Signal 2: DAD1 B, Sig=230,4 Ref=360,100

Peak #	RetTime [min]	Type	Width [min]	Area [mAU*s]	Height [mAU]	Area %
1	6.902	BB	0.1283	722.15198	85.71149	50.0730
2	8.584	BB	0.1643	720.04584	66.49401	49.9270

Totals :   1442.19781 152.20550

Signal 3: DAD1 C, Sig=254,4 Ref=360,100

Peak #	RetTime [min]	Type	Width [min]	Area [mAU*s]	Height [mAU]	Area %
1	6.902	BB	0.1279	736.79138	87.77656	49.9075
2	8.584	BB	0.1643	739.52350	68.32912	50.0925

Totals :   1476.31488 156.10568

Signal 4: DAD1 D, Sig=280,4 Ref=360,100

Signal 5: DAD1 E, Sig=310,4 Ref=360,100

===

*** End of Report ***

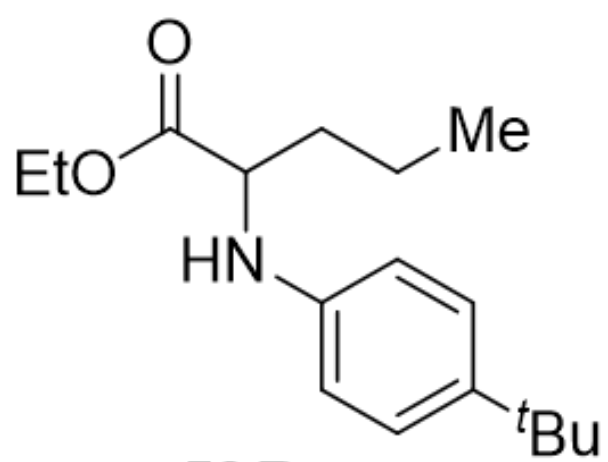

5f-Rac

Data File C:\CHEM32\1\DATA\SUN_12 2022-01-22 19-06-15\LZY-08-189-A.D
Sample Name:

===

Acq. Operator   :   Seq. Line :   6
Acq. Instrument : Instrument 1   Location : Vial 21
Injection Date : 1/22/2022 8:39:08 PM   Inj :   1
Inj Volume : 5.000 µl
Different Inj Volume from Sequence !   Actual Inj Volume : 6.000 µl
Acq. Method   : C:\CHEM32\1\DATA\SUN_12 2022-01-22 19-06-15\AD-03-20.M
Last changed   : 1/22/2022 8:55:36 PM
(modified after loading)
Analysis Method : C:\CHEM32\1\METHODS\OD-03-60-0.6.M
Last changed   : 3/8/2022 3:10:58 PM
(modified after loading)
Additional Info : Peak(s) manually integrated

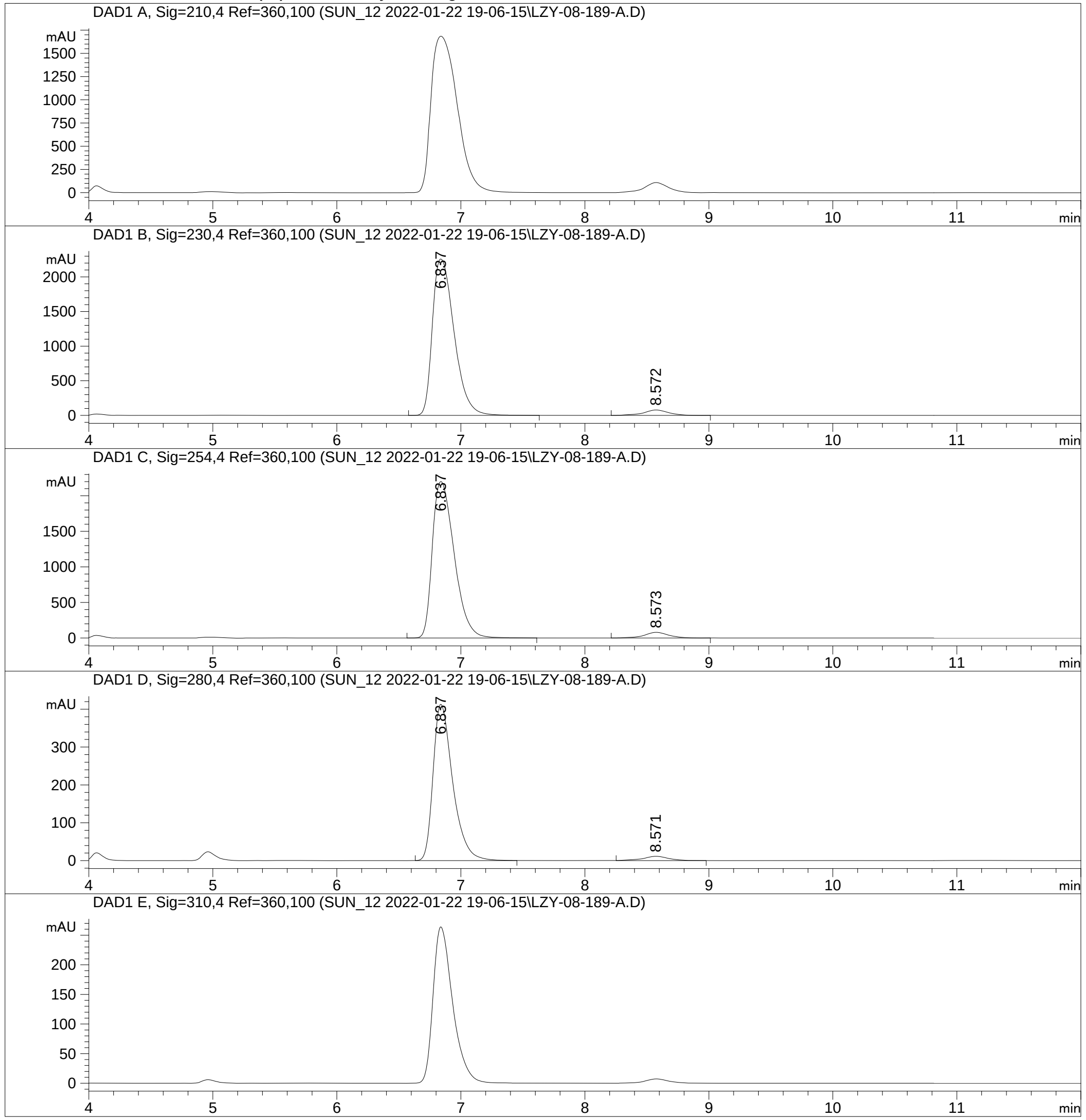

Data File C:\CHEM32\1\DATA\SUN_12 2022-01-22 19-06-15\LZY-08-189-A.D
Sample Name:

===

Area Percent Report

===

Sorted By   :   Signal
Multiplier   :   1.0000
Dilution   :   1.0000
Use Multiplier & Dilution Factor with ISTDs

Signal 1: DAD1 A, Sig=210,4 Ref=360,100

Signal 2: DAD1 B, Sig=230,4 Ref=360,100

Peak #	RetTime [min]	Type	Width [min]	Area [mAU*s]	Height [mAU]	Area %
1	6.837	BB	0.1899	2.74814e4	2259.57422	96.1805
2	8.572	BB	0.2069	1091.32593	78.13806	3.8195

Totals :   2.85727e4 2337.71228

Signal 3: DAD1 C, Sig=254,4 Ref=360,100

Peak #	RetTime [min]	Type	Width [min]	Area [mAU*s]	Height [mAU]	Area %
1	6.837	BB	0.1928	2.69223e4	2199.93921	96.1346
2	8.573	BB	0.2022	1082.50049	79.80846	3.8654

Totals :   2.80048e4 2279.74767

Signal 4: DAD1 D, Sig=280,4 Ref=360,100

Peak #	RetTime [min]	Type	Width [min]	Area [mAU*s]	Height [mAU]	Area %
1	6.837	BB	0.1662	4541.65332	413.25385	96.5081
2	8.571	BB	0.2117	164.32716	11.42931	3.4919

Totals :   4705.98048 424.68315

Signal 5: DAD1 E, Sig=310,4 Ref=360,100

===

*** End of Report ***

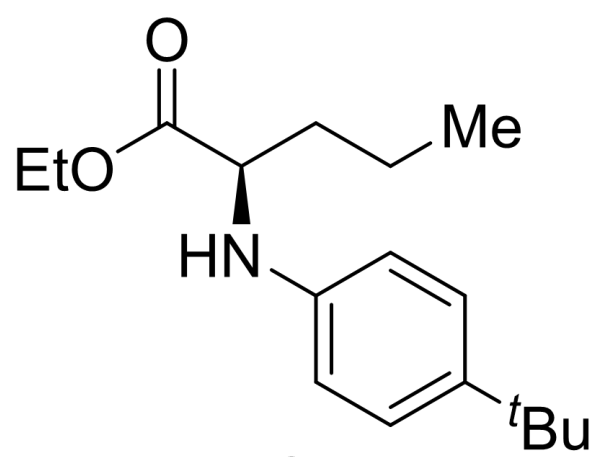

5f

===

```
Acq. Operator   :                               Seq. Line :   2
Acq. Instrument : Instrument 1                   Location : Vial 92
Injection Date  : 1/24/2022 9:47:15 AM                Inj :   1
                                               Inj Volume : 5.000 µl
Different Inj Volume from Sequence !     Actual Inj Volume : 9.000 µl
Acq. Method     : C:\CHEM32\1\DATA\SUN_12 2022-01-24 09-34-24\IC-03-30.M
Last changed    : 1/24/2022 9:46:20 AM
                  (modified after loading)
Analysis Method : C:\CHEM32\1\METHODS\OD-03-60-0.6.M
Last changed    : 3/8/2022 2:51:32 PM
                  (modified after loading)
Additional Info : Peak(s) manually integrated
```

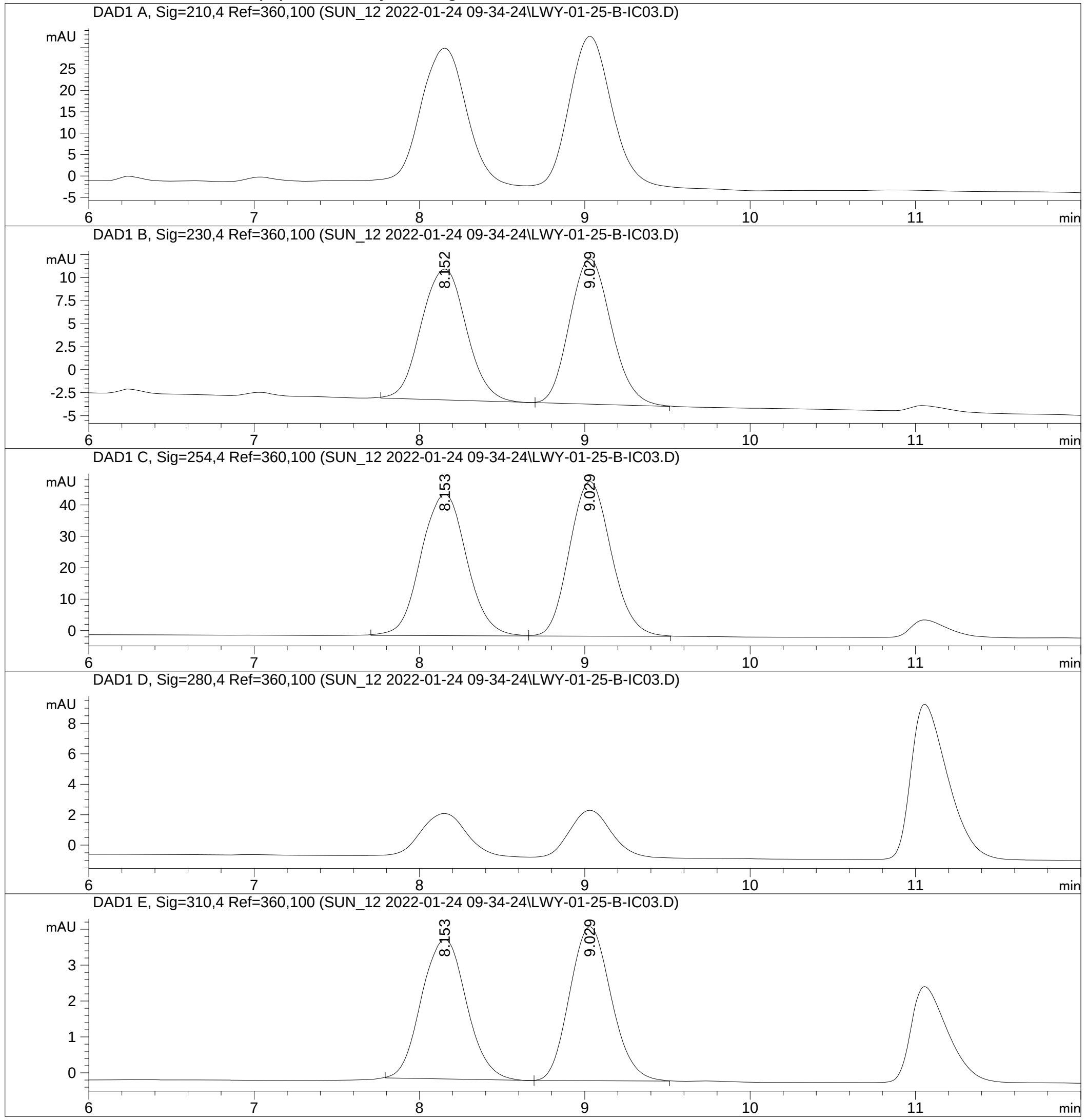

Data File C:\CHEM32\1\DATA\SUN_12 2022-01-24 09-34-24\LWY-01-25-B-IC03.D
Sample Name:

===
Area Percent Report
===

```
Sorted By             :      Signal
Multiplier            :      1.0000
Dilution              :      1.0000
Use Multiplier & Dilution Factor with ISTDs
```

Signal 1: DAD1 A, Sig=210,4 Ref=360,100

Signal 2: DAD1 B, Sig=230,4 Ref=360,100

Peak #	RetTime [min]	Type	Width [min]	Area [mAU*s]	Height [mAU]	Area %
1	8.152	BB	0.3026	268.83502	14.21569	49.3278
2	9.029	BB	0.2762	276.16174	15.77876	50.6722

Totals : 544.99677 29.99445

Signal 3: DAD1 C, Sig=254,4 Ref=360,100

Peak #	RetTime [min]	Type	Width [min]	Area [mAU*s]	Height [mAU]	Area %
1	8.153	BV	0.3057	862.93597	45.01241	49.9758
2	9.029	VB	0.2767	863.77258	49.22673	50.0242

Totals : 1726.70856 94.23914

Signal 4: DAD1 D, Sig=280,4 Ref=360,100

Signal 5: DAD1 E, Sig=310,4 Ref=360,100

Peak #	RetTime [min]	Type	Width [min]	Area [mAU*s]	Height [mAU]	Area %
1	8.153	BB	0.2999	73.28806	3.88787	49.4841
2	9.029	BB	0.2754	74.81618	4.29028	50.5159

Totals : 148.10424 8.17815

===
*** End of Report ***

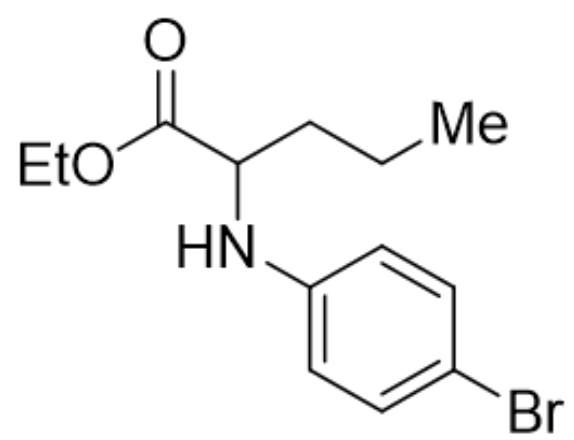

5g-Rac

```
Data File C:\CHEM32\1\DATA\SUN_12 2022-01-24 09-34-24\LZY-08-189-B-IC03.D
Sample Name:

=====================================================================
Acq. Operator   :                        Seq. Line :   5
Acq. Instrument : Instrument 1            Location : Vial 22
Injection Date  : 1/24/2022 10:40:27 AM        Inj :   1
                                        Inj Volume : 5.000 µl
Different Inj Volume from Sequence !   Actual Inj Volume : 4.000 µl
Acq. Method     : C:\CHEM32\1\DATA\SUN_12 2022-01-24 09-34-24\IC-03-30.M
Last changed    : 12/28/2012 10:25:26 PM
Analysis Method : C:\CHEM32\1\METHODS\OD-03-60-0.6.M
Last changed    : 3/8/2022 2:51:32 PM
                  (modified after loading)
Additional Info : Peak(s) manually integrated
```

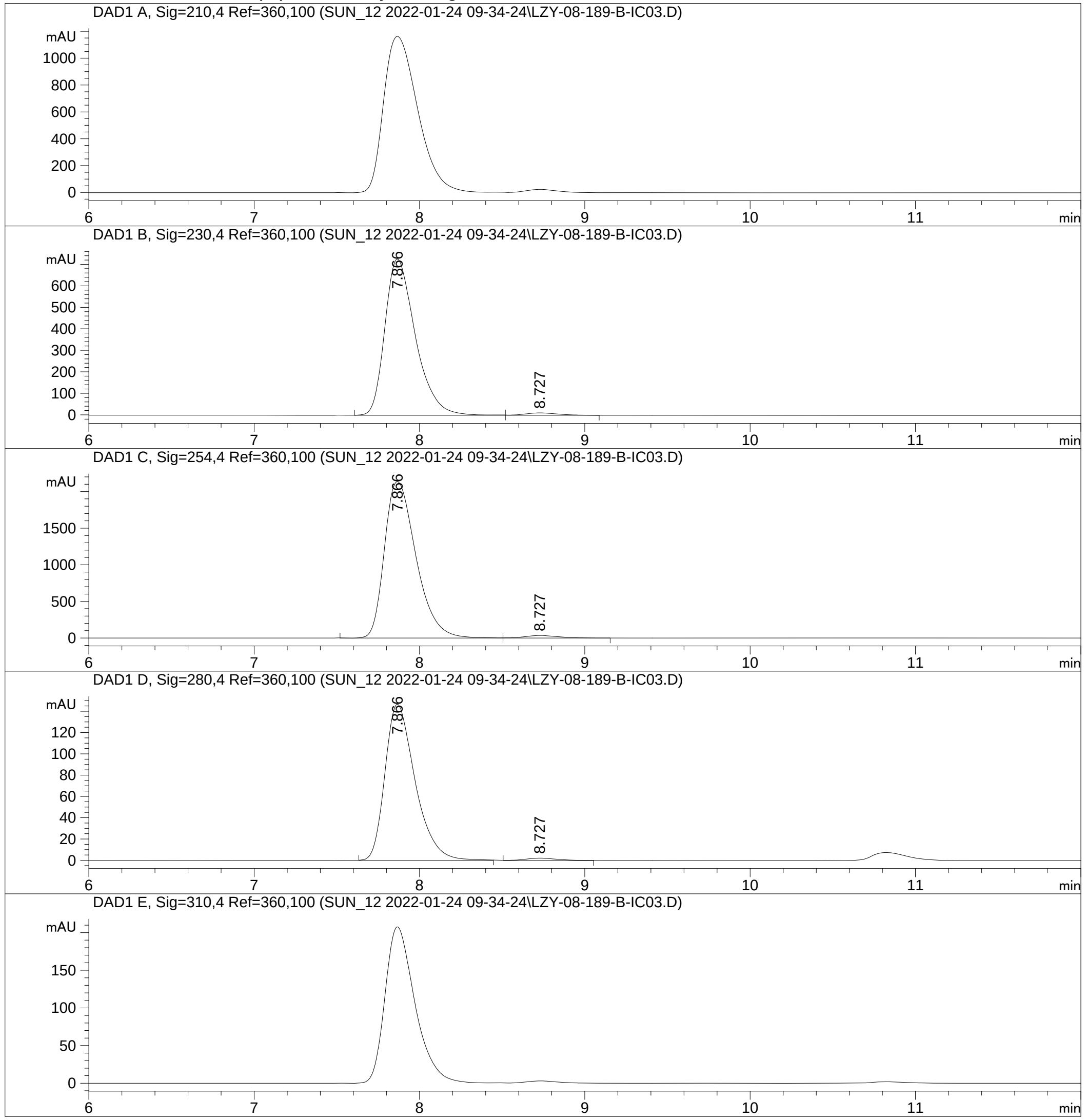

Data File C:\CHEM32\1\DATA\SUN_12 2022-01-24 09-34-24\LZY-08-189-B-IC03.D
Sample Name:

Area Percent Report

```
Sorted By            :      Signal
Multiplier           :      1.0000
Dilution             :      1.0000
Use Multiplier & Dilution Factor with ISTDs
```

Signal 1: DAD1 A, Sig=210,4 Ref=360,100

Signal 2: DAD1 B, Sig=230,4 Ref=360,100

Peak #	RetTime [min]	Type	Width [min]	Area [mAU*s]	Height [mAU]	Area %
1	7.866	BB	0.1950	9279.56543	726.81427	98.4183
2	8.727	BB	0.2053	149.13838	11.06322	1.5817
Totals :				9428.70381	737.87749	

Signal 3: DAD1 C, Sig=254,4 Ref=360,100

Peak #	RetTime [min]	Type	Width [min]	Area [mAU*s]	Height [mAU]	Area %
1	7.866	BV	0.2047	2.83248e4	2136.65088	98.3546
2	8.727	VB	0.2048	473.83878	34.80652	1.6454
Totals :				2.87986e4	2171.45740	

Signal 4: DAD1 D, Sig=280,4 Ref=360,100

Peak #	RetTime [min]	Type	Width [min]	Area [mAU*s]	Height [mAU]	Area %
1	7.866	BB	0.1940	1862.29846	146.81532	98.4233
2	8.727	BB	0.2060	29.83269	2.20340	1.5767
Totals :				1892.13115	149.01872	

Signal 5: DAD1 E, Sig=310,4 Ref=360,100

*** End of Report ***

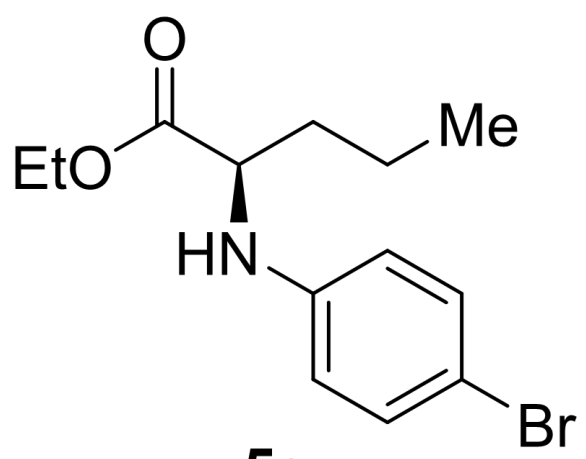

5g

Data File C:\CHEM32\1\DATA\COLUMNWASHING 2022-03-09 10-24-17\LZY-09-46-B-RAC.D
Sample Name:

===

Acq. Operator : Seq. Line : 16
Acq. Instrument : Instrument 1 Location : Vial 27
Injection Date : 3/9/2022 3:02:32 PM Inj : 1
Inj Volume : 5.000 µl
Different Inj Volume from Sequence ! Actual Inj Volume : 8.000 µl
Acq. Method : C:\CHEM32\1\DATA\COLUMNWASHING 2022-03-09 10-24-17\IC-03-30.M
Last changed : 12/28/2012 10:25:26 PM
Analysis Method : C:\CHEM32\1\METHODS\OD-03-60-0.6.M
Last changed : 3/11/2022 12:02:12 PM
(modified after loading)
Additional Info : Peak(s) manually integrated

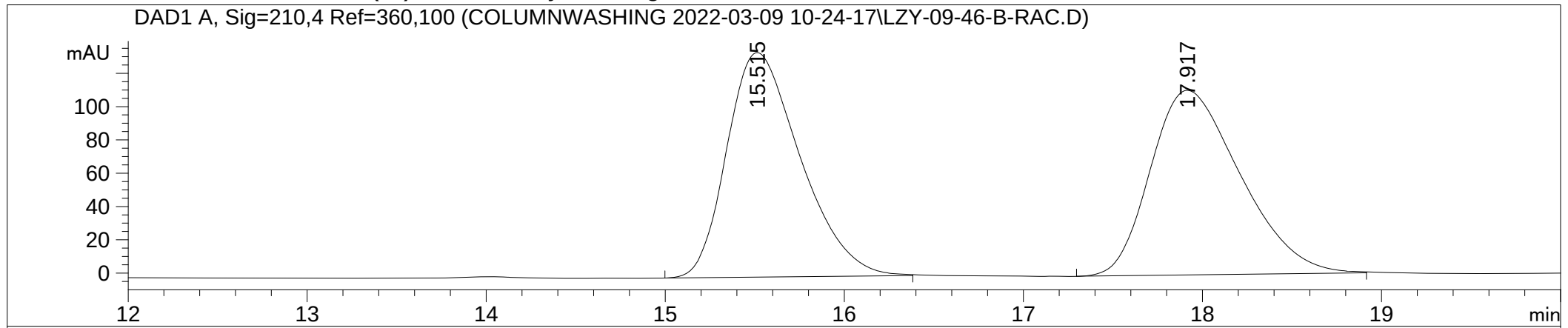

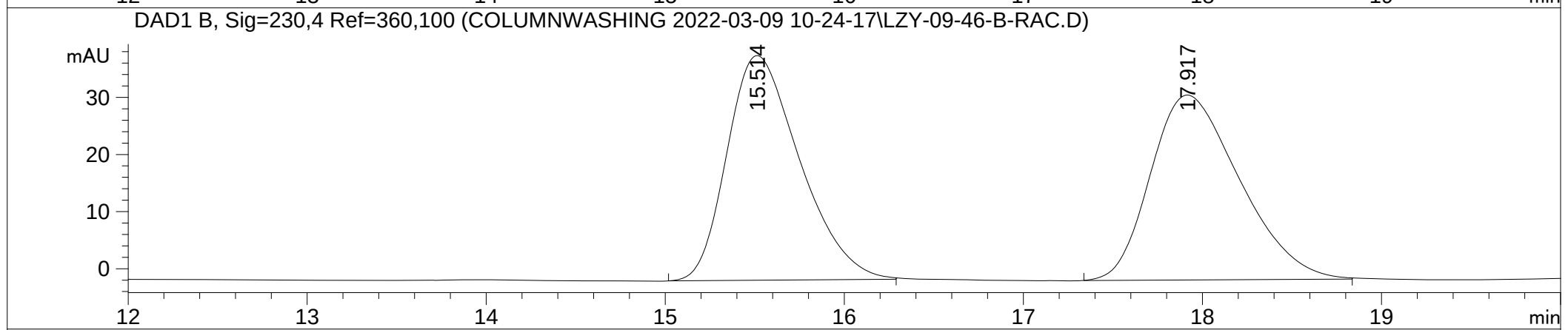

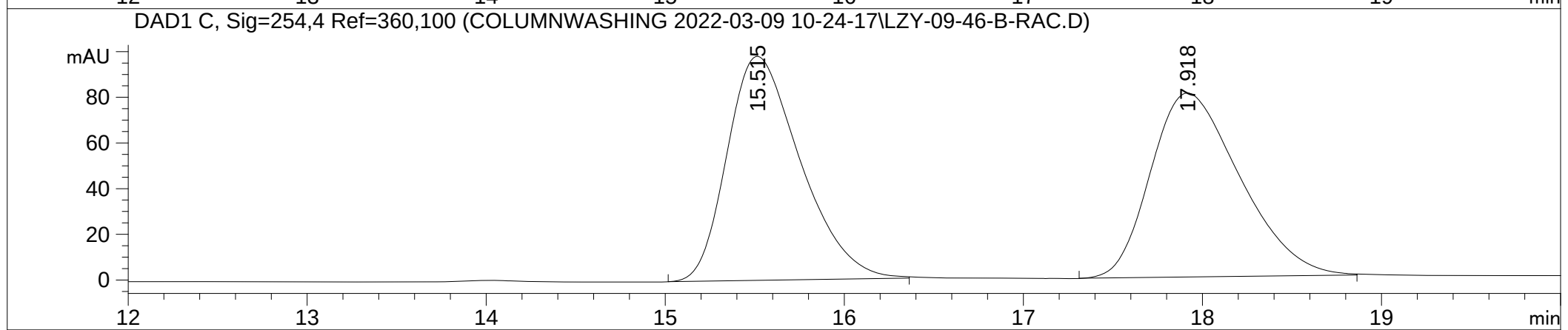

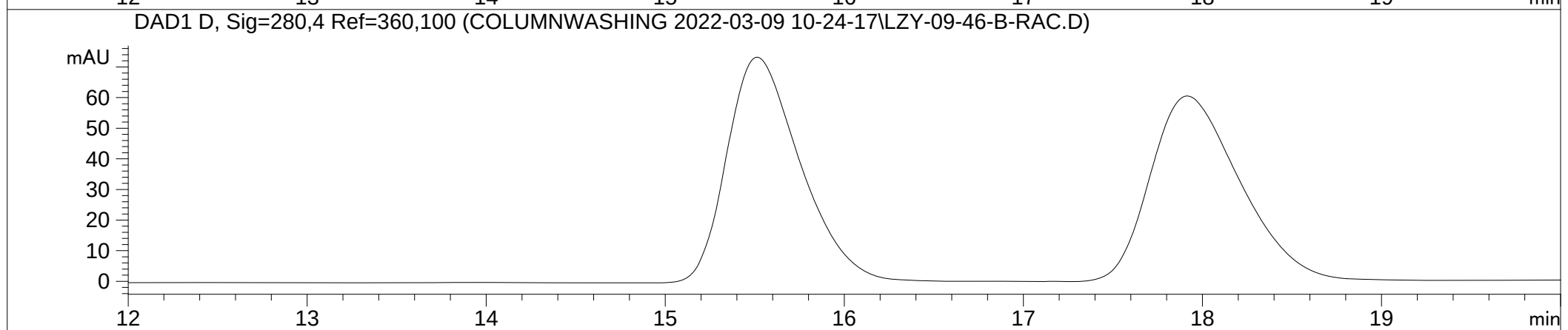

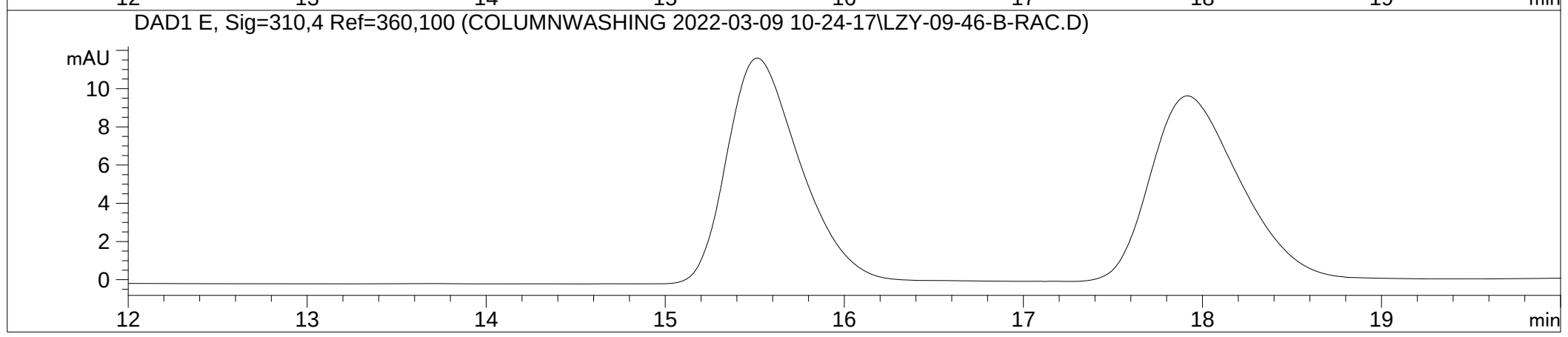

Data File C:\CHEM32\1\DATA\COLUMNWASHING 2022-03-09 10-24-17\LZY-09-46-B-RAC.D
Sample Name:

===

Area Percent Report

===

Sorted By : Signal
Multiplier : 1.0000
Dilution : 1.0000
Use Multiplier & Dilution Factor with ISTDs

Signal 1: DAD1 A, Sig=210,4 Ref=360,100

Peak #	RetTime [min]	Type	Width [min]	Area [mAU*s]	Height [mAU]	Area %
1	15.515	BB	0.4342	3841.54517	134.90642	49.8625
2	17.917	BB	0.5305	3862.73486	110.86703	50.1375

Totals : 7704.28003 245.77345

Signal 2: DAD1 B, Sig=230,4 Ref=360,100

Peak #	RetTime [min]	Type	Width [min]	Area [mAU*s]	Height [mAU]	Area %
1	15.514	BB	0.4332	1110.63562	39.35614	49.8403
2	17.917	BB	0.5349	1117.75220	32.36687	50.1597

Totals : 2228.38782 71.72301

Signal 3: DAD1 C, Sig=254,4 Ref=360,100

Peak #	RetTime [min]	Type	Width [min]	Area [mAU*s]	Height [mAU]	Area %
1	15.515	BB	0.4336	2787.89185	98.10013	49.9696
2	17.918	BB	0.5362	2791.28369	80.57265	50.0304

Totals : 5579.17554 178.67278

Signal 4: DAD1 D, Sig=280,4 Ref=360,100

Signal 5: DAD1 E, Sig=310,4 Ref=360,100

===

*** End of Report ***

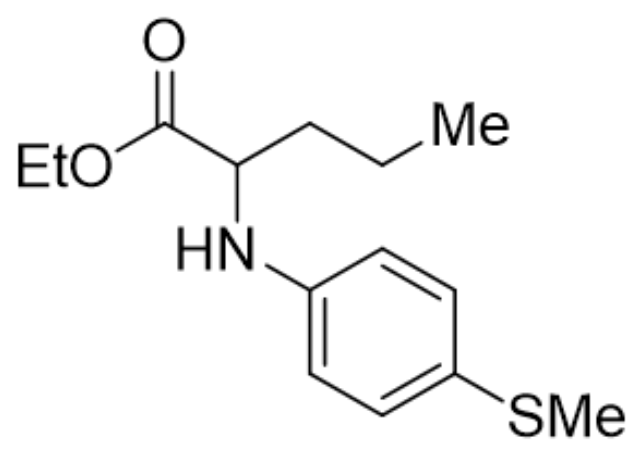

5h-Rac

===

Acq. Operator : Seq. Line : 17
Acq. Instrument : Instrument 1 Location : Vial 28
Injection Date : 3/9/2022 3:33:32 PM Inj : 1
Inj Volume : 5.000 µl
Different Inj Volume from Sequence ! Actual Inj Volume : 3.000 µl
Acq. Method : C:\CHEM32\1\DATA\COLUMNWASHING 2022-03-09 10-24-17\IC-03-30.M
Last changed : 12/28/2012 10:25:26 PM
Analysis Method : C:\CHEM32\1\METHODS\OD-03-60-0.6.M
Last changed : 3/11/2022 12:02:12 PM
(modified after loading)
Additional Info : Peak(s) manually integrated

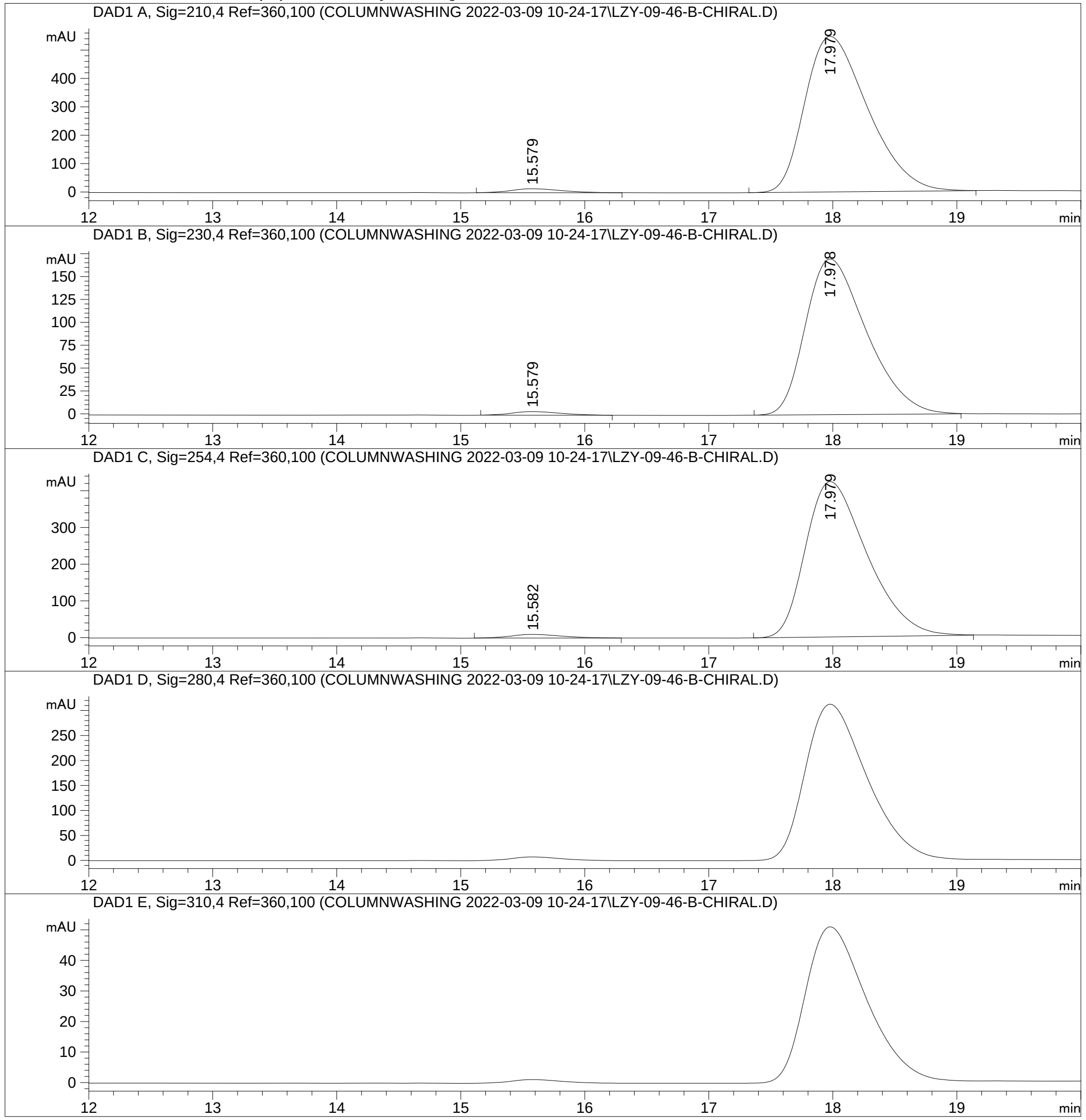

Data File C:\CHEM32\1\DATA\COLUMNWASHING 2022-03-09 10-24-17\LZY-09-46-B-CHIRAL.D
Sample Name:

===

Area Percent Report

===

Sorted By : Signal
Multiplier : 1.0000
Dilution : 1.0000
Use Multiplier & Dilution Factor with ISTDs

Signal 1: DAD1 A, Sig=210,4 Ref=360,100

Peak #	RetTime [min]	Type	Width [min]	Area [mAU*s]	Height [mAU]	Area %
1	15.579	BB	0.4178	390.91504	14.08627	1.9861
2	17.979	BB	0.5466	1.92916e4	548.19891	98.0139

Totals : 1.96825e4 562.28518

Signal 2: DAD1 B, Sig=230,4 Ref=360,100

Peak #	RetTime [min]	Type	Width [min]	Area [mAU*s]	Height [mAU]	Area %
1	15.579	BB	0.4109	107.46925	3.95614	1.8121
2	17.978	BB	0.5255	5823.26904	170.07140	98.1879

Totals : 5930.73830 174.02754

Signal 3: DAD1 C, Sig=254,4 Ref=360,100

Peak #	RetTime [min]	Type	Width [min]	Area [mAU*s]	Height [mAU]	Area %
1	15.582	BB	0.4234	274.33038	9.95746	1.8445
2	17.979	BB	0.5287	1.45989e4	422.96478	98.1555

Totals : 1.48732e4 432.92224

Signal 4: DAD1 D, Sig=280,4 Ref=360,100

Signal 5: DAD1 E, Sig=310,4 Ref=360,100

===

*** End of Report ***

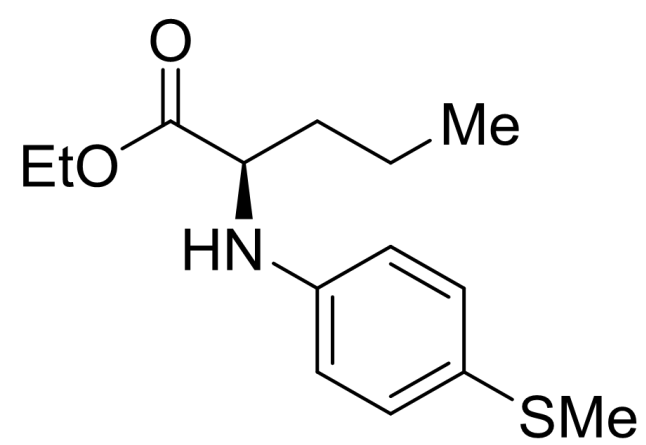

5h

===

Acq. Operator :	Seq. Line : 18
Acq. Instrument : Instrument 1	Location : Vial 29
Injection Date : 3/9/2022 4:04:34 PM	Inj : 1
	Inj Volume : 5.000 µl
Different Inj Volume from Sequence !	Actual Inj Volume : 8.000 µl

Acq. Method : C:\CHEM32\1\DATA\COLUMNWASHING 2022-03-09 10-24-17\IC-03-20.M
Last changed : 9/18/2012 8:18:47 PM
Analysis Method : C:\CHEM32\1\METHODS\OD-03-60-0.6.M
Last changed : 3/11/2022 12:05:03 PM
(modified after loading)
Additional Info : Peak(s) manually integrated

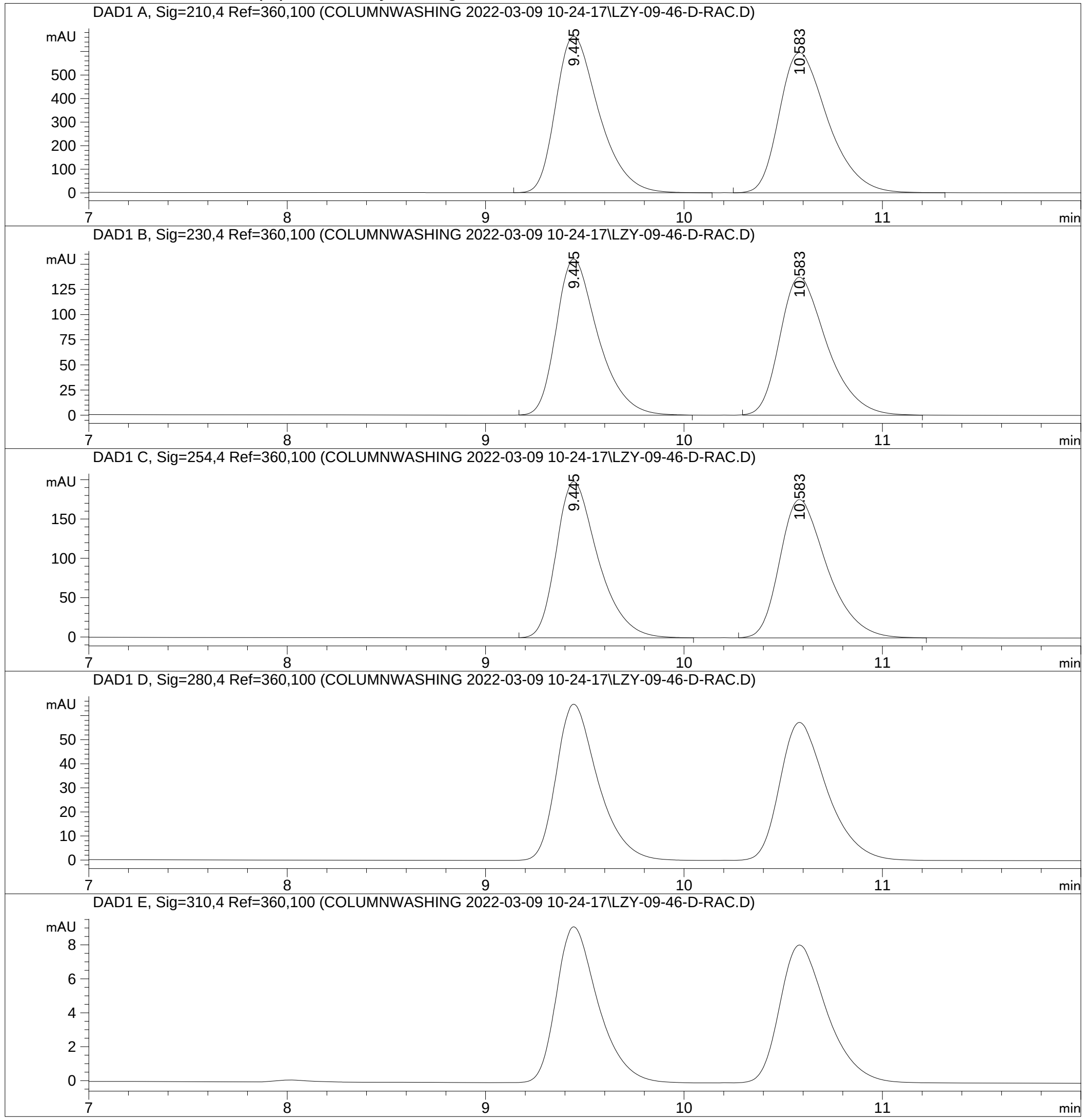

Data File C:\CHEM32\1\DATA\COLUMNWASHING 2022-03-09 10-24-17\LZY-09-46-D-RAC.D
Sample Name:

===
Area Percent Report
===

Sorted By : Signal
Multiplier : 1.0000
Dilution : 1.0000
Use Multiplier & Dilution Factor with ISTDs

Signal 1: DAD1 A, Sig=210,4 Ref=360,100

Peak #	RetTime [min]	Type	Width [min]	Area [mAU*s]	Height [mAU]	Area %
1	9.445	BB	0.2323	1.00317e4	663.80573	49.7664
2	10.583	BB	0.2605	1.01258e4	595.09601	50.2336

Totals : 2.01575e4 1258.90173

Signal 2: DAD1 B, Sig=230,4 Ref=360,100

Peak #	RetTime [min]	Type	Width [min]	Area [mAU*s]	Height [mAU]	Area %
1	9.445	BB	0.2229	2274.60083	155.30991	50.0721
2	10.583	BB	0.2510	2268.05371	137.12770	49.9279

Totals : 4542.65454 292.43761

Signal 3: DAD1 C, Sig=254,4 Ref=360,100

Peak #	RetTime [min]	Type	Width [min]	Area [mAU*s]	Height [mAU]	Area %
1	9.445	BB	0.2230	2912.13647	198.71487	49.9774
2	10.583	BB	0.2513	2914.77295	175.88425	50.0226

Totals : 5826.90942 374.59912

Signal 4: DAD1 D, Sig=280,4 Ref=360,100

Signal 5: DAD1 E, Sig=310,4 Ref=360,100

===
*** End of Report ***

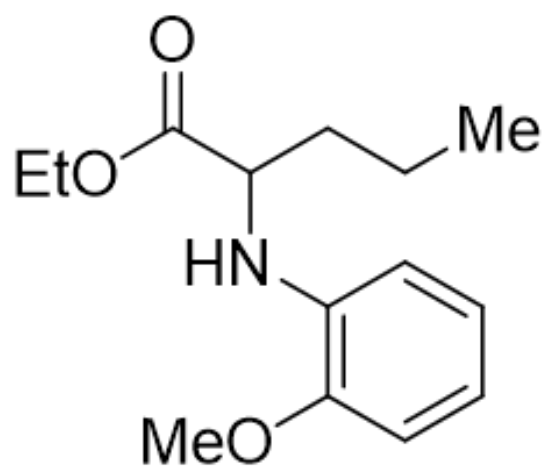

5i-Rac

Data File C:\CHEM32\1\DATA\COLUMNWASHING 2022-03-09 10-24-17\LZY-09-46-D-CHIRAL.D
Sample Name:

===

Acq. Operator : Seq. Line : 19
Acq. Instrument : Instrument 1 Location : Vial 30
Injection Date : 3/9/2022 4:25:36 PM Inj : 1
Inj Volume : 5.000 µl
Different Inj Volume from Sequence ! Actual Inj Volume : 3.000 µl
Acq. Method : C:\CHEM32\1\DATA\COLUMNWASHING 2022-03-09 10-24-17\IC-03-20.M
Last changed : 9/18/2012 8:18:47 PM
Analysis Method : C:\CHEM32\1\METHODS\OD-03-60-0.6.M
Last changed : 3/11/2022 12:05:03 PM
(modified after loading)
Additional Info : Peak(s) manually integrated

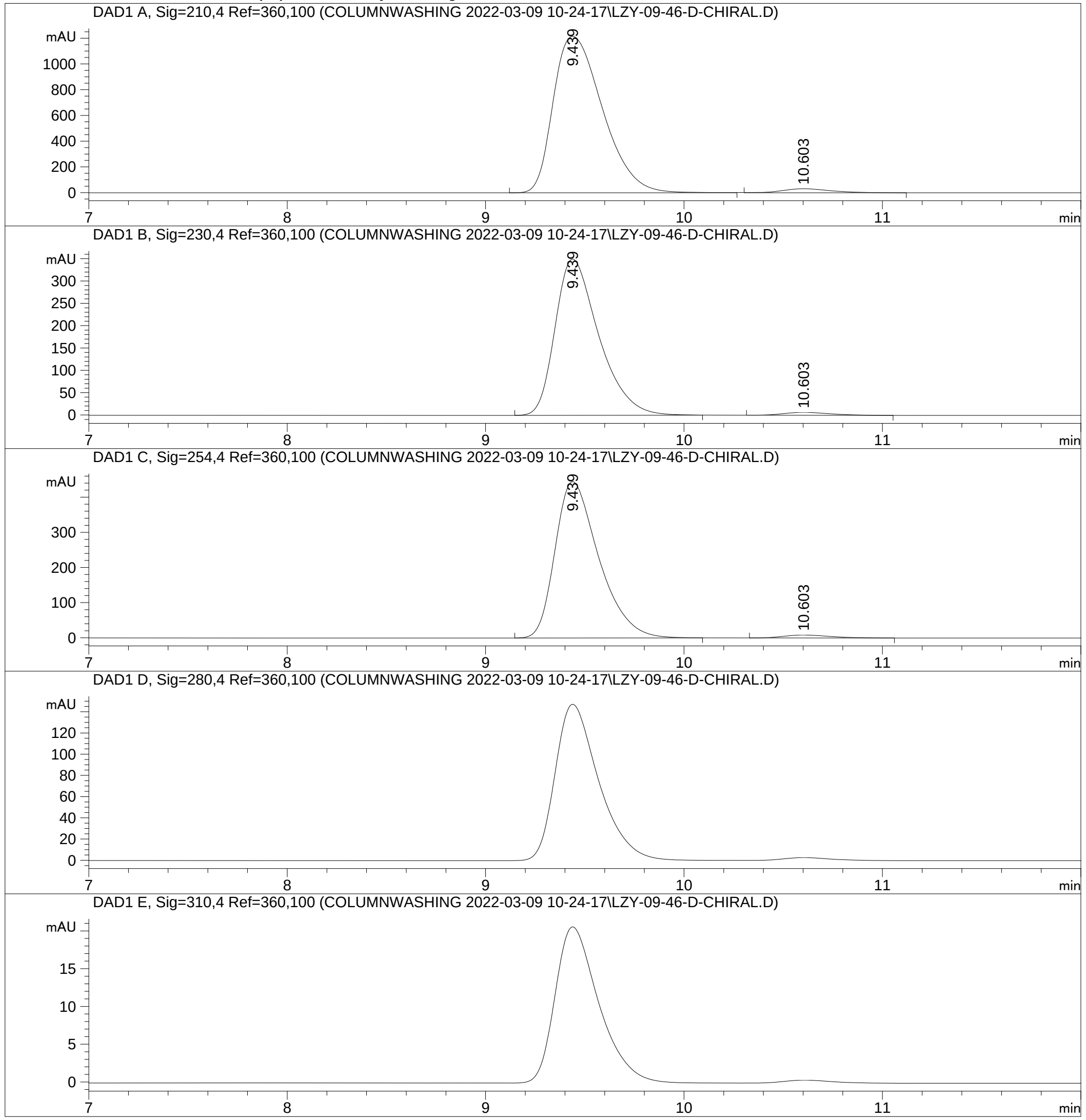

Data File C:\CHEM32\1\DATA\COLUMNWASHING 2022-03-09 10-24-17\LZY-09-46-D-CHIRAL.D
Sample Name:

===

Area Percent Report

===

Sorted By : Signal
Multiplier : 1.0000
Dilution : 1.0000
Use Multiplier & Dilution Factor with ISTDs

Signal 1: DAD1 A, Sig=210,4 Ref=360,100

Peak #	RetTime [min]	Type	Width [min]	Area [mAU*s]	Height [mAU]	Area %
1	9.439	BB	0.2710	2.09488e4	1215.78943	97.4584
2	10.603	BB	0.2590	546.31476	31.71371	2.5416

Totals : 2.14951e4 1247.50314

Signal 2: DAD1 B, Sig=230,4 Ref=360,100

Peak #	RetTime [min]	Type	Width [min]	Area [mAU*s]	Height [mAU]	Area %
1	9.439	BB	0.2319	5339.57861	350.08887	98.0373
2	10.603	BB	0.2501	106.89764	6.55886	1.9627

Totals : 5446.47626 356.64772

Signal 3: DAD1 C, Sig=254,4 Ref=360,100

Peak #	RetTime [min]	Type	Width [min]	Area [mAU*s]	Height [mAU]	Area %
1	9.439	BB	0.2326	6810.40527	444.94791	98.0178
2	10.603	BB	0.2506	137.72484	8.42884	1.9822

Totals : 6948.13011 453.37674

Signal 4: DAD1 D, Sig=280,4 Ref=360,100

Signal 5: DAD1 E, Sig=310,4 Ref=360,100

===

*** End of Report ***

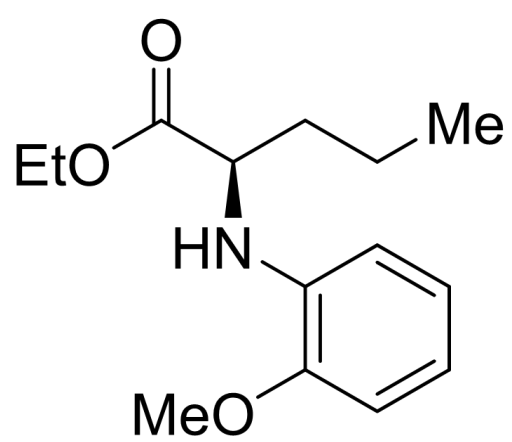

5i

Data File C:\CHEM32\1\DATA\SUN_12 2022-01-22 19-06-15\LWY-01-25-C.D
Sample Name:

===

Acq. Operator : Seq. Line : 22
Acq. Instrument : Instrument 1 Location : Vial 93
Injection Date : 1/23/2022 12:42:59 AM Inj : 1
Inj Volume : 5.000 µl
Different Inj Volume from Sequence ! Actual Inj Volume : 7.000 µl
Acq. Method : C:\CHEM32\1\DATA\SUN_12 2022-01-22 19-06-15\OD-03-30.M
Last changed : 1/31/2015 11:39:09 AM
Analysis Method : C:\CHEM32\1\METHODS\OD-03-60-0.6.M
Last changed : 3/8/2022 3:13:43 PM
(modified after loading)
Additional Info : Peak(s) manually integrated

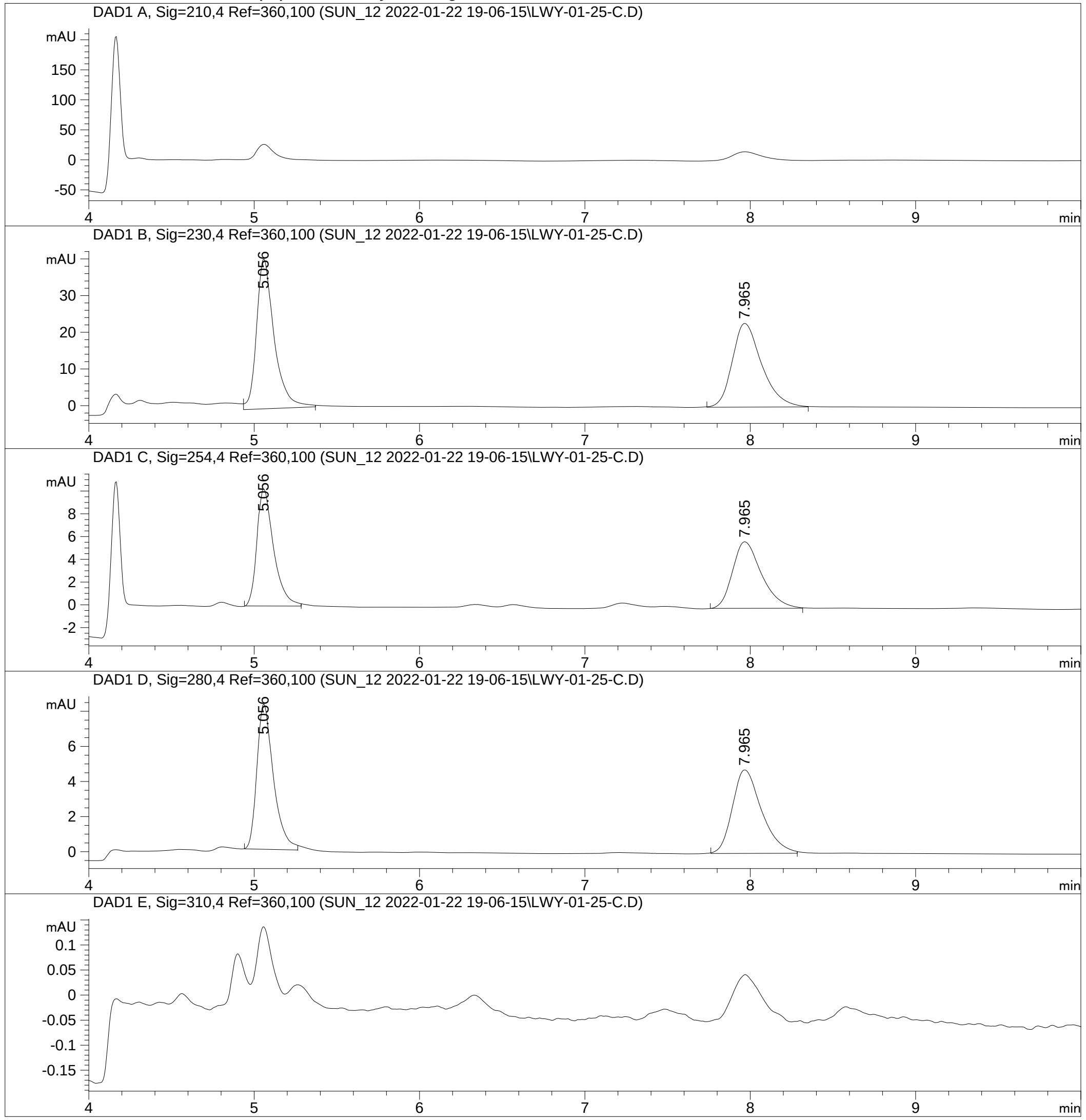

Data File C:\CHEM32\1\DATA\SUN_12 2022-01-22 19-06-15\LWY-01-25-C.D
Sample Name:

===

Area Percent Report

===

Sorted By : Signal
Multiplier : 1.0000
Dilution : 1.0000
Use Multiplier & Dilution Factor with ISTDs

Signal 1: DAD1 A, Sig=210,4 Ref=360,100

Signal 2: DAD1 B, Sig=230,4 Ref=360,100

Peak #	RetTime [min]	Type	Width [min]	Area [mAU*s]	Height [mAU]	Area %
1	5.056	BB	0.1102	302.14133	40.89255	51.6697
2	7.965	BB	0.1864	282.61411	22.83813	48.3303

Totals : 584.75543 63.73067

Signal 3: DAD1 C, Sig=254,4 Ref=360,100

Peak #	RetTime [min]	Type	Width [min]	Area [mAU*s]	Height [mAU]	Area %
1	5.056	BB	0.1059	72.31110	10.29782	50.3590
2	7.965	BB	0.1841	71.28006	5.85336	49.6410

Totals : 143.59116 16.15118

Signal 4: DAD1 D, Sig=280,4 Ref=360,100

Peak #	RetTime [min]	Type	Width [min]	Area [mAU*s]	Height [mAU]	Area %
1	5.056	BB	0.1051	57.49146	8.27496	49.6246
2	7.965	BB	0.1829	58.36125	4.76455	50.3754

Totals : 115.85271 13.03951

Signal 5: DAD1 E, Sig=310,4 Ref=360,100

===

*** End of Report ***

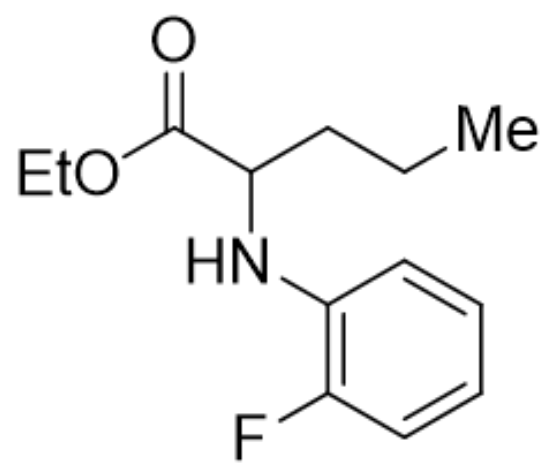

5j-Rac

===

Acq. Operator : Seq. Line : 10
Acq. Instrument : Instrument 1 Location : Vial 23
Injection Date : 1/22/2022 9:36:49 PM Inj : 1
Inj Volume : 5.000 µl
Different Inj Volume from Sequence ! Actual Inj Volume : 4.000 µl
Acq. Method : C:\CHEM32\1\DATA\SUN_12 2022-01-22 19-06-15\OD-03-10.M
Last changed : 1/22/2022 9:24:59 PM
(modified after loading)
Analysis Method : C:\CHEM32\1\METHODS\OD-03-60-0.6.M
Last changed : 3/8/2022 3:13:43 PM
(modified after loading)
Additional Info : Peak(s) manually integrated

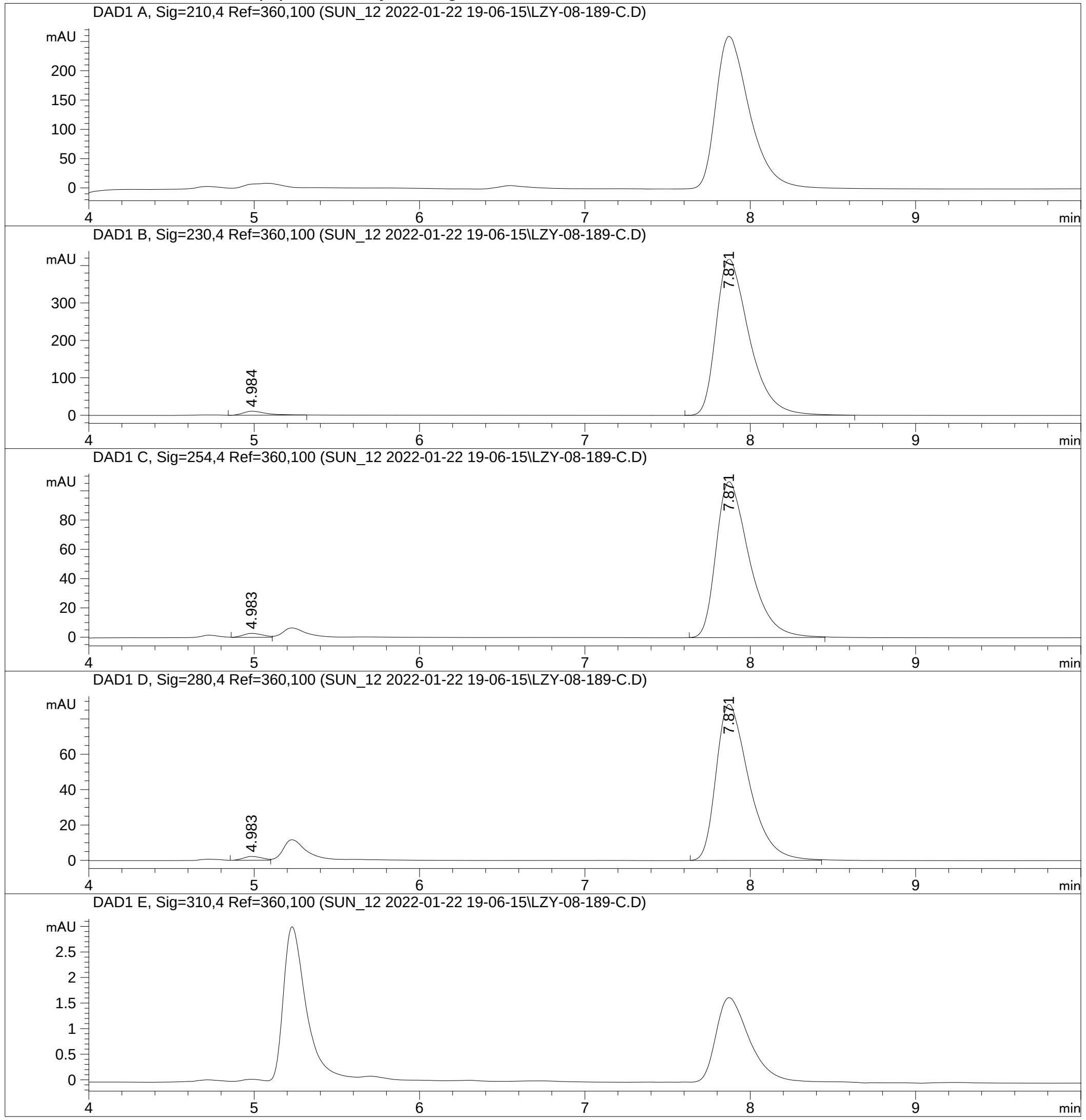

Data File C:\CHEM32\1\DATA\SUN_12 2022-01-22 19-06-15\LZY-08-189-C.D
Sample Name:

===

Area Percent Report

===

Sorted By : Signal
Multiplier : 1.0000
Dilution : 1.0000
Use Multiplier & Dilution Factor with ISTDs

Signal 1: DAD1 A, Sig=210,4 Ref=360,100

Signal 2: DAD1 B, Sig=230,4 Ref=360,100

Peak #	RetTime [min]	Type	Width [min]	Area [mAU*s]	Height [mAU]	Area %
1	4.984	VB	0.1415	102.75595	10.74801	1.7324
2	7.871	BB	0.2127	5828.65674	417.94220	98.2676

Totals : 5931.41269 428.69021

Signal 3: DAD1 C, Sig=254,4 Ref=360,100

Peak #	RetTime [min]	Type	Width [min]	Area [mAU*s]	Height [mAU]	Area %
1	4.983	VV	0.1262	22.85426	2.77269	1.5117
2	7.871	BB	0.2130	1488.98425	106.55775	98.4883

Totals : 1511.83851 109.33044

Signal 4: DAD1 D, Sig=280,4 Ref=360,100

Peak #	RetTime [min]	Type	Width [min]	Area [mAU*s]	Height [mAU]	Area %
1	4.983	VV	0.1199	16.46180	2.13629	1.3284
2	7.871	BB	0.2114	1222.73779	88.39456	98.6716

Totals : 1239.19959 90.53085

Signal 5: DAD1 E, Sig=310,4 Ref=360,100

===

*** End of Report ***

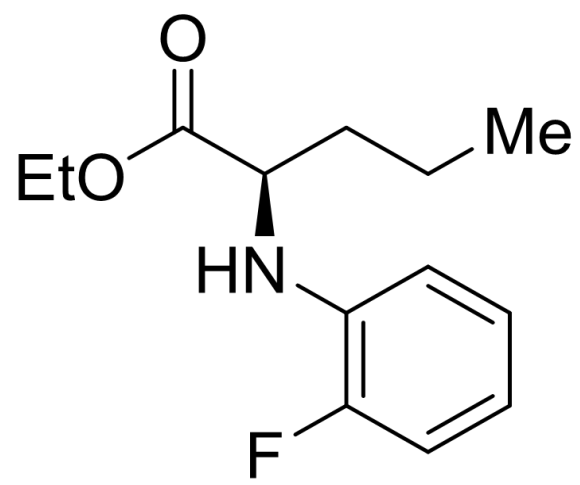

5j

Data File C:\CHEM32\1\DATA\SNAPSHOT.D
Sample Name:

```
=====================================================================
Acq. Operator   :                              Seq. Line :  14
                                                Location : Vial 25
Injection Date  : 3/9/2022 2:20:24 PM                Inj :   1
Acq. Method     : IC-02-30.M
Analysis Method : C:\CHEM32\1\METHODS\OD-03-60-0.6.M
Last changed    : 3/9/2022 2:32:55 PM
                  (modified after loading)
Additional Info : Peak(s) manually integrated
```

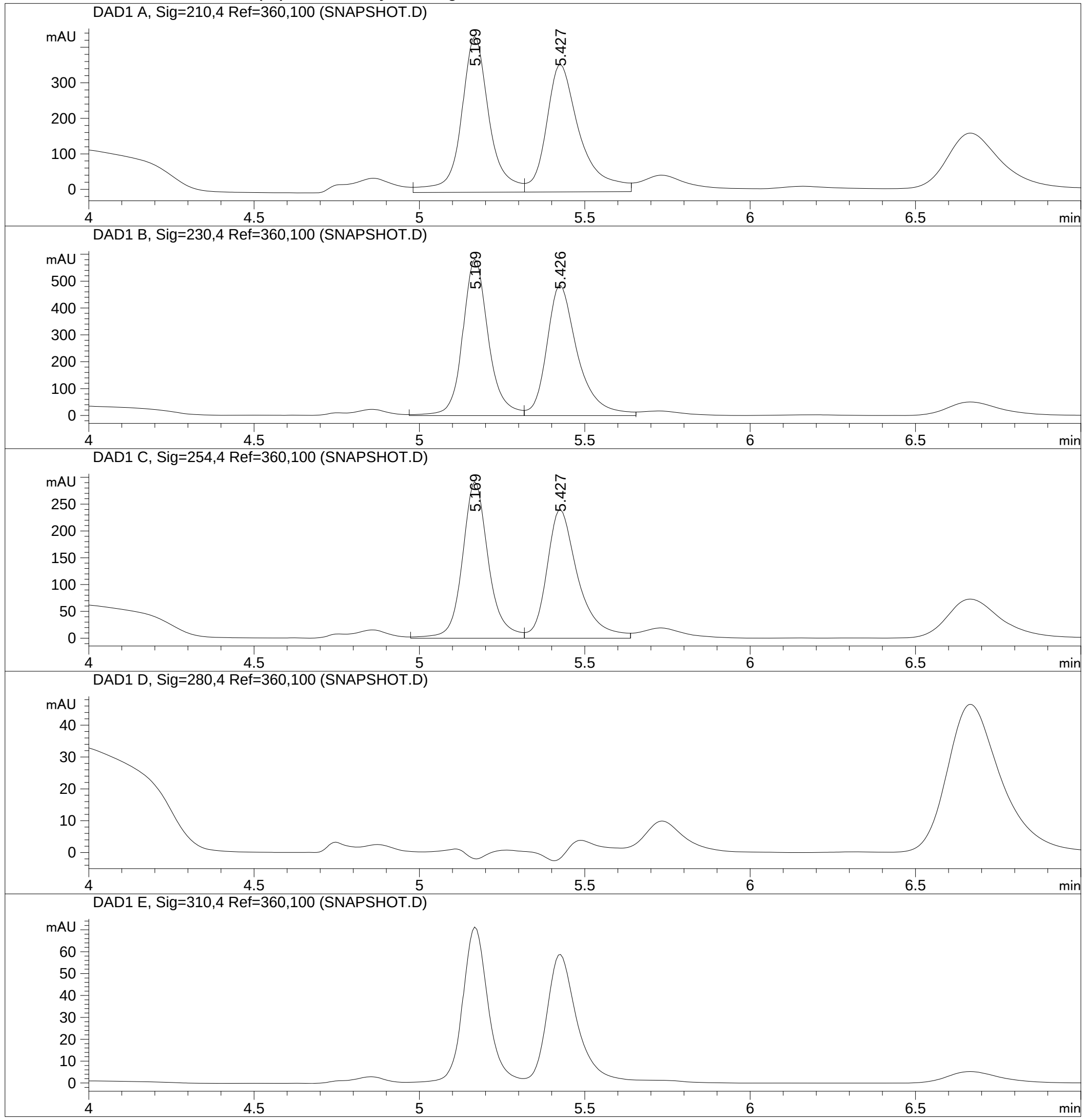

Data File C:\CHEM32\1\DATA\SNAPSHOT.D
Sample Name:

```
=====================================================================
                         Area Percent Report
=====================================================================

Sorted By             :      Signal
Multiplier            :      1.0000
Dilution              :      1.0000
Use Multiplier & Dilution Factor with ISTDs


Signal 1: DAD1 A, Sig=210,4 Ref=360,100

Peak RetTime Type  Width     Area      Height     Area
  #   [min]        [min]   [mAU*s]     [mAU]        %
----|-------|----|-------|----------|----------|--------|
   1   5.169 VV    0.0867 2583.11377  438.66153  50.5671
   2   5.427 VV    0.1020 2525.17651  359.25259  49.4329

Totals :                  5108.29028  797.91412


Signal 2: DAD1 B, Sig=230,4 Ref=360,100

Peak RetTime Type  Width     Area      Height     Area
  #   [min]        [min]   [mAU*s]     [mAU]        %
----|-------|----|-------|----------|----------|--------|
   1   5.169 VV    0.0811 3151.11230  582.93561  49.9881
   2   5.426 VV    0.0957 3152.61694  485.56699  50.0119

Totals :                  6303.72925 1068.50259


Signal 3: DAD1 C, Sig=254,4 Ref=360,100

Peak RetTime Type  Width     Area      Height     Area
  #   [min]        [min]   [mAU*s]     [mAU]        %
----|-------|----|-------|----------|----------|--------|
   1   5.169 VV    0.0817 1593.29834  291.80435  50.2822
   2   5.427 VV    0.0966 1575.41223  239.90224  49.7178

Totals :                  3168.71057  531.70659


Signal 4: DAD1 D, Sig=280,4 Ref=360,100


Signal 5: DAD1 E, Sig=310,4 Ref=360,100

=====================================================================
                          *** End of Report ***
```

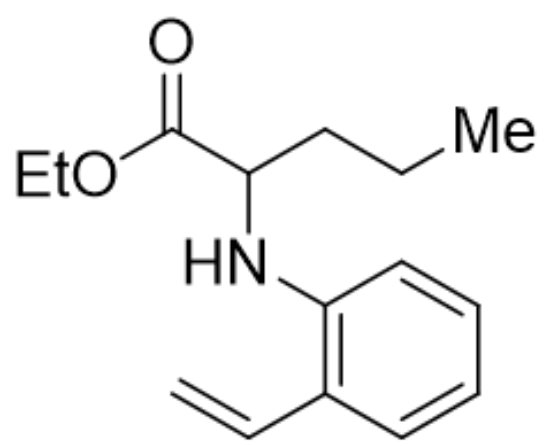

5k-Rac

Data File C:\CHEM32\1\DATA\SUN_12 2022-02-04 14-39-41\LZY-09-09-C.D
Sample Name:

===

Acq. Operator : Seq. Line : 5
Acq. Instrument : Instrument 1 Location : Vial 24
Injection Date : 2/4/2022 3:45:30 PM Inj : 1
Inj Volume : 5.000 µl
Different Inj Volume from Sequence ! Actual Inj Volume : 2.000 µl
Acq. Method : C:\CHEM32\1\DATA\SUN_12 2022-02-04 14-39-41\IC-02-35.M
Last changed : 7/12/2016 12:39:55 AM
Analysis Method : C:\CHEM32\1\METHODS\OD-03-60-0.6.M
Last changed : 3/8/2022 2:37:59 PM
(modified after loading)
Additional Info : Peak(s) manually integrated

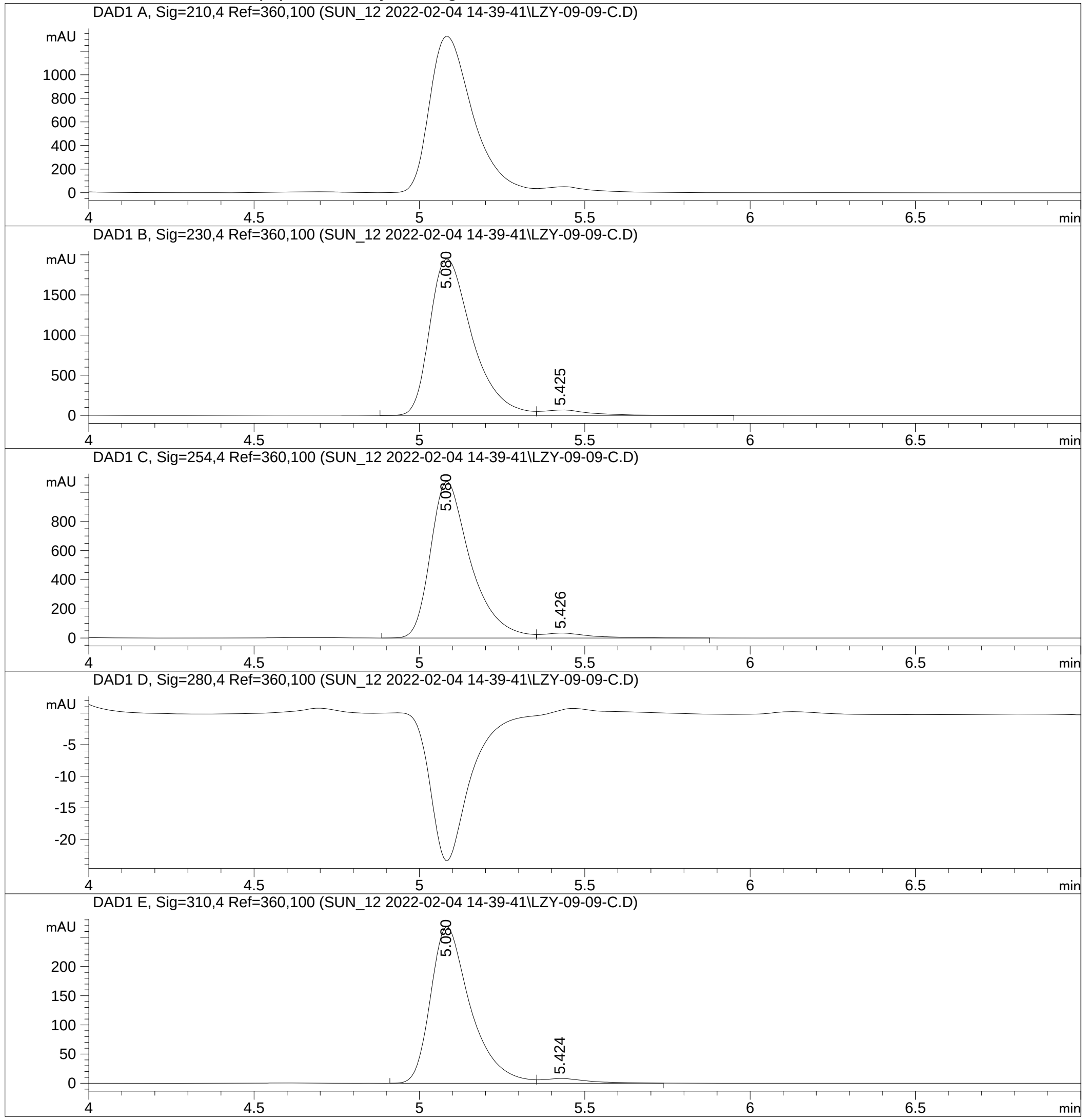

Data File C:\CHEM32\1\DATA\SUN_12 2022-02-04 14-39-41\LZY-09-09-C.D
Sample Name:

===

Area Percent Report

===

Sorted By : Signal
Multiplier : 1.0000
Dilution : 1.0000
Use Multiplier & Dilution Factor with ISTDs

Signal 1: DAD1 A, Sig=210,4 Ref=360,100

Signal 2: DAD1 B, Sig=230,4 Ref=360,100

Peak #	RetTime [min]	Type	Width [min]	Area [mAU*s]	Height [mAU]	Area %
1	5.080	VV	0.1361	1.74027e4	1950.52344	96.3261
2	5.425	VB	0.1400	663.75220	67.94395	3.6739

Totals : 1.80665e4 2018.46739

Signal 3: DAD1 C, Sig=254,4 Ref=360,100

Peak #	RetTime [min]	Type	Width [min]	Area [mAU*s]	Height [mAU]	Area %
1	5.080	VV	0.1261	9044.35352	1075.21448	96.3843
2	5.426	VB	0.1429	339.27957	33.86009	3.6157

Totals : 9383.63309 1109.07457

Signal 4: DAD1 D, Sig=280,4 Ref=360,100

Signal 5: DAD1 E, Sig=310,4 Ref=360,100

Peak #	RetTime [min]	Type	Width [min]	Area [mAU*s]	Height [mAU]	Area %
1	5.080	BV	0.1237	2247.31519	268.36661	96.6729
2	5.424	VB	0.1382	77.34428	8.04411	3.3271

Totals : 2324.65946 276.41072

===

*** End of Report ***

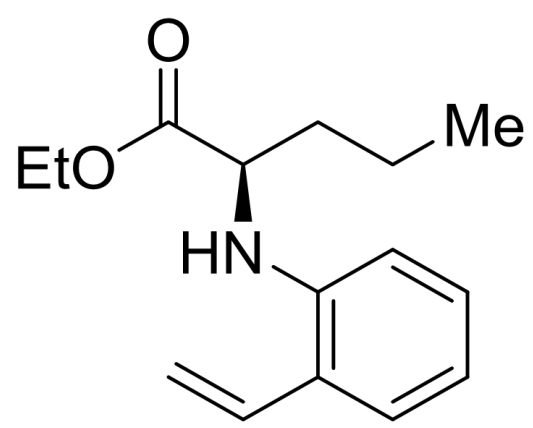

5k

```
=====================================================================
Acq. Operator   :                        Seq. Line :  23
Acq. Instrument : Instrument 1            Location : Vial 94
Injection Date  : 1/23/2022 1:14:02 AM         Inj :   1
                                        Inj Volume : 5.000 µl
Different Inj Volume from Sequence !   Actual Inj Volume : 7.000 µl
Acq. Method     : C:\CHEM32\1\DATA\SUN_12 2022-01-22 19-06-15\OD-03-30.M
Last changed    : 1/31/2015 11:39:09 AM
Analysis Method : C:\CHEM32\1\METHODS\OD-03-60-0.6.M
Last changed    : 3/8/2022 2:48:31 PM
                  (modified after loading)
Additional Info : Peak(s) manually integrated
```

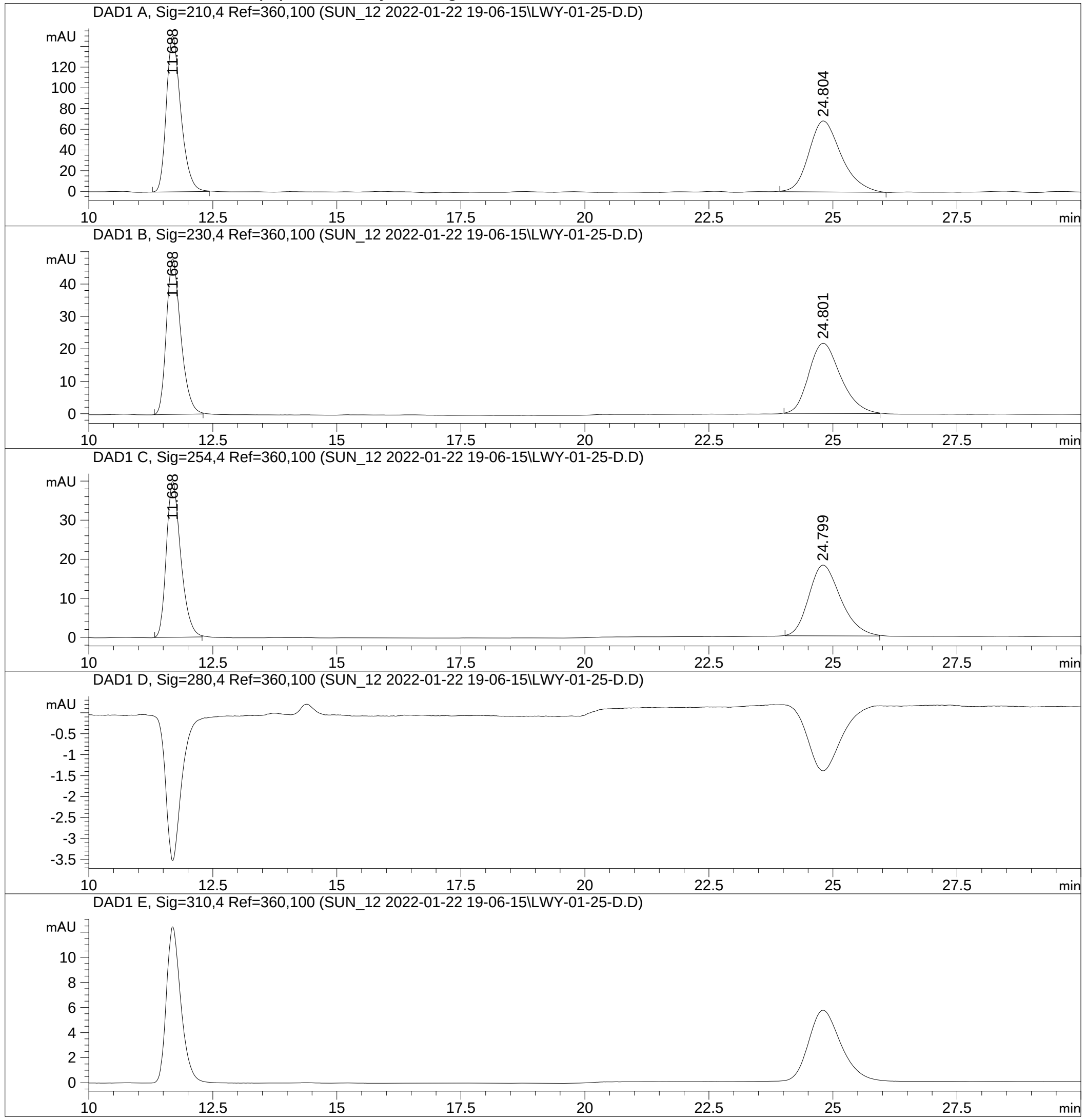


```
=====================================================================
                         Area Percent Report
=====================================================================

Sorted By             :      Signal
Multiplier            :      1.0000
Dilution              :      1.0000
Use Multiplier & Dilution Factor with ISTDs
```

Signal 1: DAD1 A, Sig=210,4 Ref=360,100

Peak #	RetTime [min]	Type	Width [min]	Area [mAU*s]	Height [mAU]	Area %
1	11.688	BB	0.3097	3057.06958	150.05870	49.5015
2	24.804	BB	0.6772	3118.64233	68.49899	50.4985

Totals : 6175.71191 218.55769

Signal 2: DAD1 B, Sig=230,4 Ref=360,100

Peak #	RetTime [min]	Type	Width [min]	Area [mAU*s]	Height [mAU]	Area %
1	11.688	BB	0.3080	970.19037	47.96761	50.0095
2	24.801	BB	0.6694	969.82318	21.70005	49.9905

Totals : 1940.01355 69.66766

Signal 3: DAD1 C, Sig=254,4 Ref=360,100

Peak #	RetTime [min]	Type	Width [min]	Area [mAU*s]	Height [mAU]	Area %
1	11.688	BB	0.3083	807.74207	39.88350	49.9254
2	24.799	BB	0.6820	810.15503	18.10564	50.0746

Totals : 1617.89709 57.98914

Signal 4: DAD1 D, Sig=280,4 Ref=360,100

Signal 5: DAD1 E, Sig=310,4 Ref=360,100

===
*** End of Report ***

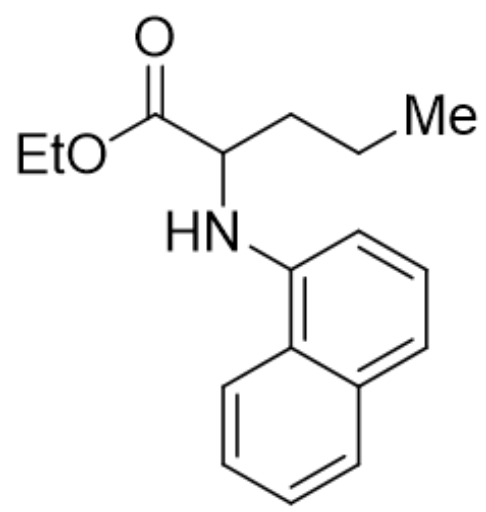

5l-Rac

Data File C:\CHEM32\1\DATA\SUN_12 2022-01-22 19-06-15\LZY-08-189-D.D
Sample Name:

```
=====================================================================
Acq. Operator   :                         Seq. Line :  11
Acq. Instrument : Instrument 1             Location : Vial 24
Injection Date  : 1/22/2022 9:47:49 PM          Inj :   1
                                         Inj Volume : 5.000 µl
Different Inj Volume from Sequence !     Actual Inj Volume : 4.000 µl
Acq. Method     : C:\CHEM32\1\DATA\SUN_12 2022-01-22 19-06-15\OD-03-30.M
Last changed    : 1/22/2022 9:46:59 PM
                  (modified after loading)
Analysis Method : C:\CHEM32\1\METHODS\OD-03-60-0.6.M
Last changed    : 3/8/2022 2:48:31 PM
                  (modified after loading)
Additional Info : Peak(s) manually integrated
```

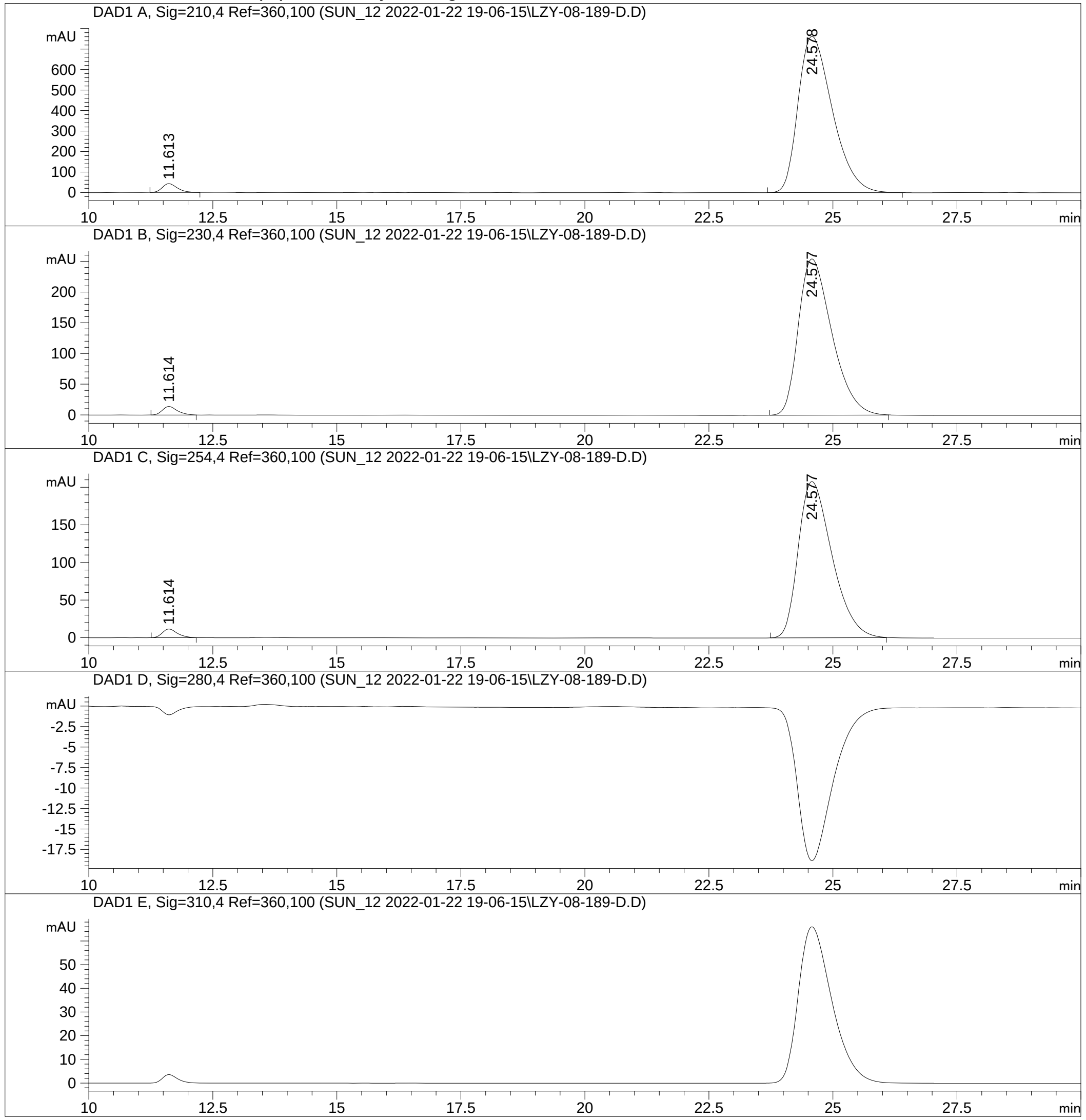

Data File C:\CHEM32\1\DATA\SUN_12 2022-01-22 19-06-15\LZY-08-189-D.D
Sample Name:

Area Percent Report

Sorted By : Signal
Multiplier : 1.0000
Dilution : 1.0000
Use Multiplier & Dilution Factor with ISTDs

Signal 1: DAD1 A, Sig=210,4 Ref=360,100

Peak #	RetTime [min]	Type	Width [min]	Area [mAU*s]	Height [mAU]	Area %
1	11.613	BB	0.3177	885.37653	42.72977	2.3632
2	24.578	BB	0.7417	3.65803e4	762.51758	97.6368

Totals : 3.74657e4 805.24735

Signal 2: DAD1 B, Sig=230,4 Ref=360,100

Peak #	RetTime [min]	Type	Width [min]	Area [mAU*s]	Height [mAU]	Area %
1	11.614	BB	0.3191	291.66940	13.88175	2.3832
2	24.577	BB	0.7220	1.19467e4	254.35556	97.6168

Totals : 1.22383e4 268.23731

Signal 3: DAD1 C, Sig=254,4 Ref=360,100

Peak #	RetTime [min]	Type	Width [min]	Area [mAU*s]	Height [mAU]	Area %
1	11.614	BB	0.3213	243.07344	11.56050	2.4145
2	24.577	BB	0.7293	9823.96777	207.90205	97.5855

Totals : 1.00670e4 219.46255

Signal 4: DAD1 D, Sig=280,4 Ref=360,100

Signal 5: DAD1 E, Sig=310,4 Ref=360,100

*** End of Report ***

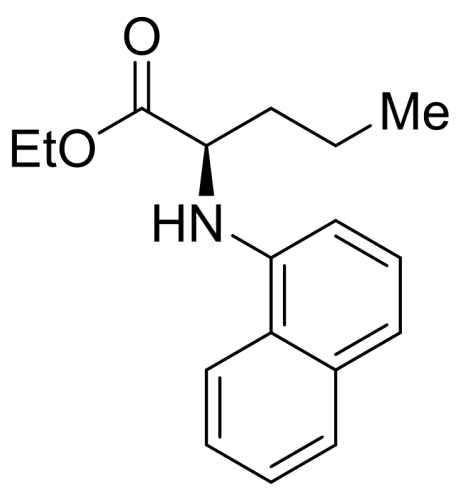

5l

Data File C:\CHEM32\1\DATA\SUN_12 2022-11-15 16-51-28\LZY-10-107-RAC.D
Sample Name:

```
=====================================================================
Acq. Operator   :                               Seq. Line :  28
Acq. Instrument : Instrument 1                   Location : Vial 94
Injection Date  : 11/16/2022 2:25:18 AM               Inj :   1
                                               Inj Volume : 5.000 µl
Different Inj Volume from Sequence !     Actual Inj Volume : 10.000 µl
Acq. Method     : C:\CHEM32\1\DATA\SUN_12 2022-11-15 16-51-28\AS-05-20.M
Last changed    : 11/16/2022 2:24:23 AM
                  (modified after loading)
Analysis Method : C:\CHEM32\1\METHODS\AY3-02-30.M
Last changed    : 11/16/2022 9:39:59 AM
                  (modified after loading)
Additional Info : Peak(s) manually integrated
```

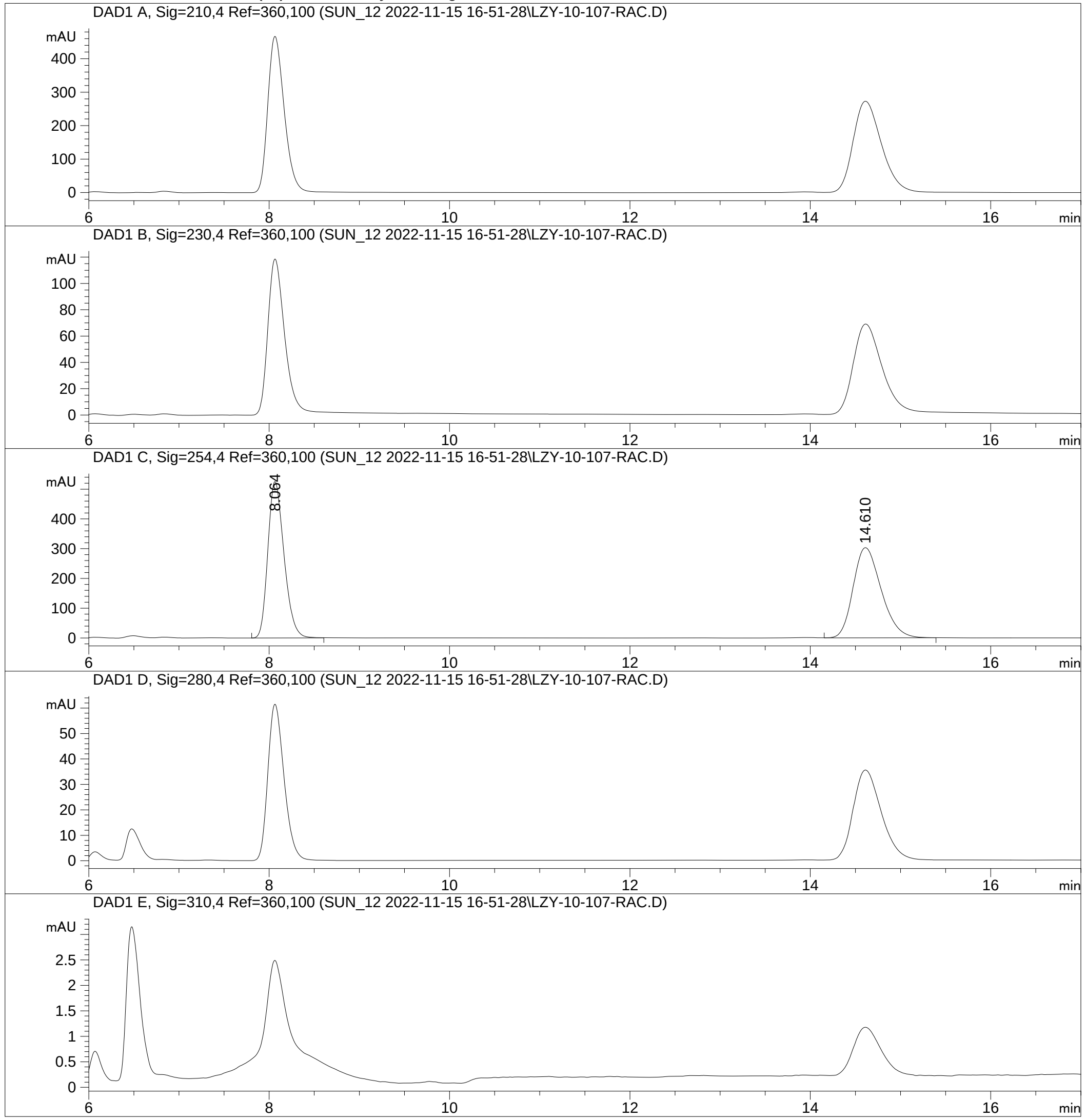

Data File C:\CHEM32\1\DATA\SUN_12 2022-11-15 16-51-28\LZY-10-107-RAC.D
Sample Name:

Area Percent Report

```
Sorted By             :      Signal
Multiplier            :      1.0000
Dilution              :      1.0000
Use Multiplier & Dilution Factor with ISTDs
```

Signal 1: DAD1 A, Sig=210,4 Ref=360,100

Signal 2: DAD1 B, Sig=230,4 Ref=360,100

Signal 3: DAD1 C, Sig=254,4 Ref=360,100

Peak #	RetTime [min]	Type	Width [min]	Area [mAU*s]	Height [mAU]	Area %
1	8.064	BB	0.1931	6537.95947	525.65533	49.9322
2	14.610	VB	0.3343	6555.71436	303.10608	50.0678

Totals : 1.30937e4 828.76141

Signal 4: DAD1 D, Sig=280,4 Ref=360,100

Signal 5: DAD1 E, Sig=310,4 Ref=360,100

*** End of Report ***

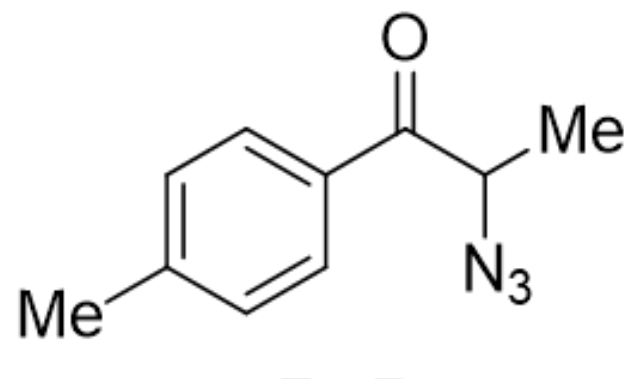

7a-Rac

===

Acq. Operator : Seq. Line : 2
Acq. Instrument : Instrument 1 Location : Vial 21
Injection Date : 7/10/2022 7:07:08 PM Inj : 1
Inj Volume : 5.000 µl
Different Inj Volume from Sequence ! Actual Inj Volume : 7.000 µl
Acq. Method : C:\CHEM32\1\DATA\SUN_12 2022-07-10 18-54-21\AS-05-20.M
Last changed : 7/10/2022 7:06:16 PM
(modified after loading)
Analysis Method : C:\CHEM32\1\METHODS\AY3-02-30.M
Last changed : 11/16/2022 9:39:59 AM
(modified after loading)
Additional Info : Peak(s) manually integrated

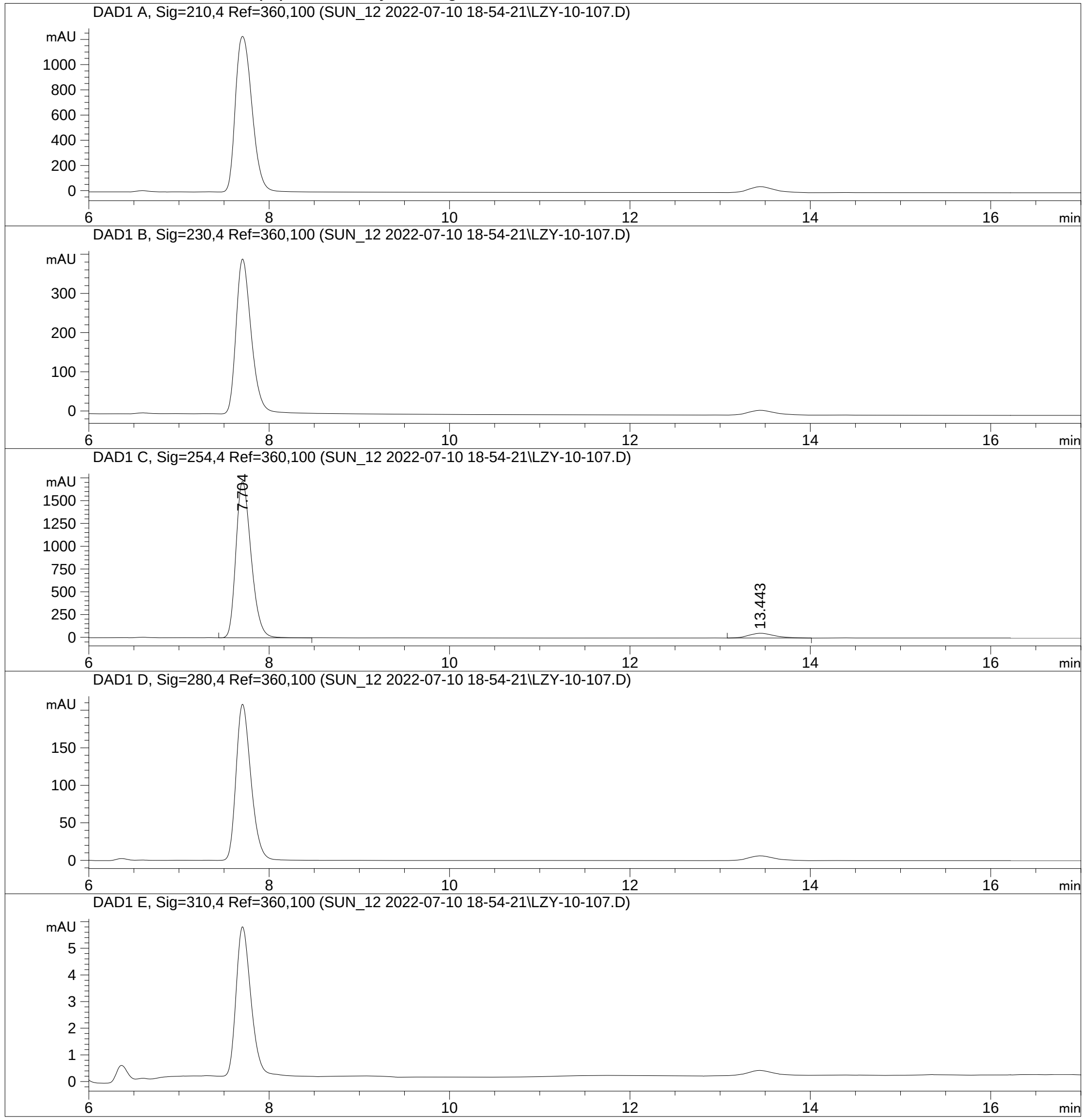

===

Area Percent Report

===

Sorted By : Signal
Multiplier : 1.0000
Dilution : 1.0000
Use Multiplier & Dilution Factor with ISTDs

Signal 1: DAD1 A, Sig=210,4 Ref=360,100

Signal 2: DAD1 B, Sig=230,4 Ref=360,100

Signal 3: DAD1 C, Sig=254,4 Ref=360,100

Peak #	RetTime [min]	Type	Width [min]	Area [mAU*s]	Height [mAU]	Area %
1	7.704	VB	0.1820	2.00028e4	1715.32593	95.2757
2	13.443	BB	0.2935	991.85284	52.23833	4.7243

Totals : 2.09947e4 1767.56425

Signal 4: DAD1 D, Sig=280,4 Ref=360,100

Signal 5: DAD1 E, Sig=310,4 Ref=360,100

===

*** End of Report ***

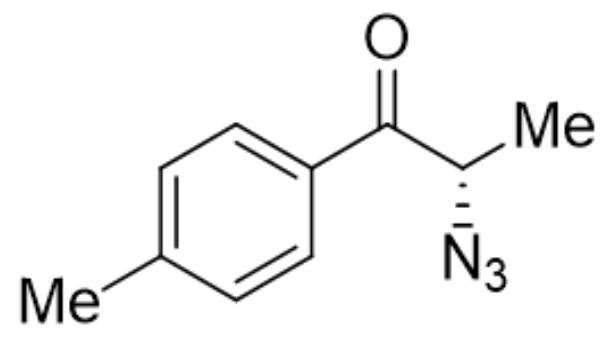

7a

===

Acq. Operator :   Seq. Line : 19
Acq. Instrument : Instrument 1   Location : Vial 28
Injection Date : 10/7/2022 5:07:56 PM   Inj : 1
                                                  Inj Volume : 5.000 µl
Different Inj Volume from Sequence !   Actual Inj Volume : 9.000 µl
Acq. Method : C:\CHEM32\1\DATA\SUN_12 2022-10-07 11-09-05\AS-05-20.M
Last changed : 10/7/2022 3:18:22 PM
(modified after loading)
Analysis Method : C:\CHEM32\1\DATA\SUN_12 2023-07-13 22-16-37\OD-03-60.M
Last changed : 8/30/2023 3:20:15 PM
(modified after loading)
Additional Info : Peak(s) manually integrated

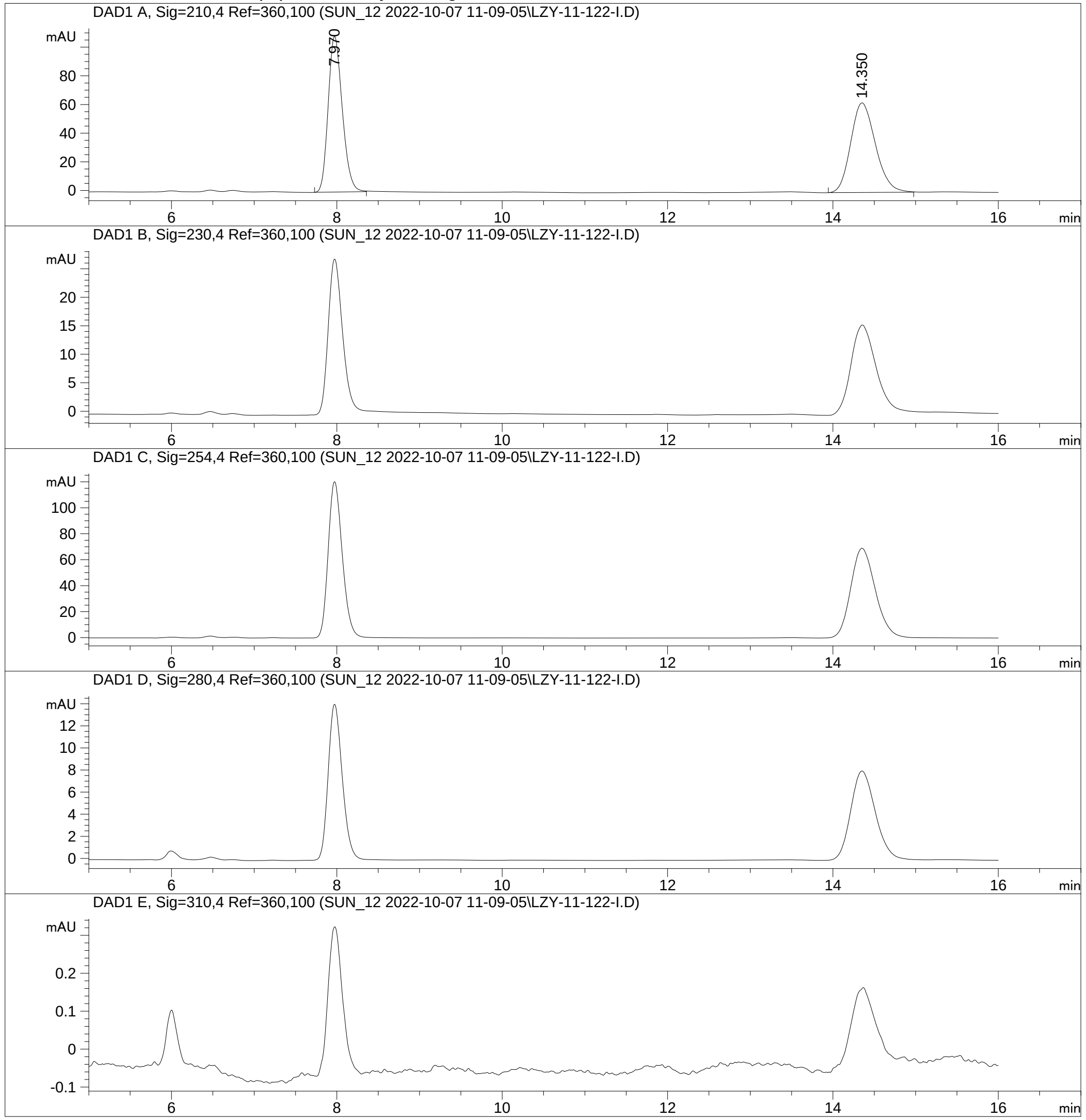

Data File C:\CHEM32\1\DATA\SUN_12 2022-10-07 11-09-05\LZY-11-122-I.D
Sample Name:

===

Area Percent Report

===

Sorted By : Signal
Multiplier : 1.0000
Dilution : 1.0000
Use Multiplier & Dilution Factor with ISTDs

Signal 1: DAD1 A, Sig=210,4 Ref=360,100

Peak #	RetTime [min]	Type	Width [min]	Area [mAU*s]	Height [mAU]	Area %
1	7.970	BB	0.1841	1285.54150	108.58537	49.7358
2	14.350	BB	0.3204	1299.19800	62.51939	50.2642

Totals : 2584.73950 171.10476

Signal 2: DAD1 B, Sig=230,4 Ref=360,100

Signal 3: DAD1 C, Sig=254,4 Ref=360,100

Signal 4: DAD1 D, Sig=280,4 Ref=360,100

Signal 5: DAD1 E, Sig=310,4 Ref=360,100

===

*** End of Report ***

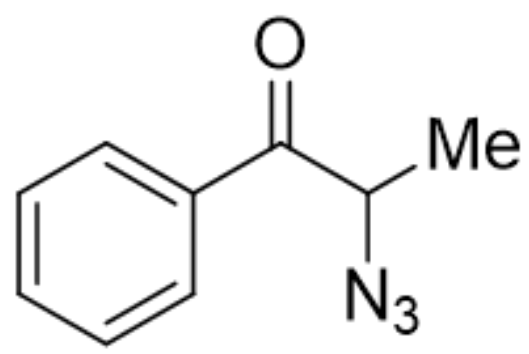

7b-Rac

```
=====================================================================
Acq. Operator   :                               Seq. Line :   4
Acq. Instrument : Instrument 1                   Location : Vial 21
Injection Date  : 10/7/2022 12:33:29 PM               Inj :   1
                                               Inj Volume : 5.000 µl
Different Inj Volume from Sequence !     Actual Inj Volume : 6.000 µl
Acq. Method     : C:\CHEM32\1\DATA\SUN_12 2022-10-07 11-09-05\AS-05-20.M
Last changed    : 10/7/2022 12:32:37 PM
                  (modified after loading)
Analysis Method : C:\CHEM32\1\DATA\SUN_12 2023-07-13 22-16-37\OD-03-60.M
Last changed    : 8/30/2023 3:10:26 PM
                  (modified after loading)
Additional Info : Peak(s) manually integrated
```

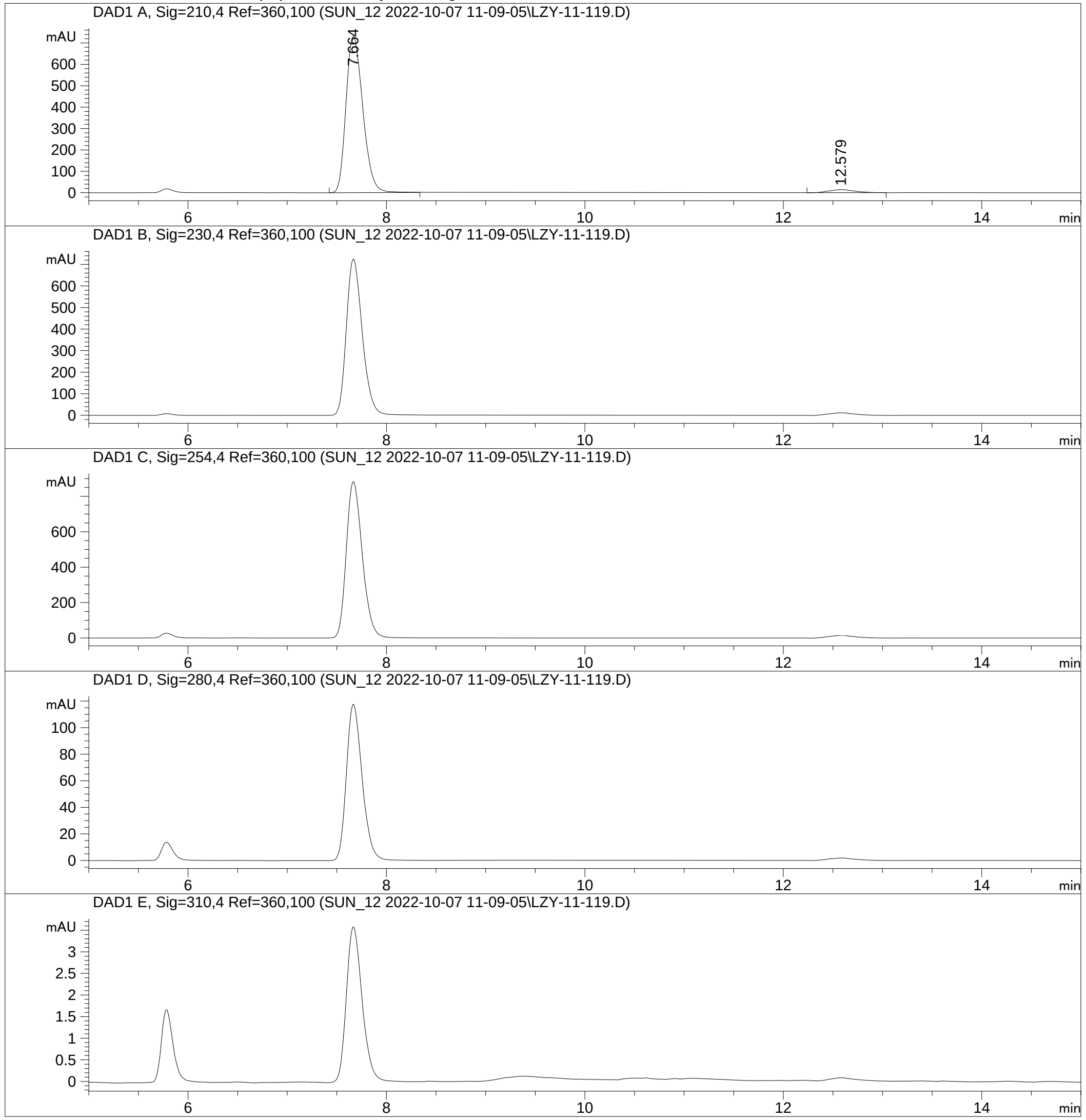

Data File C:\CHEM32\1\DATA\SUN_12 2022-10-07 11-09-05\LZY-11-119.D
Sample Name:

```
=====================================================================
                         Area Percent Report
=====================================================================

Sorted By             :      Signal
Multiplier            :      1.0000
Dilution              :      1.0000
Use Multiplier & Dilution Factor with ISTDs


Signal 1: DAD1 A, Sig=210,4 Ref=360,100
```

Peak #	RetTime [min]	Type	Width [min]	Area [mAU*s]	Height [mAU]	Area %
1	7.664	BB	0.1855	8617.97070	731.04242	97.2042
2	12.579	BB	0.2616	247.87520	14.48575	2.7958

```
Totals :                  8865.84590  745.52817



Signal 2: DAD1 B, Sig=230,4 Ref=360,100


Signal 3: DAD1 C, Sig=254,4 Ref=360,100


Signal 4: DAD1 D, Sig=280,4 Ref=360,100


Signal 5: DAD1 E, Sig=310,4 Ref=360,100

=====================================================================
                          *** End of Report ***
```

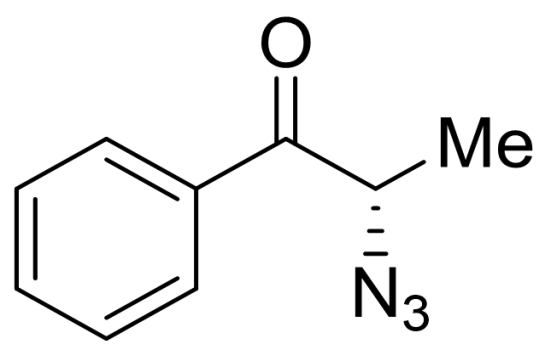

7b

===

```
Acq. Operator   :                               Seq. Line :   8
Acq. Instrument : Instrument 1                   Location : Vial 25
Injection Date  : 10/15/2022 9:33:18 PM               Inj :   1
                                               Inj Volume : 5.000 µl
Different Inj Volume from Sequence !     Actual Inj Volume : 9.000 µl
Acq. Method     : C:\CHEM32\1\DATA\SUN_12 2022-10-15 18-53-13\AS-05-20.M
Last changed    : 10/15/2022 9:54:04 PM
                  (modified after loading)
Analysis Method : C:\CHEM32\1\METHODS\AY3-02-30.M
Last changed    : 11/11/2022 5:04:37 PM
                  (modified after loading)
Additional Info : Peak(s) manually integrated
```

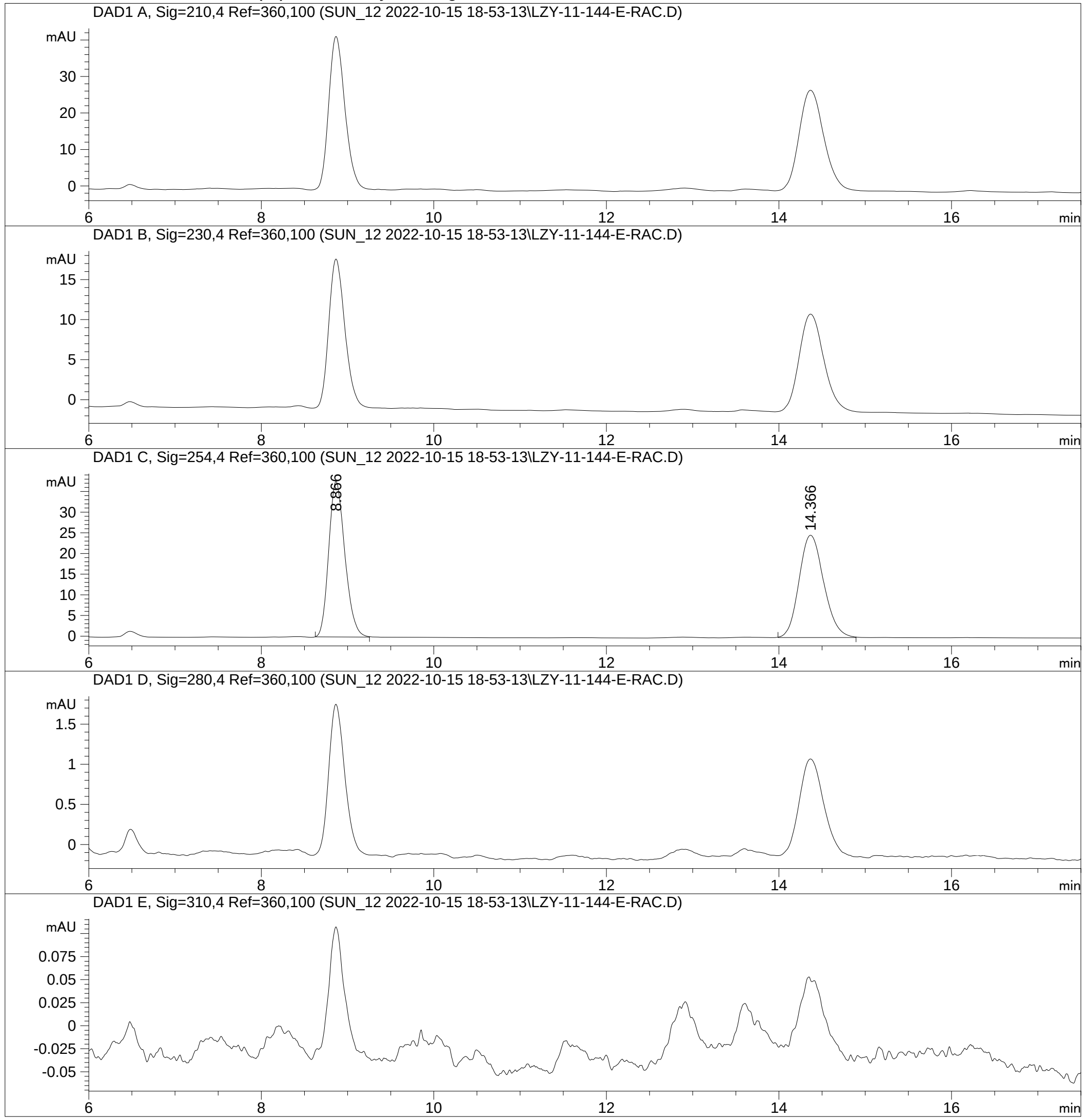

Data File C:\CHEM32\1\DATA\SUN_12 2022-10-15 18-53-13\LZY-11-144-E-RAC.D
Sample Name:

===

Area Percent Report

===

```
Sorted By            :      Signal
Multiplier           :      1.0000
Dilution             :      1.0000
Use Multiplier & Dilution Factor with ISTDs
```

Signal 1: DAD1 A, Sig=210,4 Ref=360,100

Signal 2: DAD1 B, Sig=230,4 Ref=360,100

Signal 3: DAD1 C, Sig=254,4 Ref=360,100

Peak #	RetTime [min]	Type	Width [min]	Area [mAU*s]	Height [mAU]	Area %
1	8.866	BB	0.2018	489.25656	37.59777	49.9383
2	14.366	BB	0.3088	490.46530	24.79248	50.0617
Totals :				979.72186	62.39025	

Signal 4: DAD1 D, Sig=280,4 Ref=360,100

Signal 5: DAD1 E, Sig=310,4 Ref=360,100

===

*** End of Report ***

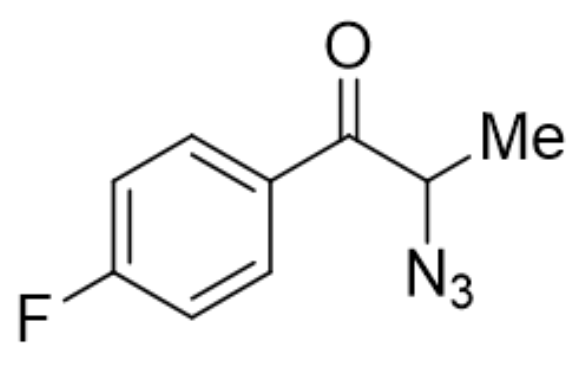

7c-Rac

Data File C:\CHEM32\1\DATA\SUN_12 2022-10-19 21-48-43\LZY-11-149.D
Sample Name:

===

Acq. Operator : Seq. Line : 2
Acq. Instrument : Instrument 1 Location : Vial 21
Injection Date : 10/19/2022 10:00:45 PM Inj : 1
Inj Volume : 3.000 µl
Different Inj Volume from Sequence ! Actual Inj Volume : 6.000 µl
Acq. Method : C:\CHEM32\1\DATA\SUN_12 2022-10-19 21-48-43\AS-05-30.M
Last changed : 10/19/2022 9:59:53 PM
(modified after loading)
Analysis Method : C:\CHEM32\1\METHODS\AY3-02-30.M
Last changed : 11/11/2022 5:04:37 PM
(modified after loading)
Additional Info : Peak(s) manually integrated

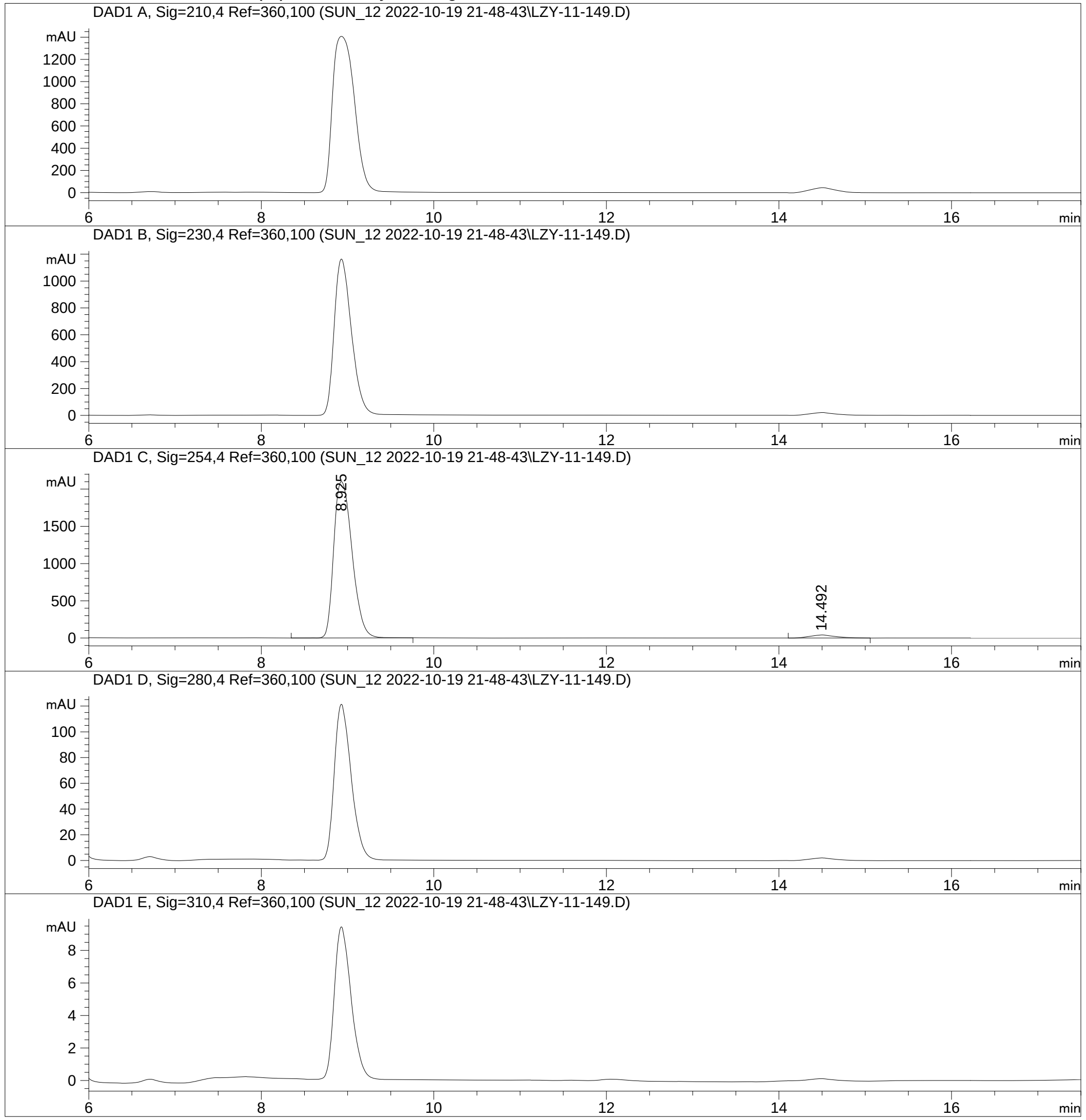

Data File C:\CHEM32\1\DATA\SUN_12 2022-10-19 21-48-43\LZY-11-149.D
Sample Name:

===
Area Percent Report
===

Sorted By : Signal
Multiplier : 1.0000
Dilution : 1.0000
Use Multiplier & Dilution Factor with ISTDs

Signal 1: DAD1 A, Sig=210,4 Ref=360,100

Signal 2: DAD1 B, Sig=230,4 Ref=360,100

Signal 3: DAD1 C, Sig=254,4 Ref=360,100

Peak #	RetTime [min]	Type	Width [min]	Area [mAU*s]	Height [mAU]	Area %
1	8.925	VB	0.2321	3.09900e4	2100.75342	97.3822
2	14.492	BB	0.3119	833.06128	41.55070	2.6178

Totals : 3.18231e4 2142.30412

Signal 4: DAD1 D, Sig=280,4 Ref=360,100

Signal 5: DAD1 E, Sig=310,4 Ref=360,100

===
*** End of Report ***

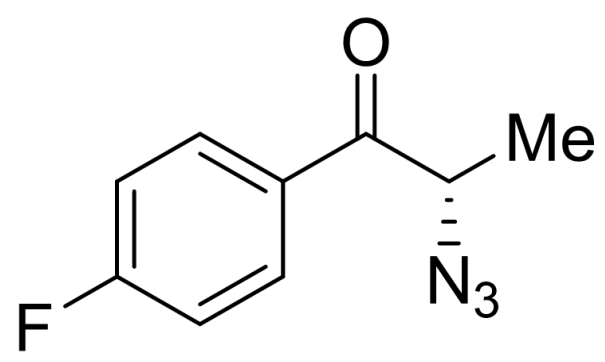

7c

===

Acq. Operator : Seq. Line : 2
Acq. Instrument : Instrument 1 Location : Vial 21
Injection Date : 11/16/2022 10:08:48 AM Inj : 1
Inj Volume : 5.000 µl
Different Inj Volume from Sequence ! Actual Inj Volume : 10.000 µl
Acq. Method : C:\CHEM32\1\DATA\SUN_12 2022-11-16 09-55-30\AS-05-20.M
Last changed : 11/16/2022 10:07:53 AM
(modified after loading)
Analysis Method : C:\CHEM32\1\METHODS\AY3-02-30.M
Last changed : 11/16/2022 11:01:14 AM
(modified after loading)
Additional Info : Peak(s) manually integrated

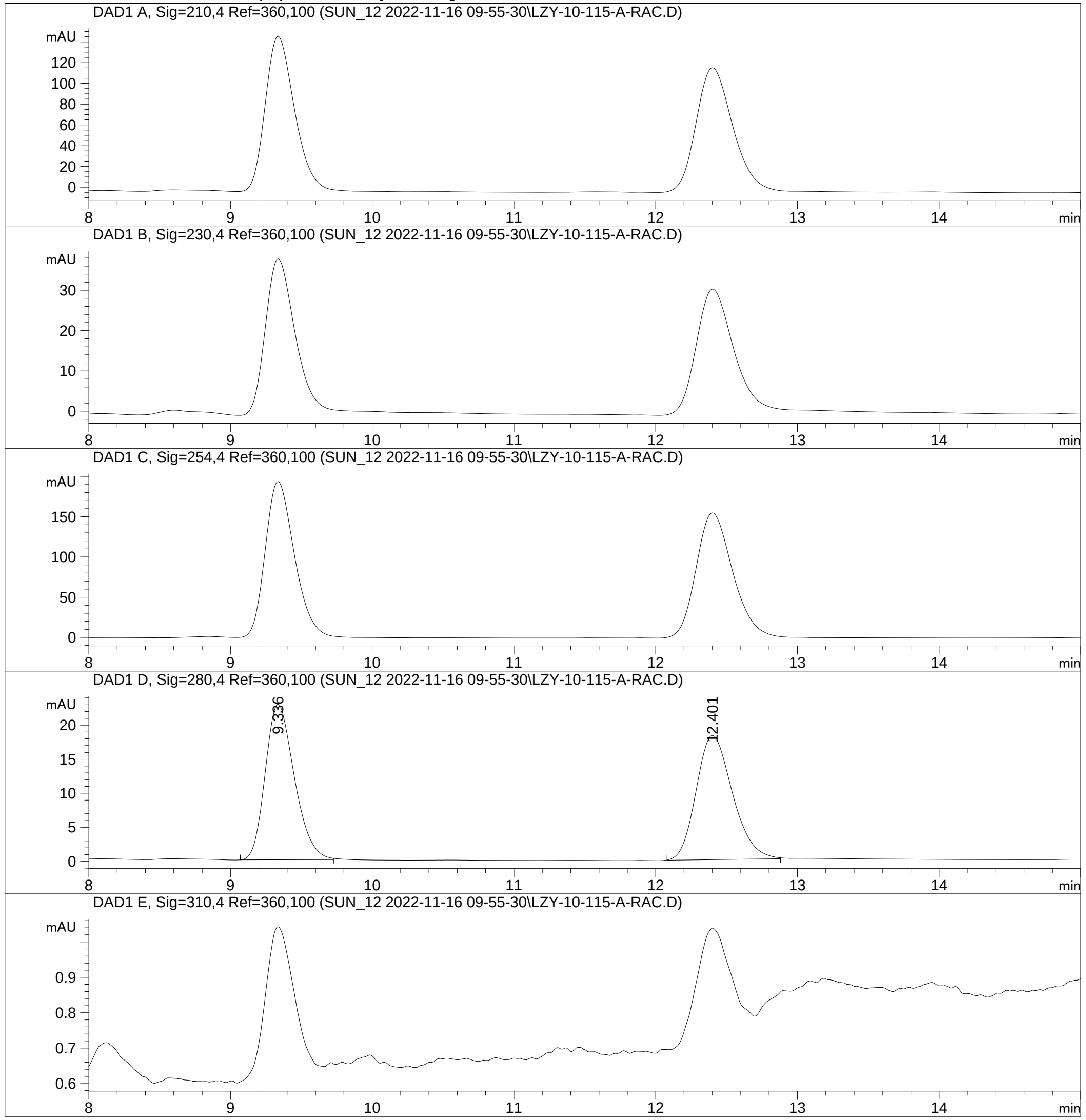

===
Area Percent Report
===

Sorted By : Signal
Multiplier : 1.0000
Dilution : 1.0000
Use Multiplier & Dilution Factor with ISTDs

Signal 1: DAD1 A, Sig=210,4 Ref=360,100

Signal 2: DAD1 B, Sig=230,4 Ref=360,100

Signal 3: DAD1 C, Sig=254,4 Ref=360,100

Signal 4: DAD1 D, Sig=280,4 Ref=360,100

Peak #	RetTime [min]	Type	Width [min]	Area [mAU*s]	Height [mAU]	Area %
1	9.336	BB	0.2201	325.32465	22.84958	50.0938
2	12.401	BB	0.2733	324.10641	18.23819	49.9062

Totals : 649.43106 41.08777

Signal 5: DAD1 E, Sig=310,4 Ref=360,100

===
*** End of Report ***

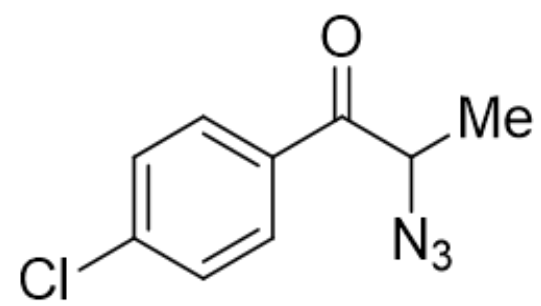

7d-Rac

```
Data File C:\CHEM32\1\DATA\SUN_12 2022-07-13 23-28-16\LZY-10-115-A.D
Sample Name:

    =====================================================================
    Acq. Operator   :                                 Seq. Line :   2
    Acq. Instrument : Instrument 1                     Location : Vial 21
    Injection Date  : 7/13/2022 11:41:07 PM                 Inj :   1
                                                    Inj Volume : 5.000 µl
    Different Inj Volume from Sequence !     Actual Inj Volume : 6.000 µl
    Acq. Method     : C:\CHEM32\1\DATA\SUN_12 2022-07-13 23-28-16\AS-05-20.M
    Last changed    : 7/13/2022 11:40:14 PM
                      (modified after loading)
    Analysis Method : C:\CHEM32\1\METHODS\AY3-02-30.M
    Last changed    : 11/16/2022 11:01:14 AM
                      (modified after loading)
    Additional Info : Peak(s) manually integrated
```

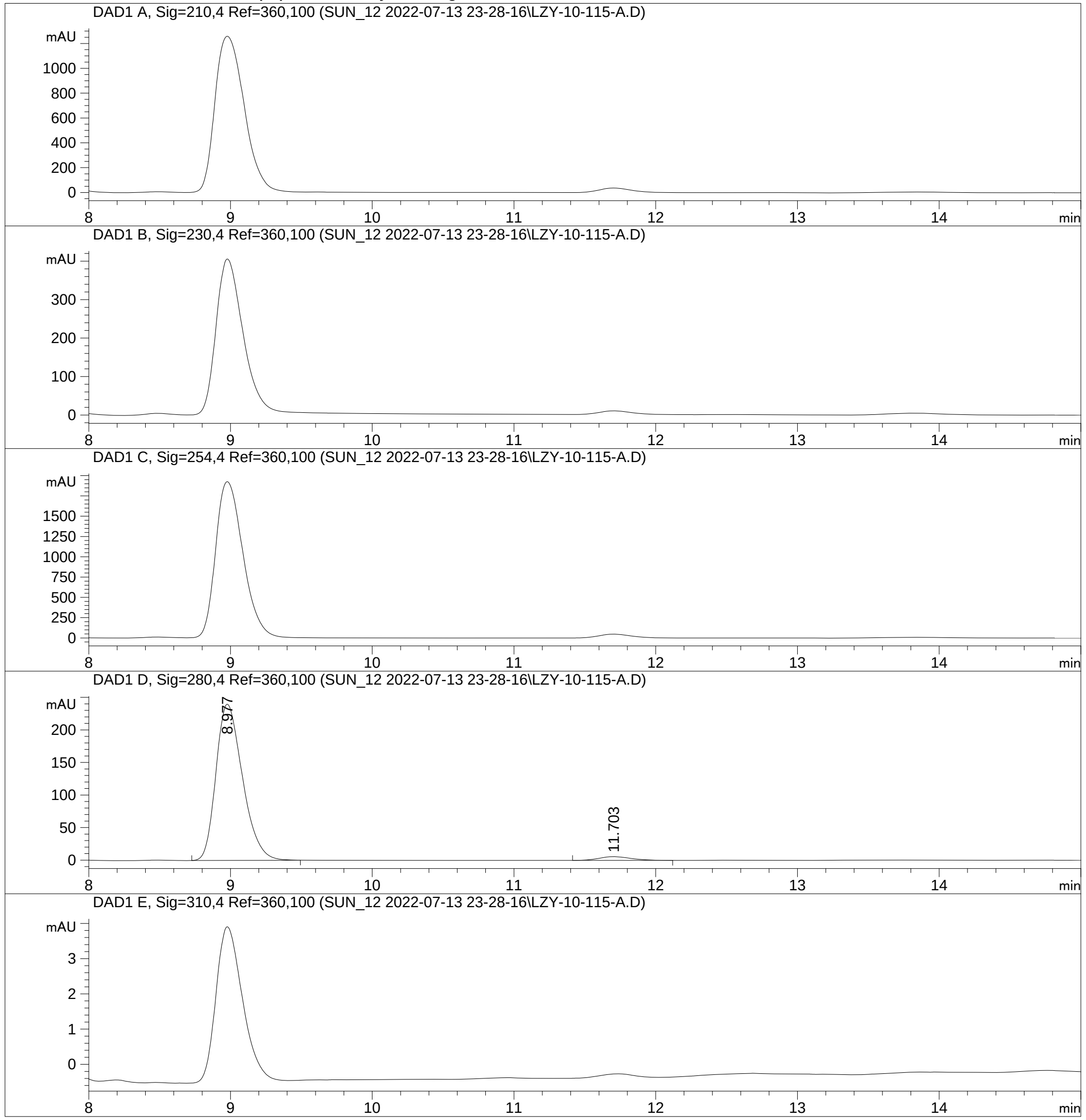

Data File C:\CHEM32\1\DATA\SUN_12 2022-07-13 23-28-16\LZY-10-115-A.D
Sample Name:

```
    =====================================================================
                             Area Percent Report
    =====================================================================

    Sorted By             :      Signal
    Multiplier            :      1.0000
    Dilution              :      1.0000
    Use Multiplier & Dilution Factor with ISTDs


    Signal 1: DAD1 A, Sig=210,4 Ref=360,100


    Signal 2: DAD1 B, Sig=230,4 Ref=360,100


    Signal 3: DAD1 C, Sig=254,4 Ref=360,100


    Signal 4: DAD1 D, Sig=280,4 Ref=360,100

    Peak RetTime Type  Width     Area      Height     Area
      #   [min]        [min]   [mAU*s]     [mAU]        %
    ----|-------|----|-------|----------|----------|--------|
       1   8.977 BB    0.2018 3123.21509  240.08701  97.2260
       2  11.703 BB    0.2427   89.10940    5.69086   2.7740

    Totals :                  3212.32449  245.77786


    Signal 5: DAD1 E, Sig=310,4 Ref=360,100

    =====================================================================
                              *** End of Report ***
```

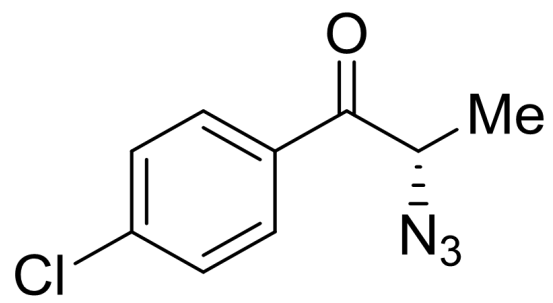

7d

```
=====================================================================
Acq. Operator   :                               Seq. Line :   8
Acq. Instrument : Instrument 1                   Location : Vial 23
Injection Date  : 9/7/2022 12:31:27 PM                Inj :   1
                                               Inj Volume : 5.000 µl
Different Inj Volume from Sequence !     Actual Inj Volume : 9.000 µl
Acq. Method     : C:\CHEM32\1\DATA\SUN_12 2022-09-07 10-50-26\AS-05-20.M
Last changed    : 9/7/2022 12:30:34 PM
                  (modified after loading)
Analysis Method : C:\CHEM32\1\METHODS\AY3-02-30.M
Last changed    : 11/14/2022 10:41:33 PM
                  (modified after loading)
Additional Info : Peak(s) manually integrated
```

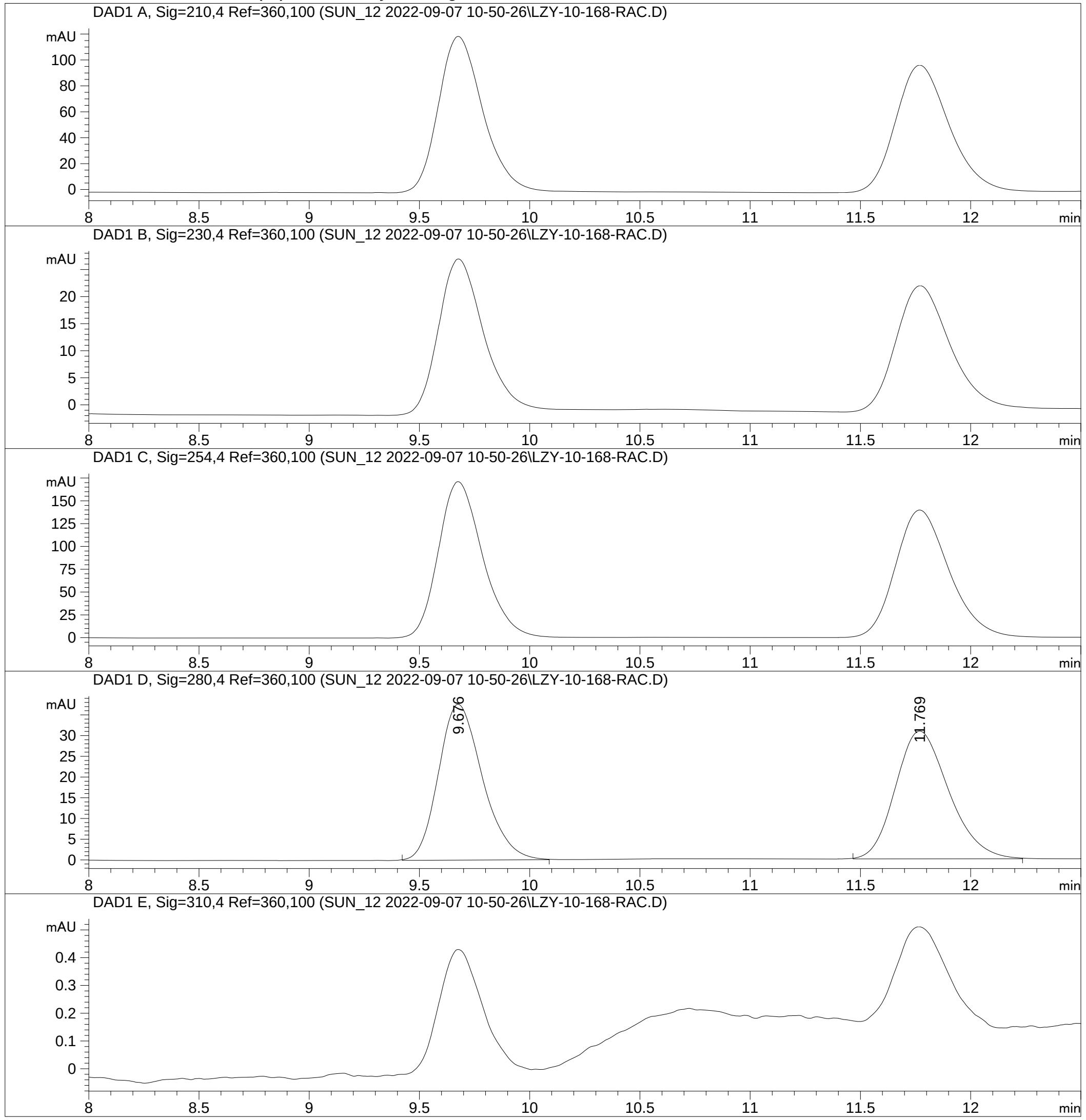

Data File C:\CHEM32\1\DATA\SUN_12 2022-09-07 10-50-26\LZY-10-168-RAC.D
Sample Name:

```
=====================================================================
                         Area Percent Report
=====================================================================

Sorted By             :      Signal
Multiplier            :      1.0000
Dilution              :      1.0000
Use Multiplier & Dilution Factor with ISTDs


Signal 1: DAD1 A, Sig=210,4 Ref=360,100


Signal 2: DAD1 B, Sig=230,4 Ref=360,100


Signal 3: DAD1 C, Sig=254,4 Ref=360,100


Signal 4: DAD1 D, Sig=280,4 Ref=360,100
```

Peak #	RetTime [min]	Type	Width [min]	Area [mAU*s]	Height [mAU]	Area %
1	9.676	BB	0.2138	521.98895	37.63641	50.1641
2	11.769	BB	0.2583	518.57452	30.82806	49.8359

```
Totals :                  1040.56348   68.46448



Signal 5: DAD1 E, Sig=310,4 Ref=360,100

=====================================================================
                          *** End of Report ***
```

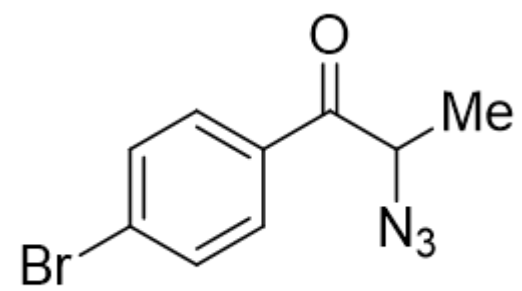

7e-Rac

===

```
Acq. Operator   :                             Seq. Line :   2
Acq. Instrument : Instrument 1                 Location : Vial 21
Injection Date  : 8/7/2022 9:23:36 PM               Inj :   1
                                             Inj Volume : 5.000 µl
Different Inj Volume from Sequence !     Actual Inj Volume : 3.000 µl
Acq. Method     : C:\CHEM32\1\DATA\SUN_12 2022-08-07 21-10-29\AS-05-20.M
Last changed    : 8/7/2022 9:22:25 PM
                  (modified after loading)
Analysis Method : C:\CHEM32\1\METHODS\AY3-02-30.M
Last changed    : 11/14/2022 10:41:33 PM
                  (modified after loading)
Additional Info : Peak(s) manually integrated
```

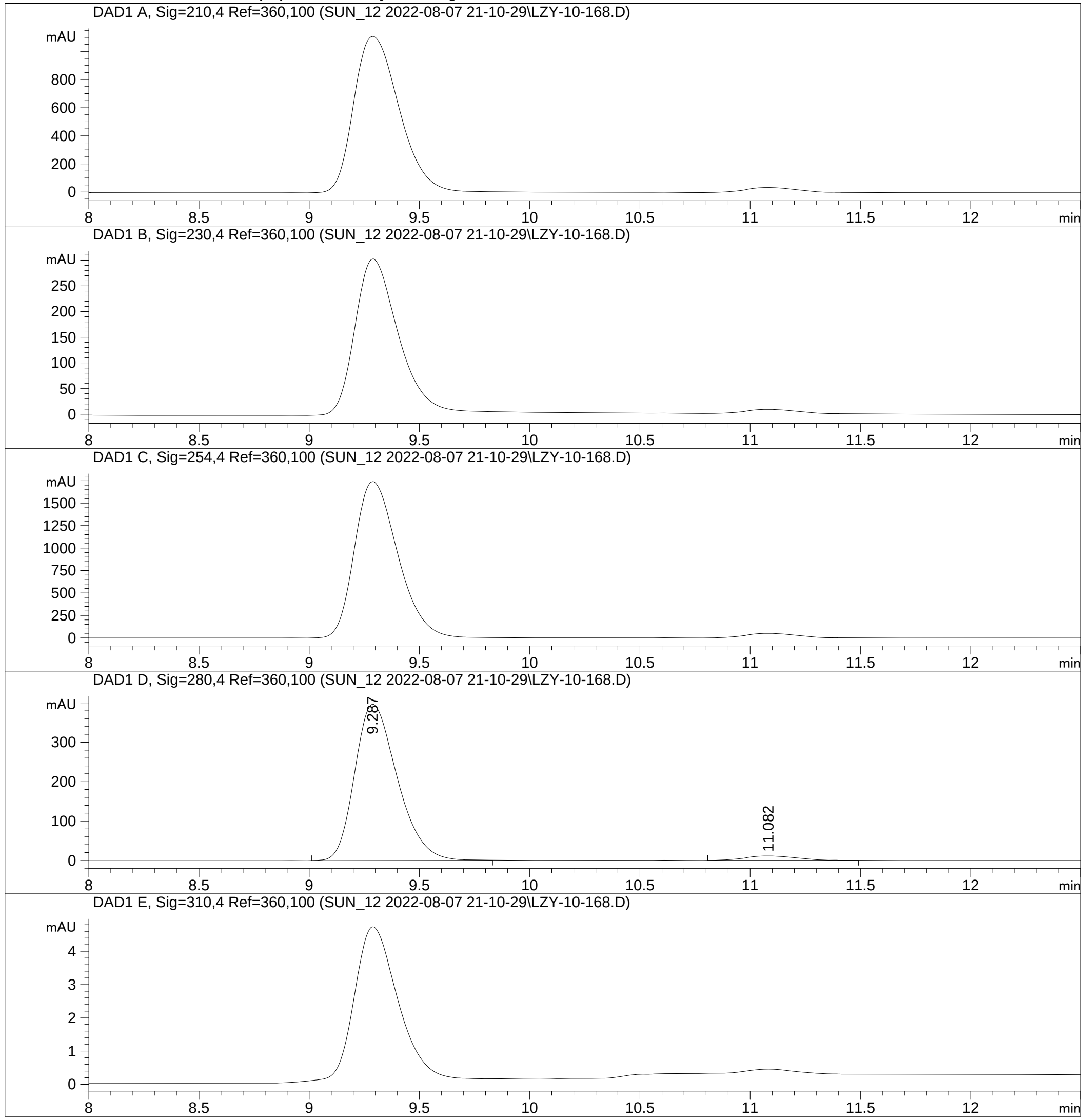

===
Area Percent Report
===

```
Sorted By             :      Signal
Multiplier            :      1.0000
Dilution              :      1.0000
Use Multiplier & Dilution Factor with ISTDs
```

Signal 1: DAD1 A, Sig=210,4 Ref=360,100

Signal 2: DAD1 B, Sig=230,4 Ref=360,100

Signal 3: DAD1 C, Sig=254,4 Ref=360,100

Signal 4: DAD1 D, Sig=280,4 Ref=360,100

Peak #	RetTime [min]	Type	Width [min]	Area [mAU*s]	Height [mAU]	Area %
1	9.287	BB	0.2083	5317.56055	397.00543	96.6706
2	11.082	BB	0.2380	183.13939	11.73809	3.3294

Totals :   5500.69994 408.74352

Signal 5: DAD1 E, Sig=310,4 Ref=360,100

===
*** End of Report ***

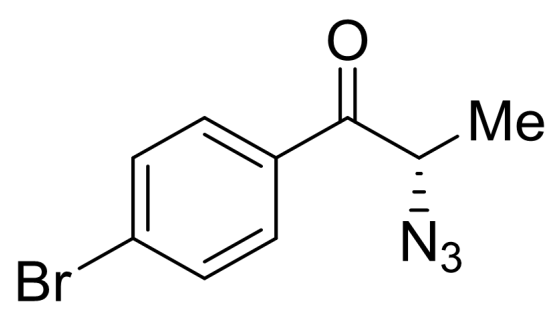

7e

```
=====================================================================
Acq. Operator   :                               Seq. Line :   5
Acq. Instrument : Instrument 1                   Location : Vial 22
Injection Date  : 10/3/2022 8:45:13 PM                Inj :   1
                                               Inj Volume : 5.000 µl
Different Inj Volume from Sequence !     Actual Inj Volume : 9.000 µl
Acq. Method     : C:\CHEM32\1\DATA\SUN_12 2022-10-03 19-45-33\IC-03-20.M
Last changed    : 10/3/2022 8:44:22 PM
                  (modified after loading)
Analysis Method : C:\CHEM32\1\METHODS\AY3-02-30.M
Last changed    : 11/14/2022 10:13:09 PM
                  (modified after loading)
Additional Info : Peak(s) manually integrated
```

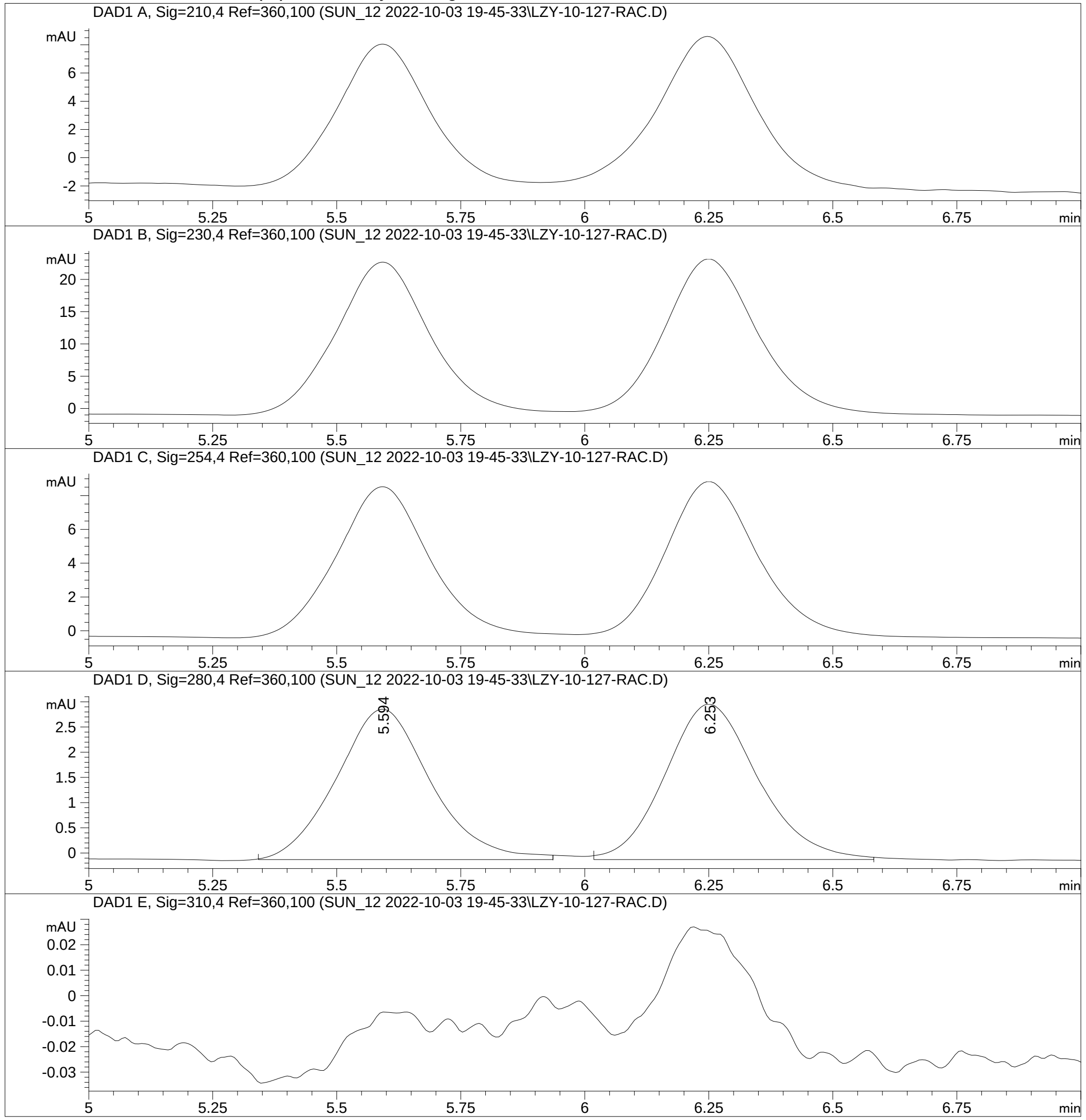


```
=====================================================================
                          Area Percent Report
=====================================================================

Sorted By             :      Signal
Multiplier            :      1.0000
Dilution              :      1.0000
Use Multiplier & Dilution Factor with ISTDs


Signal 1: DAD1 A, Sig=210,4 Ref=360,100


Signal 2: DAD1 B, Sig=230,4 Ref=360,100


Signal 3: DAD1 C, Sig=254,4 Ref=360,100


Signal 4: DAD1 D, Sig=280,4 Ref=360,100

Peak RetTime Type  Width     Area      Height     Area
  #   [min]        [min]   [mAU*s]     [mAU]        %
----|-------|----|-------|----------|----------|--------|
   1   5.594 BB    0.1968   39.59787    2.98438  49.6395
   2   6.253 BB    0.1960   40.17304    3.08412  50.3605

Totals :                    79.77091    6.06851



Signal 5: DAD1 E, Sig=310,4 Ref=360,100

=====================================================================
                           *** End of Report ***
```

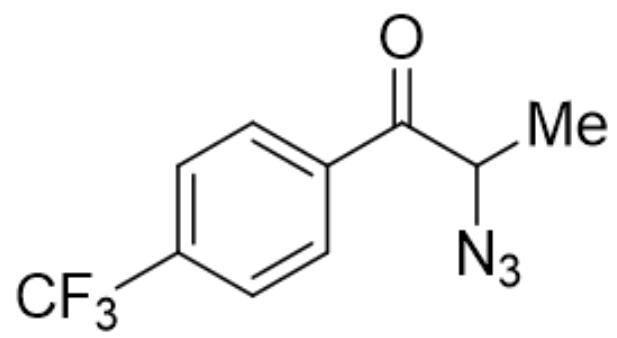

7f-Rac

```
=====================================================================
Acq. Operator   :                                Seq. Line :   2
Acq. Instrument : Instrument 1                    Location : Vial 21
Injection Date  : 11/22/2022 8:39:28 PM                Inj :   1
                                                Inj Volume : 5.000 µl
Different Inj Volume from Sequence !     Actual Inj Volume : 6.000 µl
Acq. Method     : C:\CHEM32\1\DATA\SUN_12 2022-11-22 20-26-32\IC-03-10.M
Last changed    : 6/27/2018 12:21:49 AM
Analysis Method : C:\CHEM32\1\METHODS\AY3-02-30.M
Last changed    : 11/22/2022 10:02:20 PM
                  (modified after loading)
Additional Info : Peak(s) manually integrated
```

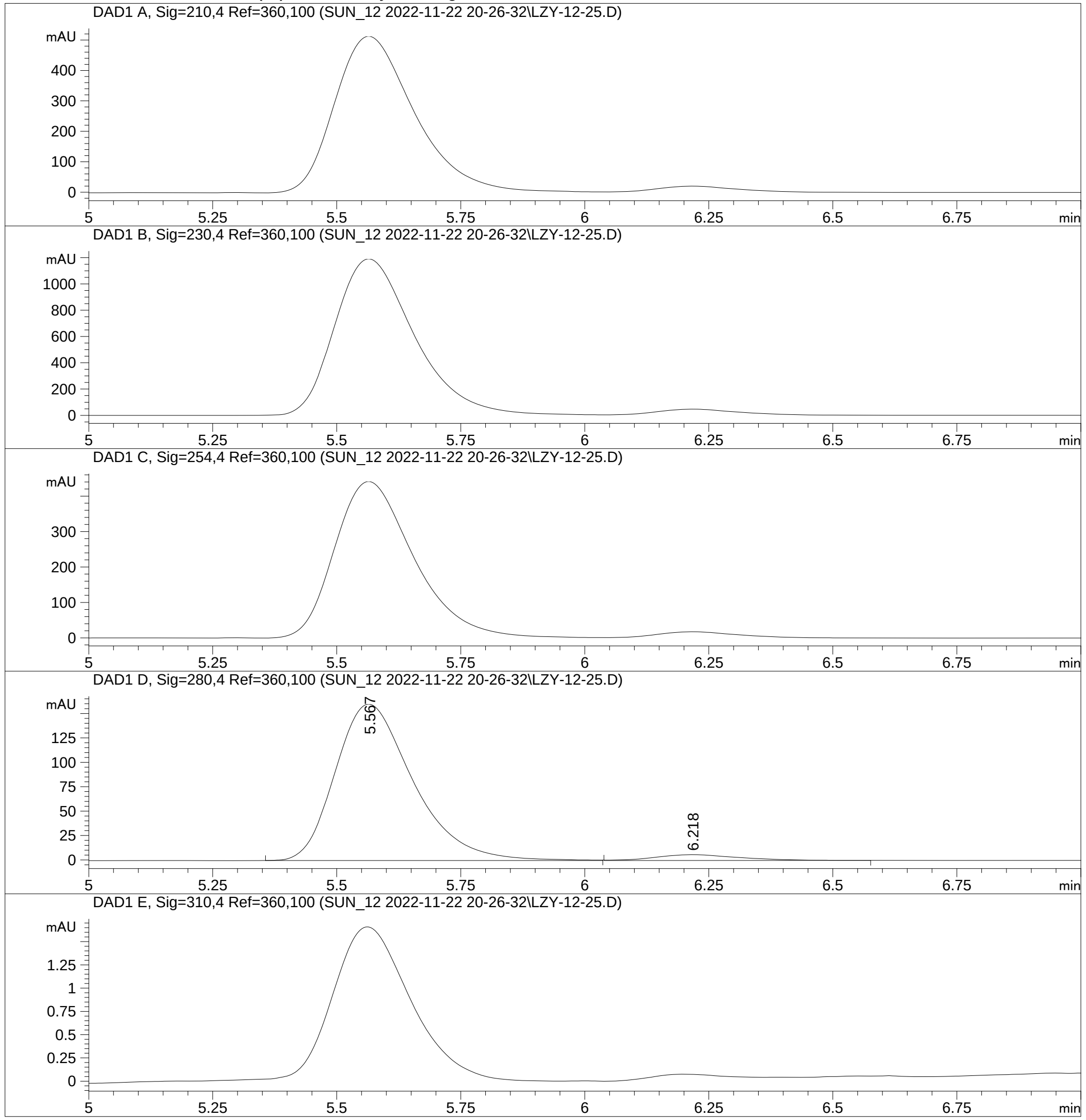


```
=====================================================================
                         Area Percent Report
=====================================================================

Sorted By             :      Signal
Multiplier            :      1.0000
Dilution              :      1.0000
Use Multiplier & Dilution Factor with ISTDs


Signal 1: DAD1 A, Sig=210,4 Ref=360,100


Signal 2: DAD1 B, Sig=230,4 Ref=360,100


Signal 3: DAD1 C, Sig=254,4 Ref=360,100


Signal 4: DAD1 D, Sig=280,4 Ref=360,100
```

Peak #	RetTime [min]	Type	Width [min]	Area [mAU*s]	Height [mAU]	Area %
1	5.567	BB	0.1672	1747.66870	160.25475	96.1922
2	6.218	BB	0.1786	69.18137	5.90963	3.8078

Totals : 1816.85007 166.16438

```
Signal 5: DAD1 E, Sig=310,4 Ref=360,100

=====================================================================
                          *** End of Report ***
```

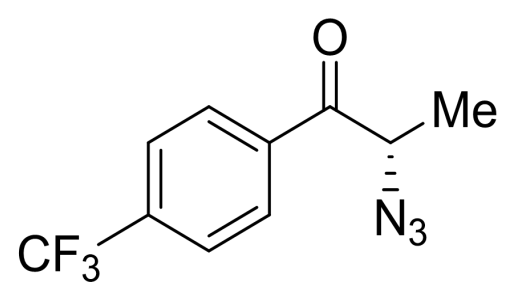

7f

```
=====================================================================
Acq. Operator   :                                Seq. Line :   2
Acq. Instrument : Instrument 1                    Location : Vial 22
Injection Date  : 9/23/2022 7:10:02 PM                 Inj :   1
                                                Inj Volume : 5.000 µl
Different Inj Volume from Sequence !     Actual Inj Volume : 9.000 µl
Acq. Method     : C:\CHEM32\1\DATA\SUN_12 2022-09-23 18-57-13\IC-05-20.M
Last changed    : 6/25/2018 1:35:20 AM
Analysis Method : C:\CHEM32\1\METHODS\AY3-02-30.M
Last changed    : 11/14/2022 10:25:15 PM
                  (modified after loading)
Additional Info : Peak(s) manually integrated
```

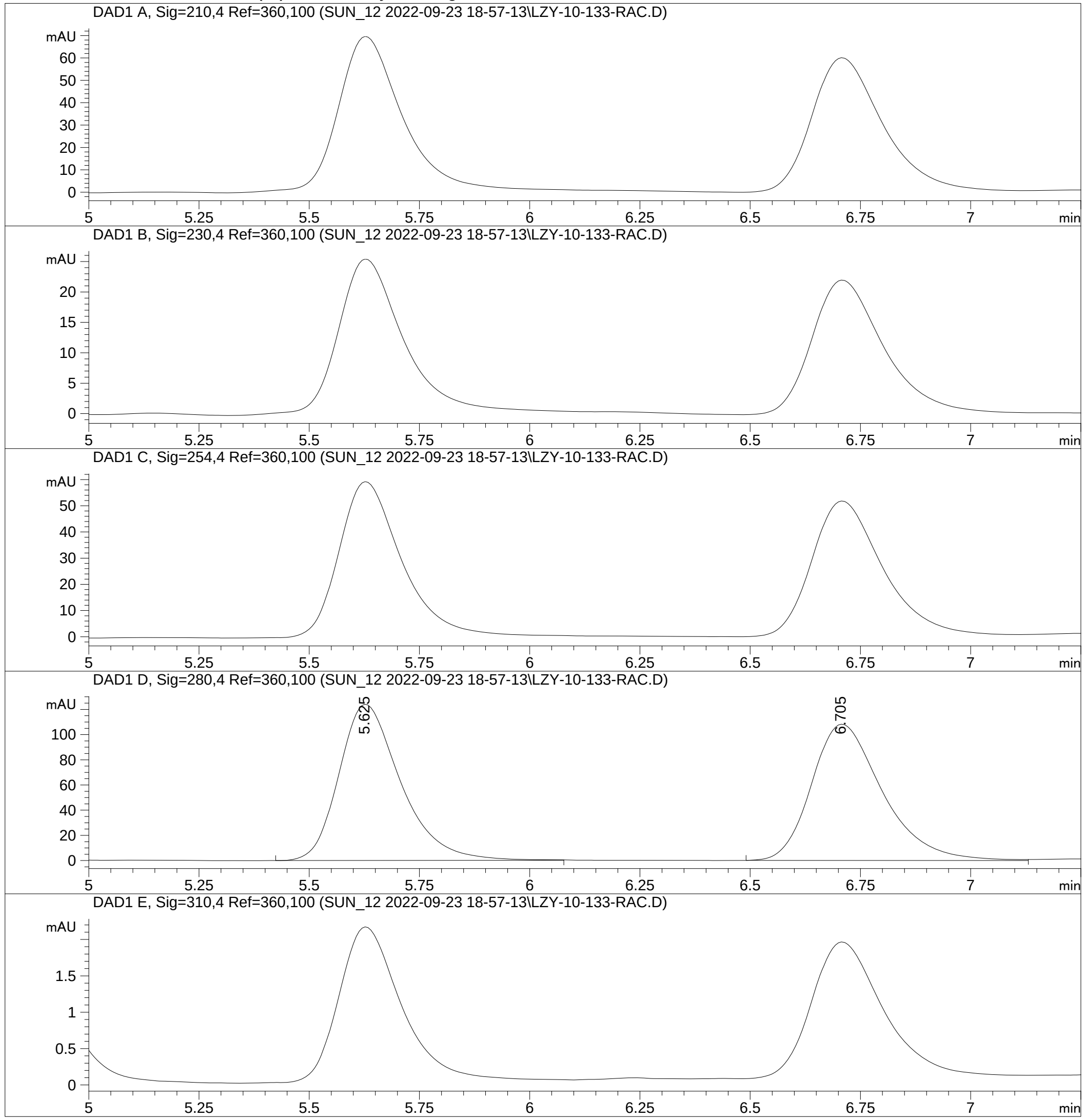

Data File C:\CHEM32\1\DATA\SUN_12 2022-09-23 18-57-13\LZY-10-133-RAC.D
Sample Name:

```
=====================================================================
                         Area Percent Report
=====================================================================

Sorted By             :      Signal
Multiplier            :      1.0000
Dilution              :      1.0000
Use Multiplier & Dilution Factor with ISTDs


Signal 1: DAD1 A, Sig=210,4 Ref=360,100


Signal 2: DAD1 B, Sig=230,4 Ref=360,100


Signal 3: DAD1 C, Sig=254,4 Ref=360,100


Signal 4: DAD1 D, Sig=280,4 Ref=360,100

Peak RetTime Type  Width     Area      Height     Area
  #   [min]        [min]   [mAU*s]     [mAU]        %
----|-------|----|-------|----------|----------|--------|
   1   5.625 BB    0.1476 1207.83118  124.03680  49.9300
   2   6.705 BB    0.1686 1211.21851  108.17992  50.0700

Totals :                  2419.04968  232.21673



Signal 5: DAD1 E, Sig=310,4 Ref=360,100

=====================================================================
                          *** End of Report ***
```

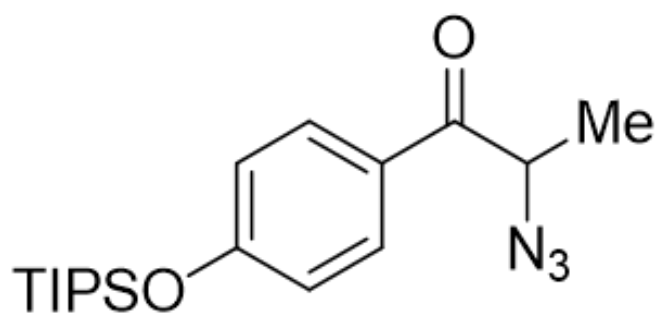

7g-Rac

```
=====================================================================
Acq. Operator   :                               Seq. Line :   7
Acq. Instrument : Instrument 1                   Location : Vial 21
Injection Date  : 7/28/2022 1:39:08 AM                Inj :   1
                                               Inj Volume : 5.000 µl
Different Inj Volume from Sequence !     Actual Inj Volume : 2.000 µl
Acq. Method     : C:\CHEM32\1\DATA\SUN_12 2022-07-27 23-10-28\IC-05-20.M
Last changed    : 6/25/2018 1:35:20 AM
Analysis Method : C:\CHEM32\1\METHODS\AY3-02-30.M
Last changed    : 11/14/2022 10:25:15 PM
                  (modified after loading)
Additional Info : Peak(s) manually integrated
```

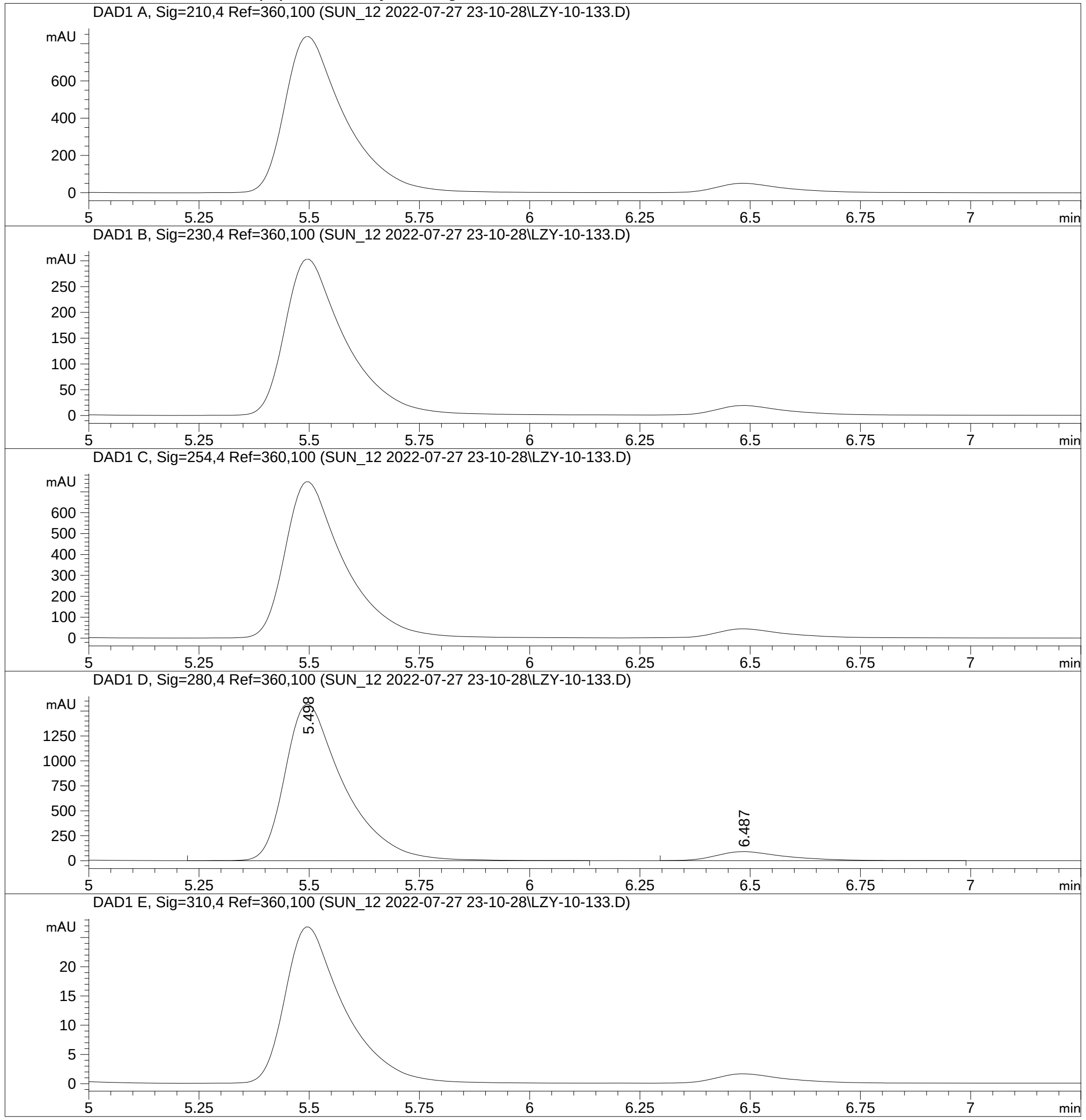

Data File C:\CHEM32\1\DATA\SUN_12 2022-07-27 23-10-28\LZY-10-133.D
Sample Name:

```
=====================================================================
                          Area Percent Report
=====================================================================

Sorted By             :      Signal
Multiplier            :      1.0000
Dilution              :      1.0000
Use Multiplier & Dilution Factor with ISTDs


Signal 1: DAD1 A, Sig=210,4 Ref=360,100


Signal 2: DAD1 B, Sig=230,4 Ref=360,100


Signal 3: DAD1 C, Sig=254,4 Ref=360,100


Signal 4: DAD1 D, Sig=280,4 Ref=360,100

Peak RetTime Type  Width     Area      Height     Area
  #   [min]        [min]   [mAU*s]     [mAU]        %
----|-------|----|-------|----------|----------|--------|
   1   5.498 VB    0.1387 1.46190e4  1569.50989  93.6284
   2   6.487 BB    0.1619  994.84601   90.76784   6.3716

Totals :                  1.56138e4  1660.27773



Signal 5: DAD1 E, Sig=310,4 Ref=360,100

=====================================================================
                          *** End of Report ***
```

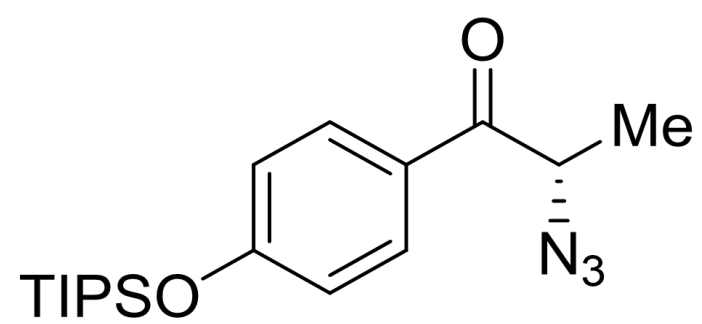

7g

Data File C:\CHEM32\1\DATA\SUN_12 2022-09-07 10-50-26\LZY-11-19-RAC.D
Sample Name:

===

Acq. Operator : Seq. Line : 2
Acq. Instrument : Instrument 1 Location : Vial 21
Injection Date : 9/7/2022 11:04:00 AM Inj : 1
Inj Volume : 5.000 µl
Different Inj Volume from Sequence ! Actual Inj Volume : 9.000 µl
Acq. Method : C:\CHEM32\1\DATA\SUN_12 2022-09-07 10-50-26\IC-10-20.M
Last changed : 6/25/2018 2:45:05 PM
Analysis Method : C:\CHEM32\1\METHODS\AY3-02-30.M
Last changed : 11/11/2022 3:56:34 PM
(modified after loading)
Additional Info : Peak(s) manually integrated

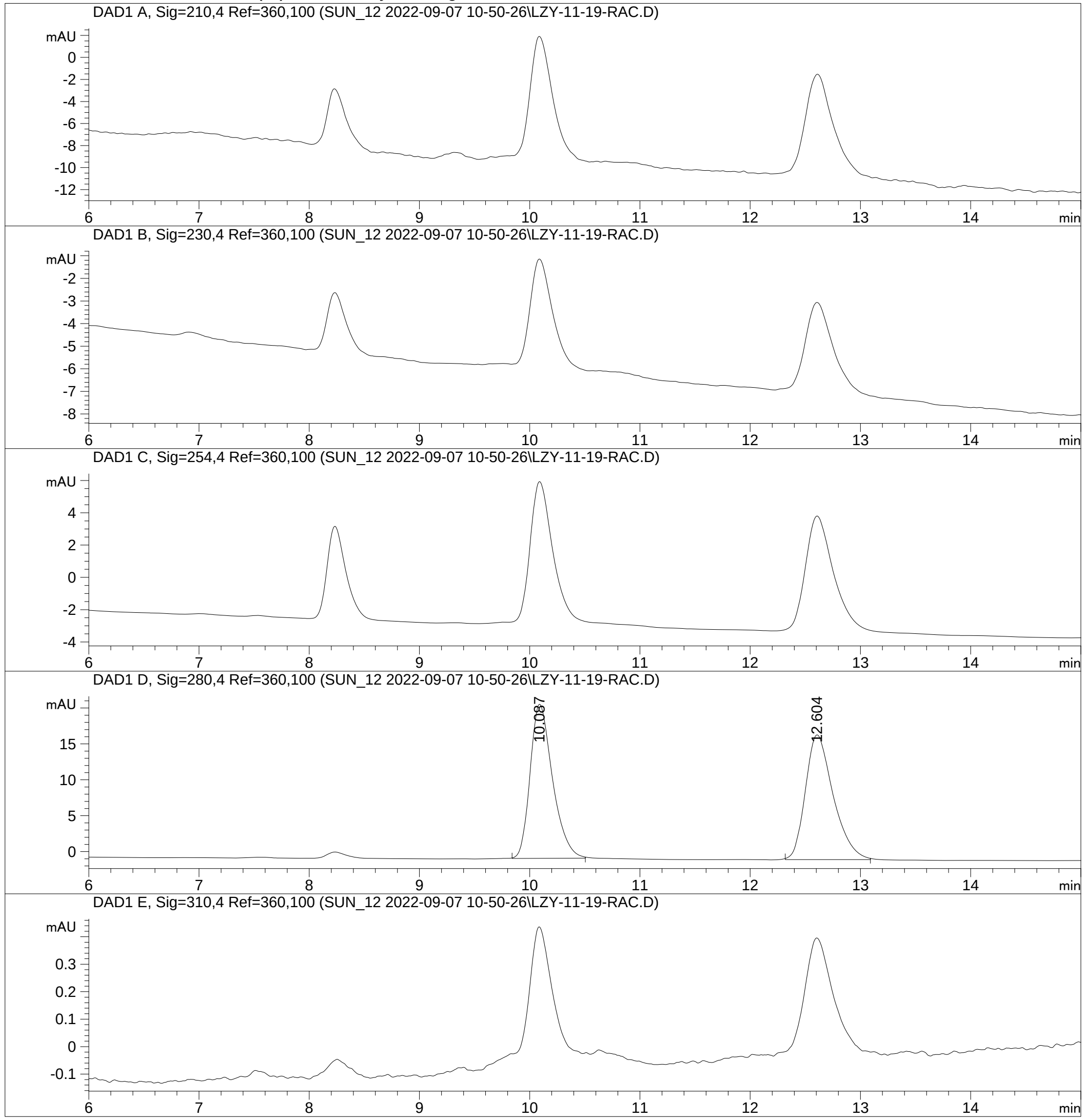

Data File C:\CHEM32\1\DATA\SUN_12 2022-09-07 10-50-26\LZY-11-19-RAC.D
Sample Name:

===

Area Percent Report

===

Sorted By : Signal
Multiplier : 1.0000
Dilution : 1.0000
Use Multiplier & Dilution Factor with ISTDs

Signal 1: DAD1 A, Sig=210,4 Ref=360,100

Signal 2: DAD1 B, Sig=230,4 Ref=360,100

Signal 3: DAD1 C, Sig=254,4 Ref=360,100

Signal 4: DAD1 D, Sig=280,4 Ref=360,100

Peak #	RetTime [min]	Type	Width [min]	Area [mAU*s]	Height [mAU]	Area %
1	10.087	BB	0.2146	302.96677	21.47423	49.8626
2	12.604	BB	0.2645	304.63596	17.38315	50.1374

Totals : 607.60272 38.85738

Signal 5: DAD1 E, Sig=310,4 Ref=360,100

===

*** End of Report ***

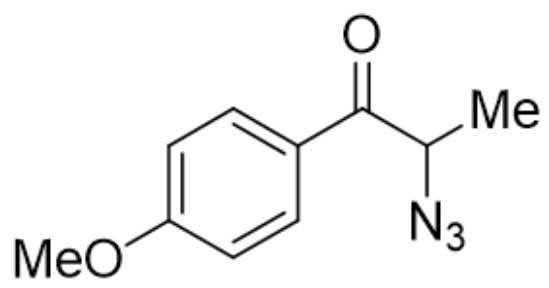

7h-Rac

Data File C:\CHEM32\1\DATA\SUN_12 2022-08-25 19-32-08\LZY-11-19.D
Sample Name:

===

```
Acq. Operator   :                              Seq. Line :   2
Acq. Instrument : Instrument 1                  Location : Vial 21
Injection Date  : 8/25/2022 7:44:57 PM               Inj :   1
                                              Inj Volume : 5.000 µl
Different Inj Volume from Sequence !     Actual Inj Volume : 3.000 µl
Acq. Method     : C:\CHEM32\1\DATA\SUN_12 2022-08-25 19-32-08\IC-10-20.M
Last changed    : 6/25/2018 2:45:05 PM
Analysis Method : C:\CHEM32\1\METHODS\AY3-02-30.M
Last changed    : 11/11/2022 3:56:34 PM
                  (modified after loading)
Additional Info : Peak(s) manually integrated
```

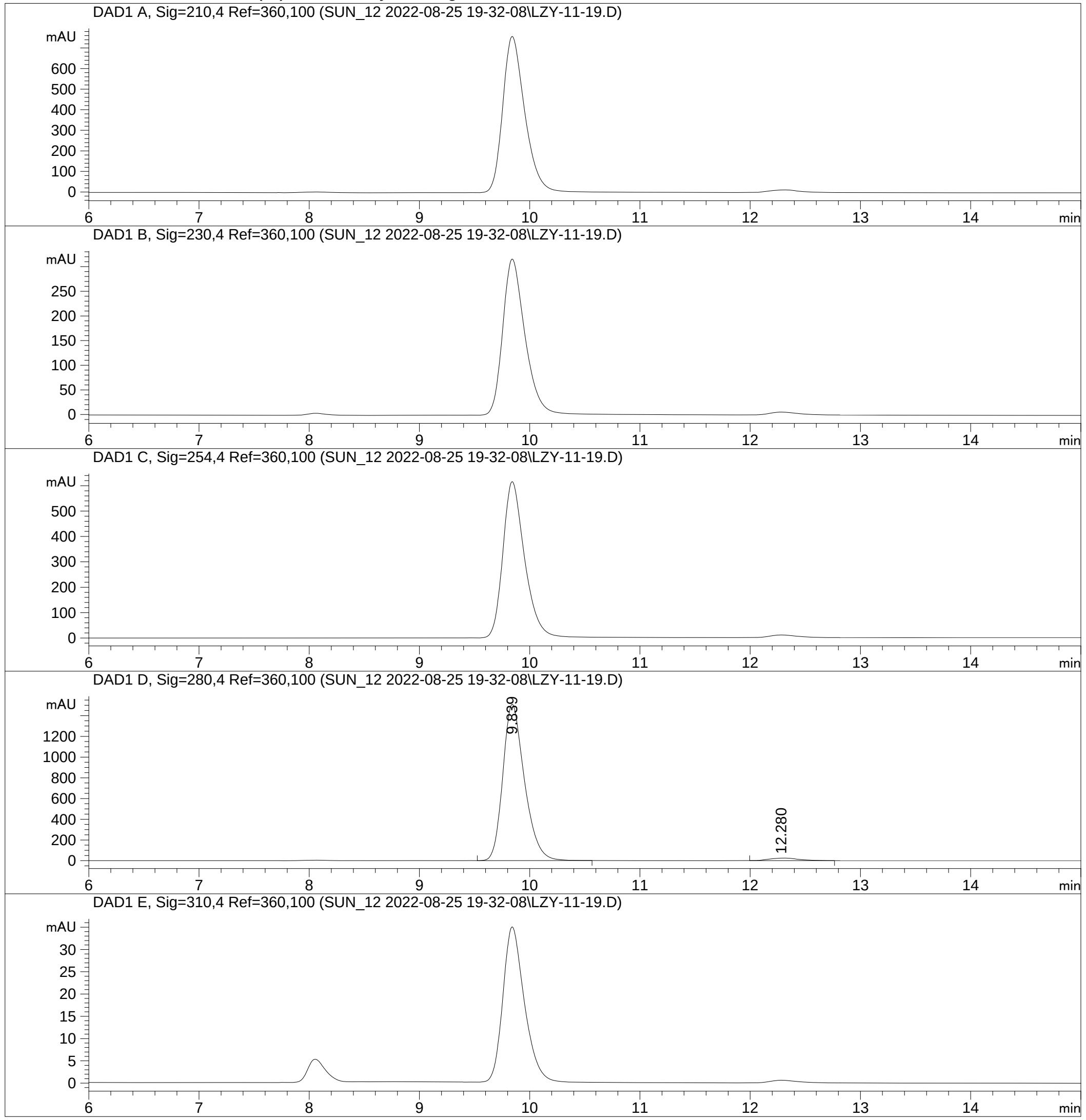

Data File C:\CHEM32\1\DATA\SUN_12 2022-08-25 19-32-08\LZY-11-19.D
Sample Name:

===

Area Percent Report

===

```
Sorted By            :      Signal
Multiplier           :      1.0000
Dilution             :      1.0000
Use Multiplier & Dilution Factor with ISTDs


Signal 1: DAD1 A, Sig=210,4 Ref=360,100


Signal 2: DAD1 B, Sig=230,4 Ref=360,100


Signal 3: DAD1 C, Sig=254,4 Ref=360,100


Signal 4: DAD1 D, Sig=280,4 Ref=360,100
```

Peak #	RetTime [min]	Type	Width [min]	Area [mAU*s]	Height [mAU]	Area %
1	9.839	BB	0.2046	2.05203e4	1509.68811	97.8996
2	12.280	BB	0.2600	440.24850	25.68012	2.1004

Totals : 2.09605e4 1535.36823

Signal 5: DAD1 E, Sig=310,4 Ref=360,100

===

*** End of Report ***

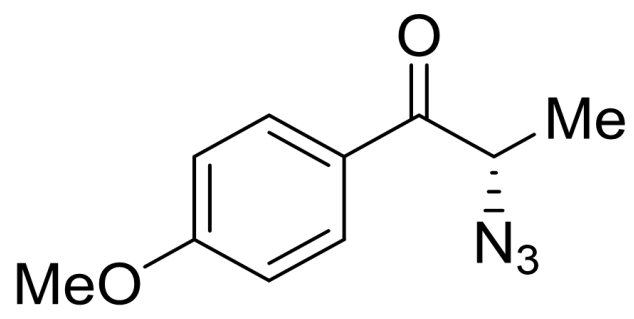

7h

Data File C:\CHEM32\1\DATA\SUN_12 2022-11-02 09-11-17\LZY-11-177-A-RAC-IC07.D
Sample Name:

```
=====================================================================
Acq. Operator   :                              Seq. Line :  11
Acq. Instrument : Instrument 1                  Location : Vial 21
Injection Date  : 11/2/2022 12:06:56 PM              Inj :   1
                                              Inj Volume : 5.000 µl
Different Inj Volume from Sequence !     Actual Inj Volume : 9.000 µl
Acq. Method     : C:\CHEM32\1\DATA\SUN_12 2022-11-02 09-11-17\IC-07-30.M
Last changed    : 8/25/2016 5:14:59 PM
Analysis Method : C:\CHEM32\1\METHODS\AY3-02-30.M
Last changed    : 11/14/2022 10:58:08 PM
                  (modified after loading)
Additional Info : Peak(s) manually integrated
```

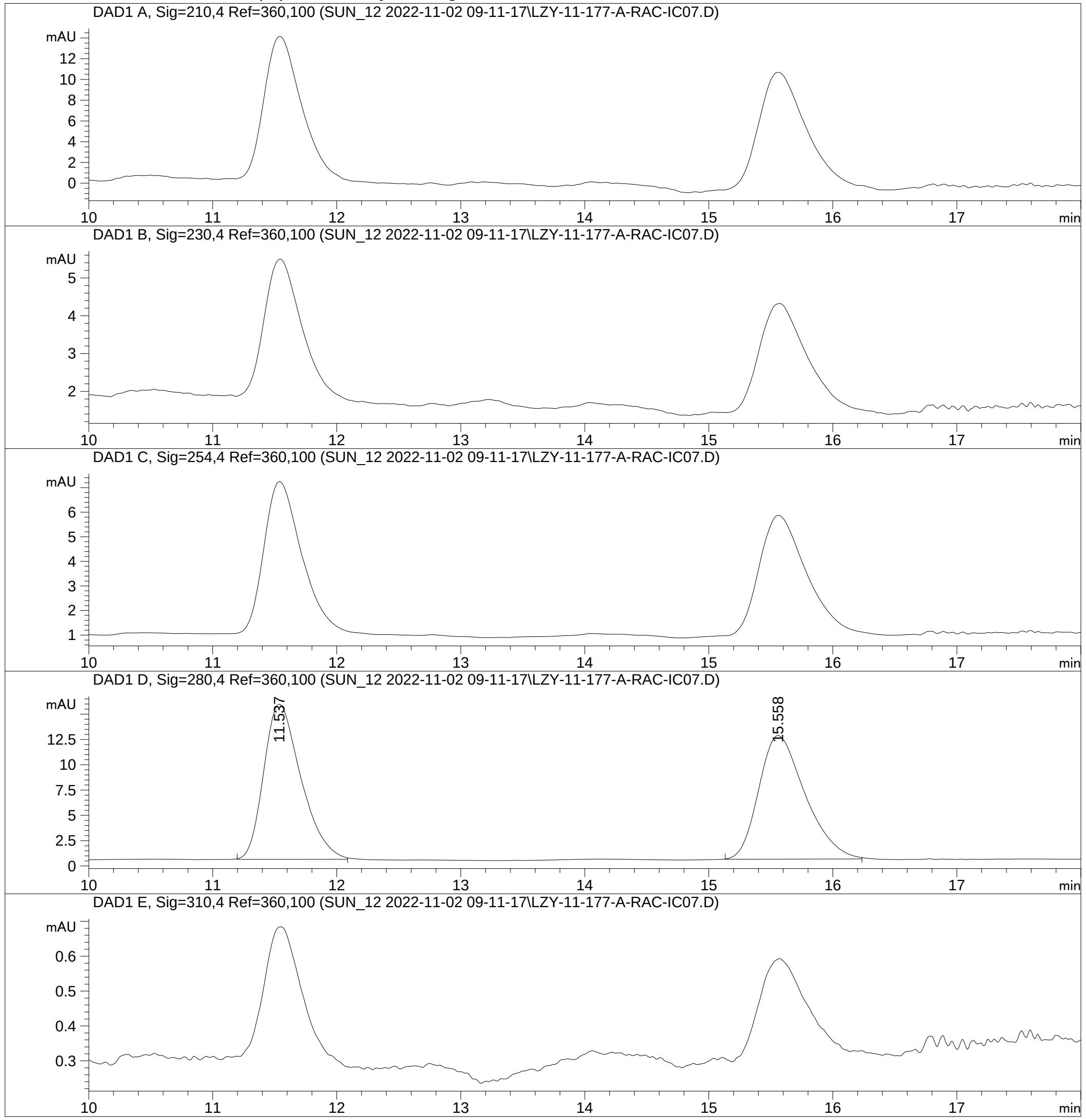

Data File C:\CHEM32\1\DATA\SUN_12 2022-11-02 09-11-17\LZY-11-177-A-RAC-IC07.D
Sample Name:

```
=====================================================================
                         Area Percent Report
=====================================================================

Sorted By             :      Signal
Multiplier            :      1.0000
Dilution              :      1.0000
Use Multiplier & Dilution Factor with ISTDs


Signal 1: DAD1 A, Sig=210,4 Ref=360,100


Signal 2: DAD1 B, Sig=230,4 Ref=360,100


Signal 3: DAD1 C, Sig=254,4 Ref=360,100


Signal 4: DAD1 D, Sig=280,4 Ref=360,100
```

Peak #	RetTime [min]	Type	Width [min]	Area [mAU*s]	Height [mAU]	Area %
1	11.537	BB	0.3169	319.05554	15.32608	50.1894
2	15.558	BB	0.3964	316.64728	12.21432	49.8106

Totals : 635.70282 27.54041

```
Signal 5: DAD1 E, Sig=310,4 Ref=360,100

=====================================================================
                        *** End of Report ***
```

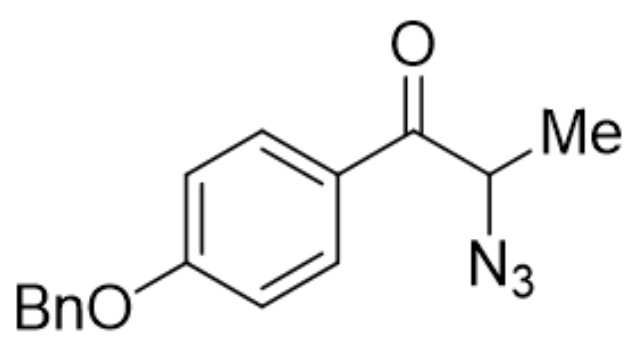

7i-Rac

Data File C:\CHEM32\1\DATA\SUN_12 2022-11-07 16-05-52\LZY-11-191.D
Sample Name:

===

Acq. Operator : Seq. Line : 27
Acq. Instrument : Instrument 1 Location : Vial 21
Injection Date : 11/7/2022 11:37:21 PM Inj : 1
Inj Volume : 5.000 µl
Different Inj Volume from Sequence ! Actual Inj Volume : 6.000 µl
Acq. Method : C:\CHEM32\1\DATA\SUN_12 2022-11-07 16-05-52\IC-07-30.M
Last changed : 8/25/2016 5:14:59 PM
Analysis Method : C:\CHEM32\1\METHODS\AY3-02-30.M
Last changed : 11/14/2022 10:58:08 PM
(modified after loading)
Additional Info : Peak(s) manually integrated

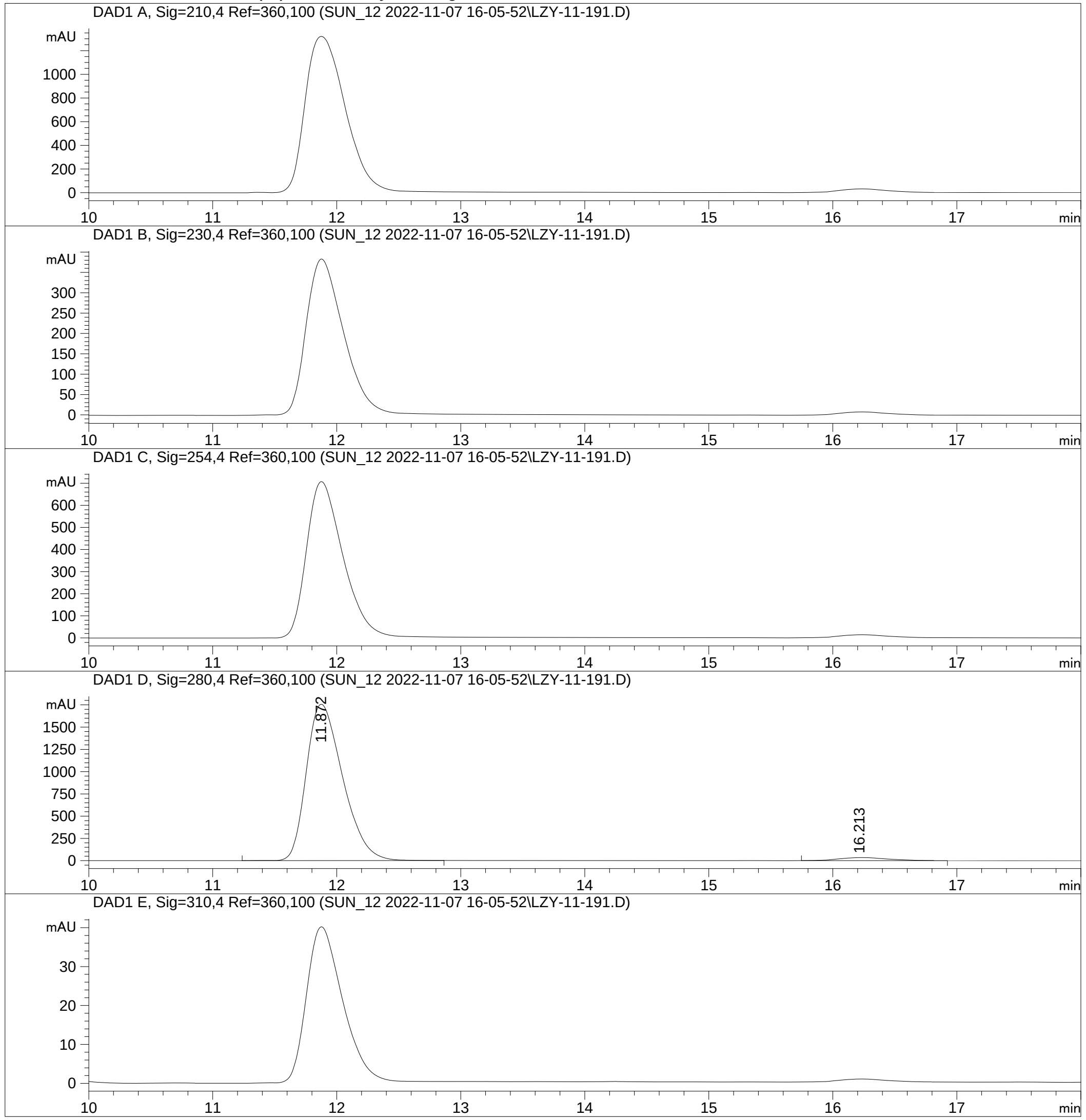

Data File C:\CHEM32\1\DATA\SUN_12 2022-11-07 16-05-52\LZY-11-191.D
Sample Name:

===

Area Percent Report

===

Sorted By : Signal
Multiplier : 1.0000
Dilution : 1.0000
Use Multiplier & Dilution Factor with ISTDs

Signal 1: DAD1 A, Sig=210,4 Ref=360,100

Signal 2: DAD1 B, Sig=230,4 Ref=360,100

Signal 3: DAD1 C, Sig=254,4 Ref=360,100

Signal 4: DAD1 D, Sig=280,4 Ref=360,100

Peak #	RetTime [min]	Type	Width [min]	Area [mAU*s]	Height [mAU]	Area %
1	11.872	BB	0.3137	3.57964e4	1756.68799	97.5000
2	16.213	BB	0.4056	917.85071	34.58976	2.5000

Totals : 3.67143e4 1791.27775

Signal 5: DAD1 E, Sig=310,4 Ref=360,100

===

*** End of Report ***

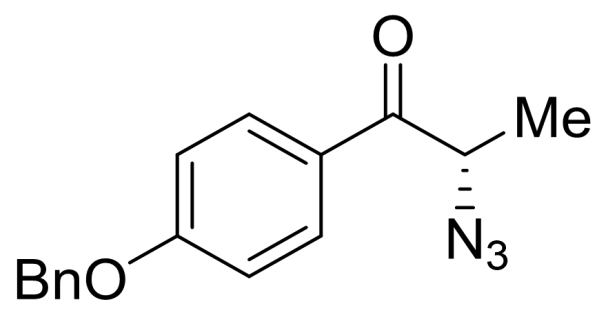

7i

Data File C:\CHEM32\1\DATA\SUN_12 2022-10-03 19-45-33\LZY-10-115-B-RAC.D
Sample Name:

```
=====================================================================
Acq. Operator   :                             Seq. Line :   2
Acq. Instrument : Instrument 1                 Location : Vial 21
Injection Date  : 10/3/2022 8:01:55 PM              Inj :   1
                                             Inj Volume : 5.000 µl
Different Inj Volume from Sequence !     Actual Inj Volume : 9.000 µl
Acq. Method     : C:\CHEM32\1\DATA\SUN_12 2022-10-03 19-45-33\IC-05-20.M
Last changed    : 6/25/2018 1:35:20 AM
Analysis Method : C:\CHEM32\1\METHODS\AY3-02-30.M
Last changed    : 11/14/2022 10:08:01 PM
                  (modified after loading)
Additional Info : Peak(s) manually integrated
```

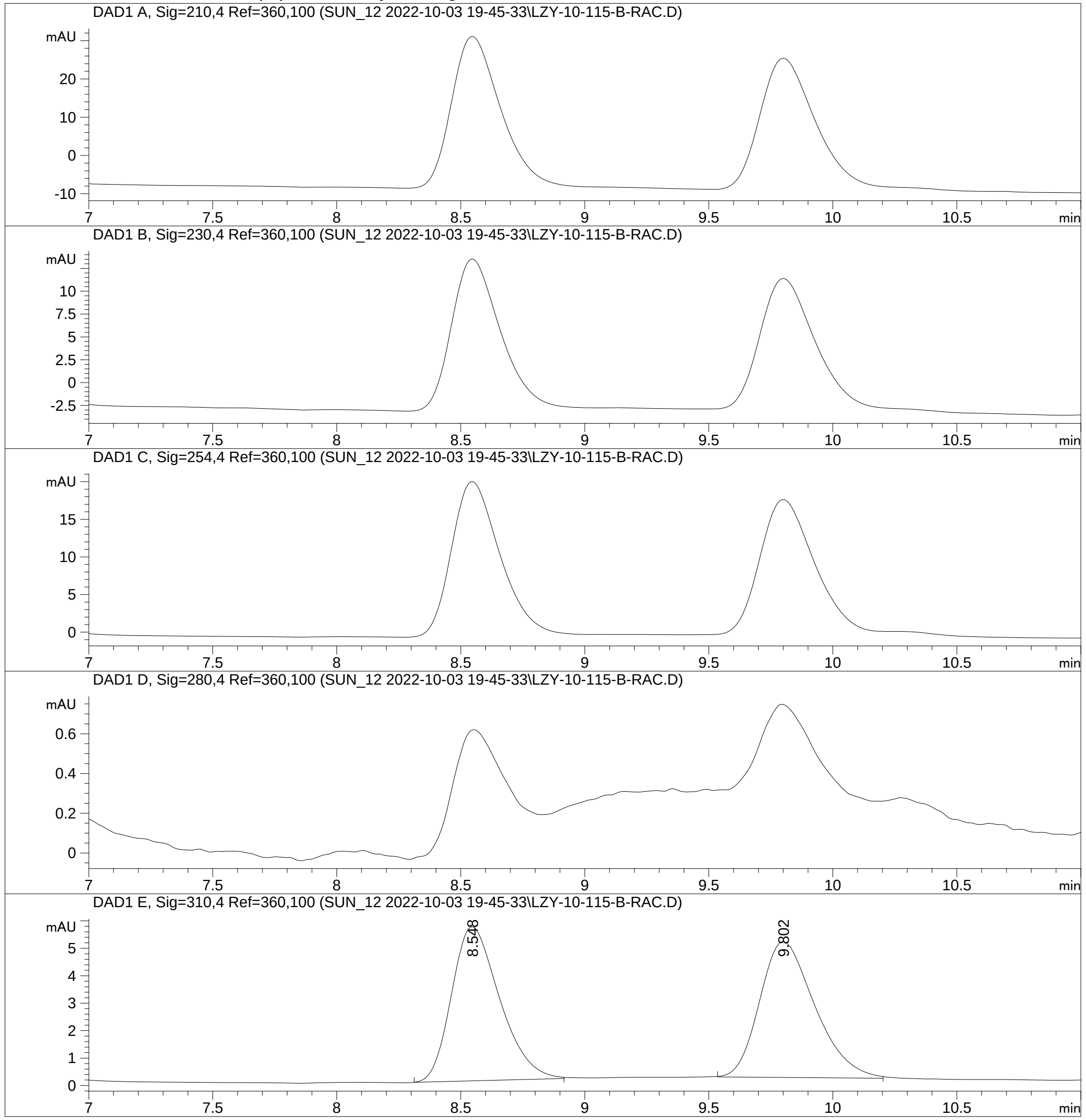

Data File C:\CHEM32\1\DATA\SUN_12 2022-10-03 19-45-33\LZY-10-115-B-RAC.D
Sample Name:

```
=====================================================================
                         Area Percent Report
=====================================================================

Sorted By             :      Signal
Multiplier            :      1.0000
Dilution              :      1.0000
Use Multiplier & Dilution Factor with ISTDs


Signal 1: DAD1 A, Sig=210,4 Ref=360,100

Signal 2: DAD1 B, Sig=230,4 Ref=360,100

Signal 3: DAD1 C, Sig=254,4 Ref=360,100

Signal 4: DAD1 D, Sig=280,4 Ref=360,100

Signal 5: DAD1 E, Sig=310,4 Ref=360,100

Peak RetTime Type  Width     Area      Height     Area
  #   [min]        [min]   [mAU*s]     [mAU]        %
----|-------|----|-------|----------|----------|--------|
   1   8.548 BB    0.2071   75.61379    5.61393  49.6949
   2   9.802 BB    0.2364   76.54230    4.94882  50.3051

Totals :                   152.15609   10.56274

=====================================================================
                          *** End of Report ***
```

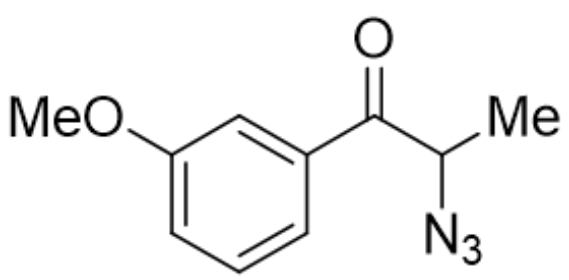

7j-Rac

Data File C:\CHEM32\1\DATA\SUN_12 2022-07-15 14-54-37\LZY-10-115-B.D
Sample Name:

```
=====================================================================
Acq. Operator   :                        Seq. Line :   2
Acq. Instrument : Instrument 1            Location : Vial 21
Injection Date  : 7/15/2022 3:07:27 PM         Inj :   1
                                        Inj Volume : 5.000 µl
Different Inj Volume from Sequence !     Actual Inj Volume : 3.000 µl
Acq. Method     : C:\CHEM32\1\DATA\SUN_12 2022-07-15 14-54-37\IC-05-20.M
Last changed    : 6/25/2018 1:35:20 AM
Analysis Method : C:\CHEM32\1\METHODS\AY3-02-30.M
Last changed    : 11/14/2022 10:08:01 PM
                  (modified after loading)
Additional Info : Peak(s) manually integrated
```

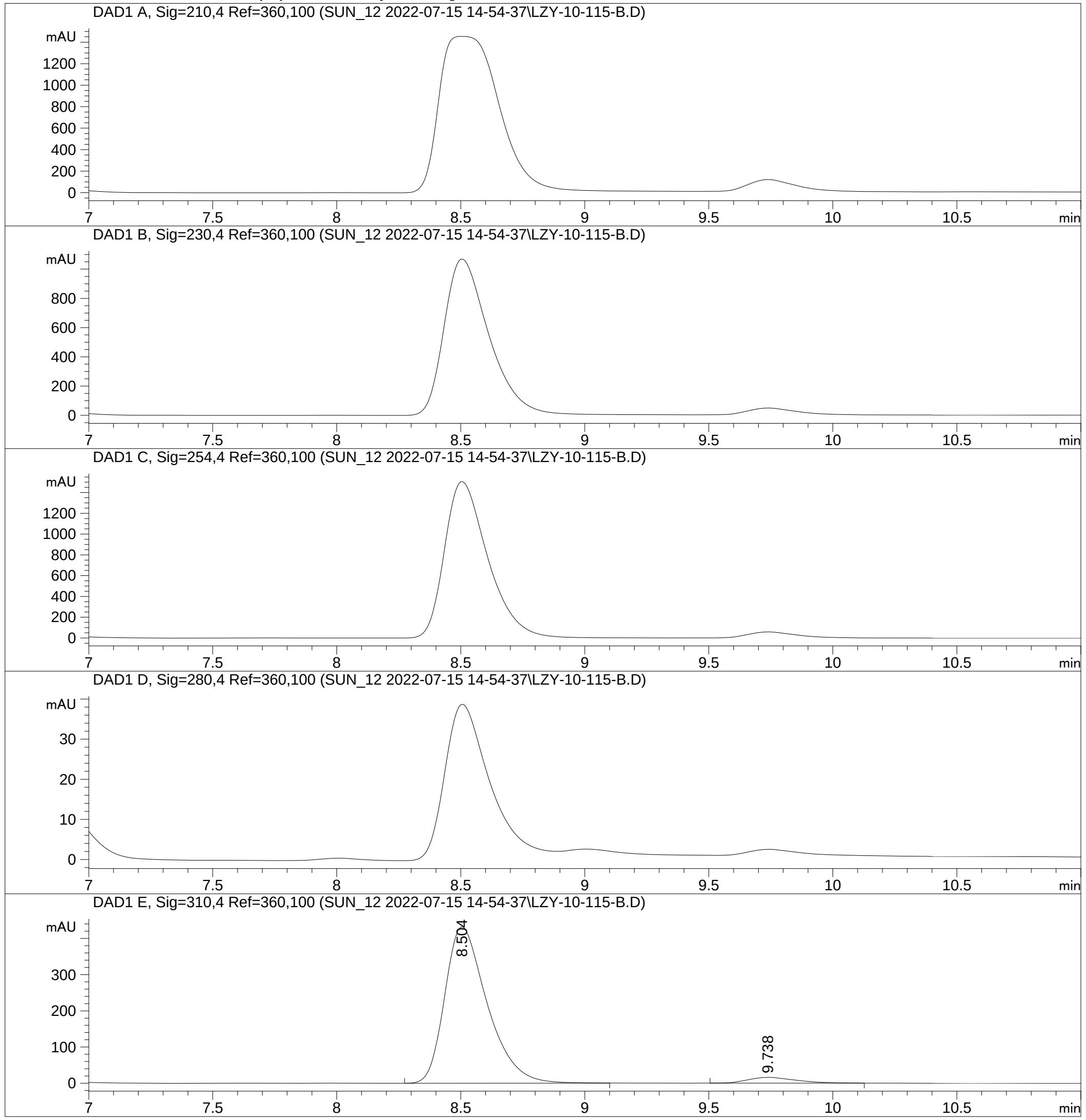

Data File C:\CHEM32\1\DATA\SUN_12 2022-07-15 14-54-37\LZY-10-115-B.D
Sample Name:

===
Area Percent Report
===

Sorted By : Signal
Multiplier : 1.0000
Dilution : 1.0000
Use Multiplier & Dilution Factor with ISTDs

Signal 1: DAD1 A, Sig=210,4 Ref=360,100

Signal 2: DAD1 B, Sig=230,4 Ref=360,100

Signal 3: DAD1 C, Sig=254,4 Ref=360,100

Signal 4: DAD1 D, Sig=280,4 Ref=360,100

Signal 5: DAD1 E, Sig=310,4 Ref=360,100

Peak #	RetTime [min]	Type	Width [min]	Area [mAU*s]	Height [mAU]	Area %
1	8.504	BB	0.1786	5132.96582	432.16959	95.9813
2	9.738	BB	0.2038	214.91335	16.09316	4.0187

Totals : 5347.87917 448.26274

===
*** End of Report ***

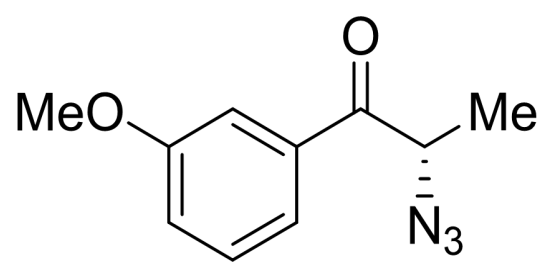

7j

===

```
Acq. Operator   :                              Seq. Line :   5
Acq. Instrument : Instrument 1                  Location : Vial 21
Injection Date  : 9/23/2022 5:06:57 PM               Inj :   1
                                              Inj Volume : 5.000 µl
Different Inj Volume from Sequence !     Actual Inj Volume : 9.000 µl
Acq. Method     : C:\CHEM32\1\DATA\SUN_12 2022-09-23 16-09-42\AS-05-20.M
Last changed    : 9/23/2022 5:06:04 PM
                  (modified after loading)
Analysis Method : C:\CHEM32\1\METHODS\AY3-02-30.M
Last changed    : 11/14/2022 10:51:31 PM
                  (modified after loading)
Additional Info : Peak(s) manually integrated
```

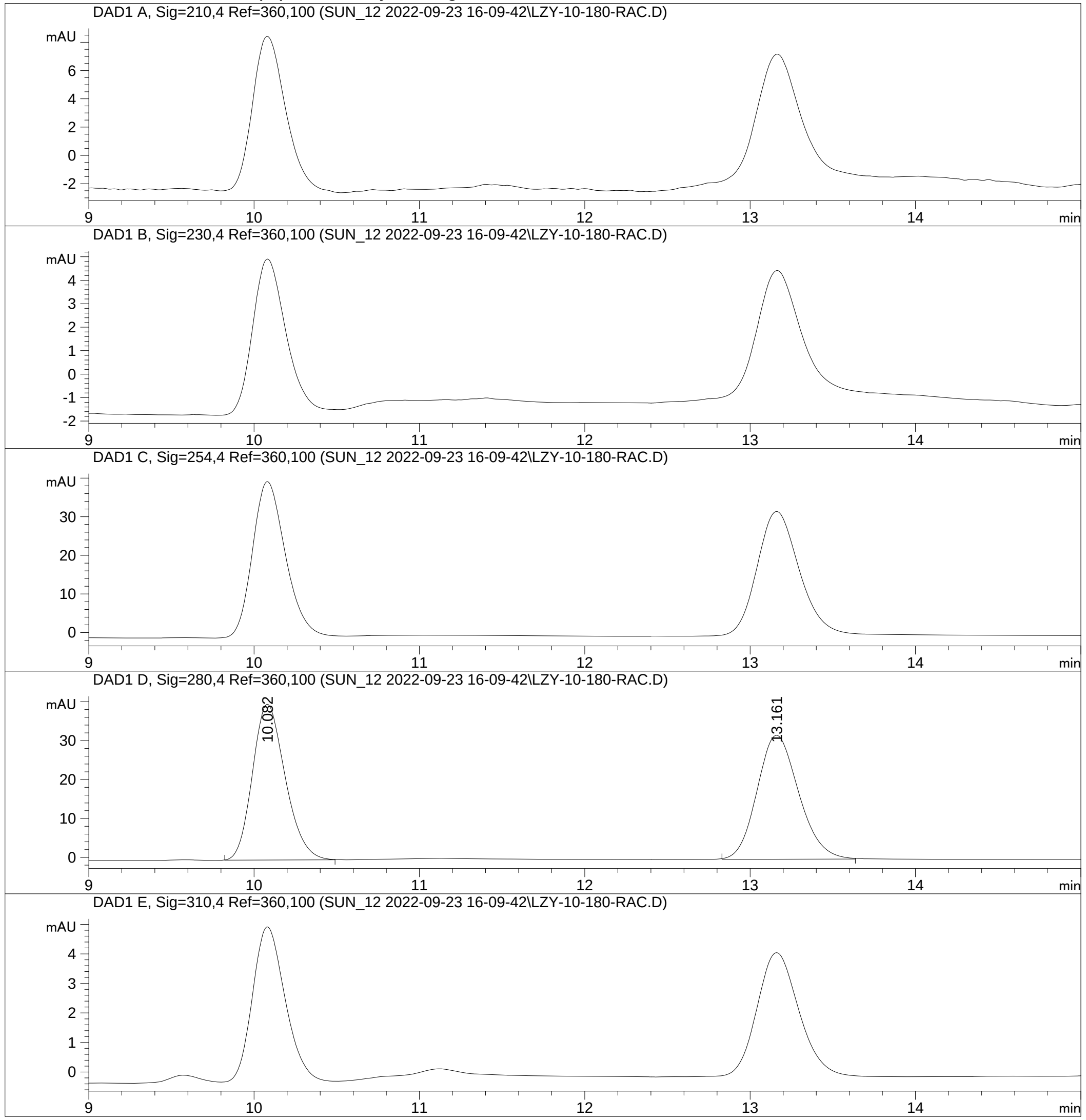

Data File C:\CHEM32\1\DATA\SUN_12 2022-09-23 16-09-42\LZY-10-180-RAC.D
Sample Name:

===

Area Percent Report

===

```
Sorted By             :      Signal
Multiplier            :      1.0000
Dilution              :      1.0000
Use Multiplier & Dilution Factor with ISTDs
```

Signal 1: DAD1 A, Sig=210,4 Ref=360,100

Signal 2: DAD1 B, Sig=230,4 Ref=360,100

Signal 3: DAD1 C, Sig=254,4 Ref=360,100

Signal 4: DAD1 D, Sig=280,4 Ref=360,100

Peak #	RetTime [min]	Type	Width [min]	Area [mAU*s]	Height [mAU]	Area %
1	10.082	BB	0.2124	550.60242	40.04538	49.7160
2	13.161	BB	0.2699	556.89313	31.86596	50.2840

Totals : 1107.49554 71.91134

Signal 5: DAD1 E, Sig=310,4 Ref=360,100

===

*** End of Report ***

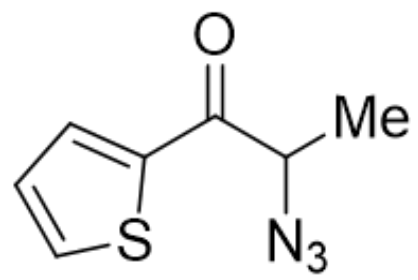

7k-Rac

===

Acq. Operator : Seq. Line : 33
Acq. Instrument : Instrument 1 Location : Vial 21
Injection Date : 8/13/2022 9:22:02 PM Inj : 1
Inj Volume : 5.000 µl
Different Inj Volume from Sequence ! Actual Inj Volume : 4.000 µl
Acq. Method : C:\CHEM32\1\DATA\SUN_12 2022-08-13 09-46-30\AS-05-20.M
Last changed : 8/13/2022 9:21:10 PM
(modified after loading)
Analysis Method : C:\CHEM32\1\METHODS\AY3-02-30.M
Last changed : 11/14/2022 10:51:31 PM
(modified after loading)
Additional Info : Peak(s) manually integrated

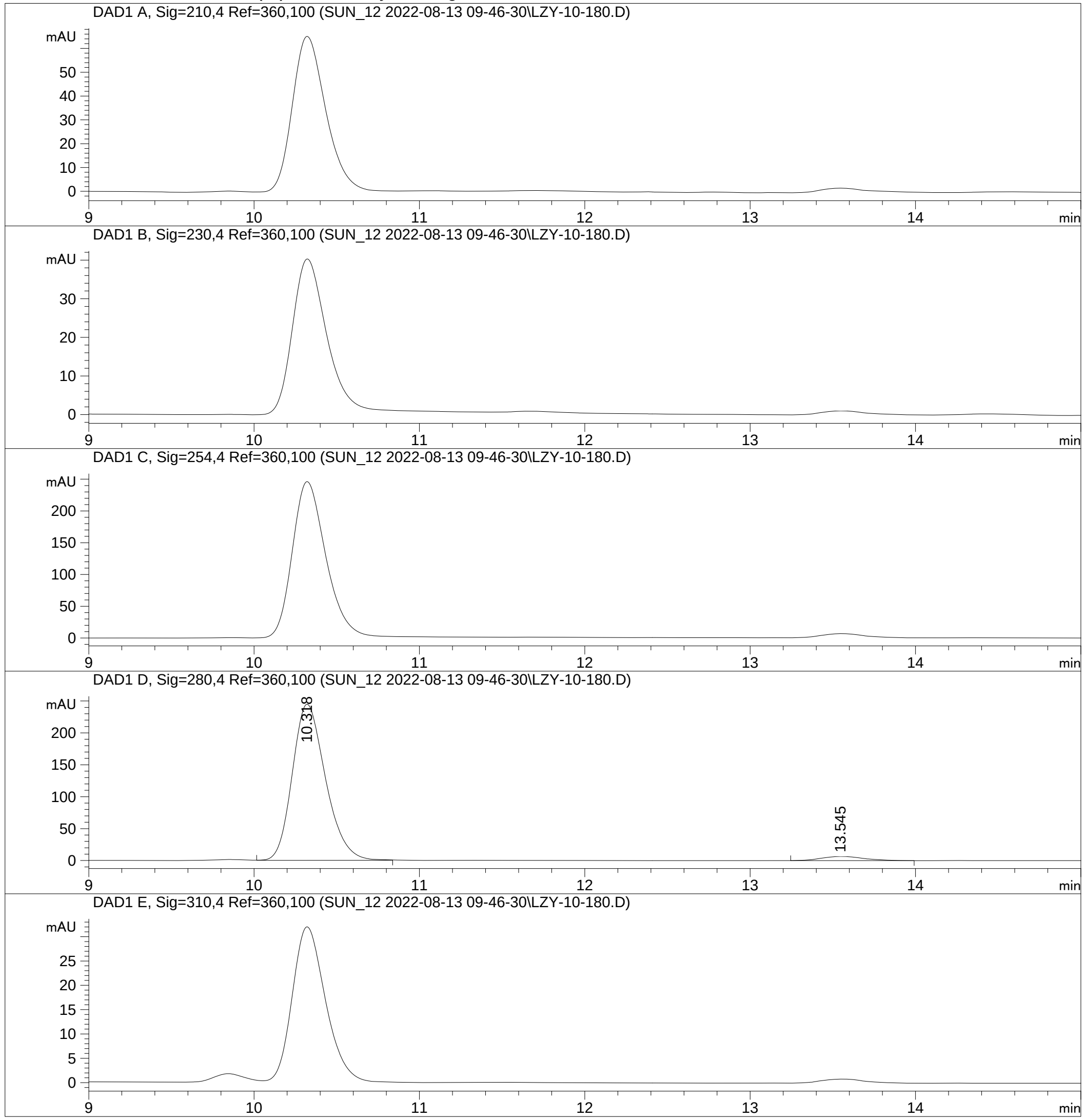

Data File C:\CHEM32\1\DATA\SUN_12 2022-08-13 09-46-30\LZY-10-180.D
Sample Name:

===

Area Percent Report

===

Sorted By : Signal
Multiplier : 1.0000
Dilution : 1.0000
Use Multiplier & Dilution Factor with ISTDs

Signal 1: DAD1 A, Sig=210,4 Ref=360,100

Signal 2: DAD1 B, Sig=230,4 Ref=360,100

Signal 3: DAD1 C, Sig=254,4 Ref=360,100

Signal 4: DAD1 D, Sig=280,4 Ref=360,100

Peak #	RetTime [min]	Type	Width [min]	Area [mAU*s]	Height [mAU]	Area %
1	10.318	VB	0.2126	3409.54761	244.66446	96.7899
2	13.545	BB	0.2630	113.08089	6.63066	3.2101

Totals : 3522.62849 251.29512

Signal 5: DAD1 E, Sig=310,4 Ref=360,100

===

*** End of Report ***

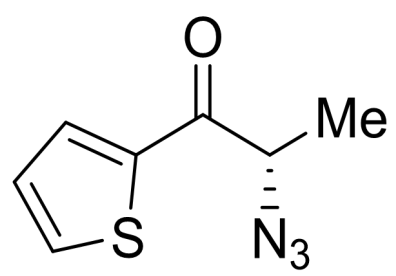

7k

===

Acq. Operator : Seq. Line : 2
Acq. Instrument : Instrument 1 Location : Vial 21
Injection Date : 10/15/2022 7:34:48 PM Inj : 1
Inj Volume : 5.000 µl
Different Inj Volume from Sequence ! Actual Inj Volume : 9.000 µl
Acq. Method : C:\CHEM32\1\DATA\SUN_12 2022-10-15 18-53-13\IC-07-30.M
Last changed : 8/25/2016 5:14:59 PM
Analysis Method : C:\CHEM32\1\METHODS\AY3-02-30.M
Last changed : 11/11/2022 4:47:23 PM
(modified after loading)
Additional Info : Peak(s) manually integrated

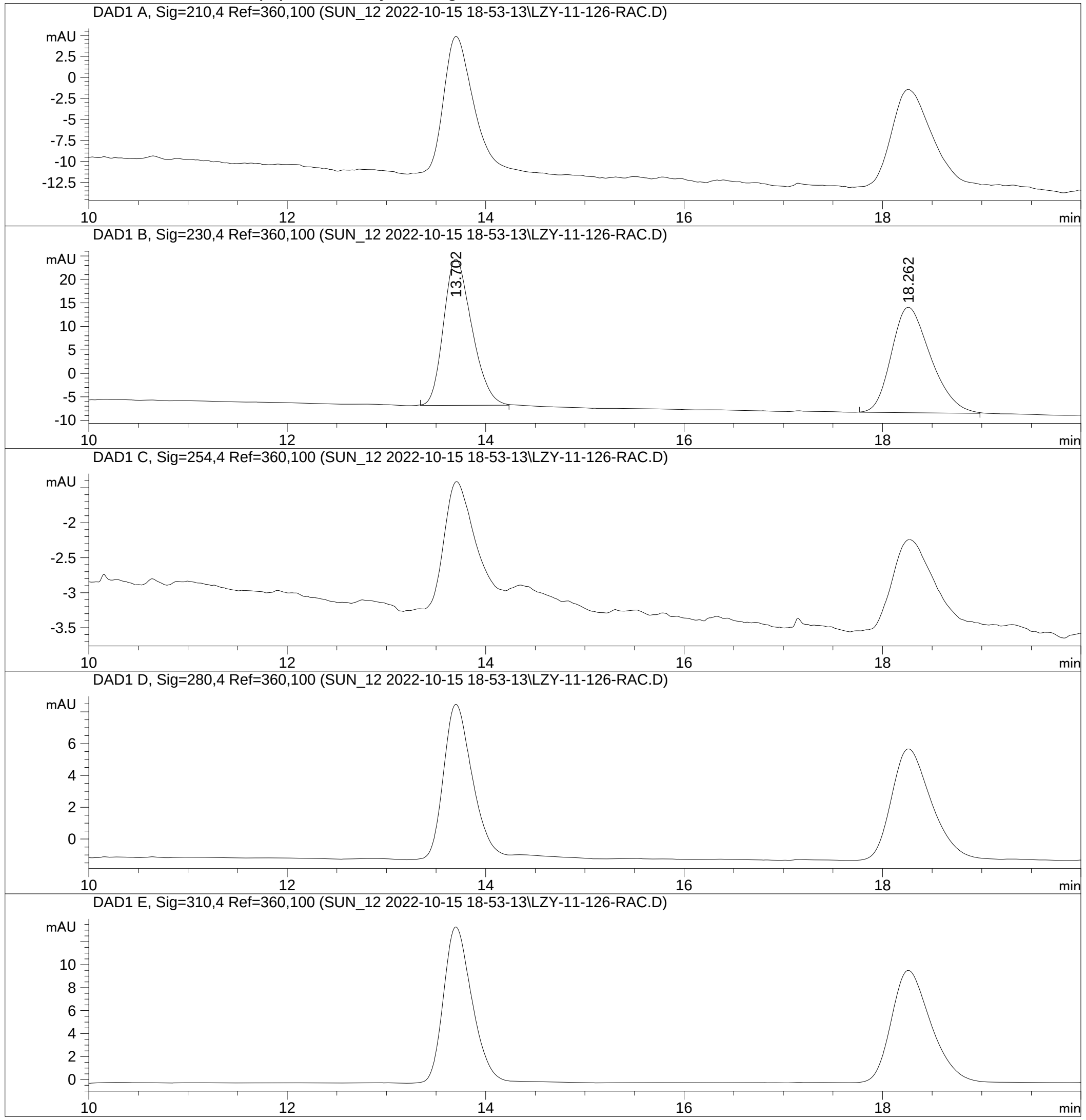

Data File C:\CHEM32\1\DATA\SUN_12 2022-10-15 18-53-13\LZY-11-126-RAC.D
Sample Name:

===

Area Percent Report

===

Sorted By : Signal
Multiplier : 1.0000
Dilution : 1.0000
Use Multiplier & Dilution Factor with ISTDs

Signal 1: DAD1 A, Sig=210,4 Ref=360,100

Signal 2: DAD1 B, Sig=230,4 Ref=360,100

Peak #	RetTime [min]	Type	Width [min]	Area [mAU*s]	Height [mAU]	Area %
1	13.702	BB	0.3046	615.86395	31.16456	49.9778
2	18.262	BB	0.4224	616.41132	22.44350	50.0222

Totals : 1232.27527 53.60806

Signal 3: DAD1 C, Sig=254,4 Ref=360,100

Signal 4: DAD1 D, Sig=280,4 Ref=360,100

Signal 5: DAD1 E, Sig=310,4 Ref=360,100

===

*** End of Report ***

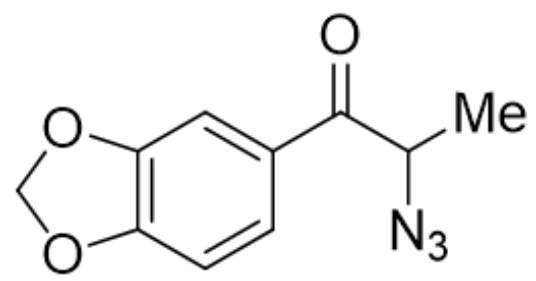

7l-Rac

Data File C:\CHEM32\1\DATA\SUN_12 2022-10-12 21-26-12\LZY-11-126.D
Sample Name:

===

Acq. Operator : Seq. Line : 2
Acq. Instrument : Instrument 1 Location : Vial 21
Injection Date : 10/12/2022 9:39:02 PM Inj : 1
Inj Volume : 5.000 µl
Different Inj Volume from Sequence ! Actual Inj Volume : 9.000 µl
Acq. Method : C:\CHEM32\1\DATA\SUN_12 2022-10-12 21-26-12\IC-07-30.M
Last changed : 8/25/2016 5:14:59 PM
Analysis Method : C:\CHEM32\1\METHODS\AY3-02-30.M
Last changed : 11/11/2022 4:47:23 PM
(modified after loading)
Additional Info : Peak(s) manually integrated

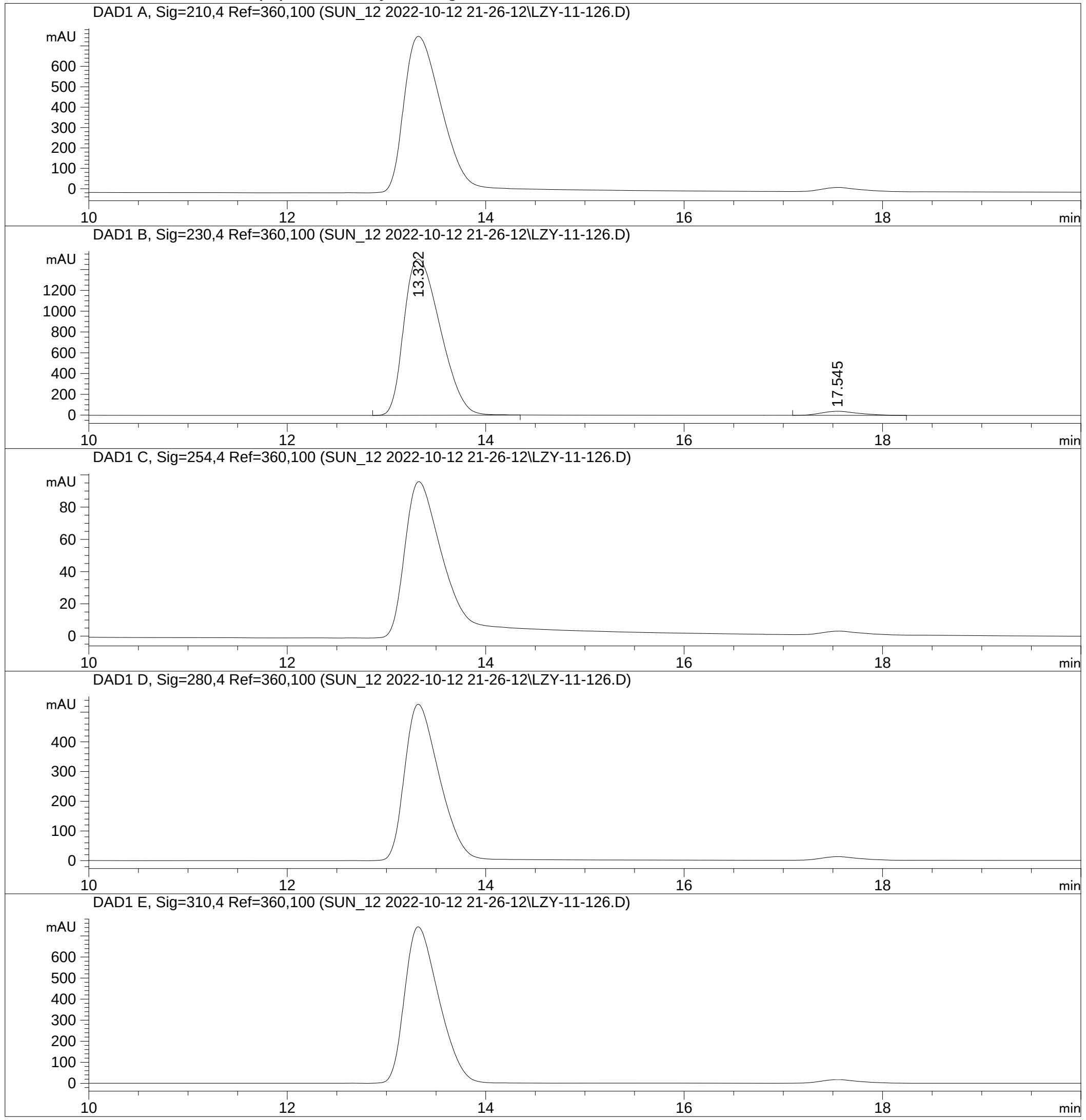

Data File C:\CHEM32\1\DATA\SUN_12 2022-10-12 21-26-12\LZY-11-126.D
Sample Name:

===

Area Percent Report

===

Sorted By : Signal
Multiplier : 1.0000
Dilution : 1.0000
Use Multiplier & Dilution Factor with ISTDs

Signal 1: DAD1 A, Sig=210,4 Ref=360,100

Signal 2: DAD1 B, Sig=230,4 Ref=360,100

Peak #	RetTime [min]	Type	Width [min]	Area [mAU*s]	Height [mAU]	Area %
1	13.322	BB	0.3983	3.82047e4	1504.26086	97.4181
2	17.545	BB	0.3976	1012.56836	39.17242	2.5819

Totals : 3.92173e4 1543.43328

Signal 3: DAD1 C, Sig=254,4 Ref=360,100

Signal 4: DAD1 D, Sig=280,4 Ref=360,100

Signal 5: DAD1 E, Sig=310,4 Ref=360,100

===

*** End of Report ***

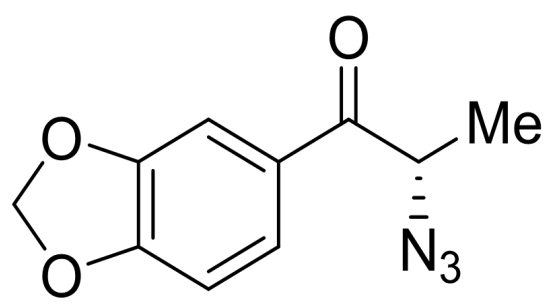

7l

```
=====================================================================
Acq. Operator   :                               Seq. Line :  10
Acq. Instrument : Instrument 1                   Location : Vial 23
Injection Date  : 11/12/2022 12:11:06 PM              Inj :   1
                                               Inj Volume : 5.000 µl
Different Inj Volume from Sequence !     Actual Inj Volume : 10.000 µl
Acq. Method     : C:\CHEM32\1\DATA\SUN_12 2022-11-12 08-25-33\AD-10-20.M
Last changed    : 11/12/2022 12:10:13 PM
                  (modified after loading)
Analysis Method : C:\CHEM32\1\METHODS\AY3-02-30.M
Last changed    : 11/14/2022 10:53:00 AM
                  (modified after loading)
Additional Info : Peak(s) manually integrated
```

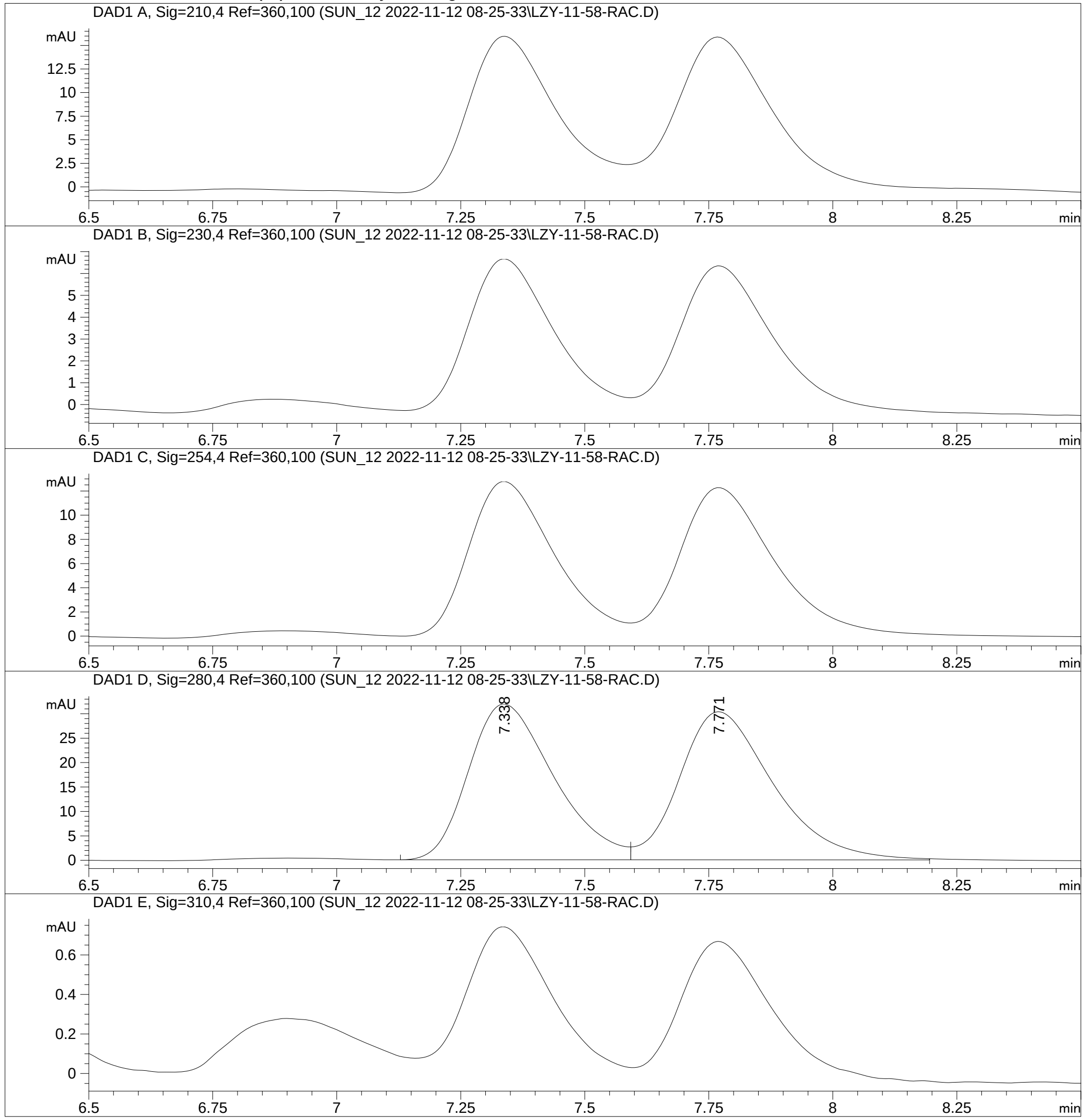


```
=====================================================================
                         Area Percent Report
=====================================================================

Sorted By             :      Signal
Multiplier            :      1.0000
Dilution              :      1.0000
Use Multiplier & Dilution Factor with ISTDs


Signal 1: DAD1 A, Sig=210,4 Ref=360,100


Signal 2: DAD1 B, Sig=230,4 Ref=360,100


Signal 3: DAD1 C, Sig=254,4 Ref=360,100


Signal 4: DAD1 D, Sig=280,4 Ref=360,100

Peak RetTime Type  Width     Area      Height     Area
  #   [min]        [min]   [mAU*s]     [mAU]        %
----|-------|----|-------|----------|----------|--------|
   1   7.338 BV    0.1836  381.44098   31.88637  48.9386
   2   7.771 VB    0.1991  397.98746   30.32810  51.0614

Totals :                   779.42844   62.21448



Signal 5: DAD1 E, Sig=310,4 Ref=360,100

=====================================================================
                          *** End of Report ***
```

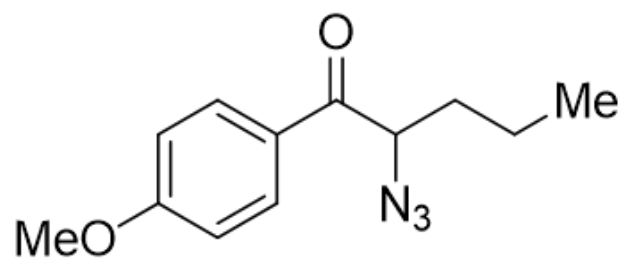

7m-Rac

===

Acq. Operator : Seq. Line : 2
Acq. Instrument : Instrument 1 Location : Vial 21
Injection Date : 9/9/2022 7:06:53 PM Inj : 1
Inj Volume : 5.000 µl
Different Inj Volume from Sequence ! Actual Inj Volume : 6.000 µl
Acq. Method : C:\CHEM32\1\DATA\SUN_12 2022-09-09 18-54-05\AD-10-20.M
Last changed : 9/9/2022 7:06:00 PM
(modified after loading)
Analysis Method : C:\CHEM32\1\METHODS\AY3-02-30.M
Last changed : 11/14/2022 10:53:00 AM
(modified after loading)
Additional Info : Peak(s) manually integrated

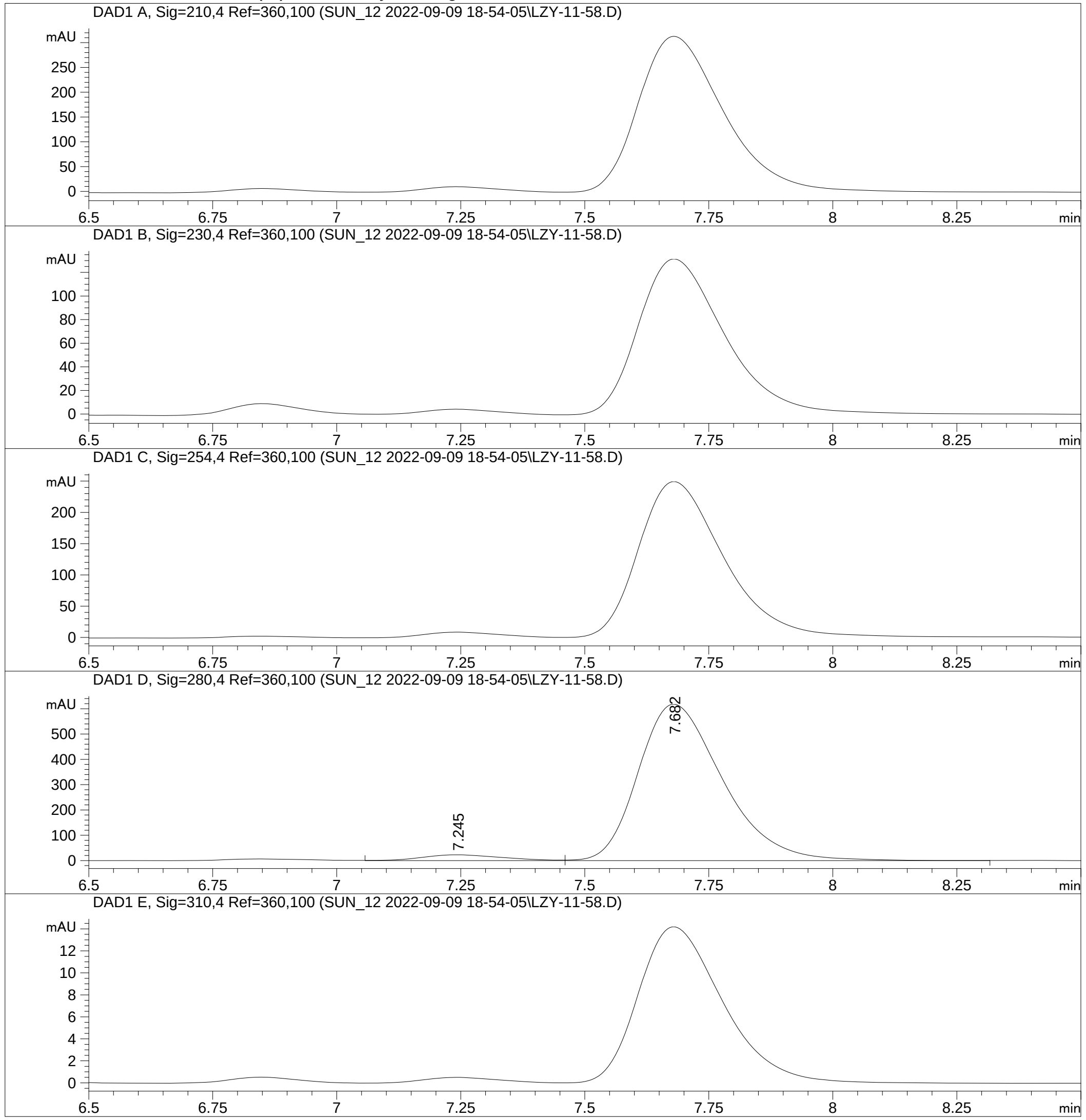

Data File C:\CHEM32\1\DATA\SUN_12 2022-09-09 18-54-05\LZY-11-58.D
Sample Name:

===

Area Percent Report

===

Sorted By : Signal
Multiplier : 1.0000
Dilution : 1.0000
Use Multiplier & Dilution Factor with ISTDs

Signal 1: DAD1 A, Sig=210,4 Ref=360,100

Signal 2: DAD1 B, Sig=230,4 Ref=360,100

Signal 3: DAD1 C, Sig=254,4 Ref=360,100

Signal 4: DAD1 D, Sig=280,4 Ref=360,100

Peak #	RetTime [min]	Type	Width [min]	Area [mAU*s]	Height [mAU]	Area %
1	7.245	VV	0.1739	255.68140	22.95938	3.3862
2	7.682	VB	0.1817	7295.03223	618.13702	96.6138

Totals : 7550.71362 641.09640

Signal 5: DAD1 E, Sig=310,4 Ref=360,100

===

*** End of Report ***

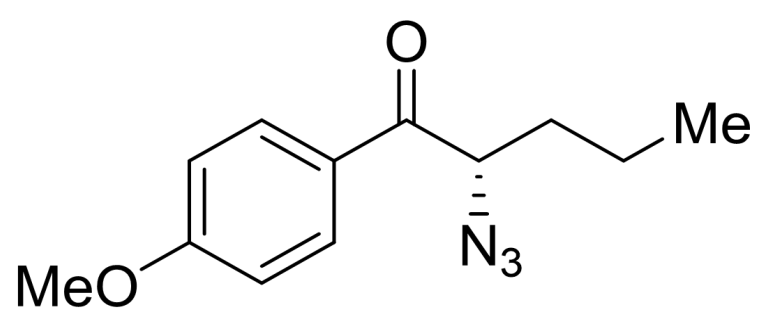

7m

Data File C:\CHEM32\1\DATA\SUN_12 2022-11-12 08-25-33\LZY-11-115-A-RAC.D
Sample Name:

===

Acq. Operator : Seq. Line : 7
Acq. Instrument : Instrument 1 Location : Vial 22
Injection Date : 11/12/2022 11:07:49 AM Inj : 1
Inj Volume : 5.000 µl
Different Inj Volume from Sequence ! Actual Inj Volume : 10.000 µl
Acq. Method : C:\CHEM32\1\DATA\SUN_12 2022-11-12 08-25-33\AD-03-20.M
Last changed : 7/2/2016 3:26:02 PM
Analysis Method : C:\CHEM32\1\METHODS\AY3-02-30.M
Last changed : 11/14/2022 10:45:40 AM
(modified after loading)
Additional Info : Peak(s) manually integrated

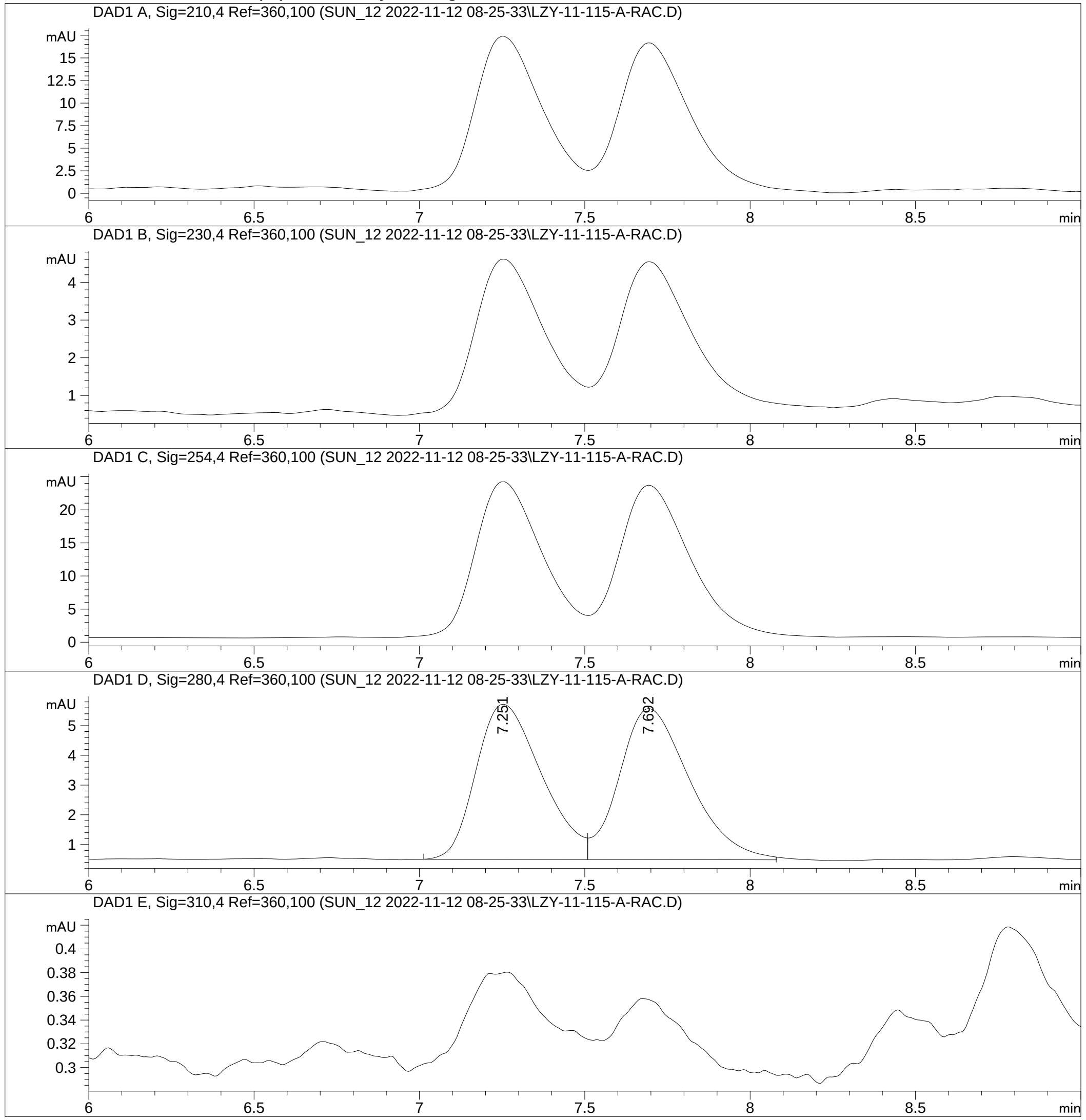

Data File C:\CHEM32\1\DATA\SUN_12 2022-11-12 08-25-33\LZY-11-115-A-RAC.D
Sample Name:

===
Area Percent Report
===

Sorted By : Signal
Multiplier : 1.0000
Dilution : 1.0000
Use Multiplier & Dilution Factor with ISTDs

Signal 1: DAD1 A, Sig=210,4 Ref=360,100

Signal 2: DAD1 B, Sig=230,4 Ref=360,100

Signal 3: DAD1 C, Sig=254,4 Ref=360,100

Signal 4: DAD1 D, Sig=280,4 Ref=360,100

Peak #	RetTime [min]	Type	Width [min]	Area [mAU*s]	Height [mAU]	Area %
1	7.251	BV	0.2128	71.96436	5.22259	48.9385
2	7.692	VB	0.2283	75.08613	5.08369	51.0615

Totals : 147.05048 10.30628

Signal 5: DAD1 E, Sig=310,4 Ref=360,100

===
*** End of Report ***

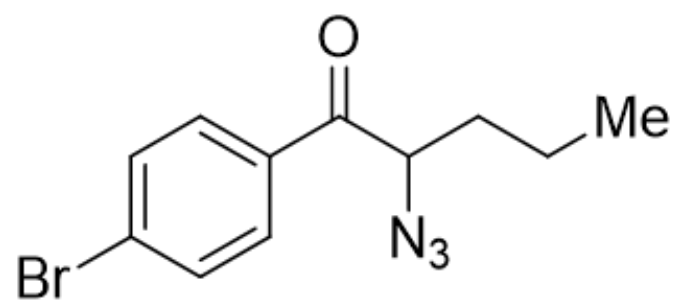

7n-Rac

```
=====================================================================
Acq. Operator   :                               Seq. Line :   2
Acq. Instrument : Instrument 1                   Location : Vial 21
Injection Date  : 10/8/2022 6:37:58 PM                Inj :   1
                                               Inj Volume : 5.000 µl
Different Inj Volume from Sequence !     Actual Inj Volume : 6.000 µl
Acq. Method     : C:\CHEM32\1\DATA\SUN_12 2022-10-08 18-25-15\AD-03-10.M
Last changed    : 10/8/2022 6:25:22 PM
                  (modified after loading)
Analysis Method : C:\CHEM32\1\METHODS\AY3-02-30.M
Last changed    : 11/14/2022 10:45:40 AM
                  (modified after loading)
Additional Info : Peak(s) manually integrated
```

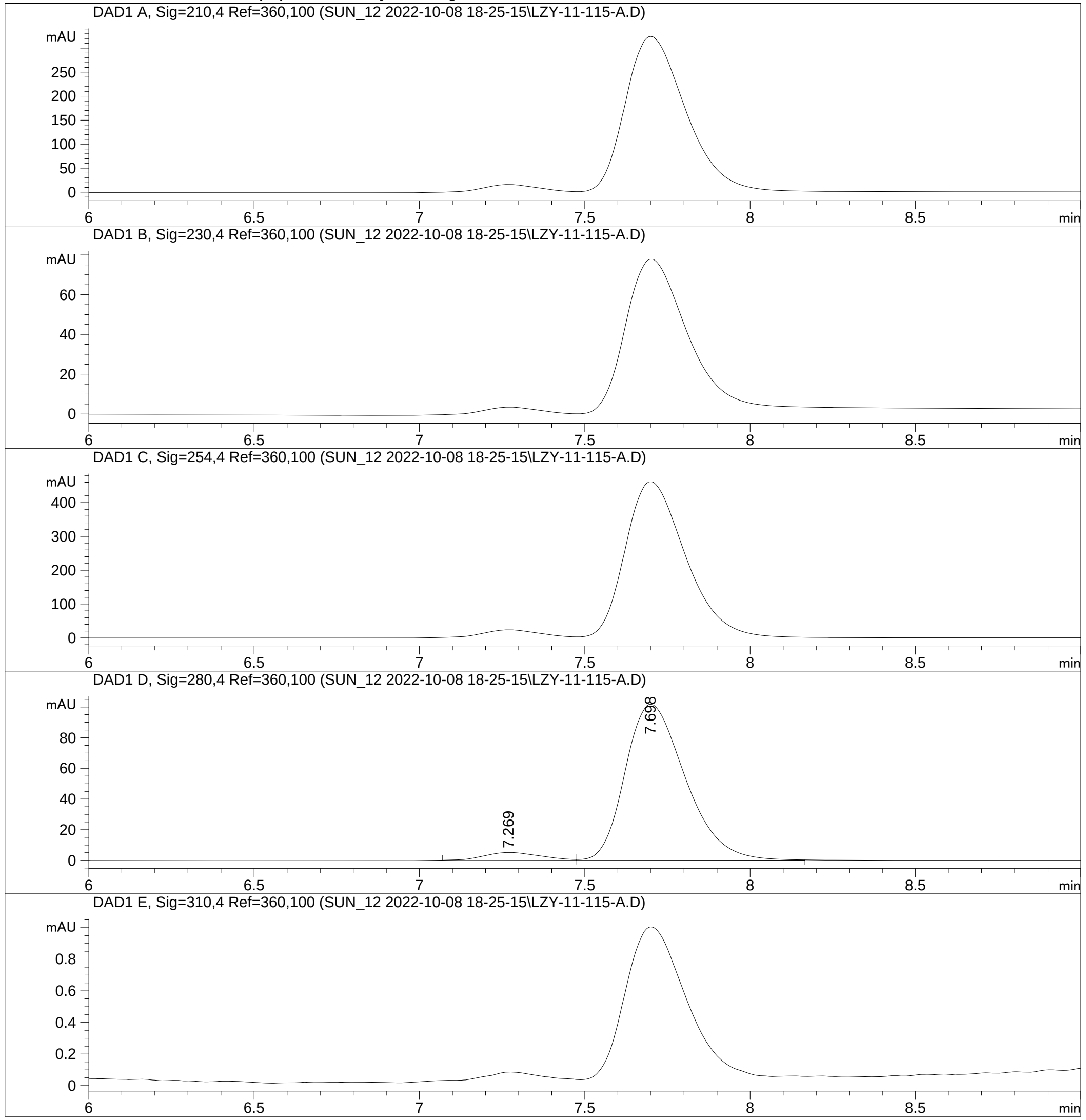


```
=====================================================================
                          Area Percent Report
=====================================================================

Sorted By             :      Signal
Multiplier            :      1.0000
Dilution              :      1.0000
Use Multiplier & Dilution Factor with ISTDs


Signal 1: DAD1 A, Sig=210,4 Ref=360,100


Signal 2: DAD1 B, Sig=230,4 Ref=360,100


Signal 3: DAD1 C, Sig=254,4 Ref=360,100


Signal 4: DAD1 D, Sig=280,4 Ref=360,100

Peak RetTime Type  Width     Area      Height     Area
  #   [min]        [min]   [mAU*s]     [mAU]        %
----|-------|----|-------|----------|----------|--------|
   1   7.269 BV    0.1871   61.73327    5.17884   4.5753
   2   7.698 VB    0.1955 1287.53149  101.82910  95.4247

Totals :                  1349.26477  107.00794



Signal 5: DAD1 E, Sig=310,4 Ref=360,100

=====================================================================
                          *** End of Report ***
```

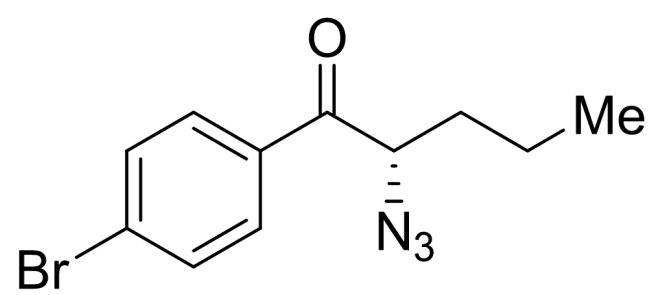

7n

```
=====================================================================
Acq. Operator   :                                Seq. Line :   8
Acq. Instrument : Instrument 1                    Location : Vial 24
Injection Date  : 11/12/2022 11:28:54 AM               Inj :   1
                                                Inj Volume : 5.000 µl
Different Inj Volume from Sequence !     Actual Inj Volume : 10.000 µl
Acq. Method     : C:\CHEM32\1\DATA\SUN_12 2022-11-12 08-25-33\AD-03-30.M
Last changed    : 11/12/2022 11:28:00 AM
                  (modified after loading)
Analysis Method : C:\CHEM32\1\METHODS\AY3-02-30.M
Last changed    : 11/14/2022 10:50:12 AM
                  (modified after loading)
Additional Info : Peak(s) manually integrated
```

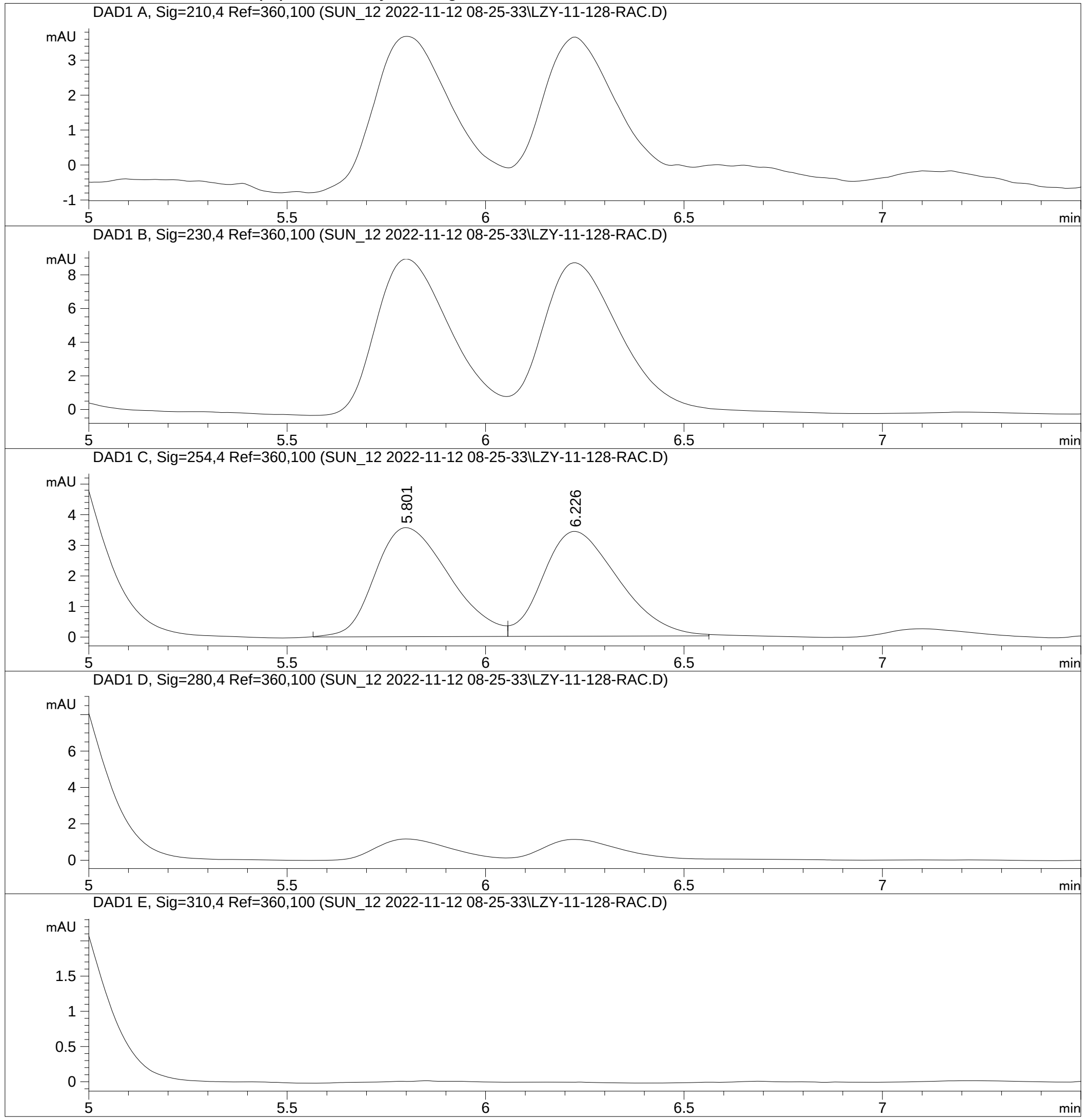

Data File C:\CHEM32\1\DATA\SUN_12 2022-11-12 08-25-33\LZY-11-128-RAC.D
Sample Name:

Area Percent Report

Sorted By : Signal
Multiplier : 1.0000
Dilution : 1.0000
Use Multiplier & Dilution Factor with ISTDs

Signal 1: DAD1 A, Sig=210,4 Ref=360,100

Signal 2: DAD1 B, Sig=230,4 Ref=360,100

Signal 3: DAD1 C, Sig=254,4 Ref=360,100

Peak #	RetTime [min]	Type	Width [min]	Area [mAU*s]	Height [mAU]	Area %
1	5.801	BV	0.2082	46.51212	3.56560	50.6177
2	6.226	VB	0.2044	45.37684	3.42766	49.3823

Totals : 91.88896 6.99326

Signal 4: DAD1 D, Sig=280,4 Ref=360,100

Signal 5: DAD1 E, Sig=310,4 Ref=360,100

*** End of Report ***

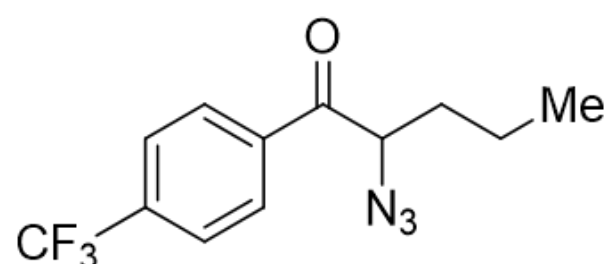

7o-Rac

Data File C:\CHEM32\1\DATA\SUN_12 2022-10-13 19-04-59\LZY-11-128.D
Sample Name:

===

Acq. Operator   :   Seq. Line : 16
Acq. Instrument : Instrument 1   Location : Vial 21
Injection Date : 10/13/2022 10:54:45 PM   Inj :   1
                                          Inj Volume : 5.000 µl
Different Inj Volume from Sequence !   Actual Inj Volume : 6.000 µl
Acq. Method   : C:\CHEM32\1\DATA\SUN_12 2022-10-13 19-04-59\AD-03-20.M
Last changed   : 7/2/2016 3:26:02 PM
Analysis Method : C:\CHEM32\1\METHODS\AY3-02-30.M
Last changed   : 11/14/2022 10:50:12 AM
                  (modified after loading)
Additional Info : Peak(s) manually integrated

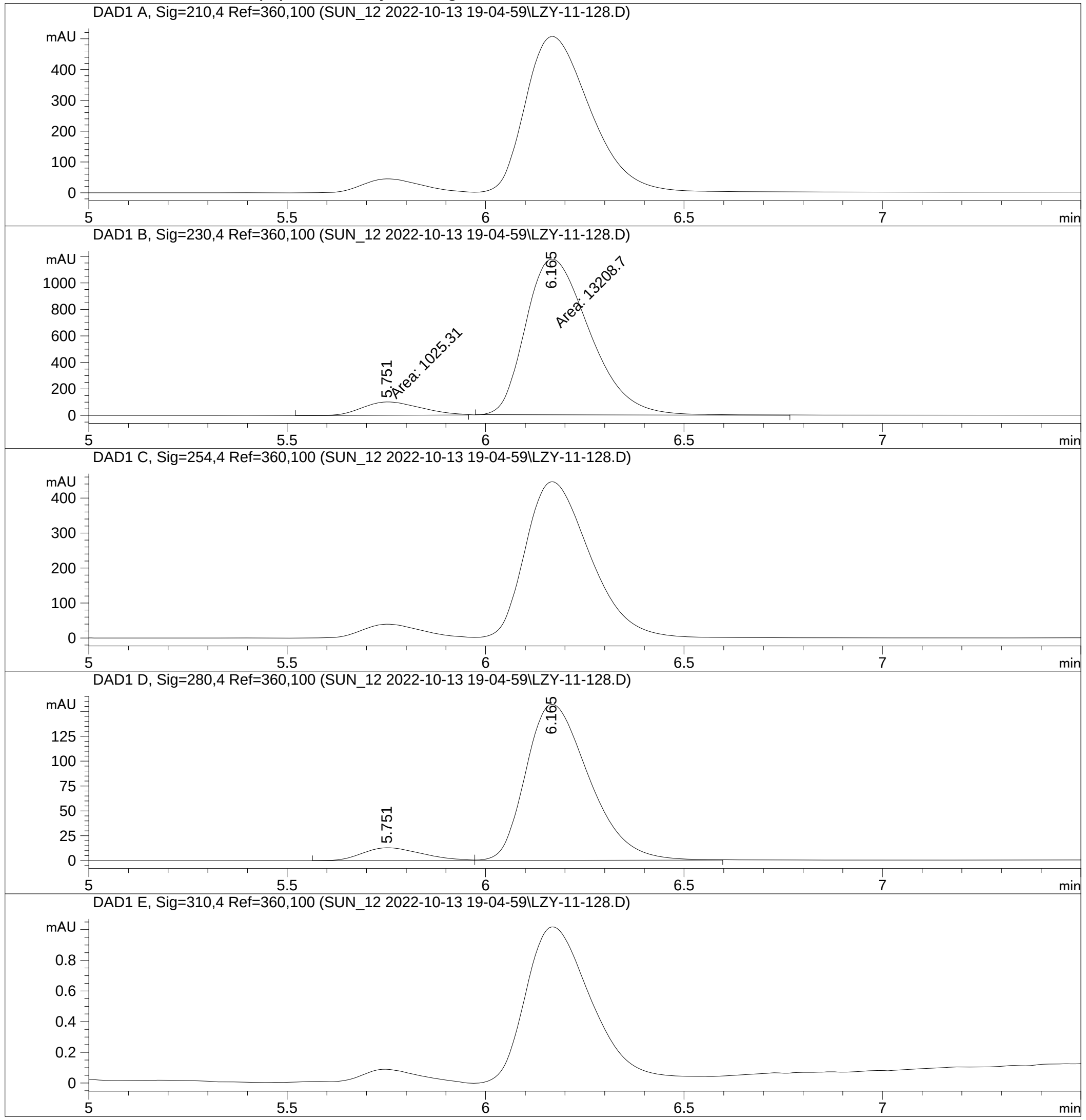

Data File C:\CHEM32\1\DATA\SUN_12 2022-10-13 19-04-59\LZY-11-128.D
Sample Name:

===
Area Percent Report
===

Sorted By   :   Signal
Multiplier   :   1.0000
Dilution   :   1.0000
Use Multiplier & Dilution Factor with ISTDs

Signal 1: DAD1 A, Sig=210,4 Ref=360,100

Signal 2: DAD1 B, Sig=230,4 Ref=360,100

Peak #	RetTime [min]	Type	Width [min]	Area [mAU*s]	Height [mAU]	Area %
1	5.751	MM	0.1704	1025.30518	100.26555	7.2032
2	6.165	MM	0.1872	1.32087e4	1176.03601	92.7968

Totals :   1.42340e4 1276.30156

Signal 3: DAD1 C, Sig=254,4 Ref=360,100

Signal 4: DAD1 D, Sig=280,4 Ref=360,100

Peak #	RetTime [min]	Type	Width [min]	Area [mAU*s]	Height [mAU]	Area %
1	5.751	BV	0.1623	132.09601	12.80821	7.0880
2	6.165	VB	0.1728	1731.56189	156.87758	92.9120

Totals :   1863.65790 169.68579

Signal 5: DAD1 E, Sig=310,4 Ref=360,100

===
*** End of Report ***

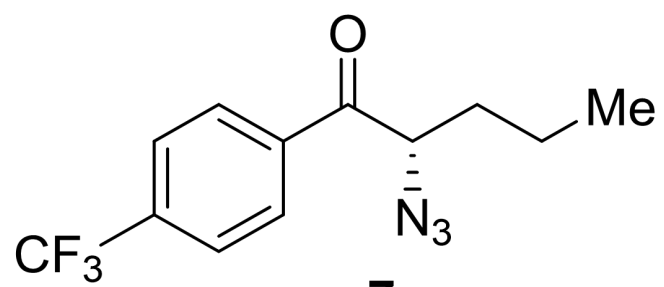

7o

```
=====================================================================
Acq. Operator   :                               Seq. Line :   4
Acq. Instrument : Instrument 1                   Location : Vial 23
Injection Date  : 9/23/2022 11:17:24 AM               Inj :   1
                                               Inj Volume : 5.000 µl
Different Inj Volume from Sequence !     Actual Inj Volume : 9.000 µl
Acq. Method     : C:\CHEM32\1\DATA\SUN_12 2022-09-23 09-59-53\AS-05-20.M
Last changed    : 9/23/2022 11:16:13 AM
                  (modified after loading)
Analysis Method : C:\CHEM32\1\METHODS\AY3-02-30.M
Last changed    : 11/11/2022 4:09:30 PM
                  (modified after loading)
Additional Info : Peak(s) manually integrated
```

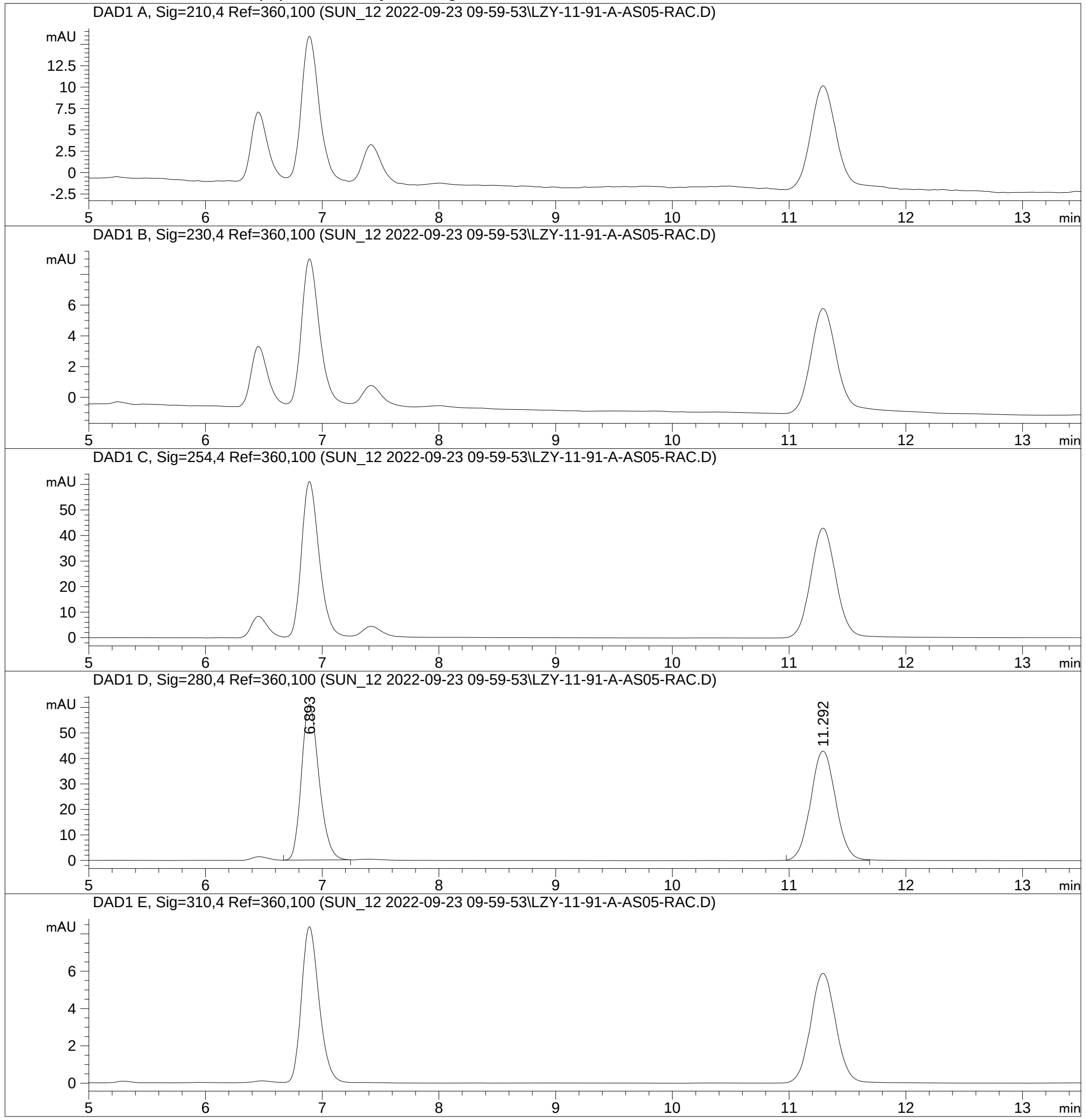

Data File C:\CHEM32\1\DATA\SUN_12 2022-09-23 09-59-53\LZY-11-91-A-AS05-RAC.D
Sample Name:

```
=====================================================================
                         Area Percent Report
=====================================================================

Sorted By             :      Signal
Multiplier            :      1.0000
Dilution              :      1.0000
Use Multiplier & Dilution Factor with ISTDs


Signal 1: DAD1 A, Sig=210,4 Ref=360,100


Signal 2: DAD1 B, Sig=230,4 Ref=360,100


Signal 3: DAD1 C, Sig=254,4 Ref=360,100

Peak RetTime Type  Width     Area      Height     Area
  #   [min]        [min]   [mAU*s]     [mAU]        %
----|-------|----|-------|----------|----------|--------|
   1   3.189 BB    0.0855    7.53351    1.19613  14.6135
   2  16.649 BB    0.3348   44.01829    2.04738  85.3865

Totals :                    51.55180    3.24351



Signal 4: DAD1 D, Sig=280,4 Ref=360,100

Peak RetTime Type  Width     Area      Height     Area
  #   [min]        [min]   [mAU*s]     [mAU]        %
----|-------|----|-------|----------|----------|--------|
   1   6.893 VB    0.1613  636.45392   61.19549  49.8786
   2  11.292 BB    0.2318  639.55121   42.92744  50.1214

Totals :                  1276.00513  104.12294



Signal 5: DAD1 E, Sig=310,4 Ref=360,100

=====================================================================
                          *** End of Report ***
```

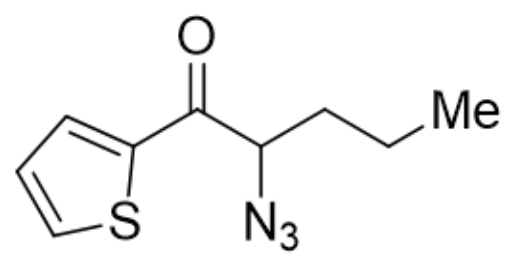

7p-Rac

Data File C:\CHEM32\1\DATA\SUN_12 2022-09-25 18-36-26\LZY-11-91-A.D
Sample Name:

```
=====================================================================
Acq. Operator   :                              Seq. Line :  12
Acq. Instrument : Instrument 1                  Location : Vial 24
Injection Date  : 9/25/2022 9:33:54 PM               Inj :   1
                                              Inj Volume : 5.000 µl
Acq. Method     : C:\CHEM32\1\DATA\SUN_12 2022-09-25 18-36-26\AS-05-20.M
Last changed    : 9/25/2022 9:33:03 PM
                  (modified after loading)
Analysis Method : C:\CHEM32\1\METHODS\AY3-02-30.M
Last changed    : 11/11/2022 4:09:30 PM
                  (modified after loading)
Additional Info : Peak(s) manually integrated
```

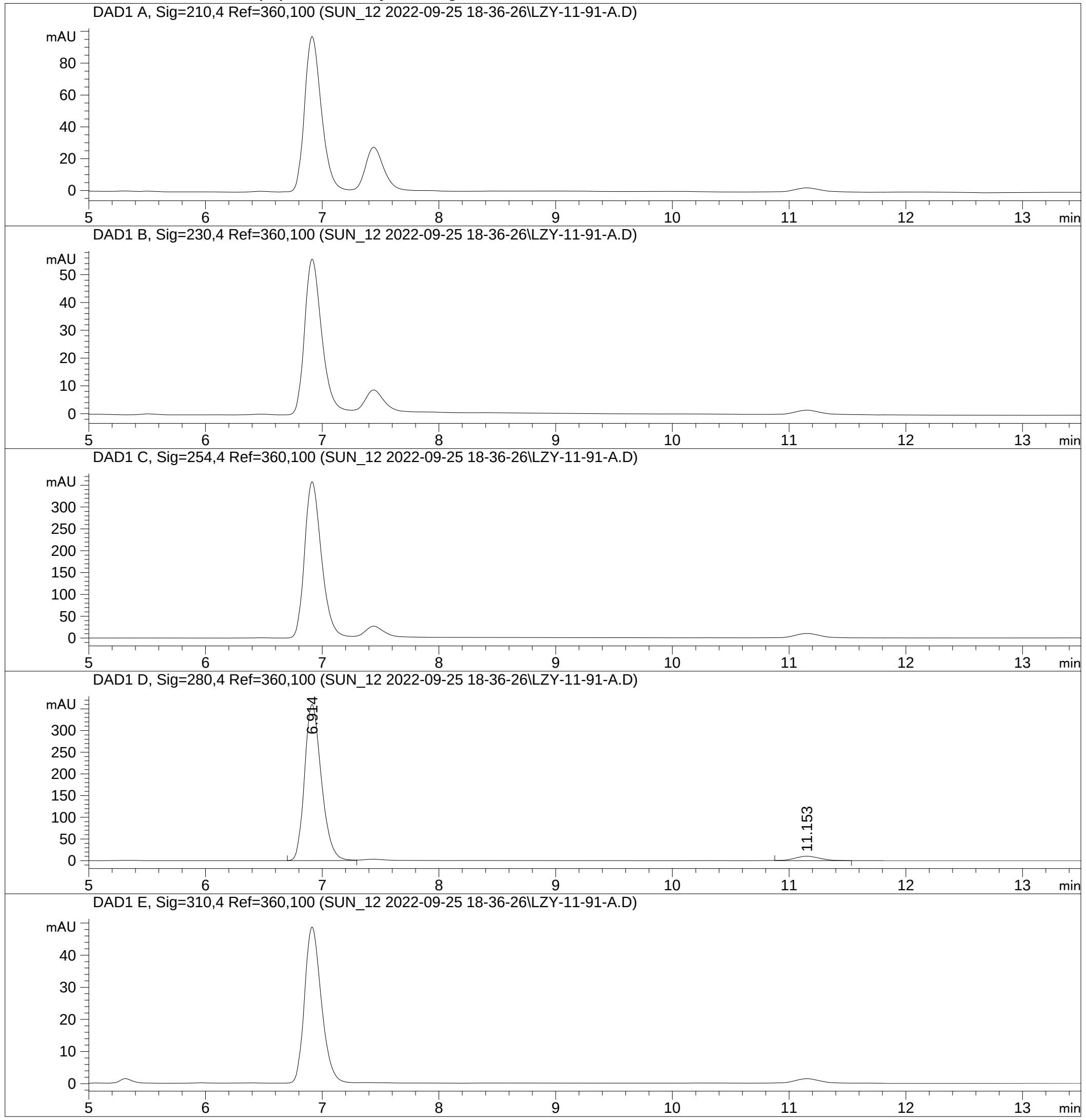

Data File C:\CHEM32\1\DATA\SUN_12 2022-09-25 18-36-26\LZY-11-91-A.D
Sample Name:

```
=====================================================================
                         Area Percent Report
=====================================================================

Sorted By             :      Signal
Multiplier            :      1.0000
Dilution              :      1.0000
Use Multiplier & Dilution Factor with ISTDs


Signal 1: DAD1 A, Sig=210,4 Ref=360,100


Signal 2: DAD1 B, Sig=230,4 Ref=360,100


Signal 3: DAD1 C, Sig=254,4 Ref=360,100


Signal 4: DAD1 D, Sig=280,4 Ref=360,100
```

Peak #	RetTime [min]	Type	Width [min]	Area [mAU*s]	Height [mAU]	Area %
1	6.914	BV	0.1543	3592.19141	360.49606	96.0690
2	11.153	BB	0.2251	146.98732	10.14066	3.9310
Totals :				3739.17873	370.63672	

```
Signal 5: DAD1 E, Sig=310,4 Ref=360,100

=====================================================================
                        *** End of Report ***
```

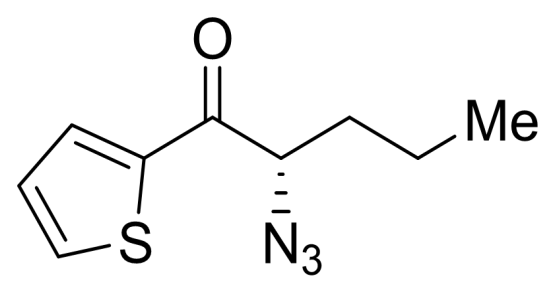

7p

Data File C:\Users\P...\ChemStation\1\Data\SUN\SUN 2023-04-11 15-47-20\OnlineEdited--010.D
Sample Name: lzy-11-81-Rac

```
=====================================================================
Acq. Operator   : SYSTEM                        Seq. Line :  10
Sample Operator : SYSTEM
Acq. Instrument : HPLC                           Location :   P2-A-09
Injection Date  : 11/4/2023 8:00:51 pm                Inj :   1
                                               Inj Volume : 2.000 µl
Different Inj Volume from Sample Entry!  Actual Inj Volume : 12.000 µl
Acq. Method     : C:\Users\Public\Documents\ChemStation\1\Data\SUN\SUN 2023-04-11 15-47-20
                  \AD3-01-30.M
Last changed    : 11/4/2023 7:55:03 pm by SYSTEM
Analysis Method : C:\Users\Public\Documents\ChemStation\1\Data\SUN\SUN 2023-04-11 15-47-20
                  \AD3-01-30.M (Sequence Method)
Last changed    : 25/8/2023 4:50:14 pm by SYSTEM
                  (modified after loading)
Additional Info : Peak(s) manually integrated
```

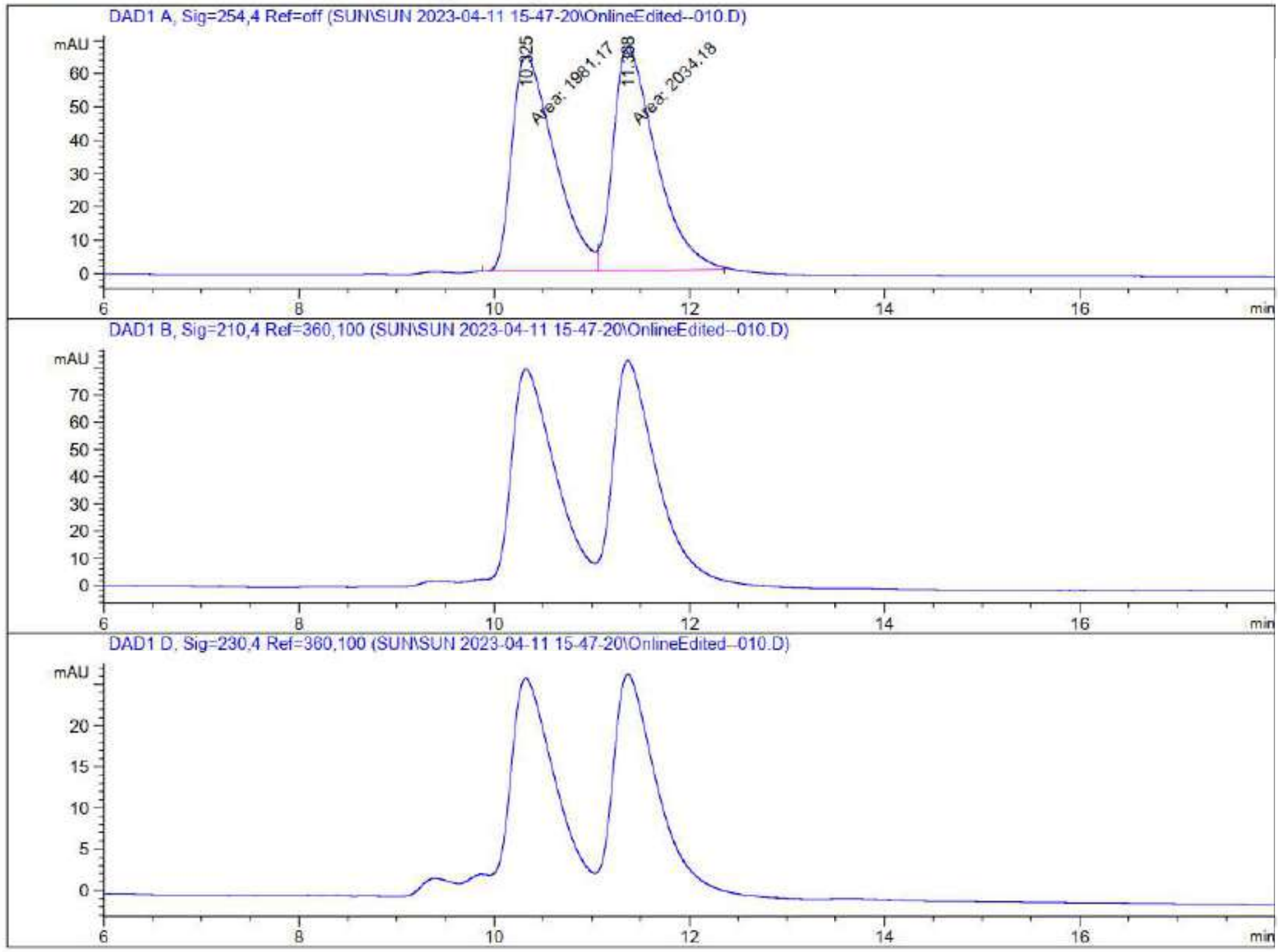

Data File C:\Users\P...\ChemStation\1\Data\SUN\SUN 2023-04-11 15-47-20\OnlineEdited--010.D
Sample Name: lzy-11-81-Rac

Area Percent Report

```
Sorted By             :      Signal
Multiplier            :      1.0000
Dilution              :      1.0000
Use Multiplier & Dilution Factor with ISTDs
```

Signal 1: DAD1 A, Sig=254,4 Ref=off

Peak #	RetTime [min]	Type	Width [min]	Area [mAU*s]	Height [mAU]	Area %
1	10.325	MM	0.5093	1981.16895	64.83823	49.3399
2	11.368	MM	0.5024	2034.18164	67.48161	50.6601

Totals : 4015.35059 132.31983

Signal 2: DAD1 B, Sig=210,4 Ref=360,100

Signal 3: DAD1 D, Sig=230,4 Ref=360,100

*** End of Report ***

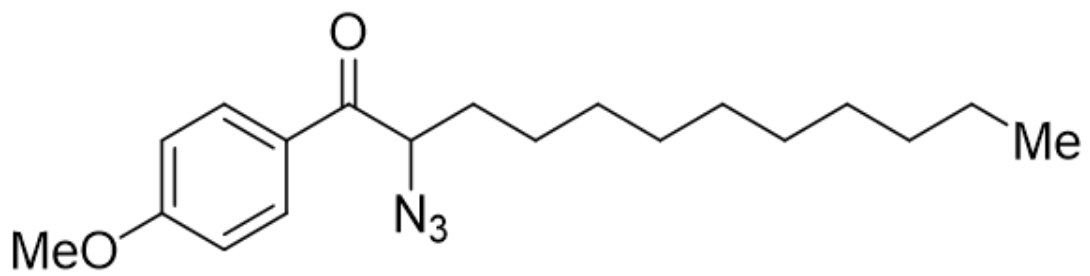

7q-Rac

===

Acq. Operator : SYSTEM Seq. Line : 11
Sample Operator : SYSTEM
Acq. Instrument : HPLC Location : P2-A-10
Injection Date : 11/4/2023 8:21:50 pm Inj : 1
Inj Volume : 2.000 µl
Different Inj Volume from Sample Entry! Actual Inj Volume : 3.000 µl
Acq. Method : C:\Users\Public\Documents\ChemStation\1\Data\SUN\SUN 2023-04-11 15-47-20\AD3-01-30.M
Last changed : 11/4/2023 7:55:03 pm by SYSTEM
Analysis Method : C:\Users\Public\Documents\ChemStation\1\Data\SUN\SUN 2023-04-11 15-47-20\AD3-01-30.M (Sequence Method)
Last changed : 25/8/2023 4:50:14 pm by SYSTEM
(modified after loading)
Additional Info : Peak(s) manually integrated

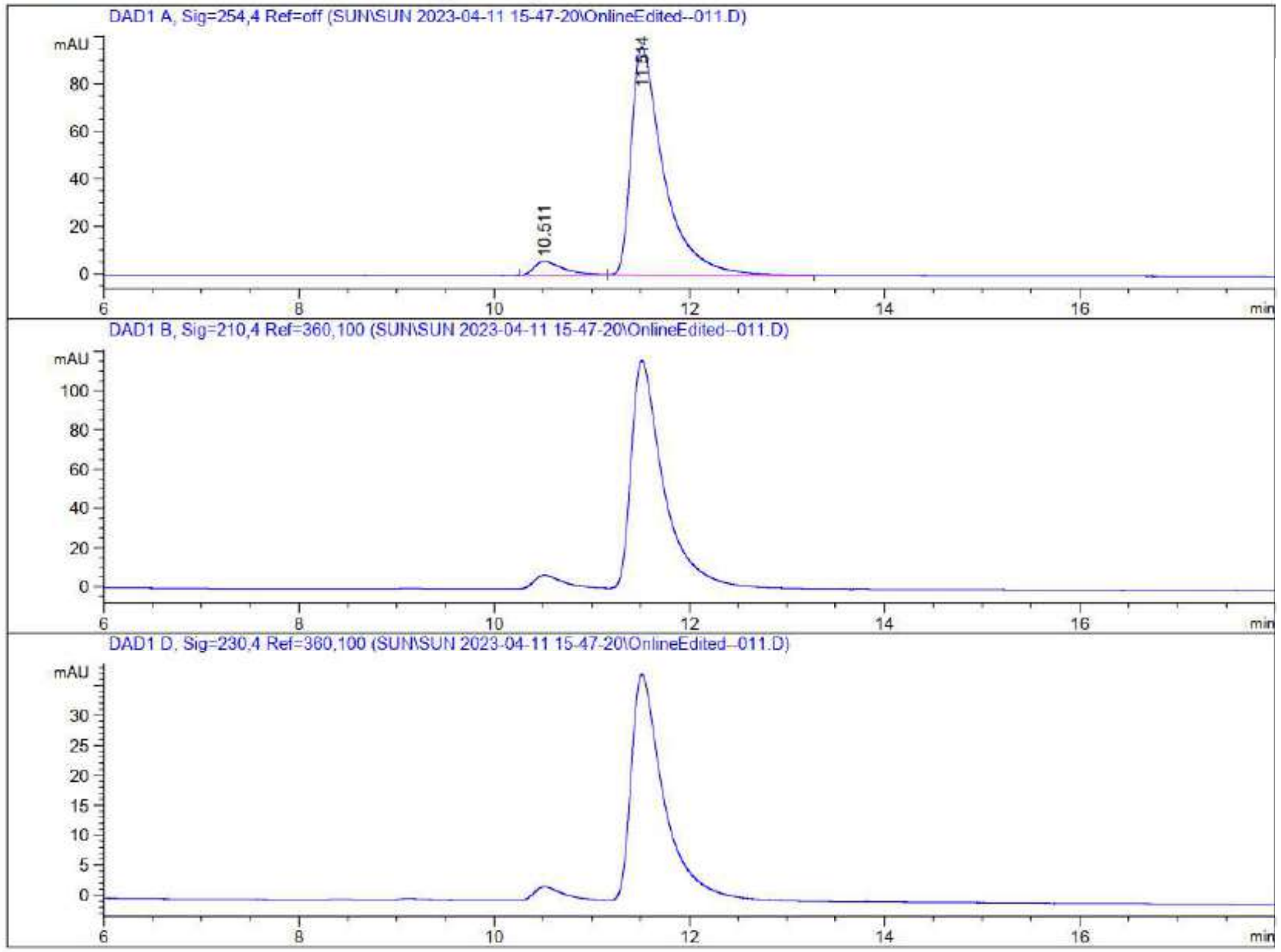

Data File C:\Users\P...\ChemStation\1\Data\SUN\SUN 2023-04-11 15-47-20\OnlineEdited--011.D
Sample Name: lzy-11-81

===

Area Percent Report

===

Sorted By : Signal
Multiplier : 1.0000
Dilution : 1.0000
Use Multiplier & Dilution Factor with ISTDs

Signal 1: DAD1 A, Sig=254,4 Ref=off

Peak #	RetTime [min]	Type	Width [min]	Area [mAU*s]	Height [mAU]	Area %
1	10.511	BB	0.2506	116.52908	5.75746	4.9927
2	11.514	BB	0.3371	2217.46631	96.15375	95.0073

Totals : 2333.99539 101.91120

Signal 2: DAD1 B, Sig=210,4 Ref=360,100

Signal 3: DAD1 D, Sig=230,4 Ref=360,100

===

*** End of Report ***

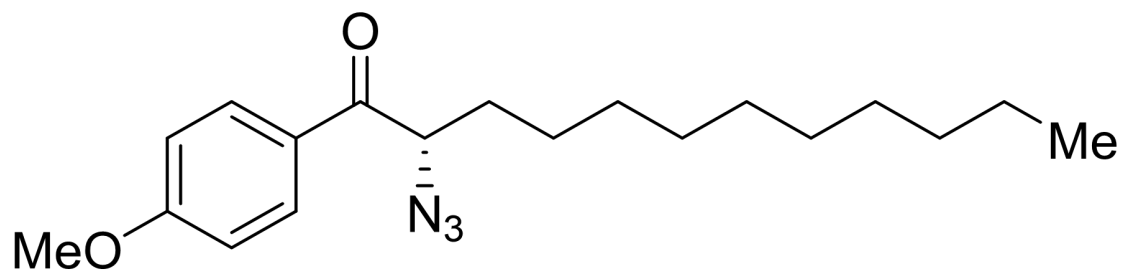

7q

```
=====================================================================
Acq. Operator   :                              Seq. Line :   2
Acq. Instrument : Instrument 1                  Location : Vial 21
Injection Date  : 10/19/2022 8:08:18 PM              Inj :   1
                                              Inj Volume : 5.000 µl
Different Inj Volume from Sequence !     Actual Inj Volume : 10.000 µl
Acq. Method     : C:\CHEM32\1\DATA\SUN_12 2022-10-19 19-55-30\AD-15-50.M
Last changed    : 10/19/2022 8:07:25 PM
                  (modified after loading)
Analysis Method : C:\CHEM32\1\METHODS\AY3-02-30.M
Last changed    : 11/11/2022 5:47:32 PM
                  (modified after loading)
Additional Info : Peak(s) manually integrated
```

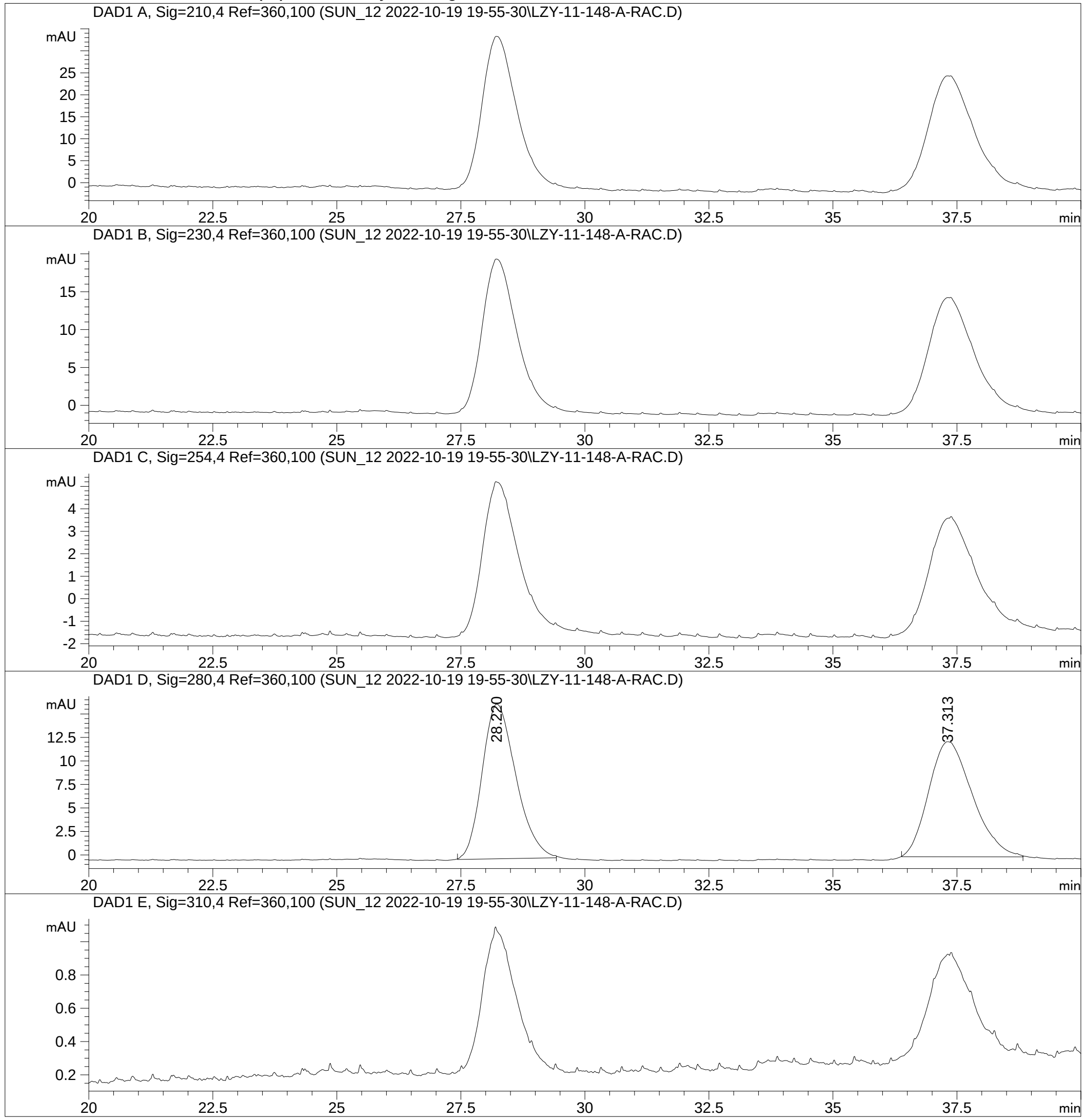


```
=====================================================================
                         Area Percent Report
=====================================================================

Sorted By             :      Signal
Multiplier            :      1.0000
Dilution              :      1.0000
Use Multiplier & Dilution Factor with ISTDs


Signal 1: DAD1 A, Sig=210,4 Ref=360,100


Signal 2: DAD1 B, Sig=230,4 Ref=360,100


Signal 3: DAD1 C, Sig=254,4 Ref=360,100


Signal 4: DAD1 D, Sig=280,4 Ref=360,100

Peak RetTime Type  Width     Area      Height     Area
  #   [min]        [min]   [mAU*s]     [mAU]        %
----|-------|----|-------|----------|----------|--------|
   1  28.220 BB    0.6577  777.43359   16.44120  50.8621
   2  37.313 BB    0.7995  751.07770   12.24603  49.1379

Totals :                   1528.51129   28.68723



Signal 5: DAD1 E, Sig=310,4 Ref=360,100

=====================================================================
                          *** End of Report ***
```

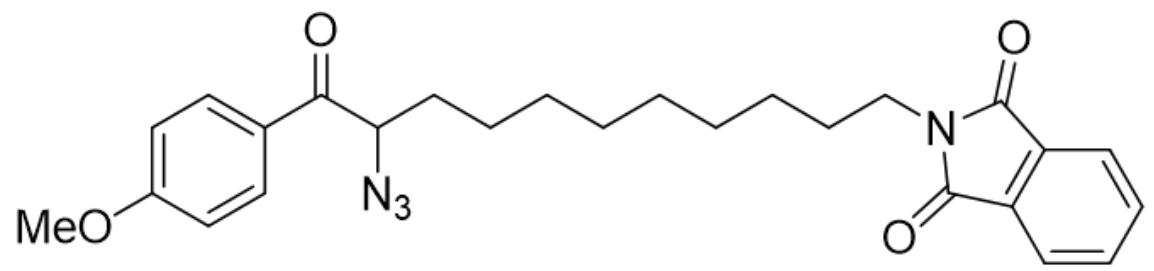

7r-Rac

===

Acq. Operator : Seq. Line : 2
Acq. Instrument : Instrument 1 Location : Vial 21
Injection Date : 10/20/2022 5:29:00 PM Inj : 1
Inj Volume : 5.000 µl
Different Inj Volume from Sequence ! Actual Inj Volume : 6.000 µl
Acq. Method : C:\CHEM32\1\DATA\SUN_12 2022-10-20 17-16-11\AD-15-40.M
Last changed : 10/20/2022 5:28:08 PM
(modified after loading)
Analysis Method : C:\CHEM32\1\METHODS\AY3-02-30.M
Last changed : 11/11/2022 5:47:32 PM
(modified after loading)
Additional Info : Peak(s) manually integrated

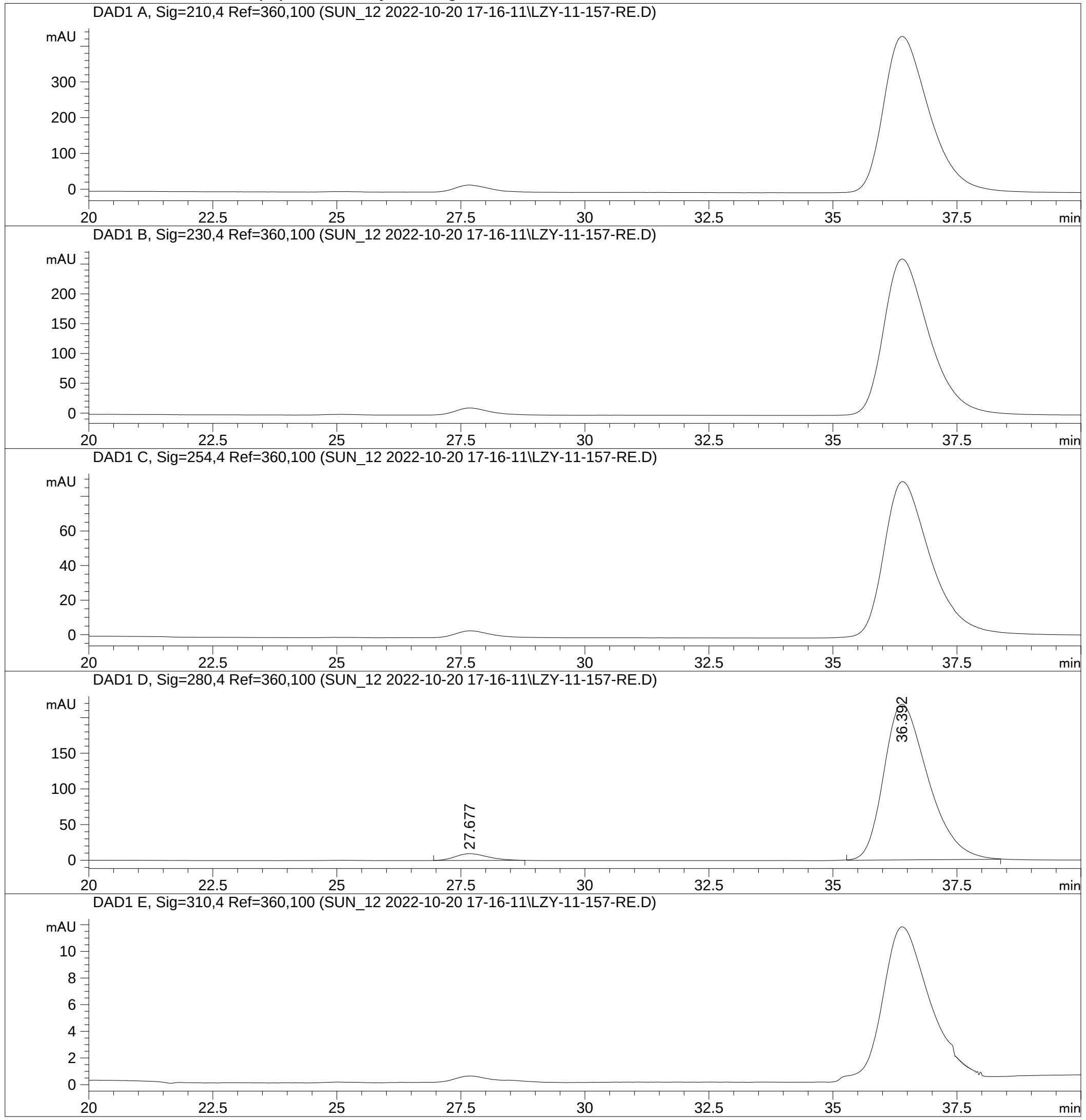

Data File C:\CHEM32\1\DATA\SUN_12 2022-10-20 17-16-11\LZY-11-157-RE.D
Sample Name:

===

Area Percent Report

===

Sorted By : Signal
Multiplier : 1.0000
Dilution : 1.0000
Use Multiplier & Dilution Factor with ISTDs

Signal 1: DAD1 A, Sig=210,4 Ref=360,100

Signal 2: DAD1 B, Sig=230,4 Ref=360,100

Signal 3: DAD1 C, Sig=254,4 Ref=360,100

Signal 4: DAD1 D, Sig=280,4 Ref=360,100

Peak #	RetTime [min]	Type	Width [min]	Area [mAU*s]	Height [mAU]	Area %
1	27.677	BB	0.6902	434.86282	9.53254	3.1090
2	36.392	BB	0.9499	1.35522e4	218.40880	96.8910

Totals : 1.39871e4 227.94134

Signal 5: DAD1 E, Sig=310,4 Ref=360,100

===

*** End of Report ***

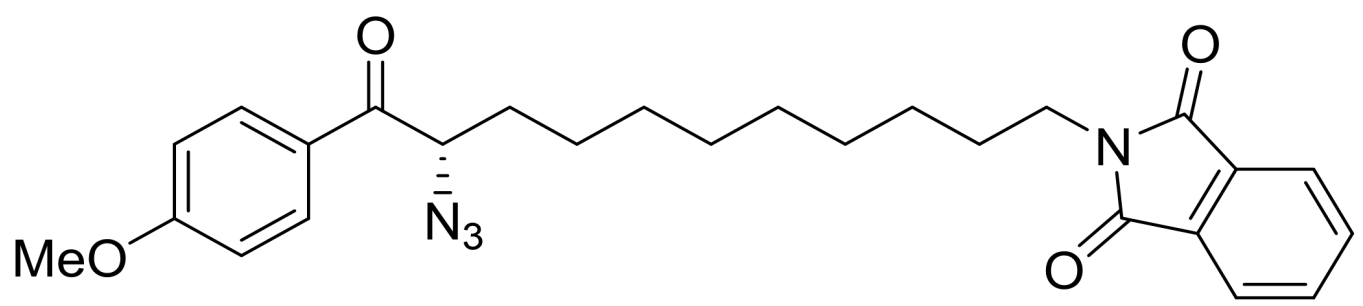

7r

Data File C:\CHEM32\1\DATA\SUN_12 2022-11-16 09-55-30\LZY-11-174-RAC.D
Sample Name:

```
=====================================================================
Acq. Operator   :                               Seq. Line :   5
Acq. Instrument : Instrument 1                   Location : Vial 22
Injection Date  : 11/16/2022 10:51:59 AM              Inj :   1
                                               Inj Volume : 5.000 µl
Different Inj Volume from Sequence !     Actual Inj Volume : 10.000 µl
Acq. Method     : C:\CHEM32\1\DATA\SUN_12 2022-11-16 09-55-30\AD-15-30.M
Last changed    : 11/16/2022 10:51:06 AM
                  (modified after loading)
Analysis Method : C:\CHEM32\1\METHODS\AY3-02-30.M
Last changed    : 11/16/2022 11:06:04 AM
                  (modified after loading)
Additional Info : Peak(s) manually integrated
```

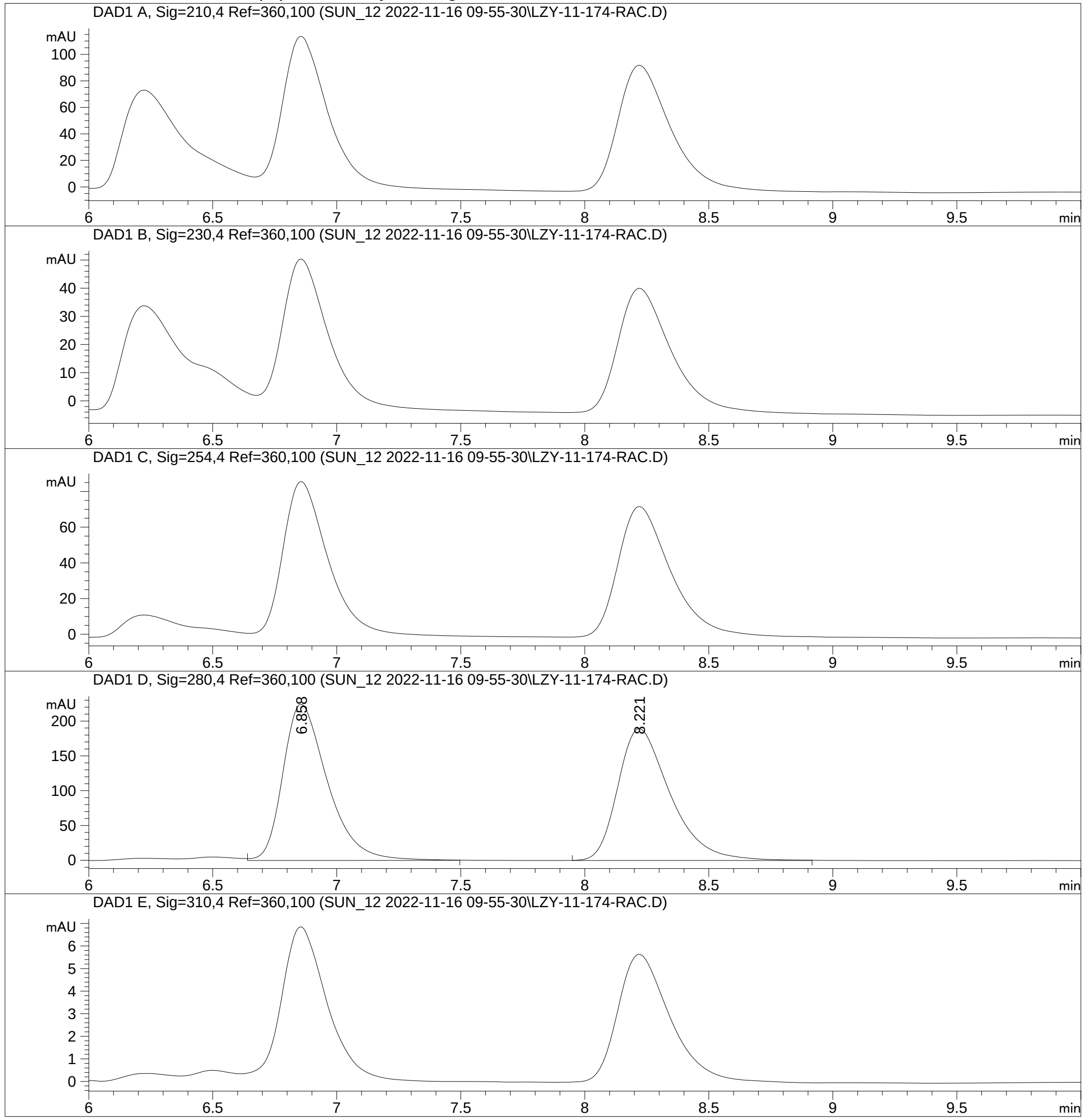

Data File C:\CHEM32\1\DATA\SUN_12 2022-11-16 09-55-30\LZY-11-174-RAC.D
Sample Name:

```
=====================================================================
                         Area Percent Report
=====================================================================

Sorted By             :      Signal
Multiplier            :      1.0000
Dilution              :      1.0000
Use Multiplier & Dilution Factor with ISTDs


Signal 1: DAD1 A, Sig=210,4 Ref=360,100


Signal 2: DAD1 B, Sig=230,4 Ref=360,100


Signal 3: DAD1 C, Sig=254,4 Ref=360,100


Signal 4: DAD1 D, Sig=280,4 Ref=360,100

Peak RetTime Type  Width     Area      Height     Area
  #   [min]        [min]   [mAU*s]     [mAU]        %
----|-------|----|-------|----------|----------|--------|
   1   6.858 VB    0.1899 2808.29663  224.55600  50.2643
   2   8.221 BB    0.2212 2778.76440  189.39059  49.7357

Totals :                  5587.06104  413.94659



Signal 5: DAD1 E, Sig=310,4 Ref=360,100

=====================================================================
                          *** End of Report ***
```

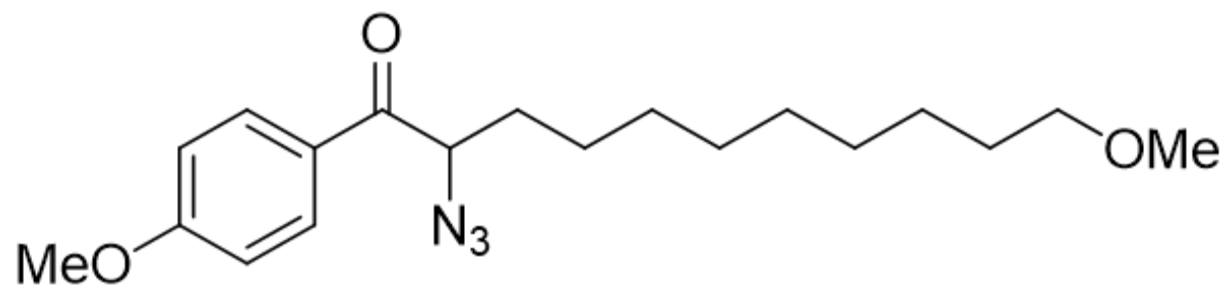

7s-Rac

Data File C:\CHEM32\1\DATA\SUN_12 2022-10-29 21-47-40\LZY-11-174.D
Sample Name:

```
=====================================================================
Acq. Operator   :                              Seq. Line :   2
Acq. Instrument : Instrument 1                  Location : Vial 21
Injection Date  : 10/29/2022 10:00:27 PM             Inj :   1
                                              Inj Volume : 5.000 µl
Different Inj Volume from Sequence !     Actual Inj Volume : 7.000 µl
Acq. Method     : C:\CHEM32\1\DATA\SUN_12 2022-10-29 21-47-40\AD-15-10.M
Last changed    : 7/23/2020 12:42:53 AM
Analysis Method : C:\CHEM32\1\METHODS\AY3-02-30.M
Last changed    : 11/14/2022 10:55:59 PM
                  (modified after loading)
Additional Info : Peak(s) manually integrated
```

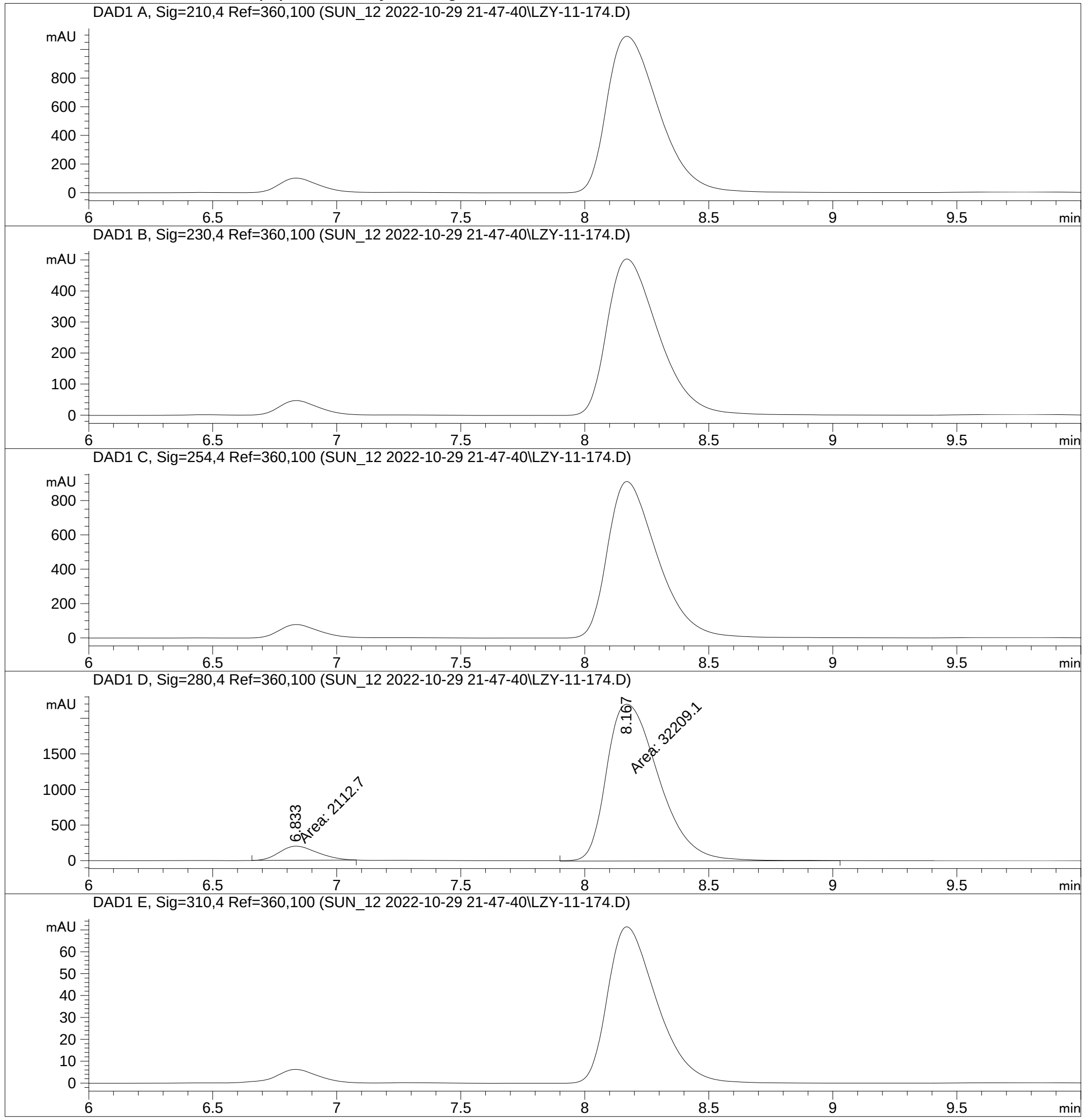

Data File C:\CHEM32\1\DATA\SUN_12 2022-10-29 21-47-40\LZY-11-174.D
Sample Name:

```
=====================================================================
                         Area Percent Report
=====================================================================

Sorted By             :      Signal
Multiplier            :      1.0000
Dilution              :      1.0000
Use Multiplier & Dilution Factor with ISTDs


Signal 1: DAD1 A, Sig=210,4 Ref=360,100


Signal 2: DAD1 B, Sig=230,4 Ref=360,100


Signal 3: DAD1 C, Sig=254,4 Ref=360,100


Signal 4: DAD1 D, Sig=280,4 Ref=360,100
```

Peak #	RetTime [min]	Type	Width [min]	Area [mAU*s]	Height [mAU]	Area %
1	6.833	MM	0.1772	2112.69849	198.74150	6.1556
2	8.167	MM	0.2435	3.22091e4	2204.39014	93.8444

Totals : 3.43218e4 2403.13164

Signal 5: DAD1 E, Sig=310,4 Ref=360,100

===
*** End of Report ***

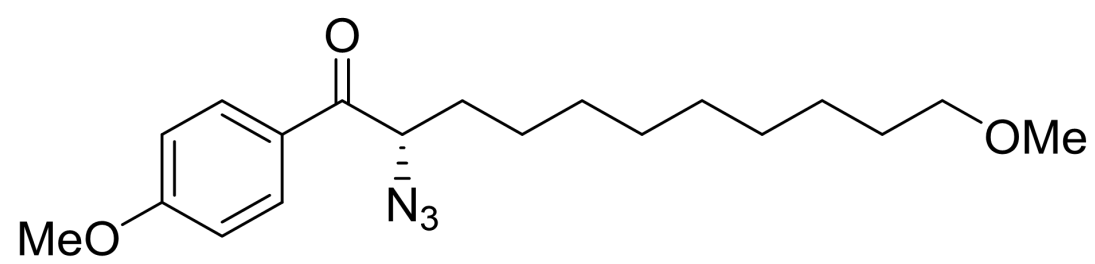

7s

===

Acq. Operator : SYSTEM Seq. Line : 16
Sample Operator : SYSTEM
Acq. Instrument : HPLC Location : P1-C-01
Injection Date : 4/7/2026 8:23:50 pm Inj : 1
Inj Volume : 2.000 µl
Different Inj Volume from Sample Entry! Actual Inj Volume : 15.000 µl
Acq. Method : C:\Users\Public\Documents\ChemStation\1\Data\SUN\SUN 2026-07-04 15-32-47\IBN3-10-40.M
Last changed : 23/7/2025 10:18:50 pm by SYSTEM
Analysis Method : C:\Users\Public\Documents\ChemStation\1\Data\SUN\SUN 2026-07-04 15-32-47\IBN3-10-40.M (Sequence Method)
Last changed : 7/7/2026 7:47:10 pm by SYSTEM
(modified after loading)
Additional Info : Peak(s) manually integrated

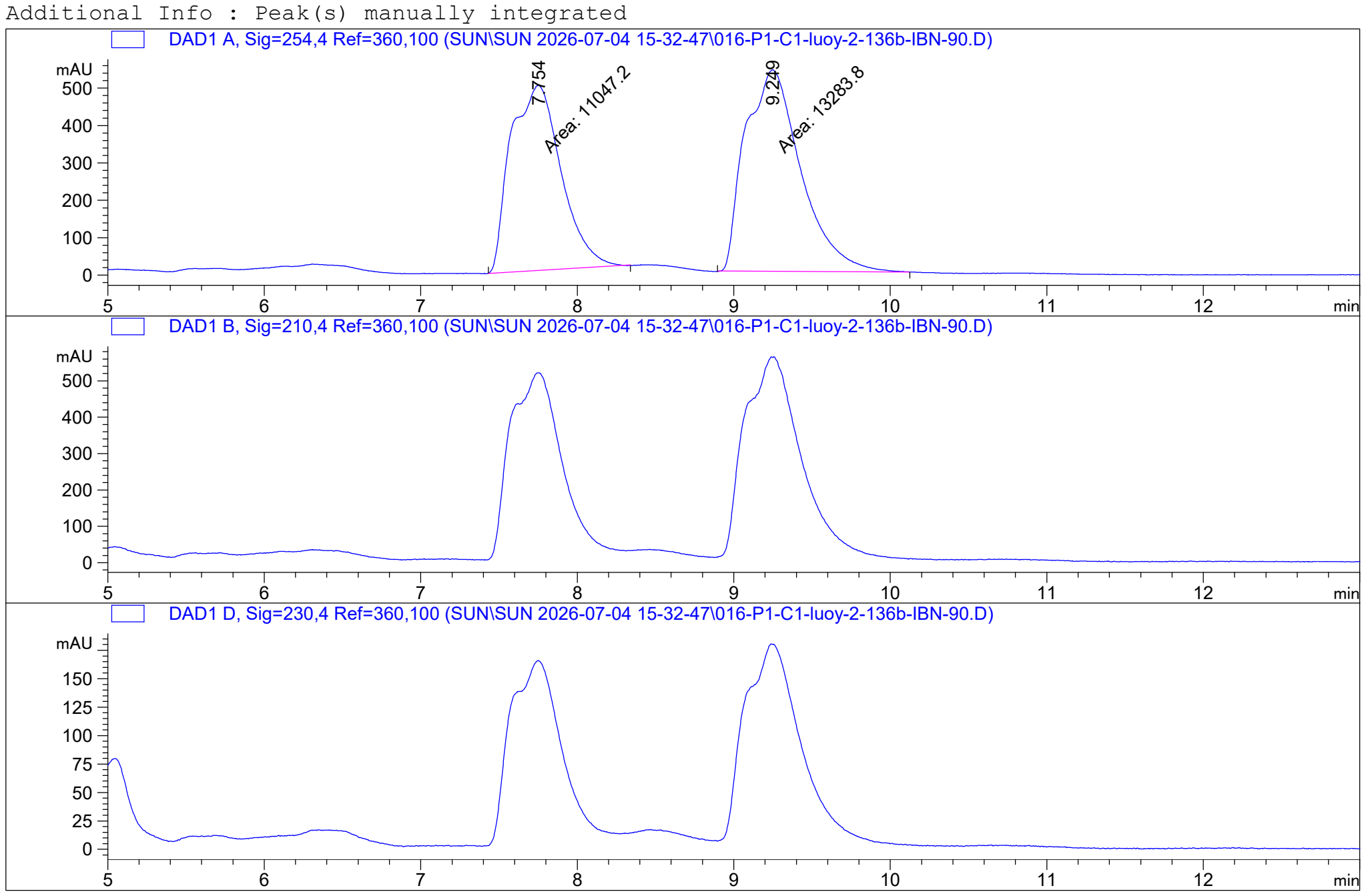

===

Area Percent Report

===

Sorted By : Signal
Multiplier : 1.0000
Dilution : 1.0000
Use Multiplier & Dilution Factor with ISTDs

Data File C:\Users\P...n\1\Data\SUN\SUN 2026-07-04 15-32-47\016-P1-C1-luoy-2-136b-IBN-90.D
Sample Name: luoy-2-136b-IBN-90

Signal 1: DAD1 A, Sig=254,4 Ref=360,100

Peak #	RetTime [min]	Type	Width [min]	Area [mAU*s]	Height [mAU]	Area %
1	7.754	MM	0.3723	1.10472e4	494.59811	45.4037
2	9.249	MM	0.4110	1.32838e4	538.71936	54.5963
Totals :				2.43310e4	1033.31747	

Signal 2: DAD1 B, Sig=210,4 Ref=360,100

Peak #	RetTime [min]	Type	Width [min]	Area [mAU*s]	Height [mAU]	Area %
1	22.855	VB	0.0280	5.22763	2.34136	49.8082
2	37.903	VV	0.0307	5.26788	2.13774	50.1918
Totals :				10.49551	4.47910	

Signal 3: DAD1 D, Sig=230,4 Ref=360,100

===

*** End of Report ***

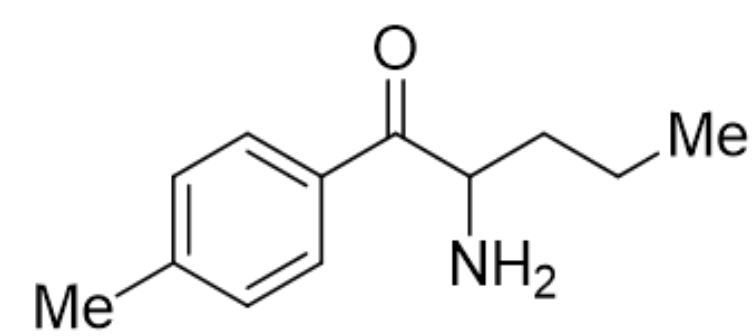

8a-Rac

Data File C:\Users\Public\Documents\ChemStation\1\Data\Snapshot.d
Sample Name:

```
=====================================================================
Acq. Operator   : SYSTEM                        Seq. Line :  24
Sample Operator : SYSTEM
Acq. Instrument : HPLC                           Location :   P1-C-02
Injection Date  : 7/7/2026 8:12:30 pm
                                               Inj Volume : 2.000 µl
Different Inj Volume from Sample Entry!  Actual Inj Volume : 10.000 µl
Acq. Method     : C:\Users\Public\Documents\ChemStation\1\Data\SUN\SUN 2026-07-07 11-15-38\IBN3-
                  10-20.M
Last changed    : 30/7/2023 1:22:19 pm by SYSTEM
Analysis Method : C:\Users\Public\Documents\ChemStation\1\Data\SUN\SUN 2026-07-07 11-15-38\IA3-
                  20-10.M (Sequence Method)
Last changed    : 7/7/2026 8:26:54 pm by SYSTEM
                  (modified after loading)
Additional Info : Peak(s) manually integrated
```

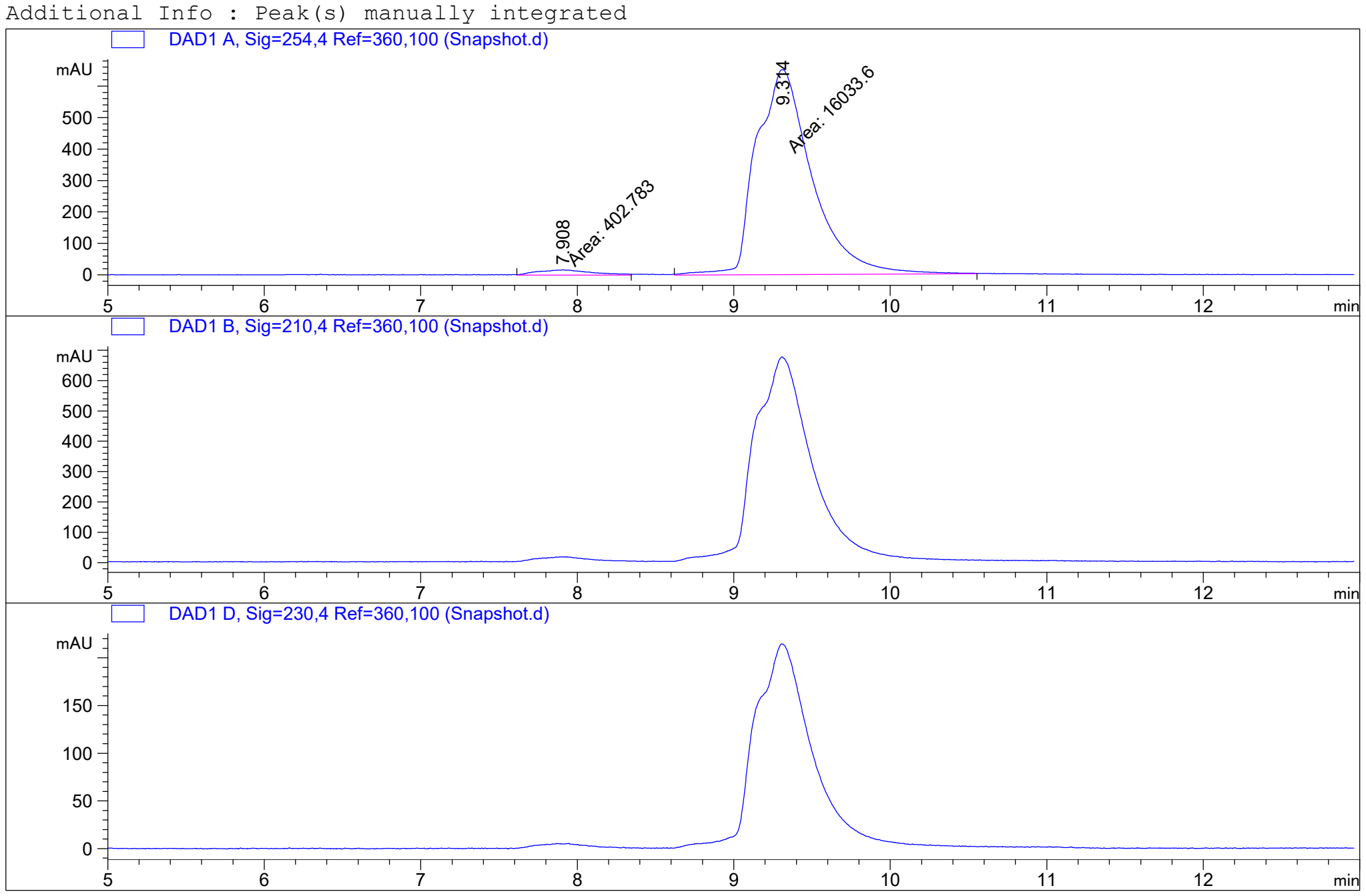


```
=====================================================================
                         Area Percent Report
=====================================================================

Sorted By             :      Signal
Multiplier            :      1.0000
Dilution              :      1.0000
Use Multiplier & Dilution Factor with ISTDs
```


Data File C:\Users\Public\Documents\ChemStation\1\Data\Snapshot.d
Sample Name:

Signal 1: DAD1 A, Sig=254,4 Ref=360,100

Peak #	RetTime [min]	Type	Width [min]	Area [mAU*s]	Height [mAU]	Area %
1	7.908	MM	0.4053	402.78265	16.56171	2.4506
2	9.314	MM	0.4105	1.60336e4	650.95575	97.5494

Totals : 1.64363e4 667.51746

Signal 2: DAD1 B, Sig=210,4 Ref=360,100

Signal 3: DAD1 D, Sig=230,4 Ref=360,100

===
*** End of Report ***

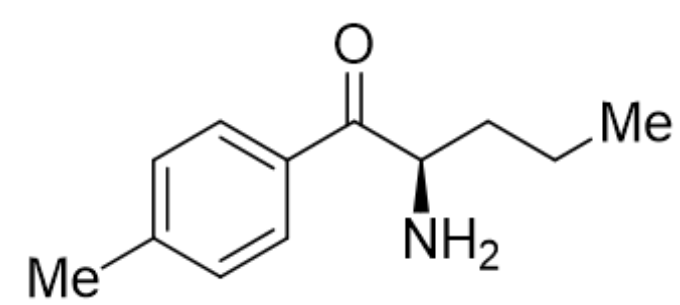

8a
